# Structure–Activity Relationship Study on Soticlestat Derivatives for the Discovery of CYP46A1 (CH24H) Inhibitors

**DOI:** 10.3390/molecules31030460

**Published:** 2026-01-28

**Authors:** Xinwei Hu, Wenqian Huang, Xiaotong Lin, Hao Zhang, Yishu Huang, Jiang Wu, Guilong Zhao

**Affiliations:** 1 Guangzhou University of Chinese Medicine, Guangzhou 510006, China; 19966405675@163.com; 2 Zhongshan Institute for Drug Discovery, Shanghai Institute of Materia Medica, Chinese Academy of Sciences, Zhongshan 528400, China; 18379981865@163.com (W.H.); linxiaotong@zidd.ac.cn (X.L.); 19003708@mail.ecust.edu.cn (H.Z.); huangyishu@simm.ac.cn (Y.H.); 3 Shanghai Institute of Materia Medica, Chinese Academy of Sciences, Shanghai 201203, China; 4 University of Chinese Academy of Sciences, Beijing 100049, China; 5 College of Pharmacy, Shenzhen Technology University, Shenzhen 518118, China

**Keywords:** CH24H, CYP46A1, soticlestat, structure-activity relationship, Dravet syndrome, Lennox-Gastaut syndrome

## Abstract

Dravet syndrome (DS) and Lennox–Gastaut syndrome (LGS) are rare, severe developmental and epileptic encephalopathies with poor prognosis, and novel drugs are urgently needed to meet clinical needs. CYP46A1 (cholesterol 24-hydroxylase, CH24H) is mainly responsible for the metabolism of cholesterol to 24(S)-hydroxycholesterol in the brain and is implicated in many brain disorders through the mediation of excitatory amino acid transporter 2 (EAAT2) and N-methyl-D-aspartate (NMDA) receptors. Inhibition of CYP46A1 is supposed to provide a novel treatment for disorders associated with neural hyperexcitation, such as epilepsy and epileptic syndromes. Soticlestat, a potent CYP46A1 inhibitor being developed by Takeda, is indicated for LGS and DS but suffers from unsatisfactory in vivo potency in animal models and clinical trials. We designed three series of soticlestat derivatives to explore the structure–activity relationship (SAR) with the aim of finding more potent CYP46A1 inhibitors and understanding the SAR of CYP46A1 inhibitors represented by soticlestat. Eventually, three compounds with a benzenesulfonamide moiety (in subseries C-4) that serves as an isostere of OH in soticlestat were discovered with very potent CYP46A1 inhibitory activities comparable to soticlestat, and an interesting flat SAR profile was observed in some subseries. The findings in the present study provide insight into the SAR of CYP46A1 inhibitors and should be valuable for the future design of novel CYP46A1 inhibitors.

## 1. Introduction

Dravet syndrome (DS) and Lennox–Gastaut syndrome (LGS) belong to rare, severe developmental and epileptic encephalopathies (DEEs), which are characterized by early-onset treatment-resistant seizures, cognitive and behavioral impairment, poor prognosis, and high mortality rates [1,2,3]. Advances have been made over the past few decades in the development of pharmacological and non-pharmacological therapies for DS and LGS, such as clobazam, fenfluramine, stiripentol, cannabidiol, the ketogenic diet, and emerging therapies targeting DNA and RNA regulation; however, there are still urgent clinical needs for novel drugs due to low response rates and severe adverse effects associated with current therapies [4,5,6].

Cholesterol is an important structural and functional component in the brain. After de novo synthesis in the brain, cholesterol is metabolized to 24(S)-hydroxycholesterol, which then crosses the blood–brain barrier (BBB) to enter the circulation and is delivered to the liver for further degradation [7]. The main enzyme responsible for the metabolism of cholesterol is cholesterol 24-hydroxylase (CH24H), also known as cholesterol 24S-hydroxylase or CYP46A1. CYP46A1 is predominantly expressed in the brain and is a crucial enzyme in brain cholesterol homeostasis. It is implicated in many neurological functions by regulating the level of 24(S)-hydroxycholesterol in the brain and is thus emerging as a novel promising therapeutic target for the treatment of a lot of brain disorders, such as Alzheimer’s disease (AD), Parkinson’s disease (PD), and Huntington’s disease (HTD) [8,9,10]. Under certain pathological conditions, CYP46A1 is upregulated and disrupts the glutamate transporter excitatory amino acid transporter 2 (EAAT2) [11], thereby impairing glutamate uptake function; in addition, 24(S)-hydroxycholesterol is a positive allosteric modulator of N-methyl-D-aspartate (NMDA) receptors, an important class of excitatory receptors in the brain [12]. Increased levels of extracellular glutamate arising from EAAT2 disruption and NMDA receptor activation by 24(S)-hydroxycholesterol can cause glutamate-evoked neuronal excitotoxicity. Therefore, CYP46A1 inhibitors are supposed to suppress neuronal excitotoxicity present in CYP46A1 pathological conditions and provide a novel treatment for disorders associated with neural hyperexcitation, such as epilepsy and epileptic syndromes.

**Figure 1:**
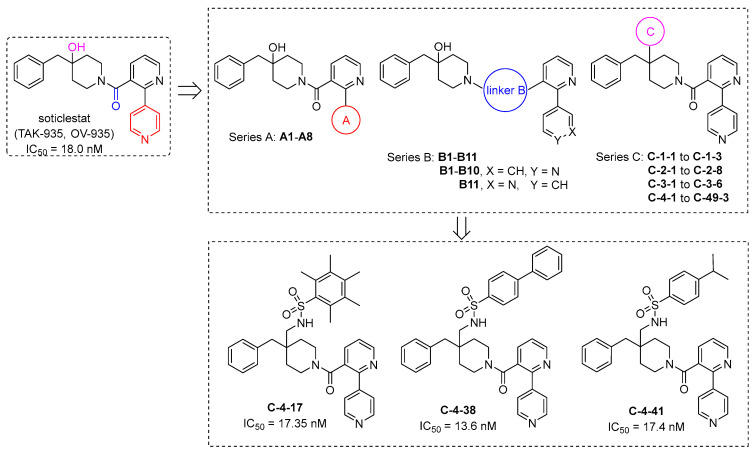
Chemical modifications based on soticlestat in the present study.

Takeda Pharmaceuticals Company has been developing soticlestat (TAK-935, OV-935), a potent CYP46A1 inhibitor, for the treatment of LGS and DS (Figure 1) [13,14]. However, it suffers from unsatisfactory in vivo potency in animals and clinical trials. In a preclinical study, soticlestat reduced seizures by only 42% and lowered brain 24(S)-hydroxycholesterol levels by only 55% at 30 mg/kg (po), a dose associated with near-maximal efficacy [15]. In major clinical trials, soticlestat, at doses of up to 600 mg/day, did not show significantly higher efficacy in reducing convulsive seizures in patients with LGS, although such significance was observed in patients with DS [14]. Encouraged by these aforementioned shortcomings, we initiated a program aiming to find more potent CYP46A1 inhibitors based on soticlestat as a chemical template. A total of three series with 85 derivatives were designed through the modification of the distal pyridine ring in soticlestat (Series A), modification of the linker connecting the bipyridine and piperidine ring in soticlestat (Series B), and replacement of the hydroxyl group in soticlestat using a bioisosterism strategy (Series C, further categorized into four subseries, C-1, C-2, C-3, and C-4). These compounds were evaluated for in vitro CYP46A1 inhibitory activity, and some CYP46A1 inhibitors with potent inhibitory activities (IC_50_) comparable to that of soticlestat were discovered (Figure 1). These findings should be valuable for the future design of novel CYP46A1 inhibitors.

## 2. Results and Discussion

### 2.1. Chemistry

**Scheme 1:**
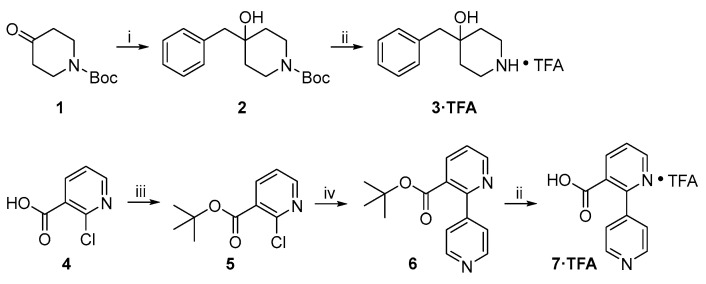
Synthetic routes to intermediates **3·TFA** and **7·TFA**. Reagents and conditions: (i) BnMgCl, dried THF, 0 °C-rt, N_2_, 48%; (ii) TFA, CH_2_Cl_2_, 0 °C-rt; (iii) (a) (COCl)_2_, DMF (cat.), dried CH_2_Cl_2_, 0 °C-rt, N_2_; (b) *t*-BuOK, dried THF, −10 °C-rt, N_2_, 60% (over two steps); (iv) pyridine-4-boronic acid, Pd(PPh_3_)_4_, Na_2_CO_3_, DME/H_2_O, reflux, N_2_, 50%.

The synthetic routes to two key intermediates **3** and **7** (both as trifluoroacetic acid (TFA) salts) are shown in Scheme 1. Commercially available ketone **1** was treated with BnMgCl to give tertiary alcohol **2**, and the Boc protecting group in the latter was then cleaved by TFA to give **3** as a TFA salt. On the other hand, treatment of commercially available carboxylic acid **4** with (COCl)_2_ in the presence of a catalytic amount of N,N-dimethylformamide (DMF) afforded the corresponding carbonyl chloride, which was subsequently treated with *t*-BuOK to give the corresponding *tert*-butyl ester. Suzuki coupling of **5** and pyridine-4-boronic acid in the presence of Pd(PPh_3_)_4_ as a catalyst and Na_2_CO_3_ as a base in refluxing DME (1,2-dimethoxyethane)/H_2_O produced bipyridine **6**. Cleavage of the *tert*-butyl ester moiety with TFA gave the corresponding carboxylic acid **7** as a TFA salt.

**Scheme 2:**
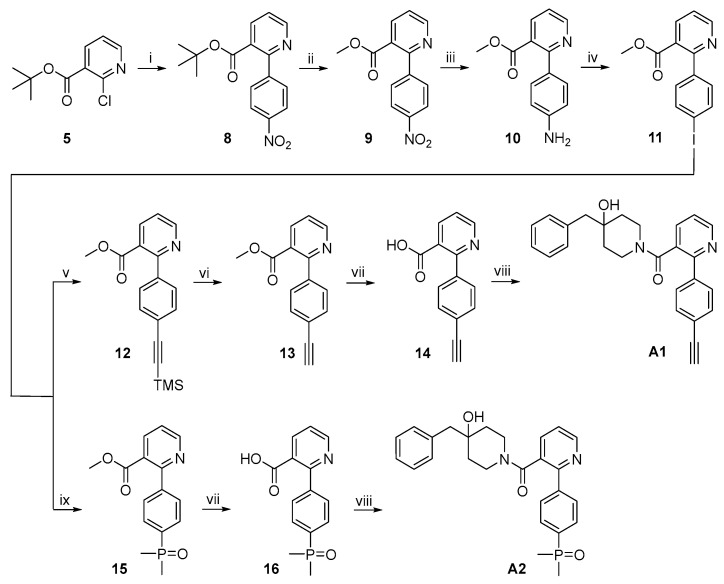
Synthetic routes to target compounds **A1** and **A2**. Reagents and conditions: (i) 4-nitrophenylboronic acid, Pd(PPh_3_)_4_, Na_2_CO_3_, DME/H_2_O, reflux, N_2_, 86%; (ii) (a) TFA, CH_2_Cl_2_, 0 °C-rt; (b) SOCl_2_/MeOH, reflux, N_2_, 76% (over two steps); (iii) H_2_, 10%Pd/C, MeOH, rt, 75%; (iv) (a) NaNO_2_, HCl/H_2_O, 0 °C; (b) KI, 0 °C-rt, 44%; (v) trimethylsilylacetylene, CuI/PdCl_2_(PPh_3_)_2_, Et_3_N, dried THF, rt, N_2_, 73%; (vi) TBAF, dried THF, 0 °C-rt, N_2_, 65%; (vii) aqNaOH, MeOH, rt; (viii) **3·TFA**, EDCI/HOBT, DIPEA, dried THF, 0 °C-rt, N_2_, 44% (overall for **13** to **A1**), 18% (overall for **15** to **A2**); (ix) HP(=O)Me_2_, Xantphos, Pd_2_(dba)_3_, Et_3_N, dioxane, rt, N_2_, 59%.

The synthetic routes to target compounds **A1** and **A2** are shown in Scheme 2. Suzuki coupling of **5** and 4-nitrophenylboronic acid under the same conditions used above produced **8**. The *tert*-butyl ester functionality in **8** was converted to the corresponding methyl ester functionality in **9** by cleavage with TFA followed by acid-catalyzed esterification of the carboxylic acid intermediate in refluxing MeOH. Pd-catalyzed hydrogenolysis of NO_2_ in **9** at atmospheric pressure and room temperature smoothly afforded aniline **10**, which was subjected to a Sandmeyer reaction to give iodobenzene **11**. CuI/PdCl_2_(PPh_3_)_2_-mediated Sonogashira coupling of **11** and trimethylsilylacetylene furnished **12**, which was treated with tetra-*n*-butylammonium fluoride (TBAF) to remove the trimethylsilyl (TMS) protecting group to give **13**. NaOH-mediated saponification of methyl ester **13** followed by 1-ethyl-3-(3-dimethylaminopropyl)carbodiimide hydrochloride (EDCI)/1-hydroxybenzotriazole (HOBT)-mediated amide formation with **3**·**TFA** produced target compound **A1** [16,17]. Pd-mediated cross-coupling of **11** with dimethylphosphine oxide in the presence of Pd_2_(dba)_3_/Xantphos afforded **15** [18], which was converted to target compound **A1** by a procedure identical to that used for the synthesis of **A1** from **13**.

**Scheme 3:**
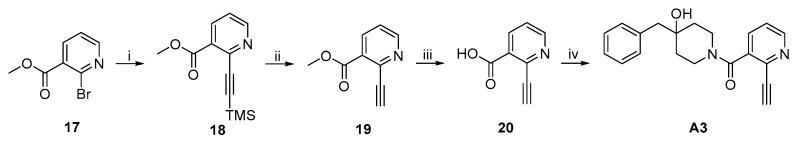
Synthetic route to target compound **A3**. Reagents and conditions: (i) trimethylsilylacetylene, CuI/PdCl_2_(PPh_3_)_2_, Et_3_N, dried THF, rt, N_2_, 74%; (ii) TBAF, dried THF, 0 °C-rt, N_2_, 66%; (iii) aqNaOH, MeOH, rt; (iv) **3·TFA**, EDCI/HOBT, DIPEA, dried THF, 0 °C-rt, N_2_, 38% (overall for **19** to **A3**).

The synthetic route to **A3** is shown in Scheme 3. Sonogashira coupling of commercially available **17** and trimethylsilylacetylene under the same conditions described above gave **18**, which was converted to target compound **A3** following the same procedure used for the synthesis of **A1** from **12**.

**Scheme 4:**
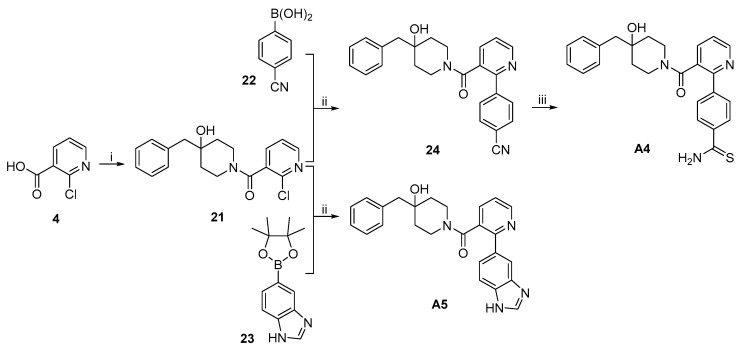
Synthetic routes to target compounds **A4** and **A5**. Reagents and conditions: (i) (a) (COCl)_2_, DMF (cat.), dried CH_2_Cl_2_, 0 °C-rt, N_2_; (b) **3·TFA**, Et_3_N, dried THF, 0 °C-rt, N_2_, 31% (over two steps); (ii) Pd(PPh_3_)_4_, Na_2_CO_3_, DME/H_2_O, reflux, N_2_, 61% for **24**, 10% for **A5**; (iii) (NH_4_)_2_S, Et_3_N, pyridine, 50 °C, N_2_, 87%.

The synthetic routes to target compounds **A4** and **A5** are shown in Scheme 4. Treatment of **4** with (COCl)_2_ in the presence of a catalytic amount of DMF afforded the corresponding carbonyl chloride, which was reacted with **3**·**TFA** in the presence of Et_3_N to give amide **21**. Suzuki coupling of **21** and boronic acid **22** under the same conditions used above produced **24**, which was heated with (NH_4_)_2_S in the presence of Et_3_N in pyridine at 50 °C to give target compound **A4** [19]. Suzuki coupling of **21** and boronic acid **23** under the same conditions above produced target compound **A5**.

**Scheme 5:**
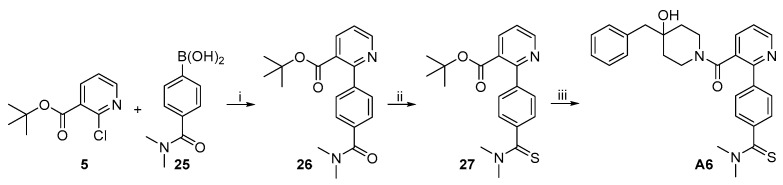
Synthetic route to target compound **A6**. Reagents and conditions: (i) Pd(PPh_3_)_4_, Na_2_CO_3_, DME/H_2_O, reflux, N_2_, 76%; (ii) Lawesson reagent, PhCH_3_, reflux, N_2_, 57%; (iii) (a) TFA, CH_2_Cl_2_, 0 °C-rt; (b) **3·TFA**, EDCI/HOBT, DIPEA, dried THF, 0 °C-rt, N_2_, 45% (over two steps).

The synthetic route to target compound **A6** is depicted in Scheme 5. Suzuki coupling of **5** and boronic acid **25** under the same conditions used above produced **26**. The amide moiety in **26** was transformed into the corresponding thioamide with Lawesson reagent in refluxing toluene to give **27** [20], which was further converted to target compound **A6** by the same procedure used for the synthesis of **A1** from **14**.

**Scheme 6:**
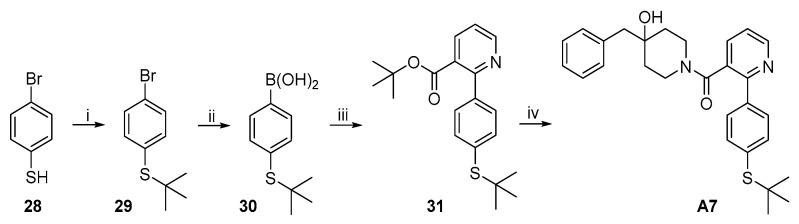
Synthetic route to target compound **A7**. Reagents and conditions: (i) *t*-BuCl, anhydrous AlCl_3_, MeCN, rt, N_2_, 80%; (ii) (a) *n*-BuLi, dried THF, −78 °C, N_2_; (b) B(OCH_3_)_3_, −78 °C-rt, N_2_, 72%; (iii) **5**, Pd(PPh_3_)_4_, K_2_CO_3_, dioxane/H_2_O, reflux, N_2_, 80%; (iv) (a) TFA, CH_2_Cl_2_, 0 °C-rt; (b) **3·TFA**, EDCI/HOBT, DIPEA, dried THF, 0 °C-rt, N_2_, 58% (over two steps).

The synthetic route to target compound **A7** is shown in Scheme 6. Treatment of commercially available thiophenol **28** with *t*-BuCl in the presence of anhydrous AlCl_3_ produced thioether **29** [21]. Lithiation of phenyl bromide **29** by treatment with *n*-BuLi at −78 °C followed by trapping of the phenyl cabanion with B(OMe)_3_ afforded phenyl boronic acid **30**. Suzuki coupling of **30** and **5** under the same conditions described above gave **31**, which was converted to target compound **A7** by the same procedure used for the synthesis of **A6** from **27**.

**Scheme 7:**
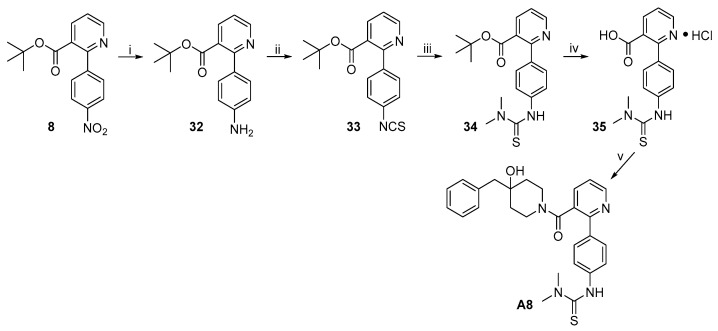
Synthetic route to target compound **A8**. Reagents and conditions: (i) H_2_, 10%Pd/C, MeOH, 71%; (ii) CSCl_2_, Et_3_N, dried CH_2_Cl_2_, 0 °C-rt, N_2_, 78%; (iii) NH(CH_3_)_2_·HCl, Et_3_N, dried THF, 0 °C-rt, N_2_, 82%; (iv) (a) TFA, CH_2_Cl_2_; (b) SOCl_2_/MeOH, 0 °C-rt; (v) **3·TFA**, EDCI/HOBT, DIPEA, dried THF, 0 °C-rt, N_2_, 62% (overall for **34** to **A8**).

The synthetic route to target compound **A8** is outlined in Scheme 7. Hydrogenolysis of nitrobenzene **8** to aniline **32** was achieved under the same conditions described above, and the latter was transformed into thiourea **34** by initial treatment with CSCl_2_ to give isothiocyanate **33** and subsequent treatment with dimethylamine. Cleavage of the *tert*-butyl ester moiety with TFA followed by treatment with HCl in MeOH generated in situ by SOCl_2_ in MeOH gave **35** as an HCl salt. EDCI/HOBT-mediated amide formation of **35** and **3·TFA** under the same conditions described above produced target compound **A8**.

**Scheme 8:**
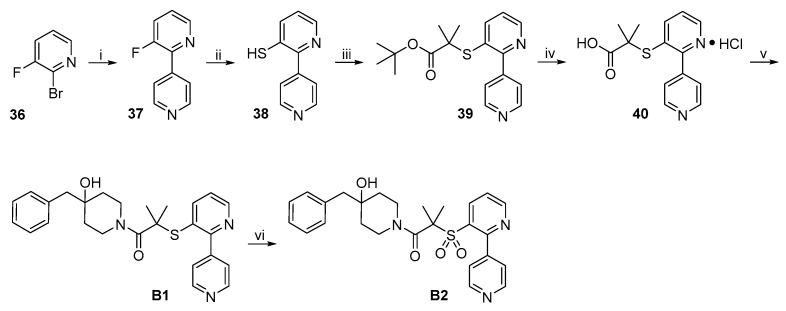
Synthetic route to target compounds **B1** and **B2**. Reagents and conditions: (i) pyridine-4-boronic acid, Pd(PPh_3_)_4_, K_2_CO_3_, dioxane/H_2_O, reflux, N_2_, 79%; (ii) Na_2_S, K_2_CO_3_, DMF, 120 °C, N_2_, 21%; (iii) BrC(CH_3_)_2_CO_2_Bu^t^, K_2_CO_3_, DMF, 45 °C, N_2_, 36%; (iv) (a) TFA, CH_2_Cl_2_, 0 °C-rt; (b) SOCl_2_/MeOH, 0 °C-rt; (v) **3·TFA**, EDCI/HOBT, DIPEA, dried THF, 0 °C-rt, N_2_, 41% (overall for **39** to **B1**); (vi) *m*CPBA, CH_2_Cl_2_, 0 °C-rt, N_2_, 82%.

The synthetic route to target compounds **B1** and **B2** is shown in Scheme 8. Suzuki coupling of commercially available **36** and pyridine-4-boronic acid afforded **37** under the same conditions used above. Treatment of fluoropyridine **37** with Na_2_S in the presence of K_2_CO_3_ in DMF at 120 °C led to the formation of thiophenol **38** via aromatic nucleophilic substitution. Substitution reaction of **38** with *tert*-butyl 2-bromo-2-methylpropanoate in the presence of K_2_CO_3_ in DMF at 45 °C gave **39**, which was converted to target compound **B1** by the same procedure described above. Oxidation of thioether **B1** to the corresponding sulfone **B2** was achieved by treatment with *m*-chloroperoxybenzoic acid (*m*CPBA) in CH_2_Cl_2_ at room temperature.

**Scheme 9:**
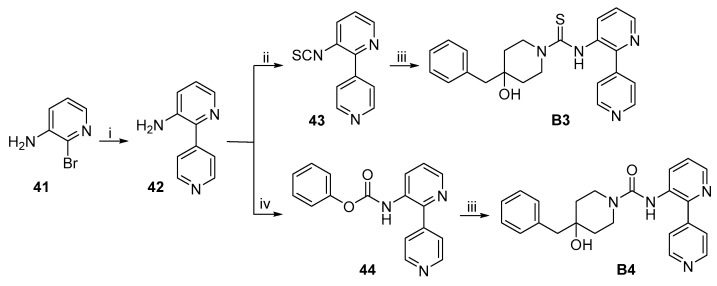
Synthetic routes to target compounds **B3** and **B4**. Reagents and conditions: (i) pyridine-4-boronic acid, Pd(PPh_3_)_4_, K_2_CO_3_, dioxane/H_2_O, reflux, N_2_, 37%; (ii) CSCl_2_, Et_3_N, dried CH_2_Cl_2_, 0 °C-rt, N_2_; (iii) **3·TFA**, Et_3_N, dried THF, 0 °C-rt, N_2_, 65% for **B3**, 78% for **B4**; (iv) PhOCOCl, pyridine, MeCN, 0 °C-rt, N_2_, 84%.

The synthetic routes to target compounds **B3** and **B4** are shown in Scheme 9. Suzuki coupling of commercially available **41** and pyridine-4-boronic acid afforded **42** under the same conditions used above. The amine **42** was converted to isothiocyanate **43** by treatment with CSCl_2_ as described above. Treatment of **43** with **3**·**TFA** in the presence of Et_3_N afforded target compound **B3**. Treatment of amine **42** with phenyl chloroformate in the presence of pyridine gave benzyl carbamate **44**, which was subsequently treated with **3**·**TFA** in the presence of Et_3_N to produce target compound **B4**.

**Scheme 10:**
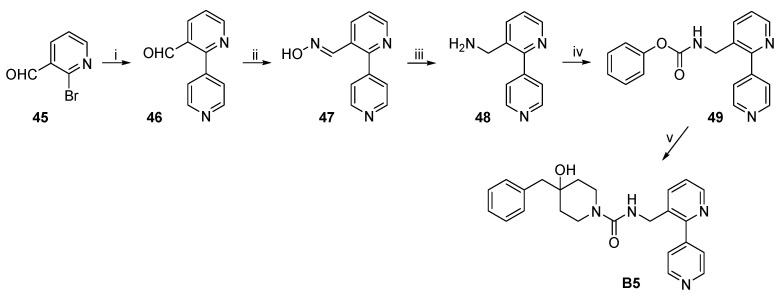
Synthetic route to target compound **B5**. Reagents and conditions: (i) pyridine-4-boronic acid, Pd(PPh_3_)_4_, K_2_CO_3_, dioxane/H_2_O, reflux, N_2_, 57%; (ii) NH_2_OH·HCl, NaOH, EtOH/H_2_O, reflux, N_2_, 55%; (iii) Zn, AcONH_4_, NH_3_·H_2_O, MeCN, reflux, N_2_; (iv) PhOCOCl, pyridine, MeCN, 0 °C-rt, N_2_; (v) **3·TFA**, Et_3_N, dried THF, 0 °C-rt, N_2_, 76% (overall for **47** to **B5**).

The synthetic route to target compound **B5** is shown in Scheme 10. Suzuki coupling of commercially available **45** and pyridine-4-boronic acid, as described above, afforded **46**. Aldehyde **46** was treated with hydroxylamine to give oxime **47**, which was then reduced to primary amine **48** with zinc powder in the presence of AcONH_4_ and aqueous ammonia in refluxing MeCN. Treatment of **48** with phenyl chloroformate followed by reaction with **3·TFA** produced **B5** by the same procedure described above.

**Scheme 11:**
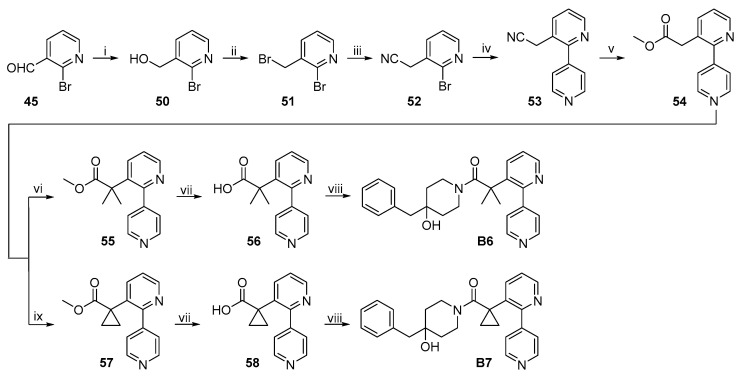
Synthetic routes to target compounds **B6** and **B7**. Reagents and conditions: (i) NaBH_4_, MeOH, 0 °C-rt, N_2_, 96%; (ii) PBr_3_, dried CH_2_Cl_2_, 0 °C-reflux, N_2_, 51%; (iii) TMSCN, TBAF, MeCN, 0 °C-rt, N_2_, 85%; (iv) pyridine-4-boronic acid, Pd(PPh_3_)_4_, Na_2_CO_3_, DME/H_2_O, reflux, N_2_, 56%; (v) (a) H_2_SO_4_/H_2_O/AcOH (1/1/10 by *v*/*v*/*v*), reflux, N_2_; (b) SOCl_2_, MeOH, 0 °C-reflux, N_2_, 47% (over two steps); (vi) (a) LiHMDS, dried THF, −78 °C, N_2_; (b) CH_3_I, dried THF, −78 °C-rt, N_2_, 84%; (vii) aqNaOH, MeOH, 45 °C, 34% for **56**, 76% for **58**; (viii) (a) (COCl)_2_, DMF (cat.), dried CH_2_Cl_2_, 0 °C-rt, N_2_; (b) **3·TFA**, Et_3_N, dried THF, 0 °C-rt, N_2_, 19% for **B6** (over two steps), 38% for **B7** (over two steps); (ix) (a) LiHMDS, dried THF, −78 °C, N_2_; (b) 1,3,2-dioxathiolane 2,2-dioxide, dried THF, −78 °C-rt, N_2_, 60%.

The synthetic routes to target compounds **B6** and **B7** are shown in Scheme 11. Aldehyde **45** was reduced by NaBH_4_ to the corresponding primary alcohol **50**, and the latter was subsequently converted to the corresponding bromide **51** by treatment with PBr_3_ in refluxing CH_2_Cl_2_. TBAF-mediated substitution of **51** with TMSCN afforded nitrile **52**. Suzuki coupling of **52** and pyridine-4-boronic acid under the same conditions described above gave **53**. Acidic hydrolysis of nitrile **53** with H_2_SO_4_ in refluxing aqueous acetic acid gave corresponding carboxylic acid, which was then esterified to the corresponding methyl ester **54** in refluxing HCl/MeOH. Deprotonation of **54** with excess lithium bis(trimethylsilyl)amide (LiHMDS) followed by *gem*-dimethylation with excess MeI produced *gem*-dimethylated ester **55** [22]. Saponification of **55** with NaOH led to the corresponding acid **56**, which was converted to target compound **B6** by a procedure identical to that used for the synthesis of **21** from **4**. Deprotonation of **54** with excess LiHMDS followed by treatment with 1,3,2-dixoathiolane 2,2-dioxide produced a cyclopropane counterpart **57** [23], which was converted to target compound **B7** following the same procedure used for **B6**.

**Scheme 12:**
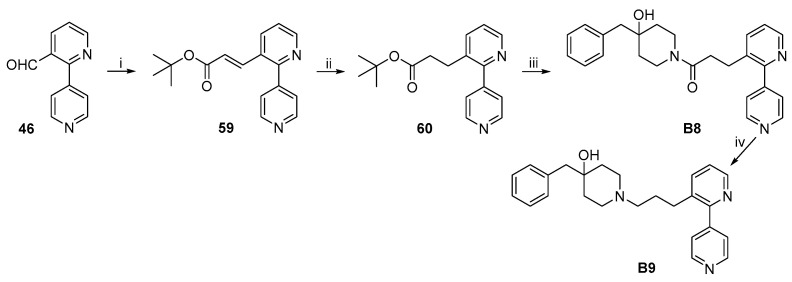
Synthetic route to target compounds **B8** and **B9**. Reagents and conditions: (i) Ph_3_P=CHCOOBu*^t^*, DBU, dried THF, 0 °C-rt, N_2_, 50%; (ii) H_2_, 10%Pd/C, MeOH, 83%; (iii) (a) TFA, CH_2_Cl_2_, 0 °C-rt; (b) **3·TFA**, EDCI/HOBT, DIPEA, dried THF, 0 °C-rt, N_2_, 41% (over two steps); (iv) LiAlH_4_, dried THF, 0–50 °C, N_2_, 50%.

**Scheme 13:**
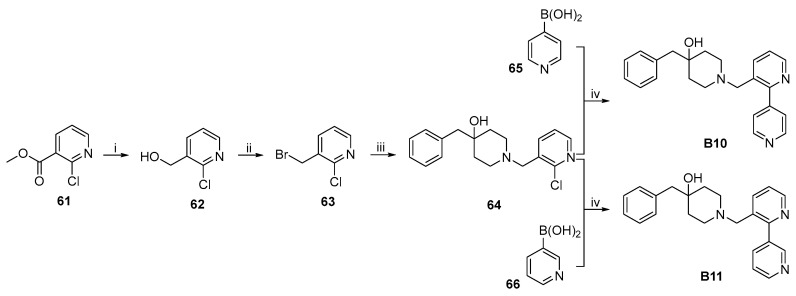
Synthetic routes to target compounds **B10** and **B11**. Reagents and conditions: (i) LiAlH_4_, dried THF, 0 °C-rt, N_2_, 27%; (ii) PBr_3_, dried CH_2_Cl_2_, 0 °C-rt, N_2_, 47%; (iii) **3·TFA**, aqNaOH, dioxane, 0 °C-rt, 60%; (iv) Pd(PPh_3_)_4_, K_2_CO_3_, dioxane/H_2_O, reflux, N_2_, 46% for **B10**, 54% for **B11**.

The synthetic routes to target compounds **B8**–**B11** are shown in Scheme 12 and Scheme 13. The experimental details have been published in our earlier work [24].

**Scheme 14:**
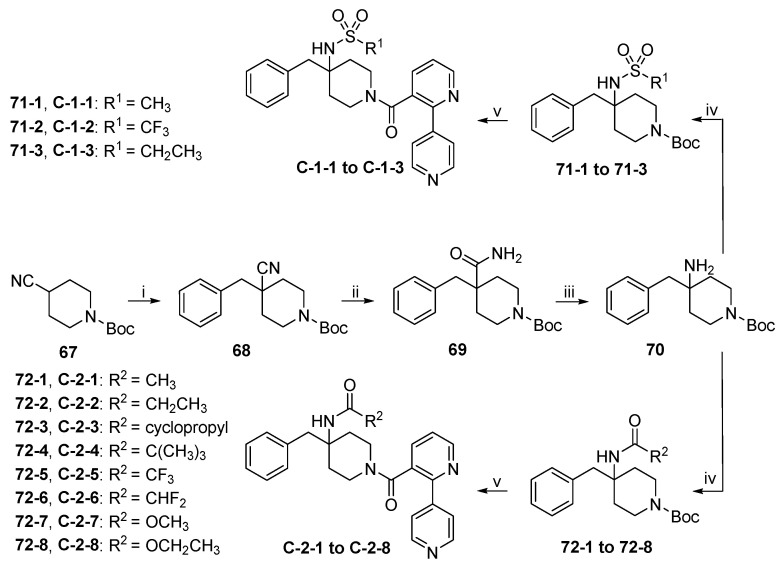
Synthetic routes to target compounds **C-1-1 to C-1-3** and **C-2-1 to C-2-8**. Reagents and conditions: (i) (a) LDA, dried THF, −78 °C, N_2_; (b) BnBr, dried THF, −78 °C-rt, N_2_, 82%; (ii) K_2_CO_3_, H_2_O_2_, DMSO, 0 °C-rt, 94%; (iii) PIFA, MeCN/H_2_O, rt, 55%; (iv) for **71-3**, **72-1**, **72-3**, and **72-4**: carbonyl or sulfonyl chlorides, Et_3_N, dried CH_2_Cl_2_, 0 °C-rt, N_2_, 6% for **71-3**, 52% for **72-1**, 35% for **72-3**, 78% for **72-4**; for the others: carbonyl or sulfonyl anhydrides, DMAP, Et_3_N, dried CH_2_Cl_2_, 0 °C-rt, N_2_, 47% for **71-1**, 50% for **71-2**, 72% for **72-2**, 60% for **72-5**, 80% for **72-6**; 77% for **72-7**, 72% for **72-8**; (v) (a) TFA, CH_2_Cl_2_, 0 °C-rt; (b) **7·TFA**, EDCI/HOBT, DIPEA, dried THF, 0 °C-rt, N_2_, 50% for **C-1-1**, 49% for **C-1-2**, 45% for **C-1-3**, 45% for **C-2-1**, 47% for **C-2-2**, 61% for **C-2-3**, 16% for **C-2-4**, 60% for **C-2-5**, 17% for **C-2-6**, 24% for **C-2-7**, 55% for **C-2-8**, all yields were for two steps.

The synthetic routes to target compounds **C-1-1** to **C-1-3** and **C-2-1** to **C-2-8** are shown in Scheme 14. Deprotonation of commercially available **67** with lithium diisopropylamide (LDA) at −78 °C followed by benzylation with BnBr afforded **68**, which then gave rise to amide **69** upon treatment with H_2_O_2_/K_2_CO_3_ in DMSO [25]. Phenyliodine (III) bis(trifluoroacetate) (PIFA)-mediated Hofmann rearrangement of amide **69** in MeCN/H_2_O at room temperature smoothly led to the formation of tertiary amine **70** [26]. Reactions of amine **70** with sulfonyl chlorides or anhydrides produced sulfonamides **71-1** to **71-3**, while reactions with carbonyl chlorides or anhydrides produced amides **72-1** to **72-8**. Cleavage of Boc in **71-1** to **71-3** and **72-1** to **72-8** followed by EDCI/HOBT-mediated amide formation with **7**·**TFA,** as described above, gave target compounds **C-1-1** to **C-1-3** and **C-2-1** to **C-2-8**, respectively.

**Scheme 15:**
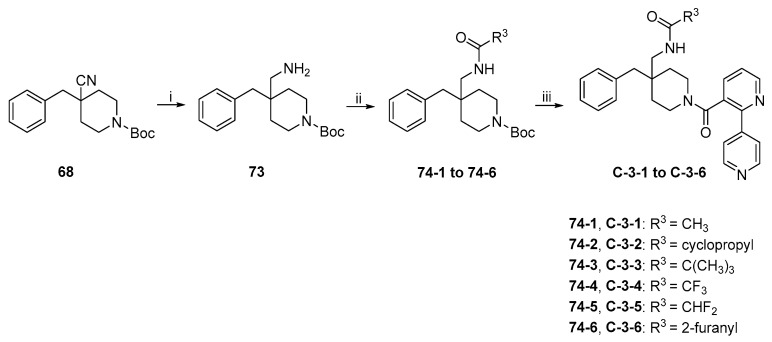
Synthetic route to target compounds **C-3-1 to C-3-6**. Reagents and conditions: (i) NaBH_4_, NiCl·6H_2_O, MeOH, 0 °C-rt, N_2_, 88%; (ii) for **74-4** and **74-5**: carbonyl anhydrides, DMAP, Et_3_N, dried CH_2_Cl_2_, 0 °C-rt, N_2_, 73% for **74-4**, 70% for **74-5**; for the others: carbonyl chlorides, Et_3_N, dried CH_2_Cl_2_, 0 °C-rt, N_2_, 95% for **74-1**, 71% for **74-2**, 90% for **74-3**, 88% for **74-6**; (iii) (a) TFA, CH_2_Cl_2_, 0 °C-rt; (b) **7·TFA**, EDCI/HOBT, DIPEA, dried THF, 0 °C-rt, N_2_, 14% for **C-3-1**, 55% for **C-3-2**, 62% for **C-3-3**, 55% for **C-3-4**, 48% for **C-3-5**, 41% for **C-3-6**, all yields were for two steps.

The synthetic route to target compounds **C-3-1** to **C-3-6** is shown in Scheme 15. Nitrile **68** was reduced with NaBH_4_ mediated by NiCl_2_·6H_2_O in MeOH to give primary amine **73**, which was converted to amides **74-1** to **74-6** by the same procedure described above for **72-1** to **72-8**. Cleavage of Boc in **74-1** to **74-6** followed by EDCI/HOBT-mediated amide formation with **7·TFA**, using the same procedure as above, led to the formation of target compounds **C-3-1** to **C-3-6**.

**Scheme 16:**
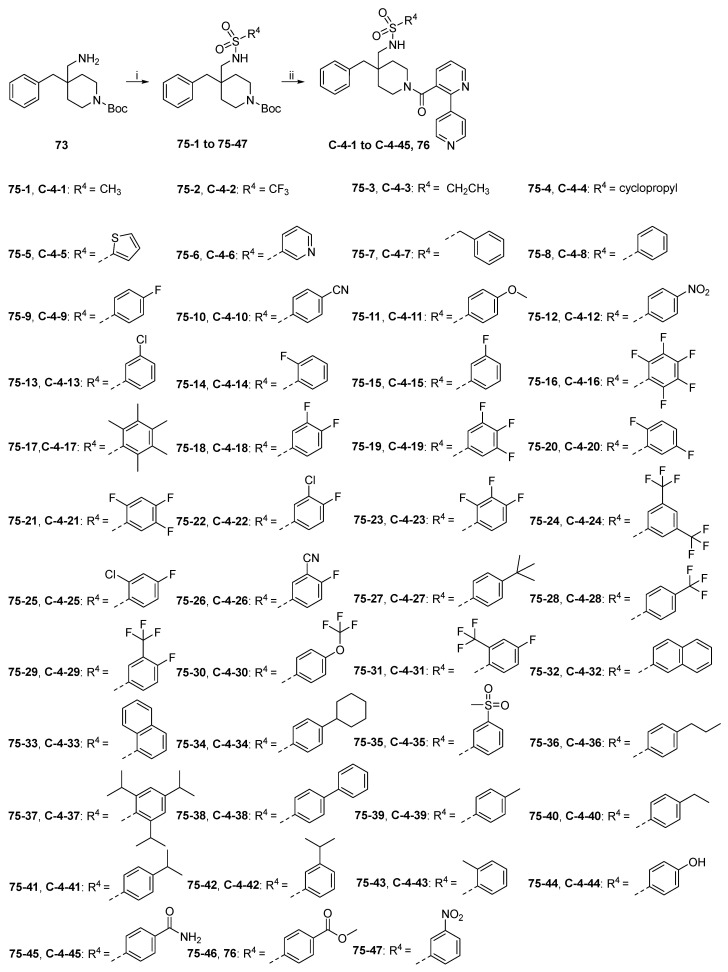
Synthetic route to target compounds **C-4-1** to **C-4-45** and **76**. Reagents and conditions: (i) for **75-1**: Ms_2_O, DMAP, Et_3_N, dried CH_2_Cl_2_, 0 °C-rt, N_2_, 35%; for the others: acid chlorides, Et_3_N, dried CH_2_Cl_2_, 0 °C-rt, N_2_, for isolated yields, see experimental section. (ii) (a) TFA, CH_2_Cl_2_, 0 °C-rt; (b) **7·TFA**, EDCI/HOBT, DIPEA, dried THF, 0 °C-rt, N_2_, for isolated yields, see experimental section.

The synthetic route to target compounds **C-4-1** to **C-4-45** is shown in Scheme 16. Primary amine **73** was converted to **C-4-1** to **C-4-45** by a procedure identical to that used for **C-3-1** to **C-3-6**, as discussed above.

**Scheme 17:**
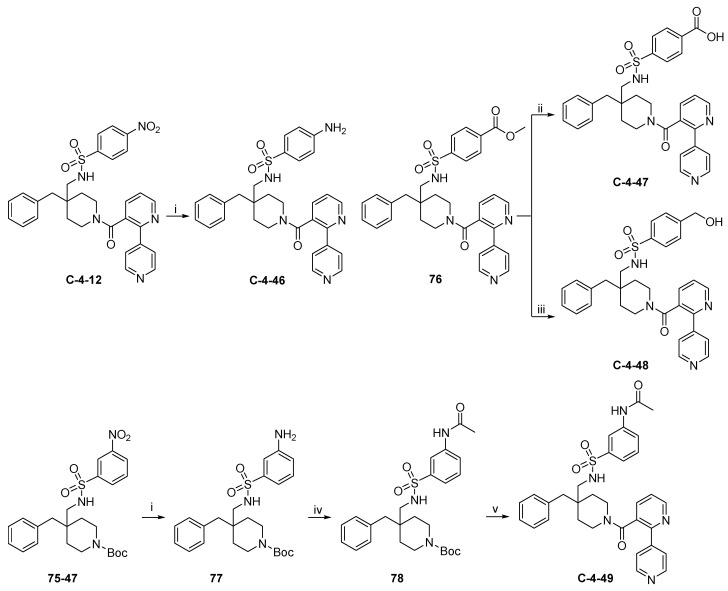
Synthetic routes to target compounds **C-4-46** to **C-4-49**. Reagents and conditions: (i) H_2_, 10%Pd/C, MeOH, rt, 40% for **C-4-46**, 87% for **77**; (ii) aqNaOH, MeOH, rt, 41%; (iii) LiAlH_4_, dried THF, 0 °C-rt, N_2_, 12%; (iv) AcCl, Et_3_N, dried CH_2_Cl_2_, 0 °C-rt, N_2_, 78%; (v) (a) TFA, CH_2_Cl_2_, 0 °C-rt; (b) **7·TFA**, EDCI/HOBT, DIPEA, dried THF, 0 °C-rt, N_2_, 82%.

The synthetic routes to target compounds **C-4-46** to **C-4-49** are shown in Scheme 17. **C-4-12** was reduced to **C-4-46** by Pd-catalyzed hydrogenolysis under the same condition used above. Saponification of the ester moiety in **76** with aqueous NaOH in MeOH led to the formation of **C-4-47**, while reduction of **76** with LiAlH_4_ produced **C-4-48**. Reduction of **75-47** by Pd-catalyzed hydrogenolysis gave aniline **77**, which was acylated with AcCl in the presence of Et_3_N to afford **78**. Compound **78** was converted to target compound **C-4-49** by the identical procedure used above.

It should be noted that all target compounds in Series A and Series C in the present study exhibited unusual NMR characteristics because there are four stable conformations at room temperature, which exist as two diastereomers, each as a pair of enantiomers, due to restricted bond rotations around two axes, i.e., the amide CO-N and CO-pyridine. This phenomenon has been intensively investigated by variable-temperature ^13^C NMR, molecular mechanics, and quantum mechanics in our earlier study [24], and will not be further discussed here.

### 2.2. In Vitro CYP46A1 Inhibition

The in vitro CYP46A1 inhibitory activity of the target compounds was determined by the inhibition of CYP46A1-mediated metabolism of testosterone to 16β-hydroxyltestosterone by the compounds tested and expressed as the half-maximal inhibitory concentration (IC_50_) using soticlestat as a positive control [27]. The results are summarized in Table 1.

As shown in Figure 1, Series A focuses on the modification of the distal pyridine ring in soticlestat with the aim of finding a moiety that can bind to the heme iron in CYP46A1 with higher affinity, because the N in the distal pyridine ring in soticlestat was believed to be a critical moiety for ligation to the CYP46A1 heme iron [13]. Unfortunately, among all the moieties screened in **A1**–**A8**, none were superior to the distal pyridine N atom in soticlestat; in fact, all modifications led to complete loss of CYP46A1 inhibition (IC_50_ values > 10,000 nM).

Series B focuses on modification of the linker connecting the bipyridine and piperidine rings, which is C=O in soticlestat. Thus, all linkers in **B1**–**B11** were associated with weaker CYP46A1 inhibitory activity than soticlestat, and the most potent one was **B10** (IC_50_ = 140.9 nM), with a CH_2_ linker compared to the C=O linker in soticlestat (IC_50_ = 18.0 nM). It should be noted that the orientation of the distal pyridine N is very important, as indicated by the dramatic difference in CYP46A1 inhibitory activity between **B10** (IC_50_ = 140.9 nM) and **B11** (IC_50_ > 10,000 nM).

Series C focuses on modification of the hydroxyl group in soticlestat using a bioisosterism strategy and is further categorized into four subseries, i.e., C-1, C-2, C-3, and C-4. Series C-1 included three compounds designed by replacement of the OH group in soticlestat with RSO_2_NH; however, none of them showed stronger CYP46A1 inhibitory activity than parent soticlestat, with the most potent one being **C-1-1,** with an IC_50_ of 102 nM. In Series C-2, the OH in soticlestat was replaced by RCONH and eight compounds were designed; like Series C-1, none of them were stronger than soticlestat, and the most potent one was **C-2-7,** with an IC_50_ of 111.8 nM. The same result was obtained in Series C-3, where the OH in soticlestat was replaced by RCONHCH_2_ and six compounds were designed, with the most potent one being C-3-4, with an IC_50_ of 156.1 nM. In Series C-4, we introduced RSO_2_NHCH_2_ to replace the OH in soticlestat. Four small alkyl substituents (the “R” in RSO_2_NHCH_2_) were initially introduced in RSO_2_NHCH_2_ to explore the space (**C-4-1** to **C-4-4**), but all of them were associated with a dramatic decrease in activity. Four different types of aromatic substituents were then introduced (**C-4-5** to **C-4-8**), and fortunately, when R = benzene ring, the CYP46A1 inhibitory activity was only slightly less potent than that of soticlestat (IC_50_ = 41.8 nM for **C-4-8** vs. 18.0 for soticlestat). Inspired by this encouraging observation, some electron-withdrawing and electron-donating groups were then explored by attaching them to the benzene ring (**C-4-9** to **C-4-13**), and it turned out that F, CN, and Cl were associated with potent activity, whereas MeO and NO_2_ had opposite effects. Based on this, a large number of derivatives with PhSO_2_NHCH_2_, where the Ph ring was substituted with F, Cl, CN, CF_3_, OCF_3_,and their various combinations, were further designed (**C-4-14** to **C-4-31**), which also included two derivatives in which the benzene ring was substituted with bulky alkyl groups, i.e., pentamethyl (**C-4-17**) and 4-*tert*-butyl (**C-4-27**), and the results showed that the SAR exhibited a flat profile, as indicated by the observation that CYP46A1 inhibitory activity did not change significantly with structural modification in these compounds, and most of them exhibited very potent CYP46A1 inhibitory activity with IC_50_ < 100 nM. However, among them, the two most potent compounds had the Ph ring substituted with pentamethyl (**C-4-17**, IC_50_ = 17.35 nM) and 4-*tert*-butyl (**C-4-27**, IC_50_ = 27.5 nM), although many other counterparts substituted with multiple halogens also exhibited IC_50_ values of < 50 nM. Inspired by this surprising result, we turned to further design many compounds with PhSO_2_NHCH_2_ where the Ph ring was substituted with a variety of alkyl groups (**C-4-32** to **C-4-34** and **C-4-36** to **C-4-43**). The assay results showed that a similar flat SAR was observed, with all alkyl-substituted compounds being very potent (IC_50_ < 100 nM), and two compounds exhibited CYP46A1 inhibitory activity slightly more potent than soticlestat (IC_50_ = 13.6 nM for **C-4-38** and 17.4 nM for **C-4-41** vs. 18.0 nM for soticlestat). Notably, in subseries C-4, the introduction of hydrophilic substituents to the benzene ring led to a dramatic decrease in activity (**C-4-35** and **C-4-44** to **C-4-49**).

### 2.3. In Vitro Inhibitory Activities of Target Compounds Against CYP1A2, CYP2C9, CYP2C19, CYP2D6, and CYP3A4

In order to evaluate the off-target inhibition of compounds in the present study against other CYP enzymes, four potent compounds, **C-4-9**, **C-4-32**, **C-4-38**, and **C-4-41**, were selected to test their inhibition against five main CYP enzymes, CYP1A2, CYP2C9, CYP2C19, CYP2D6, and CYP3A4/5. As shown in Table 2, all four selected compounds displayed weak inhibitory activities against CYP1A2, with IC_50_ values > 50 μM and high selectivities > 1000, and moderate inhibitory activities against CYP2C9, 2C19, and 2D6, with IC_50_ values of 1.54–14.7 μM and moderate selectivities of 80–406. However, these four compounds showed strong CYP3A4/5 inhibitory activities (IC_50_ values = 0.0402 to 0.403 μM), which led to low selectivities ranging from 3.0 to 11. The exact reason or chemical moieties responsible for the strong inhibition against CYP3A4/5 are unknown, since CYP3A4/5 enzymes are known to be associated with broad substrate specificity encompassing a wide range of chemical structures. However, this poor selectivity will be optimized in future work to minimize the potential for drug–drug interactions, since CYP3A4/5 is a predominant drug-metabolizing enzyme for both antiepileptic drugs and many other drugs.

## 3. Materials and Methods

### 3.1. Chemistry

Unless stated otherwise, all chemical reagents were obtained commercially and used without further purification. All dried solvents were prepared by standard methods. Reaction progress was monitored by thin-layer chromatography (TLC) on commercially available precoated TLC silica gel plates or by liquid chromatography–mass spectrometry (LC-MS), and separation and purification by flash column chromatography were performed on silica gel columns (200–300 mesh) eluting with EtOAc/petroleum ether (PE) or MeOH/CH_2_Cl_2_ (as *v*/*v*). Melting points were measured in open capillaries with an SGW X-4A microscopic melting point apparatus (Shanghai INESA Physico-Optical Instrument Co., Ltd., Shanghai, China) and are uncorrected. NMR spectra were recorded on Bruker Ascend 500, 600, or 800 NMR spectrometers (Bruker Switzerland AG, Fällanden, Switzerland) using CDCl_3_, DMSO-*d*_6_, or CD_3_OD as solvent and TMS (for ^1^H NMR) or the known carbon chemical shifts in deuterated solvents (for ^13^C NMR) as internal standards. High-resolution mass spectra (HR-MS) were determined with a Thermo Scientific Exactive Plus mass spectrometer (Thermo Fisher Scientific, Bremen, Germany) using electrospray ionization (ESI) and Orbitrap techniques. Unless stated otherwise, evaporation on a rotary evaporator was conducted at temperatures below 40 °C.

The copies of ^1^H NMR, ^13^C NMR, ^19^F NMR, ^31^P NMR and HR-MS spectra of the synthesized intermediates and target compounds can be seen in Supplementary Materials.

Synthesis of *tert*-butyl 4-benzyl-4-hydroxypiperidine-1-carboxylate (**2**). To a stirred solution of **1** (3.00 g, 15.1 mmol) in dried THF (30 mL), cooled at 0 °C under N_2_, a solution of BnMgCl in THF(11.3 mL, 18.1 mmol, 1.6 M in THF) was added dropwise. After addition, the reaction mixture was stirred at room temperature overnight. TLC analysis indicated completion of the reaction, after which the mixture was poured into ice–water (50 mL). The resulting mixture was extracted with EtOAc (100 mL × 3), and the combined extracts were washed successively with 0.1 M HCl (100 mL) and brine (100 mL), dried (MgSO_4_), and evaporated on a rotary evaporator to afford a residue, which was purified by column chromatography (EtOAc/PE = 1/3, by *v*/*v*) to give **2** as a pale yellow oil, 2.10 g (48%). ^1^H NMR (500 MHz, CDCl_3_) *δ* 7.34–7.31 (m, 2H), 7.28–7.25 (m, 1H), 7.20–7.18 (m, 2H), 3.87–3.83 (m, 2H), 3.12–3.07 (m, 2H), 2.76 (s, 2H), 1.63–1.57 (m, 2H), 1.51–1.49 (m, 2H), 1.46 (s, 9H). The ^1^H NMR data were consistent with those reported in the literature [28].

Synthesis of 4-benzylpiperidin-4-ol trifluoroacetic acid salt (**3·TFA**). To a stirred solution of **2** (6.30 g, 21.6 mmol) in CH_2_Cl_2_ (63 mL), cooled to 0 °C, TFA was added (31.5 mL). After addition, the reaction mixture was stirred at room temperature overnight. TLC analysis indicated completion of the reaction, and the mixture was evaporated on a rotary evaporator to dryness to give crude **3** as the trifluoroacetic acid salt. The crude **3** was used directly in the next step without further purification or characterization.

Synthesis of *tert*-butyl 2-chloronicotinate (**5**). To a stirred solution of **4** (10.00 g, 63.5 mmol) in dried CH_2_Cl_2_ (100 mL) in a 250 mL round-bottomed flask equipped with a drying tube fitted with anhydrous CaCl_2_ and cooled at 0 °C, (COCl)_2_ (16.1 g, 127 mmol) was added dropwise, followed by addition of DMF (three drops). The reaction mixture was thus stirred at room temperature until completion, as indicated by TLC analysis (typically within 2–3 h). The reaction mixture was evaporated on a rotary evaporator to remove all volatiles, and the residue thus obtained was dissolved in dried THF (100 mL). The resulting solution was cooled to −10 °C, and a solution of *t*-BuOK in THF (152.4 mL, 152.4 mmol, 1.0 M in THF) was added dropwise. After addition, the reaction mixture was stirred at room temperature overnight until TLC analysis indicated completion of the reaction. Saturated aqueous NaHCO_3_ was added dropwise to the reaction mixture (100 mL), and the resulting mixture was extracted with CH_2_Cl_2_ (150 mL × 3). The combined extracts were washed with brine (100 mL), dried (MgSO_4_), and evaporated on a rotary evaporator to afford a residue, which was purified by column chromatography (EtOAc/PE = 1/5, by *v*/*v*) to give **5** as a pale yellow oil, 8.20 g (60%). ^1^H NMR (500 MHz, CDCl_3_) *δ* 8.48 (dd, *J* = 2.3 Hz and 4.8 Hz, 1H), 8.07 (dd, *J* = 2.0 Hz and 7.5 Hz, 1H), 7.34–7.31 (m, 1H), 1.62 (s, 9H). The ^1^H NMR data were consistent with those reported in the literature [29].

Synthesis of *tert*-butyl [2,4′-bipyridine]-3-carboxylate (**6**). A mixture of **5** (15.00 g, 70.2 mmol), pyridine-4-boronic acid (10.34 g, 84.2 mmol), Pd(PPh_3_)_4_ (2.43 g, 2.10 mmol), and Na_2_CO_3_ (23.2 g, 210.6 mmol) in a mixed solvent of DME (100 mL) and water (100 mL) was refluxed under N_2_ overnight until TLC analysis indicated completion of the reaction. After cooling to room temperature, the reaction mixture was diluted with water (100 ml) and extracted with EtOAc (150 mL × 3). The combined extracts were washed with brine (100 mL), dried (MgSO_4_), and evaporated on a rotary evaporator to afford a residue, which was purified by column chromatography (EtOAc/PE = 1/1, by *v*/*v*) to give **6** as a pale yellow solid, 9.00 g (50%). m.p. 78.1–79.9 °C. ^1^H NMR (500 MHz, CDCl_3_) *δ* 8.77 (dd, *J* = 1.8 Hz and 4.8 Hz, 1H), 8.72 (dd, *J* = 1.8 Hz and 4.3 Hz, 2H), 8.17 (dd, *J* = 1.5 Hz and 8.0 Hz, 1H), 7.44 (dd, *J* = 1.5 Hz and 8.0 Hz, 2H), 7.42 (t, *J* = 4.0 Hz, 1H), 1.32 (s, 9H); ^13^C NMR (151 MHz, CDCl_3_) *δ* 165.78, 156.09, 150.90, 149.26, 148.10, 137.96, 128.49, 123.16, 122.54, 82.47, 27.28; ESI-HR-MS: (*m*/*z*) calcd. for C_15_H_17_N_2_O_2_ ([M+H]^+^) 257.1285, found 257.1289.

Synthesis of [2,4′-bipyridine]-3-carboxylic acid trifluoroacetic acid salt (**7·TFA**). Following the procedure for the synthesis of **3·TFA** from **2**, **7·TFA** was synthesized from **6** (5.00 g, 19.5 mmol) in CH_2_Cl_2_ (15 mL) and TFA (25 mL) at room temperature. After evaporation of all volatiles on a rotary evaporator, the residue was triturated with THF (30 mL) to afford **7·TFA** (4.92 g). The sample was used directly in the next step without characterization.

Synthesis of *tert*-butyl 2-(4-nitrophenyl)nicotinate (**8**). Following the procedure for the synthesis of **6** from **5** and pyridine-4-boronic acid, **8** was prepared from **5** (2.00 g, 9.36 mmol) and 4-nitrophenylboronic acid (1.87 g, 11.2 mmol) in the presence of Pd(PPh_3_)_4_ (0.33 g, 0.281 mmol) and Na_2_CO_3_ (2.98 g, 28.1 mmol) in a refluxing mixture of DME/H_2_O (20 mL/20 mL) under N_2_. Product **8** was isolated and purified by column chromatography (EtOAc/PE = 1/5, by *v*/*v*). Pale yellow solid, 2.42 g (86%). m.p. 112.9–113.8 °C. ^1^H NMR (500 MHz, CDCl_3_) *δ* 8.78 (dd, *J* = 1.5 Hz and 5.0 Hz, 1H), 8.32 (d, *J* = 8.5 Hz, 1H), 8.19 (dd, *J* = 1.8 Hz and 7.8 Hz, 1H), 7.70 (d, *J* = 8.5 Hz, 1H), 7.44 (dd, *J* = 4.8 Hz and 7.8 Hz, 1H), 1.34 (s, 9H); ^13^C NMR (151 MHz, CDCl_3_) *δ* 166.16, 156.76, 151.27, 147.86, 147.16, 138.43, 129.91, 128.91, 123.35, 122.88, 83.00, 27.77; ESI-HR-MS: (*m*/*z*) calcd. for C_16_H_17_N_2_O_4_ ([M+H]^+^) 301.1183, found 301.1182.

Synthesis of methyl 2-(4-nitrophenyl)nicotinate (**9**). To a stirred solution of **8** (1.00 g, 3.33 mmol) in CH_2_Cl_2_ (3 mL), cooled to 0 °C, TFA was added dropwise (5 mL). After addition, the reaction mixture was stirred at room temperature overnight. TLC analysis indicated completion of the reaction, and the mixture was subsequently evaporated to dryness on a rotary evaporator. The residue was dissolved in MeOH (10 mL) and the resulting mixture was cooled to 0 °C, followed by dropwise addition of SOCl_2_ (1.3 mL). The reaction mixture was refluxed under N_2_ until completion, as indicated by TLC analysis (typically within 4 h). After cooling to room temperature, the reaction mixture was poured into saturated aqueous NaHCO_3_ (50 mL) and extracted with EtOAc (50 mL × 3). The combined extracts were washed with brine, dried (MgSO_4_), and evaporated on a rotary evaporator to afford a solid, which was triturated with *n*-hexane to give **9**. White solid, 0.65 g (76%). m.p. 113.3–113.9 °C. ^1^H NMR (500 MHz, DMSO-*d*_6_) *δ* 8.87 (dd, *J* = 1.8 Hz and 4.8 Hz, 1H), 8.32–8.30 (m, 2H), 8.27 (dd, *J* = 1.8 Hz and 7.8 Hz, 1H), 7.78–7.76 (m, 2H), 7.63 (dd, *J* = 4.8 Hz and 7.8 Hz, 1H), 3.67 (s, 3H). The ^1^H NMR data were consistent with those reported in the literature [30].

Synthesis of methyl 2-(4-aminophenyl)nicotinate (**10**). A suspension of **9** (0.30 g, 1.16 mmol) and 10% Pd/C (30 mg) in MeOH (3 mL) was subjected to standard procedure of hydrogenation at atmospheric pressure (balloon) and room temperature overnight until TLC analysis indicated completion of the reaction. The reaction mixture was filtered through Celite and the filtrate was poured into water (20 mL). The resulting mixture was extracted with EtOAc (30 mL × 3), and the combined extracts were washed with brine, dried (MgSO_4_), and evaporated on a rotary evaporator to afford a residue, which was purified by column chromatography (EtOAc/PE = 1/3, by *v*/*v*) to give **10**. Pale yellow solid, 0.20 g (75%). m.p. 107.8–108.6 °C. ^1^H NMR (500 MHz, DMSO-*d*_6_) *δ* 8.67 (dd, *J* = 1.8 Hz and 4.8 Hz, 1H), 7.95 (dd, *J* = 1.8 Hz and 7.8 Hz, 1H), 7.31 (dd, *J* = 4.8 Hz and 7.8 Hz, 1H), 7.25 (d, *J* = 8.5 Hz, 2H), 6.60 (d, *J* = 8.5 Hz, 2H), 3.70 (s, 3H); ^13^C NMR (151 MHz, CDCl_3_) *δ* 169.36, 158.25, 151.03, 147.47, 137.67, 129.85, 129.65, 126.26, 120.47, 114.58, 52.31; ESI-HR-MS: (*m*/*z*) calcd. for C_13_H_13_N_2_O_2_ ([M+H]^+^) 229.0972, found 229.0971.

Synthesis of methyl 2-(4-iodophenyl)nicotinate (**11**). To stirred concentrated HCl (2.5 mL) at 0 °C, **10** was added (1.00 g, 4.38 mmol) in portions, followed by the addition of water (2.5 mL). A solution of NaNO_2_ (0.36 g, 5.26 mmol) in a minimum amount of water was added dropwise. After that, stirring was continued for 15 min at this temperature, followed by dropwise addition of a solution of KI (1.45 g, 8.76 mmol) in a minimum amount of water. After addition, the reaction mixture was stirred at room temperature overnight until TLC analysis indicated completion of the reaction, then poured into water (30 mL). The resulting mixture was brought to pH = 8–9 and extracted with EtOAc (50 mL × 3). The combined extracts were washed successively with 10% Na_2_S_2_O_3_ (50 mL) and brine, dried (MgSO_4_), and evaporated on a rotary evaporator to afford a residue, which was purified by column chromatography (EtOAc/PE = 1/3, by *v*/*v*) to give **11**. Pale yellow oil, 0.65 g (44%). ^1^H NMR (500 MHz, DMSO-*d*_6_) *δ* 8.80 (dd, *J* = 1.8 Hz and 4.8 Hz, 1H), 8.15 (dd, *J* = 1.8 Hz and 7.8 Hz, 1H), 7.84–7.81 (m, 2H), 7.53 (dd, *J* = 4.8 Hz and 7.8 Hz, 1H), 7.32–7.29 (m, 2H), 3.69 (s, 3H); ^13^C NMR (151 MHz, CDCl_3_) *δ* 168.04, 157.78, 151.47, 139.50, 138.11, 137.23, 130.38, 126.63, 121.88, 95.16, 52.49; ESI-HR-MS: (*m*/*z*) calcd. for C_13_H_11_INO_2_ ([M+H]^+^) 339.9829, found 339.9833.

Synthesis of methyl 2-(4-((trimethylsilyl)ethynyl)phenyl)nicotinate (**12**). A mixture of **11** (1.50 g, 4.42 mmol), trimethylsilylacetylene (1.41 g, 14.4 mmol), CuI (42.0 mg, 0.719 mmol), PdCl_2_(PPh_3_)_2_ (0.47 g, 0.663 mmol), and Et_3_N (6 mL, 43.5 mmol) in dried THF (15 mL) was stirred at room temperature under N_2_ until TLC analysis indicated the completion of reaction. The reaction mixture was poured into water (30 mL) and extracted with EtOAc (50 mL × 3). The combined extracts were washed with brine, dried (MgSO_4_), and evaporated on a rotary evaporator to afford a residue, which was purified by column chromatography (EtOAc/PE = 1/3, by *v*/*v*) to give **12**. Black oil, 1.00 g (73%). ^1^H NMR (500 MHz, DMSO-*d*_6_) *δ* 8.81 (dd, *J* = 1.8 Hz and 4.8 Hz, 1H), 8.16 (dd, *J* = 1.8 Hz and 7.8 Hz, 1H), 7.55 (s, 1H), 7.54–7.52 (m, 2H), 7.51–7.49 (m, 2H), 3.67 (s, 3H), 0.25 (s, 9H); ^13^C NMR (151 MHz, CDCl_3_) *δ* 168.39, 157.91, 151.37, 139.99, 138.04, 131.78, 128.49, 127.01, 123.58, 121.85, 104.87, 95.51, 52.43, 0.01; ESI-HR-MS: (*m*/*z*) calcd. for C_18_H_20_NO_2_Si ([M+H]^+^) 310.1258, found 310.1263.

Synthesis of methyl 2-(4-ethynylphenyl)nicotinate (**13**). To a stirred solution of **12** (1.00 g, 3.23 mmol) in dried THF (10 mL), cooled to 0 °C under N_2_, a solution of TBAF in THF (3.23 mL, 3.23 mmol, 1.0 M in THF) was added dropwise. After addition, the reaction mixture was stirred at room temperature overnight until TLC analysis indicated completion of the reaction, after which the mixture was poured into water (30 mL). The resulting mixture was extracted with EtOAc (50 mL × 3), and the combined extracts were washed with brine, dried (MgSO_4_), and evaporated on a rotary evaporator to afford a residue, which was purified by column chromatography (EtOAc/PE = 1/3, by *v*/*v*) to give **13**. Dark brown oil, 0.50 g (65%). ^1^H NMR (500 MHz, DMSO-*d*_6_) *δ* 8.81 (dd, *J* = 1.8 Hz and 4.8 Hz, 1H), 8.17 (dd, *J* = 1.8 Hz and 7.8 Hz, 1H), 7.57 (s, 1H), 7.56–7.54 (m, 2H), 7.53–7.51 (m, 2H), 4.30 (s, 1H), 3.68 (s, 3H); ^13^C NMR (151 MHz, CDCl_3_) *δ* 168.35, 158.06, 151.54, 140.49, 138.19, 132.05, 128.66, 127.01, 122.62, 122.00, 83.53, 78.38, 52.56; ESI-HR-MS: (*m*/*z*) calcd. for C_15_H_12_NO_2_ ([M+H]^+^) 238.0863, found 238.0867.

Synthesis of 2-(4-ethynylphenyl)nicotinic acid (**14**). To a stirred solution of **13** (0.40 g, 1.69 mmol) in MeOH (4 mL) a solution of NaOH (0.135 g, 3.38 mmol) in water (0.3 mL) was added. After addition, the reaction mixture was stirred at room temperature overnight until TLC analysis indicated completion of the reaction. The reaction mixture was cooled to 0 °C and concentrated HCl (0.28 mL) was added. A stirred white slurry was obtained, and the precipitate was collected via suction filtration and dried in vacuo at room temperature to give crude **14**. White solid, 0.55 g. The crude product **14** was used directly in the next step without further purification or characterization.

Synthesis of (4-benzyl-4-hydroxypiperidin-1-yl)(2-(4-ethynylphenyl)pyridin-3-yl)methanone (**A1**). To a stirred solution of **14** (0.55 g, 2.36 mmol) in dried THF (5.5 mL), cooled to 0 °C, under N_2_, EDCI (1.13 g, 5.90 mmol), HOBT (24 mg, 0.177 mmol), and DIPEA (1.83 g, 14.2 mmol) were added successively. After addition, a solution of **3·TFA** (0.82 g, 2.83 mmol) in dried THF (2 mL) was added dropwise. After that, the reaction mixture was stirred at room temperature overnight until TLC analysis indicated completion of the reaction, after which the mixture was poured into water (15 mL). The resulting mixture was extracted with EtOAc (20 mL × 3), and the combined extracts were washed with brine, dried (MgSO_4_), and evaporated on a rotary evaporator to afford a residue, which was purified by column chromatography (EtOAc) to give **A1**. White solid, 295 mg (overall 44% from **13** to **A1**). m.p. 155.5–156.9 °C. ^1^H NMR (500 MHz, DMSO-*d*_6_) *δ*: 8.73 (dd, *J* = 1.8 Hz and 4.8 Hz, 1H), 7.81–7.76 (m, 1H), 7.71–7.69 (m, 1H), 7.64–7.55 (m, 3H), 7.48–7.46 (m, 1H), 7.25–7.21 (m, 2H), 7.19–7.15 (m, 2H), 7.08–7.06 (m, 1H), 4.36–4.29 (m, 3H), 4.20–4.17 (m, 0.41H), 3.02–2.96 (m, 0.71H), 2.93–2.87 (m, 1H), 2.81–2.76 (m, 1H), 2.67–2.61 (m, 1H), 2.42–2.39 (m, 0.57H), 2.27–2.24 (m, 0.56H), 1.48–1.28 (m, 2H), 1.19–1.06 (m, 1H), 0.80–0.77 (m, 0.58H), −0.08–−0.15(m, 0.56H); ^13^C NMR (151 MHz, CDCl_3_) *δ* 167.88, 167.63, 153.91, 153.27, 150.18, 150.12, 139.39, 139.14, 136.26, 136.11, 135.75, 135.71, 132.22, 132.19, 131.45, 130.98, 130.40, 130.31, 129.37, 128.46, 128.36, 128.34, 126.86, 126.79, 123.16, 122.86, 122.62, 122.48, 83.28, 82.88, 79.21, 78.81, 69.06, 68.87, 49.13, 49.06, 42.92, 42.38, 37.63, 37.53, 36.55, 36.13, 35.62, 35.43; ESI-HR-MS: (*m*/*z*) calcd. for C_26_H_25_N_2_O_2_ ([M+H]^+^) 397.1911, found 397.1919.

Synthesis of methyl 2-(4-(dimethylphosphoryl)phenyl)nicotinate (**15**). Flask 1 was charged with **11** (1.00 g, 2.95 mmol), HP(=O)Me_2_ (0.23 g, 2.95 mmol), and dioxane (5 mL), and the mixture thus obtained was stirred under N_2_ at room temperature. Flask 2 was charged with Pd_2_(dba)_3_ (0.14 g, 0.148 mmol), XantPhos (0.17 g, 0.295 mmol), and dioxane (5 mL), and the mixture was stirred under N_2_ at room temperature for 10 min and subsequently added to Flask 1. The mixture thus obtained was stirred, followed by addition of Et_3_N (0.36 g, 3.54 mmol). Stirring was continued at room temperature overnight until TLC analysis indicated completion of the reaction. The reaction mixture was poured into water (30 mL) and extracted with EtOAc (50 mL × 3). The combined extracts were washed with brine, dried (MgSO_4_), and evaporated on a rotary evaporator to afford a residue, which was purified by column chromatography (EtOAc) to give **15**. White solid, 295 mg (44%). Pale yellow oil, 0.50 g (59%). ^1^H NMR (500 MHz, DMSO-*d*_6_) *δ* 8.83 (dd, *J* = 1.8 Hz and 4.8 Hz, 1H), 8.20 (dd, *J* = 1.5 Hz and 8.0 Hz, 1H), 7.85 (dd, *J* = 8.3 Hz and 11.3 Hz, 2H), 7.63 (dd, *J* = 2.3 Hz and 8.3 Hz, 2H), 7.56 (dd, *J* = 4.5 Hz and 8.0 Hz, 1H), 3.70 (s, 3H), 1.71 (s, 3H), 1.68 (s, 3H); ^13^C NMR (151 MHz, CDCl_3_) *δ* 167.84, 157.89, 151.63, 143.49 (d, *J* = 3.0 Hz), 138.30, 134.76 (d, *J* = 98.6 Hz), 129.53 (d, *J* = 10.0 Hz), 128.96 (d, *J* = 11.9 Hz), 126.88, 122.30, 52.58, 18.11 (d, *J* = 71.6 Hz); ^31^P NMR (202 MHz, DMSO-*d*_6_) *δ* 32.40; ESI-HR-MS: (*m*/*z*) calcd. for C_15_H_17_NO_3_P ([M+H]^+^) 290.0941, found 290.0938.

Synthesis of 2-(4-(dimethylphosphoryl)phenyl)nicotinic acid (**16**). Following the procedure for the synthesis of **14** from **13**, **16** was prepared from **15** (0.44 g, 1.52 mmol) and NaOH (0.12 g, 3.04 mmol) in MeOH (4.4 mL) and water (0.3 mL). Neutralization with concentrated HCl (0.25 mL) afforded crude **16** (471 mg). The crude **16** sample was used directly in the next step without further purification or characterization.

Synthesis of (4-benzyl-4-hydroxypiperidin-1-yl)(2-(4-(dimethylphosphoryl)phenyl)pyridin-3-yl)methanone (**A2**). Following the procedure for the synthesis of **A1** from **14** and **3·TFA**, **A2** was prepared from **16** (0.40 g, 1.45 mmol) and **3·TFA** (0.63 g, 2.18 mmol) using EDCI (0.70 g, 3.63 mmol), HOBT (15 mg, 0.109 mmol), and DIPEA (1.13 g, 8.72 mmol) in dried THF (4 mL). White foam, 0.12 g (overall 18% from **15** to **A2**). ^1^H NMR (500 MHz, DMSO-*d*_6_) *δ* 8.76–8.74 (m, 1H), 7.95–7.91 (m, 1H), 7.86–7.79 (m, 3H), 7.71–7.70 (m, 1H), 7.51–7.48 (m, 1H), 7.24–7.21 (m, 2H), 7.18–7.15 (m, 2H), 7.05–7.04 (m, 1H), 4.38–4.32 (m, 1H), 4.30–4.08 (m, 1H), 3.06–2.98 (m, 1H), 2.93–2.83 (m, 1.67H), 2.76–2.64 (m, 1H), 2.32–2.24 (m, 1H), 1.73–1.66 (m, 6H), 1.49–1.43 (m, 0.36H), 1.37–1.31 (m, 1H), 1.10–0.99 (m, 1H), 0.91–0.89 (m, 0.61H), 0.06–0.01 (m, 0.53H); ^13^C NMR (151 MHz, CDCl_3_) *δ* 167.79, 167.65, 153.66, 153.18, 150.30, 150.24, 142.51, 142.49, 142.26, 142.24, 136.15, 136.11, 135.94, 135.91, 135.44, 135.26, 134.80, 131.60, 131.36, 130.46, 130.40, 129.89, 129.86, 129.82, 129.79, 129.58, 129.50, 128.96, 128.88, 128.46, 128.37, 126.92, 126.84, 122.92, 122.88, 69.05, 68.66, 49.09, 48.66, 43.14, 42.70, 37.77, 37.66, 36.51, 36.15, 36.07, 35.71, 18.51, 18.47, 18.16, 18.15, 18.03, 18.00, 17.69, 17.67; ^31^P NMR (203 MHz, DMSO-*d*_6_) *δ* 32.39, 32.10; ESI-HR-MS: (*m*/*z*) calcd. for C_26_H_30_N_2_O_3_P ([M+H]^+^) 449.1989, found 449.1989.

Synthesis of methyl 2-((trimethylsilyl)ethynyl)nicotinate (**18**). Following the procedure for the synthesis of **12** from **11**, **18** was prepared from **17** (3.00 g, 13.9 mmol) and trimethylsilylacetylene (4.43 g, 45.1 mmol) using PdCl_2_(PPh_3_)_2_ (1.47 g, 2.09 mmol), CuI (0.13 g, 0.695 mmol), and Et_3_N (12 mL, 87 mmol) in dried THF (30 mL). Purification by column chromatography (EtOAc/PE = 1/5, by *v*/*v*) gave **18**. Black oil, 2.40 g (74%). ^1^H NMR (500 MHz, CDCl_3_) *δ* 8.71–8.70 (m, 1H), 8.22–8.20 (m, 1H), 7.34–7.31 (m, 1H), 3.96 (s, 3H), 0.30 (s, 9H). The ^1^H NMR data were consistent with those reported in the literature [31].

Synthesis of methyl 2-ethynylnicotinate (**19**). Following the procedure for the synthesis of **13** from **12**, **19** was prepared from **18** (2.40 g, 10.3 mmol) using TBAF (10.3 mL, 10.3 mmol, 1.0 M in THF) in dried THF (24 mL). Purification by column chromatography (EtOAc/PE = 1/5, by *v*/*v*) gave **19**. Brown oil, 1.10 g (66%). ^1^H NMR (500 MHz, DMSO-*d*_6_), *δ* 8.74 (dd, *J* = 1.5 Hz and 5.0 Hz, 1H), 8.21 (dd, *J* = 1.5 Hz and 8.0 Hz, 1H), 7.55 (dd, *J* = 5.0 Hz and 8.0 Hz, 1H), 4.55 (s, 1H), 3.87 (s, 3H). The ^1^H NMR data were consistent with those reported in the literature [31].

Synthesis of 2-ethynylnicotinic acid (**20**). Following the procedure for the synthesis of **14** from **13**, **20** was prepared from **19** (0.80 g, 4.96 mmol) using NaOH (0.40 g, 9.92 mmol) in MeOH (8 mL). Yellow solid, 0.72 g. This sample was used directly in the next step without further purification or characterization.

Synthesis of (4-benzyl-4-hydroxypiperidin-1-yl)(2-ethynylpyridin-3-yl)methanone (**A3**). Following the procedure for the synthesis of **A1** from **14** and **3·TFA**, **A3** was prepared from **20** (0.30 g, 2.04 mmol) and **3·TFA** (0.71 g, 2.45 mmol) using EDCI (0.98 g, 5.10 mmol), HOBT (21 mg, 0.109 mmol), and DIPEA (1.58 g, 12.2 mmol) in dried THF (3 mL). White solid, 0.20 g (overall 38% from **19** to **A3**). m.p. 138.2–139.8 °C. ^1^H NMR (500 MHz, DMSO-*d*_6_) *δ* 8.58 (d, *J* = 4.5 Hz, 1H), 7.74 (s, 1H), 7.49–7.46 (m, 1H), 7.28–7.19 (m, 5H), 4.52–4.00 (m, 3H), 3.28–3.22 (m, 0.72H), 3.10–3.01 (m, 2H), 2.69 (s, 2H), 1.66–1.39 (m, 3.47H), 1.32–1.29 (m, 1H); ^13^C NMR (151 MHz, CDCl_3_) *δ* 166.01, 149.97, 138.15, 136.38, 136.14, 135.91, 134.41, 133.98, 130.58, 128.29, 126.63, 123.52, 81.16, 80.34, 69.67, 69.31, 49.92, 49.29, 43.58, 42.99, 37.77, 36.86, 36.32, 36.09, 35.43; ESI-HR-MS: (*m*/*z*) calcd. for C_20_H_21_N_2_O_2_ ([M+H]^+^) 321.1598, found 321.1605.

Synthesis of (4-benzyl-4-hydroxypiperidin-1-yl)(2-chloropyridin-3-yl)methanone (**21**). The corresponding carboxylic acid chloride of **4** was prepared from **4** (3.0 g, 19.0 mmol) following the procedure described above using (COCl)_2_ (4.82 g, 38.0 mmol) and DMF (2 drops) in dried CH_2_Cl_2_ (30 mL). The crude carboxylic acid chloride of **4** was dissolved in dried THF (30 mL), and the resulting mixture was cooled at 0 °C and stirred under N_2_, followed by addition of **3·TFA** (6.05 g, 20.9 mmol) in one portion and Et_3_N (10.6 mL, 76.0 mmol) in a dropwise manner. After addition, the reaction mixture was stirred at room temperature overnight until TLC analysis indicated completion of the reaction, after which the mixture was poured into saturated aqueous NaHCO_3_ (100 mL). The mixture thus obtained was extracted with EtOAc (50 mL × 3), and the combined extracts were washed with brine, dried (MgSO_4_), and evaporated on a rotary evaporator to afford a residue, which was purified by column chromatography (EtOAc/PE = 1/1, by *v*/*v*) to give **21**. White solid, 1.98 g (31%). m.p. 138.4–138.9 °C. ^1^H NMR (500 MHz, DMSO-*d*_6_), *δ* 8.47–8.43 (m, 1H), 7.88–7.81 (m, 1H), 7.52–7.47 (m, 1H), 7.27–7.24 (m, 2H), 7.21–7.17 (m, 3H), 4.54 (d, *J* = 5.5 Hz, 1H), 4.26–4.22 (m, 1H), 3.30–3.21 (m, 1H), 3.13–3.02 (m, 2H), 2.71–2.70 (m, 2H), 1.58–1.40 (m, 3H), 1.36–1.29 (m, 1H). The ^1^H NMR data were consistent with those reported in the literature [13].

Synthesis of 4-(3-(4-benzyl-4-hydroxypiperidine-1-carbonyl)pyridin-2-yl)benzonitrile (**24**). Following the procedure for the synthesis of **6** from **5** and pyridine-4-boronic acid, **24** was prepared from **21** (1.00 g, 9.36 mmol) and **22** (0.53 g, 3.63 mmol) using Pd(PPh_3_)_4_ (0.11 g, 0.093 mmol) and Na_2_CO_3_ (0.96 g, 9.06 mmol) in DME/H_2_O (10 mL/10 mL). Purification by column chromatography (EtOAc/PE = 1/1, by *v*/*v*) gave **24**. White solid, 0.72 g (61%). m.p. 188.8–190.4 °C. ^1^H NMR (500 MHz, DMSO-*d*_6_), *δ* 8.77–8.75 (m, 1H), 7.97–7.93 (m, 2H), 7.86–7.75 (m, 3H), 7.54–7.51 (m, 1H), 7.25–7.15 (m, 4H), 7.06–7.05 (m, 1H), 4.41–4.39 (m, 1H), 4.29–4.15 (m, 1H), 3.09–3.03 (m, 0.73H), 2.96–2.91 (m, 1H), 2.85–2.83 (m, 1H), 2.72–2.64 (m, 1.43H), 2.44–2.32 (m, 1H), 1.51–1.31 (m, 2H), 1.13–1.05 (m, 1H), 0.93–0.90 (m, 0.58H), 0.06-0.03 (m, 0.54H); ^13^C NMR (151 MHz, CDCl_3_) *δ* 167.38, 167.26, 152.68, 152.13, 150.28, 150.18, 143.25, 143.08, 136.03, 135.98, 135.56, 135.43, 132.14, 132.03, 131.58, 131.25, 130.41, 130.39, 130.31, 130.19, 129.77, 129.14, 128.31, 126.91, 126.82, 123.19, 123.15, 118.42, 118.26, 112.58, 68.95, 68.74, 49.26, 49.03, 42.94, 42.43, 37.53, 37.50, 36.46, 36.01, 35.74, 35.44; ESI-HR-MS: (*m*/*z*) calcd. for C_25_H_24_N_3_O_2_ ([M+H]^+^) 398.1863, found 398.1863.

Synthesis of 4-(3-(4-benzyl-4-hydroxypiperidine-1-carbonyl)pyridin-2-yl)benzothioamide (**A4**). A mixture of **24** (0.50 g, 1.26 mmol), (NH_4_)_2_S (95 mg, 1.39 mmol), and Et_3_N (0.14 g, 1.39 mmol) in pyridine (5 mL) was stirred at 50 °C under N_2_ overnight until TLC analysis indicated completion of the reaction. After cooling to room temperature, the reaction mixture was poured into water (15 mL) and extracted with EtOAc (20 mL × 3). The combined extracts were washed with brine, dried (MgSO_4_), and evaporated on a rotary evaporator to afford a residue, which was purified by column chromatography (EtOAc/PE = 1/1, by *v*/*v*) to give **A4**. Yellow solid, 0.47 g (87%). m.p. 136.2–137.5 °C. ^1^H NMR (500 MHz, DMSO-*d*_6_) *δ* 9.99–9.93 (m, 1H), 9.65–9.56 (m, 1H), 8.74–8.73 (m, 1H), 8.07–7.95 (m, 2H), 7.81–7.77 (m, 1H), 7.71–7.60 (m, 2H), 7.49–7.47 (m, 1H), 7.23–7.04 (m, 5H), 4.37–4.18 (m, 2H), 3.02–2.96 (m, 1H), 2.91–2.85 (m, 0.67H), 2.82–2.77 (m, 1H), 2.64 (m, 0.63H), 2.36–2.26 (m, 1H), 1.47–1.27 (m, 2H), 1.26–1.22 (m, 0.66H), 1.17–1.10 (m, 1H), 0.87–0.84 (m, 0.60H), 0.79–0.76 (m, 0.64H), −0.08–−0.14 (m, 0.67H); ^13^C NMR (151 MHz, CDCl_3_) *δ* 201.86, 201.73, 167.98, 167.71, 153.60, 153.15, 150.33, 150.24, 141.82, 141.74, 140.30, 139.71, 136.42, 136.24, 135.83, 135.73, 131.61, 131.16, 130.59, 130.57, 129.20, 128.54, 128.45, 127.47, 127.36, 126.96, 126.88, 123.05, 122.94, 69.47, 68.97, 48.89, 48.74, 43.12, 42.74, 37.89, 37.76, 36.50, 36.08, 35.74, 35.50, 31.66, 22.73, 14.21; ESI-HR-MS: (*m*/*z*) calcd for C_25_H_26_N_3_O_2_S ([M+H]^+^) 432.1740, found 432.1741.

Synthesis of (2-(1H-benzo[d]imidazol-5-yl)pyridin-3-yl)(4-benzyl-4-hydroxypiperidin-1-yl)methanone (**A5**). Following the procedure for the synthesis of **6** from **5** and pyridine-4-boronic acid, **A5** was prepared from **21** (0.56 g, 1.69 mmol) and **23** (0.50 g, 2.03 mmol) using Pd(PPh_3_)_4_ (98 mg, 0.085 mmol) and Na_2_CO_3_ (0.54 g, 5.07 mmol) in DME/H_2_O (6 mL/6 mL). Purification by column chromatography (EtOAc) gave **A5**. White solid, 70 mg (10%). m.p. 137.8–138.9 °C. ^1^H NMR (500 MHz, DMSO-*d*_6_) *δ* 12.71 (s, 1H), 8.72–8.70 (m, 1H), 8.40–8.26 (m, 1H), 7.93–7.62 (m, 4H), 7.43–7.40 (m, 1H), 7.23–7.10 (m, 4H), 6.74–6.72 (m, 1H), 4.35–4.16 (m, 2H), 2.95–2.70 (m, 3.40H), 2.64–2.54 (m, 1H), 1.93–1.90 (m, 0.60H), 1.59–1.57 (m, 0.68H), 1.44–1.19 (m, 1.68H), 1.04–0.95 (m, 1H), 0.47–0.45 (m, 0.64H), −0.72–−0.78 (m, 0.69H); ^13^C NMR (151 MHz, CD_3_OD + CDCl_3_) *δ* 168.43, 168.21, 155.31, 154.49, 149.77, 142.57, 142.21, 136.57, 136.29, 136.09, 135.65, 133.35, 133.21, 131.41, 131.02, 130.35, 130.05, 127.99, 127.81, 126.46, 126.33, 124.01, 123.22, 122.19, 122.07, 116.34, 115.86, 115.56, 114.92, 68.81, 68.40, 48.79, 43.00, 42.54, 37.66, 37.52, 36.06, 35.59, 35.44, 34.66; ESI-HR-MS: (*m*/*z*) calcd. for C_25_H_25_N_4_O_2_ ([M+H]^+^) 413.1972, found 413.1981.

Synthesis of tert-butyl 2-(4-(dimethylcarbamoyl)phenyl)nicotinate (**26**). Following the procedure for the synthesis of **6** from **5** and pyridine-4-boronic acid, **26** was prepared from **5** (2.00 g, 9.36 mmol) and **25** (2.16 g, 11.2 mmol) using Pd(PPh_3_)_4_ (0.32 g, 0.281 mmol) and Na_2_CO_3_ (2.98 g, 28.1 mmol) in DME/H_2_O (20 mL/20 mL). Purification by column chromatography (EtOAc/PE = 1/5, by *v*/*v*) gave **A5**. White solid, 2.82 g (76%). m.p. 85.2–86.4 °C. ^1^H NMR (500 MHz, DMSO-*d*_6_) *δ* 8.78 (dd, *J* = 1.8 Hz and 4.8 Hz, 1H), 8.12 (dd, *J* = 1.8 Hz and 7.8 Hz, 1H), 7.54–7.49 (m, 5H), 3.01 (s, 3H), 2.96 (s, 3H), 1.28 (s, 9H); ^13^C NMR (151 MHz, CDCl_3_) *δ* 170.64, 166.42, 157.30, 150.43, 141.44, 137.49, 135.96, 128.52, 128.34, 126.56, 121.62, 81.94, 39.12, 34.95, 27.19; ESI-HR-MS: (*m*/*z*) calcd. for C_19_H_23_N_2_O_3_ ([M+H]^+^) 327.1703, found 327.1702.

Synthesis of tert-butyl 2-(4-(dimethylcarbamothioyl)phenyl)nicotinate (**27**). A mixture of **26** (2.00 g, 6.13 mmol) and Lawesson reagent (1.49 g, 11.2 mmol) in toluene (20 mL) was refluxed overnight under N_2_ until TLC analysis indicated completion of the reaction. After cooling to room temperature, the reaction mixture was poured into water (50 mL) and extracted with EtOAc (50 mL × 3). The combined extracts were washed with brine, dried (MgSO_4_), and evaporated on a rotary evaporator to afford a residue, which was purified by column chromatography (EtOAc) to give **27**. Pale yellow solid, 1.20 g (57%). m.p. 96.8–97.9 °C. ^1^H NMR (500 MHz, DMSO-*d*_6_) *δ* 8.77 (dd, *J* = 1.8 Hz and 4.8 Hz, 1H), 8.11 (dd, *J* = 1.8 Hz and 7.8 Hz, 1H), 7.53–7.48 (m, 3H), 7.41–7.39 (m, 2H), 3.53 (s, 3H), 3.20 (s, 3H), 1.30 (s, 9H); ^13^C NMR (151 MHz, CDCl_3_) *δ* 200.32, 166.45, 157.19, 143.35, 139.92, 138.27, 128.91, 128.56, 125.55, 122.03, 82.53, 44.05, 43.08, 27.42; ESI-HR-MS: (*m*/*z*) calcd. for C_19_H_23_N_2_O_2_S ([M+H]^+^) 343.1475, found 343.1474.

Synthesis of 4-(3-(4-benzyl-4-hydroxypiperidine-1-carbonyl)pyridin-2-yl)-N,N-dimethylbenzothioamide (**A6**). To a stirred solution of **27** (2.00 g, 5.84 mmol) in CH_2_Cl_2_ (6 mL), cooled to 0 °C, TFA was added dropwise (10 mL). After addition, the reaction mixture was stirred at room temperature overnight until TLC analysis indicated completion of the reaction, after which the mixture was evaporated on a rotary evaporator to dryness. The residue thus obtained was dissolved in dried THF (20 mL), followed by addition of EDCI (2.80 g, 14.6 mmol), HOBT (59 mg, 0.438 mmol), and DIPEA (4.52 g, 35.0 mmol). The resulting mixture was stirred at 0 °C under N_2_, and a solution of **3·TFA** (2.03 g, 7.10 mmol) in dried THF (3 mL) was added dropwise. After addition, the reaction mixture was stirred at room temperature overnight until TLC analysis indicated completion of the reaction, after which the mixture was poured into water (30 mL). The mixture thus obtained was extracted with EtOAc (50 mL × 3), and the combined extracts were washed with brine, dried (MgSO_4_), and evaporated on a rotary evaporator to afford a residue, which was purified by column chromatography (EtOAc) to give **A6**. Yellow solid, 1.21 g (overall 45% from **27** to **A6**). m.p. 89.4–91.3 °C. ^1^H NMR (500 MHz, DMSO-*d*_6_) *δ* 8.73–8.72 (m, 1H), 7.81–7.76 (m, 1H), 7.70–7.58 (m, 2H), 7.49–7.35 (m, 3H), 7.25–7.18 (m, 3H), 7.17–7.11 (m, 2H), 4.31–4.09 (m, 2H), 3.60–3.51 (m, 3H), 3.21–3.13 (m, 3H), 3.06–2.96 (m, 1.43H), 2.94–2.81 (m, 2H), 2.66–2.63 (m, 1H), 2.47–2.46 (m, 0.61H), 1.47–1.29 (m, 2H), 1.12–1.00 (m, 1H), 0.94–0.91 (m, 0.52H), 0.26–0.20 (m, 0.60H). ^13^C NMR (151 MHz, CDCl_3_) *δ* 201.60, 201.44, 167.90, 167.64, 153.50, 153.05, 150.24, 150.14, 141.56, 141.45, 140.20, 139.67, 136.40, 136.21, 135.92, 135.82, 131.44, 131.03, 130.57, 130.54, 129.03, 128.40, 128.29, 127.48, 127.41, 126.79, 126.72, 123.02, 122.92, 69.43, 68.94, 48.80, 48.68, 43.06, 42.68, 37.85, 37.73, 36.29, 35.89, 35.54, 35.36, 31.58, 22.66, 14.15; ESI-HR-MS: (*m*/*z*) calcd. for C_27_H_30_N_3_O_2_S ([M+H]^+^) 460.2053, found 460.2055.

Synthesis of (4-bromophenyl)(tert-butyl)sulfane (**29**). To a stirred solution of **28** (5.00 g, 26.5 mmol) and *t*-BuCl (3.93 g, 56 mmol) in MeCN (50 mL) at room temperature under N_2_, anhydrous AlCl_3_ (0.18 g, 1.33 mmol) was added in portions. After addition, the reaction mixture was stirred at room temperature until completion, as indicated by TLC analysis (typically within 2 h). The reaction mixture was slowly poured into ice–water (150 mL) and extracted with *n*-hexane (100 mL × 3). The combined extracts were washed with brine, dried (MgSO_4_), and evaporated on a rotary evaporator to give a residue, which was purified by column chromatography (PE) to give **27**. Pale yellow oil, 5.19 g (80%). ^1^H NMR (500 MHz, DMSO-*d*_6_) *δ* 7.57 (dd, *J* = 1.5 Hz and 8.5 Hz, 1H), 7.41 (dd, *J* = 1.3 Hz and 8.3 Hz, 1H), 1.22 (s, 9H). The ^1^H NMR data were consistent with those reported in the literature [32].

Synthesis of (4-(*tert*-butylthio)phenyl)boronic acid (**30**). To a stirred solution of **29** (6.48 g, 26.5 mmol) in dried THF (65 mL), cooled to −78 °C under N_2_, a solution of *n*-BuLi in *n*-hexane (18.3 mL, 29.2 mmol, 1.6 M in *n*-hexane) was added dropwise. After addition, the reaction mixture was stirred at this temperature for an additional 1 h, followed by dropwise addition of B(OMe)_3_ (5.51 g, 53.0 mmol). After addition, stirring was continued at this temperature for an additional 1 h and then at room temperature until TLC analysis indicated completion of the reaction. The reaction mixture was poured into ice–water (100 mL) and extracted with EtOAc (100 mL × 3). The combined extracts were washed with brine, dried (MgSO_4_), and evaporated on a rotary evaporator to afford a residue, which was purified by column chromatography (EtOAc/PE = 1/3, by *v*/*v*) to give **30**. White solid, 4.00 g (72%). m.p. 172.3–173.2 °C. ^1^H NMR (500 MHz, DMSO-*d*_6_) *δ* 8.13 (s, 2H), 7.78 (d, *J* = 8.0 Hz, 2H), 7.45 (d, *J* = 8.0 Hz, 2H), 1.24 (s, 9H). The ^1^H NMR data were consistent with those reported in the literature [33].

Synthesis of tert-butyl 2-(4-(tert-butylthio)phenyl)nicotinate (**31**). Following the procedure for the synthesis of **6** from **5** and pyridine-4-boronic acid, **31** was prepared from **30** (0.20 g, 0.95 mmol) and **5** (0.24 g, 1.14 mmol) using Pd(PPh_3_)_4_ (55 mg, 0.048 mmol) and K_2_CO_3_ (0.39 g, 2.85 mmol) in refluxing dioxane/H_2_O (2 mL/2 mL) under N_2_. Purification by column chromatography (EtOAc) gave **31**. White solid, 0.26 g (80%). m.p. 72.3–74.2 °C. ^1^H NMR (500 MHz, DMSO-*d*_6_) *δ* 8.76 (dd, *J* = 1.5 Hz and 5.0 Hz, 1H), 8.10 (dd, *J* = 1.8 Hz and 7.8 Hz, 1H), 7.59 (dd, *J* = 2.0 Hz and 6.5 Hz, 2H), 7.53–7.49 (m, 3H), 1.29 (s, 9H), 1.26 (s, 9H); ^13^C NMR (126 MHz, CDCl_3_) *δ* 167.14, 158.03, 150.84, 141.08, 137.93, 137.13, 133.21, 129.05, 128.90, 121.89, 82.45, 46.22, 31.02, 27.63; ESI-HR-MS: (*m*/*z*) calcd. for C_20_H_26_NO_2_S ([M+H]^+^) 344.1679, found 344.1688.

Synthesis of (4-benzyl-4-hydroxypiperidin-1-yl)(2-(4-(tert-butylthio)phenyl)pyridin-3-yl)methanone (**A7**). Following the procedure for the synthesis of **A6** from **27**, **A7** was prepared from **31**. Thus, **31** (2.00 g, 5.82 mmol) was first treated with TFA (10 mL) in CH_2_Cl_2_ (6 mL) at room temperature to give the corresponding carboxylic acid, which was then reacted with **3·TFA** (2.02 g, 6.98 mmol) using EDCI (2.80 g, 14.6 mmol), HOBT (59 mg, 0.437 mmol), and DIPEA (4.51 g, 34.9 mmol) in dried THF (20 mL). Purification by column chromatography (EtOAc) gave **A7**. White solid, 1.55 g (overall 58% from **31** to **A7**). ^1^H NMR (500 MHz, DMSO-*d*_6_) *δ* 8.73–8.72 (m, 1H), 7.81–7.76 (m, 1H), 7.72–7.70 (m, 1H), 7.64–7.55 (m, 3H), 7.49–7.46 (m, 1H), 7.24–7.21 (m, 2H), 7.18–7.13 (m, 2H), 7.03–7.02 (m, 1H), 4.33–4.16 (m, 2H), 3.05–2.99 (m, 0.65H), 2.95–2.87 (m, 1H), 2.81–2.71 (m, 1H), 2.62–2.55 (m, 1H), 2.37–2.23 (m, 1H), 1.46–1.36 (m, 1H), 1.33–1.26 (m, 10H), 1.23–1.16 (m, 0.41H), 1.12–1.06 (m,1H), 0.91–0.87 (m, 0.59H), 0.11–0.05 (m, 0.60H); ^13^C NMR (151 MHz, DMSO-*d*_6_) *δ* 166.80, 166.73, 153.07, 152.53, 149.89, 149.84, 139.27, 139.23, 137.31, 136.94, 136.64, 135.98, 135.82, 133.30, 132.97, 131.36, 131.12, 130.51, 130.33, 129.00, 128.42, 127.54, 127.52, 125.89, 125.84, 122.71, 68.13, 67.76, 48.43, 48.31, 46.07, 45.97, 42.52, 42.10, 40.06, 37.00, 36.93, 35.89, 35.38, 35.31, 30.73; ESI-HR-MS: (*m*/*z*) calcd. for C_28_H_33_N_2_O_2_S ([M+H]^+^) 461.2257, found 461.2267.

Synthesis of tert-butyl 2-(4-aminophenyl)nicotinate (**32**). Following the procedure for the synthesis of **10** from **9**, **32** was prepared from **8** (6.30 g, 21.0 mmol) under standard hydrogenolysis condition using 10% Pd/C (0.63 g) in MeOH (63 mL) at atmospheric pressure and room temperature. Purification by column chromatography (EtOAc/PE = 2/1, by *v*/*v*) gave **32**. White solid, 4.00 g (71%). m.p. 129.4–130.7 °C. ^1^H NMR (500 MHz, DMSO-*d*_6_) *δ* 8.64 (dd, *J* = 1.8 Hz and 4.8 Hz, 1H), 7.90 (dd, *J* = 1.8 Hz and 7.8 Hz, 1H), 7.30 (dd, *J* = 4.8 Hz and 7.8 Hz, 1H), 7.24 (dd, *J* = 2.0 Hz and 6.5 Hz, 2H), 6.61 (dd, *J* = 2.0 Hz and 6.5 Hz, 2H), 5.39 (s, 2H), 1.37 (s, 9H); ^13^C NMR (151 MHz, CDCl_3_) *δ* 167.77, 158.12, 150.24, 147.31, 137.31, 129.89, 129.84, 128.16, 120.38, 114.23, 81.83, 27.49; ESI-HR-MS: (*m*/*z*) calcd. for C_16_H_19_N_2_O_2_ ([M+H]^+^) 271.1441, found 271.1446.

Synthesis of tert-butyl 2-(4-isothiocyanatophenyl)nicotinate (**33**). To a stirred solution of **32** (1.00 g, 3.70 mmol) and Et_3_N (0.75 g, 7.41 mmol) in CH_2_Cl_2_ (10 mL), cooled to 0 °C under N_2_, CSCl_2_ (0.85 g, 7.41 mmol) was added in a dropwise manner. After addition, the reaction mixture was stirred at room temperature until completion, as indicated by TLC analysis (typically within 1 h), and then poured into ice–water (15 mL). The resulting mixture was brought to pH = 7–8 with saturated aqueous NaHCO_3_ and extracted with EtOAc (50 mL × 3). The combined extracts were washed with brine, dried (MgSO_4_), and evaporated on a rotary evaporator to afford a residue, which was purified by column chromatography (EtOAc/PE = 1/4, by *v*/*v*) to give **33**. Pale yellow solid, 0.90 g (78%). m.p. 67.4–69.8 °C. ^1^H NMR (500 MHz, DMSO-*d*_6_) *δ* 8.77 (dd, *J* = 1.8 Hz and 4.8 Hz, 1H), 8.12 (dd, *J* = 1.8 Hz and 7.8 Hz, 1H), 7.56–7.51 (m, 5H), 1.31 (s, 9H); ^13^C NMR (151 MHz, DMSO-*d*_6_) *δ* 166.24, 155.99, 151.03, 139.46, 137.70, 134.18, 130.14, 130.11, 128.26, 125.57, 122.50, 81.96, 27.21; ESI-HR-MS: (*m*/*z*) calcd. for C_17_H_17_N_2_O_2_S ([M+H]^+^) 313.1005, found 313.1011.

Synthesis of tert-butyl 2-(4-(3,3-dimethylthioureido)phenyl)nicotinate (**34**). To a stirred solution of **33** (1.16 g, 3.71 mmol) and Et_3_N (1.12 g, 11.1 mmol) in dried THF, cooled to 0 °C under N_2_, Me_2_NH·HCl was added in portions. After addition, the reaction mixture was stirred at room temperature until completion, as indicated by TLC analysis (typically within 1 h), and then poured into ice–water (50 mL). The resulting mixture was extracted with EtOAc (50 mL × 3). The combined extracts were washed with brine, dried (MgSO_4_), and evaporated on a rotary evaporator to afford a residue, which was purified by column chromatography (EtOAc/PE = 2/1, by *v*/*v*) to give **34**. White solid, 1.08 g (82%). ^1^H NMR (500 MHz, DMSO-*d*_6_) *δ* 9.13 (s, 1H), 8.74 (d, *J* = 5.0 Hz, 1H), 8.04 (d, *J* = 7.5 Hz), 7.46–7.42 (m, 5H), 3.30 (s, 6H), 1.34 (s, 9H); ^13^C NMR (151 MHz, DMSO-*d*_6_) *δ* 181.22, 167.02, 156.74, 150.71, 141.59, 137.26, 135.46, 128.43, 128.16, 124.59, 121.73, 81.90, 40.97, 27.27; ESI-HR-MS: (*m*/*z*) calcd. for C_19_H_24_N_3_O_2_S ([M+H]^+^) 358.1584, found 358.1591.

Synthesis of 2-(4-(3,3-dimethylthioureido)phenyl)nicotinic acid hydrochloric acid salt (**35**). To a stirred solution of **34** (1.46 g, 4.08 mmol) in CH_2_Cl_2_ (4.5 mL), cooled to 0 °C, TFA (7.5 mL) was added in a dropwise manner. After addition, the reaction mixture was stirred at room temperature overnight until TLC analysis indicated completion of the reaction, after which the mixture was evaporated on a rotary evaporator to give a residue. Another flask, cooled to 0 °C, was charged with MeOH (10 mL), to which SOCl_2_ (2 mL) was added dropwise. The mixture thus obtained was stirred at this temperature for another 30 min, followed by the slow addition of the residue prepared above. The resulting mixture was stirred at room temperature for another 2 h and subsequently evaporated on a rotary evaporator to give **35** as a hydrochloric acid salt. The product was obtained as a pale yellow solid. This sample was used directly in the next step without further purification or characterization.

Synthesis of 3-(4-(3-(4-benzyl-4-hydroxypiperidine-1-carbonyl)pyridin-2-yl)phenyl)-1,1-dimethylthiourea (**A8**). Following the procedure for the synthesis of **A1** from **14** and **3·TFA**, **A8** was prepared from **35** (1.60 g, 4.74 mmol) and **3·TFA** (1.09 g, 5.69 mmol) using EDCI (2.28 g, 11.9 mmol), HOBT (48 mg, 0.356 mmol), and DIPEA (3.67 g, 28.4 mmol) in dried THF (16 mL). Purification by column chromatography (EtOAc) gave **A8**. White solid, 1.40 g (62%). m.p. 119.6–122.3 °C. ^1^H NMR (500 MHz, DMSO-*d*_6_) *δ* 9.19–9.13 (m, 2H), 8.70–8.68 (m, 1H), 7.76–7.72 (m, 1H), 7.63–7.61 (m, 1H), 7.54–7.51 (m, 2H), 7.42–7.40 (m, 1H), 7.23–7.07 (m, 5H), 4.29–4.27 (m, 1H), 4.22–4.17 (m, 1H), 3.30–3.29 (m, 6H), 3.02–2.73 (m, 3H), 2.45–2.44 (m, 1H), 1.49–1.06 (m, 4H), 0.87–0.82 (m, 1.41H), 0.28–0.22 (m, 0.61H); ^13^C NMR (126 MHz, DMSO-*d*_6_) *δ* 181.10, 181.02, 167.12, 167.07, 153.42, 153.12, 149.72, 149.66, 142.18, 141.74, 137.41, 137.34, 135.90, 135.61, 134.51, 134.18, 131.04, 130.71, 130.59, 130.55, 128.22, 127.80, 127.58, 127.37, 125.88, 125.67, 124.56, 124.08, 122.07, 122.03, 68.36, 67.95, 48.22, 48.11, 42.51, 42.22, 41.00, 37.03, 36.90, 35.97, 35.53, 35.27, 35.23, 30.96, 22.07, 13.97; ESI-HR-MS: (*m*/*z*) calcd. for C_27_H_31_N_4_O_2_S ([M+H]^+^) 475.2162, found 475.2174.

Synthesis of 3-fluoro-2,4′-bipyridine (**37**). Following the procedure for the synthesis of **6** from **5** and pyridine-4-boronic acid, **37** was prepared from **36** (2.00 g, 11.4 mmol) and pyridine-4-boronic acid (1.54 g, 12.5 mmol) using Pd(PPh_3_)_4_ (0.66 g, 0.57 mmol) and K_2_CO_3_ (4.73 g, 34.2 mmol) in dioxane/H_2_O (66 mL/33 mL). Purification by column chromatography (EtOAc/PE = 1/2, by *v*/*v*) gave **37**. White solid, 1.56 g (79%). m.p. 73.3–75.2 °C. ^1^H NMR (500 MHz, DMSO-*d*_6_) *δ* 8.74–8.73 (m, 2H), 8.62–8.61 (m, 1H), 7.94–7.88 (m, 3H), 7.62–7.58 (m, 1H); ^13^C NMR (126 MHz, CDCl_3_) *δ* 158.31 (d, *J* = 263.3 Hz), 150.27, 145.90 (d, *J* = 5.3 Hz), 143.27 (d, *J* = 10.0 Hz), 142.66 (d, *J* = 5.4 Hz), 125.28 (d, *J* = 4.3 Hz), 124.70 (d, *J* = 20.5 Hz), 122.91 (d, *J* = 6.6 Hz); ^19^F NMR (471 MHz, CDCl_3_) *δ* −121.62; ESI-HR-MS: (*m*/*z*) calcd. for C_10_H_8_N_2_F ([M+H]^+^) 175.0667, found 175.0671.

Synthesis of [2,4′-bipyridine]-3-thiol (**38**). A mixture of **37** (2.00 g, 11.5 mmol), Na_2_S (1.35 g, 12.76 mmol), and K_2_CO_3_ (3.18 g, 23.0 mmol) in DMF (50 mL) was stirred overnight at 120 °C under N_2_ until TLC analysis indicated completion of the reaction. After cooling to room temperature, the reaction mixture was evaporated on a rotary evaporator to remove most of the solvent under highly reduced pressure and the residue thus obtained was diluted with water (50 mL). The resulting mixture thus obtained was extracted with EtOAc (100 mL × 3), and the combined extracts were washed with brine, dried (MgSO_4_) and evaporated on a rotary evaporator to afford a residue, which was purified by column chromatography (MeOH/CH_2_Cl_2_ = 1/10, by *v*/*v*) to give **38**. Pale yellow solid, 0.42 g (21%). m.p. 149.0–151.0 °C. ^1^H NMR (500 MHz, DMSO-*d*_6_) *δ* 8.59–8.57 (m, 3H), 7.67 (dd, *J* = 1.5 Hz and 8.0 Hz, 1H), 7.40–7.37 (m, 3H); ^13^C NMR (126 Hz, DMSO-*d*_6_) *δ* 156.33, 149.38, 148.64, 146.14, 140.94, 129.81, 124.34, 123.35; ESI-HR-MS: (*m*/*z*) calcd. for C_10_H_9_N_2_S ([M+H]^+^) 189.0481, found: 189.0486.

Synthesis of tert-butyl 2-([2,4′-bipyridin]-3-ylthio)-2-methylpropanoate (**39**). A mixture of **38** (2.70 g, 14.3 mmol), *tert*-butyl 2-bromo-2-methylpropanoate (4.80 g, 21.5 mmol) and K_2_CO_3_ (5.93 g, 12.9 mmol) in DMF (135 mL) was stirred overnight at 45 °C under N_2_, when TLC analysis indicated the completion of reaction. On cooling to room temperature, the reaction mixture was evaporated on a rotary evaporator to give a residue, which was diluted with water (50 mL). The resulting mixture was extracted with EtOAc (100 mL × 3), and the combined extracts were washed with brine, dried (MgSO_4_), and evaporated on a rotary evaporator to afford a residue, which was purified by column chromatography (EtOAc/PE = 1/1, by *v*/*v*) to give **39**. Pale yellow oil, 1.71 g (36%). ^1^H NMR (500 MHz, DMSO-*d*_6_) *δ* 8.73 (dd, *J* = 1.8 Hz and 4.3 Hz, 2H), 8.57 (dd, *J* = 1.5 Hz and 5.0 Hz, 1H), 8.32 (dd, *J* = 1.5 Hz and 8.0 Hz, 1H), 7.59 (dd, *J* = 1.5 Hz and 4.4 Hz, 2H), 7.56 (dd, *J* = 4.5 Hz and 8.0 Hz, 1H), 1.33 (s, 6H), 1.20 (s, 9H); ^13^C NMR (126 Hz, DMSO-*d*_6_) *δ* 172.64, 160.26, 149.36, 149.29, 147.58, 144.97, 128.54, 125.07, 123.02, 81.63, 53.02, 27.85, 27.83, 26.17; ESI-HR-MS: (*m*/*z*) calcd. for C_18_H_23_N_2_O_2_ ([M+H]^+^) 331.1475, found 331.1482.

Synthesis of 2-([2,4′-bipyridin]-3-ylthio)-2-methylpropanoic acid hydrochloric acid salt (**40**). Following the procedure for the synthesis of **35** from **34**, **39** (2.00 g, 6.05 mmol) was first cleaved with TFA (10 mL) in CH_2_Cl_2_ (10 mL), and then the corresponding carboxylic acid was converted to the hydrochloric acid salt **40** with HCl/MeOH generated by SOCl_2_ (2 mL) in MeOH (10 mL). The sample was used directly in the next step without further purification or characterization.

Synthesis of 2-([2,4′-bipyridin]-3-ylthio)-1-(4-benzyl-4-hydroxypiperidin-1-yl)-2-methylpropan-1-one (**B1**). Following the procedure for the synthesis of **6** from **5** and **3·TFA**, **B1** was prepared from **40** (1.50 g, 4.83 mmol) and **3·TFA** (2.79 g, 9.66 mmol) using EDCI (2.42 g, 12.6 mmol), HOBT (49 mg, 0.362 mmol), and DIPEA (2.18 g, 16.9 mmol) in dried THF (75 mL). Purification by column chromatography (EtOAc) gave **B1**. White solid, 1.12 g (overall 41% for **39** to **B1**). m.p. 71.0–73.0 °C. ^1^H NMR (500 MHz, DMSO-*d*_6_) *δ* 8.67 (dd, *J* = 1.8 Hz and 4.3 Hz, 2H), 8.61 (dd, *J* = 1.5 Hz and 4.5 Hz, 1H), 7.75 (dd, *J* = 1.5 Hz and 3.0 Hz, 1H), 7.51 (dd, *J* = 1.8 Hz and 4.3 Hz, 2H), 7.40 (dd, *J* = 4.5 Hz and 8.0 Hz, 1H), 7.25–7.22 (m, 2H), 7.19–7.16 (m, 3H), 4.46 (s, 1H), 4.07–4.04 (m, 2H), 3.07–2.82 (m, 2H), 2.64 (m, 2H), 1.35–1.34 (m, 4H), 1.27 (s, 6H); ^13^C NMR (151 MHz, CDCl_3_) *δ* 170.80, 158.63, 149.30, 148.50, 147.76, 141.66, 135.84, 130.57, 129.31, 128.64, 127.13, 124.81, 123.51, 69.45, 51.59, 49.36, 37.16, 28.24; ESI-HR-MS: (*m*/*z*) calcd. for C_26_H_30_N_3_O_2_S ([M+H]^+^) 448.2053, found 448.2065.

Synthesis of 2-([2,4′-bipyridin]-3-ylsulfonyl)-1-(4-benzyl-4-hydroxypiperidin-1-yl)-2-methylpropan-1-one (**B2**). To a stirred solution of **B1** (1.00 g, 2.23 mmol) in CH_2_Cl_2_ (10 mL), cooled to 0 °C, *m*CPBA was added portionwise (1.16 g, 6.70 mmol). After addition, the reaction temperature was slowly raised to room temperature until TLC analysis indicated completion of the reaction. The reaction mixture was poured into aqueous 10% Na_2_S_2_O_3_ (30 mL) with ice, and the resulting mixture was extracted with EtOAc (50 mL × 3). The combined extracts were washed successively with saturated aqueous NaHCO_3_ (50 mL) and brine, dried (MgSO_4_), and evaporated on a rotary evaporator to afford a residue, which was purified by column chromatography (EtOAc) to give **B2**. White solid, 0.88 g (82%). m.p. 139.0–141.0 °C. ^1^H NMR (500 MHz, DMSO-*d*_6_) *δ* 8.93 (dd, *J* = 1.8 Hz and 4.8 Hz, 1H), 8.31 (dd, *J* = 1.8 Hz and 8.3 Hz, 1H), 8.26–8.24 (m, 2H), 7.68 (dd, *J* = 4.5 Hz and 8.0 Hz, 2H), 7.49–7.47 (m, 2H), 7.28–7.17 (m, 5H), 4.45 (s, 1H), 3.93–3.91 (m, 2H), 3.00 (s, 2H), 2.67 (s, 2H), 1.40 (s, 6H), 1.36–1.34 (m, 4H); ^13^C NMR (151 MHz, CDCl_3_) *δ* 165.74, 156.15, 153.49, 141.55, 137.74, 136.96, 136.26, 131.58, 130.63, 128.66, 128.36, 126.77, 122.94, 71.57, 69.06, 49.34, 42.24, 36.76, 23.59; ESI-HR-MS: (*m*/*z*) calcd. for C_26_H_30_N_3_O_4_S ([M+H]^+^) 480.1952, found 480.1960.

Synthesis of [2,4′-bipyridin]-3-amine (**42**). Following the procedure for the synthesis of **6** from **5** and pyridine-4-boronic acid, **42** was prepared from **41** (1.00 g, 5.78 mmol) and pyridine-4-boronic acid (0.71 g, 5.78 mmol) using Pd(PPh_3_)_4_ (0.33 g, 0.289 mmol) and K_2_CO_3_ (1.20 g, 8.67 mmol) in dioxane/H_2_O (32 mL/16 mL). Purification by column chromatography (EtOAc) gave **42**. White solid, 0.39 g (37%). m.p. 134–141 °C. ^1^H NMR (500 MHz, DMSO-*d*_6_) *δ* 8.93 (dd, *J* = 1.8 Hz and 4.8 Hz, 1H), 8.31 (dd, *J* = 1.8 Hz and 8.3 Hz, 1H), 8.26–8.24 (m, 2H), 7.68 (dd, *J* = 4.5 Hz and 8.0 Hz, 2H), 7.49–7.47 (m, 2H), 7.28–7.17 (m, 5H), 4.45 (s, 1H), 3.93–3.91 (m, 2H), 3.00 (s, 2H), 2.67 (s, 2H), 1.40 (s, 6H), 1.36–1.34 (m, 4H); ^13^C NMR (151 MHz, CDCl_3_) *δ* 165.74, 156.15, 153.49, 141.55, 137.74, 136.96, 136.26, 131.58, 130.63, 128.66, 128.36, 126.77, 122.94, 71.57, 69.06, 49.34, 42.24, 36.76, 23.59; ESI-HR-MS: (*m*/*z*) calcd. for C_10_H_10_N_3_ ([M+H]^+^) 172.0869, found 172.0873.

Synthesis of 3-isothiocyanato-2,4′-bipyridine (**43**). Following the procedure for the synthesis of **33** from **32**, **43** was prepared from **42** (2.37 g, 13.8 mmol) using CSCl_2_ (3.17 g, 27.6 mmol) and Et_3_N (2.79 g, 27.6 mmol) in dried CH_2_Cl_2_ (48 mL). Crude **43**, a brown solid (1.83 g), was used directly in the next step without further purification or characterization.

Synthesis of N-([2,4′-bipyridin]-3-yl)-4-benzyl-4-hydroxypiperidine-1-carbothioamide (**B3**). Following the procedure for the synthesis of **34** from **33** and Me_2_NH·HCl, **B3** was prepared from **43** (1.50 g, 7.03 mmol) and **3·TFA** (2.44 g, 8.44 mmol) using Et_3_N (2.14 g, 21.1 mmol) in dried THF (48 mL). Purification by column chromatography (EtOAc) gave **B3**. White solid, 1.85 g (65%). m.p. 190.0–192.0 °C. ^1^H NMR (500 MHz, DMSO-*d*_6_) *δ* 9.22 (s, 1H), 8.58–8.56 (m, 3H), 7.67 (dd, *J* = 1.5 Hz and 8.0 Hz, 1H), 7.60 (dd, *J* = 1.8 Hz and 4.3 Hz, 2H), 7.46 (dd, *J* = 4.5 Hz and 8.0 Hz, 1H), 7.30–7.27 (m, 2H), 7.24–7.22 (m, 3H), 4.55 (s, 1H), 4.40 (s, 2H), 3.37–3.30 (m, 2H), 2.71 (s, 2H), 1.41–1.38 (m, 4H); ^13^C NMR (126 MHz, DMSO-*d*_6_) *δ* 180.57, 152.85, 149.37, 147.53, 146.20, 139.33, 137.44, 135.88, 130.59, 127.71, 126.02, 123.69, 123.13, 68.44, 48.57, 44.39, 36.48; ESI-HR-MS: (*m*/*z*) calcd. for C_23_H_25_N_4_OS ([M+H]^+^) 405.1744, found 405.1754.

Synthesis of phenyl [2,4′-bipyridin]-3-ylcarbamate (**44**). To a stirred solution of PhOCOCl (1.50 g, 8.76 mmol) in MeCN, cooled to 0 °C under N_2_, pyridine (1.23 g, 17.5 mmol) and a solution of **42** (1.00 g, 5.84 mmol) in MeCN (3 mL) were added dropwise in sequence. After addition, the reaction mixture was stirred at room temperature until TLC indicated analysis completion of the reaction (typically within 1 h) and then poured into ice–water (50 mL). The resulting mixture was extracted with EtOAc (50 mL × 3), and the combined extracts were washed with brine, dried (MgSO_4_), and evaporated on a rotary evaporator to afford a residue, which was purified by column chromatography (EtOAc/PE = 2/1, by *v*/*v*) to give **44**. White solid, 1.43 g (84%). m.p. 133.0–134.0 °C. ^1^H NMR (500 MHz, DMSO-*d*_6_) *δ* 8.86–8.80 (m, 2H), 8.50–8.47 (m, 0.69H), 7.73–7.70 (m, 0.70H), 7.67–7.64 (m, 1.49H), 7.42–7.39 (m, 3H), 7.30–7.27 (m, 1.46H), 7.00–6.97 (m, 3H); ^13^C NMR (126 MHz, CDCl_3_) *δ* 154.52, 150.54, 150.45, 150.37, 150.16, 145.38, 137.36, 132.94, 129.74, 126.73, 124.50, 123.05, 120.97; ESI-HR-MS: (*m*/*z*) calcd. for C_17_H_14_N_3_O_2_ ([M+H]^+^) 292.1081, found 292.1084.

Synthesis of N-([2,4′-bipyridin]-3-yl)-4-benzyl-4-hydroxypiperidine-1-carboxamide (**B4**). Following the procedure for the synthesis of **B3** from **43** and **3·TFA**, **B4** was prepared from **44** (0.60 g, 2.06 mmol) and **3·TFA** (0.79 g, 4.12 mmol) using Et_3_N (0.63 g, 6.18 mmol) in dried THF (10 mL). Purification by column chromatography (EtOAc) gave **B4**. White solid, 0.62 g (78%). m.p. 99.0–101.1 °C. ^1^H NMR (500 MHz, DMSO-*d*_6_) *δ* 8.56 (dd, *J* = 1.5 Hz and 4.5 Hz, 2H), 8.47 (dd, *J* = 1.5 Hz and 5.0 Hz, 1H), 8.40 (s, 1H), 7.70 (dd, *J* = 1.5 Hz and 8.0 Hz, 1H), 7.57 (dd, *J* = 1.5 Hz and 4.5 Hz, 2H), 7.41 (dd, *J* = 4.5 Hz and 8.0 Hz, 1H), 7.30–7.27 (m, 2H), 7.23–7.19 (m, 3H), 4.40 (s, 1H), 3.70–3.66 (m, 2H), 3.07–3.01 (m, 2H), 2.68 (m, 2H), 1.33–1.30 (m, 4H); ^13^C NMR (126 MHz, CDCl_3_) *δ* 154.35, 150.52, 146.64, 146.02, 144.76, 135.74, 133.60, 130.57, 130.54, 128.63, 127.10, 124.12, 123.49, 69.23, 49.39, 40.47, 36.60; ESI-HR-MS: (*m*/*z*) calcd. for C_23_H_25_N_4_O_2_ ([M+H]^+^) 389.1973, found 389.1980.

Synthesis of [2,4′-bipyridine]-3-carbaldehyde (**46**). Following the procedure for the synthesis of **6** from **5** and pyridine-4-boronic acid, **46** was prepared from **45** (10.00 g, 54.3 mmol) and pyridine-4-boronic acid (7.35 g, 59.8 mmol) using Pd(PPh_3_)_4_ (3.14 g, 2.72 mmol) and K_2_CO_3_ (22.51 g, 16.3 mmol) in dioxane/H_2_O (200 mL/100 mL). Purification by column chromatography (EtOAc) gave **46**. White solid, 5.20 g (57%). m.p. 108.0–110.0 °C. ^1^H NMR (500 MHz, DMSO-*d*_6_) *δ* 10.00 (s, 1H), 8.95 (dd, *J* = 2.0 and 5.0 Hz, 1H), 8.75–8.74 (m, 2H), 8.34 (dd, *J* = 1.75 and 7.8 Hz, 1H), 7.70 (dd, *J* = 5.0 and 8.0 Hz, 1H), 7.66–7.65 (m, 2H). The ^1^H NMR data were consistent with those reported in the literature [24].

Synthesis of (E)-[2,4′-bipyridine]-3-carbaldehyde oxime (**47**). To a stirred solution of **46** (5.00 g, 27.1 mmol) in EtOH/H_2_O (25 mL/25 mL) at room temperature, NH_2_OH·HCl (2.07 g, 29.8 mmol) and NaOH (1.08 g, 27.1 mmol) were added successively in a portionwise manner. After addition, the reaction mixture was stirred at reflux overnight until TLC analysis indicated completion of the reaction. After cooling to room temperature, the reaction mixture was poured into ice–water (50 mL) and extracted with EtOAc (50 mL × 3). The combined extracts were washed with brine, dried (MgSO_4_), and evaporated on a rotary evaporator to afford a residue, which was purified by column chromatography (EtOAc) to give **47**. White solid, 3.00 g (55%). m.p. 156.0–157.2 °C. ^1^H NMR (500 MHz, DMSO-*d*_6_) *δ* 11.73 (s, 1H), 8.72–8.71 (m, 3H), 8.24 (dd, *J* = 2.0 Hz and 8.0 Hz, 1H), 8.01 (s, 1H), 7.54–7.52 (m, 3H); ^13^C NMR (126 MHz, CDCl_3_) *δ* 155.05, 150.45, 149.64, 146.83, 146.34, 135.16, 127.19, 124.63, 123.75; ESI-HR-MS: (*m*/*z*) calcd. for C_11_H_10_N_3_ O ([M+H]^+^) 200.0809, found 200.0823.

Synthesis of [2,4′-bipyridin]-3-ylmethanamine (**48**). To a stirred solution of **47** (0.50 g, 2.51 mmol) in EtOH/H_2_O (5 mL/5 mL), cooled to 0 °C under N_2_, AcONH_4_ (0.48 g, 6.27 mmol) and concentrated aqueous ammonia (7.5 mL) were added successively, followed by addition of zinc powder (0.82 g, 12.6 mmol). After addition, the reaction mixture was refluxed overnight until TLC analysis indicated completion of the reaction. After cooling to room temperature, the reaction mixture was evaporated on a rotary evaporator to remove EtOH and then diluted with saturated brine (50 mL). The resulting mixture was extracted with EtOAc (50 mL × 3), and the combined extracts were washed with saturated brine, dried (MgSO_4_), and evaporated on a rotary evaporator to afford a residue, which was purified by column chromatography (MeOH/CH_2_Cl_2_ = 1/15, by *v*/*v*) to give **48**. Pale yellow oil (0.24 g). This sample was directly used in the next step without further purification or characterization.

Synthesis of phenyl ([2,4′-bipyridin]-3-ylmethyl)carbamate (**49**). Following the procedure for the synthesis of **44** from **42**, **49** was prepared from **48** (0.24 g, 1.32 mmol) and PhOCOCl (0.31 g, 1.98 mmol) using pyridine (0.52 g, 6.64 mmol) in MeCN (5 mL). Purification by column chromatography (EtOAc) gave **49**. Colorless oil, 0.05 g. This sample was directly used in the next step without characterization.

Synthesis of N-([2,4′-bipyridin]-3-ylmethyl)-4-benzyl-4-hydroxypiperidine-1-carboxamide (**B5**). Following the procedure for the synthesis of **B3** from **43** and **3·TFA**, **B5** was prepared from **49** (0.10 g, 0.328 mmol) and **3·TFA** (0.19 g, 0.656 mmol) using Et_3_N (0.10 g, 0.984 mmol) in dried THF (10 mL). Purification by column chromatography (EtOAc) gave **B5**. White solid, 50 mg (overall 76% for **47** to **B5**). m.p. 181.3–183.4 °C. ^1^H NMR (500 MHz, DMSO-*d*_6_) *δ* 8.67 (d, *J* = 5.0 Hz, 2H), 8.55 (d, *J* = 4.5 Hz, 1H), 7.76 (d, *J* = 8.0 Hz, 1H), 7.57 (d, *J* = 5.0 Hz, 2H), 7.43 (dd, *J* = 4.5 Hz and 8.0 Hz, 1H), 7.27–7.24 (m, 2H), 7.21–7.18 (m, 3H), 7.01 (t, *J* = 5.5 Hz, 1H), 4.35 (s, 1H), 4.24–4.23 (m, 2H), 3.63–3.59 (m, 2H), 3.03–2.97 (m, 2H), 2.67 (s, 2H), 1.36–1.28 (m, 4H); ^13^C NMR (126 MHz, CDCl_3_) *δ* 157.31, 155.39, 150.01, 148.48, 147.68, 137.15, 135.91, 133.35, 130.61, 128.59, 127.03, 123.77, 123.60, 69.35, 49.40, 41.95, 40.22, 36.55; ESI-HR-MS: (*m*/*z*) calcd. for C_24_H_27_N_4_O_2_ ([M+H]^+^) 403.2199, found 403.2139.

Synthesis of (2-bromopyridin-3-yl)methanol (**50**). To a stirred solution of **45** (3.00 g, 16.1 mmol) in MeOH (30 mL), cooled to 0 °C under N_2_, NaBH_4_ (0.73 g, 19.3 mmol) was added portionwise. After addition, the reaction temperature was slowly raised to room temperature until TLC analysis indicated completion of the reaction. The reaction mixture was poured into saturated aqueous NH_4_Cl (100 mL), and the resulting mixture was extracted with EtOAc (100 mL × 3). The combined extracts were washed with saturated brine, dried (MgSO_4_), and evaporated on a rotary evaporator to afford a residue, which was purified by column chromatography (EtOAc/PE = 1/3, by *v*/*v*) to give **50**. White solid, 2.90 g (96%). m.p. 66.0–69.0 °C. ^1^H NMR (500 MHz, DMSO-*d*_6_) *δ* 8.28–8.26 (m, 1H), 7.90–7.88 (m, 1H), 7.49–7.46 (m, 1H), 5.60 (s, 1H), 4.49 (s, 2H). The ^1^H NMR data were consistent with those reported in the literature [34].

Synthesis of 2-bromo-3-(bromomethyl)pyridine (**51**). To a stirred solution of **50** (2.82 g, 15.0 mmol) in dried CH_2_Cl_2_ (30 mL), cooled to 0 °C under N_2_, PBr_3_ (8.12 g, 30.0 mmol) was added in a dropwise manner. After addition, the reaction mixture was stirred at reflux until completion, as indicated by TLC analysis (typically within 3 h). After cooling to room temperature, the reaction mixture was slowly poured into saturated aqueous NaHCO_3_ (100 mL), and the resulting mixture was extracted with EtOAc (100 mL × 3). The combined extracts were washed with saturated brine, dried (MgSO_4_), and evaporated on a rotary evaporator to afford a residue, which was purified by column chromatography (EtOAc/PE = 1/5, by *v*/*v*) to give **51**. Pale yellow oil, 1.90 g (51%). ^1^H NMR (500 MHz, CD_3_OD) *δ* 8.28 (dd, *J* = 1.8 Hz and 4.8 Hz, 1H), 7.95 (dd, *J* = 1.8 Hz and 7.8 Hz, 1H), 7.41 (dd, *J* = 5.0 Hz and 7.5 Hz, 1H), 4.65 (s, 2H). The ^1^H NMR data were consistent with those reported in the literature [35].

Synthesis of 2-(2-bromopyridin-3-yl)acetonitrile (**52**). To a stirred solution of **51** (3.40 g, 13.6 mmol) in MeCN (34 mL), cooled to 0 °C under N_2_, TMSCN (4.05 g, 40.8 mmol) was added slowly, followed by dropwise addition of a solution of TBAF in THF (17.7 mL, 17.7 mmol, 1.0 M in THF). After addition, the reaction mixture was stirred at room temperature overnight until TLC analysis indicated completion of the reaction and was then poured into ice–water (100 mL). The resulting mixture was extracted with EtOAc (100 mL × 3), and the combined extracts were washed with saturated brine, dried (MgSO_4_), and evaporated on a rotary evaporator to afford a residue, which was purified by column chromatography (EtOAc/PE = 1/4, by *v*/*v*) to give **52**. White solid, 2.30 g (85%). m.p. 80.0–82.0 °C. ^1^H NMR (500 MHz, DMSO-*d*_6_) *δ* 8.39 (dd, *J* = 1.8 Hz and 4.8 Hz, 1H), 7.96 (dd, *J* = 1.8 Hz and 7.8 Hz, 1H), 7.53 (dd, *J* = 4.5 Hz and 7.5 Hz, 1H), 4.11 (s, 2H). The ^1^H NMR data were consistent with those reported in the literature [36].

Synthesis of 2-([2,4′-bipyridin]-3-yl)acetonitrile (**53**). Following the procedure for the synthesis of **6** from **5** and pyridine-4-boronic acid, **53** was prepared from **52** (1.85 g, 9.39 mmol) and pyridine-4-boronic acid (1.39 g, 11.3 mmol) using Pd (PPh_3_)_4_ (0.34 g, 0.282 mmol) and Na_2_CO_3_ (2.99 g, 28.2 mmol) in DME/H_2_O (20 mL/20 mL). White solid, 1.02 g (56%). m.p. 150.0–152.1 °C. ^1^H NMR (500 MHz, DMSO-*d*_6_) *δ* 8.72 (dd, *J* = 3.5 Hz and 4.3 Hz, 2H), 8.69 (dd, *J* = 1.5 Hz and 5.0 Hz, 1H), 8.01 (dd, *J* = 1.5 Hz and 8.0 Hz, 1H), 7.57–7.54 (m, 3H), 4.14 (s, 2H); ^13^C NMR (126 MHz, CDCl_3_) *δ* 155.93, 150.44, 149.75, 146.36, 137.73, 124.21, 123.96, 123.39, 116.94, 21.77; ESI-HR-MS: (*m*/*z*) calcd. for C_12_H_10_N_3_ ([M+H]^+^) 196.0870, found 196.0870.

Synthesis of methyl 2-([2,4′-bipyridin]-3-yl)acetate (**54**). To a stirred solution of **53** (1.00 g, 5.12 mmol) in AcOH (20 mL)/H_2_O (2 mL), cooled to 0 °C under N_2_, concentrated H_2_SO_4_ (2 mL) was added dropwise. After addition, the reaction mixture was refluxed overnight until TLC analysis indicated completion of the reaction. Upon cooling to room temperature, the reaction mixture was evaporated on a rotary evaporator to afford a residue, which was diluted with a mixed solvent of MeOH (4 mL) and CH_2_Cl_2_ (40 mL). The mixture thus obtained was dried (MgSO_4_) and evaporated on a rotary evaporator to afford a residue, which was dissolved in MeOH (10 mL). The resulting solution was cooled at 0 °C, followed by dropwise addition of SOCl_2_ (2 mL), and the reaction mixture was then refluxed under N_2_ overnight, until TLC analysis indicated completion of the reaction. Upon cooling to room temperature, saturated aqueous NaHCO_3_ was slowly added to the reaction mixture until pH > 8. The mixture thus obtained was extracted with EtOAc (50 mL × 3), and the combined extracts were washed with saturated brine, dried (MgSO_4_), and evaporated on a rotary evaporator to afford a residue, which was purified by column chromatography (EtOAc/PE = 1/1, by *v*/*v*) to give **54**. Pale yellow oil, 0.55 g (47%). ^1^H NMR (500 MHz, CDCl_3_) *δ* 8.73–8.72 (m, 2H), 8.64 (dd, *J* = 1.8 Hz and 4.8 Hz, 1H), 7.76 (dd, *J* = 1.8 Hz and 7.8 Hz, 1H), 7.44 (d, *J* = 6.0 Hz, 2H), 7.35 (dd, *J* = 4.8 Hz and 7.8 Hz, 1H), 3.68 (s, 3H), 3.70 (s, 2H); ^13^C NMR (126 MHz, CDCl_3_) *δ* 171.20, 156.53, 149.91, 148.70, 147.53, 139.13, 127.78, 123.79, 123.40, 52.39, 37.90; ESI-HR-MS: (*m*/*z*) calcd. for C_13_H_13_N_2_O_2_ ([M+H]^+^) 229.0972, found 229.0971.

Synthesis of methyl 2-([2,4′-bipyridin]-3-yl)-2-methylpropanoate (**55**). To a stirred solution of **54** (0.50 g, 2.19 mmol) in dried THF (5 mL), cooled to −78 °C under N_2_, a solution of LiHMDS in THF (6.6 mL, 6.6 mmol, 1.0 M in THF) was added dropwise. After addition, the resulting mixture was stirred at this temperature for another 1 h, followed by dropwise addition of a solution of MeI (0.93 g, 6.57 mmol) in dried THF (5 mL). After addition, the reaction mixture was stirred at this temperature for 1 h and then at room temperature overnight until LC-MS analysis indicated completion of the reaction and was then poured into ice–water (20 mL). The mixture thus obtained was extracted with EtOAc (30 mL × 3), and the combined extracts were washed with brine, dried (MgSO_4_), and evaporated on a rotary evaporator to afford a residue, which was purified by column chromatography (EtOAc/PE = 1/1, by *v*/*v*) to give **55**. White solid, 0.47 g (84%). m.p 103.1–105.3 °C. ^1^H NMR (500 MHz, CDCl_3_) *δ* 8.68 (d, *J* = 5.0 Hz, 2H), 8.57 (dd, *J* = 1.3 Hz and 4.8 Hz, 1H), 7.88 (dd, *J* = 1.5 Hz and 8.0 Hz, 1H), 7.38 (dd, *J* = 4.8 Hz and 8.3 Hz, 1H), 7.27–7.26 (m, 2H), 3.37 (s, 3H), 1.53 (s, 6H); ^13^C NMR (126 MHz, CDCl_3_) *δ* 176.49, 156.26, 149.42, 149.30, 147.32, 138.62, 134.84, 124.17, 123.18, 52.13, 45.84, 28.28; ESI-HR-MS: (*m*/*z*) calcd. for C_15_H_17_N_2_O_2_ ([M+H]^+^) 257.1285, found 257.1285.

Synthesis of 2-([2,4′-bipyridin]-3-yl)-2-methylpropanoic acid (**56**). To a stirred solution of **55** (0.43 g, 1.68 mmol) in MeOH (5 mL) a solution of NaOH (0.13 g, 3.36 mmol) in H_2_O (1 mL) was added. After addition, the reaction mixture was stirred at 45 °C overnight until TLC analysis indicated completion of the reaction. Upon cooling to room temperature, the reaction mixture was cooled in an ice–water bath, followed by dropwise addition of concentrated HCl (0.28 mL, 3.36 mmol). After that, the mixture became a white slurry, and stirring was continued for another 1 h. The precipitate was collected via suction filtration and dried in vacuo at room temperature to give **56**. White solid, 0.12 g (34%). m.p. 135.4–137.1 °C. ^1^H NMR (500 MHz, DMSO-*d*_6_) *δ* 12.44 (s, 1H), 8.59–8.58 (m, 2H), 8.51–8.49 (m, 1H), 7.99–7.97 (m, 1H), 7.47 (dd, *J* = 4.8 Hz and 8.3 Hz, 1H), 7.27–7.26 (m, 2H), 1.36 (s, 6H); ^13^C NMR (151 MHz, CD_3_OD) δ 179.88, 157.11, 151.31, 149.57, 147.52, 140.69, 137.16, 126.45, 125.01, 46.69, 28.17; ESI-HR-MS: (*m*/*z*) calcd. for C_14_H_15_N_2_O_2_ ([M+H]^+^) 243.1129, found 243.1129.

Synthesis of 2-([2,4′-bipyridin]-3-yl)-1-(4-benzyl-4-hydroxypiperidin-1-yl)-2-methylpropan-1-one (**B6**). Following the procedure for the synthesis of **21** from **4** and **3·TFA**, **56** (0.60 g, 2.48 mmol) was first treated with (COCl)_2_ (0.94 g, 7.44 mmol) using DMF (one drop) as catalyst in dried CH_2_Cl_2_ (6 mL) to give the corresponding carboxylic acid chloride, which was subsequently treated with **3·TFA** (0.93 g, 3.22 mmol) using Et_3_N (1.51 g, 14.9 mmol) in dried THF (8 mL) to give **B6** after column chromatography (EtOAc/PE = 1/1, by *v*/*v*). White solid, 0.13 g (16%). m.p 150.3–152.4 °C. ^1^H NMR (500 MHz, DMSO-*d*_6_) *δ* 8.53–8.51 (m, 3H), 7.91 (dd, *J* = 1.8 Hz and 8.3 Hz, 1H), 7.45 (dd, *J* = 4.8 Hz and 8.3 Hz, 1H), 7.25–7.13 (m, 7H), 4.34 (s, 1H), 3.61–3.58 (m, 1H), 2.90–2.87 (m, 1H), 2.73–2.69 (m, 1H), 2.60 (s, 2H), 2.48–2.36 (m, 1H), 1.41 (s, 3H), 1.37 (s, 3H), 1.27–1.12 (m, 4H); ^13^C NMR (126 MHz, CDCl_3_) *δ* 172.37, 154.92, 148.95, 148.81, 147.36, 139.92, 135.90, 134.56, 130.53, 128.54, 126.97, 123.78, 123.64, 69.23, 49.43, 45.98, 42.27, 37.99, 36.71, 36.25, 31.52, 28.52; ESI-HR-MS: (*m*/*z*) calcd. for C_26_H_30_N_3_O_2_ ([M+H]^+^) 416.2333, found: 416.2332.

Synthesis of methyl 1-([2,4′-bipyridin]-3-yl)cyclopropane-1-carboxylate (**57**). To a stirred solution of **54** (3.00 g, 13.1 mmol) in dried THF (30 mL), cooled to −78 °C under N_2_, a solution of LiHMDS in THF (15.7 mL, 15.7 mmol, 1.0 M in THF) was added dropwise. After addition, the resulting mixture was stirred at this temperature for another 1 h, followed by dropwise addition of a solution of 1,3,2-dioxathiolane 2,2-dioxide (1.95 g, 15.7 mmol) in dried THF (6 mL). After addition, the resulting mixture was stirred at −20 °C for 1.5 h and then cooled to −78 °C, followed by addition of a solution of LiHMDS in THF (15.7 mL, 15.7 mmol, 1.0 M in THF). After that, the reaction mixture was stirred at room temperature overnight until LC-MS analysis indicated completion of the reaction, and poured into ice–water (100 mL). The mixture thus obtained was extracted with EtOAc (50 mL × 3), and the combined extracts were washed with brine, dried (MgSO_4_), and evaporated on a rotary evaporator to afford a residue, which was purified by column chromatography (EtOAc/PE = 1/1, by *v*/*v*). White solid, 2.00 g (60%). m.p. 89.1–91.5 °C. ^1^H NMR (500 MHz, DMSO-*d*_6_) *δ* 8.66 (dd, *J* = 1.5 Hz and 4.5 Hz, 2H), 8.61 (dd, *J* = 1.5 Hz and 5.0 Hz, 1H), 7.91 (dd, *J* = 1.5 Hz and 8.0 Hz, 1H), 7.46 (dd, *J* = 4.8 Hz and 7.8 Hz, 1H), 7.40 (dd, *J* = 1.8 Hz and 4.3 Hz, 1H), 3.52 (s, 3H), 1.39 (d, *J* = 3.5 Hz, 2H), 1.08 (d, *J* = 3.0 Hz, 2H); ^13^C NMR (126 MHz, CD_3_OD) *δ* 177.23, 157.41, 152.88, 149.20, 147.94, 142.91, 136.27, 126.37, 125.46, 28.26, 19.41; ESI-HR-MS: (*m*/*z*) calcd. for C_15_H_15_N_2_O_2_ ([M+H]^+^) 255.1129, found 255.1127.

Synthesis of 1-([2,4′-bipyridin]-3-yl)cyclopropane-1-carboxylic acid (**58**). Following the procedure for the synthesis of **56** from **55**, **58** was prepared from **57** (2.00 g, 7.87 mmol) first by treatment with NaOH (0.94 g, 23.6 mmol) in a mixture of MeOH (20 mL)/H_2_O (2 mL), and subsequent acidification with concentrated HCl (1.97 mL, 23.6 mmol). White solid, 1.43 g (76%). ^1^H NMR (500 MHz, DMSO-*d*_6_) *δ* 12.69 (s, 1H), 8.77 (d, *J* = 5.5 Hz, 2H), 8.63 (dd, *J* = 1.8 Hz and 4.8 Hz, 1H), 7.94 (dd, *J* = 1.5 Hz and 7.5 Hz, 1H), 7.65 (d, *J* = 5.0 Hz, 2H), 7.49 (dd, *J* = 4.8 Hz and 7.8 Hz, 1H), 1.39 (s, 2H), 0.97 (s, 2H); ^13^C NMR (126 MHz, CD_3_OD) δ 177.18, 157.42, 152.90, 149.21, 147.94, 142.89, 136.28, 126.38, 125.44, 28.26, 19.40; ESI-HR-MS: (*m*/*z*) calcd. for C_14_H_13_N_2_O_2_ ([M+H]^+^) 241.0972, found 241.0971.

Synthesis of (1-([2,4′-bipyridin]-3-yl)cyclopropyl)(4-benzyl-4-hydroxypiperidin-1-yl)methanone (**B7**). Following the procedure for the synthesis of **B6** from **56**, **58** (0.50 g, 2.08 mmol) was first treated with (COCl)_2_ (0.53 g, 4.16 mmol) in the presence of DMF (one drop) in dried CH_2_Cl_2_ (5 mL) to form the corresponding carboxylic acid chloride, which was subsequently treated with **3·TFA** (0.78 g, 2.70 mmol) in the presence of Et_3_N (1.26 g, 12.5 mmol) in dried THF (5 mL) to give **B7** after column chromatography (EtOAc). White solid, 0.33 g (38%). m.p. 122.5–124.1 °C. ^1^H NMR (500 MHz, DMSO-*d*_6_) *δ* 8.56 (dd, *J* = 1.3 Hz and 4.8 Hz, 1H), 8.46 (dd, *J* = 1.5 Hz and 4.5 Hz, 2H), 7.87 (dd, *J* = 1.5 Hz and 8.0 Hz, 1H), 7.43 (dd, *J* = 4.5 Hz and 8.0 Hz, 1H), 7.31–7.28 (m, 2H), 7.24–7.21 (m, 1H), 7.17–7.16 (m, 2H), 7.08–7.07 (m, 2H), 4.31 (s, 1H), 3.26–3.22 (m, 2H), 2.64–2.55 (m, 4H), 1.43–1.33 (m, 4H), 1.00–0.82 (m, 4H); ^13^C NMR (126 MHz, CDCl_3_) *δ* 169.52, 157.14, 149.35, 147.81, 147.28, 135.98, 135.14, 134.17, 130.54, 128.55, 127.05, 124.12, 123.44, 69.49, 49.57, 35.76, 27.85, 16.19; ESI-HR-MS: (*m*/*z*) calcd. for C_26_H_28_N_3_O_2_ ([M+H]^+^) 414.2177, found 414.2174.

Synthesis of *tert*-butyl 4-benzyl-4-cyanopiperidine-1-carboxylate (**68**). To a stirred solution of **67** (3.00 g, 14.3 mmol) in dried THF (30 mL), cooled to −78 °C under N_2_, a solution of LDA in THF (9.3 mL, 18.6 mmol, 2.0 M in THF) was added dropwise. After addition, the mixture thus obtained was stirred at this temperature for 40 min, followed by dropwise addition of BnBr (3.18 g, 18.6 mmol). After that, the reaction mixture was stirred at room temperature overnight until TLC analysis indicated completion of the reaction, and poured into ice–water (50 mL). The resulting mixture was extracted with EtOAc (50 mL × 3), and the combined extracts were washed with brine, dried (MgSO_4_), and evaporated on a rotary evaporator to afford a residue, which was purified by column chromatography (EtOAc/PE = 1/10, by *v*/*v*) to give **68**. White solid, 3.50 g (82%). m.p. 85.1–86.5 °C. ^1^H NMR (500 MHz, DMSO-*d*_6_) *δ* 7.36–7.33 (m, 2H), 7.30–7.27 (m, 3H), 4.00–3.97 (m, 2H), 2.91–2.78 (m, 4H), 1.76–1.73 (m, 2H), 1.57–1.51 (m, 2H), 1.40 (s, 9H); ^13^C NMR (126 MHz, CDCl_3_) *δ* 154.54, 134.42, 130.39, 128.62, 127.68, 121.95, 80.15, 45.95, 40.93, 39.24, 34.77, 28.50; ESI-HR-MS: (*m*/*z*) calcd. for C_18_H_24_N_2_NaO_2_ ([M+Na]^+^) 323.1730, found 323.1737.

Synthesis of *tert*-butyl 4-benzyl-4-carbamoylpiperidine-1-carboxylate (**69**). To a stirred solution of **68** (2.00 g, 6.66 mmol) in DMSO (20 mL), cooled to 0 °C, K_2_CO_3_ (1.82 g, 13.2 mmol) and H_2_O_2_ (1.13 g, 33.3 mmol) were added successively. After addition, the reaction mixture was stirred at room temperature overnight until TLC analysis indicated completion of the reaction, and poured into ice–water (60 mL). The resulting mixture was extracted with EtOAc (50 mL × 3), and the combined extracts were washed with brine, dried (MgSO_4_), and evaporated on a rotary evaporator to afford a residue, which was purified by column chromatography (EtOAc/PE = 1/5, by *v*/*v*) to give **69**. White solid, 2.00 g (94%). m.p. 159.9–162.1 °C. ^1^H NMR (500 MHz, DMSO-*d*_6_) δ 7.29–7.23 (m, 3H), 7.21–7.18 (m, 1H), 7.14–7.12 (m, 2H), 7.05 (br, 1H), 3.71–3.68 (m, 2H), 2.84–2.77 (m, 4H), 1.91–1.89 (m, 2H), 1.37 (s, 9H), 1.30–1.24 (m, 2H); ^13^C NMR (126 MHz, CDCl_3_) *δ* 177.05, 154.96, 136.32, 130.30, 128.29, 126.98, 79.75, 46.79, 46.24, 41.46, 41.03, 40.70, 33.55, 28.53; ESI-HR-MS: (*m*/*z*) calcd. for C_18_H_27_N_2_O_3_ ([M+H]^+^) 319.2017, found 319.2017.

Synthesis of *tert*-butyl 4-amino-4-benzylpiperidine-1-carboxylate (**70**). To a stirred solution of **69** (1.93 g, 6.06 mmol) in MeCN/H_2_O (10 mL/10 mL) at room temperature, PIFA (2.61 g, 6.06 mmol) was added. After addition, the reaction mixture was stirred at room temperature overnight until TLC analysis indicated completion of the reaction, and poured into ice–water (50 mL). The resulting mixture was extracted with EtOAc (50 mL × 3), and the combined extracts were washed with brine, dried (MgSO_4_), and evaporated on a rotary evaporator to afford a residue, which was purified by column chromatography (EtOAc) to give **70**. White solid, 0.96 g (55%). m.p. 163.4–166.2 °C. ^1^H NMR (500 MHz, DMSO-*d*_6_) *δ* 7.83 (br, 2H), 7.38–7.31 (m, 3H), 7.25–7.24 (m, 2H), 3.50–3.46 (m, 4H), 2.98–2.97 (m, 2H), 1.64–1.58 (m, 4H), 1.38 (s, 9H); ^13^C NMR (126 MHz, CDCl_3_) *δ* 154.90, 137.50, 130.39, 128.02, 126.17, 79.30, 45.84, 40.98, 39.80, 39.12, 36.46, 31.99, 28.37; ESI-HR-MS: (*m*/*z*) calcd. for C_17_H_27_N_2_O_2_ ([M+H]^+^) 291.2067, found 291.2065.

General Procedure 1: Synthesis of **71-1**, **72-1**, **72-3**, and **72-4**. To a stirred solution of **70** (1.0 eq) in dried CH_2_Cl_2_ (10 *v*/*w*, based on **70**), cooled to 0 °C under N_2_, Et_3_N (2–3 eq) was added, followed by dropwise addition of carbonyl or sulfonyl chloride (1.2–1.5 eq). After that, the reaction mixture was stirred at room temperature until completion, as indicated by TLC analysis (typically within 2–3 h), and poured into ice–water. The resulting mixture thus obtained was extracted with CH_2_Cl_2_ in three portions, and the combined extracts were washed with brine, dried (MgSO_4_), and evaporated on a rotary evaporator to afford a residue, which was purified by column chromatography to give the desired product.

General Procedure 2: Synthesis of **71-2**, **71-3**, **72-2**, and **72-5** to **72-8**. To a stirred solution of **70** (1.0 eq) and DMAP (0.2 eq) in dried CH_2_Cl_2_ (10 *v*/*w*, based on **70**), cooled to 0 °C under N_2_, Et_3_N (2–3 eq) was added, followed by dropwise addition of carbonyl or sulfonyl anhydride (1.2–1.5 eq). after that, the reaction mixture was stirred at room temperature until completion, as indicated by TLC analysis (typically within 2–3 h), and poured into ice–water. The resulting mixture thus obtained was extracted with CH_2_Cl_2_ in three portions, and the combined extracts were washed with brine, dried (MgSO_4_), and evaporated on a rotary evaporator to afford a residue, which was purified by column chromatography to give the desired product.

*tert*-butyl 4-benzyl-4-(methylsulfonamido)piperidine-1-carboxylate (**71-1**). Compound **71-1** was prepared according to General Procedure 2 from **70** (2.00 g, 6.89 mmol) using Ms_2_O (1.44 g, 8.27 mmol), DMAP (0.17 g, 1.38 mmol), and Et_3_N (1.41 g, 13.8 mmol) in dried CH_2_Cl_2_ (20 mL). Purification by column chromatography (EtOAc/PE = 1/5, by *v*/*v*) gave **71-1**. White foam. 1.20 g (47%). ^1^H NMR (500 MHz, DMSO-*d*_6_) *δ* 7.31–7.27 (m, 4H), 7.23–7.20 (m, 1H), 6.74 (s, 1H), 3.62 (d, *J* = 13.5 Hz, 2H), 3.12 (br, 2H), 3.01 (s, 5H), 1.81 (d, *J* = 13.5 Hz, 2H), 1.45–1.39 (m, 2H), 1.36 (s, 9H); ^13^C NMR (151 MHz, CDCl_3_) *δ* 154.63, 135.80, 131.19, 128.17, 126.77, 79.78, 58.53, 45.66, 45.04, 39.91, 39.17, 34.40, 28.38; ESI-HR-MS: (*m*/*z*) calcd. for C_18_H_28_NaN_2_O_4_S ([M+Na]^+^) 391.1662, found 391.1664.

*tert*-butyl 4-benzyl-4-((trifluoromethyl)sulfonamido)piperidine-1-carboxylate (**71-2**). Compound **71-2** was prepared according to General Procedure 2 from **70** (1.50 g, 5.17 mmol) using Tf_2_O (1.75 g, 6.20 mmol), DMAP (0.13 g, 1.03 mmol), and Et_3_N (1.04 g, 10.3 mmol) in dried CH_2_Cl_2_ (15 mL). Purification by column chromatography (EtOAc/PE = 1/5, by *v*/*v*) gave **71-2**. White foam, 1.10 g (50%). ^1^H NMR (500 MHz, DMSO-*d*_6_) *δ* 7.37 (s, 1H), 7.31–7.22 (m, 5H), 3.66–3.62 (m, 2H), 2.98–2.92 (m, 4H), 1.90–1.87 (m, 1H), 1.68–1.65 (m, 1H), 1.60–1.54 (m, 1H), 1.48–1.42 (m, 1H), 1.34 (s, 9H); ^19^F NMR (471 MHz, DMSO-*d*_6_) *δ* −76.26; ^13^C NMR (151 MHz, CDCl_3_) *δ* 154.63, 134.38, 128.60, 127.31, 123.81 (q, *J* = 332.6 Hz), 79.96, 57.55, 49.67, 39.40, 38.59, 35.61, 33.93, 28.44; ESI-HR-MS: (*m*/*z*) calcd. for C_18_H_24_F_3_N_2_O_4_S ([M-H]^−^) 421.1414, found 421.1416.

*tert*-butyl 4-benzyl-4-(ethylsulfonamido)piperidine-1-carboxylate (**71-3**). Compound **71-3** was prepared according to General Procedure 1 from **70** (1.50 g, 5.17 mmol) using CH_3_CH_2_SO_2_Cl (0.80 g, 6.20 mmol) and Et_3_N (1.04 g, 10.3 mmol) in dried CH_2_Cl_2_ (15 mL). Purification by column chromatography (EtOAc/PE = 1/5, by *v*/*v*) gave **71-3**. White foam, 0.11 g (6%). ^1^H NMR (500 MHz, DMSO-*d*_6_) *δ* 7.32–7.27 (m, 4H), 7.23–7.20 (m, 1H), 6.67 (s, 1H), 3.62–3.58 (m, 2H), 3.08–3.00 (m, 2H), 1.82–1.79 (m, 2H), 1.45–1.39 (m, 2H), 1.36 (s, 9H), 1.22 (t, *J* = 7.3 Hz, 3H); ^13^C NMR (151 MHz, CDCl_3_) *δ* 154.60, 135.79, 131.22, 128.18, 126.78, 79.78, 58.43, 50.98, 45.70, 39.94, 30.24, 34.46, 28.38, 8.52; ESI-HR-MS: (*m*/*z*) calcd. for C_19_H_30_N_2_NaO_4_S ([M+Na]^+^) 405.1818, found 405.1815.

*tert*-butyl 4-acetamido-4-benzylpiperidine-1-carboxylate (**72-1**). Compound **72-1** was prepared according to General Procedure 1 from **70** (0.30 g, 1.03 mmol) using AcCl (97 mg, 1.24 mmol) and Et_3_N (0.21 g, 2.06 mmol) in dried CH_2_Cl_2_ (3 mL). Purification by column chromatography (EtOAc/PE = 1/5, by *v*/*v*) gave **72-1**. White foam, 0.18 g (52%). ^1^H NMR (500 MHz, DMSO-*d*_6_) *δ* 7.30–7.27 (m, 2H), 7.22–7.19 (m, 1H), 7.17 (s, 1H), 7.09–7.07 (m, 2H), 3.69–3.66 (m, 2H), 2.96 (s, 2H), 2.85 (s, 2H), 2.00–1.96 (m, 2H), 1.83 (s, 3H), 1.38 (s, 9H), 1.36–1.32 (m, 2H); ^13^C NMR (151 MHz, CDCl_3_) *δ* 170.68, 154.78, 136.82, 130.47, 127.98, 126.43, 79.58, 54.86, 43.18, 39.93, 38.96, 34.09, 28.39, 24.25; ESI-HR-MS: (*m*/*z*) calcd. for C_19_H_28_N_2_NaO_3_ ([M+Na]^+^) 355.1992, found 355.1990.

*tert*-butyl 4-benzyl-4-propionamidopiperidine-1-carboxylate (**72-2**). Compound **72-2** was prepared according to General Procedure 2 from **70** (1.50 g, 5.17 mmol) using (CH_3_CH_2_CO)_2_O (0.81 g, 6.20 mmol), DMAP (0.13 g, 1.03 mmol), and Et_3_N (1.04 g, 10.3 mmol) in dried CH_2_Cl_2_ (15 mL). Purification by column chromatography (EtOAc/PE = 1/5, by *v*/*v*) gave **72-2**. White foam, 1.28 g (72%). ^1^H NMR (500 MHz, DMSO-*d*_6_) *δ* 7.29–7.26 (m, 2H), 7.22–7.18 (m, 1H), 7.08–7.06 (m, 2H), 3.70–3.67 (m, 2H), 2.96 (s, 2H), 2.84 (s, 2H), 2.10–2.06 (m, 2H), 2.02-1.99 (m, 2H), 1.38 (s, 9H), 1.02 (t, *J* = 7.5 Hz, 3H); ^13^C NMR (151 MHz, CDCl_3_) *δ* 174.18, 154.54, 136.63, 130.32, 127.71, 126.20, 79.29, 54.33, 43.13, 39.76, 38.77, 33.88, 30.22, 28.19, 9.95; ESI-HR-MS: (*m*/*z*) calcd. for C_20_H_30_N_2_NaO_3_ ([M+Na]^+^) 369.2149, found 369.2146.

*tert*-butyl 4-benzyl-4-(cyclopropanecarboxamido)piperidine-1-carboxylate (**72-3**). Compound **72-3** was prepared according to General Procedure 1 from **70** (0.30 g, 1.03 mmol) using cyclopropanecarbonyl chloride (0.65 g, 6.20 mmol) and Et_3_N (1.04 g, 10.3 mmol) in dried CH_2_Cl_2_ (15 mL). Purification by column chromatography (EtOAc/PE = 1/5, by *v*/*v*) gave **72-3**. White foam, 0.65 g (35%). ^1^H NMR (500 MHz, DMSO-*d*_6_) *δ* 7.40 (s, 1H), 7.29–7.26 (m, 2H), 7.22–7.19 (m, 2H), 7.08–7.06 (m, 2H), 3.70–3.68 (m, 2H), 2.96 (s, 2H), 2.87 (s, 2H), 2.01–1.99 (m, 2H), 1.65–1.60 (m, 1H), 1.38 (s, 9H), 1.36–1.35 (m, 2H), 0.73–0.70 (m, 2H), 0.63–0.59 (m, 2H); ^13^C NMR (151 MHz, CDCl_3_) *δ* 173.78, 154.91, 136.85, 130.68, 128.02, 126.49, 79.65, 54.87, 43.46, 39.94, 39.01, 34.41, 28.46, 15.38, 6.87; ESI-HR-MS: (*m*/*z*) calcd. for C_21_H_31_N_2_O_3_ ([M+H]^+^) 359.2329, found 1359.2325.

*tert*-butyl 4-benzyl-4-pivalamidopiperidine-1-carboxylate (**72-4**). Compound **72-4** was prepared according to General Procedure 1 from **70** (0.20 g, 0.689 mmol) using pivaloyl chloride (0.12 g, 1.03 mmol) and Et_3_N (0.21 g, 2.07 mmol) in dried CH_2_Cl_2_ (2 mL). Purification by column chromatography (EtOAc/PE = 1/5, by *v*/*v*) gave **72-4**. White solid, 0.20 g (78%). m.p. 91.3–93.1 °C. ^1^H NMR (500 MHz, DMSO-*d*_6_) *δ* 7.27–7.24 (m, 2H), 7.21–7.17 (m, 1H), 7.07–7.05 (m, 2H), 6.22 (s, 1H), 3.71–3.68 (m, 2H), 2.95 (s, 2H), 2.79 (s, 2H), 2.14–2.11 (m, 1H), 1.38 (s, 9H), 1.35–1.29 (m, 2H), 1.09 (s, 9H); ^13^C NMR (126 MHz, CDCl_3_) *δ* 178.37, 154.89, 136.78, 130.77, 127.99, 126.60, 79.81, 54.19, 43.50, 39.56, 34.34, 28.52, 27.87, 27.19; ESI-HR-MS: (*m*/*z*) calcd. for C_22_H_35_N_2_O_3_ ([M+H]^+^) 375.2643, found 375.2650.

*tert*-butyl 4-benzyl-4-(2,2,2-trifluoroacetamido)piperidine-1-carboxylate (**72-5**). Compound **72-5** was prepared according to General Procedure 2 from **70** (0.30 g, 1.03 mmol) using (CF_3_CO)_2_O (0.26 g, 1.24 mmol), DMAP (25 mg, 0.206 mmol), and Et_3_N (0.21 g, 2.06 mmol) in dried CH_2_Cl_2_ (3 mL). Purification by column chromatography (EtOAc/PE = 1/5, by *v*/*v*) gave **72-5**. White foam, 0.24 g (60%). ^1^H NMR (500 MHz, DMSO-*d*_6_) *δ* 8.57 (s, 1H), 7.32–7.28 (m, 2H), 7.25–7.22 (m, 1H), 7.08–7.06 (m, 2H), 3.74–3.71 (m, 2H), 2.99 (s, 2H), 2.82 (s, 2H), 2.14–2.11 (m, 2H), 1.53–1.47 (m, 2H), 1.39 (s, 9H); ^13^C NMR (151 MHz, CDCl_3_) *δ* 156.94 (q, *J* = 36.3 Hz), 154.69, 135.40, 130.43, 128.45, 127.13, 115.53 (q, *J* = 289.6 Hz), 80.02, 56.49, 42.96, 39.78, 38.90, 33.81, 28.44; ^19^F NMR (471 MHz, DMSO-*d*_6_) *δ* −73.69; ESI-HR-MS: (*m*/*z*) calcd. for C_19_H_24_F_3_N_2_O_3_ ([M-H]^−^) 385.1745, found 385.1743.

*tert*-butyl 4-benzyl-4-(2,2-difluoroacetamido)piperidine-1-carboxylate (**72-6**). Compound **72-6** was prepared according to General Procedure 2 from **70** (0.10 g, 0.344 mmol) using (CHF_2_CO)_2_O (90 mg, 0.516 mmol), DMAP (8 mg, 0.069 mmol), and Et_3_N (0.10 g, 1.03 mmol) in dried CH_2_Cl_2_ (1 mL). Purification by column chromatography (EtOAc/PE = 1/5, by *v*/*v*) gave **72-6**. White solid, 0.10 g (80%). m.p. 87.9–90.4 °C. ^1^H NMR (500 MHz, DMSO-*d*_6_) *δ* 8.09 (s, 1H), 7.30–7.27 (m, 2H), 7.24–7.21 (m, 1H), 7.09–7.07 (m, 2H), 6.10 (t, *J* = 54.0 Hz, 1H), 3.73–3.70 (m, 2H), 2.99 (s, 2H), 2.83 (s, 2H), 2.05–2.02 (m, 2H), 1.51–1.43 (m, 2H), 1.38 (s, 9H); ^13^C NMR (126 MHz, CDCl_3_) *δ* 162.57 (t, *J* = 24.1 Hz), 154.74, 135.73, 130.49, 128.31, 126.94, 108.52 (t, *J* = 254.1 Hz), 79.91, 55.70, 43.22, 39.70, 38.96, 33.88, 28.44; ^19^F NMR (471 MHz, DMSO-*d*_6_) δ −124.41; ESI-HR-MS: (*m*/*z*) calcd. for C_19_H_26_N_2_F_2_NaO_3_ ([M+Na]^+^) 391.1804, found 391.1804.

*tert*-butyl 4-benzyl-4-((methoxycarbonyl)amino)piperidine-1-carboxylate (**72-7**). Compound **72-7** was prepared according to General Procedure 2 from **70** (0.50 g, 1.72 mmol) using (CH_3_OCO)_2_O (0.28 g, 2.06 mmol), DMAP (42 mg, 0.342 mmol), and Et_3_N (0.35 g, 3.42 mmol) in dried CH_2_Cl_2_ (5 mL). Purification by column chromatography (EtOAc/PE = 1/5, by *v*/*v*) gave **72-7**. White foam, 0.46 g (77%). ^1^H NMR (500 MHz, DMSO-*d*_6_) *δ* 7.29–7.26 (m, 2H), 7.22–7.19 (m, 1H), 7.07 (d, *J* = 7.5 Hz, 2H), 6.77 (s, 1H), 3.67–3.65 (m, 2H), 3.56 (s, 3H), 2.91 (br, 4H), 1.94–1.91 (m, 2H), 1.38 (s, 9H), 1.36–1.30 (m, 2H); ^13^C NMR (151 MHz, CDCl_3_) *δ* 155.62, 154.74, 136.55, 130.46, 128.02, 126.46, 79.46, 53.69, 51.70, 44.09, 39.77, 38.90, 34.23, 28.38; ESI-HR-MS: (*m*/*z*) calcd. for C_19_H_28_N_2_NaO_4_ ([M+Na]^+^) 371.1941, found 371.1939.

*tert*-butyl 4-benzyl-4-((ethoxycarbonyl)amino)piperidine-1-carboxylate (**72-8**). Compound **72-8** was prepared according to General Procedure 2 from **70** (0.50 g, 1.72 mmol) using (CH_3_CH_2_OCO)_2_O (0.33 g, 2.06 mmol), DMAP (42 mg, 0.342 mmol), and Et_3_N (0.35 g, 3.42 mmol) in dried CH_2_Cl_2_ (5 mL). Purification by column chromatography (EtOAc/PE = 1/5, by *v*/*v*) gave **72-8**. White foam, 0.45 g (72%). ^1^H NMR (500 MHz, DMSO-*d*_6_) *δ* 7.28–7.25 (m, 2H), 7.22–7.19 (m, 1H), 7.09–7.07 (m, 2H), 6.70 (s, 1H), 4.03–4.01 (m, 2H), 3.68–3.65 (m, 2H), 2.91 (br, 4H), 1.94 (br, 2H), 1.39–1.34 (s, 11H), 1.20 (t, *J* = 7.0 Hz, 3H); ^13^C NMR (151 MHz, CDCl_3_) *δ* 155.23, 154.76, 136.61, 130.49, 128.00, 126.45, 79.44, 60.28, 53.64, 44.10, 39.76, 38.88, 34.29, 28.40, 14.70; ESI-HR-MS: (*m*/*z*) calcd. for C_20_H_30_N_2_NaO_4_ ([M+Na]^+^) 385.2098, found 385.2096.

General Procedure 3: Synthesis of **C-1-1** to **C-1-3** and **C-2-1** to **C-2-8**. To a stirred solution of **72-1** to **72-3** or **72-2-1** to **72-2-8** (1.0 eq) in CH_2_Cl_2_, cooled to 0 °C, TFA (about 1.67 *v*/*v* based on CH_2_Cl_2_) was added dropwise. After addition, the reaction mixture was stirred at room temperature overnight until TLC analysis indicated completion of the reaction, and then evaporated on a rotary evaporator to afford a residue. Another flask, charged with **7·TFA** (1.2 eq), EDCI (2.5 eq), HOBT (0.075 eq), DIPEA (6 eq), and dried THF, was stirred at 0 °C, followed by dropwise addition of a solution of the residue obtained above in a small amount of dried THF. After addition, the resulting mixture was stirred at room temperature overnight until TLC analysis indicated completion of the reaction, and then poured into water. The mixture thus obtained was extracted with EtOAc in three portions, and the combined extracts were washed with brine, dried (MgSO_4_), and evaporated on a rotary evaporator to afford a residue, which was purified by column chromatography to afford the target products.

N-(1-([2,4′-bipyridine]-3-carbonyl)-4-benzylpiperidin-4-yl)methanesulfonamide (**C-1-1**). Following General Procedure 3, **71-1** (0.90 g, 2.44 mmol) was first treated with TFA (4.5 mL) in CH_2_Cl_2_ (2.7 mL), and the intermediate obtained was reacted with **7·TFA** (0.87 g, 2.93 mmol) using EDCI (1.22 g, 6.34 mmol), HOBT (25 mg, 0.183 mmol), and DIPEA (1.89 g, 14.6 mmol) in dried THF (9 mL). Purification by column chromatography (EtOAc) gave **C-1-1**. White foam, 0.55 g (50%). ^1^H NMR (500 MHz, DMSO-*d*_6_) *δ* 8.79–8.67 (m, 3H), 7.87–7.84 (m, 1H), 7.60–7.56 (m, 3H), 7.30–7.14 (m, 5H), 6.73–6.69 (m, 1H), 4.26–4.05 (m, 1H), 3.13–2.88 (m, 7H), 2.82–2.79 (m, 1H), 2.60–2.58 (m, 0.58H), 1.95–1.76 (m, 1H), 1.54–1.41 (m, 1.66H), 1.23–1.17 (m, 0.59H), 0.29–0.26 (m, 1H); ^13^C NMR (151 MHz, CDCl_3_ + CD_3_OD) *δ* 167.36, 167.19, 151.47, 151.37, 150.38, 150.30, 149.31, 146.48, 146.30, 136.10, 135.36, 135.12, 131.07, 130.93, 130.80, 130.73, 130.57, 127.97, 126.72, 126.65, 123.75, 123.66, 123.25, 123.12, 57.74, 57.64, 46.34, 45.49, 44.28, 42.78, 42.45, 37.42, 37.33, 33.86, 33.71, 33.46, 33.26; ESI-HR-MS: (*m*/*z*) calcd. for C_24_H_27_N_4_O_3_S ([M+H]^+^) 451.1798, found 451.1810.

N-(1-([2,4′-bipyridine]-3-carbonyl)-4-benzylpiperidin-4-yl)-1,1,1-trifluoromethanesulfonamide (**C-1-2**). Following General Procedure 3, **71-2** (1.10 g, 2.60 mmol) was first treated with TFA (5.5 mL) in CH_2_Cl_2_ (3.3 mL), and the intermediate obtained was reacted with **7·TFA** (0.93 g, 3.12 mmol) using EDCI (1.25 g, 6.50 mmol), HOBT (26 mg, 0.195 mmol), and DIPEA (2.02 g, 15.6 mmol) in dried THF (11 mL). Purification by column chromatography (EtOAc) gave **C-1-2**. White foam, 0.64 g (49%). ^1^H NMR (500 MHz, DMSO-*d*_6_) *δ* 8.78 (s, 1H), 8.65–8.56 (m, 2H), 7.87–7.84 (m, 1H), 7.55–7.51 (m, 3H), 7.34–7.11 (m, 6H), 4.26–4.14 (m, 1H), 3.06–2.92 (m, 2H), 2.88–2.72 (m, 2H), 2.66–2.54 (m, 1H), 2.02–1.91 (m, 0.69H), 1.78–1.52 (m, 1.78H), 1.38–1.23 (m, 1.60H), 0.71–0.42 (m, 0.45H); ^13^C NMR (151 MHz, CDCl_3_) *δ* 167.61, 167.34, 167.28, 152.16, 151.59, 151.51, 150.66, 150.63, 150.05, 150.03, 149.92, 146.30, 146.27, 146.08, 136.28, 136.19, 136.06, 133.89, 133.67, 133.59, 131.14, 131.08, 130.84, 128.57, 128.52, 127.39, 127.33, 123.63, 123.56, 123.44, 122.72, 122.68, 57.23, 57.10, 57.02, 56.93, 49.75, 49.67, 49.54, 42.23, 42.13, 41.61, 41.47, 36.90, 36.71, 35.39, 34.96, 34.80, 34.39, 33.69, 33.37, 33.01, 32.93; ^19^F NMR (471 MHz, DMSO-*d*_6_) *δ* −76.07, −76.15, −76.38, −76.42; ESI-HR-MS: (*m*/*z*) calcd. for C_24_H_24_F_3_N_4_O_3_S ([M+H]^+^) 505.1516, found 505.1516.

N-(1-([2,4′-bipyridine]-3-carbonyl)-4-benzylpiperidin-4-yl)ethanesulfonamide (**C-1-3**). Following General Procedure 3, **71-3** (0.60 g, 1.57 mmol) was first treated with TFA (3 mL) in CH_2_Cl_2_ (1.8 mL), and the intermediate obtained was reacted with **7·TFA** (0.56 g, 1.88 mmol) using EDCI (0.75 g, 3.93 mmol), HOBT (16 mg, 0.118 mmol), and DIPEA (1.22 g, 9.42 mmol) in dried THF (11 mL). Purification by column chromatography (EtOAc) gave **C-1-3**. White foam, 0.33 g (45%). ^1^H NMR (500 MHz, DMSO-*d*_6_) *δ* 8.78–8.77 (m, 1H), 8.68–8.66 (m, 2H), 7.87–7.85 (m, 1H), 7.59–7.55 (m, 3H), 7.28–7.15 (m, 5H), 6.64–6.58 (m, 1H), 4.24–4.03 (m, 1H), 3.09–2.89 (m, 5H), 2.88–2.80 (m, 1.4H), 2.67–2.55 (m, 1H), 1.95–1.74 (m, 1H), 1.52–1.40 (m, 2H), 1.22–1.17 (m, 2H), 1.10–1.07 (m, 1H), 0.28–0.23 (m, 0.52H); ^13^C NMR (151 MHz, CDCl_3_) *δ* 167.54, 167.37, 151.92, 151.81, 150.71, 150.65, 150.11, 150.04, 146.32, 146.19, 136.26, 136.22, 135.25, 135.01, 131.17, 131.12, 131.00, 128.36, 127.07, 123.67, 123.61, 123.35, 123.07, 58.12, 58.07, 51.04, 50.99, 46.28, 45.40, 42.90, 42.48, 37.64, 37.58, 34.37, 34.30, 33.91, 33.66, 8.51; ESI-HR-MS: (*m*/*z*) calcd. for C_25_H_29_N_4_O_3_S ([M+H]^+^) 465.1955, found 465.1954.

N-(1-([2,4′-bipyridine]-3-carbonyl)-4-benzylpiperidin-4-yl)acetamide (**C-2-1**). Following General Procedure 3, **72-1** (0.63 g, 1.90 mmol) was first treated with TFA (9.5 mL) in CH_2_Cl_2_ (5.7 mL), and the intermediate obtained was reacted with **7·TFA** (0.68 g, 2.28 mmol) using EDCI (0.91 g, 4.75 mmol), HOBT (19 mg, 0.143 mmol), and DIPEA (1.47 g, 11.4 mmol) in dried THF (6.3 mL). Purification by column chromatography (EtOAc) gave **C-2-1**. White foam, 0.35 g (45%). ^1^H NMR (500 MHz, DMSO-*d*_6_) *δ* 8.79–8.65 (m, 3H), 7.93–7.83 (m, 1H), 7.67–7.54 (m, 3H), 7.29–7.14 (m, 4H), 7.04–6.90 (m, 2H), 4.32–4.21 (m, 1H), 3.34 (s, 1H), 3.05–2.64 (m, 4.66H), 2.46–2.17 (m, 1H), 2.04–2.01 (m, 0.43H), 1.79–1.76 (m, 3H), 1.50–1.41 (m, 1H), 1.10–1.05 (d, 0.51H), 0.07–0.00 (m, 0.52H); ^13^C NMR (151 MHz, CDCl_3_) *δ* 170.88, 170.78, 167.37, 167.19, 151.79, 151.51, 150.52, 150.45, 149.85, 149.79, 146.33, 146.26, 136.25, 136.17, 135.98, 135.94, 131.34, 131.11, 130.32, 130.27, 128.06, 128.03, 126.60, 126.56, 123.58, 123.54, 123.48, 122.94, 54.68, 54.44, 43.23, 42.95, 42.82, 42.42, 37.38, 33.92, 33.89, 33.42, 33.11, 24.11; ESI-HR-MS: (*m*/*z*) calcd. for C_25_H_27_N_4_O_2_ ([M+H]^+^) 415.2129, found 415.2138.

N-(1-([2,4′-bipyridine]-3-carbonyl)-4-benzylpiperidin-4-yl)propionamide (**C-2-2**). Following General Procedure 3, **72-2** (1.23 g, 3.55 mmol) was first treated with TFA (6.2 mL) in CH_2_Cl_2_ (3.7 mL), and the intermediate obtained was reacted with **7·TFA** (1.27 g, 4.26 mmol) using EDCI (1.70 g, 8.88 mmol), HOBT (36 mg, 0.266 mmol), and DIPEA (2.75 g, 21.3 mmol) in dried THF (12.3 mL). Purification by column chromatography (EtOAc) gave **C-2-2**. White foam, 0.71 g (47%). ^1^H NMR (500 MHz, DMSO-*d*_6_) *δ* 8.79–8.77 (m, 2H), 8.65–8.64 (m, 1H), 7.93–7.83 (m, 1H), 7.68–7.66 (m, 1H), 7.58–7.53 (m, 2H), 7.27–7.24 (m, 2H), 7.20–7.17 (m, 1H), 7.08–7.01 (m, 2H), 6.91–6.89 (m, 1H), 4.32–4.21 (m, 1H), 3.02–2.67 (m, 5H), 2.46–2.18 (m, 1H), 2.05–2.00 (m, 2H), 1.84–1.81 (m, 0.37H), 1.53–1.49 (m, 1H), 1.42–1.03 (m, 1H), 1.00–0.94 (m, 3H), 0.06–0.01 (m, 0.54H); ^13^C NMR (151 MHz, CDCl_3_) *δ* 174.24, 167.12, 166.84, 151.47, 151.14, 150.27, 150.22, 149.51, 146.07, 136.06, 135.98, 135.69, 131.10, 130.87, 130.16, 130.10, 127.74, 126.33, 123.35, 123.29, 122.67, 54.13, 53.86, 43.11, 42.89, 42.58, 42.17, 37.19, 33.66, 33.62, 33.28, 32.76, 29.99, 9.74; ESI-HR-MS: (*m*/*z*) calcd. for C_26_H_29_N_4_O_2_ ([M+H]^+^) 429.2285, found 429.2285.

N-(1-([2,4′-bipyridine]-3-carbonyl)-4-benzylpiperidin-4-yl)cyclopropanecarboxamide (**C-2-3**). Following General Procedure 3, **72-3** (0.60 g, 1.67 mmol) was first treated with TFA (3 mL) in CH_2_Cl_2_ (1.8 mL), and the intermediate obtained was reacted with **7·TFA** (0.60 g, 2.00 mmol) using EDCI (0.80 g, 4.18 mmol), HOBT (17 mg, 0.125 mmol), and DIPEA (1.29 g, 10.0 mmol) in dried THF (6 mL). Purification by column chromatography (EtOAc) gave **C-2-3**. White foam, 0.45 g (61%). ^1^H NMR (500 MHz, DMSO-*d*_6_) *δ* 8.79–8.65 (m, 3H), 7.94–7.84 (m, 1H), 7.67–7.54 (m, 3H), 7.41–7.37 (m, 1H), 7.28–7.18 (m, 3H), 7.04–6.90 (m, 2H), 4.32–4.22 (m, 1H), 3.07–2.67 (m, 4.40H), 2.46–1.80 (m, 2H), 1.59–1.44 (m, 2.42H), 1.12–1.07 (m, 0.52H), 0.72–0.58 (m, 4H), 0.12–0.06 (m, 0.53H); ^13^C NMR (151 MHz, CDCl_3_) *δ* 173.99, 173.88, 167.10, 166.88, 151.53, 151.15, 150.25, 150.21, 149.52, 146.10, 136.11, 136.02, 135.77, 135.64, 131.16, 130.93, 130.28, 130.19, 127.78, 126.33, 123.38, 123.32, 122.71, 54.44, 54.10, 43.26, 43.10, 42.62, 42.32, 37.25, 33.95, 33.86, 33.43, 32.92, 14.71, 14.64, 6.69, 6.59, 6.53; ESI-HR-MS: (*m*/*z*) calcd. for C_27_H_29_N_4_O_2_ ([M+H]^+^) 441.2285, found 441.2284.

N-(1-([2,4′-bipyridine]-3-carbonyl)-4-benzylpiperidin-4-yl)pivalamide (**C-2-4**). Following General Procedure 3, **72-4** (1.50 g, 4.00 mmol) was first treated with TFA (7.5 mL) in CH_2_Cl_2_ (4.5 mL), and the intermediate obtained was reacted with **7·TFA** (1.43 g, 4.80 mmol) using EDCI (1.92 g, 10.0 mmol), HOBT (41 mg, 0.300 mmol), and DIPEA (3.10 g, 24.0 mmol) in dried THF (15 mL). Purification by column chromatography (EtOAc) gave **C-2-4**. White foam, 0.30 g (16%). ^1^H NMR (500 MHz, DMSO-*d*_6_) *δ* 8.79–8.77 (m, 2H), 8.63–8.62 (m, 1H), 7.93–7.84 (m, 1H), 7.68–7.67 (m, 1H), 7.59–7.51 (m, 2H), 7.26–7.17 (m, 3H), 7.00–6.88 (m, 2H), 6.23–6.14 (m, 1H), 4.33–4.22 (m, 1H), 2.95–2.55 (m, 4H), 2.47–2.10 (m, 2H), 1.93–1.65 (m, 1H), 1.46–1.24 (m, 1.47H), 1.06–1.00 (m, 9H), 0.04–0.01 (m, 0.53H); ^13^C NMR (126 MHz, CDCl_3_) *δ* 178.50, 167.82, 152.04, 150.74, 150.10, 149.89, 146.57, 136.34, 136.16, 131.33, 130.63, 128.12, 126.84, 123.70, 123.22, 53.92, 43.44, 43.11, 42.44, 39.56, 37.60, 34.17, 33.80, 33.59, 31.70, 27.80, 22.77, 14.24; ESI-HR-MS: (*m*/*z*) calcd. for C_28_H_33_N_4_O_2_ ([M+H]^+^) 457.2598, found 457.2601.

N-(1-([2,4′-bipyridine]-3-carbonyl)-4-benzylpiperidin-4-yl)-2,2,2-trifluoroacetamide (**C-2-5**). Following General Procedure 3, **72-5** (0.90 g, 2.33 mmol) was first treated with TFA (4.5 mL) in CH_2_Cl_2_ (2.7 mL), and the intermediate obtained was reacted with **7·TFA** (0.84 g, 2.80 mmol) using EDCI (1.12 g, 5.83 mmol), HOBT (24 mg, 0.175 mmol), and DIPEA (1.81 g, 14.0 mmol) in dried THF (15 mL). Purification by column chromatography (EtOAc) gave **C-2-5**. White foam, 0.66 g (60%). ^1^H NMR (500 MHz, DMSO-*d*_6_) *δ* 8.80–8.52 (m, 4H), 7.96–7.85 (m, 1H), 7.67–7.53 (m, 3H), 7.30–7.22 (m, 3H), 7.03–6.91 (m, 2H), 4.33–4.24 (m, 1H), 3.35–3.34 (m, 0.53H), 3.11–2.54 (m, 5H), 2.30–2.15 (m, 1H), 1.90–1.71 (m, 1H), 1.66–1.56 (m, 0.75H), 1.25–0.22 (m, 1H); ^13^C NMR (151 MHz, CDCl_3_) *δ* 171.27, 167.41, 167.20, 157.64 (q, *J* = 35.8 Hz), 151.75, 151.37, 150.61, 150.58, 149.68, 149.46, 146.28, 136.05, 135.91, 135.03, 134.92, 131.07, 130.82, 130.26, 130.20, 128.29, 127.01, 123.61, 123.50, 122.84, 115.37 (q, *J* = 289.6 Hz), 60.35, 56.13, 55.91, 43.02, 42.76, 42.63, 42.21, 37.26, 33.38, 33.25, 32.87, 32.59, 20.86, 14.05; ^19^F NMR (471 MHz, DMSO-*d*_6_) *δ* −73.73, −73.76; ESI-HR-MS: (*m*/*z*) calcd. for C_25_H_24_F_3_N_4_O_2_ ([M+H]^+^) 469.1846, found 469.1856.

N-(1-([2,4′-bipyridine]-3-carbonyl)-4-benzylpiperidin-4-yl)-2,2-difluoroacetamide (**C-2-6**). Following General Procedure 3, **72-6** (0.10 g, 0.271 mmol) was first treated with TFA (0.5 mL) in CH_2_Cl_2_ (0.3 mL), and the intermediate obtained was reacted with **7·TFA** (97 mg, 0.325 mmol) using EDCI (0.13 g, 0.676 mmol), HOBT (3 mg, 0.020 mmol), and DIPEA (0.21 g, 1.63 mmol) in dried THF (5 mL). Purification by column chromatography (EtOAc) gave **C-2-6**. White solid, 21 mg (17%). m.p. 97.9–99.4 °C. ^1^H NMR (500 MHz, DMSO-*d*_6_) *δ* 8.80–8.78 (m, 2H), 8.65 (s, 1H), 8.10–8.06 (m, 1H), 7.95–7.84 (m, 1H), 7.67–7.54 (m, 3H), 7.29–7.20 (m, 3H), 7.04–6.91 (m, 2H), 6.17–5.92 (m, 1H), 4.34–4.24 (m, 1H), 3.11–3.08 (m, 0.44H), 2.97–2.94 (m, 2H), 2.84–2.63 (m, 2H), 2.22–2.07 (m, 1H), 1.83–1.53 (m, 2H), 1.27–1.15 (m, 2H), 0.87–0.84 (m, 1H), 0.16–0.12 (m, 0.53H); ^13^C NMR (126 MHz, CDCl_3_) *δ* 167.56, 162.89, 162.70, 152.24, 151.85, 150.77, 150.28, 150.20, 146.38, 136.34, 136.23, 135.06, 131.37, 131.09, 130.44, 130.36, 128.51, 128.48, 127.22, 123.74, 123.65, 123.02, 110.58, 108.56, 108.38, 106.54, 55.56, 55.41, 43.50, 43.08, 42.82, 42.37, 37.43, 33.83, 33.33, 33.19, 29.81; ^19^F NMR (471 MHz, DMSO-*d*_6_) *δ* −124.47; ESI-HR-MS: (*m*/*z*) calcd. for C_25_H_25_F_2_N_4_O_2_ ([M+H]^+^) 451.1940, found 451.1940.

methyl (1-([2,4′-bipyridine]-3-carbonyl)-4-benzylpiperidin-4-yl)carbamate (**C-2-7**). Following General Procedure 3, **72-7** (0.44 g, 1.27 mmol) was first treated with TFA (2.2 mL) in CH_2_Cl_2_ (1.3 mL), and the intermediate obtained was reacted with **7·TFA** (0.45 g, 1.52 mmol) using EDCI (0.61 g, 3.18 mmol), HOBT (13 mg, 0.095 mmol), and DIPEA (0.98 g, 7.62 mmol) in dried THF (4.4 mL). Purification by column chromatography (EtOAc) gave **C-2-7**. White foam, 0.13 g (24%). ^1^H NMR (500 MHz, DMSO-*d*_6_) *δ* 8.79–8.65 (m, 3H), 7.92–7.83 (m, 1H), 7.67–7.54 (m, 3H), 7.28–7.18 (m, 3H), 7.03–6.89 (m, 2H), 6.76–6.74 (m, 1H), 4.31–4.20 (m, 1H), 3.53 (s, 3H), 3.03–2.69 (m, 4.50H), 2.40–2.38 (m, 0.50H), 2.13–1.95 (m, 1H), 1.74–1.40 (m, 2H), 1.13–0.04 (m, 1H); ^13^C NMR (151 MHz, CDCl_3_) *δ* 167.20, 167.09, 155.66, 151.84, 151.47, 150.38, 150.32, 149.83, 149.77, 146.15, 136.00, 135.94, 135.89, 135.85, 131.37, 131.06, 130.25, 130.18, 128.02, 127.98, 126.56, 126.50, 123.45, 123.41, 122.75, 53.55, 53.25, 51.70, 44.06, 43.94, 42.64, 42.27, 37.26, 34.15, 33.96, 33.55, 33.12; ESI-HR-MS: (*m*/*z*) calcd. for C_25_H_27_N_4_O_3_ ([M+H]^+^) 431.2078, found 431.2077.

ethyl (1-([2,4′-bipyridine]-3-carbonyl)-4-benzylpiperidin-4-yl)carbamate (**C-2-8**). Following General Procedure 3, **72-8** (0.95 g, 2.64 mmol) was first treated with TFA (13.2 mL) in CH_2_Cl_2_ (7.9 mL), and the intermediate obtained was reacted with **7·TFA** (0.95 g, 3.17 mmol) using EDCI (1.27 g, 6.60 mmol), HOBT (27 mg, 0.198 mmol), and DIPEA (2.04 g, 15.8 mmol) in dried THF (9.5 mL). Purification by column chromatography (EtOAc) gave **C-2-8**. White foam, 0.64 g (55%). ^1^H NMR (500 MHz, DMSO-*d*_6_) *δ* 8.79–8.65 (m, 3H), 7.92–7.82 (m, 1H), 7.67–7.54 (m, 3H), 7.27–7.18 (m, 3H), 7.05–6.90 (m, 2H), 6.70–6.67 (m, 1H), 4.31–4.20 (m, 1H), 4.03–3.95 (m, 2H), 3.03–2.69 (m, 4.5H), 2.41–2.37 (m, 0.54H), 2.15–1.98 (m, 1H), 1.77–1.39 (m, 2H), 1.18–1.15 (m, 3H), 1.10–1.07 (m, 0.58H), 0.09–0.02 (m, 0.51H); ^13^C NMR (151 MHz, CDCl_3_) *δ* 167.01, 166.88, 155.16, 151.63, 151.24, 150.19, 150.14, 149.61, 149.54, 145.99, 135.89, 135.83, 135.81, 135.69, 131.23, 130.93, 130.12, 130.05, 127.81, 127.77, 126.35, 126.30, 123.31, 123.26, 122.58, 60.07, 53.36, 53.02, 43.88, 42.47, 42.15, 37.11, 34.03, 33.83, 33.42, 32.92, 14.44, 14.41; ESI-HR-MS: (*m*/*z*) calcd. for C_26_H_29_N_4_O_3_ ([M+H]^+^) 445.2234, found 445.2234.

Synthesis of *tert*-butyl 4-(aminomethyl)-4-benzylpiperidine-1-carboxylate (**73**). To a stirred solution of **68** (10.00 g, 33.3 mmol) in MeOH (100 mL), cooled to 0 °C under N_2_, NiCl_2_·6H_2_O (15.8 g, 66.6 mmol) in one portion and NaBH_4_ (12.6 g, 333 mmol) in portions were added successively. After addition, the reaction mixture was stirred at room temperature until completion, as indicated by TLC analysis (typically within 5 h), filtered through Celite, and poured into ice–water (100 mL). The resulting mixture was extracted with EtOAc (150 mL × 3), and the combined extracts were washed with brine, dried (MgSO_4_), and evaporated on a rotary evaporator to afford a residue, which was purified by column chromatography (MeOH/CH_2_Cl_2_ = 1/10, by *v*/*v*) to give **73**. Colorless oil, 8.88 g (88%). ^1^H NMR (500 MHz, DMSO-*d*_6_) *δ* 7.28–7.25 (m, 2H), 7.20–7.16 (m, 3H), 3.45–3.40 (m, 2H), 3.27–3.20 (m, 2H), 2.63 (s, 2H), 2.38 (s, 2H), 1.37 (s, 9H), 1.33–1.28 (m, 2H), 1.25–1.19 (m, 2H); ^13^C NMR (126 MHz, CDCl_3_) *δ* 155.05, 137.68, 130.52, 128.16, 126.30, 79.45, 46.05, 41.13, 39.91, 39.24, 36.65, 32.16, 28.51; ESI-HR-MS: (*m*/*z*) calcd. for C_18_H_29_N_2_O_2_ ([M+H]^+^) 305.2224 found 305.2221.

Synthesis of *tert*-butyl 4-(acetamidomethyl)-4-benzylpiperidine-1-carboxylate (**74-1**). Compound **74-1** was prepared according to General Procedure 1 described above from **73** (0.80 g, 2.63 mmol) using AcCl (0.25 g, 3.16 mmol) and Et_3_N (0.80 g, 7.89 mmol) in dried CH_2_Cl_2_ (8 mL). Purification by column chromatography (EtOAc/PE = 1/3, by *v*/*v*) gave **74-1**. White solid, 0.85 g (95%). m.p. 48.2–50.5 °C. ^1^H NMR (500 MHz, DMSO-*d*_6_) *δ* 7.26 (t, *J* = 6.0 Hz, 1H), 7.29–7.26 (m, 2H), 7.22–7.17 (m, 3H), 3.46–3.41 (m, 2H), 3.26 (s, 2H), 3.02 (d, *J* = 6.0 Hz, 2H), 2.61 (s, 2H), 1.89 (s, 3H), 1.35 (s, 9H), 1.26–1.17 (m, 4H); ^13^C NMR (126 MHz, CDCl_3_) *δ* 170.23, 155.04, 137.49, 130.38, 128.58, 126.83, 79.57, 44.19, 44.00, 36.89, 32.59, 28.56, 23.47; ESI-HR-MS: (*m*/*z*) calcd. for C_20_H_30_N_2_NaO_3_ ([M+Na]^+^) 369.2149, found 369.2145.

Synthesis of *tert*-butyl 4-benzyl-4-(cyclopropanecarboxamidomethyl)piperidine-1-carboxylate (**74-2**). Compound **74-2** was prepared according to General Procedure 1 described above from **73** (0.80 g, 2.63 mmol) using cyclopropanecarbonyl chloride (0.33 g, 3.16 mmol) and Et_3_N (0.80 g, 7.89 mmol) in dried CH_2_Cl_2_ (8 mL). Purification by column chromatography (EtOAc/PE = 1/3, by *v*/*v*) gave **74-2**. White solid, 0.85 g (95%). White solid, 0.70 g (71%). m.p 66.6–69.2 °C. ^1^H NMR (500 MHz, CDCl_3_) *δ* 7.33–7.30 (m, 2H), 7.27–7.24 (m, 1H), 7.16–7.14 (m, 2H), 5.48 (t, *J* = 6.3 Hz, 1H), 3.59–3.56 (m, 2H), 3.39–3.17 (m, 4H), 2.65 (s, 2H), 1.44 (s, 9H), 1.43–1.41 (m, 3H), 1.21–1.16 (m, 1H), 0.91–0.88 (m, 2H), 0.71–0.67 (m, 2H); ^13^C NMR (126 MHz, CDCl_3_) *δ* 173.79, 155.03, 137.52, 130.42, 128.52, 126.74, 79.48, 44.08, 43.96, 36.98, 32.51, 28.54, 14.84, 7.22; ESI-HR-MS: (*m*/*z*) calcd. for C_22_H_32_N_2_NaO_3_ ([M+Na]^+^) 395.2305, found 395.2304.

Synthesis of *tert*-butyl 4-benzyl-4-(pivalamidomethyl)piperidine-1-carboxylate (**74-3**). Compound **74-3** was prepared according to General Procedure 1 described above from **73** (0.80 g, 2.63 mmol) using pivaloyl chloride (0.38 g, 3.16 mmol) and Et_3_N (0.80 g, 7.89 mmol) in dried CH_2_Cl_2_ (8 mL). Purification by column chromatography (EtOAc/PE = 1/3, by *v*/*v*) gave **74-3**. White solid, 0.95 g (90%). m.p. 66–69 °C. ^1^H NMR (500 MHz, DMSO-*d*_6_) *δ* 7.30–7.27 (m, 2H), 7.22–7.21 (m, 1H), 7.18–7.17 (m, 2H), 7.06 (t, *J* = 6.3 Hz, 1H), 3.36 (s, 4H), 3.08 (d, *J* = 6.0 Hz, 2H), 2.63 (s, 2H), 1.34 (s, 9H), 1.29–1.24 (m, 2H), 1.19–1.14 (m, 2H), 1.08 (s, 9H); ^13^C NMR (151 MHz, CDCl_3_) *δ* 178.15, 154.74, 137.78, 130.02, 130.00, 128.66, 128.60, 126.73, 79.19, 45.50, 43.51, 43.49, 39.98, 38.94, 38.52, 37.17, 37.13, 32.78, 28.36, 28.33, 27.28, 27.25; ESI-HR-MS: (*m*/*z*) calcd. for C_23_H_36_N_2_NaO_3_ ([M+Na]^+^) 411.2618, found 411.2618.

Synthesis of *tert*-butyl 4-benzyl-4-((2,2,2-trifluoroacetamido)methyl)piperidine-1-carboxylate (**74-4**). Compound **74-4** was prepared according to General Procedure 2 from **73** (0.80 g, 2.63 mmol) using (CF_3_CO)_2_O (0.66 g, 3.16 mmol), DMAP (65 mg, 0.53 mmol), and Et_3_N (0.80 g, 7.89 mmol) in dried CH_2_Cl_2_ (8 mL). Purification by column chromatography (EtOAc/PE = 1/3, by *v*/*v*) gave **74-4**. White solid, 0.80 g (73%). m.p. 66–69 °C. ^1^H NMR (500 MHz, DMSO-*d*_6_) *δ* 9.29 (t, *J* = 6.3 Hz, 1H), 7.30–7.27 (m, 2H), 7.23–7.17 (m, 3H), 3.41–3.37 (m, 4H), 3.20 (d, *J* = 6.0 Hz, 2H), 2.67 (s, 2H), 1.34 (s, 9H), 1.31–1.26 (m, 2H), 1.23–1.18 (m, 2H); ^13^C NMR (151 MHz, CDCl_3_) *δ* 157.53 (q, *J* = 36.8 Hz), 154.87, 136.91, 130.07, 128.81, 127.14, 115.83 (q, *J* = 288.2 Hz), 79.69, 44.85, 43.93, 39.90, 38.95, 37.06, 32.41, 28.42; ^19^F NMR (471 MHz, DMSO-*d*_6_) *δ* −73.79; ESI-HR-MS: (*m*/*z*) calcd. for C_20_H_27_F_3_N_2_NaO_3_ ([M+Na]^+^) 423.1866, found 423.1866.

Synthesis of *tert*-butyl 4-benzyl-4-((2,2-difluoroacetamido)methyl)piperidine-1-carboxylate (**74-5**). Compound **74-5** was prepared according to General Procedure 2 from **73** (0.80 g, 2.63 mmol) using (CHF_2_CO)_2_O (0.55 g, 3.16 mmol), DMAP (65 mg, 0.53 mmol), and Et_3_N (0.80 g, 7.89 mmol) in dried CH_2_Cl_2_ (8 mL). Purification by column chromatography (EtOAc/PE = 1/3, by *v*/*v*) gave **74-5**. White solid, 0.70 g (70%). m.p 66–69 °C. ^1^H NMR (500 MHz, DMSO-*d*_6_) *δ* 8.67 (t, *J* = 6.3 Hz, 1H), 7.30–7.27 (m, 2H), 7.23–7.17 (m, 3H), 6.27 (t, *J* = 53.8 Hz, 1H), 3.44–3.40 (m, 2H), 3.33 (s, 2H), 3.14 (d, *J* = 6.0 Hz, 2H), 2.65 (s, 2H), 1.35 (s, 9H), 1.31–1.26 (m, 2H), 1.24–1.19 (m, 2H); ^13^C NMR (151 MHz, CDCl_3_) *δ* 146.35 (t, *J* = 24.5 Hz), 154.88, 136.91, 130.13, 128.70, 127.00, 108.44 (t, *J* = 168.4 Hz), 79.60, 43.88, 43.81, 39.92, 38.97, 36.94, 32.43, 28.44; ^19^F NMR (471 MHz, DMSO-*d*_6_) *δ* −125.00; ESI-HR-MS: (*m*/*z*) calcd. for C_20_H_28_F_2_N_2_NaO_3_ ([M+Na]^+^) 405.1960, found 405.1962.

Synthesis of *tert*-butyl 4-benzyl-4-((furan-2-carboxamido)methyl)piperidine-1-carboxylate (**74-6**). Compound **74-6** was prepared according to General Procedure 1 described above from **73** (0.80 g, 2.63 mmol) using furan-2-carbonyl chloride (0.41 g, 3.16 mmol) and Et_3_N (0.80 g, 7.89 mmol) in dried CH_2_Cl_2_ (8 mL). Purification by column chromatography (EtOAc/PE = 1/3, by *v*/*v*) gave **74-6**. Pale yellow foam, 0.92 g (88%). m.p. 66–69 °C. ^1^H NMR (500 MHz, DMSO-*d*_6_) *δ* 8.12 (t, *J* = 6.3 Hz, 1H), 7.83–7.82 (m, 1H), 7.30–7.27 (m, 2H), 7.22–7.19 (m, 3H), 7.14 (d, *J* = 3.5 Hz, 1H), 6.62–6.61 (m, 1H), 3.44–3.46 (m, 4H), 3.26 (d, *J* = 6.5 Hz, 2H), 2.68 (s, 2H), 1.34–1.33 (m, 2H), 1.32 (s, 9H), 1.25–1.19 (m, 2H); ^13^C NMR (151 MHz, CDCl_3_) *δ* 158.44, 154.83, 147.66, 143.80, 137.15, 130.20, 128.43, 126.56, 114.08, 112.06, 79.30, 43.89, 43.14, 39.95, 38.98, 37.05, 32.37, 28.34; ESI-HR-MS: (*m*/*z*) calcd. for C_23_H_30_N_2_NaO_4_ ([M+Na]^+^) 421.2098, found 421.2100.

Synthesis of N-((1-([2,4′-bipyridine]-3-carbonyl)-4-benzylpiperidin-4-yl)methyl)acetamide (**C-3-1**). Following General Procedure 3, **74-1** (0.85 g, 2.45 mmol) was first treated with TFA (4.3 mL) in CH_2_Cl_2_ (2.6 mL), and the intermediate obtained was reacted with **7·TFA** (0.88 g, 2.94 mmol) using EDCI (1.18 g, 6.13 mmol), HOBT (25 mg, 0.184 mmol), and DIPEA (1.90 g, 14.7 mmol) in dried THF (8.5 mL). Purification by column chromatography (EtOAc) gave **C-3-1**. White solid, 0.15 g (14%). m.p. 146.3–148.5 °C. ^1^H NMR (500 MHz, CD_3_OD) *δ* 8.77 (dd, *J* = 1.5 Hz and 5.0 Hz, 1H), 8.67–8.58 (m, 2H), 7.87–7.80 (m, 1H), 7.70–7.67 (m, 2H), 7.57 (dd, *J* = 4.8 Hz and 7.8 Hz, 1H), 7.30–7.20 (m, 3H), 7.14–7.05 (m, 2H), 4.09–3.85 (m, 1H), 3.58–3.42 (m, 1H), 3.26–3.16 (m, 2H), 3.06–2.98 (m, 2H), 2.65 (s, 2H), 2.50–2.40 (m, 1H), 1.99 (s, 3H), 1.46–1.44 (m, 1H), 1.31–1.11 (m, 2H), 0.81–0.63 (m, 1H); ^13^C NMR (126 MHz, CDCl_3_) *δ* 170.53, 170.22, 167.55, 167.39, 152.14, 151.88, 150.52, 150.46, 150.25, 150.19, 146.45, 146.30, 136.88, 136.78, 136.30, 136.13, 131.78, 131.62, 130.32, 130.00, 128.69, 128.56, 127.05, 126.92, 123.63, 123.58, 123.51, 123.32, 45.84, 44.30, 43.17, 42.96, 42.07, 37.65, 36.98, 36.90, 32.33, 32.29, 31.73, 31.45, 23.36, 23.30; ESI-HR-MS: (*m*/*z*) calcd. for C_26_H_29_N_4_O_2_ ([M+H]^+^) 429.2286, found 429.2283.

Synthesis of N-((1-([2,4′-bipyridine]-3-carbonyl)-4-benzylpiperidin-4-yl)methyl)cyclopropanecarboxamide (**C-3-2**). Following General Procedure 3, **74-2** (0.70 g, 1.88 mmol) was first treated with TFA (3.5 mL) in CH_2_Cl_2_ (2.1 mL), and the intermediate obtained was reacted with **7·TFA** (0.67 g, 2.26 mmol) using EDCI (0.90 g, 4.70 mmol), HOBT (19 mg, 0.141 mmol), and DIPEA (1.46 g, 11.3 mmol) in dried THF (7 mL). Purification by column chromatography (EtOAc) gave **C-3-2**. White solid, 0.47 g (55%). m.p. 161.5–162.3 °C. ^1^H NMR (500 MHz, CD_3_OD) *δ* 8.77 (dd, *J* = 1.5 Hz and 5.0 Hz, 1H), 8.67–8.58 (m, 2H), 7.86–7.80 (m, 1H), 7.70–7.67 (m, 2H), 7.56 (dd, *J* = 5.0 Hz and 7.5 Hz, 1H), 7.30–7.20 (m, 3H), 7.15–7.06 (m, 2H), 4.08–3.84 (m, 1H), 3.62–3.35 (m, 1.53H), 3.26–3.22 (m, 2H), 3.05–2.99 (m, 2H), 2.68–2.43 (m, 2H), 1.72–1.67 (m, 1H), 1.46–1.44 (m, 1H), 1.34–1.12 (m, 2H), 0.85–0.75 (m, 4H), 0.69–0.64 (m, 0.56H); ^13^C NMR (126 MHz, CDCl_3_) *δ* 174.22, 173.87, 167.45, 167.39, 152.03, 151.86, 150.52, 150.43, 150.29, 150.17, 146.43, 146.35, 136.94, 136.82, 136.30, 136.23, 131.78, 131.67, 130.36, 130.05, 128.67, 128.52, 127.00, 126.85, 123.61, 123.49, 123.42, 45.81, 44.71, 43.22, 43.01, 42.41, 42.08, 37.65, 37.10, 36.96, 32.37, 32.18, 31.32, 31.17, 14.70, 14.64, 7.51, 7.43, 7.28; ESI-HR-MS: (*m*/*z*) calcd. for C_28_H_31_N_4_O_2_ ([M+H]^+^) 455.2442, found 455.2448.

Synthesis of N-((1-([2,4′-bipyridine]-3-carbonyl)-4-benzylpiperidin-4-yl)methyl)pivalamide (**C-3-3**). Following General Procedure 3, **74-3** (0.83 g, 2.14 mmol) was first treated with TFA (4.2 mL) in CH_2_Cl_2_ (2.4 mL), and the intermediate obtained was reacted with **7·TFA** (0.77 g, 2.57 mmol) using EDCI (1.03 g, 5.35 mmol), HOBT (22 mg, 0.161 mmol), and DIPEA (1.65 g, 12.8 mmol) in dried THF (8.3 mL). Purification by column chromatography (EtOAc) gave **C-3-3**. White foam, 0.62 g (62%). ^1^H NMR (500 MHz, DMSO-*d*_6_) *δ* 8.77–8.76 (m, 1H), 8.66–8.58 (m, 2H), 7.79–7.73 (m, 1H), 7.59–7.53 (m, 3H), 7.30–7.16 (m, 4H), 7.10–6.94 (m, 2H), 3.86–3.75 (m, 1H), 3.48–3.40 (m, 1H), 3.21–2.94 (m, 4H), 2.63–2.56 (m, 0.85H), 2.47–2.40 (m, 1.21H), 1.37–1.12 (m, 3H), 1.07 (s, 9H), 0.86–0.61 (m, 1H); ^13^C NMR (151 MHz, CDCl_3_) *δ* 178.19, 167.12, 166.97, 151.90, 151.37, 150.09, 149.82, 149.77, 146.14, 145.91, 137.11, 137.06, 135.98, 135.55, 131.47, 131.24, 129.78, 129.56, 128.60, 128.53, 126.82, 126.76, 123.35, 123.25, 123.14, 122.69, 47.00, 45.31, 42.93, 42.87, 42.69, 41.75, 38.38, 38.30, 37.52, 37.34, 37.17, 37.04, 32.83, 32.48, 32.05, 31.87, 27.12, 27.02; ESI-HR-MS: (*m*/*z*) calcd. for C_29_H_35_N_4_O_2_ ([M+H]^+^) 471.2755, found 471.2761.

Synthesis of N-((1-([2,4′-bipyridine]-3-carbonyl)-4-benzylpiperidin-4-yl)methyl)-2,2,2-trifluoroacetamide (**C-3-4**). Following General Procedure 3, **74-4** (0.72 g, 1.80 mmol) was first treated with TFA (3.6 mL) in CH_2_Cl_2_ (2.2 mL), and the intermediate obtained was reacted with **7·TFA** (0.64 g, 2.16 mmol) using EDCI (0.86 g, 4.50 mmol), HOBT (18 mg, 0.135 mmol), and DIPEA (1.40 g, 10.8 mmol) in dried THF (7.2 mL). Purification by column chromatography (EtOAc) gave **C-3-4**. White solid, 0.48 g (55%). ^1^H NMR (500 MHz, DMSO-*d*_6_) *δ* 9.30–9.22 (m, 1H), 8.78–8.76 (m, 1H), 8.65–8.56 (m, 2H), 7.82–7.74 (m, 1H), 7.58–7.52 (m, 3H), 7.30–7.09 (m, 5H), 3.82–3.68 (m, 1H), 3.60–3.46 (m, 1H), 3.39–3.37 (m, 2H), 3.22–3.16 (m, 2H), 3.06–2.97 (m, 1.60H), 2.69–2.53 (m, 1H), 2.49–2.46 (m, 0.46H), 1.40–1.12 (m, 3H), 0.84–0.73 (m, 1H); ^13^C NMR (151 MHz, CDCl_3_) *δ* 167.31, 167.19, 157.98, 157.73, 157.49, 157.24, 151.50, 150.29, 149.70, 146.16, 146.10, 136.07, 135.98, 135.85, 135.68, 131.14, 131.08, 129.97, 129.77, 128.52, 128.45, 126.99, 126.89, 123.41, 123.35, 123.12, 122.90, 118.48, 116.61, 116.57, 114.70, 114.66, 44.46, 44.32, 43.25, 42.76, 42.68, 42.48, 37.32, 37.27, 36.88, 36.80, 31.66, 31.62, 31.54, 31.20; ^19^F NMR (471 MHz, DMSO-*d*_6_) *δ* −73.66, −73.71; ESI-HR-MS: (*m*/*z*) calcd. for C_26_H_26_F_3_N_4_O_2_ ([M+H]^+^) 483.2002, found 483.2007.

Synthesis of N-((1-([2,4′-bipyridine]-3-carbonyl)-4-benzylpiperidin-4-yl)methyl)-2,2-difluoroacetamide (**C-3-5**). Following general procedure 3, **74-5** (0.57 g, 1.49 mmol) was first treated with TFA (2.9 mL) in CH_2_Cl_2_ (1.7 mL), and the intermediate obtained was reacted with **7·TFA** (0.53 g, 1.79 mmol) using EDCI (0.72 g, 3.73 mmol), HOBT (15 mg, 0.112 mmol), and DIPEA (1.16 g, 8.94 mmol) in dried THF (5.7 mL). Purification by column chromatography (EtOAc) gave **C-3-5**. White foam, 0.33 g (48%). ^1^H NMR (500 MHz, CD_3_OD) *δ* 8.77 (dd, *J* = 1.8 Hz and 4.8 Hz, 1H), 8.66–8.65 (m, 1H), 8.56–8.55 (m, 1H), 7.86–7.79 (m, 1H), 7.69–7.64 (m, 2H), 7.56 (dd, *J* = 4.8 Hz and 7.8 Hz, 1H), 7.31–7.22 (m, 3H), 7.15–7.06 (m, 2H), 6.21–5.97 (m, 1H), 4.03–3.81 (m, 1H), 3.65–3.45 (m, 1H), 3.35–3.32 (m, 1H), 3.28–3.20 (m, 1H), 3.12–3.00 (m, 2H), 2.68 (s, 1H), 2.55–2.47 (m, 1H), 1.50–1.47 (m, 1H), 1.34–1.17 (m, 2H), 0.81–0.75 (m, 1H); ^13^C NMR (151 MHz, CDCl_3_) *δ* 167.30, 167.13, 163.22, 163.11, 163.06, 162.94, 162.89, 162.78, 151.66, 151.47, 150.22, 149.71, 146.11, 146.01, 136.08, 135.99, 135.86, 135.76, 131.19, 131.12, 129.98, 129.76, 128.42, 128.33, 126.85, 126.73, 123.37, 123.11, 122.92, 109.95, 109.79, 108.28, 108.13, 106.62, 106.46, 44.29, 43.91, 42.69, 42.47, 42.17, 37.28, 36.72, 36.65, 31.69, 31.54, 31.45, 30.97; ^19^F NMR (471 MHz, CD_3_OD) *δ* −127.59; ESI-HR-MS: (*m*/*z*) calcd. for C_26_H_27_F_2_N_4_O_2_ ([M+H]^+^) 465.2097, found 465.2104.

Synthesis of N-((1-([2,4′-bipyridine]-3-carbonyl)-4-benzylpiperidin-4-yl)methyl)furan-2-carboxamide (**C-3-6**). Following General Procedure 3, **74-6** (0.78 g, 1.96 mmol) was first treated with TFA (9.8 mL) in CH_2_Cl_2_ (5.9 mL), and the intermediate obtained was reacted with **7·TFA** (0.70 g, 2.35 mmol) using EDCI (0.94 g, 4.90 mmol), HOBT (20 mg, 0.147 mmol), and DIPEA (1.53 g, 11.8 mmol) in dried THF (7.8 mL). Purification by column chromatography (EtOAc) gave **C-3-6**. White solid, 0.39 g (41%). ^1^H NMR (500 MHz, CD_3_OD) *δ* 8.76 (dd, *J* = 1.8 Hz and 4.8 Hz, 1H), 8.56–8.53 (m, 2H), 7.86–7.77 (m, 1H), 7.67–7.63 (m, 3H), 7.55 (dd, *J* = 5.0 Hz and 8.0 Hz, 1H), 7.31–7.23 (m, 3H), 7.19–7.09 (m, 3H), 6.59–6.58 (m, 1H), 4.06–3.88 (m, 1H), 3.62–3.50 (m, 1H), 3.44–3.41 (m, 1H), 3.35–3.33 (m, 1H), 3.27 (s, 1.44H), 3.11–3.08 (m, 1H), 2.74 (s, 0.56H), 2.60–2.52 (m, 1H), 1.56–1.24 (m, 3H), 1.08–0.79 (m, 1H); ^13^C NMR (151 MHz, CDCl_3_) *δ* 167.00, 166.86, 158.26, 158.19, 151.64, 151.32, 149.92, 149.60, 147.15, 147.04, 145.93, 145.77, 143.72, 136.33, 136.28, 135.74, 135.41, 131.24, 131.08, 129.89, 129.66, 128.22, 128.13, 126.44, 126.37, 123.15, 123.03, 122.97, 122.60, 113.87, 111.75, 44.94, 43.11, 42.77, 42.72, 42.53, 41.27, 37.30, 37.19, 36.93, 36.82, 31.84, 31.66, 31.35; ESI-HR-MS: (*m*/*z*) calcd. for C_29_H_29_N_4_O_3_ ([M+H]^+^) 481.2234, found 481.2239.

Synthesis of *tert*-butyl 4-benzyl-4-(methylsulfonamidomethyl)piperidine-1-carboxylate (**75-1**). Compound **75-1** was prepared according to General Procedure 2 from **73** (0.80 g, 2.63 mmol) using Ms_2_O (0.55 g, 3.16 mmol), DMAP (65 mg, 0.53 mmol), and Et_3_N (0.80 g, 7.89 mmol) in dried CH_2_Cl_2_ (8 mL). Purification by column chromatography (EtOAc/PE = 1/2, by *v*/*v*) gave **75-1**. White solid, 0.35 g (35%). m.p 160.2–162.0 °C. ^1^H NMR (500 MHz, DMSO-*d*_6_) *δ* 7.29–7.26 (m, 2H), 7.22–7.18 (m, 3H), 6.99 (t, *J* = 6.8 Hz, 1H), 3.46–3.41 (m, 2H), 3.26 (s, 2H), 2.91 (s, 3H), 2.86 (d, *J* = 7.0 Hz, 2H), 2.63 (s, 2H), 1.36 (s, 9H), 1.35–1.33 (m, 2H), 1.24–1.19 (m, 2H); ^13^C NMR (126 MHz, CDCl_3_) *δ* 154.98, 136.73, 130.44, 128.52, 126.87, 79.68, 47.75, 42.22, 39.93, 36.32, 32.21, 28.52; ESI-HR-MS: (*m*/*z*) calcd. for C_19_H_30_N_2_NaO_4_S ([M+Na]^+^) 405.1818, found 405.1815.

Synthesis of *tert*-butyl 4-benzyl-4-(((trifluoromethyl)sulfonamido)methyl)piperidine-1-carboxylate (**75-2**). Compound **75-2** was prepared according to General Procedure 1 from **73** (0.80 g, 2.63 mmol) using TfCl (0.53 g, 3.16 mmol) and Et_3_N (0.80 g, 7.89 mmol) in dried CH_2_Cl_2_ (8 mL). Purification by column chromatography (EtOAc/PE = 1/5, by *v*/*v*) gave **75-2**. White solid, 0.65 g (57%). m.p 161.3–162.1 °C. ^1^H NMR (500 MHz, DMSO-*d*_6_) *δ* 9.33 (t, *J* = 6.0 Hz, 1H), 7.31–7.28 (m, 2H), 7.24–7.20 (m, 1H), 7.18–7.16 (m, 2H), 3.43–3.38 (m, 2H), 3.33–3.30 (m, 2H), 3.05 (d, *J* = 6.0 Hz, 2H), 2.70 (s, 2H), 1.40–1.38 (m, 2H), 1.36 (s, 9H), 1.27–1.22 (m, 2H); ^13^C NMR (126 MHz, CDCl_3_) *δ* 154.99, 136.34, 130.29, 128.85, 127.27, 121.15, 118.59, 80.08, 49.76, 42.39, 36.47, 31.86, 28.50; ESI-HR-MS: (*m*/*z*) calcd. for C_19_H_27_F_3_N_2_NaO_4_S ([M+Na]^+^) 459.1536, found 459.1530.

Synthesis of *tert*-butyl 4-benzyl-4-(ethylsulfonamidomethyl)piperidine-1-carboxylate (**75-3**). Compound **75-3** was prepared according to General Procedure 1 from **73** (0.80 g, 2.63 mmol) using CH_3_CH_2_SO_2_Cl (0.41 g, 3.16 mmol) and Et_3_N (0.80 g, 7.89 mmol) in dried CH_2_Cl_2_ (8 mL). Purification by column chromatography (EtOAc/PE = 1/5, by *v*/*v*) gave **75-3**. White solid, 0.75 g (72%). m.p. 124.6–126.1 °C. ^1^H NMR (500 MHz, DMSO-*d*_6_) *δ* 7.29–7.26 (m, 2H), 7.21–7.19 (m, 3H), 7.04 (t, *J* = 6.8 Hz, 1H), 3.45–3.34 (m, 2H), 3.25 (s, 2H), 3.04–3.00 (m, 2H), 2.84 (d, *J* = 6.5 Hz, 2H), 2.64 (s, 2H), 1.38–1.36 (m, 2H), 1.35 (s, 9H), 1.23–1.19 (m, 5H); ^13^C NMR (126 MHz, CDCl_3_) *δ* 155.02, 136.82, 130.36, 128.67, 127.02, 79.74, 47.73, 46.72, 42.76, 36.54, 32.39, 28.57, 8.45; ESI-HR-MS: (*m*/*z*) calcd. for C_30_H_33_N_2_O_4_S ([M+H]^+^) 397.2156, found 397.2158.

Synthesis of *tert*-butyl 4-benzyl-4-(cyclopropanesulfonamidomethyl)piperidine-1-carboxylate (**75-4**). Compound **75-4** was prepared according to General Procedure 1 from **73** (0.80 g, 2.63 mmol) using cyclopropanesulfonyl chloride (0.44 g, 3.16 mmol) and Et_3_N (0.80 g, 7.89 mmol) in dried CH_2_Cl_2_ (8 mL). Purification by column chromatography (EtOAc/PE = 1/5, by *v*/*v*) gave **75-4**. White solid, 0.55 g (51%). m.p 66.3–69.7 °C. ^1^H NMR (500 MHz, CDCl_3_) *δ* 7.32–7.29 (m, 2H), 7.27–7.23 (m, 1H), 7.17–7.15 (m, 2H), 4.48 (t, *J* = 6.8 Hz, 1H), 3.60–3.55 (m, 2H), 3.39–3.34 (m, 2H), 3.04–3.02 (m, 2H), 2.68 (s, 2H), 2.41–2.36 (m, 1H), 1.50–1.48 (m, 4H), 1.45 (s, 9H), 1.17–1.13 (m, 2H), 1.00–0.96 (m, 2H); ^13^C NMR (126 MHz, CDCl_3_) *δ* 154.99, 136.83, 130.46, 128.53, 126.87, 79.67, 47.72, 42.38, 36.35, 32.31, 29.75, 28.54, 5.39; ESI-HR-MS: (*m*/*z*) calcd. for C_21_H_32_N_2_NaO_4_S ([M+Na]^+^) 431.1975, found 431.1971.

Synthesis of *tert*-butyl 4-benzyl-4-((thiophene-2-sulfonamido)methyl)piperidine-1-carboxylate (**75-5**). Compound **75-5** was prepared according to General Procedure 1 from **73** (0.80 g, 2.63 mmol) using thiophene-2-sulfonyl chloride (0.58 g, 3.16 mmol) and Et_3_N (0.80 g, 7.89 mmol) in dried CH_2_Cl_2_ (8 mL). Purification by column chromatography (EtOAc/PE = 1/5, by *v*/*v*) gave **75-5**. White solid, 1.02 g (86%). m.p 131.2–131.7 °C. ^1^H NMR (500 MHz, DMSO-*d*_6_) *δ* 7.94 (dd, *J* = 1.0 Hz and 5.0 Hz, 1H), 7.85 (t, *J* = 6.5 Hz, 1H), 7.60 (dd, *J* = 1.3 Hz and 3.8 Hz, 1H), 7.23–7.16 (m, 4H), 7.11–7.09 (m, 2H), 3.43–3.33 (m, 2H), 3.18 (s, 2H), 2.71 (d, *J* = 6.5 Hz, 2H), 2.64 (s, 2H), 1.35 (s, 9H), 1.33–1.31 (m, 2H), 1.24–1.19 (m, 2H); ^13^C NMR (151 MHz, CDCl_3_) *δ* 154.92, 140.51, 136.58, 132.28, 132.05, 130.41, 128.40, 127.57, 126.73, 79.63, 47.63, 42.39, 39.79, 38.88, 36.10, 32.32, 28.50; ESI-HR-MS: (*m*/*z*) calcd. for C_22_H_30_N_2_NaO_4_S_2_ ([M+Na]^+^) 473.1539, found 473.1540.

Synthesis of *tert*-butyl 4-benzyl-4-((pyridine-3-sulfonamido)methyl)piperidine-1-carboxylate (**75-6**). Compound **75-6** was prepared according to General Procedure 1 from **73** (0.80 g, 2.63 mmol) using pyridine-3-sulfonyl chloride (0.56 g, 3.16 mmol) and Et_3_N (0.80 g, 7.89 mmol) in dried CH_2_Cl_2_ (8 mL). Purification by column chromatography (EtOAc/PE = 1/5, by *v*/*v*) gave **75-6**. White solid, 1.02 g (87%). m.p. 160.5–161.3 °C. ^1^H NMR (500 MHz, DMSO-*d*_6_) *δ* 8.97 (d, *J* = 2.5 Hz, 1H), 8.83 (dd, *J* = 1.5 Hz and 5.0 Hz, 1H), 8.21–8.19 (m, 1H), 7.87 (t, *J* = 6.0 Hz, 1H), 7.66 (dd, *J* = 5.0 Hz and 8.0 Hz, 1H), 7.22–7.15 (m, 3H), 7.10–7.08 (m, 2H), 3.44–3.39 (m, 2H), 3.18 (s, 2H), 2.68 (d, *J* = 5.5 Hz, 2H), 2.63 (s, 2H), 1.35 (s, 9H), 1.32–1.30 (m, 2H), 1.23–1.18 (m, 2H); ^13^C NMR (151 MHz, CDCl_3_) *δ* 154.83, 153.17, 147.83, 136.52, 136.40, 134.71, 130.29, 128.29, 126.67, 123.86, 79.61, 47.68, 41.96, 39.70, 38.80, 36.10, 31.95, 28.38; ESI-HR-MS: (*m*/*z*) calcd. for C_23_H_31_N_3_NaO_4_S ([M+Na]^+^) 468.1927, found 468.1931.

Synthesis of *tert*-butyl 4-benzyl-4-(((phenylmethyl)sulfonamido)methyl)piperidine-1-carboxylate (**75-7**). Compound **75-7** was prepared according to General Procedure 1 from **73** (0.80 g, 2.63 mmol) using phenylmethanesulfonyl chloride (0.60 g, 3.16 mmol) and Et_3_N (0.80 g, 7.89 mmol) in dried CH_2_Cl_2_ (8 mL). Purification by column chromatography (EtOAc/PE = 1/5, by *v*/*v*) gave **75-7**. White solid, 0.72 g (60%). m.p. 150.5–151.9 °C. ^1^H NMR (500 MHz, DMSO-*d*_6_) *δ* 7.41–7.35 (m, 5H), 7.29–7.26 (m, 2H), 7.22–7.19 (m, 1H), 7.16–7.11 (m, 3H), 4.36 (s, 2H), 3.43–3.38 (m, 2H), 3.23 (s, 2H), 2.72 (d, *J* = 7.0 Hz, 2H), 2.57 (s, 2H), 1.36 (s, 9H), 1.33–1.28 (m, 2H), 1.19–1.13 (m, 2H); ^13^C NMR (151 MHz, CDCl_3_) *δ* 154.93, 136.68, 130.60, 130.32, 129.33, 129.03, 128.94, 128.43, 126.73, 79.56, 58.74, 47.79, 42.47, 39.78, 38.88, 36.49, 36.48, 32.10, 32.07, 28.51; ESI-HR-MS: (*m*/*z*) calcd. for C_25_H_34_N_2_NaO_4_S ([M+Na]^+^) 481.2131, found 481.2130.

Synthesis of *tert*-butyl 4-benzyl-4-(phenylsulfonamidomethyl)piperidine-1-carboxylate (**75-8**). Compound **75-8** was prepared according to General Procedure 1 from **73** (0.80 g, 2.63 mmol) using benzenesulfonyl chloride (0.56 g, 3.16 mmol) and Et_3_N (0.80 g, 7.89 mmol) in dried CH_2_Cl_2_ (8 mL). Purification by column chromatography (EtOAc/PE = 1/5, by *v*/*v*) gave **75-8**. White solid, 0.72 g (62%). m.p. 76.5–77.3 °C. ^1^H NMR (500 MHz, DMSO-*d*_6_) *δ* 7.83–7.81 (m, 2H), 7.67–7.59 (m, 4H), 7.21–7.14 (m, 3H), 7.09–7.07 (m, 2H), 3.43–3.32 (m, 2H), 3.15 (s, 2H), 2.62–2.61 (m, 4H), 1.35 (s, 9H), 1.31–1.29 (m, 2H), 1.22–1.17 (m, 2H); ^13^C NMR (126 MHz, CDCl_3_) *δ* 154.97, 139.62, 136.67, 132.95, 130.37, 129.36, 128.51, 127.14, 126.85, 79.70, 47.50, 42.68, 36.25, 32.46, 28.55; ESI-HR-MS: (*m*/*z*) calcd. for C_24_H_33_N_2_O_4_S ([M+H]^+^) 445.2156, found 445.2151.

Synthesis of *tert*-butyl 4-benzyl-4-(((4-fluorophenyl)sulfonamido)methyl)piperidine-1-carboxylate (**75-9**). Compound **75-9** was prepared according to General Procedure 1 from **73** (0.80 g, 2.63 mmol) using 4-fluorobenzenesulfonyl chloride (0.61 g, 3.16 mmol) and Et_3_N (0.80 g, 7.89 mmol) in dried CH_2_Cl_2_ (8 mL). Purification by column chromatography (EtOAc/PE = 1/5, by *v*/*v*) gave **75-9**. White solid, 1.10 g (90%). m.p. 176.4–177.8 °C. ^1^H NMR (500 MHz, DMSO-*d*_6_) *δ* 7.89–7.87 (m, 2H), 7.67 (t, *J* = 6.5 Hz, 1H), 7.45 (t, *J* = 8.8 Hz, 2H), 7.22–7.15 (m, 3H), 7.09–7.08 (m, 2H), 3.43–3.39 (m, 2H), 3.17–3.15 (m, 2H), 2.62–2.61 (m, 4H), 1.35 (s, 9H), 1.33–1.28 (m, 2H), 1.23–1.17 (m, 2H); ^13^C NMR (151 MHz, CDCl_3_) *δ* 166.03, 164.34, 154.93, 136.59, 135.73 (d, *J* = 3.2 Hz), 130.36, 129.84 (d, *J* = 9.4 Hz), 128.43, 126.81, 116.54 (d, *J* = 22.5 Hz), 79.68, 47.52, 42.36, 36.18, 32.33, 28.50; ^19^F NMR (471 MHz, DMSO-*d*_6_) *δ* −107.10; ESI-HR-MS: (*m*/*z*) calcd. for C_24_H_31_FN_2_NaO_4_S ([M+Na]^+^) 485.1881, found 485.1880.

Synthesis of *tert*-butyl 4-benzyl-4-(((4-cyanophenyl)sulfonamido)methyl)piperidine-1-carboxylate (**75-10**). Compound **75-10** was prepared according to General Procedure 1 from **73** (1.00 g, 3.28 mmol) using 4-cyanobenzenesulfonyl chloride (0.79 g, 3.94 mmol) and Et_3_N (1.00 g, 9.84 mmol) in dried CH_2_Cl_2_ (10 mL). Purification by column chromatography (EtOAc/PE = 1/5, by *v*/*v*) gave **75-10**. White foam, 1.10 g (73%). ^1^H NMR (500 MHz, DMSO-*d*_6_) *δ* 8.12–8.10 (m, 2H), 7.99–7.97 (m, 2H), 7.92 (t, *J* = 6.3 Hz, 1H), 7.22–7.15 (m, 3H), 7.10–7.08 (m, 2H), 3.43–3.39 (m, 2H), 3.17–3.15 (m, 2H), 2.67–2.62 (m, 4H), 1.35 (s, 9H), 1.32–1.29 (m, 2H), 1.23–1.17 (m, 2H); ^13^C NMR (151 MHz, CDCl_3_) *δ* 154.94, 144.08, 136.50, 133.13, 130.29, 128.57, 127.73, 127.01, 117.35, 116.57, 79.84, 48.03, 42.37, 39.75, 38.94, 36.33, 32.26, 28.51; ESI-HR-MS: (*m*/*z*) calcd. for C_25_H_31_N_3_NaO_4_S ([M+Na]^+^) 492.1927, found 492.1926.

Synthesis of *tert*-butyl 4-benzyl-4-(((4-methoxyphenyl)sulfonamido)methyl)piperidine-1-carboxylate (**75-11**). Compound **75-11** was prepared according to General Procedure 1 from **73** (0.80 g, 2.63 mmol) using 4-methoxybenzenesulfonyl chloride (0.65 g, 3.16 mmol) and Et_3_N (0.80 g, 7.89 mmol) in dried CH_2_Cl_2_ (8 mL). Purification by column chromatography (EtOAc/PE = 1/5, by *v*/*v*) gave **75-11**. White solid, 0.99 g (79%). m.p. 113.1–114.2 °C. ^1^H NMR (500 MHz, DMSO-*d*_6_) *δ* 7.75 (d, *J* = 10.0 Hz, 2H), 7.47 (t, *J* = 6.5 Hz, 1H), 7.21–7.16 (m, 3H), 7.13–7.07 (m, 4H), 3.84 (s, 3H), 3.43–3.33 (m, 2H), 3.14 (s, 2H), 2.62–2.57 (m, 4H), 1.35 (s, 9H), 1.30–1.28 (m, 2H), 1.22–1.17 (m, 2H); ^13^C NMR (151 MHz, CDCl_3_) *δ* 163.01, 154.90, 136.69, 131.15, 130.44, 129.25, 128.30, 126.62, 114.42, 79.54, 55.70, 47.20, 42.32, 39.74, 38.92, 36.08, 32.33, 28.48; ESI-HR-MS: (*m*/*z*) calcd. for C_25_H_34_N_2_NaO_5_S ([M+Na]^+^) 497.2081, found 497.2078.

Synthesis of *tert*-butyl 4-benzyl-4-(((4-nitrophenyl)sulfonamido)methyl)piperidine-1-carboxylate (**75-12**). Compound **75-12** was prepared according to General Procedure 1 from **73** (0.80 g, 2.63 mmol) using 4-nitrobenzenesulfonyl chloride (0.70 g, 3.16 mmol) and Et_3_N (0.80 g, 7.89 mmol) in dried CH_2_Cl_2_ (8 mL). Purification by column chromatography (EtOAc/PE = 1/5, by *v*/*v*) gave **75-12**. White solid, 1.10 g (85%). m.p. 164.1–164.9 °C. ^1^H NMR (500 MHz, DMSO-*d*_6_) *δ* 8.45–8.42 (m, 2H), 8.09–7.99 (m, 2H), 7.71 (t, *J* = 6.5 Hz, 1H), 7.22–7.15 (m, 3H), 7.11–7.09 (m, 2H), 3.44–3.39 (m, 2H), 3.20–3.15 (m, 2H), 2.69 (d, *J* = 6.5 Hz, 2H), 2.63 (s, 2H), 1.34 (s, 9H), 1.32–1.30 (m, 2H), 1.24–1.18 (m, 2H); ^13^C NMR (151 MHz, CDCl_3_) *δ* 154.95, 150.19, 145.64, 136.50, 130.29, 128.58, 128.36, 127.02, 124.56, 79.86, 48.17, 42.34, 39.76, 38.95, 36.35, 32.23, 28.51; ESI-HR-MS: (*m*/*z*) calcd. for C_24_H_31_N_3_NaO_6_S ([M+Na]^+^) 512.1826, found 512.1830.

Synthesis of *tert*-butyl 4-benzyl-4-(((3-chlorophenyl)sulfonamido)methyl)piperidine-1-carboxylate (**75-13**). Compound **75-13** was prepared according to General Procedure 1 from **73** (0.80 g, 2.63 mmol) using 3-chlorobenzenesulfonyl chloride (0.67 g, 3.16 mmol) and Et_3_N (0.80 g, 7.89 mmol) in dried CH_2_Cl_2_ (8 mL). Purification by column chromatography (EtOAc/PE = 1/5, by *v*/*v*) gave **75-13**. White solid, 1.10 g (87%). m.p. 185.2–185.8 °C. ^1^H NMR (500 MHz, DMSO-*d*_6_) *δ* 7.84 (t, *J* = 1.8 Hz, 1H), 7.79–7.74 (m, 3H), 7.65 (t, *J* = 8.0 Hz, 1H), 7.22–7.15 (m, 3H), 7.10–7.08 (m, 2H), 3.44–3.39 (m, 2H), 3.17–3.15 (m, 2H), 2.65–2.63 (m, 4H), 1.35 (s, 9H), 1.32–1.30 (m, 2H), 1.23–1.17 (m, 2H); ^13^C NMR (151 MHz, CDCl_3_) *δ* 154.94, 141.43, 136.58, 135.54, 133.01, 130.63, 130.35, 128.51, 127.20, 126.89, 125.19, 79.71, 47.67, 42.52, 39.81, 38.94, 36.25, 32.37, 28.53; ESI-HR-MS: (*m*/*z*) calcd. for C_24_H_31_ClN_2_NaO_4_S ([M+Na]^+^) 501.1585, found 501.1586.

Synthesis of *tert*-butyl 4-benzyl-4-(((2-fluorophenyl)sulfonamido)methyl)piperidine-1-carboxylate (**75-14**). Compound **75-14** was prepared according to General Procedure 1 from **73** (0.80 g, 2.63 mmol) using 2-fluorobenzenesulfonyl chloride (0.61 g, 3.16 mmol) and Et_3_N (0.80 g, 7.89 mmol) in dried CH_2_Cl_2_ (8 mL). Purification by column chromatography (EtOAc/PE = 1/5, by *v*/*v*) gave **75-14**. White solid, 1.06 g (87%). m.p. 161.3–162.6 °C. ^1^H NMR (500 MHz, DMSO-*d*_6_) *δ* 7.97 (s, 1H), 7.81–7.78 (m, 1H), 7.73–7.69 (m, 1H), 7.48–7.44 (m, 1H), 7.40–7.37 (m, 1H), 7.24–7.21 (m, 2H), 7.19–7.16 (m, 1H), 7.12–1.10 (m, 2H), 3.42–3.38 (m, 2H), 3.16 (s, 2H), 2.76 (s, 2H), 2.65 (s, 2H), 1.35 (s,9H), 1.33–1.30 (m, 2H), 1.23–1.17 (m, 2H); ^13^C NMR (126 MHz, DMSO-*d*_6_) δ 159.14, 157.13, 153.93, 137.09, 135.24 (d, *J* = 8.4 Hz), 130.63, 129.78, 128.31 (d, *J* = 14.2 Hz), 127.79, 126.09, 124.91 (d, *J* = 3.7 Hz), 117.26 (d, *J* = 21.3 Hz), 78.49, 47.07, 40.67, 35.53, 30.92, 28.05; ^19^F NMR (471 MHz, DMSO-*d*_6_) *δ* −110.11; ESI-HR-MS: (*m*/*z*) calcd. for C_24_H_31_FN_2_NaO_4_S ([M+Na]^+^) 485.1881, found 485.1881.

Synthesis of *tert*-butyl 4-benzyl-4-(((3-fluorophenyl)sulfonamido)methyl)piperidine-1-carboxylate (**75-15**). Compound **75-15** was prepared according to General Procedure 1 from **73** (0.80 g, 2.63 mmol) using 3-fluorobenzenesulfonyl chloride (0.61 g, 3.16 mmol) and Et_3_N (0.80 g, 7.89 mmol) in dried CH_2_Cl_2_ (8 mL). Purification by column chromatography (EtOAc/PE = 1/5, by *v*/*v*) gave **75-15**. White solid, 1.04 g (86%). m.p. 151.0–152.2 °C. ^1^H NMR (500 MHz, DMSO-*d*_6_) *δ* 7.76 (s, 1H), 7.70–7.66 (m, 2H), 7.63–7.61 (m, 1H), 7.55–7.51 (m, 1H), 7.22–7.15 (m, 3H), 7.10–7.08 (m, 2H), 3.44–3.40 (m, 2H), 3.17 (s, 2H), 2.65–2.62 (m, 4H), 1.35 (s, 9H), 1.32–1.29 (m, 2H), 1.23–1.18 (m, 2H); ^13^C NMR (151 MHz, CDCl_3_) *δ* 163.41, 161.74, 154.94, 141.75 (d, *J* = 6.5 Hz), 136.57, 131.16 (d, *J* = 7.7 Hz), 130.36, 128.45, 126.84, 122.85, 122.83, 120.09 (d, *J* = 21.3 Hz), 114.50 (d, *J* = 24.3 Hz), 79.70, 47.64, 42.40, 39.79, 38.91, 36.22, 32.32, 28.51; ^19^F NMR (471 MHz, DMSO-*d*_6_) *δ* −110.62; ESI-HR-MS: (*m*/*z*) calcd. for C_24_H_31_FN_2_NaO_4_S ([M+Na]^+^) 485.1881, found 485.1881.

Synthesis of *tert*-butyl 4-benzyl-4-(((perfluorophenyl)sulfonamido)methyl)piperidine-1-carboxylate (**75-16**). Compound **75-16** was prepared according to General Procedure 1 from **73** (0.80 g, 2.63 mmol) using perfluorobenzenesulfonyl chloride (0.84 g, 3.16 mmol) and Et_3_N (0.80 g, 7.89 mmol) in dried CH_2_Cl_2_ (8 mL). Purification by column chromatography (EtOAc/PE = 1/5, by *v*/*v*) gave **75-16**. White solid, 1.04 g (74%). m.p. 170.1–170.8 °C. ^1^H NMR (500 MHz, DMSO-*d*_6_) *δ* 8.72 (s, 1H), 7.28–7.24 (m, 2H), 7.22–7.16 (m, 3H), 3.44–3.39 (m, 2H), 3.24 (s, 2H), 2.89 (s, 2H), 2.68 (s, 2H), 1.37–1.35 (m, 11H), 1.26–1.21 (m, 2H); ^13^C NMR (151 MHz, CDCl_3_) *δ* 154.95, 145.12 (t, *J* = 35.0 Hz), 143.40 (t, *J* = 37.4 Hz), 138.85 (d, *J* = 12.5 Hz), 137.19 (t, *J* = 17.8 Hz), 136.52, 130.20, 128.80, 127.24, 116.14 (t, *J* = 15.3 Hz), 79.86, 48.14, 46.01, 42.97, 39.74, 38.90, 36.54, 32.50, 28.55; ^19^F NMR (471 MHz, DMSO-*d*_6_) *δ* −137.97–−138.07 (m), −148.02 (tt, *J* = 5.7 and 22.6 Hz), −159.92–−160.05 (m); ESI-HR-MS: (*m*/*z*) calcd. for C_24_H_27_F_5_N_2_NaO_4_S ([M+Na]^+^) 557.1504, found 557.1505.

Synthesis of *tert*-butyl 4-benzyl-4-(((2,3,4,5,6-pentamethylphenyl)sulfonamido)methyl)piperidine-1-carboxylate (**75-17**). Compound **75-17** was prepared according to General Procedure 1 from **73** (0.80 g, 2.63 mmol) using 2,3,4,5,6-pentamethylbenzenesulfonyl chloride (0.78 g, 3.16 mmol) and Et_3_N (0.80 g, 7.89 mmol) in dried CH_2_Cl_2_ (8 mL). Purification by column chromatography (EtOAc/PE = 1/5, by *v*/*v*) gave **75-17**. White solid, 1.01g (75%). m.p. 177.5–178.0 °C. ^1^H NMR (500 MHz, DMSO-*d*_6_) *δ* 7.39 (t, *J* = 6.5 Hz, 1H), 7.22–7.15 (m, 3H), 7.07–7.06 (m, 2H), 3.37–3.36 (m, 2H), 3.13–3.08 (m, 2H), 2.61–2.59 (m, 4H), 2.48 (s, 6H), 2.23 (s, 3H), 2.18 (s, 6H), 1.34 (s, 9H), 1.32–1.27 (m, 2H), 1.17–1.12 (m, 2H); ^13^C NMR (151 MHz, CDCl_3_) *δ* 154.93, 139.73, 136.95, 135.72, 134.99, 134.11, 130.41, 128.46, 126.78, 79.61, 46.93, 42.70, 39.82, 38.97, 36.31, 32.55, 28.54, 19.01, 17.89, 17.17; ESI-HR-MS: (*m*/*z*) calcd. for C_29_H_42_N_2_NaO_4_S ([M+Na]^+^) 537.2757, found 537.2759.

Synthesis of *tert*-butyl 4-benzyl-4-(((3,4-difluorophenyl)sulfonamido)methyl)piperidine-1-carboxylate (**75-18**). Compound **75-18** was prepared according to General Procedure 1 from **73** (0.80 g, 2.63 mmol) using 3,4-difluorobenzenesulfonyl chloride (0.67 g, 3.16 mmol) and Et_3_N (0.80 g, 7.89 mmol) in dried CH_2_Cl_2_ (8 mL). Purification by column chromatography (EtOAc/PE = 1/5, by *v*/*v*) gave **75-18**. White solid, 1.09 g (86%). m.p. 185.9–186.3 °C. ^1^H NMR (500 MHz, DMSO-*d*_6_) *δ* 7.89–7.86 (m, 1H), 7.77 (s, 1H), 7.72–7.69 (m, 2H), 7.23–7.16 (m, 3H), 7.11–7.09 (m, 2H), 3.43–3.39 (m, 2H), 3.18 (s, 2H), 2.66–2.62 (m, 4H), 1.35 (s, 9H), 1.32–1.29 (m, 2H), 1.23–1.17 (m, 2H); ^13^C NMR (151 MHz, DMSO-*d*_6_) *δ* 153.95, 152.68 (d, *J* = 12.4 Hz), 151.00 (d, *J* = 12.4 Hz), 150.06 (d, *J* = 13.4 Hz), 148.40 (d, *J* = 13.3 Hz), 137.34 (t, *J* = 4.2 Hz), 130.64, 127.72, 126.09, 124.40 (q, *J* = 3.9 Hz), 118.63 (d, *J* = 18.3 Hz), 116.44 (d, *J* = 19.6 Hz), 78.43, 67.00, 46.97, 40.83, 35.51, 28.01; ESI-HR-MS: (*m*/*z*) calcd. for C_24_H_30_F_2_N_2_NaO_4_S ([M+Na]^+^) 503.1787, found 503.1787.

Synthesis of *tert*-butyl 4-benzyl-4-(((3,4,5-trifluorophenyl)sulfonamido)methyl)piperidine-1-carboxylate (**75-19**). Compound **75-19** was prepared according to General Procedure 1 from **73** (0.80 g, 2.63 mmol) using 3,4,5-trifluorobenzenesulfonyl chloride (0.73 g, 3.16 mmol) and Et_3_N (0.80 g, 7.89 mmol) in dried CH_2_Cl_2_ (8 mL). Purification by column chromatography (EtOAc/PE = 1/3, by *v*/*v*) gave **75-19**. White solid, 1.03 g (79%). m.p. 197.9–198.5 °C. ^1^H NMR (500 MHz, DMSO-*d*_6_) *δ* 7.86 (s, 1H), 7.77 (t, *J* = 6.5 Hz, 2H), 7.24–7.12 (m, 3H), 7.14–7.12 (m, 2H), 3.45–3.40 (m, 2H), 3.19 (s, 2H), 2.70 (s, 2H), 2.62 (s, 2H), 1.35 (s, 9H), 1.33–1.30 (m, 2H), 1.24–1.18 (m, 2H); ^13^C NMR (151 MHz, CDCl_3_) *δ* 154.98, 151.27 (ddd, *J* = 3.2, 10.6 and 257.2 Hz), 142.789 (dt, *J* = 15.0 and 185.4 Hz), 136.57, 135.96 (q, *J* = 5.6 Hz), 130.28, 128.69, 127.16, 112.31 (dd, *J* = 5.6 and 18.3 Hz), 79.91, 48.29, 42.61, 39.80, 38.99, 36.39, 32.41, 28.54; ^19^F NMR (471 MHz, DMSO-*d*_6_) *δ* −131.39 (d, *J* = 21.2 Hz), −154.40 (d, *J* = 21.2 Hz); ESI-HR-MS: (*m*/*z*) calcd. for C_24_H_29_F_3_N_2_NaO_4_S ([M+Na]^+^) 521.1692, found 521.1694.

Synthesis of *tert*-butyl 4-benzyl-4-(((2,5-difluorophenyl)sulfonamido)methyl)piperidine-1-carboxylate (**75-20**). Compound **75-20** was prepared according to General Procedure 1 from **73** (0.80 g, 2.63 mmol) using 2,5-difluorobenzenesulfonyl chloride (0.67 g, 3.16 mmol) and Et_3_N (0.80 g, 7.89 mmol) in dried CH_2_Cl_2_ (8 mL). Purification by column chromatography (EtOAc/PE = 1/3, by *v*/*v*) gave **75-20**. White solid, 1.07 g (85%). m.p. 155.5–156.3 °C. ^1^H NMR (500 MHz, DMSO-*d*_6_) *δ* 8.12 (t, *J* = 6.0 Hz, 1H), 7.62–7.53 (m, 3H), 7.24–7.21 (m, 2H), 7.19–7.16 (m, 1H), 7.13–7.11 (m, 2H), 3.43–3.38 (m, 2H), 3.19 (s, 2H), 2.79 (d, *J* = 5.5 Hz, 2H), 1.35 (s, 9H), 1.33–1.31 (m, 2H), 1.24–1.18 (m, 2H); ^13^C NMR (151 MHz, CDCl_3_) *δ* 157.96 (dd, *J* = 2.5 and 248.5 Hz), 154.88, 136.55, 130.32, 128.99 (dd, *J* = 6.7 and 16.2 Hz), 128.48, 126.84, 121.66 (dd, *J* = 8.5 and 24.1 Hz), 118.45 (dd, *J* = 7.9 and 24.3 Hz), 117.37, 117.19, 79.64, 47.52, 42.48, 39.73, 38.85, 36.24, 32.25, 28.48; ^19^F NMR (471 MHz, DMSO-*d*_6_) *δ* −115.64 (d, *J* = 18.8 Hz), −116.31 (d, *J* = 18.8 Hz); ESI-HR-MS: (*m*/*z*) calcd. for C_24_H_30_F_2_N_2_NaO_4_S ([M+Na]^+^) 503.1787, found 503.1786.

Synthesis of *tert*-butyl 4-benzyl-4-(((2,4,5-trifluorophenyl)sulfonamido)methyl)piperidine-1-carboxylate (**75-21**). Compound **75-21** was prepared according to General Procedure 1 from **73** (0.80 g, 2.63 mmol) using 2,4,5-trifluorobenzenesulfonyl chloride (0.73 g, 3.16 mmol) and Et_3_N (0.80 g, 7.89 mmol) in dried CH_2_Cl_2_ (8 mL). Purification by column chromatography (EtOAc/PE = 1/3, by *v*/*v*) gave **75-21**. White solid, 1.13 g (86%). m.p. 134.9–135.6 °C. ^1^H NMR (500 MHz, DMSO-*d*_6_) *δ* 8.15 (t, *J* = 5.8 Hz, 1H), 7.91–7.82 (m, 2H), 7.25–7.11 (m, 5H), 3.43–3.39 (m, 2H), 3.21–3.17 (m, 2H), 2.78 (d, *J* = 5.5 Hz, 2H), 2.65 (s, 2H), 1.35 (s, 9H), 1.32–1.30 (m, 2H), 1.24–1.19 (m, 2H); ^13^C NMR (151 MHz, CDCl_3_) *δ* 155.23 (dd, *J* = 2.9 Hz and 10.1 Hz), 154.49, 154.15 (dd, *J* = 2.9 Hz and 10.2 Hz), 153.55 (dd, *J* = 2.9 Hz and 10.2 Hz), 136.53, 130.30, 128.54, 126.93, 124.37 (dt, *J* = 4.2 Hz and 16.2 Hz), 119.04 (dt, *J* = 1.6 Hz and 20.9 Hz), 107.47 (dd, *J* = 21.7 Hz and 27.3 Hz), 79.72, 47.67, 42.47, 39.71, 38.88, 36.28, 32.27, 28.49; ^19^F NMR (471 MHz, DMSO-*d*_6_) *δ* −110.11 (dd, *J* = 8.0 and 16.5 Hz), −126.54 (dd, *J* = 8.2 and 22.4 Hz), −140.55 (dd, *J* = 15.8 and 22.4 Hz); ESI-HR-MS: (*m*/*z*) calcd. for C_24_H_29_F_3_N_2_NaO_4_S ([M+Na]^+^) 521.1692, found 521.1697.

Synthesis of *tert*-butyl 4-benzyl-4-(((3-chloro-4-fluorophenyl)sulfonamido)methyl)piperidine-1-carboxylate (**75-22**). Compound **75-22** was prepared according to General Procedure 1 from **73** (0.80 g, 2.63 mmol) using 3-chloro-4-fluorobenzenesulfonyl chloride (0.72 g, 3.16 mmol) and Et_3_N (0.80 g, 7.89 mmol) in dried CH_2_Cl_2_ (8 mL). Purification by column chromatography (EtOAc/PE = 1/3, by *v*/*v*) gave **75-22**. White solid, 1.13 g (87%). m.p 178.5–179.2 °C. ^1^H NMR (500 MHz, DMSO-*d*_6_) *δ* 8.01 (dd, *J* = 2.3 Hz and 6.8 Hz, 1H), 7.85–7.82 (m, 1H), 7.77 (t, *J* = 6.5 Hz, 1H), 7.77 (t, *J* = 6.5 Hz, 1H), 7.23–7.15 (m, 3H), 7.11–7.09 (m, 2H), 3.44–3.39 (m, 2H), 3.19–3.16 (m, 2H), 2.66–2.62 (m, 4H), 1.35 (s, 9H), 1.31–1.29 (m, 2H), 1.23–1.18 (m, 2H); ^13^C NMR (151 MHz, CDCl_3_) *δ* 161.54, 159.84, 154.96, 136.85 (d, *J* = 3.8 Hz), 136.59, 130.33, 130.06, 128.56, 127.59 (d, *J* = 8.5 Hz), 126.99, 122.65 (d, *J* = 18.7 Hz), 117.58 (d, *J* = 22.2 Hz), 79.79, 47.87, 42.54, 39.80, 38.95, 36.29, 32.39, 28.54; ^19^F NMR (471 MHz, DMSO-*d*_6_) *δ* −109.99; ESI-HR-MS: (*m*/*z*) calcd. for C_24_H_30_ClFN_2_NaO_4_S ([M+Na]^+^) 519.1491, found 519.1495.

Synthesis of *tert*-butyl 4-benzyl-4-(((2,3,4-trifluorophenyl)sulfonamido)methyl)piperidine-1-carboxylate (**75-23**). Compound **75-23** was prepared according to General Procedure 1 from **73** (0.80 g, 2.63 mmol) using 2,3,4-trifluorobenzenesulfonyl chloride (0.73 g, 3.16 mmol) and Et_3_N (0.80 g, 7.89 mmol) in dried CH_2_Cl_2_ (8 mL). Purification by column chromatography (EtOAc/PE = 1/3, by *v*/*v*) gave **75-23**. White solid, 1.12 g (85%). m.p. 151.7–152.6 °C. ^1^H NMR (500 MHz, DMSO-*d*_6_) *δ* 8.19 (t, *J* = 6.3 Hz, 1H), 7.68–7.64 (m, 1H), 7.55–7.50 (m, 1H), 7.25–7.22 (m, 2H), 7.20–7.17 (m, 1H), 7.14–7.13 (m, 2H), 3.43–3.38 (m, 2H), 3.22–3.17 (m, 2H), 2.78 (d, *J* = 6.0 Hz, 2H), 2.66 (s, 2H), 1.35 (s, 9H), 1.34–1.31 (m, 2H), 1.24–1.19 (m, 2H); ^13^C NMR (151 MHz, CDCl_3_) *δ* 154.93, 154.48 (ddd, *J* = 3.0, 10.2 and 259.3 Hz), 148.45 (ddd, *J* = 3.8, 11.5 and 258.0 Hz), 140.41 (dt, *J* = 15.3 and 257.4 Hz), 136.55, 130.25, 128.64, 127.05, 125.62 (d, *J* = 9.8 Hz), 124.60 (q, *J* = 3.2 Hz), 112.71 (dd, *J* = 3.6 and 18.1 Hz), 79.76, 47.70, 42.73, 39.76, 38.89, 36.37, 32.39, 28.53; ^19^F NMR (471 MHz, CDCl_3_) *δ* −124.18 (dd, *J* = 13.0 and 20.0 Hz), −131.04 (dd, *J* = 13.0 and 21.4 Hz), −156.25 (t, *J* = 21.0 Hz); ESI-HR-MS: (*m*/*z*) calcd. for C_24_H_29_F_3_N_2_NaO_4_S ([M+Na]^+^) 521.1692, found 521.1700.

Synthesis of *tert*-butyl 4-benzyl-4-(((3,5-bis(trifluoromethyl)phenyl)sulfonamido)methyl)piperidine-1-carboxylate (**75-24**). Compound **75-24** was prepared according to General Procedure 1 from **73** (0.80 g, 2.63 mmol) using 3,5-bis(trifluoromethyl)benzenesulfonyl chloride (0.99 g, 3.16 mmol) and Et_3_N (0.80 g, 7.89 mmol) in dried CH_2_Cl_2_ (8 mL). Purification by column chromatography (EtOAc/PE = 1/3, by *v*/*v*) gave **75-24**. White solid, 1.31 g (86%). m.p. 178.6–179.7 °C. ^1^H NMR (500 MHz, DMSO-*d*_6_) *δ* 8.50 (s, 1H), 8.42–8.41 (m, 2H), 8.02 (t, *J* = 6.0 Hz, 1H), 7.20–7.15 (m, 3H), 7.10–7.08 (m, 2H), 3.43–3.38 (m, 2H), 3.23–3.17 (m, 2H), 2.71 (d, *J* = 6.0 Hz, 2H), 2.64 (s, 2H), 1.35 (s, 9H), 1.32–1.30 (m, 2H), 1.24–1.18 (m, 2H); ^13^C NMR (151 MHz, CD_3_OD) *δ* 156.60, 145.17, 138.11, 133.92 (q, *J* = 34.2 Hz), 131.73, 129.03, 128.56, 128.54, 127.50, 127.22 (t, *J* = 3.7 Hz), 124.17 (q, *J* = 272.7 Hz), 81.03, 42.36, 41.05, 40.12, 37.17, 32.54, 28.65; ^19^F NMR (471 MHz, DMSO-*d*_6_) *δ* −61.49; ESI-HR-MS: (*m*/*z*) calcd. for C_26_H_30_F_6_N_2_NaO_4_S ([M+Na]^+^) 603.1723, found 603.1724.

Synthesis of *tert*-butyl 4-benzyl-4-(((2-chloro-4-fluorophenyl)sulfonamido)methyl)piperidine-1-carboxylate (**75-25**). Compound **75-25** was prepared according to General Procedure 1 from **73** (0.80 g, 2.63 mmol) using 2-chloro-4-fluorobenzenesulfonyl chloride (0.72 g, 3.16 mmol) and Et_3_N (0.80 g, 7.89 mmol) in dried CH_2_Cl_2_ (8 mL). Purification by column chromatography (EtOAc/PE = 1/3, by *v*/*v*) gave **75-25**. White solid, 1.18 g (90%). m.p. 204.4–206.3 °C. ^1^H NMR (500 MHz, DMSO-*d*_6_) *δ* 8.01 (dd, *J* = 6.0 Hz and 9.0 Hz,1H), 7.97 (t, *J* = 6.3 Hz, 1H), 7.71 (dd, *J* = 2.5 Hz and 8.5 Hz,1H), 7.43–7.39 (m, 1H), 7.25–7.22 (m, 2H), 7.19–7.18 (m, 1H), 7.14–7.12 (m, 2H), 3.43–3.38 (m, 2H), 3.18–3.12 (m, 2H), 2.76 (d, *J* = 6.5 Hz, 2H), 2.65 (s, 2H), 1.34 (s, 9H), 1.32–1.30 (m, 2H), 1.23–1.17 (m, 2H); ^13^C NMR (151 MHz, CDCl_3_) *δ* 165.69 (d, *J* = 258.7 Hz), 154.87, 136.63, 133.51 (d, *J* = 9.7 Hz), 133.28 (d, *J* = 3.62 Hz), 132.99 (d, *J* = 11.0 Hz), 130.37, 128.53, 126.87, 119.29 (d, *J* = 25.5 Hz), 114.74 (d, *J* = 21.4 Hz), 79.65, 47.43, 42.56, 39.71, 38.87, 36.32, 32.41, 28.50; ^19^F NMR (471 MHz, DMSO-*d*_6_) *δ* −105.21; ESI-HR-MS: (*m*/*z*) calcd. for C_24_H_30_ClFN_2_NaO_4_S ([M+Na]^+^) 519.1491, found 519.1499.

Synthesis of *tert*-butyl 4-benzyl-4-(((3-cyano-4-fluorophenyl)sulfonamido)methyl)piperidine-1-carboxylate (**75-26**). Compound **75-26** was prepared according to General Procedure 1 from **73** (0.80 g, 2.63 mmol) using 3-cyano-4-fluorobenzenesulfonyl chloride (0.69 g, 3.16 mmol) and Et_3_N (0.80 g, 7.89 mmol) in dried CH_2_Cl_2_ (8 mL). Purification by column chromatography (EtOAc/PE = 1/3, by *v*/*v*) gave **75-26**. White solid, 0.74 g (58%). m.p. 207.8–209.5 °C. ^1^H NMR (500 MHz, DMSO-*d*_6_) *δ* 8.36 (dd, *J* = 2.5 Hz and 6.0 Hz,1H), 8.21–8.18 (m, 1H), 7.85–7.77 (m, 2H), 7.24–7.17 (m, 3H), 7.12–7.11 (m, 2H), 3.45–3.40 (m, 2H), 3.20–3.18 (m, 2H), 2.69 (d, *J* = 6.5 Hz, 2H), 2.62 (s, 2H), 1.35 (s, 9H), 1.31–1.29 (m, 2H), 1.23–1.18 (m, 2H); ^13^C NMR (151 MHz, CDCl_3_) *δ* 165.13 (d, *J* = 9.5 Hz), 154.97, 137.60, 137.58, 136.58, 133.98, 133.91, 133.03, 130.23, 128.77, 127.26, 117.84 (d, *J* = 20.7 Hz), 112.35, 103.12 (d, *J* = 16.6 Hz), 79.93, 48.28, 42.77, 39.78, 38.97, 36.44, 32.51, 28.56; ^19^F NMR (471 MHz, DMSO-*d*_6_) *δ* −102.49; ESI-HR-MS: (*m*/*z*) calcd. for C_25_H_30_FN_3_NaO_4_S ([M+Na]^+^) 510.1833, found 510.1839.

Synthesis of *tert*-butyl 4-benzyl-4-(((4-(*tert*-butyl)phenyl)sulfonamido)methyl)piperidine-1-carboxylate (**75-27**). Compound **75-27** was prepared according to General Procedure 1 from **73** (0.80 g, 2.63 mmol) using 4-*tert*-butylbenzenesulfonyl chloride (0.73 g, 3.16 mmol) and Et_3_N (0.80 g, 7.89 mmol) in dried CH_2_Cl_2_ (8 mL). Purification by column chromatography (EtOAc/PE = 1/3, by *v*/*v*) gave **75-27**. White solid, 1.10 g (84%) m.p. 190.0–190.9 °C. ^1^H NMR (500 MHz, DMSO-*d*_6_) *δ* 7.76–7.73 (m, 2H), 7.63–7.60 (m, 2H), 7.57 (t, *J* = 6.3 Hz, 1H), 7.19–7.13 (m, 3H), 7.08–7.06 (m, 2H), 3.43–3.38 (m, 2H), 3.12–3.06 (m, 2H), 2.62–2.61 (m, 4H), 1.35 (s, 9H), 1.31 (s, 9H), 1.29–1.27 (m, 2H), 1.22–1.17 (m, 2H); ^13^C NMR (151 MHz, CDCl_3_) *δ* 156.66, 154.93, 136.70, 136.55, 130.44, 128.36, 127.03, 126.66, 126.29, 79.59, 47.22, 42.45, 39.76, 38.91, 36.18, 35.27, 32.38, 31.19, 28.52; ESI-HR-MS: (*m*/*z*) calcd. for C_28_H_41_N_2_O_4_S ([M+H]^+^) 501.2782, found 501.2790.

Synthesis of *tert*-butyl 4-benzyl-4-(((4-(trifluoromethyl)phenyl)sulfonamido)methyl)piperidine-1-carboxylate (**75-28**). Compound **75-28** was prepared according to General Procedure 1 from **73** (0.80 g, 2.63 mmol) using 4-(trifluoromethyl)benzenesulfonyl chloride (0.77 g, 3.16 mmol) and Et_3_N (0.80 g, 7.89 mmol) in dried CH_2_Cl_2_ (8 mL). Purification by column chromatography (EtOAc/PE = 1/3, by *v*/*v*) gave **75-28**. White solid, 1.20 g (89%). m.p. 131.2–132.9 °C. ^1^H NMR (500 MHz, DMSO-*d*_6_) *δ* 8.05–8.00 (m, 4H), 7.89 (t, *J* = 5.5 Hz, 1H), 7.20–7.14 (m, 3H), 7.08–7.06 (m, 2H), 3.44–3.39 (m, 2H), 3.18–3.13 (m, 2H), 2.67–2.63 (m, 4H), 1.35 (s, 9H), 1.32–1.30 (m, 2H), 1.24–1.18 (m, 2H); ^13^C NMR (151 MHz, CDCl_3_) *δ* 154.95, 143.31, 136.54, 134.63 (q, *J* = 33.1 Hz), 130.30, 128.55, 127.65, 126.97, 126.51 (q, *J* = 5.6 Hz), 123.30 (q, *J* = 273.1 Hz), 79.79, 47.83, 42.48, 39.77, 38.96, 36.31, 32.38, 28.52; ^19^F NMR (471 MHz, DMSO-*d*_6_) *δ* −61.57; ESI-HR-MS: (*m*/*z*) calcd. for C_25_H_31_F_3_N_2_NaO_4_S ([M+Na]^+^) 535.1849, found 535.1851.

Synthesis of *tert*-butyl 4-benzyl-4-(((4-fluoro-3-(trifluoromethyl)phenyl)sulfonamido)methyl)piperidine-1-carboxylate (**75-29**). Compound **75-29** was prepared according to General Procedure 1 from **73** (0.80 g, 2.63 mmol) using 4-fluoro-3-(trifluoromethyl)benzenesulfonyl chloride (0.83 g, 3.16 mmol) and Et_3_N (0.80 g, 7.89 mmol) in dried CH_2_Cl_2_ (8 mL). Purification by column chromatography (EtOAc/PE = 1/3, by *v*/*v*) gave **75-29**. White solid, 0.96 g (69%). m.p. 196.2–197.1 °C. ^1^H NMR (500 MHz, DMSO-*d*_6_) *δ* 8.21–8.16 (m, 2H), 7.86 (t, *J* = 6.3 Hz, 1H), 7.81–7.77 (m, 1H), 7.21–7.17 (m, 3H), 7.10–7.08 (m, 2H), 3.43–3.38 (m, 2H), 3.20–3.15 (m, 2H), 2.66–2.63 (m, 4H), 1.35 (s, 9H), 1.31–1.29 (m, 2H), 1.23–1.18 (m, 2H); ^13^C NMR (151 MHz, CDCl_3_) *δ* 161.70 (d, *J* = 264.1 Hz), 155.15, 136.93 (d, *J* = 3.9 Hz), 136.44, 133.10 (d, *J* = 9.8 Hz), 130.33, 128.05, 126.62, 126.49, 122.00 (q, *J* = 237.8 Hz), 119.56–118.88 (m), 117.99 (d, *J* = 27.4 Hz), 77.94, 47.49, 41.49, 39.69, 38.73, 35.89, 31.57, 28.14; ^19^F NMR (471 MHz, CD_3_OD) *δ* −59.72 (d, *J* = 11.8 Hz), −108.93 (q, *J* = 12.2 Hz); ESI-HR-MS: (*m*/*z*) calcd. for C_25_H_30_F_4_N_2_NaO_4_S ([M+Na]^+^) 553.1755, found 553.1755.

Synthesis of *tert*-butyl 4-benzyl-4-(((4-(trifluoromethoxy)phenyl)sulfonamido)methyl)piperidine-1-carboxylate (**75-30**). Compound **75-30** was prepared according to General Procedure 1 from **73** (0.80 g, 2.63 mmol) using 4-(trifluoromethoxy)benzenesulfonyl chloride (0.82 g, 3.16 mmol) and Et_3_N (0.80 g, 7.89 mmol) in dried CH_2_Cl_2_ (8 mL). Purification by column chromatography (EtOAc/PE = 1/3, by *v*/*v*) gave **75-30**. White solid, 1.20 g (86%). m.p. 149.8–150.7 °C. ^1^H NMR (500 MHz, DMSO-*d*_6_) *δ* 7.97–7.95 (m, 2H), 7.78 (t, *J* = 6.0 Hz, 1H), 7.61 (d, *J* = 8.5 Hz, 2H), 7.20–7.14 (m, 3H), 7.09–7.07 (m, 2H), 3.44–3.39 (m, 2H), 3.17–3.12 (m, 2H), 2.66–2.63 (m, 4H), 1.35 (s, 9H), 1.31–1.29 (m, 2H), 1.23–1.18 (m, 2H); ^13^C NMR (151 MHz, CDCl_3_) *δ* 154.94, 152.26 (q, *J* = 1.9 Hz), 138.08, 136.55, 130.34, 129.28, 128.44, 126.84, 121.17, 120.33 (q, *J* = 259.6 Hz), 79.71, 47.62, 42.32, 39.77, 38.88, 36.23, 32.31, 28.49; ^19^F NMR (471 MHz, DMSO-*d*_6_) *δ* −56.77; ESI-HR-MS: (*m*/*z*) calcd. for C_25_H_31_F_3_N_2_NaO_5_S ([M+Na]^+^) 551.1798, found 551.1804.

Synthesis of *tert*-butyl 4-benzyl-4-(((4-fluoro-2-(trifluoromethyl)phenyl)sulfonamido)methyl)piperidine-1-carboxylate (**75-31**). Compound **75-31** was prepared according to General Procedure 1 from **73** (0.80 g, 2.63 mmol) using 4-fluoro-2-(trifluoromethyl)benzenesulfonyl chloride (0.82 g, 3.16 mmol) and Et_3_N (0.83 g, 7.89 mmol) in dried CH_2_Cl_2_ (8 mL). Purification by column chromatography (EtOAc/PE = 1/3, by *v*/*v*) gave **75-31**. White solid, 1.22 g (87%). m.p. 187.5–188.5 °C. ^1^H NMR (500 MHz, DMSO-*d*_6_) *δ* 8.16–8.11 (m, 2H), 7.91 (dd, *J* = 2.8 Hz and 9.3 Hz, 1H), 7.82–7.78 (m, 1H), 7.27–7.24 (m, 2H), 7.21–7.17 (m, 1H), 7.15–7.13 (m, 2H), 3.43–3.39 (m, 2H), 3.21–2.18 (m, 2H), 2.81 (d, *J* = 6.0 Hz, 2H), 2.66 (s, 2H), 1.38–1.35 (m, 2H), 1.35 (s, 9H), 1.24–1.19 (m, 2H); ^13^C NMR (151 MHz, CDCl_3_) *δ* 165.19, 163.48, 154.94, 136.59, 134.97 (d, *J* = 8.8 Hz), 134.59 (d, *J* = 3.9 Hz), 130.23, 128.63, 126.99, 122.22 (qd, *J* = 2.1 and 274.3 Hz), 119.11 (d, *J* = 20.8 Hz), 116.94 (dq, *J* = 6.4 and 26.1 Hz), 79.76, 47.87, 42.45, 39.75, 38.90, 36.47, 32.45, 28.54; ^19^F NMR (471 MHz, DMSO-*d*_6_) *δ* −56.52, −105.12; ESI-HR-MS: (*m*/*z*) calcd. for C_25_H_30_F_4_N_2_NaO_4_S ([M+Na]^+^) 553.1755, found 553.1754.

Synthesis of *tert*-butyl 4-benzyl-4-((naphthalene-2-sulfonamido)methyl)piperidine-1-carboxylate (**75-32**). Compound **75-32** was prepared according to General Procedure 1 from **73** (0.80 g, 2.63 mmol) using naphthalene-2-sulfonyl chloride (0.72 g, 3.16 mmol) and Et_3_N (0.83 g, 7.89 mmol) in dried CH_2_Cl_2_ (8 mL). Purification by column chromatography (EtOAc/PE = 1/3, by *v*/*v*) gave **75-32**. White solid, 1.12 g (86%). m.p. 134.1–134.8 °C. ^1^H NMR (500 MHz, DMSO-*d*_6_) *δ* 8.44 (s, 1H), 8.16 (d, *J* = 8.5 Hz, 2H), 8.06 (d, *J* = 8.0 Hz, 1H), 7.88 (dd, *J* = 2.0 Hz and 3.5 Hz, 1H), 7.74–7.66 (m, 3H), 7.11–7.05 (m, 5H), 3.42–3.37 (m, 2H), 3.17–3.12 (m, 2H), 2.51–2.79 (m, 4H), 1.33 (s, 9H), 1.31–1.29 (m, 2H), 1.22–1.17 (m, 2H); ^13^C NMR (151 MHz, CDCl_3_) *δ* 154.92, 136.64, 136.39, 134.94, 132.24, 130.39, 129.77, 129.34, 129.02, 128.59, 128.35, 128.04, 127.78, 126.70, 122.24, 79.60, 47.43, 42.44, 39.79, 38.90, 36.18, 32.36, 28.50; ESI-HR-MS: (*m*/*z*) calcd. for C_28_H_35_N_2_O_4_S ([M+H]^+^) 495.2312, found 495.2308.

Synthesis of *tert*-butyl 4-benzyl-4-((naphthalene-1-sulfonamido)methyl)piperidine-1-carboxylate (**75-33**). Compound **75-33** was prepared according to General Procedure 1 from **73** (0.80 g, 2.63 mmol) using naphthalene-1-sulfonyl chloride (0.72 g, 3.16 mmol) and Et_3_N (0.83 g, 7.89 mmol) in dried CH_2_Cl_2_ (8 mL). Purification by column chromatography (EtOAc/PE = 1/3, by *v*/*v*) gave **75-33**. White solid, 1.03 g (79%). m.p. 149.9–151.1 °C. ^1^H NMR (500 MHz, DMSO-*d*_6_) *δ* 8.80 (d, *J* = 8.5 Hz, 1H), 8.23 (d, *J* = 8.0 Hz, 1H), 8.12–8.09 (m, 2H), 7.96 (t, *J* = 6.3 Hz, 1H), 7.78–7.74 (m, 1H), 7.72–7.68 (m, 1H), 7.65–7.62 (m, 1H), 7.11–7.04 (m, 3H), 6.97–6.95 (m, 2H), 3.36–3.31 (m, 2H), 3.00 (s, 2H), 2.63 (d, *J* = 6.0 Hz, 2H), 2.58 (s, 2H), 1.33 (s, 9H), 1.27–1.22 (m, 2H), 1.17–1.11 (m, 2H); ^13^C NMR (151 MHz, CDCl_3_) *δ* 154.84, 136.57, 134.49, 134.37, 134.29, 130.26, 129.87, 129.34, 128.62, 128.32, 128.15, 127.10, 126.63, 124.29, 124.27, 79.53, 47.07, 42.84, 39.68, 38.88, 36.13, 32.37, 28.49; ESI-HR-MS: (*m*/*z*) calcd. for C_28_H_35_N_2_O_4_S ([M+H]^+^) 495.2312, found 495.2300.

Synthesis of *tert*-butyl 4-benzyl-4-(((4-cyclohexylphenyl)sulfonamido)methyl)piperidine-1-carboxylate (**75-34**). Compound **75-34** was prepared according to General Procedure 1 from **73** (0.80 g, 2.63 mmol) using 4-cyclohexylbenzenesulfonyl chloride (0.82 g, 3.16 mmol) and Et_3_N (0.83 g, 7.89 mmol) in dried CH_2_Cl_2_ (8 mL). Purification by column chromatography (EtOAc/PE = 1/3, by *v*/*v*) gave **75-34**. White solid, 1.16 g (84%). m.p. 169.4–170.5 °C. ^1^H NMR (500 MHz, DMSO-*d*_6_) *δ* 7.72 (d, *J* = 8.5 Hz, 2H), 7.55 (t, *J* = 6.5 Hz, 1H), 7.45 (d, *J* = 8.0 Hz, 2H), 7.20–7.13 (m, 3H), 7.08–7.06 (m, 2H), 3.43–3.38 (m, 2H), 3.12–3.06 (m, 2H), 2.61–2.58 (m, 5H), 1.81–1.78 (m, 4H), 1.73–1.70 (m, 1H), 1.47–1.37 (m, 4H), 1.35 (s, 9H), 1.32–1.28 (m, 2H), 1.23–1.17 (m, 2H); ^13^C NMR (151 MHz, CDCl_3_) *δ* 154.93, 153.54, 136.84, 136.70, 130.43, 128.40, 127.78, 127.26, 126.70, 79.60, 47.26, 44.66, 42.54, 39.77, 38.93, 36.20, 34.22, 32.41, 28.53, 26.76, 26.06; ESI-HR-MS: (*m*/*z*) calcd. for C_30_H_42_N_2_O_4_S ([M+H]^+^) 549.2763, found 549.2758.

Synthesis of *tert*-butyl 4-benzyl-4-(((3-(methylsulfonyl)phenyl)sulfonamido)methyl)piperidine-1-carboxylate (**75-35**). Compound **75-35** was prepared according to General Procedure 1 from **73** (0.80 g, 2.63 mmol) using 3-(methylsulfonyl)benzenesulfonyl chloride (0.80 g, 3.16 mmol) and Et_3_N (0.83 g, 7.89 mmol) in dried CH_2_Cl_2_ (8 mL). Purification by column chromatography (EtOAc/PE = 1/3, by *v*/*v*) gave **75-35**. White solid, 1.04 g (76%). m.p. 155.3–156.1 °C. ^1^H NMR (500 MHz, DMSO-*d*_6_) *δ* 8.33–8.32 (m, 1H), 8.24–8.22 (m, 1H), 8.16–8.14 (m, 1H), 7.93–7.88 (m, 2H), 7.22–7.16 (m, 3H), 7.09–7.08 (m, 2H), 3.43–3.38 (m, 2H), 3.32 (s, 3H), 3.18 (br, 2H), 2.68–2.63 (m, 4H), 1.35 (s, 9H), 1.32–1.30 (m, 2H), 1.23–1.18 (m, 2H); ^13^C NMR (151 MHz, CDCl_3_) *δ* 154.94, 142.19, 141.85, 136.55, 132.04, 131.33, 130.67, 130.37, 128.54, 126.93, 126.28, 79.75, 47.89, 44.46, 42.41, 39.76, 38.91, 36.29, 32.22, 28.52; ESI-HR-MS: (*m*/*z*) calcd. for C_25_H_34_N_2_NaO_6_S_2_ ([M+Na]^+^) 545.1750, found 545.1750.

Synthesis of *tert*-butyl 4-benzyl-4-(((4-propylphenyl)sulfonamido)methyl)piperidine-1-carboxylate (**75-36**). Compound **75-36** was prepared according to General Procedure 1 from **73** (0.80 g, 2.63 mmol) using 4-*n*-propylbenzenesulfonyl chloride (0.69 g, 3.16 mmol) and Et_3_N (0.83 g, 7.89 mmol) in dried CH_2_Cl_2_ (8 mL). Purification by column chromatography (EtOAc/PE = 1/3, by *v*/*v*) gave **75-36**. White solid, 1.13 g (88%). m.p. 161.5–162.7 °C. ^1^H NMR (500 MHz, DMSO-*d*_6_) *δ* 7.72 (d, *J* = 8.0 Hz, 2H), 7.55 (t, *J* = 6.5 Hz, 1H), 7.41 (d, *J* = 8.0 Hz, 2H), 7.20–7.14 (m, 3H), 7.09–7.07 (m, 2H), 3.43–3.38 (m, 2H), 3.14–3.09 (m, 2H), 2.66–2.60 (m, 6H), 1.66–1.58 (m, 2H), 1.35 (s, 9H), 1.32–1.28 (m, 2H), 1.21–1.16 (m, 2H), 0.893 (t, *J* = 7.3 Hz, 3H); ^13^C NMR (151 MHz, CDCl_3_) *δ* 154.94, 148.36, 136.82, 136.71, 130.42, 129.34, 128.41, 127.19, 126.73, 79.62, 47.32, 42.55, 39.79, 38.93, 37.96, 36.20, 32.41, 28.53, 24.29, 13.83; ESI-HR-MS: (*m*/*z*) calcd. for C_27_H_38_N_2_NaO_4_S ([M+Na]^+^) 509.2444, found 509.2444.

Synthesis of *tert*-butyl 4-benzyl-4-(((2,4,6-triisopropylphenyl)sulfonamido)methyl)piperidine-1-carboxylate (**75-37**). Compound **75-37** was prepared according to General Procedure 1 from **73** (0.80 g, 2.63 mmol) using 2,4,6-triisopropylbenzenesulfonyl chloride (0.96 g, 3.16 mmol) and Et_3_N (0.83 g, 7.89 mmol) in dried CH_2_Cl_2_ (8 mL). Purification by column chromatography (EtOAc/PE = 1/3, by *v*/*v*) gave **75-37**. White solid, 1.16 g (77%). m.p. 129.5–130.3 °C. ^1^H NMR (500 MHz, DMSO-*d*_6_) *δ* 7.51 (t, *J* = 6.5 Hz, 1H), 7.22 (s, 2H), 7.20–7.14 (m, 3H), 7.08–7.06 (m, 2H), 4.18–4.13 (m, 2H), 3.37–3.33 (m, 2H), 3.14–3.09 (m, 2H), 2.94–2.88 (m, 1H), 2.70 (d, *J* = 6.0 Hz, 2H), 2.63 (s, 2H), 1.34 (s, 9H), 1.31–1.29 (m, 2H), 1.20–1.16 (m, 20H); ^13^C NMR (151 MHz, CDCl_3_) *δ* 154.89, 152.99, 150.29, 136.77, 132.24, 130.36, 128.45, 126.71, 123.99, 79.62, 46.99, 42.29, 39.81, 38.96, 36.30, 34.22, 32.34, 29.87, 28.50, 25.09, 23.65; ESI-HR-MS: (*m*/*z*) calcd. for C_33_H_50_N_2_NaO_4_S ([M+Na]^+^) 593.3383, found 593.3381.

Synthesis of *tert*-butyl 4-(([1,1′-biphenyl]-4-sulfonamido)methyl)-4-benzylpiperidine-1-carboxylate (**75-38**). Compound **75-38** was prepared according to General Procedure 1 from **73** (0.80 g, 2.63 mmol) using [1,1′-biphenyl]-4-sulfonyl chloride (0.80 g, 3.16 mmol) and Et_3_N (0.83 g, 7.89 mmol) in dried CH_2_Cl_2_ (8 mL). Purification by column chromatography (EtOAc/PE = 1/3, by *v*/*v*) gave **75-38**. White solid, 1.23 g (90%). m.p. 146.3–147.9 °C. ^1^H NMR (500 MHz, DMSO-*d*_6_) *δ* 7.92–7.88 (m, 4H), 7.76–7.75 (m, 2H), 7.69 (t, *J* = 6.5 Hz, 1H), 7.54–7.51 (m, 2H), 7.47–7.43 (m, 1H), 7.20–7.13 (m, 3H), 7.10–7.08 (m, 2H), 3.44–3.39 (m, 2H), 3.18–3.14 (m, 2H), 2.67–2.64 (m, 4H), 1.34 (s, 9H), 1.32–1.31 (m, 2H), 1.24–1.19 (m, 2H); ^13^C NMR (151 MHz, CDCl_3_) *δ* 154.93, 145.82, 139.28, 138.16, 136.66, 130.42, 129.18, 128.64, 128.39, 127.92, 127.66, 127.41, 126.72, 79.61, 47.41, 42.40, 39.79, 38.95, 36.21, 32.38, 28.51; ESI-HR-MS: (*m*/*z*) calcd. for C_30_H_36_N_2_NaO_4_S_2_ ([M+Na]^+^) 543.2288, found 543.2289.

Synthesis of *tert*-butyl 4-benzyl-4-(((4-methylphenyl)sulfonamido)methyl)piperidine-1-carboxylate (**75-39**). Compound **75-39** was prepared according to General Procedure 1 from **73** (0.80 g, 2.63 mmol) using 4-toluenesulfonyl chloride (0.60 g, 3.16 mmol) and Et_3_N (0.83 g, 7.89 mmol) in dried CH_2_Cl_2_ (8 mL). Purification by column chromatography (EtOAc/PE = 1/3, by *v*/*v*) gave **75-39**. White solid, 1.04 g (86%). m.p. 172.5–173.7 °C. ^1^H NMR (500 MHz, DMSO-*d*_6_) *δ* 7.70 (d, *J* = 8.0 Hz, 2H), 7.54 (t, *J* = 6.3 Hz, 1H), 7.40 (d, *J* = 8.0 Hz, 2H), 7.21–7.15 (m, 3H), 7.09–7.07 (m, 2H), 3.43–3.38 (m, 2H), 3.14 (s, 2H), 2.62–2.58 (m, 4H), 2.39 (s, 3H), 1.35 (s, 9H), 1.32–1.28 (m, 2H), 1.22–1.17 (m, 2H); ^13^C NMR (151 MHz, CDCl_3_) *δ* 154.92, 143.66, 136.69, 136.62, 130.44, 129.90, 128.33, 127.15, 126.65, 79.56, 47.26, 42.39, 39.77, 38.89, 36.12, 32.34, 28.50, 21.62; ESI-HR-MS: (*m*/*z*) calcd. for C_25_H_34_N_2_NaO_4_S ([M+Na]^+^) 481.2131, found 481.2128.

Synthesis of *tert*-butyl 4-benzyl-4-(((4-ethylphenyl)sulfonamido)methyl)piperidine-1-carboxylate (**75-40**). Compound **75-40** was prepared according to General Procedure 1 from **73** (0.80 g, 2.63 mmol) using 4-ethylbenzenesulfonyl chloride (0.65 g, 3.16 mmol) and Et_3_N (0.83 g, 7.89 mmol) in dried CH_2_Cl_2_ (8 mL). Purification by column chromatography (EtOAc/PE = 1/3, by *v*/*v*) gave **75-40**. White solid, 1.07 g (86%). m.p 166.2–167.2 °C. ^1^H NMR (500 MHz, DMSO-*d*_6_) *δ* 7.73 (d, *J* = 8.0 Hz, 2H), 7.55 (t, *J* = 6.5 Hz, 1H), 7.44 (d, *J* = 8.0 Hz, 2H), 7.20–7.14 (m, 3H), 7.09–7.07 (m, 2H), 3.43–3.38 (m, 2H), 3.15–3.10 (m, 2H), 2.72–2.67 (m, 2H), 2.62–2.60 (m, 4H), 1.35 (s, 9H), 1.33–1.28 (m, 2H), 1.22–1.17 (m, 2H); ^13^C NMR (151 MHz, CDCl_3_) *δ* 154.94, 149.80, 136.79, 136.69, 130.43, 128.76, 128.35, 127.26, 126.67, 79.61, 47.26, 42.42, 39.78, 38.90, 36.15, 32.36, 28.90, 28.51, 15.23; ESI-HR-MS: (*m*/*z*) calcd. for C_26_H_36_N_2_NaO_4_S ([M+Na]^+^) 495.2288, found 495.2284.

Synthesis of *tert*-butyl 4-benzyl-4-(((4-isopropylphenyl)sulfonamido)methyl)piperidine-1-carboxylate (**75-41**). Compound **75-41** was prepared according to General Procedure 1 from **73** (0.80 g, 2.63 mmol) using 4-isopropylbenzenesulfonyl chloride (0.69 g, 3.16 mmol) and Et_3_N (0.83 g, 7.89 mmol) in dried CH_2_Cl_2_ (8 mL). Purification by column chromatography (EtOAc/PE = 1/3, by *v*/*v*) gave **75-41**. White solid, 1.04 g (81%). m.p. 186.0–186.5 °C. ^1^H NMR (500 MHz, DMSO-*d*_6_) *δ* 7.75–7.72 (m, 2H), 7.56 (t, *J* = 6.3 Hz, 1H), 7.48–7.46 (m, 2H), 7.20–7.13 (m, 3H), 7.08–7.06 (m, 2H), 3.43–3.38 (m, 2H), 3.13–3.08 (m, 2H), 3.01–2.96 (m, 1H), 2.62–2.60 (m, 4H), 1.35 (s, 9H), 1.32–1.28 (m, 2H), 1.23 (d, *J* = 7.0 Hz, 6H), 1.20–1.17 (m, 2H); ^13^C NMR (151 MHz, CDCl_3_) *δ* 154.91, 154.36, 136.90, 136.69, 130.44, 128.33, 127.38, 127.30, 126.64, 79.56, 47.20, 42.40, 39.77, 38.89, 36.16, 34.26, 32.36, 28.51, 23.75; ESI-HR-MS: (*m*/*z*) calcd. for C_27_H_38_N_2_NaO_4_S ([M+Na]^+^) 509.2444, found 509.2442.

Synthesis of *tert*-butyl 4-benzyl-4-(((3-isopropylphenyl)sulfonamido)methyl)piperidine-1-carboxylate (**75-42**). Compound **75-42** was prepared according to General Procedure 1 from **73** (0.80 g, 2.63 mmol) using 3-isopropylbenzenesulfonyl chloride (0.69 g, 3.16 mmol) and Et_3_N (0.83 g, 7.89 mmol) in dried CH_2_Cl_2_ (8 mL). Purification by column chromatography (EtOAc/PE = 1/3, by *v*/*v*) gave **75-42**. White solid, 1.01 g (79%). m.p. 158.2–160.2 °C. ^1^H NMR (500 MHz, DMSO-*d*_6_) *δ* 7.69 (s, 1H), 7.63–7.61 (m, 1H), 7.58 (t, *J* = 6.3 Hz, 1H), 7.55–7.51 (m, 2H), 7.20–7.16 (m, 3H), 7.08–7.06 (m, 2H), 3.41–3.36 (m, 2H), 3.14–3.10 (m, 2H), 3.03–2.97 (m, 1H), 2.62–2.60 (m, 4H), 1.35 (s, 9H), 1.32–1.28 (m, 2H), 1.23 (d, *J* = 7.0 Hz, 6H), 1.20–1.16 (m, 2H); ^13^C NMR (201 MHz, CDCl_3_) *δ* 154.91, 150.50, 139.50, 136.64, 131.14, 130.40, 129.30, 128.35, 126.67, 124.99, 124.58, 79.58, 47.20, 42.42, 39.83, 38.87, 36.13, 34.19, 32.35, 28.50, 23.87; ESI-HR-MS: (*m*/*z*) calcd. for C_27_H_38_N_2_NaO_4_S ([M+Na]^+^) 509.2444, found 509.2444.

Synthesis of *tert*-butyl 4-benzyl-4-(((2-methylphenyl)sulfonamido)methyl)piperidine-1-carboxylate (**75-43**). Compound **75-43** was prepared according to General Procedure 1 from **73** (0.80 g, 2.63 mmol) using 2-methylbenzenesulfonyl chloride (0.60 g, 3.16 mmol) and Et_3_N (0.83 g, 7.89 mmol) in dried CH_2_Cl_2_ (8 mL). Purification by column chromatography (EtOAc/PE = 1/3, by *v*/*v*) gave **75-43**. White solid, 1.07 g (89%). m.p. 174.2–175.3 °C. ^1^H NMR (500 MHz, DMSO-*d*_6_) *δ* 7.79–7.77 (m, 1H), 7.73–7.69 (m, 1H), 7.53–7.50 (m, 1H), 7.42–7.36 (m, 2H), 7.24–7.15 (m, 3H), 7.10–7.07 (m, 2H), 3.40–3.32 (m, 2H), 3.14 (s, 2H), 2.69–2.68 (m, 1H), 2.63–2.58 (m, 5H), 2.39 (s, 1H), 1.34 (s, 9H), 1.30–1.28 (m, 2H), 1.20–1.15 (m, 2H); ^13^C NMR (151 MHz, CDCl_3_) *δ* 154.93, 137.66, 136.90, 136.82, 133.02, 132.76, 130.31, 129.59, 128.58, 126.89, 126.43, 79.68, 47.36, 42.91, 39.79, 38.91, 36.36, 32.48, 28.54, 20.44; ESI-HR-MS: (*m*/*z*) calcd. for C_25_H_34_N_2_NaO_4_S ([M+Na]^+^) 481.2131, found 481.2128.

Synthesis of *tert*-butyl 4-benzyl-4-(((4-hydroxyphenyl)sulfonamido)methyl)piperidine-1-carboxylate (**75-44**). Compound **75-44** was prepared according to General Procedure 1 from **73** (0.80 g, 2.63 mmol) using 4-hydroxybenzenesulfonyl chloride (0.61 g, 3.16 mmol) and Et_3_N (0.83 g, 7.89 mmol) in dried CH_2_Cl_2_ (8 mL). Purification by column chromatography (EtOAc/PE = 1/3, by *v*/*v*) gave **75-44**. White solid, 1.06 g (88%). m.p. 200.0–203.1 °C. ^1^H NMR (500 MHz, DMSO-*d*_6_) *δ* 10.36 (s, 1H), 7.64–7.62 (m, 2H), 7.36 (t, *J* = 6.5 Hz, 1H), 7.21–7.14 (m, 3H), 7.08–7.06 (m, 2H), 6.92–6.90 (m, 2H), 3.43–3.38 (m, 2H), 3.17–3.13 (m, 2H), 2.61–2.56 (m, 4H), 1.35 (s, 9H), 1.32–1.28 (m, 2H), 1.21–1.16 (m, 2H); ^13^C NMR (151 MHz, CDCl_3_ + CD_3_OD) *δ* 161.04, 155.06, 136.46, 130.22, 129.60, 128.88, 127.79, 126.12, 115.43, 79.73, 46.84, 41.62, 39.59, 38.61, 35.59, 31.53, 27.88; ESI-HR-MS: (*m*/*z*) calcd. for C_24_H_32_N_2_NaO_5_S ([M+Na]^+^) 483.1924, found 483.1920.

Synthesis of *tert*-butyl 4-benzyl-4-(((4-carbamoylphenyl)sulfonamido)methyl)piperidine-1-carboxylate (**75-45**). Compound **75-45** was prepared according to General Procedure 1 from **73** (0.80 g, 2.63 mmol) using 4-carbamoylbenzenesulfonyl chloride (0.69 g, 3.16 mmol) and Et_3_N (0.83 g, 7.89 mmol) in dried CH_2_Cl_2_ (8 mL). Purification by column chromatography (EtOAc/PE = 1/3, by *v*/*v*) gave **75-45**. White solid, 1.06 g (83%). m.p. 157.5–158.6 °C. ^1^H NMR (500 MHz, DMSO-*d*_6_) *δ* 8.17 (s, 1H), 8.06–8.04 (m, 2H), 7.89–7.87 (m, 2H), 7.74 (t, *J* = 6.5 Hz, 1H), 7.61 (s, 1H), 7.21–7.14 (m, 3H), 7.09–7.07 (m, 2H), 3.43–3.39 (m, 2H), 3.19–3.15 (m, 2H), 2.65–2.62 (m, 4H), 1.35 (s, 9H), 1.32–1.29 (m, 2H), 1.23–1.17 (m, 2H); ^13^C NMR (151 MHz, CDCl_3_) *δ* 168.39, 155.03, 142.89, 137.43, 136.64, 130.47, 128.54, 128.42, 127.28, 126.81, 79.82, 47.72, 42.22, 39.83, 38.94, 36.22, 32.13, 28.53; ESI-HR-MS: (*m*/*z*) calcd. for C_25_H_33_N_3_NaO_5_S ([M+Na]^+^) 510.2033, found 510.2032.

Synthesis of *tert*-butyl 4-benzyl-4-(((4-(methoxycarbonyl)phenyl)sulfonamido)methyl)piperidine-1-carboxylate (**75-46**). Compound **75-46** was prepared according to General Procedure 1 from **73** (1.50 g, 4.93 mmol) using methyl 4-(chlorosulfonyl)benzoate (1.39 g, 5.92 mmol) and Et_3_N (1.50 g, 14.8 mmol) in dried CH_2_Cl_2_ (15 mL). Purification by column chromatography (EtOAc/PE = 1/3, by *v*/*v*) gave **75-46**. White solid, 2.06 g (83%). m.p. 174.0–175.2 °C. ^1^H NMR (500 MHz, DMSO-*d*_6_) *δ* 8.17–8.14 (m, 2H), 7.97–7.95 (m, 2H), 7.84 (t, *J* = 6.5 Hz, 1H), 7.21–7.14 (m, 3H), 7.10–7.08 (m, 2H), 3.90 (s, 3H), 3.42–3.38 (m, 2H), 3.18–3.13 (m, 2H), 2.67–2.62 (m, 4H), 1.34 (s, 9H), 1.31–1.28 (m, 2H), 1.22–1.17 (m, 2H); ^13^C NMR (151 MHz, CDCl_3_) *δ* 165.67, 154.92, 143.63, 136.57, 134.06, 130.52, 130.33, 128.51, 127.12, 126.90, 79.71, 52.80, 47.73, 42.53, 39.77, 38.92, 36.27, 32.34, 28.51; ESI-HR-MS: (*m*/*z*) calcd. for C_26_H_34_N_2_NaO_6_S ([M+Na]^+^) 525.2030, found 525.2027.

Synthesis of *tert*-butyl 4-benzyl-4-(((3-nitrophenyl)sulfonamido)methyl)piperidine-1-carboxylate (**75-47**). Compound **75-47** was prepared according to General Procedure 1 from **73** (2.00 g, 6.57 mmol) using methyl 3-nitrobenzenesulfonyl chloride (1.75 g, 7.88 mmol) and Et_3_N (2.00 g, 19.7 mmol) in dried CH_2_Cl_2_ (20 mL). Purification by column chromatography (EtOAc/PE = 1/3, by *v*/*v*) gave **75-47**. White solid, 2.67 g (83%). m.p. 20.1–208.4 °C. ^1^H NMR (500 MHz, DMSO-*d*_6_) *δ* 8.57 (t, *J* = 2.0 Hz, 1H)), 8.51–8.48 (m, 1H), 8.25–8.23 (m, 1H), 7.96–7.91 (m, 2H), 7.22–7.15 (m, 3H), 7.12–7.10 (m, 2H), 3.43–3.38 (m, 2H), 3.21–3.16 (m, 2H), 2.67–2.63 (m, 4H), 1.35 (s, 9H), 1.32–1.30 (m, 2H), 1.23–1.18 (m, 2H); ^13^C NMR (151 MHz, CDCl_3_) *δ* 155.04, 148.12, 142.26, 136.30, 132.25, 130.40, 130.22, 127.79, 126.66, 126.21, 121.73, 79.77, 47.33, 41.25, 39.53, 38.58, 35.72, 31.28, 27.83; ESI-HR-MS: (*m*/*z*) calcd. for C_24_H_31_N_3_NaO_6_S ([M+Na]^+^) 512.1826, found 512.1827.

Synthesis of N-((1-([2,4′-bipyridine]-3-carbonyl)-4-benzylpiperidin-4-yl)methyl)methanesulfonamide (**C-4-1**). Following General Procedure 3, **75-1** (0.30 g, 0.784 mmol) was first treated with TFA (1.5 mL) in CH_2_Cl_2_ (0.9 mL), and the intermediate obtained was reacted with **7·TFA** (0.28 g, 0.941 mmol) using EDCI (0.38 g, 1.96 mmol), HOBT (8 mg, 0.059 mmol), and DIPEA (0.61 g, 4.70 mmol) in dried THF (3 mL). Purification by column chromatography (EtOAc) gave **C-1-1**. White solid, 0.26 g (71%). m.p. 112.1–114.5 °C. ^1^H NMR (500 MHz, DMSO-*d*_6_) *δ* 8.57 (t, *J* = 2.0 Hz, 1H)), 8.51–8.48 (m, 1H), 8.25–8.23 (m, 1H), 7.96–7.91 (m, 2H), 7.22–7.15 (m, 3H), 7.12–7.10 (m, 2H), 3.43–3.38 (m, 2H), 3.21–3.16 (m, 2H), 2.67–2.63 (m, 4H), 1.35 (s, 9H), 1.32–1.30 (m, 2H), 1.23–1.18 (m, 2H); ^13^C NMR (151 MHz, CDCl_3_) *δ* 155.04, 148.12, 142.26, 136.30, 132.25, 130.40, 130.22, 127.79, 126.66, 126.21, 121.73, 79.77, 47.33, 41.25, 39.53, 38.58, 35.72, 31.28, 27.83; ESI-HR-MS: (*m*/*z*) calcd. for C_25_H_29_N_4_O_3_S ([M+H]^+^) 465.1955 found 465.1953.

Synthesis of N-((1-([2,4′-bipyridine]-3-carbonyl)-4-benzylpiperidin-4-yl)methyl)-1,1,1-trifluoromethanesulfonamide (**C-4-2**). Following General Procedure 3, **75-2** (0.65 g, 1.49 mmol) was first treated with TFA (3.3 mL) in CH_2_Cl_2_ (2 mL), and the intermediate obtained was reacted with **7·TFA** (0.53 g, 1.79 mmol) using EDCI (0.72 g, 3.73 mmol), HOBT (15 mg, 0.112 mmol), and DIPEA (1.16 g, 8.94 mmol) in dried THF (6.5 mL). Purification by column chromatography (EtOAc) gave **C-4-2**. White solid, 0.33 g (43%). m.p. 116.2–118.3 °C. ^1^H NMR (500 MHz, CDCl_3_) *δ* 8.77–8.76 (m, 1H)), 8.68–8.64 (m, 2H), 7.72–7.64 (m, 3H), 7.42–7.39 (m, 1H), 7.27–7.25 (m, 3H), 7.01–6.93 (m, 2H), 3.95–3.92 (m, 0.50H), 3.76–3.62 (m, 1H), 3.46–3.39 (m, 0.73H), 3.15–2.81 (m, 4H), 2.62 (s, 1H), 2.42–2.32 (m, 1H), 1.53–1.43 (m, 1H), 1.34–1.09 (m, 2H), 0.71–0.50 (m, 1H); ^13^C NMR (126 MHz, CDCl_3_) *δ* 167.74, 167.62, 151.99, 151.80, 150.67, 149.96, 146.61, 146.56, 136.24, 136.10, 135.85, 135.63, 131.30, 130.33, 130.12, 128.72, 128.67, 127.27, 127.16, 123.73, 123.48, 123.38, 121.26, 118.69, 50.71, 49.72, 47.91, 43.00, 42.66, 41.06, 37.49, 36.29, 36.19, 31.65, 31.29, 30.98, 30.62; ESI-HR-MS: (*m*/*z*) calcd. for C_25_H_26_F_3_N_4_O_3_S ([M+H]^+^) 519.1673, found 519.1667.

Synthesis of N-((1-([2,4′-bipyridine]-3-carbonyl)-4-benzylpiperidin-4-yl)methyl)ethanesulfonamide (**C-4-3**). Following General Procedure 3, **75-3** (0.75 g, 1.89 mmol) was first treated with TFA (3.8 mL) in CH_2_Cl_2_ (2.3 mL), and the intermediate obtained was reacted with **7·TFA** (0.68 g, 2.27 mmol) using EDCI (0.91 g, 4.73 mmol), HOBT (19 mg, 0.142 mmol), and DIPEA (1.46 g, 11.3 mmol) in dried THF (7.5 mL). Purification by column chromatography (EtOAc) gave **C-4-3**. White solid, 0.75 g (82%). m.p. 104.6–106.2 °C. ^1^H NMR (500 MHz, DMSO-*d*_6_) *δ* 8.78 (dd, *J* = 1.5 Hz and 5.0 Hz, 1H), 8.68–8.64 (m, 2H), 7.84–7.79 (m, 1H), 7.59–7.54 (m, 3H), 7.28–7.20 (m, 4H), 7.11–7.03 (m, 2H), 3.92–3.70 (m, 1H), 3.48–3.46 (m, 0.43H), 3.34–3.33 (m, 1H), 3.18–3.07 (m, 1H), 3.02–2.97 (m, 3H), 2.87–2.76 (m, 1H), 2.64 (s, 1.65H), 2.47–2.34 (m, 1H), 1.51–1.34 (m, 1.54H), 1.20 (t, *J* = 7.3 Hz, 3H), 1.16–1.10 (m, 1.41H), 0.88–0.51 (m, 1H); ^13^C NMR (126 MHz, CDCl_3_) *δ* 167.62, 167.56, 152.08, 151.92, 150.57, 150.31, 150.21, 146.38, 136.27, 136.16, 136.10, 131.54, 130.30, 130.05, 128.68, 128.60, 127.15, 127.02, 123.64, 123.43, 123.31, 47.95, 46.78, 46.60, 46.10, 43.81, 42.76, 42.69, 41.25, 37.57, 37.47, 36.39, 31.94, 31.75, 31.59, 31.30; ESI-HR-MS: (*m*/*z*) calcd. for C_26_H_31_N_4_O_3_S ([M+H]^+^) 479.2112, found 479.2109.

Synthesis of N-((1-([2,4′-bipyridine]-3-carbonyl)-4-benzylpiperidin-4-yl)methyl)cyclopropanesulfonamide (**C-4-4**). Following General Procedure 3, **75-4** (0.55 g, 1.35 mmol) was first treated with TFA (2.76 mL) in CH_2_Cl_2_ (1.7 mL), and the intermediate obtained was reacted with **7·TFA** (0.48 g, 1.62 mmol) using EDCI (0.65 g, 3.38 mmol), HOBT (14 mg, 0.101 mmol), and DIPEA (1.05 g, 8.10 mmol) in dried THF (5.5 mL). Purification by column chromatography (EtOAc) gave **C-4-4**. White solid, 0.45 g (68%). m.p. 134.5–137.1 °C. ^1^H NMR (500 MHz, CD_3_OD) *δ* 8.78–8.77 (m, 1H), 8.68–8.60 (m, 2H), 7.88–7.84 (m, 1H), 7.69–7.68 (m, 2H), 7.59–7.56 (m, 1H), 7.30–7.08 (m, 5H), 4.59 (m, 0.51H), 4.01–3.80 (m, 1H), 3.60–3.41 (m, 1H), 3.22–3.17 (m, 1H), 3.07–2.98 (m, 2H), 2.86–2.79 (m, 1H), 2.69 (s, 1H), 2.55–2.44 (m, 2H), 1.60–1.23 (m, 3H), 1.04–0.97 (m, 4H), 0.70 (m, 0.55H); ^13^C NMR (126 MHz, CD_3_OD) *δ* 169.08, 151.65, 150.57, 148.32, 137.95, 137.66, 132.94, 131.82, 129.18, 127.65, 125.28, 124.88, 47.43, 44.23, 43.52, 41.61, 38.82, 37.20, 32.65, 31.94, 30.31, 5.51, 5.47; ESI-HR-MS: (*m*/*z*) calcd. for C_27_H_31_N_4_O_3_S ([M+H]^+^) 491.2112, found 491.2110.

Synthesis of N-((1-([2,4′-bipyridine]-3-carbonyl)-4-benzylpiperidin-4-yl)methyl)thiophene-2-sulfonamide (**C-4-5**). Following General Procedure 3, **75-5** (0.82 g, 1.82 mmol) was first treated with TFA (4.1 mL) in CH_2_Cl_2_ (2.5 mL), and the intermediate obtained was reacted with **7·TFA** (0.65 g, 2.18 mmol) using EDCI (0.87 g, 4.55 mmol), HOBT (19 mg, 0.137 mmol), and DIPEA (1.41 g, 10.9 mmol) in dried THF (8.2 mL). Purification by column chromatography (EtOAc) gave **C-4-5**. White foam, 0.51 g (53%). ^1^H NMR (500 MHz, DMSO-*d*_6_) *δ* 8.77 (dd, *J* = 1.5 Hz and 4.5 Hz, 1H), 8.67–8.64 (m, 2H), 7.95–7.93 (m, 1H), 7.86–7.81 (m, 2H), 7.60–7.54 (m, 4H), 7.22–7.16 (m, 4H), 7.08–6.98 (m, 2H), 4.12–4.09 (m, 0.25H), 3.86–3.69 (m, 1H), 3.38–3.35 (m, 0.42H), 3.30–3.25 (m, 0.76H), 3.18–3.13 (m, 1H), 3.04–2.99 (m, 1H), 2.81–2.72 (m, 1H), 2.66–2.53 (m, 2H), 2.37–2.35 (m, 0.60H), 1.49–1.46 (m, 0.60H), 1.32–1.22 (m, 1H), 1.14–1.07 (m, 1.65H), 0.84–0.55 (m, 1H); ^13^C NMR (151 MHz, CDCl_3_) *δ* 167.47, 167.37, 151.90, 151.70, 150.40, 149.97, 149.93, 146.26, 146.23, 140.60, 140.46, 136.12, 136.01, 135.99, 135.79, 132.18, 131.98, 131.91, 131.85, 131.33, 130.26, 130.08, 128.28, 127.52, 127.46, 126.74, 126.69, 123.51, 123.28, 123.08, 47.46, 46.27, 42.90, 42.66, 42.51, 41.51, 37.45, 37.39, 35.98, 35.84, 31.83, 31.54, 31.42, 31.19; ESI-HR-MS: (*m*/*z*) calcd. for C_28_H_29_N_4_O_3_S_2_ ([M+H]^+^) 533.1676, found 533.1679.

Synthesis of N-((1-([2,4′-bipyridine]-3-carbonyl)-4-benzylpiperidin-4-yl)methyl)pyridine-3-sulfonamide (**C-4-6**). Following General Procedure 3, **75-6** (0.86 g, 1.93 mmol) was first treated with TFA (4.3 mL) in CH_2_Cl_2_ (2.6 mL), and the intermediate obtained was reacted with **7·TFA** (0.69 g, 2.32 mmol) using EDCI (0.93 g, 4.83 mmol), HOBT (20 mg, 0.145 mmol), and DIPEA (1.50 g, 11.6 mmol) in dried THF (8.6 mL). Purification by column chromatography (EtOAc) gave **C-4-6**. White foam, 0.59 g (58%). ^1^H NMR (500 MHz, DMSO-*d*_6_) *δ* 8.97–8.96 (m, 1H), 8.85–8.83 (m, 1H), 8.78–8.77 (m, 1H), 8.66–8.63 (m, 2H), 8.20–8.19 (m, 1H), 7.89–7.78 (m, 2H), 7.67–7.65 (m, 1H), 7.57–7.54 (m, 3H), 7.19–7.16 (m, 3H), 7.07–6.97 (m, 2H), 3.85–3.68 (m, 1H), 3.42–3.41 (m, 0.40H), 3.29–3.27 (m, 0.60H), 3.17–3.14 (m, 1H), 3.04–2.99 (m, 1H), 2.84–2.70 (m, 1H), 2.63–2.60 (m, 1.49H), 2.46–2.34 (m, 1.51H), 1.47–1.45 (m, 0.56H), 1.33–1.23 (m, 1H), 1.13–1.06 (m, 1.55H), 0.79–0.55 (m, 1H); ^13^C NMR (201 MHz, CDCl_3_ + CD_3_OD) *δ* 167.45, 167.34, 162.43, 162.25, 152.45, 151.58, 151.40, 150.26, 149.39, 149.29, 147.16, 146.40, 137.01, 136.10, 136.01, 135.85, 135.62, 134.91, 134.83, 131.14, 130.13, 130.00, 128.00, 126.52, 126.47, 123.98, 123.67, 123.24, 123.13, 116.96, 115.51, 47.15, 46.13, 42.66, 42.54, 42.03, 40.96, 37.41, 37.34, 35.84, 35.70, 31.31, 30.97, 30.79, 30.64; ESI-HR-MS: (*m*/*z*) calcd. for C_29_H_30_N_5_O_3_S ([M+H]^+^) 528.2064, found 528.2067.

Synthesis of N-((1-([2,4′-bipyridine]-3-carbonyl)-4-benzylpiperidin-4-yl)methyl)-1-phenylmethanesulfonamide (**C-4-7**). Following General Procedure 3, **75-7** (0.63 g, 1.37 mmol) was first treated with TFA (3.2 mL) in CH_2_Cl_2_ (1.9 mL), and the intermediate obtained was reacted with **7·TFA** (0.49 g, 1.64 mmol) using EDCI (0.66 g, 3.43 mmol), HOBT (14 mg, 0.103 mmol), and DIPEA (1.06 g, 8.22 mmol) in dried THF (6.3 mL). Purification by column chromatography (EtOAc) gave **C-4-7**. White foam, 0.49 g (66%). ^1^H NMR (500 MHz, DMSO-*d*_6_) *δ* 8.78–8.77 (m, 1H), 8.71–8.65 (m, 2H), 7.84–7.79 (m, 1H), 7.60–7.54 (m, 3H), 7.40–7.36 (m, 5H), 7.29–7.10 (m, 5H), 7.03–7.01 (m, 1H), 4.35–4.34 (m, 2H), 4.13–3.87 (m, 1H), 3.73–3.38 (m, 1H), 3.31–3.25 (m, 0.61H), 3.18–3.14 (m, 1H), 3.05–3.01 (m, 0.60H), 2.96–2.89 (m, 1H), 2.79–2.68 (m, 1H), 2.58–2.55 (m, 1.58H), 2.43–2.27 (m, 1H), 1.45–1.42 (m, 0.51H), 1.30–1.29 (m, 0.69H), 1.10–1.03 (m, 1.57H), 0.91–0.45 (m, 1H); ^13^C NMR (201 MHz, CDCl_3_) *δ* 167.29, 167.21, 151.76, 151.55, 150.29, 150.26, 149.83, 149.78, 146.16, 136.06, 136.03, 135.93, 135.81, 131.31, 131.26, 130.52, 130.45, 130.15, 129.96, 129.24, 129.13, 128.72, 128.68, 128.59, 128.19, 128.16, 126.64, 126.55, 123.44, 123.42, 123.20, 123.03, 58.57, 58.48, 47.77, 46.01, 43.03, 42.53, 42.43, 41.11, 37.31, 37.23, 36.17, 36.04, 31.41, 31.07, 30.87; ESI-HR-MS: (*m*/*z*) calcd. for C_31_H_33_N_4_O_3_S ([M+H]^+^) 541.2268, found 541.2268.

Synthesis of N-((1-([2,4′-bipyridine]-3-carbonyl)-4-benzylpiperidin-4-yl)methyl)benzenesulfonamide (**C-4-8**). Following General Procedure 3, **75-8** (0.70 g, 1.57 mmol) was first treated with TFA (3.5 mL) in CH_2_Cl_2_ (2.1 mL), and the intermediate obtained was reacted with **7·TFA** (0.56 g, 1.88 mmol) using EDCI (0.75 g, 3.93 mmol), HOBT (16 mg, 0.118 mmol), and DIPEA (1.22 g, 9.42 mmol) in dried THF (7 mL). Purification by column chromatography (EtOAc) gave **C-4-8**. White solid, 0.33 g (40%). m.p. 105–108 °C. ^1^H NMR (500 MHz, CD_3_OD) *δ* 8.76 (dd, *J* = 1.8 Hz and 4.8 Hz, 1H), 8.67–8.58 (m, 2H), 7.86–7.81 (m, 3H), 7.67–7.53 (m, 6H), 7.19–7.17 (m, 3H), 7.05–6.93 (m, 2H), 7.03–7.01 (m, 1H), 3.98–3.78 (m, 1H), 3.44–3.33 (m, 1H), 3.19–3.16 (m, 0.49H), 3.11–2.98 (m, 1H), 2.88–2.72 (m, 1.59H), 2.63–2.38 (m, 3H), 1.53–1.50 (m, 0.59H), 1.44–1.28 (m, 1H), 1.25–0.98 (m, 2H), 0.65–0.60 (m, 0.54H); ^13^C NMR (126 MHz, CDCl_3_) *δ* 167.55, 152.19, 151.94, 150.58, 150.32, 150.22, 146.38, 146.29, 139.61, 136.27, 136.15, 135.96, 132.96, 131.54, 131.46, 130.27, 130.03, 129.37, 128.55, 127.04, 126.94, 123.64, 123.59, 123.43, 123.20, 47.37, 46.11, 43.67, 42.78, 42.62, 41.96, 37.53, 37.46, 36.27, 36.12, 32.08, 31.83, 31.48; ESI-HR-MS: (*m*/*z*) calcd. for C_30_H_31_N_4_O_3_S ([M+H]^+^) 527.2112, found 527.2109.

Synthesis of N-((1-([2,4′-bipyridine]-3-carbonyl)-4-benzylpiperidin-4-yl)methyl)-4-fluorobenzenesulfonamide (**C-4-9**). Following General Procedure 3, **75-9** (0.90 g, 1.95 mmol) was first treated with TFA (4.5 mL) in CH_2_Cl_2_ (2.7 mL), and the intermediate obtained was reacted with **7·TFA** (0.70 g, 2.34 mmol) using EDCI (0.94 g, 4.88 mmol), HOBT (20 mg, 0.146 mmol), and DIPEA (1.51 g, 11.7 mmol) in dried THF (9 mL). Purification by column chromatography (EtOAc) gave **C-4-9**. White foam, 0.93 g (88%). ^1^H NMR (500 MHz, DMSO-*d*_6_) *δ* 8.77 (dd, *J* = 1.8 Hz and 4.8 Hz, 1H), 8.65–8.64 (m, 2H), 7.89–7.79 (m, 3H), 7.70–7.64 (m, 1H), 7.58–7.54 (m, 3H), 7.49–7.43 (m, 2H), 7.19–7.16 (m, 3H), 7.07–7.05 (m, 1H), 6.98–6.96 (m, 1H), 3.87–3.69 (m, 1H), 3.38 (m, 0.34H), 3.27–3.22 (m, 0.63H), 3.17–3.12 (m, 1H), 3.00–2.94 (m, 1H), 2.79 (m, 0.42H), 2.67–2.54 (m, 2H), 2.39–2.33 (m, 1.44H), 1.47–1.45 (m, 0.58H), 1.35–1.23 (m, 1H), 1.13–1.04 (m, 1.68H), 0.80–0.50 (m, 1H); ^13^C NMR (201 MHz, CDCl_3_) *δ* 167.49, 167.47, 167.39, 167.36, 165.51, 164.23, 151.80, 151.64, 150.41, 149.90, 146.23, 136.08, 135.98, 135.81, 135.74, 131.24, 130.20, 130.06, 129.62, 129.58, 129.51, 129.47, 128.24, 128.18, 126.68, 123.51, 123.25, 123.10, 116.42, 116.37, 116.30, 47.26, 45.98, 42.64, 42.48, 41.23, 37.43, 37.37, 36.00, 35.96, 35.85, 35.81, 31.73, 31.36, 31.08, 31.03; ^19^F NMR (471 MHz, DMSO-*d*_6_) *δ* −106.93, −107.00; ESI-HR-MS: (*m*/*z*) calcd. for C_30_H_30_FN_4_O_3_S ([M+H]^+^) 545.2017, found 545.2026.

Synthesis of N-((1-([2,4′-bipyridine]-3-carbonyl)-4-benzylpiperidin-4-yl)methyl)-4-cyanobenzenesulfonamide (**C-4-10**). Following General Procedure 3, **75-10** (1.00 g, 2.13 mmol) was first treated with TFA (5 mL) in CH_2_Cl_2_ (3 mL), and the intermediate obtained was reacted with **7·TFA** (0.76 g, 2.56 mmol) using EDCI (1.02 g, 5.33 mmol), HOBT (22 mg, 0.160 mmol), and DIPEA (1.65 g, 12.8 mmol) in dried THF (10 mL). Purification by column chromatography (EtOAc) gave **C-4-10**. White foam, 0.77 g (66%). ^1^H NMR (500 MHz, DMSO-*d*_6_) *δ* 8.78–8.77 (m, 1H), 8.65–8.63 (m, 2H), 8.14–8.10 (m, 2H), 7.98–7.95 (m, 3H), 7.83–7.78 (m, 1H), 7.57–7.55 (m, 3H), 7.19–7.17 (m, 3H), 7.06–6.97 (m, 2H), 3.86–3.68 (m, 1H), 3.40 (m, 0.60H), 3.28–3.12 (m, 1H), 2.99–2.98 (m, 1H), 2.80 (m, 0.49H), 2.70–2.58 (m, 2H), 2.42–2.34 (m, 1.59H), 1.47–1.44 (m, 0.58H), 1.39 (m, 0.81H), 1.32–1.23 (m, 1H), 1.17–1.13 (m, 1.55H), 0.76–0.55 (m, 1H); ^13^C NMR (151 MHz, CDCl_3_) *δ* 167.51, 167.43, 151.78, 151.65, 150.49, 149.92, 149.88, 146.31, 146.27, 144.13, 136.06, 136.03, 135.89, 135.64, 133.05, 132.98, 131.19, 130.15, 129.99, 128.31, 127.49, 127.38, 126.87, 126.81, 123.55, 123.27, 123.21, 117.34, 117.24, 116.14, 47.86, 46.30, 42.65, 42.61, 42.50, 40.94, 37.44, 37.38, 36.08, 35.94, 31.68, 31.29, 31.17, 30.91, 28.64; ESI-HR-MS: (*m*/*z*) calcd. for C_31_H_30_N_5_O_3_S ([M+H]^+^) 552.2064, found 552.2067.

Synthesis of N-((1-([2,4′-bipyridine]-3-carbonyl)-4-benzylpiperidin-4-yl)methyl)-4-methoxybenzenesulfonamide (**C-4-11**). Following General Procedure 3, **75-11** (0.84 g, 1.85 mmol) was first treated with TFA (4.2 mL) in CH_2_Cl_2_ (2.5 mL), and the intermediate obtained was reacted with **7·TFA** (0.66 g, 2.22 mmol) using EDCI (0.89 g, 4.63 mmol), HOBT (19 mg, 0.139 mmol), and DIPEA (1.43 g, 11.1 mmol) in dried THF (8.4 mL). Purification by column chromatography (EtOAc) gave **C-4-11**. White foam, 0.84 g (81%). ^1^H NMR (500 MHz, DMSO-*d*_6_) *δ* 8.77 (dd, *J* = 1.8 Hz and 4.8 Hz, 1H), 8.66–8.64 (m, 2H), 7.84–7.79 (m, 1H), 7.74–7.73 (m, 2H), 7.58–7.54 (m, 3H), 7.49–7.45 (m, 1H), 7.17–7.10 (m, 5H), 7.05–6.96 (m, 2H), 4.11–4.08 (m, 0.29H), 3.85–3.84 (m, 4H), 3.70 (m, 0.49H), 3.25–3.20 (m, 0.65H), 3.18–3.12 (m, 1H), 2.96 (s, 1H), 2.77–2.61 (m, 2H), 2.37–2.31 (m, 1.71H), 1.47–1.44 (m, 0.61H), 1.30–1.23 (m, 1H), 1.11–1.03 (m, 1.63H), 0.80–0.51 (m, 1H); ^13^C NMR (151 MHz, CDCl_3_) *δ* 167.43, 167.31, 162.78, 151.85, 151.66, 150.34, 149.91, 146.20, 146.17, 136.09, 136.06, 135.95, 135.85, 131.30, 131.14, 130.25, 130.09, 128.91, 128.19, 128.13, 126.57, 123.47, 123.22, 123.03, 114.36, 114.33, 114.27, 55.59, 46.94, 45.80, 42.64, 42.48, 41.46, 37.42, 37.35, 35.94, 35.78, 31.78, 31.53, 31.34, 31.15; ESI-HR-MS: (*m*/*z*) calcd. for C_31_H_33_N_4_O_4_S ([M+H]^+^) 557.2217, found 557.2217.

Synthesis of N-((1-([2,4′-bipyridine]-3-carbonyl)-4-benzylpiperidin-4-yl)methyl)-4-nitrobenzenesulfonamide (**C-4-12**). Following General Procedure 3, **75-12** (1.00 g, 2.04 mmol) was first treated with TFA (5 mL) in CH_2_Cl_2_ (3 mL), and the intermediate obtained was reacted with **7·TFA** (0.73 g, 2.45 mmol) using EDCI (0.98 g, 5.10 mmol), HOBT (21 mg, 0.153 mmol), and DIPEA (1.58 g, 12.2 mmol) in dried THF (10 mL). Purification by column chromatography (EtOAc) gave **C-4-12**. Pale yellow foam, 0.92 g (79%). ^1^H NMR (500 MHz, DMSO-*d*_6_) *δ* 8.77 (dd, *J* = 1.5 Hz and 5.0 Hz, 1H), 8.64–8.63 (m, 2H), 8.47–8.42 (m, 2H), 8.07–7.96 (m, 3H), 7.83–7.78 (m, 1H), 7.57–7.53 (m, 3H), 7.19–7.17 (m, 3H), 7.09–6.98 (m, 2H), 3.86–3.68 (m, 1H), 3.42 (m, 0.49H), 3.31–3.25 (m, 0.66H), 3.17–3.13 (m, 1.57H), 3.03–2.96 (m, 1H), 2.80–2.71 (m, 1H), 2.64–2.63 (m, 1H), 2.44–2.35 (m, 1.51H), 1.48–1.45 (m, 0.59H), 1.34–1.23 (m, 1H), 1.13–1.06 (m, 1.66H), 0.76–0.53 (m, 1H); ^13^C NMR (201 MHz, CDCl_3_ + CD_3_OD) *δ* 167.40, 167.28, 151.47, 151.33, 150.17, 149.66, 149.30, 149.15, 146.34, 145.82, 135.99, 135.80, 135.55, 131.07, 130.07, 129.95, 127.86, 127.82, 126.41, 126.34, 124.10, 124.07, 123.63, 123.16, 123.07, 47.13, 46.16, 42.58, 42.44, 41.90, 40.76, 37.32, 37.24, 35.76, 35.62, 31.16, 30.83, 30.61, 30.52; ESI-HR-MS: (*m*/*z*) calcd. for C_30_H_30_N_5_O_5_S ([M+H]^+^) 572.1962, found 572.1965.

Synthesis of N-((1-([2,4′-bipyridine]-3-carbonyl)-4-benzylpiperidin-4-yl)methyl)-3-chlorobenzenesulfonamide (**C-4-13**). Following General Procedure 3, **75-13** (0.80 g, 1.67 mmol) was first treated with TFA (4 mL) in CH_2_Cl_2_ (2.4 mL), and the intermediate obtained was reacted with **7·TFA** (0.60 g, 2.00 mmol) using EDCI (0.80 g, 4.18 mmol), HOBT (17 mg, 0.125 mmol), and DIPEA (1.29 g, 10.0 mmol) in dried THF (8 mL). Purification by column chromatography (EtOAc) gave **C-4-13**. White foam, 0.79 g (84%). ^1^H NMR (500 MHz, DMSO-*d*_6_) *δ* 8.78–8.77 (m, 1H), 8.66–8.63 (m, 2H), 7.85–7.73 (m, 5H), 7.68–7.63 (m, 1H), 7.58–7.54 (m, 3H), 7.19–7.17 (m, 3H), 7.07–6.97 (m, 2H), 3.87–3.69 (m, 1H), 3.39 (m, 0.48H), 3.29–3.25 (m, 0.58H), 3.18–3.12 (m, 1H), 3.03–2.95 (m, 1H), 2.82 (m, 0.47H), 2.70–2.56 (m, 2H), 2.43–2.34 (m, 1.44H), 1.48–1.46 (m, 0.58H), 1.34–1.22 (m, 1H), 1.15–1.06 (m, 1.51H), 0.82–0.51 (m, 1H); ^13^C NMR (201 MHz, CDCl_3_) *δ* 167.46, 167.34, 151.80, 151.58, 150.37, 149.90, 149.82, 146.19, 146.14, 141.46, 136.05, 135.92, 135.86, 135.63, 135.14, 132.69, 131.18, 131.15, 130.50, 130.46, 130.14, 129.99, 128.19, 126.77, 126.70, 126.65, 124.98, 124.81, 123.47, 123.44, 123.21, 123.03, 47.32, 46.01, 42.66, 42.59, 42.48, 41.24, 37.43, 37.33, 35.96, 35.80, 31.68, 31.33, 31.26, 31.01; ESI-HR-MS: (*m*/*z*) calcd. for C_30_H_30_ClN_4_O_3_S ([M+H]^+^) 561.1722, found 561.1724.

Synthesis of N-((1-([2,4′-bipyridine]-3-carbonyl)-4-benzylpiperidin-4-yl)methyl)-2-fluorobenzenesulfonamide (**C-4-14**). Following General Procedure 3, **75-14** (0.85 g, 1.84 mmol) was first treated with TFA (4.3 mL) in CH_2_Cl_2_ (2.6 mL), and the intermediate obtained was reacted with **7·TFA** (0.66 g, 2.21 mmol) using EDCI (0.88 g, 4.60 mmol), HOBT (19 mg, 0.138 mmol), and DIPEA (1.42 g, 11.0 mmol) in dried THF (8.5 mL). Purification by column chromatography (EtOAc) gave **C-4-14**. White foam, 0.83 g (83%). ^1^H NMR (500 MHz, CD_3_OD) *δ* 8.76 (dd, *J* = 1.5 Hz and 5.0 Hz, 1H), 8.67–8.58 (m, 2H), 7.85–7.83 (m, 2H), 7.67–7.65 (m, 3H), 7.56–7.54 (m, 1H), 7.37–7.30 (m, 2H), 7.22–7.17 (m, 3H), 7.09–6.98 (m, 2H), 4.59 (m, 0.24H), 3.98–3.80 (m, 1H), 3.43–3.32 (m, 1H), 3.18–3.08 (m, 1H), 3.04–3.00 (m, 0.53H), 2.90–2.83 (m, 1.49H), 2.68–2.64 (m, 1.78H), 2.52–2.41 (m, 1H), 1.55–1.52 (m, 0.56H), 1.43–1.40 (m, 1H), 1.26–1.20 (m, 1.51H), 1.04–0.62 (m, 1H); ^13^C NMR (126 MHz, CDCl_3_) *δ* 167.41, 167.27, 159.48, 157.46, 151.85, 151.65, 150.32, 149.88, 146.20, 146.13, 136.05, 136.01, 135.92, 135.82, 135.09, 135.02, 131.33, 131.24, 130.40, 130.14, 129.96, 128.26, 127.64, 127.53, 127.44, 127.34, 126.70, 124.64, 124.54, 123.44, 123.23, 122.96, 116.95, 116.78, 46.86, 45.77, 43.11, 42.58, 42.37, 41.75, 37.33, 37.27, 36.01, 35.90, 31.71, 31.54, 31.35, 31.15; ^19^F NMR (471 MHz, CD_3_OD) *δ* −111.64, −111.70; ESI-HR-MS: (*m*/*z*) calcd. for C_30_H_30_FN_4_O_3_S ([M+H]^+^) 545.2017, found 545.2019.

Synthesis of N-((1-([2,4′-bipyridine]-3-carbonyl)-4-benzylpiperidin-4-yl)methyl)-3-fluorobenzenesulfonamide (**C-4-15**). Following General Procedure 3, **75-15** (0.90 g, 1.95 mmol) was first treated with TFA (4.5 mL) in CH_2_Cl_2_ (2.7 mL), and the intermediate obtained was reacted with **7·TFA** (0.70 g, 2.34 mmol) using EDCI (0.94 g, 4.88 mmol), HOBT (20 mg, 0.146 mmol), and DIPEA (1.51 g, 11.7 mmol) in dried THF (9 mL). Purification by column chromatography (EtOAc) gave **C-4-15**. White foam, 0.91 g (86%). ^1^H NMR (500 MHz, CD_3_OD) *δ* 8.76 (dd, *J* = 1.5 Hz and 5.0 Hz, 1H), 8.66–8.58 (m, 2H), 7.84–7.81 (m, 1H), 7.68–7.53 (m, 6H), 7.39–7.38 (m, 1H), 7.20–7.15 (m, 3H), 7.05–6.93 (m, 2H), 3.98–3.75 (m, 1H), 3.50–3.34 (m, 1H), 3.21–3.08 (m, 1H), 3.03–2.87 (m, 1H), 2.81–2.74 (m, 1H), 2.63–2.52 (m, 2H), 2.49–2.38 (m, 1H), 1.54–1.50 (m, 0.53H), 1.44–1.31 (m, 1H), 1.26–1.18 (m, 1.56H), 0.99–0.61 (m, 1H); ^13^C NMR (151 MHz, CDCl_3_) *δ* 167.54, 167.44, 163.25, 163.22, 161.59, 161.55, 151.92, 151.72, 150.46, 150.01, 149.95, 146.27, 141.82, 141.78, 136.13, 136.00, 135.97, 135.74, 131.30, 131.28, 131.12, 131.08, 131.04, 130.20, 130.03, 128.31, 126.83, 126.77, 123.53, 123.29, 123.12, 122.70, 122.55, 119.99, 119.85, 114.26, 114.10, 47.47, 46.14, 42.93, 42.67, 42.54, 41.40, 37.47, 37.39, 36.07, 35.92, 31.81, 31.47, 31.43, 31.14; ^19^F NMR (471 MHz, CD_3_OD) *δ* −111.98, −111.99; ESI-HR-MS: (*m*/*z*) calcd. for C_30_H_30_FN_4_O_3_S ([M+H]^+^) 545.2017, found 545.2021.

Synthesis of N-((1-([2,4′-bipyridine]-3-carbonyl)-4-(cyclohexa-1,5-dien-1-ylmethyl)piperidin-4-yl)methyl)-2,3,4,5,6-pentafluorobenzenesulfonamide (**C-4-16**). Following General Procedure 3, **75-16** (0.80 g, 1.50 mmol) was first treated with TFA (4 mL) in CH_2_Cl_2_ (2.4 mL), and the intermediate obtained was reacted with **7·TFA** (0.54 g, 1.80 mmol) using EDCI (0.72 g, 3.75 mmol), HOBT (15 mg, 0.113 mmol), and DIPEA (1.16 g, 9.00 mmol) in dried THF (8 mL). Purification by column chromatography (EtOAc) gave **C-4-16**. White foam, 0.78 g (85%). ^1^H NMR (500 MHz, CD_3_OD) *δ* 8.76 (dd, *J* = 1.5 Hz and 5.0 Hz, 1H), 8.64–8.58 (m, 2H), 7.86–7.82 (m, 1H), 7.70–7.66 (m, 2H), 7.57–7.54 (m, 1H), 7.26–7.03 (m, 5H), 4.59 (m, 0.27H), 3.40–3.79 (m, 1H), 3.59–3.37 (m, 1H), 3.23–3.14 (m, 1H), 3.06–2.97 (m, 2H), 2.83–2.69 (m, 1.72H), 2.55–2.45 (m, 1H), 1.59–1.46 (m, 1H), 1.40–1.25 (m, 2H), 1.00–0.69 (m, 1H); ^13^C NMR (126 MHz, CDCl_3_) *δ* 167.59, 167.50, 152.02, 151.73, 150.57, 150.03, 149.98, 146.47, 146.33, 145.27, 144.84, 143.23, 142.76, 138.93, 136.89, 136.25, 136.06, 135.95, 135.75, 131.35, 131.31, 130.23, 129.93, 128.56, 127.14, 127.04, 123.66, 123.57, 123.46, 123.24, 116.38, 116.25, 116.13, 48.26, 46.34, 43.44, 42.65, 41.12, 37.49, 37.39, 36.28, 36.20, 31.88, 31.60, 31.47, 31.20; ^19^F NMR (471 MHz, CD_3_OD) *δ* −139.47–−139.53 (m), −150.30–−150.39 (m), −162.19–−162.34 (m); ESI-HR-MS: (*m*/*z*) calcd. for C_30_H_26_F_5_N_4_O_3_S ([M+H]^+^) 617.1640, found 617.1643.

Synthesis of N-((1-([2,4′-bipyridine]-3-carbonyl)-4-(cyclohexa-1,5-dien-1-ylmethyl)piperidin-4-yl)methyl)-2,3,4,5,6-pentamethylbenzenesulfonamide (**C-4-17**). Following General Procedure 3, **75-17** (0.87 g, 1.69 mmol) was first treated with TFA (4.4 mL) in CH_2_Cl_2_ (2.6 mL), and the intermediate obtained was reacted with **7·TFA** (0.61 g, 2.03 mmol) using EDCI (0.81 g, 4.23 mmol), HOBT (17 mg, 0.128 mmol), and DIPEA (1.31 g, 10.1 mmol) in dried THF (8.7 mL). Purification by column chromatography (EtOAc) gave **C-4-17**. White solid, 0.85 g (84%). m.p. 212.3–214.1 °C. ^1^H NMR (500 MHz, DMSO-*d*_6_) *δ* 8.77 (dd, *J* = 1.8 Hz and 4.8 Hz, 1H), 8.64–8.60 (m, 2H), 7.82–7.78 (m, 1H), 7.57–7.53 (m, 3H), 7.41–7.36 (m, 1H), 7.19–7.17 (m, 3H), 7.06–6.94 (m, 2H), 3.79–3.65 (m, 1H), 3.26–3.23 (m, 1H), 3.08–3.06 (m, 0.50H), 2.92–2.90 (m, 1H), 2.70–2.61 (m, 2H), 2.55–2.54 (m, 0.67H), 2.48 (m, 6.64H), 2.36–2.33 (m, 1.67H), 2.22–2.17 (m, 10H), 1.46–1.23 (m, 0.62H), 1.30–1.23 (m, 1H), 0.75–0.47 (m, 1H); ^13^C NMR (126 MHz, CDCl_3_) *δ* 167.50, 167.40, 152.18, 151.88, 150.45, 150.15, 146.31, 146.12, 139.68, 136.41, 136.27, 136.20, 136.03, 135.87, 135.65, 134.98, 134.92, 133.89, 133.82, 131.53, 131.38, 130.22, 130.03, 128.46, 126.91, 123.53, 123.48, 123.38, 123.00, 46.35, 45.36, 43.89, 42.76, 42.60, 42.44, 37.44, 36.34, 36.19, 32.11, 32.05, 31.95, 31.72, 18.91, 17.84, 17.81, 17.12, 17.08; ESI-HR-MS: (*m*/*z*) calcd. for C_30_H_26_F_5_N_4_O_3_S ([M+H]^+^) 597.2894, found 597.2899.

Synthesis of N-((1-([2,4′-bipyridine]-3-carbonyl)-4-(cyclohexa-1,5-dien-1-ylmethyl)piperidin-4-yl)methyl)-3,4-difluorobenzenesulfonamide (**C-4-18**). Following General Procedure 3, **75-18** (0.88 g, 1.83 mmol) was first treated with TFA (4.4 mL) in CH_2_Cl_2_ (2.6 mL), and the intermediate obtained was reacted with **7·TFA** (0.66 g, 2.20 mmol) using EDCI (0.87 g, 4.56 mmol), HOBT (19 mg, 0.137 mmol), and DIPEA (1.42 g, 11.0 mmol) in dried THF (8.8 mL). Purification by column chromatography (EtOAc) gave **C-4-18**. White foam, 0.79 g (77%). ^1^H NMR (500 MHz, CD_3_OD) *δ* 8.77 (dd, *J* = 1.8 Hz and 4.8 Hz, 1H), 8.67–8.59 (m, 2H), 7.86–7.77 (m, 2H), 7.70–7.65 (m, 3H), 7.58–7.48 (m, 2H), 7.22–7.19 (m, 3H), 7.06–6.95 (m, 2H), 4.59–3.94 (m, 1H), 3.78–3.53 (m, 1H), 3.38–3.34 (m, 0.60H), 3.20–3.11 (m, 1H), 3.05–2.91 (m, 1H), 2.81–2.74 (m, 1H), 2.65 (m, 0.74H), 2.55–2.40 (m, 2H), 1.56–1.52 (m, 0.51H), 1.43–1.34 (m, 1H), 1.28–1.19 (m, 1.62H), 0.96–0.64 (m, 1H); ^13^C NMR (126 MHz, CDCl_3_) *δ* 167.65, 167.59, 154.16, 154.06, 152.12, 152.01, 151.84, 151.25, 150.61, 150.11, 149.23, 146.38, 136.65, 136.22, 136.03, 135.80, 131.36, 130.21, 130.02, 128.51, 127.09, 127.00, 124.21, 124.00, 123.63, 123.39, 123.30, 118.47, 118.32, 116.95, 116.79, 47.95, 46.37, 43.26, 42.72, 42.62, 41.32, 37.54, 37.47, 36.20, 36.07, 31.95, 31.65, 31.48, 31.19; ^19^F NMR (471 MHz, CD_3_OD) *δ* −133.31–−133.43 (m), −136.87–−136.96 (m); ESI-HR-MS: (*m*/*z*) calcd. for C_30_H_29_F_2_N_4_O_3_S ([M+H]^+^) 563.1923, found 563.1928.

Synthesis of N-((1-([2,4′-bipyridine]-3-carbonyl)-4-(cyclohexa-1,5-dien-1-ylmethyl)piperidin-4-yl)methyl)-3,4,5-trifluorobenzenesulfonamide (**C-4-19**). Following General Procedure 3, **75-19** (0.80 g, 1.60 mmol) was first treated with TFA (4 mL) in CH_2_Cl_2_ (2.4 mL), and the intermediate obtained was reacted with **7·TFA** (0.57 g, 1.92 mmol) using EDCI (0.77 g, 4.00 mmol), HOBT (16 mg, 0.120 mmol), and DIPEA (1.24 g, 9.60 mmol) in dried THF (8 mL). Purification by column chromatography (EtOAc) gave **C-4-19**. White foam, 0.67 g (72%). ^1^H NMR (500 MHz, CD_3_OD) *δ* 8.77 (dd, *J* = 1.8 Hz and 4.8 Hz, 1H), 8.67–8.58 (m, 2H), 7.86–7.82 (m, 1H), 7.68–7.63 (m, 4H), 7.58–7.55 (m, 1H), 7.23–7.20 (m, 3H), 7.08–7.00 (m, 2H), 4.59 (s, 1H), 4.00–3.95 (m, 0.53H), 3.77–3.57 (m, 1H), 3.40–3.35 (m, 1H), 3.22–3.02 (m, 1.52H), 2.93–2.76 (m, 1.62H), 2.66 (m, 0.69H), 2.59–2.41 (m, 2H), 1.55–1.21 (m, 3H), 0.91–0.66 (m, 1H); ^13^C NMR (151 MHz, CDCl_3_ + CD_3_OD) *δ* 167.46, 167.36, 151.69, 151.61, 151.40, 150.23, 149.96, 149.35, 149.22, 146.40, 143.04, 141.31, 136.33, 136.03, 135.83, 135.60, 131.14, 130.12, 130.02, 127.93, 126.49, 123.67, 123.23, 123.12, 111.85, 111.73, 47.26, 46.24, 42.63, 41.95, 40.83, 37.34, 35.79, 35.68, 31.24, 30.94, 30.71, 30.62; ^19^F NMR (471 MHz, CD_3_OD) *δ* −132.96–−132.02 (m), −156.32–−156.48 (m); ESI-HR-MS: (*m*/*z*) calcd. for C_30_H_28_F_3_N_4_O_3_S ([M+H]^+^) 581.1829, found 581.1835.

Synthesis of N-((1-([2,4′-bipyridine]-3-carbonyl)-4-(cyclohexa-1,5-dien-1-ylmethyl)piperidin-4-yl)methyl)-2,5-difluorobenzenesulfonamide (**C-4-20**). Following General Procedure 3, **75-20** (0.80 g, 1.66 mmol) was first treated with TFA (4 mL) in CH_2_Cl_2_ (2.4 mL), and the intermediate obtained was reacted with **7·TFA** (0.59 g, 1.99 mmol) using EDCI (0.80 g, 4.15 mmol), HOBT (17 mg, 0.125 mmol), and DIPEA (1.29 g, 9.96 mmol) in dried THF (8 mL). Purification by column chromatography (EtOAc) gave **C-4-20**. White foam, 0.71 g (76%). ^1^H NMR (500 MHz, DMSO-*d*_6_) *δ* 8.77 (dd, *J* = 1.8 Hz and 4.8 Hz, 1H), 8.64–8.63 (m, 2H), 8.16–8.10 (m, 1H), 7.83–7.78 (m, 1H), 7.59–7.53 (m, 6H), 7.23–7.17 (m, 3H), 7.11–7.01 (m, 2H), 3.85–3.69 (m, 1H), 3.33–3.27 (m, 1H), 3.18–3.14 (m, 0.76H), 3.04–2.97 (m, 1H), 2.84–2.80 (m, 1H), 2.74–2.66 (m, 1.53H), 2.56–2.53 (m, 1.46H), 2.40–2.37 (m, 0.61H), 1.51–1.47 (m, 0.60H), 1.35–1.28 (m, 1H), 1.16–1.09 (m, 1.60H), 0.84–0.55 (m, 1H); ^13^C NMR (126 MHz, CDCl_3_) *δ* 167.57, 167.44, 158.84, 156.86, 155.54, 153.56, 152.02, 151.78, 150.49, 150.10, 150.03, 146.33, 146.22, 136.18, 136.03, 135.84, 131.41, 131.33, 130.20, 129.97, 129.04, 126.96, 126.91, 123.57, 123.52, 123.36, 123.09, 121.76, 121.70, 121.57, 121.50, 118.57, 118.51, 118.38, 118.31, 117.36, 117.16, 116.95, 47.40, 46.05, 43.40, 42.66, 42.50, 41.68, 37.43, 37.36, 36.18, 36.08, 31.87, 31.66, 31.56, 31.27; ^19^F NMR (471 MHz, DMSO-*d*_6_) δ −115.57–−115.67 (m), −116.19–−116.23 (m); ESI-HR-MS: (*m*/*z*) calcd. for C_30_H_29_F_2_N_4_O_3_S ([M+H]^+^) 563.1923, found 563.1931.

Synthesis of N-((1-([2,4′-bipyridine]-3-carbonyl)-4-(cyclohexa-1,5-dien-1-ylmethyl)piperidin-4-yl)methyl)-2,4,5-trifluorobenzenesulfonamide (**C-4-21**). Following General Procedure 3, **75-21** (0.91 g, 1.83 mmol) was first treated with TFA (4.6 mL) in CH_2_Cl_2_ (2.7 mL), and the intermediate obtained was reacted with **7·TFA** (0.66 g, 2.20 mmol) using EDCI (0.87 g, 4.56 mmol), HOBT (19 mg, 0.137 mmol), and DIPEA (1.42 g, 11.0 mmol) in dried THF (9.1 mL). Purification by column chromatography (EtOAc) gave **C-4-21**. White foam, 0.84 g (79%). ^1^H NMR (500 MHz, DMSO-*d*_6_) *δ* 8.77 (dd, *J* = 1.8 Hz and 4.8 Hz, 1H), 8.63 (dd, *J* = 2.0 Hz and 4.5 Hz, 2H), 8.19–8.12 (m, 1H), 7.90–7.78 (m, 3H), 7.57–7.53 (m, 3H), 7.24–7.18 (m, 3H), 7.12–7.01 (m, 2H), 3.85–3.44 (m, 2H), 3.31–3.27 (m, 0.59H), 3.17–3.14 (m, 0.65H), 3.04–2.99 (m, 1H), 2.82–2.78 (m, 1H), 2.73–2.65 (m, 1.49H), 2.55–2.52 (m, 1H), 2.40–2.37 (m, 0.60H), 1.49–1.46 (m, 0.60H), 1.35–1.24 (m, 1H), 1.16–1.08 (m, 1.61H), 0.77–0.58 (m, 1H); ^13^C NMR (126 MHz, CDCl_3_) *δ* 167.61, 167.49, 155.33, 155.25, 154.24, 153.31, 153.24, 152.16, 151.98, 151.80, 150.55, 150.04, 147.36, 146.39, 145.36, 136.20, 136.11, 136.02, 135.83, 131.40, 130.19, 129.95, 128.55, 128.52, 127.07, 126.99, 124.47, 124.33, 123.60, 123.40, 123.20, 119.13, 118.91, 117.73, 107.65, 107.48, 107.44, 107.26, 47.66, 46.19, 43.42, 42.66, 42.51, 41.51, 37.46, 37.38, 36.20, 36.13, 31.91, 31.61, 31.25; ^19^F NMR (471 MHz, DMSO-*d*_6_) *δ* −110.07–−110.16 (m), −126.25–−126.39 (m), −140.39–−140.47 (m); ESI-HR-MS: (*m*/*z*) calcd. for C_30_H_28_F_3_N_4_O_3_S ([M+H]^+^) 581.1829, found 581.1837.

Synthesis of N-((1-([2,4′-bipyridine]-3-carbonyl)-4-(cyclohexa-1,5-dien-1-ylmethyl)piperidin-4-yl)methyl)-3-chloro-4-fluorobenzenesulfonamide (**C-4-22**). Following General Procedure 3, **75-22** (0.90 g, 1.81 mmol) was first treated with TFA (4.5 mL) in CH_2_Cl_2_ (2.7 mL), and the intermediate obtained was reacted with **7·TFA** (0.65 g, 2.17 mmol) using EDCI (0.87 g, 4.53 mmol), HOBT (18 mg, 0.136 mmol), and DIPEA (1.41 g, 10.9 mmol) in dried THF (9 mL). Purification by column chromatography (EtOAc) gave **C-4-22**. White foam, 0.54 g (51%). ^1^H NMR (500 MHz, CD_3_OD) *δ* 8.76 (dd, *J* = 1.8 Hz and 4.8 Hz, 1H), 8.67–8.59 (m, 2H), 7.99–7.96 (m, 1H), 7.85–7.78 (m, 2H), 7.67–7.66 (m, 2H), 7.56–7.54 (m, 1H), 7.48–7.42 (m, 1H), 7.21–7.18 (m, 3H), 7.05–6.94 (m, 2H), 3.98–3.76 (m, 1H), 3.55–3.33 (m, 1H), 3.22–3.10 (m, 1H), 3.04–2.89 (m, 1H), 2.80–2.73 (m, 1H), 2.64–2.63 (m, 1H), 2.57–2.39 (m, 2H), 1.54–1.51 (m, 0.52H), 1.43–1.34 (m, 1H), 1.26–1.19 (m, 1.52H), 0.96–0.62 (m, 1H); ^13^C NMR (126 MHz, CDCl_3_) *δ* 167.63, 161.64, 159.59, 151.86, 150.63, 150.11, 146.44, 136.90, 136.23, 136.05, 135.83, 131.40, 130.21, 130.01, 129.87, 128.57, 127.60, 127.39, 127.11, 123.67, 123.42, 122.66, 122.52, 117.68, 117.51, 48.05, 46.39, 43.42, 42.71, 41.37, 37.50, 36.24, 36.11, 32.00, 31.74, 31.48, 31.21; ^19^F NMR (471 MHz, CD_3_OD) *δ* −110.77, −110.81; ESI-HR-MS: (*m*/*z*) calcd. for C_30_H_29_ClFN_4_O_3_S ([M+H]^+^) 579.1627, found 579.1637.

Synthesis of N-((1-([2,4′-bipyridine]-3-carbonyl)-4-(cyclohexa-1,5-dien-1-ylmethyl)piperidin-4-yl)methyl)-2,3,4-trifluorobenzenesulfonamide (**C-4-23**). Following General Procedure 3, **75-23** (0.85 g, 1.70 mmol) was first treated with TFA (4.3 mL) in CH_2_Cl_2_ (2.6 mL), and the intermediate obtained was reacted with **7·TFA** (0.61 g, 2.04 mmol) using EDCI (0.81 g, 4.25 mmol), HOBT (17 mg, 0.128 mmol), and DIPEA (1.32 g, 10.2 mmol) in dried THF (8.5 mL). Purification by column chromatography (EtOAc) gave **C-4-23**. White foam, 0.85 g (86%). ^1^H NMR (500 MHz, CD_3_OD) *δ* 8.78–8.76 (m, 1H), 8.68–8.58 (m, 2H), 7.87–7.84 (m, 1H), 7.68–7.66 (m, 3H), 7.58–7.54 (m, 1H), 7.32–7.19 (m, 4H), 7.10–7.01 (m, 2H), 3.98–3.80 (m, 1H), 3.50–3.35 (m, 1.44H), 3.20–3.05 (m, 1.59H), 2.92–2.85 (m, 1.51H), 2.68–2.65 (m, 1.60H), 2.54–2.44 (m, 1H), 1.55–1.44 (m, 1H), 1.37–1.34 (m, 0.51H), 1.30–1.24 (m, 1.61H), 0.99–0.69 (m, 1H); ^13^C NMR (151 MHz, CDCl_3_) *δ* 167.71, 167.61, 155.21, 153.50, 152.06, 151.93, 150.66, 150.16, 149.25, 147.53, 146.49, 141.25, 139.55, 136.28, 136.07, 135.89, 131.49, 130.14, 129.91, 128.75, 127.30, 127.20, 125.63, 125.39, 124.83, 124.40, 123.70, 123.51, 123.36, 112.82, 47.73, 46.31, 43.95, 42.72, 42.54, 41.87, 37.47, 36.36, 36.29, 32.04, 31.89, 31.73, 31.42, 29.81; ^19^F NMR (471 MHz, CD_3_OD) *δ* −128.46–−128.60 (m), −132.66–−160.75 (m), −160.30–−160.47 (m); ESI-HR-MS: (*m*/*z*) calcd. for C_30_H_28_F_3_N_4_O_3_S ([M+H]^+^) 581.1829, found 581.1837.

Synthesis of N-((1-([2,4′-bipyridine]-3-carbonyl)-4-(cyclohexa-1,5-dien-1-ylmethyl)piperidin-4-yl)methyl)-3,5-bis(trifluoromethyl)benzenesulfonamide (**C-4-24**). Following General Procedure 3, **75-24** (0.90 g, 1.55 mmol) was first treated with TFA (4.5 mL) in CH_2_Cl_2_ (2.7 mL), and the intermediate obtained was reacted with **7·TFA** (0.55 g, 1.86 mmol) using EDCI (0.74 g, 3.88 mmol), HOBT (16 mg, 0.116 mmol), and DIPEA (1.20 g, 9.30 mmol) in dried THF (9 mL). Purification by column chromatography (EtOAc) gave **C-4-24**. White foam, 0.78 g (76%). ^1^H NMR (500 MHz, CD_3_OD) *δ* 8.77 (dd, *J* = 1.8 Hz and 4.8 Hz, 1H), 8.66–8.59 (m, 2H), 8.39–8.38 (m, 2H), 8.30 (s, 1H), 7.86–7.83 (m, 1H), 7.68–7.67 (m, 2H), 7.57–7.55 (m, 1H), 7.19–7.16 (m, 3H), 7.04–6.93 (m, 2H), 3.95–3.92 (m, 0.52H), 3.76–3.64 (m, 1H), 3.45–3.40 (m, 1H), 3.28–3.12 (m, 1H), 3.05–2.98 (m, 1H), 2.85–2.78 (m, 1H), 2.67–2.58 (m, 1.66H), 2.52–2.41 (m, 1H), 1.56–1.53 (m, 0.54H), 1.44–1.37 (m, 1H), 1.30–1.20 (m, 1.58H), 0.97–0.66 (m, 1H); ^13^C NMR (151 MHz, CDCl_3_) *δ* 167.72, 152.02, 151.78, 150.66, 150.09, 150.04, 146.39, 143.05, 136.25, 136.14, 135.88, 135.63, 133.34, 133.11, 133.08, 132.88, 132.85, 132.65, 131.25, 131.21, 130.16, 129.97, 128.50, 127.32, 127.15, 127.07, 126.22, 125.19, 123.66, 123.59, 123.45, 123.40, 123.31, 121.64, 121.56, 119.75, 48.47, 46.65, 42.94, 42.72, 40.95, 37.60, 37.49, 36.19, 36.09, 31.82, 31.49, 31.23, 31.00; ^19^F NMR (471 MHz, CD_3_OD) *δ* −64.29, −64.33; ESI-HR-MS: (*m*/*z*) calcd. for C_32_H_29_F_6_N_4_O_3_S ([M+H]^+^) 663.1859, found 663.1865.

Synthesis of N-((1-([2,4′-bipyridine]-3-carbonyl)-4-(cyclohexa-1,5-dien-1-ylmethyl)piperidin-4-yl)methyl)-2-chloro-4-fluorobenzenesulfonamide (**C-4-25**). Following General Procedure 3, **75-25** (0.90 g, 1.81 mmol) was first treated with TFA (4.5 mL) in CH_2_Cl_2_ (2.7 mL), and the intermediate obtained was reacted with **7·TFA** (0.65 g, 2.17 mmol) using EDCI (0.87 g, 4.53 mmol), HOBT (18 mg, 0.136 mmol), and DIPEA (1.41 g, 10.9 mmol) in dried THF (9 mL). Purification by column chromatography (EtOAc) gave **C-4-25**. White foam, 0.81 g (77%). ^1^H NMR (500 MHz, CD_3_OD) *δ* 8.77–8.75 (m, 1H), 8.67–8.59 (m, 2H), 8.07–8.05 (m, 1H), 7.85–7.83 (m, 1H), 7.67–7.66 (m, 2H), 7.57–7.54 (m, 1H), 7.49–7.46 (m, 1H), 7.23–7.19 (m, 4H), 7.10–6.99 (m, 2H), 3.96–3.80 (m, 1H), 3.38–3.32 (m, 1H), 3.17–3.14 (m, 0.49H), 3.10–2.98 (m, 1H), 2.84–2.77 (m, 1.54H), 2.65 (s, 1H), 2.55–2.40 (m, 2H), 1.54–1.51 (m, 0.54H), 1.43–1.33 (m, 1H), 1.25–1.20 (m, 1.59H), 0.98–0.62 (m, 1H); ^13^C NMR (126 MHz, CDCl_3_) *δ* 167.66, 167.50, 165.71, 163.73, 163.65, 152.04, 151.89, 150.58, 150.16, 146.37, 136.23, 136.16, 135.96, 133.86, 133.78, 133.36, 133.28, 132.93, 132.81, 131.48, 131.40, 130.17, 130.01, 128.65, 127.10, 127.05, 123.61, 123.44, 123.19, 119.41, 119.21, 115.08, 114.91, 114.83, 114.66, 47.04, 46.00, 43.82, 42.68, 42.43, 42.14, 37.39, 36.32, 36.27, 32.04, 31.97, 31.87, 31.60; ^19^F NMR (471 MHz, CD_3_OD) *δ* −106.24, −106.34; ESI-HR-MS: (*m*/*z*) calcd. for C_30_H_29_ClFN_4_O_3_S ([M+H]^+^) 579.1627, found 579.1633.

Synthesis of N-((1-([2,4′-bipyridine]-3-carbonyl)-4-(cyclohexa-1,5-dien-1-ylmethyl)piperidin-4-yl)methyl)-3-cyano-4-fluorobenzenesulfonamide (**C-4-26**). Following General Procedure 3, **75-26** (0.64 g, 1.31 mmol) was first treated with TFA (3.2 mL) in CH_2_Cl_2_ (1.9 mL), and the intermediate obtained was reacted with **7·TFA** (0.47 g, 1.57 mmol) using EDCI (0.63 g, 3.28 mmol), HOBT (13 mg, 0.098 mmol), and DIPEA (1.02 g, 7.86 mmol) in dried THF (6.4 mL). Purification by column chromatography (EtOAc) gave **C-4-26**. White foam, 0.55 g (74%). ^1^H NMR (500 MHz, CD_3_OD) *δ* 8.77–8.76 (m, 1H), 8.67–8.58 (m, 2H), 8.26–8.24 (m, 1H), 8.18–8.14 (m, 1H), 7.86–7.82 (m, 1H), 7.68–7.66 (m, 2H), 7.62–7.55 (m, 2H), 7.22–7.19 (m, 3H), 7.07–6.96 (m, 2H), 3.98–3.74 (m, 1H), 3.58–3.35 (m, 1H), 3.23–3.12 (m, 1H), 3.06–2.92 (m, 1H), 2.83–2.76 (m, 1H), 2.65 (s, 1H), 2.58–2.40 (m, 2H), 1.54–1.52 (m, 0.53H), 1.46–1.45 (m, 1H), 1.34–1.18 (m, 1.60H), 0.92–0.65 (m, 1H); ^13^C NMR (126 MHz, CDCl_3_) *δ* 167.66, 166.03, 163.92, 151.88, 151.86, 150.66, 150.12, 146.51, 146.35, 137.67, 136.20, 136.00, 135.76, 134.11, 134.04, 133.85, 133.77, 133.02, 132.89, 131.35, 131.28, 130.23, 130.04, 128.55, 128.47, 127.17, 127.06, 123.67, 123.38, 123.34, 117.88, 117.71, 112.53, 112.42, 102.93, 102.81, 52.44, 48.44, 46.65, 43.06, 42.71, 42.65, 40.86, 37.59, 37.50, 36.23, 36.14, 31.88, 31.58, 31.28, 31.05, 28.82; ^19^F NMR (471 MHz, CDCl_3_) δ −99.05, −99.22, −107.07; ESI-HR-MS: (*m*/*z*) calcd. for C_31_H_29_FN_5_O_3_S ([M+H]^+^) 570.1970, found 570.1976.

Synthesis of N-((1-([2,4′-bipyridine]-3-carbonyl)-4-(cyclohexa-1,5-dien-1-ylmethyl)piperidin-4-yl)methyl)-4-(*tert*-butyl)benzenesulfonamide (**C-4-27**). Following General Procedure 3, **75-27** (0.90 g, 1.80 mmol) was first treated with TFA (4.5 mL) in CH_2_Cl_2_ (2.7 mL), and the intermediate obtained was reacted with **7·TFA** (0.64 g, 2.16 mmol) using EDCI (0.86 g, 4.50 mmol), HOBT (18 mg, 0.135 mmol), and DIPEA (1.40 g, 10.8 mmol) in dried THF (9 mL). Purification by column chromatography (EtOAc) gave **C-4-27**. White foam, 0.75 g (72%). ^1^H NMR (500 MHz, CD_3_OD) *δ* 8.76 (dd, *J* = 1.8 Hz and 4.8 Hz, 1H), 8.67–8.59 (m, 2H), 7.84–7.81 (m, 1H), 7.78–7.74 (m, 2H), 7.67–7.66 (m, 2H), 7.63–7.59 (m, 2H), 7.56–7.53 (m, 1H), 7.18–7.16 (m, 3H), 7.04–6.93 (m, 2H), 3.99–3.78 (m, 1H), 3.43–3.35 (m, 0.67H), 3.28–3.26 (m, 0.46H), 3.20–3.05 (m, 1H), 3.02–2.87 (m, 1H), 2.78–2.71 (m, 1H), 2.63 (s, 1H), 2.58–2.51 (m, 1H), 2.48–2.37 (m, 1H), 1.52–1.41 (m, 1.55H), 1.36–1.34 (m, 9H), 1.24–1.18 (m, 1.62H), 1.01–0.58 (m, 1H); ^13^C NMR (126 MHz, CDCl_3_) *δ* 167.53, 167.42, 156.60, 152.08, 151.83, 150.45, 150.11, 150.05, 146.30, 136.56, 136.17, 136.02, 135.91, 131.46, 130.29, 130.10, 128.32, 126.82, 126.76, 126.28, 126.22, 123.53, 123.33, 123.12, 47.11, 45.85, 43.19, 42.72, 42.61, 41.72, 37.51, 37.41, 36.17, 35.98, 35.18, 31.93, 31.69, 31.60, 31.31, 31.10; ESI-HR-MS: (*m*/*z*) calcd. for C_34_H_39_N_4_O_3_S ([M+H]^+^) 583.2737, found 583.2740.

Synthesis of N-((1-([2,4′-bipyridine]-3-carbonyl)-4-(cyclohexa-1,5-dien-1-ylmethyl)piperidin-4-yl)methyl)-4-(trifluoromethyl)benzenesulfonamide (**C-4-28**). Following General Procedure 3, **75-28** (0.80 g, 1.56 mmol) was first treated with TFA (4 mL) in CH_2_Cl_2_ (2.4 mL), and the intermediate obtained was reacted with **7·TFA** (0.56 g, 1.87 mmol) using EDCI (0.75 g, 3.90 mmol), HOBT (16 mg, 0.117 mmol), and DIPEA (1.21 g, 9.36 mmol) in dried THF (8 mL). Purification by column chromatography (EtOAc) gave **C-4-28**. White foam, 0.67 g (72%). ^1^H NMR (500 MHz, CD_3_OD) *δ* 8.77–8.75 (m, 1H), 8.67–8.58 (m, 2H), 8.04–8.00 (m, 2H), 7.92–7.82 (m, 3H), 7.68–7.66 (m, 2H), 7.56–7.54 (m, 1H), 7.19–7.16 (m, 3H), 7.04–6.93 (m, 2H), 3.98–3.77 (m, 1H), 3.50–3.35 (m, 1H), 3.22–3.09 (m, 1H), 3.04–2.91 (m, 1H), 2.93–2.76 (m, 1H), 2.64–2.61 (m, 1H), 2.58–2.53 (m, 1H), 2.50–2.39 (m, 1H), 1.54–1.51 (m, 0.53H), 1.45–1.32 (m, 1H), 1.28–1.19 (m, 1.67H), 0.99–0.62 (m, 1H); ^13^C NMR (126 MHz, CDCl_3_) *δ* 167.63, 167.54, 151.99, 151.80, 150.57, 150.06, 146.37, 143.39, 136.18, 136.11, 135.99, 135.74, 134.81, 134.55, 134.28, 131.34, 130.18, 130.00, 128.44, 127.50, 127.39, 127.00, 126.92, 126.43, 124.27, 123.60, 123.37, 123.28, 122.10, 47.81, 46.26, 43.12, 42.69, 42.59, 41.34, 37.52, 37.45, 36.20, 36.04, 31.89, 31.58, 31.42, 31.13; ^19^F NMR (471 MHz, DMSO-*d*_6_) δ −65.23, −65.26; ESI-HR-MS: (*m*/*z*) calcd. for C_31_H_30_F_3_N_4_O_3_S ([M+H]^+^) 595.1985, found 595.1986.

Synthesis of N-((1-([2,4′-bipyridine]-3-carbonyl)-4-(cyclohexa-1,5-dien-1-ylmethyl)piperidin-4-yl)methyl)-4-fluoro-3-(trifluoromethyl)benzenesulfonamide (**C-4-29**). Following General Procedure 3, **75-29** (0.80 g, 1.51 mmol) was first treated with TFA (4 mL) in CH_2_Cl_2_ (2.4 mL), and the intermediate obtained was reacted with **7·TFA** (0.54 g, 1.81 mmol) using EDCI (0.72 g, 3.78 mmol), HOBT (15 mg, 0.113 mmol), and DIPEA (1.17 g, 9.06 mmol) in dried THF (8 mL). Purification by column chromatography (EtOAc) gave **C-4-29**. White foam, 0.76 g (82%). ^1^H NMR (500 MHz, CD_3_OD) *δ* 8.78–8.76 (m, 1H), 8.67–8.59 (m, 2H), 8.17–8.14 (m, 2H), 7.86–7.83 (m, 1H), 7.68–7.67 (m, 2H), 7.59–7.55 (m, 2H), 7.20–7.18 (m, 3H), 7.05–6.94 (m, 2H), 3.97–3.77 (m, 1H), 3.58–3.35 (m, 1H), 3.22–3.11 (m, 1H), 3.05–2.92 (m, 1H), 2.81–2.74 (m, 1H), 2.66 (s, 1H), 2.59–2.54 (m, 1H), 2.51–2.41 (m, 1H), 1.55–1.52 (m, 0.53H), 1.46–1.33 (m, 1H), 1.28–1.21 (m, 1.56H), 0.96–0.65 (m, 1H); ^13^C NMR (126 MHz, CDCl_3_) *δ* 167.65, 163.01, 160.91, 151.99, 151.84, 150.64, 150.12, 146.48, 146.39, 136.72, 136.69, 136.23, 136.17, 135.98, 135.76, 133.41, 133.34, 133.10, 133.02, 131.34, 130.18, 129.97, 128.54, 127.16, 127.06, 126.74, 123.65, 123.40, 122.76, 120.59, 119.82, 119.72, 119.55, 119.44, 118.40, 118.21, 48.17, 46.47, 43.25, 42.70, 41.20, 37.56, 37.47, 36.22, 36.10, 31.95, 31.66, 31.41, 31.13; ^19^F NMR (471 MHz, CD_3_OD) δ −63.08–−63.15 (m), −110.16–−110.26; ESI-HR-MS: (*m*/*z*) calcd. for C_31_H_29_F_4_N_4_O_3_S ([M+H]^+^) 613.1891, found 613.1885.

Synthesis of N-((1-([2,4′-bipyridine]-3-carbonyl)-4-(cyclohexa-1,5-dien-1-ylmethyl)piperidin-4-yl)methyl)-4-(trifluoromethoxy)benzenesulfonamide (**C-4-30**). Following General Procedure 3, **75-30** (0.90 g, 1.70 mmol) was first treated with TFA (4.5 mL) in CH_2_Cl_2_ (2.7 mL), and the intermediate obtained was reacted with **7·TFA** (0.61 g, 2.04 mmol) using EDCI (0.81 g, 4.25 mmol), HOBT (17 mg, 0.128 mmol), and DIPEA (1.32 g, 10.2 mmol) in dried THF (9 mL). Purification by column chromatography (EtOAc) gave **C-4-30**. White foam, 0.73 g (70%). ^1^H NMR (500 MHz, CD_3_OD) *δ* 8.77–8.76 (m, 1H), 8.68–8.59 (m, 2H), 7.97–7.92 (m, 2H), 7.86–7.82 (m, 1H), 7.68–7.67 (m, 2H), 7.57–7.54 (m, 1H), 7.51–7.47 (m, 2H), 7.20–7.17 (m, 3H), 7.05–6.94 (m, 2H), 3.99–3.78 (m, 1H), 3.49–3.34 (m, 1H), 3.22–3.09 (m, 1H), 3.03–2.88 (m, 1H), 2.81–2.75 (m, 1H), 2.64 (s, 1H), 2.60–2.52 (m, 1H), 2.50–2.40 (m, 1H), 1.54–1.42 (m, 1H), 1.36–1.33 (m, 0.52H), 1.27–1.20 (m, 1.49H), 1.00–0.62 (m, 1H); ^13^C NMR (126 MHz, CDCl_3_) *δ* 167.53, 152.15, 152.14, 152.02, 151.84, 150.56, 150.10, 146.37, 138.13, 136.19, 136.11, 136.04, 135.79, 131.38, 130.21, 130.01, 129.16, 129.04, 128.43, 126.98, 126.90, 123.58, 123.37, 123.26, 121.27, 121.18, 121.11, 47.68, 46.18, 43.19, 42.70, 42.59, 41.43, 37.52, 37.44, 36.19, 36.04, 31.94, 31.62, 31.52, 31.19; ^19^F NMR (471 MHz, CD_3_OD) *δ* −59.27, −59.30; ESI-HR-MS: (*m*/*z*) calcd. for C_31_H_30_F_3_N_4_O_4_S ([M+H]^+^) 611.1934, found 611.1926.

Synthesis of N-((1-([2,4′-bipyridine]-3-carbonyl)-4-(cyclohexa-1,5-dien-1-ylmethyl)piperidin-4-yl)methyl)-4-fluoro-2-(trifluoromethyl)benzenesulfonamide (**C-4-31**). Following General Procedure 3, **75-31** (0.90 g, 1.70 mmol) was first treated with TFA (4.5 mL) in CH_2_Cl_2_ (2.7 mL), and the intermediate obtained was reacted with **7·TFA** (0.61 g, 2.04 mmol) using EDCI (0.81 g, 4.25 mmol), HOBT (17 mg, 0.128 mmol), and DIPEA (1.32 g, 10.2 mmol) in dried THF (9 mL). Purification by column chromatography (EtOAc) gave **C-4-31**. White solid, 0.71 g (68%), m.p. 102.4–103.8 °C. ^1^H NMR (500 MHz, CD_3_OD) *δ* 8.77 (dd, *J* = 1.8 Hz and 4.8 Hz, 1H), 8.67–8.58 (m, 2H), 8.18–8.11 (m, 1H), 7.85–7.82 (m, 1H), 7.75–7.73 (m, 1H), 7.67–7.66 (m, 2H), 7.62–7.55 (m, 2H), 7.24–7.18 (m, 3H), 7.09–6.99 (m, 2H), 3.96–3.78 (m, 1H), 3.49–3.35 (m, 1H), 3.20–3.11 (m, 1H), 3.05–2.86 (m, 2H), 2.67–2.65 (m, 2H), 2.54–2.44 (m, 1H), 1.57–1.54 (m, 0.56H), 1.45–1.34 (m, 1H), 1.30–1.25 (m, 1.63H), 0.99–0.67 (m, 1H); ^13^C NMR (126 MHz, CDCl_3_) *δ* 167.62, 167.51, 165.30, 165.22, 163.26, 163.17, 151.95, 151.87, 150.56, 150.14, 146.34, 136.20, 136.16, 136.09, 135.86, 135.22, 135.15, 134.63, 134.52, 134.46, 134.22, 131.40, 130.00, 129.84, 128.61, 127.08, 127.01, 123.60, 123.38, 123.27, 119.43, 119.27, 119.15, 118.98, 116.98, 116.93, 116.78, 116.72, 47.61, 46.39, 43.55, 42.67, 42.46, 41.51, 37.43, 37.38, 36.36, 36.30, 31.91, 31.86, 31.80, 31.42; ^19^F NMR (471 MHz, CD_3_OD) *δ* −59.22, −106.31, −106.37; ESI-HR-MS: (*m*/*z*) calcd. for C_31_H_29_F_4_N_4_O_3_S ([M+H]^+^) 613.1891, found 613.1882.

Synthesis of N-((1-([2,4′-bipyridine]-3-carbonyl)-4-(cyclohexa-1,5-dien-1-ylmethyl)piperidin-4-yl)methyl)naphthalene-2-sulfonamide (**C-4-32**). Following General Procedure 3, **75-32** (0.90 g, 1.82 mmol) was first treated with TFA (4.5 mL) in CH_2_Cl_2_ (2.7 mL), and the intermediate obtained was reacted with **7·TFA** (0.65 g, 2.18 mmol) using EDCI (0.87 g, 4.55 mmol), HOBT (19 mg, 0.137 mmol), and DIPEA (1.41 g, 10.9 mmol) in dried THF (9 mL). Purification by column chromatography (EtOAc) gave **C-4-32**. White foam, 0.66 g (63%). ^1^H NMR (500 MHz, CD_3_OD) *δ* 8.74–8.73 (m, 1H), 8.63–8.56 (m, 2H), 8.40–8.39 (m, 1H), 8.05–7.95 (m, 3H), 7.87–7.76 (m, 2H), 7.66–7.61 (m, 4H), 7.54–7.49 (m, 1H), 7.11–7.05 (m, 3H), 6.99–6.87 (m, 2H), 3.96–3.75 (m, 1H), 3.45–3.35 (m, 0.68H), 3.29–3.27 (m, 0.44H), 3.17–3.00 (m, 1H), 2.95–2.86 (m, 1H), 2.81–2.75 (m, 1H), 2.62–2.54 (m, 2H), 2.47–2.36 (m, 1H), 1.51–1.49 (m, 0.62H), 1.40–1.33 (m, 1H), 1.22–1.15 (m, 1.75H), 1.00–0.55 (m, 1H); ^13^C NMR (201 MHz, CDCl_3_) *δ* 167.45, 167.29, 151.90, 151.64, 150.31, 149.99, 149.87, 146.17, 146.13, 136.36, 136.03, 135.98, 135.93, 135.73, 134.64, 132.07, 131.96, 131.27, 130.17, 130.02, 129.58, 129.22, 129.06, 128.85, 128.31, 128.12, 127.83, 127.62, 126.61, 126.55, 123.43, 123.20, 123.01, 121.90, 47.10, 45.91, 42.80, 42.60, 42.47, 41.48, 37.42, 37.33, 36.00, 35.82, 31.74, 31.43, 31.35, 31.09; ESI-HR-MS: (*m*/*z*) calcd. for C_34_H_33_N_4_O_3_S ([M+H]^+^) 577.2268, found 577.2266.

Synthesis of N-((1-([2,4′-bipyridine]-3-carbonyl)-4-(cyclohexa-1,5-dien-1-ylmethyl)piperidin-4-yl)methyl)naphthalene-1-sulfonamide (**C-4-33**). Following General Procedure 3, **75-33** (0.82 g, 1.66 mmol) was first treated with TFA (4.1 mL) in CH_2_Cl_2_ (2.5 mL), and the intermediate obtained was reacted with **7·TFA** (0.59 g, 1.99 mmol) using EDCI (0.80 g, 4.15 mmol), HOBT (17 mg, 0.127 mmol), and DIPEA (1.29 g, 9.96 mmol) in dried THF (8.2 mL). Purification by column chromatography (EtOAc) gave **C-4-33**. White solid, 0.69 g (72%). m.p. 121.2–122.8 °C. ^1^H NMR (500 MHz, CD_3_OD) *δ* 8.80–8.77 (m, 1H), 8.74 (dd, *J* = 1.5 Hz and 5.0 Hz, 1H), 8.65–8.56 (m, 2H), 8.16–8.11 (m, 2H), 8.02–8.00 (m, 1H), 7.79–7.74 (m, 1H), 7.72–7.69 (m, 1H), 7.65–7.61 (m, 3H), 7.58–7.51 (m, 2H), 7.10–7.06 (m, 1H), 7.05–7.02 (m, 2H), 6.90–6.77 (m, 2H), 3.88–3.72 (m, 1H), 3.12–3.02 (m, 1.49H), 2.86–2.85 (m, 1H), 2.73–2.63 (m, 1.58H), 2.55 (s, 1H), 2.46–2.27 (m, 2H), 1.41–1.39 (m, 0.57H), 1.34–1.24 (m, 1H), 1.15–1.02 (m, 1.53H), 0.93–0.42 (m, 1H); ^13^C NMR (201 MHz, CDCl_3_) *δ* 167.40, 167.27, 151.94, 151.68, 150.36, 150.05, 149.95, 146.23, 146.12, 136.07, 135.94, 135.73, 134.35, 134.30, 134.20, 134.11, 131.36, 131.26, 130.08, 129.91, 129.73, 129.40, 129.22, 128.41, 128.35, 128.20, 127.90, 127.01, 126.94, 126.63, 124.34, 124.14, 124.11, 123.47, 123.44, 123.29, 122.99, 46.70, 45.66, 43.35, 42.58, 42.38, 42.06, 37.29, 37.24, 35.99, 35.85, 31.84, 31.78, 31.48, 31.28; ESI-HR-MS: (*m*/*z*) calcd. for C_34_H_33_N_4_O_3_S ([M+H]^+^) 577.2268, found 577.2267.

Synthesis of N-((1-([2,4′-bipyridine]-3-carbonyl)-4-(cyclohexa-1,5-dien-1-ylmethyl)piperidin-4-yl)methyl)-4-cyclohexylbenzenesulfonamide (**C-4-34**). Following General Procedure 3, **75-34** (0.90 g, 1.71 mmol) was first treated with TFA (4.5 mL) in CH_2_Cl_2_ (2.7 mL), and the intermediate obtained was reacted with **7·TFA** (0.61 g, 2.05 mmol) using EDCI (0.82 g, 4.28 mmol), HOBT (17 mg, 0.128 mmol), and DIPEA (1.33 g, 10.3 mmol) in dried THF (9 mL). Purification by column chromatography (EtOAc) gave **C-4-34**. White foam, 0.88 g (85%). ^1^H NMR (500 MHz, CD_3_OD) *δ* 8.76–8.75 (m, 1H), 8.67–8.58 (m, 2H), 7.84–7.80 (m, 1H), 7.76–7.72 (m, 2H), 7.67–7.65 (m, 2H), 7.56–7.53 (m, 1H), 7.43–7.39 (m, 2H), 7.18–7.16 (m, 3H), 7.04–6.92 (m, 2H), 3.99–3.78 (m, 1H), 3.39–3.35 (m, 0.74H), 3.27–3.15 (m, 1H), 3.09–2.97 (m, 1H), 2.88–2.85 (m, 0.48H), 2.77–2.70 (m, 1H), 2.62–2.60 (m, 2H), 2.57–2.53 (m, 1H), 2.47–2.37 (m, 1H), 1.87–1.83 (m, 4H), 1.78–1.75 (m, 1H), 1.51–1.43 (m, 5H), 1.34–1.17 (m, 3H), 1.00–0.56 (m, 1H); ^13^C NMR (201 MHz, CDCl_3_) *δ* 167.44, 167.31, 153.31, 151.96, 151.70, 150.34, 150.02, 149.92, 146.20, 146.14, 136.82, 136.09, 135.94, 135.83, 131.36, 131.30, 130.23, 130.06, 128.18, 127.65, 127.59, 126.96, 126.89, 126.62, 126.56, 123.45, 123.42, 123.24, 123.02, 46.98, 45.74, 44.40, 42.92, 42.64, 42.51, 41.54, 37.44, 37.34, 36.03, 35.84, 34.02, 33.99, 31.80, 31.55, 31.41, 31.17, 26.55, 25.85; ESI-HR-MS: (*m*/*z*) calcd. for C_36_H_41_N_4_O_3_S ([M+H]^+^) 609.2894, found 609.2893.

Synthesis of N-((1-([2,4′-bipyridine]-3-carbonyl)-4-(cyclohexa-1,5-dien-1-ylmethyl)piperidin-4-yl)methyl)-3-(methylsulfonyl)benzenesulfonamide (**C-4-35**). Following General Procedure 3, **75-35** (0.90 g, 1.72 mmol) was first treated with TFA (4.5 mL) in CH_2_Cl_2_ (2.7 mL), and the intermediate obtained was reacted with **7·TFA** (0.61 g, 2.06 mmol) using EDCI (0.82 g, 4.30 mmol), HOBT (17 mg, 0.129 mmol), and DIPEA (1.33 g, 10.3 mmol) in dried THF (9 mL). Purification by column chromatography (EtOAc) gave **C-4-35**. White foam, 0.60 g (58%). ^1^H NMR (500 MHz, CD_3_OD) *δ* 8.76–8.75 (m, 1H), 8.67–8.58 (m, 2H), 8.40 (s, 1H), 8.23–8.13 (m, 2H), 7.85–7.82 (m, 2H), 7.67–7.65 (m, 2H), 7.56–7.54 (m, 1H), 7.19–7.17 (m, 3H), 7.04–6.93 (m, 2H), 3.95–3.74 (m, 1H), 3.55–3.37 (m, 1H), 3.21–3.17 (m, 3.52H), 3.13–3.08 (m, 0.60H), 3.02–2.91 (m, 1H), 2.83–2.76 (m, 1H), 2.64–2.53 (m, 2H), 2.49–2.39 (m, 1H), 1.53–1.50 (m, 0.57H), 1.42–1.33 (m, 1H), 1.26–1.19 (m, 1.62H), 0.96–0.66 (m, 1H); ^13^C NMR (151 MHz, CDCl_3_) *δ* 167.57, 167.49, 151.90, 151.76, 150.49, 150.06, 149.98, 146.30, 141.99, 141.85, 136.19, 136.12, 136.02, 135.76, 131.94, 131.78, 131.31, 131.15, 131.12, 130.67, 130.63, 130.27, 130.11, 128.37, 126.88, 126.81, 126.01, 123.59, 123.28, 123.24, 47.97, 46.42, 44.26, 44.20, 42.67, 42.57, 40.89, 37.52, 37.42, 36.12, 35.99, 31.75, 31.34, 31.25, 30.99; ESI-HR-MS: (*m*/*z*) calcd. for C_31_H_33_N_4_O_5_S_2_ ([M+H]^+^) 605.1887, found 605.1887.

Synthesis of N-((1-([2,4′-bipyridine]-3-carbonyl)-4-(cyclohexa-1,5-dien-1-ylmethyl)piperidin-4-yl)methyl)-4-n-propylbenzenesulfonamide (**C-4-36**). Following General Procedure 3, **75-36** (0.90 g, 1.85 mmol) was first treated with TFA (4.5 mL) in CH_2_Cl_2_ (2.7 mL), and the intermediate obtained was reacted with **7·TFA** (0.66 g, 2.22 mmol) using EDCI (0.89 g, 4.63 mmol), HOBT (19 mg, 0.139 mmol), and DIPEA (1.43 g, 11.1 mmol) in dried THF (9 mL). Purification by column chromatography (EtOAc) gave **C-4-36**. White foam, 0.81 g (77%). ^1^H NMR (500 MHz, CD_3_OD) *δ* 8.75–8.74 (m, 1H), 8.66–8.58 (m, 2H), 7.83–7.80 (m, 1H), 7.76–7.71 (m, 2H), 7.66–7.65 (m, 2H), 7.55–7.52 (m, 1H), 7.39–7.35 (m, 2H), 7.18–7.16 (m, 3H), 7.04–6.92 (m, 2H), 3.97–3.76 (m, 1H), 3.43–3.35 (m, 0.59H), 3.29–3.26 (m, 0.43H), 3.19–3.15 (m, 0.50H), 3.09–2.96 (m, 1H), 2.90–2.86 (m, 0.51H), 2.77–2.73 (m, 1H), 2.69–2.62 (m, 3H), 2.57–2.50 (m, 1H), 2.47–2.37 (m, 1H), 1.69–1.63 (m, 2H), 1.52–1.49 (m, 0.62H), 1.40–1.33 (m, 1H), 1.24–1.17 (m, 1.65H), 1.01–0.92 (m, 3.59H), 0.62–0.58 (m, 0.58H); ^13^C NMR (151 MHz, CDCl_3_ + CD_3_OD) *δ* 168.74, 168.58, 152.88, 152.61, 151.35, 150.44, 150.32, 148.98, 147.84, 138.62, 137.57, 137.32, 137.23, 132.55, 132.50, 131.45, 131.40, 130.08, 128.90, 127.81, 127.35, 127.30, 124.92, 124.56, 124.40, 48.23, 47.07, 43.90, 43.83, 43.37, 41.88, 38.53, 38.47, 36.90, 36.76, 32.48, 32.35, 31.82, 25.12, 14.07; ESI-HR-MS: (*m*/*z*) calcd. for C_33_H_37_N_4_O_3_S ([M+H]^+^) 569.2581, found 569.2582.

Synthesis of N-((1-([2,4′-bipyridine]-3-carbonyl)-4-(cyclohexa-1,5-dien-1-ylmethyl)piperidin-4-yl)methyl)-2,4,6-triisopropylbenzenesulfonamide (**C-4-37**). Following General Procedure 3, **75-37** (0.90 g, 1.58 mmol) was first treated with TFA (4.5 mL) in CH_2_Cl_2_ (2.7 mL), and the intermediate obtained was reacted with **7·TFA** (0.57 g, 1.90 mmol) using EDCI (0.76 g, 3.95 mmol), HOBT (16 mg, 0.119 mmol), and DIPEA (1.23 g, 9.48 mmol) in dried THF (9 mL). Purification by column chromatography (EtOAc) gave **C-4-37**. White foam. 0.88 g (85%). ^1^H NMR (500 MHz, DMSO-*d*_6_) *δ* 8.77 (dd, *J* = 1.5 Hz and 5.0 Hz, 1H), 8.66–8.60 (m, 2H), 7.82–7.81 (m, 1H), 7.59–7.48 (m, 4H), 7.22 (s, 2H), 7.05–6.92 (m, 3H), 4.16–4.10 (m, 2H), 3.79–3.74 (m, 1H), 3.28–3.09 (m, 1.47H), 2.94–2.89 (m, 2H), 2.73–2.69 (m, 1H), 2.64–2.62 (m, 1.48H), 2.53–2.52 (m, 0.43H), 2.36–2.33 (m, 0.58H), 1.51–1.49 (m, 0.55H), 1.35–1.28 (m, 1H), 1.20–1.18 (m, 6.60H), 1.17–1.07 (m, 14H), 0.86–0.49 (m, 1H); ^13^C NMR (151 MHz, CDCl_3_) *δ* 167.39, 152.96, 152.14, 151.80, 150.42, 150.05, 146.30, 146.09, 136.17, 136.01, 135.94, 132.20, 131.43, 131.29, 130.14, 129.98, 128.41, 126.78, 123.93, 123.54, 123.43, 123.34, 122.94, 46.03, 45.38, 43.28, 42.74, 42.65, 42.50, 37.43, 36.37, 36.12, 34.10, 32.16, 31.91, 31.70, 31.63, 29.69, 25.00, 24.98, 23.56, 23.55; ESI-HR-MS: (*m*/*z*) calcd. for C_39_H_49_N_4_O_3_S ([M+H]^+^) 653.3520, found 653.3521.

Synthesis of N-((1-([2,4′-bipyridine]-3-carbonyl)-4-(cyclohexa-1,5-dien-1-ylmethyl)piperidin-4-yl)methyl)-[1,1′-biphenyl]-4-sulfonamide (**C-4-38**). Following General Procedure 3, **75-38** (0.90 g, 1.73 mmol) was first treated with TFA (4.5 mL) in CH_2_Cl_2_ (2.7 mL), and the intermediate obtained was reacted with **7·TFA** (0.62 g, 2.08 mmol) using EDCI (0.83 g, 4.33 mmol), HOBT (18 mg, 0.130 mmol), and DIPEA (1.34 g, 10.4 mmol) in dried THF (9 mL). Purification by column chromatography (EtOAc) gave **C-4-38**. White solid, 0.63 g (60%). m.p. 215.5–216.4 °C. ^1^H NMR (500 MHz, DMSO-*d*_6_) *δ* 8.77–8.76 (m, 1H), 8.65–8.64 (m, 2H), 7.93–7.87 (m, 4H), 7.83–7.67 (m, 4H), 7.57–7.51 (m, 5H), 7.46–7.43 (m, 1H), 7.17–7.14 (m, 3H), 7.07–6.97 (m, 2H), 3.88–3.71 (m, 1H), 3.39–3.37 (m, 0.44H), 3.28–3.24 (m, 0.60H), 3.18–3.13 (m, 0.51H), 3.00–2.98 (m, 1H), 2.81–2.68 (m, 1H), 2.64–2.60 (m, 2H), 2.45–2.44 (m, 1H), 2.38–2.35 (m, 0.53H), 1.50–1.47 (m, 0.57H), 1.33–1.29 (m, 1H), 1.15–1.06 (m, 1.62H), 0.82–0.51 (m, 1H); ^13^C NMR (201 MHz, CDCl_3_) *δ* 167.61, 167.51, 152.14, 151.88, 150.52, 150.24, 150.13, 146.33, 146.27, 145.80, 145.75, 139.21, 139.10, 138.14, 136.21, 136.16, 136.10, 135.92, 131.47, 131.42, 130.28, 130.08, 129.19, 128.71, 128.64, 128.46, 128.44, 127.95, 127.87, 127.55, 127.47, 127.38, 127.33, 126.93, 126.85, 123.59, 123.55, 123.38, 123.18, 47.39, 46.08, 43.40, 42.75, 42.62, 41.77, 37.55, 37.46, 36.25, 36.07, 32.01, 31.73, 31.37; ESI-HR-MS: (*m*/*z*) calcd. for C_36_H_35_N_4_O_3_S ([M+H]^+^) 603.2424, found 603.2425.

Synthesis of N-((1-([2,4′-bipyridine]-3-carbonyl)-4-(cyclohexa-1,5-dien-1-ylmethyl)piperidin-4-yl)methyl)-4-methylbenzenesulfonamide (**C-4-39**). Following General Procedure 3, **75-39** (1.00 g, 2.18 mmol) was first treated with TFA (5 mL) in CH_2_Cl_2_ (3 mL), and the intermediate obtained was reacted with **7·TFA** (0.78 g, 2.62 mmol) using EDCI (1.04 g, 5.45 mmol), HOBT (22 mg, 0.164 mmol), and DIPEA (1.69 g, 13.1 mmol) in dried THF (10 mL). Purification by column chromatography (EtOAc) gave **C-4-39**. White foam, 0.95 g (81%). ^1^H NMR (500 MHz, DMSO-*d*_6_) *δ* 8.77 (d, *J* = 5.0 Hz, 1H), 8.66–8.64 (m, 2H), 7.84–7.79 (m, 1H), 7.70–7.67 (m, 2H), 7.57–7.52 (m, 4H), 7.42–7.39 (m, 2H), 7.18–7.16 (m, 3H), 7.06–6.96 (m, 2H), 3.86–3.70 (m, 1H), 3.25–3.11 (m, 1H), 2.96–2.77 (m, 1.71H), 2.64–2.54 (m, 2H), 2.40–2.32 (m, 5.46H), 1.47–1.44 (m, 0.61H), 1.31–1.27 (m, 1H), 1.11–1.04 (m, 1.66H), 0.81–0.51 (m, 1H); ^13^C NMR (201 MHz, CDCl_3_) *δ* 167.38, 167.25, 151.82, 151.60, 150.28, 149.89, 149.82, 146.15, 146.10, 143.40, 136.56, 136.55, 136.01, 135.88, 135.77, 131.27, 131.21, 130.19, 130.02, 129.72, 129.67, 128.09, 126.80, 126.73, 126.55, 126.49, 123.41, 123.17, 122.96, 46.89, 45.75, 42.74, 42.58, 42.43, 41.44, 37.36, 37.28, 35.92, 35.75, 31.70, 31.45, 31.28, 31.08, 21.40, 21.39; ESI-HR-MS: (*m*/*z*) calcd. for C_31_H_33_N_4_O_3_S ([M+H]^+^) 541.2268, found 541.2265.

Synthesis of N-((1-([2,4′-bipyridine]-3-carbonyl)-4-(cyclohexa-1,5-dien-1-ylmethyl)piperidin-4-yl)methyl)-4-ethylbenzenesulfonamide (**C-4-40**). Following General Procedure 3, **75-40** (0.90 g, 1.90 mmol) was first treated with TFA (4.5 mL) in CH_2_Cl_2_ (2.7 mL), and the intermediate obtained was reacted with **7·TFA** (0.68 g, 2.28 mmol) using EDCI (0.91 g, 4.75 mmol), HOBT (19 mg, 0.143 mmol), and DIPEA (1.47 g, 11.4 mmol) in dried THF (9 mL). Purification by column chromatography (EtOAc) gave **C-4-40**. White foam, 0.73 g (69%). ^1^H NMR (500 MHz, DMSO-*d*_6_) *δ* 8.77 (dd, *J* = 1.5 Hz and 4.5 Hz, 1H), 8.66–8.64 (m, 2H), 7.84–7.79 (m, 1H), 7.72–7.69 (m, 2H), 7.58–7.54 (m, 4H), 7.46–7.42 (m, 2H), 7.17–7.15 (m, 3H), 7.05–6.95 (m, 2H), 3.87–3.70 (m, 1H), 3.24–3.11 (m, 1H), 2.96–2.75 (m, 1.72H), 2.71–2.66 (m, 2H), 2.64–2.61 (m, 1.56H), 2.55–2.52 (m, 0.62H), 2.39–2.32 (m, 1.69H), 1.47–1.44 (m, 0.64H), 1.31–1.26 (m, 1H), 1.22–1.18 (m, 3.49H), 1.11–1.04 (m, 2H), 0.80–0.50 (m, 1H); ^13^C NMR (151 MHz, CDCl_3_) *δ* 167.43, 167.31, 151.92, 151.68, 150.33, 149.97, 149.89, 149.57, 146.20, 146.15, 136.78, 136.06, 135.93, 135.82, 131.33, 131.28, 130.22, 130.05, 128.63, 128.58, 128.16, 126.96, 126.89, 126.60, 126.55, 123.45, 123.22, 123.01, 46.97, 45.79, 42.86, 42.63, 42.49, 41.54, 37.42, 37.33, 35.99, 35.82, 31.79, 31.54, 31.38, 31.16, 28.68, 15.06; ESI-HR-MS: (*m*/*z*) calcd. for C_32_H_35_N_4_O_3_S ([M+H]^+^) 555.2424, found 555.2421.

Synthesis of N-((1-([2,4′-bipyridine]-3-carbonyl)-4-(cyclohexa-1,5-dien-1-ylmethyl)piperidin-4-yl)methyl)-4-isopropylbenzenesulfonamide (**C-4-41**). Following General Procedure 3, **75-41** (1.00 g, 2.05 mmol) was first treated with TFA (5 mL) in CH_2_Cl_2_ (3 mL), and the intermediate obtained was reacted with **7·TFA** (0.73 g, 2.46 mmol) using EDCI (0.98 g, 5.13 mmol), HOBT (21 mg, 0.154 mmol), and DIPEA (1.59 g, 12.3 mmol) in dried THF (10 mL). Purification by column chromatography (EtOAc) gave **C-4-41**. White foam, 0.62 g (53%). ^1^H NMR (500 MHz, DMSO-*d*_6_) *δ* 8.77 (dd, *J* = 1.5 Hz and 5.0 Hz, 1H), 8.66–8.64 (m, 2H), 7.84–7.79 (m, 1H), 7.73–7.71 (m, 2H), 7.58–7.53 (m, 4H), 7.49–7.45 (m, 2H), 7.16–7.14 (m, 3H), 7.06–6.95 (m, 2H), 3.89–3.70 (m, 1H), 3.23–3.10 (m, 1H), 3.00–2.95 (m, 2H), 2.78–2.75 (m, 0.48H), 2.66–2.53 (m, 2H), 2.47–2.32 (m, 2H), 1.47–1.44 (m, 0.58H), 1.31–1.21 (m, 7.51H), 1.14–1.04 (m, 1.66H), 0.82–0.47 (m, 1H); ^13^C NMR (201 MHz, CDCl_3_) *δ* 167.49, 167.36, 154.20, 152.00, 151.75, 150.39, 150.07, 149.99, 146.25, 146.19, 136.94, 136.13, 135.98, 135.87, 131.40, 131.34, 130.27, 130.10, 128.21, 127.33, 127.27, 127.06, 126.99, 126.64, 123.51, 123.46, 123.28, 123.06, 47.00, 45.79, 42.96, 42.69, 42.56, 41.61, 37.48, 37.38, 36.07, 35.89, 34.10, 31.85, 31.61, 31.46, 31.23, 23.64, 23.62; ESI-HR-MS: (*m*/*z*) calcd. for C_33_H_37_N_4_O_3_S ([M+H]^+^) 569.2581, found 569.2581.

Synthesis of N-((1-([2,4′-bipyridine]-3-carbonyl)-4-(cyclohexa-1,5-dien-1-ylmethyl)piperidin-4-yl)methyl)-3-isopropylbenzenesulfonamide (**C-4-42**). Following General Procedure 3, **75-42** (0.90 g, 1.85 mmol) was first treated with TFA (4.5 mL) in CH_2_Cl_2_ (2.7 mL), and the intermediate obtained was reacted with **7·TFA** (0.66 g, 2.22 mmol) using EDCI (0.89 g, 4.63 mmol), HOBT (19 mg, 0.139 mmol), and DIPEA (1.43 g, 11.1 mmol) in dried THF (9 mL). Purification by column chromatography (EtOAc) gave **C-4-42**. White foam, 0.80 g (76%). ^1^H NMR (500 MHz, DMSO-*d*_6_) *δ* 8.77 (dd, *J* = 1.5 Hz and 1.8 Hz, 1H), 8.65 (dd, *J* = 2.0 Hz and 4.5 Hz, 2H), 7.83–7.79 (m, 1H), 7.68–7.67 (m, 1H), 7.62–7.60 (m, 2H), 7.57–7.53 (m, 5H), 7.17–7.15 (m, 3H), 7.05–6.95 (m, 2H), 3.84–3.68 (m, 1H), 3.23–3.12 (m, 1H), 3.02–2.93 (m, 2H), 2.77–2.75 (m, 0.47H), 2.64–2.59 (m, 1.54H), 2.56–2.53 (m, 0.65H), 2.48–2.41 (m, 1.43H), 2.34–2.32 (m, 0.59H), 1.47–1.44 (m, 0.58H), 1.31–1.28 (m, 1H), 1.22–1.21 (m, 6.51H), 1.13–1.03 (m, 1.63H), 0.85–0.47 (m, 1H); ^13^C NMR (151 MHz, CDCl_3_) *δ* 167.55, 167.45, 152.10, 151.83, 150.47, 150.16, 150.08, 146.31, 146.20, 139.53, 136.19, 136.11, 136.02, 135.88, 131.46, 131.38, 131.10, 130.25, 130.06, 129.27, 129.25, 128.36, 126.83, 126.77, 124.81, 124.74, 124.45, 124.32, 123.56, 123.49, 123.35, 123.09, 47.12, 45.91, 43.24, 42.70, 42.60, 41.81, 37.50, 37.40, 36.15, 35.97, 34.08, 31.97, 31.74, 31.60, 31.38, 23.80, 23.77; ESI-HR-MS: (*m*/*z*) calcd. for C_33_H_37_N_4_O_3_S ([M+H]^+^) 569.2581, found 569.2582.

Synthesis of N-((1-([2,4′-bipyridine]-3-carbonyl)-4-(cyclohexa-1,5-dien-1-ylmethyl)piperidin-4-yl)methyl)-2-methylbenzenesulfonamide (**C-4-43**). Following General Procedure 3, **75-43** (0.90 g, 1.96 mmol) was first treated with TFA (4.5 mL) in CH_2_Cl_2_ (2.7 mL), and the intermediate obtained was reacted with **7·TFA** (0.70 g, 2.35 mmol) using EDCI (0.94 g, 4.90 mmol), HOBT (20 mg, 0.147 mmol), and DIPEA (1.53 g, 11.8 mmol) in dried THF (9 mL). Purification by column chromatography (EtOAc) gave **C-4-43**. White foam, 0.50 g (47%). ^1^H NMR (500 MHz, DMSO-*d*_6_) *δ* 8.77 (dd, *J* = 1.5 Hz and 5.0 Hz, 1H), 8.66–8.62 (m, 2H), 7.82–7.69 (m, 3H), 7.57–7.51 (m, 4H), 7.42–7.37 (m, 2H), 7.21–7.17 (m, 3H), 7.08–6.98 (m, 2H), 3.83–3.69 (m, 1H), 3.25–3.20 (m, 1H), 3.10–3.07 (m, 0.44H), 2.96 (s, 1H), 2.74–2.70 (m, 1H), 2.62–2.53 (m, 4.65H), 2.47–2.35 (m, 2H), 1.48–1.45 (m, 0.59H), 1.33–1.23 (m, 1H), 1.13–1.05 (m, 1.63H), 0.83–0.52 (m, 1H); ^13^C NMR (201 MHz, CDCl_3_) *δ* 167.46, 167.34, 151.97, 151.74, 150.41, 150.06, 149.99, 146.26, 146.15, 143.55, 137.75, 137.56, 136.74, 136.69, 136.17, 136.12, 136.00, 132.86, 132.83, 132.67, 132.64, 131.39, 131.27, 130.25, 130.16, 130.06, 129.97, 129.83, 129.78, 129.22, 128.96, 128.41, 128.37, 128.26, 126.92, 126.84, 126.78, 126.40, 126.25, 123.51, 123.48, 123.31, 123.28, 123.02, 46.84, 45.83, 43.45, 42.66, 42.45, 42.12, 37.34, 36.18, 36.06, 31.86, 31.77, 31.58, 31.50, 31.35, 20.23; ESI-HR-MS: (*m*/*z*) calcd. for C_31_H_33_N_4_O_3_S ([M+H]^+^) 541.2268, found 541.2282.

Synthesis of N-((1-([2,4′-bipyridine]-3-carbonyl)-4-(cyclohexa-1,5-dien-1-ylmethyl)piperidin-4-yl)methyl)-4-hydroxybenzenesulfonamide (**C-4-44**). Following General Procedure 3, **75-44** (0.90 g, 1.95 mmol) was first treated with TFA (4.5 mL) in CH_2_Cl_2_ (2.7 mL), and the intermediate obtained was reacted with **7·TFA** (0.70 g, 2.34 mmol) using EDCI (0.94 g, 4.88 mmol), HOBT (20 mg, 0.146 mmol), and DIPEA (1.51 g, 11.7 mmol) in dried THF (9 mL). Purification by column chromatography (EtOAc) gave **C-4-44**. White foam, 0.11 g (10%). ^1^H NMR (600 MHz, DMSO-*d*_6_) *δ* 10.38 (s, 1H), 8.78–8.77 (m, 1H), 8.65–8.64 (m, 2H), 7.84–7.79 (m, 1H), 7.62–7.55 (m, 5H), 7.37–7.35 (m, 1H), 7.17–7.16 (m, 3H), 7.05–7.04 (m, 1H), 6.96–6.89 (m, 3H), 3.86–3.70 (m, 1H), 3.23–3.17 (m, 1H), 3.12–3.10 (m, 0.45H), 2.96 (s, 1H), 2.78–2.58 (m, 2H), 2.48–2.31 (m, 2.46H), 1.46–1.43 (m, 0.54H), 1.30–1.23 (m, 1H), 1.10–1.02 (m, 1.52H), 0.81–0.49 (m, 1H); ^13^C NMR (151 MHz, CDCl_3_) *δ* 167.60, 161.61, 151.69, 150.65, 149.51, 149.24, 147.20, 146.91, 136.29, 136.11, 135.97, 131.40, 130.41, 130.28, 129.77, 129.32, 128.45, 126.90, 123.97, 123.88, 123.72, 116.38, 116.22, 46.73, 46.12, 43.20, 42.99, 42.69, 42.38, 37.68, 36.09, 36.02, 31.98, 31.49, 29.89, 29.81, 29.72, 29.63, 29.43; ESI-HR-MS: (*m*/*z*) calcd. for C_30_H_31_N_4_O_4_S ([M+H]^+^) 543.2061, found 543.2061.

Synthesis of 4-(N-((1-([2,4′-bipyridine]-3-carbonyl)-4-(cyclohexa-1,5-dien-1-ylmethyl)piperidin-4-yl)methyl)sulfamoyl)benzamide (**C-4-45**). Following General Procedure 3, **75-45** (0.90 g, 1.85 mmol) was first treated with TFA (4.5 mL) in CH_2_Cl_2_ (2.7 mL), and the intermediate obtained was reacted with **7·TFA** (0.66 g, 2.22 mmol) using EDCI (0.89 g, 4.63 mmol), HOBT (19 mg, 0.139 mmol), and DIPEA (1.43 g, 11.1 mmol) in dried THF (9 mL). Purification by column chromatography (EtOAc) gave **C-4-45**. White foam, 0.77 g (73%). ^1^H NMR (500 MHz, DMSO-*d*_6_) *δ* 8.77 (d, *J* = 4.5 Hz, 1H), 8.66–8.63 (m, 2H), 8.22–8.19 (m, 1H), 8.09–8.05 (m, 2H), 7.89–7.86 (m, 2H), 7.83–7.74 (m, 2H), 7.63 (s, 1H), 7.57–7.53 (m, 3H), 7.18–7.15 (m, 3H), 7.05–6.96 (m, 2H), 3.88–3.68 (m, 1H), 3.27–3.11 (m, 1H), 3.02–2.80 (m, 2H), 2.70–2.56 (m, 2.42H), 2.43–2.34 (m, 1.51H), 1.48–1.45 (m, 0.59H), 1.22–1.19 (m, 1H), 1.16–1.05 (m, 1.58H), 0.82–0.51 (m, 1H); ^13^C NMR (151 MHz, CDCl_3_) *δ* 169.04, 168.77, 167.51, 167.41, 151.51, 150.37, 149.40, 146.61, 146.44, 142.77, 142.49, 137.16, 136.17, 135.98, 135.78, 131.22, 130.28, 130.11, 128.44, 128.10, 126.80, 126.58, 123.80, 123.30, 54.08, 47.11, 46.25, 42.75, 42.48, 42.26, 41.16, 37.45, 35.83, 31.45, 31.09, 30.94, 30.76, 17.55; ESI-HR-MS: (*m*/*z*) calcd. for C_31_H_32_N_5_O_4_S ([M+H]^+^) 570.2170, found 570.2171.

Synthesis of methyl 4-(N-((1-([2,4′-bipyridine]-3-carbonyl)-4-(cyclohexa-1,5-dien-1-ylmethyl)piperidin-4-yl)methyl)sulfamoyl)benzoate (**76**). Following General Procedure 3, **75-46** (1.90 g, 3.78 mmol) was first treated with TFA (9.5 mL) in CH_2_Cl_2_ (5.7 mL), and the intermediate obtained was reacted with **7·TFA** (1.35 g, 4.54 mmol) using EDCI (1.81 g, 9.45 mmol), HOBT (38 mg, 0.284 mmol), and DIPEA (2.93 g, 22.7 mmol) in dried THF (19 mL). Purification by column chromatography (EtOAc) gave **76**. White foam, 1.91 g (86%). ^1^H NMR (500 MHz, DMSO-*d*_6_) *δ* 8.77 (dd, *J* = 1.5 Hz and 5.0 Hz, 1H), 8.64–8.63 (m, 2H), 8.19–8.14 (m, 2H), 7.95–7.92 (m, 2H), 7.86–7.78 (m, 2H), 7.57–7.53 (m, 3H), 7.18–7.17 (m, 3H), 7.11–6.97 (m, 2H), 3.91 (s, 3H), 3.86–3.68 (m, 1H), 3.26–2.98 (m, 2.58H), 2.79–2.67 (m, 1H), 2.64–2.59 (m, 2H), 2.44–2.34 (m, 1.50H), 1.45–1.30 (m, 1H), 1.12–1.05 (m, 1.67H), 0.80–0.54 (m, 1H); ^13^C NMR (201 MHz, CDCl_3_) *δ* 167.48, 167.36, 165.56, 165.47, 151.85, 151.63, 150.40, 149.95, 149.87, 146.21, 146.19, 143.68, 136.07, 135.96, 135.92, 135.67, 133.72, 131.22, 130.33, 130.29, 130.15, 129.99, 128.23, 126.84, 126.75, 126.68, 123.48, 123.24, 123.07, 60.37, 52.64, 47.46, 46.09, 42.77, 42.60, 42.48, 41.25, 37.41, 37.33, 36.02, 35.86, 31.70, 31.45, 31.30, 30.99, 22.53, 20.98, 14.10, 14.05; ESI-HR-MS: (*m*/*z*) calcd. for C_32_H_33_N_4_O_5_S ([M+H]^+^) 585.2166, found 585.2164.

Synthesis of N-((1-([2,4′-bipyridine]-3-carbonyl)-4-benzylpiperidin-4-yl)methyl)-4-aminobenzenesulfonamide (**C-4-46**). Following the procedure for the synthesis of **10** from **9**, **C-4-46** was prepared from **C-4-12** (0.40 g, 0.700 mmol) using 10% Pd/C (40 mg) in MeOH (4 mL). Purification by column chromatography (EtOAc) gave **C-4-46**. Pale yellow solid, 0.15 g (40%). m.p. 125.4–127.6 °C. 1H NMR (500 MHz, CD_3_OD) δ 8.77–8.76 (m, 1H), 8.68–8.60 (m, 2H), 7.85–7.83 (m, 1H), 7.67–7.66 (m, 2H), 7.58–7.55 (m, 1H), 7.52–7.47 (m, 2H), 7.21–7.19 (m, 3H), 7.06–6.93 (m, 2H), 6.72–6.68 (m, 2H), 3.99–3.96 (m, 1H), 3.26–3.17 (m, 1H), 3.05–2.99 (m, 1H), 2.86–2.83 (m, 0.58H), 2.73–2.63 (m, 2H), 2.50–2.37 (m, 2H), 1.53–1.50 (m, 0.67H), 1.42–1.34 (m, 1H), 1.23–1.20 (m, 1.61H), 1.03–0.86 (m, 1H), 0.62–0.60 (m, 0.58H); ^13^C NMR (151 MHz, CD_3_OD) *δ* 169.06, 168.94, 154.09, 153.23, 152.90, 151.60, 151.58, 150.62, 150.54, 148.25, 138.00, 137.77, 137.61, 137.51, 132.90, 132.83, 131.69, 129.96, 129.08, 127.50, 127.22, 125.22, 124.88, 124.69, 114.50, 48.20, 47.12, 44.17, 43.61, 42.14, 38.74, 37.03, 36.90, 32.84, 32.75, 32.11; ESI-HR-MS: (*m*/*z*) calcd. for C_30_H_32_N_5_O_3_S ([M+H]^+^) 542.2220, found 542.2219.

Synthesis of 4-(N-((1-([2,4′-bipyridine]-3-carbonyl)-4-benzylpiperidin-4-yl)methyl)sulfamoyl)benzoic acid (**C-4-47**). Following the procedure for the synthesis of **14** from **13**, **C-4-47** was prepared from **76** (0.50 g, 0.855 mmol) using NaOH (68 mg, 1.71 mmol) dissolved in H_2_O (0.12 mL) in MeOH (4 mL), followed by acidification by concentrated HCl (0.14 mL). White solid, 0.20 g (41%). ^1^H NMR (500 MHz, DMSO-*d*_6_) *δ* 8.94–8.85 (m, 3H), 8.14–8.08 (m, 4H), 7.94–7.92 (m, 4H), 7.69–7.66 (m, 1H), 7.18–7.16 (m, 3H), 7.02 (s, 2H), 3.77–3.40 (m, 2H), 3.12–3.02 (m, 2H), 2.59 (s, 4H), 1.40–1.12 (m, 4H); ^13^C NMR (151 MHz, CD_3_OD) *δ* 168.16, 168.00, 156.42, 152.14, 149.96, 145.45, 143.41, 137.84, 137.64, 135.57, 133.62, 131.76, 131.47, 129.09, 128.04, 127.54, 127.48, 126.69, 44.38, 41.89, 38.84, 37.31, 32.34, 31.40; ESI-HR-MS: (*m*/*z*) calcd. for C_31_H_31_N_4_O_5_S ([M+H]^+^) 571.2010, found 571.2008.

Synthesis of N-((1-([2,4′-bipyridine]-3-carbonyl)-4-benzylpiperidin-4-yl)methyl)-4-(hydroxymethyl)benzenesulfonamide (**C-4-48**). Following the procedure for the synthesis of **B9** from **B8**, **C-4-48** was prepared from **76** (0.50 g, 0.855 mmol) using LiAlH_4_ (39 mg, 1.03 mmol) in dried THF (5 mL) at room temperature. Purification by column chromatography (EtOAc) gave **C-4-48**. White solid, 55 mg (12%). m.p. 125.4–126.7 °C. ^1^H NMR (500 MHz, DMSO-*d*_6_) *δ* 8.77 (d, *J* = 4.5 Hz, 1H), 8.66–8.64 (m, 2H), 7.84–7.75 (m, 3H), 7.57–7.51 (m, 7H), 7.18–7.16 (m, 3H), 7.06–6.96 (m, 2H), 5.45–5.41 (m, 1H), 4.61–4.58 (m, 2H), 3.86–3.70 (m, 1H), 3.26–3.12 (m, 1.69H), 2.97 (s, 1H), 2.79–2.62 (m, 2H), 2.39–2.32 (m, 2H), 1.45–1.23 (m, 2H), 1.12–1.04 (m, 1.62H), 0.81–0.52 (m, 1H); ^13^C NMR (151 MHz, DMSO-*d*_6_) *δ* 166.49, 166.32, 151.41, 151.27, 150.17, 149.87, 147.41, 145.89, 138.54, 136.84, 136.60, 135.88, 135.78, 131.55, 131.44, 130.53, 130.47, 127.82, 126.74, 126.45, 126.16, 123.67, 122.88, 122.71, 62.24, 46.78, 45.67, 42.32, 42.18, 41.42, 36.83, 36.75, 35.55, 35.50, 31.32, 30.79; ESI-HR-MS: (*m*/*z*) calcd. for C_31_H_33_N_4_O_4_S ([M+H]^+^) 557.2217, found 557.2222.

Synthesis of *tert*-butyl 4-(((3-aminophenyl)sulfonamido)methyl)-4-benzylpiperidine-1-carboxylate (**77**). Following the procedure for the synthesis of **10** from **9**, **77** was prepared from **75** to **47** (2.50 g, 5.11 mmol) using 10% Pd/C (0.25 g) in MeOH (25 mL). Column chromatography (EtOAc/PE = 1/2 by *v*/*v*) gave **77**. White solid, 2.04 g (87%). m.p. 159.1–160.3 °C. ^1^H NMR (500 MHz, DMSO-*d*_6_) *δ* 7.44 (t, *J* = 6.3 Hz, 1H), 7.23–7.15 (m, 4H), 7.10–7.08 (m, 2H), 6.99 (t, *J* = 2.0 Hz, 1H), 6.92–6.89 (m, 1H), 6.76–6.74 (m, 1H), 5.56 (s, 2H), 3.44–3.39 (m, 2H), 3.16–3.14 (m, 2H), 2.62–2.61 (m, 4H), 1.35 (s, 9H), 1.33–1.30 (m, 2H), 1.22–1.17 (m, 2H); ^13^C NMR (201 MHz, CDCl_3_) *δ* 154.92, 147.56, 140.21, 136.68, 130.47, 130.13, 128.28, 126.58, 118.93, 116.32, 112.66, 79.54, 47.30, 42.27, 39.81, 38.84, 36.06, 32.18, 28.48; ESI-HR-MS: (*m*/*z*) calcd. for C_24_H_33_N_3_NaO_4_S ([M+Na]^+^) 482.2084, found 482.2084.

Synthesis of *tert*-butyl 4-(((3-acetamidophenyl)sulfonamido)methyl)-4-benzylpiperidine-1-carboxylate (**78**). Compound **78** was prepared according to General Procedure 1 from **77** (1.50 g, 5.11 mmol) using AcCl (0.31 g, 3.91 mmol) and Et_3_N (0.99 g, 9.78 mmol) in dried CH_2_Cl_2_ (15 mL). Purification by column chromatography (EtOAc/PE = 1/1, by *v*/*v*) gave **78**. White foam, 1.27 g (78%). ^1^H NMR (500 MHz, DMSO-*d*_6_) *δ* 10.25 (s, 1H), 8.15 (s, 1H), 7.76–7.74 (m, 1H), 7.66 (t, *J* = 6.5 Hz, 1H), 7.54–7.50 (m, 1H), 7.48–7.46 (m, 1H), 7.21–7.16 (m, 3H), 7.10–7.08 (m, 2H), 3.43–3.38 (m, 2H), 3.15 (s, 2H), 2.64–2.62 (m, 4H), 2.07 (s, 3H), 1.35 (s, 9H), 1.33–1.30 (m, 2H), 1.22–1.17 (m, 2H); ^13^C NMR (201 MHz, CDCl_3_) *δ* 169.50, 155.09, 139.99, 139.51, 136.64, 130.51, 129.88, 128.37, 126.71, 124.11, 122.31, 117.88, 79.80, 47.57, 42.28, 39.88, 38.93, 36.14, 32.13, 28.53, 24.55; ESI-HR-MS: (*m*/*z*) calcd. for C_26_H_35_N_3_NaO_5_S ([M+Na]^+^) 524.2190, found 524.2190.

Synthesis of N-(3-(N-((1-([2,4′-bipyridine]-3-carbonyl)-4-benzylpiperidin-4-yl)methyl)sulfamoyl)phenyl)acetamide (**C-4-49**). Following General Procedure 3, **78** (1.10 g, 2.19 mmol) was first treated with TFA (5.5 mL) in CH_2_Cl_2_ (3.3 mL), and the intermediate obtained was reacted with **7·TFA** (0.78 g, 2.63 mmol) using EDCI (1.05 g, 5.48 mmol), HOBT (22 mg, 0.164 mmol), and DIPEA (1.69 g, 13.1 mmol) in dried THF (11 mL). Purification by column chromatography (EtOAc) gave **C-4-49**. Pale yellow foam, 1.05 g (82%). ^1^H NMR (500 MHz, DMSO-*d*_6_) *δ* 10.27–10.24 (m, 1H), 8.77 (dd, *J* = 1.8 Hz and 4.8 Hz, 1H), 8.65–8.64 (m, 2H), 8.16 (s, 1H), 7.84–7.65 (m, 3H), 7.58–7.50 (m, 4H), 7.47–7.45 (m, 1H), 7.18–7.17 (m, 3H), 7.06–6.97 (m, 2H), 3.83–3.69 (m, 1H), 3.26–3.14 (m, 1H), 2.97 (s, 1H), 2.82–2.63 (m, 2H), 2.57–2.56 (m, 1H), 2.44–2.33 (m, 1.46H), 2.07 (s, 3H), 1.45–1.23 (m, 2H), 1.14–1.05 (m, 1.58H), 0.85–0.53 (m, 1H); ^13^C NMR (201 MHz, CDCl_3_) *δ* 169.89, 169.43, 167.56, 167.51, 151.85, 151.32, 150.71, 150.48, 149.96, 149.39, 147.43, 146.39, 140.15, 139.85, 139.55, 139.50, 136.54, 136.22, 135.95, 131.59, 131.42, 130.53, 130.48, 130.42, 130.26, 129.75, 128.37, 128.35, 126.73, 124.27, 124.12, 124.01, 123.92, 123.61, 123.34, 121.96, 121.22, 117.79, 47.75, 46.28, 42.85, 42.81, 42.38, 40.63, 37.59, 37.52, 36.18, 35.99, 31.24, 30.95, 24.36, 24.26; ESI-HR-MS: (*m*/*z*) calcd. for C_32_H_34_N_5_O_4_S ([M+H]^+^) 584.2326, found 584.2328.

### 3.2. In Vitro CYP46A1 Inhibition

Chemical Materials: The chemicals used in this assay were obtained commercially: terfenadine (Sigma-Aldrich, T9652, Shanghai, China), tolbutamide (Sigma-Aldrich, T0891, Shanghai, China), buspirone (MedChemExpress, HY-B1115A, Shanghai, China), KH_2_PO_4_ (Shanghai Titanchem, 7778-77-0, Shanghai, China), NADPH (ACROS, 328742500, Shanghai, China), and testosterone (Adamas Reagent, 171265, Shanghai, China).

Study Procedure: Recombinant human CYP46A1R bactosomes (Cypex, 1000 nM) were incubated with testosterone (15 µM as substrate) and a series of test compounds across a seven-point concentration range (0, 0.31, 2.44, 19.5, 156, 1250, 10,000 nM) in 50 mM KH_2_PO_4_ buffer (pH 7.4) in a 96-deep well plate. The reaction mixture was incubated at 38 °C for 10 min after initiation with 5 mM NADPH and was then terminated with an MeCN quenching solution containing terfenadine, buspirone, and tolbutamide as internal standards. Following centrifugation, the supernatants were collected for LC-MS/MS analysis.

LC-MS/MS Conditions: The LC-MS/MS analysis was performed on an API 5500 mass spectrometer in positive ESI mode with unit resolution for both Q1 and Q3. Chromatographic separation used a Kinetex Polar C_18_ column (2.6 μm, 2.1 × 50 mm) with a 3 min gradient at a flow rate of 0.7 mL/min, where mobile phase A was 0.1% formic acid in water and mobile phase B was 0.1% formic acid in acetonitrile. Key monitored multiple reaction monitoring (MRM) transitions included terfenadine (472.4/436.4), tolbutamide (271.025/91), 16β-hydroxytestosterone (305.203/97.1 and 305.203/109.2), and buspirone (386.2/122).

Calculation: The enzyme activity for each concentration was calculated as the percentage of the substrate metabolite peak area ratio at a given concentration (0, 0.31, 2.44, 19.5, 156, 1250, 10,000 nM) relative to the peak area ratio at the zero-concentration control (0 nM). Correspondingly, the inhibition percentage was derived as follows: Inhibition (%) = 100% − Enzyme Activity (%). The half-maximal inhibitory concentration (IC_50_) values were subsequently determined by analyzing the concentration–response curve using nonlinear regression in GraphPad Prism software (version 5.03, GraphPad Software, Inc., Boston, MA, USA).

### 3.3. In Vitro Inhibitory Activities of Target Compounds Against CYP1A2, CYP2C9, CYP2C19, CYP2D6, and CYP3A4

Chemical Materials: The study utilized pooled mixed-gender human liver microsomes (Corning, 452117, Suzhou, China). A panel of chemical inhibitors and substrates for specific CYP isoforms was obtained commercially from various vendors: terfenadine (Sigma-Aldrich, T9652, Shanghai, China), tolbutamide (Sigma-Aldrich, T0891, Shanghai, China), buspirone (MedChemExpress, HY-B1115A, Shanghai, China), phenacetin (Sigma-Aldrich, 101690303, Shanghai, China), diclofenac (MCE, HY-15036, Shanghai, China), mephenytoin (Glpbio, GC14486, Shanghai, China), dextromethorphan (Sigma-Aldrich, D9684-5G, Shanghai, China), midazolam (Shanghai yuanye Bio-Technology, PVJT-0H9Z, Shanghai, China), K_2_HPO_4_ (SCR, 20032117, Shanghai, Chinna), and NADPH (ABCONE, N99640-100MG, Shanghai, China).

Study Procedure: The incubation was automated on a TECAN Fluent 780 workstation. A liver microsome working solution containing a 5-in-1 substrate cocktail (phenacetin, diclofenac, mephenytoin, dextromethorphan, and midazolam) was pre-incubated at 38 °C for 7 min. Test compounds were then added at six non-zero concentrations (0, 0.049, 0.20, 0.78, 3.1, 12.5, 50 µM). The reaction was initiated with 5 mM NADPH and incubated at 38 °C for 10 min before termination with an MeCN quenching solution containing terfenadine, tolbutamide, and buspirone as internal standards. Following centrifugation, the supernatants were collected for LC-MS/MS analysis.

LC-MS/MS Conditions: Analysis was performed on an AB Sciex API 5500 mass spectrometer (AB SCIEX, Singapore) in positive ESI mode. Chromatographic separation used a Kinetex Polar C_18_ column (2.6 μm, 2.1 × 50 mm, Phenomenex, Torrance, CA, USA) with a 2.5 min gradient of 0.1% formic acid in water (mobile phase A) and 0.1% formic acid in acetonitrile (mobile phase B) at a flow rate of 0.7 mL/min. Analytes were monitored via specific MRM transitions: acetaminophen (metabolite of phenacetin; 152.1/110.0), 4′-hydroxydiclofenac (metabolite of diclofenac; 312.1/230.0), 4′-hydroxymephenytoin (metabolite of mephenytoin; 235.0/150.0), dextrorphan (metabolite of dextromethorphan; 258.2/199.1), and 1′-hydroxymidazolam (metabolite of midazolam; 342.1/203.0).

Calculation: The enzyme activity for each compound concentration was calculated as the percentage of the substrate metabolite peak area ratio at a given concentration (0, 0.049, 0.20, 0.78, 3.1, 12.5, 50 µM) relative to the peak area ratio at the zero-concentration control (0 nM). Correspondingly, the inhibition percentage was derived as follows: Inhibition (%) = 100% − Enzyme Activity (%). The half-maximal inhibitory concentration (IC_50_) values were subsequently determined by analyzing the concentration–response curve using nonlinear regression in GraphPad Prism software (version 5.03, GraphPad Software, Inc., Boston, MA, USA).

## 4. Conclusions

In conclusion, three series of soticlestat derivatives were designed and evaluated for their in vitro CYP46A1 inhibitory activity. Among the designed series, Series A, B, and subseries C-1 to C-3 were associated with significantly decreased CYP46A1 inhibitory activity, while a lot of compounds with benzenesulfonamide moiety (subseries C-4) as an isostere of OH in soticlestat were discovered to possess CYP46A1 inhibitory activities comparable to that of soticlestat. The most potent compound was **C-4-38**, with an IC_50_ of 13.6 nM, compared with an IC_50_ of 18.0 nM for soticlestat. An interesting flat SAR profile was also observed in some compounds in subseries C-4. Although four selected compounds displayed low selectivity toward CYP3A4/5 inhibition, which will be optimized in future work to minimize potential drug–drug interactions, the findings in the present study still remain very valuable for the design of potent CYP46A1 inhibitors.

## Figures and Tables

**Table 1:** In vitro CYP46A1 inhibitory activity (IC_50_) of target compounds **A1**–**A8**, **B1**–**B11**, **C-1-1** to **C-1-3**, **C-2-1** to **C-2-8**, **C-3-1** to **C-3-6**, and **C-4-1** to **C-4-49**.

Compound	IC_50_ (nM)	Compd	IC_50_ (nM)	Compd	IC_50_ (nM)	Compd	IC_50_ (nM)
Soticlestat	18.0	**C-1-3**	296.3	**C-4-8**	41.8	**C-4-30**	36.47
**A1**	>10,000	**C-2-1**	3562	**C-4-9**	36.2	**C-4-31**	56.13
**A2**	>10,000	**C-2-2**	>10,000	**C-4-10**	79.4	**C-4-32**	25.7
**A3**	>10,000	**C-2-3**	>10,000	**C-4-11**	113.2	**C-4-33**	82.3
**A4**	>10,000	**C-2-4**	>10,000	**C-4-12**	168.6	**C-4-34**	32.5
**A5**	>10,000	**C-2-5**	5900	**C-4-13**	63.76	**C-4-35**	371.4
**A6**	>10,000	**C-2-6**	4346	**C-4-14**	117.3	**C-4-36**	51.2
**A7**	>10,000	**C-2-7**	111.8	**C-4-15**	63.02	**C-4-37**	24.2
**A8**	>10,000	**C-2-8**	>10,000	**C-4-16**	54.49	**C-4-38**	13.6
**B1**	3375	**C-3-1**	>10,000	**C-4-17**	17.35	**C-4-39**	117.7
**B2**	>10,000	**C-3-2**	2193	**C-4-18**	56.15	**C-4-40**	63.0
**B3**	1023	**C-3-3**	226.7	**C-4-19**	65.67	**C-4-41**	17.4
**B4**	>10,000	**C-3-4**	156.1	**C-4-20**	50.92	**C-4-42**	48.6
**B5**	>10,000	**C-3-5**	1634	**C-4-21**	41.7	**C-4-43**	127.5
**B6**	>10,000	**C-3-6**	372.6	**C-4-22**	43.99	**C-4-44**	150.6
**B7**	>10,000	**C-4-1**	526.9	**C-4-23**	62.05	**C-4-45**	317.9
**B8**	>10,000	**C-4-2**	151.5	**C-4-24**	56.38	**C-4-46**	147.4
**B9**	>10,000	**C-4-3**	1849	**C-4-25**	52.68	**C-4-47**	3240
**B10**	140.9	**C-4-4**	982.6	**C-4-26**	74.22	**C-4-48**	379.5
**B11**	>10,000	**C-4-5**	133	**C-4-27**	27.5	**C-4-49**	166.1
**C-1-1**	102	**C-4-6**	126.1	**C-4-28**	41.23		
**C-1-2**	5583	**C-4-7**	526.3	**C-4-29**	33.31		

**Table 2:** Inhibitory activities of selected compounds **C-4-9**, **C-4-32**, **C-4-38**, and **C-4-41** against off-target CYP enzymes (1A2, 2C9, 2C19, 2D6, and 3A4/5).

Compd	1A2		2C9		2C19		2D6		3A4/5	
	IC_50_ (μM)	SR ^a^	IC_50_ (μM)	SR ^a^	IC_50_ (μM)	SR ^a^	IC_50_ (μM)	SR ^a^	IC_50_ (μM)	SR ^a^
**C-4-9**	>50	>1381	6.29	173	14.7	406	2.90	80	0.403	11
**C-4-32**	>50	>1946	4.27	166	3.34	130	5.81	226	0.0948	3.7
**C-4-38**	>50	>3676	1.85	136	1.54	113	4.04	297	0.0402	3.0
**C-4-41**	>50	>2874	5.20	299	4.59	264	2.95	170	0.0898	5.2

^a^ SR = selectivity ratio = IC_50_ (off-target CYP)/IC_50_ (CYP46A1).

## Data Availability

The data presented in this study are available on request from the corresponding authors.

## Supplementary Materials

The following supporting information can be downloaded at: https://www.mdpi.com/article/10.3390/molecules31030460/s1, the copies of all the ^1^H NMR, ^13^C NMR, ^19^F NMR, ^31^P NMR, and HR-MS spectra of synthesized intermediates and target compounds.

## Abbreviations

The following abbreviations are used in this manuscript:

AD	Alzheimer’s disease
BBB	blood–brain barrier
CH24H	cholesterol 24-hydroxylase
dba	dibenzylideneacetone
DEE	developmental and epileptic encephalopathy
DME	1,2-dimethoxyethane
DMF	N,N-dimethylformamide
DS	Dravet syndrome
EAAT2	excitatory amino acid transporter 2
EDCI	1-ethyl-3-(3-dimethylaminopropyl)carbodiimide hydrochloride
ESI	electrospray ionization
HOBT	1-hydroxybenzotriazole
HR-MS	high-resolution mass spectrometry
HTD	Huntington’s disease
IC_50_	Half-maximal inhibitory concentration
LDA	lithium diisopropylamide
LGS	Lennox–Gastaut syndrome
LiHMDS	lithium bis(trimethylsilyl)amide
*m*CPBA	*m*-chloroperoxybenzoic acid
NMDA	N-methyl-D-aspartate
PD	Parkinson’s disease
PE	petroleum ether
PIFA	phenyliodine (III) bis(trifluoroacetate)
TBAF	tetra-*n*-butylammonium fluoride
TFA	trifluoroacetic acid
TLC	thin-layer chromatography
TMS	trimethylsilyl
Xantphos	9,9-dimethyl-4,5-bis(diphenylphosphino)xanthene
