# Supplementary material for: Structure–Activity Relationship Study on Soticlestat Derivatives for the Discovery of CYP46A1 (CH24H) Inhibitors
Source: Molecules. 2026 Jan 28;31(3):460. doi: 10.3390/molecules31030460 (PMC12899276; doi:10.3390/molecules31030460)
Supplement: Supplementary file 1 [file molecules-31-00460-s001.zip › molecules-4122710-supplementary.pdf]

Structure–Activity Relationship Study on Soticlestat Derivatives for the Discovery of CYP46A1 (CH24H) Inhibitors

Xinwei Hu[1,2,†], Wenqian Huang[2,3,4,†], Xiaotong Lin[2], Hao Zhang[2,3,4], Yishu Huang[2,3,4], Jiang Wu[5,*], Guilong Zhao[1,2,3,4,*]

[1]Guangzhou University of Chinese Medicine, Guangzhou 510006, China; 19966405675@163.com (X.H.)
[2]Zhongshan Institute for Drug Discovery, Shanghai Institute of Materia Medica, Chinese Academy of Sciences, Zhongshan 528400, China; 18379981865@163.com (W.H.); linxiaotong@zidd.ac.cn (X.L.); 19003708@mail.ecust.edu.cn (H.Z.); huangyishu@simm.ac.cn (Y.H.)
[3]Shanghai Institute of Materia Medica, Chinese Academy of Sciences, Shanghai 201203, China;
[4]University of Chinese Academy of Sciences, Beijing 100049, China;
[5]College of Pharmacy, Shenzhen Technology University, Shenzhen 518118, China. wujiang@sztu.edu.cn (J.W.)

*Correspondence: wujiang@sztu.edu.cn (J.W.); zhao_guilong@126.com (G.Z.)
[†]These authors contributed equally to this work

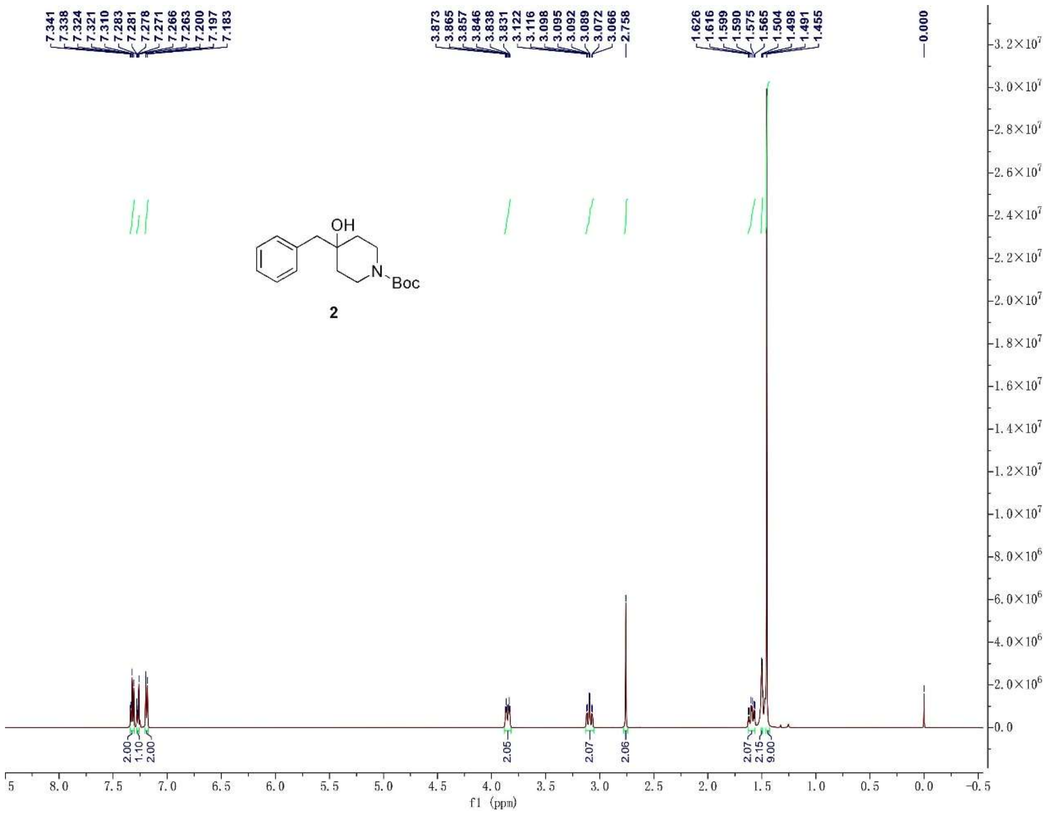

Figure S1: ^{1}H NMR spectrum of **2**

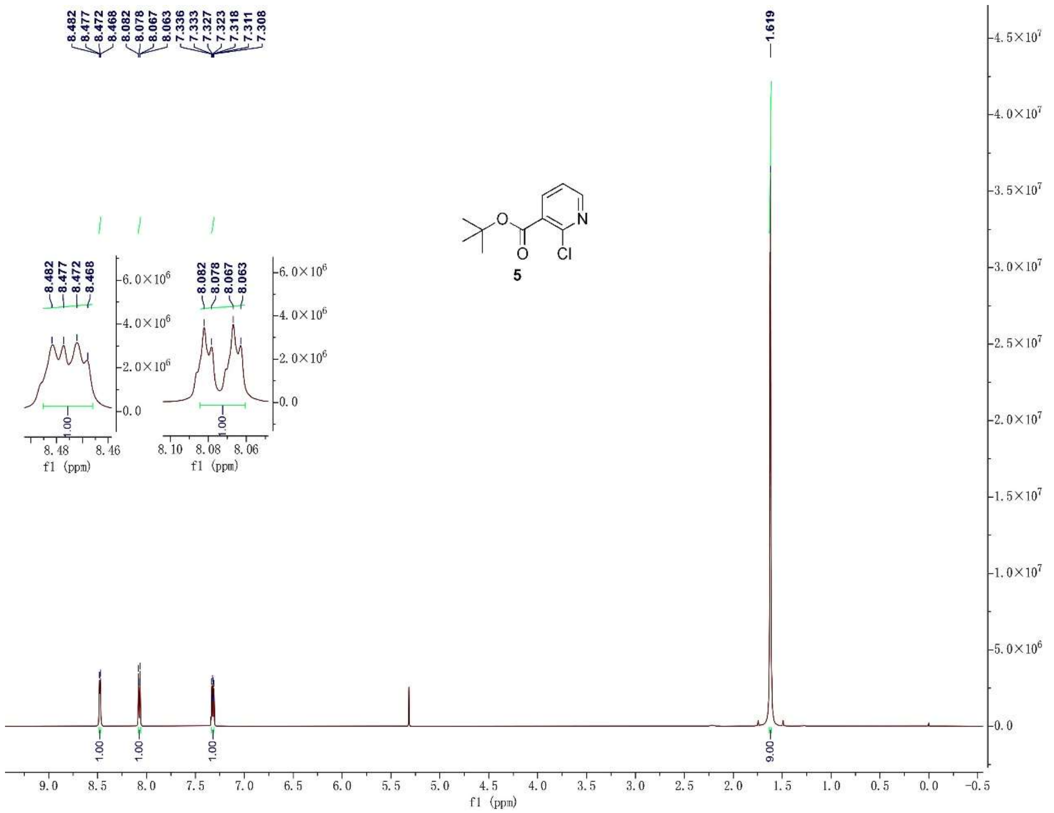

Figure S2: ^{1}HNMR spectrum of **5**

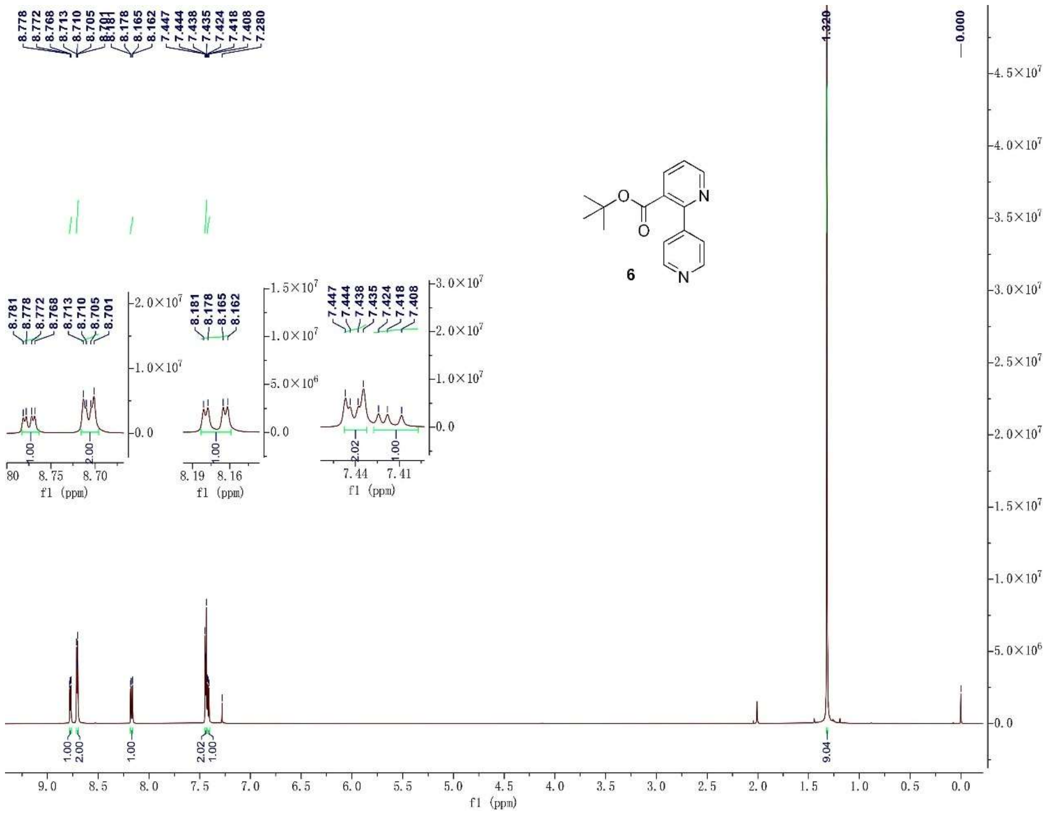

Figure S3: ^{1}HNMR spectrum of **6**

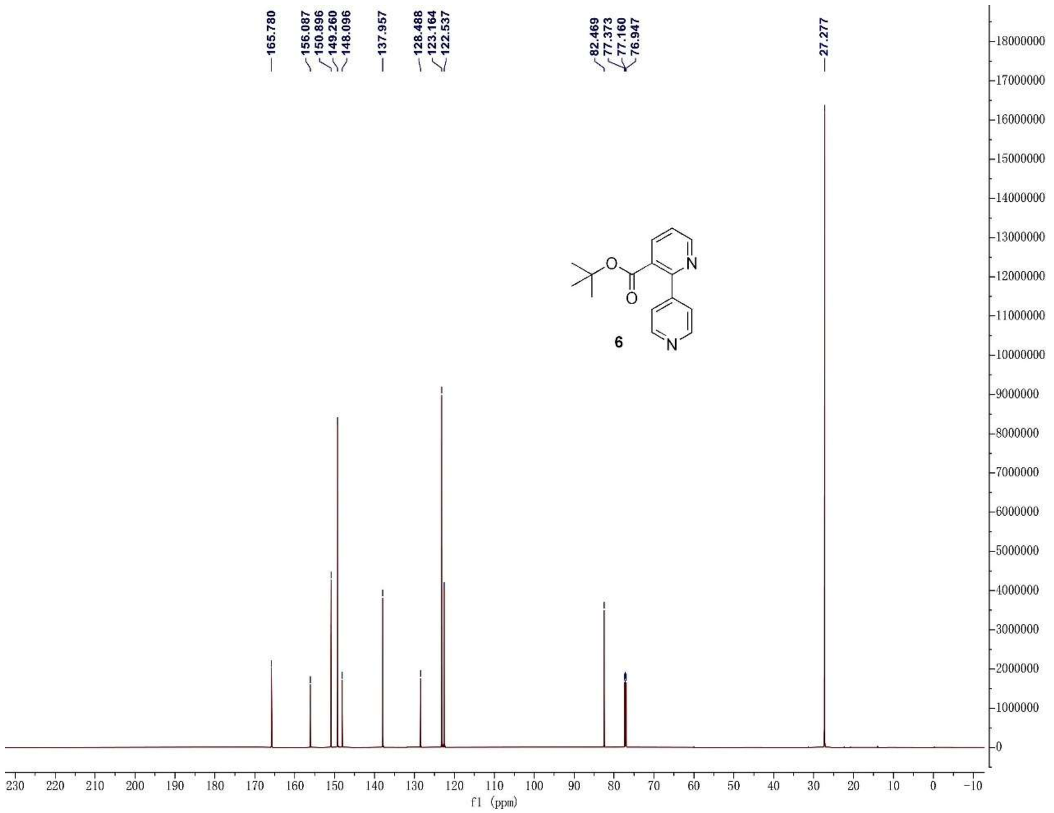

Figure S4: ^{13}C NMR spectrum of **6**

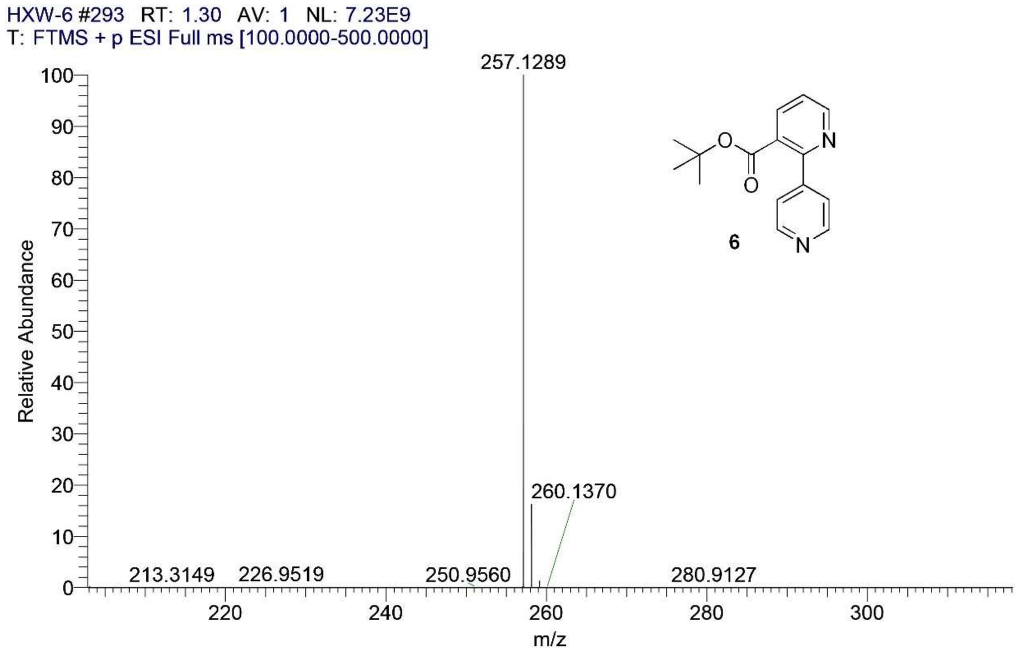

Figure S5: HR-MS (ESI/ion trap) spectrum of **6**

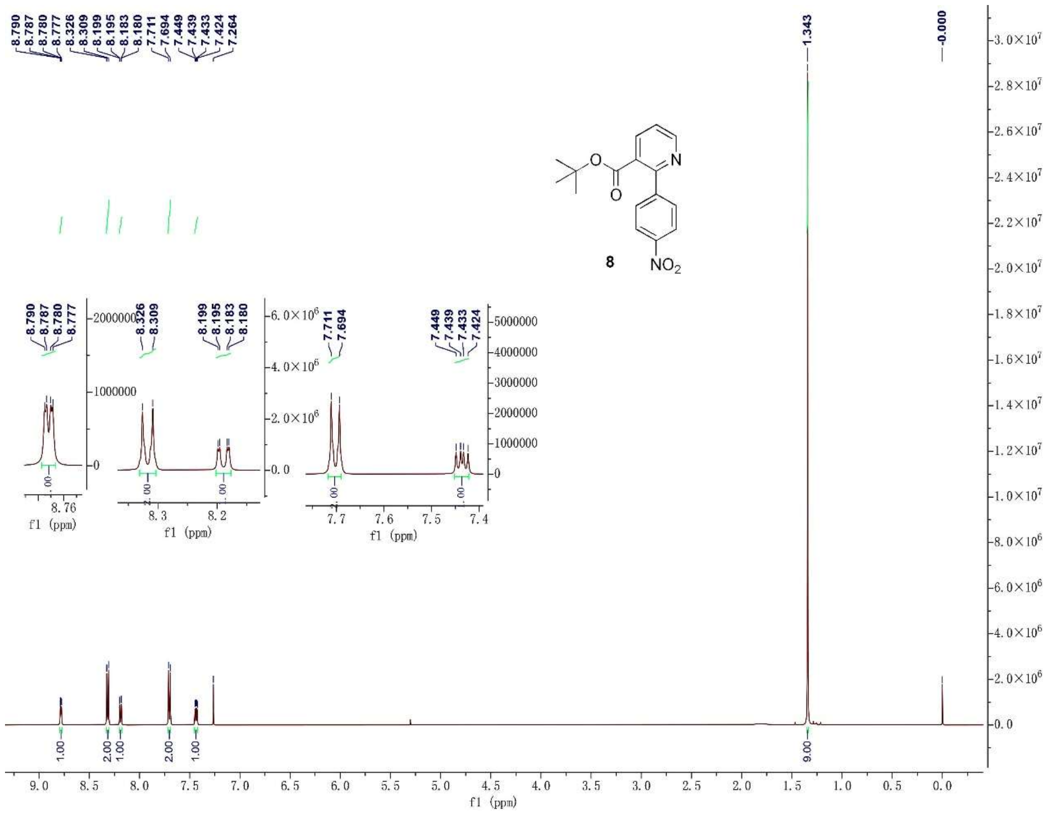

Figure S6: ^{1}H NMR spectrum of **8**

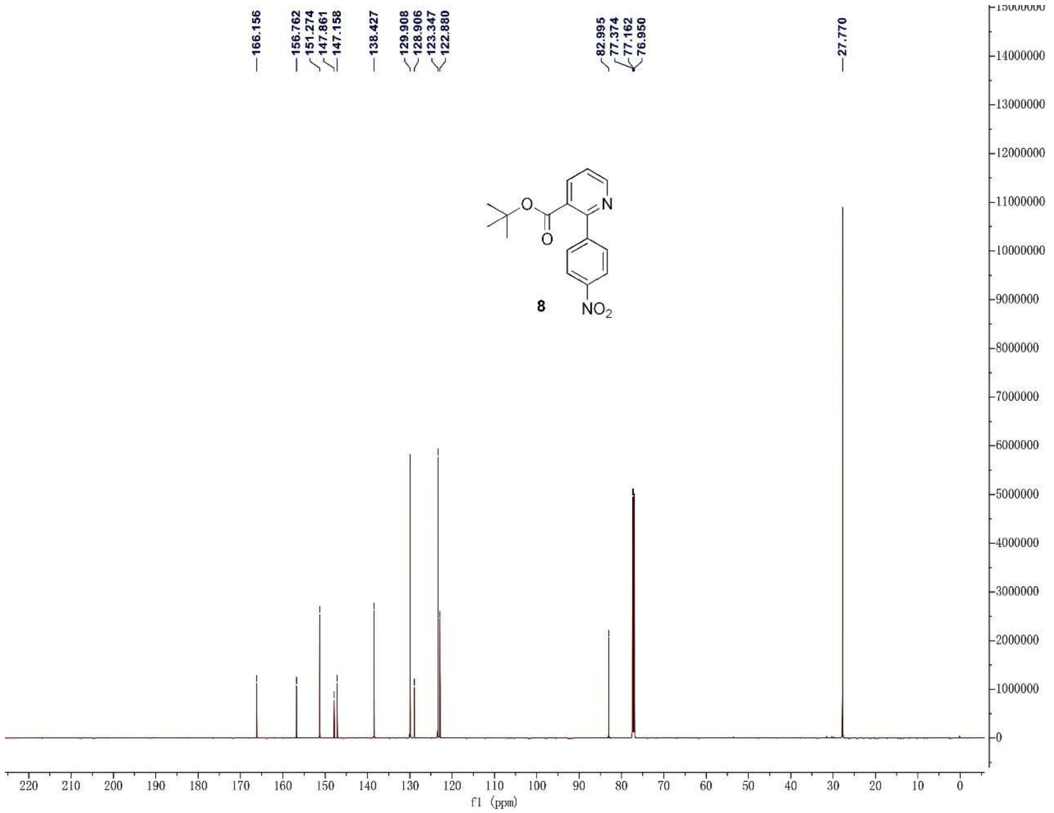

Figure S7: ^{13}C NMR spectrum of **8**

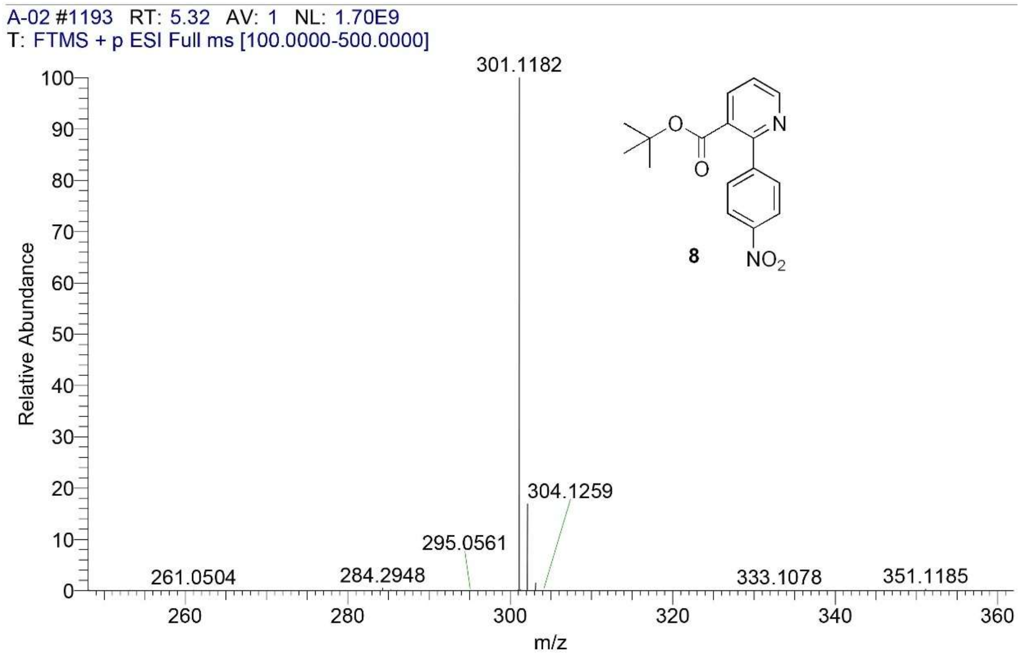

Figure S8: HR-MS (ESI/ion trap) spectrum of **8**

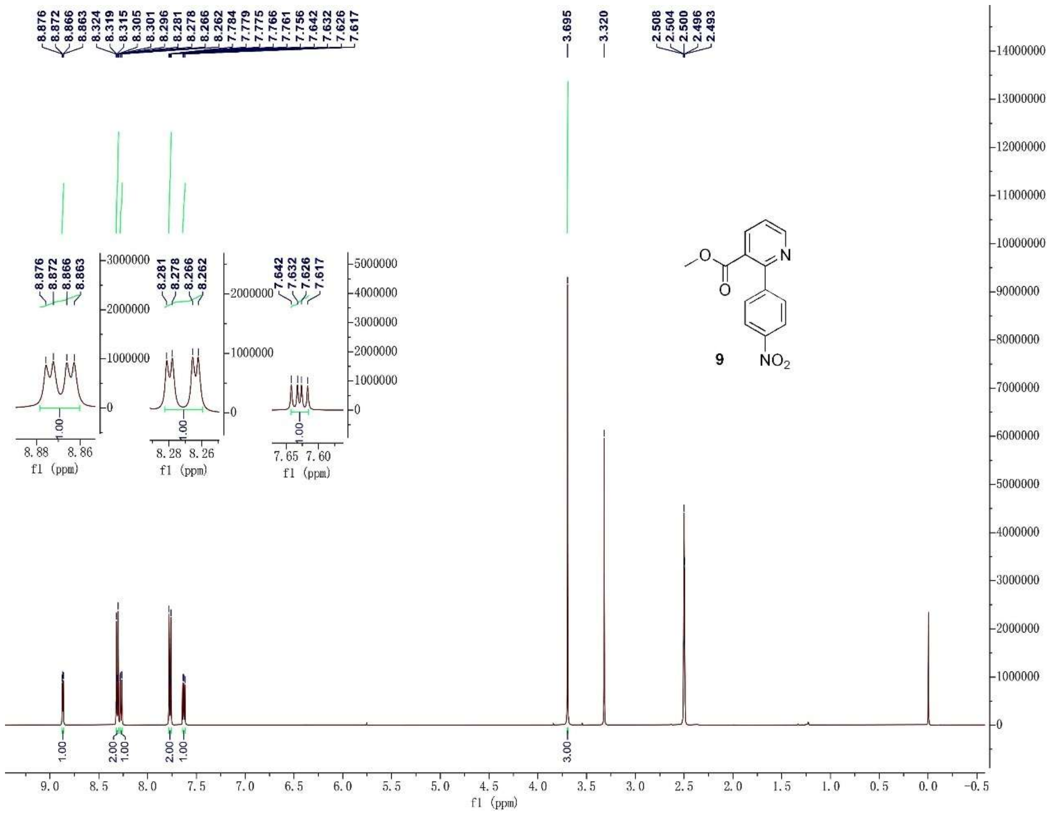

Figure S9: ^{1}H NMR spectrum of **9**

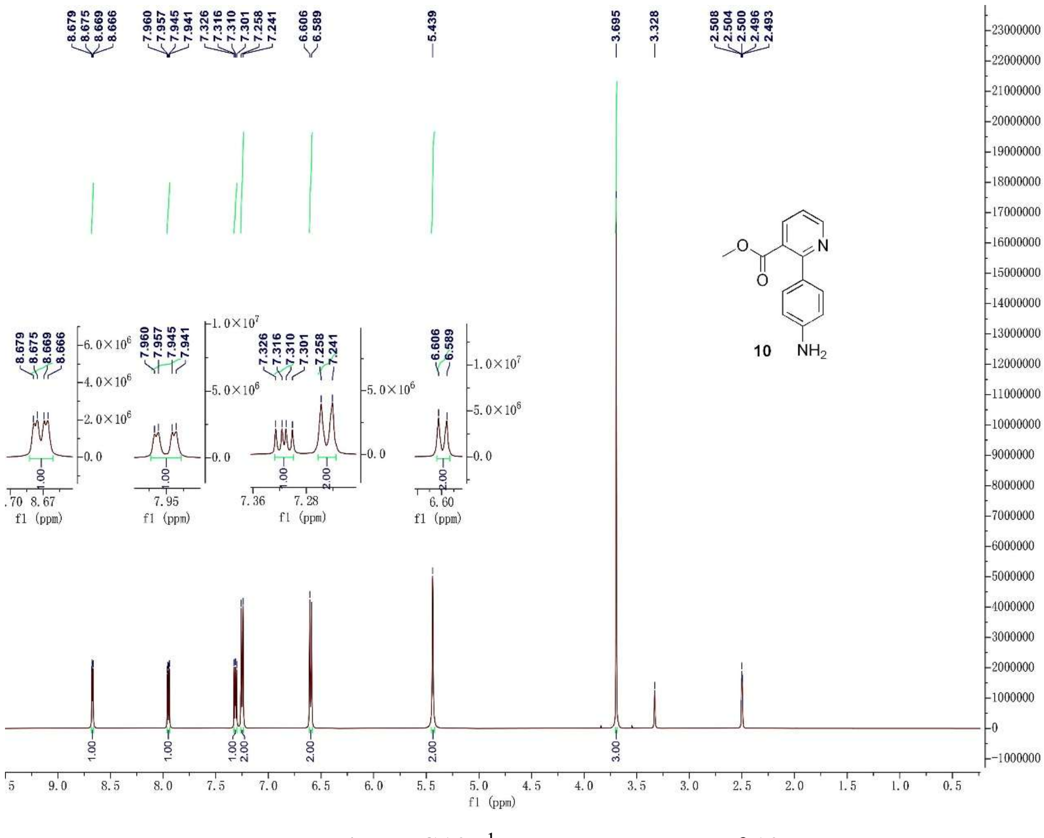

Figure S10: ^{1}H NMR spectrum of **10**

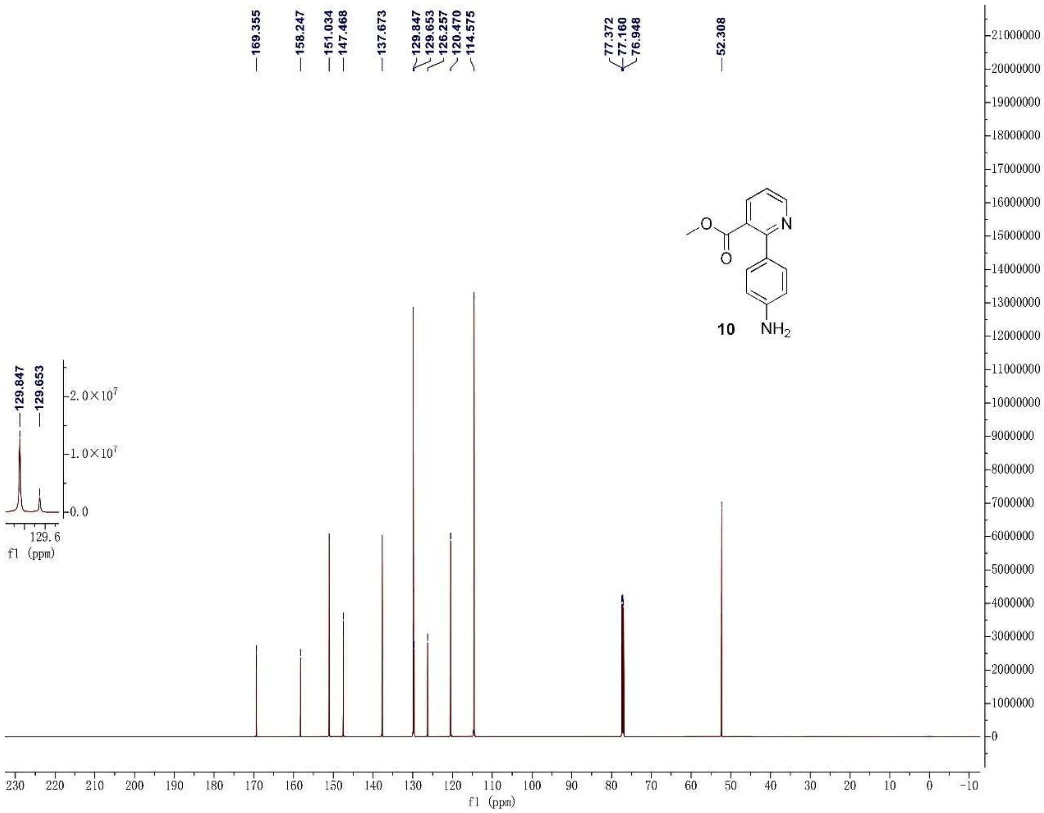

Figure S11: ^{13}C NMR spectrum of **10**

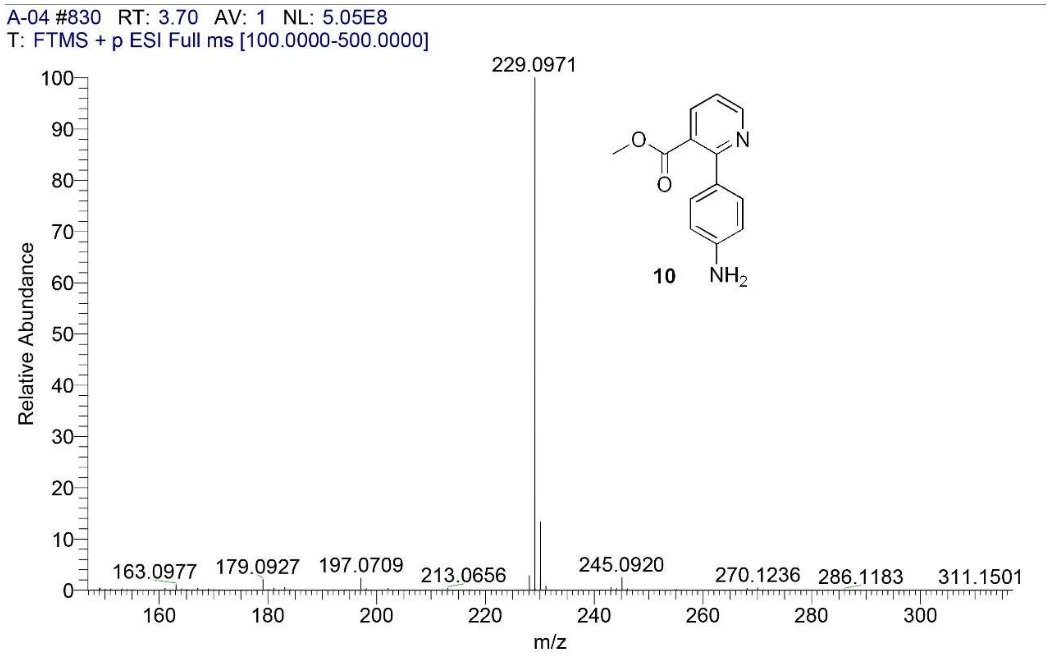

Figure S12: HR-MS (ESI/ion trap) spectrum of **10**

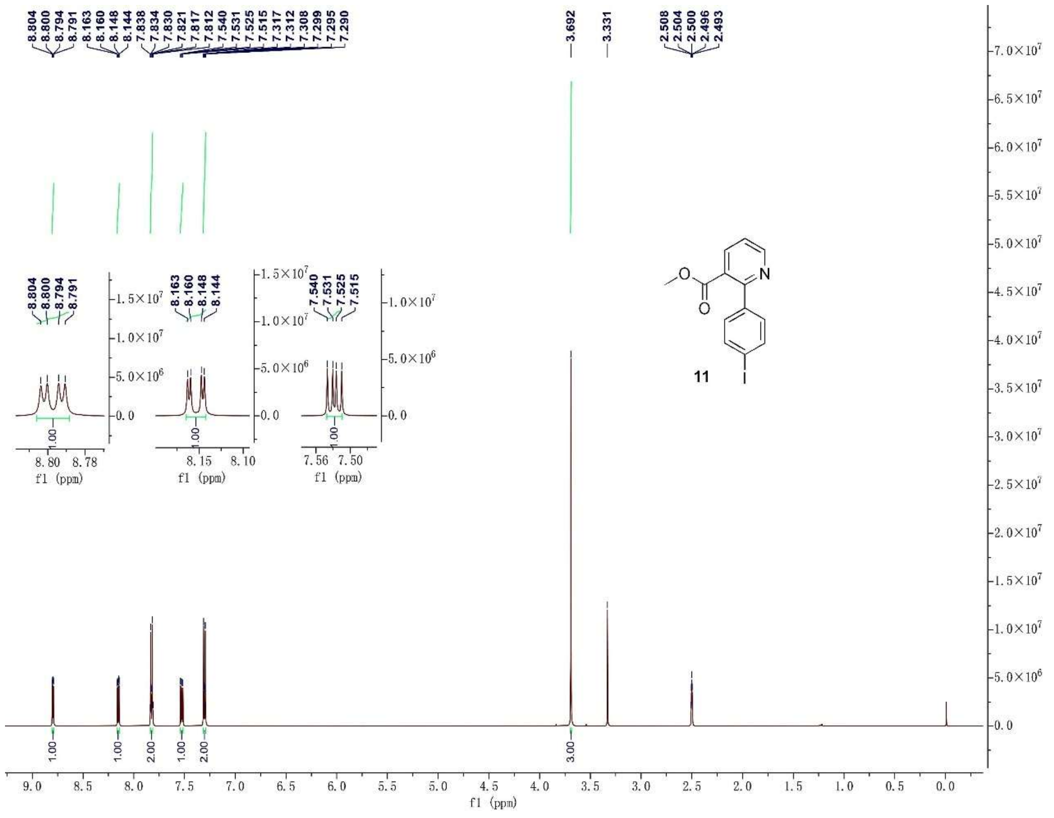

Figure S13: ^{1}H NMR spectrum of **11**

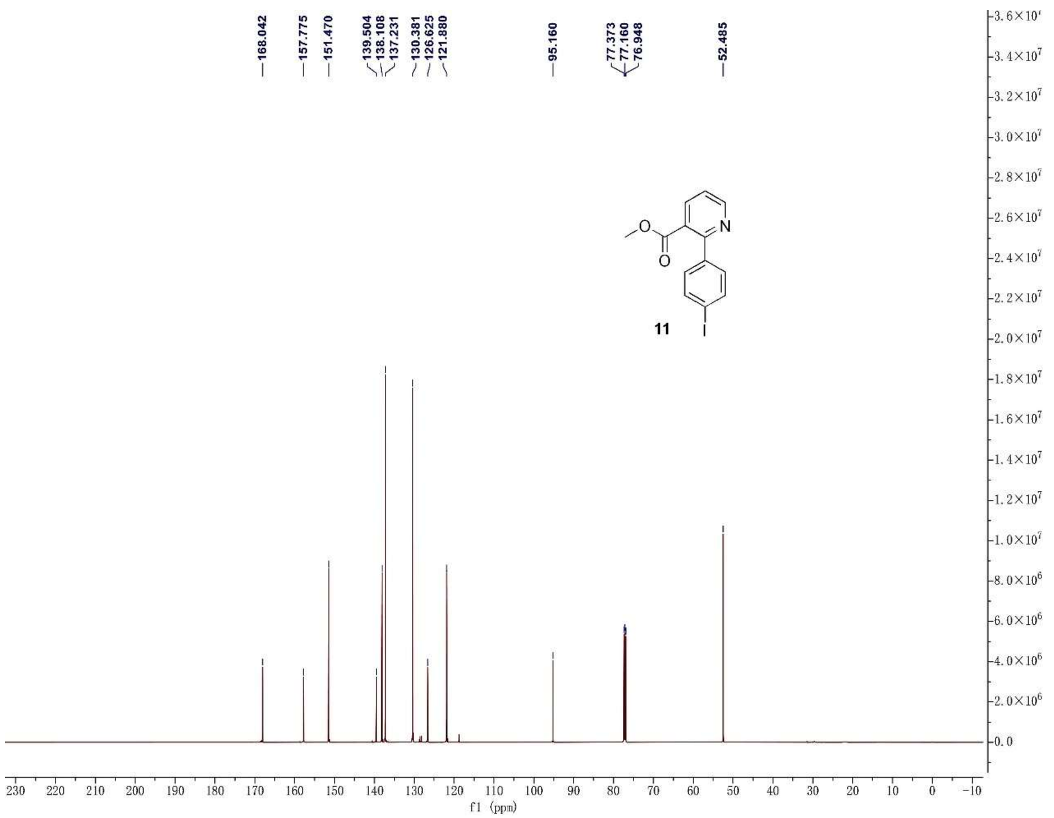

Figure S14: ^{13}C NMR spectrum of **11**

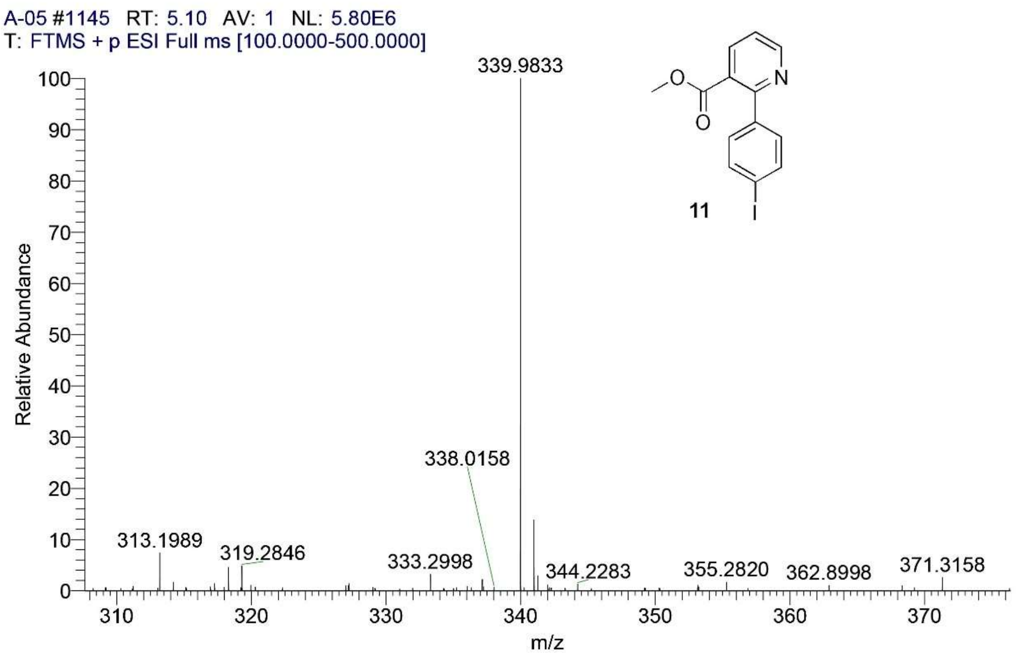

Figure S15: HR-MS (ESI/ion trap) spectrum of **11**

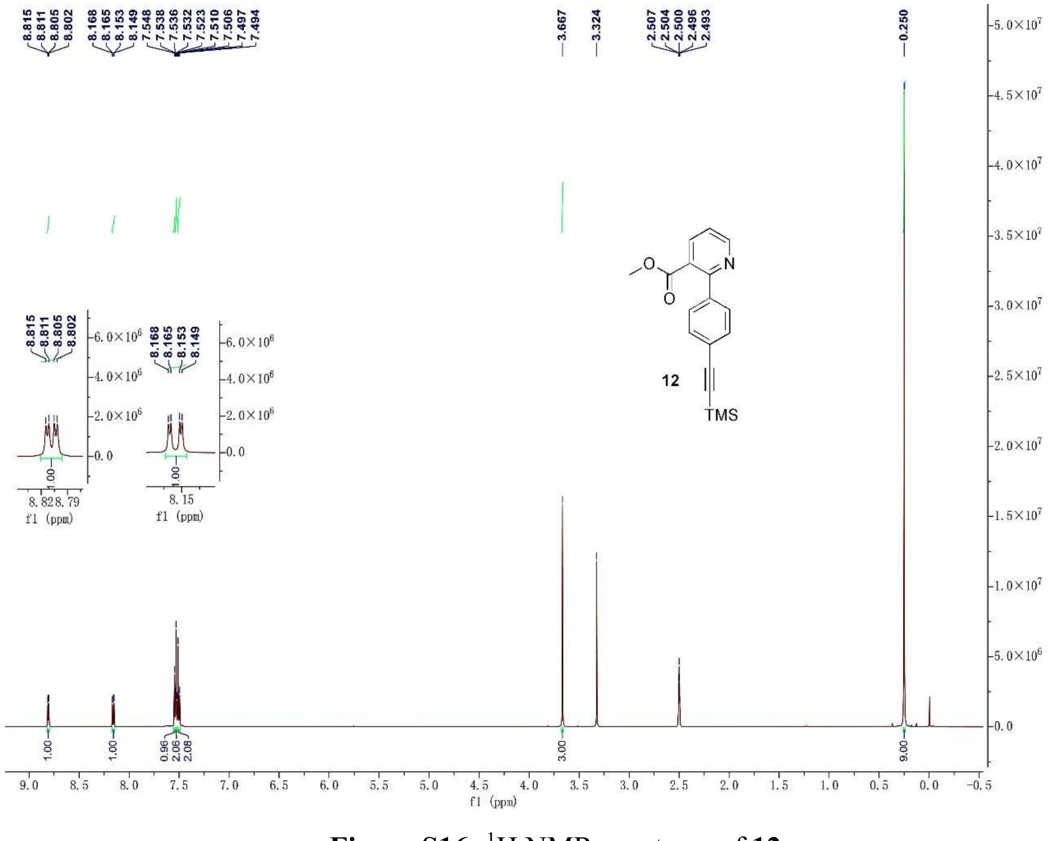

Figure S16: ^{1}H NMR spectrum of **12**

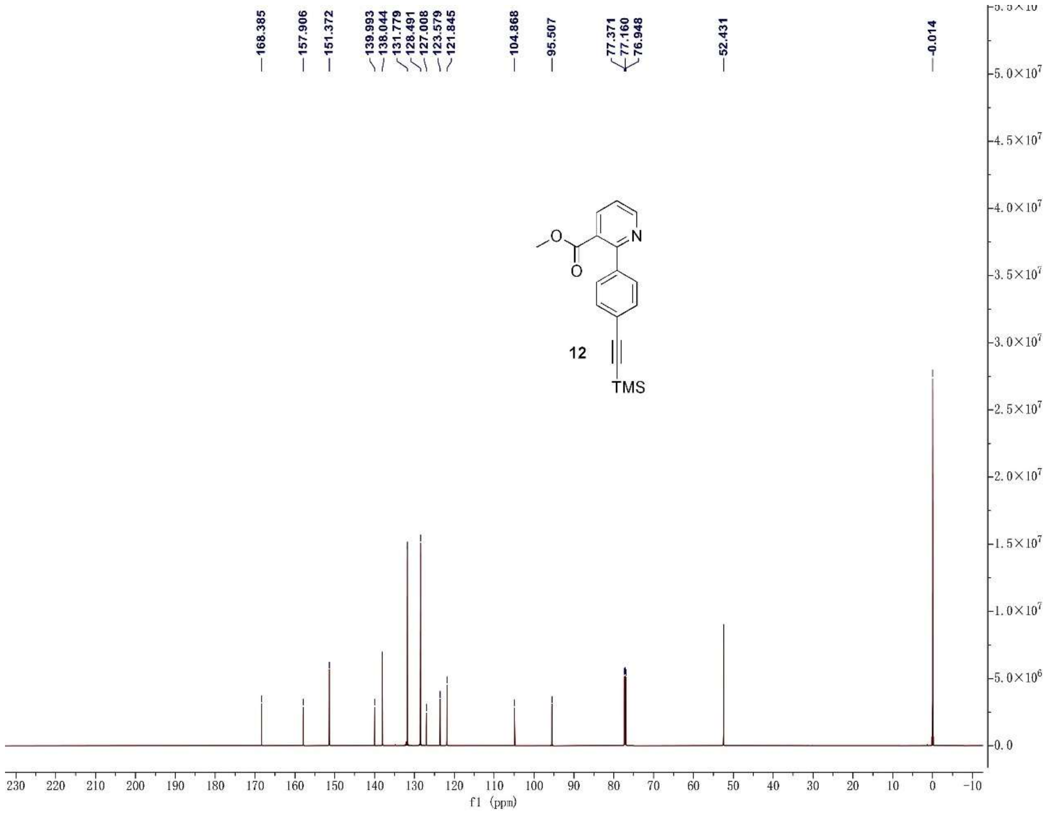

Figure S17: ^{13}C NMR spectrum of **12**

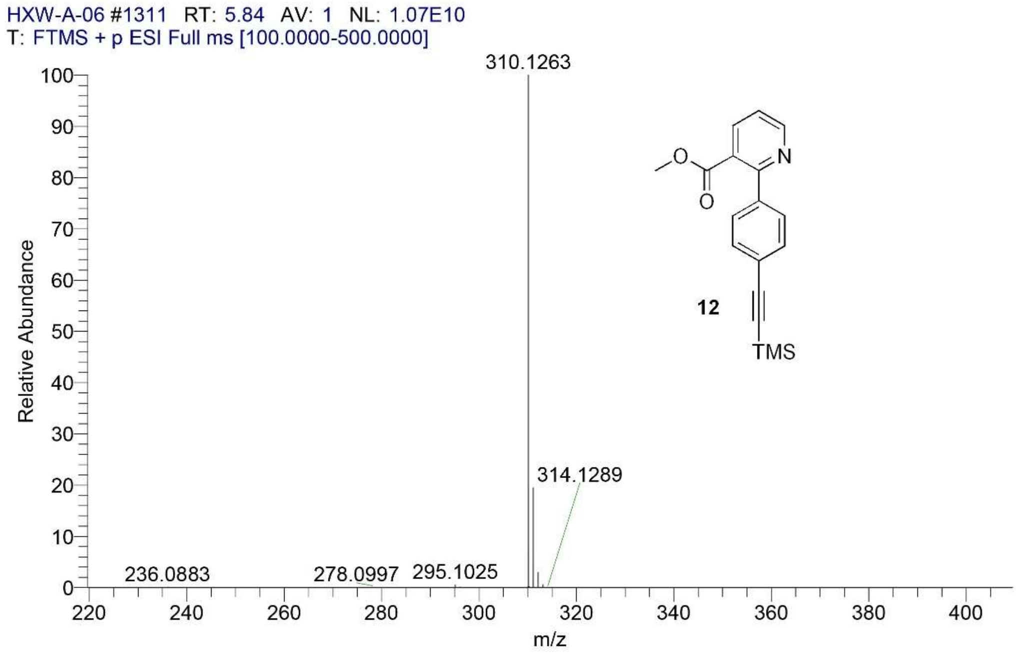

Figure S18: HR-MS (ESI/ion trap) spectrum of **12**

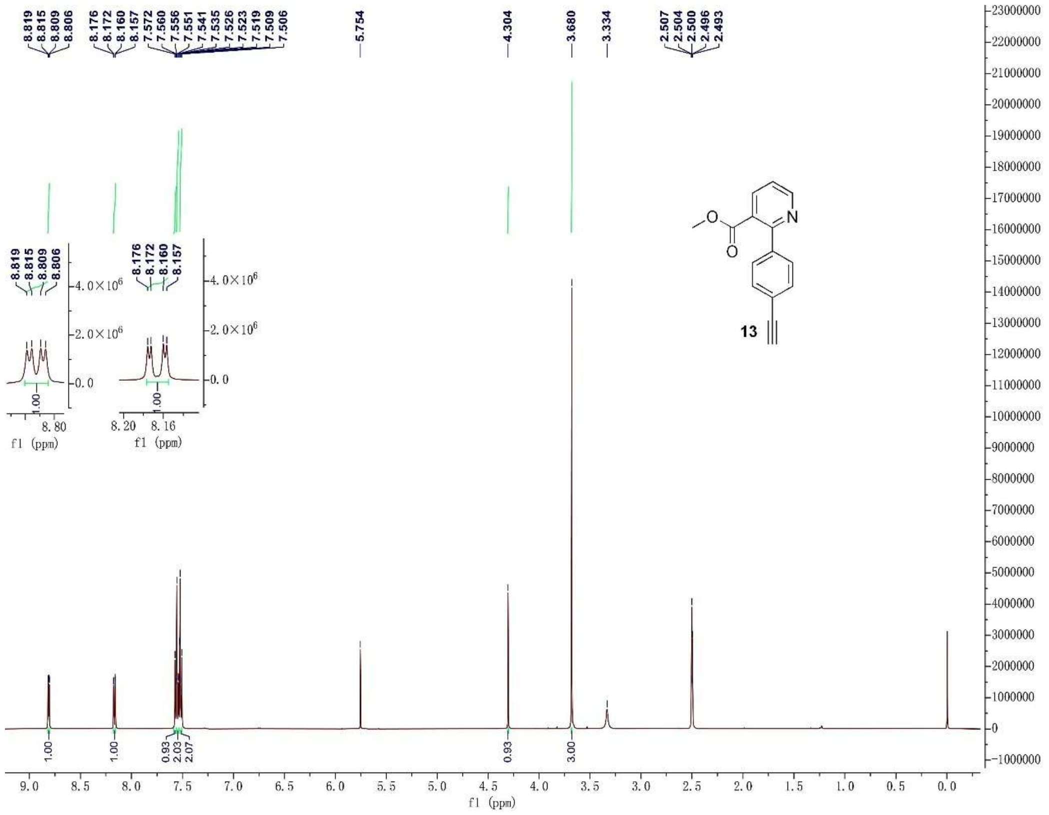

Figure S19: ^{1}H NMR spectrum of **13**

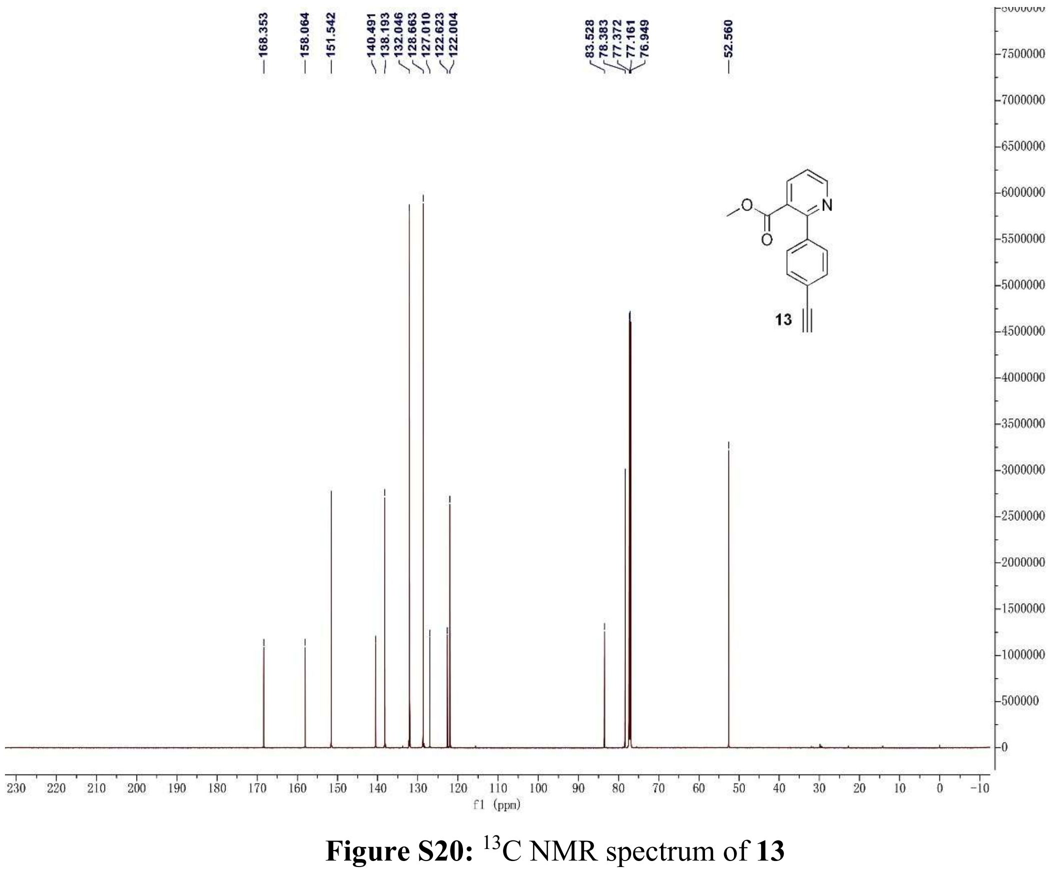

Figure S20: ^{13}C NMR spectrum of **13**

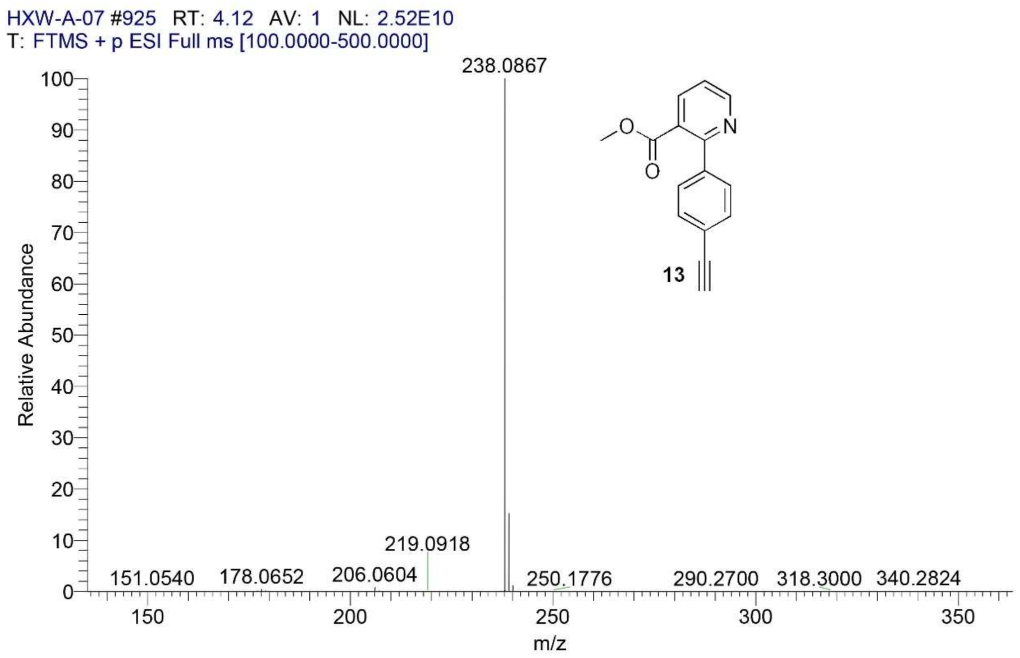

Figure S21: HR-MS (ESI/ion trap) spectrum of **13**

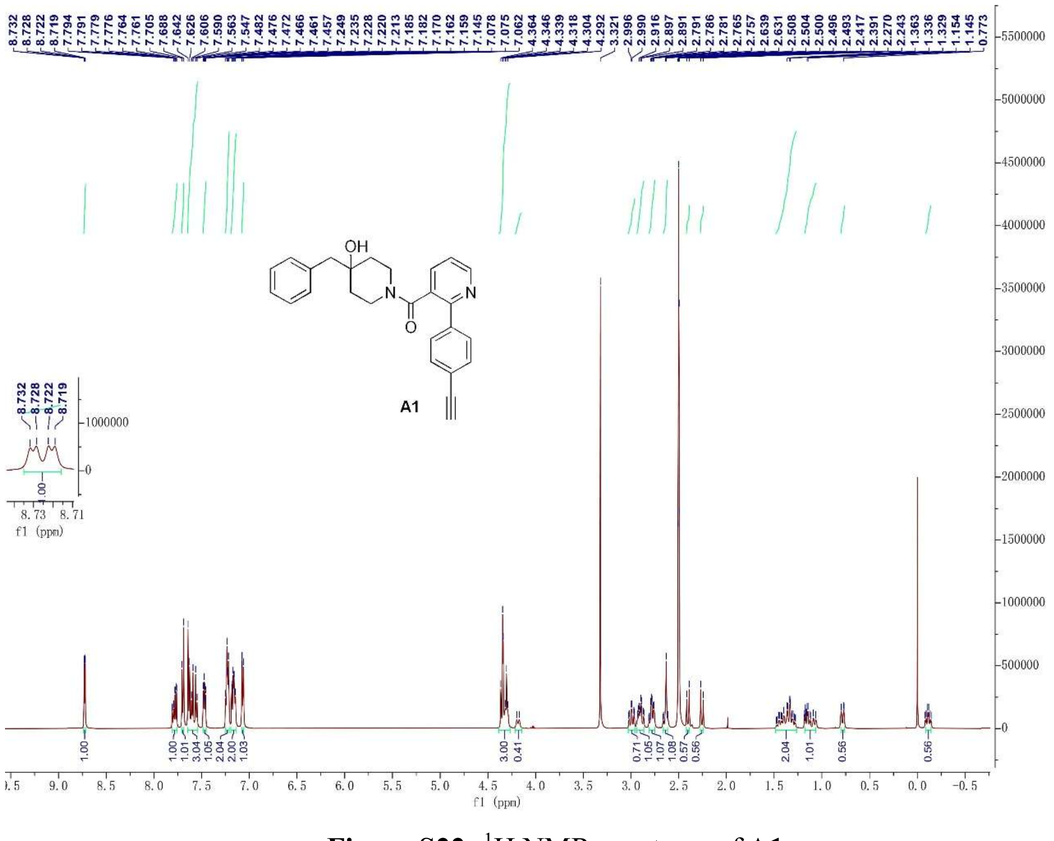

Figure S22: ^{1}H NMR spectrum of **A1**

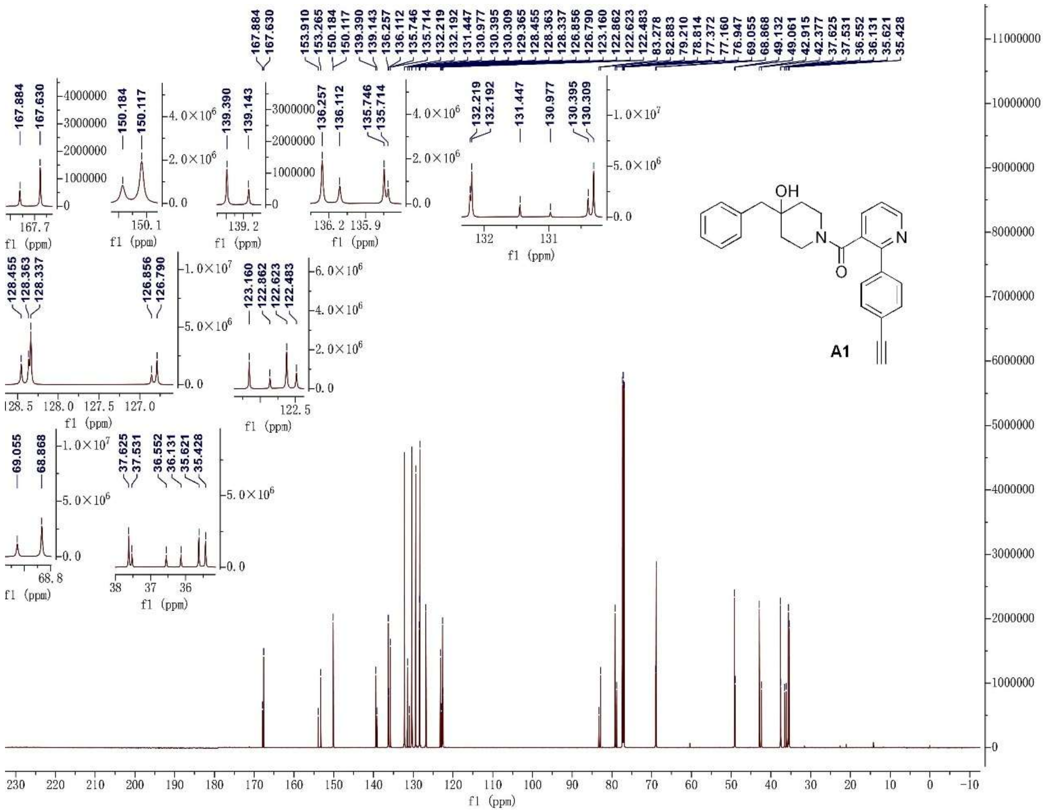

Figure S23: ^{13}C NMR spectrum of **A1**

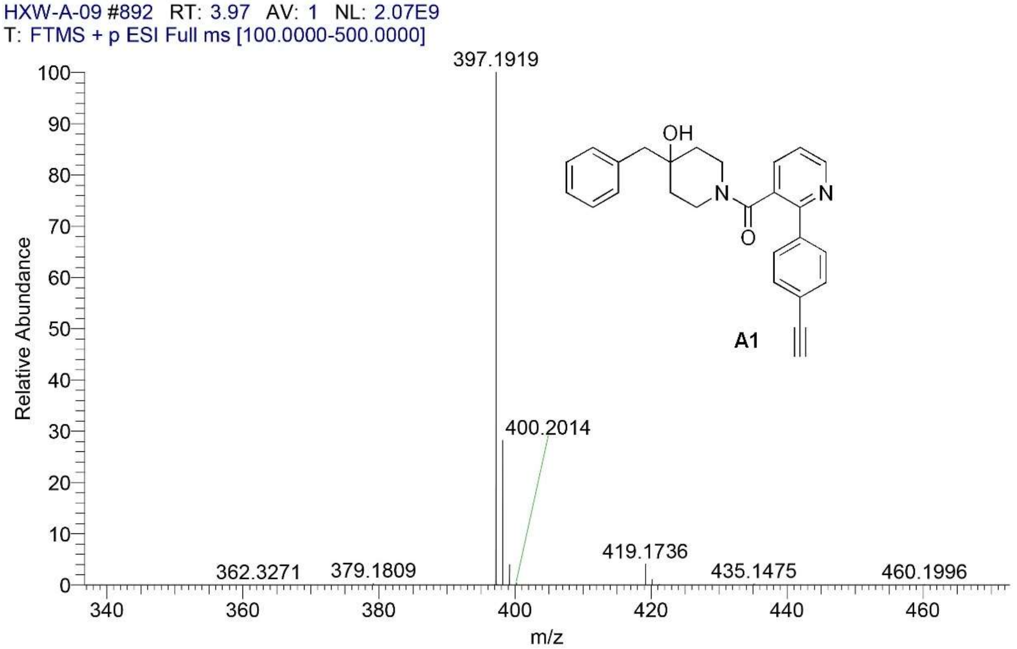

Figure S24: HR-MS (ESI/ion trap) spectrum of **A1**

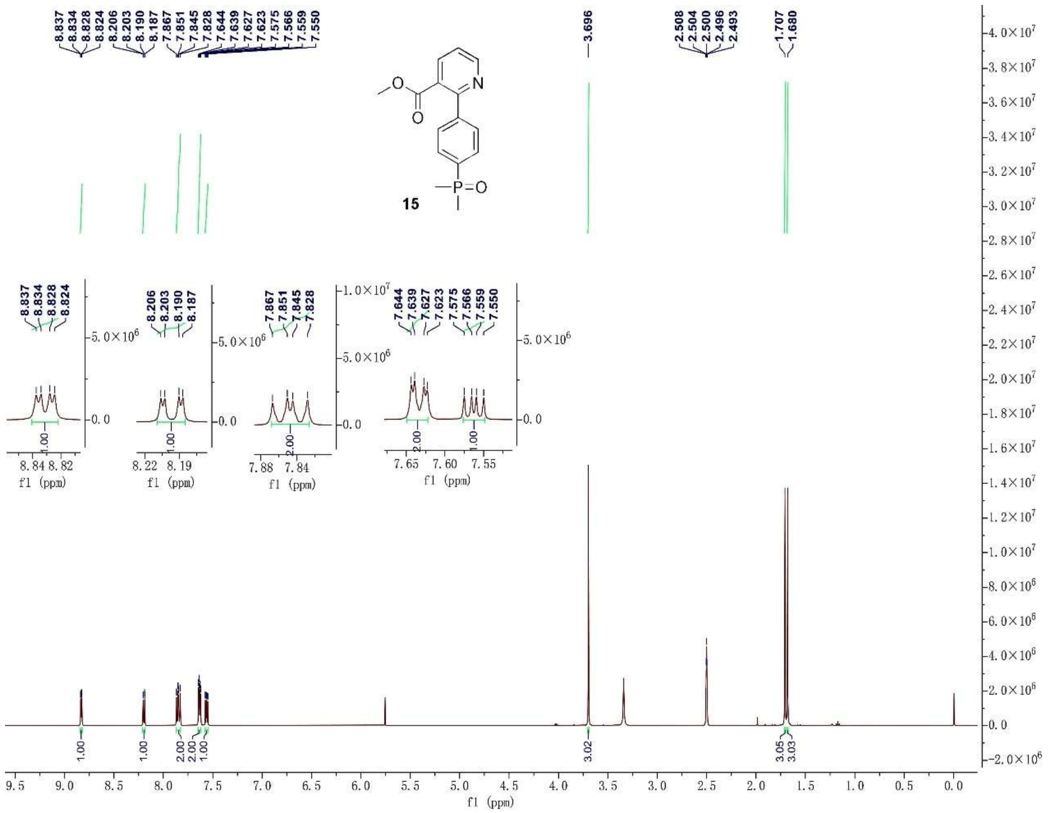

Figure S25: ^{1}H NMR spectrum of **15**

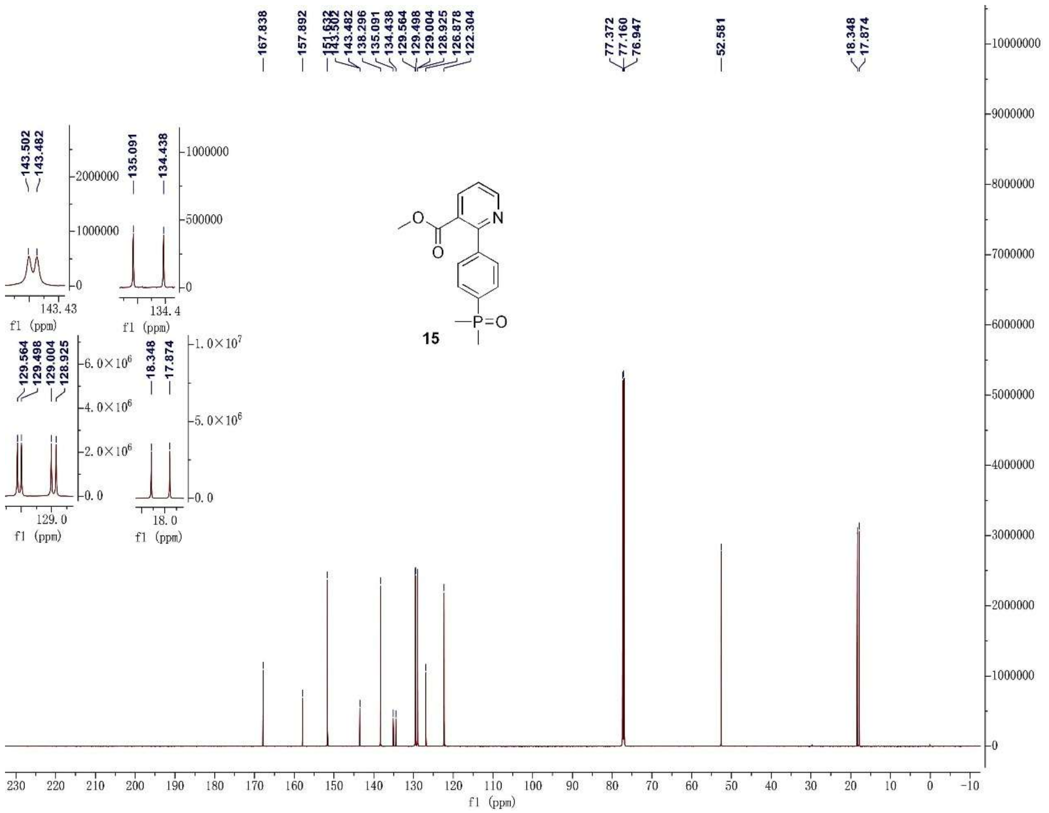

Figure S26: ^{13}C NMR spectrum of **15**

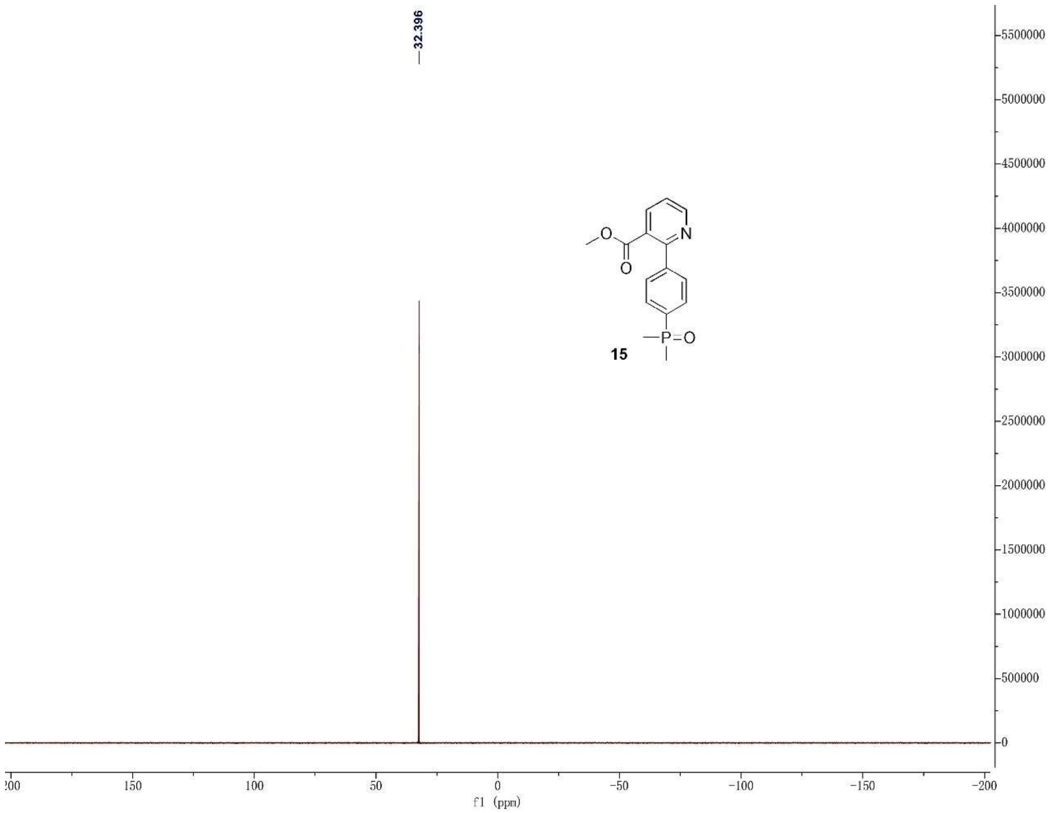

Figure S27: ^{31}P NMR spectrum of **15**

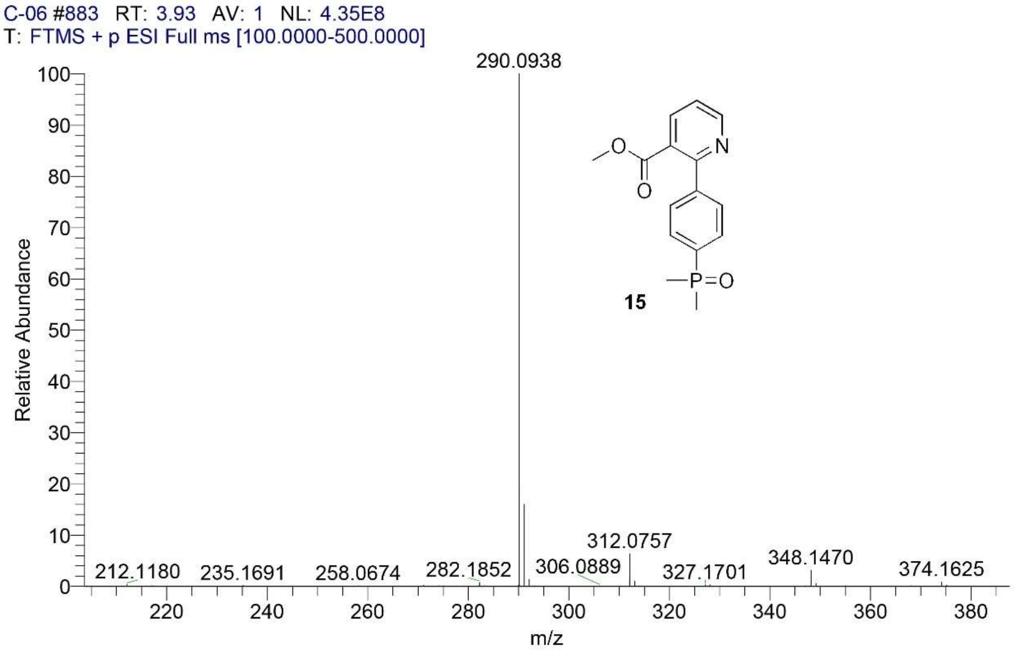

Figure S28: HR-MS (ESI/ion trap) spectrum of **15**

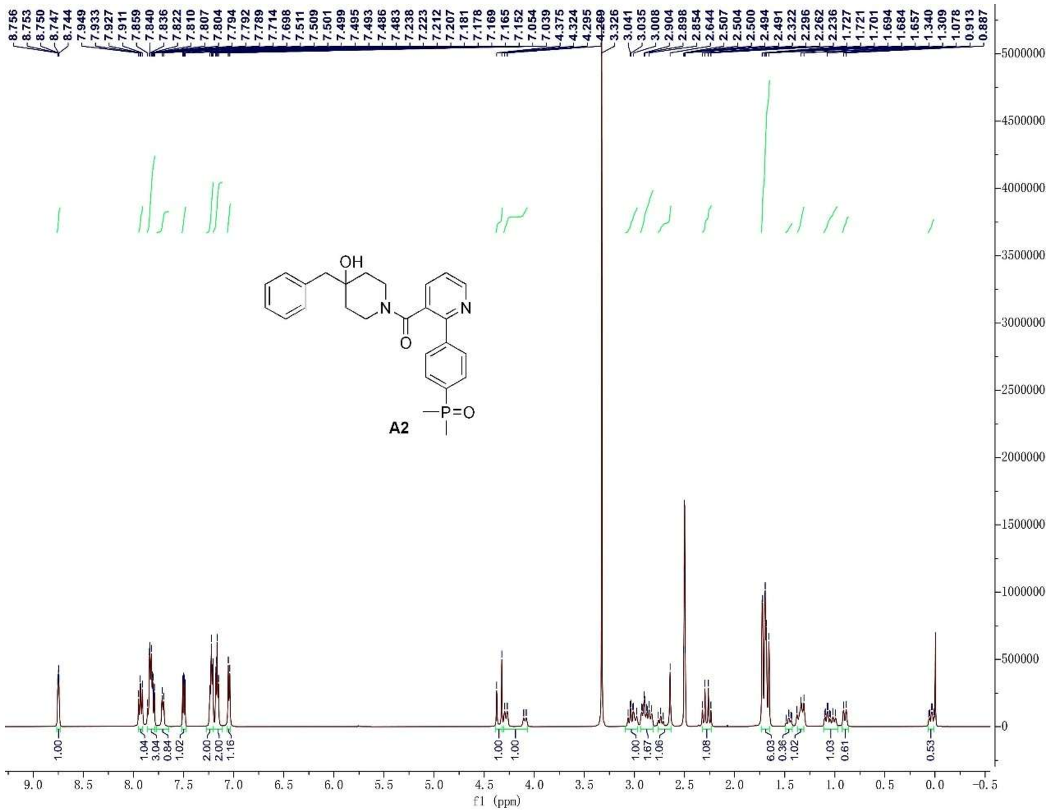

Figure S29: ^{1}H NMR spectrum of **A2**

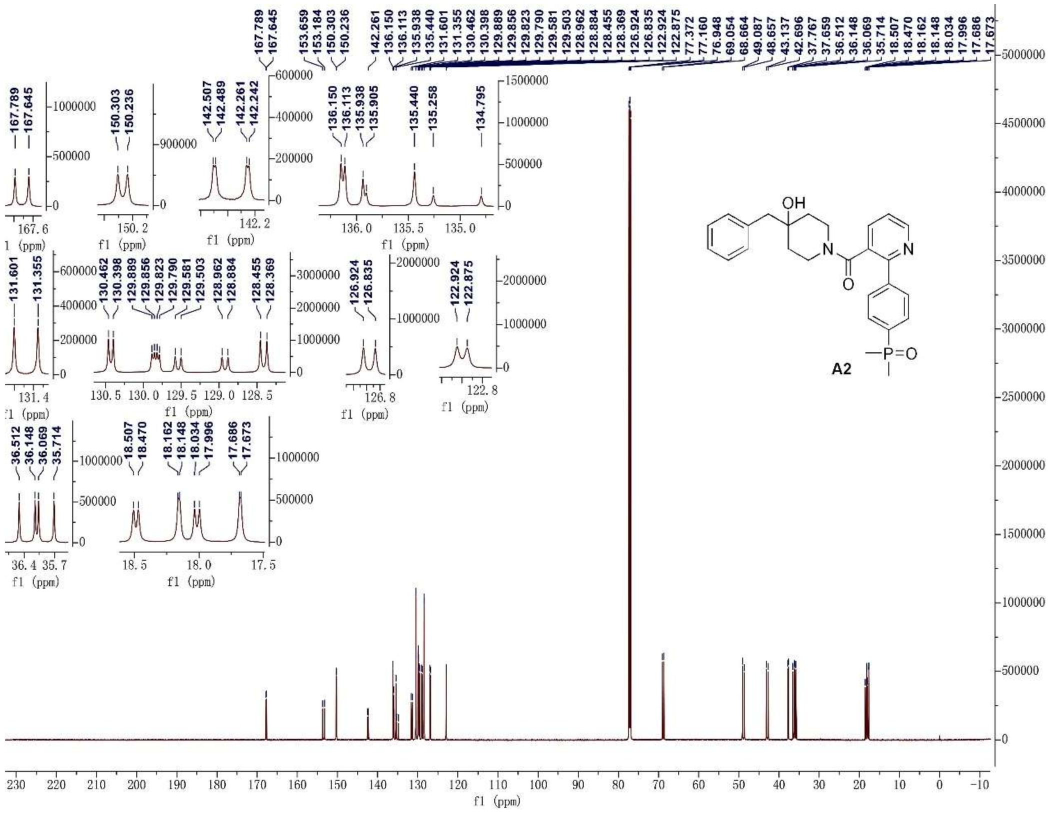

Figure S30: ^{13}C NMR spectrum of **A2**

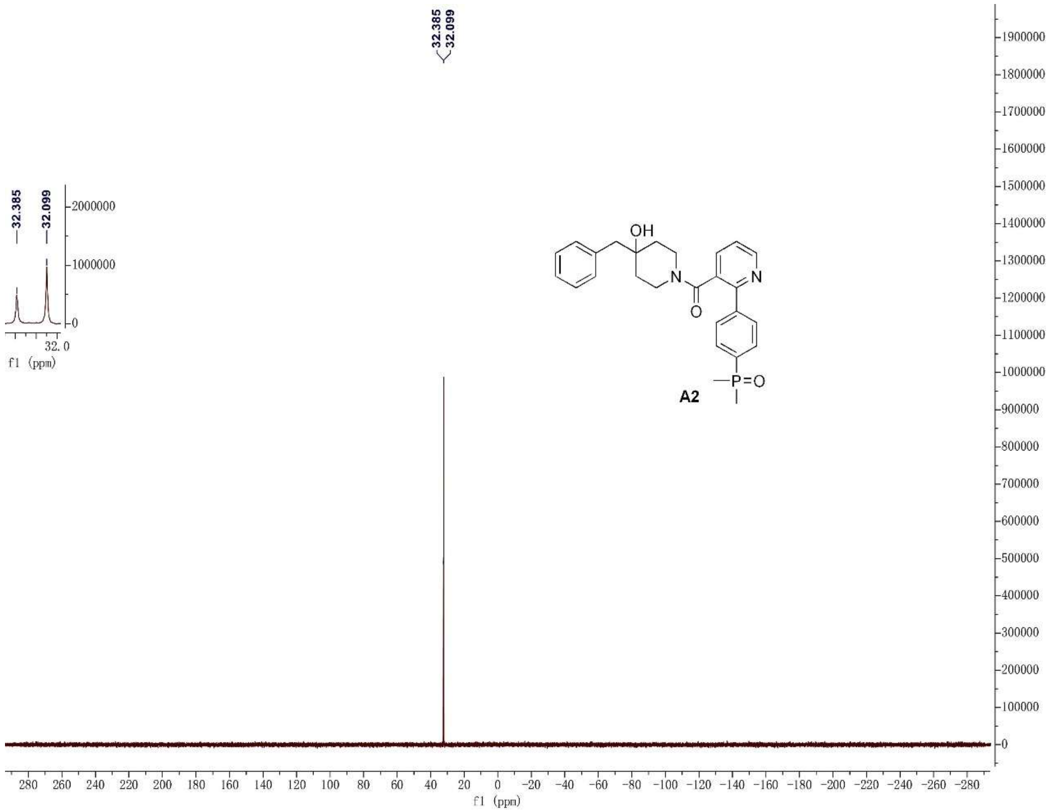

Figure S31: ^{31}P NMR spectrum of **A2**

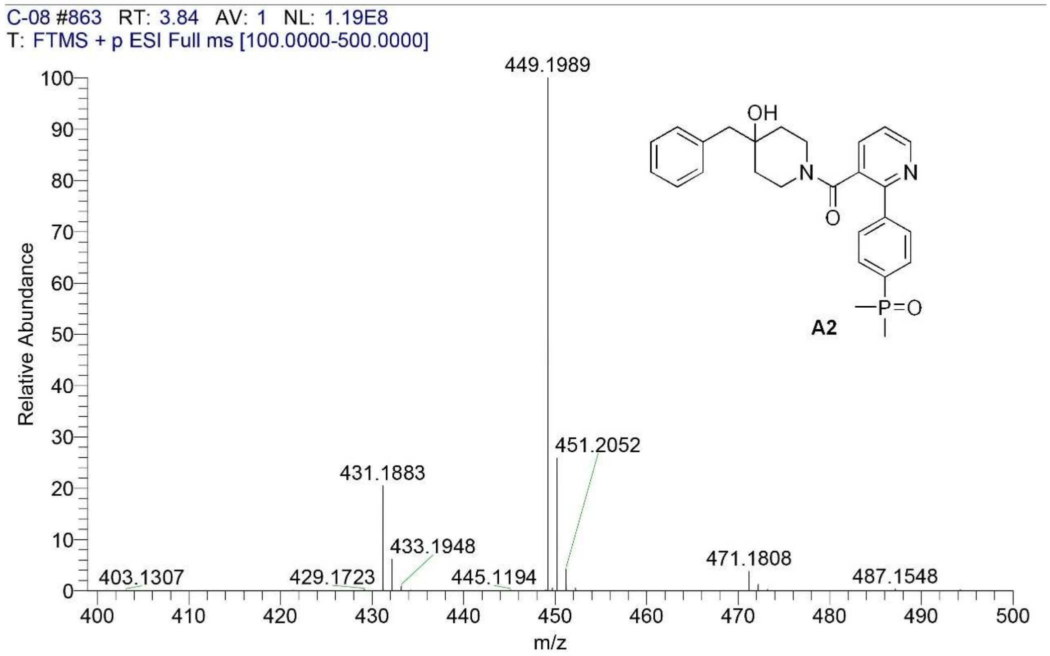

Figure S32: HR-MS (ESI/ion trap) spectrum of **A2**

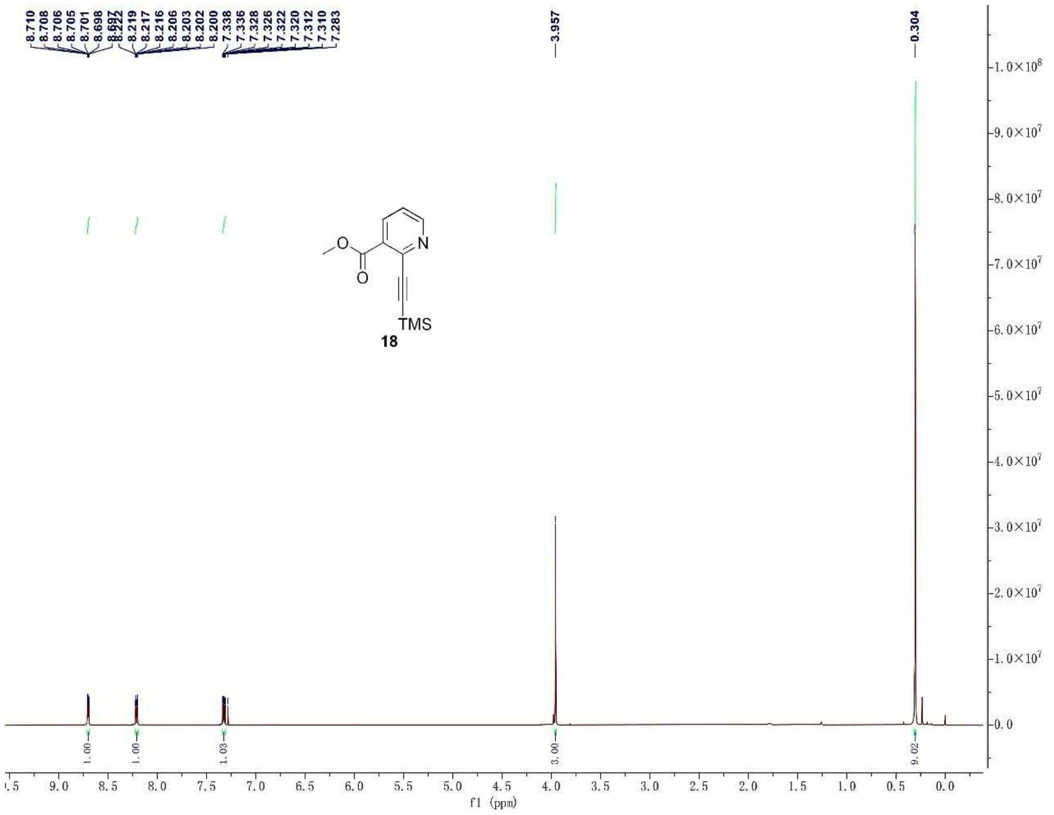

Figure S33: ^{1}H NMR spectrum of **18**

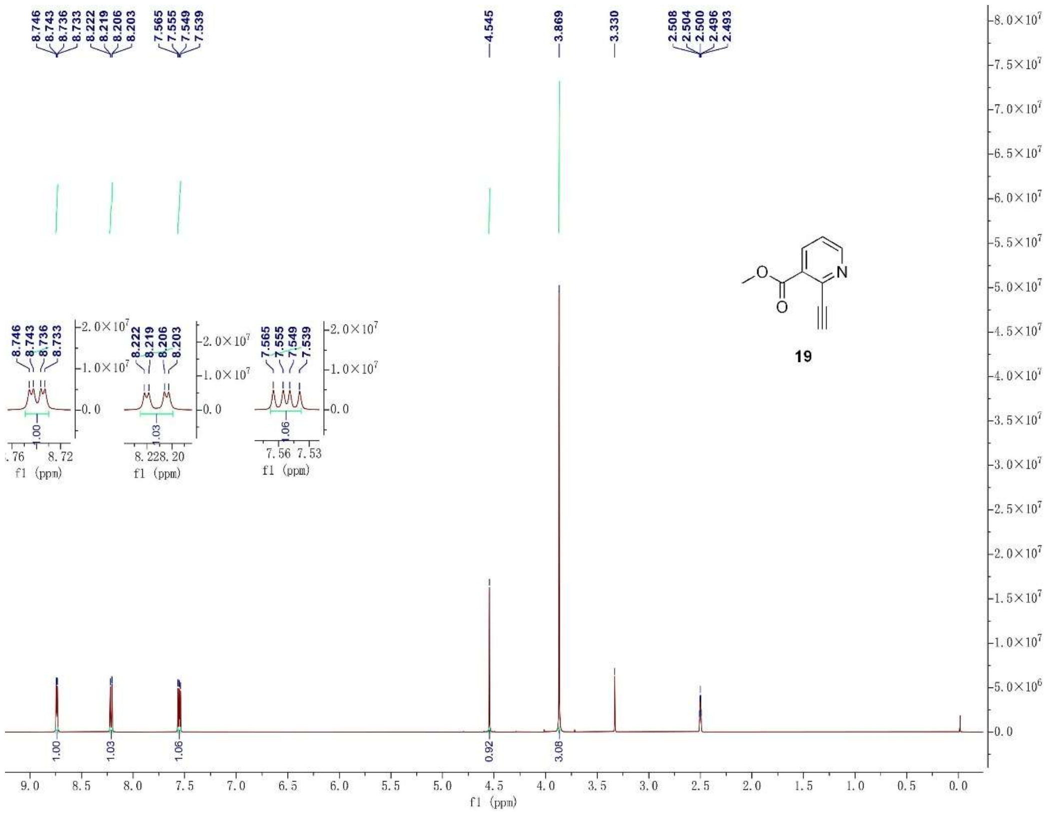

Figure S34: ^{1}H NMR spectrum of **19**

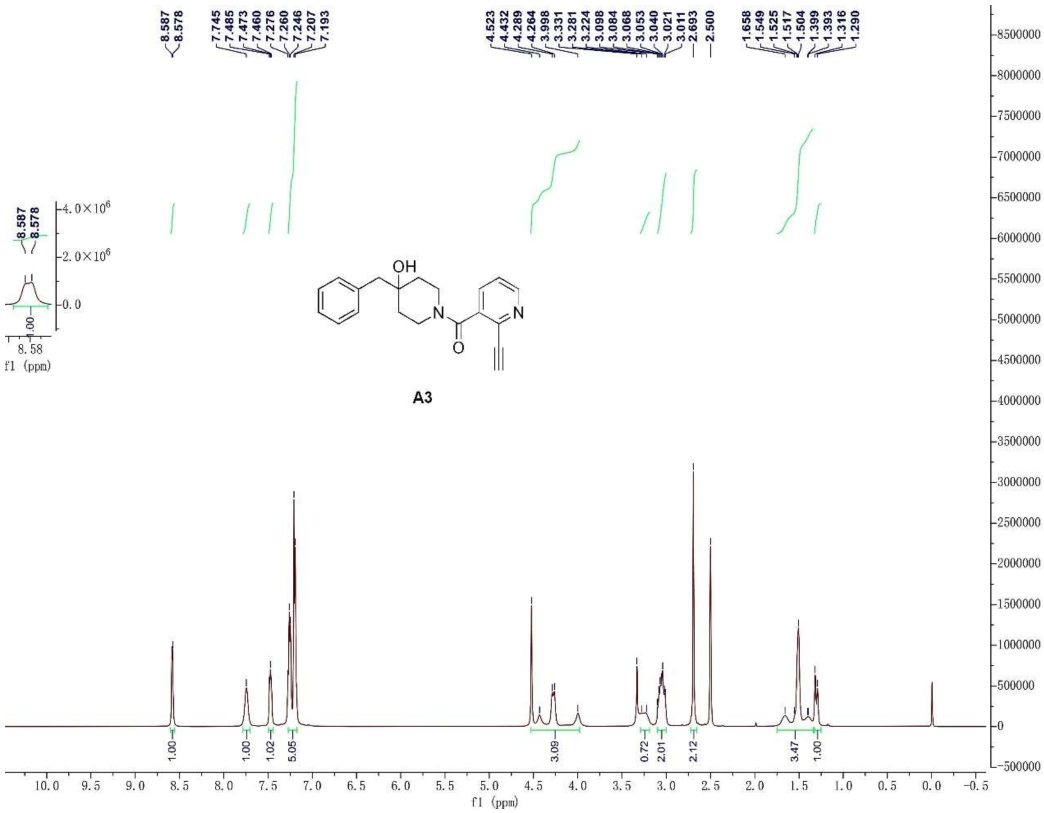

Figure S35: ^{1}H NMR spectrum of **A3**

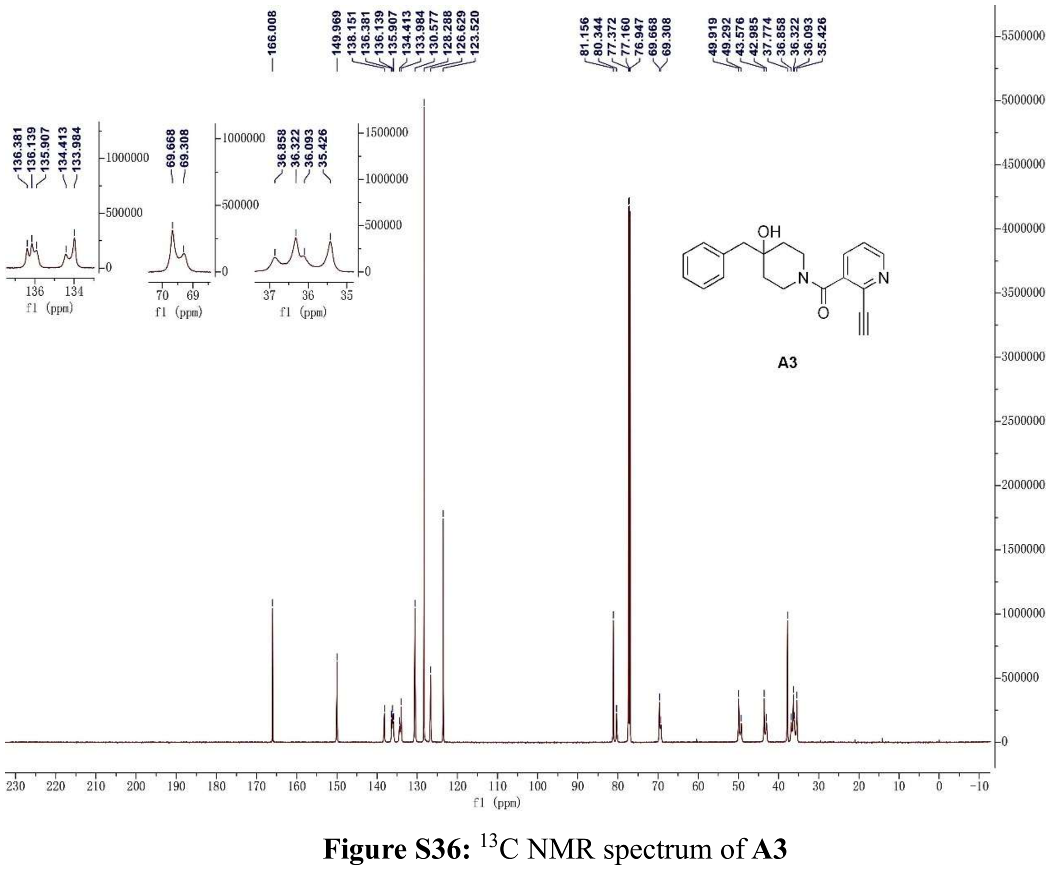

Figure S36: ^{13}C NMR spectrum of **A3**

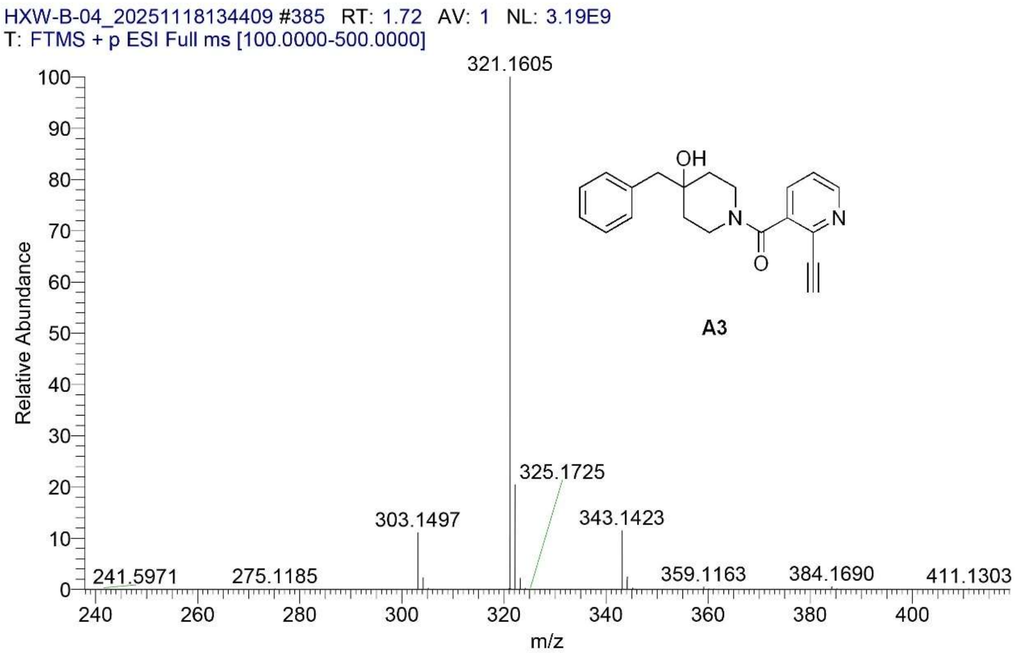

Figure S37: HR-MS (ESI/ion trap) spectrum of **A3**

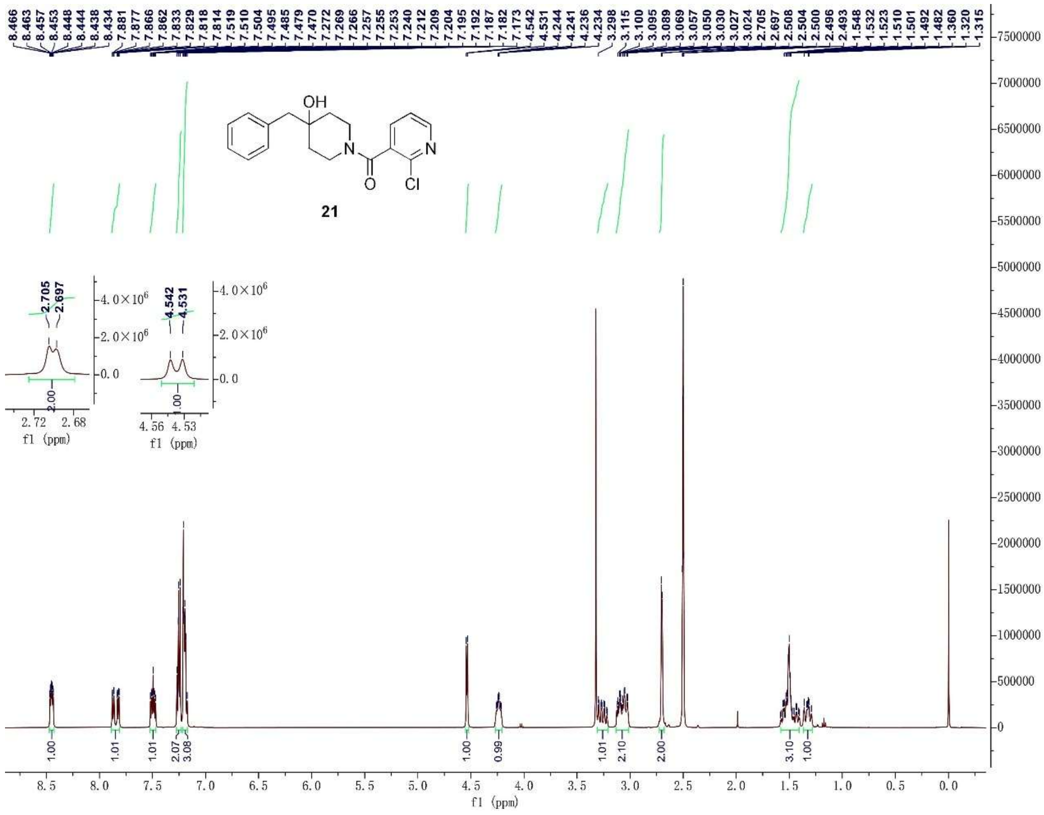

Figure S38: ^{1}H NMR spectrum of **21**

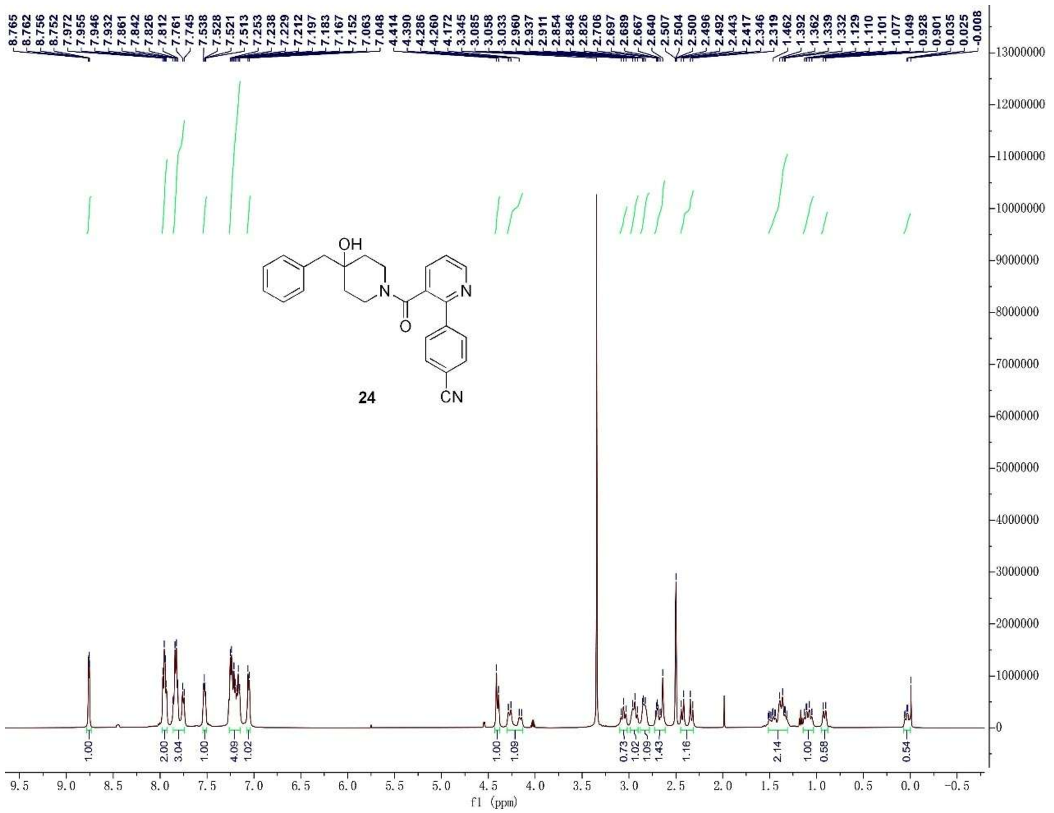

Figure S39: ^{1}H NMR spectrum of **24**

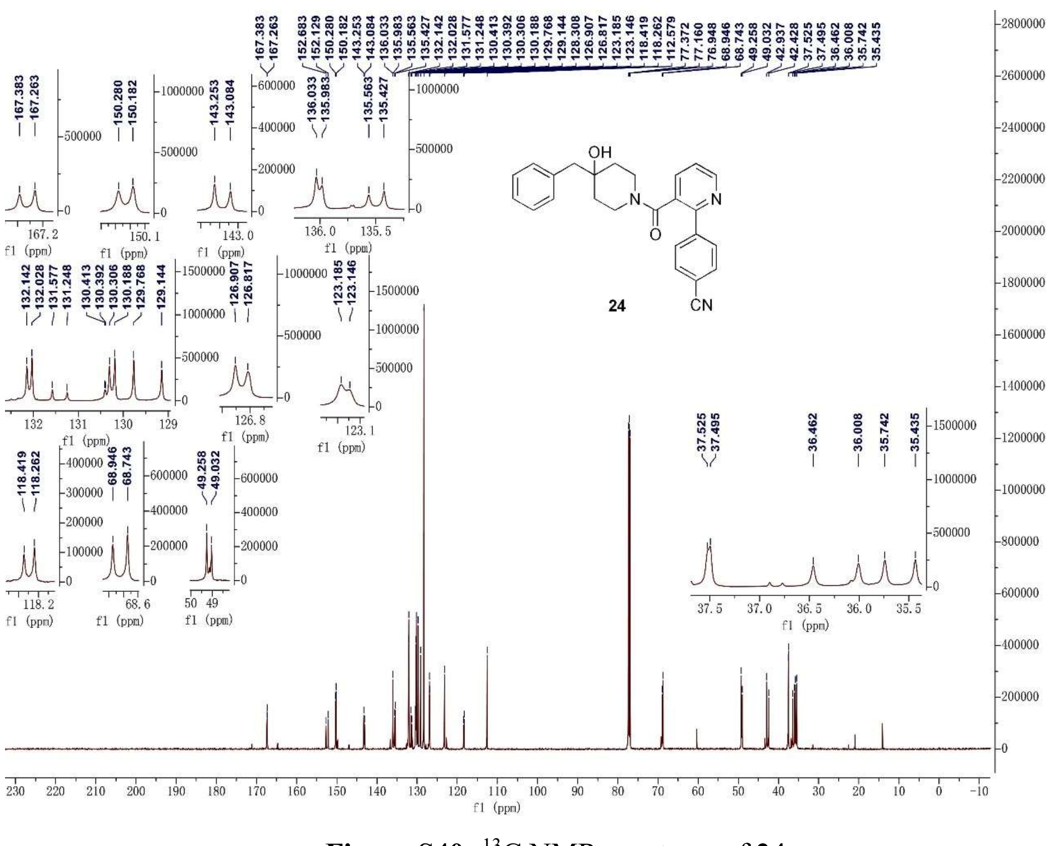

Figure S40: ^{13}C NMR spectrum of **24**

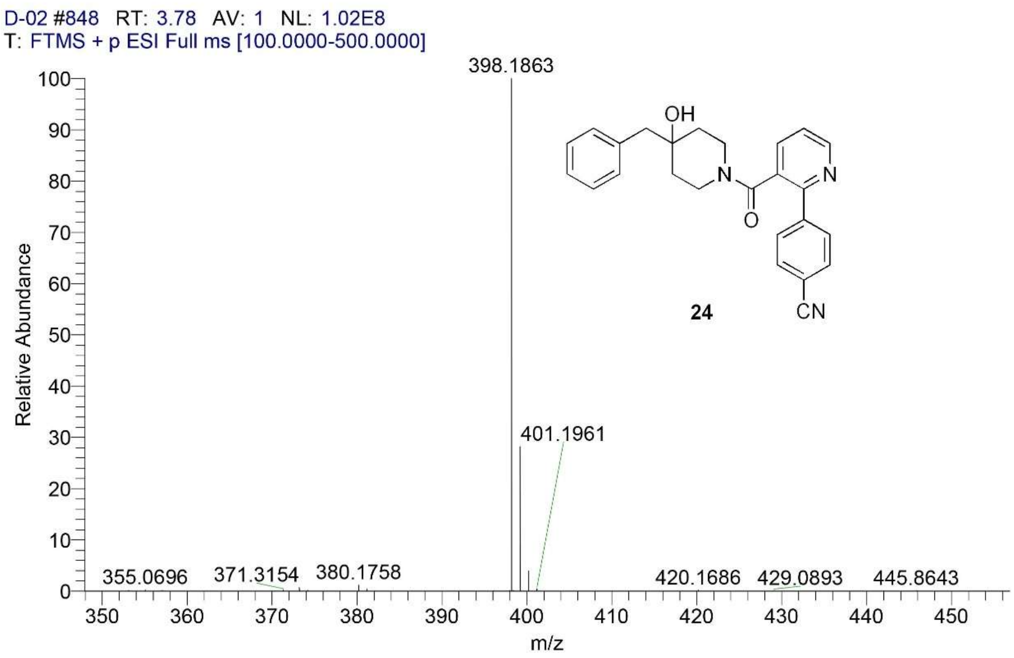

Figure S41: HR-MS (ESI/ion trap) spectrum of **24**

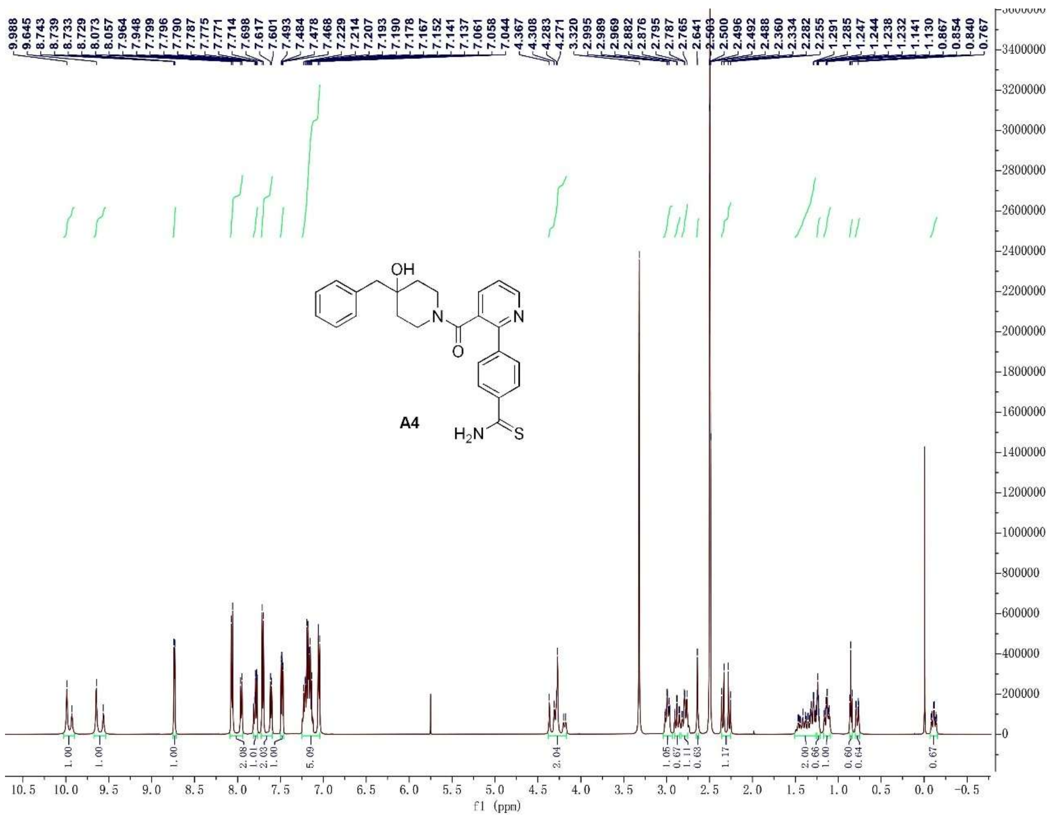

Figure S42: ^{1}H NMR spectrum of **A4**

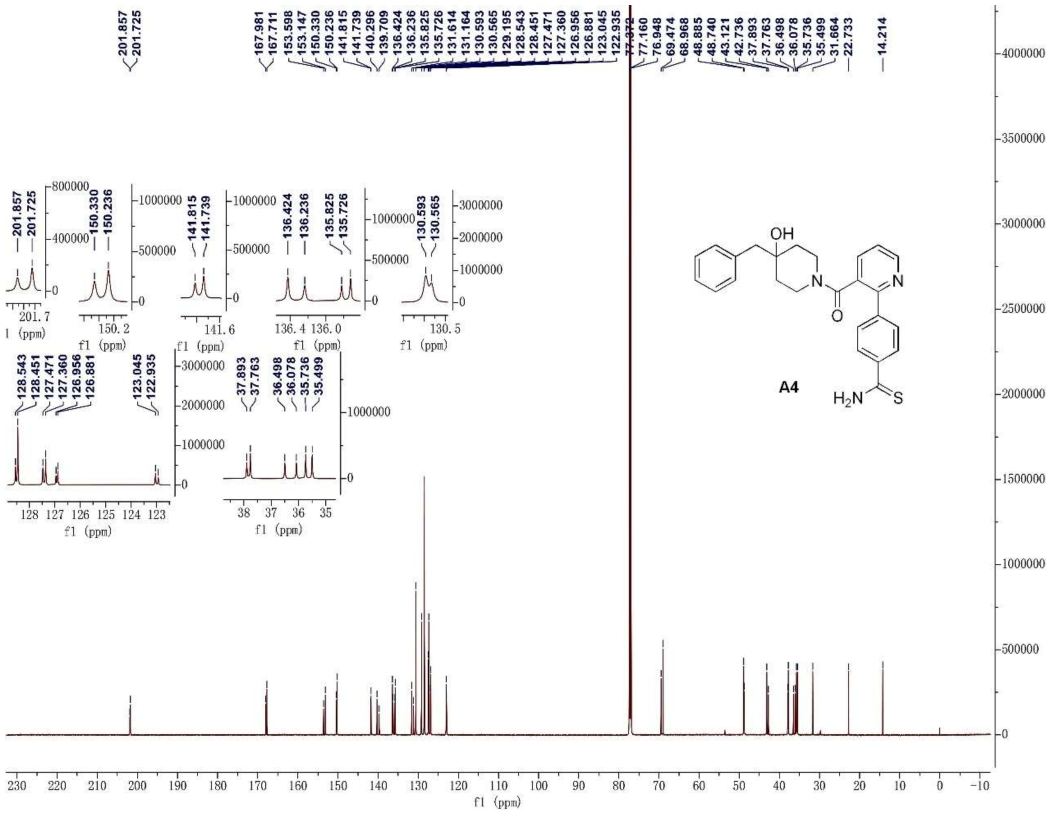

Figure S43: ^{13}C NMR spectrum of **A4**

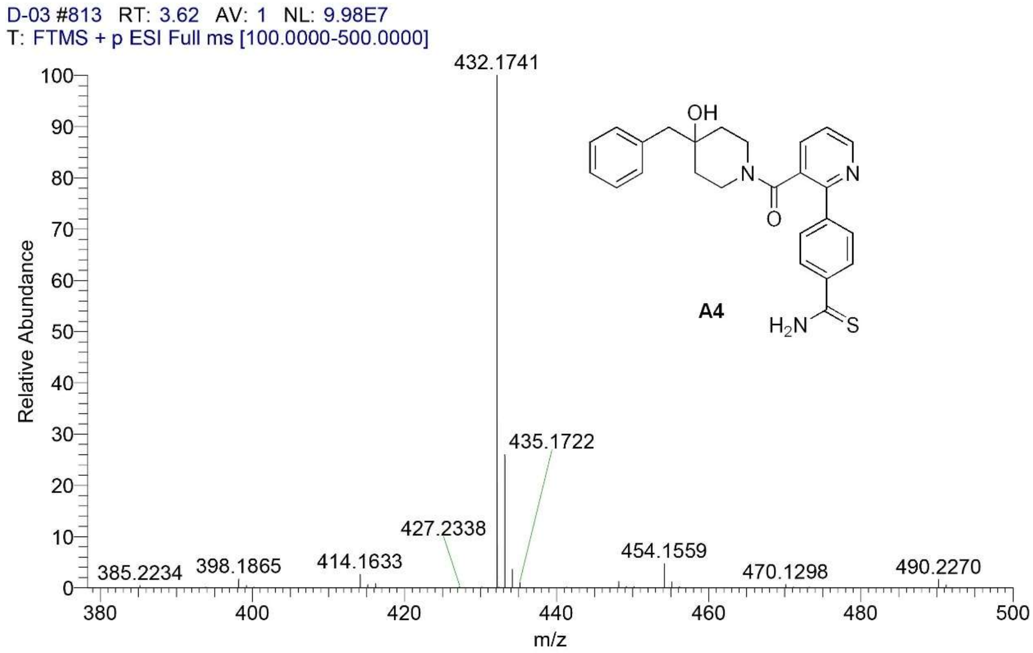

Figure S44: HR-MS (ESI/ion trap) spectrum of **A4**

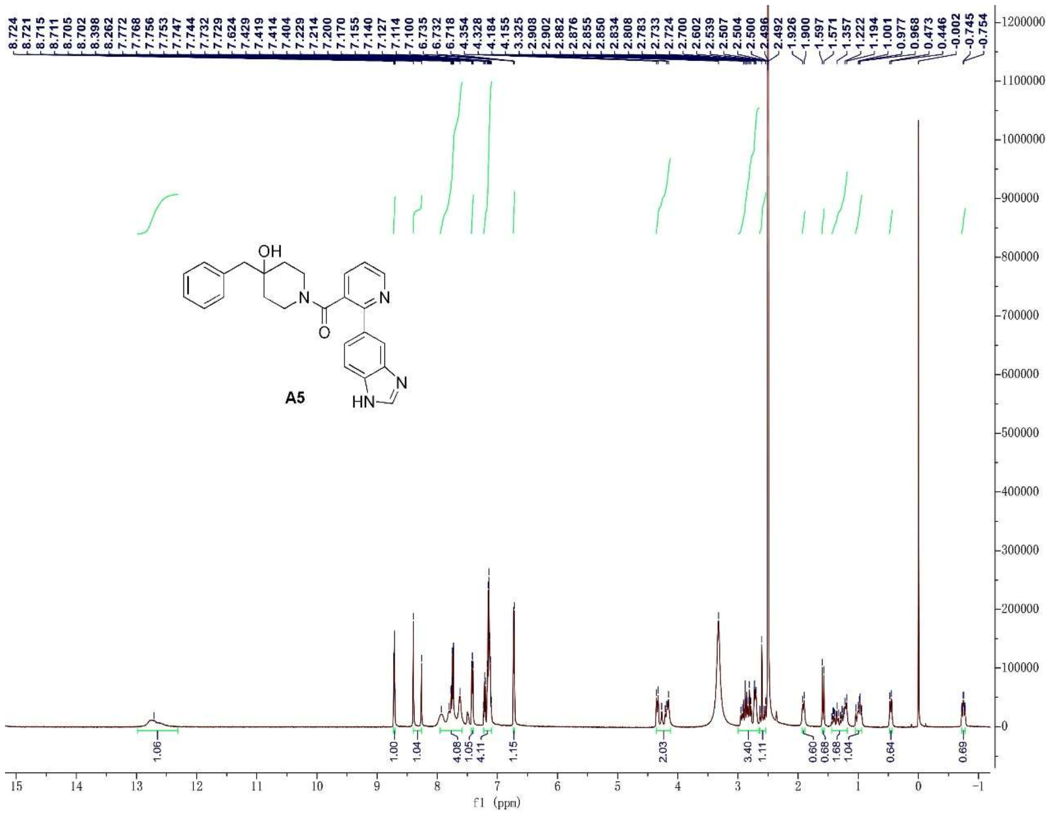

Figure S45: ^{1}H NMR spectrum of **A5**

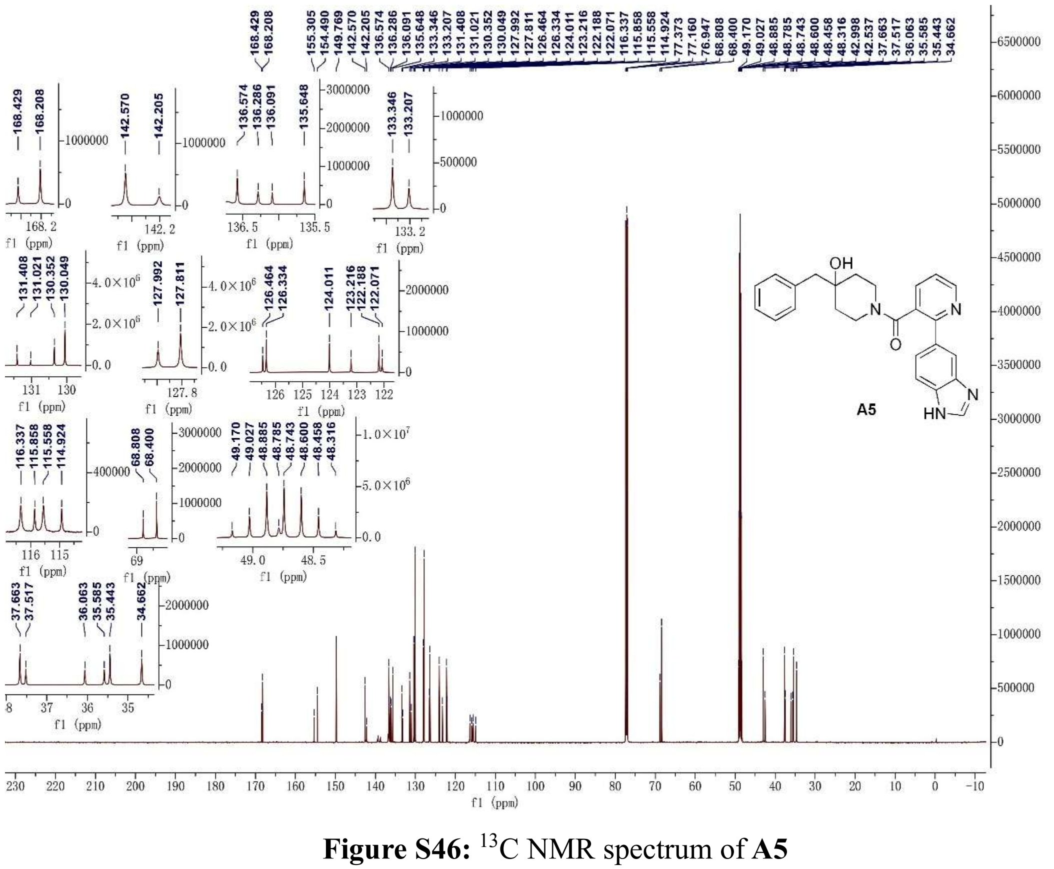

Figure S46: ^{13}C NMR spectrum of **A5**

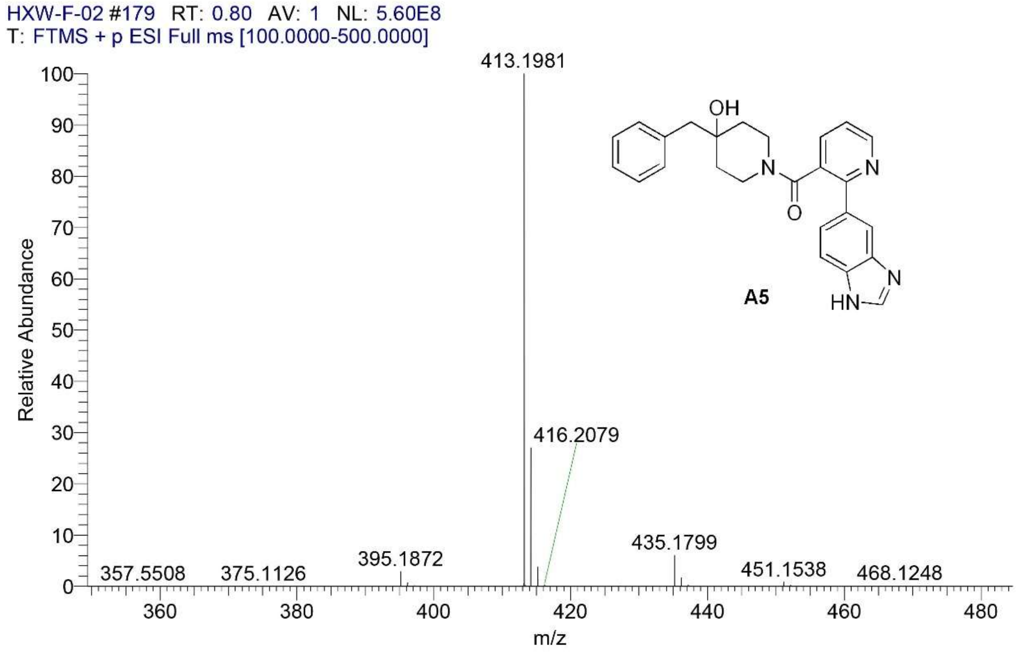

Figure S47: HR-MS (ESI/ion trap) spectrum of **A5**

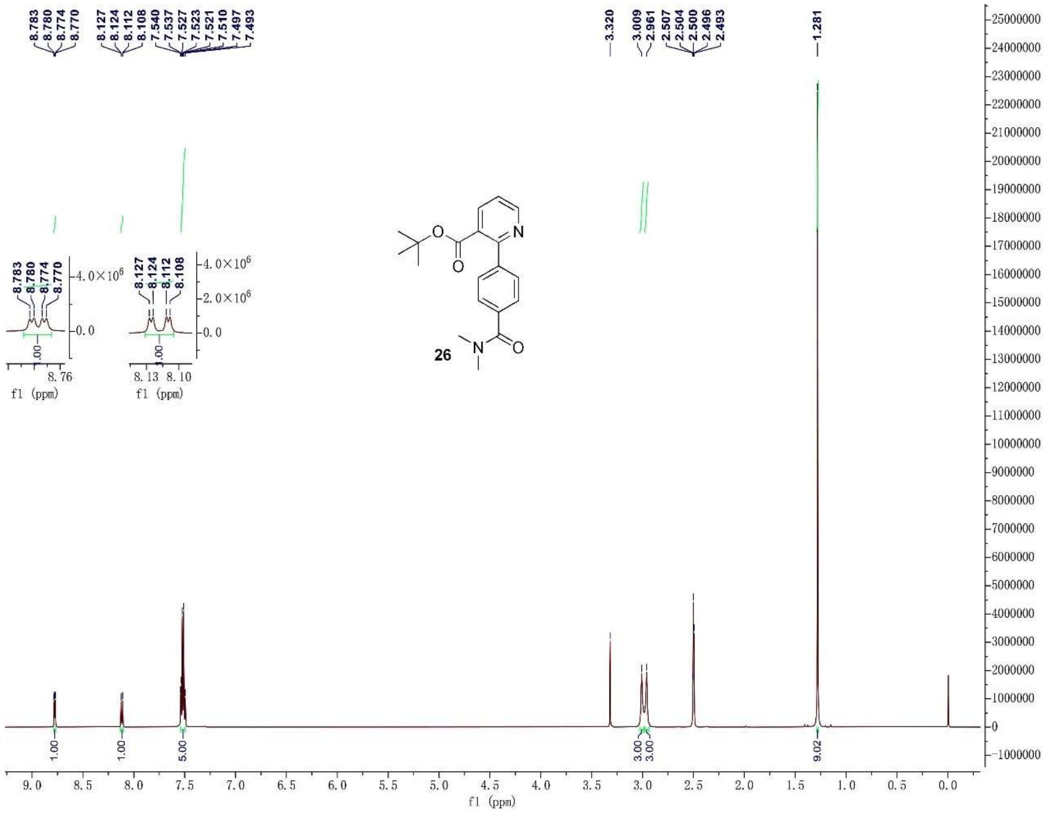

Figure S48: ^{1}H NMR spectrum of **26**

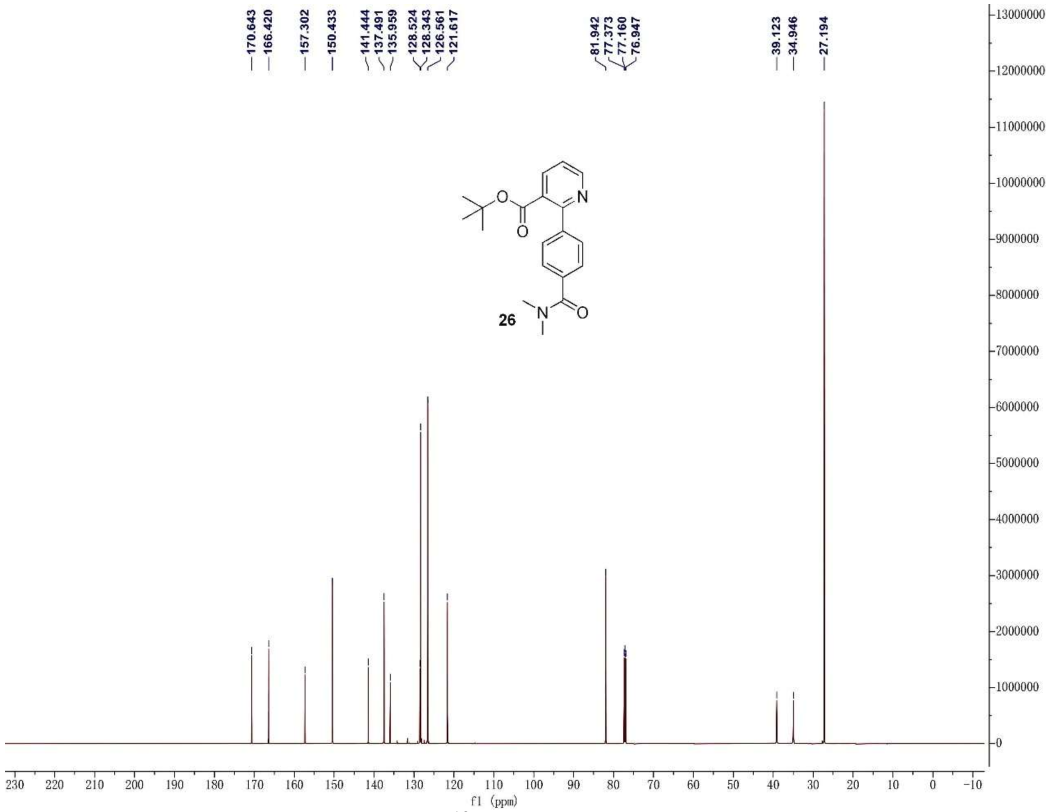

Figure S49: ^{13}C NMR spectrum of **26**

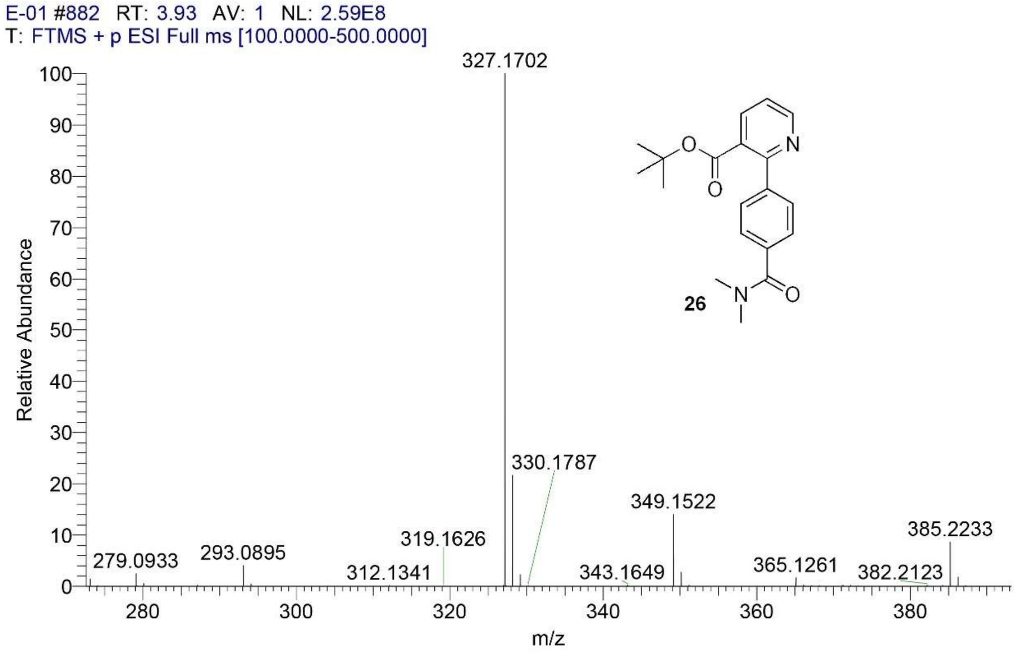

Figure S50: HR-MS (ESI/ion trap) spectrum of **26**

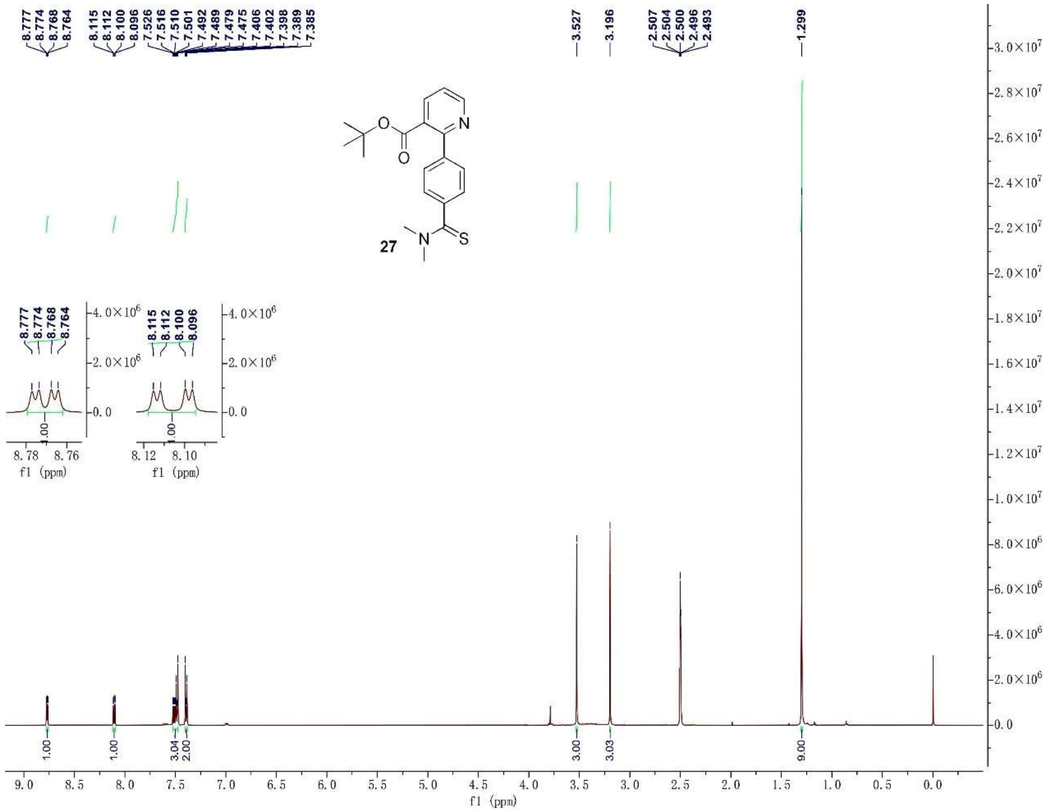

Figure S51: ^{1}H NMR spectrum of **27**

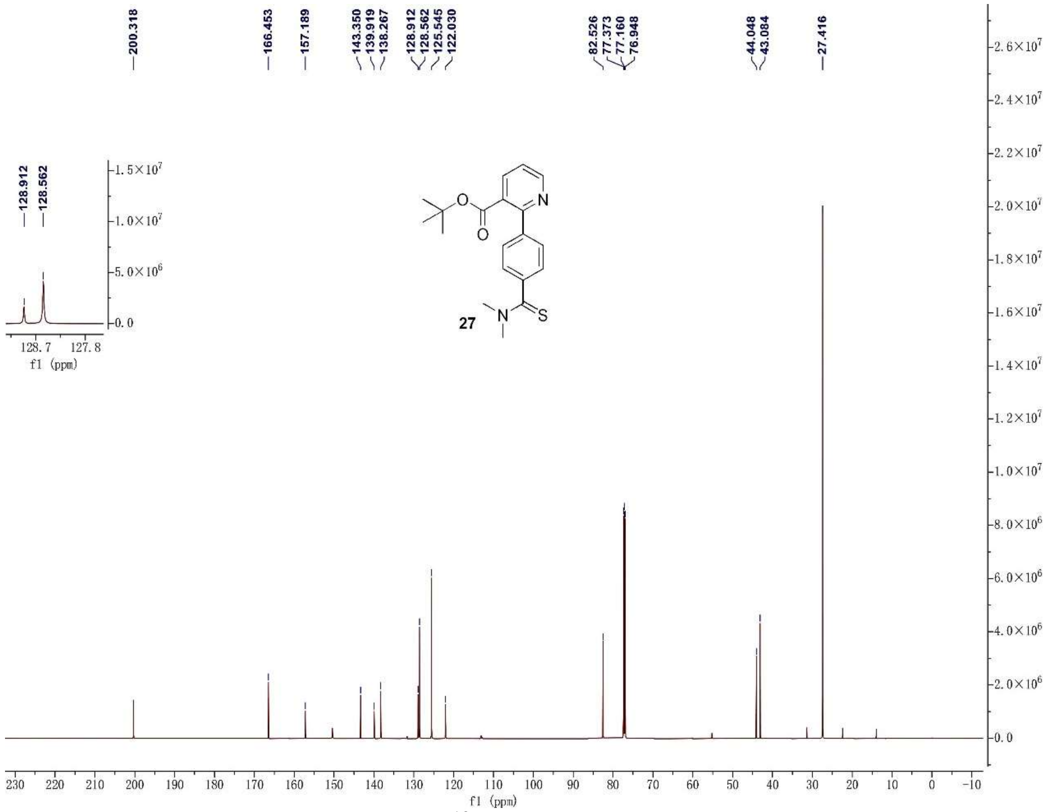

Figure S52: ^{13}C NMR spectrum of **27**

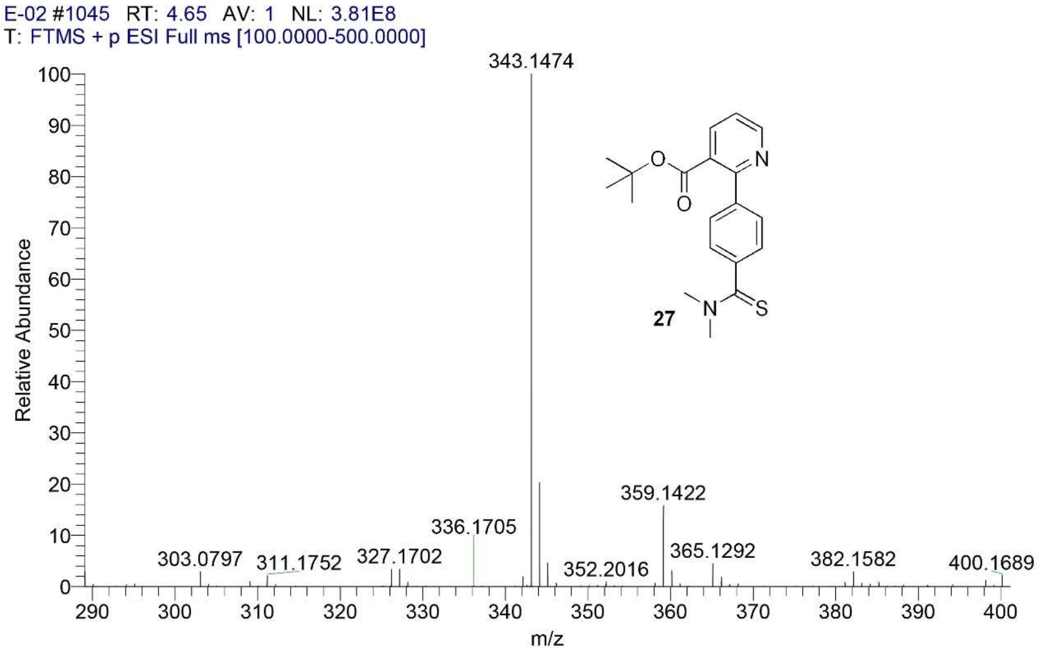

Figure S53: HR-MS (ESI/ion trap) spectrum of **27**

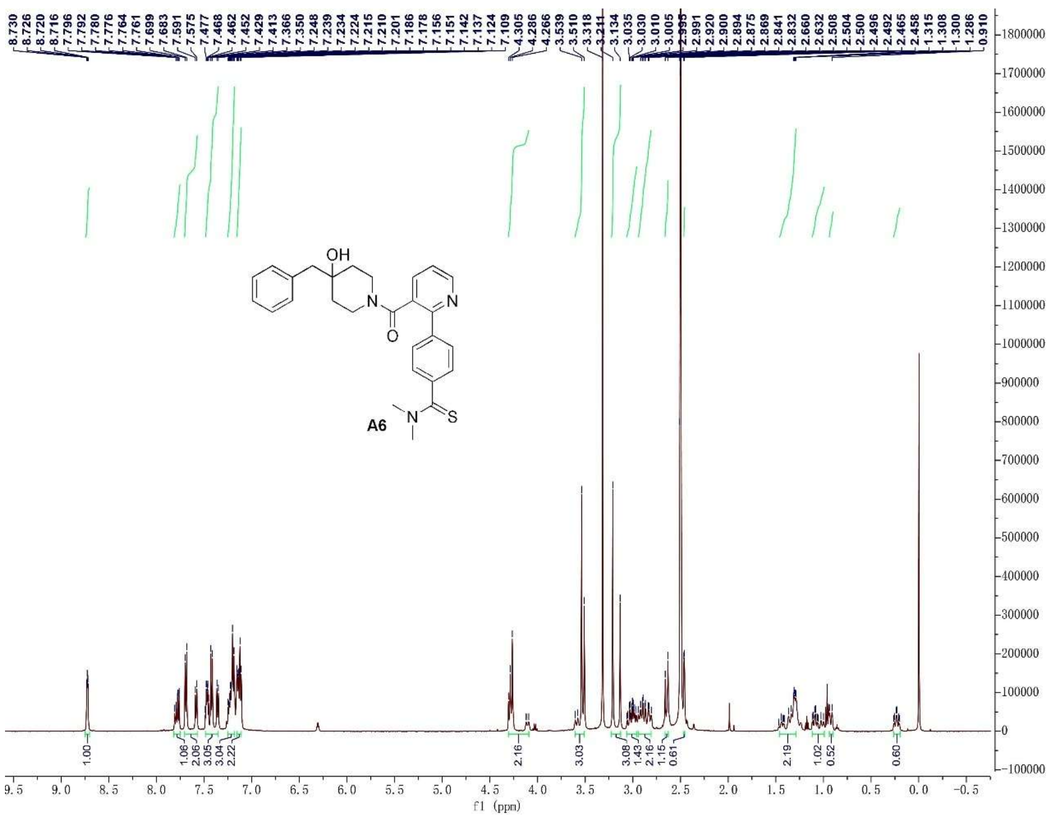

Figure S54: ^{1}H NMR spectrum of **A6**

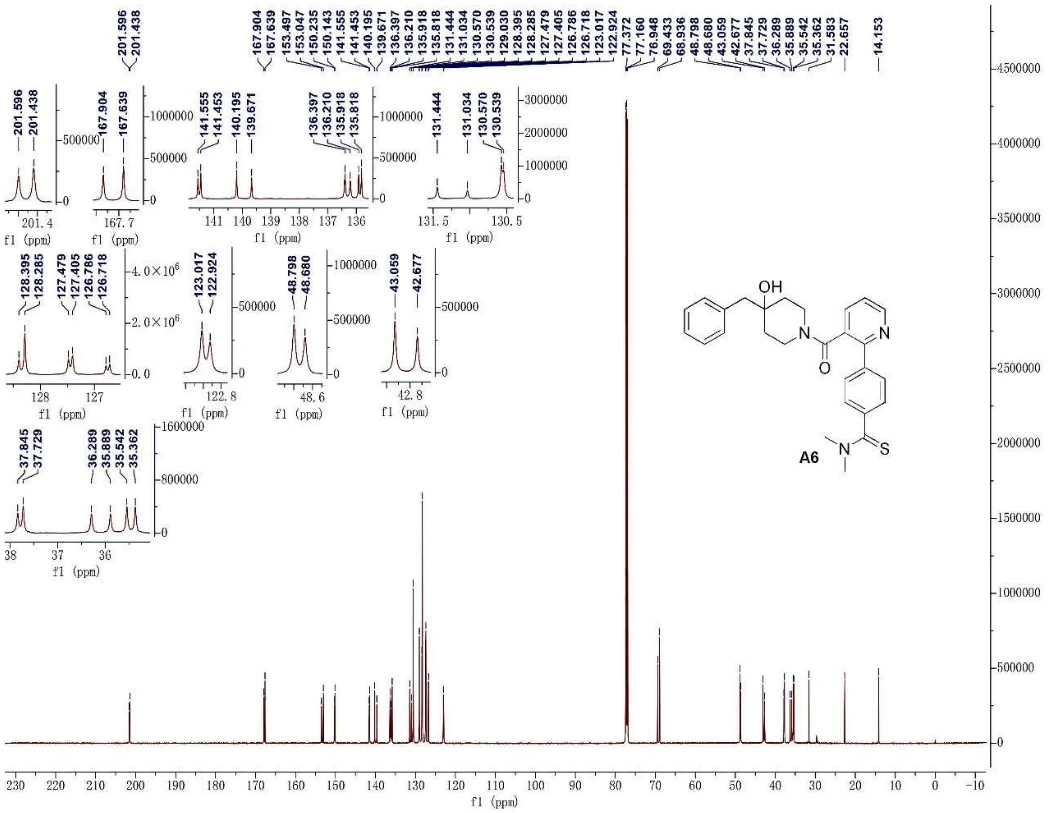

Figure S55: ^{13}C NMR spectrum of **A6**

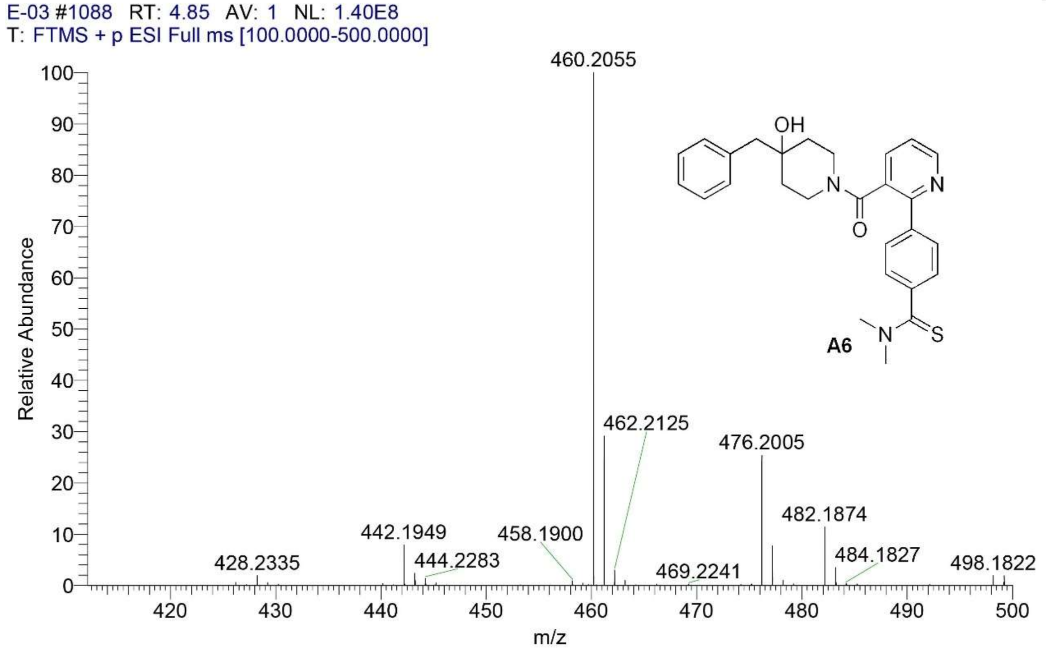

Figure S56: HR-MS (ESI/ion trap) spectrum of **A6**

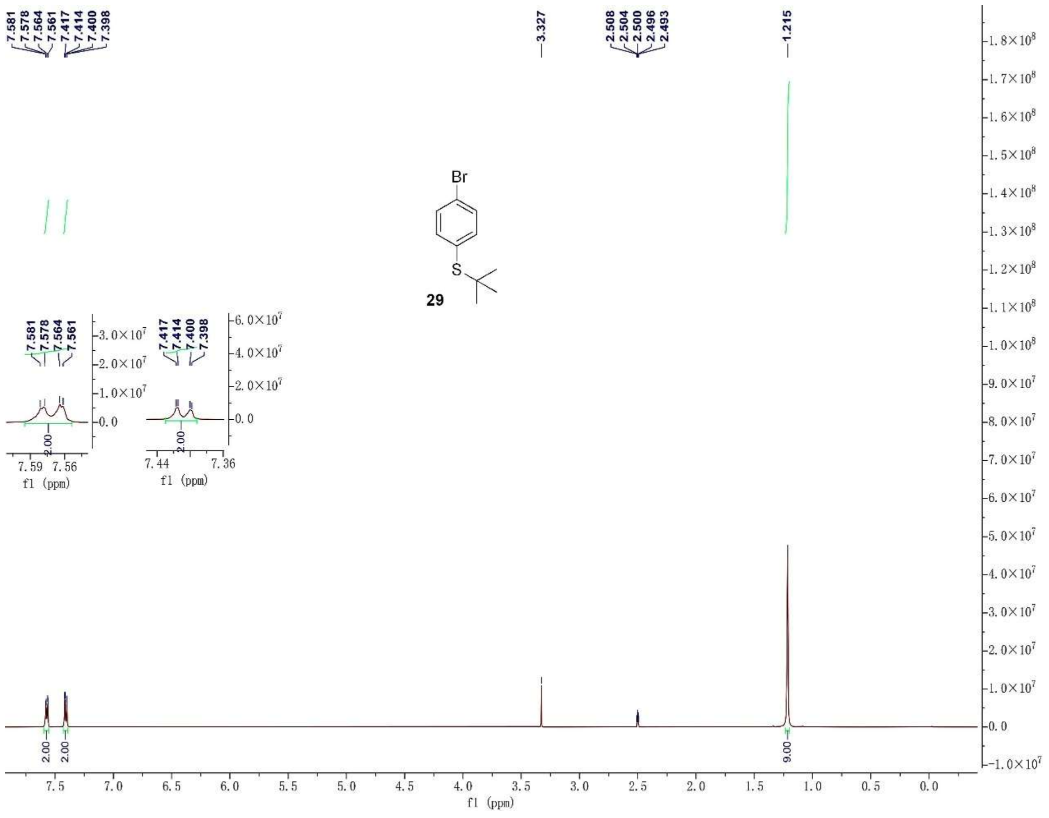

Figure S57: ^{1}H NMR spectrum of **29**

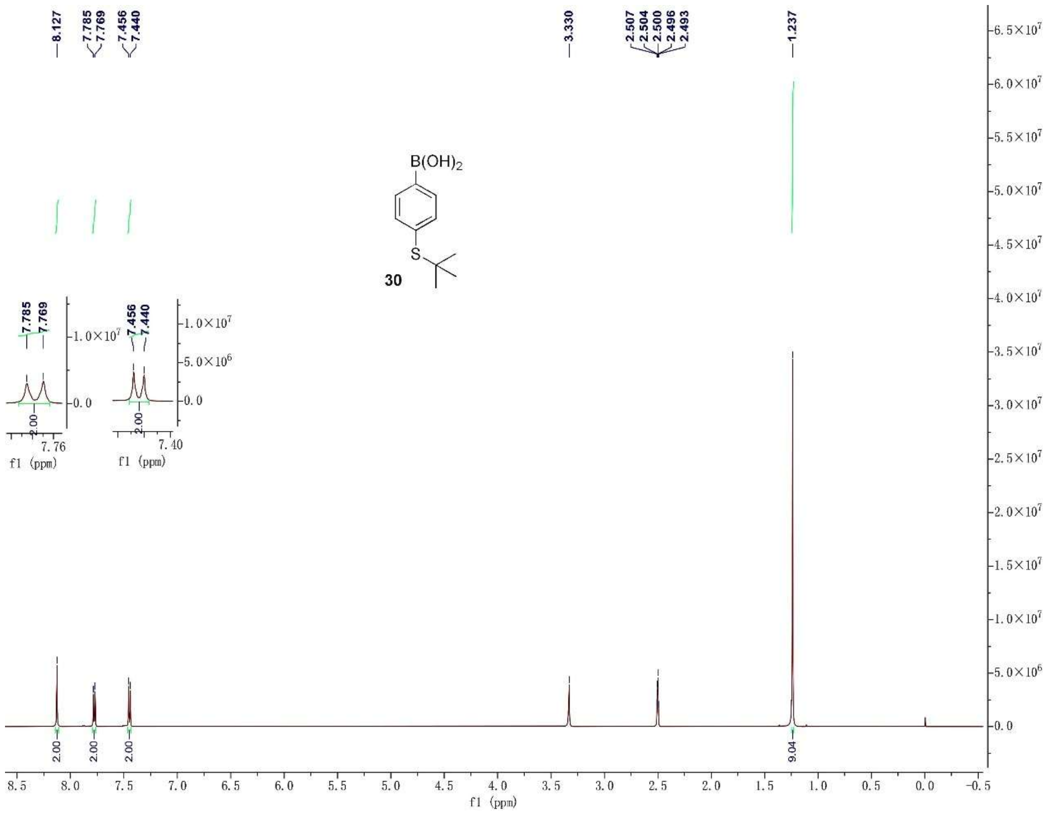

Figure S58: ^{1}H NMR spectrum of **30**

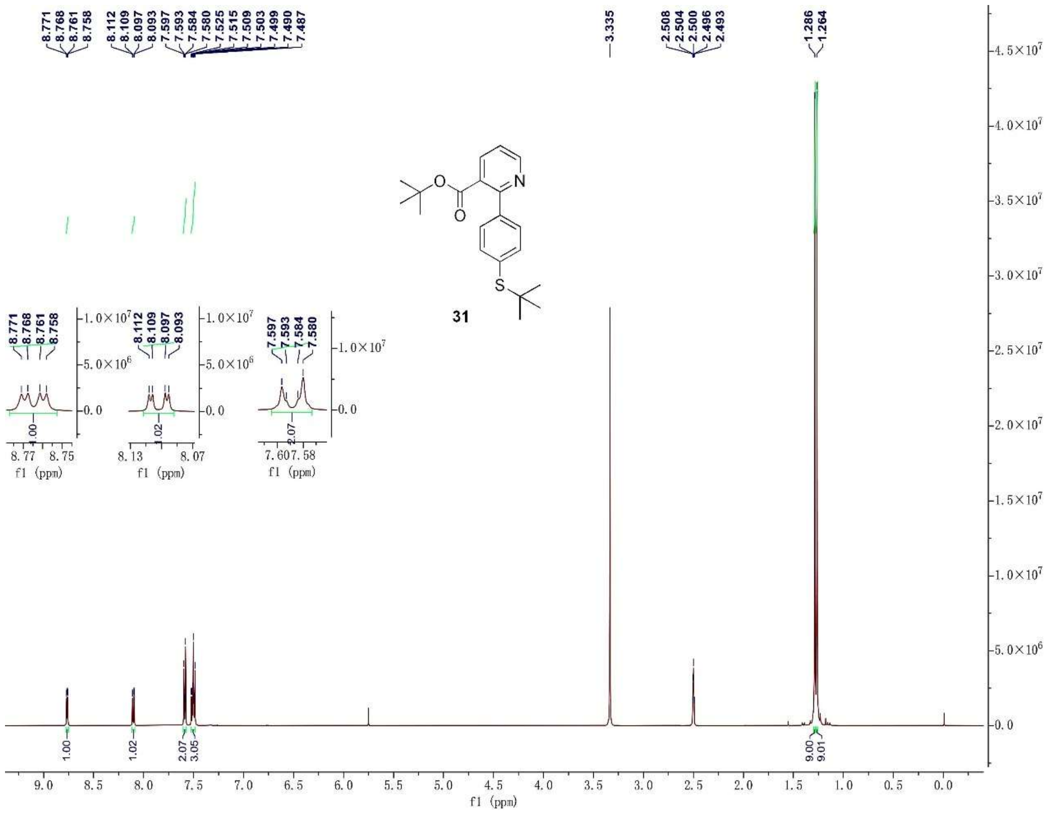

Figure S59: ^{1}H NMR spectrum of **31**

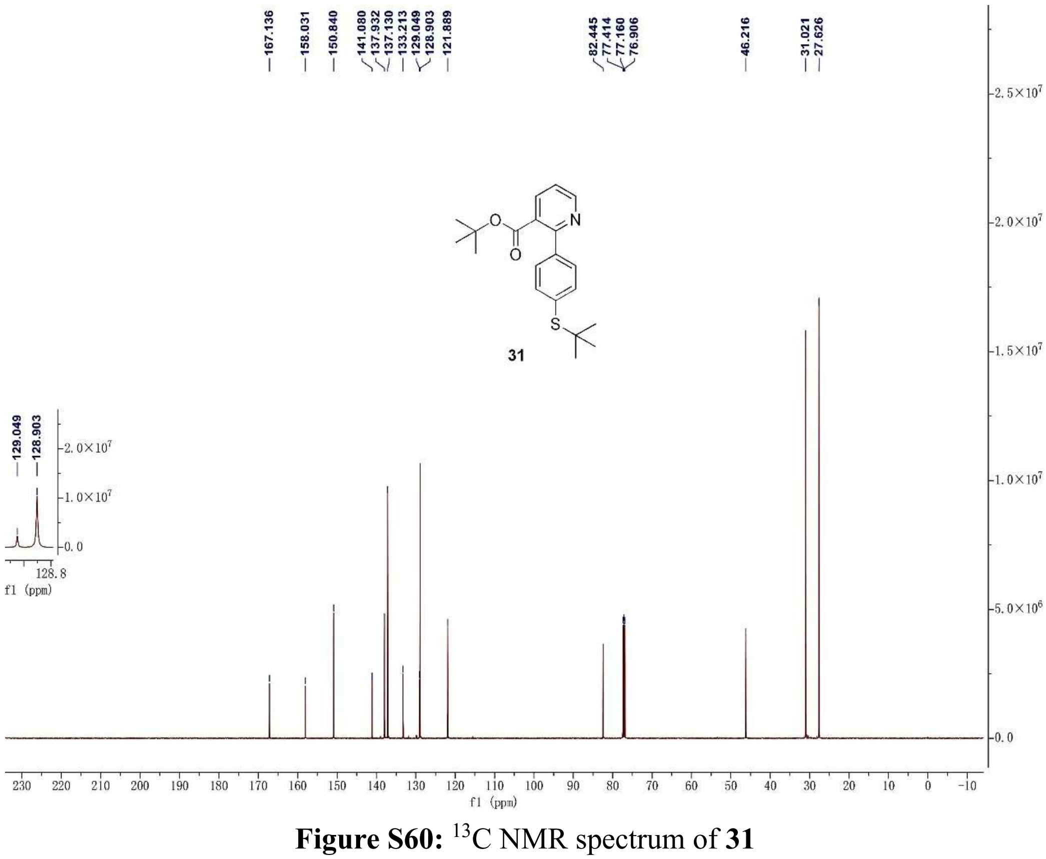

Figure S60: ^{13}C NMR spectrum of **31**

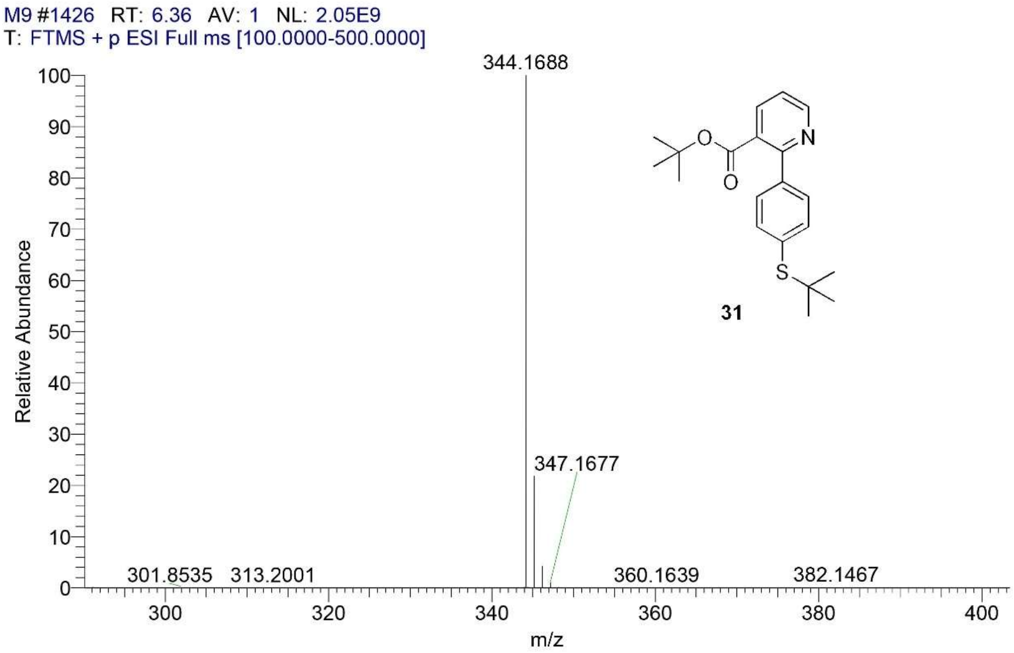

Figure S61: HR-MS (ESI/ion trap) spectrum of **31**

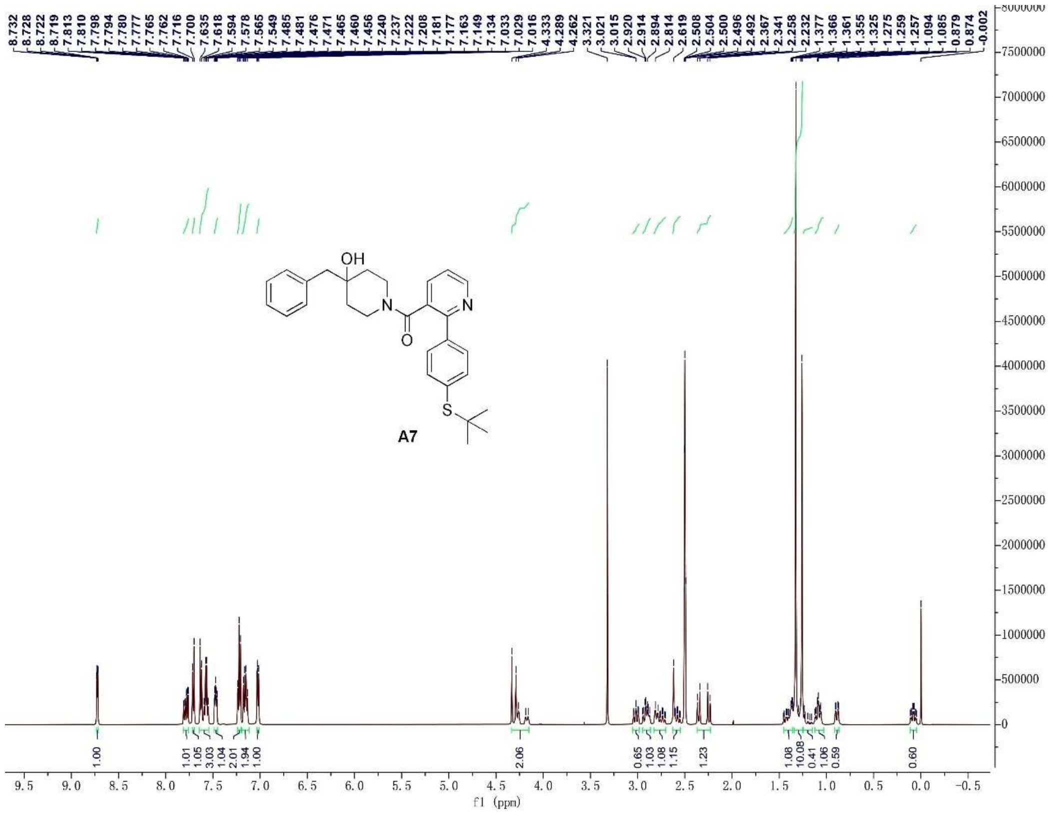

Figure S62: ^{1}H NMR spectrum of **A7**

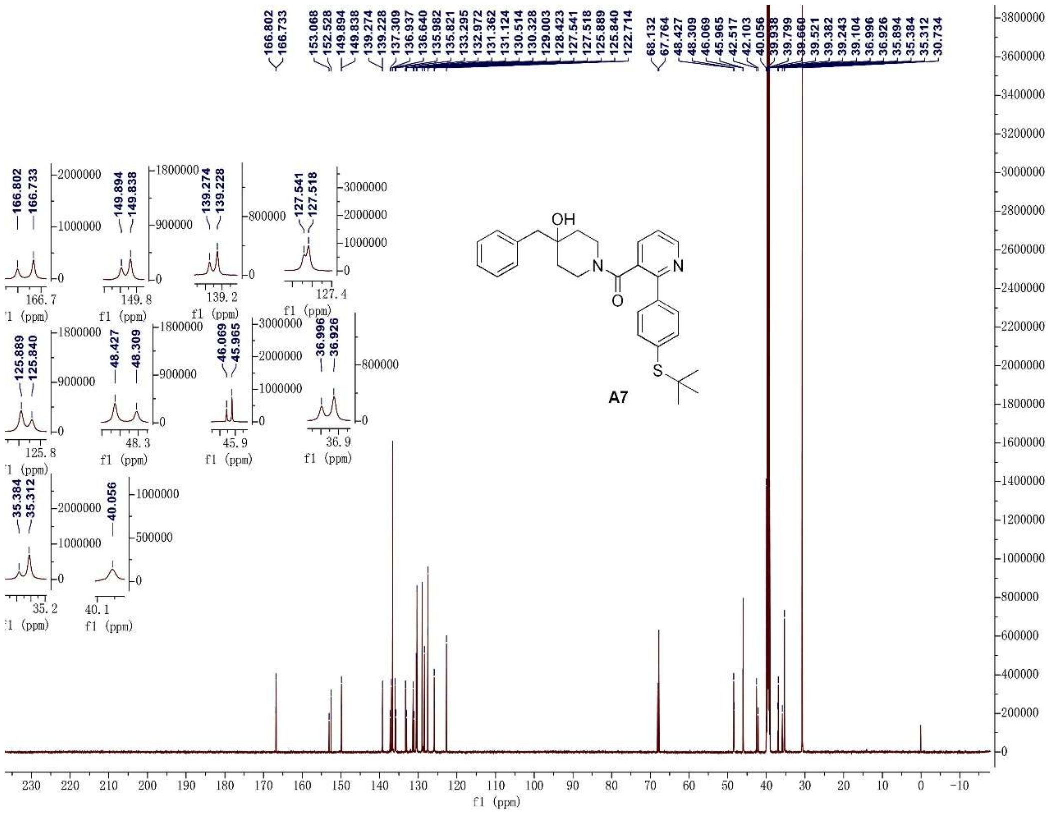

Figure S63: ^{13}C NMR spectrum of **A7**

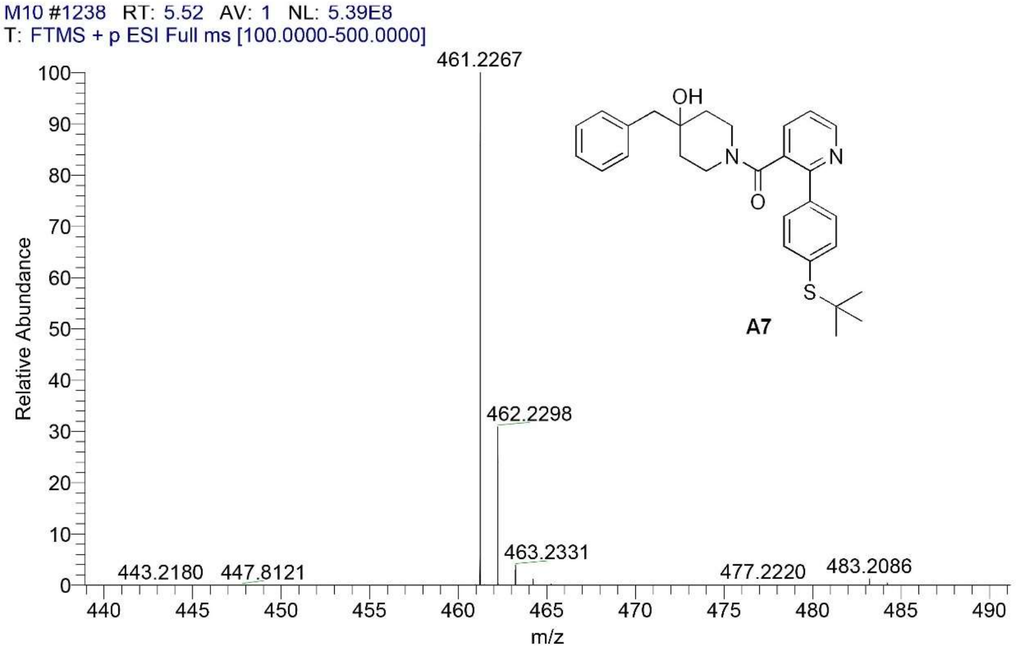

Figure S64: HR-MS (ESI/ion trap) spectrum of **A7**

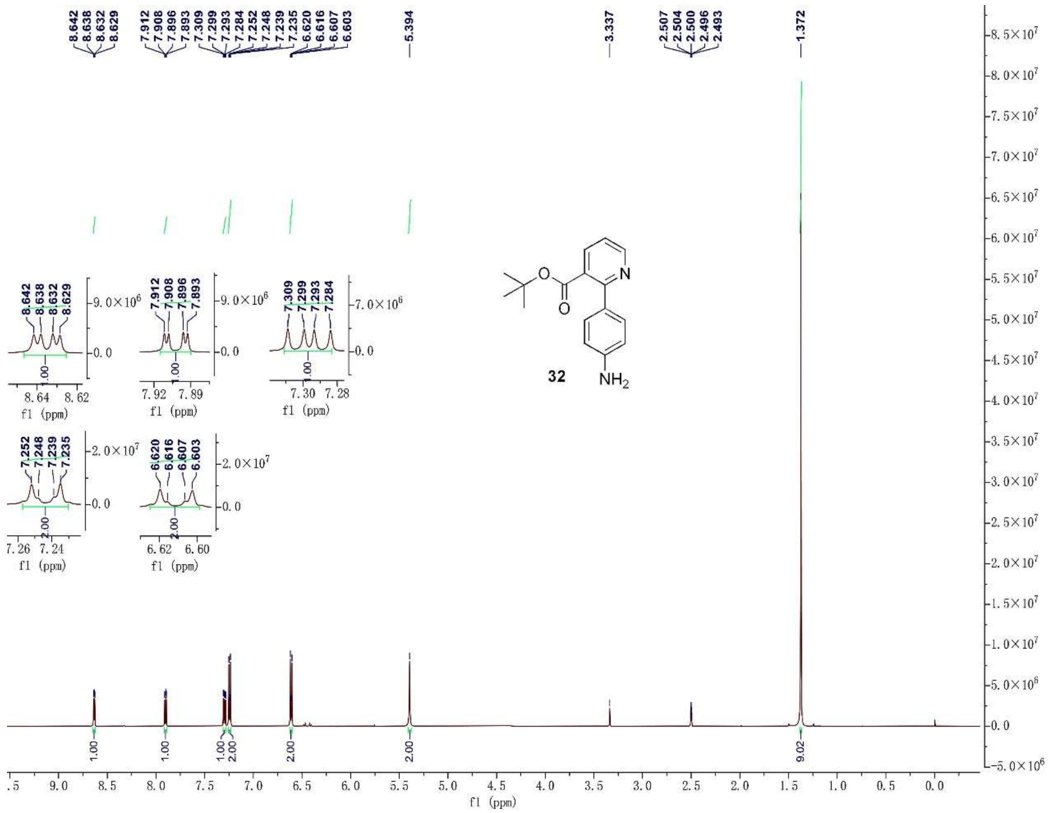

Figure S65: ^{1}H NMR spectrum of **32**

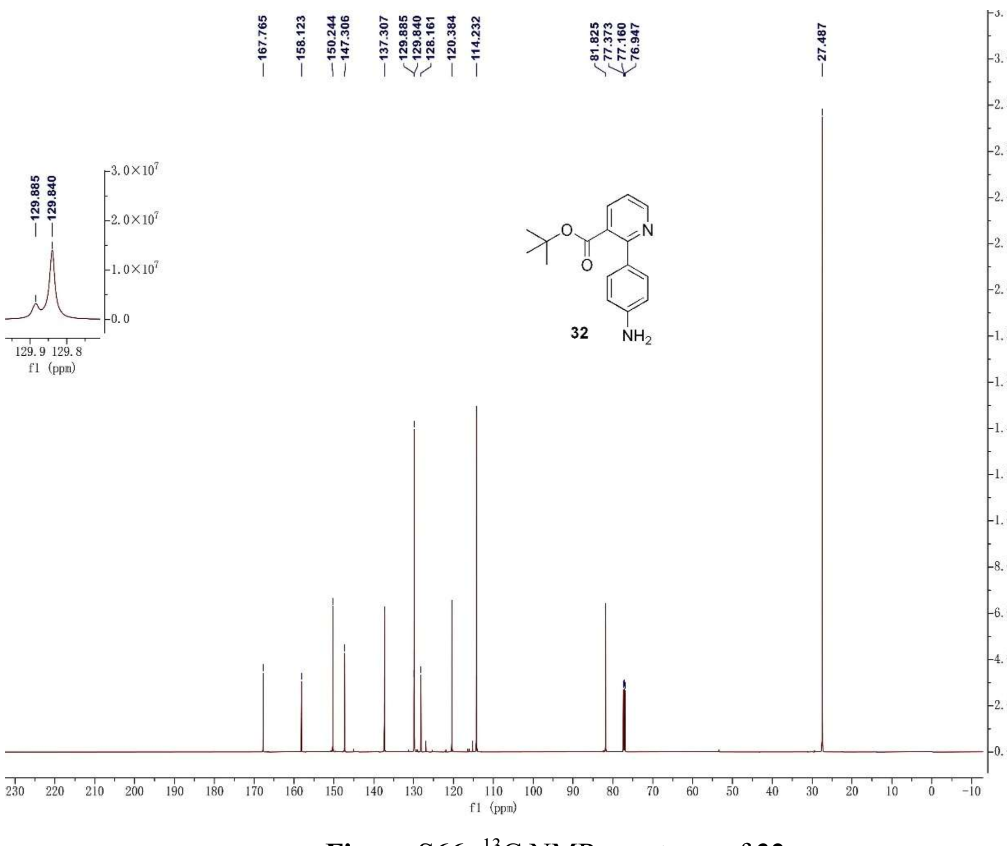

Figure S66: ^{13}C NMR spectrum of **32**

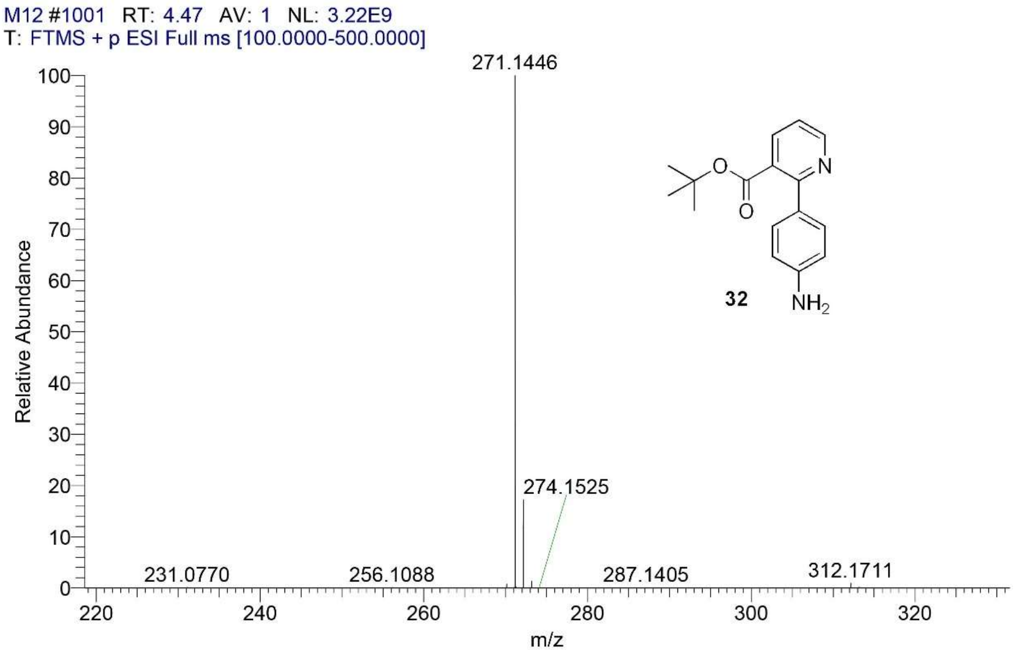

Figure S67: HR-MS (ESI/ion trap) spectrum of **32**

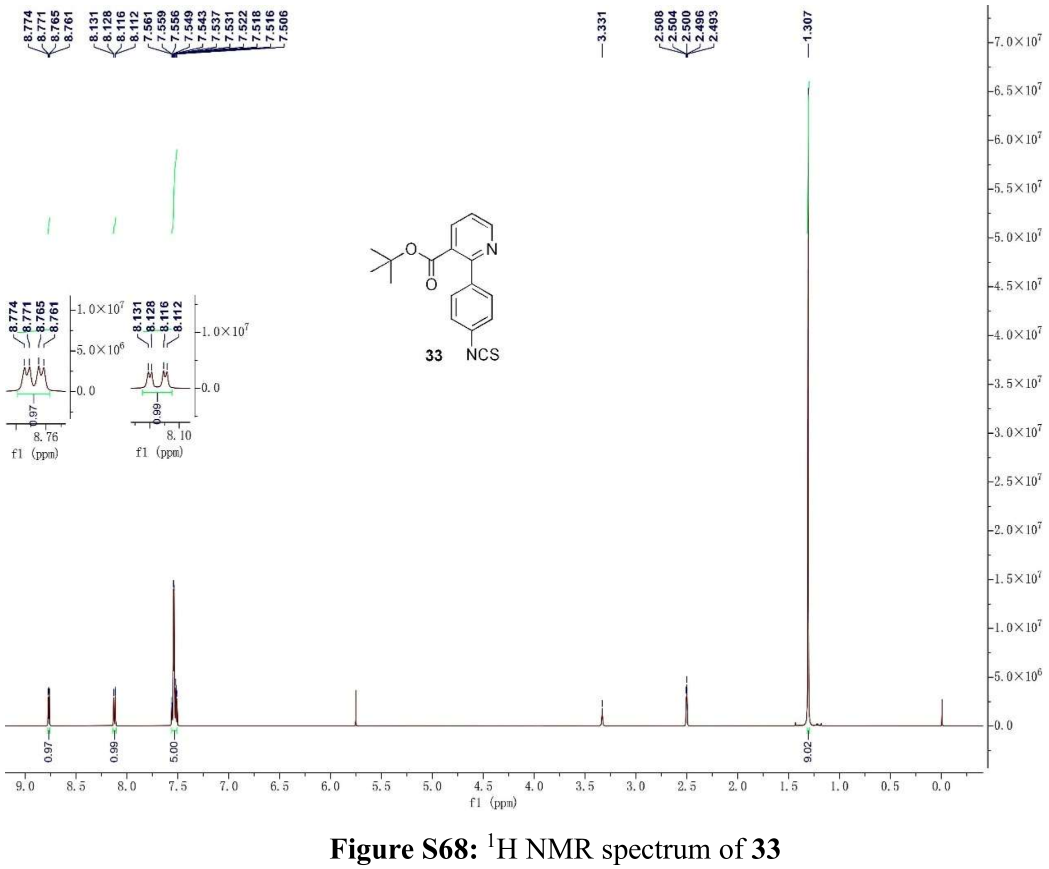

Figure S68: ^{1}H NMR spectrum of **33**

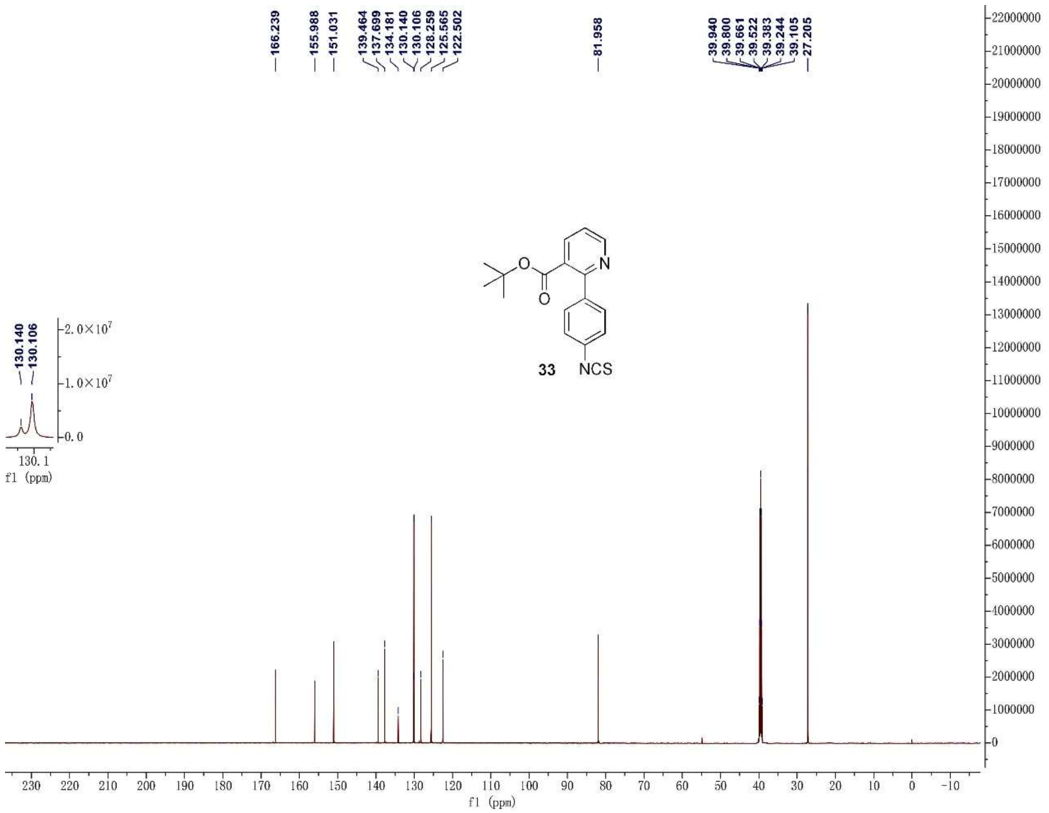

Figure S69: ^{13}C NMR spectrum of **33**

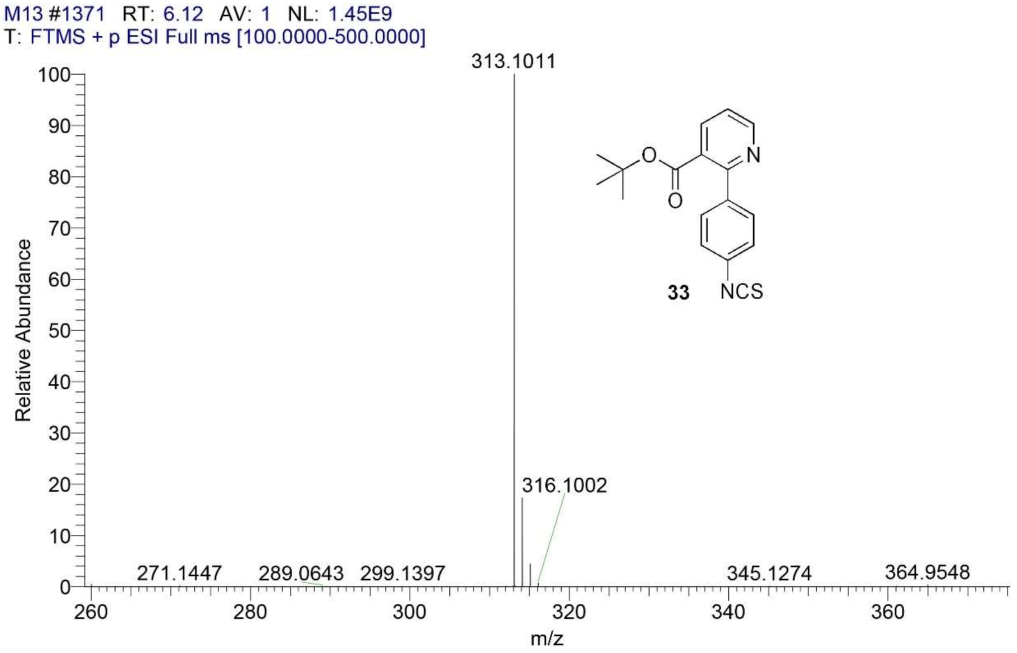

Figure S70: HR-MS (ESI/ion trap) spectrum of **33**

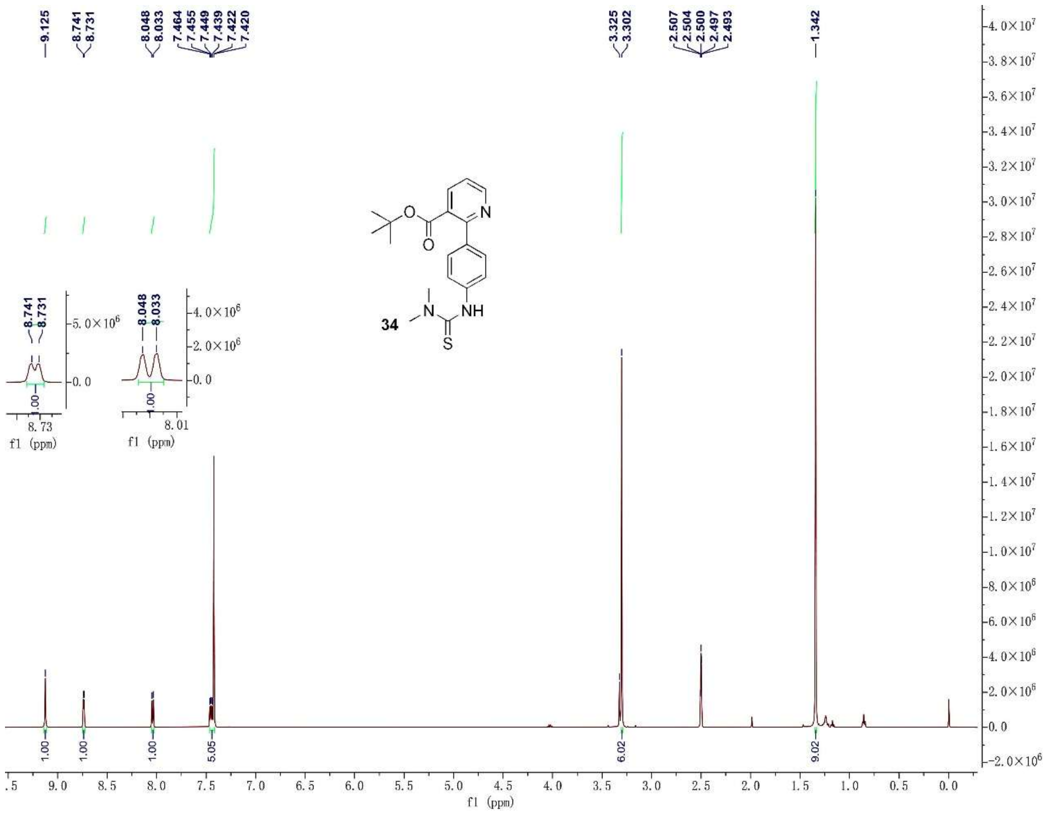

Figure S71: ^{1}H NMR spectrum of **34**

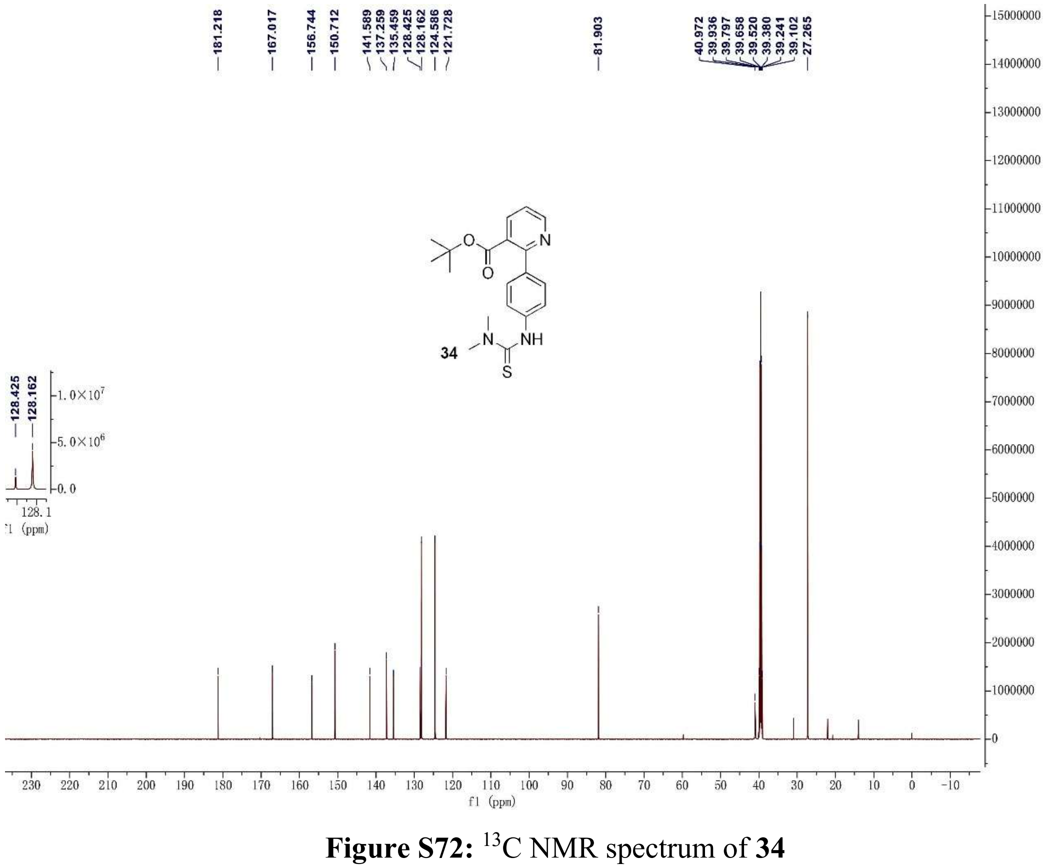

Figure S72: ^{13}C NMR spectrum of **34**

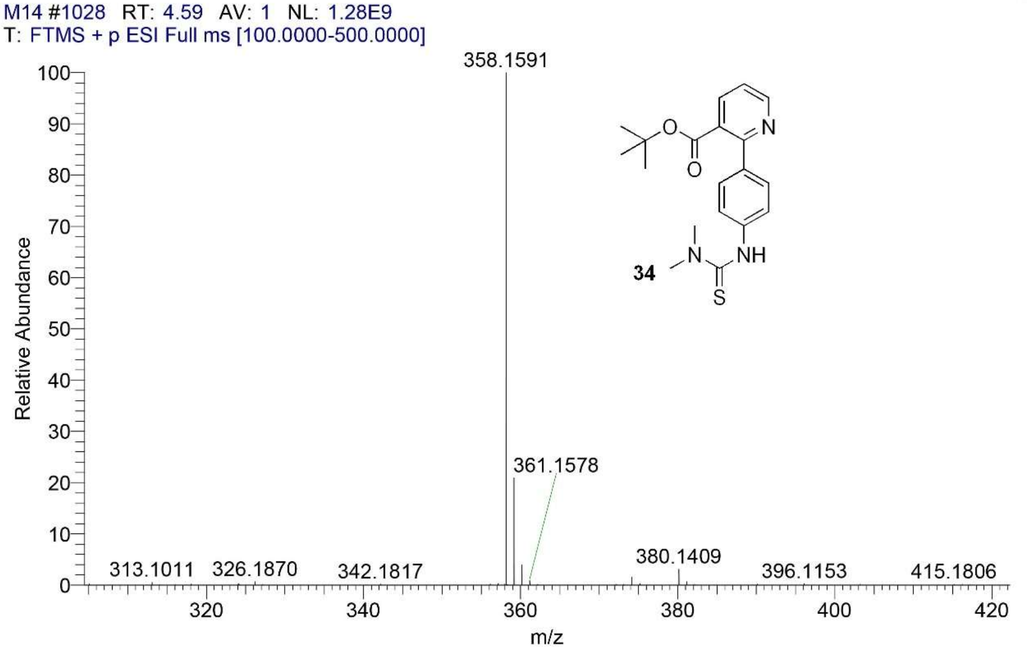

Figure S73: HR-MS (ESI/ion trap) spectrum of **34**

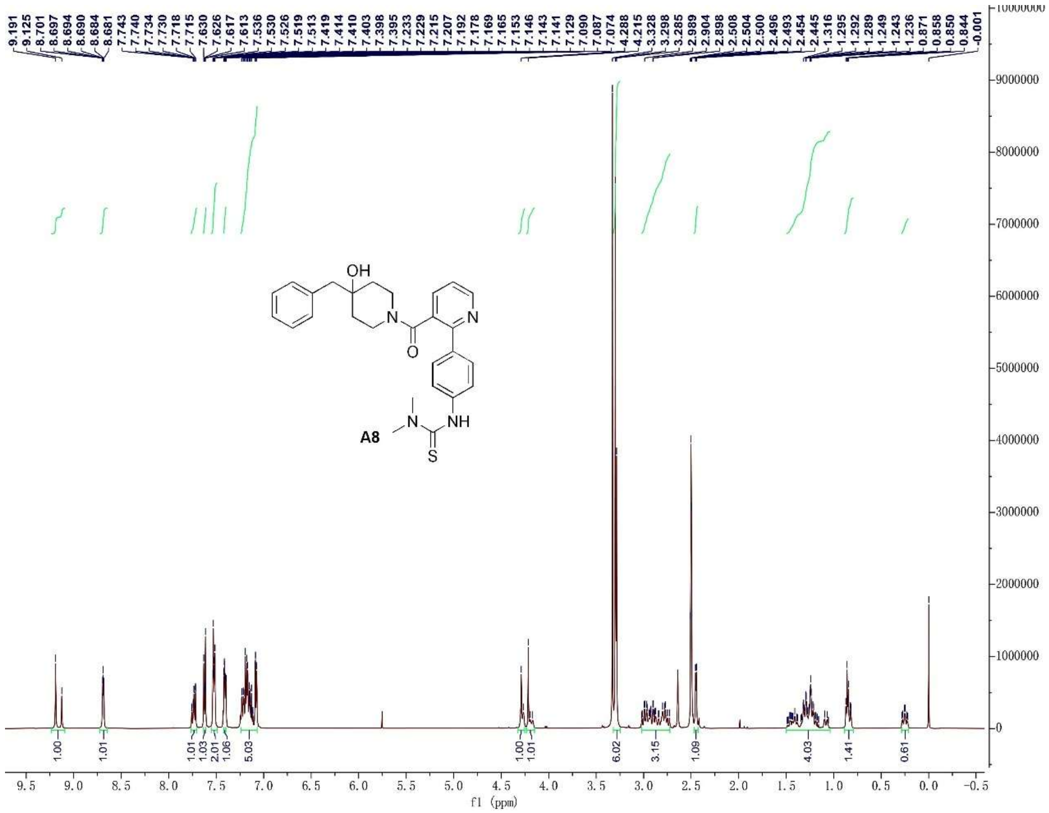

Figure S74: ^{13}C NMR spectrum of **A8**

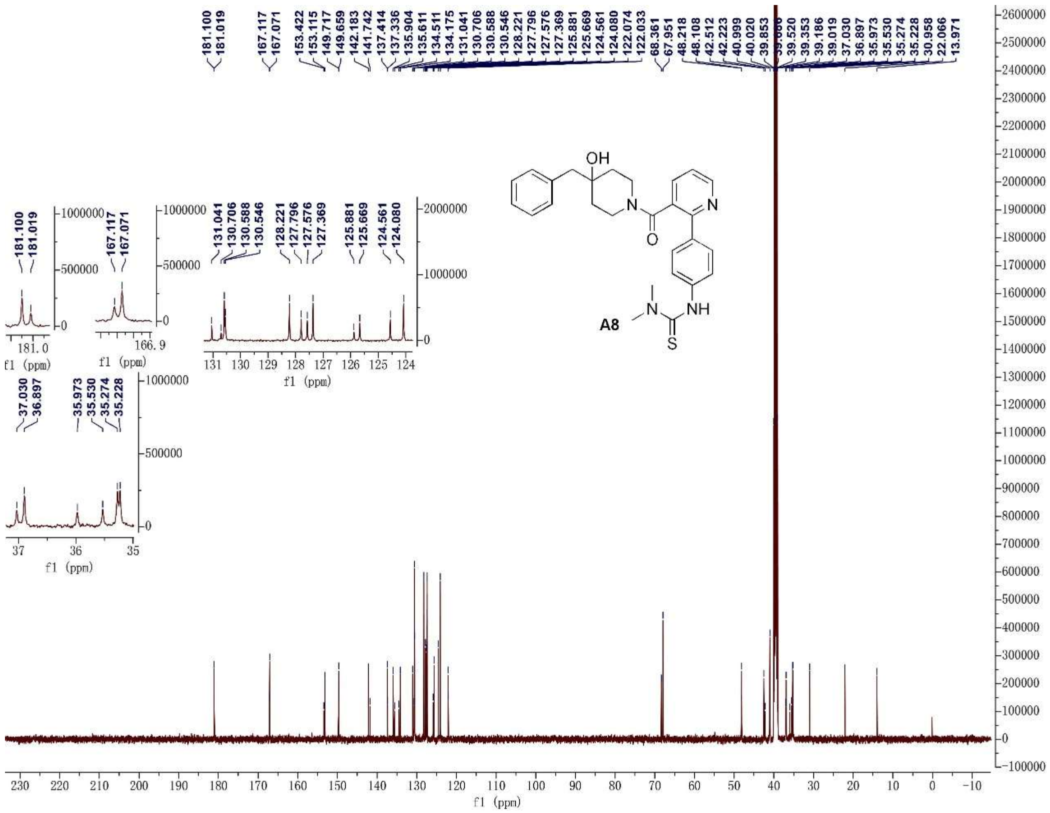

Figure S75: ^{13}C NMR spectrum of **A8**

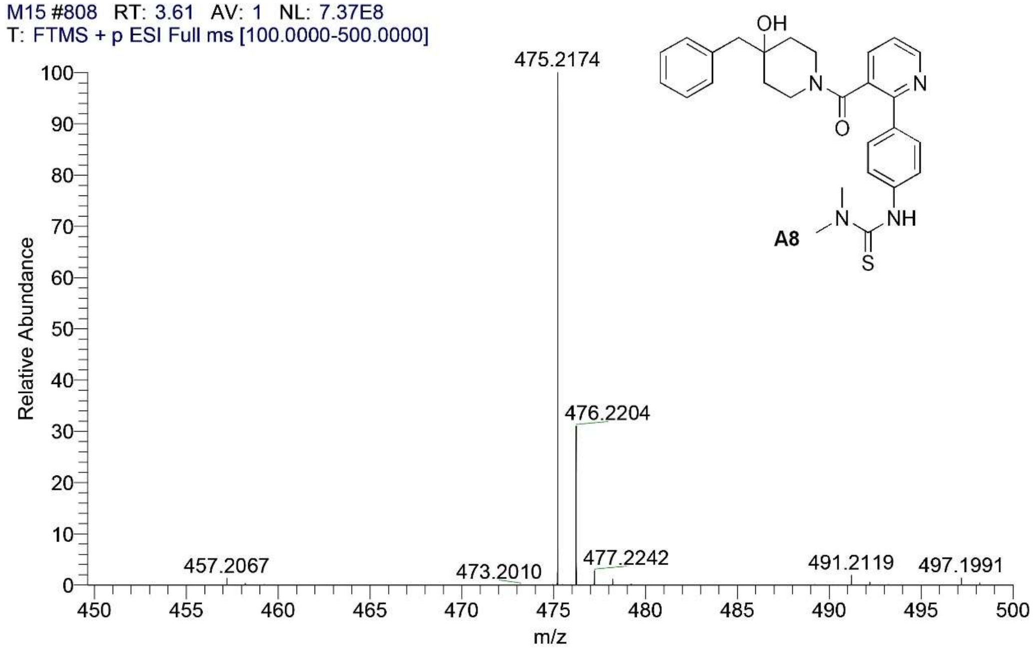

Figure S76: HR-MS (ESI/ion trap) spectrum of A**8**

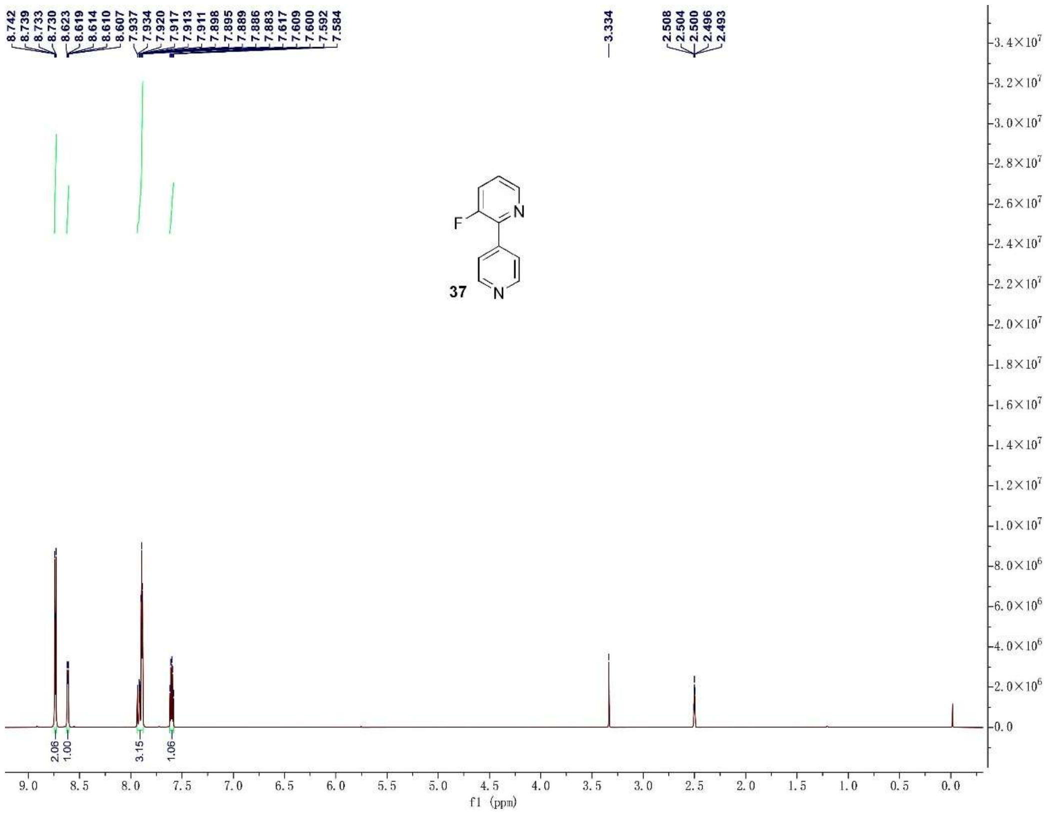

Figure S77: ^{1}H NMR spectrum of **37**

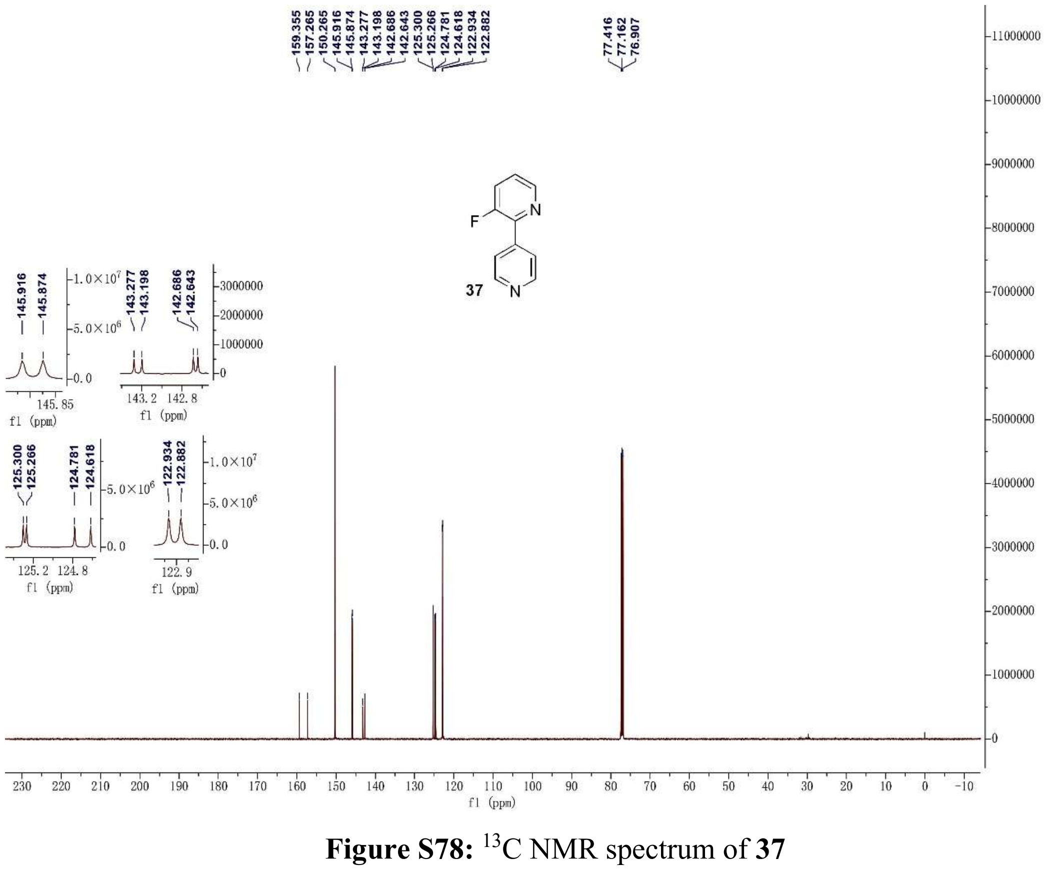

Figure S78: ^{13}C NMR spectrum of **37**

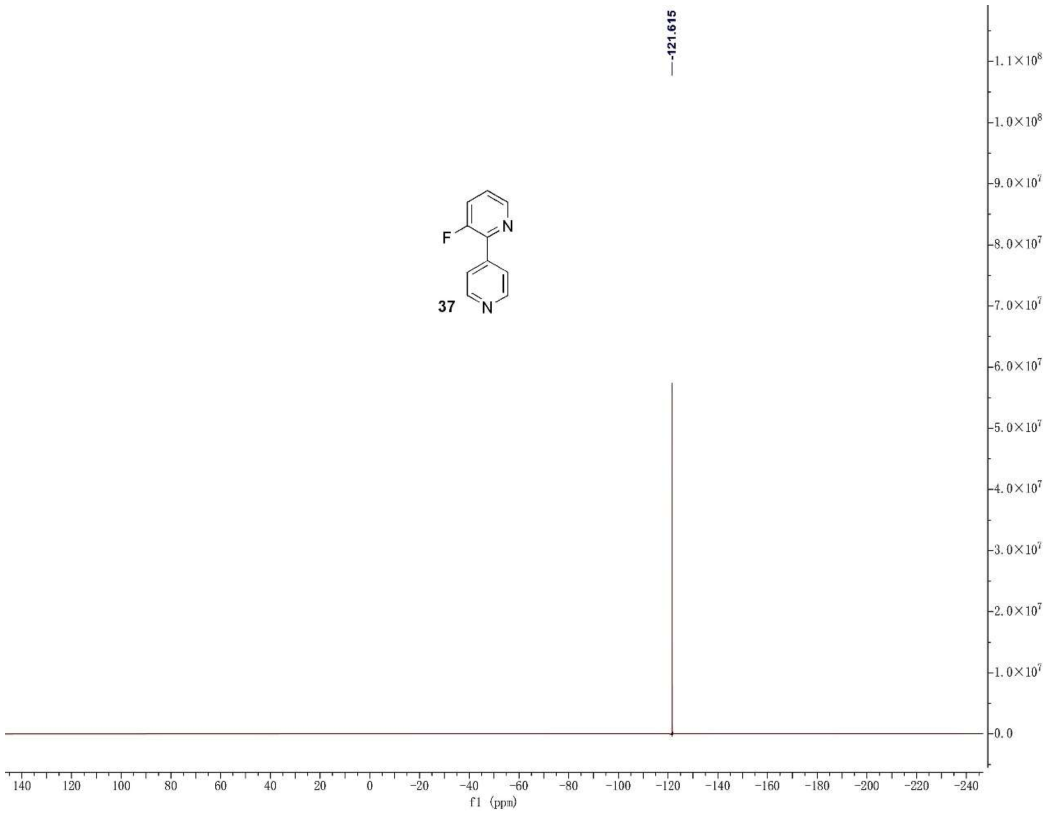

Figure S79: ^{19}F NMR spectrum of **37**

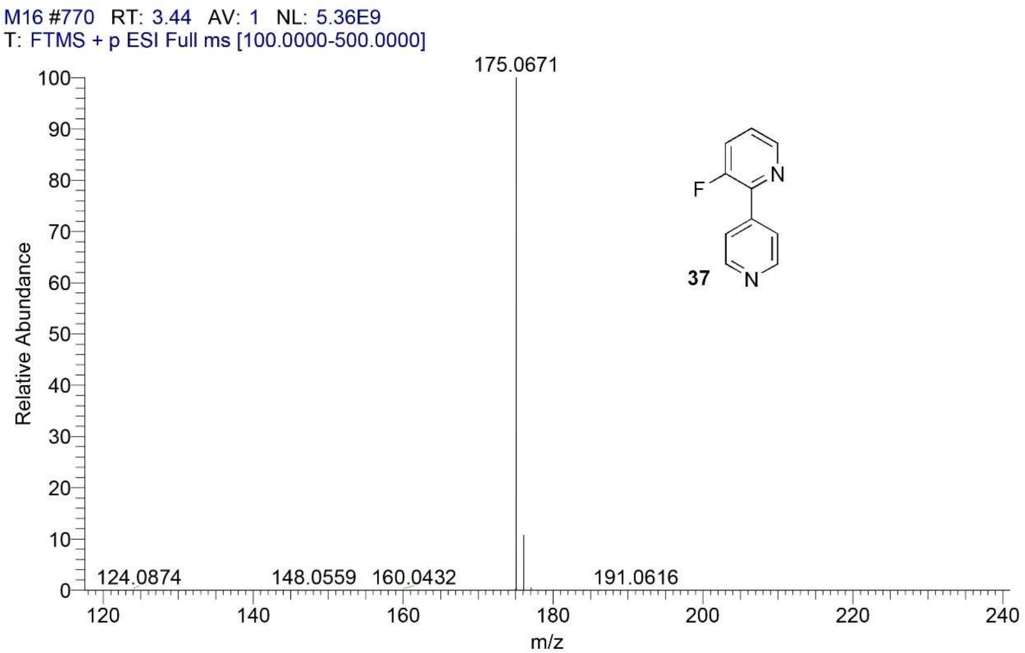

Figure S80: HR-MS (ESI/ion trap) spectrum of **37**

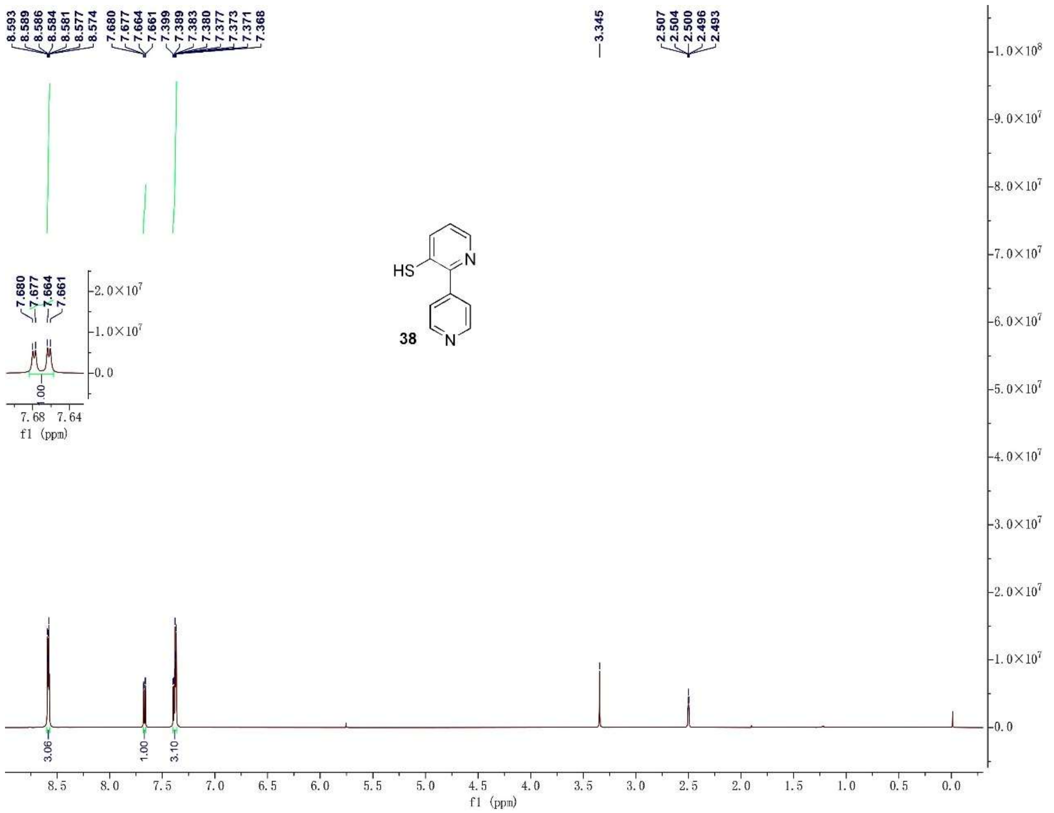

Figure S81: ^{1}H NMR spectrum of **38**

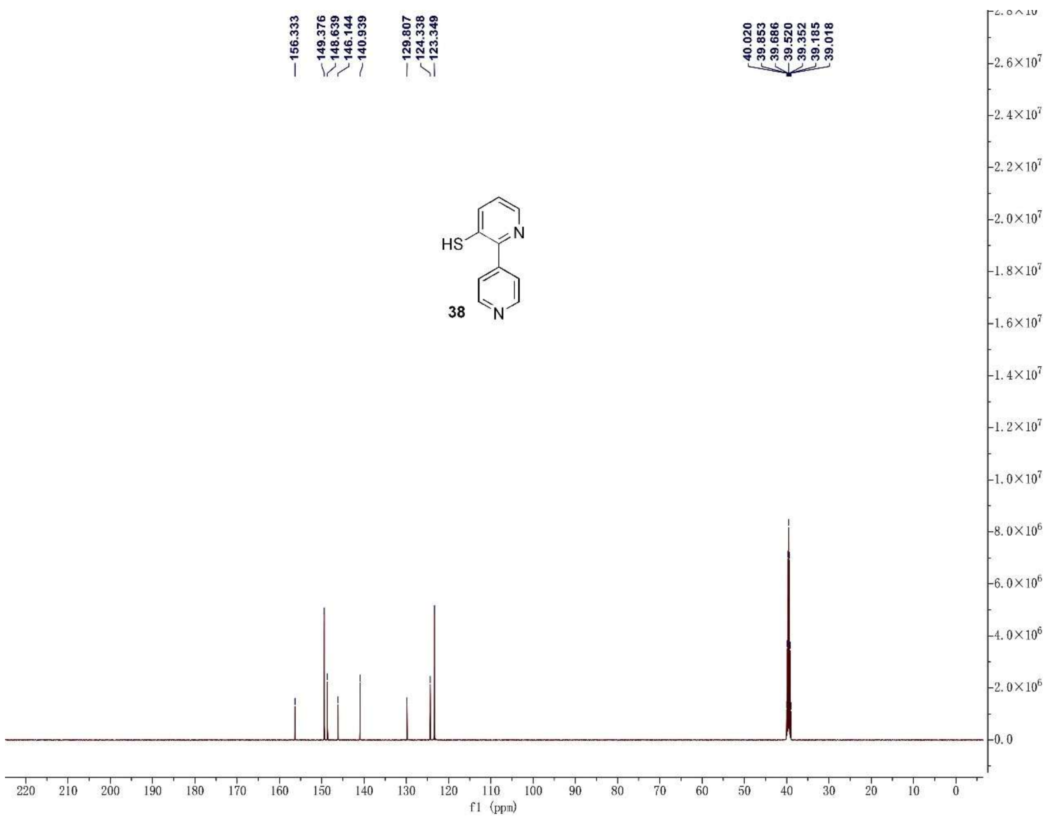

Figure S82: ^{13}C NMR spectrum of **38**

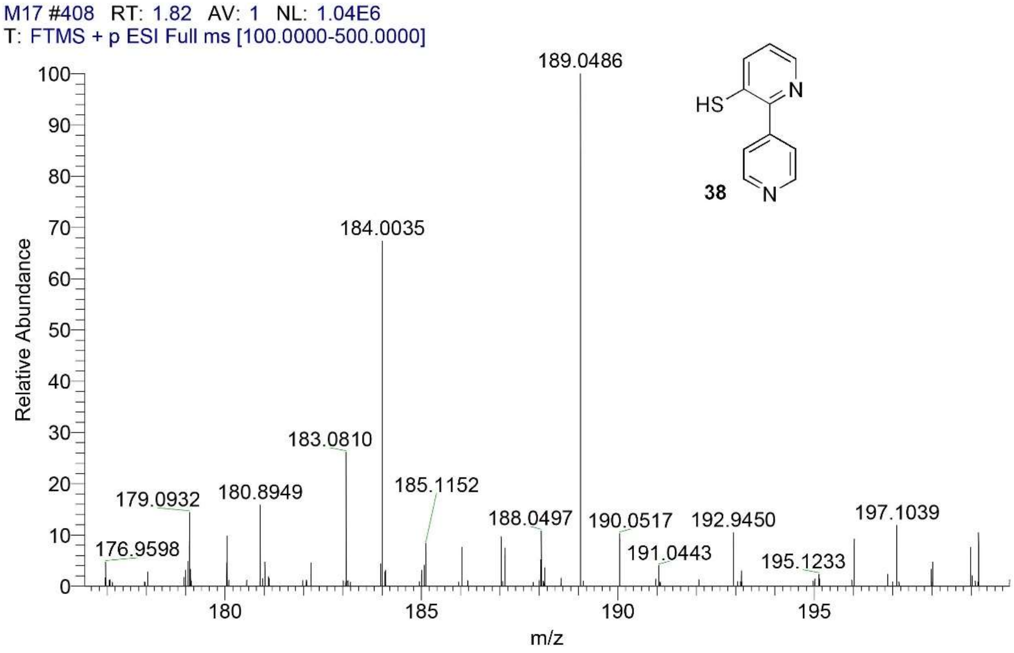

Figure S83: HR-MS (ESI/ion trap) spectrum of 3**8**

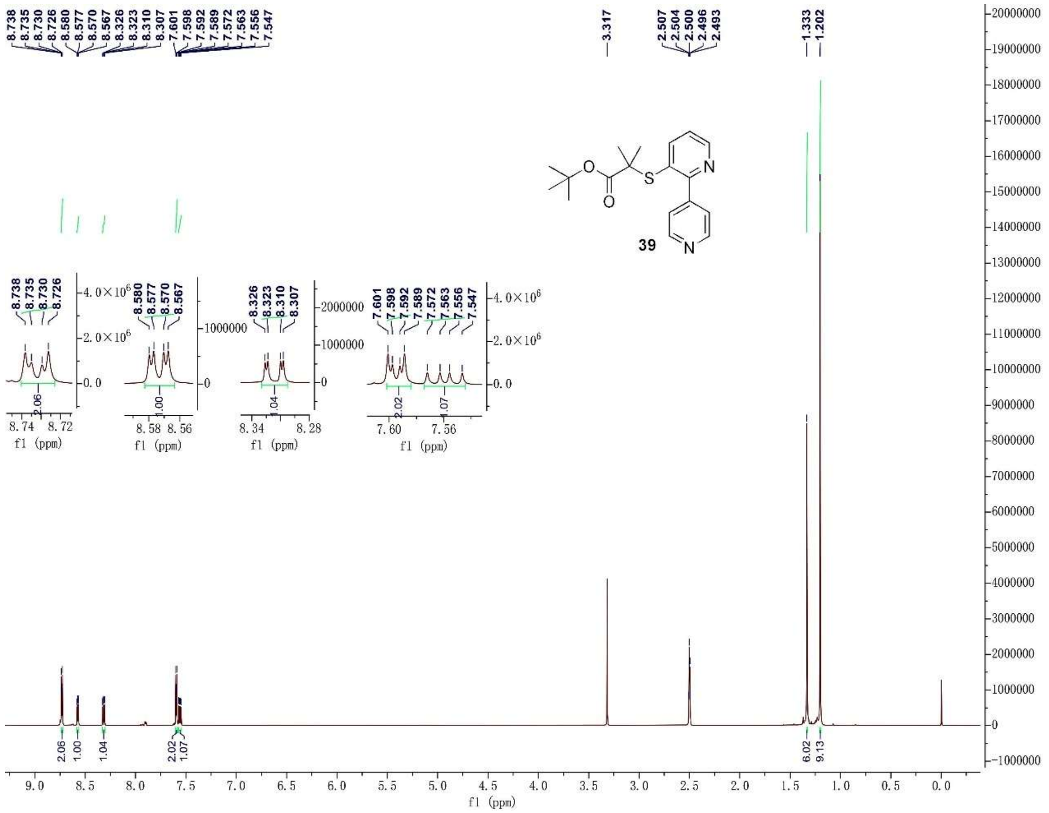

Figure S84: ^{1}H NMR spectrum of **39**

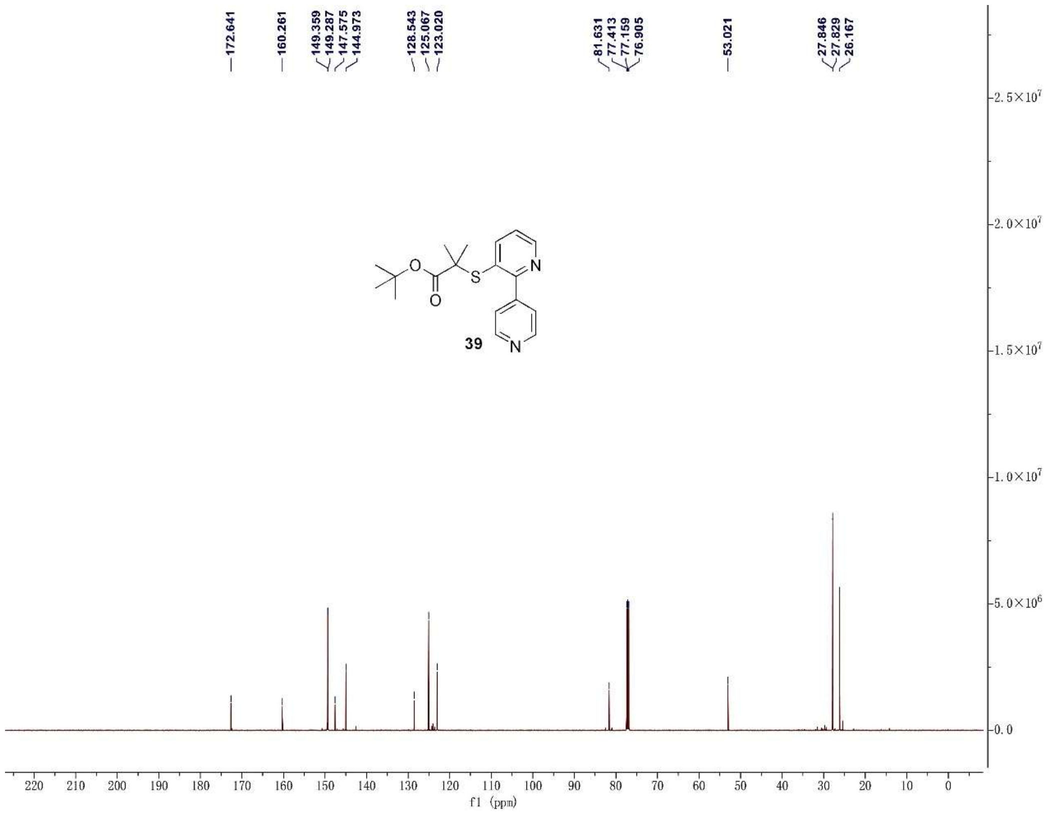

Figure S85: ^{13}C NMR spectrum of **39**

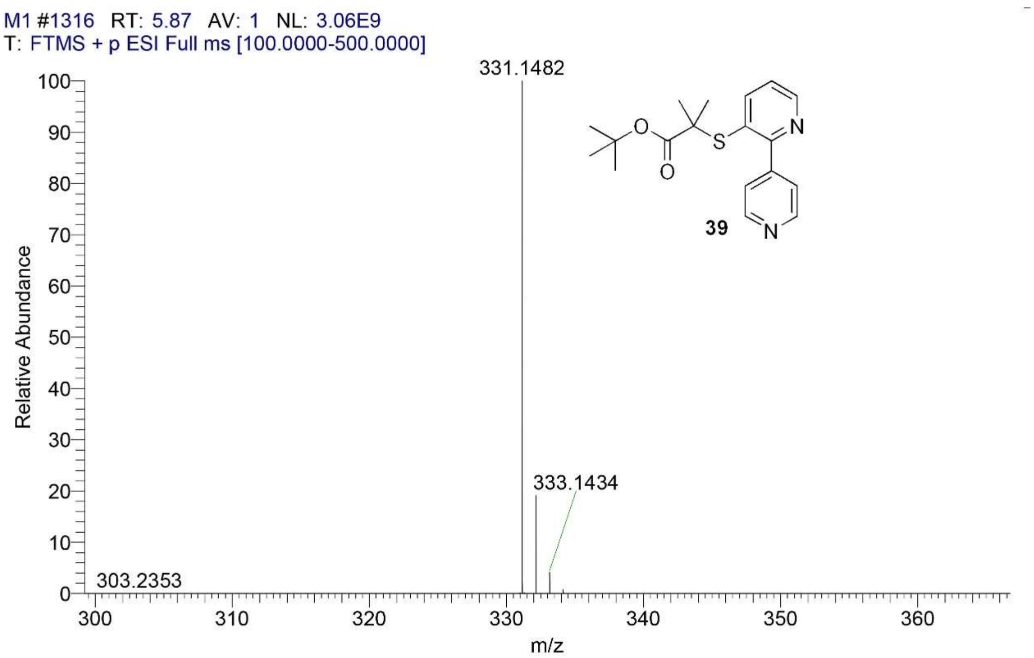

Figure S86: HR-MS (ESI/ion trap) spectrum of **39**

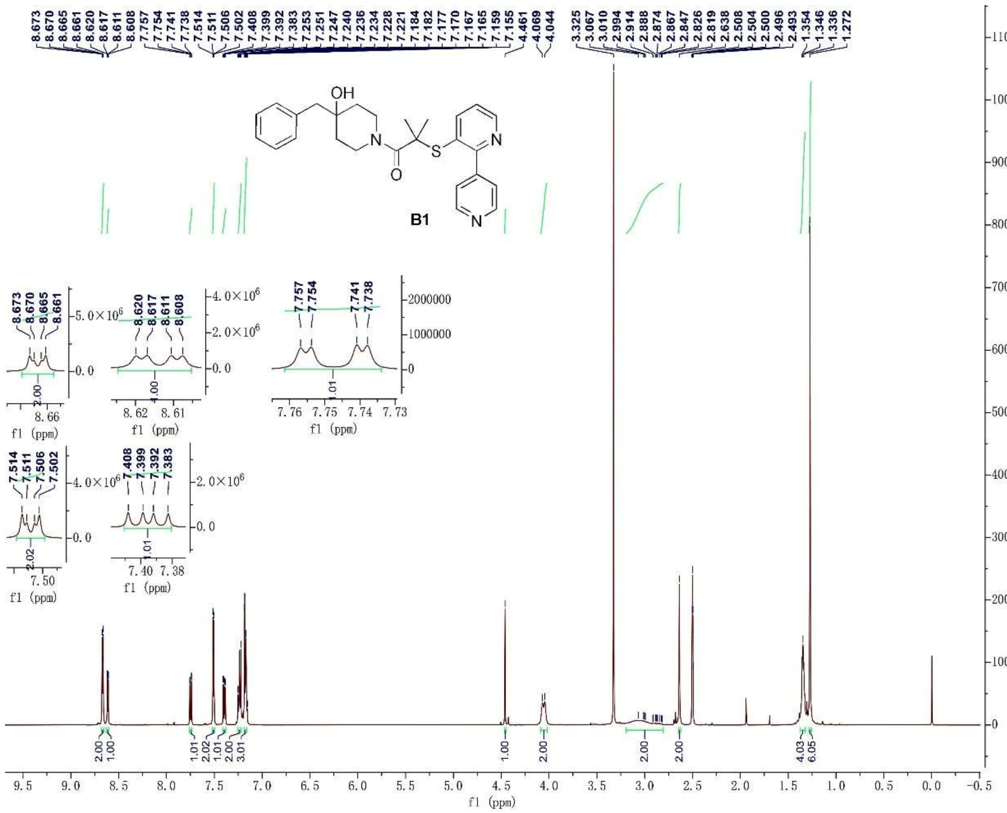

Figure S87: ^{1}H NMR spectrum of **B1**

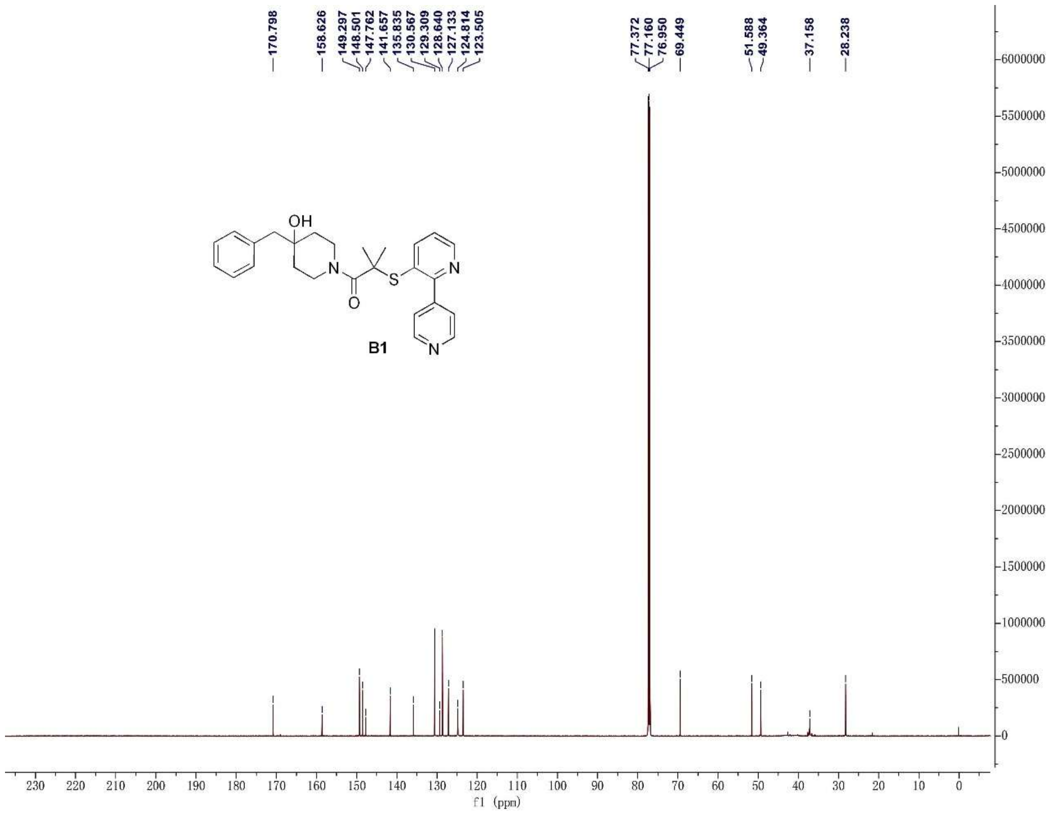

Figure S88: ^{13}C NMR spectrum of **B1**

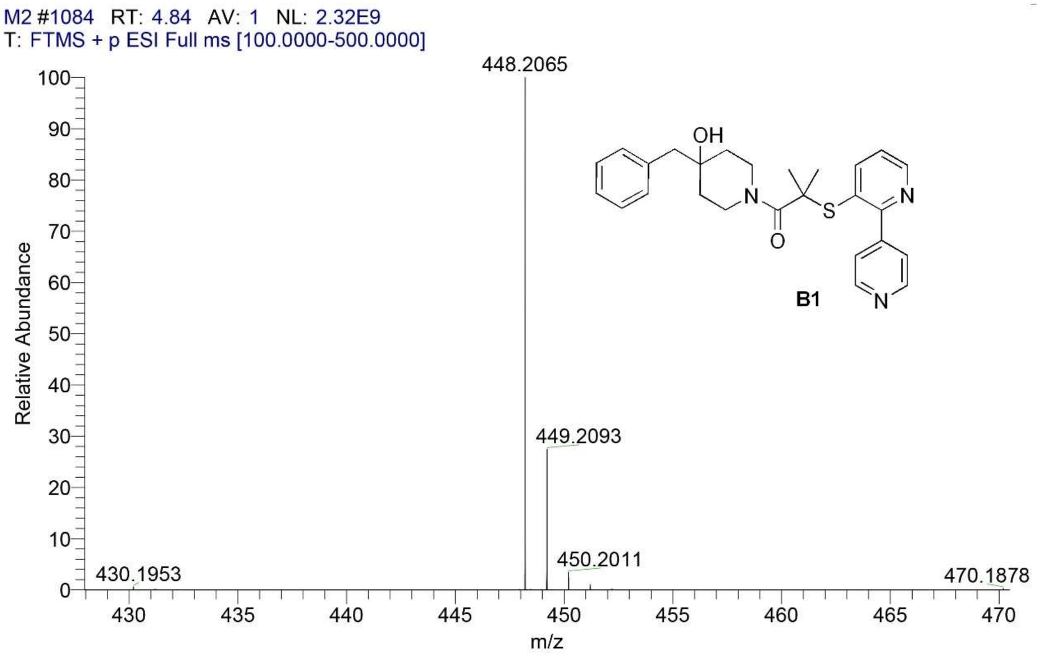

Figure S89: HR-MS (ESI/ion trap) spectrum of **B1**

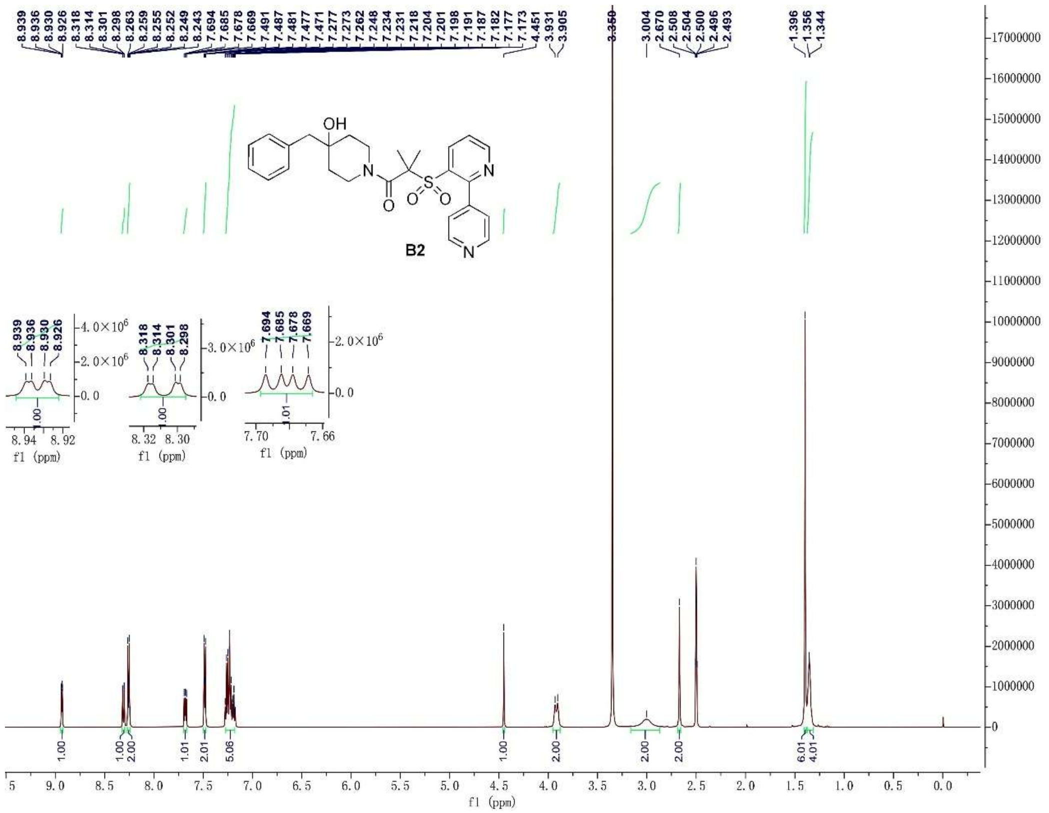

Figure S90: ^{1}H NMR spectrum of **B2**

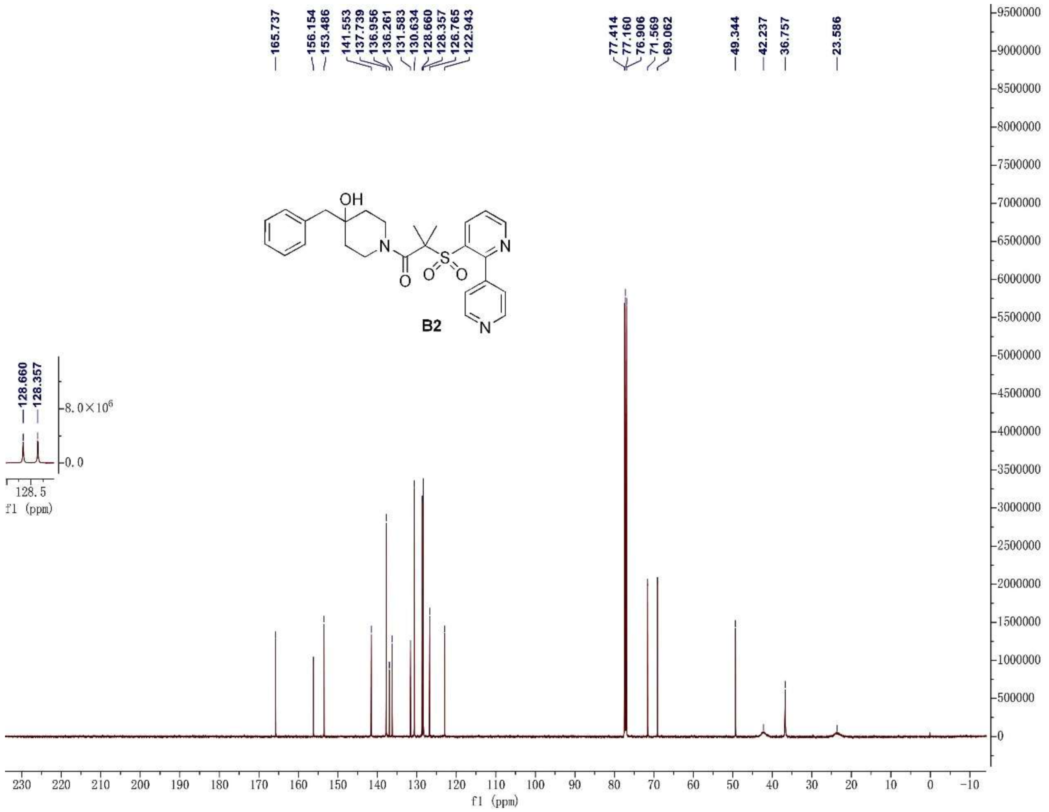

Figure S91: ^{13}C NMR spectrum of **B2**

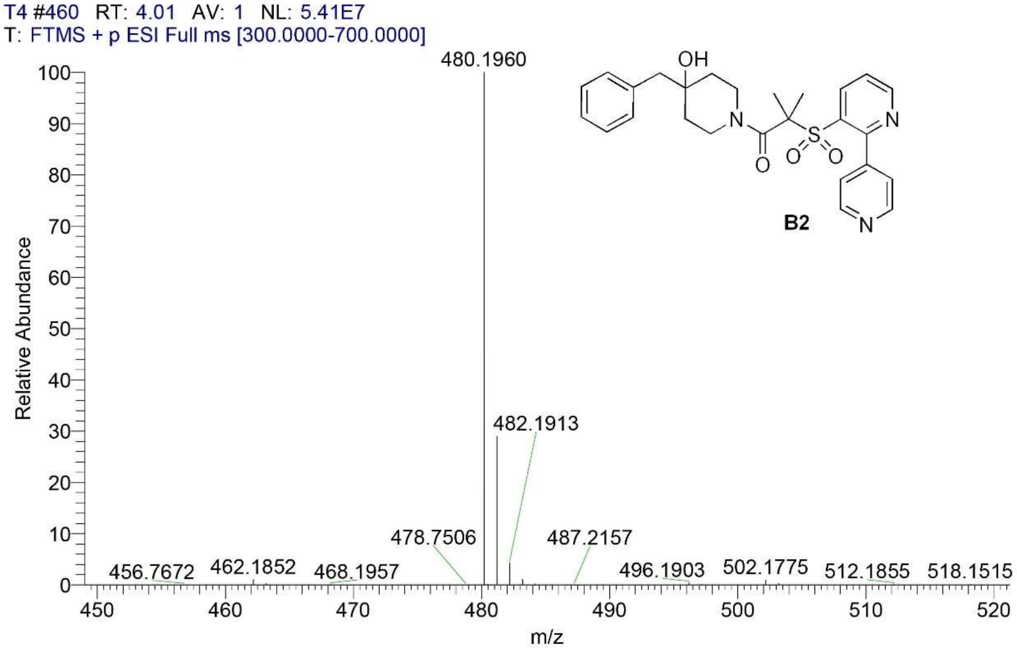

Figure S92: HR-MS (ESI/ion trap) spectrum of **B2**

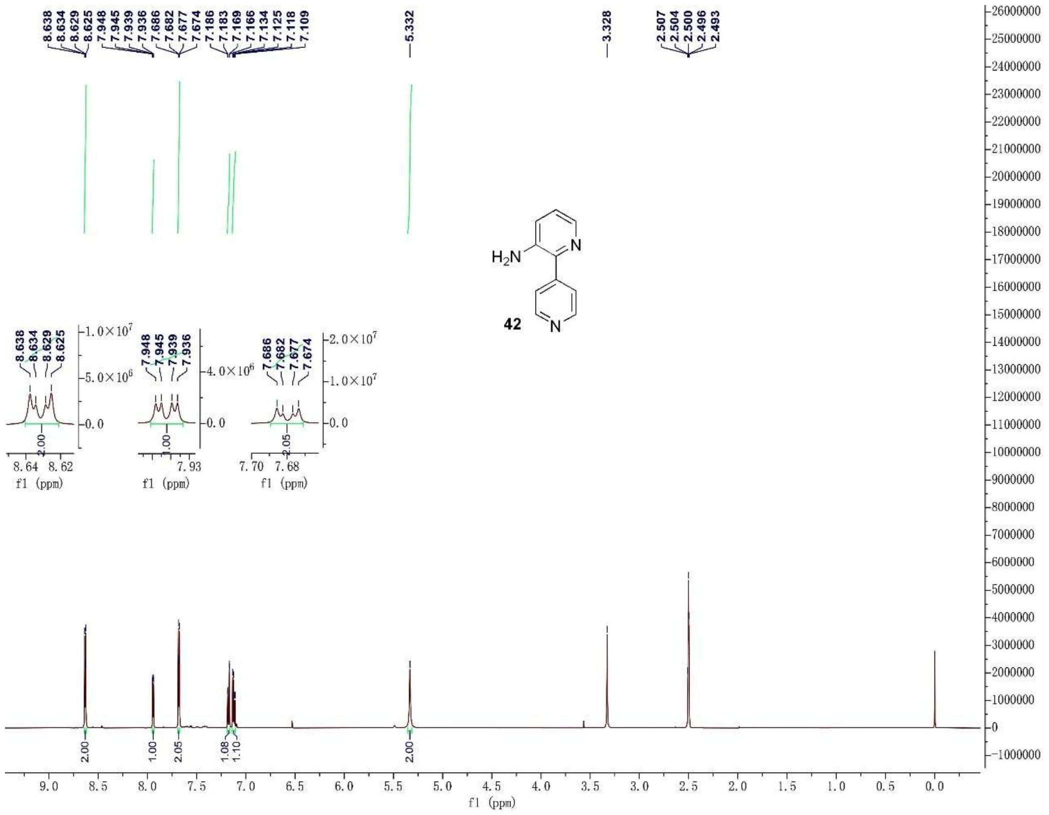

Figure S93: ^{1}H NMR spectrum of **42**

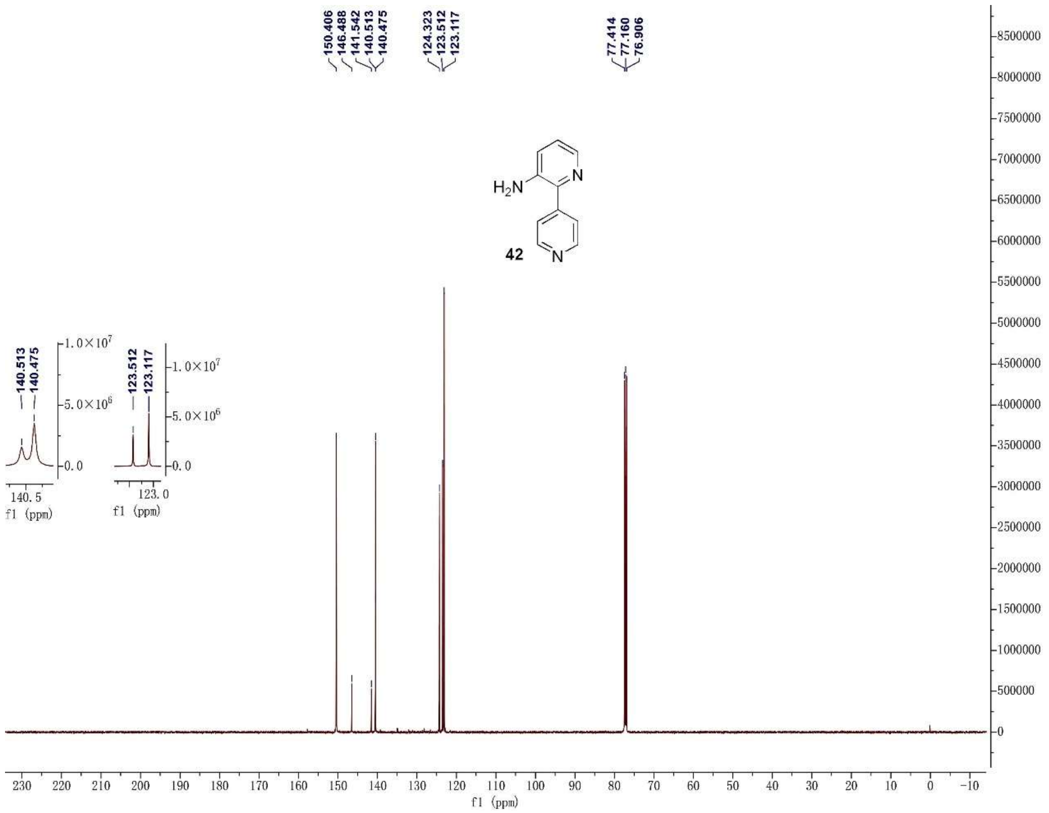

Figure S94: ^{13}C NMR spectrum of **42**

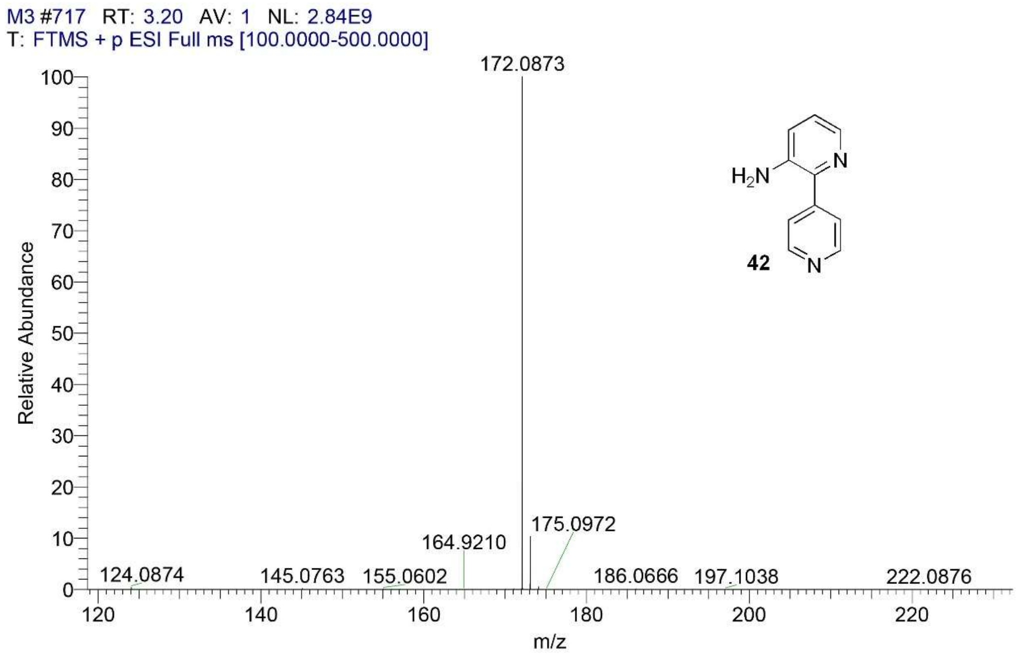

Figure S95: HR-MS (ESI/ion trap) spectrum of **42**

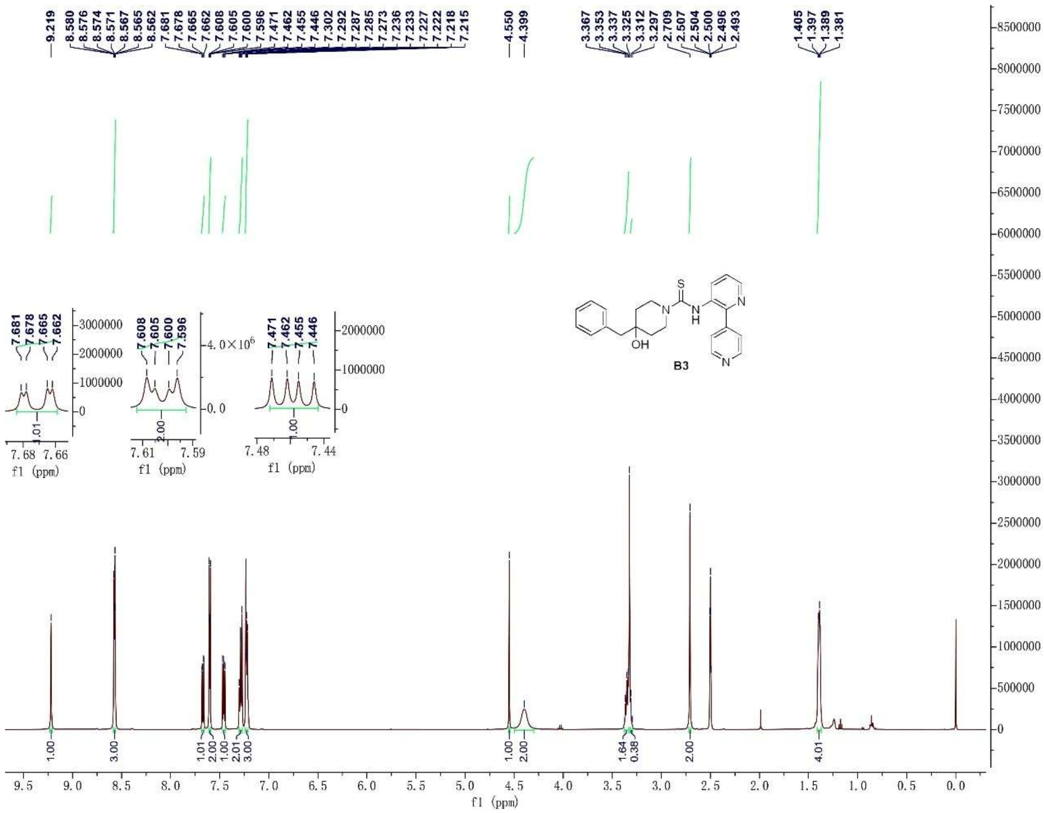

Figure S96: ^{1}H NMR spectrum of **B3**

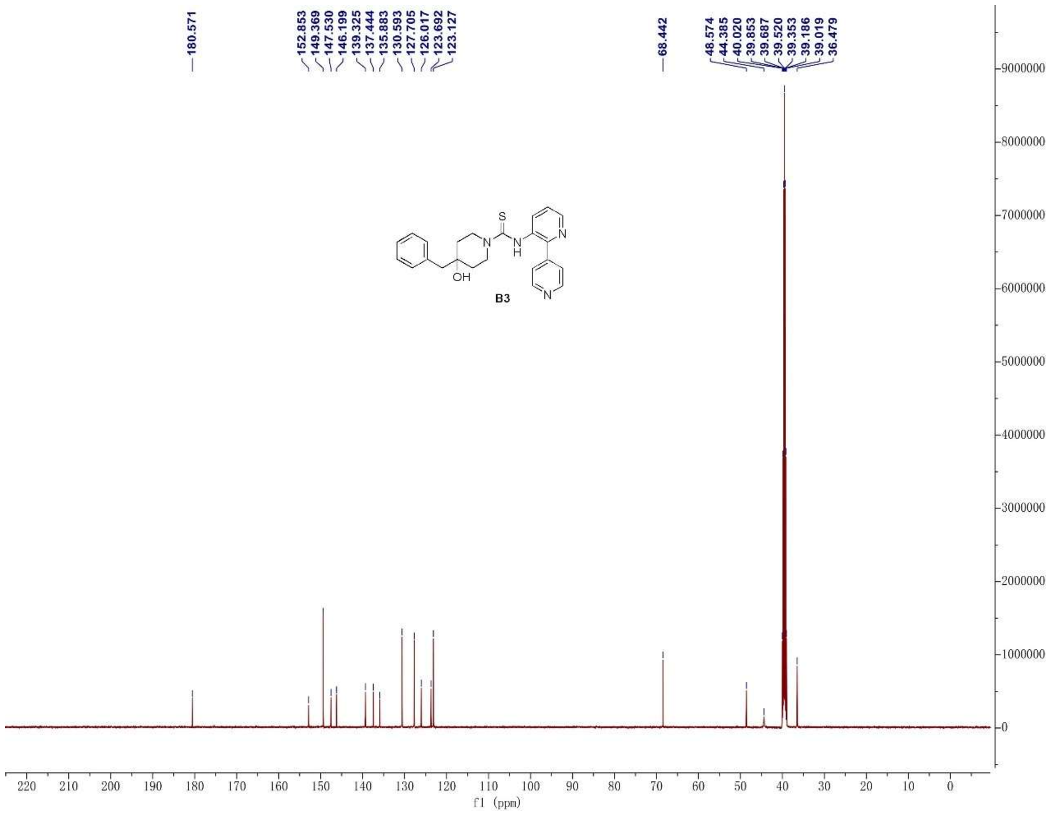

Figure S97: ^{13}C NMR spectrum of **B3**

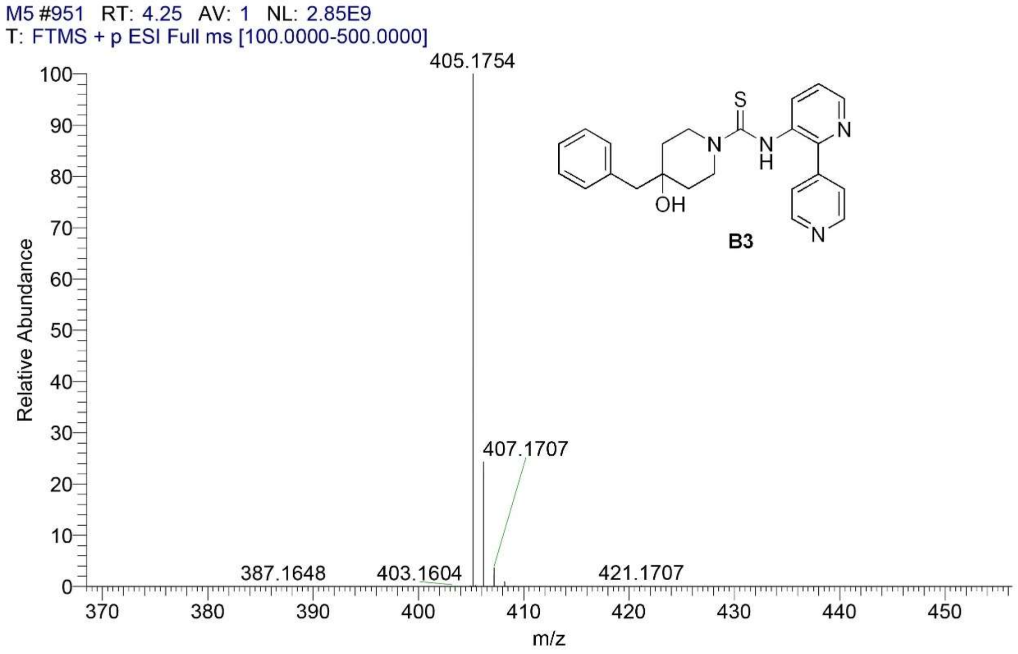

Figure S98: HR-MS (ESI/ion trap) spectrum of **B3**

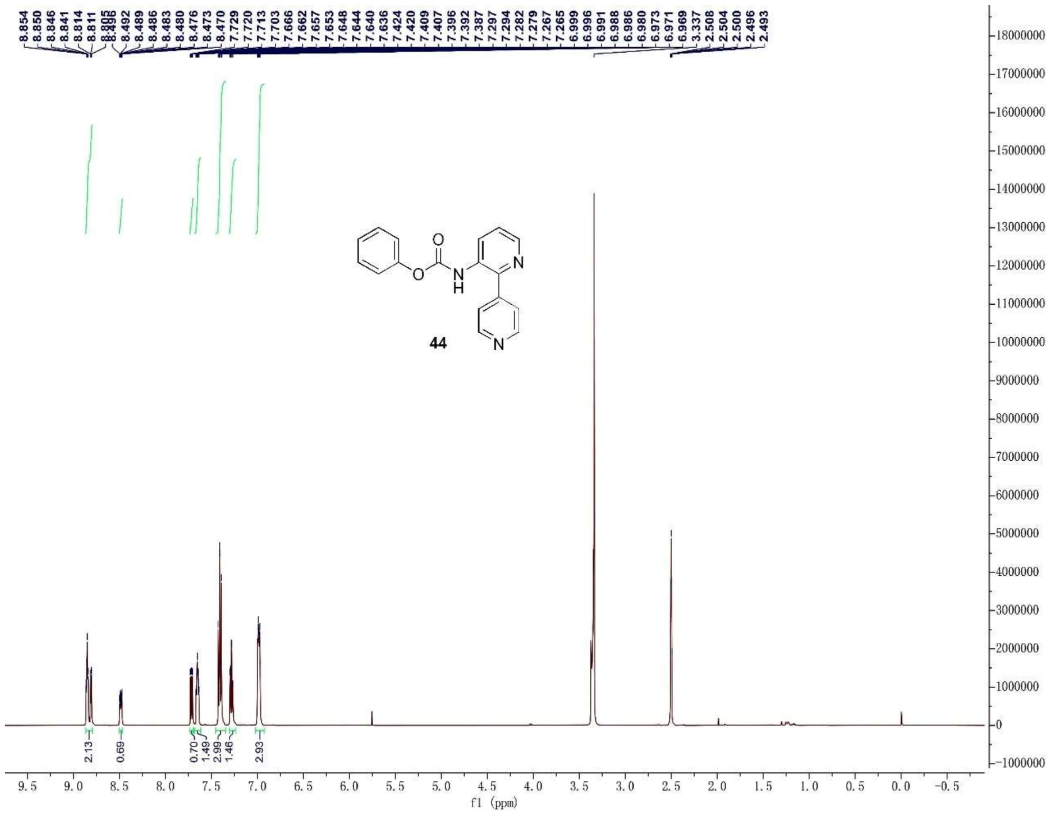

Figure S99: ^{1}H NMR spectrum of **44**

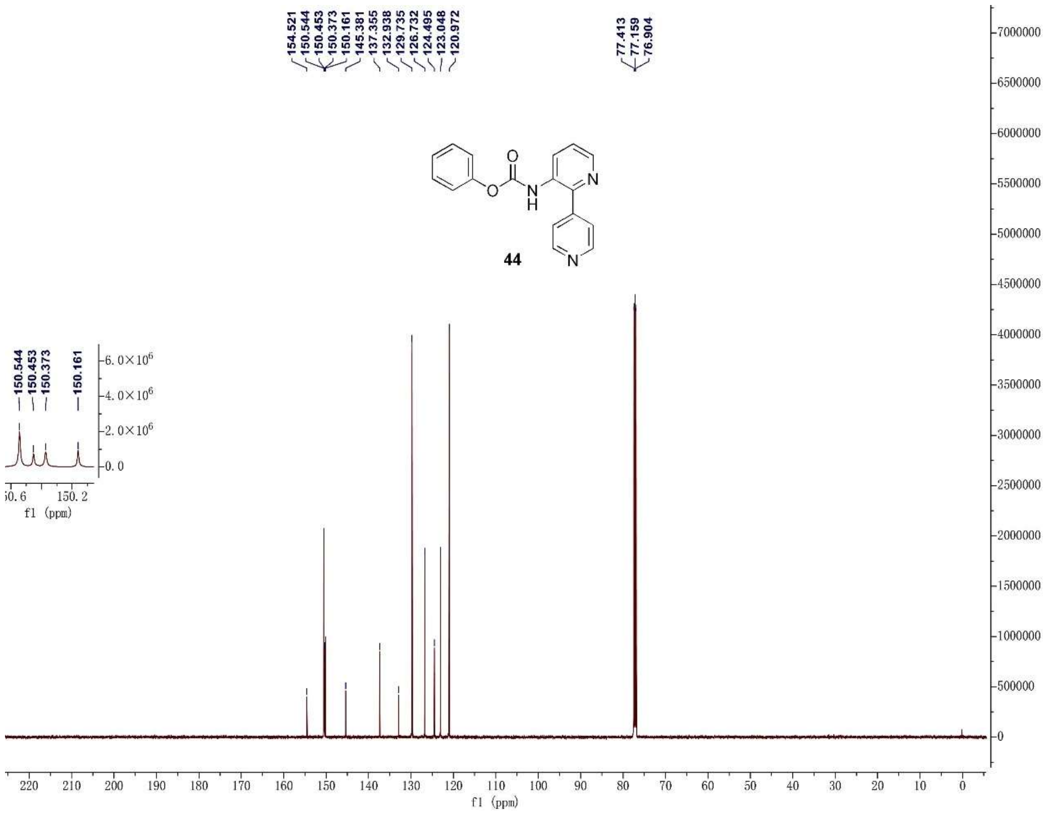

Figure S100: ^{13}C NMR spectrum of **44**

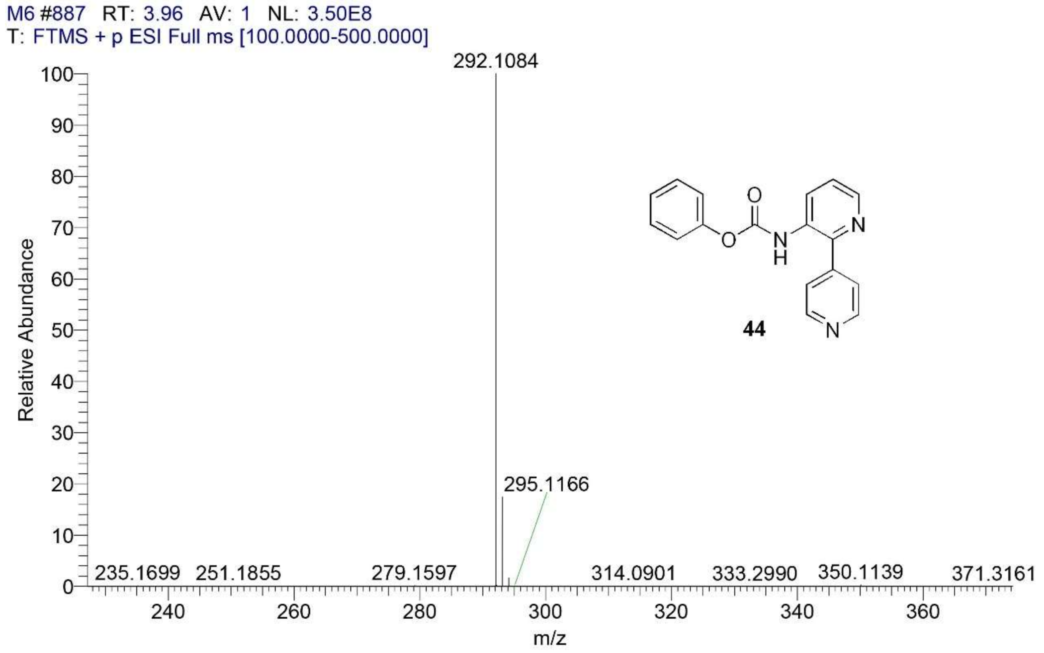

Figure S101: HR-MS (ESI/ion trap) spectrum of **44**

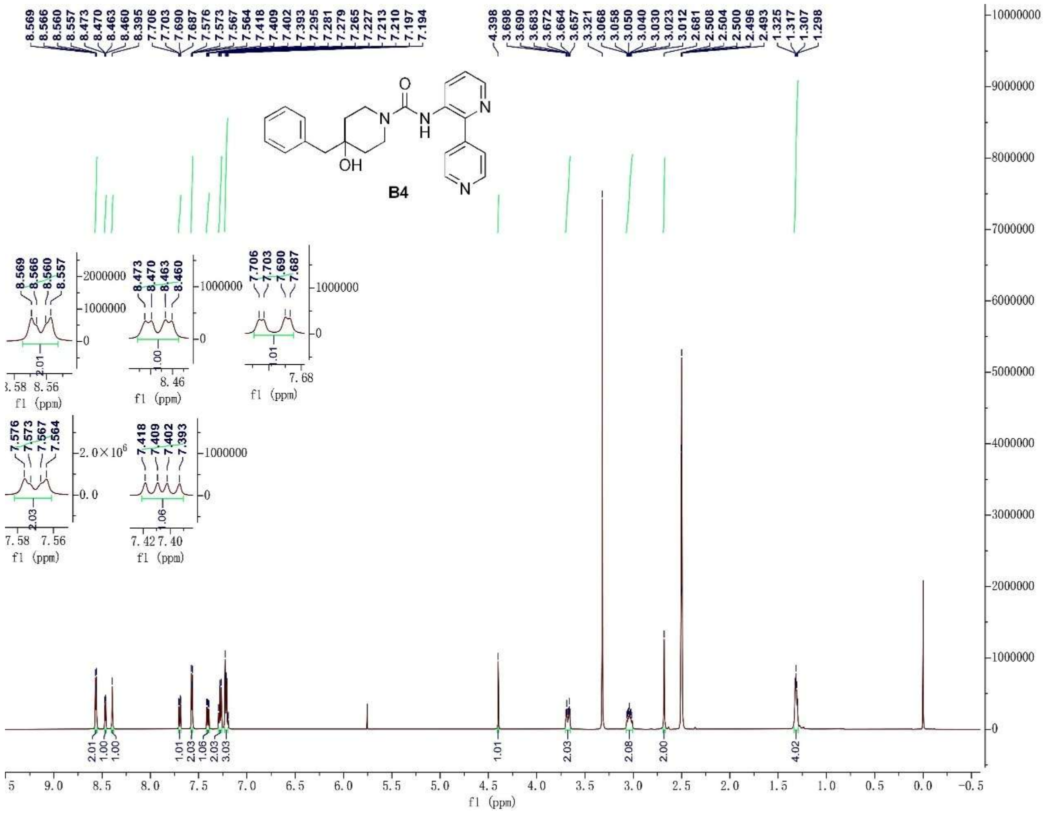

Figure S102: ^{1}H NMR spectrum of **B4**

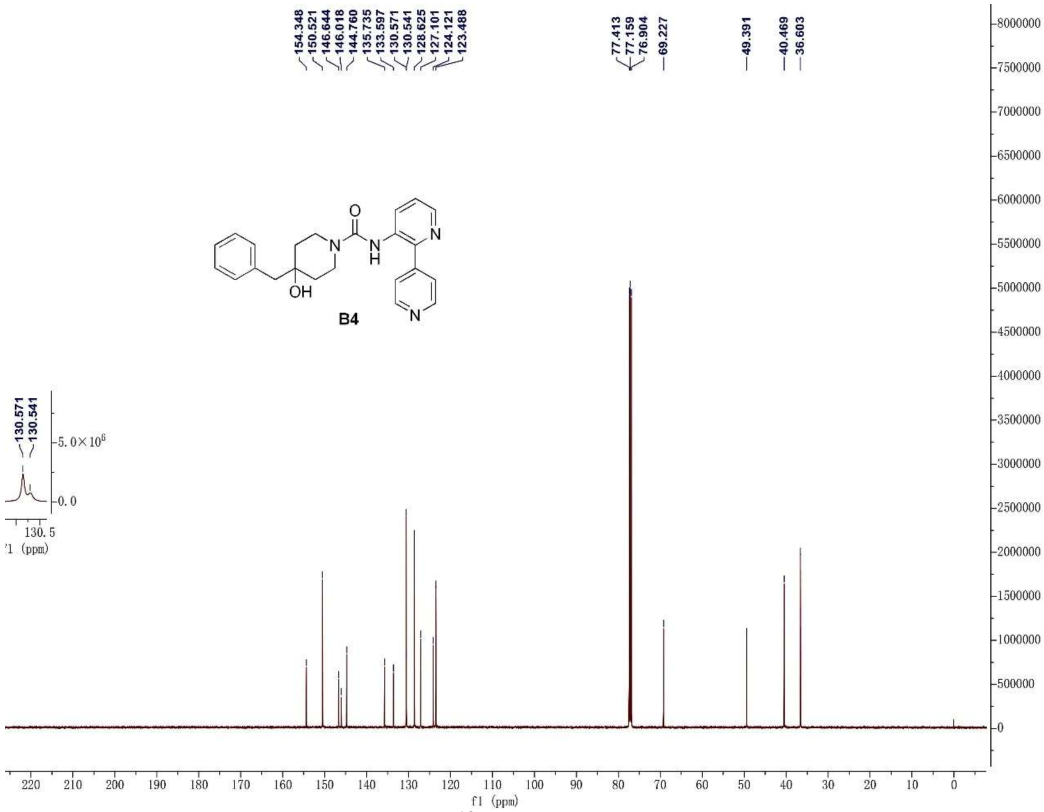

Figure S103: ^{13}C NMR spectrum of **B4**

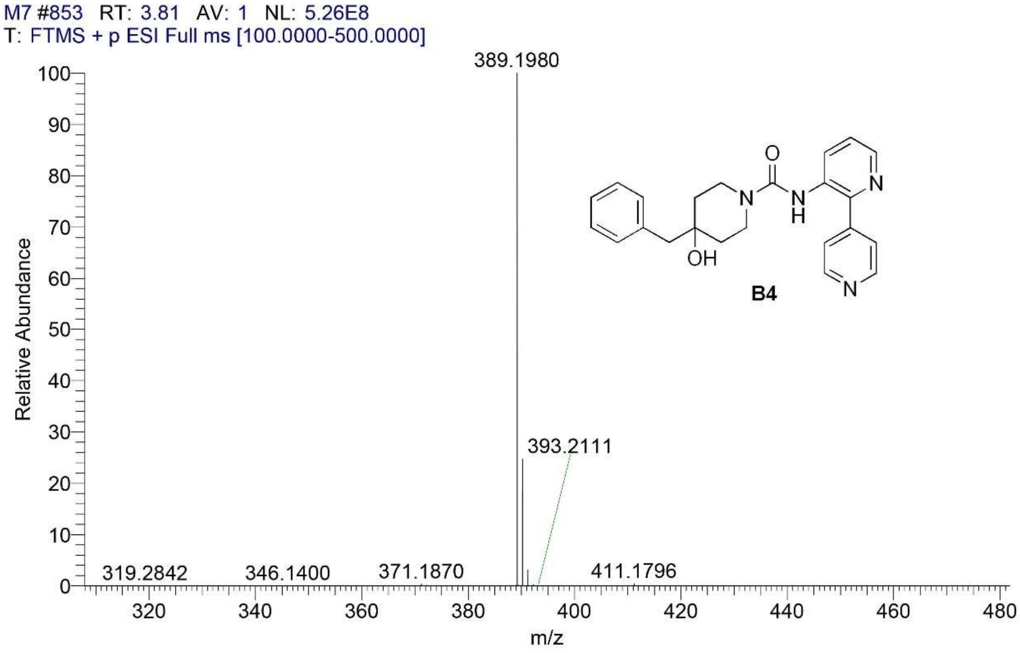

Figure S104: HR-MS (ESI/ion trap) spectrum of **B4**

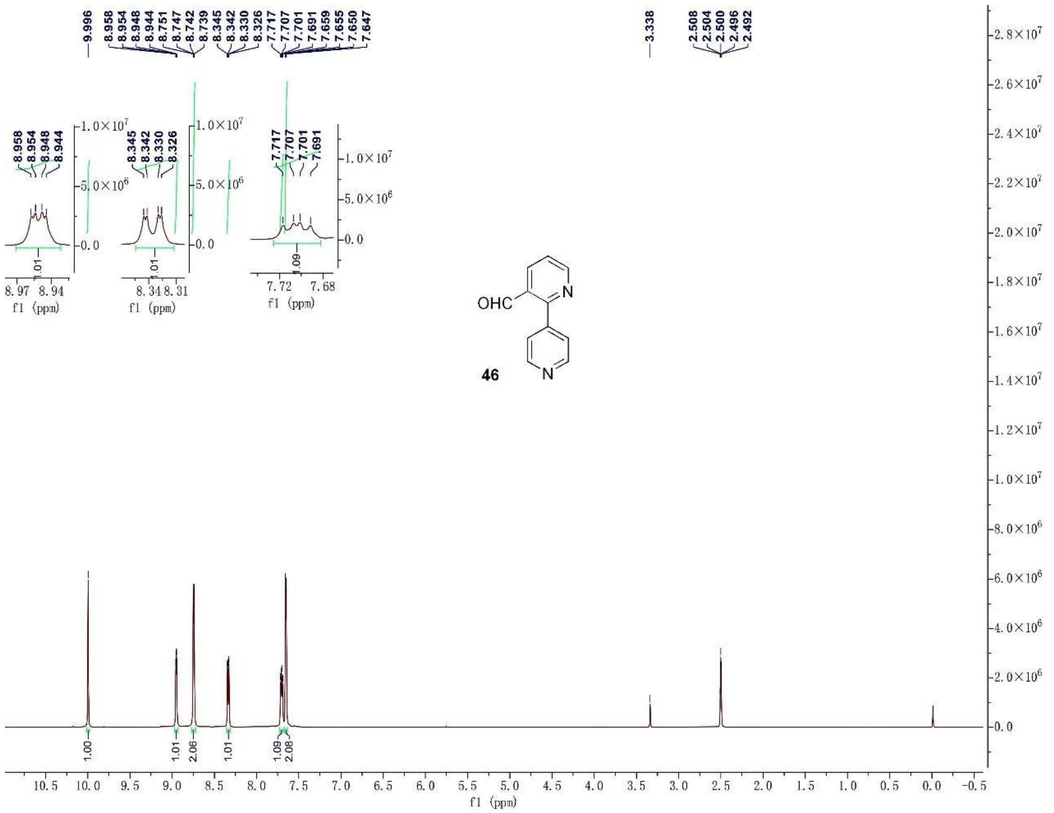

Figure S105: ^{1}H NMR spectrum of **46**

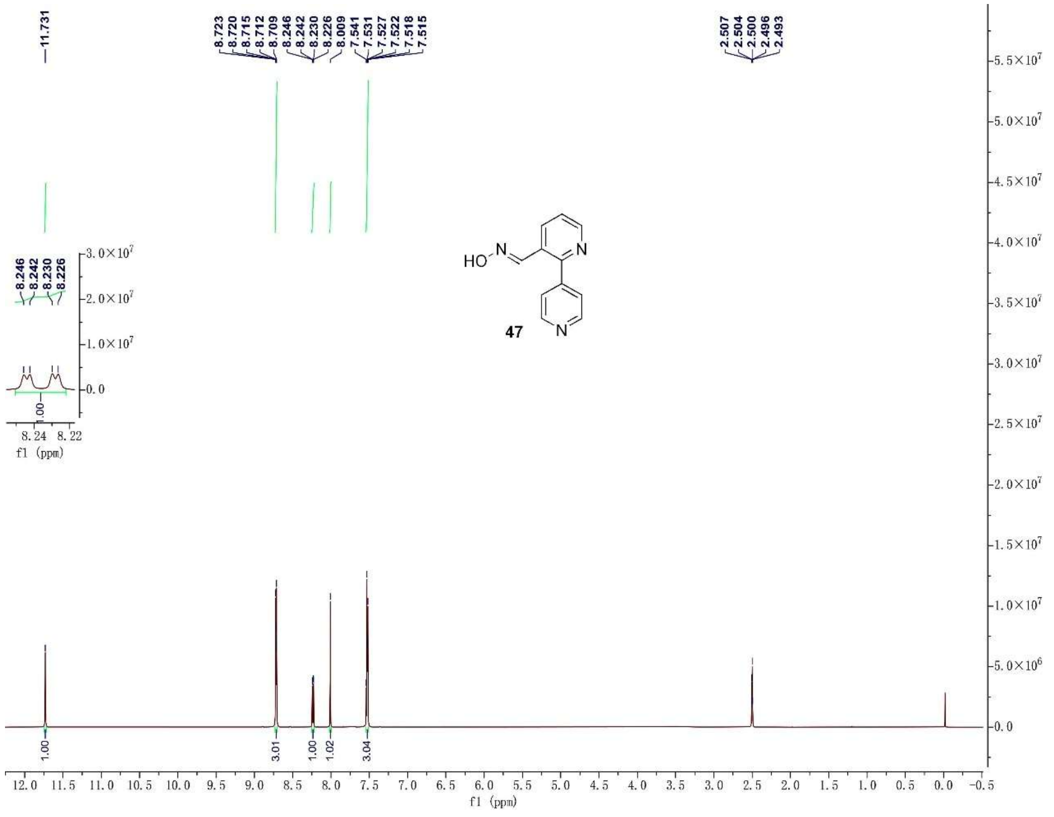

Figure S106: ^{1}H NMR spectrum of **47**

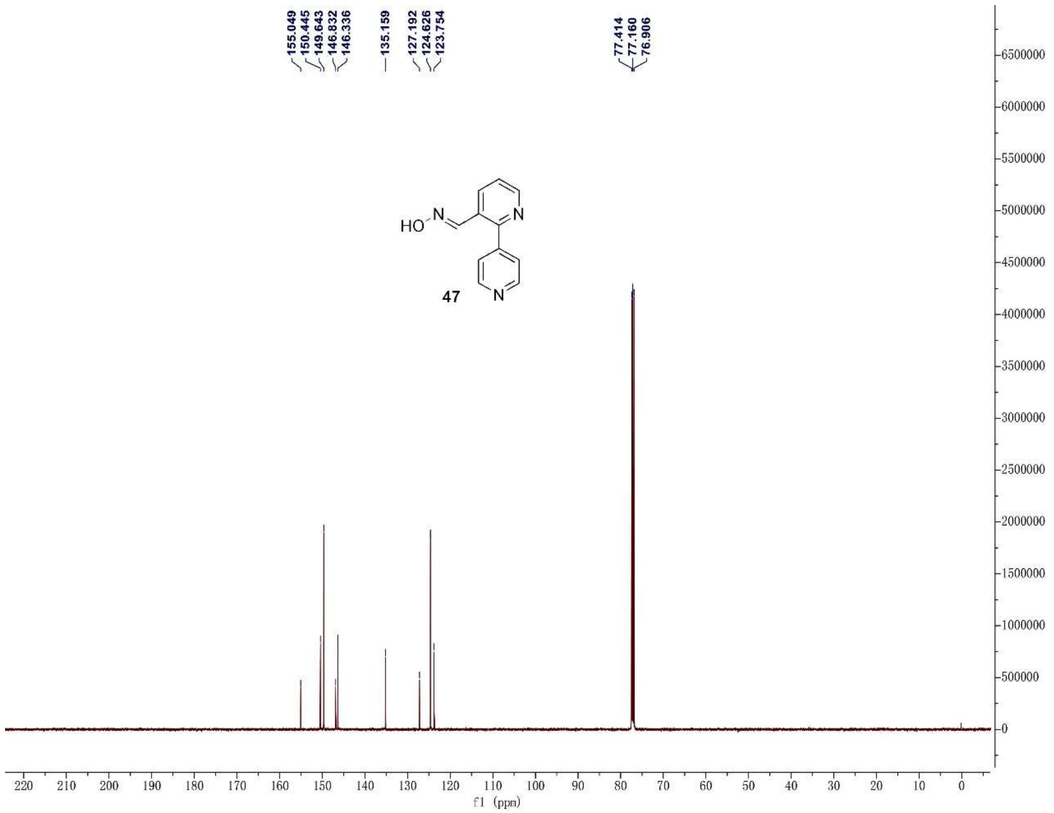

Figure S107: ^{13}C NMR spectrum of **47**

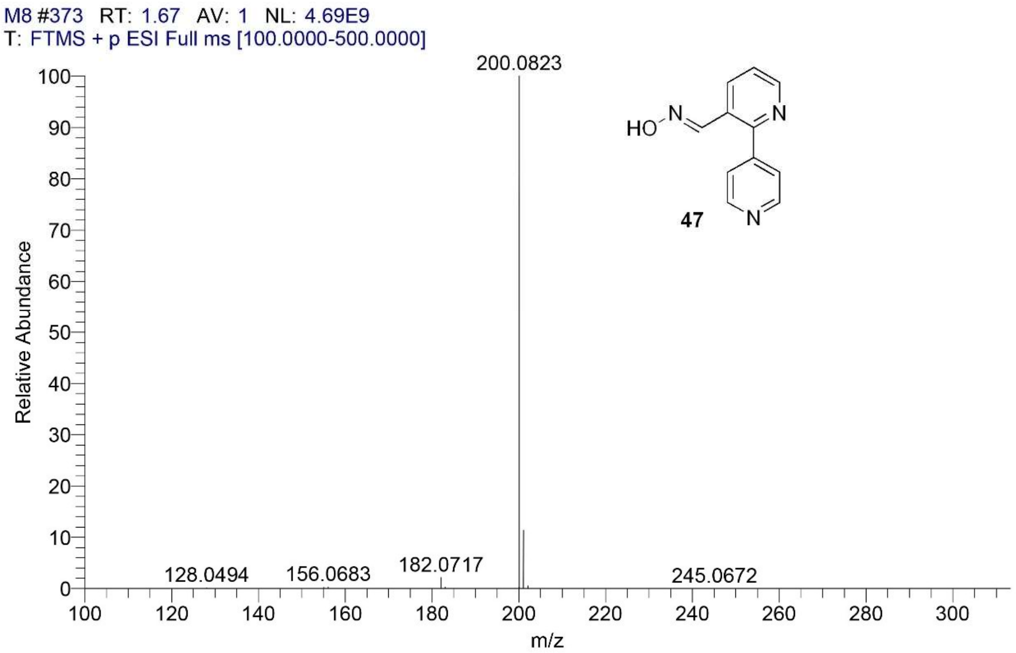

Figure S108: HR-MS (ESI/ion trap) spectrum of **47**

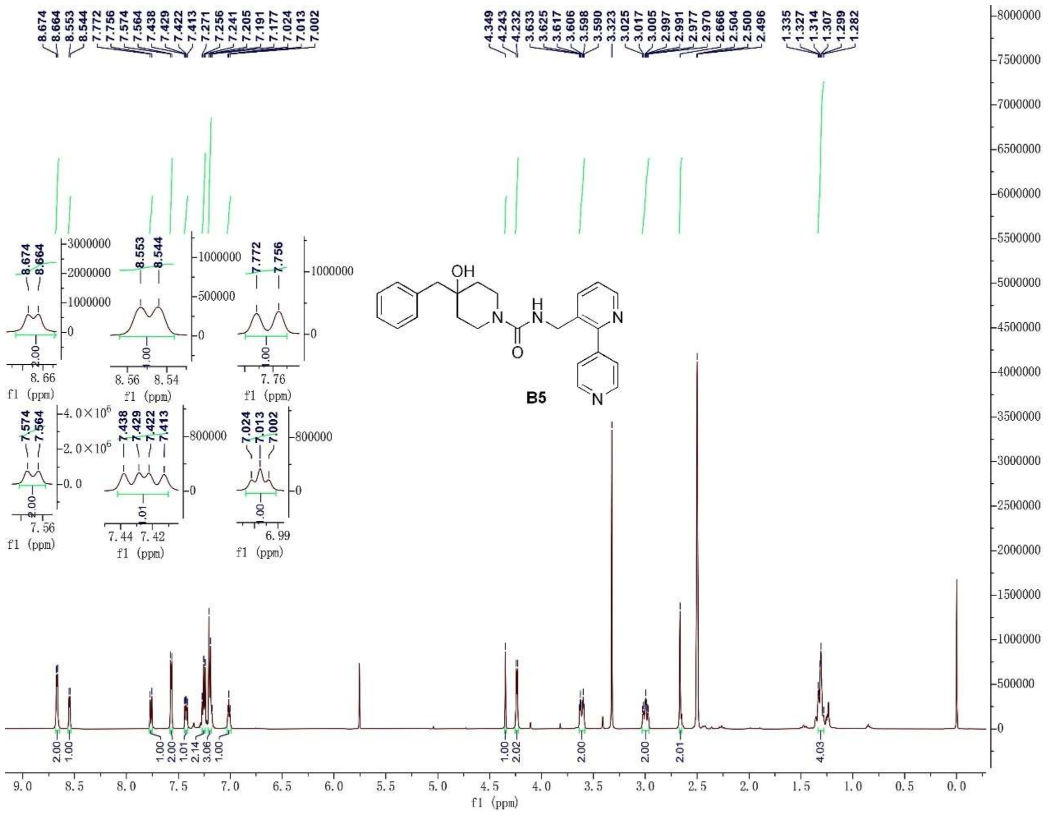

Figure S109: ^{1}H NMR spectrum of **B5**

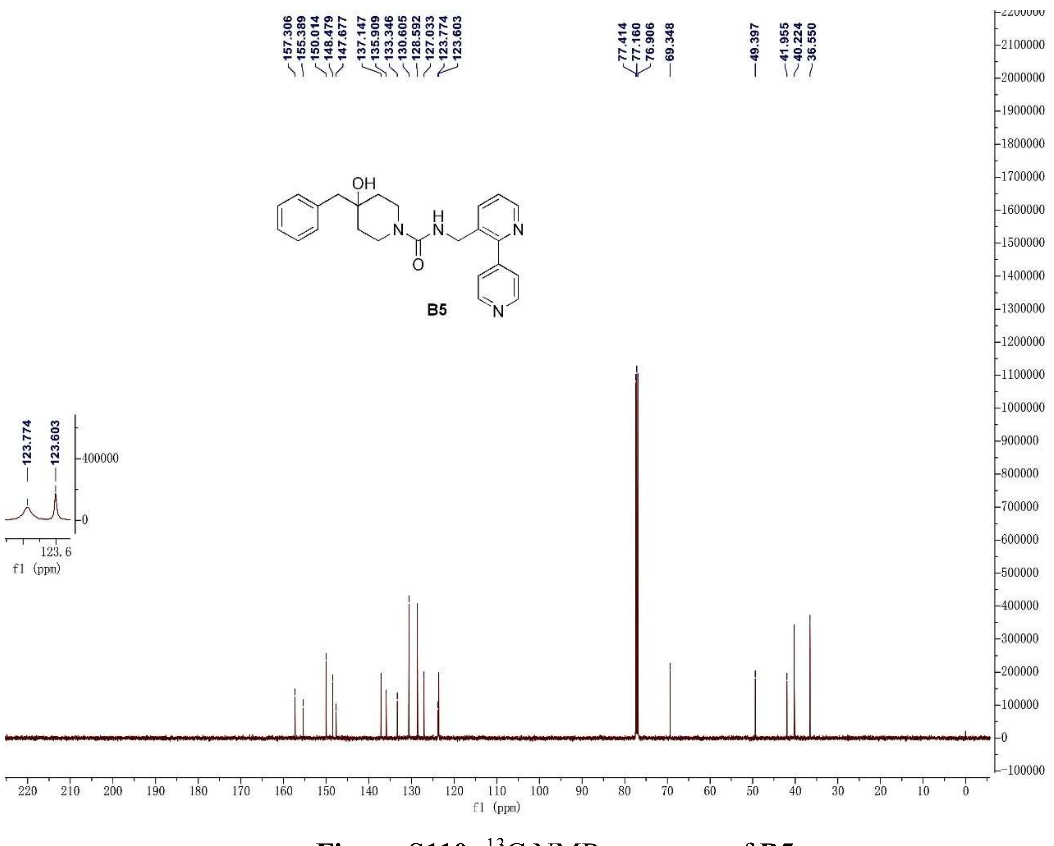

Figure S110: ^{13}C NMR spectrum of **B5**

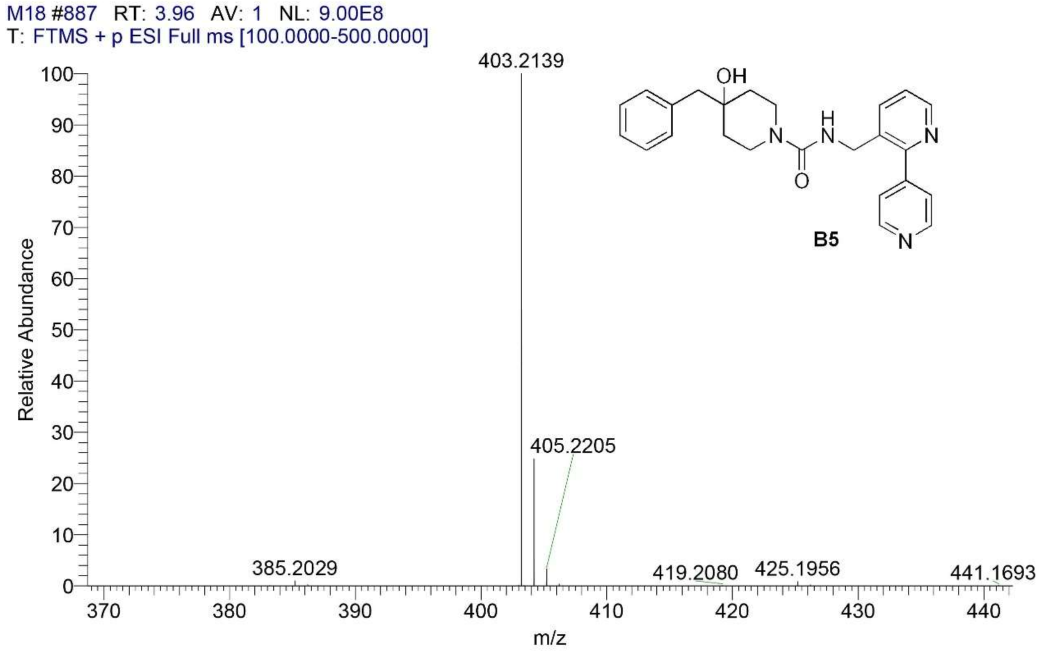

Figure S111: HR-MS (ESI/ion trap) spectrum of **B5**

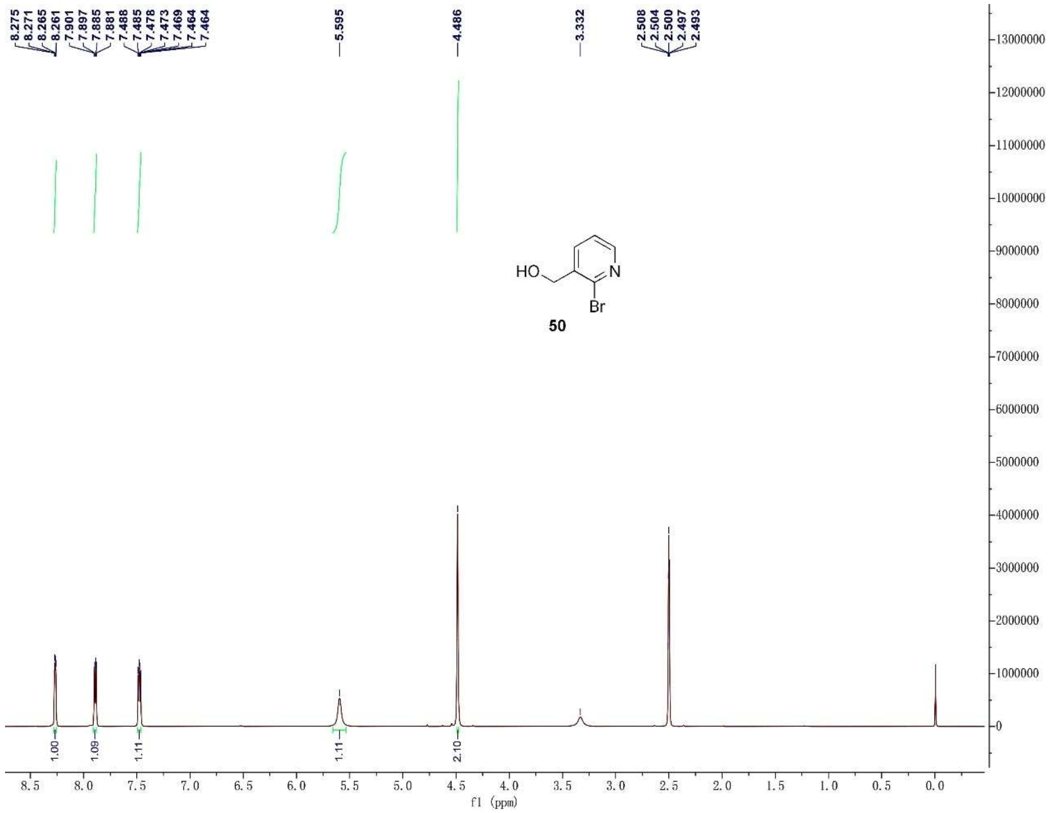

Figure S112: ^{1}H NMR spectrum of **50**

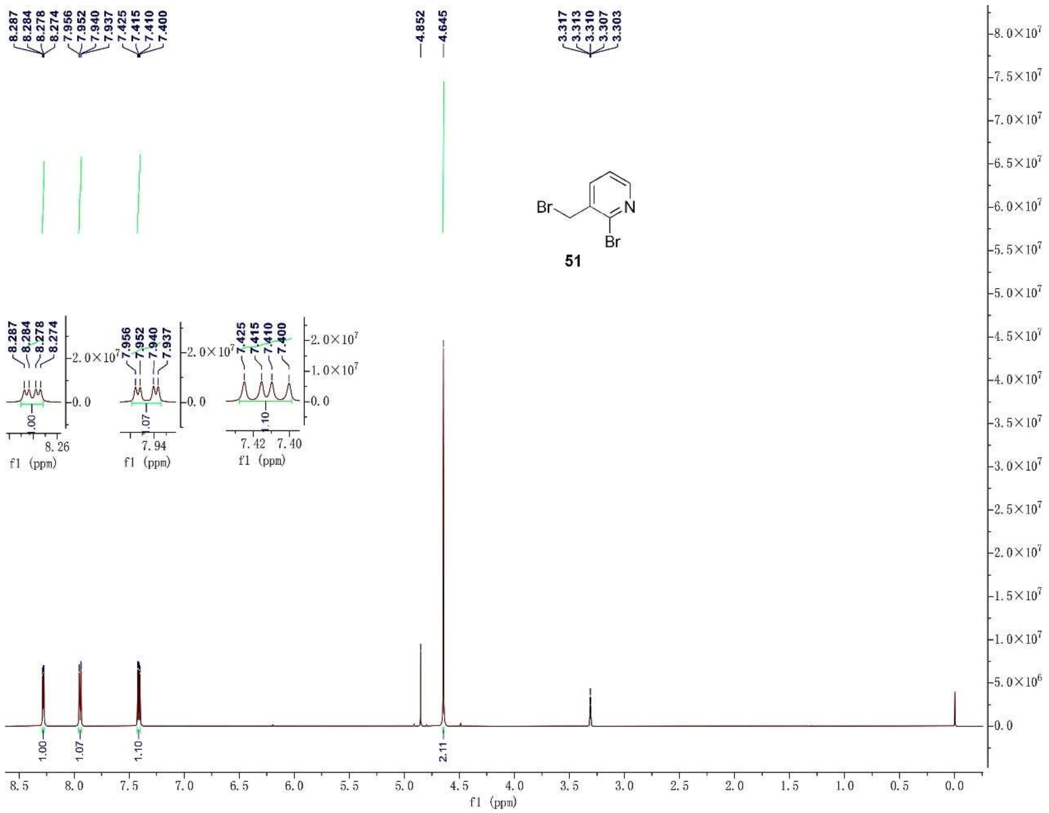

Figure S113: ^{1}H NMR spectrum of **51**

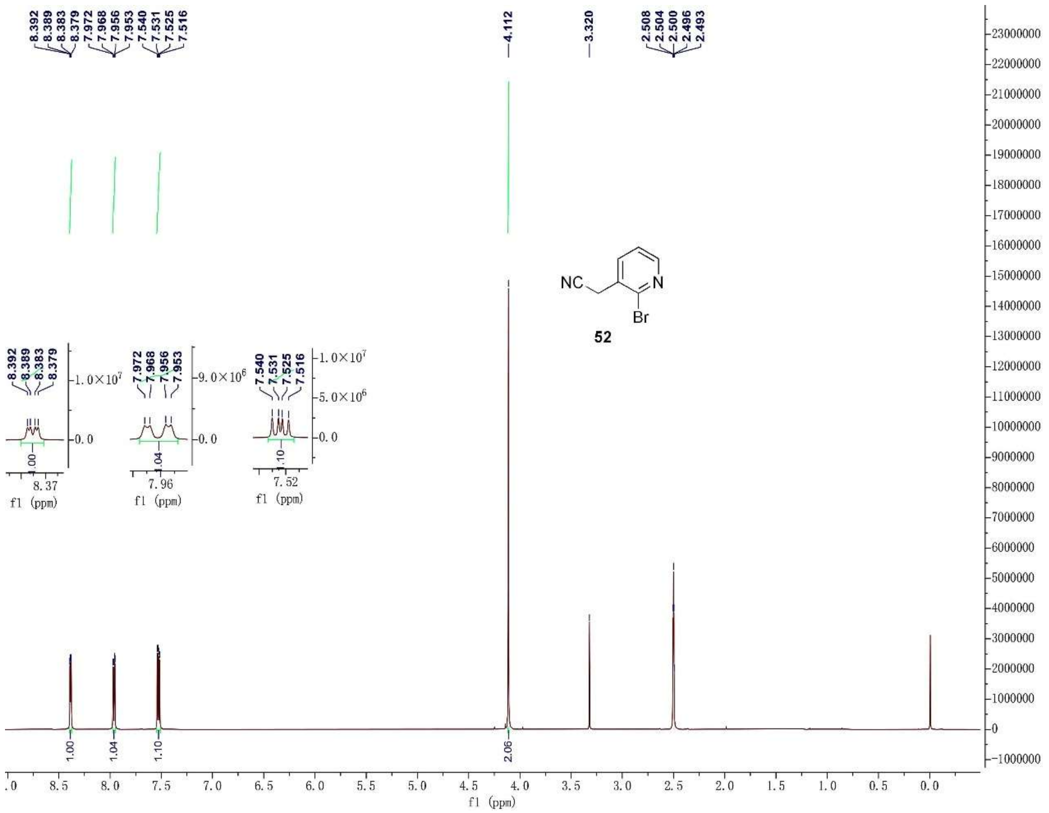

Figure S114: ^{1}H NMR spectrum of **52**

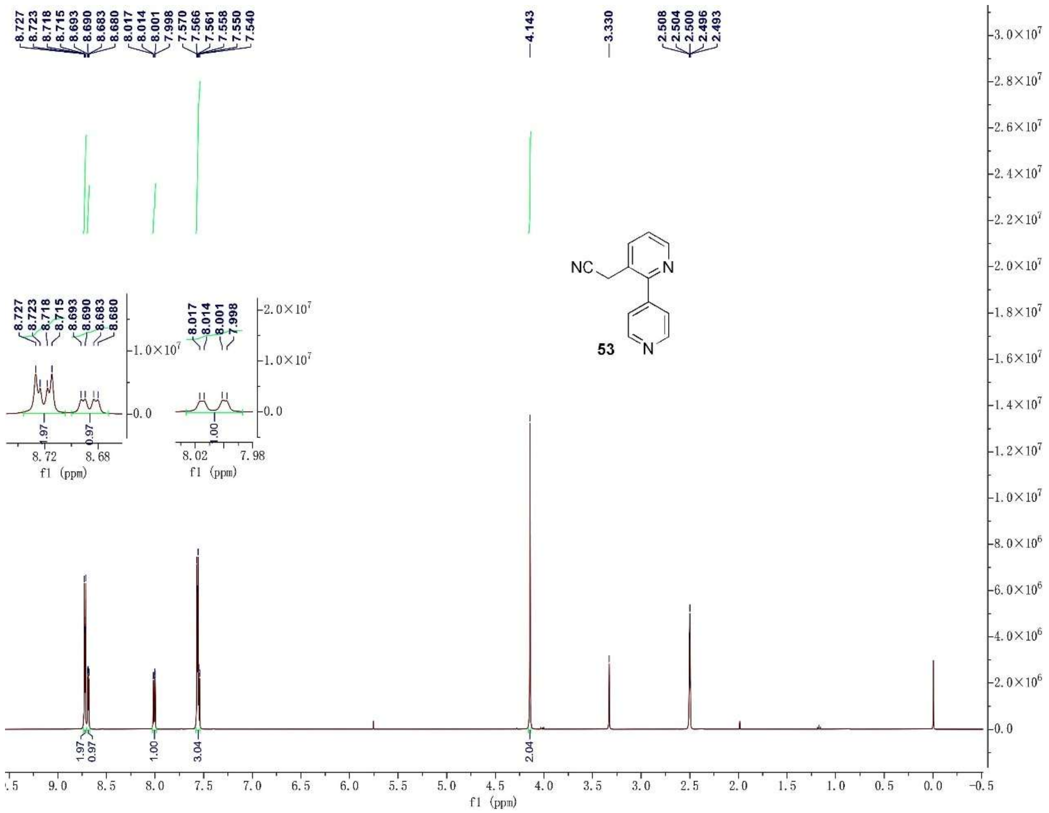

Figure S115: ^{1}H NMR spectrum of **53**

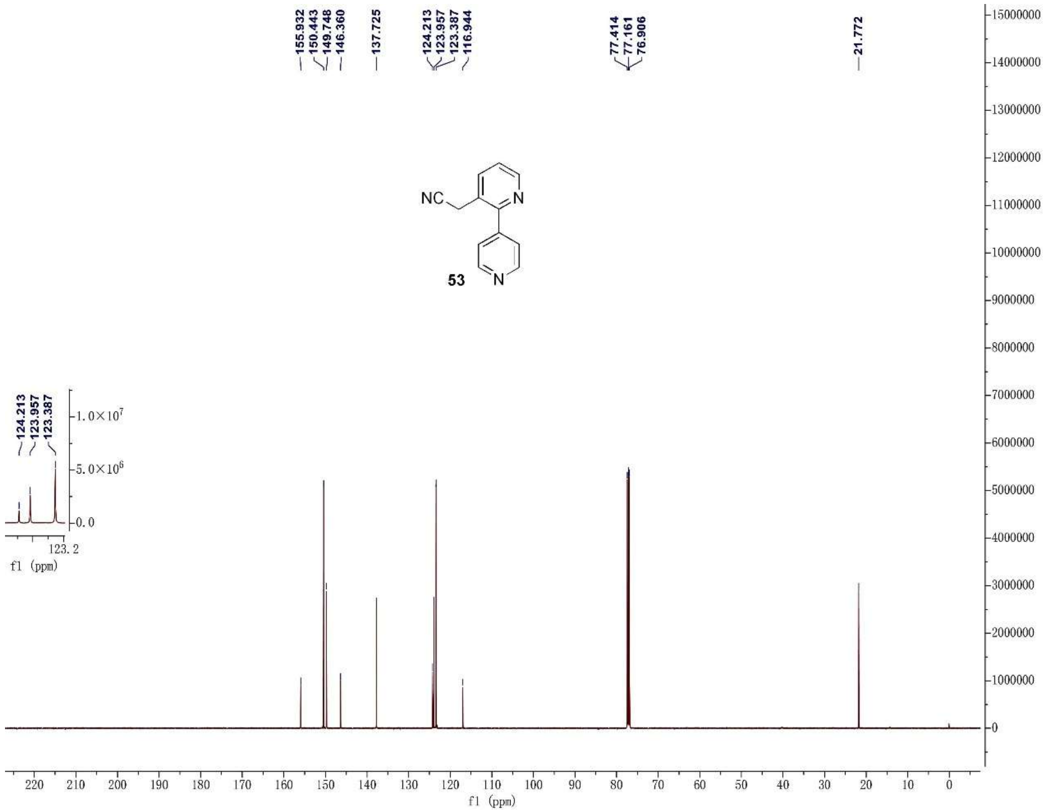

Figure S116: ^{13}C NMR spectrum of **53**

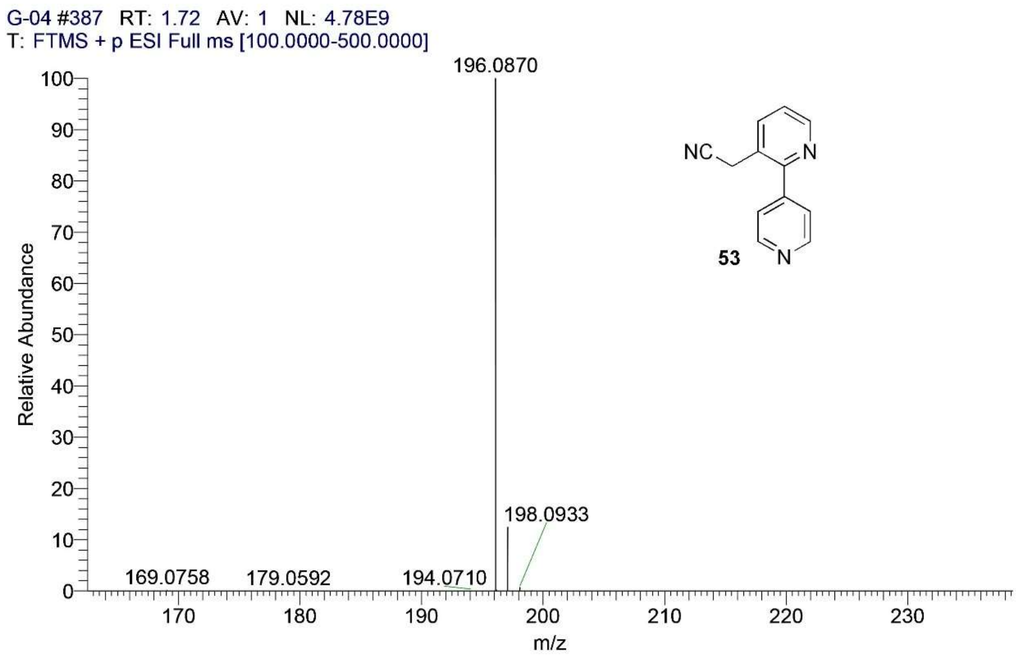

Figure S117: HR-MS (ESI/ion trap) spectrum of **53**

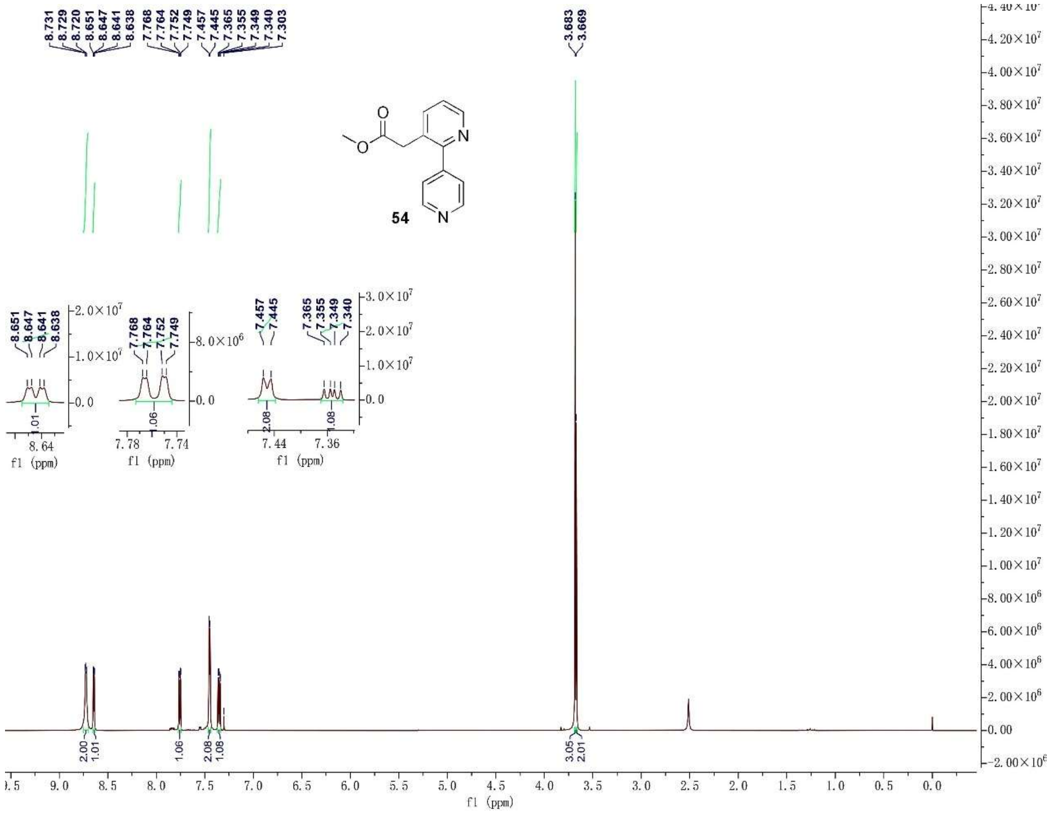

Figure S118: ^{1}H NMR spectrum of **54**

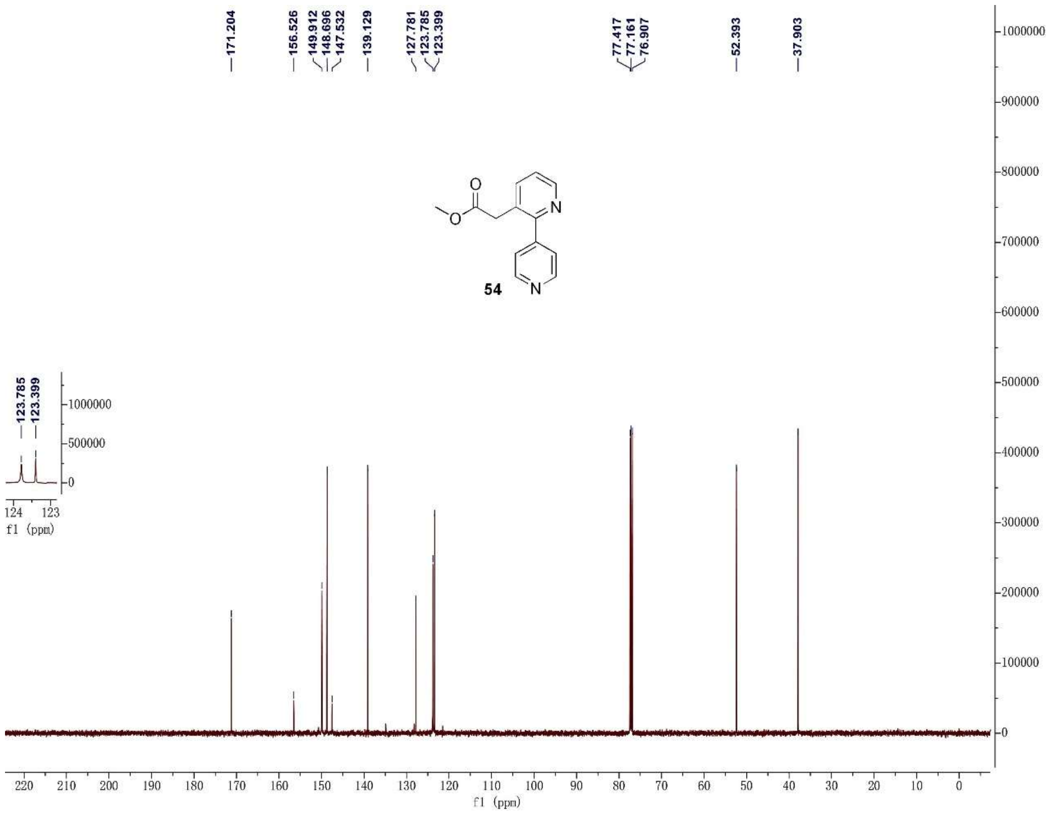

Figure S119: ^{13}C NMR spectrum of **54**

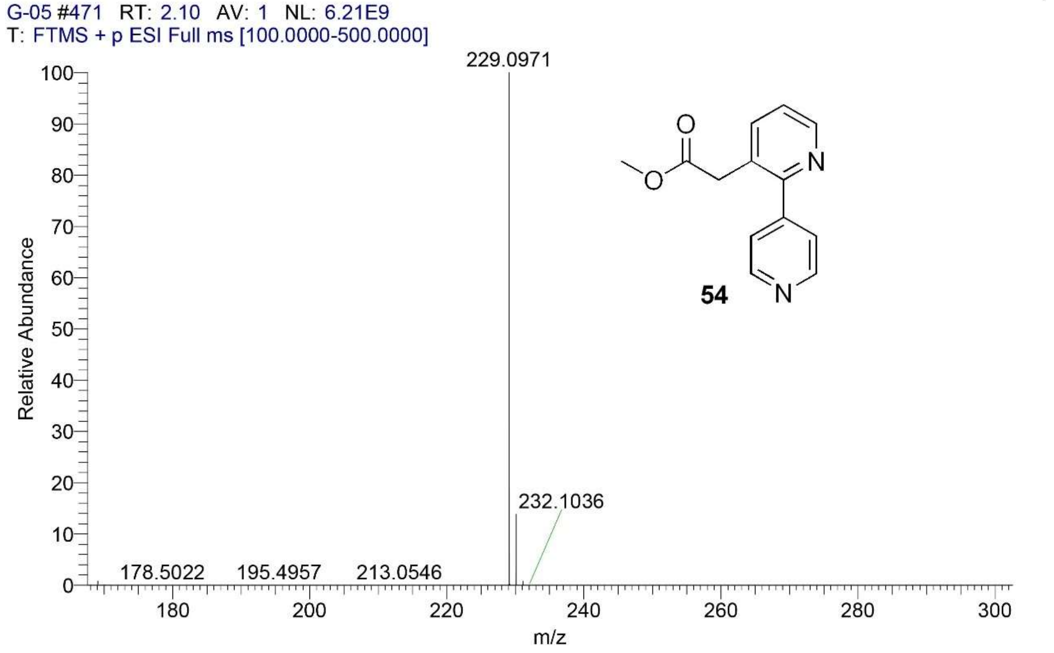

Figure S120: HR-MS (ESI/ion trap) spectrum of **54**

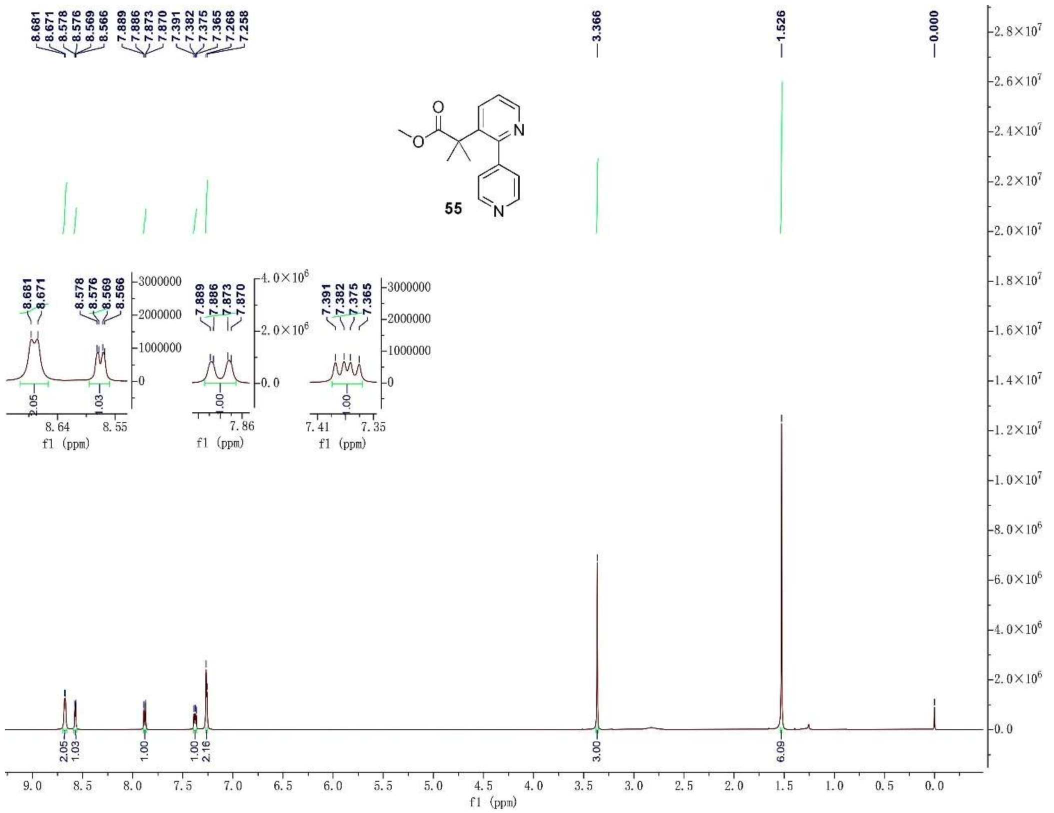

Figure S121: ^{1}H NMR spectrum of **55**

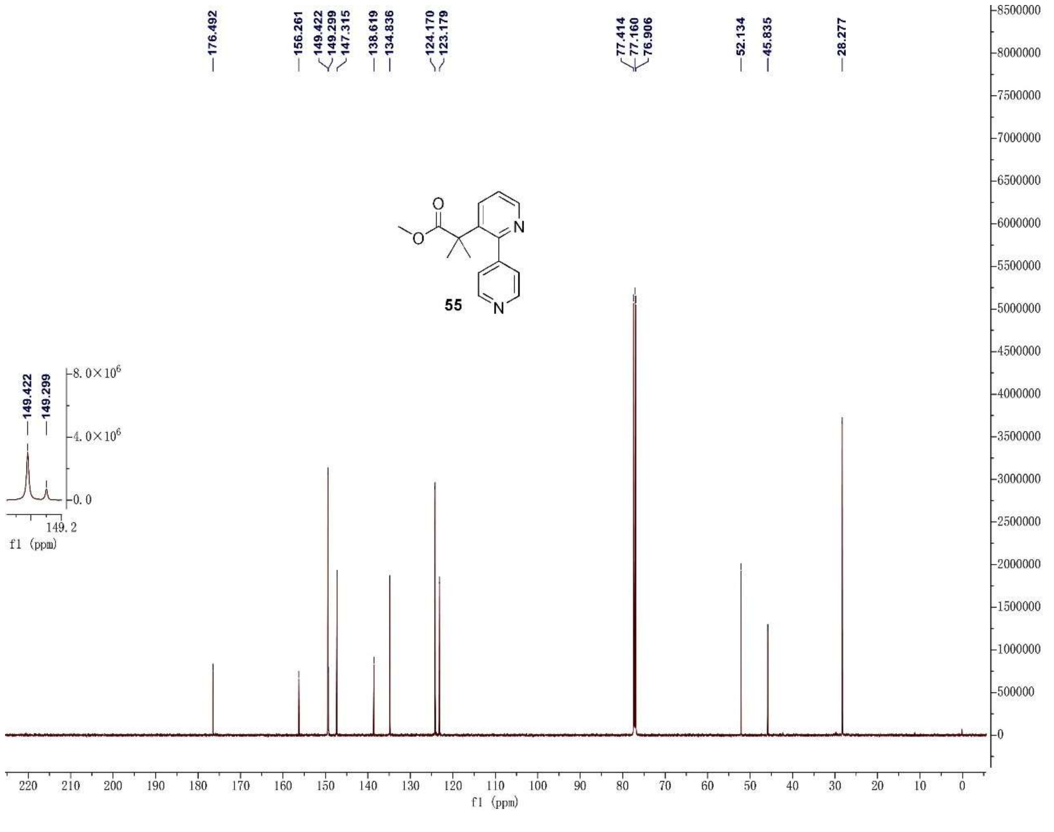

Figure S122: ^{13}C NMR spectrum of **55**

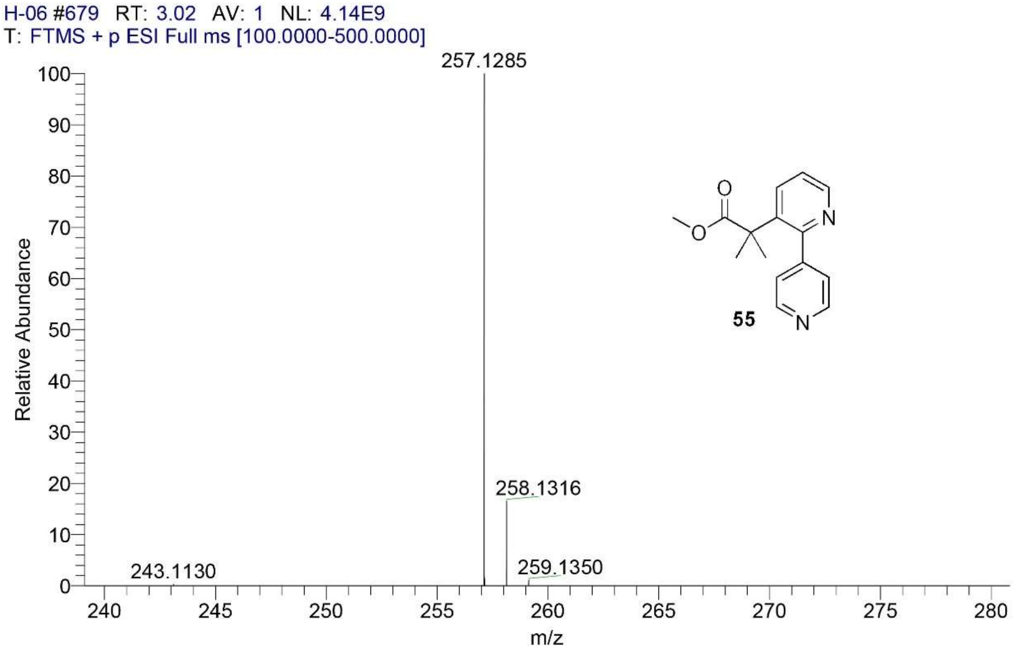

Figure S123: HR-MS (ESI/ion trap) spectrum of **55**

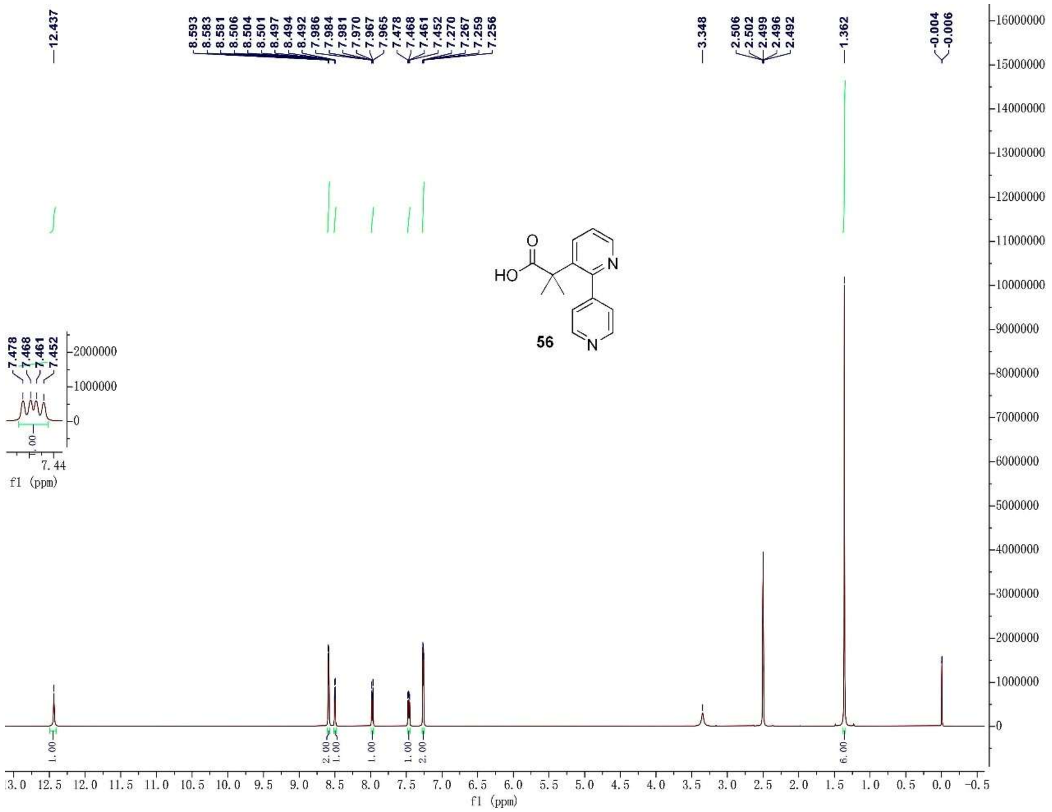

Figure S124: ^{1}H NMR spectrum of **56**

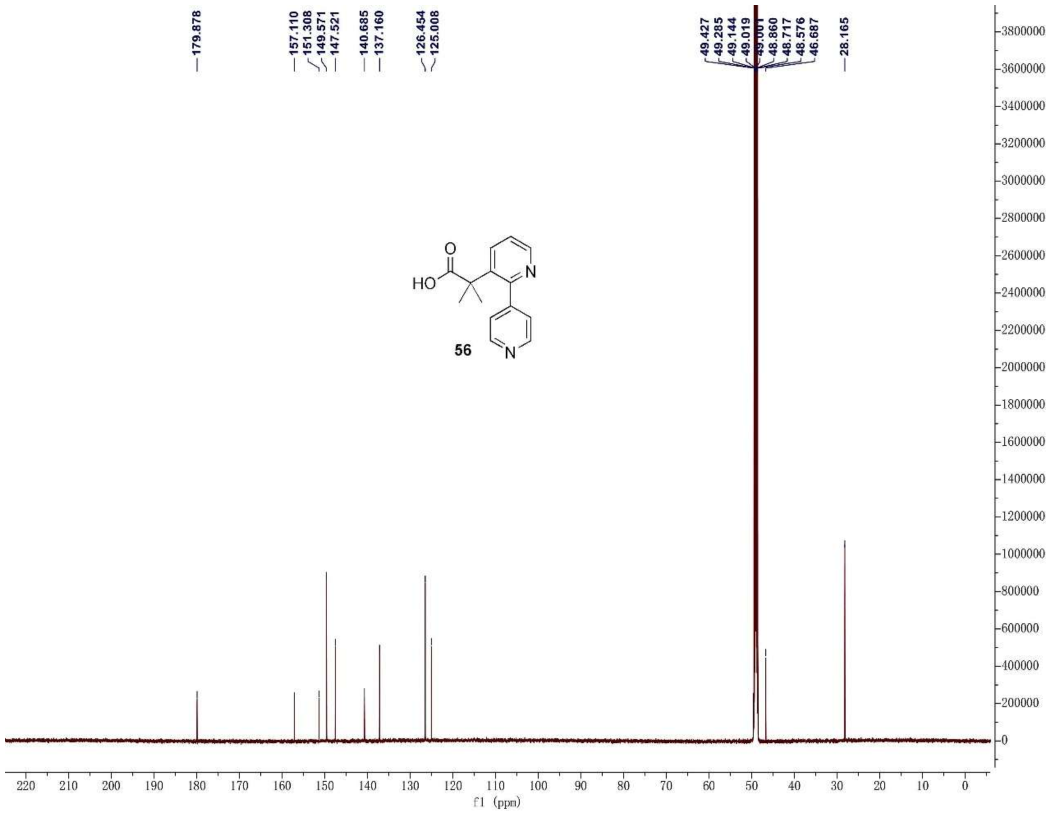

Figure S125: ^{13}C NMR spectrum of **56**

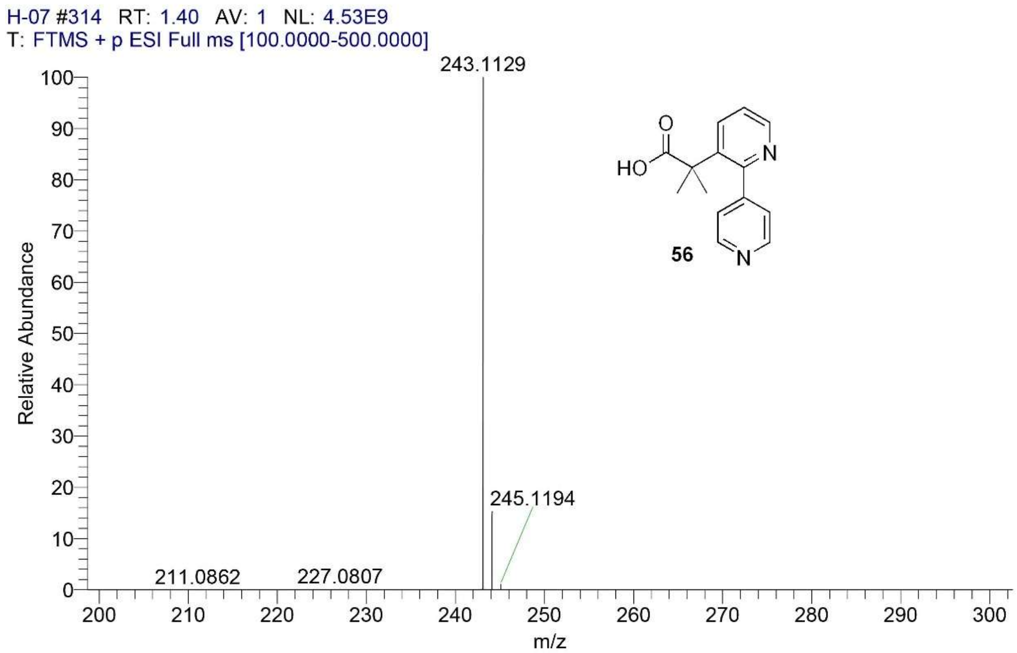

Figure S126: HR-MS (ESI/ion trap) spectrum of **56**

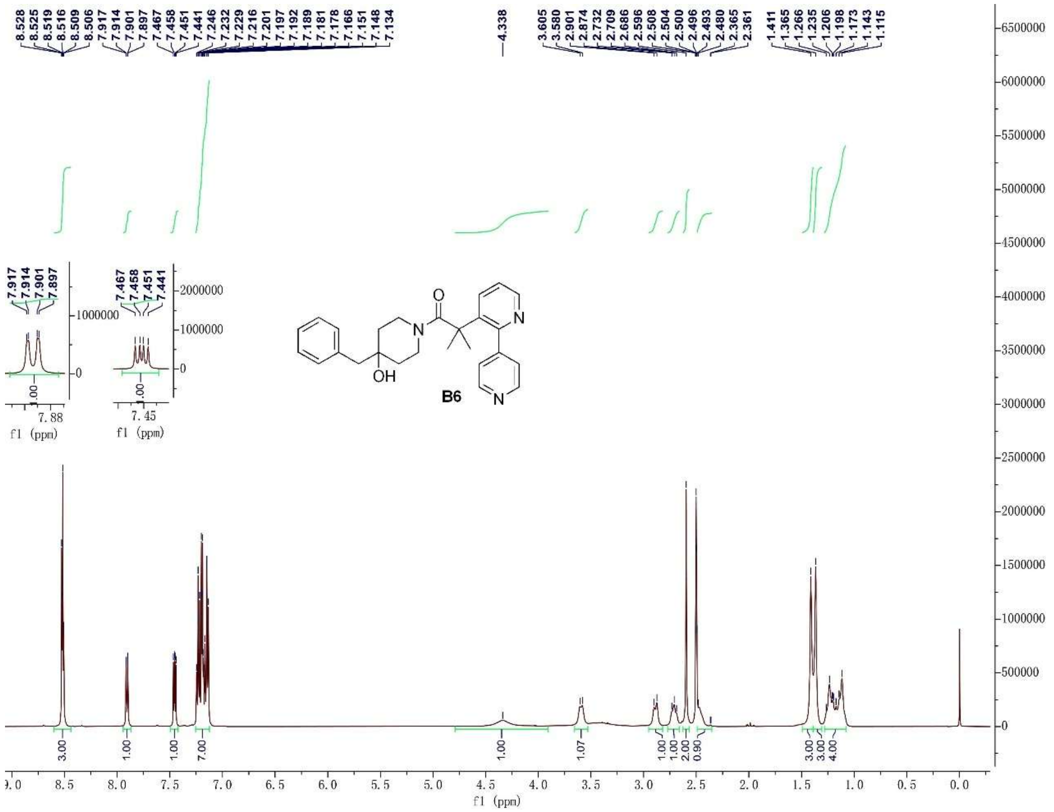

Figure S127: ^{1}H NMR spectrum of **B6**

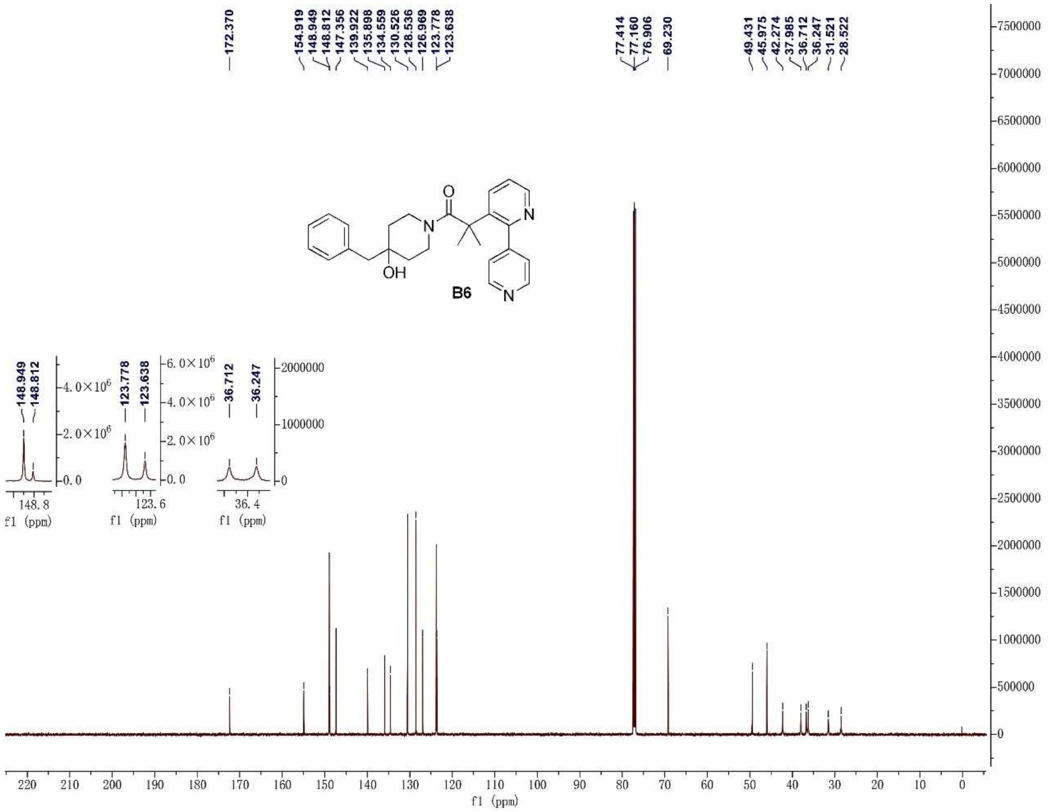

Figure S128: ^{13}C NMR spectrum of **B6**

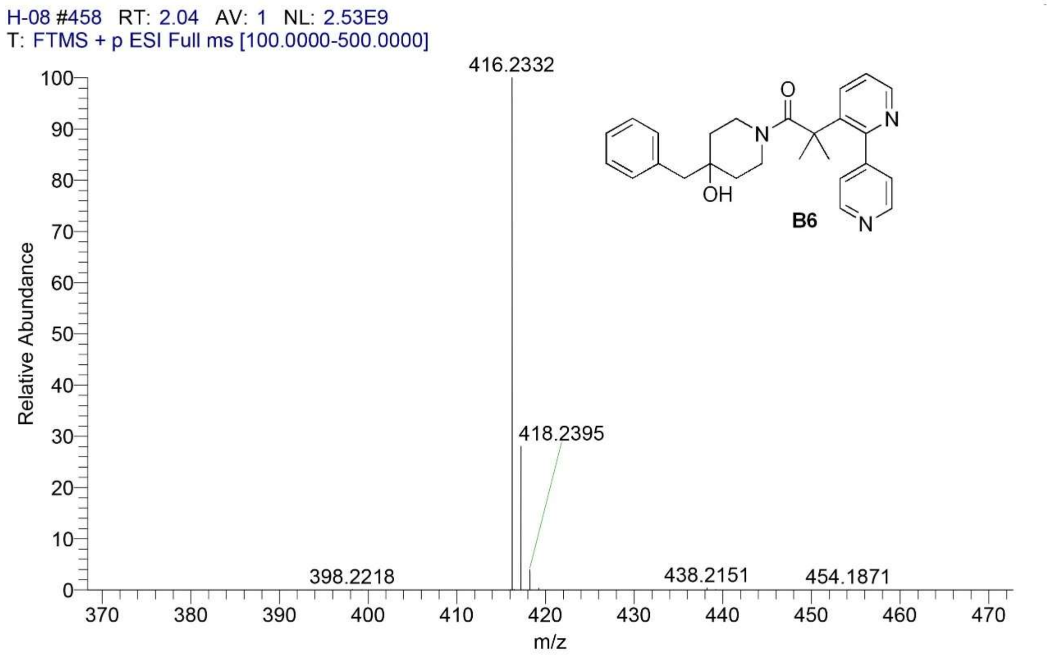

Figure S129: HR-MS (ESI/ion trap) spectrum of **B6**

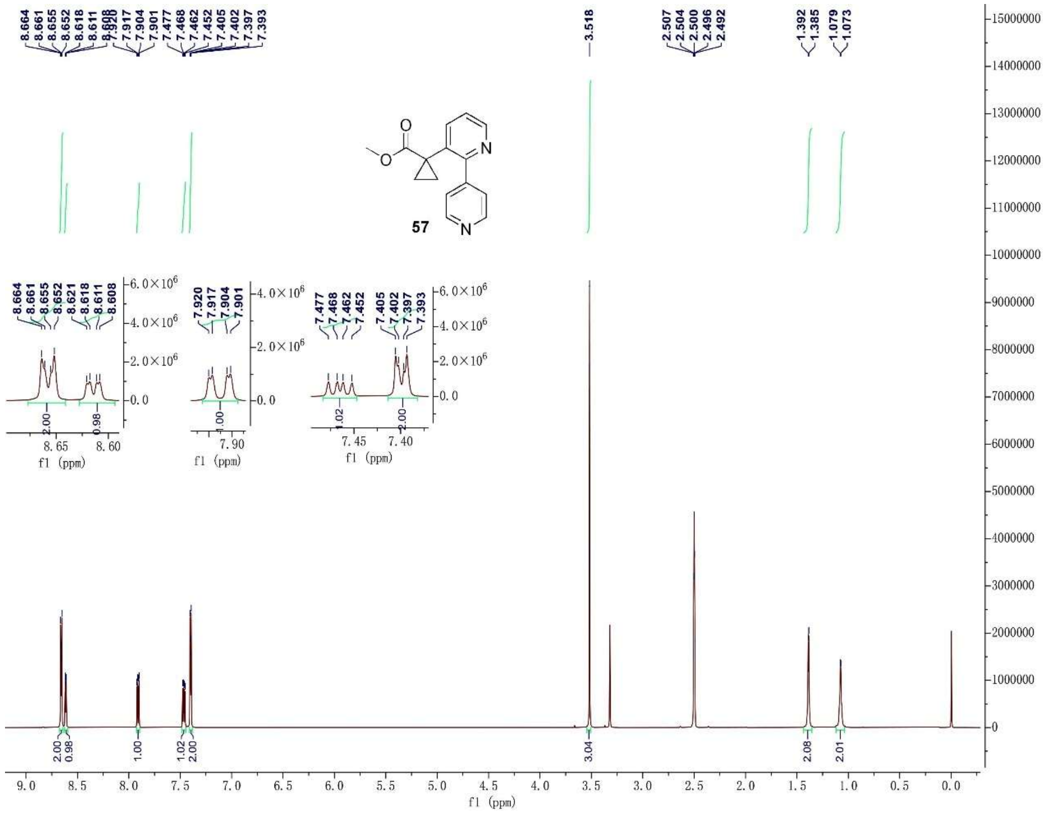

Figure S130: ^{1}H NMR spectrum of **57**

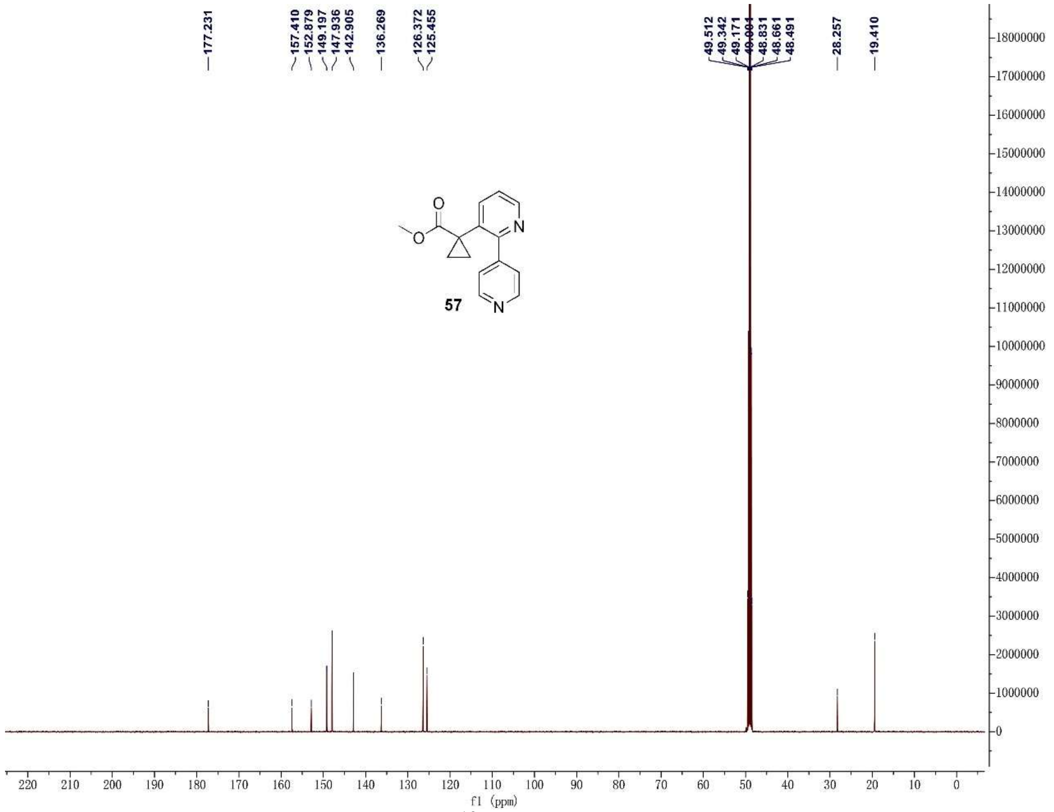

Figure S131: ^{13}C NMR spectrum of **57**

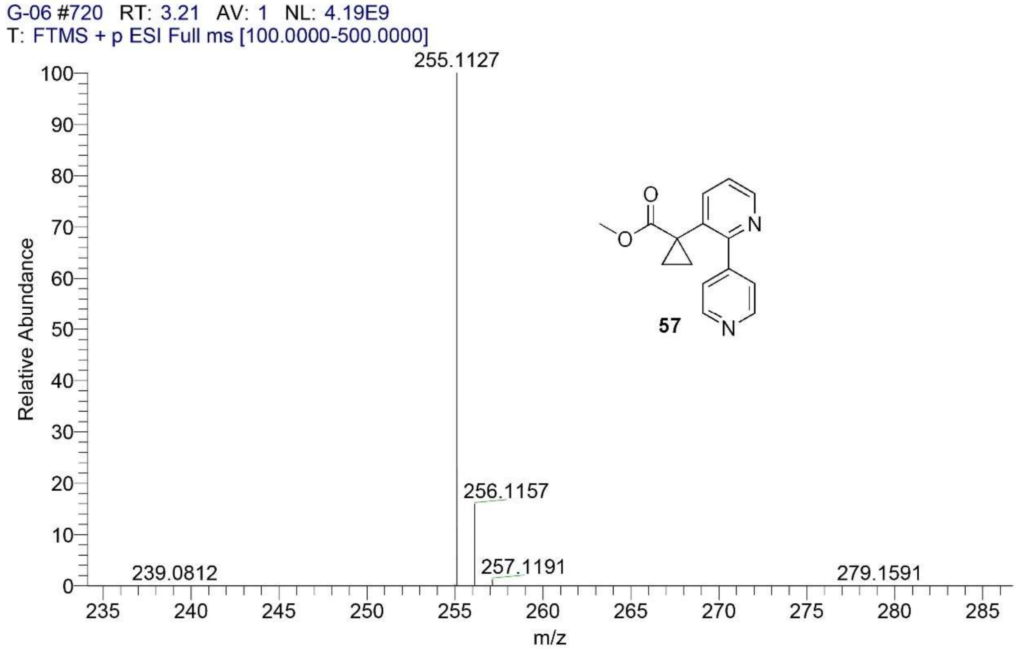

Figure S132: HR-MS (ESI/ion trap) spectrum of **57**

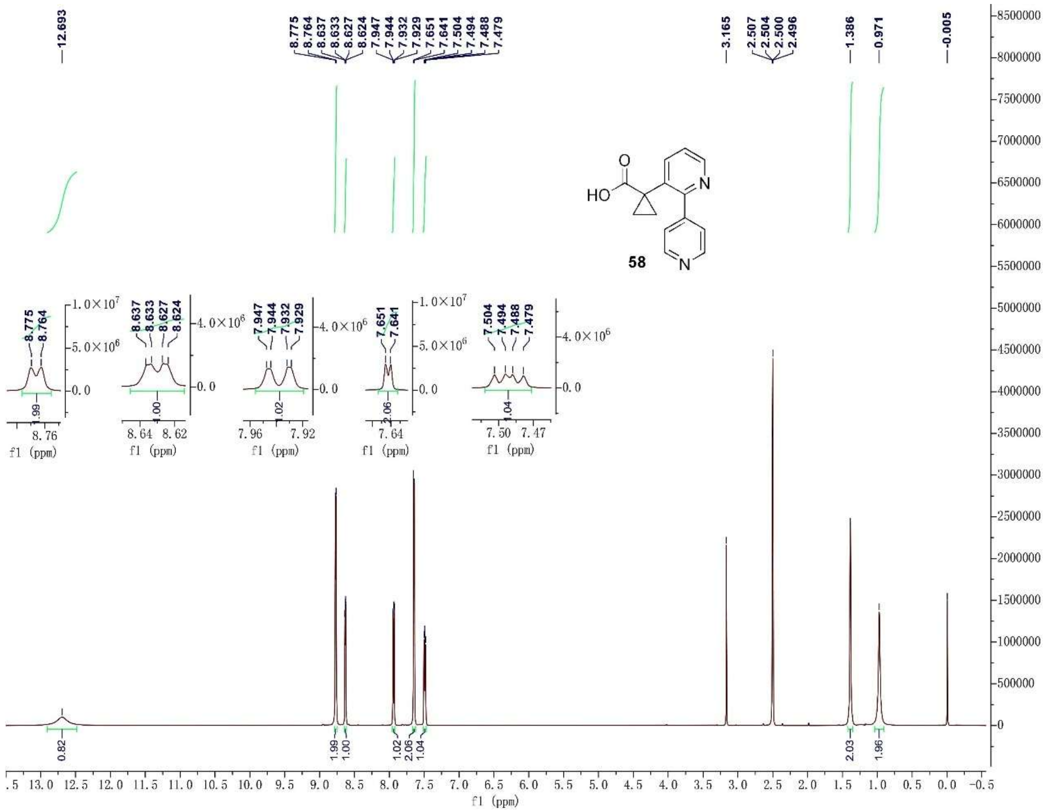

Figure S133: ^{1}H NMR spectrum of **58**

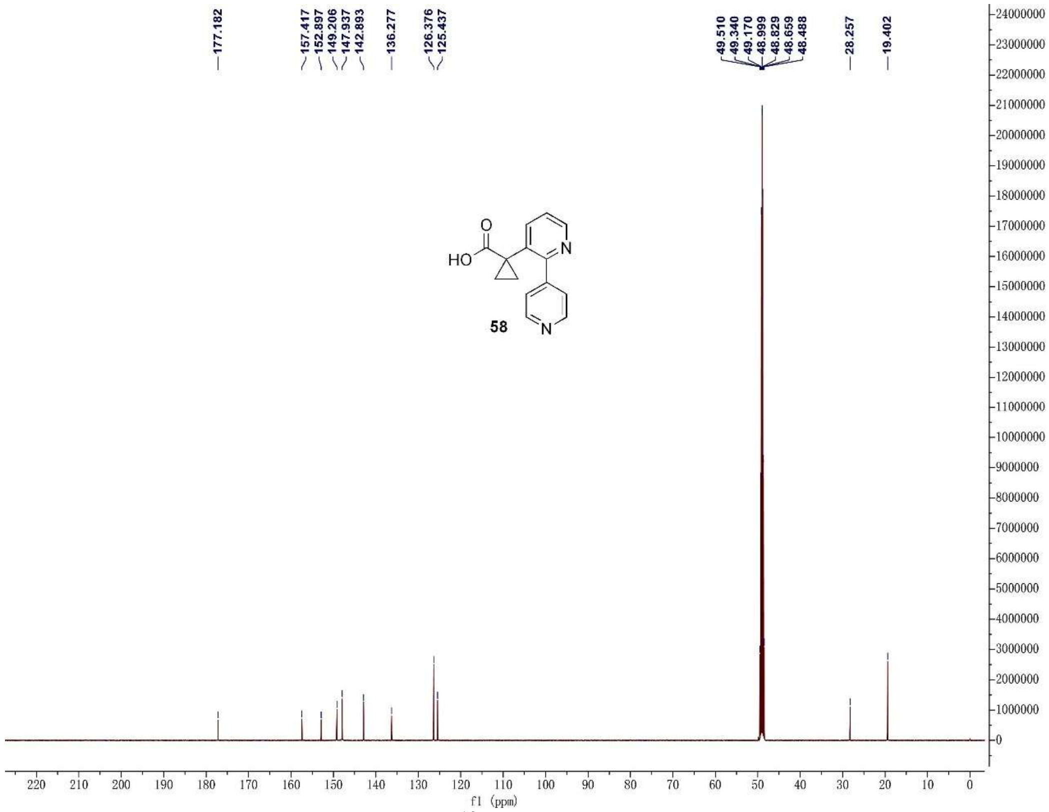

Figure S134: ^{13}C NMR spectrum of **58**

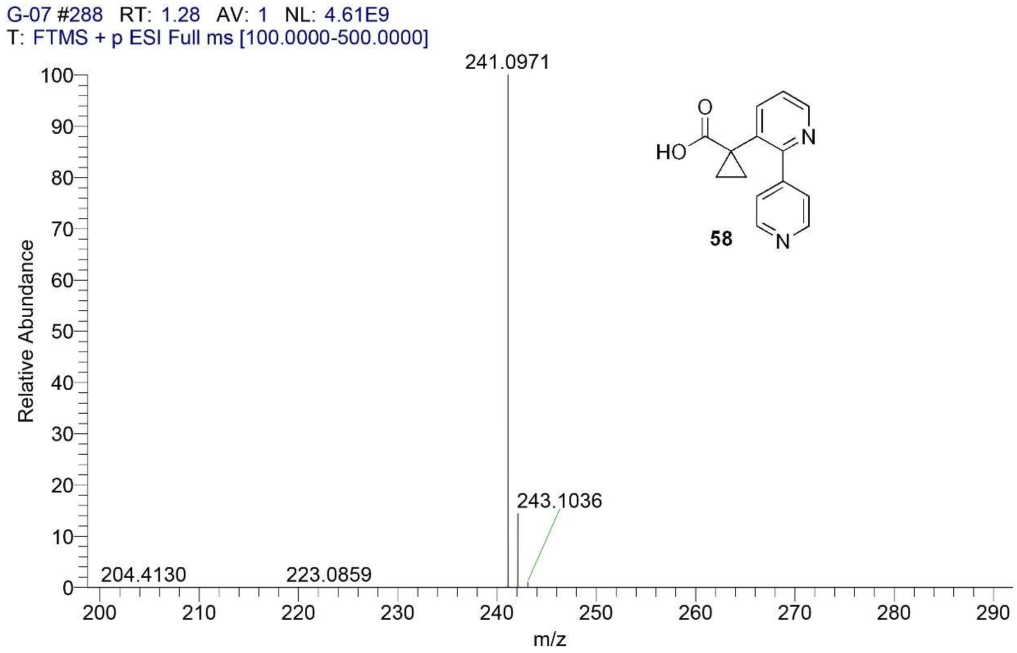

Figure S135: HR-MS (ESI/ion trap) spectrum of **58**

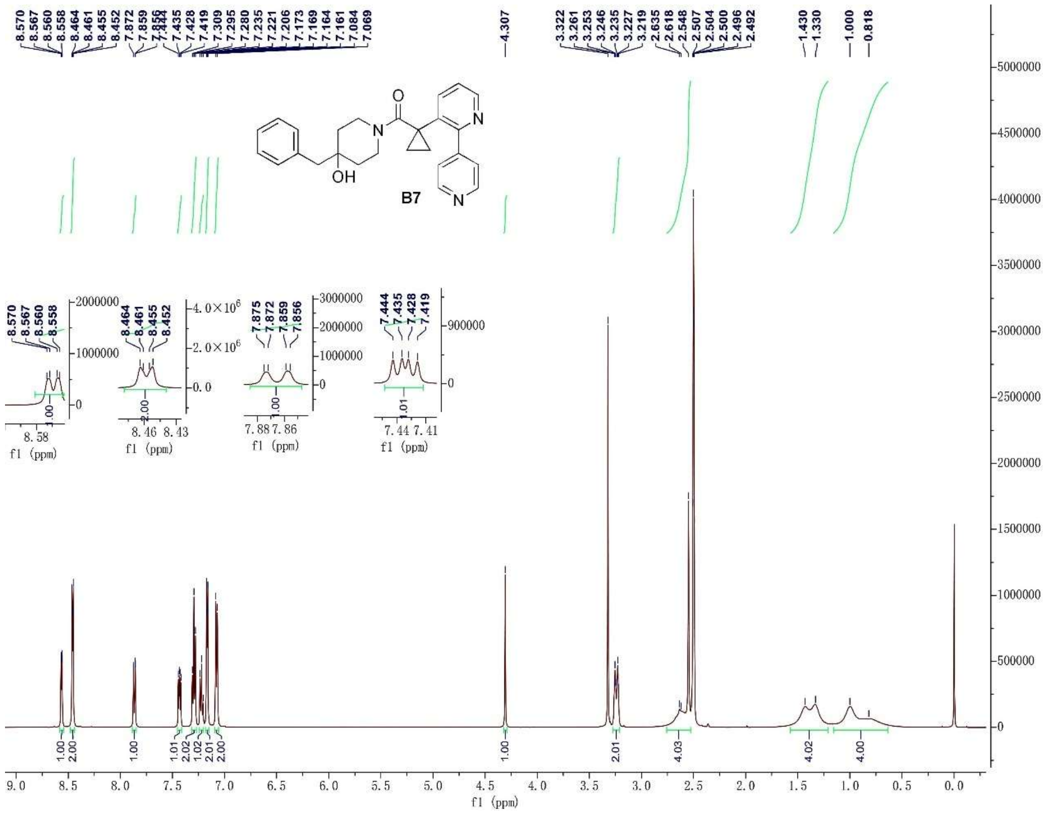

Figure S136: ^{1}H NMR spectrum of **B7**

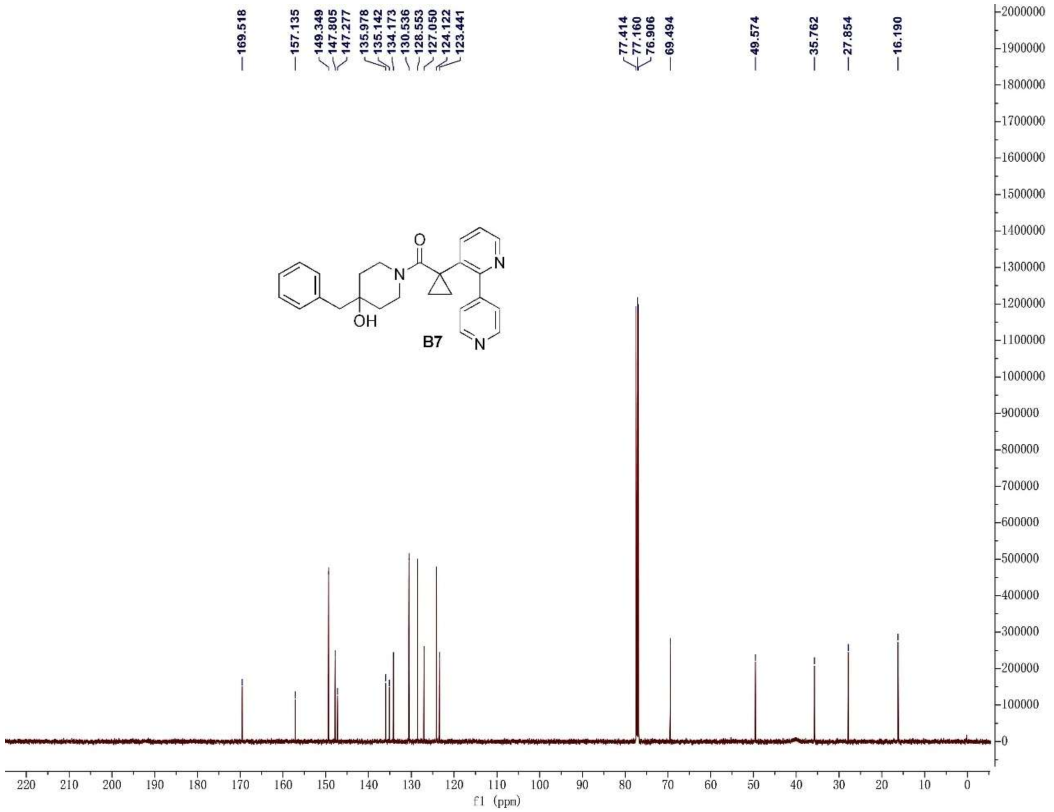

Figure S137: ^{13}C NMR spectrum of **B7**

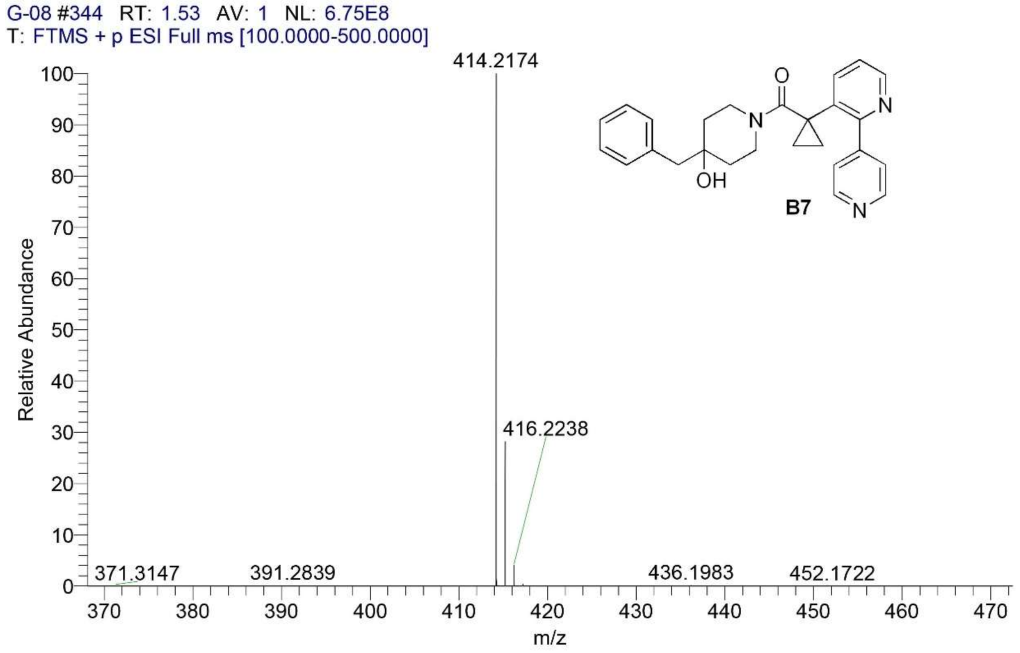

Figure S138: HR-MS (ESI/ion trap) spectrum of **B7**

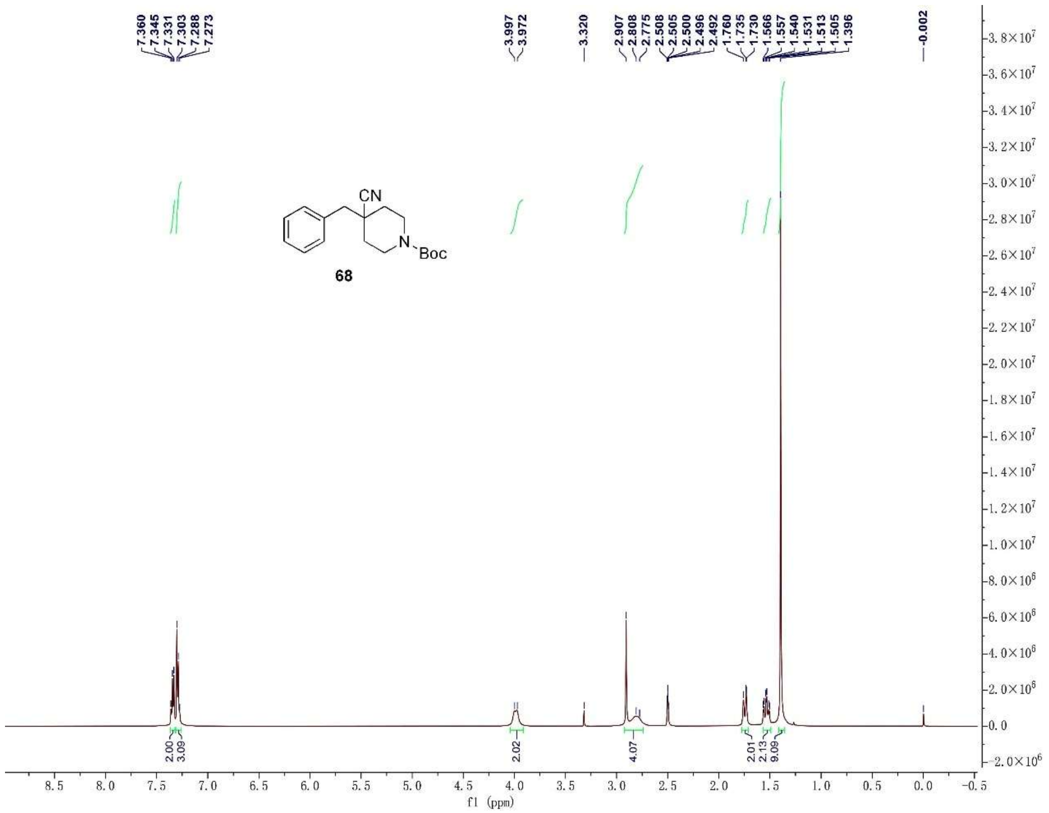

Figure S139: ^{1}H NMR spectrum of **68**

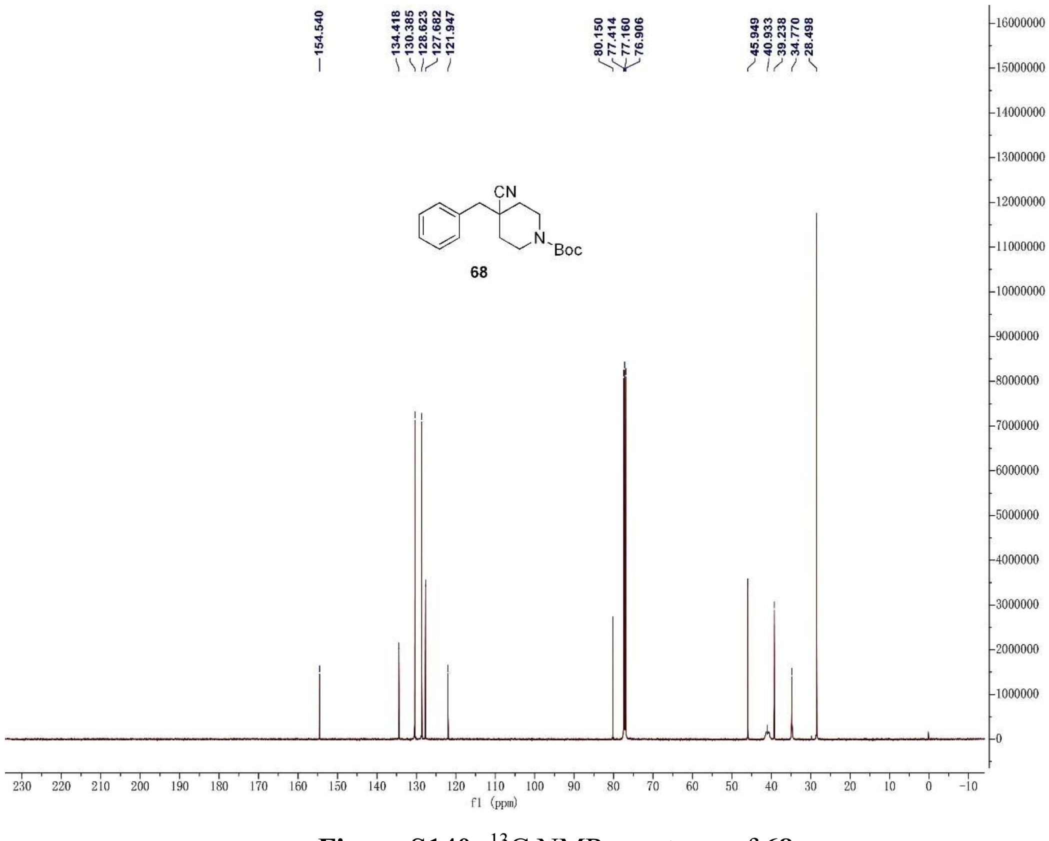

Figure S140: ^{13}C NMR spectrum of **68**

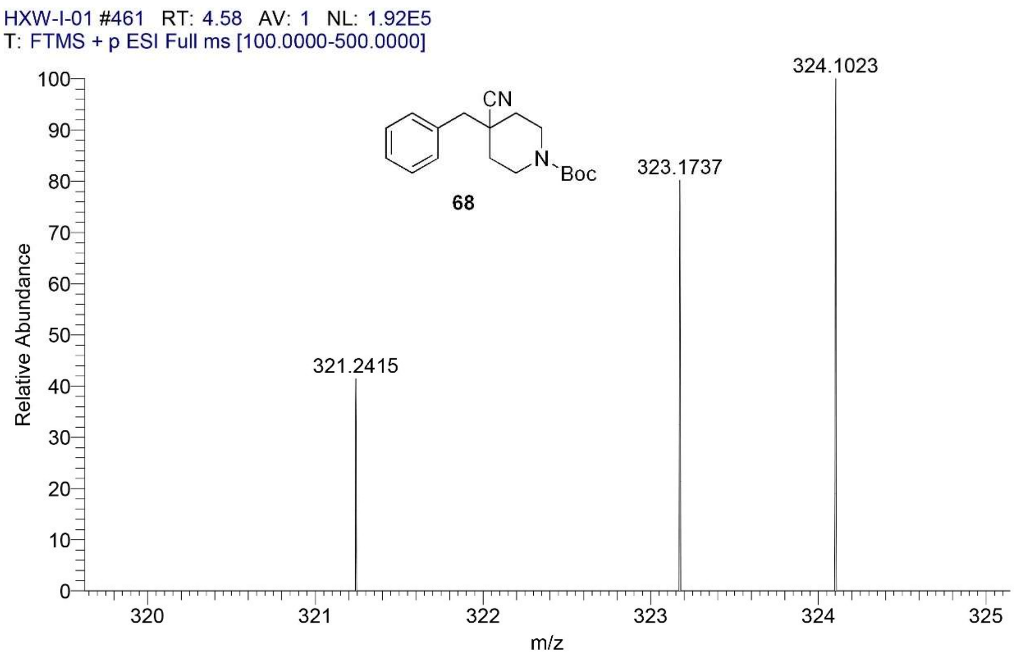

Figure S141: HR-MS (ESI/ion trap) spectrum of **68**

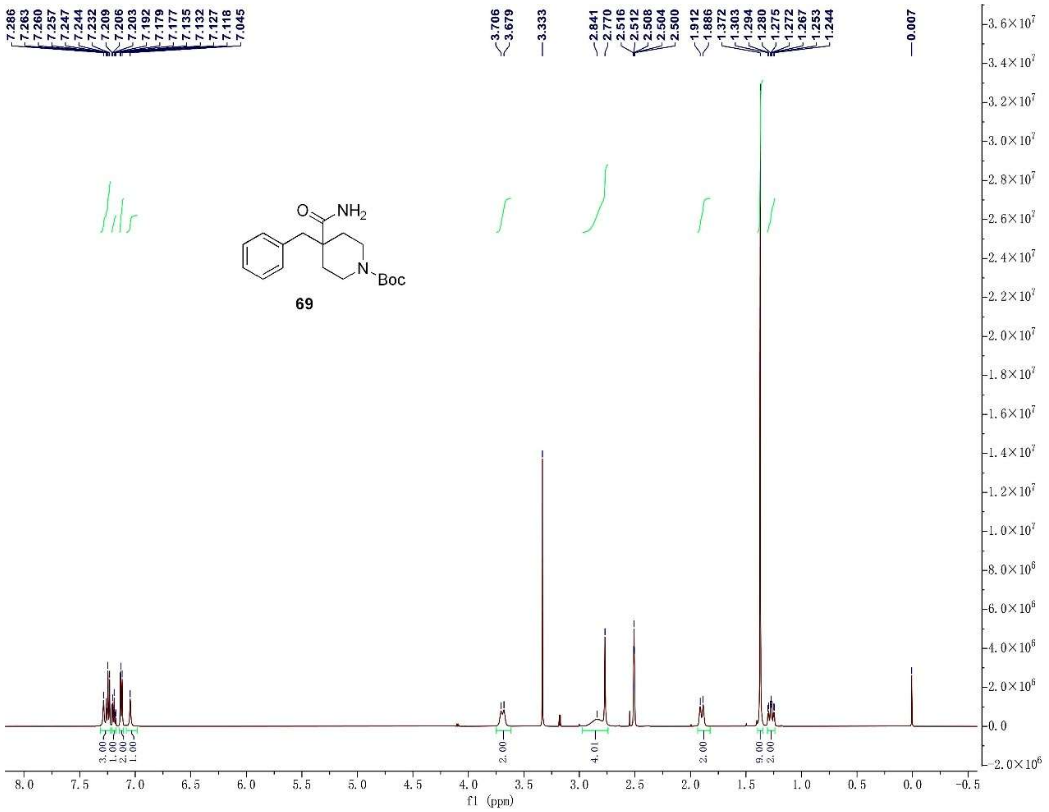

Figure S142: ^{1}H NMR spectrum of **69**

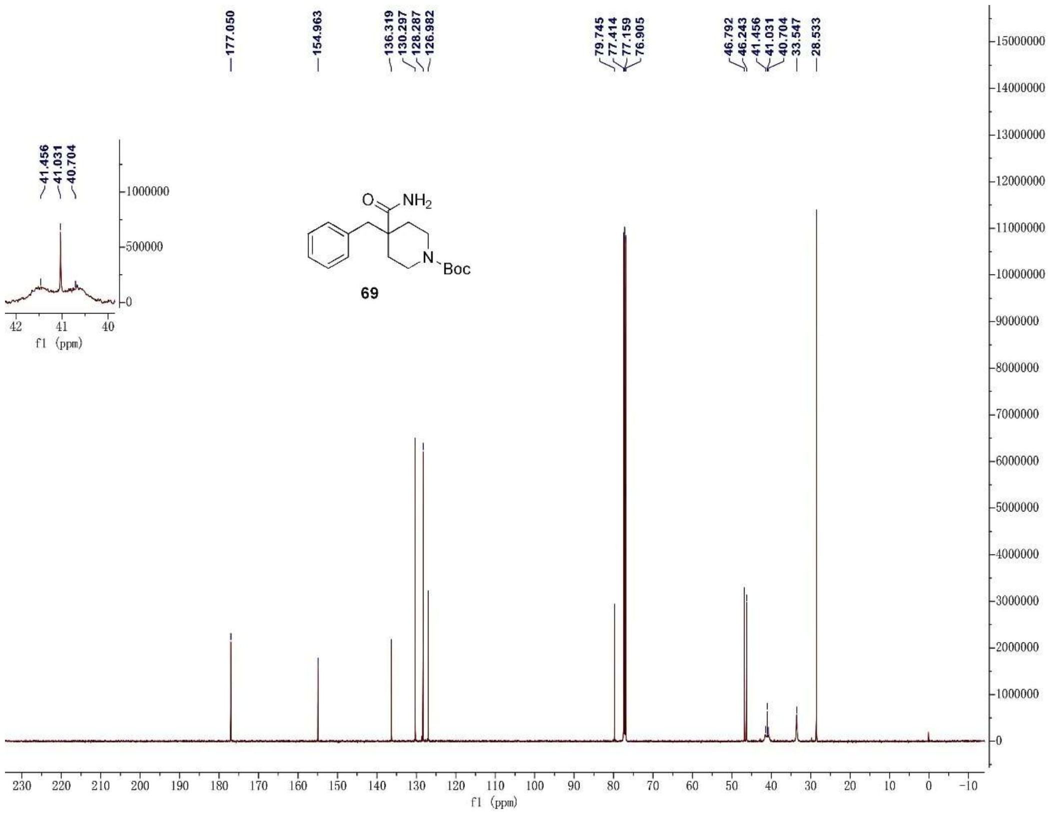

Figure S143: ^{13}C NMR spectrum of **69**

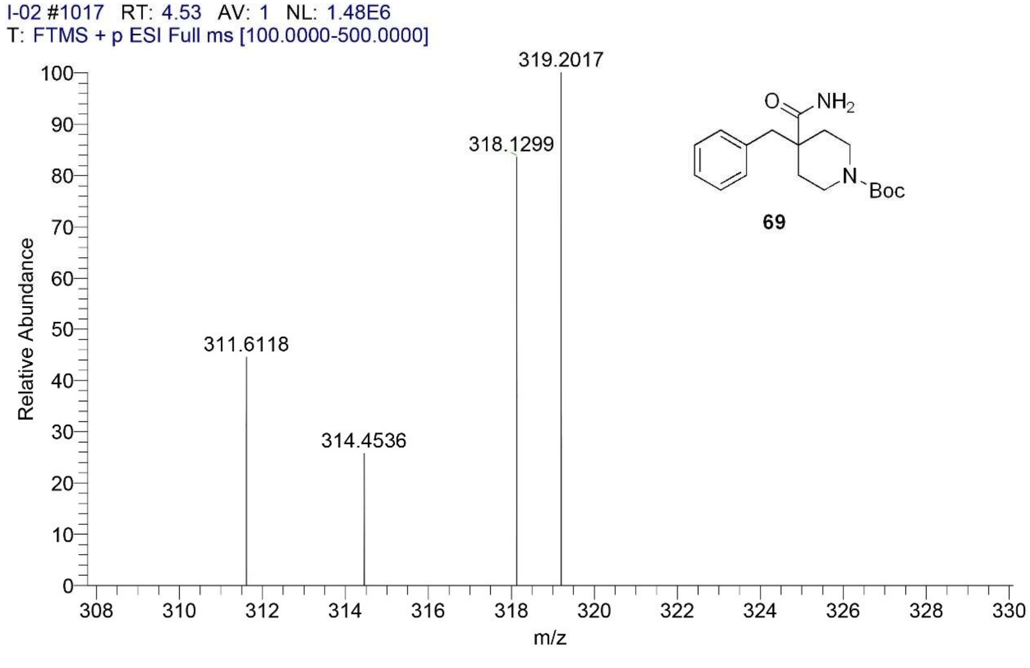

Figure S144: HR-MS (ESI/ion trap) spectrum of **69**

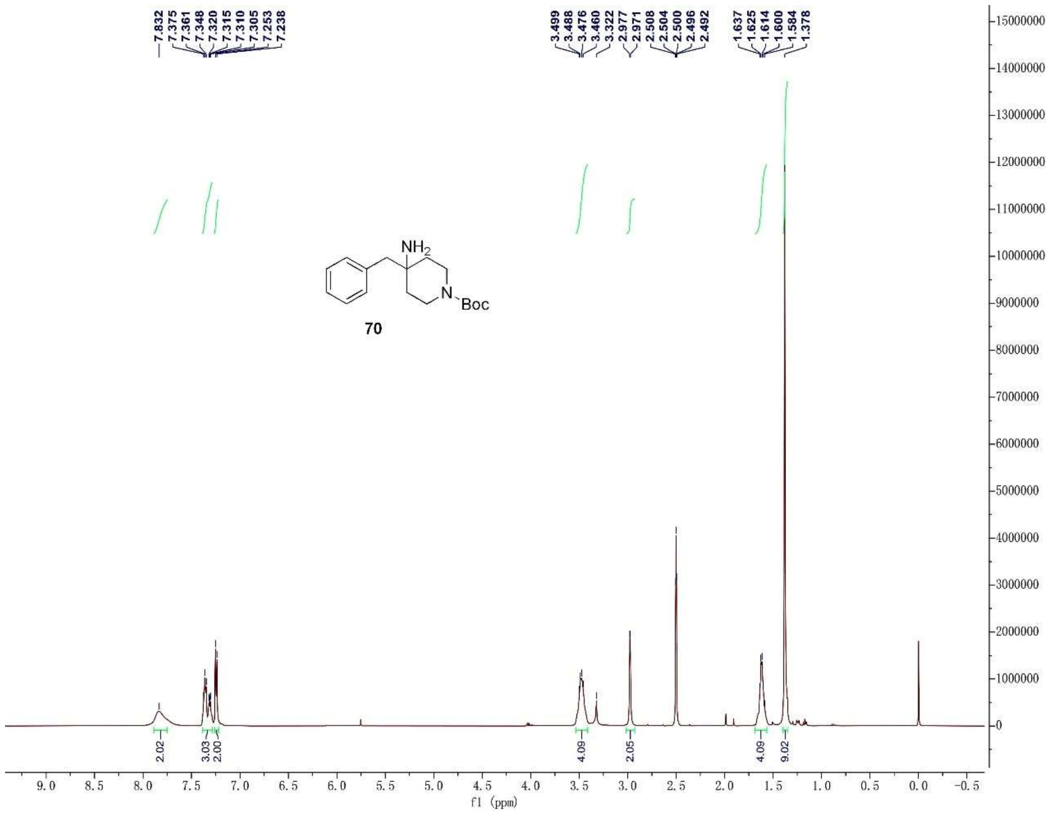

Figure S145: ^{1}H NMR spectrum of **70**

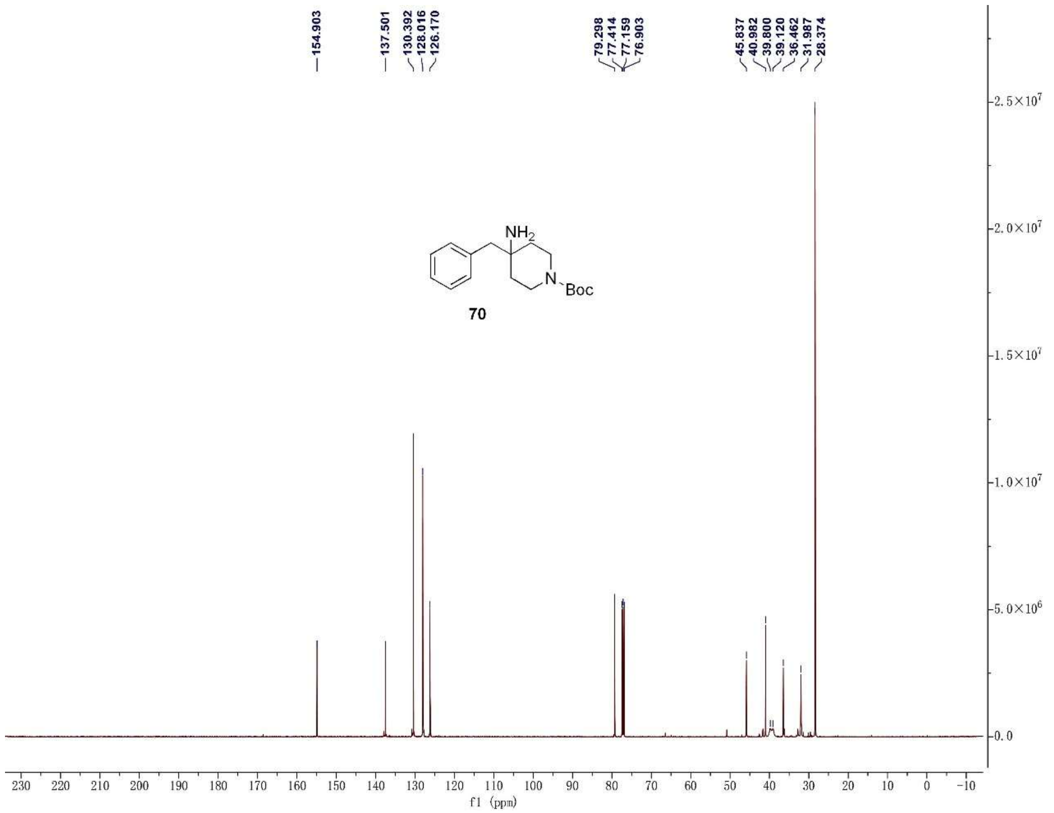

Figure S146: ^{13}C NMR spectrum of **70**

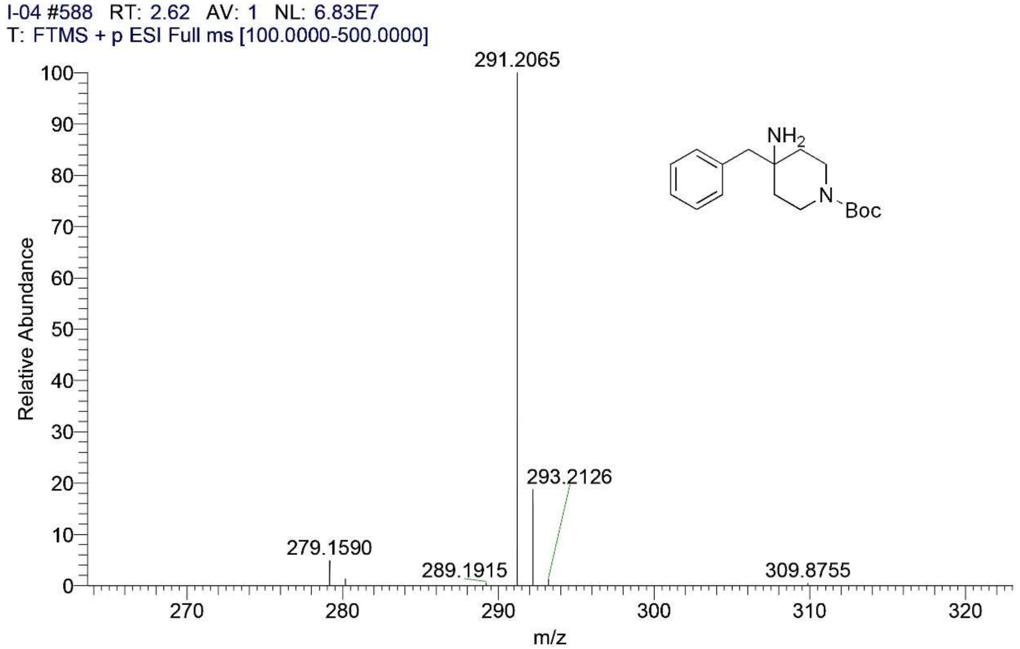

Figure S147: HR-MS (ESI/ion trap) spectrum of **70**

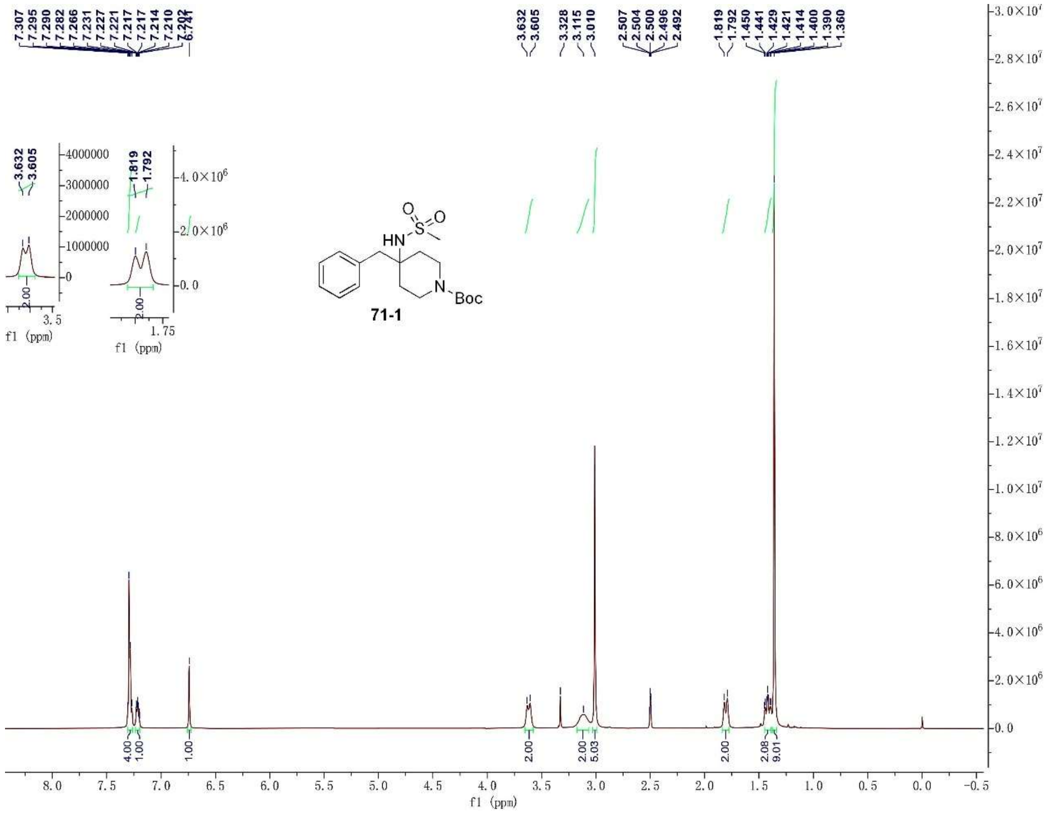

Figure S148: ^{1}H NMR spectrum of **71-1**

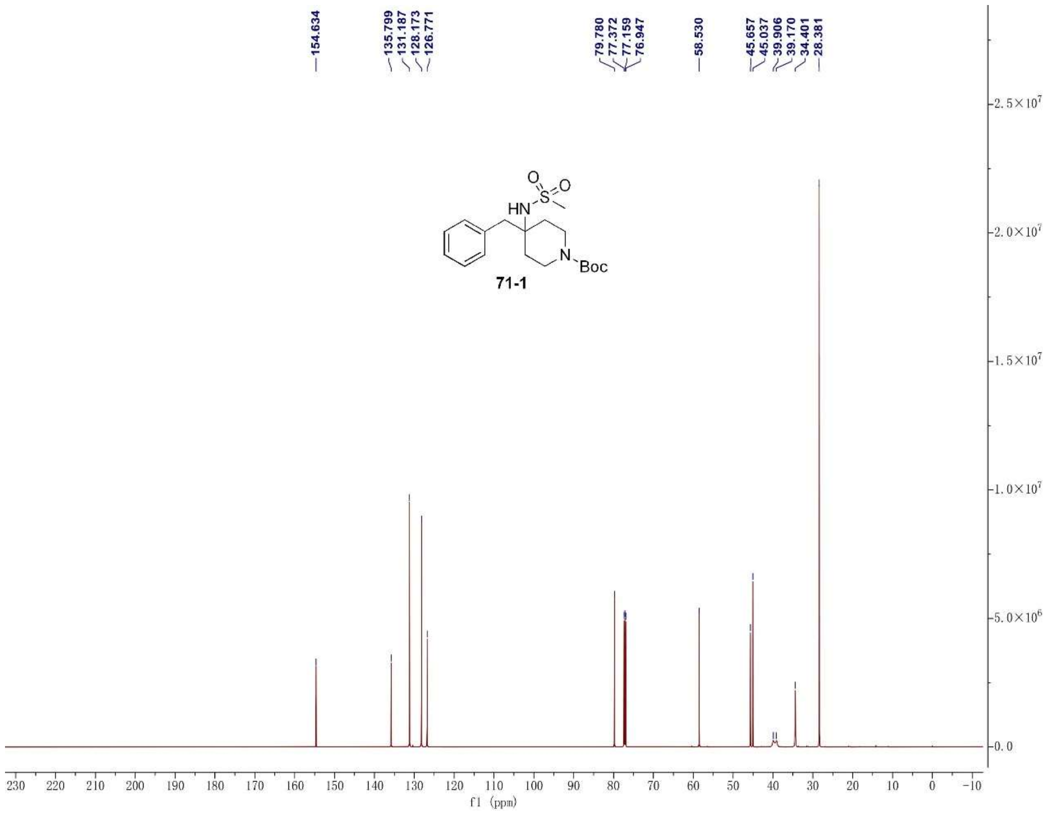

Figure S149: ^{13}C NMR spectrum of **71-1**

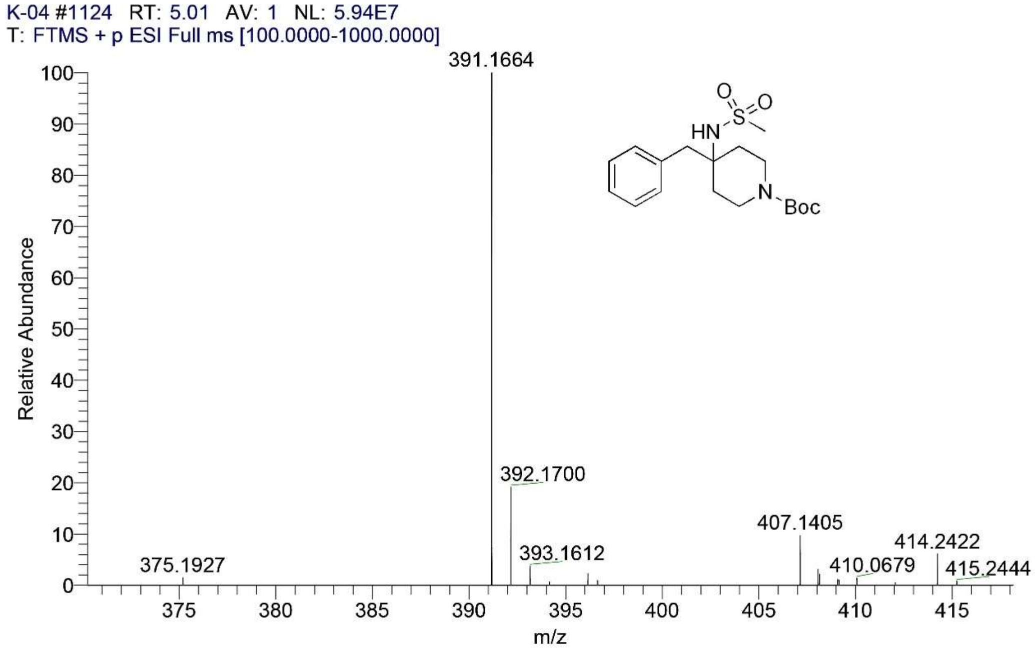

Figure S150: HR-MS (ESI/ion trap) spectrum of **71-1**

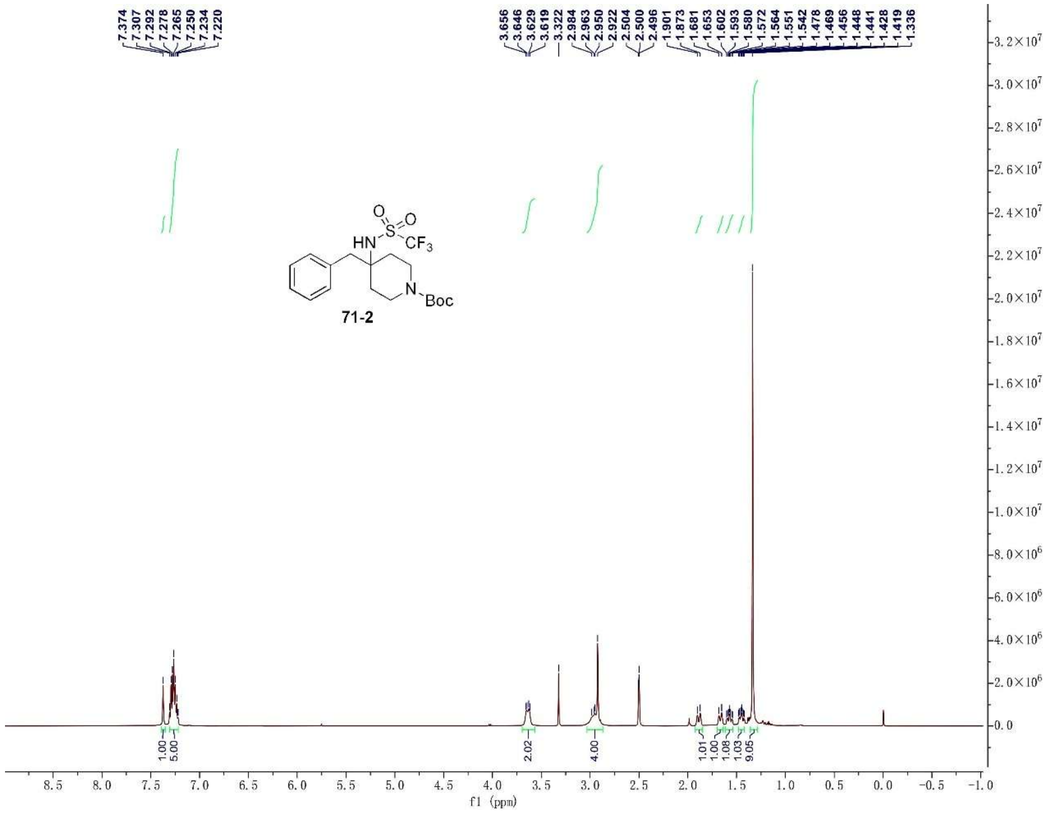

Figure S151: ^{1}H NMR spectrum of **71-2**

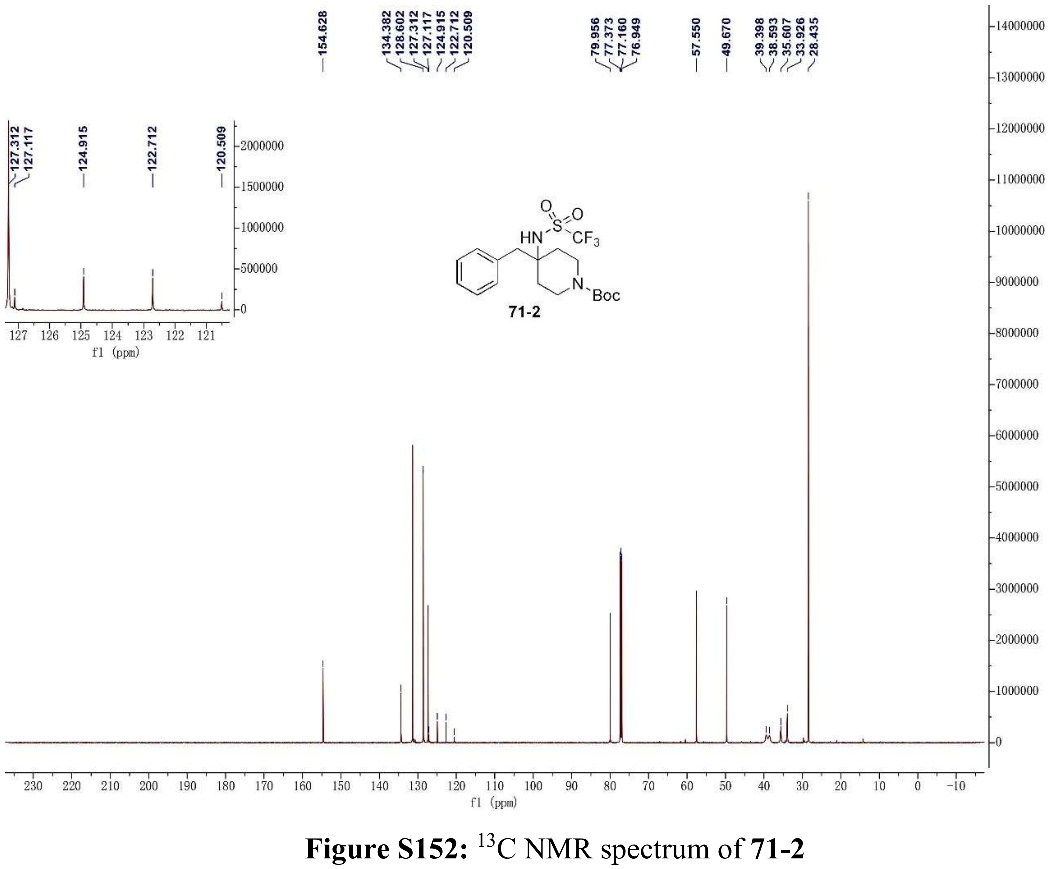

Figure S152: ^{13}C NMR spectrum of **71-2**

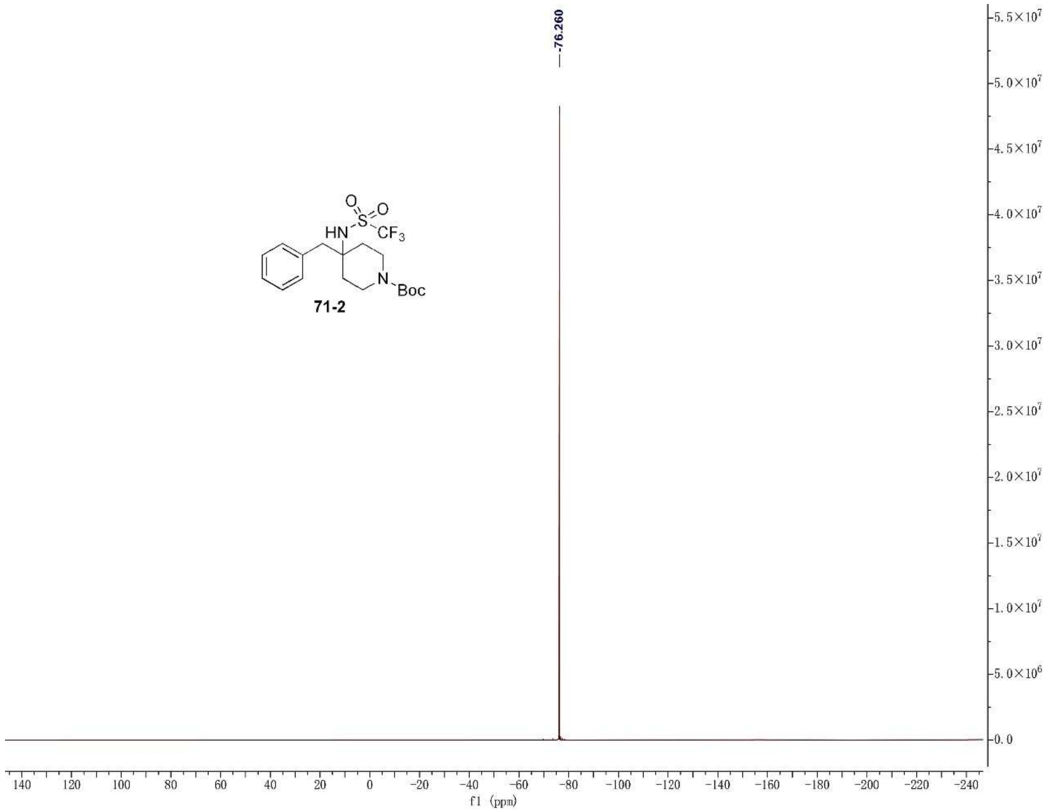

Figure S153: ^{19}F NMR spectrum of **71-2**

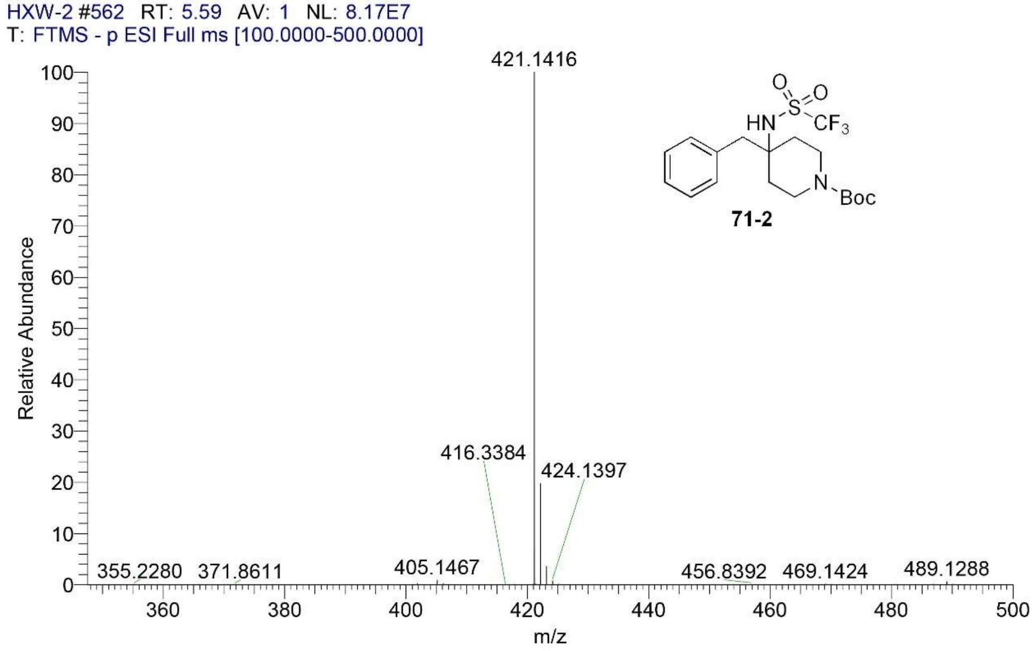

Figure S154: HR-MS (ESI/ion trap) spectrum of **71-2**

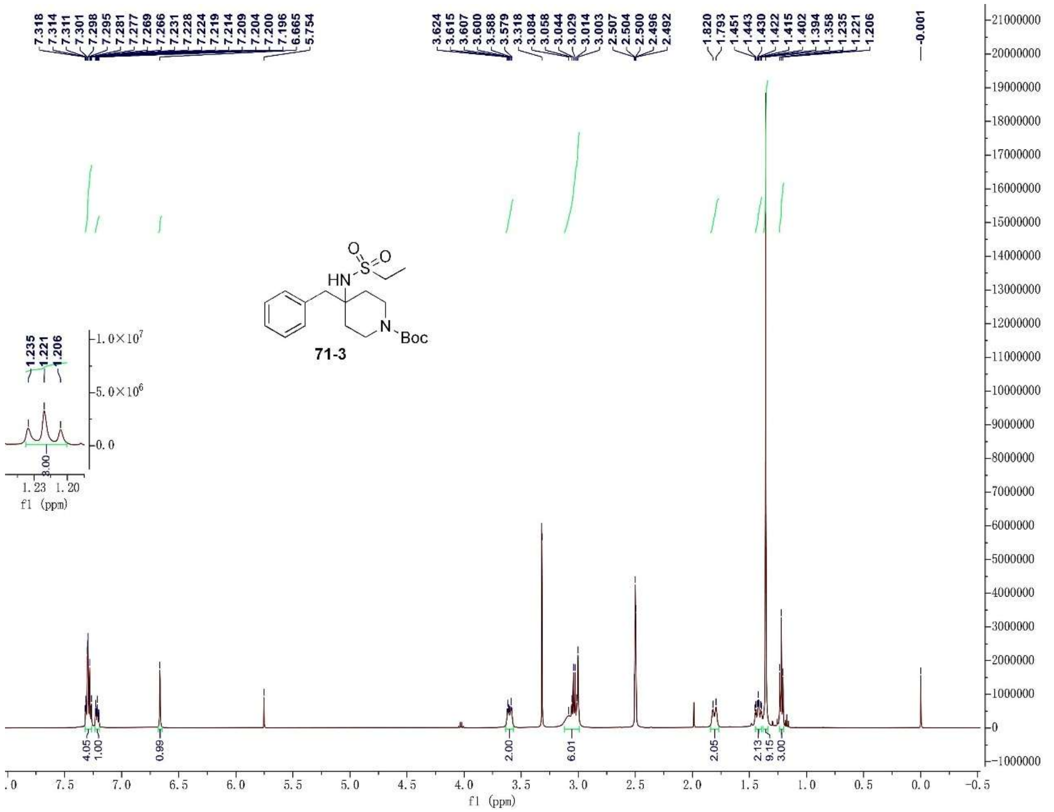

Figure S155: ^{1}H NMR spectrum of **71-3**

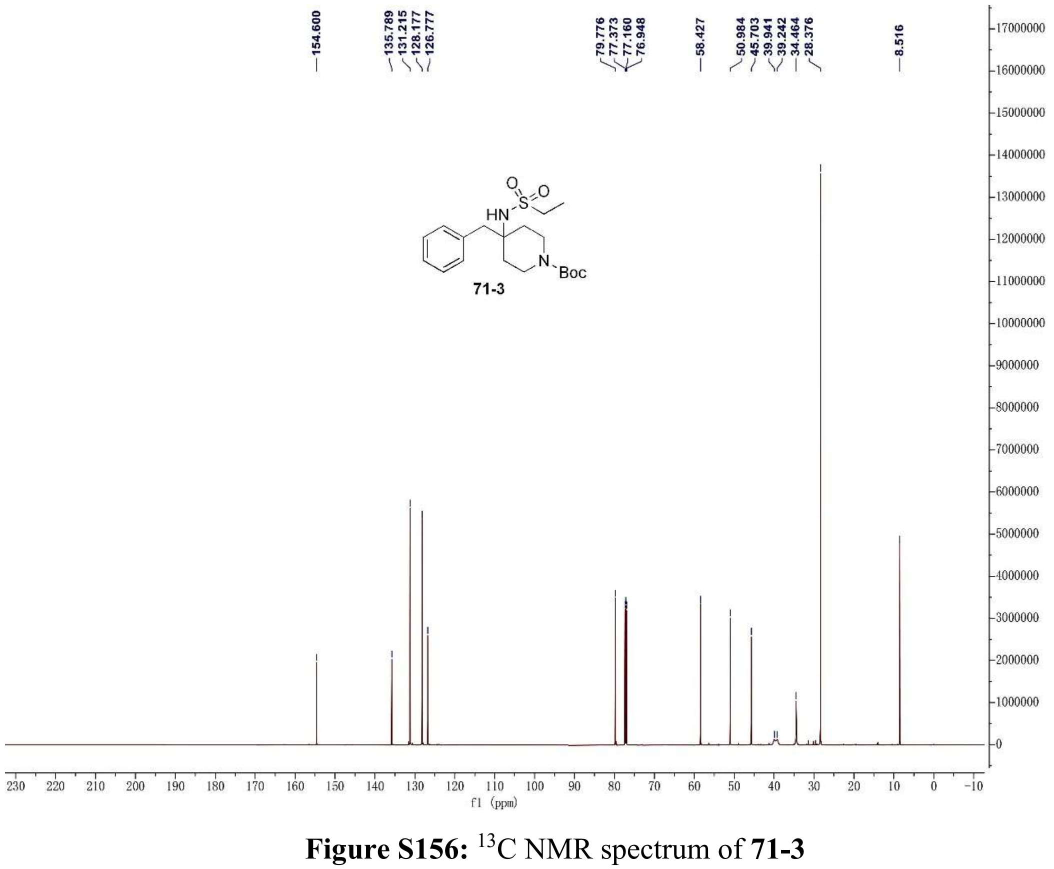

Figure S156: ^{13}C NMR spectrum of **71-3**

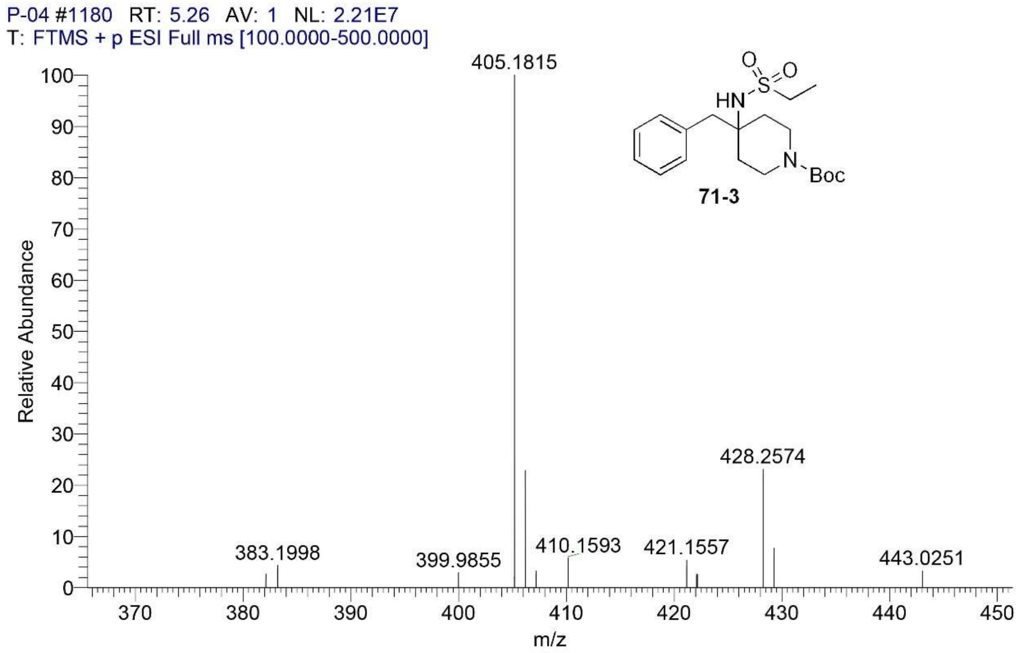

Figure S157: HR-MS (ESI/ion trap) spectrum of **71-3**

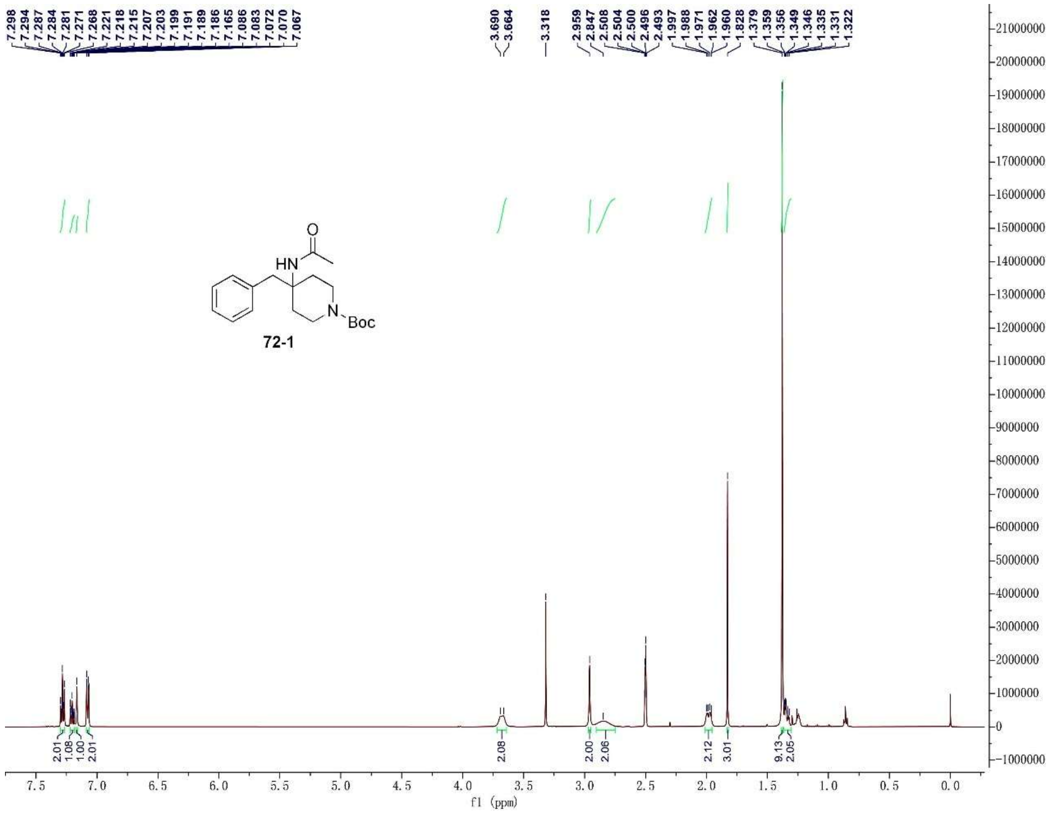

Figure S158: ^{1}H NMR spectrum of **72-1**

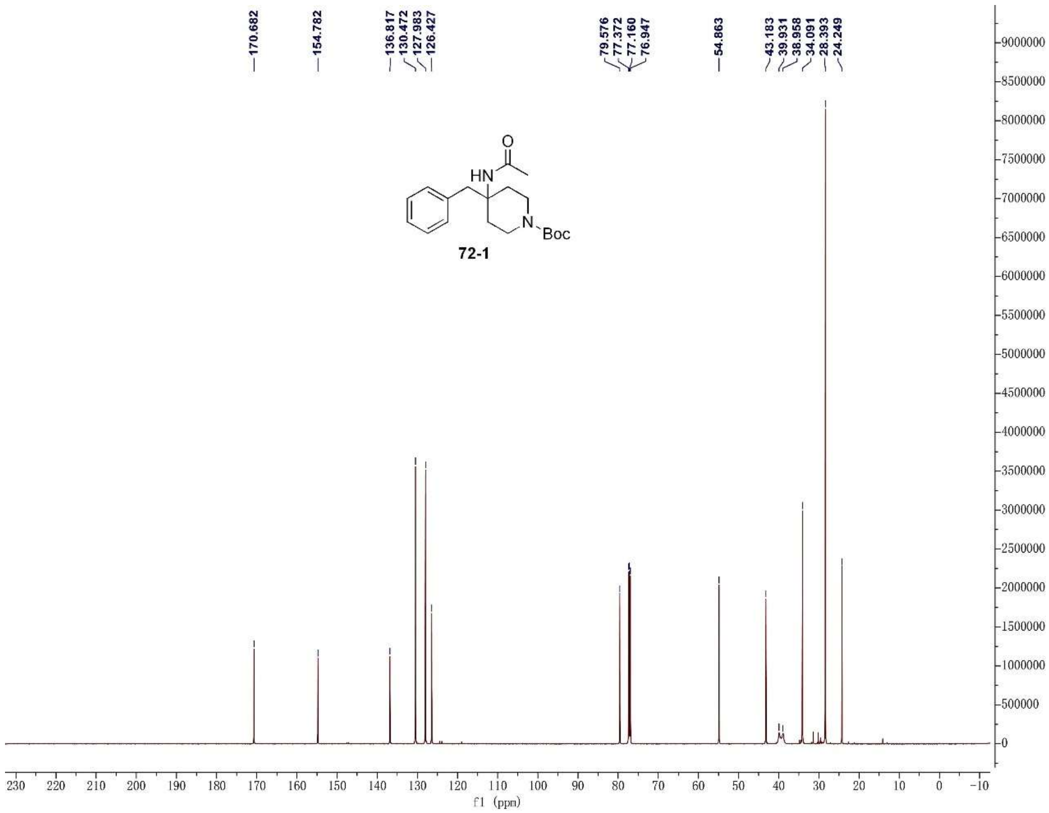

Figure S159: ^{13}C NMR spectrum of **72-1**

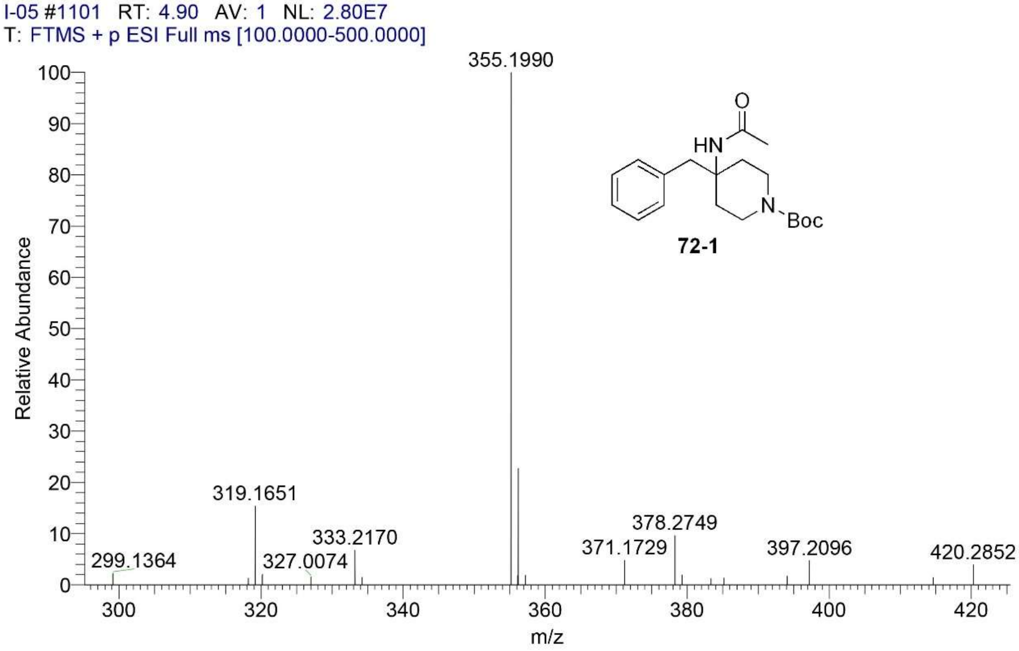

Figure S160: HR-MS (ESI/ion trap) spectrum of **72-1**

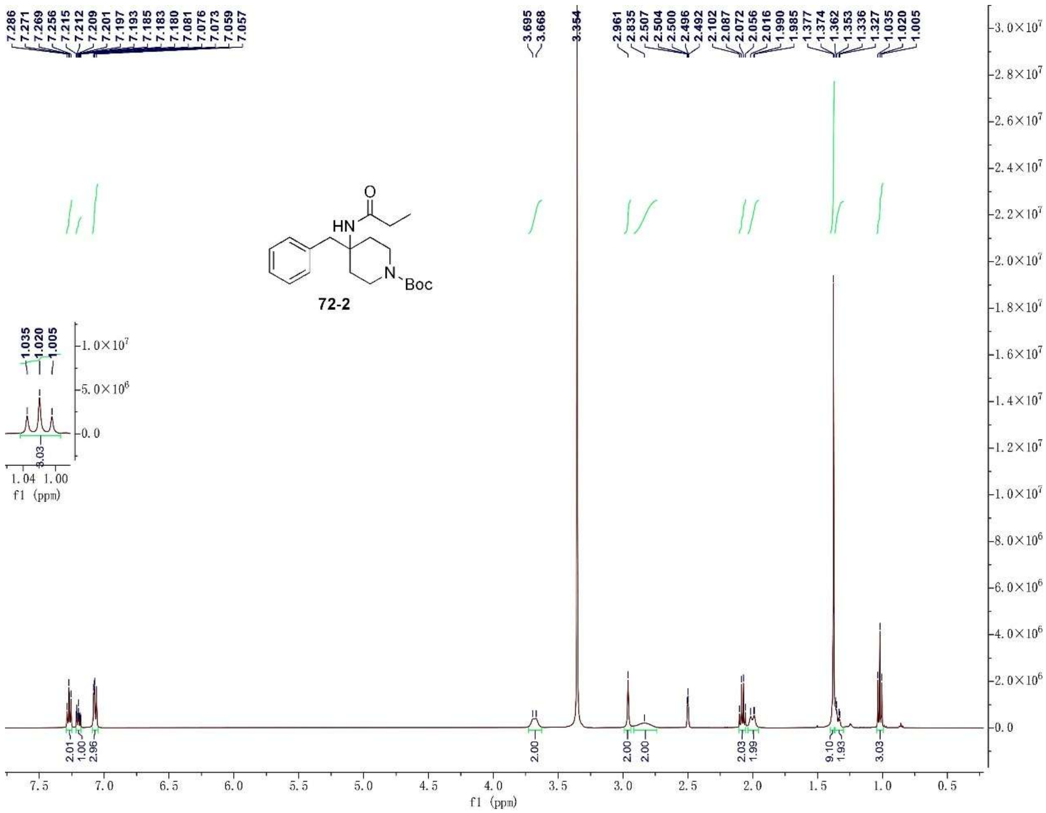

Figure S161: ^{1}H NMR spectrum of **72-2**

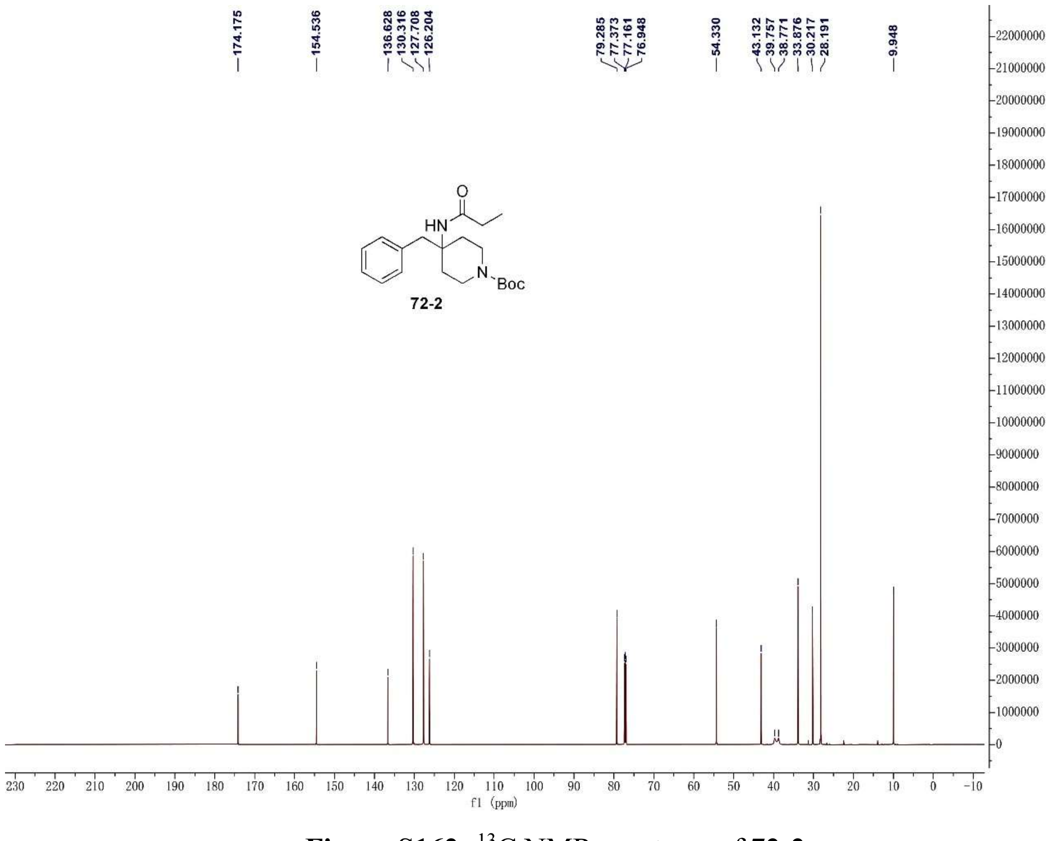

Figure S162: ^{13}C NMR spectrum of **72-2**

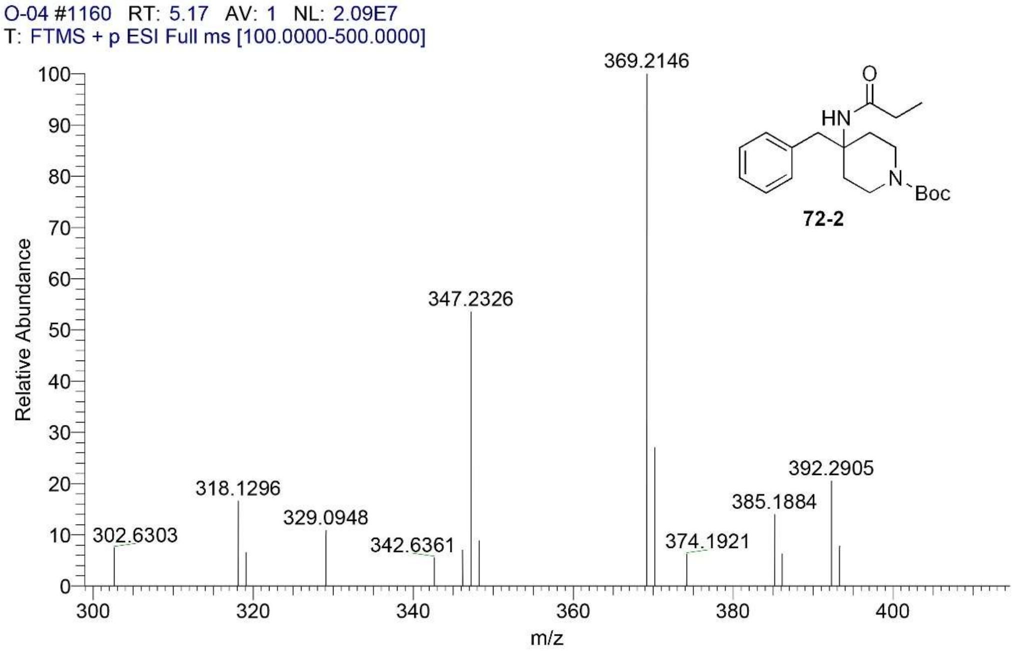

Figure S163: HR-MS (ESI/ion trap) spectrum of **72-2**

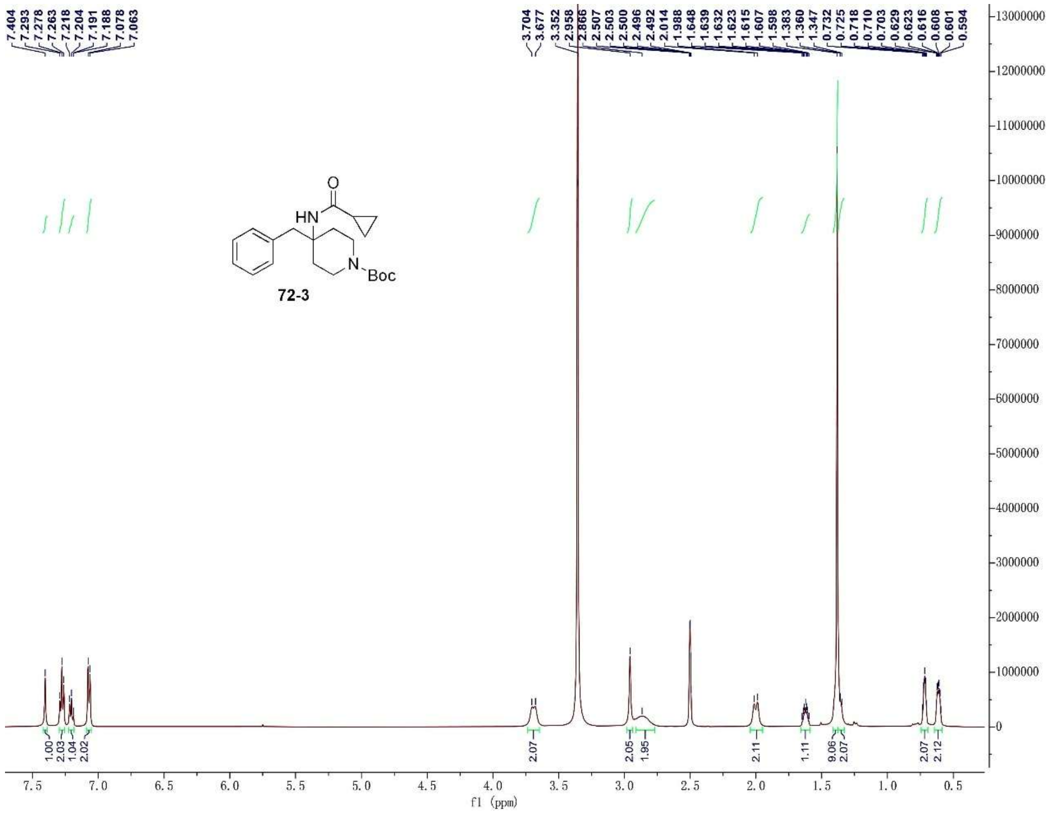

Figure S164: ^{1}H NMR spectrum of **72-3**

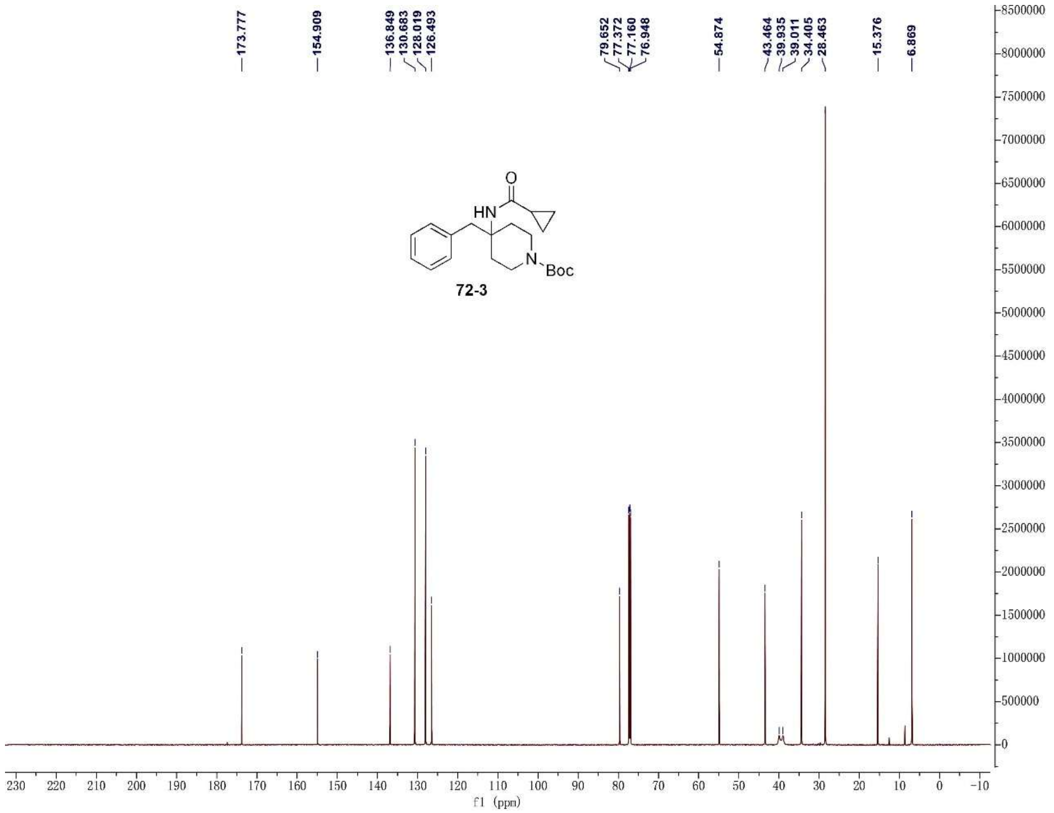

Figure S165: ^{13}C NMR spectrum of **72-3**

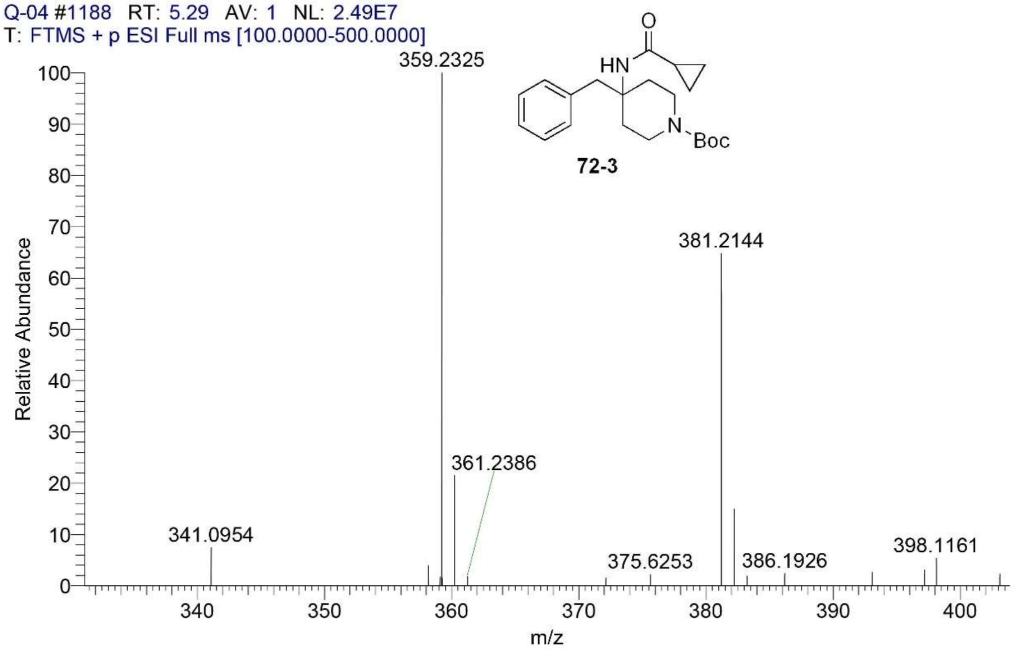

Figure S166: HR-MS (ESI/ion trap) spectrum of **72-3**

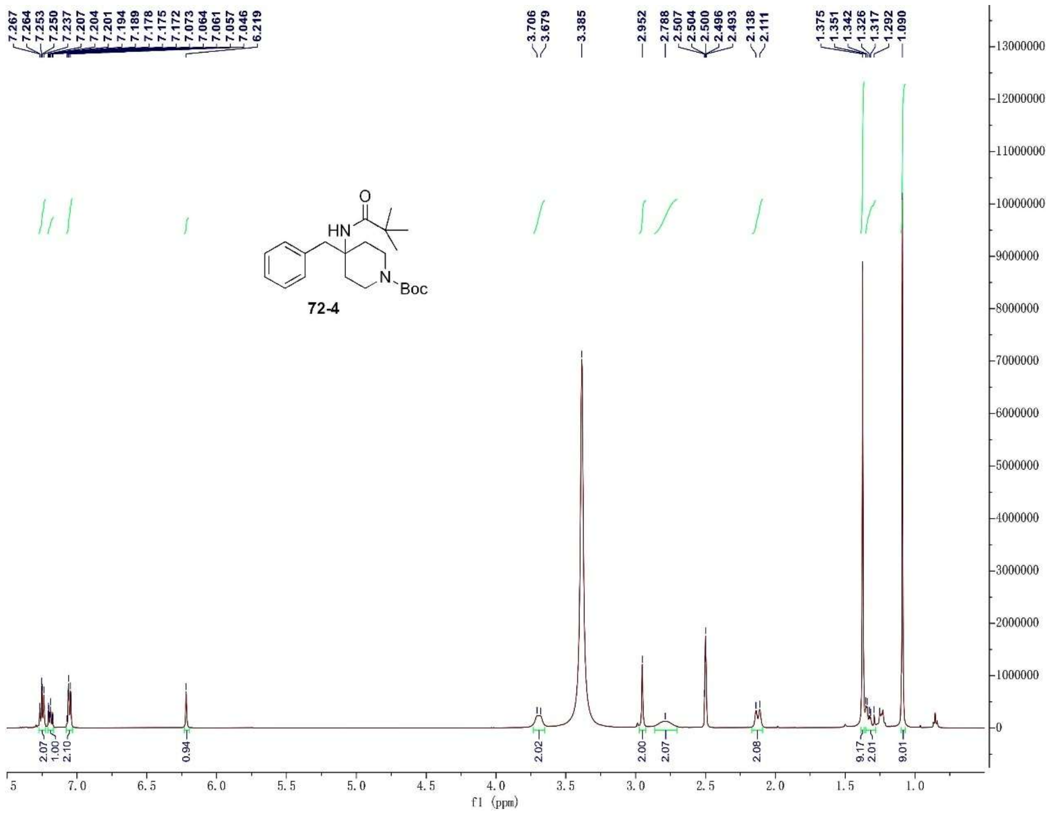

Figure S167: ^{1}H NMR spectrum of **72-4**

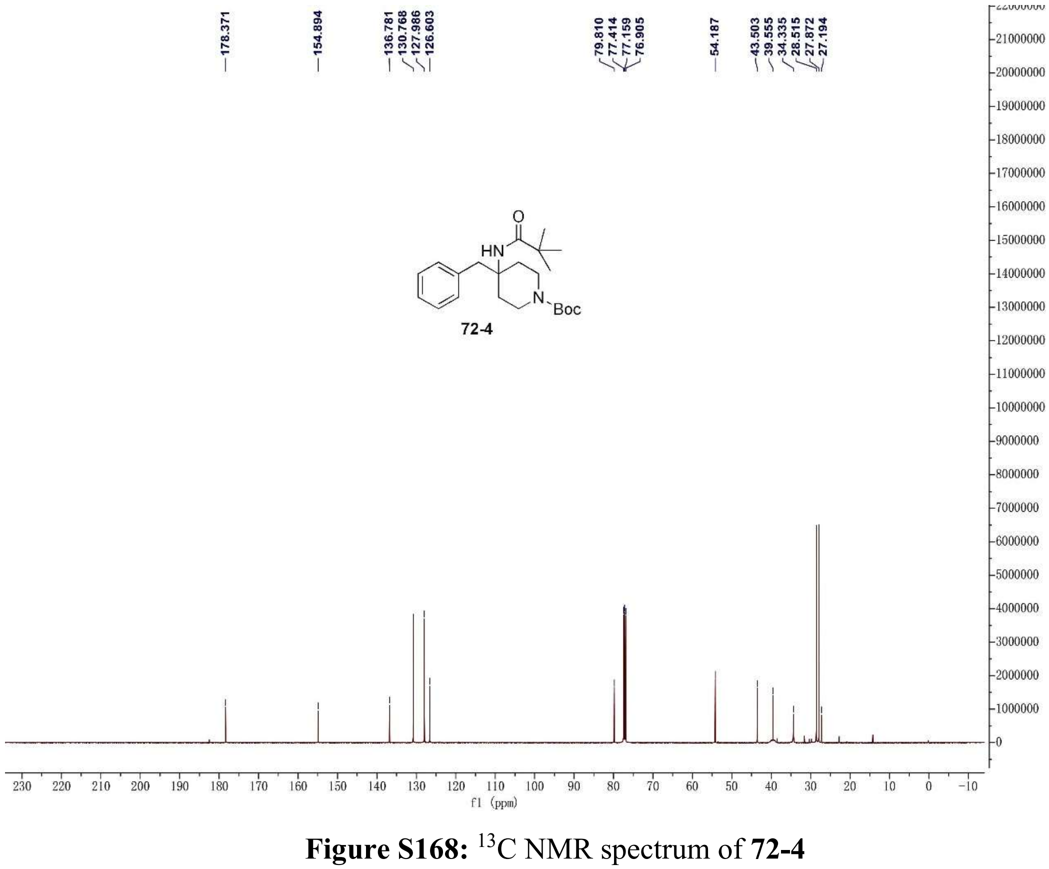

Figure S168: ^{13}C NMR spectrum of **72-4**

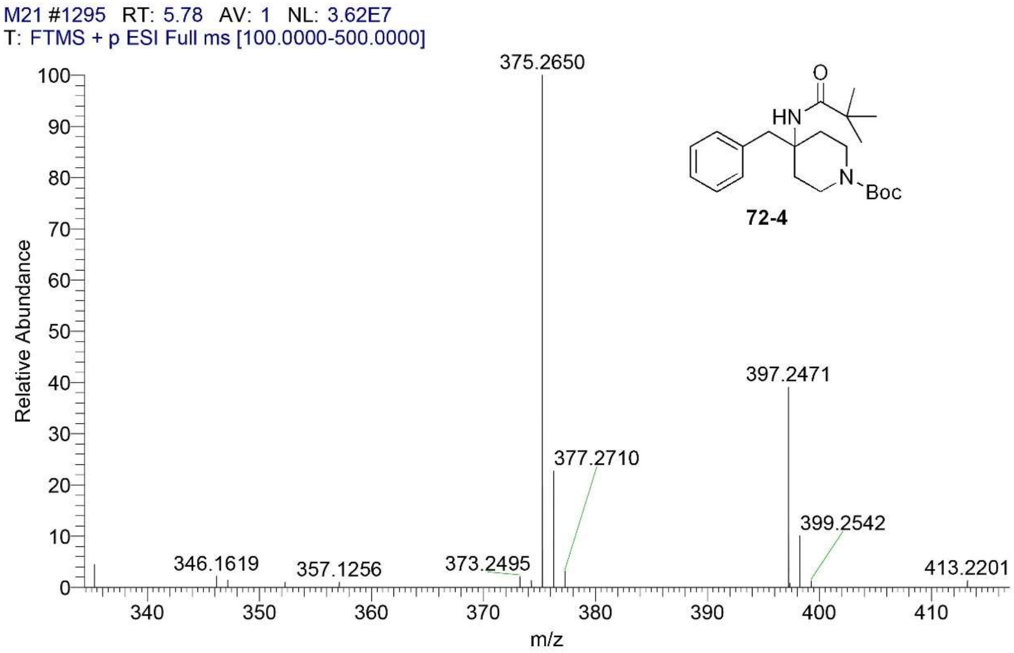

Figure S169: HR-MS (ESI/ion trap) spectrum of **72-4**

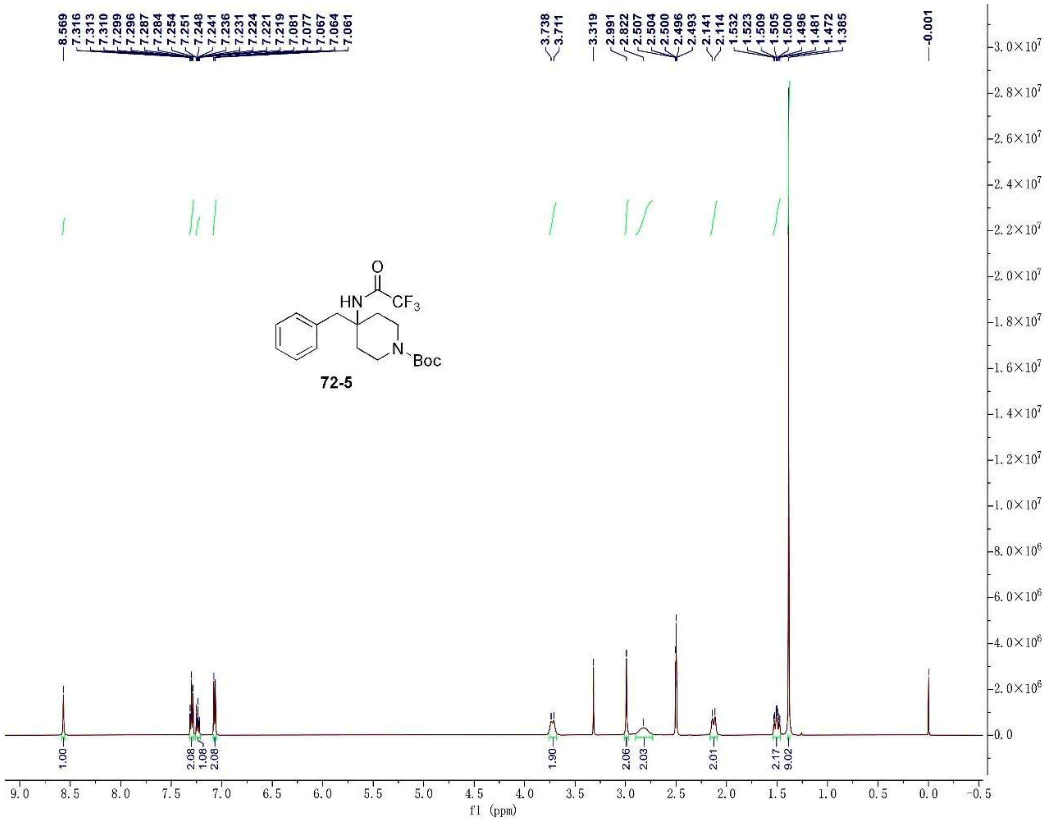

Figure S170: ^{1}H NMR spectrum of **72-5**

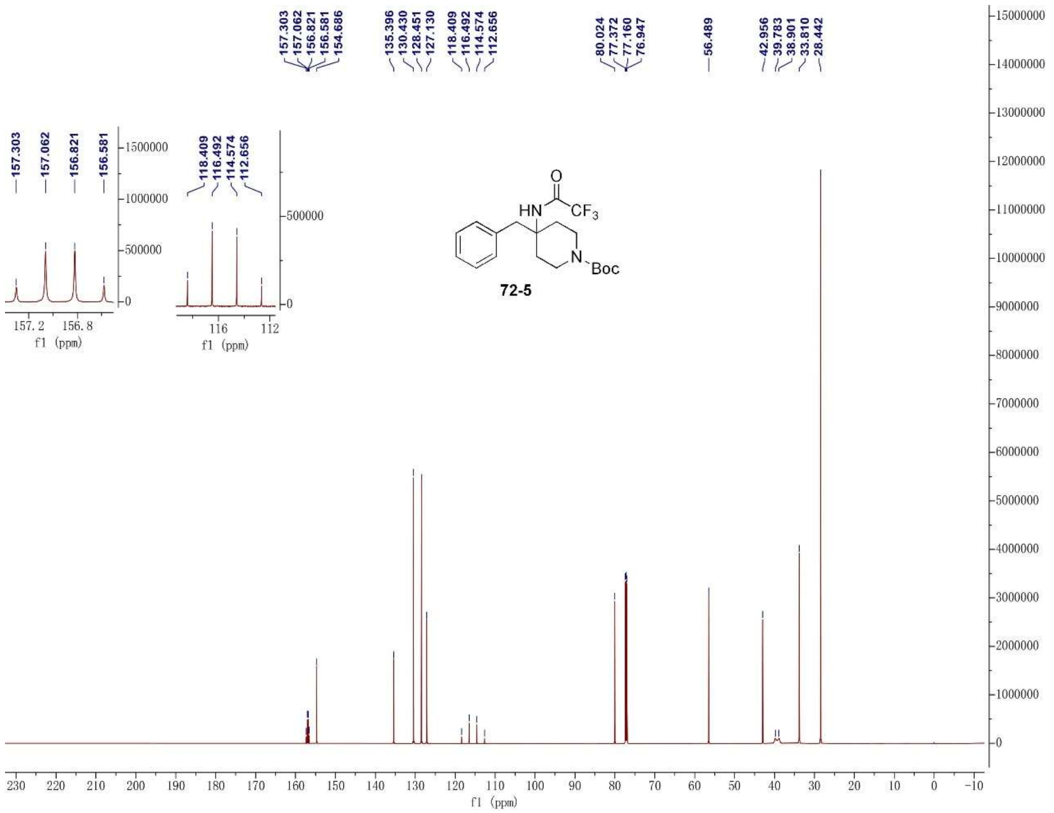

Figure S171: ^{13}C NMR spectrum of **72-5**

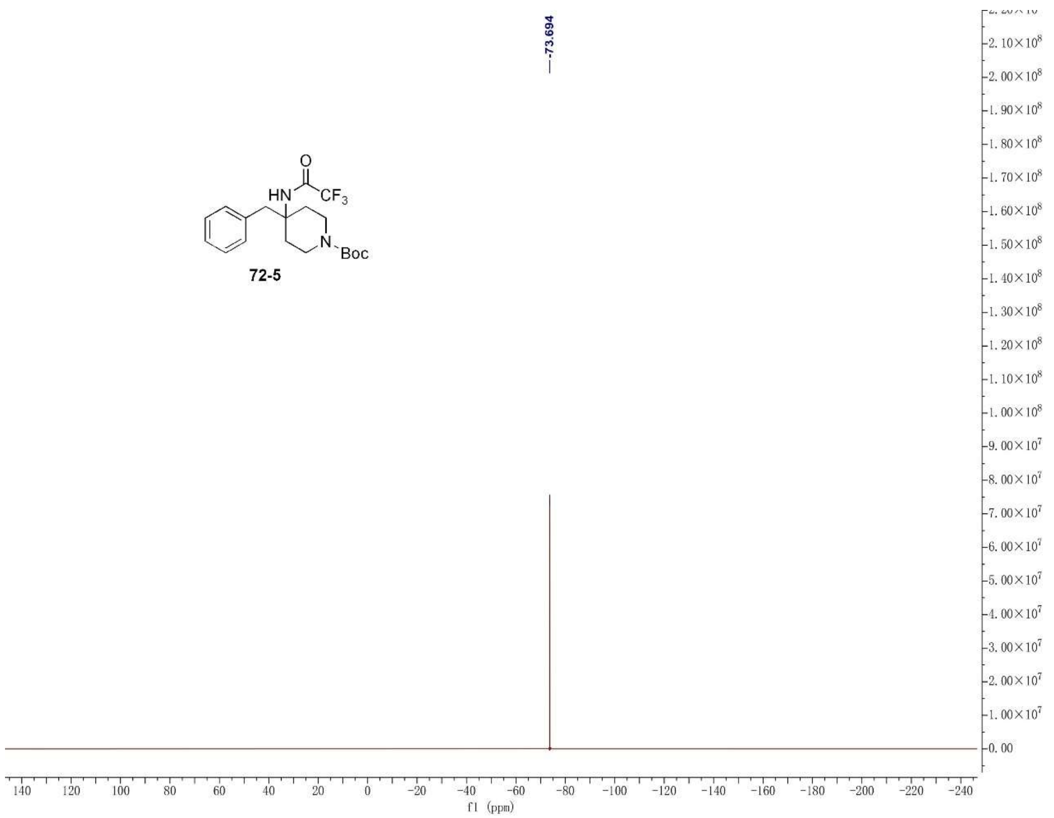

Figure S172: ^{19}F NMR spectrum of **72-5**

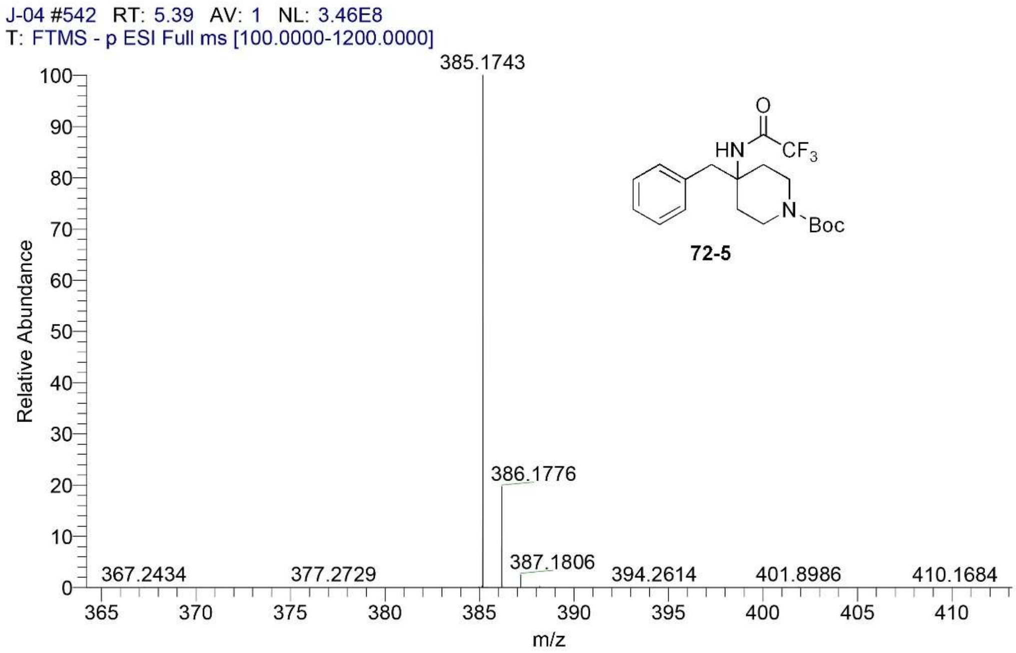

Figure S173: HR-MS (ESI/ion trap) spectrum of **72-5**

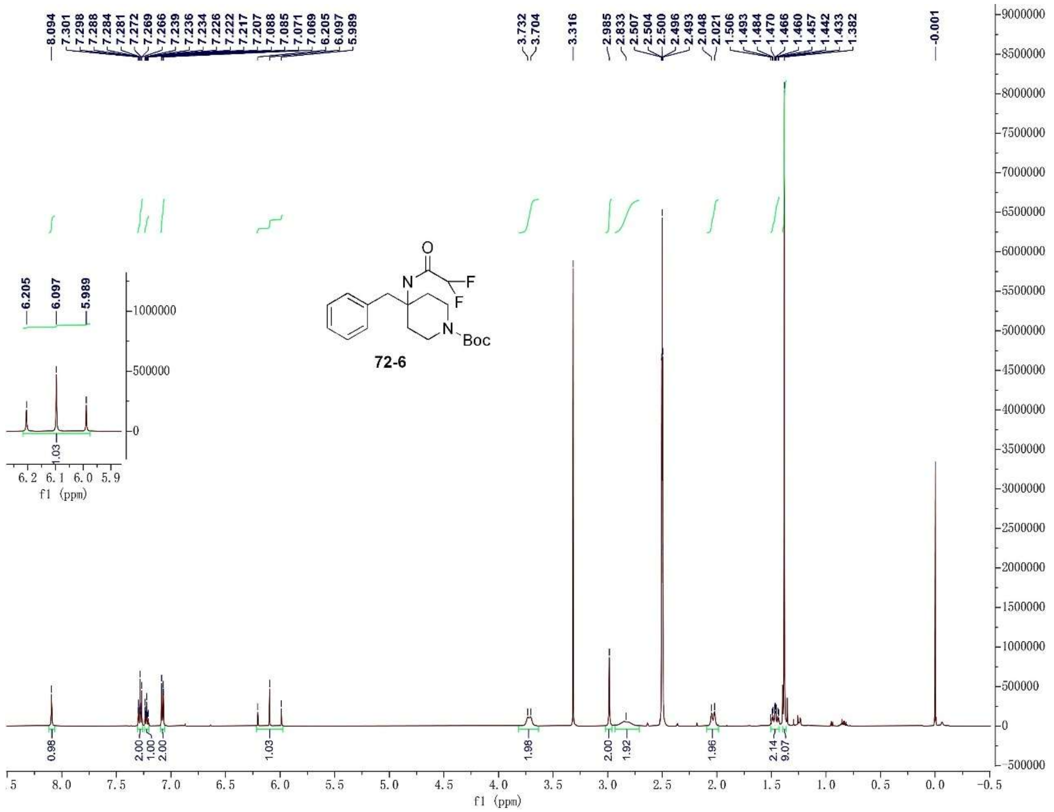

Figure S174: ^{1}H NMR spectrum of **72-6**

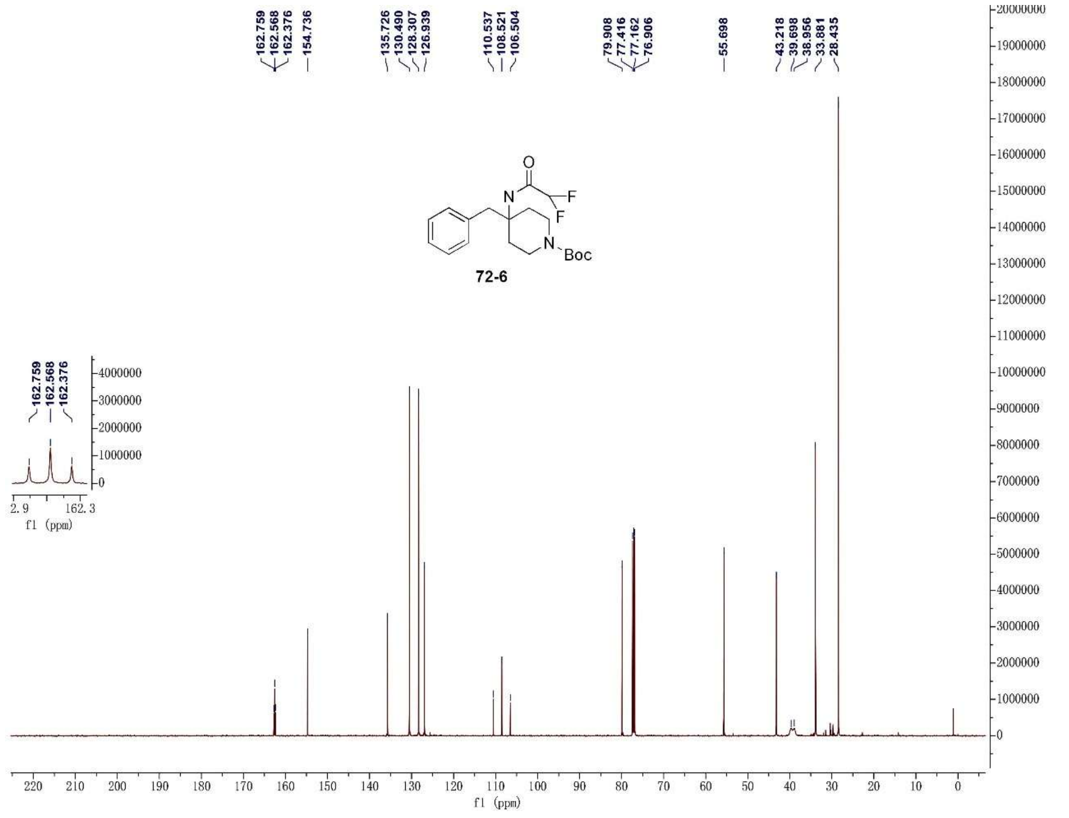

Figure S175: ^{13}C NMR spectrum of **72-6**

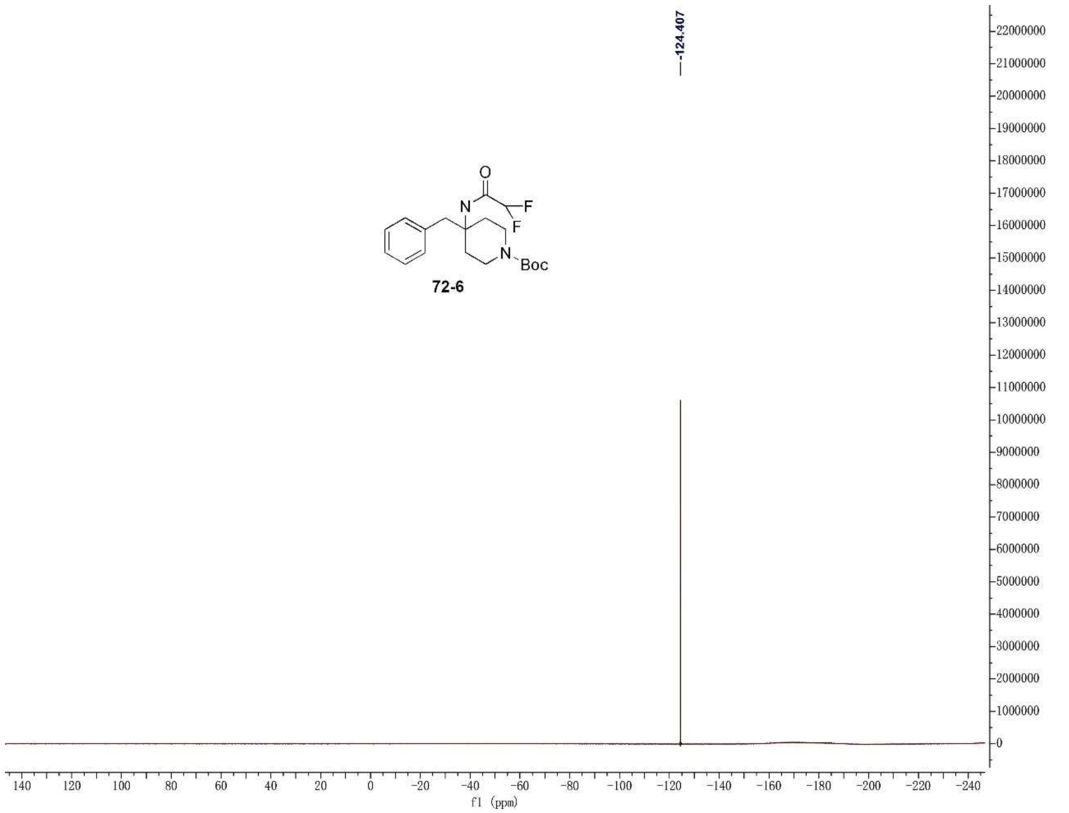

Figure S176: ^{19}F NMR spectrum of **72-6**

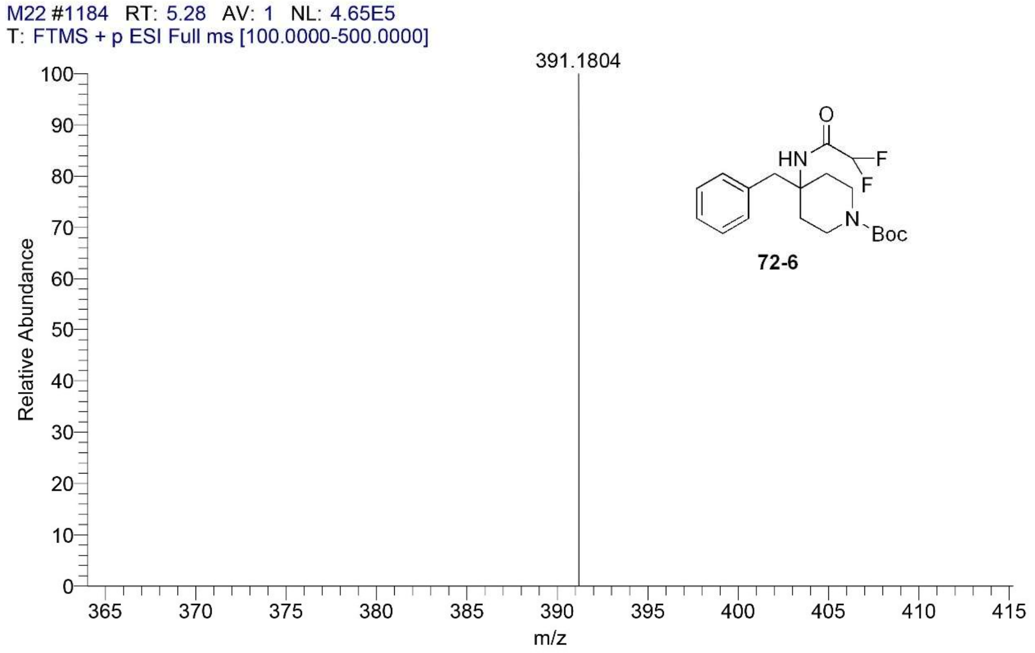

Figure S177: HR-MS (ESI/ion trap) spectrum of **72-6**

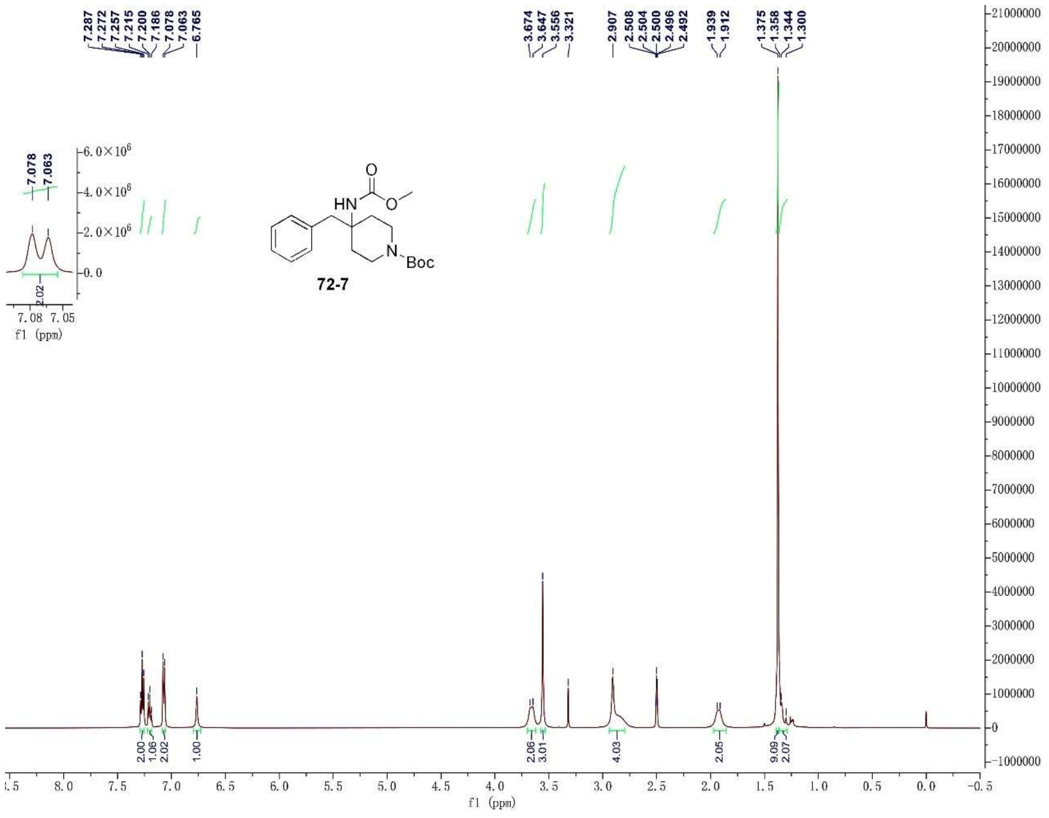

Figure S178: ^{1}H NMR spectrum of **72-7**

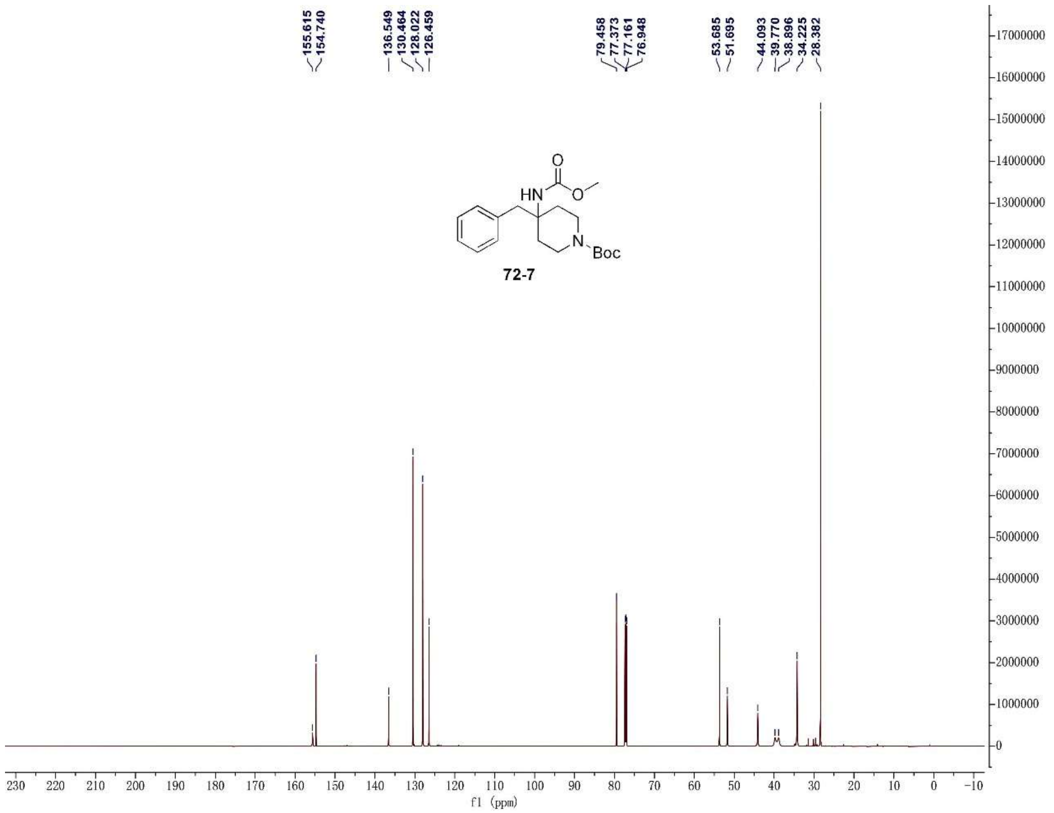

Figure S179: ^{13}C NMR spectrum of **72-7**

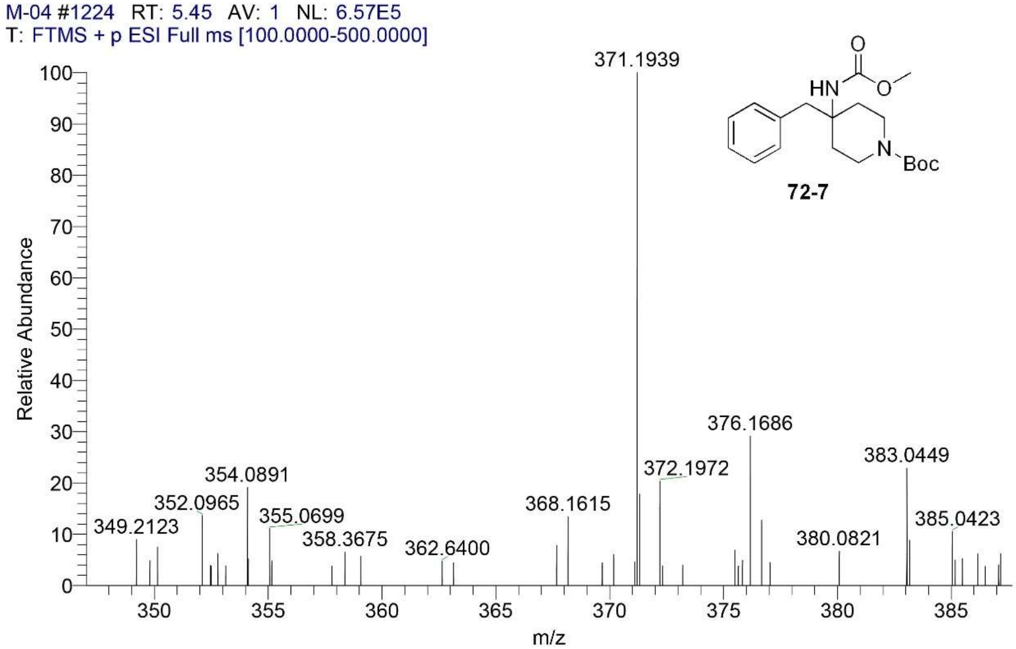

Figure S180: HR-MS (ESI/ion trap) spectrum of **72-7**

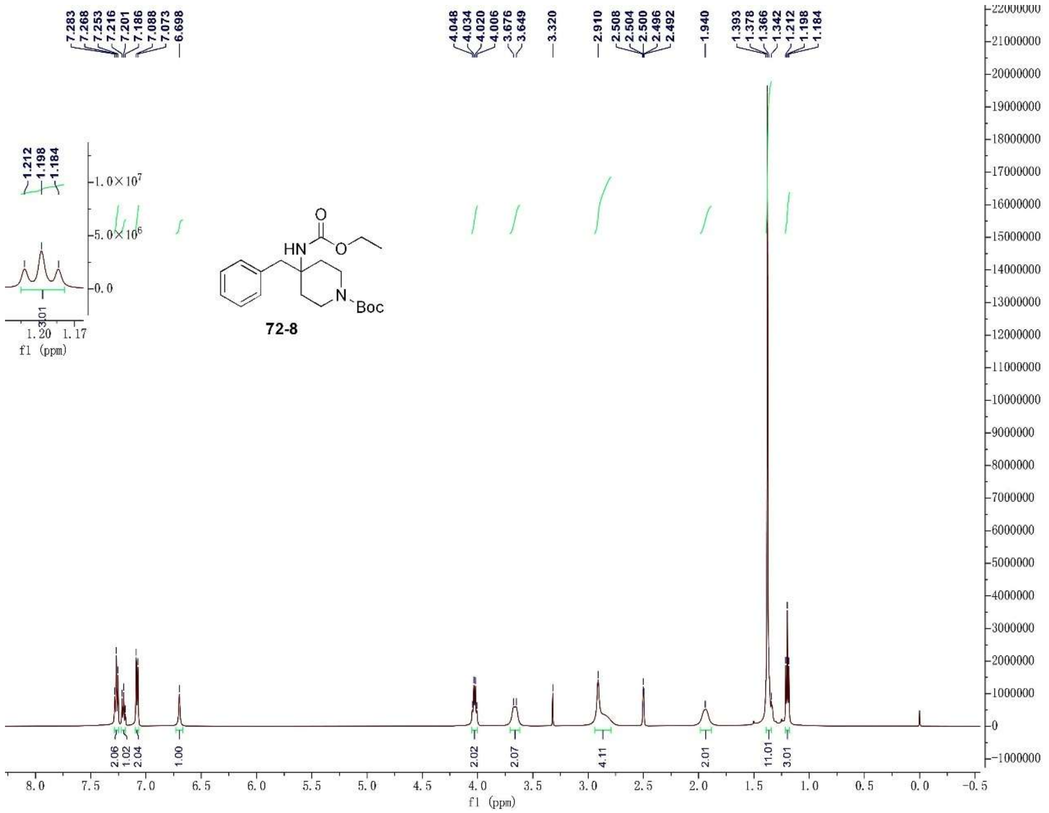

Figure S181: ^{1}H NMR spectrum of **72-8**

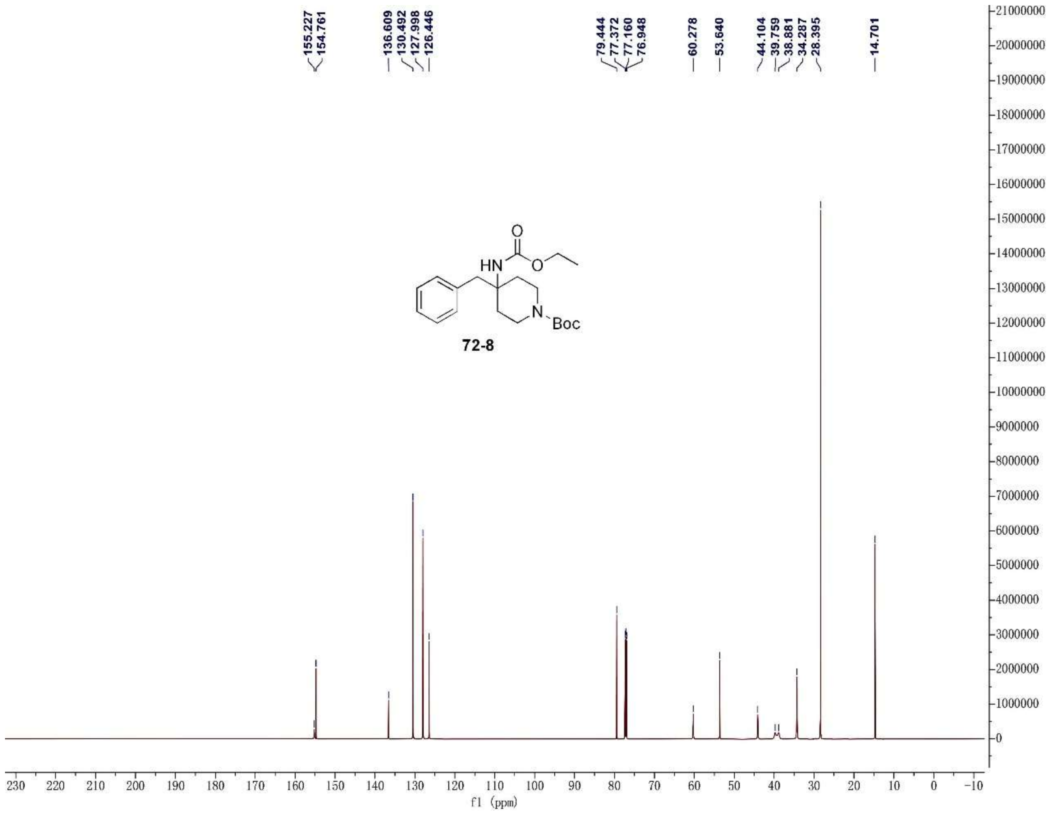

Figure S182: ^{13}C NMR spectrum of **72-8**

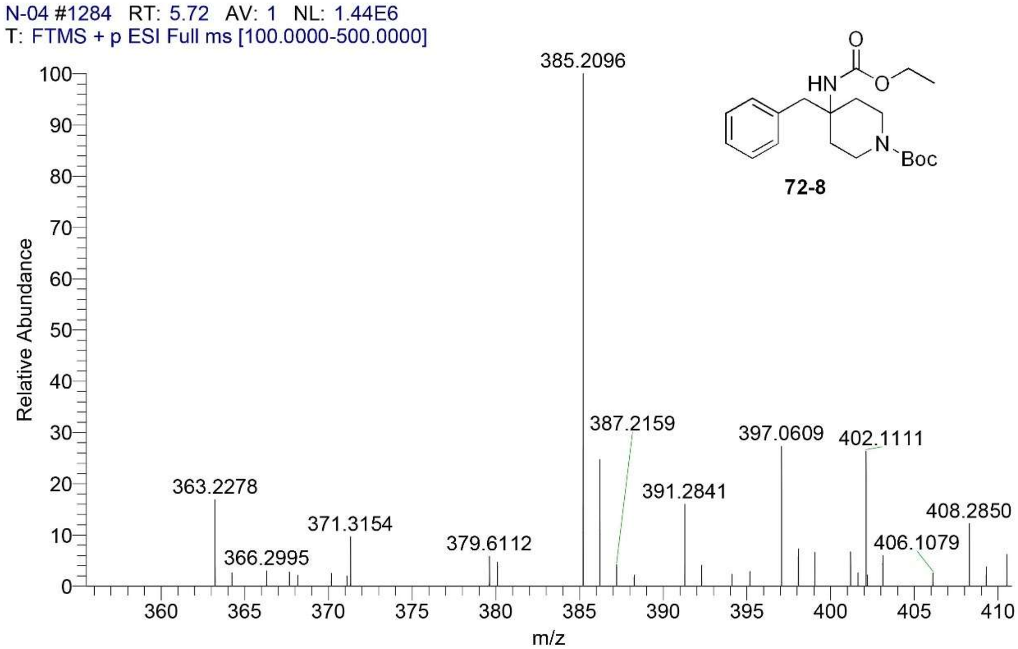

Figure S183: HR-MS (ESI/ion trap) spectrum of **72-8**

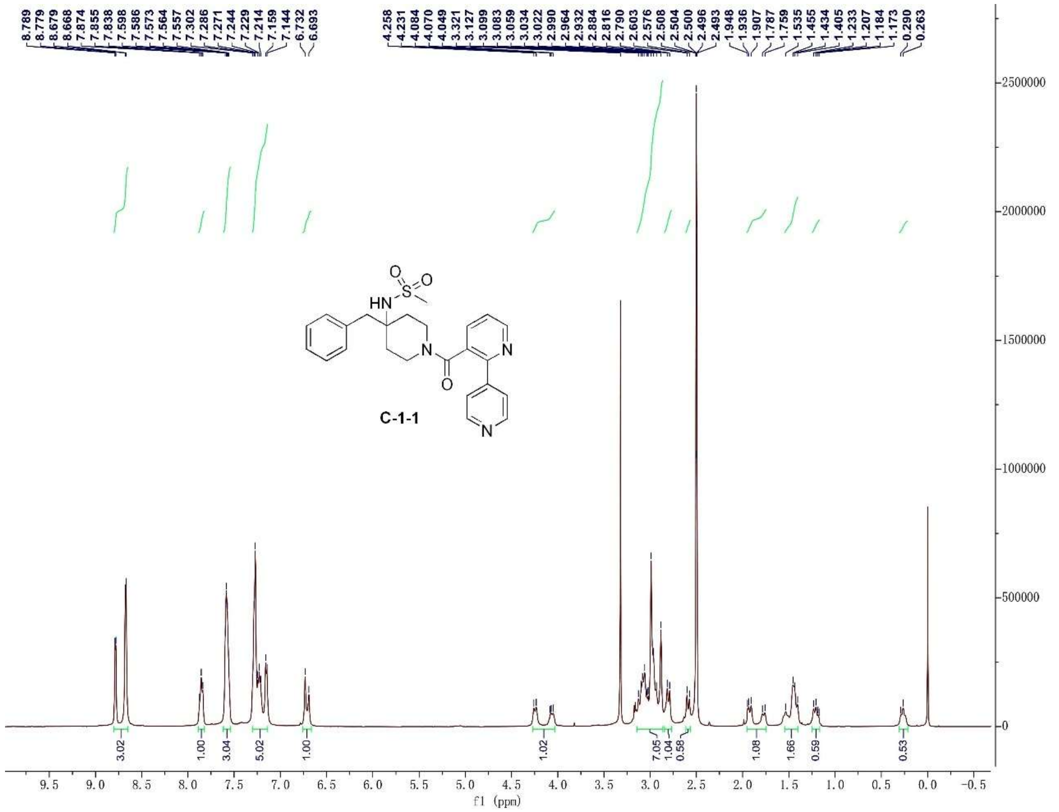

Figure S184: ^{1}H NMR spectrum of **C-1-1**

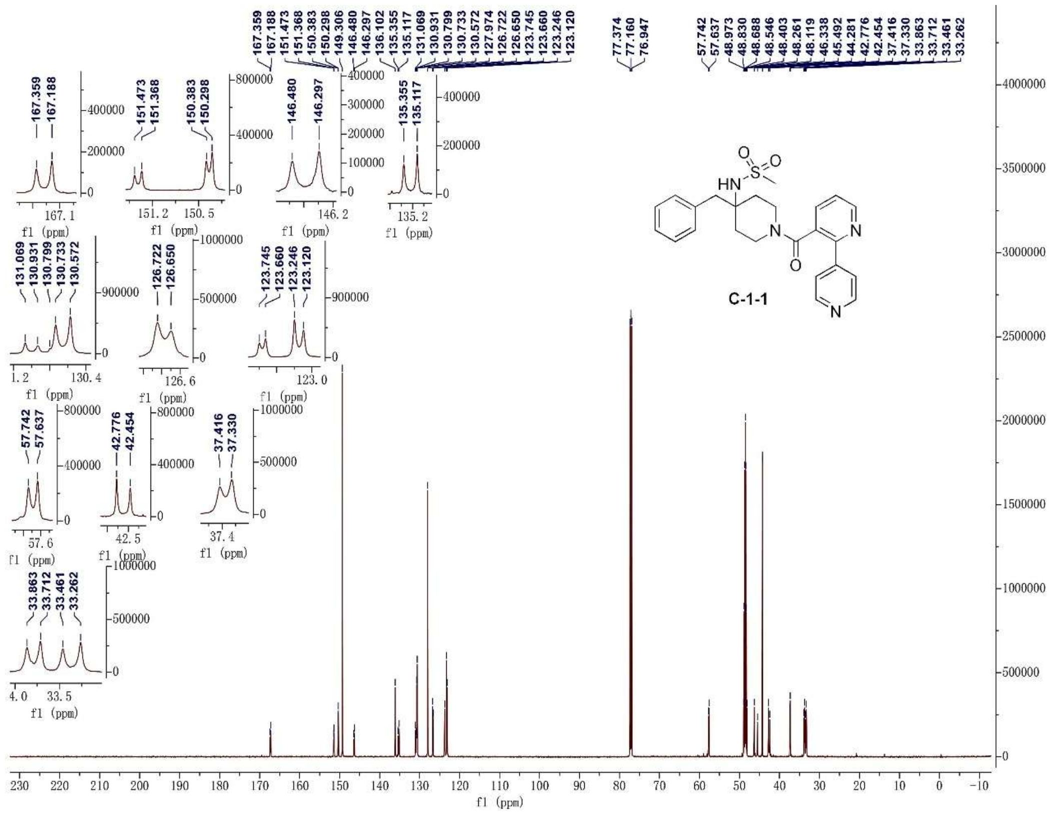

Figure S185: ^{13}C NMR spectrum of **C-1-1**

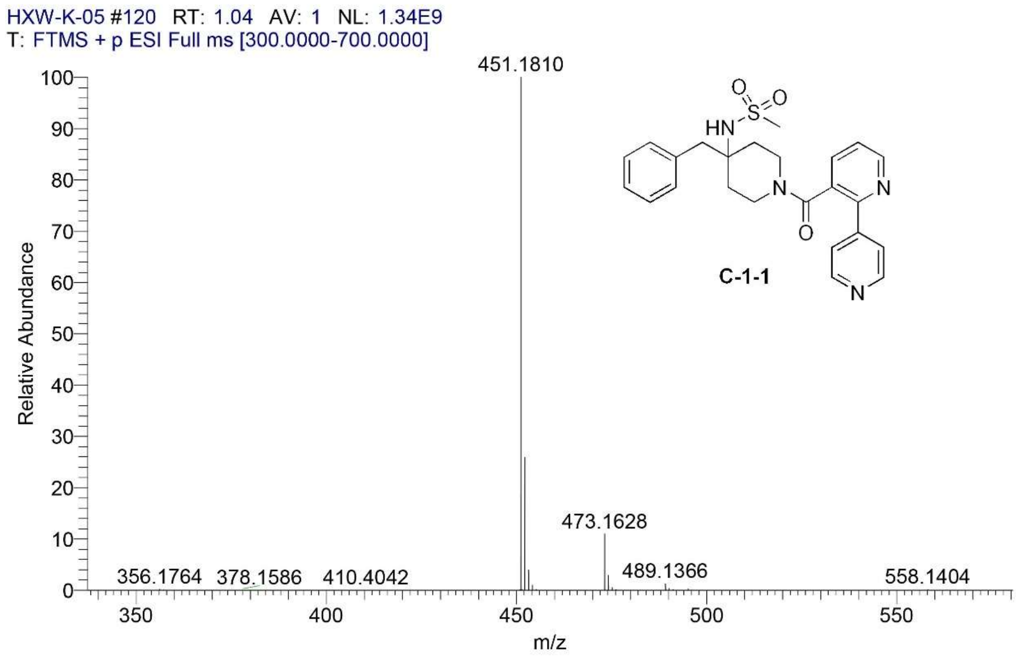

Figure S186: HR-MS (ESI/ion trap) spectrum of **C-1-1**

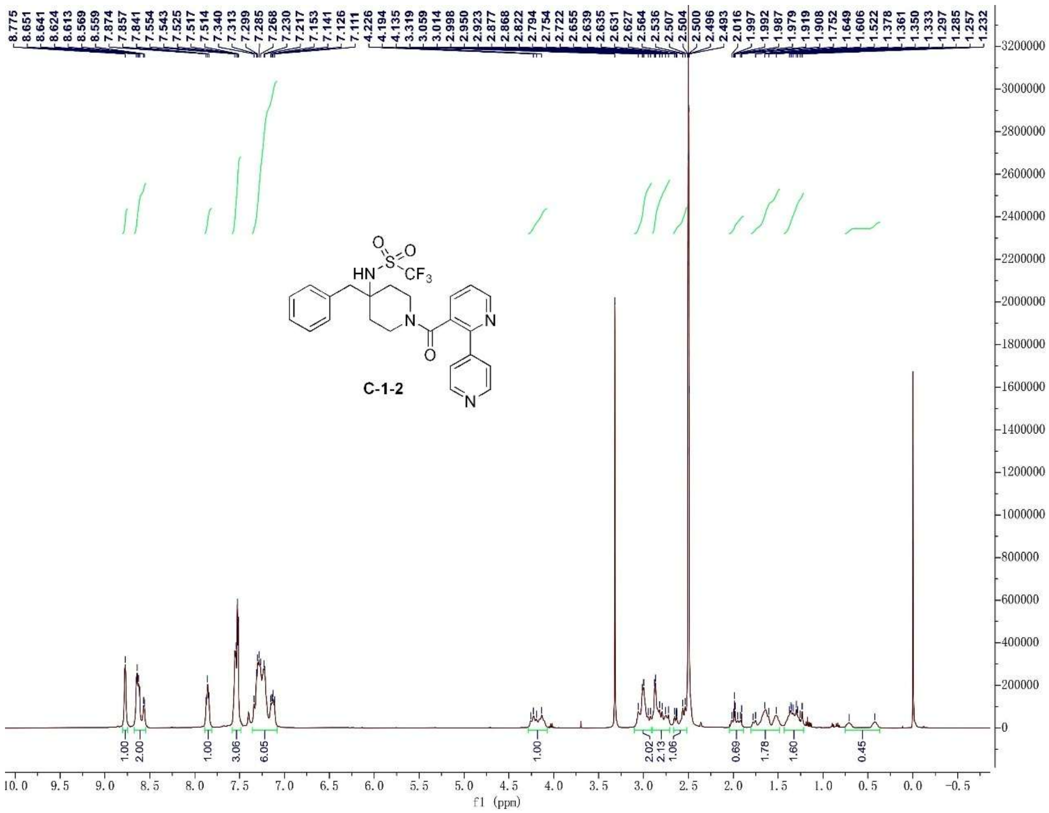

Figure S187: ^{1}H NMR spectrum of **C-1-2**

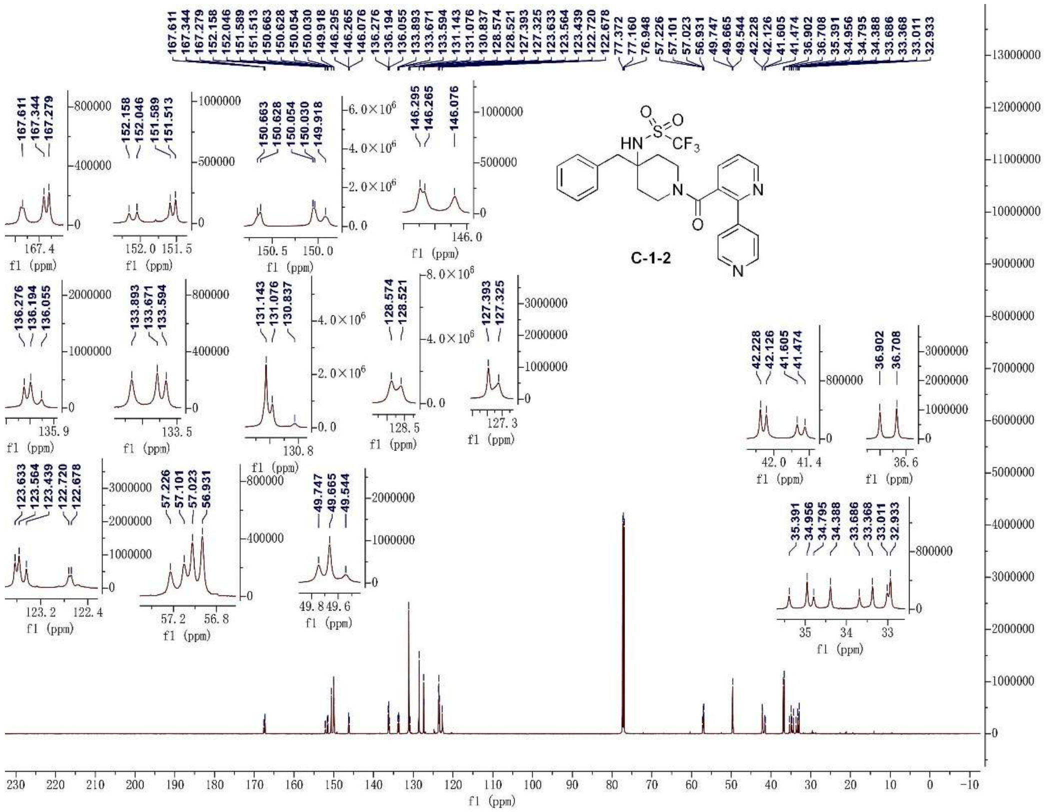

Figure S188: ^{13}C NMR spectrum of **C-1-2**

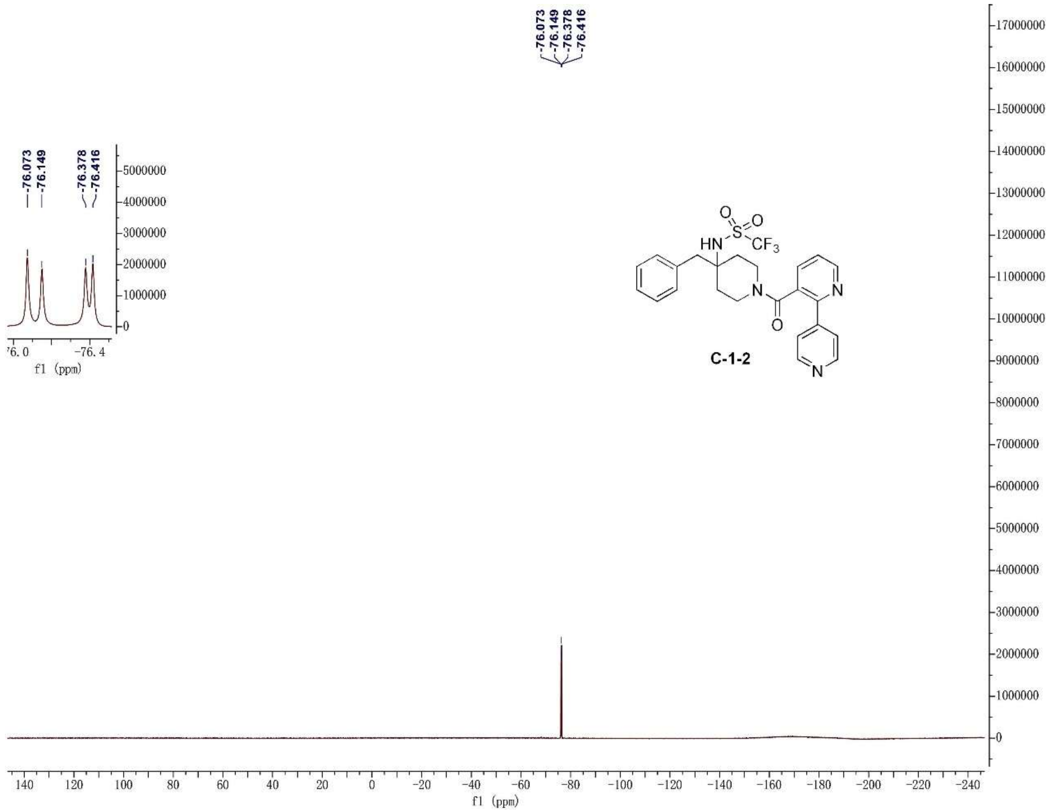

Figure S189: ^{19}F NMR spectrum of **C-1-2**

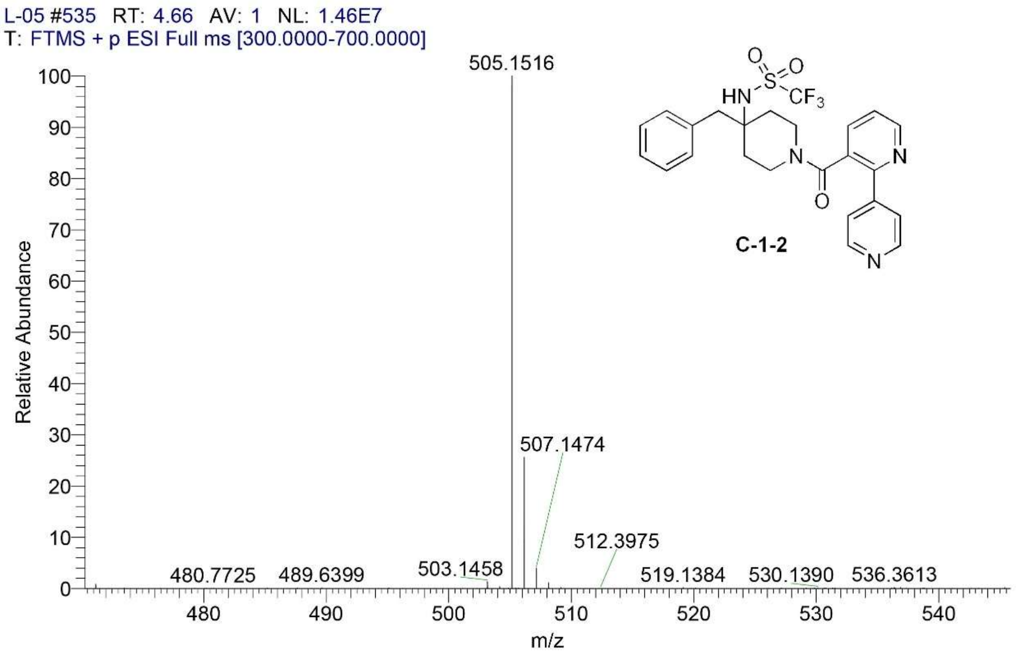

Figure S190: HR-MS (ESI/ion trap) spectrum of **C-1-2**

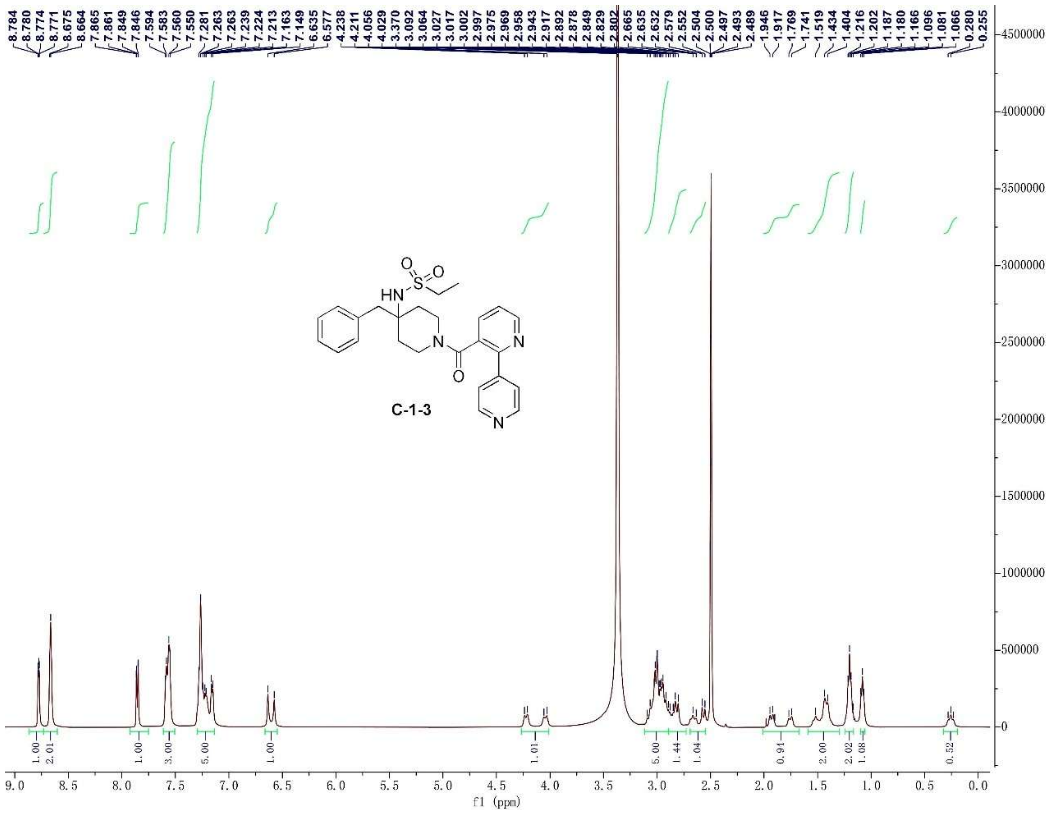

Figure S191: ^{1}H NMR spectrum of **C-1-3**

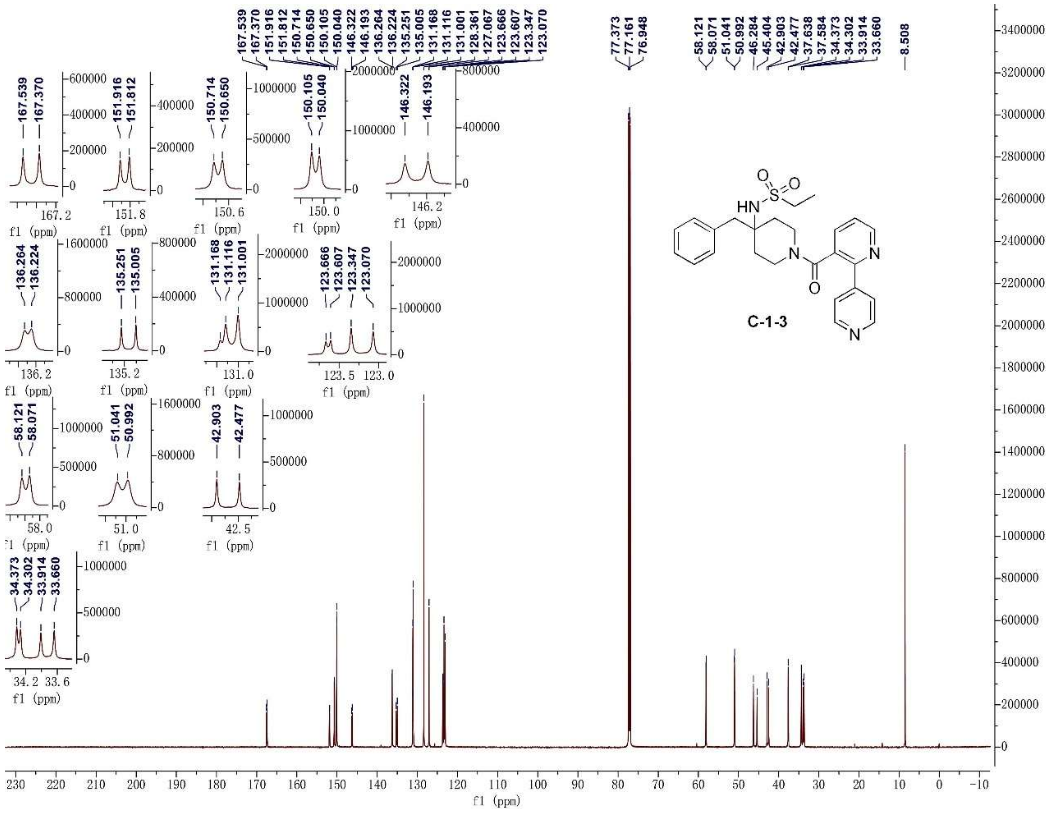

Figure S192: ^{13}C NMR spectrum of **C-1-3**

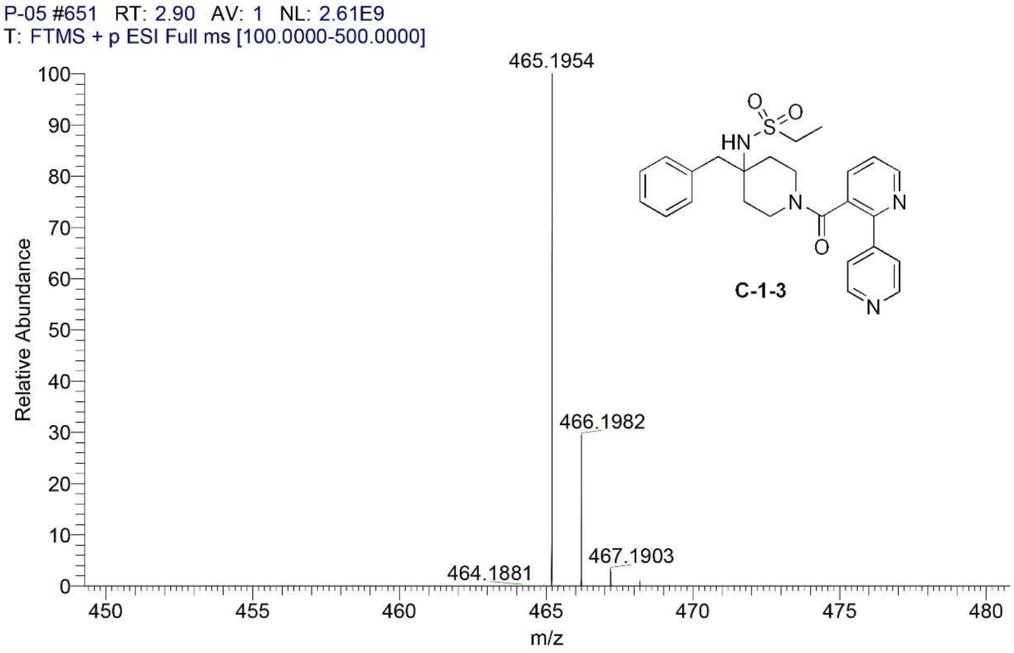

Figure S193: HR-MS (ESI/ion trap) spectrum of **C-1-3**

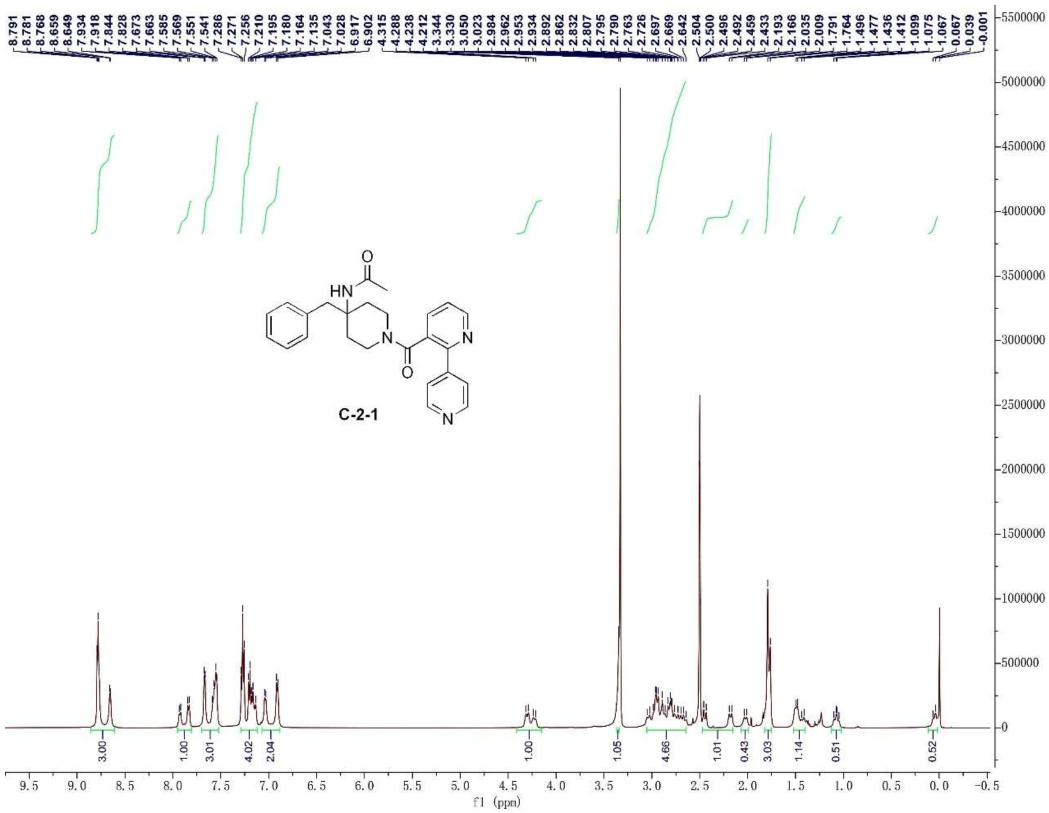

Figure S194: ^{1}H NMR spectrum of **C-2-1**

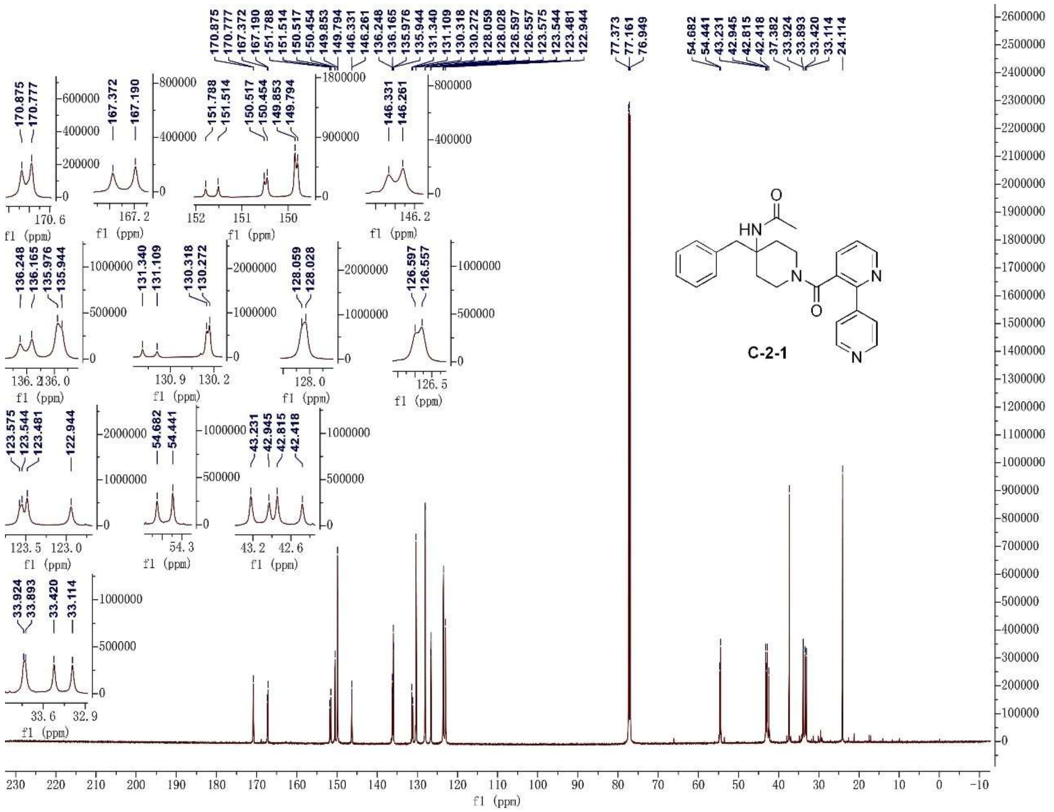

Figure S195: ^{13}C NMR spectrum of **C-2-1**

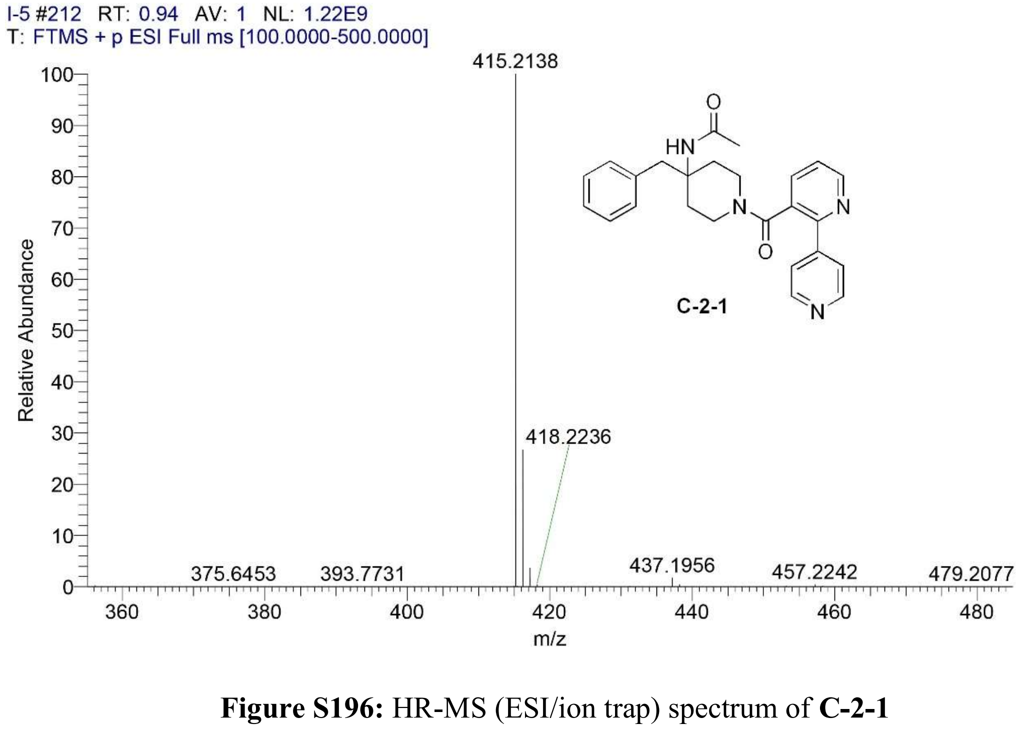

Figure S196: HR-MS (ESI/ion trap) spectrum of **C-2-1**

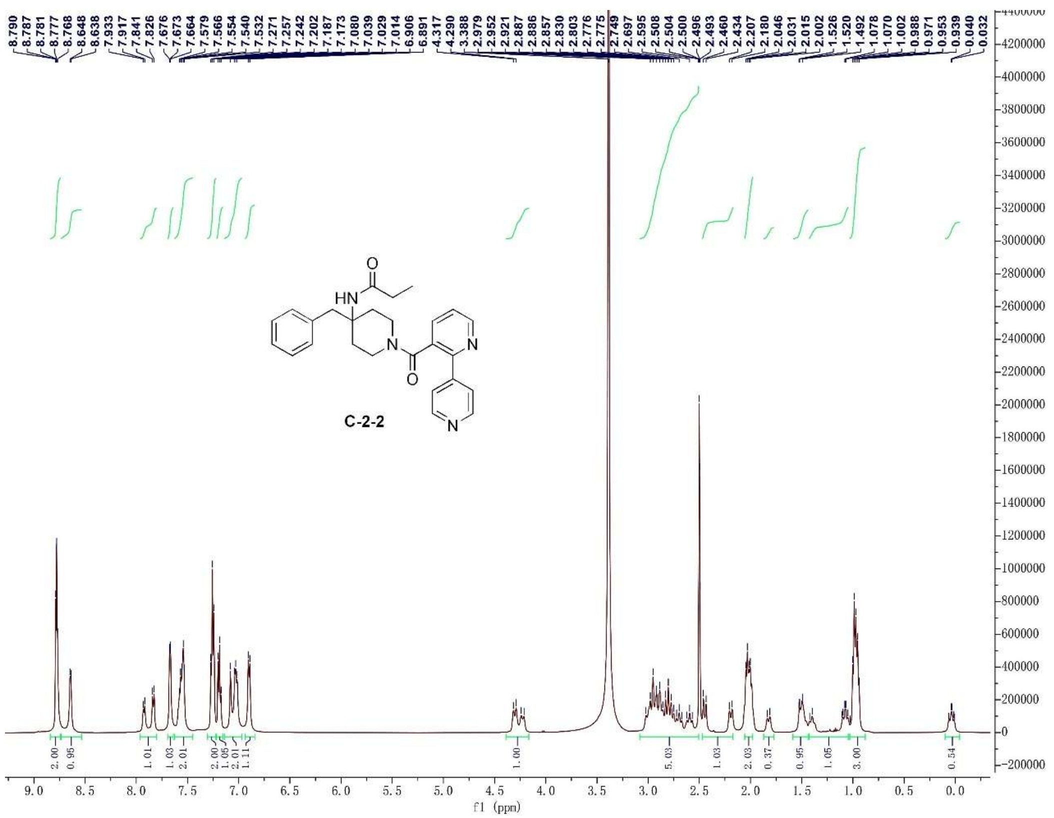

Figure S197: ^{1}H NMR spectrum of **C-2-2**

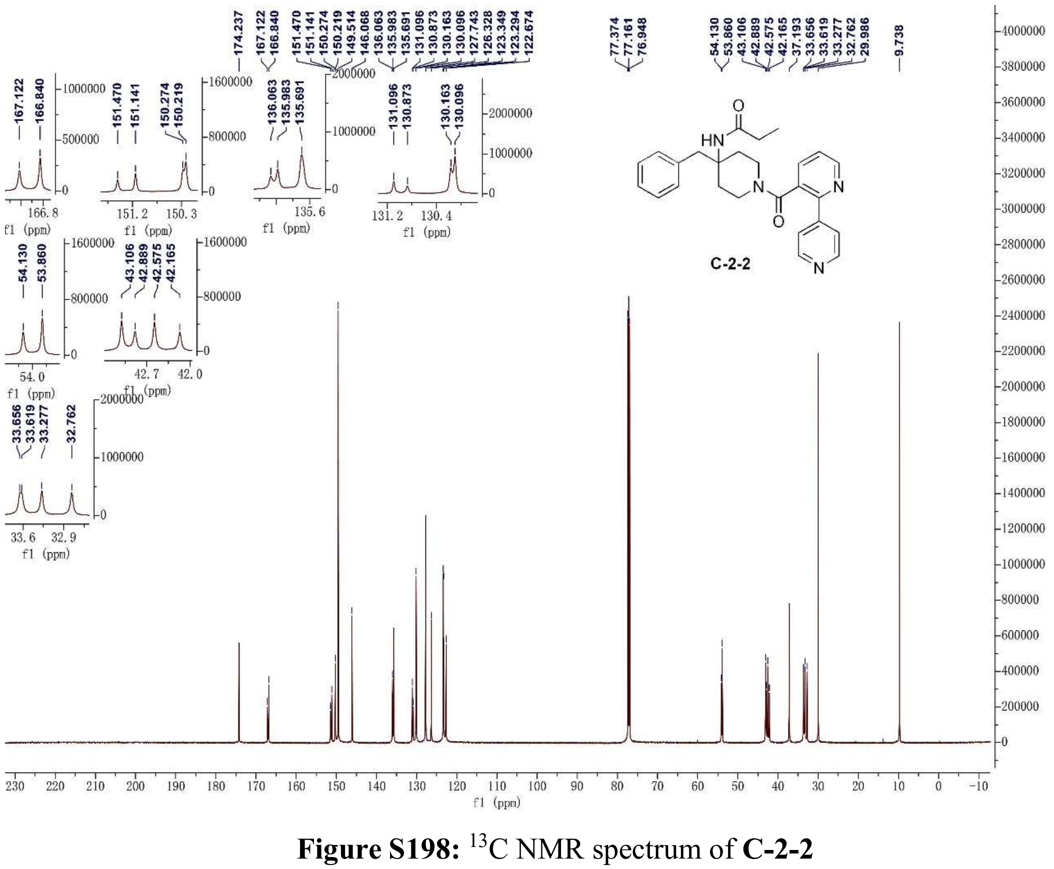

Figure S198: ^{13}C NMR spectrum of **C-2-2**

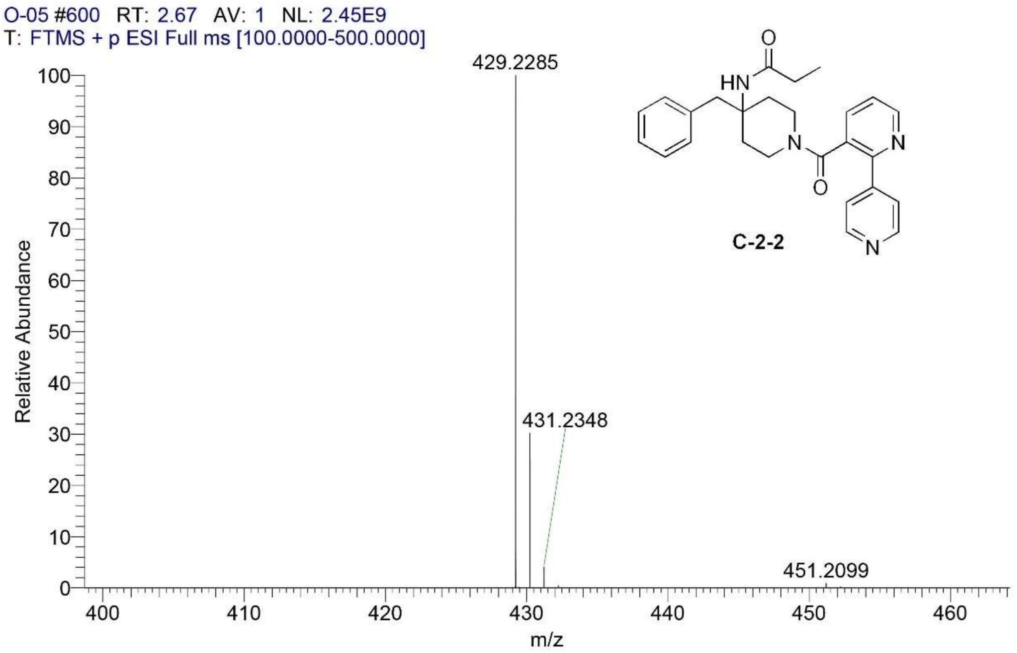

Figure S199: HR-MS (ESI/ion trap) spectrum of **C-2-2**

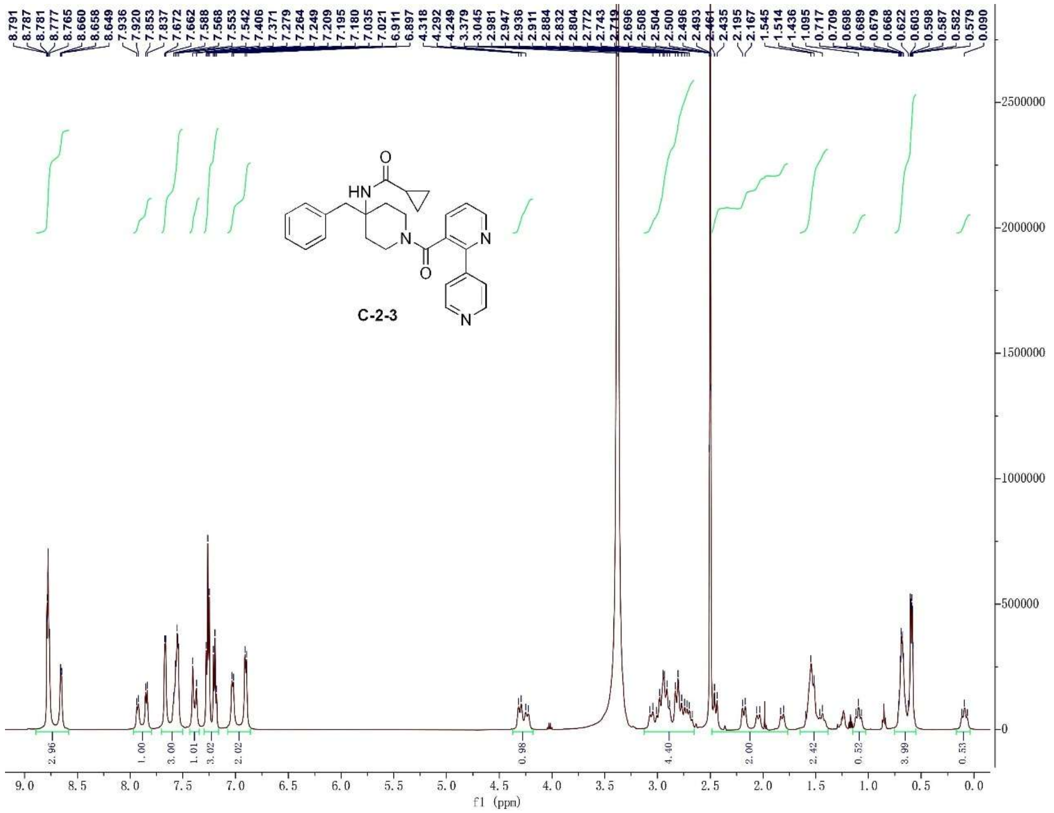

Figure S200: ^{1}H NMR spectrum of **C-2-3**

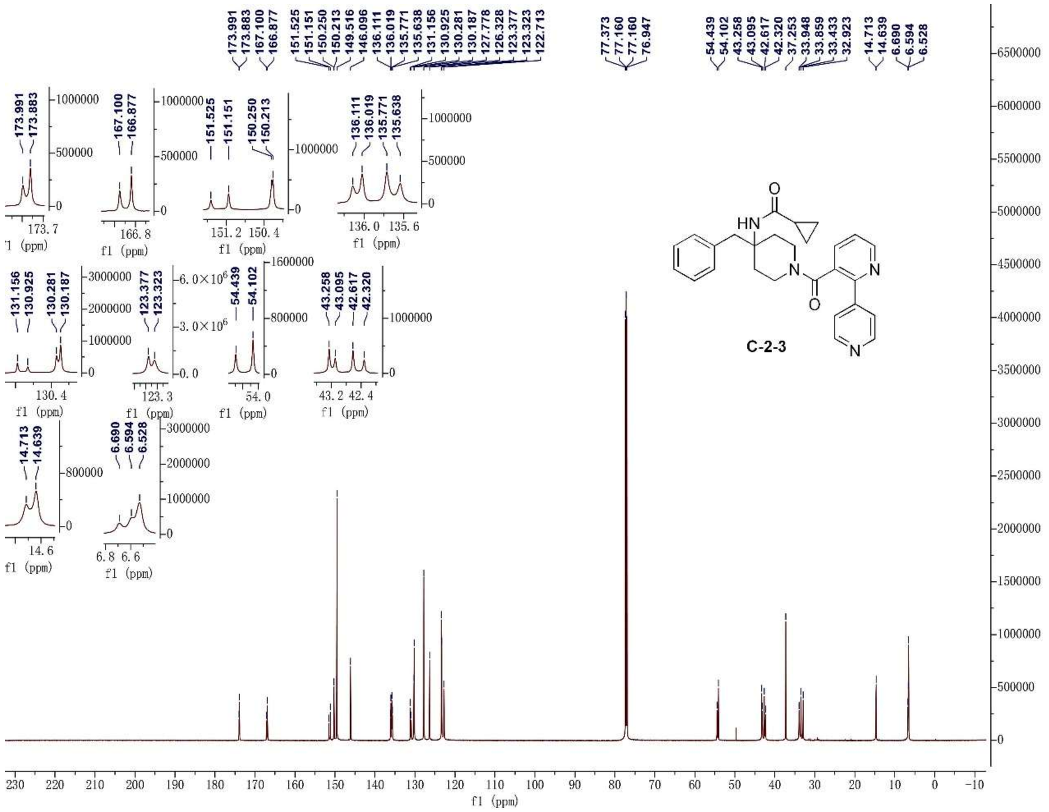

Figure S201: ^{13}C NMR spectrum of **C-2-3**

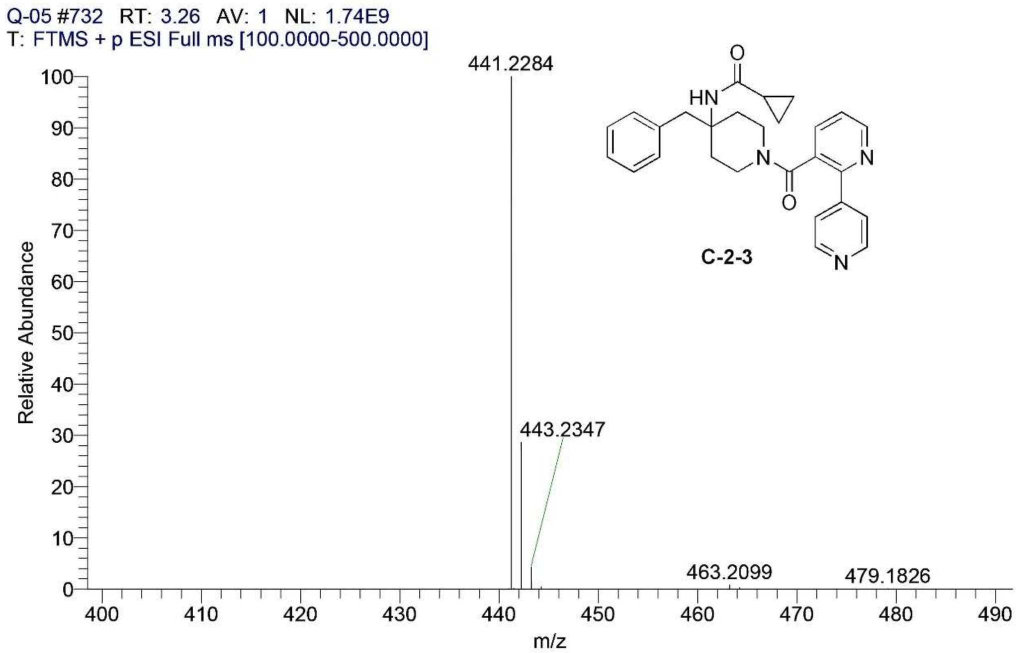

Figure S202: HR-MS (ESI/ion trap) spectrum of **C-2-3**

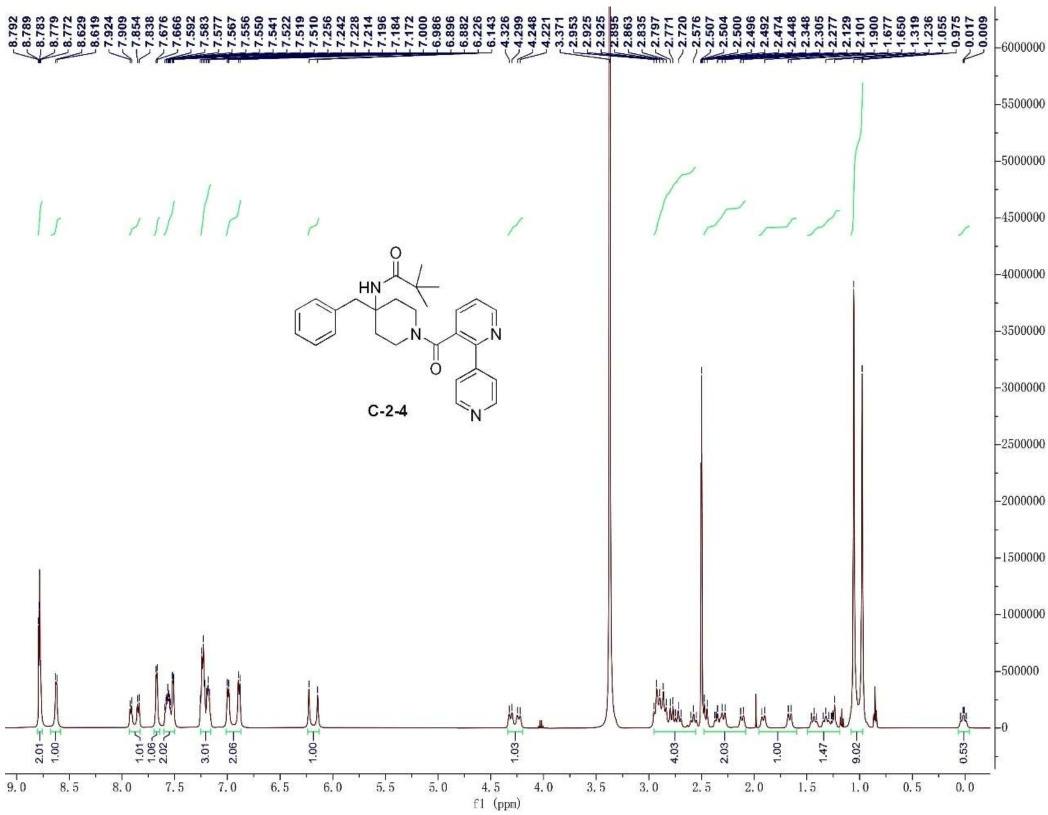

Figure S203: ^{1}H NMR spectrum of **C-2-4**

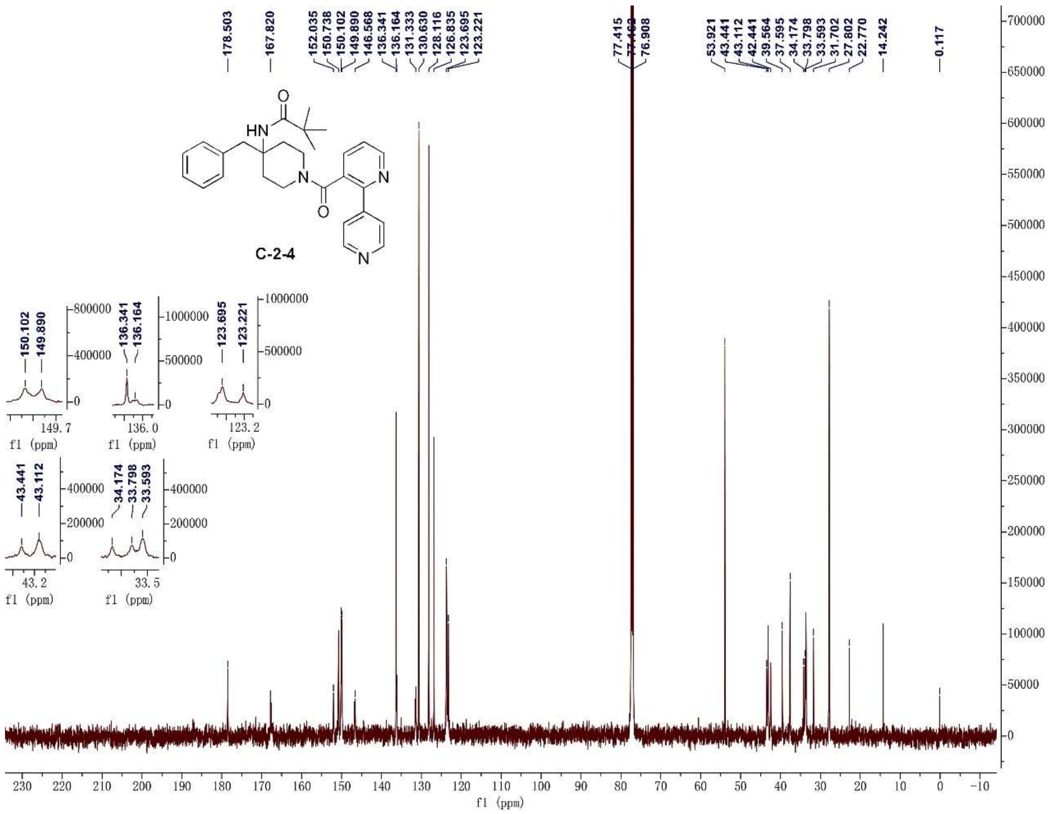

Figure S204: ^{13}C NMR spectrum of **C-2-4**

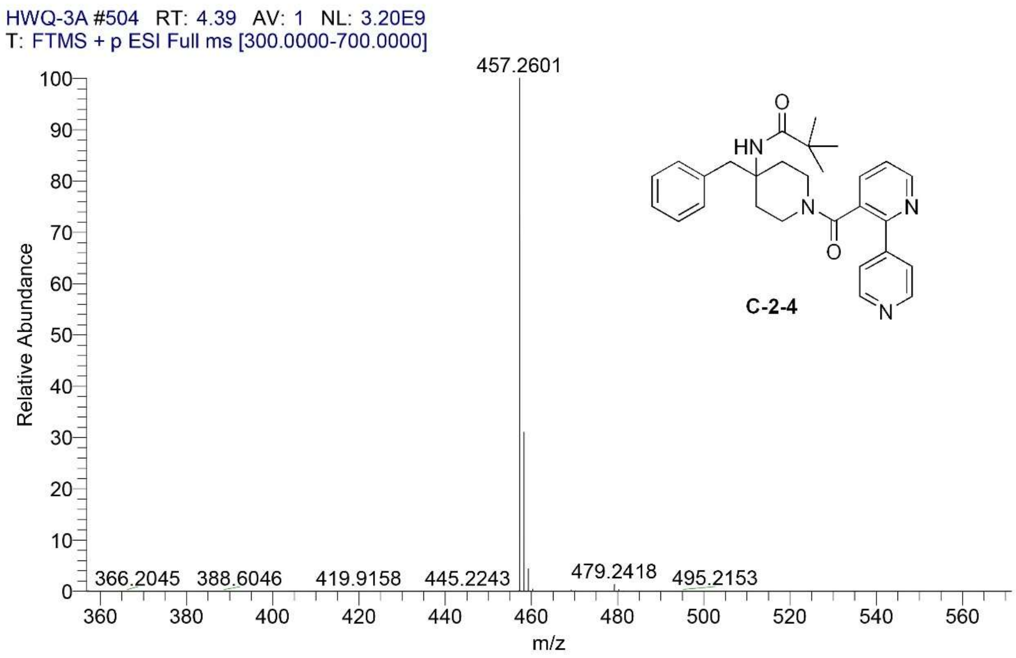

Figure S205: HR-MS (ESI/ion trap) spectrum of **C-2-4**

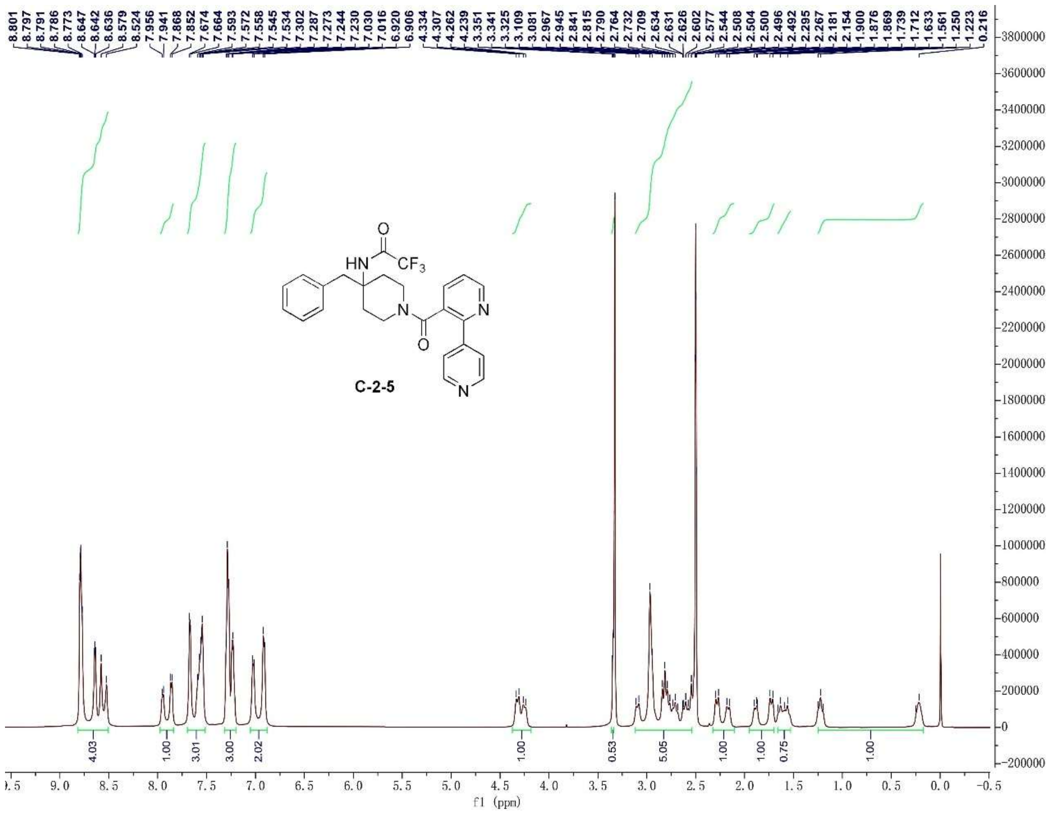

Figure S206: ^{1}H NMR spectrum of **C-2-5**

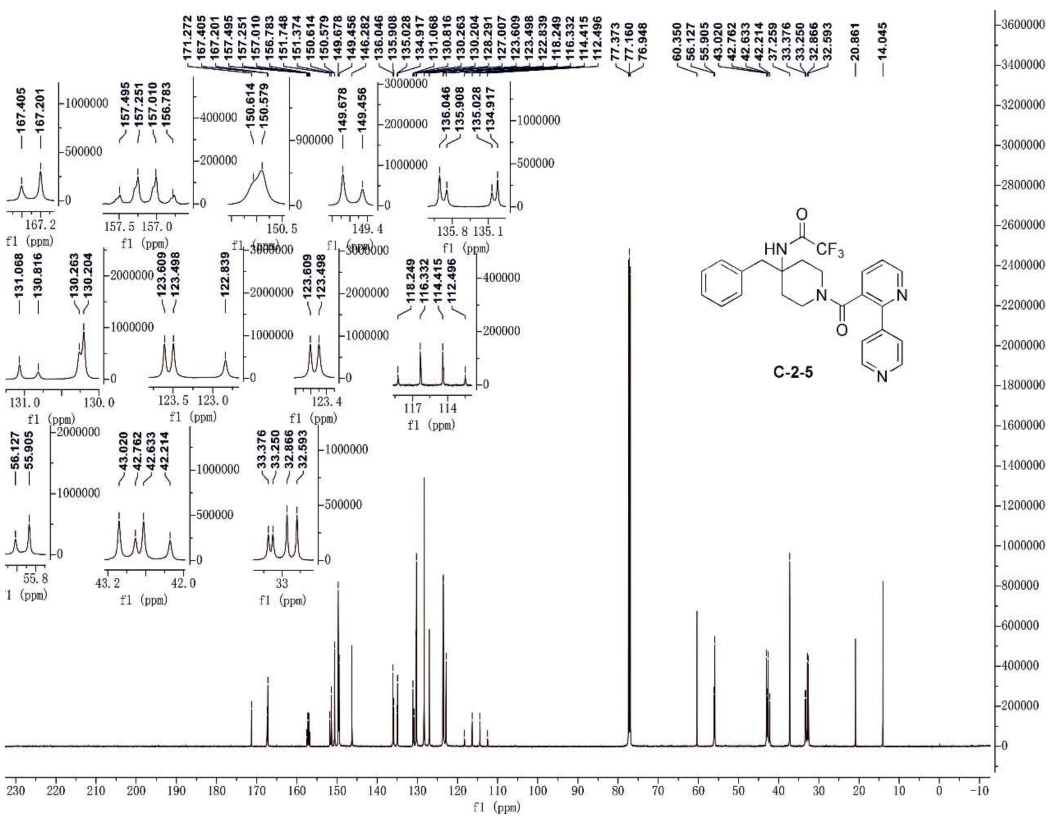

Figure S207: ^{13}C NMR spectrum of **C-2-5**

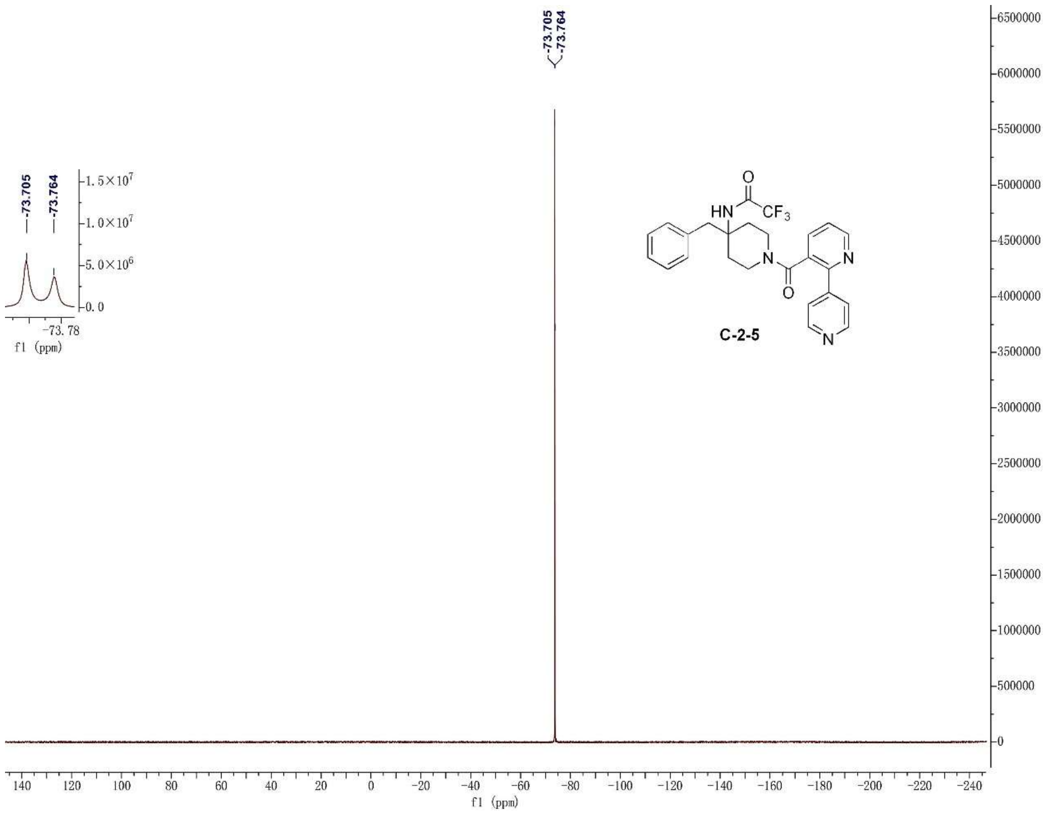

Figure S208: ^{19}F NMR spectrum of **C-2-5**

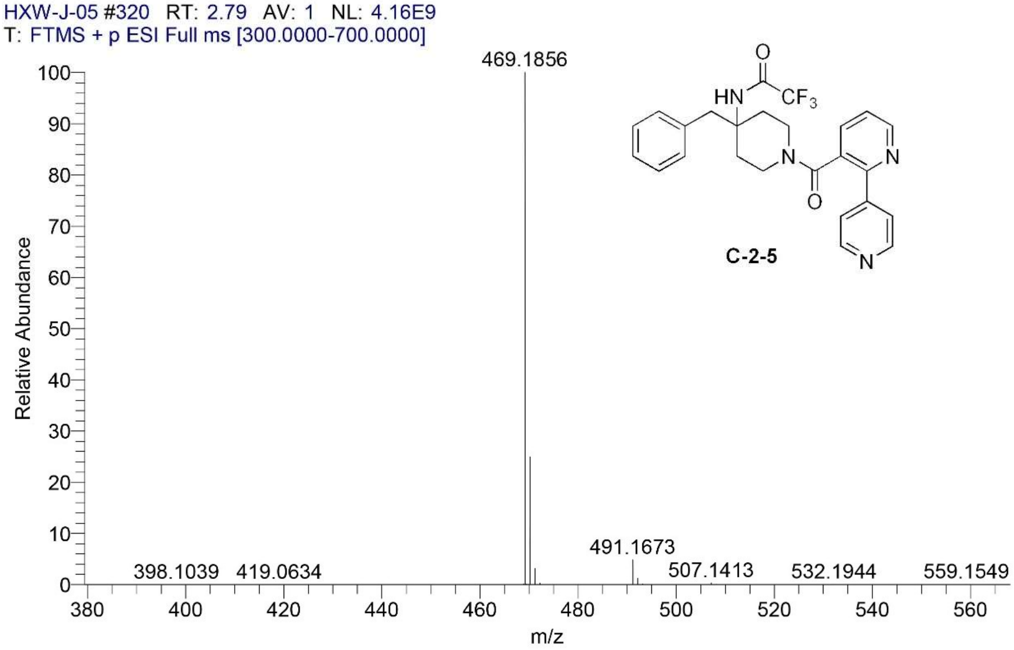

Figure S209: HR-MS (ESI/ion trap) spectrum of **C-2-5**

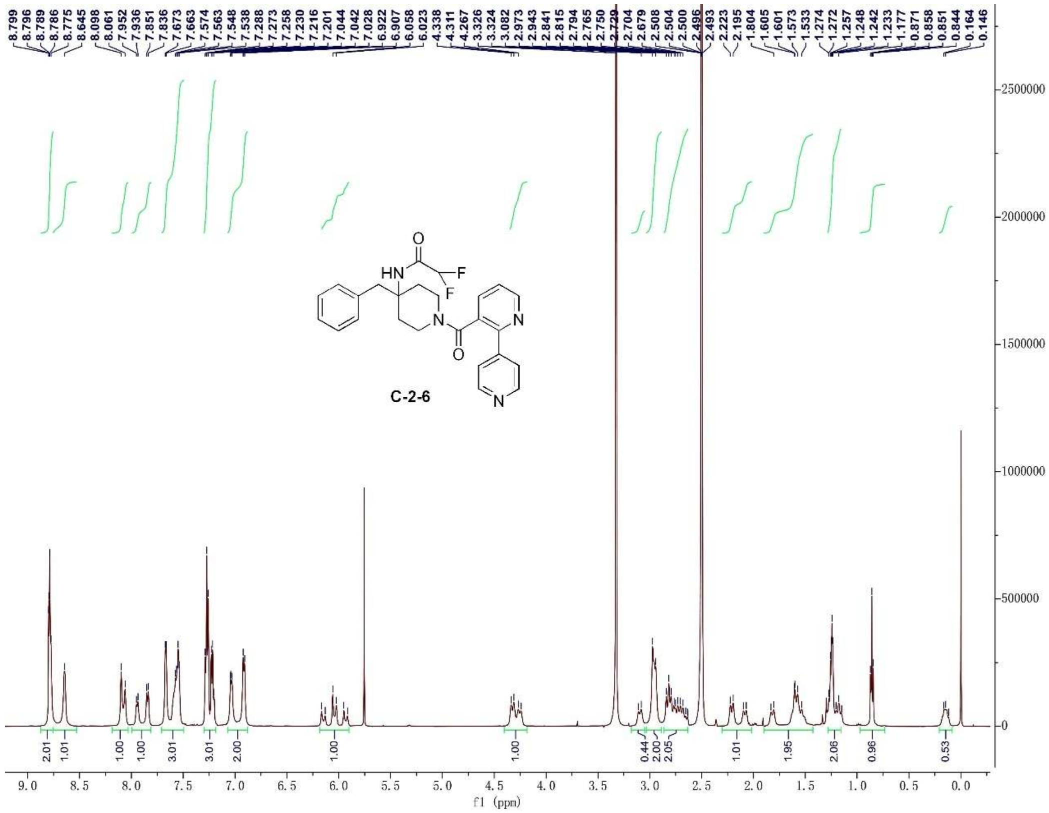

Figure S210: ^{1}H NMR spectrum of **C-2-6**

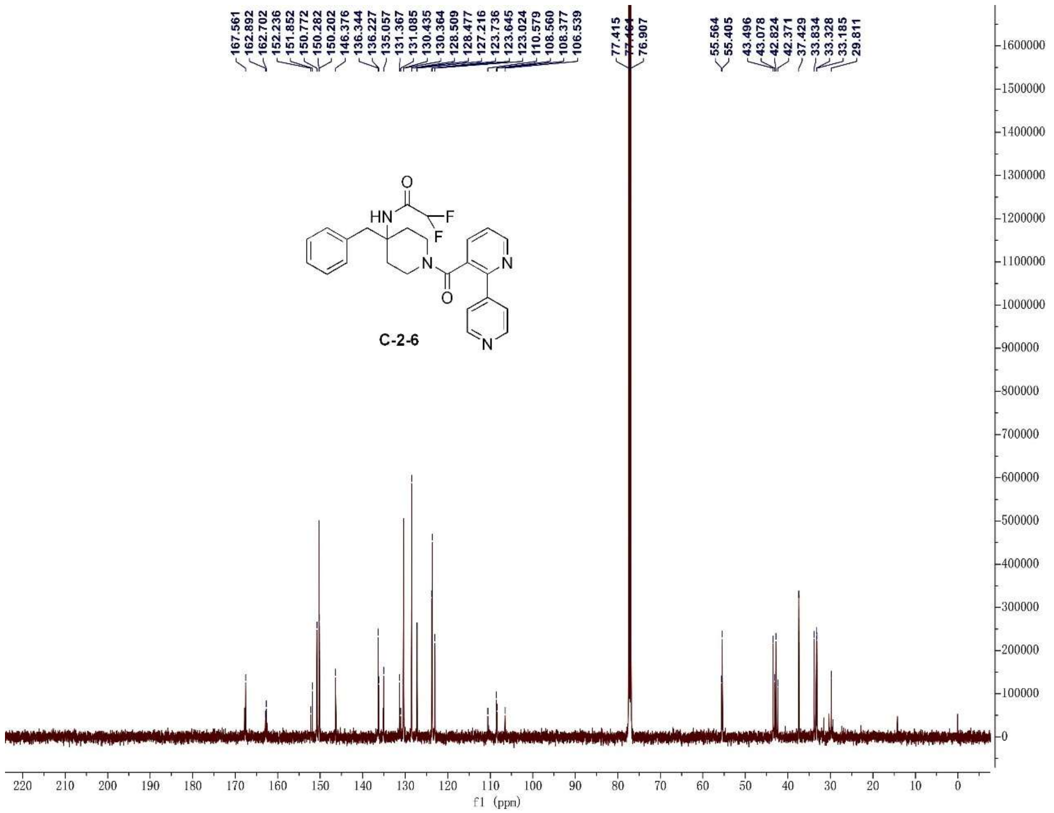

Figure S211: ^{13}C NMR spectrum of **C-2-6**

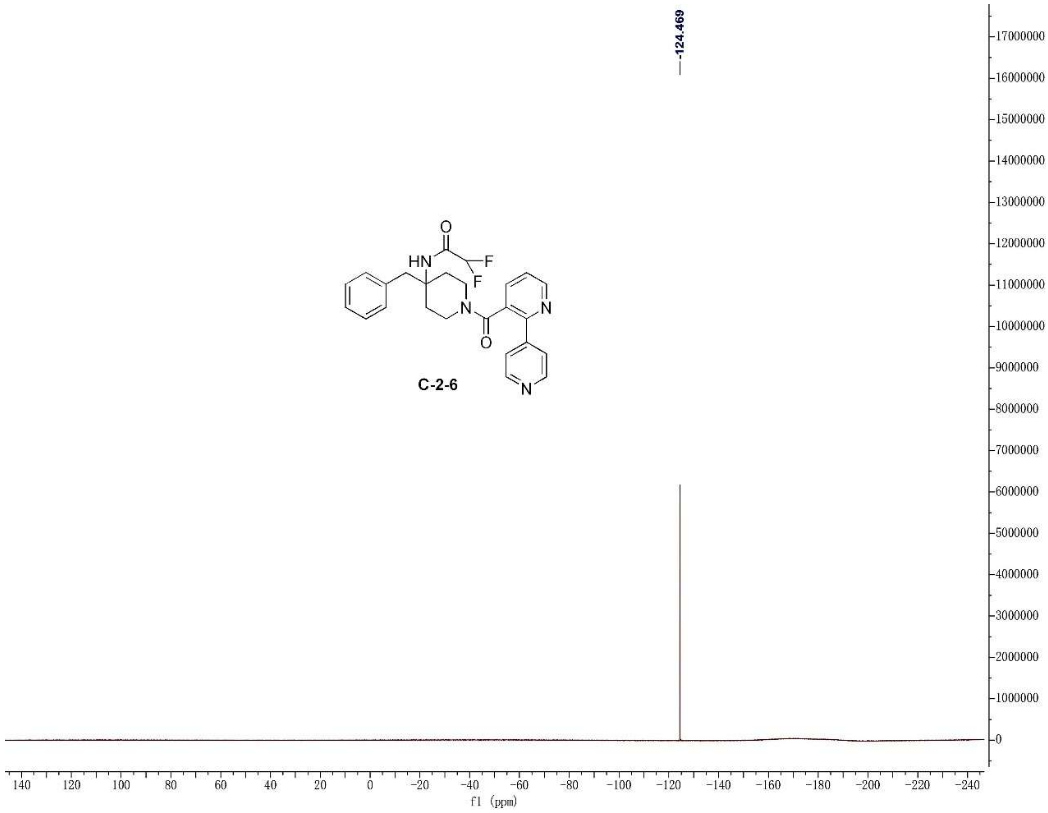

Figure S212: ^{13}C NMR spectrum of **C-2-6**

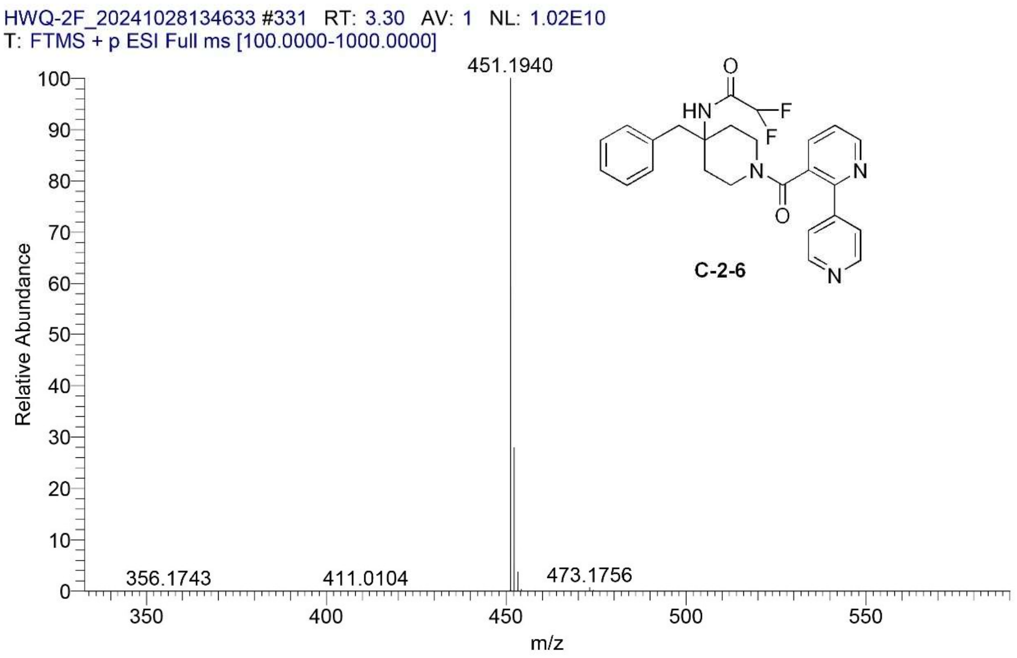

Figure S213: HR-MS (ESI/ion trap) spectrum of **C-2-6**

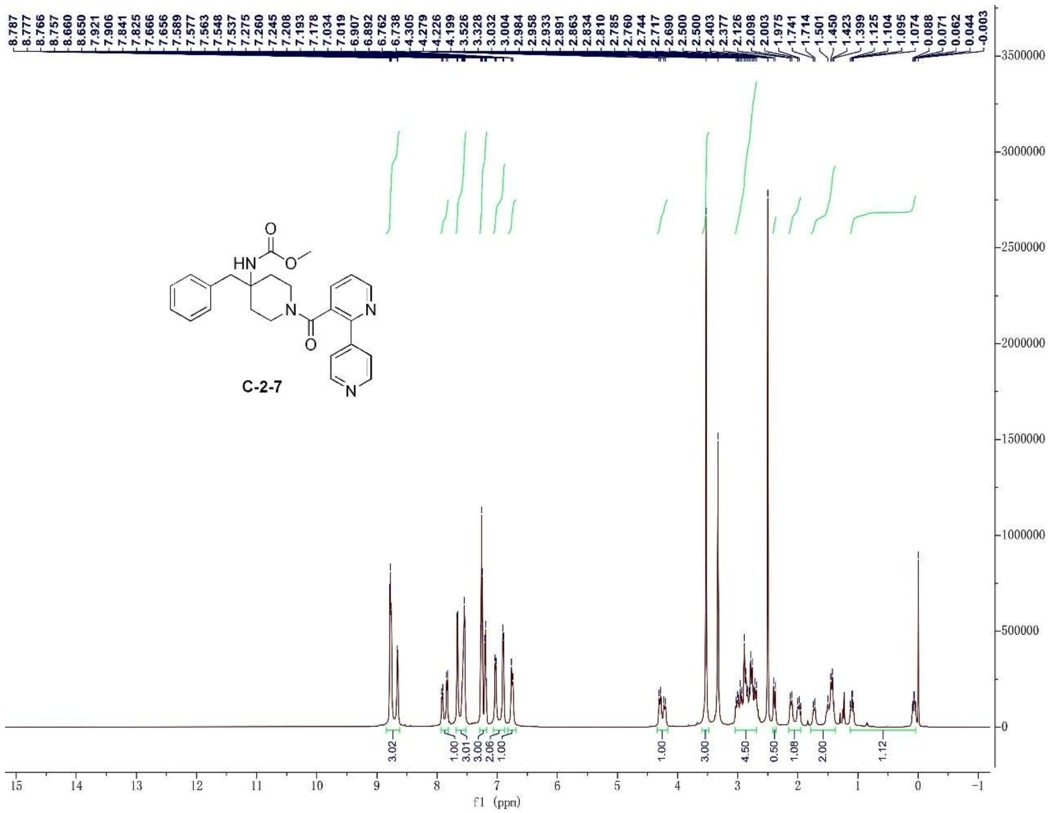

Figure S214: ^{1}H NMR spectrum of **C-2-7**

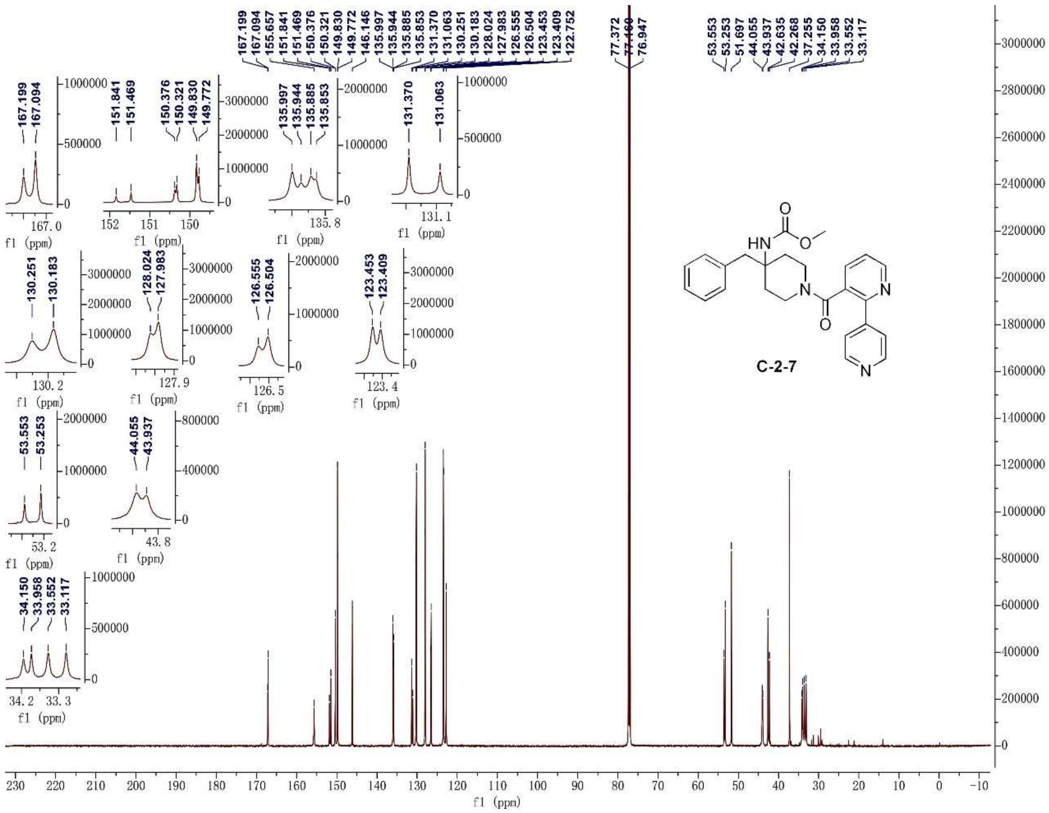

Figure S215: ^{13}C NMR spectrum of **C-2-7**

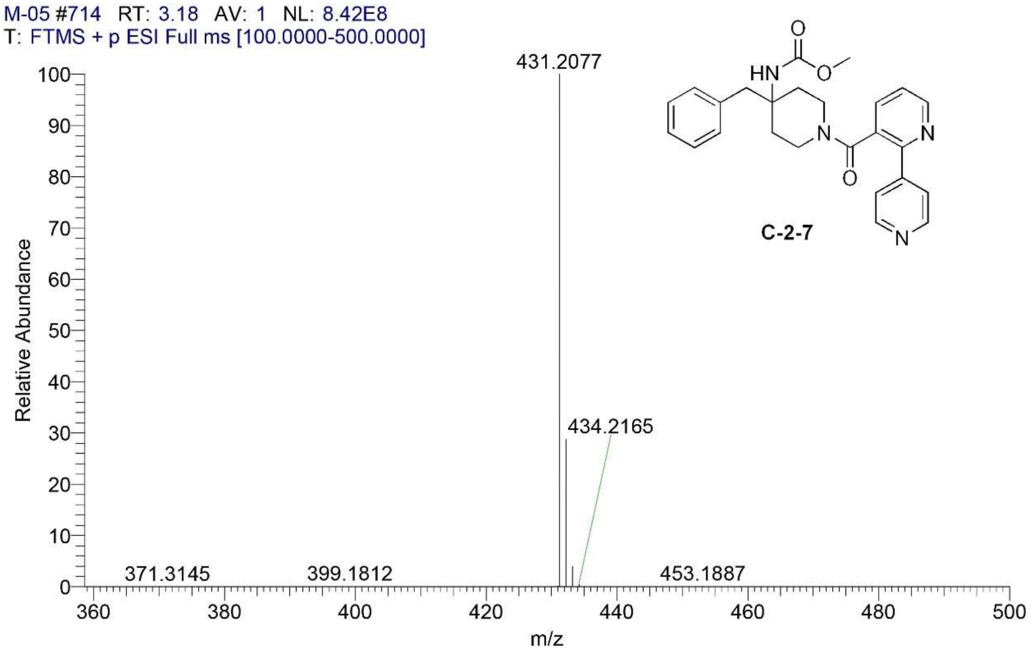

Figure S216: HR-MS (ESI/ion trap) spectrum of **C-2-7**

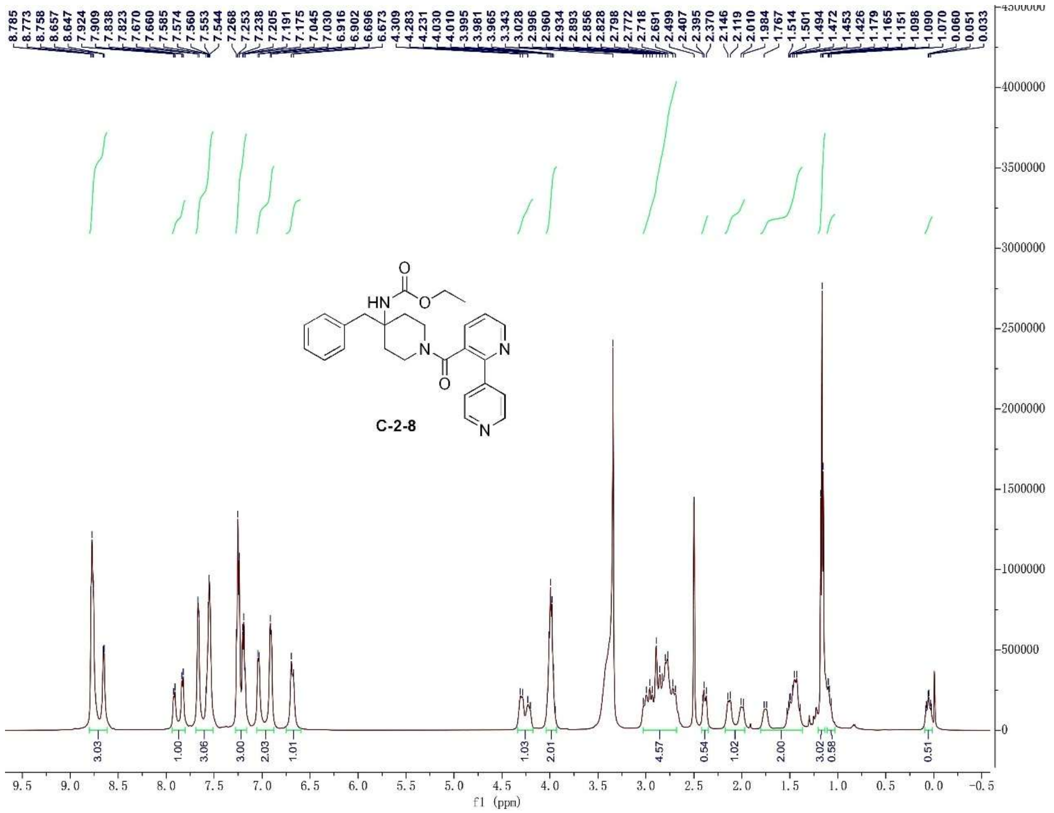

Figure S217: ^{1}H NMR spectrum of **C-2-8**

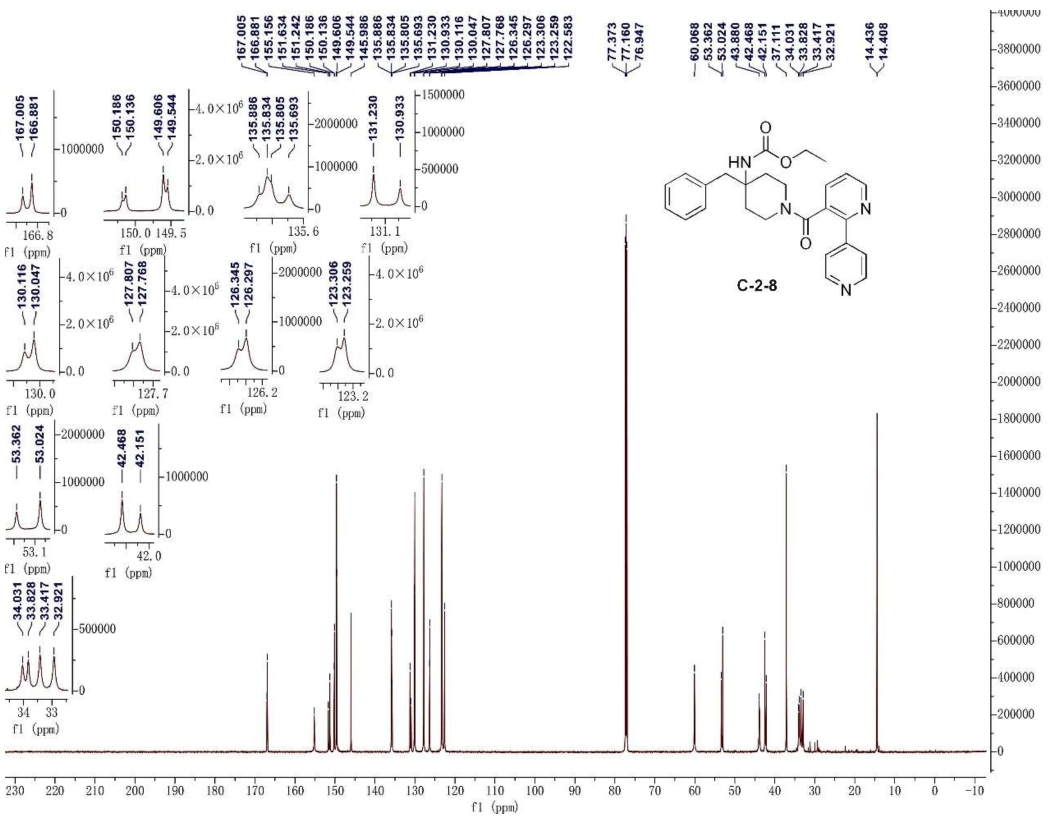

Figure S218: ^{13}C NMR spectrum of **C-2-8**

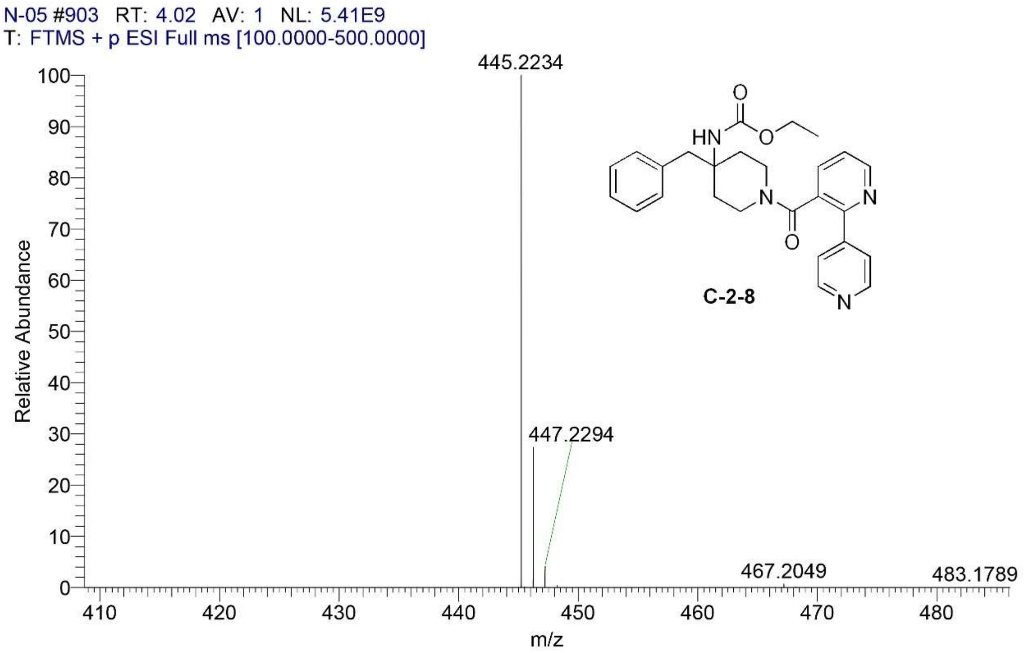

Figure S219: HR-MS (ESI/ion trap) spectrum of **C-2-8**

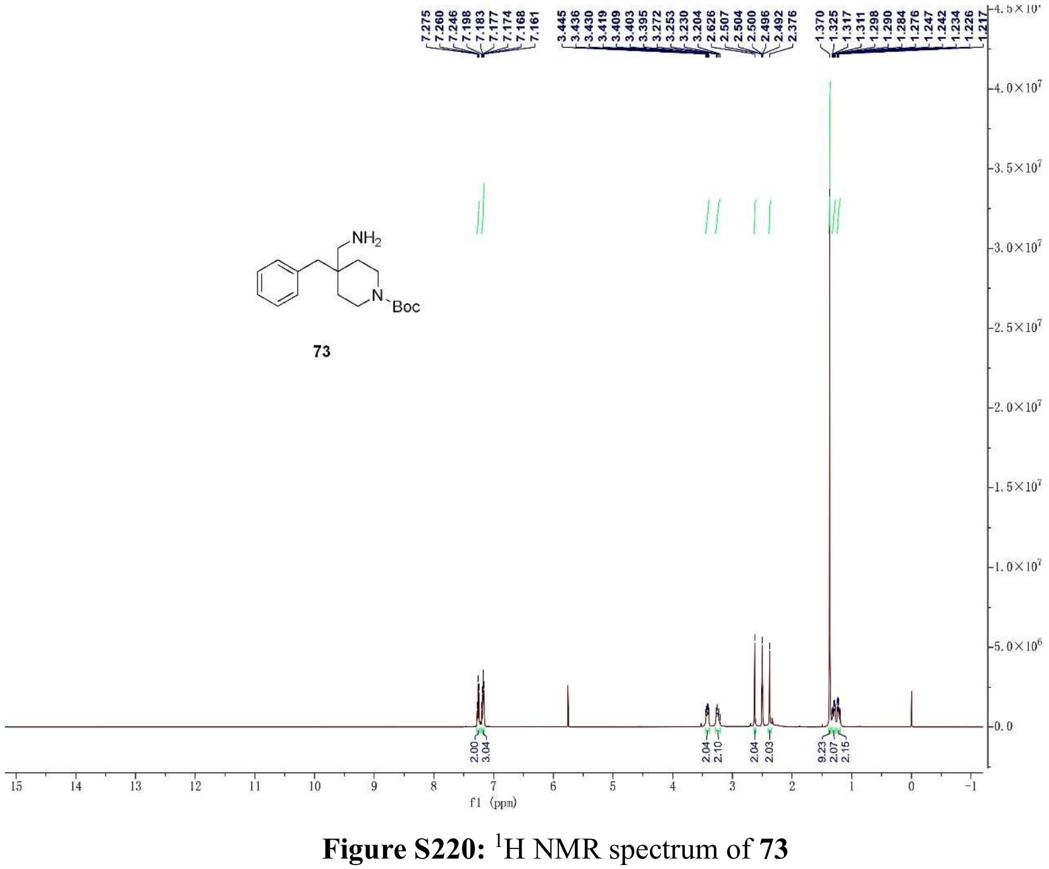

Figure S220: ^{1}H NMR spectrum of **73**

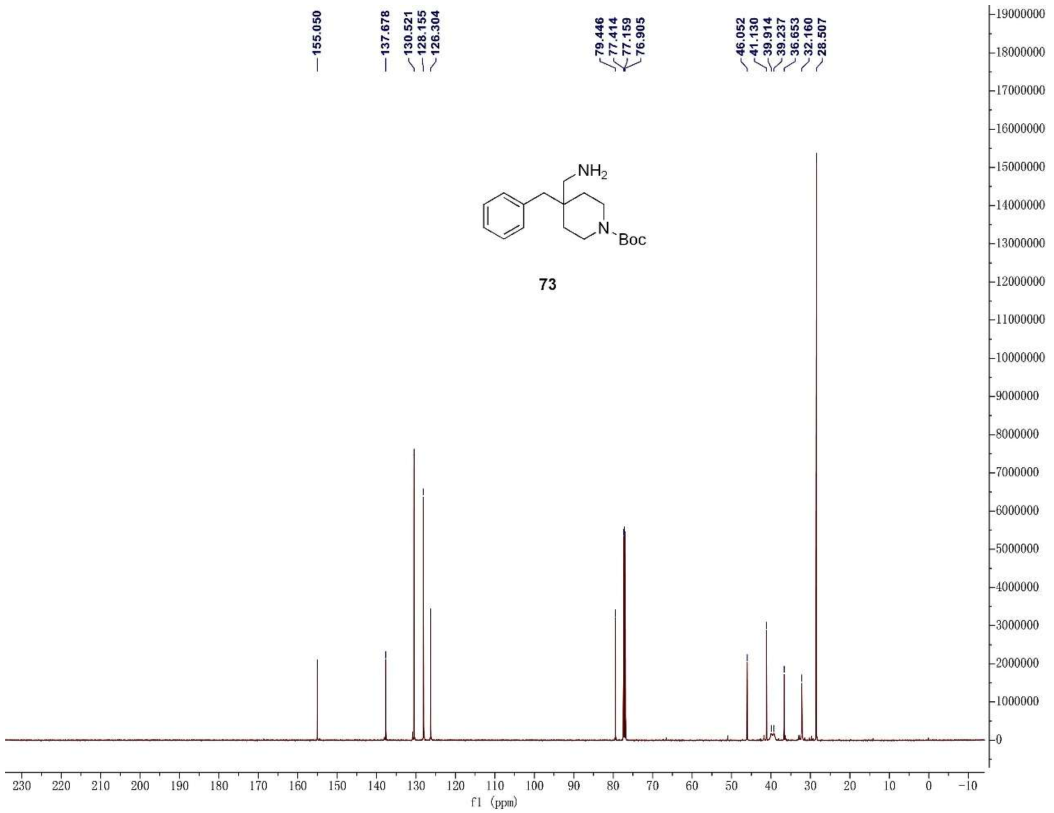

Figure S221: ^{13}C NMR spectrum of **73**

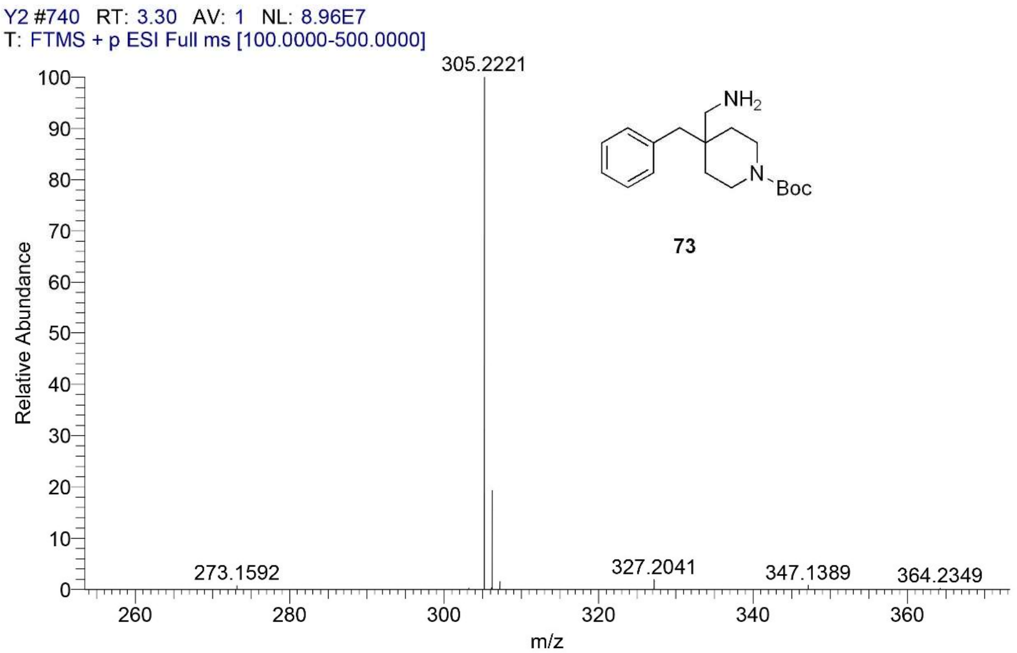

Figure S222: HR-MS (ESI/ion trap) spectrum of **73**

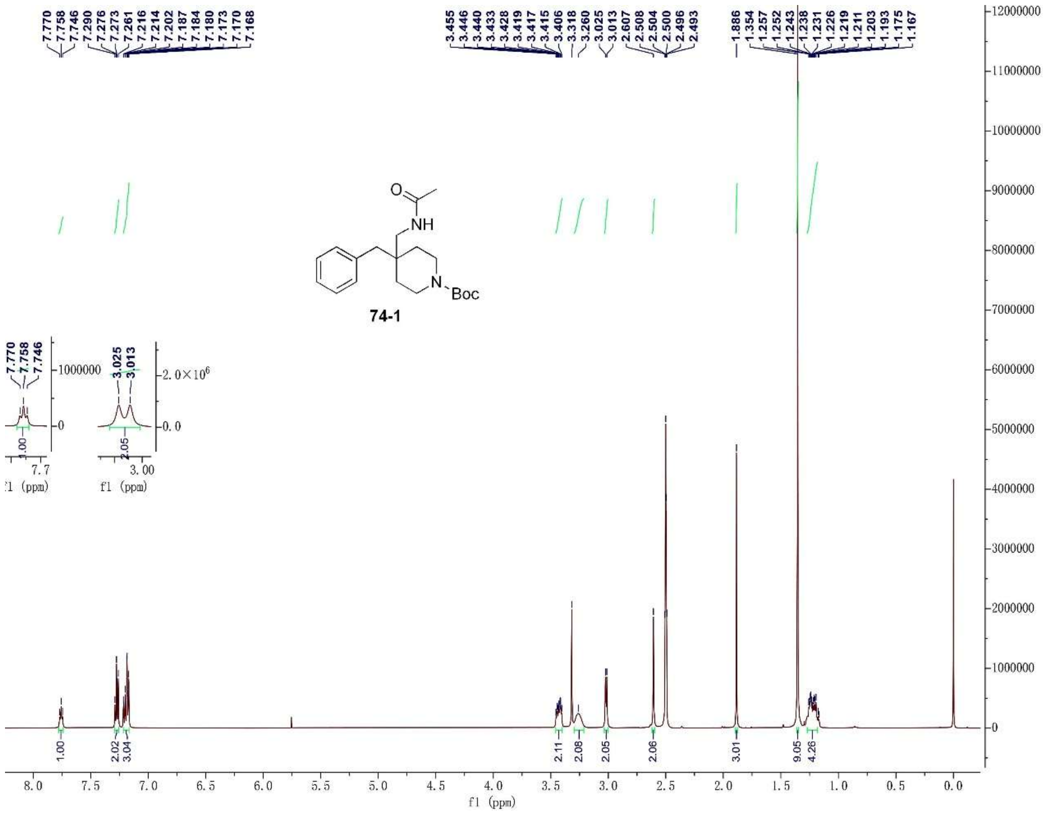

Figure S223: ^{1}H NMR spectrum of **74-1**

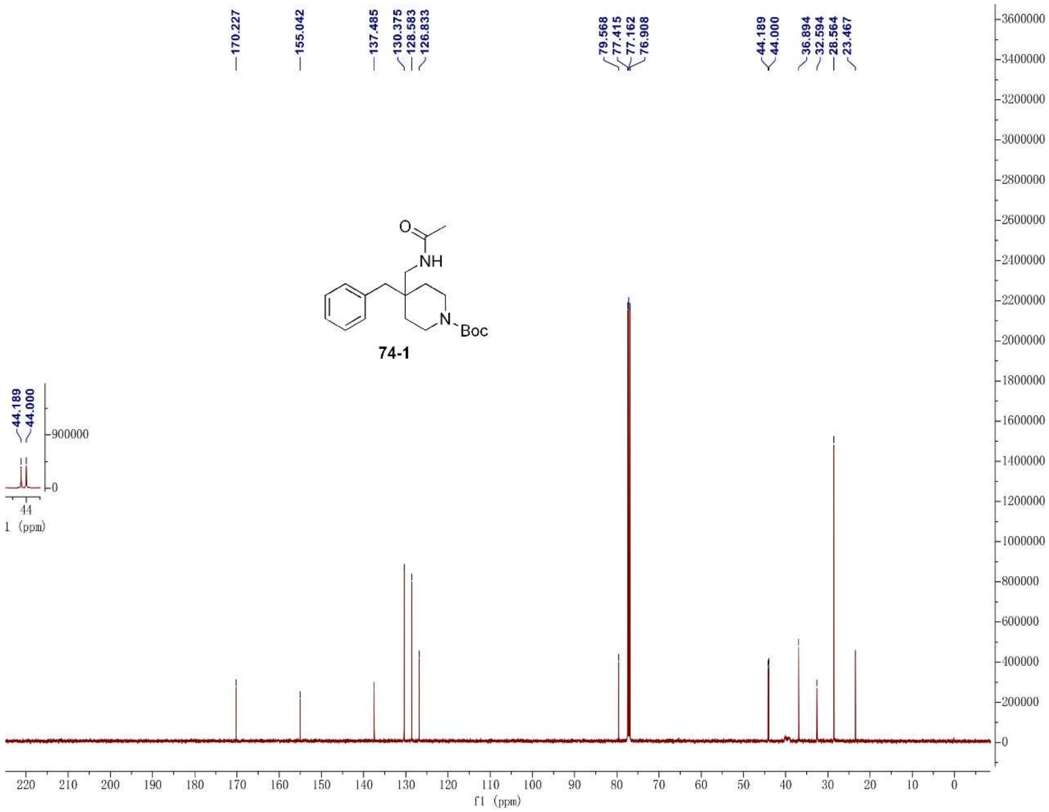

Figure S224: ^{13}C NMR spectrum of **74-1**

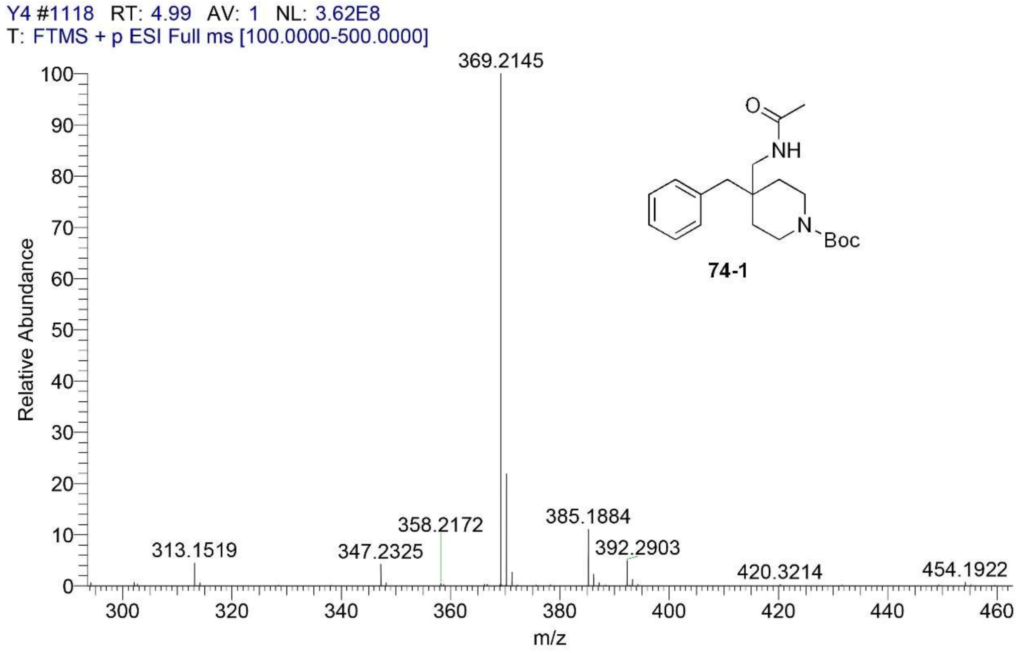

Figure S225: HR-MS (ESI/ion trap) spectrum of **74-1**

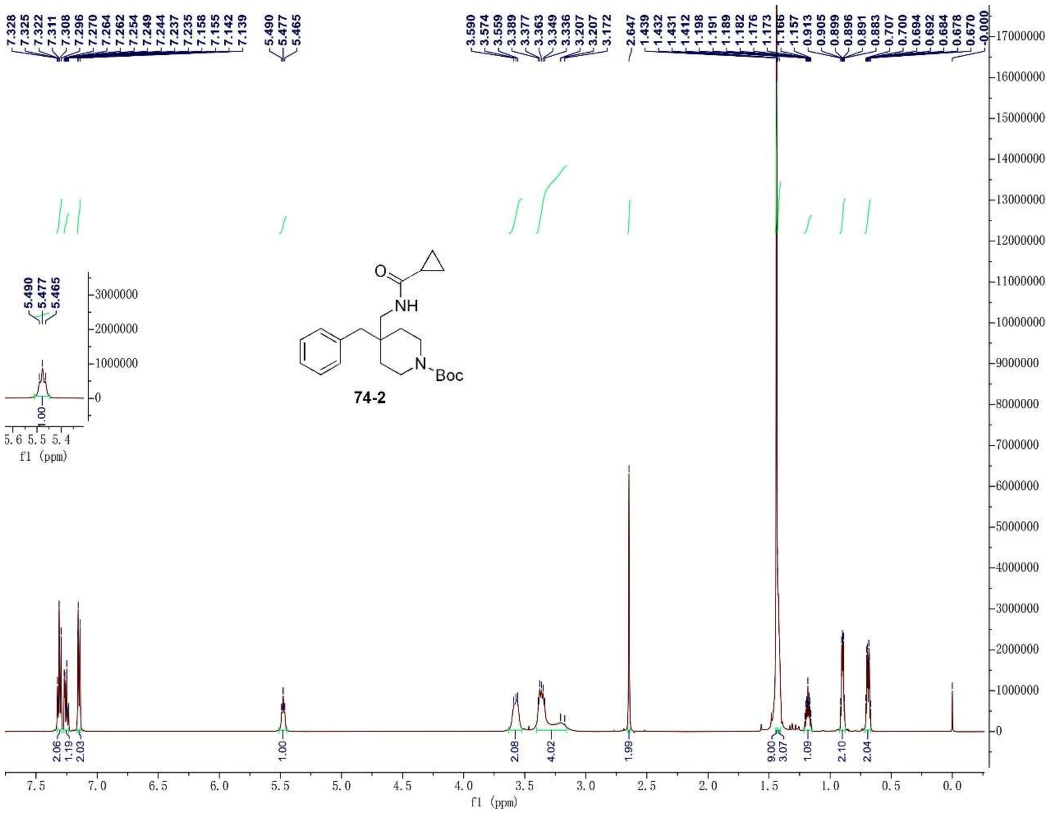

Figure S226: ^{1}H NMR spectrum of **74-2**

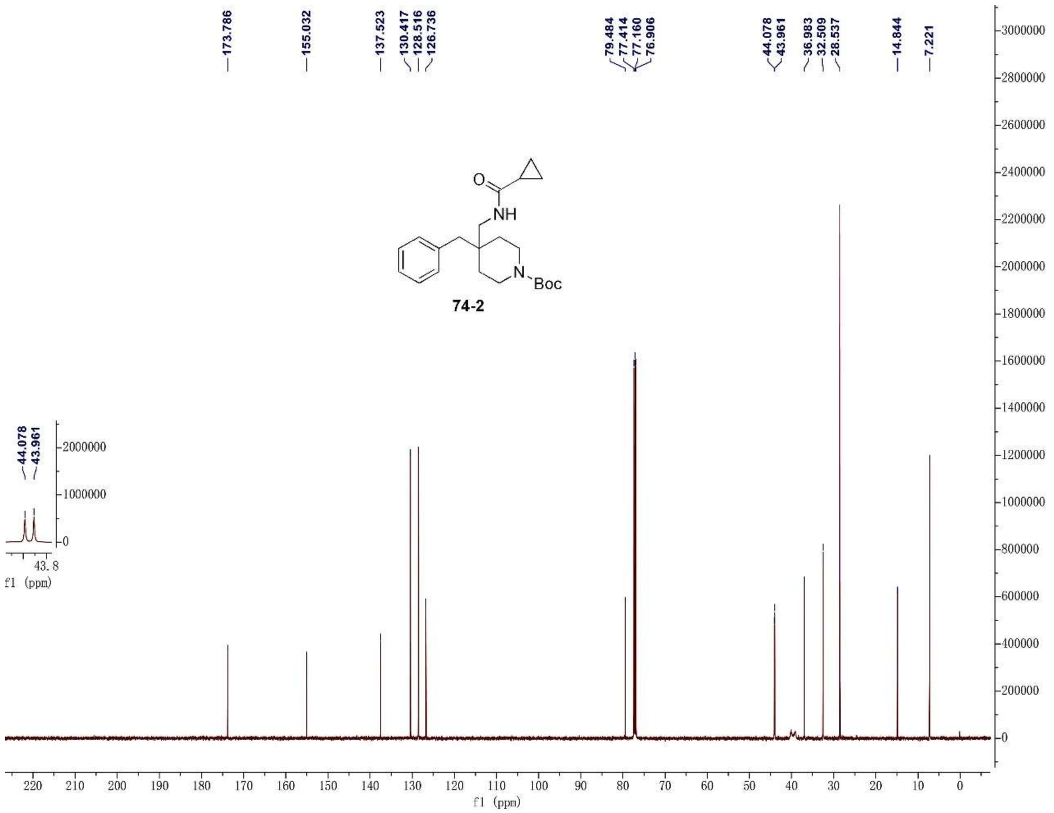

Figure S227: ^{13}C NMR spectrum of **74-2**

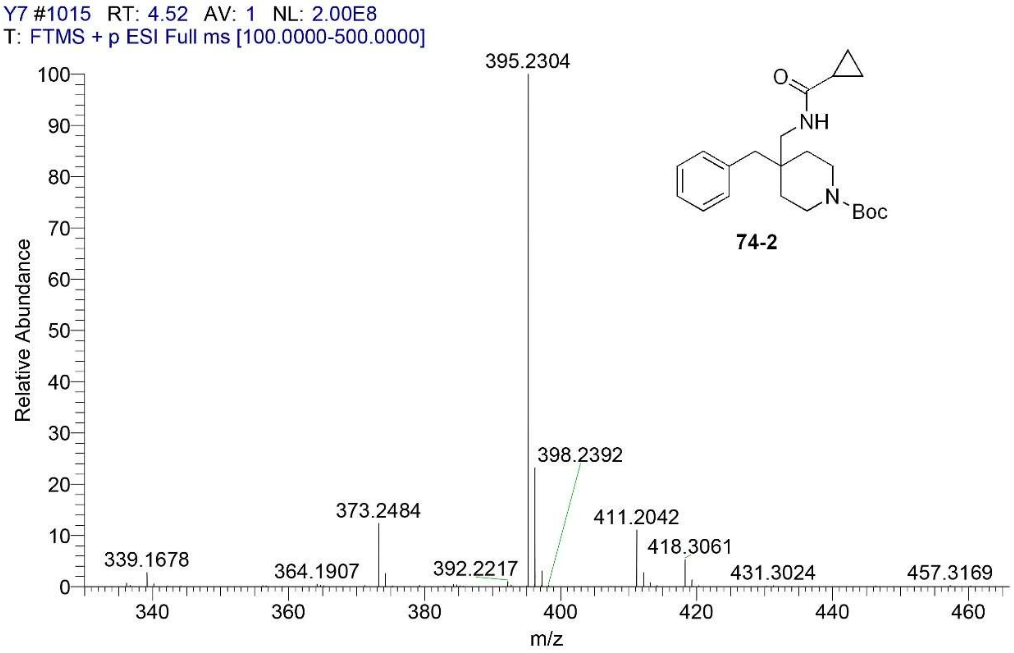

Figure S228: HR-MS (ESI/ion trap) spectrum of **74-2**

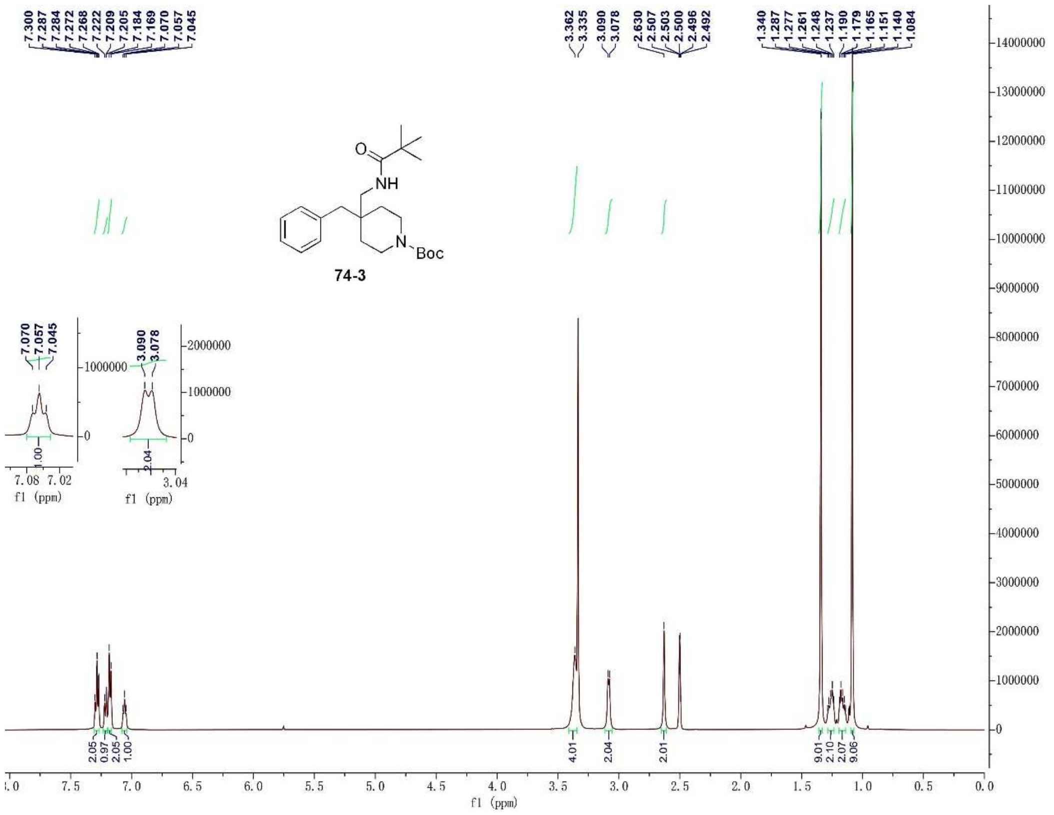

Figure S229: ^{1}H NMR spectrum of **74-3**

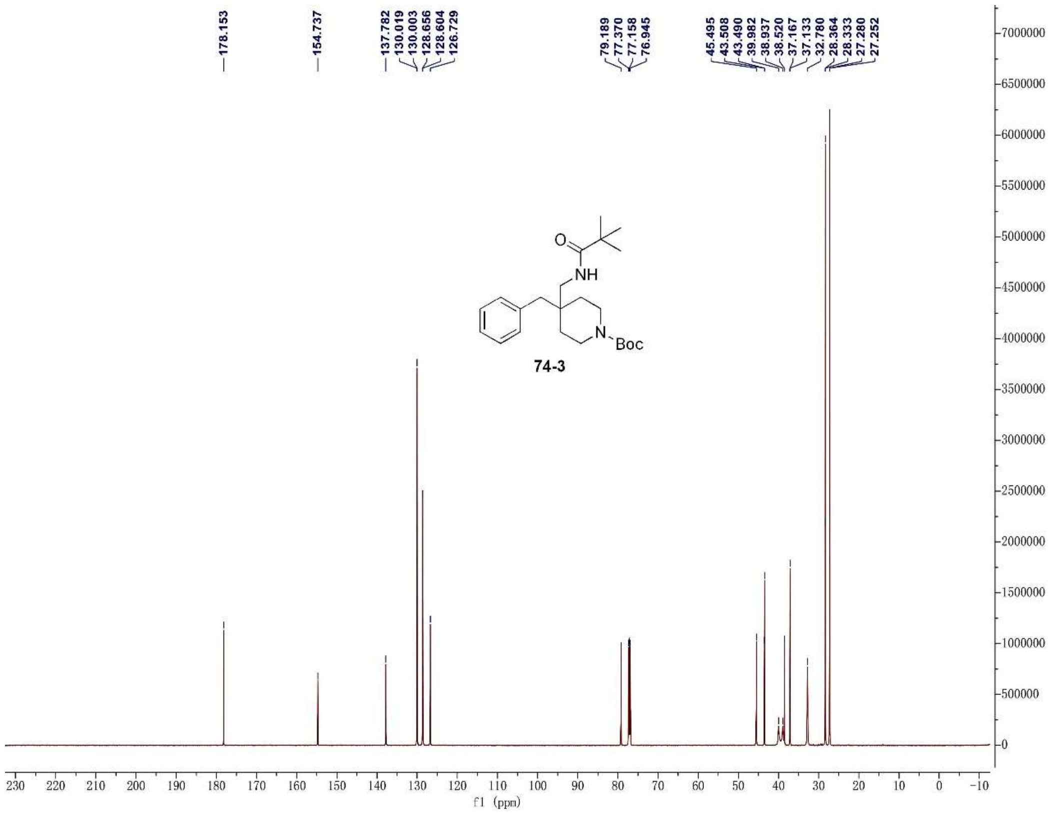

Figure S230: ^{13}C NMR spectrum of **74-3**

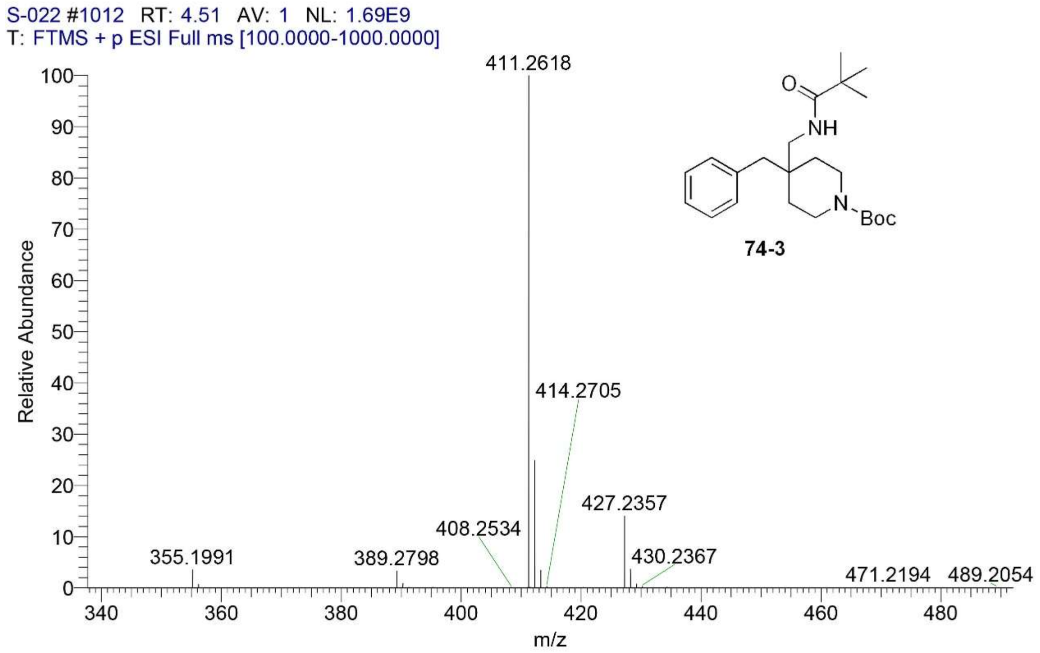

Figure S231: HR-MS (ESI/ion trap) spectrum of **74-3**

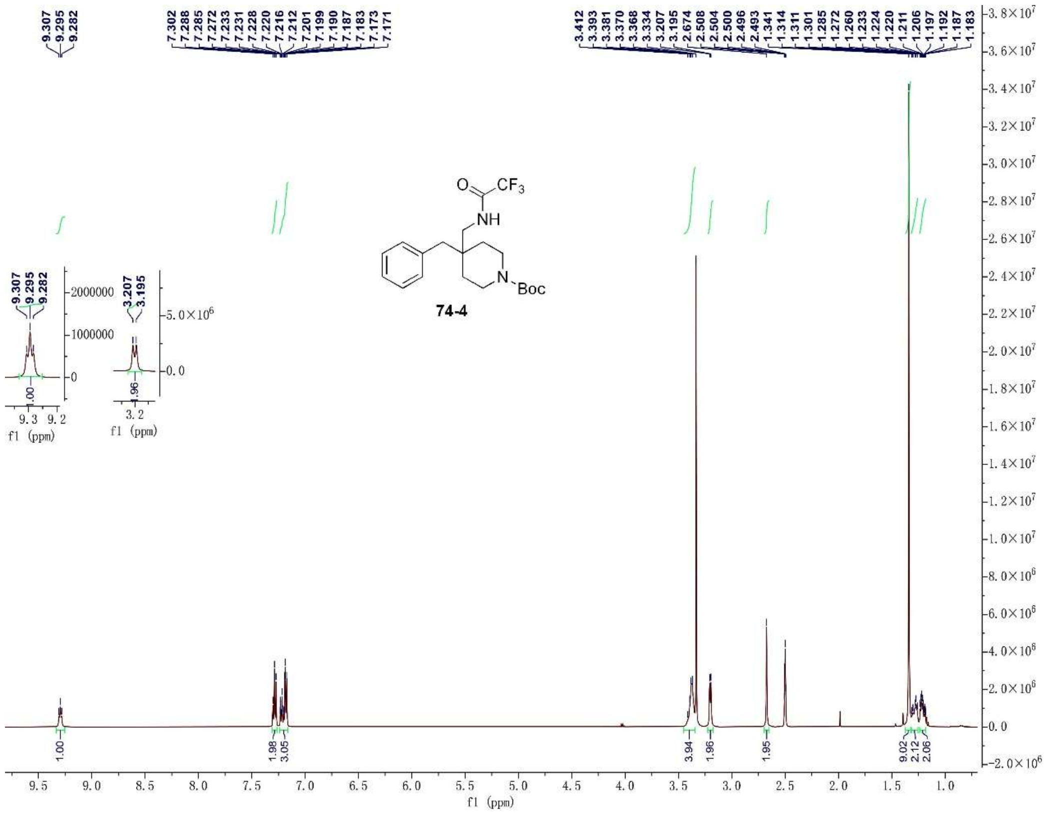

Figure S232: ^{1}H NMR spectrum of **74-4**

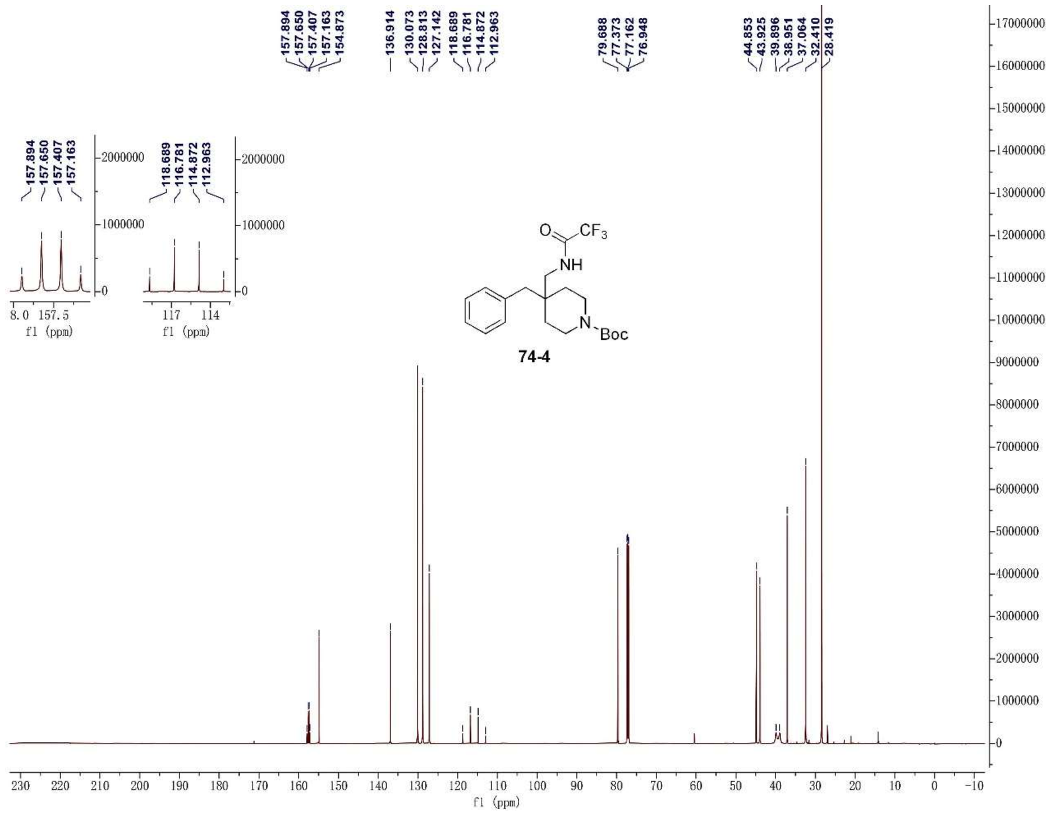

Figure S233: ^{13}C NMR spectrum of **74-4**

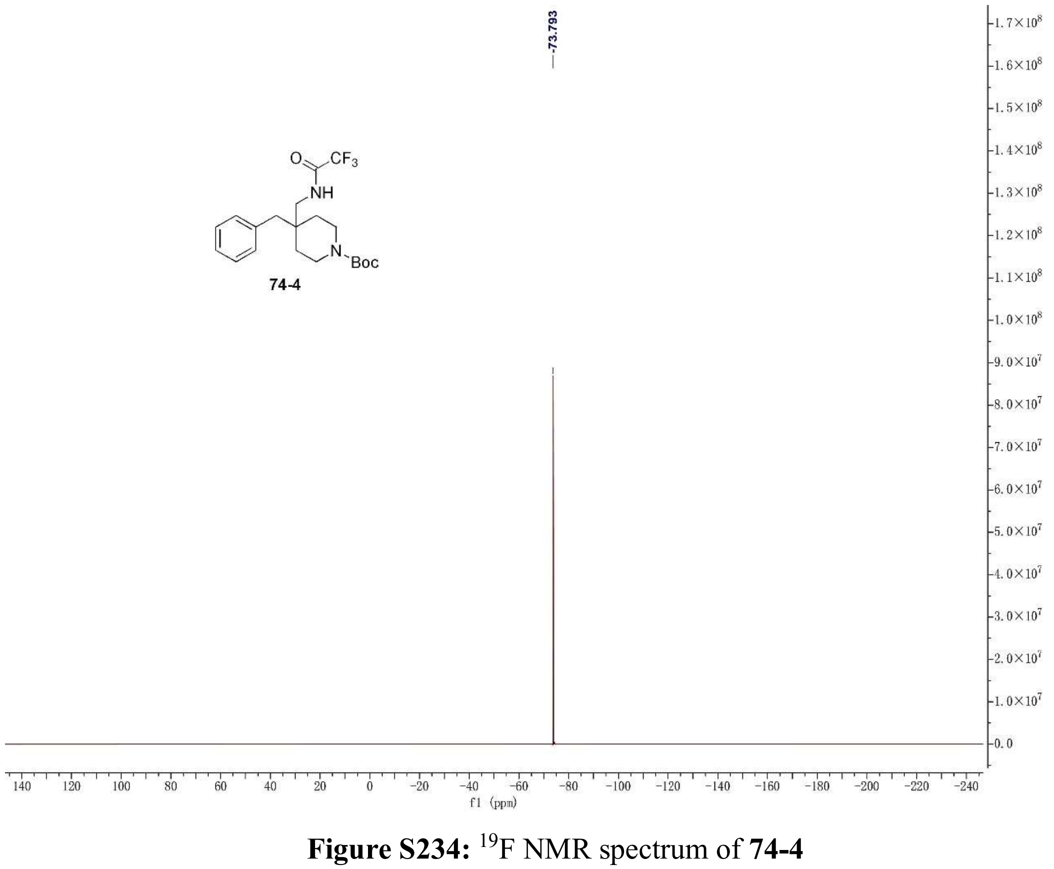

Figure S234: ^{19}F NMR spectrum of **74-4**

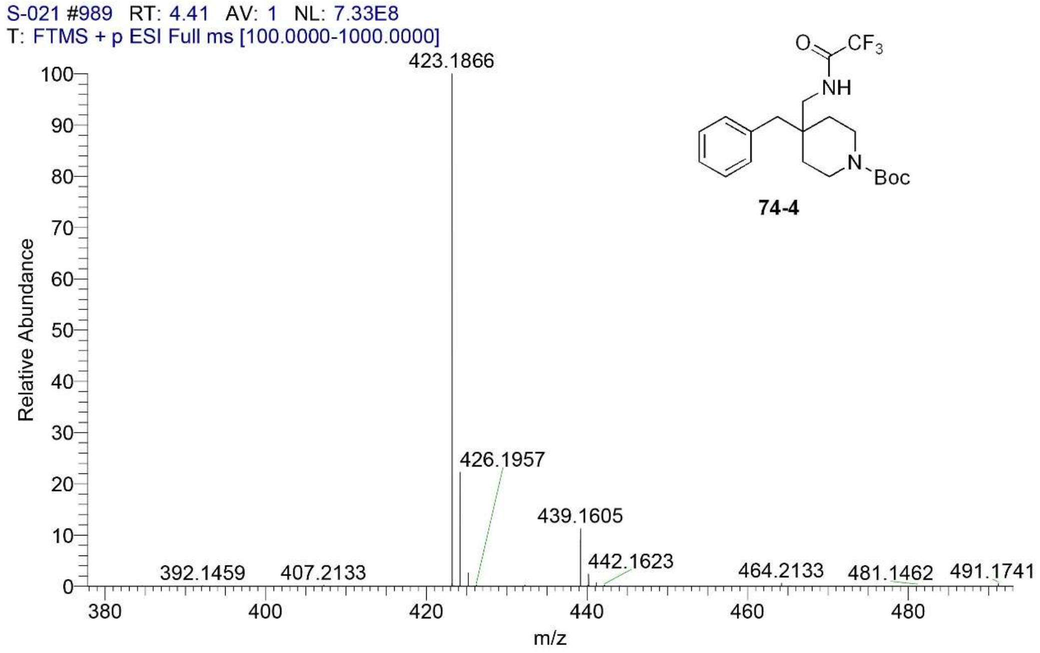

Figure S235: HR-MS (ESI/ion trap) spectrum of **74-4**

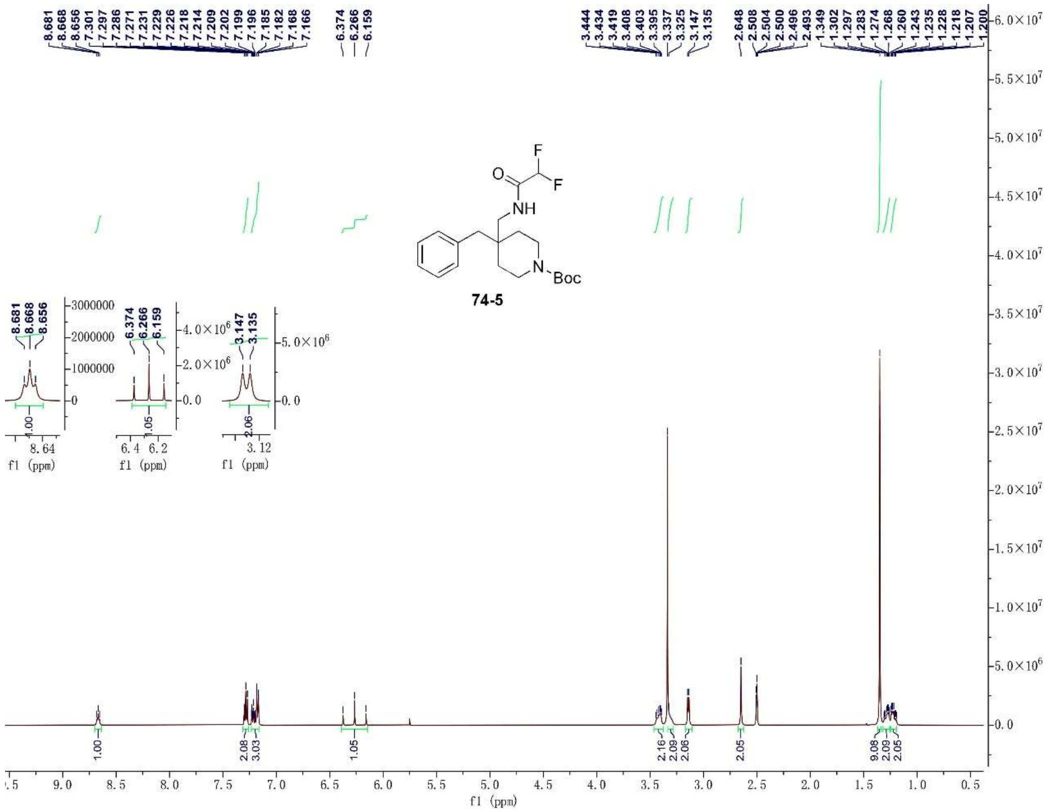

Figure S236: ^{1}H NMR spectrum of **74-5**

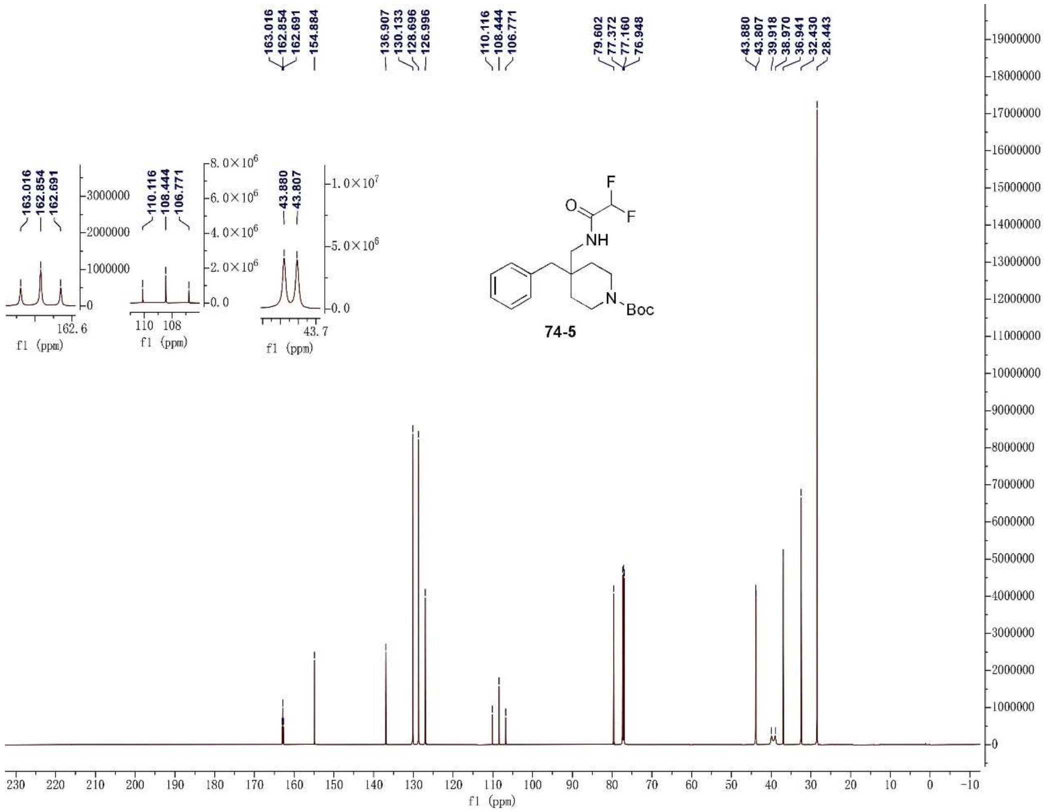

Figure S237: ^{13}C NMR spectrum of **74-5**

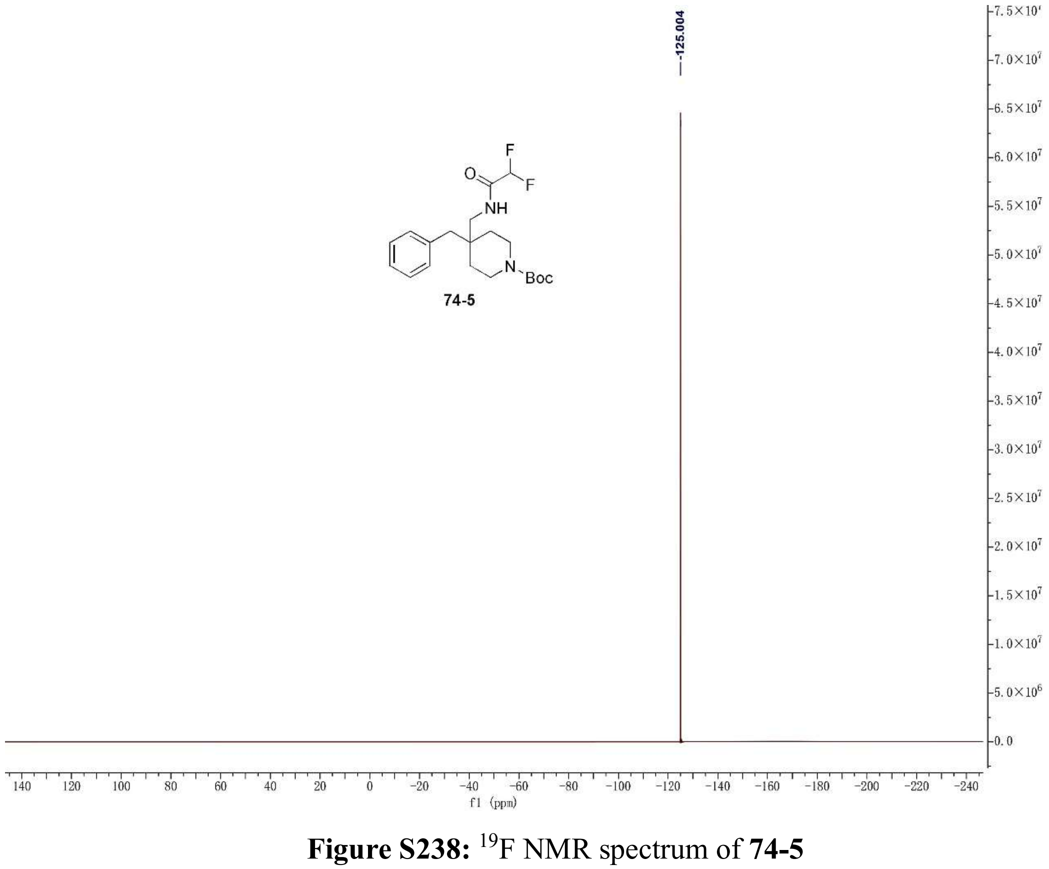

Figure S238: ^{19}F NMR spectrum of **74-5**

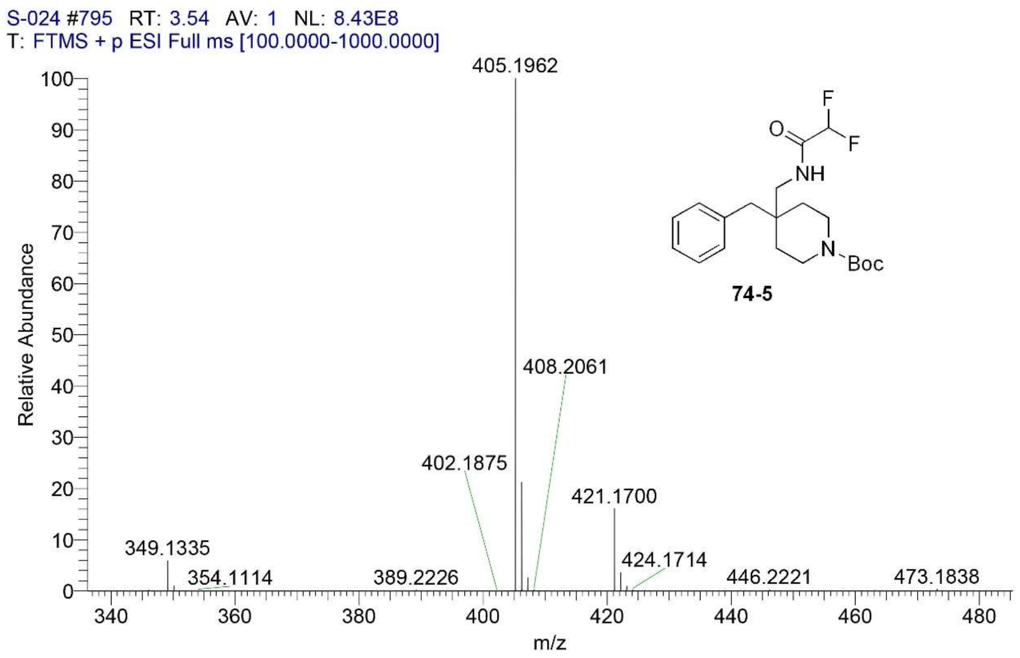

Figure S239: HR-MS (ESI/ion trap) spectrum of **74-5**

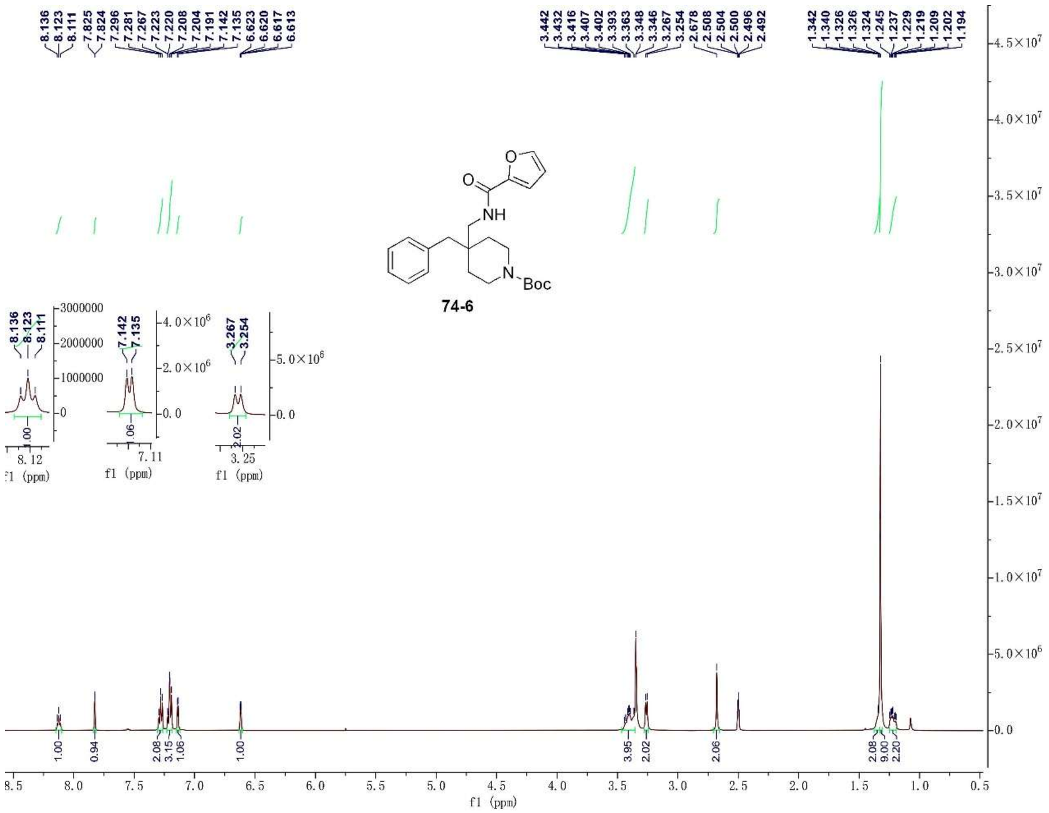

Figure S240: ^{1}H NMR spectrum of **74-6**

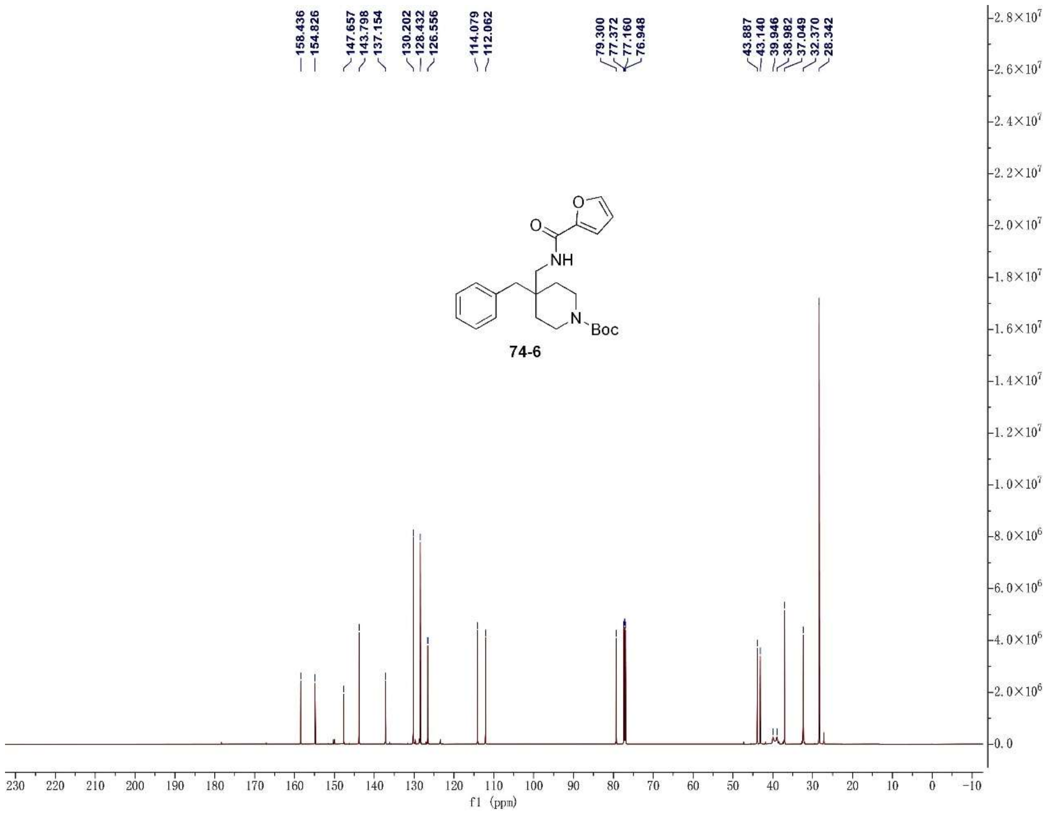

Figure S241: ^{13}C NMR spectrum of **74-6**

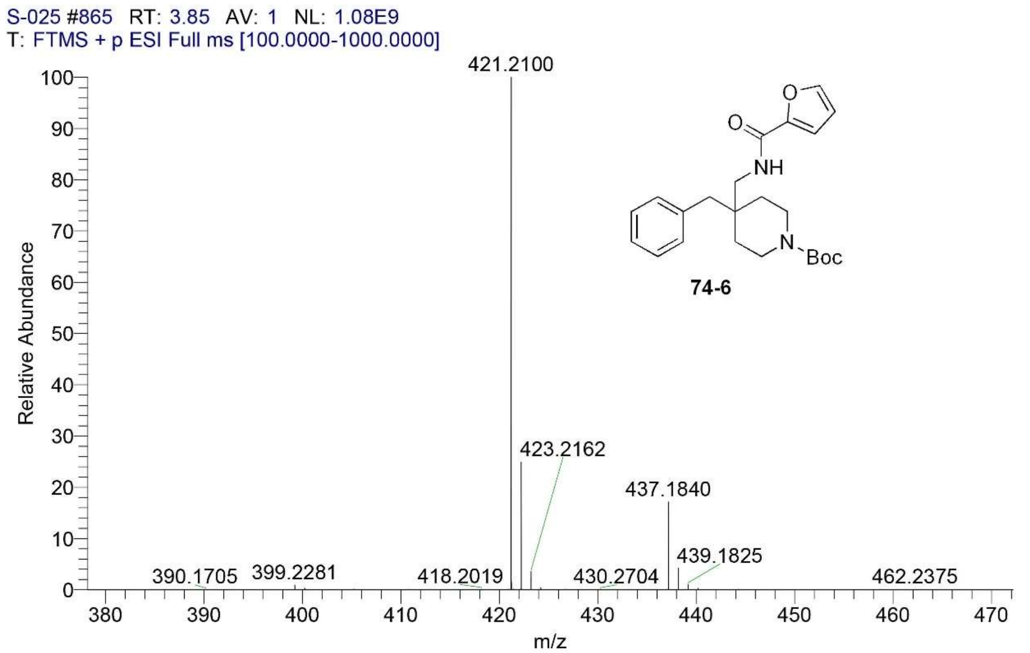

Figure S242: HR-MS (ESI/ion trap) spectrum of **74-6**

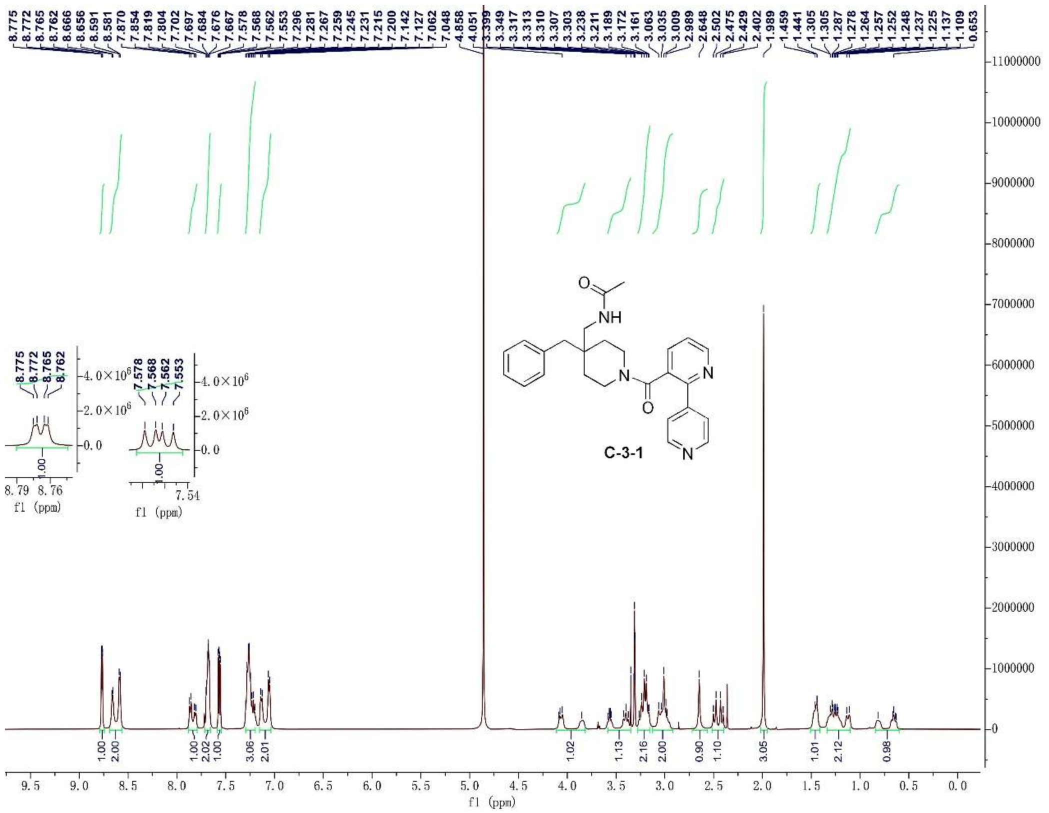

Figure S243: ^{1}H NMR spectrum of **C-3-1**

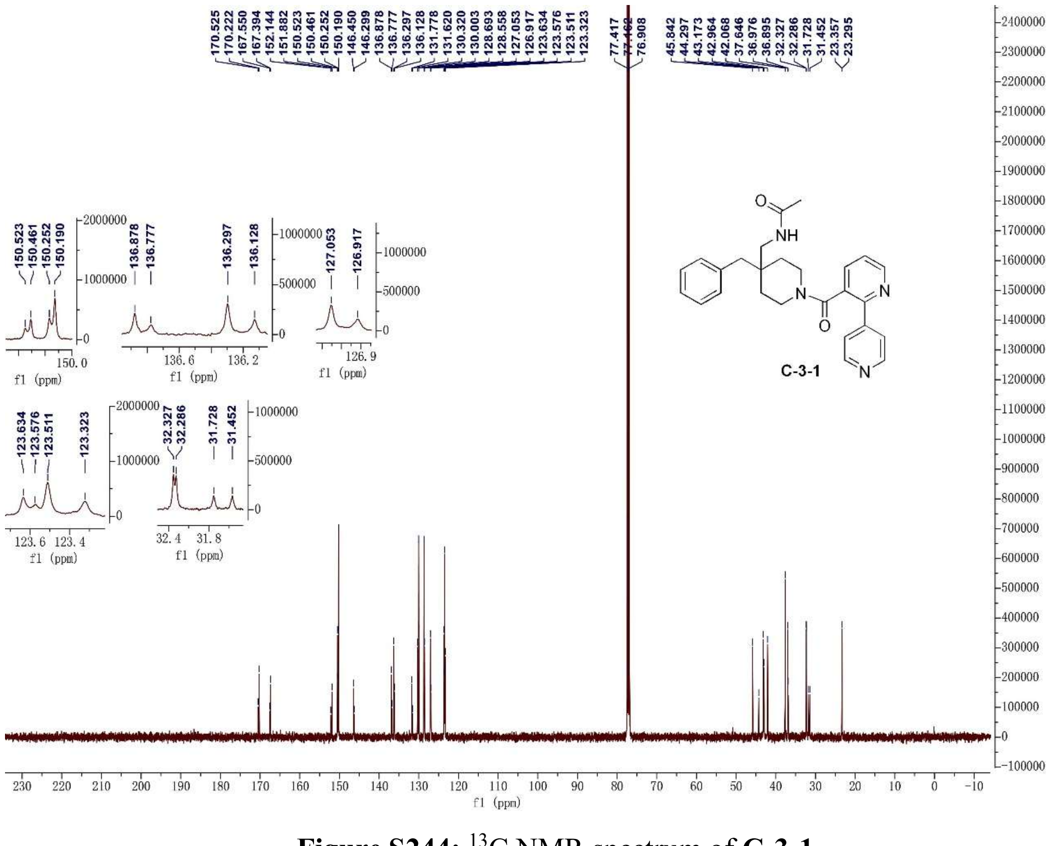

Figure S244: ^{13}C NMR spectrum of **C-3-1**

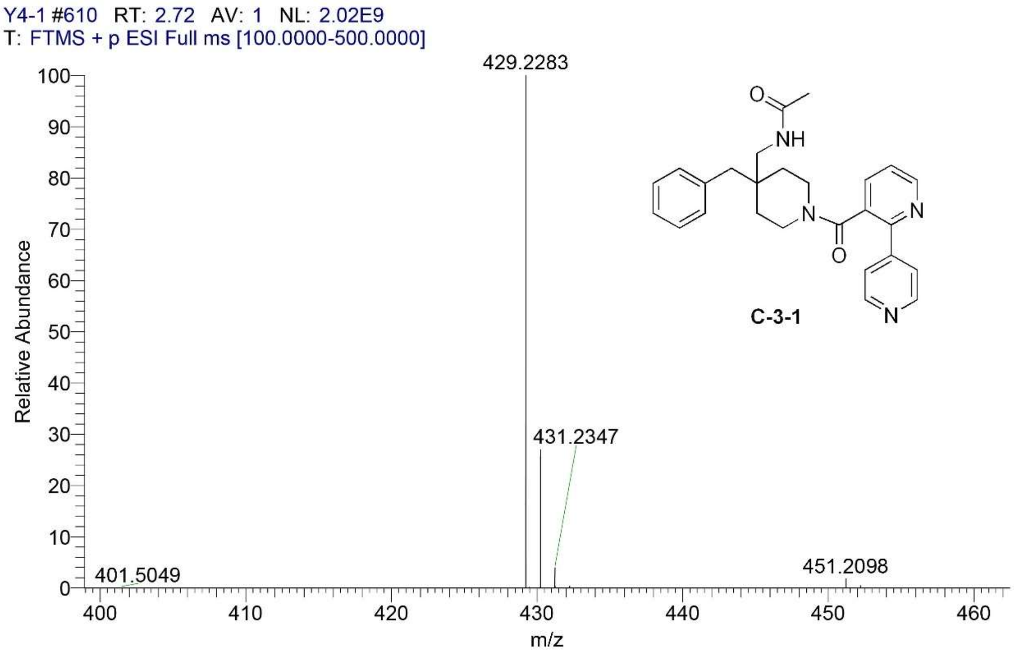

Figure S245: HR-MS (ESI/ion trap) spectrum of **C-3-1**

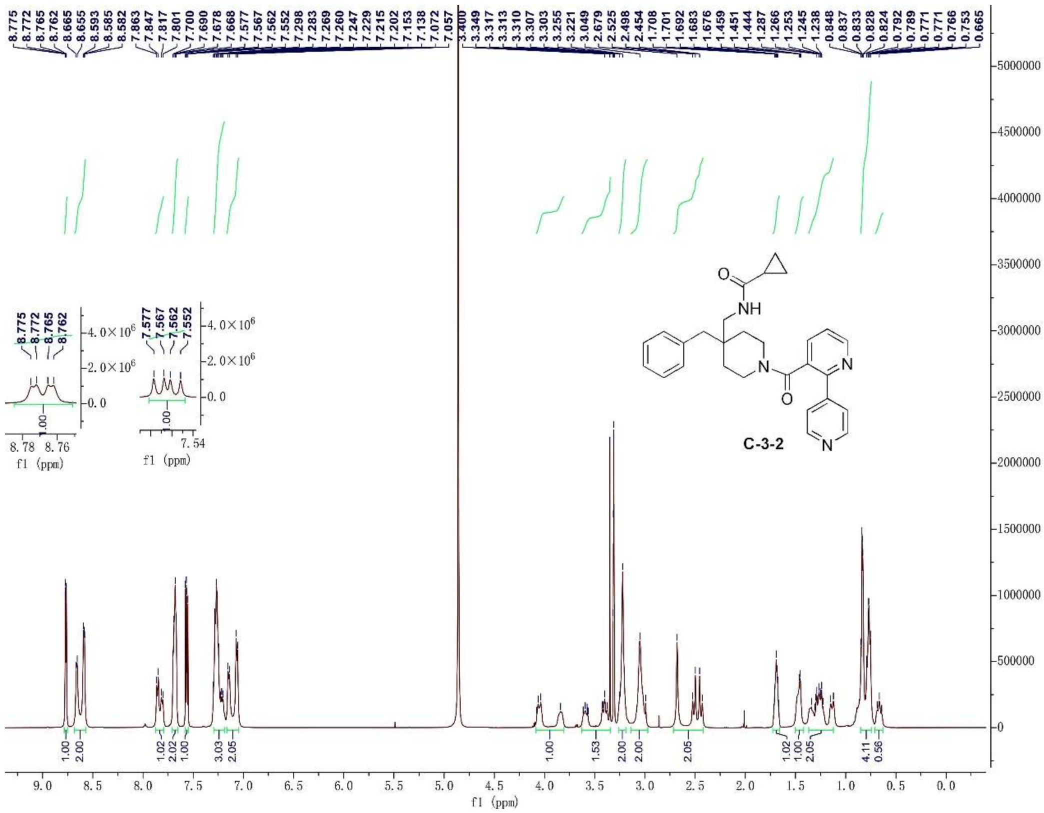

Figure S246: ^{1}H NMR spectrum of **C-3-2**

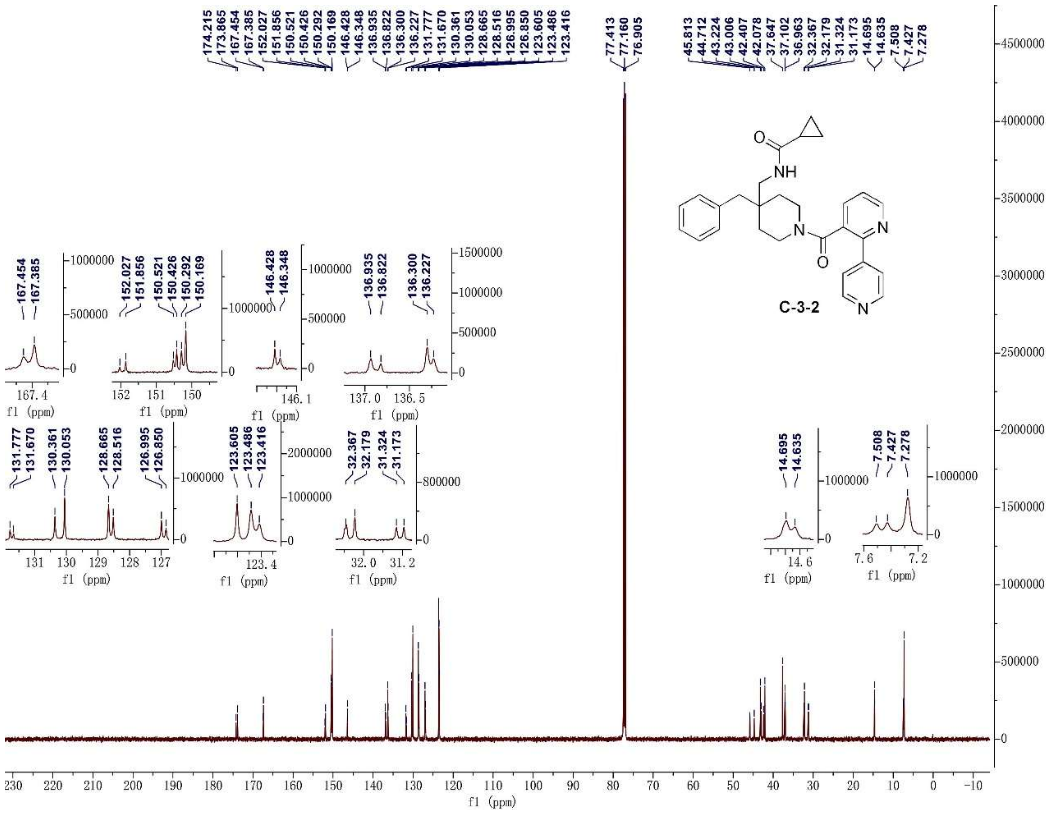

Figure S247: ^{13}C NMR spectrum of **C-3-2**

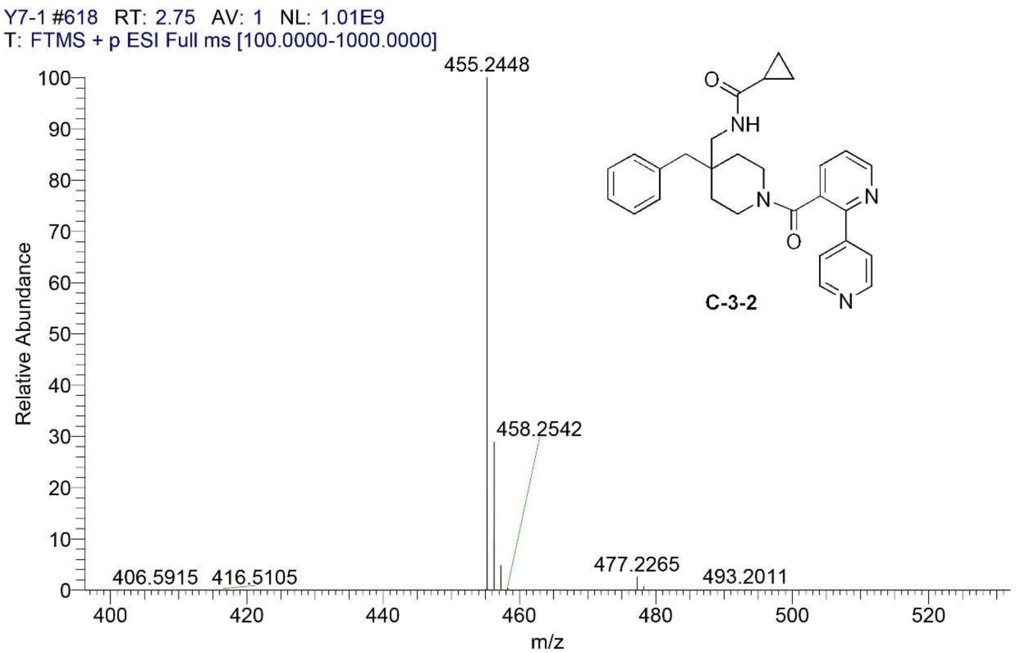

Figure S248: HR-MS (ESI/ion trap) spectrum of **C-3-2**

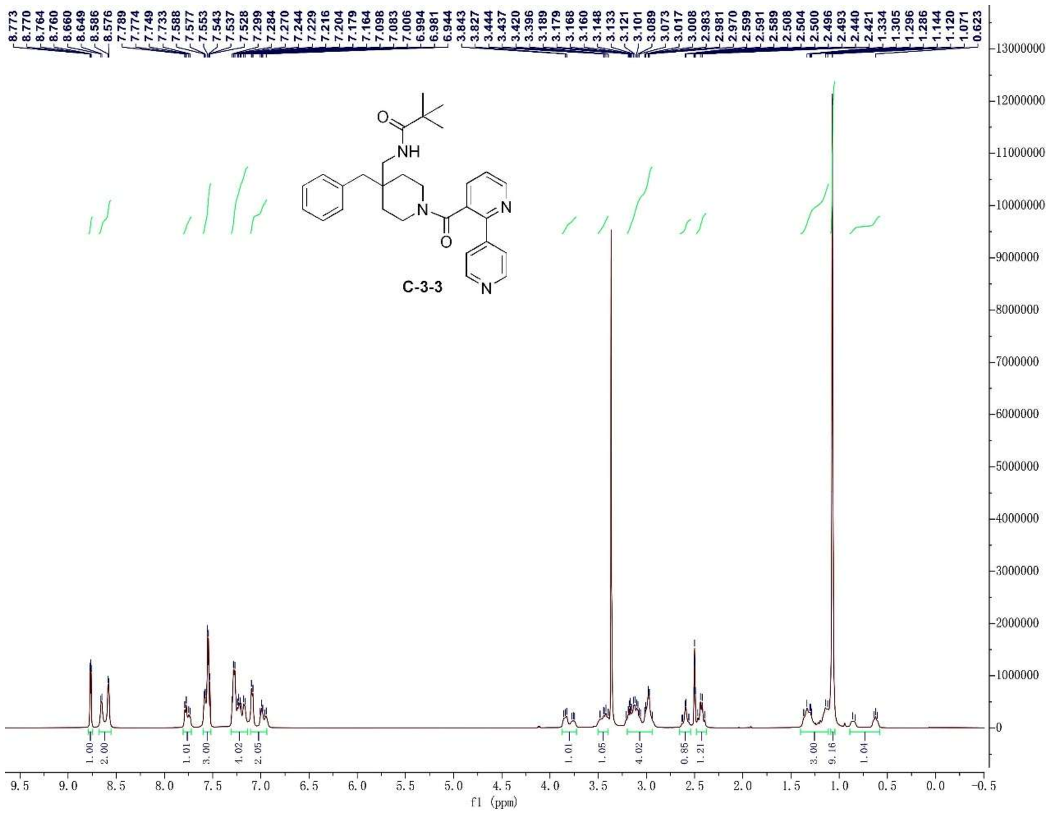

Figure S249: ^{1}H NMR spectrum of **C-3-3**

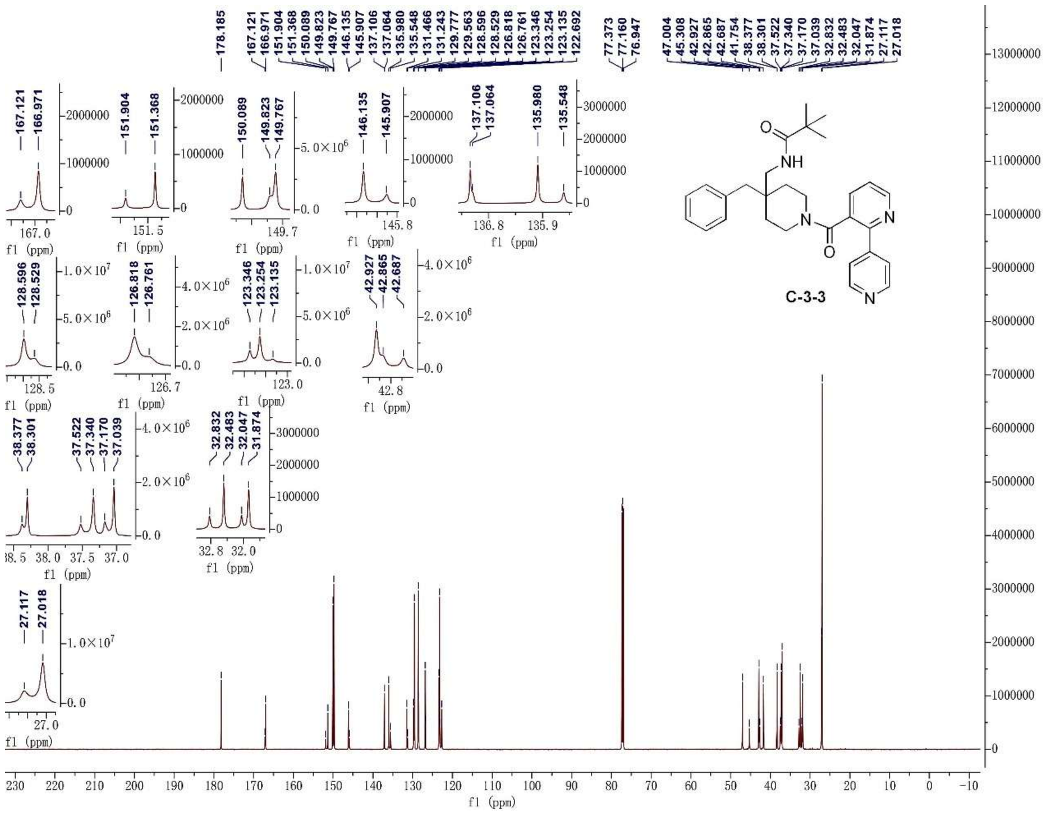

Figure S250: ^{13}C NMR spectrum of **C-3-3**

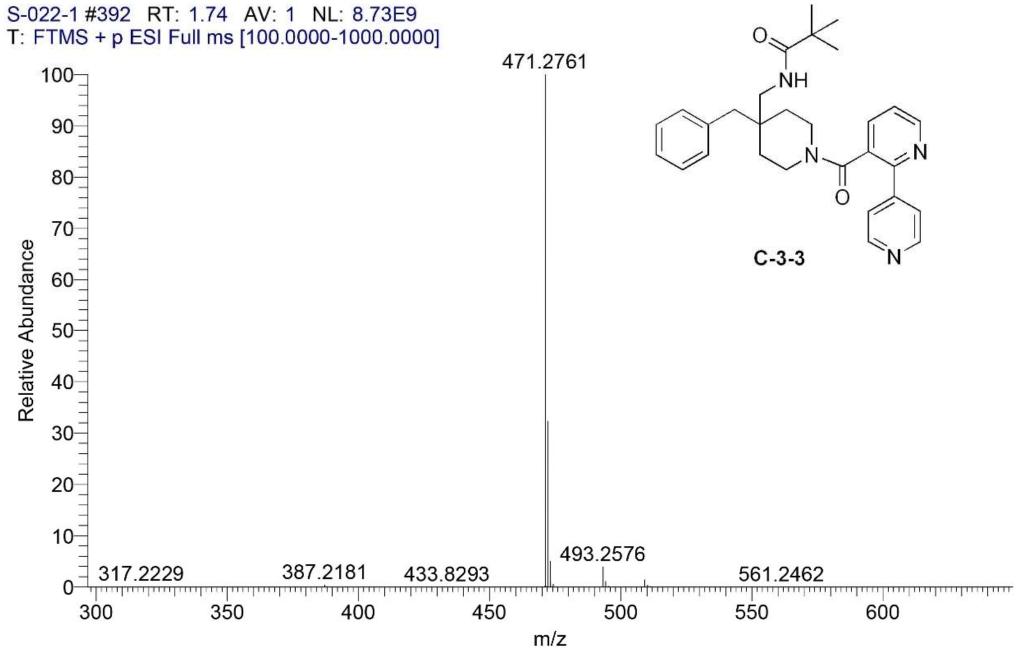

Figure S251: HR-MS (ESI/ion trap) spectrum of **C-3-3**

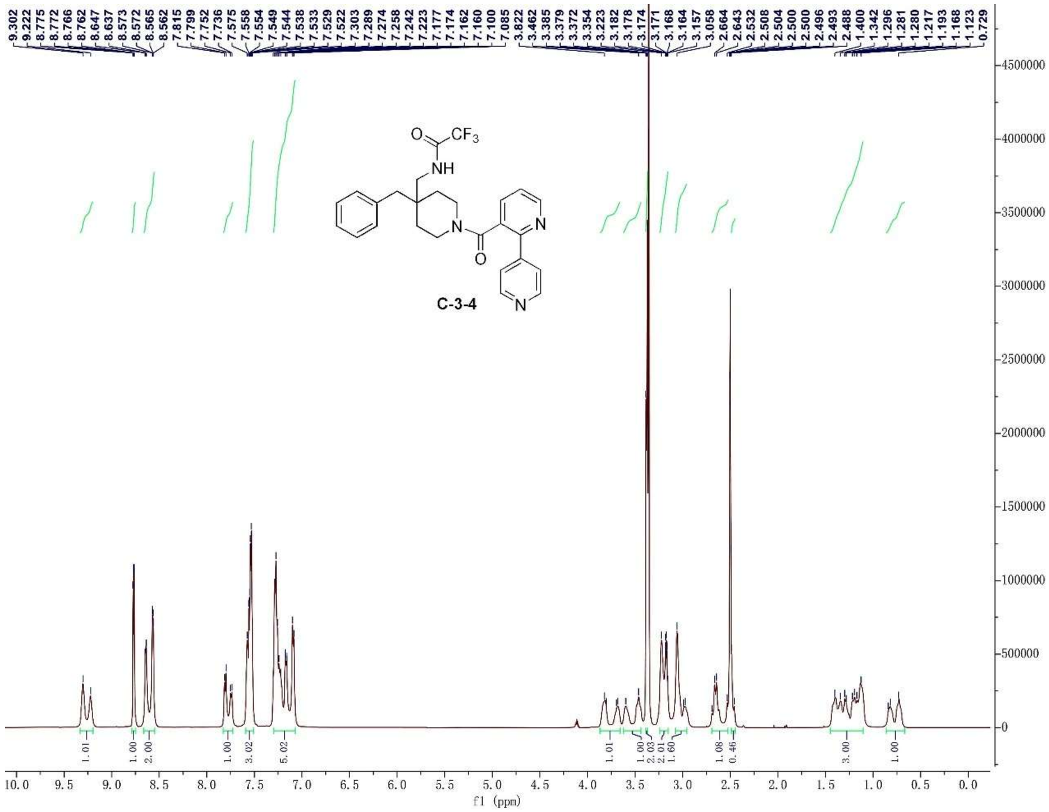

Figure S252: ^{1}H NMR spectrum of **C-3-4**

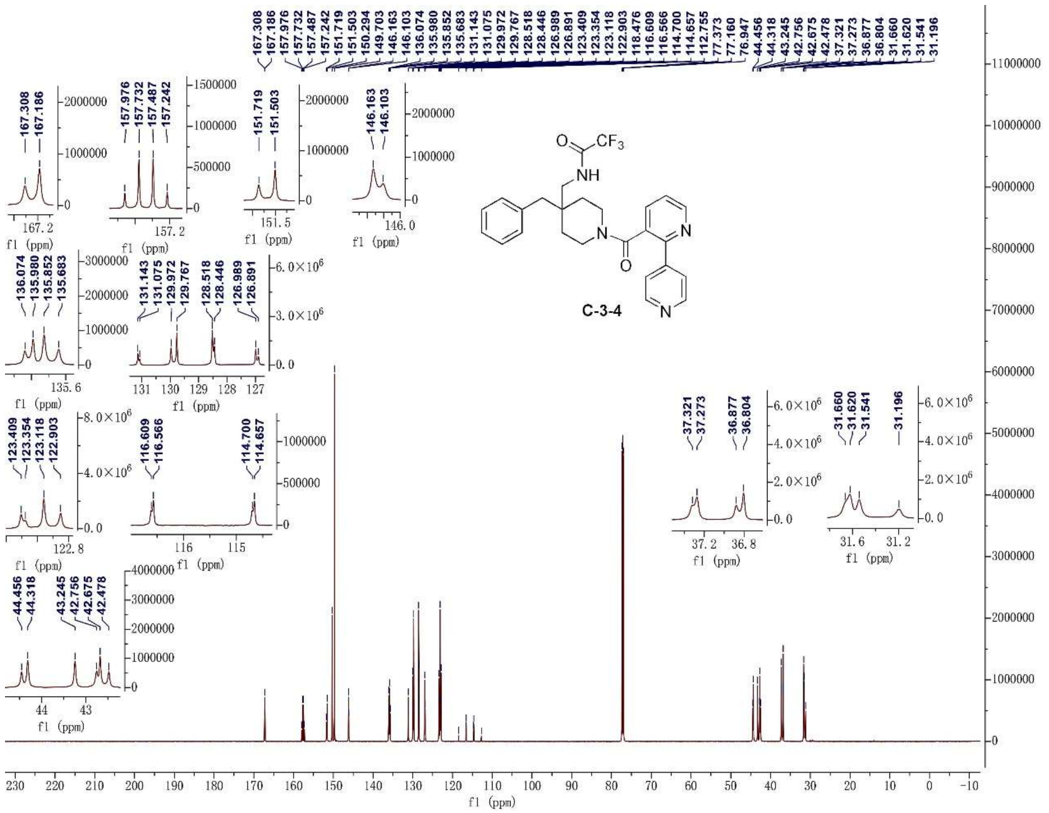

Figure S253: ^{13}C NMR spectrum of **C-3-4**

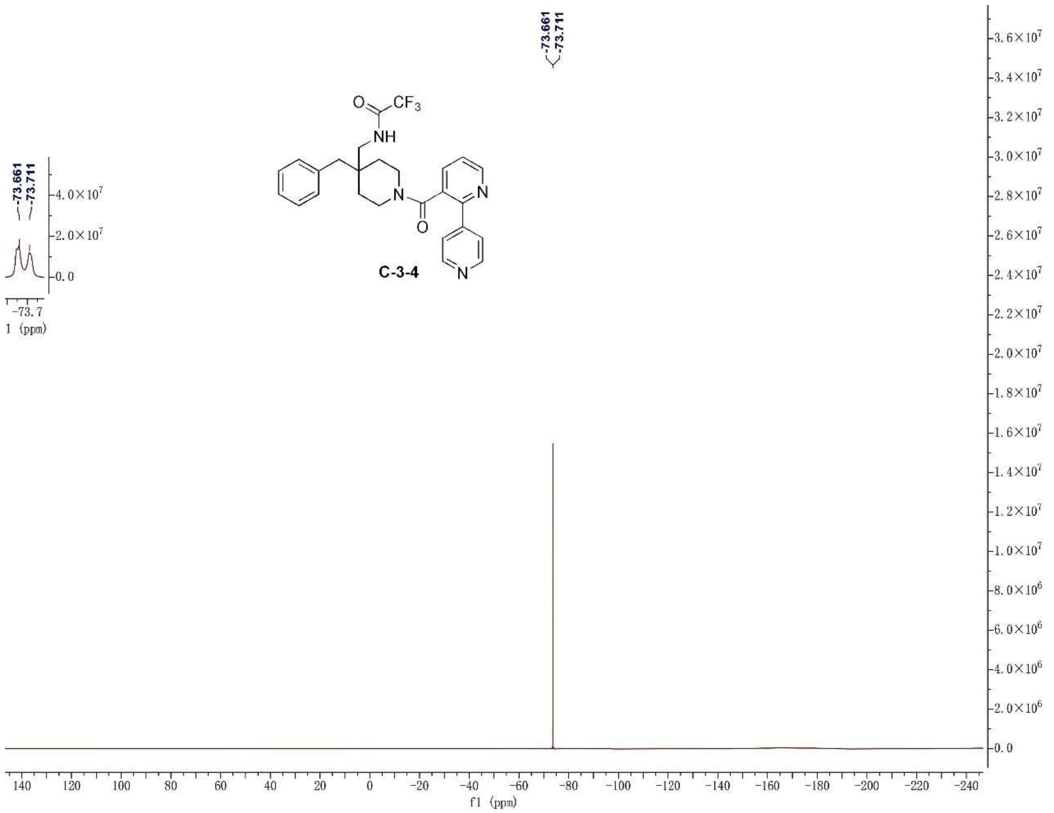

Figure S254: ^{19}F NMR spectrum of **C-3-4**

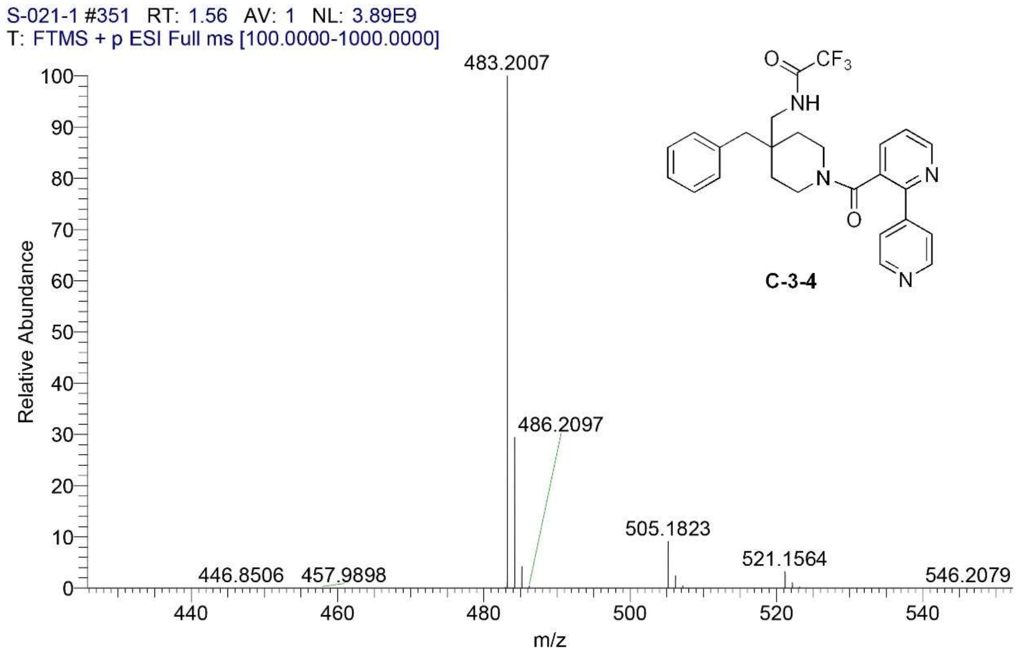

Figure S255: HR-MS (ESI/ion trap) spectrum of **C-3-4**

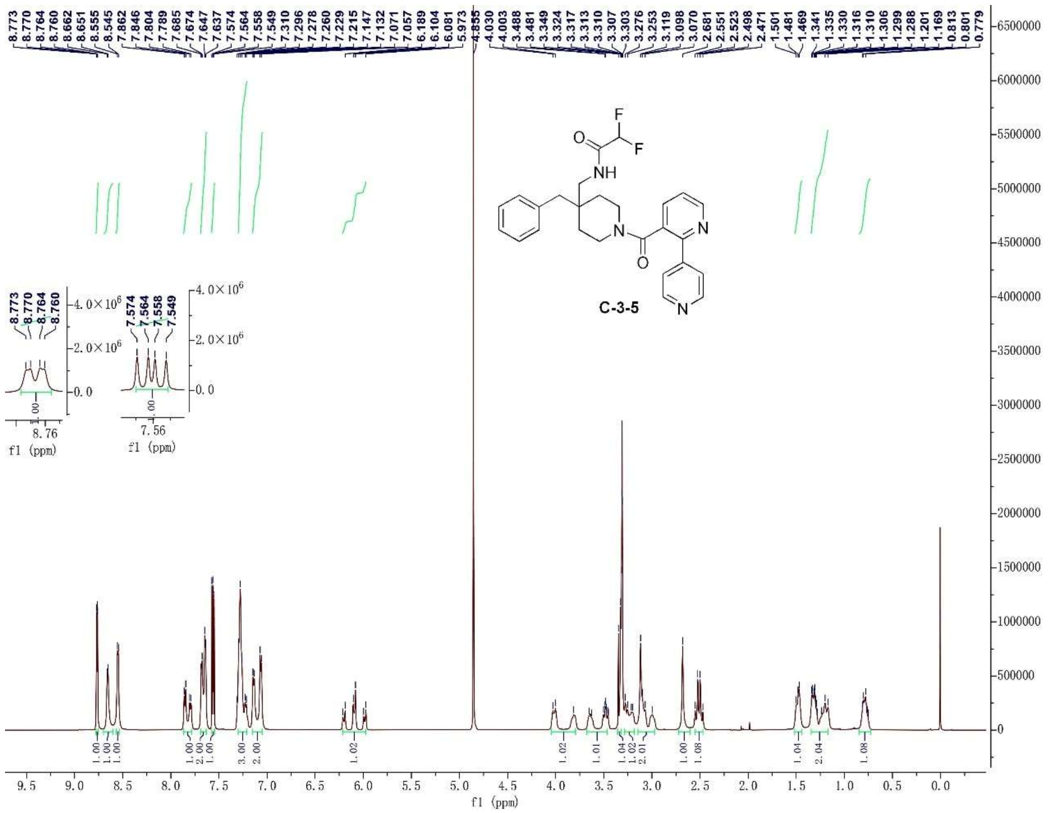

Figure S256: ^{1}H NMR spectrum of **C-3-5**

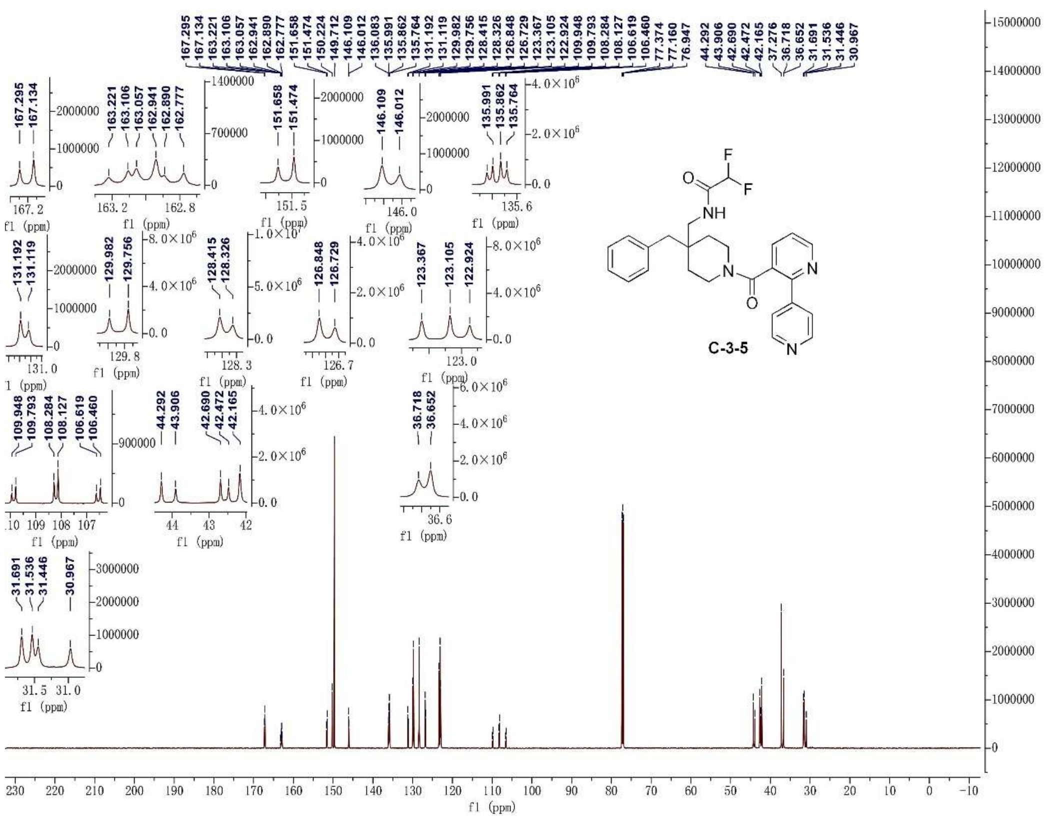

Figure S257: ^{13}C NMR spectrum of **C-3-5**

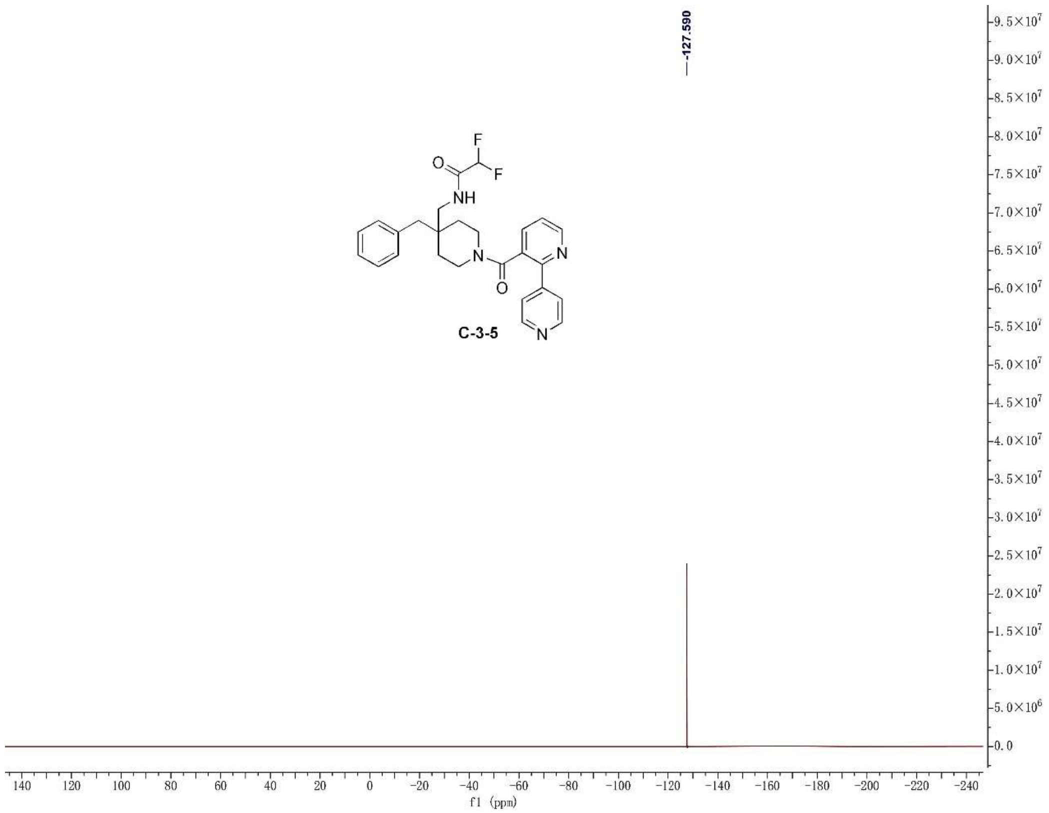

Figure S258: ^{19}F NMR spectrum of **C-3-5**

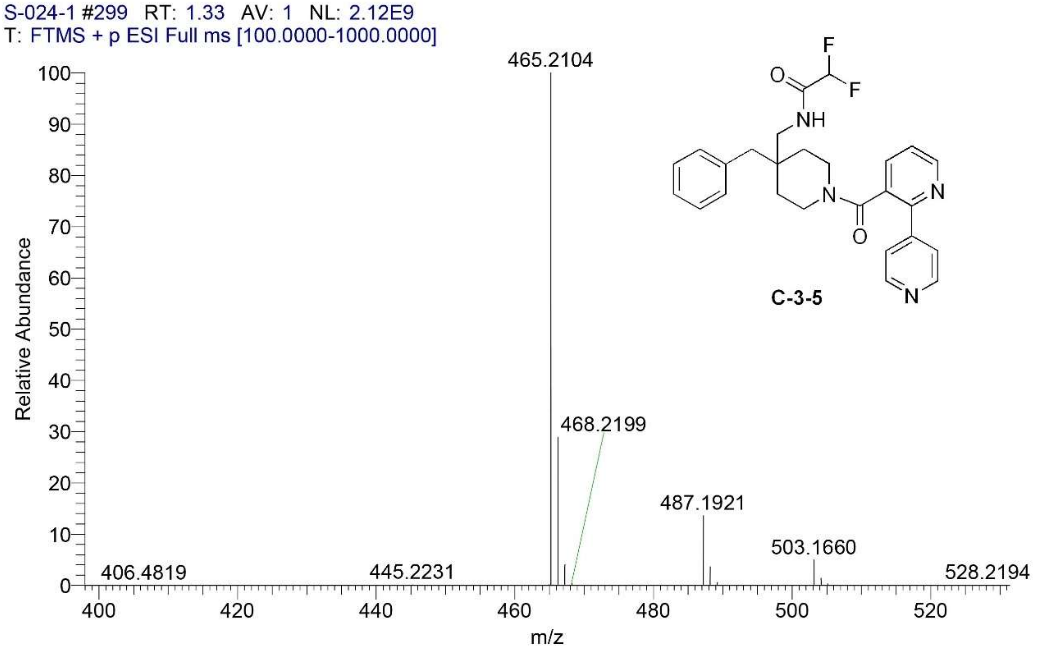

Figure S259: HR-MS (ESI/ion trap) spectrum of **C-3-5**

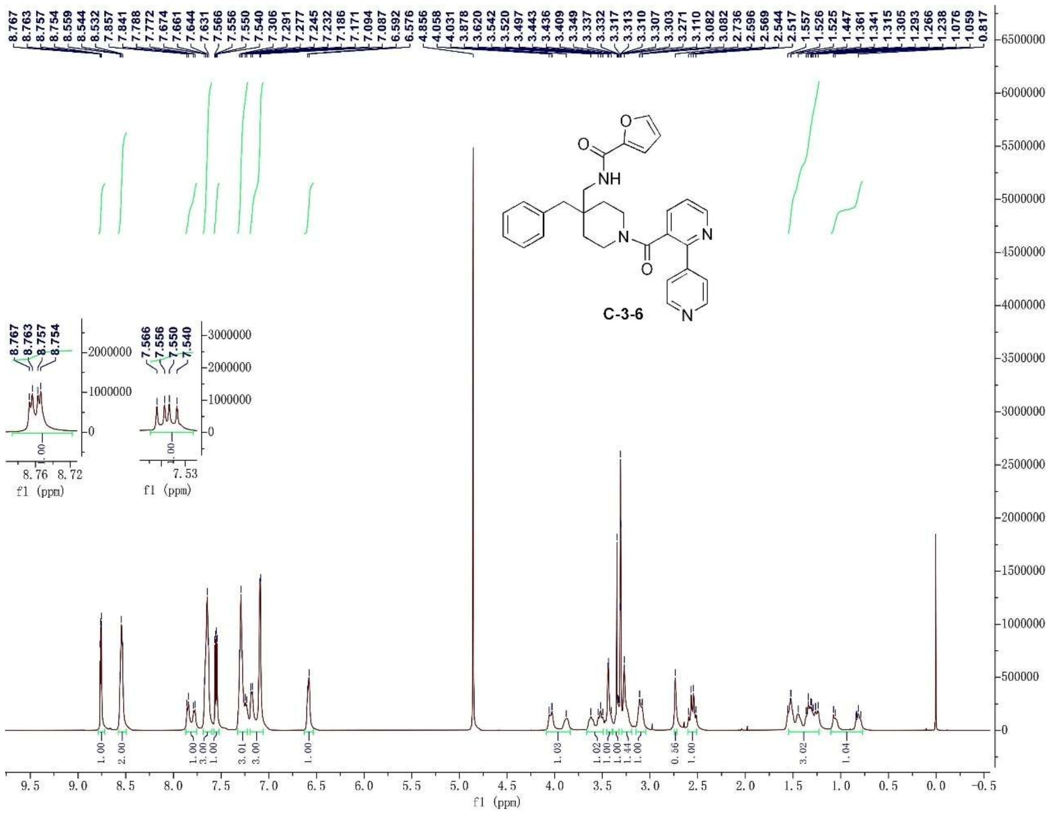

Figure S260: ^{1}H NMR spectrum of **C-3-6**

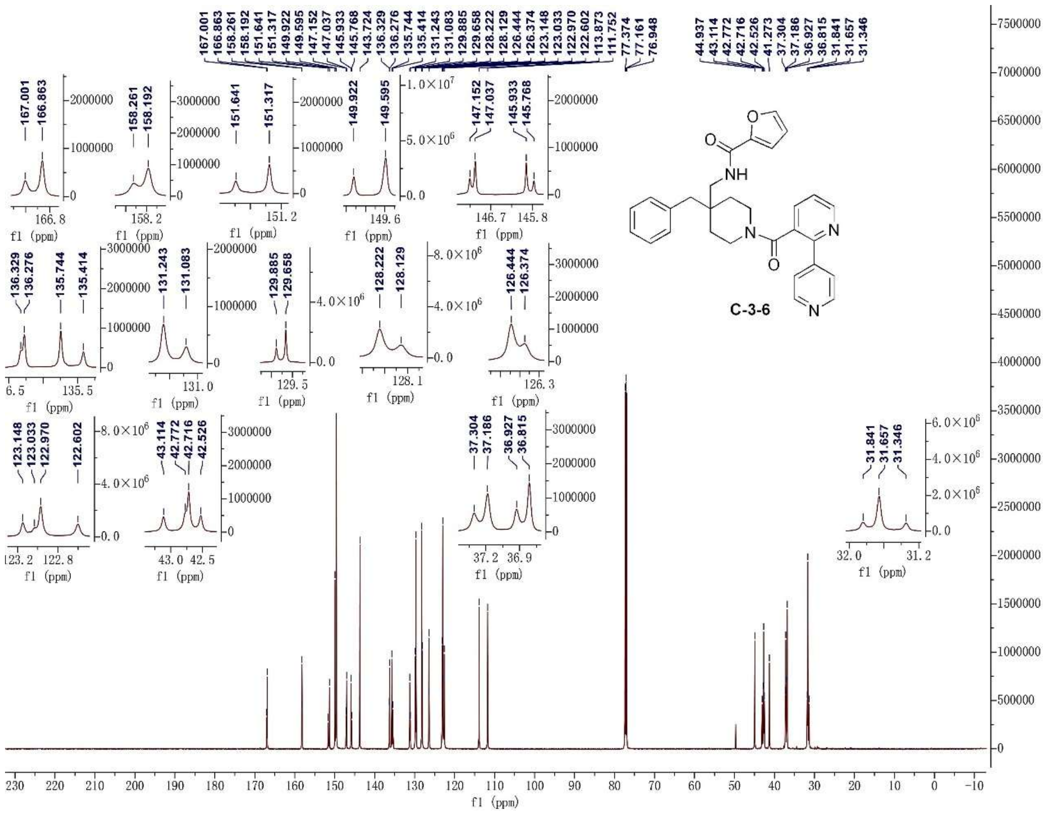

Figure S261: ^{13}C NMR spectrum of **C-3-6**

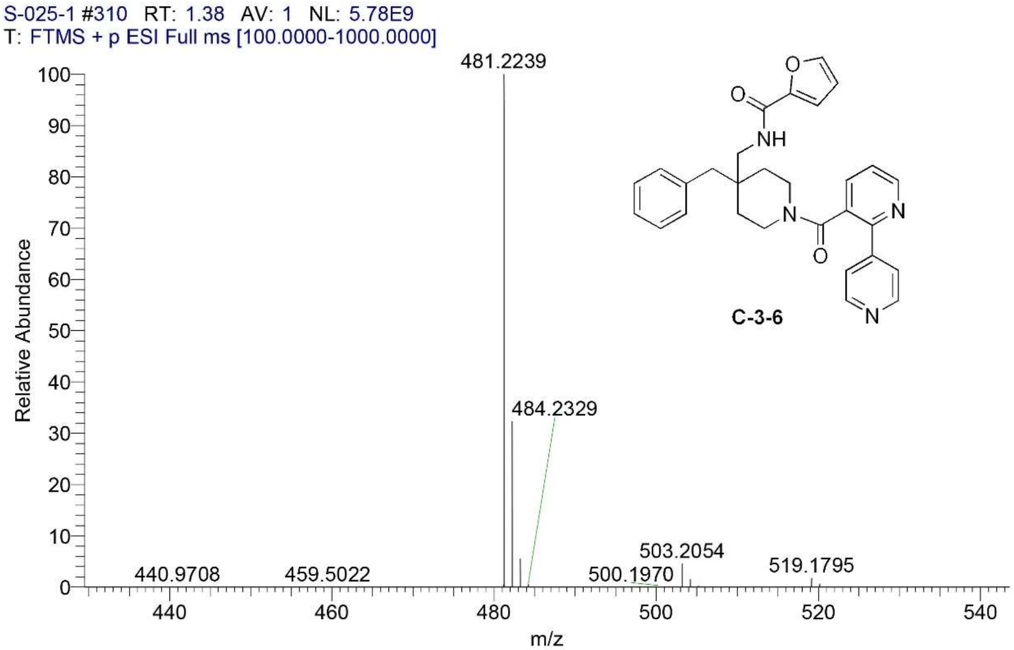

Figure S262: HR-MS (ESI/ion trap) spectrum of **C-3-6**

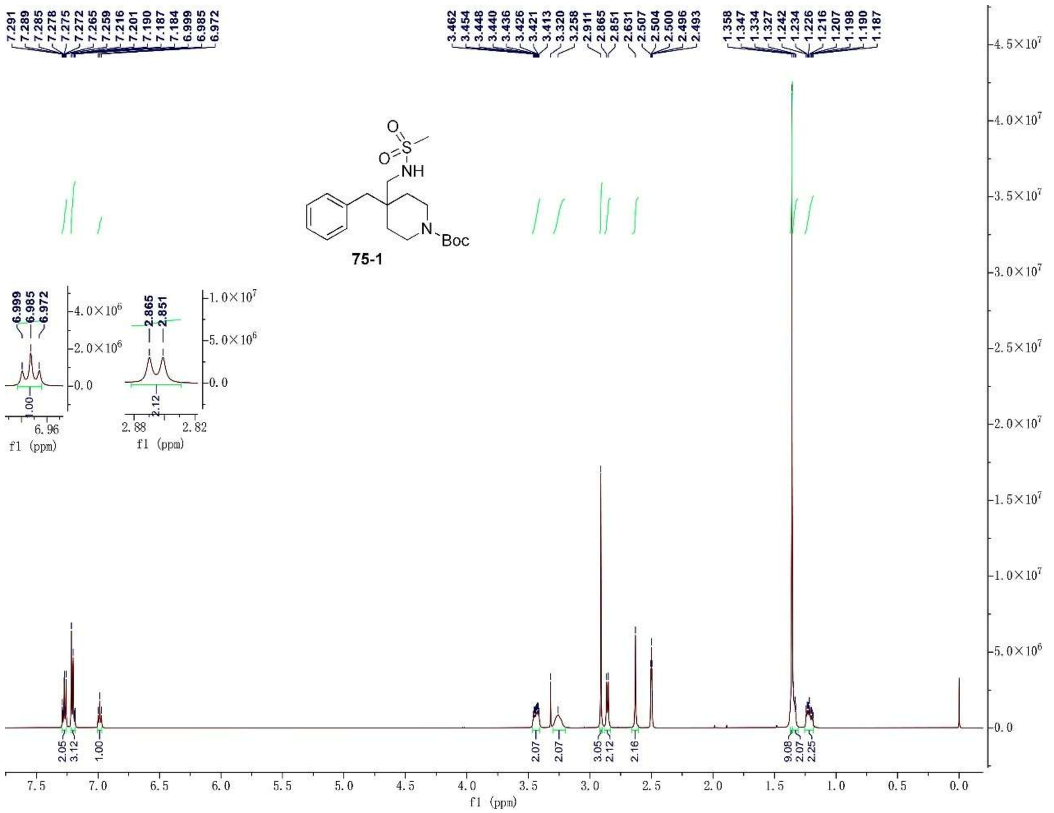

Figure S263: ^{1}H NMR spectrum of **75-1**

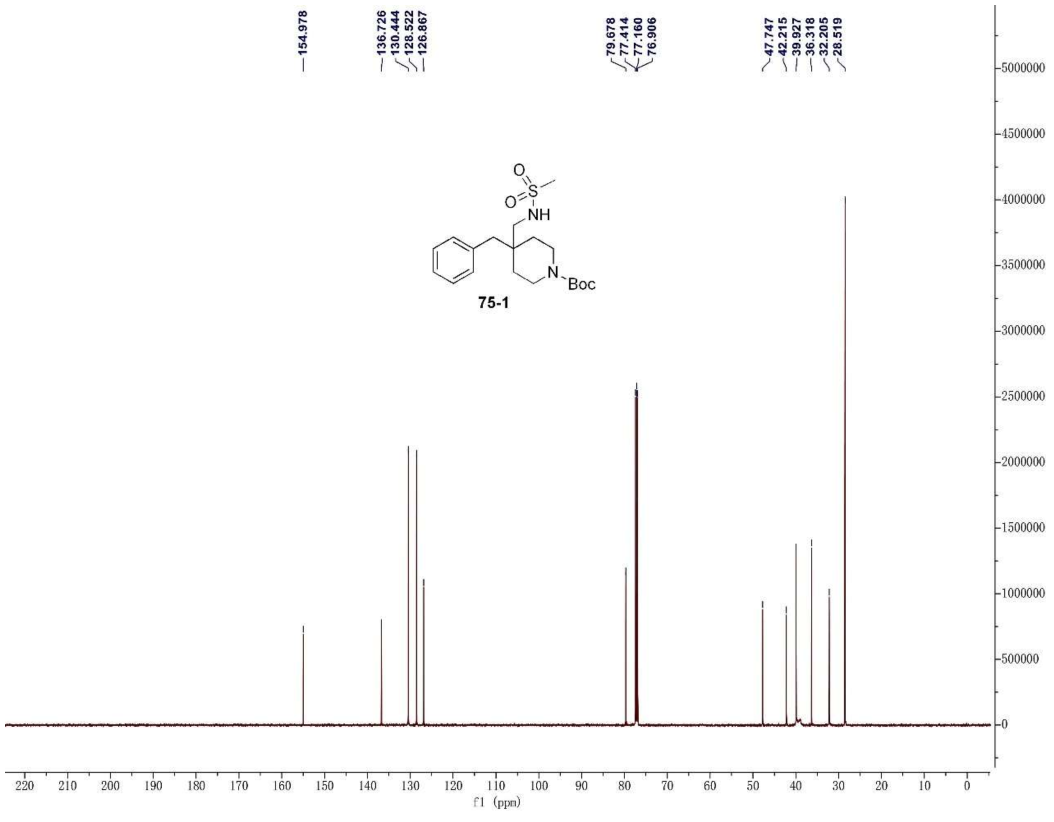

Figure S264: ^{13}C NMR spectrum of **75-1**

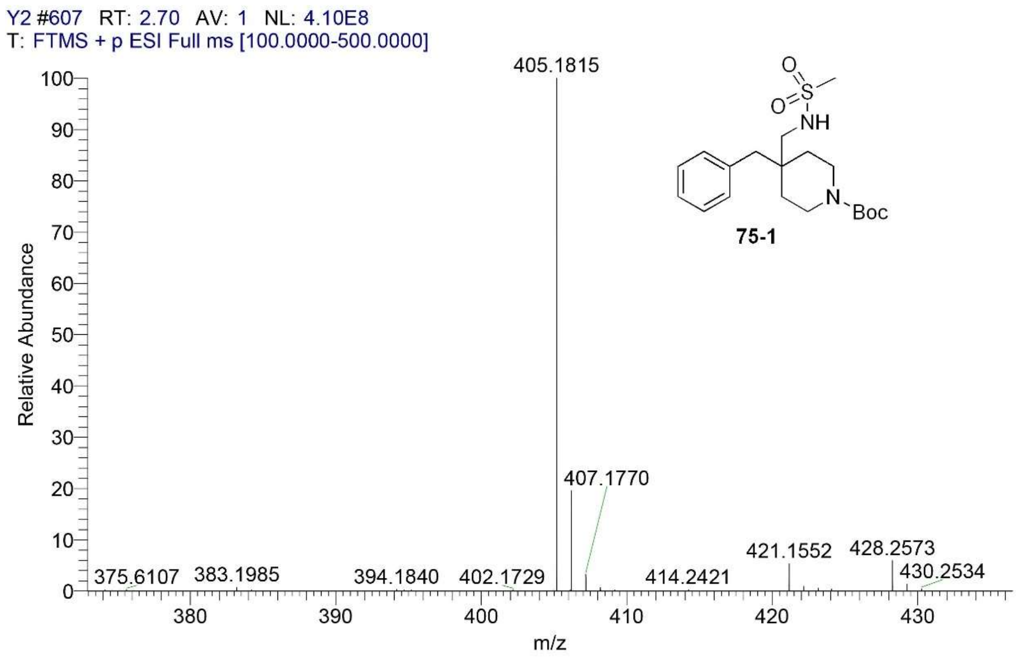

Figure S265: HR-MS (ESI/ion trap) spectrum of **75-1**

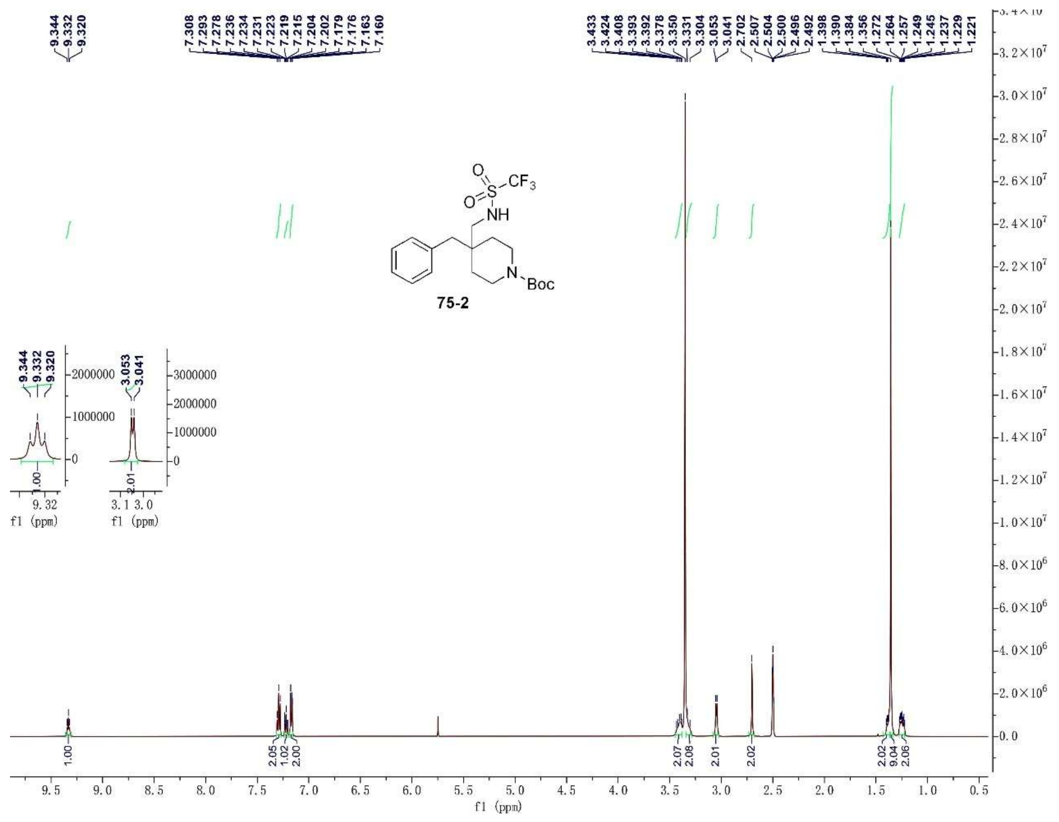

Figure S266: ^{1}H NMR spectrum of **75-2**

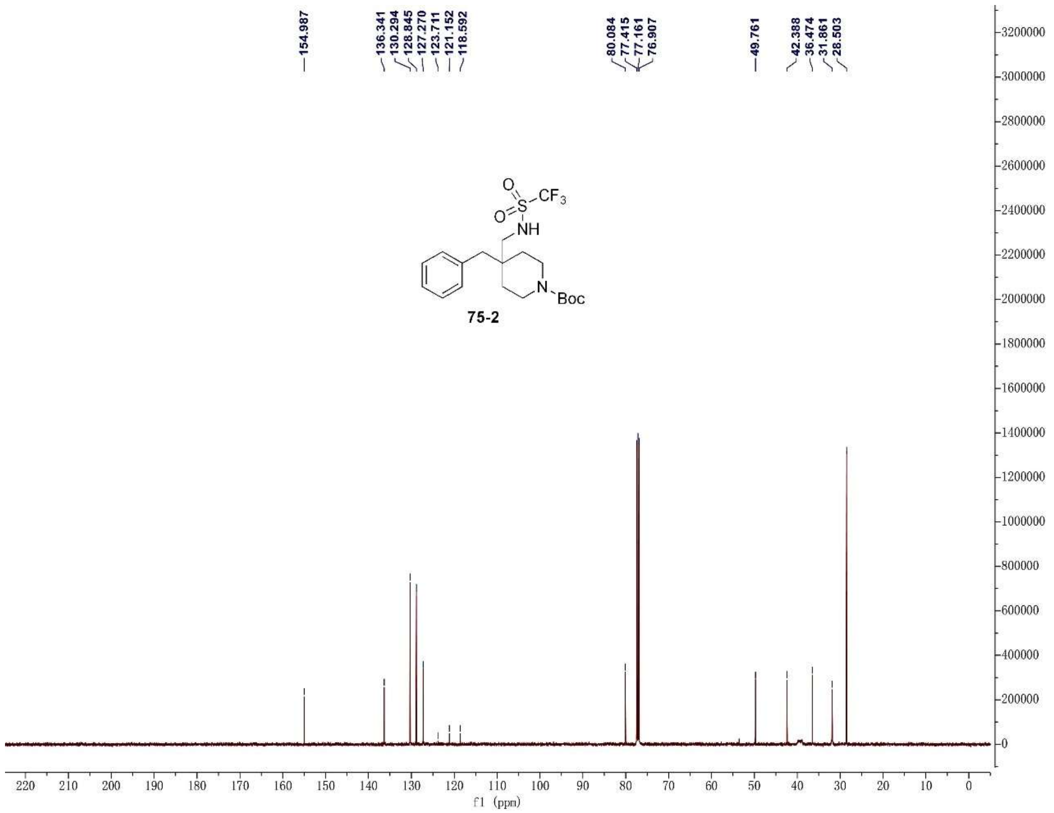

Figure S267: ^{13}C NMR spectrum of **75-2**

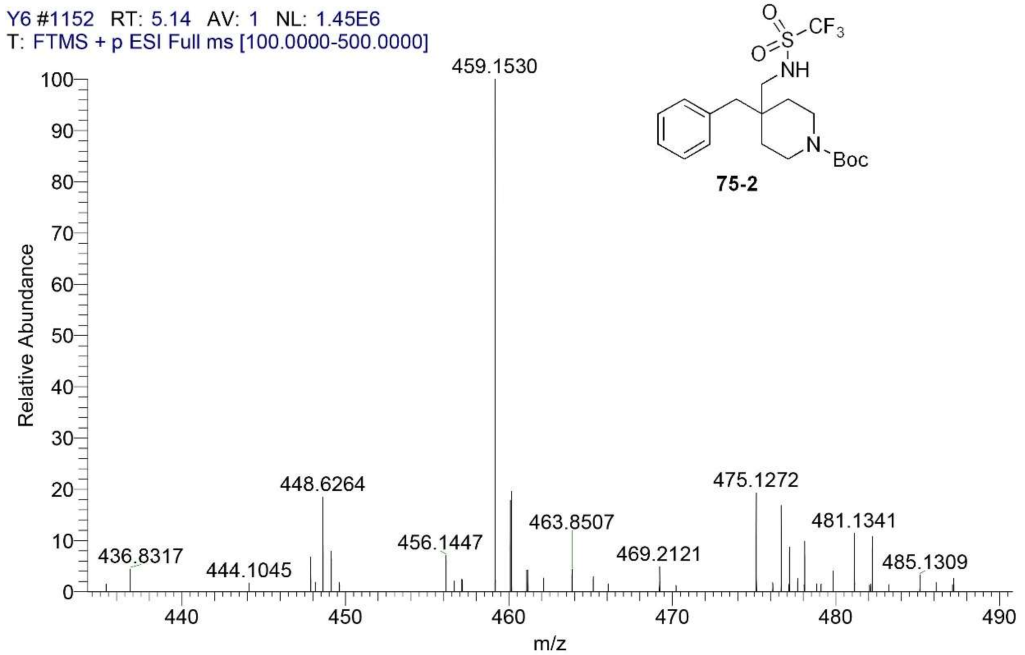

Figure S268: HR-MS (ESI/ion trap) spectrum of **75-2**

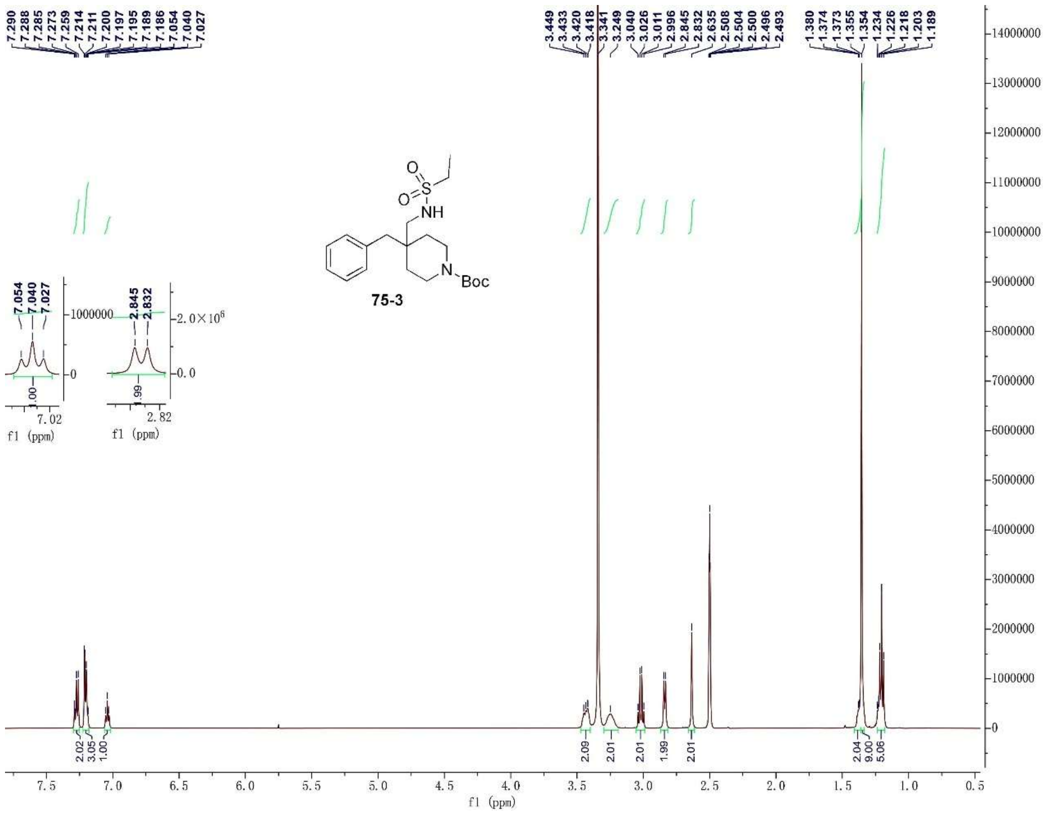

Figure S269: ^{1}H NMR spectrum of **75-3**

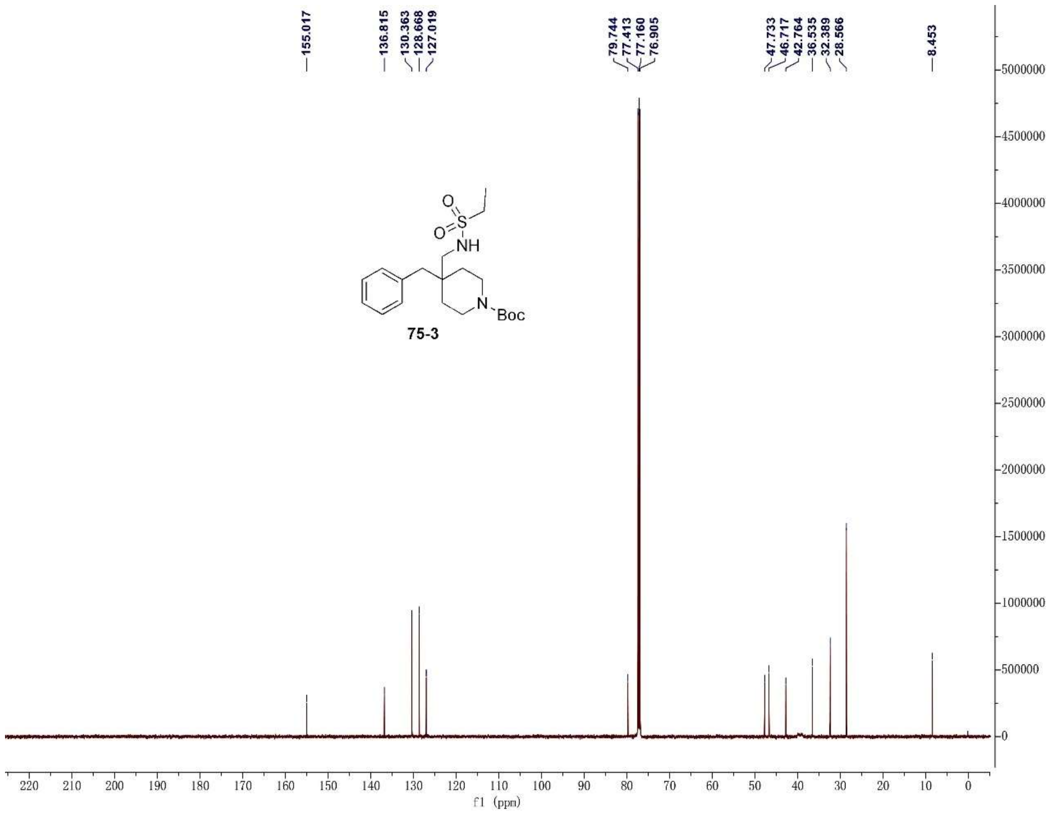

Figure S270: ^{13}C NMR spectrum of **75-3**

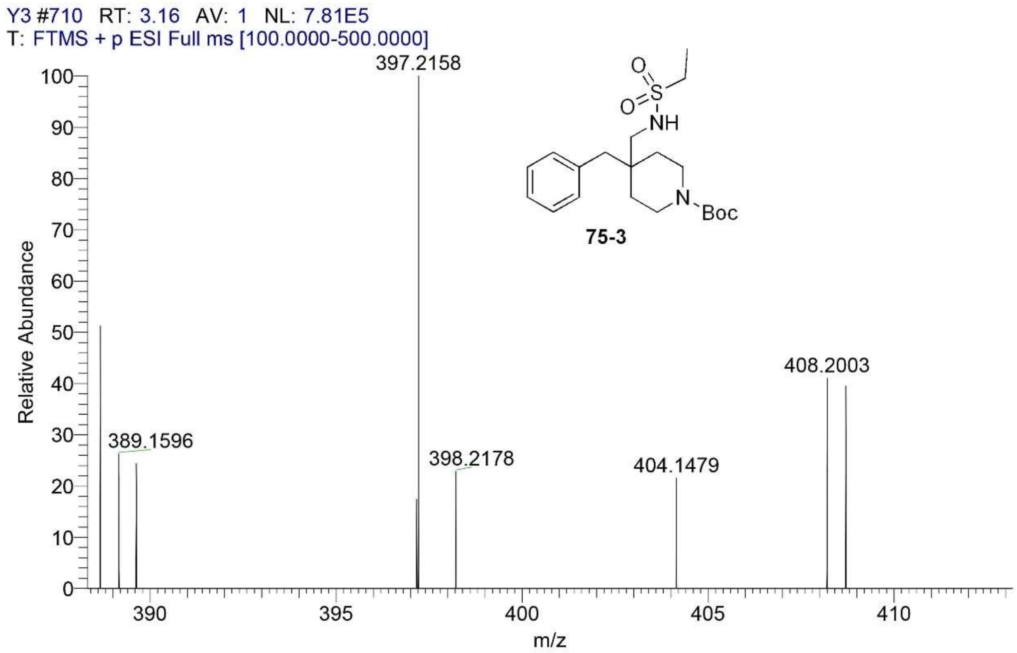

Figure S271: HR-MS (ESI/ion trap) spectrum of **75-3**

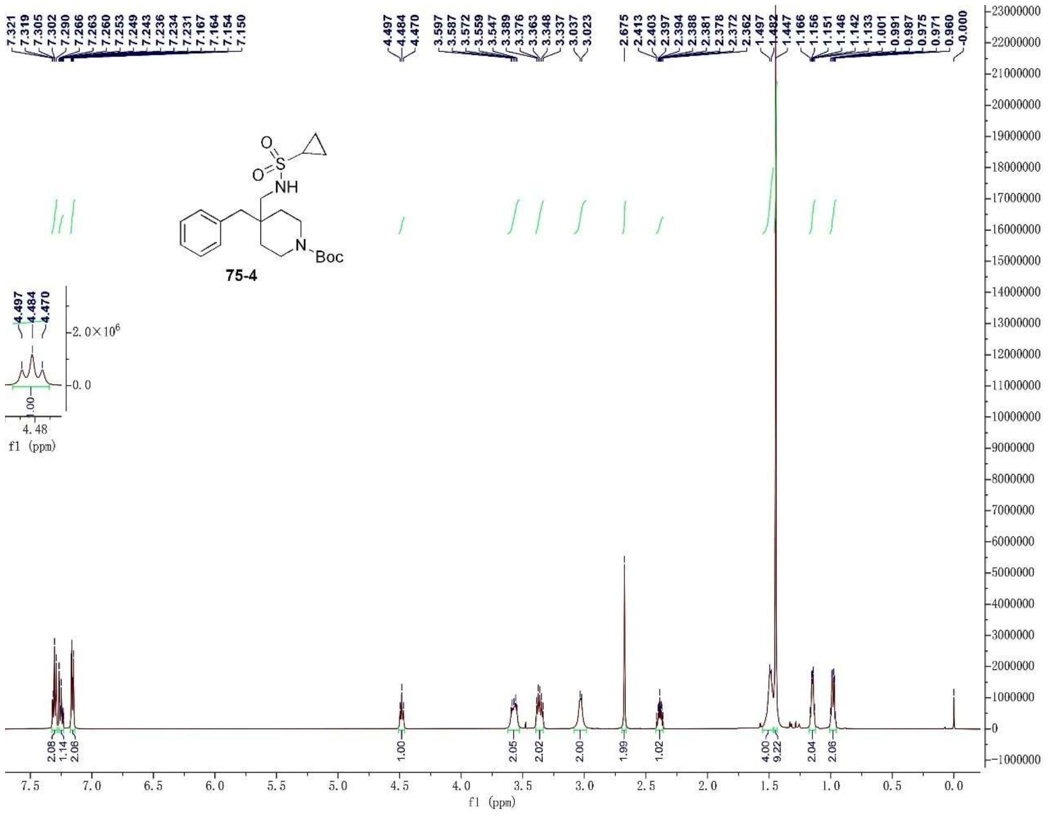

Figure S272: ^{1}H NMR spectrum of **75-4**

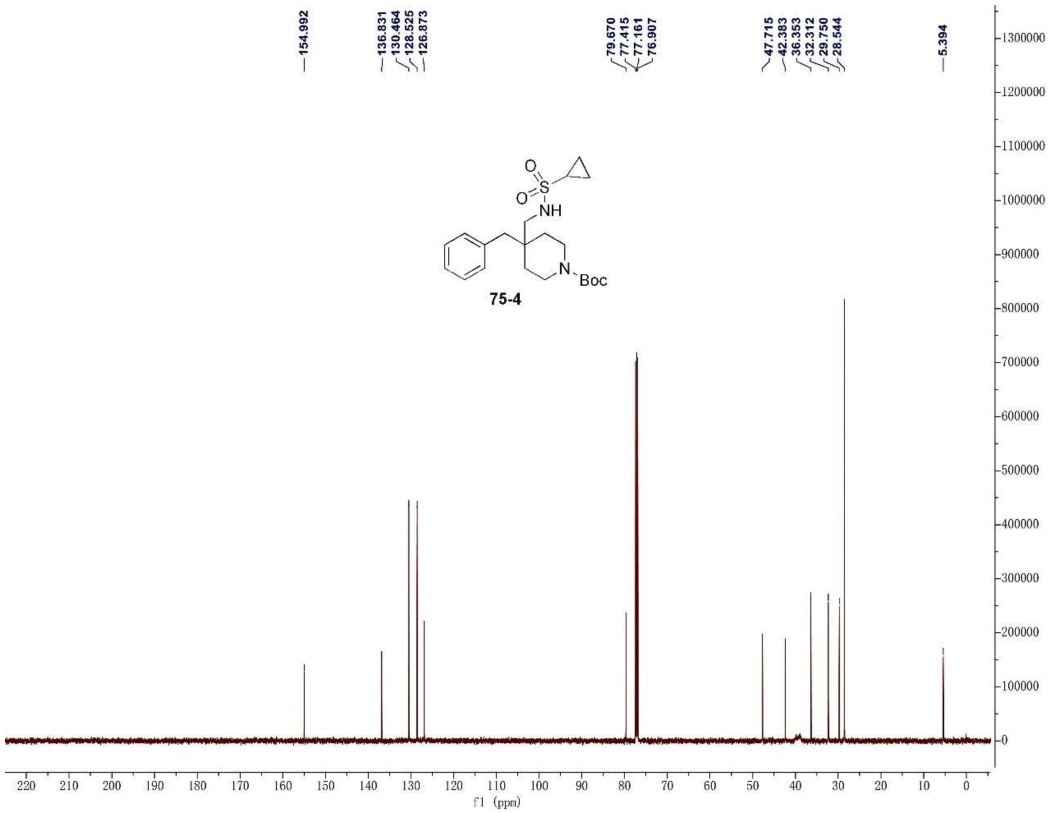

Figure S273: ^{13}C NMR spectrum of **75-4**

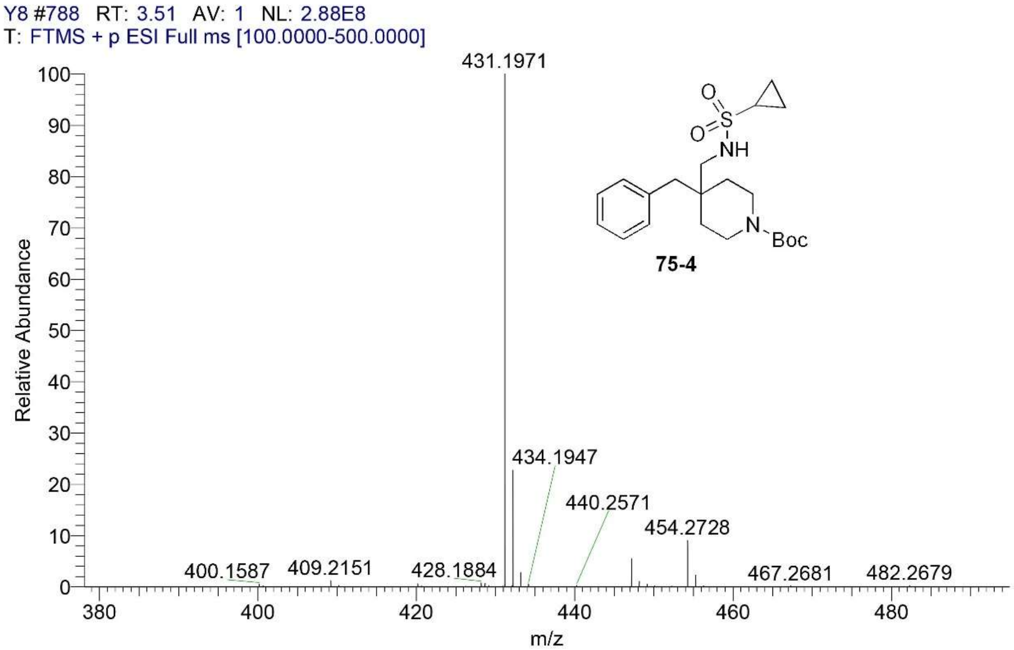

Figure S274: HR-MS (ESI/ion trap) spectrum of **75-4**

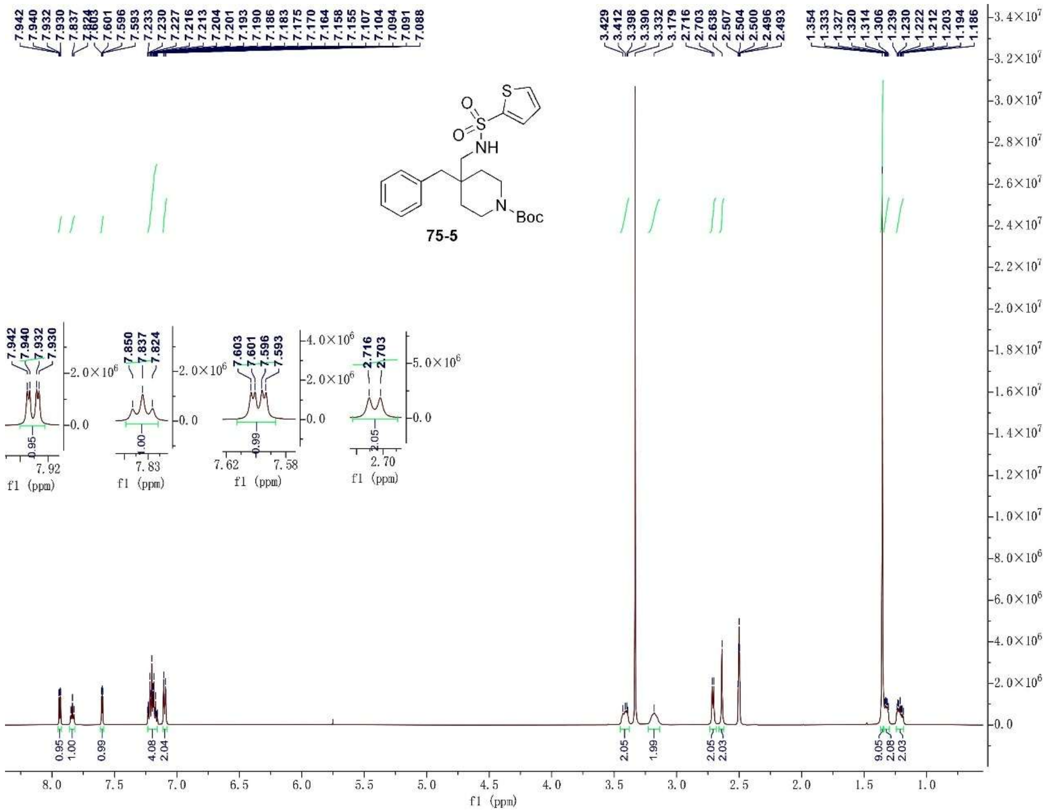

Figure S275: ^{1}H NMR spectrum of **75-5**

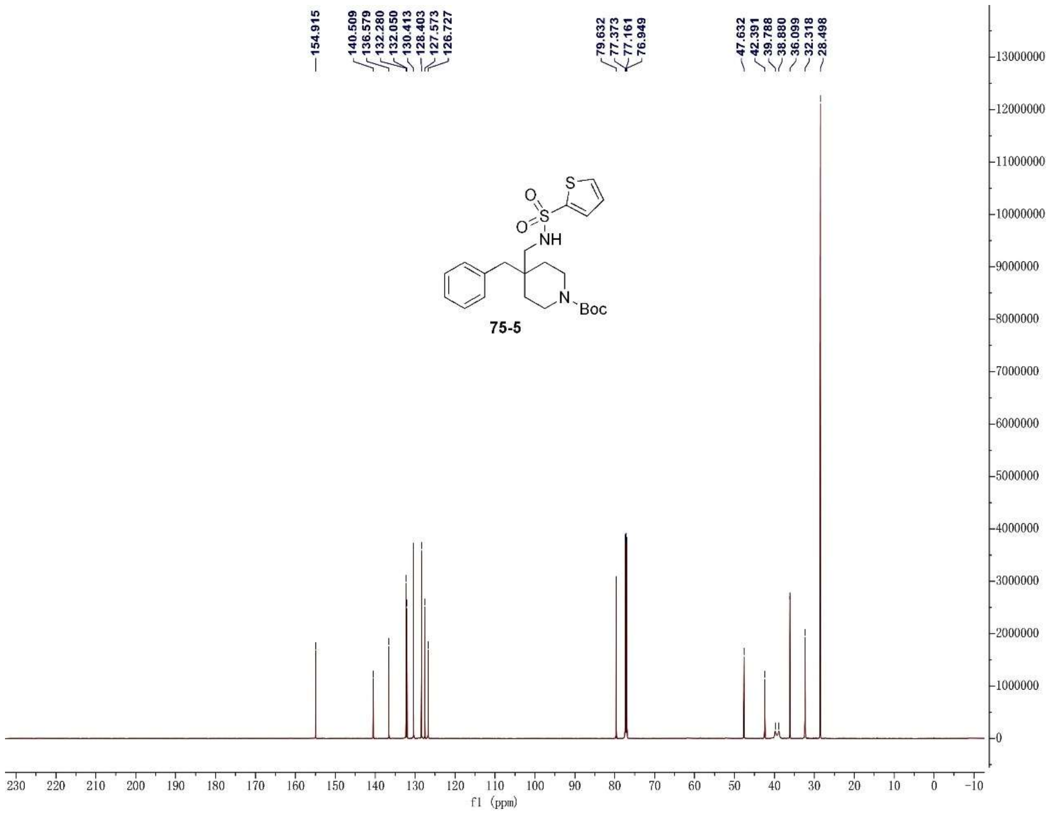

Figure S276: ^{13}C NMR spectrum of **75-5**

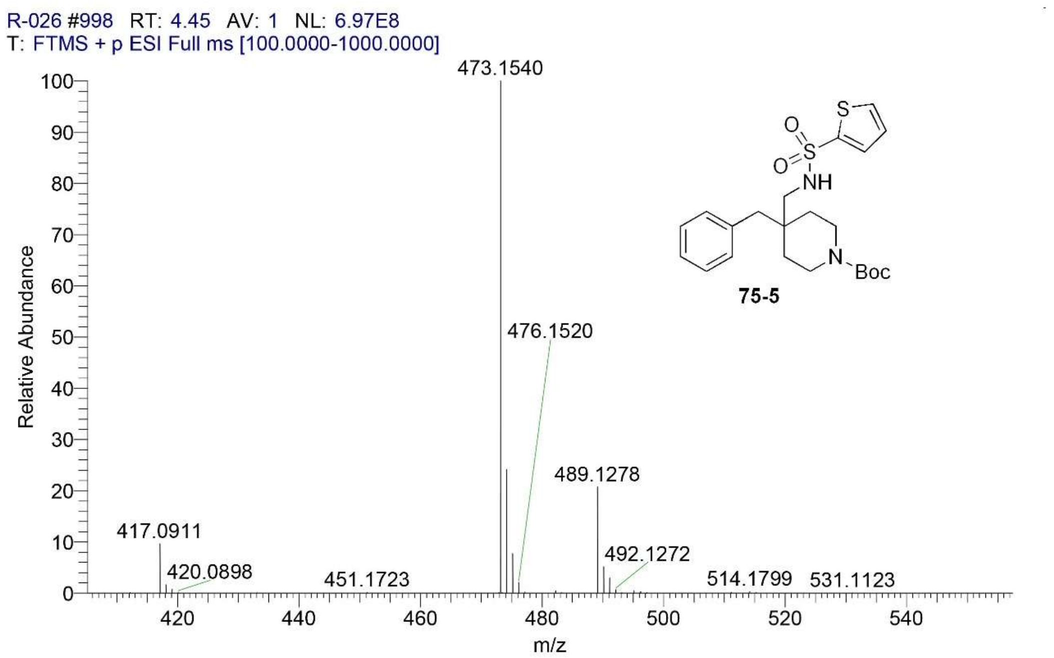

Figure S277: HR-MS (ESI/ion trap) spectrum of **75-5**

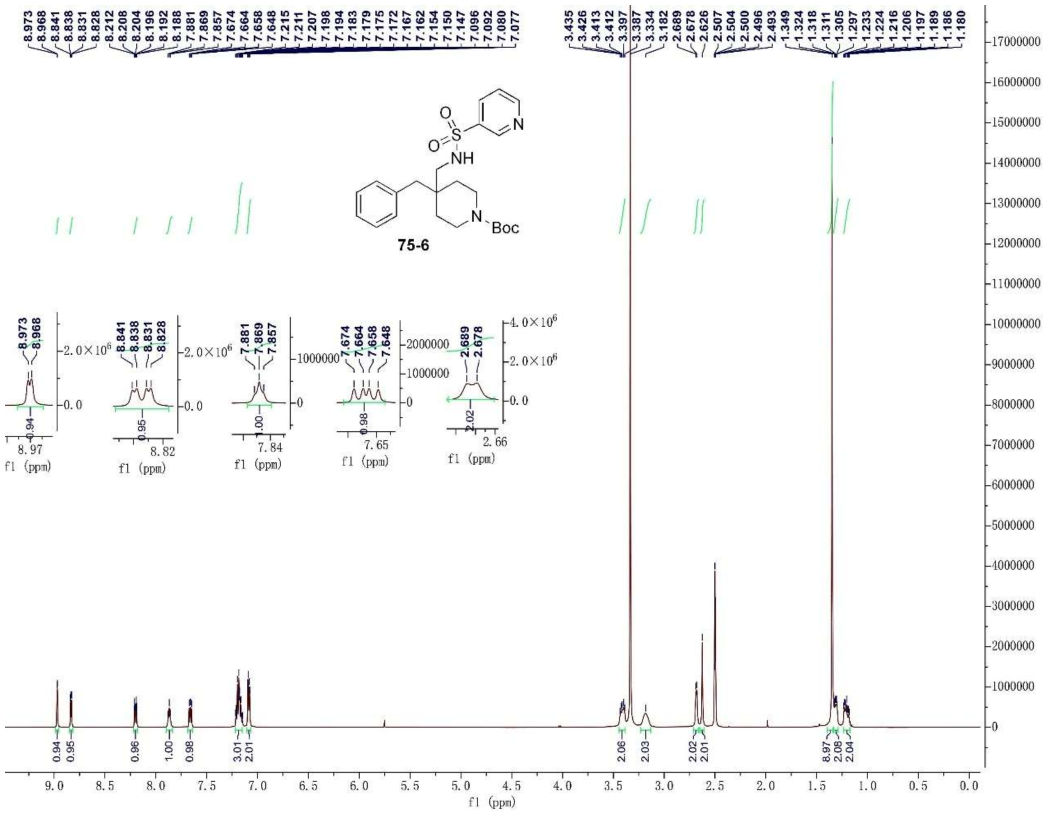

Figure S278: ^{1}H NMR spectrum of **75-6**

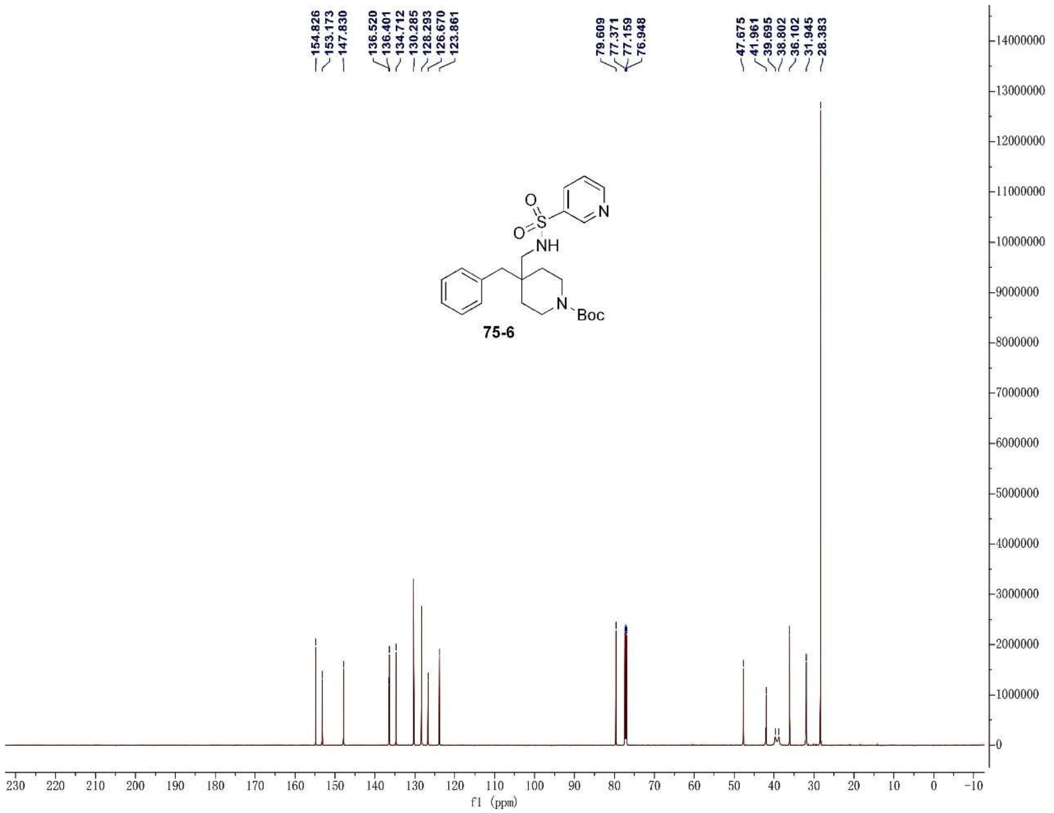

Figure S279: ^{13}C NMR spectrum of **75-6**

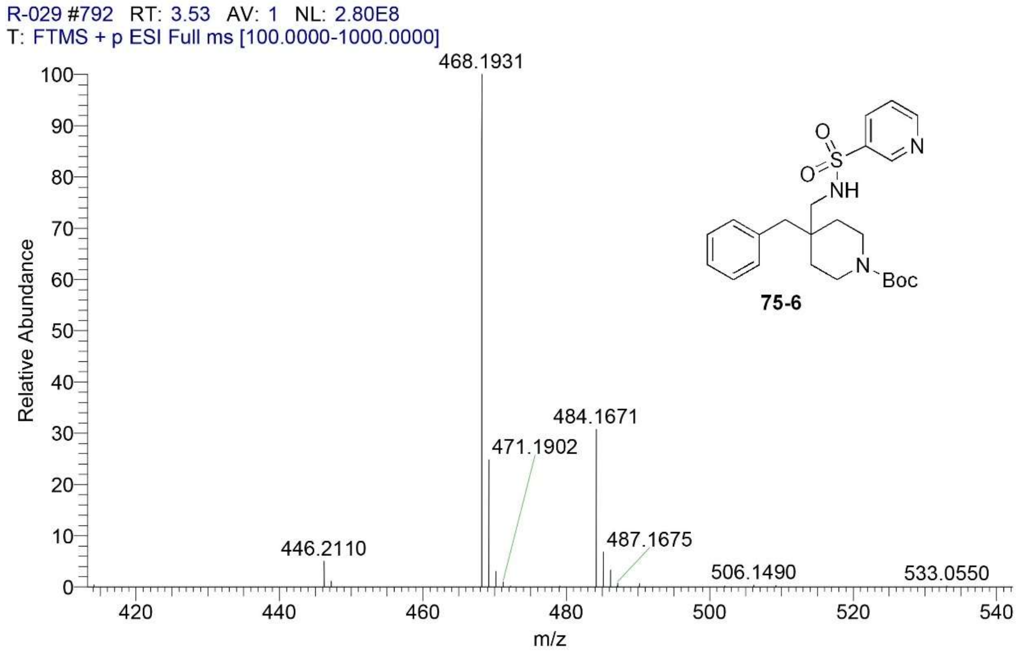

Figure S280: HR-MS (ESI/ion trap) spectrum of **75-6**

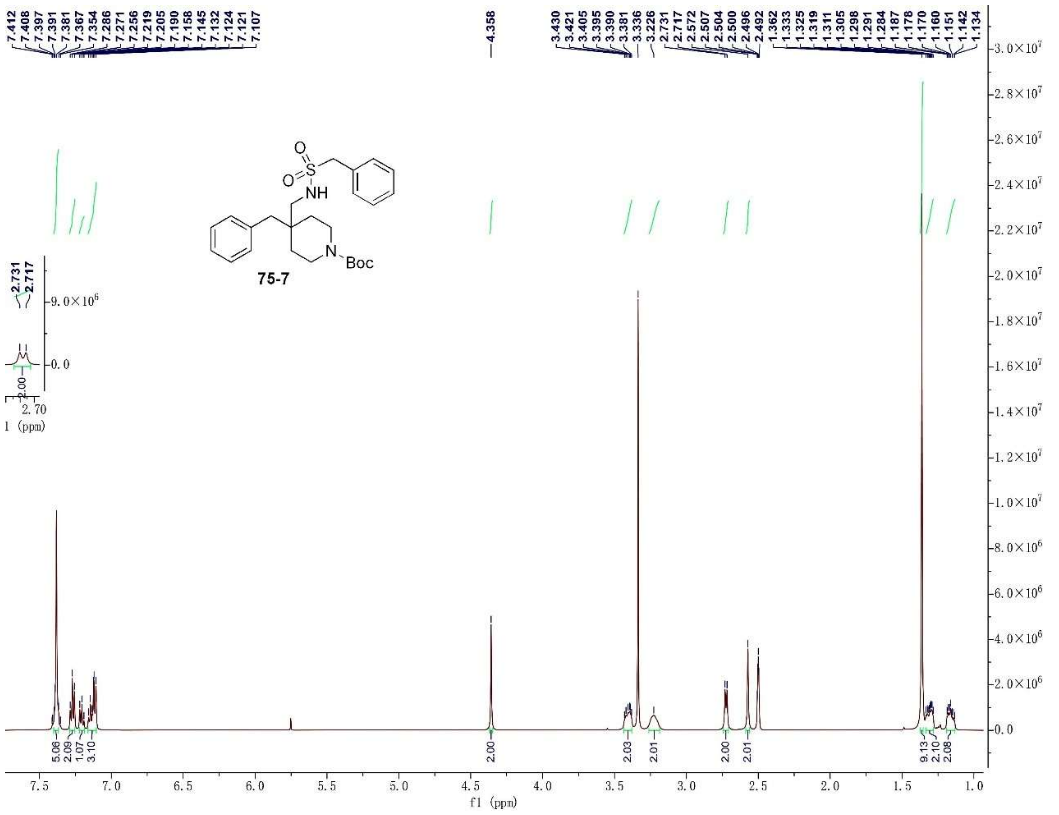

Figure S281: ^{1}H NMR spectrum of **75-7**

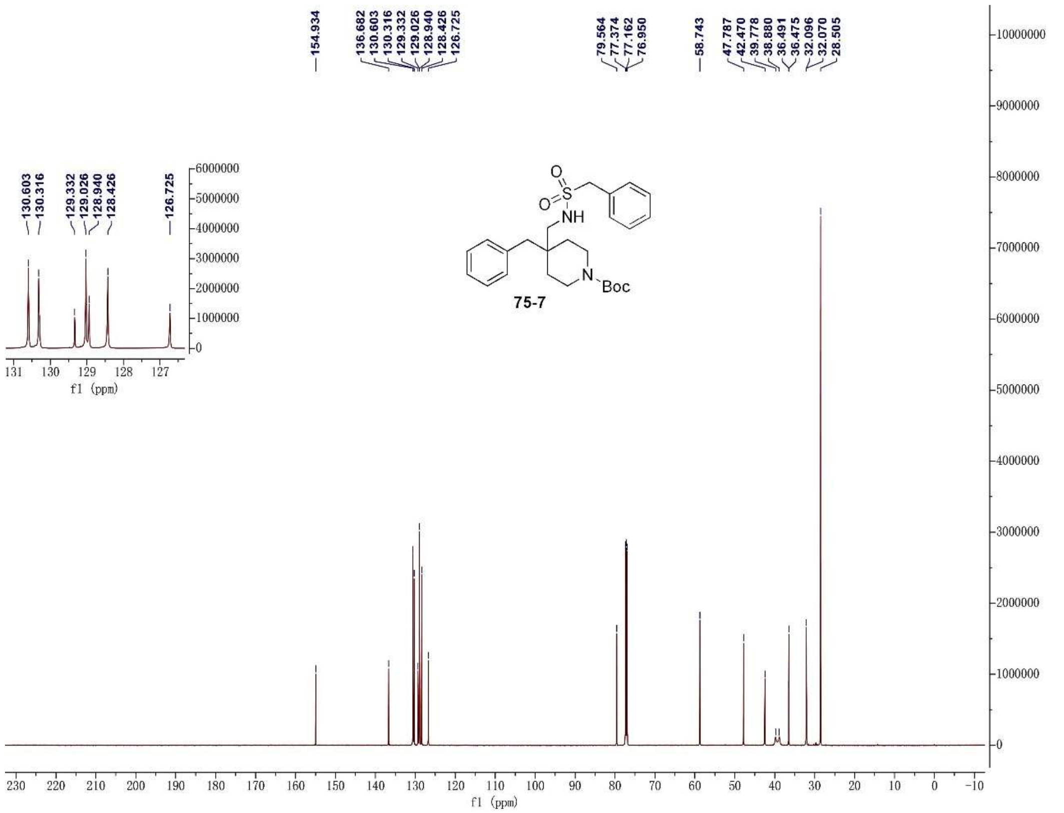

Figure S282: ^{13}C NMR spectrum of **75-7**

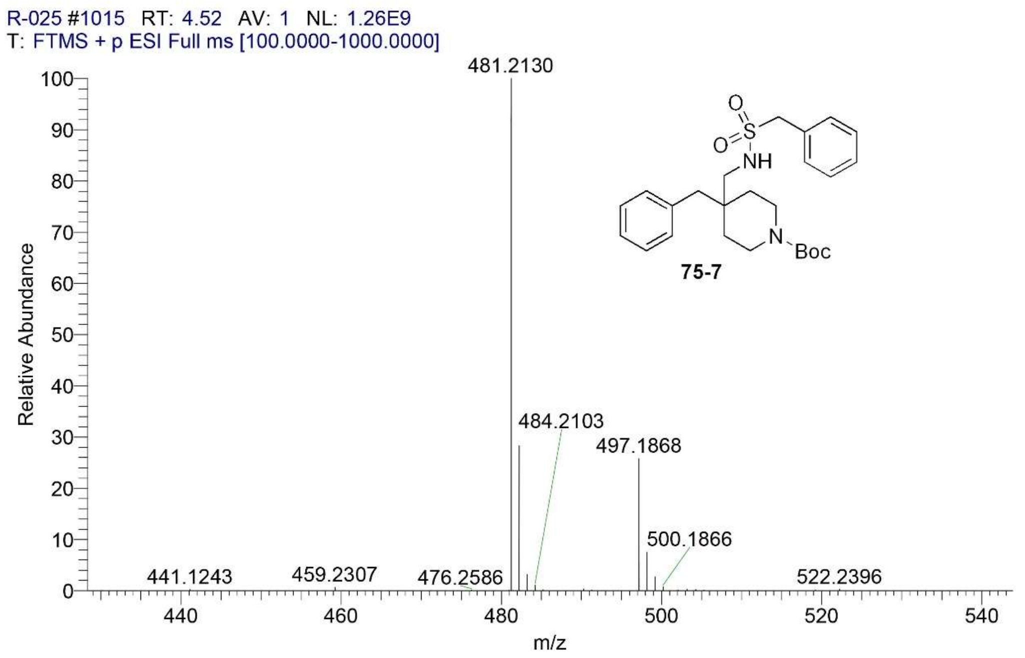

Figure S283: HR-MS (ESI/ion trap) spectrum of **75-7**

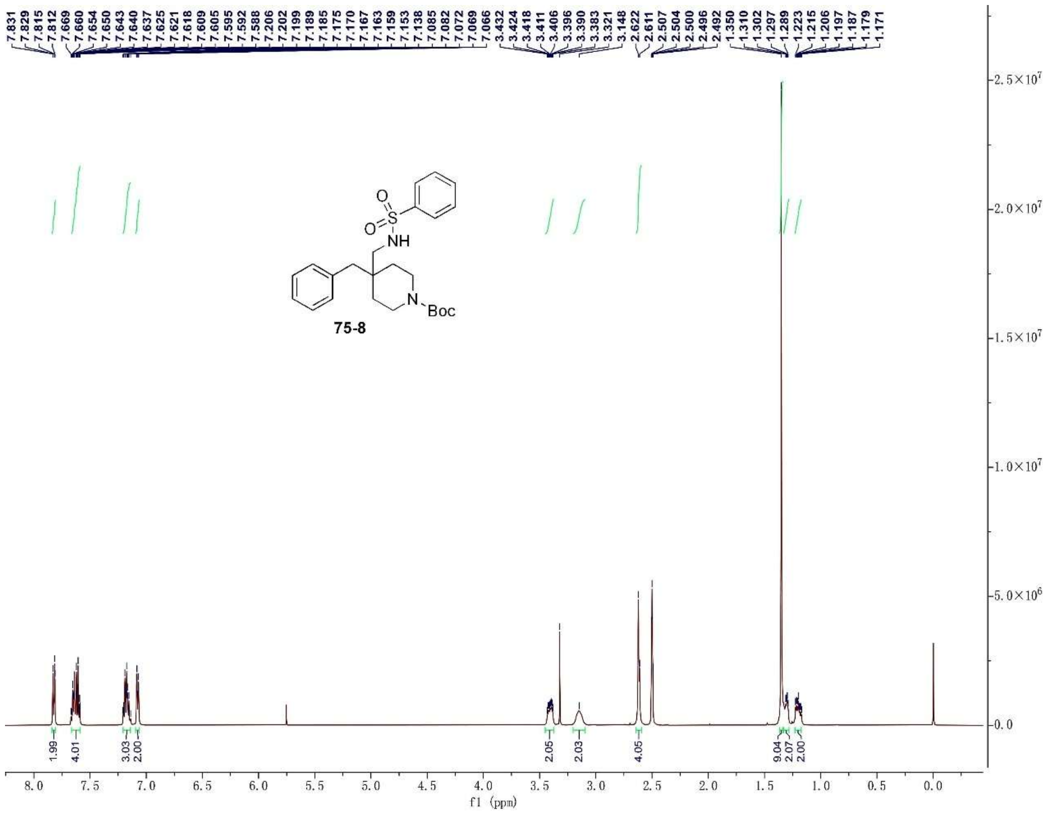

Figure S284: ^{1}H NMR spectrum of **75-8**

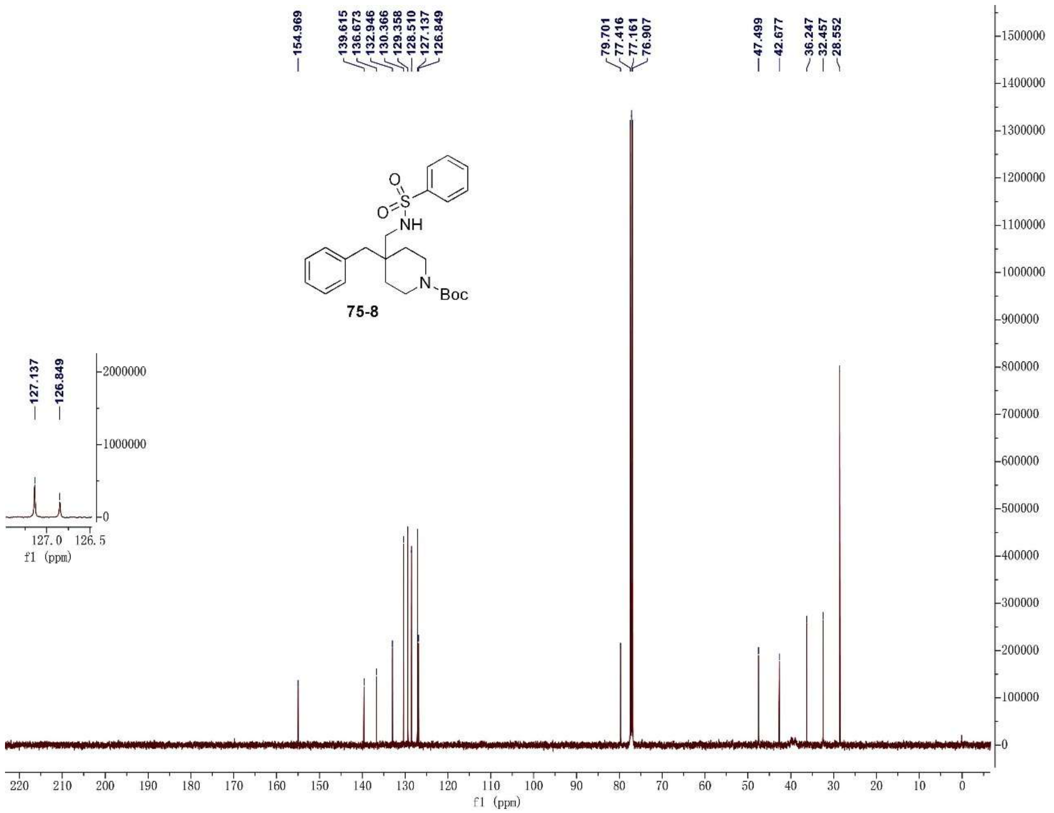

Figure S285: ^{13}C NMR spectrum of **75-8**

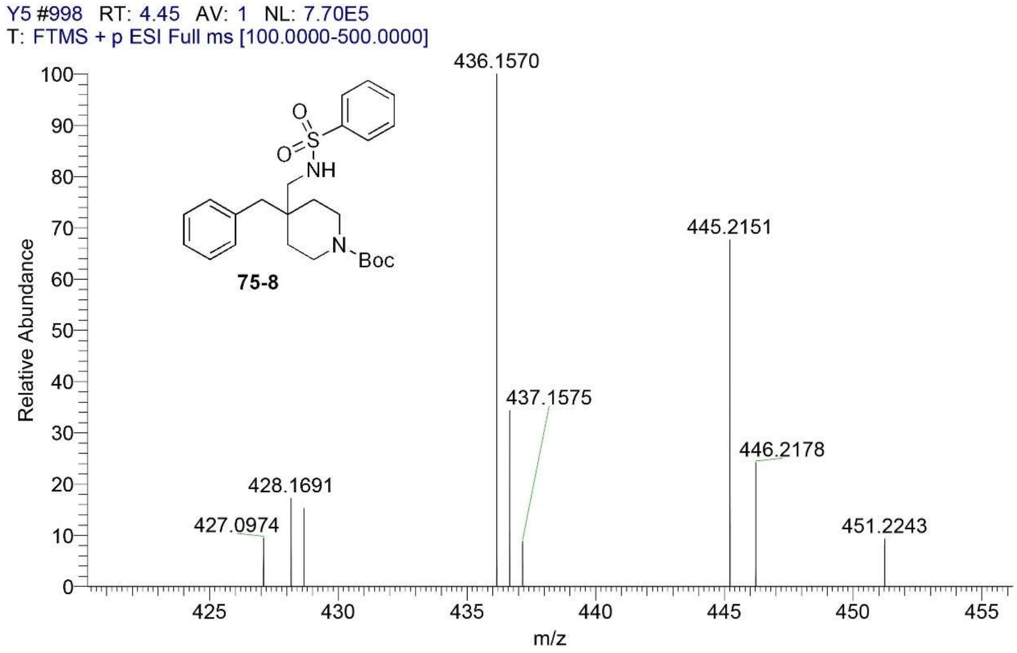

Figure S286: HR-MS (ESI/ion trap) spectrum of **75-8**

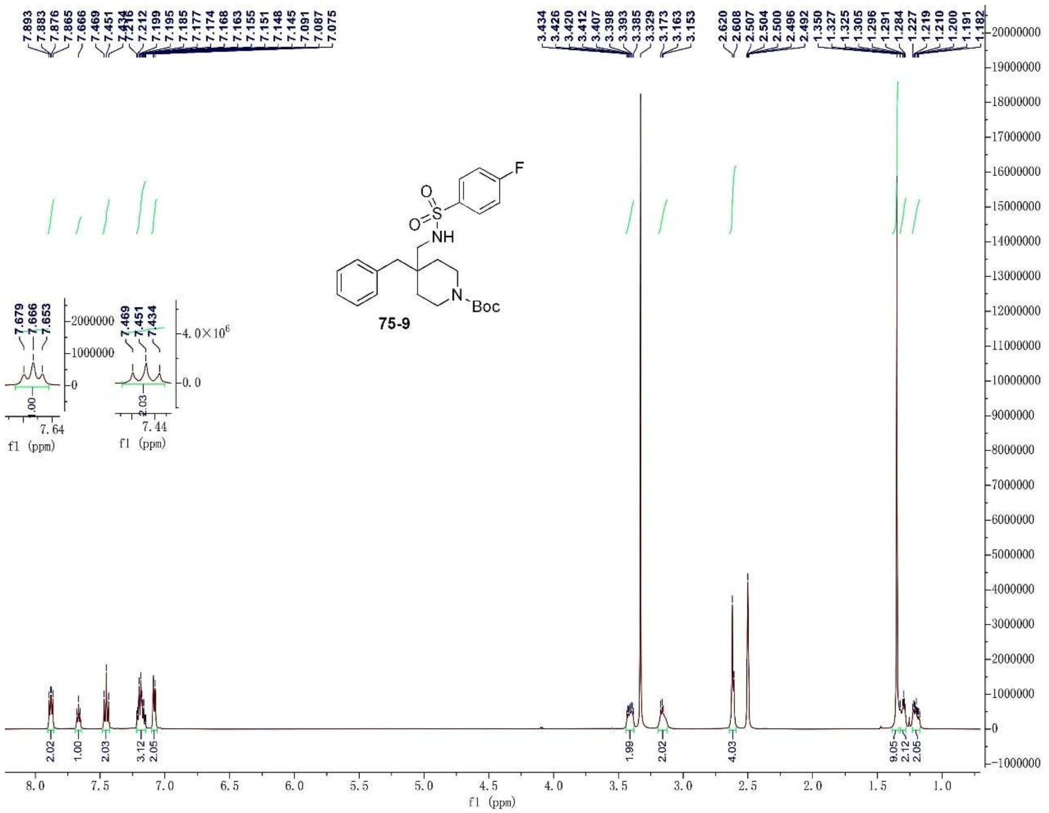

Figure S287: ^{1}H NMR spectrum of **75-9**

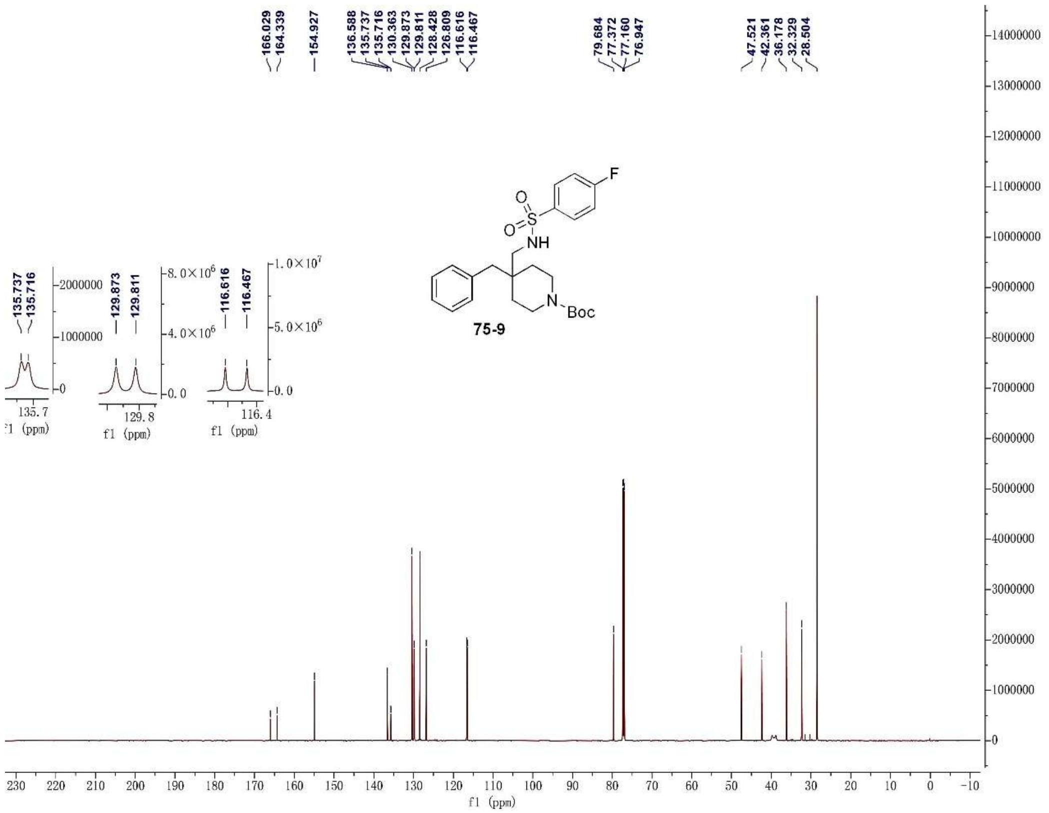

Figure S288: ^{13}C NMR spectrum of **75-9**

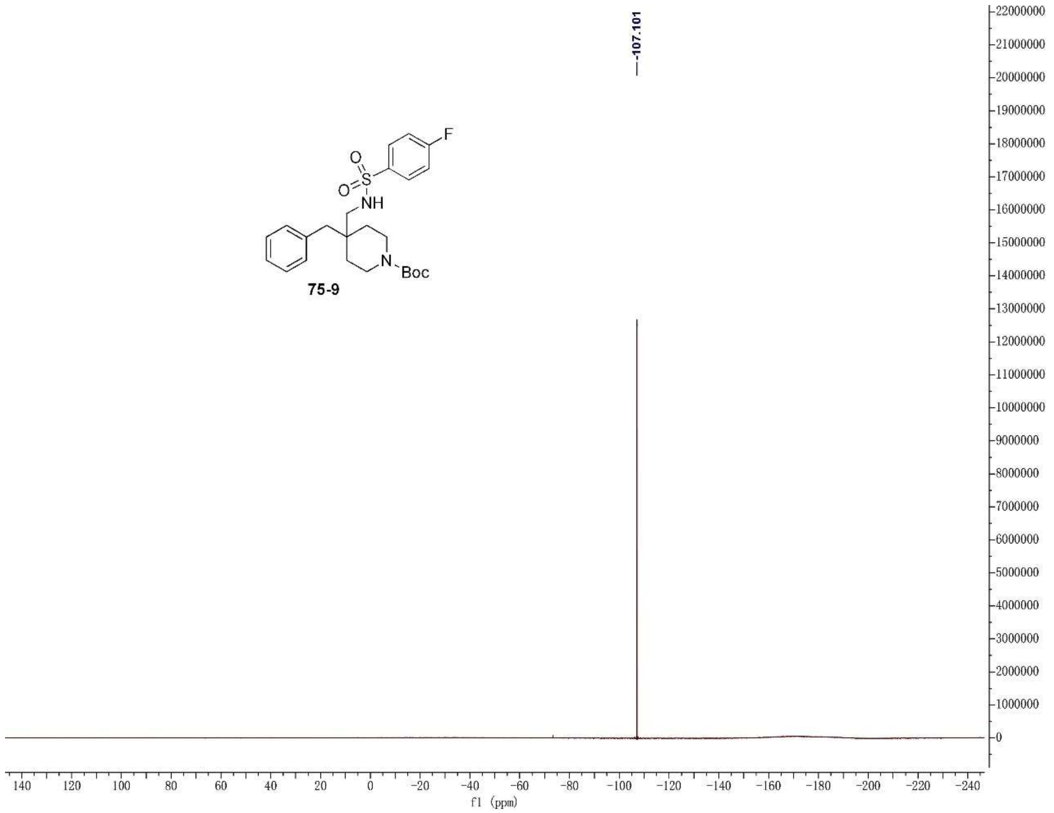

Figure S289: ^{19}F NMR spectrum of **75-9**

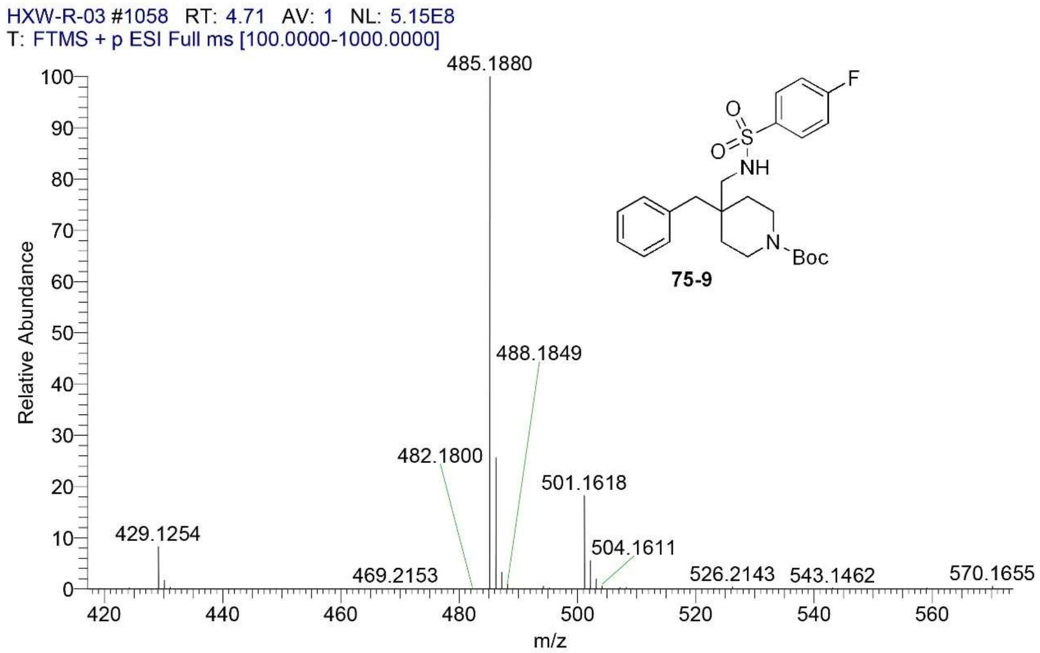

Figure S290: HR-MS (ESI/ion trap) spectrum of **75-9**

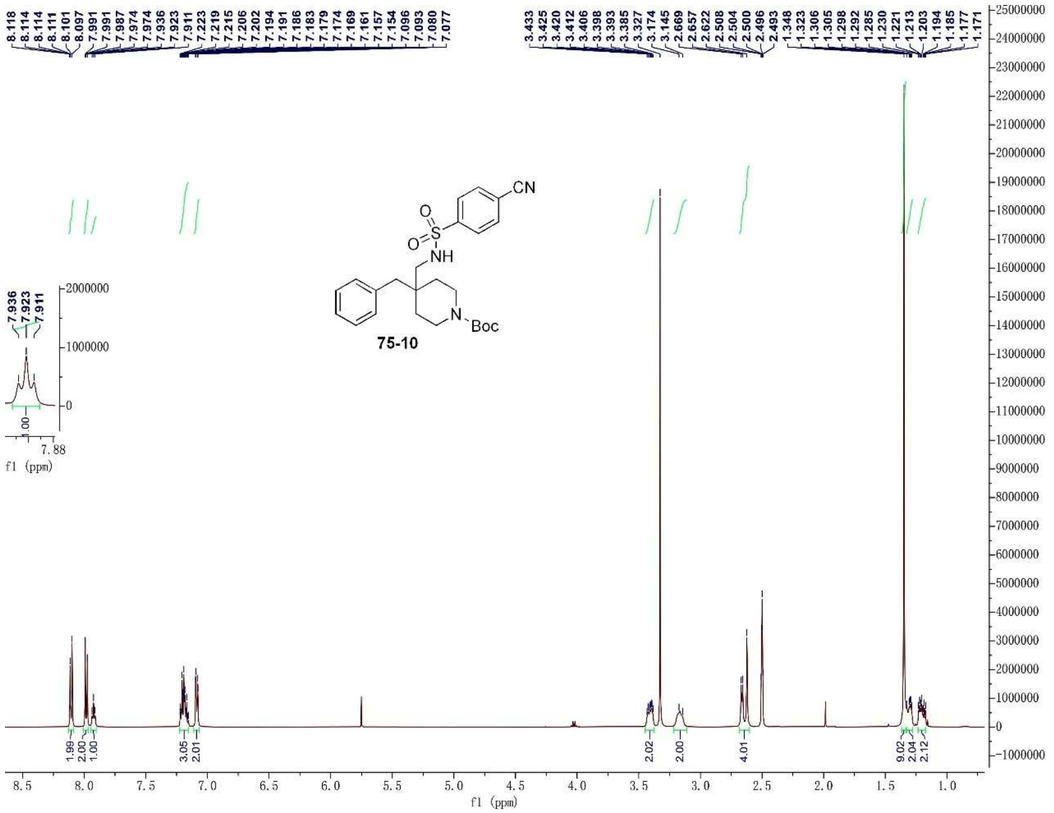

Figure S291: ^{1}H NMR spectrum of **75-10**

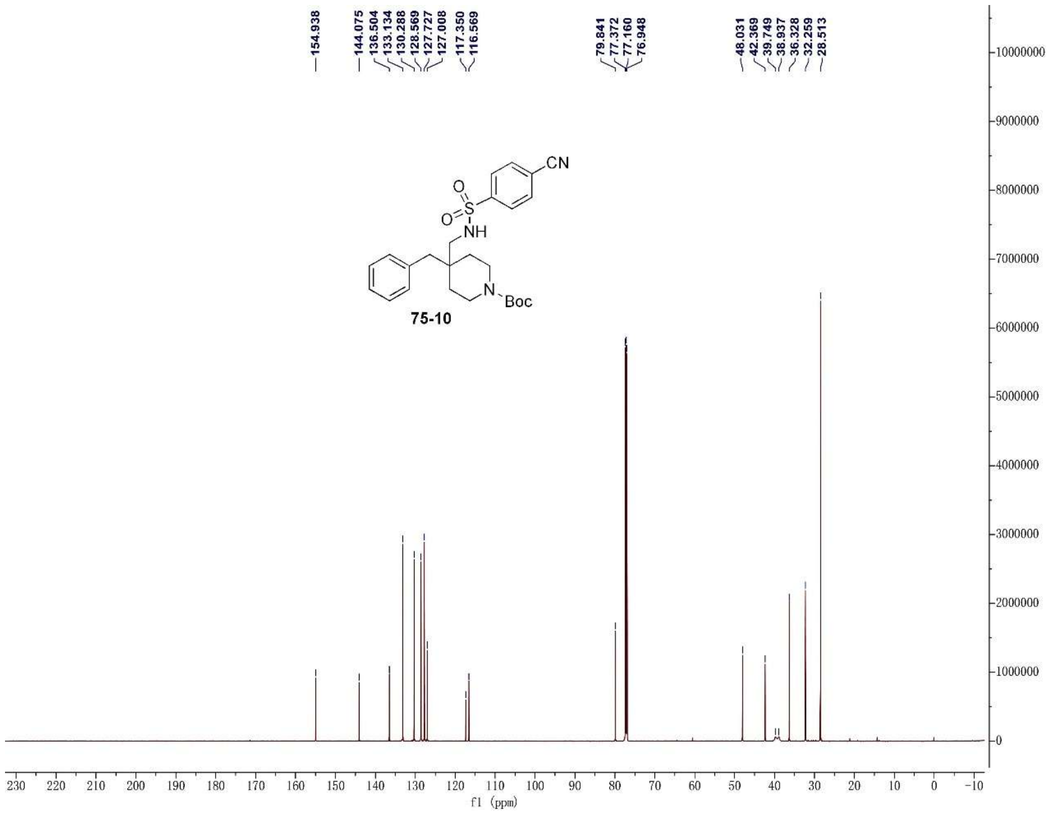

Figure S292: ^{13}C NMR spectrum of **75-10**

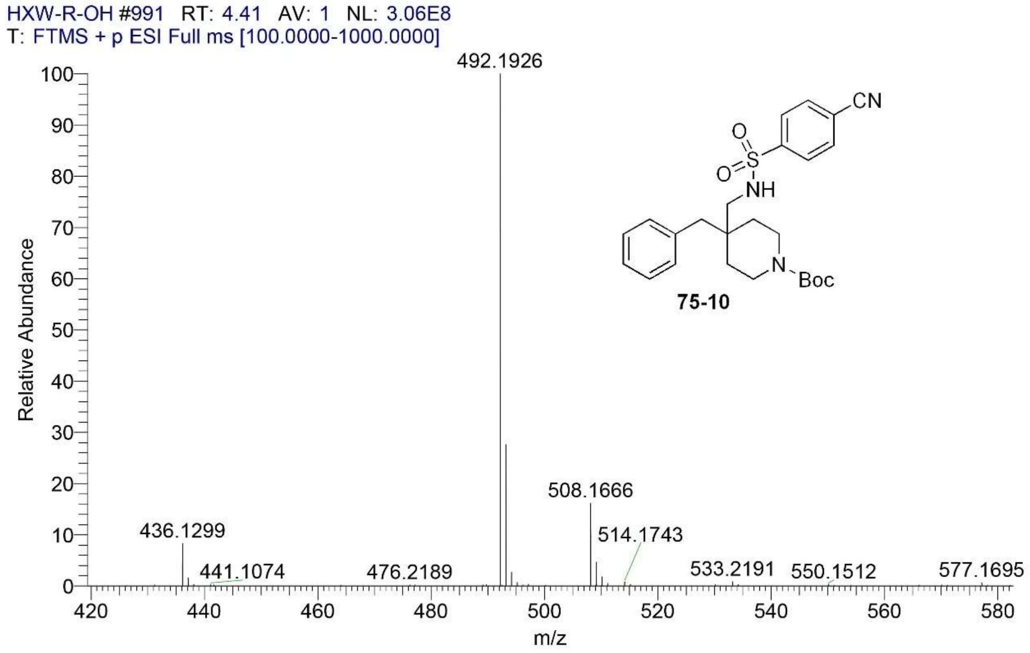

Figure S293: HR-MS (ESI/ion trap) spectrum of **75-10**

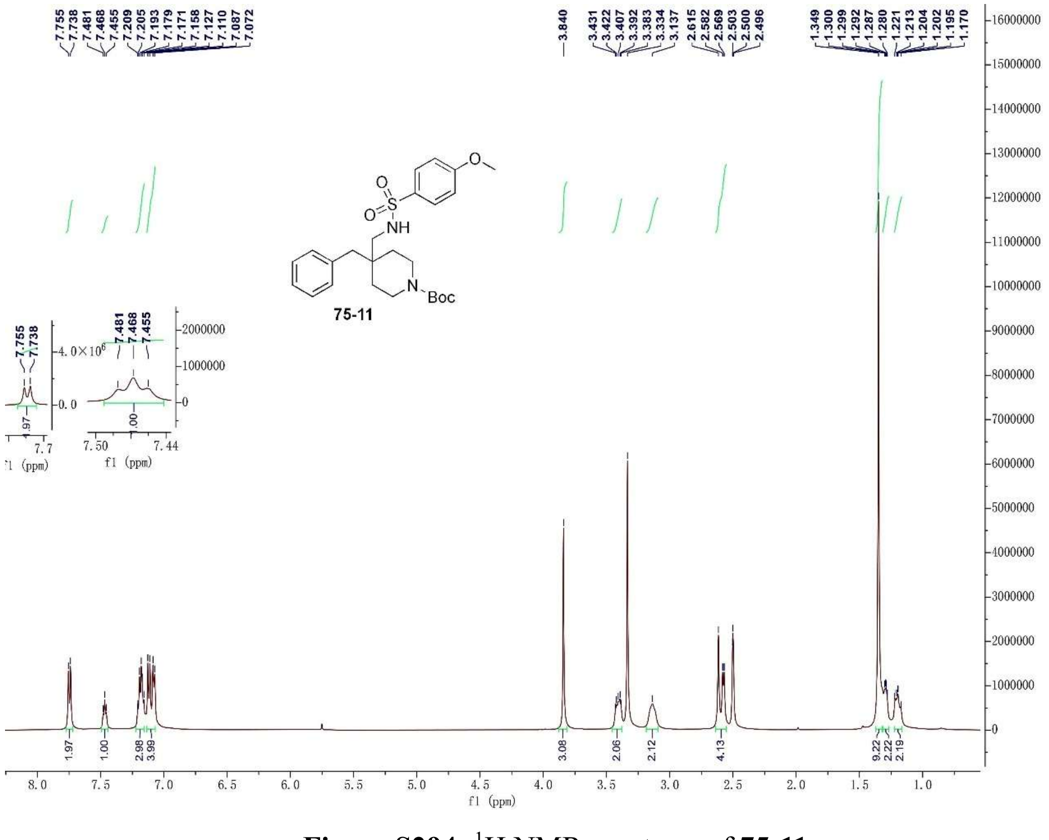

Figure S294: ^{1}H NMR spectrum of **75-11**

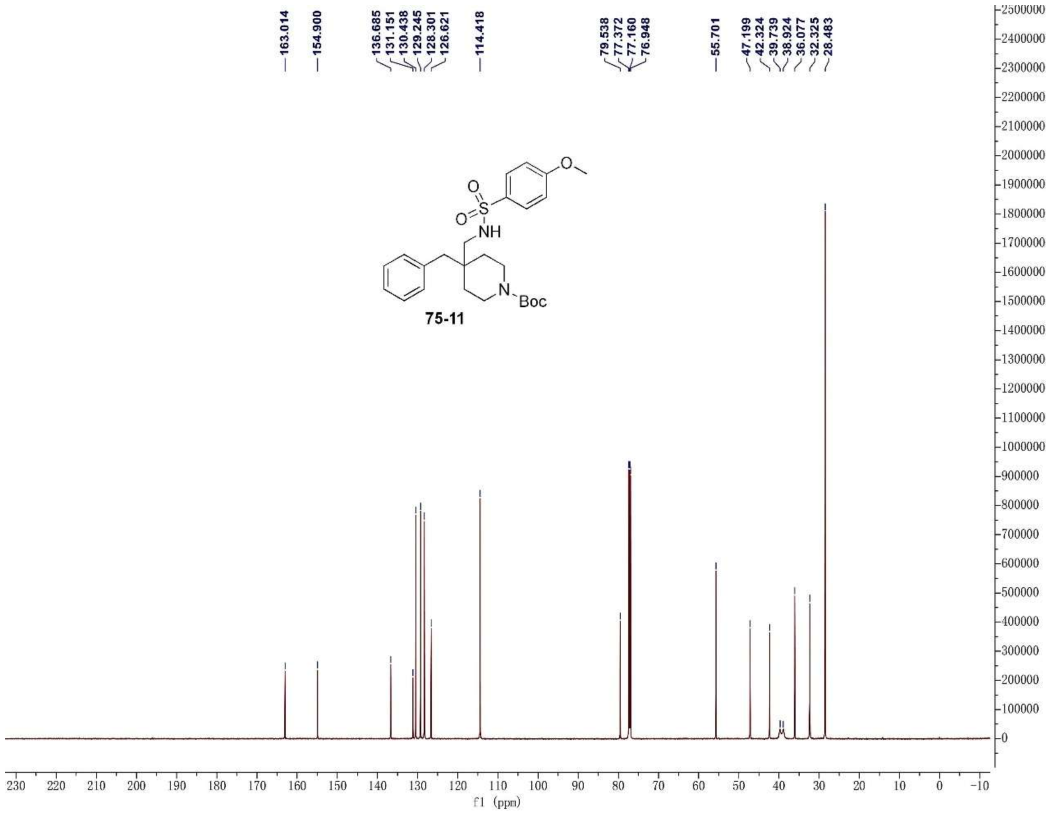

Figure S295: ^{13}C NMR spectrum of **75-11**

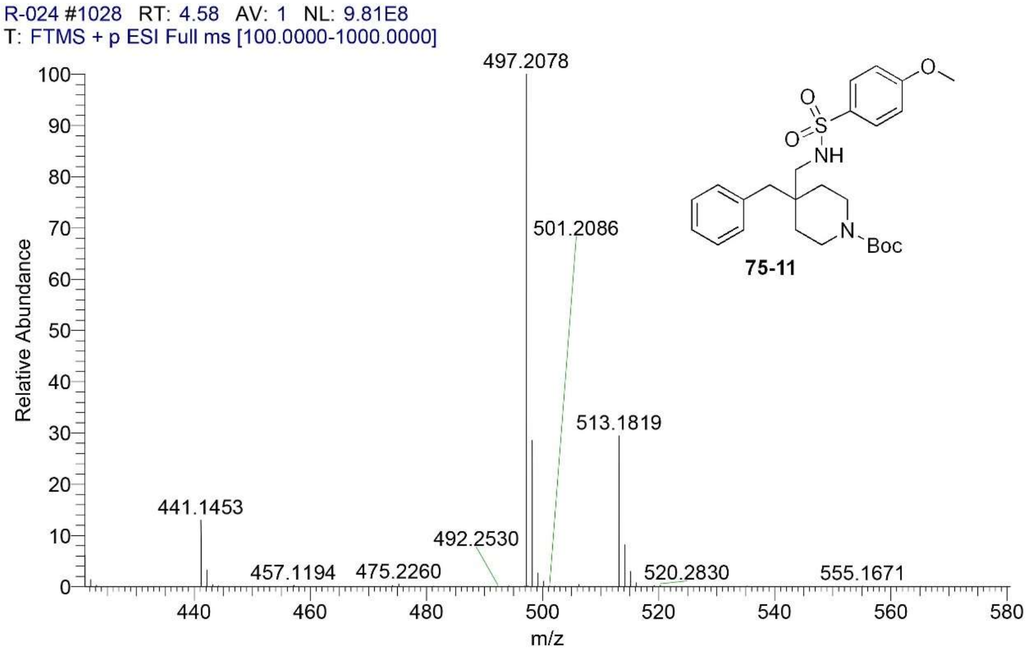

Figure S296: HR-MS (ESI/ion trap) spectrum of **75-11**

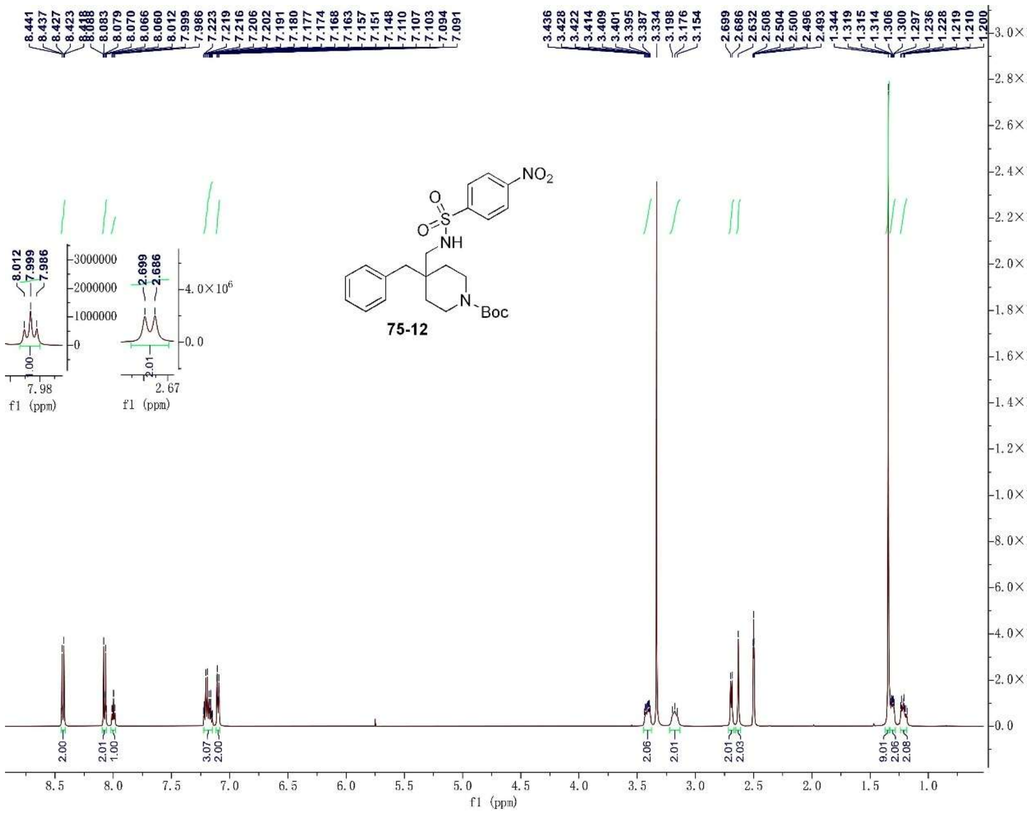

Figure S297: ^{1}H NMR spectrum of **75-12**

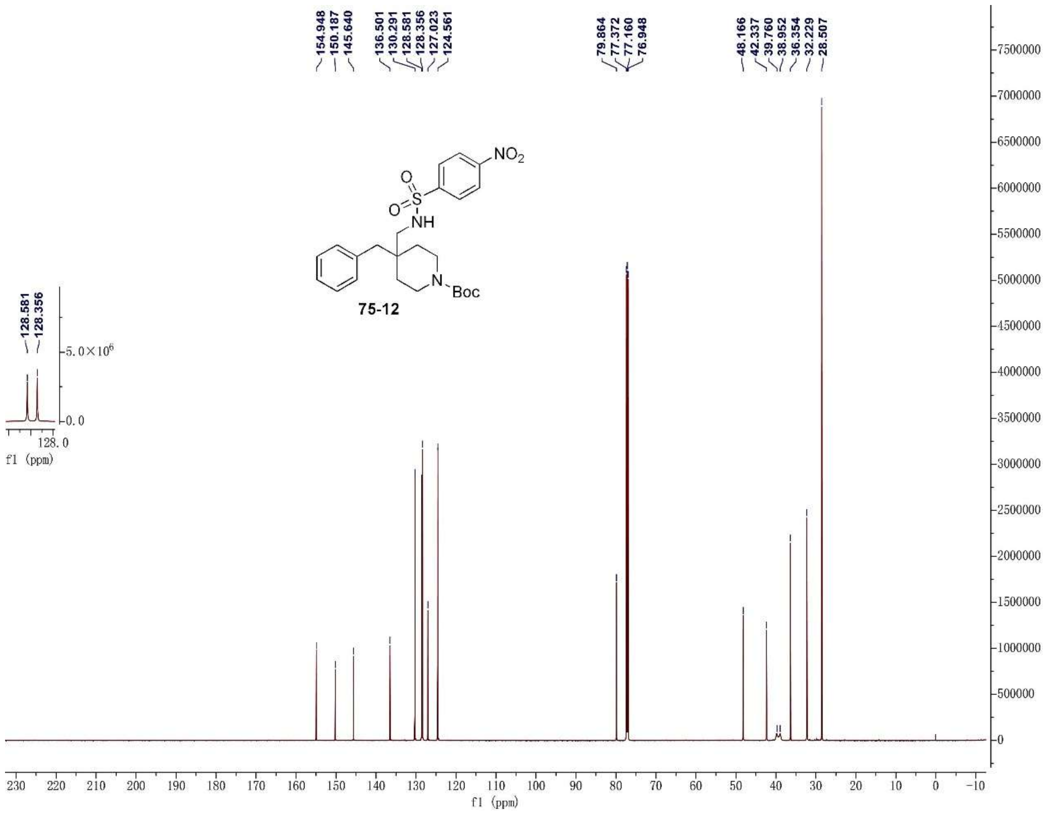

Figure S298: ^{13}C NMR spectrum of **75-12**

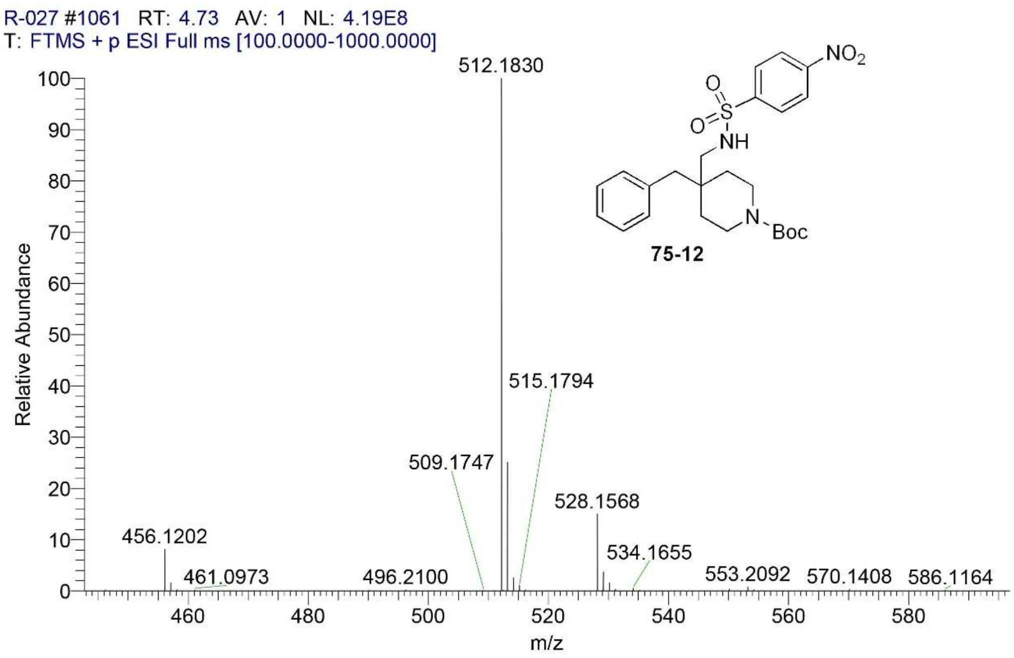

Figure S299: HR-MS (ESI/ion trap) spectrum of **75-12**

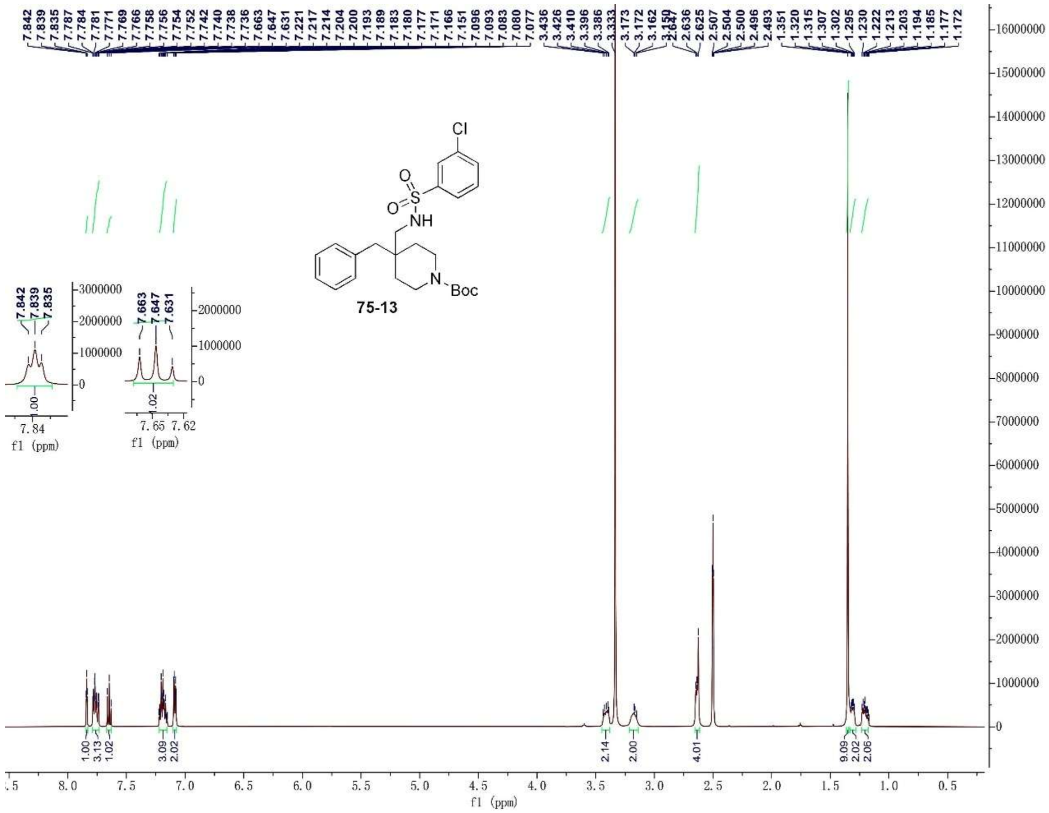

Figure S300: ^{1}H NMR spectrum of **75-13**

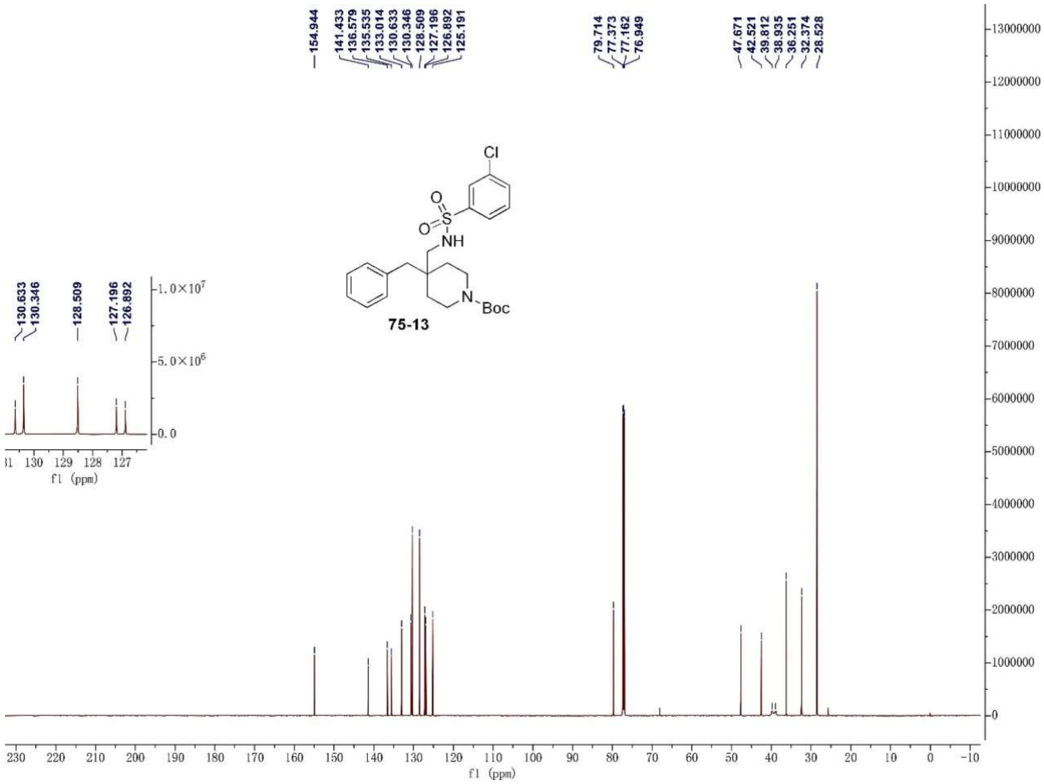

Figure S301: ^{13}C NMR spectrum of **75-13**

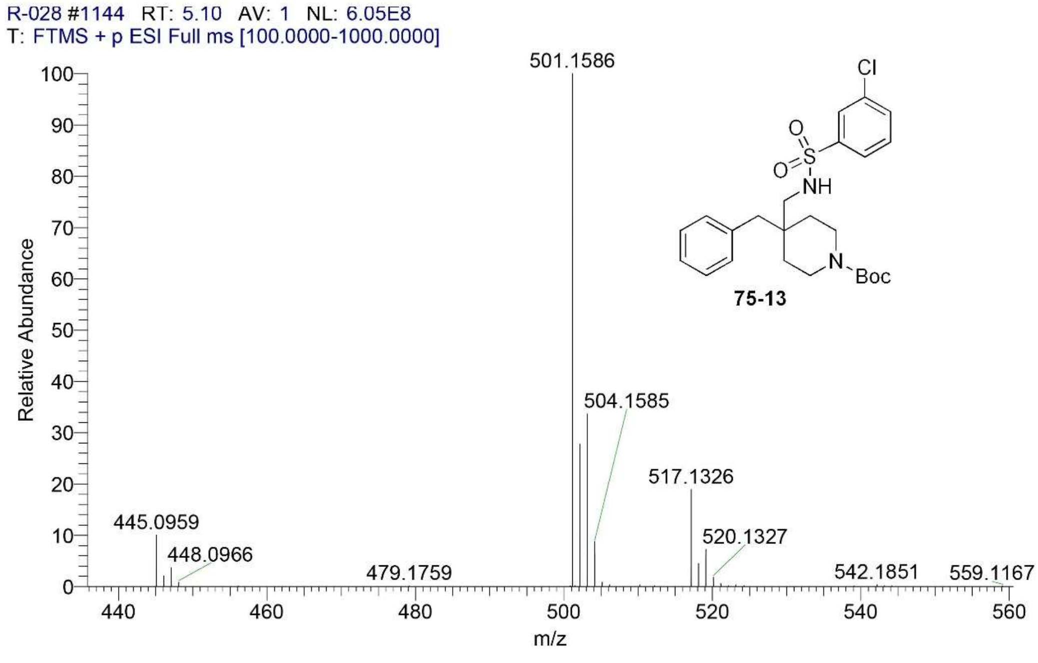

Figure S302: HR-MS (ESI/ion trap) spectrum of **75-13**

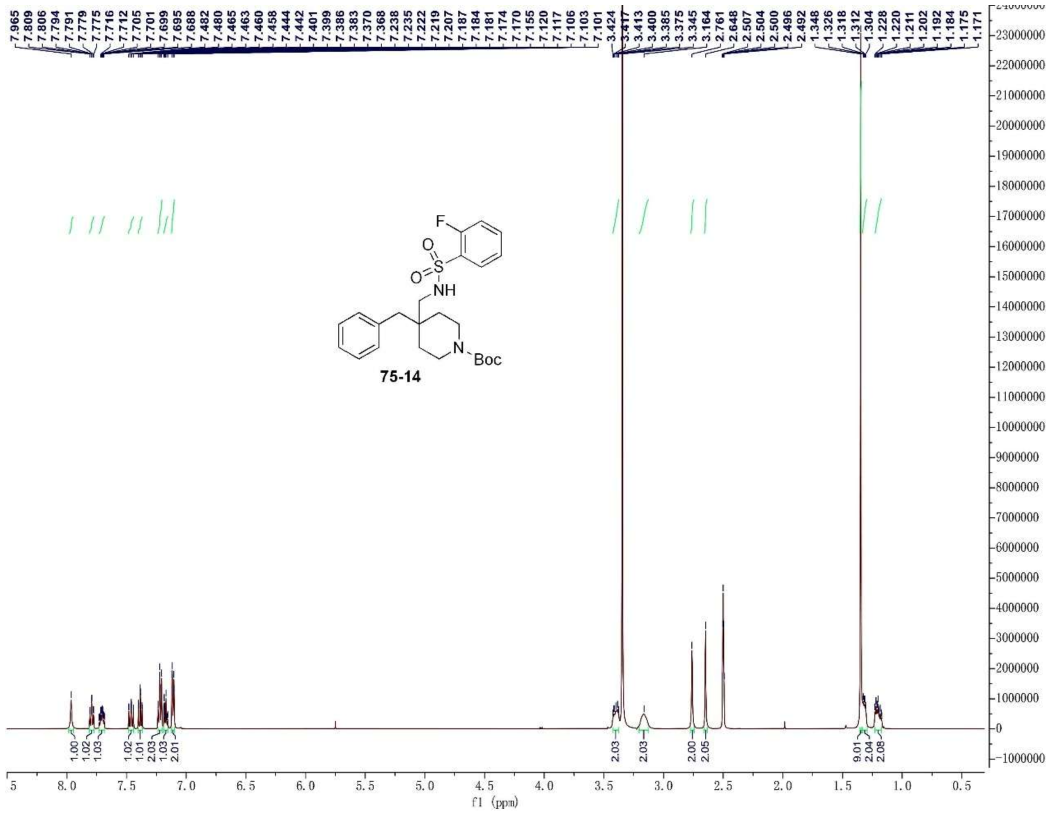

Figure S303: ^{1}H NMR spectrum of **75-14**

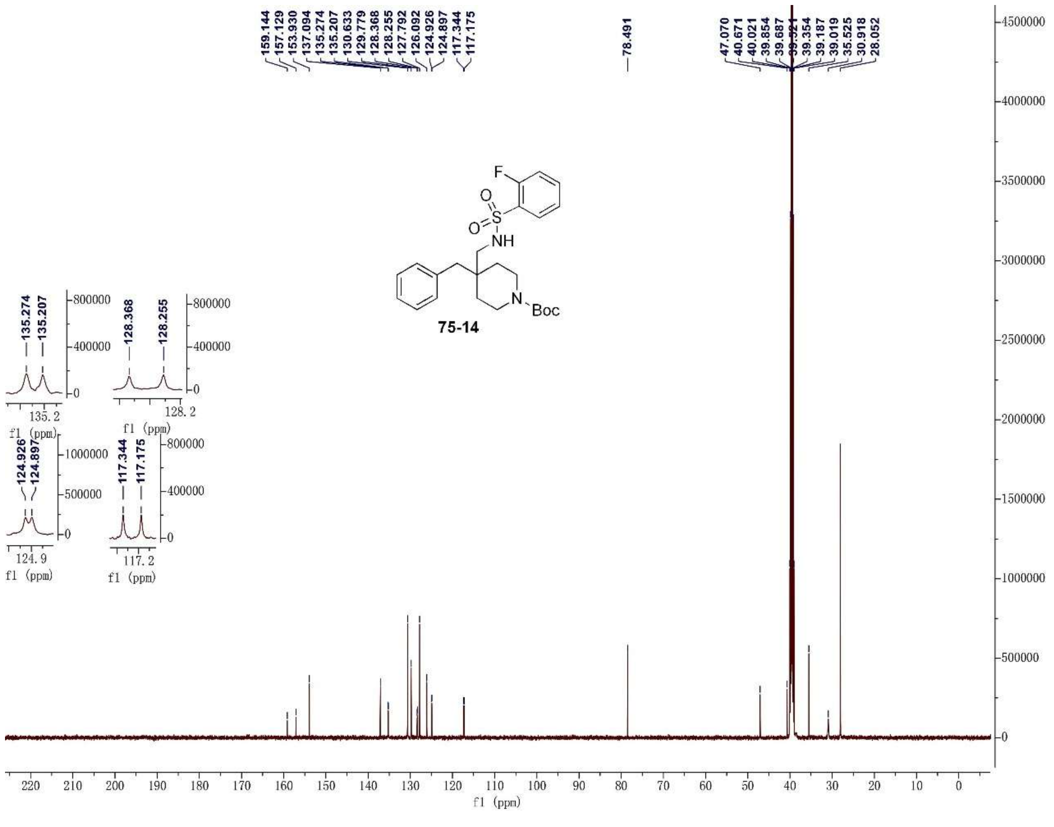

Figure S304: ^{13}C NMR spectrum of **75-14**

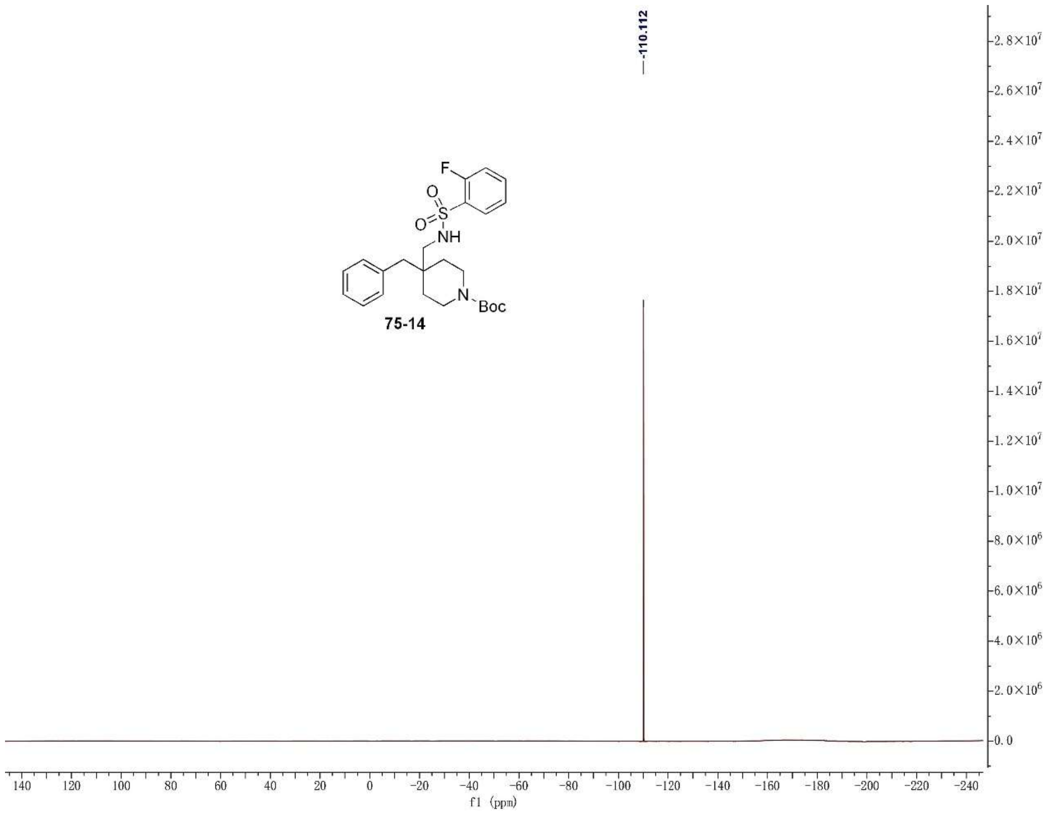

Figure S305: ^{19}F NMR spectrum of **75-14**

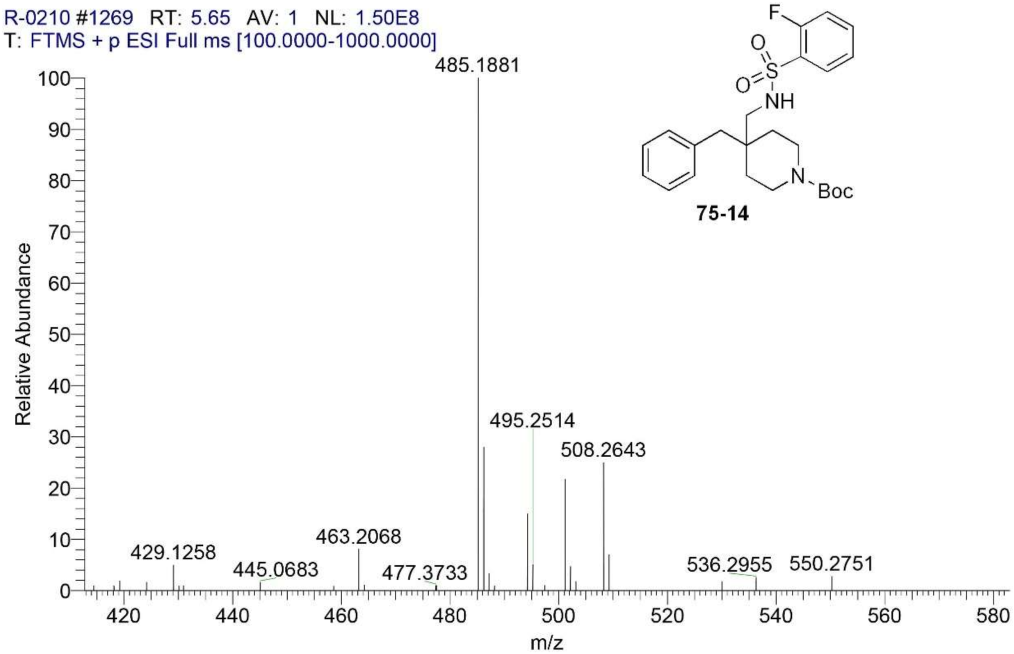

Figure S306: HR-MS (ESI/ion trap) spectrum of **75-14**

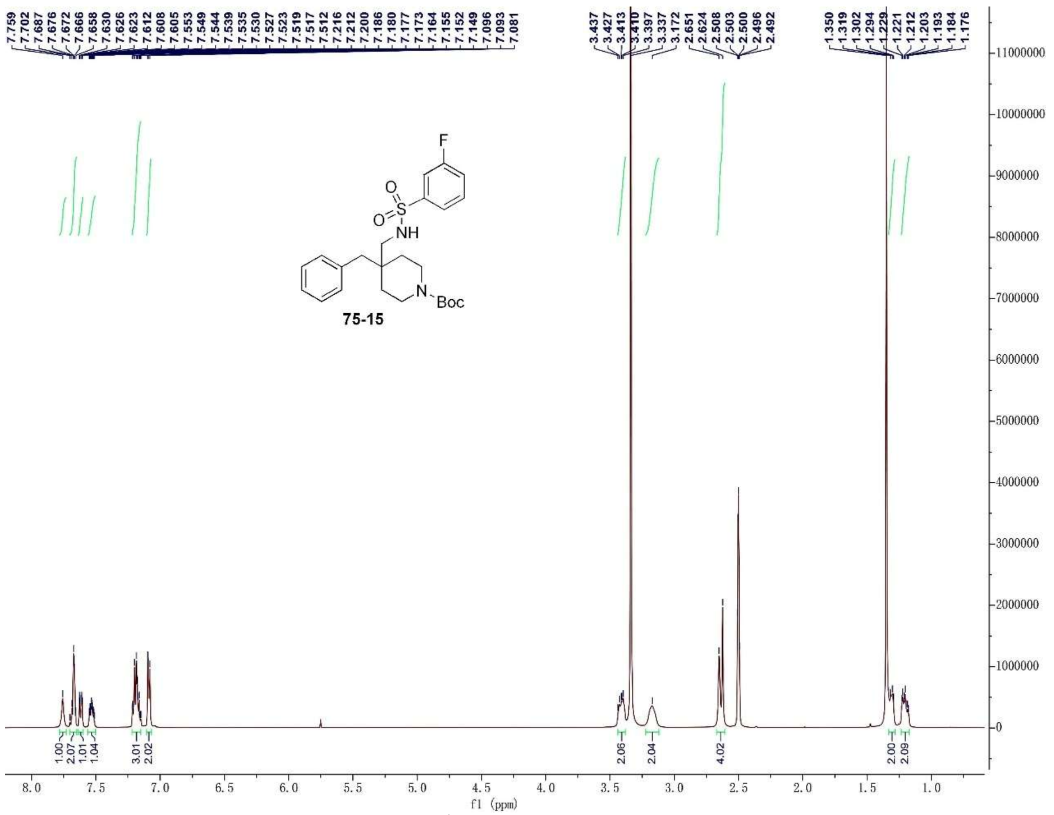

Figure S307: ^{1}H NMR spectrum of **75-15**

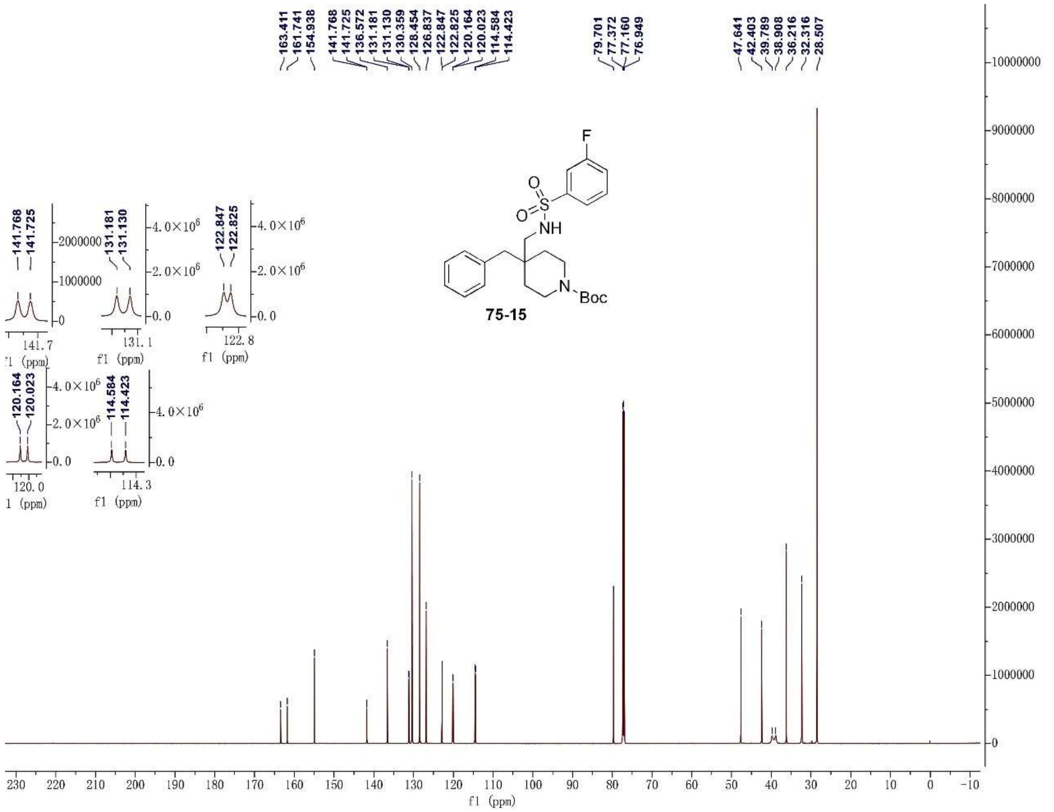

Figure S308: ^{13}C NMR spectrum of **75-15**

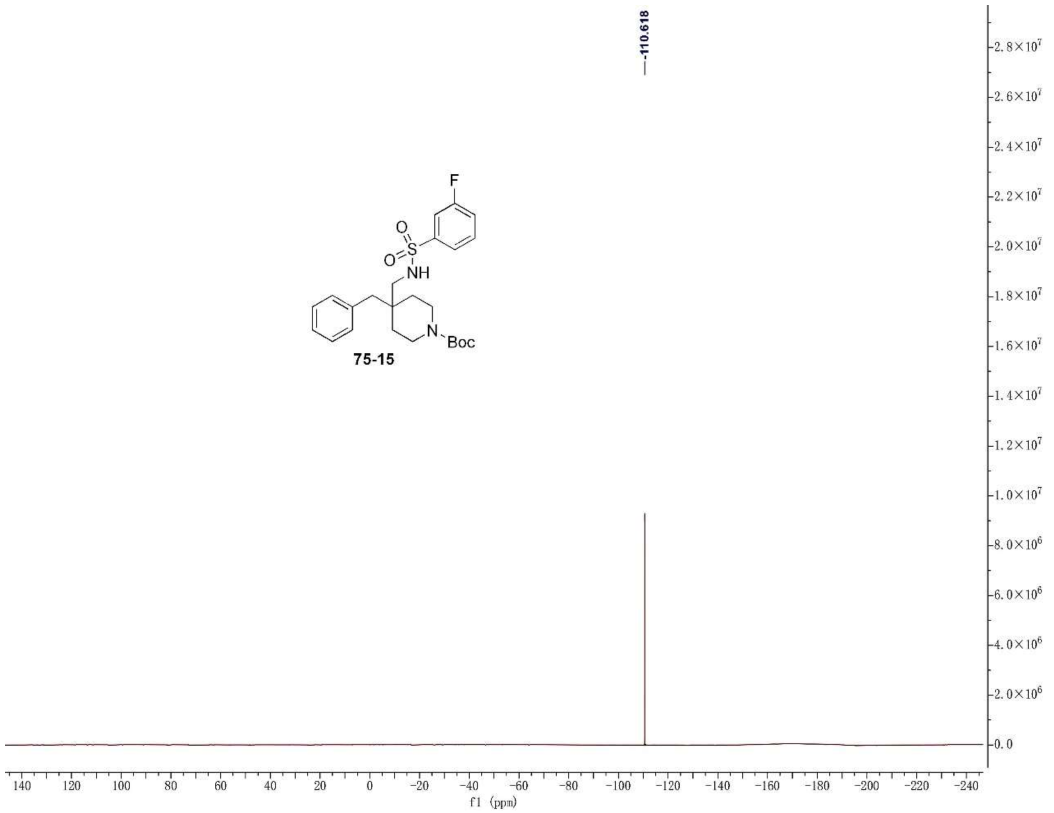

Figure S309: ^{19}F NMR spectrum of **75-15**

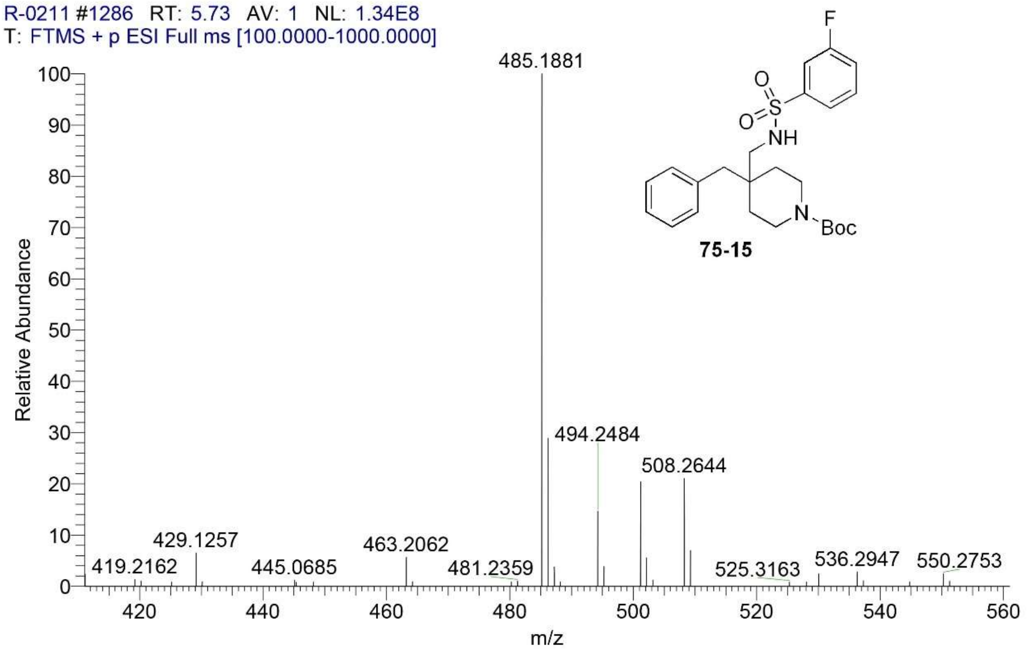

Figure S310: HR-MS (ESI/ion trap) spectrum of **75-15**

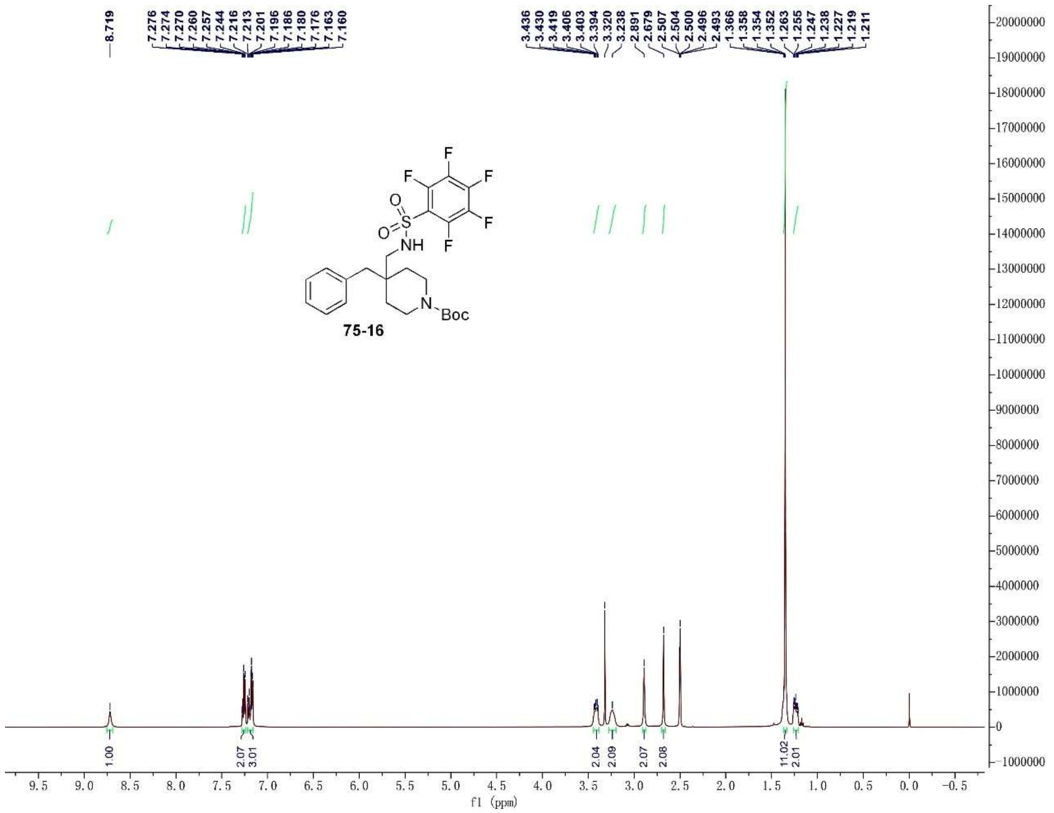

Figure S311: ^{1}H NMR spectrum of **75-16**

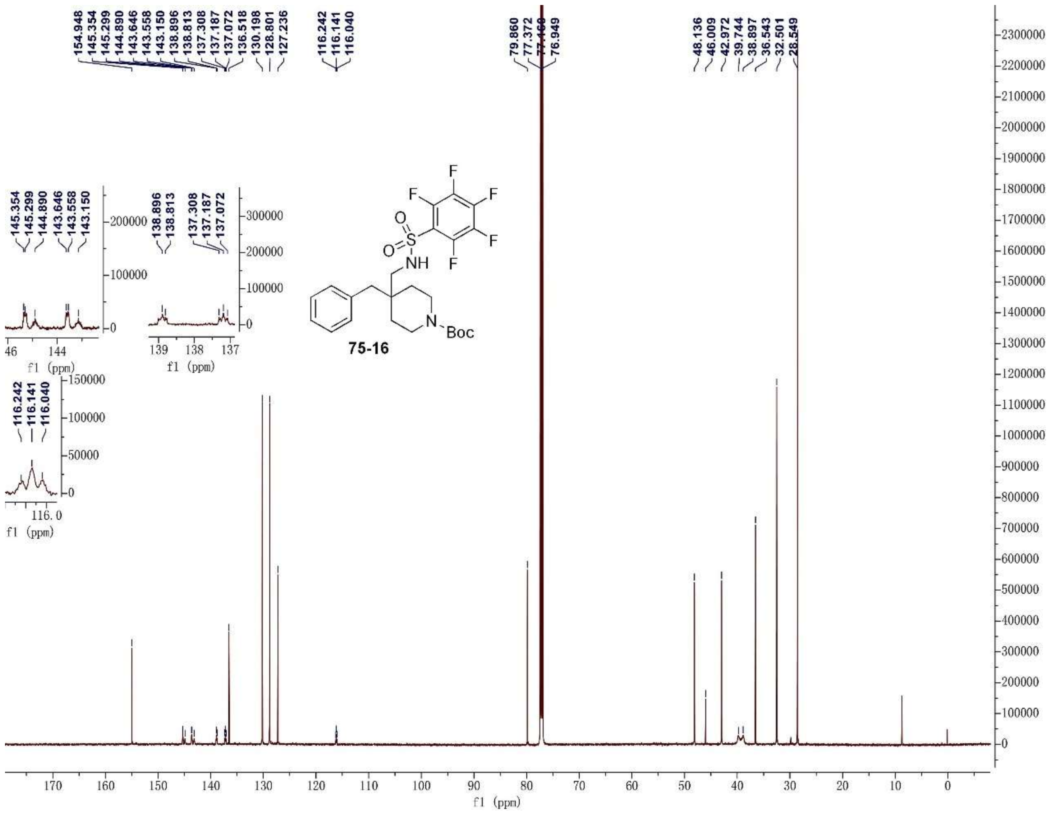

Figure S312: ^{13}C NMR spectrum of **75-16**

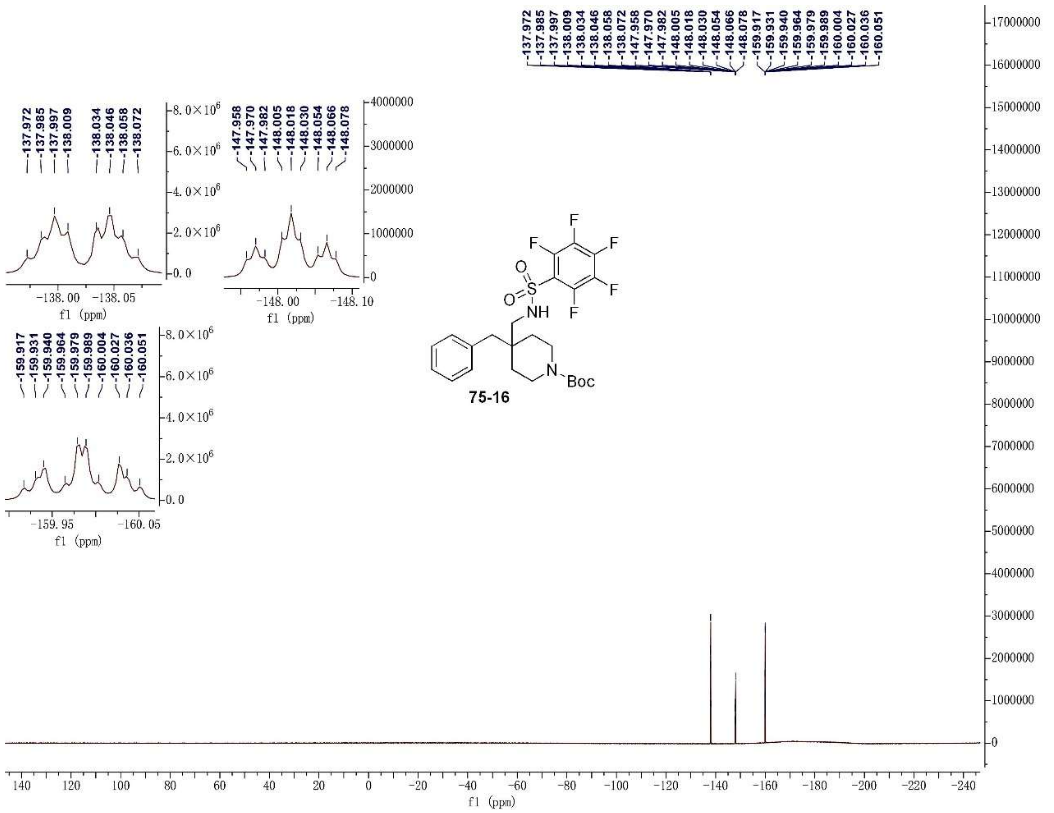

Figure S313: ^{19}F NMR spectrum of **75-16**

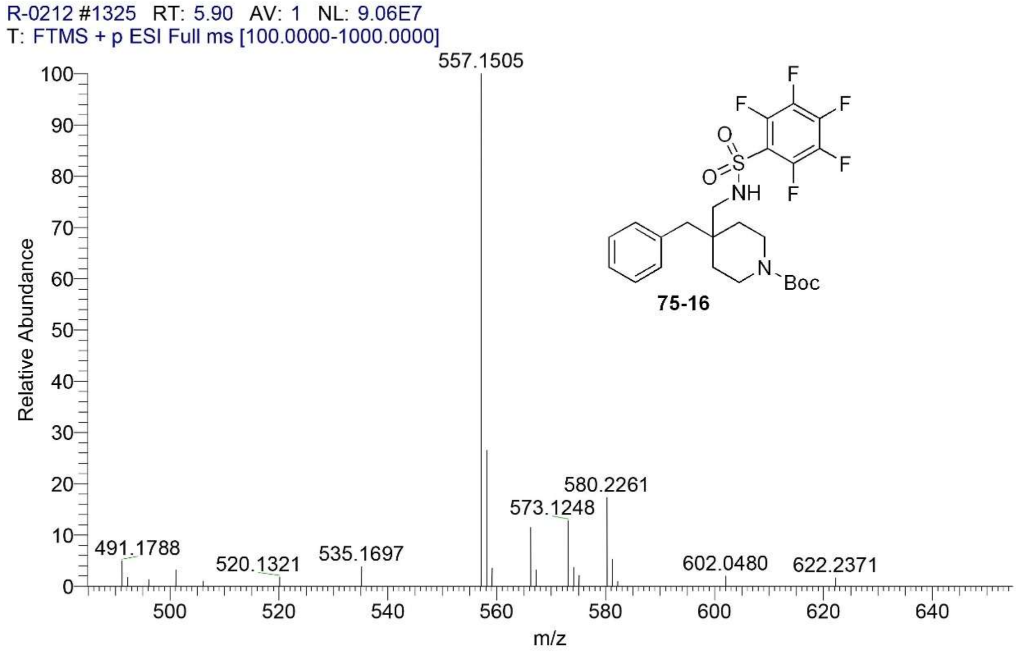

Figure S314: HR-MS (ESI/ion trap) spectrum of **75-16**

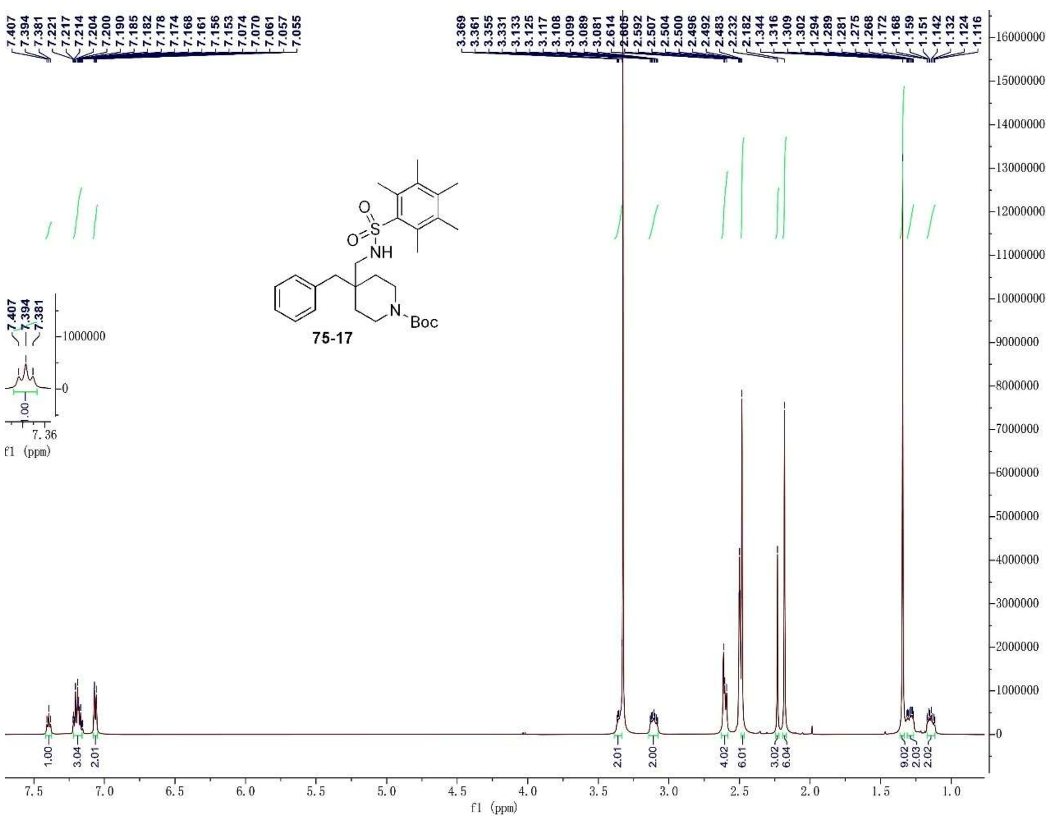

Figure S315: ^{1}H NMR spectrum of **75-17**

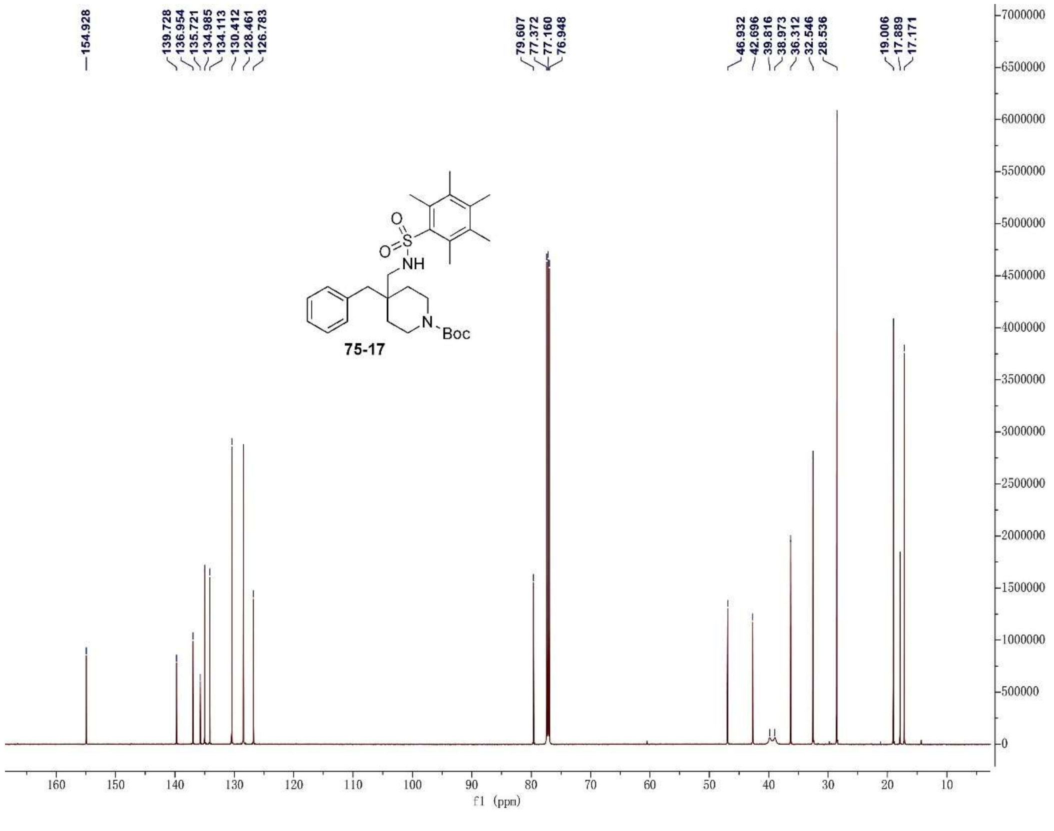

Figure S316: ^{13}C NMR spectrum of **75-17**

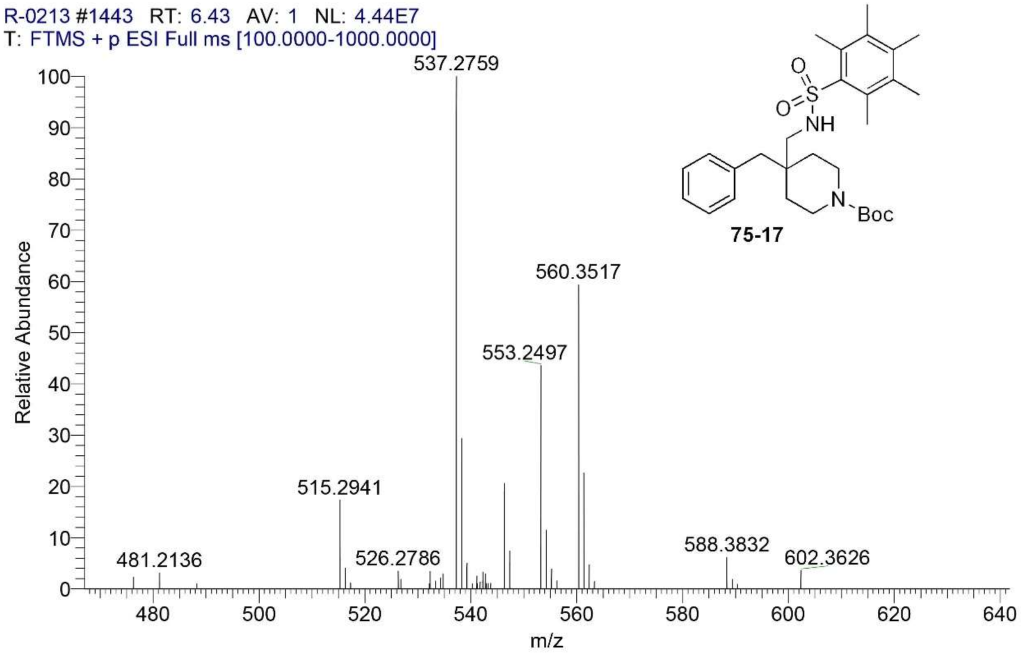

Figure S317: HR-MS (ESI/ion trap) spectrum of **75-17**

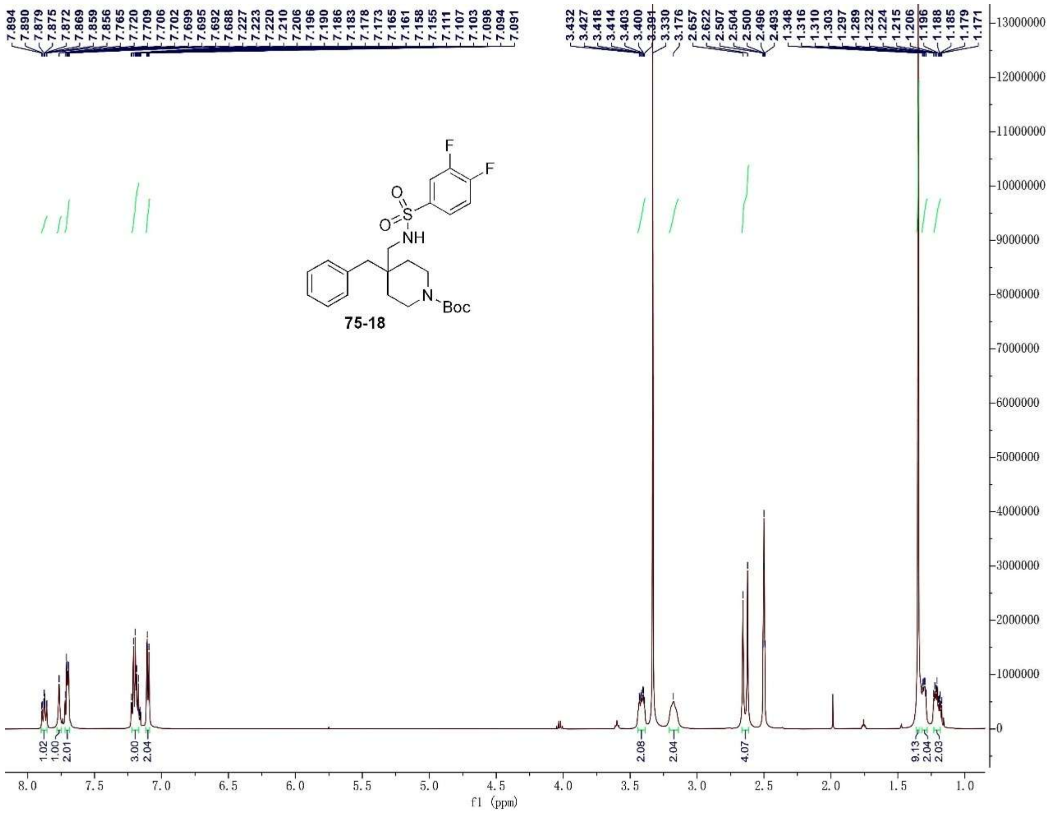

Figure S318: ^{1}H NMR spectrum of **75-18**

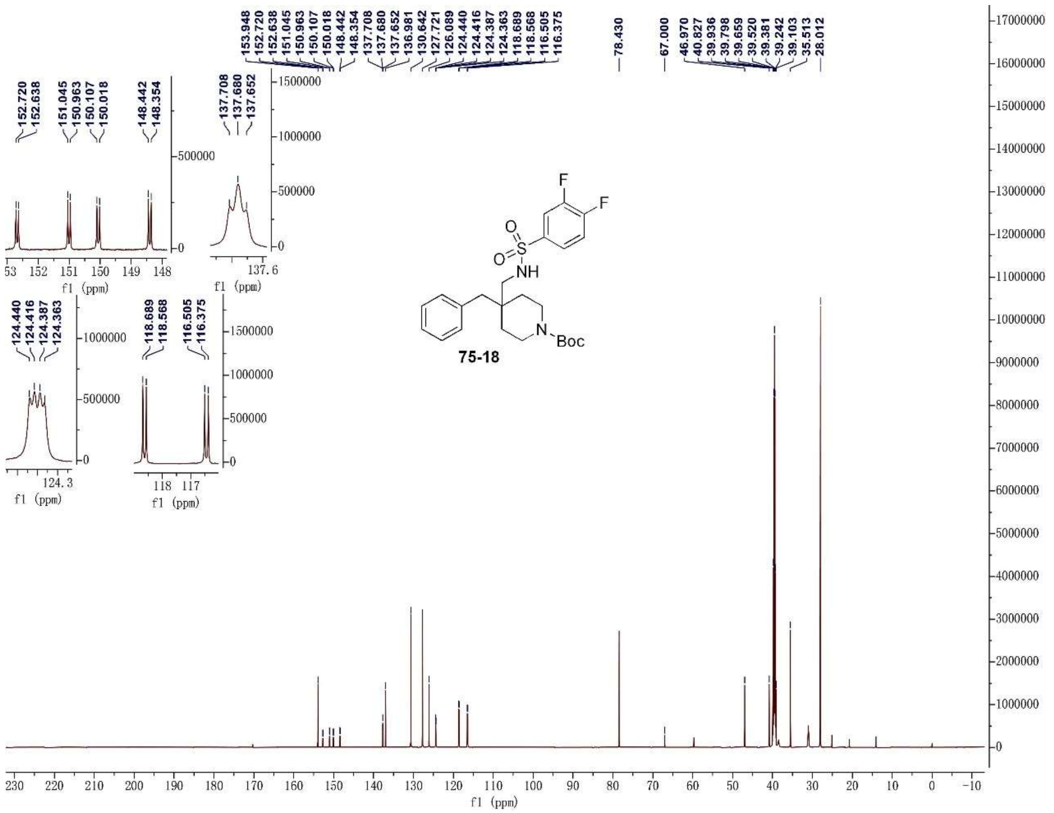

Figure S319: ^{13}C NMR spectrum of **75-18**

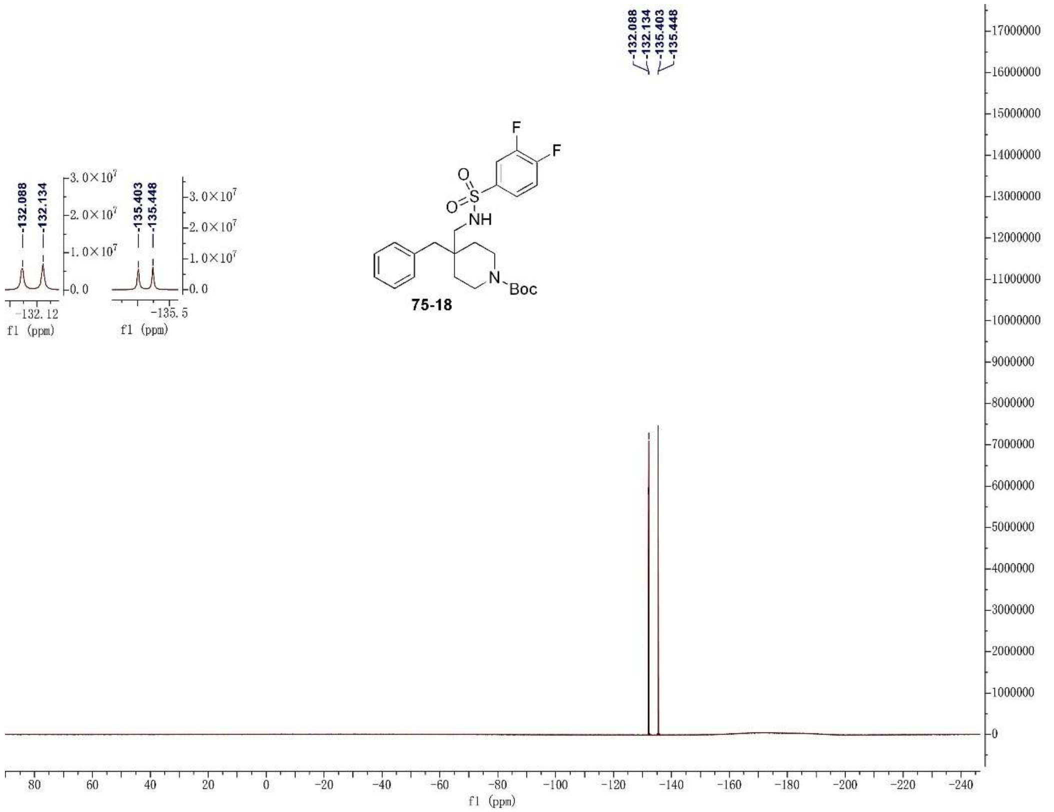

Figure S320: ^{19}F NMR spectrum of **75-18**

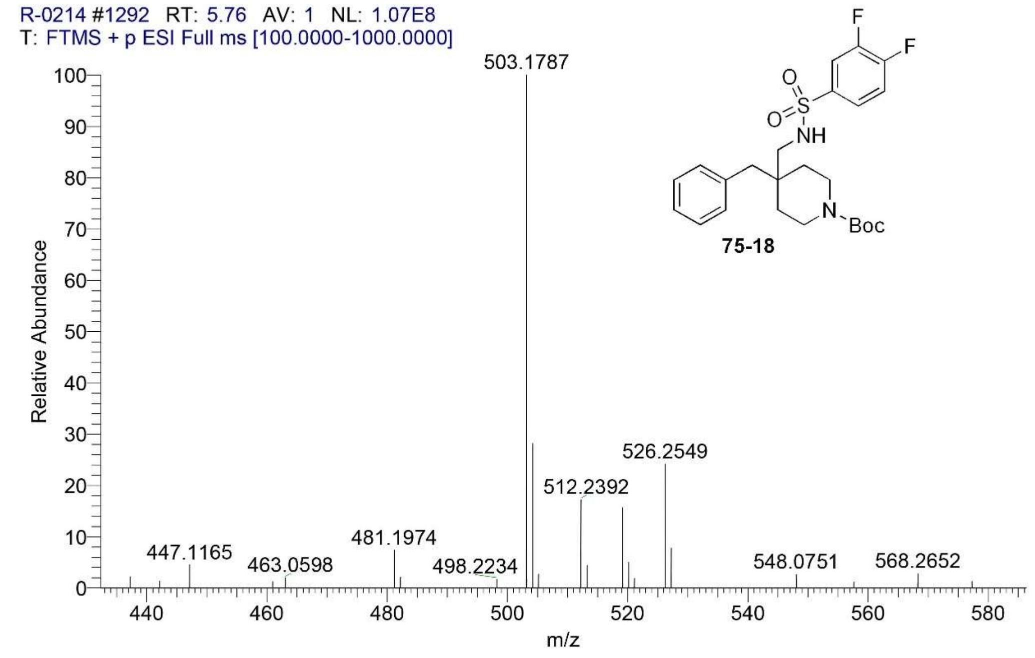

Figure S321: HR-MS (ESI/ion trap) spectrum of **75-18**

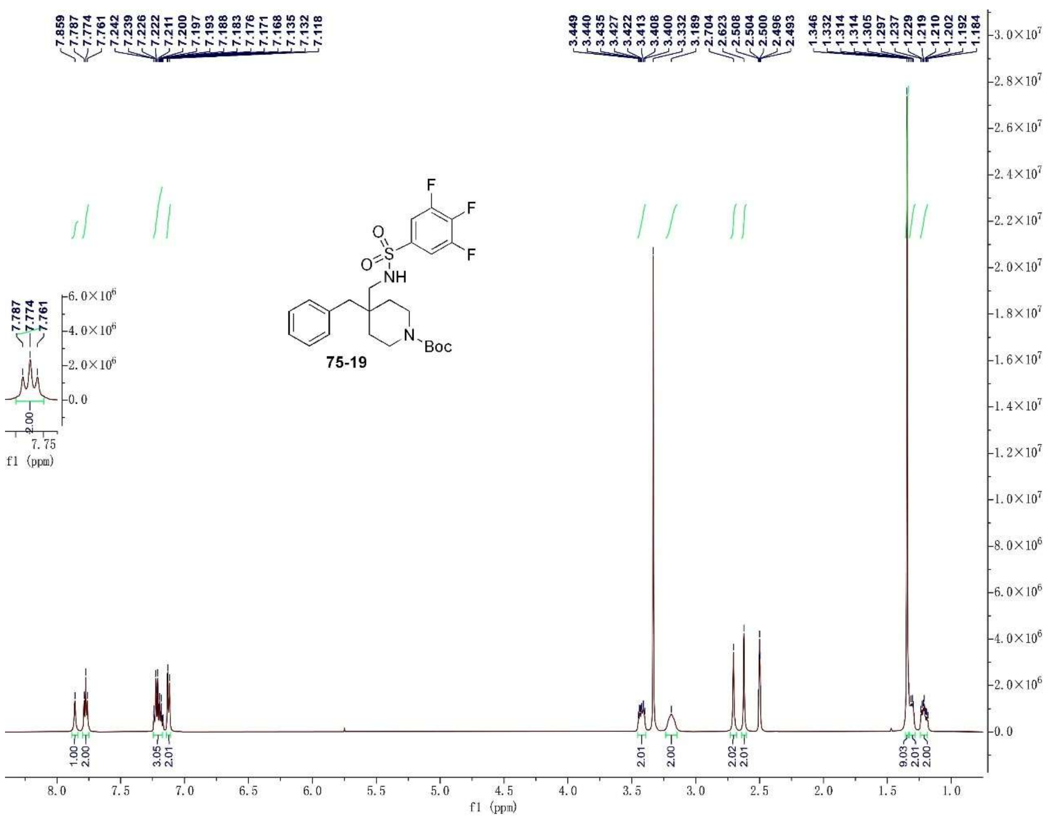

Figure S322: ^{1}H NMR spectrum of **75-19**

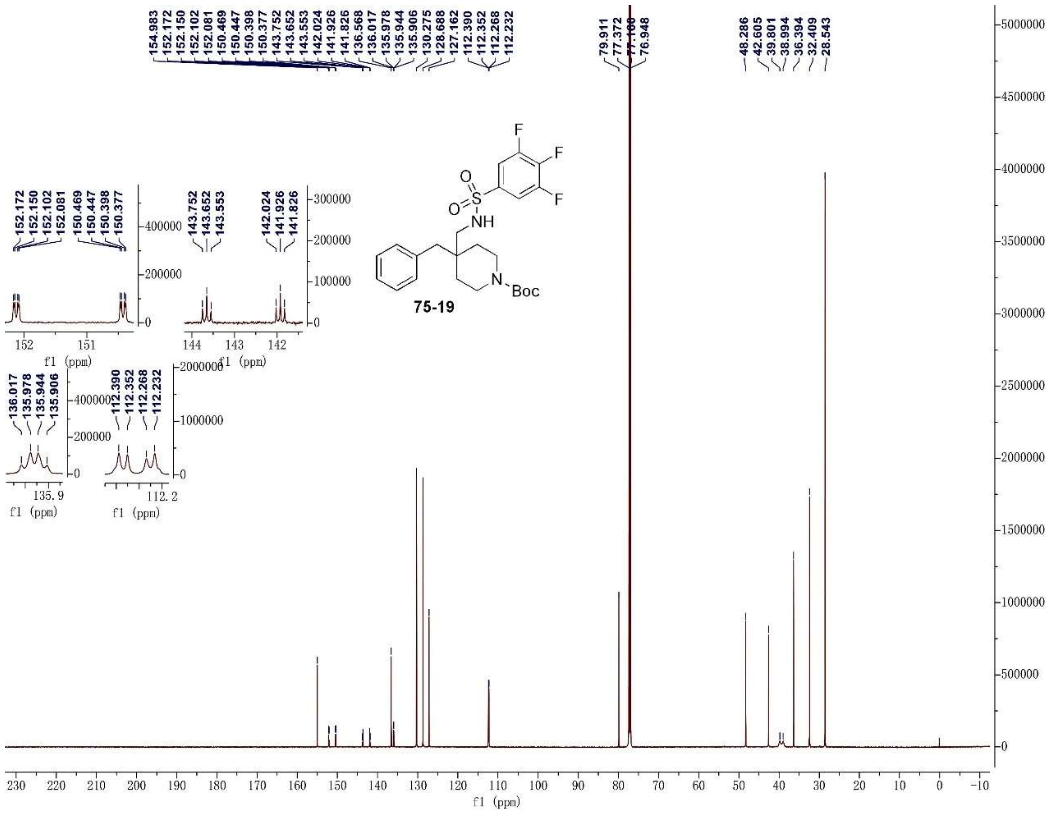

Figure S323: ^{13}C NMR spectrum of **75-19**

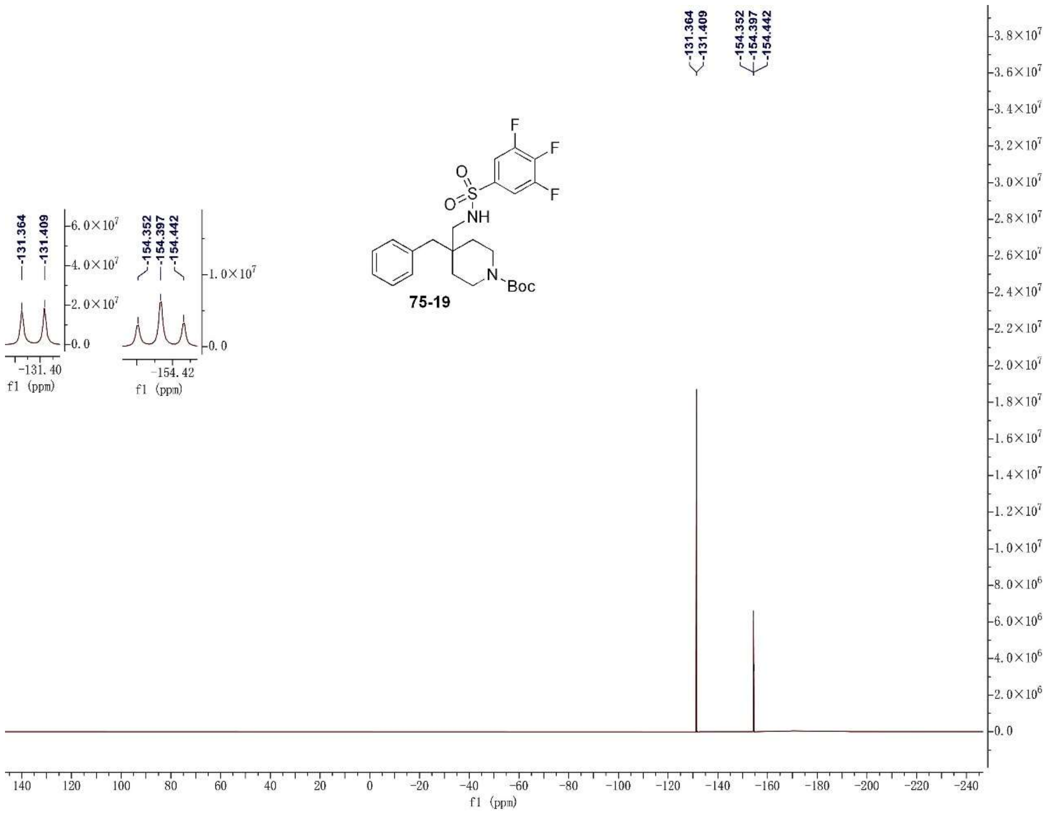

Figure S324: ^{19}F NMR spectrum of **75-19**

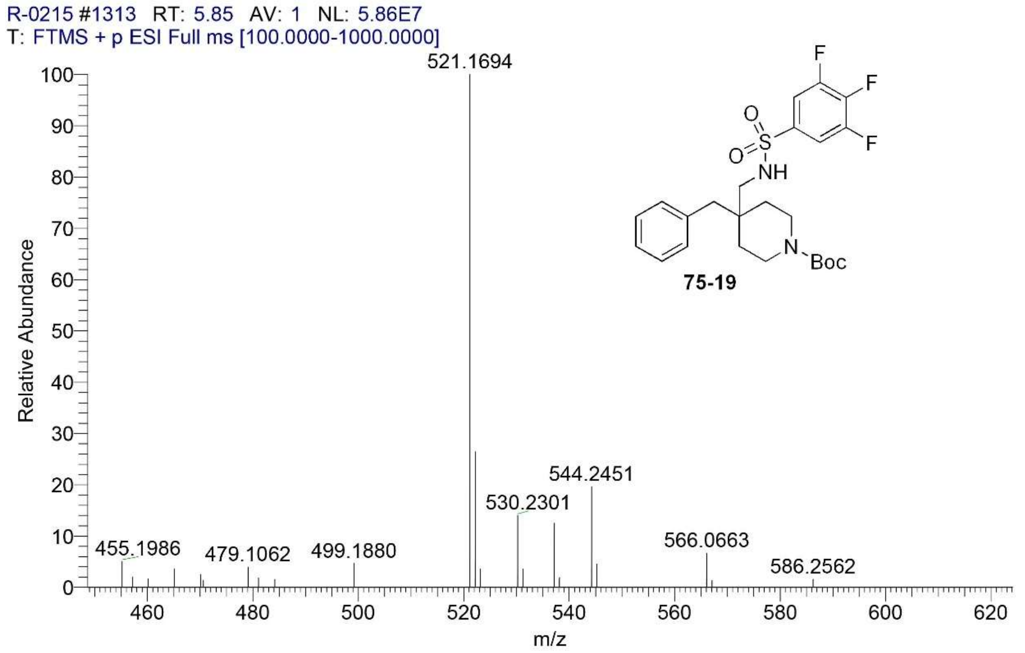

Figure S325: HR-MS (ESI/ion trap) spectrum of **75-19**

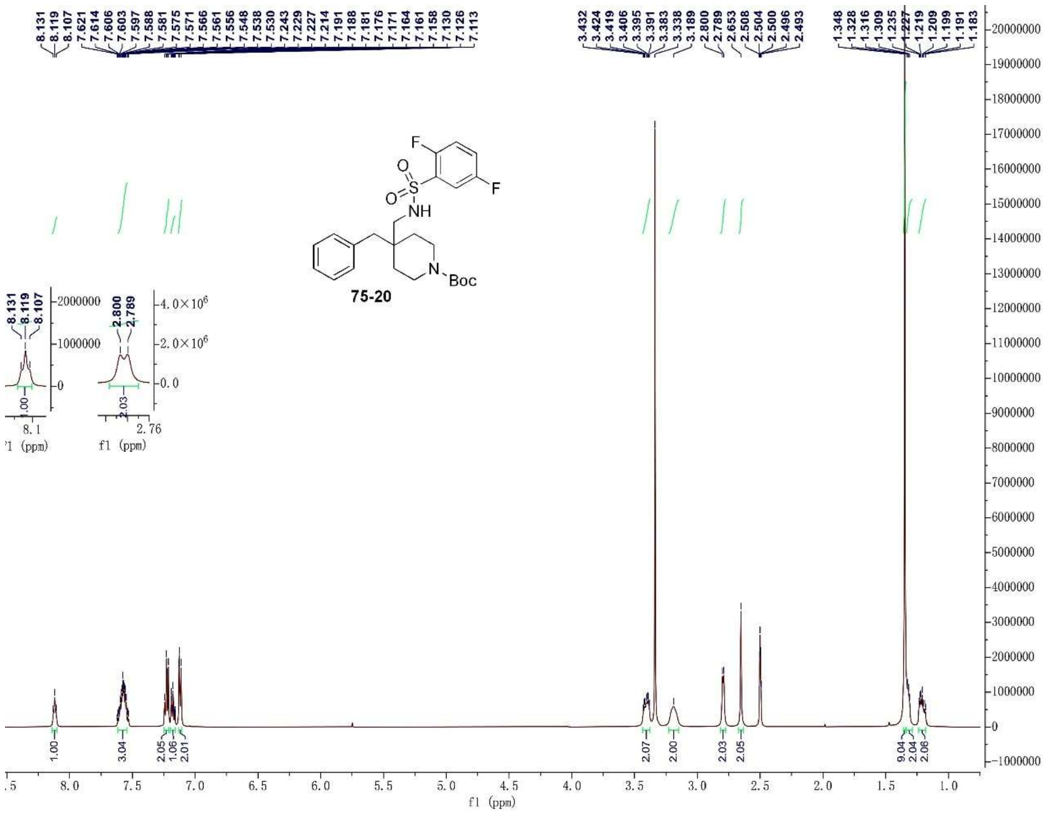

Figure S326: ^{1}H NMR spectrum of **75-20**

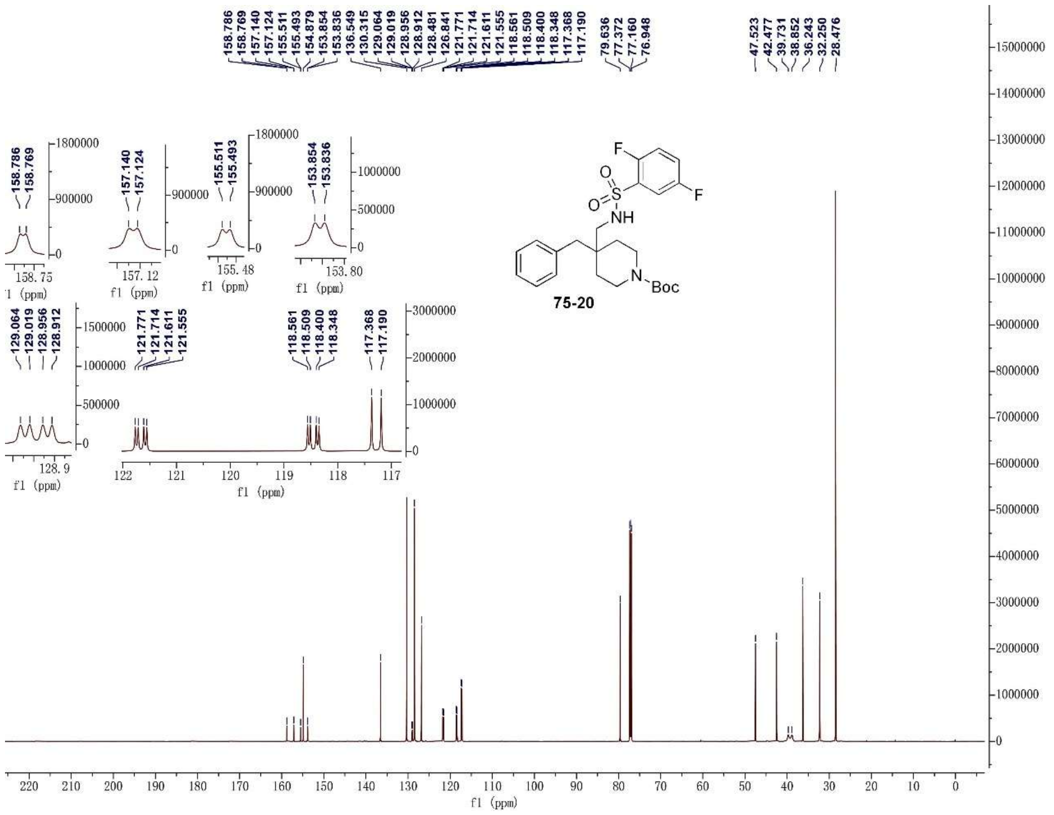

Figure S327: ^{13}C NMR spectrum of **75-20**

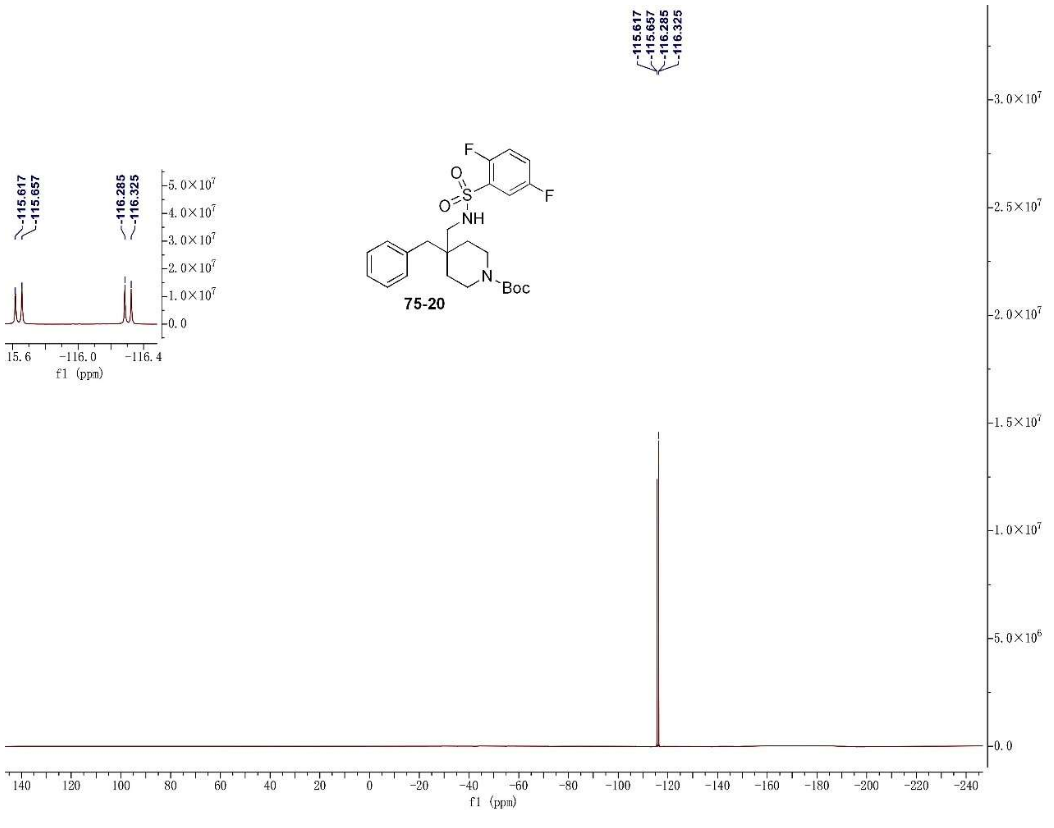

Figure S328: ^{19}F NMR spectrum of **75-20**

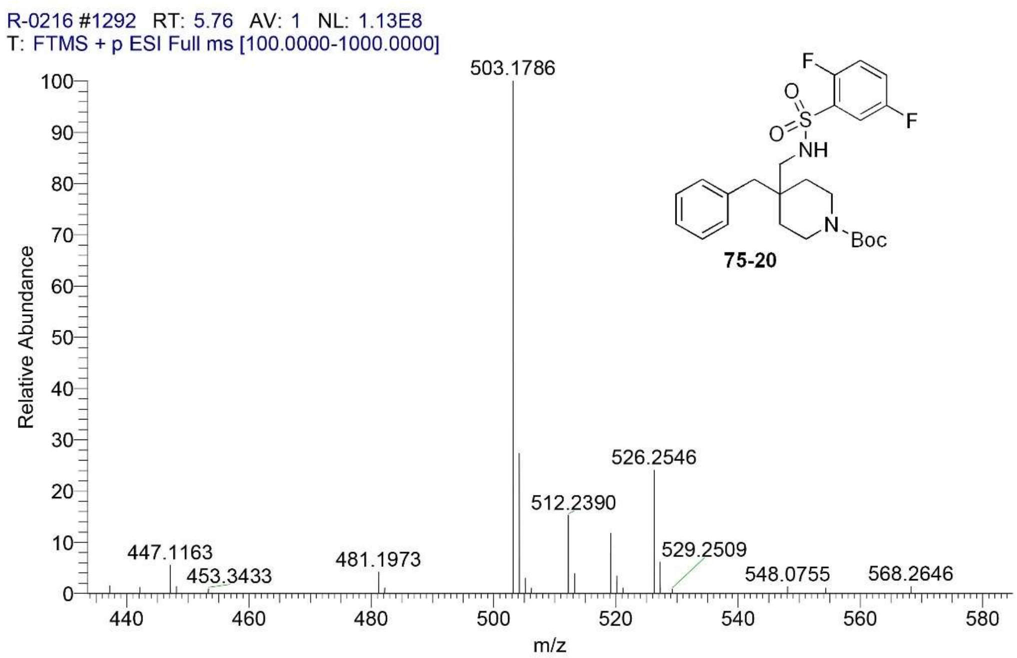

Figure S329: HR-MS (ESI/ion trap) spectrum of **75-20**

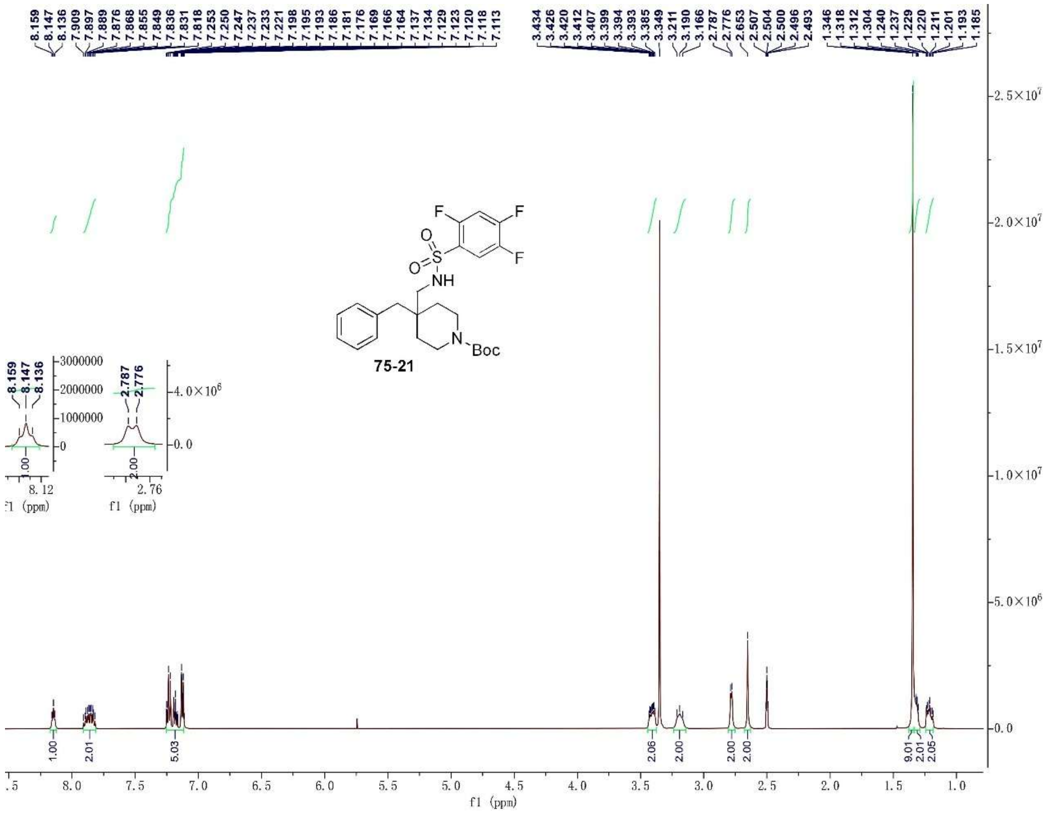

Figure S330: ^{1}H NMR spectrum of **75-21**

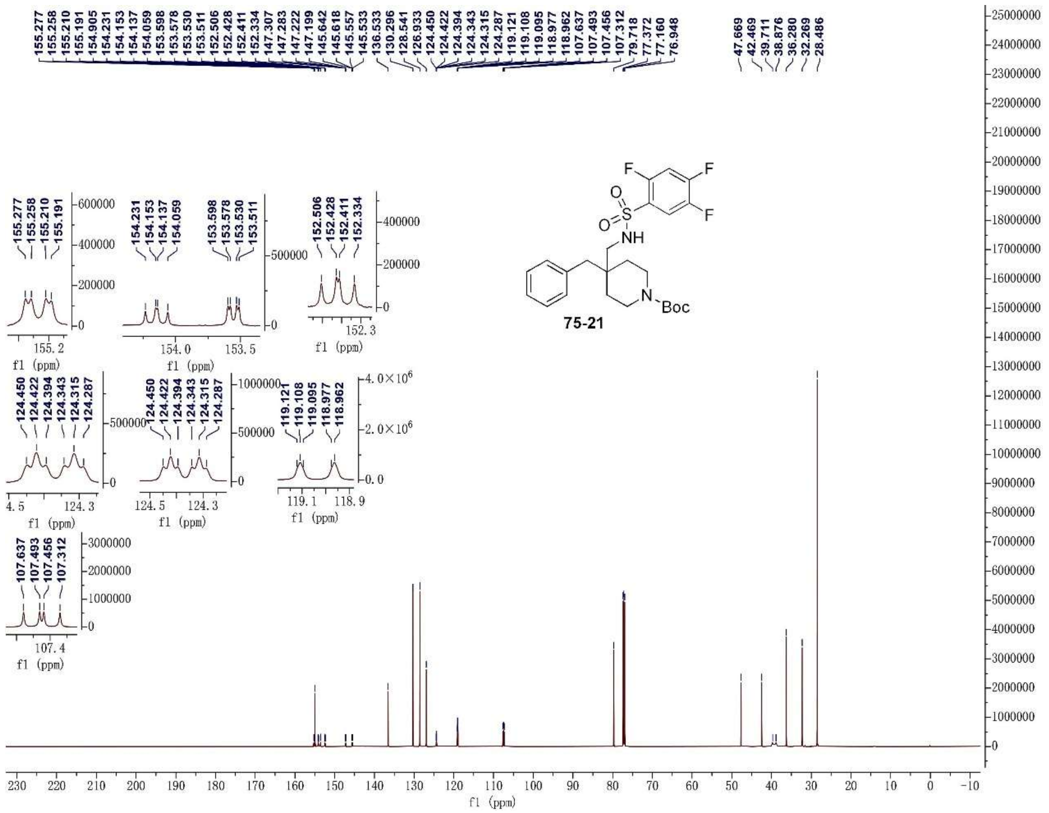

Figure S331: ^{13}C NMR spectrum of **75-21**

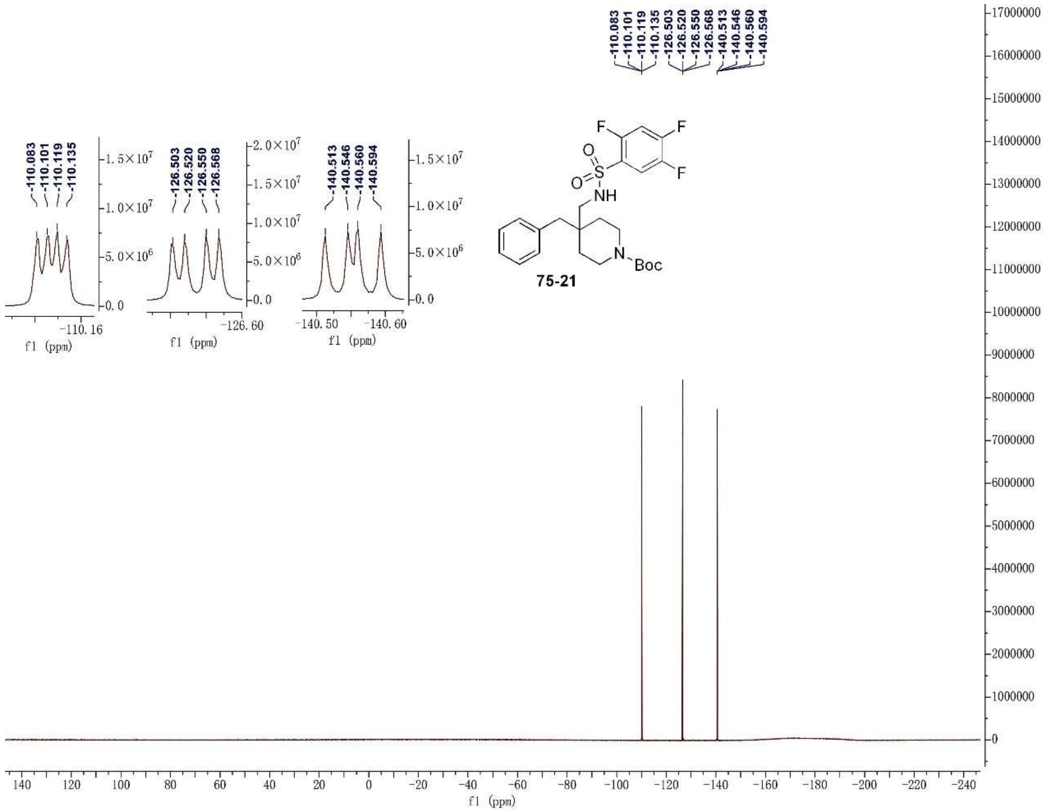

Figure S332: ^{19}F NMR spectrum of **75-21**

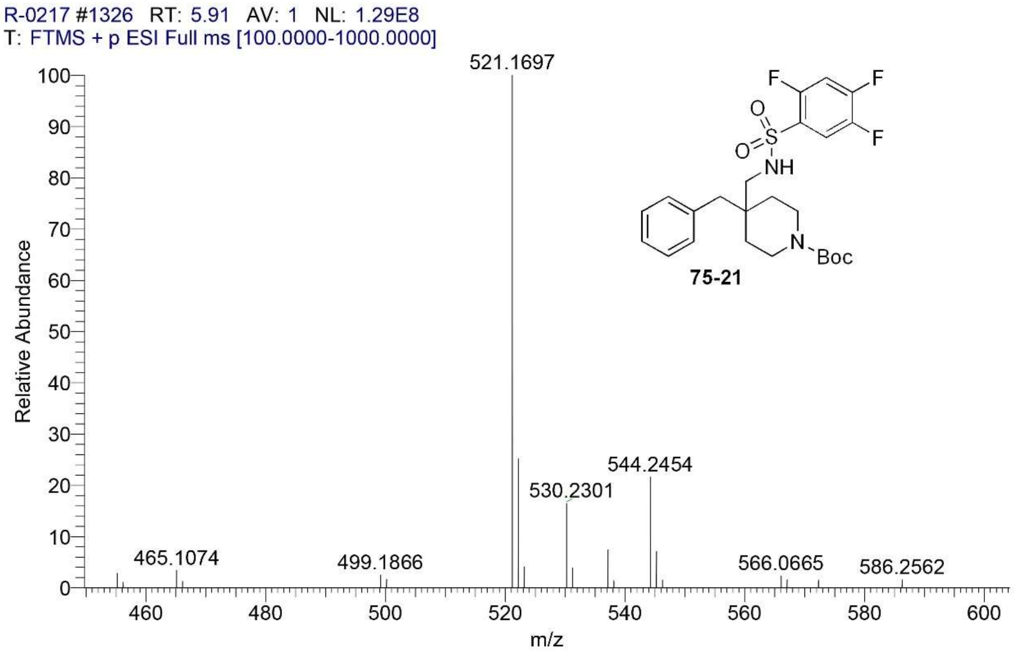

Figure S333: HR-MS (ESI/ion trap) spectrum of **75-21**

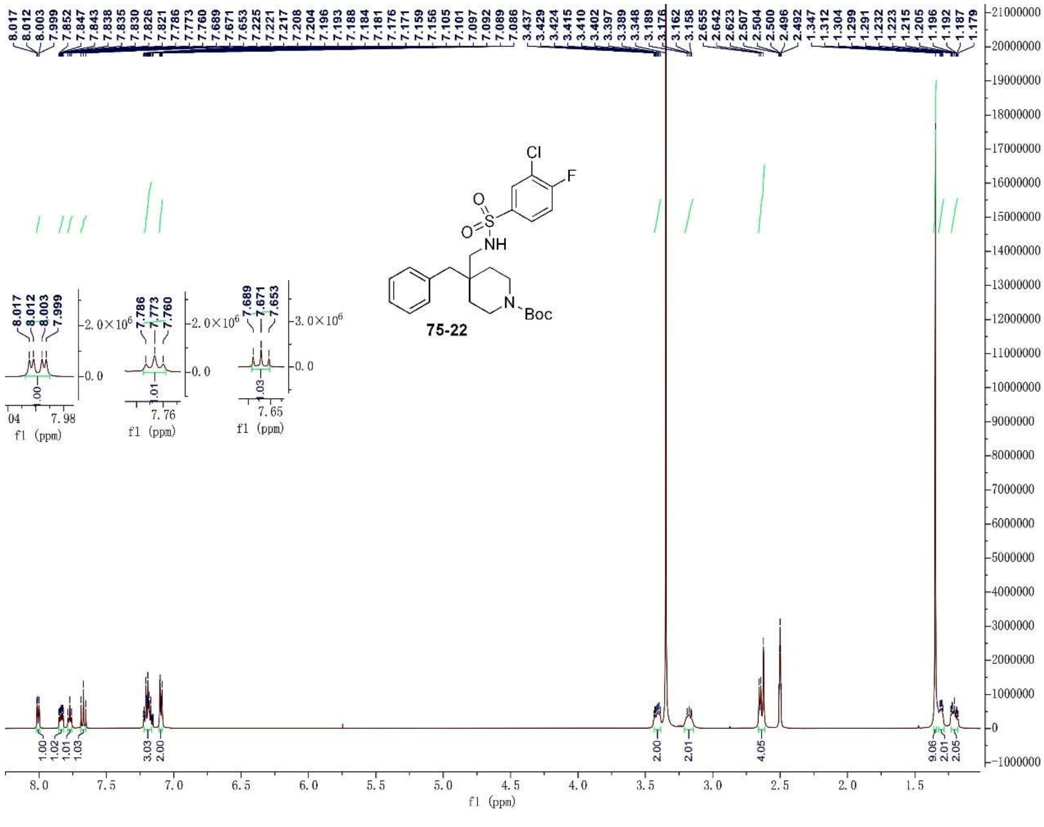

Figure S334: ^{1}H NMR spectrum of **75-22**

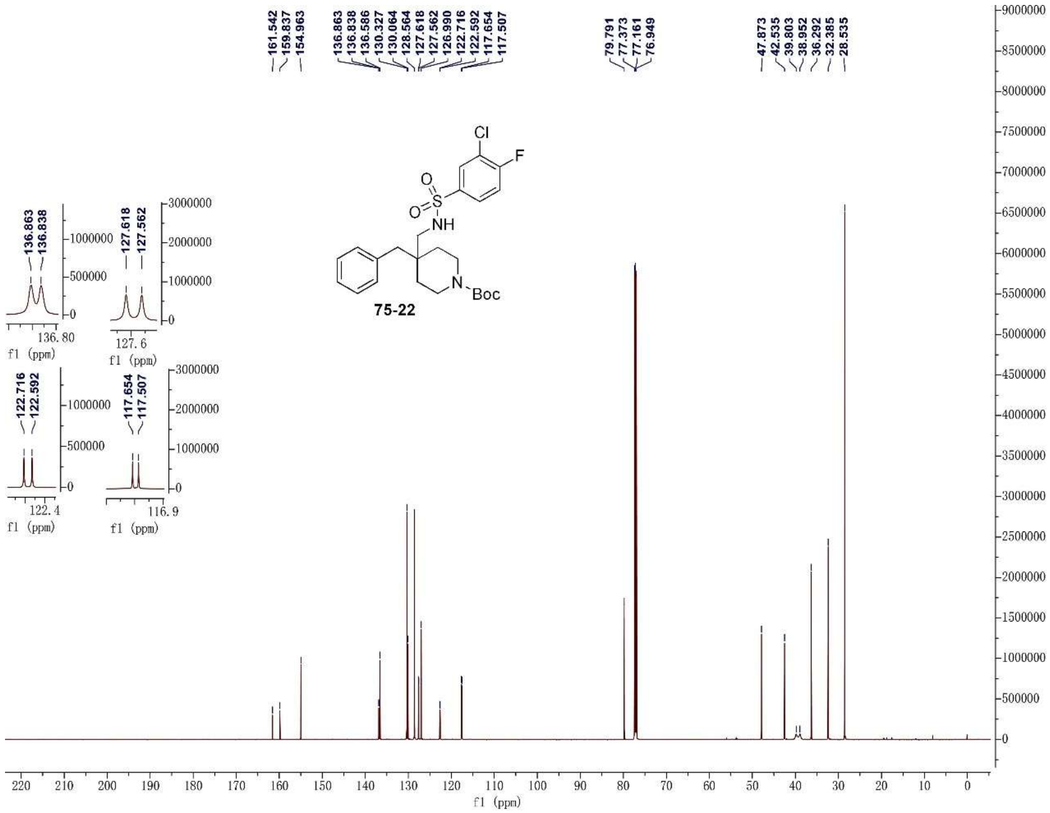

Figure S335: ^{13}C NMR spectrum of **75-22**

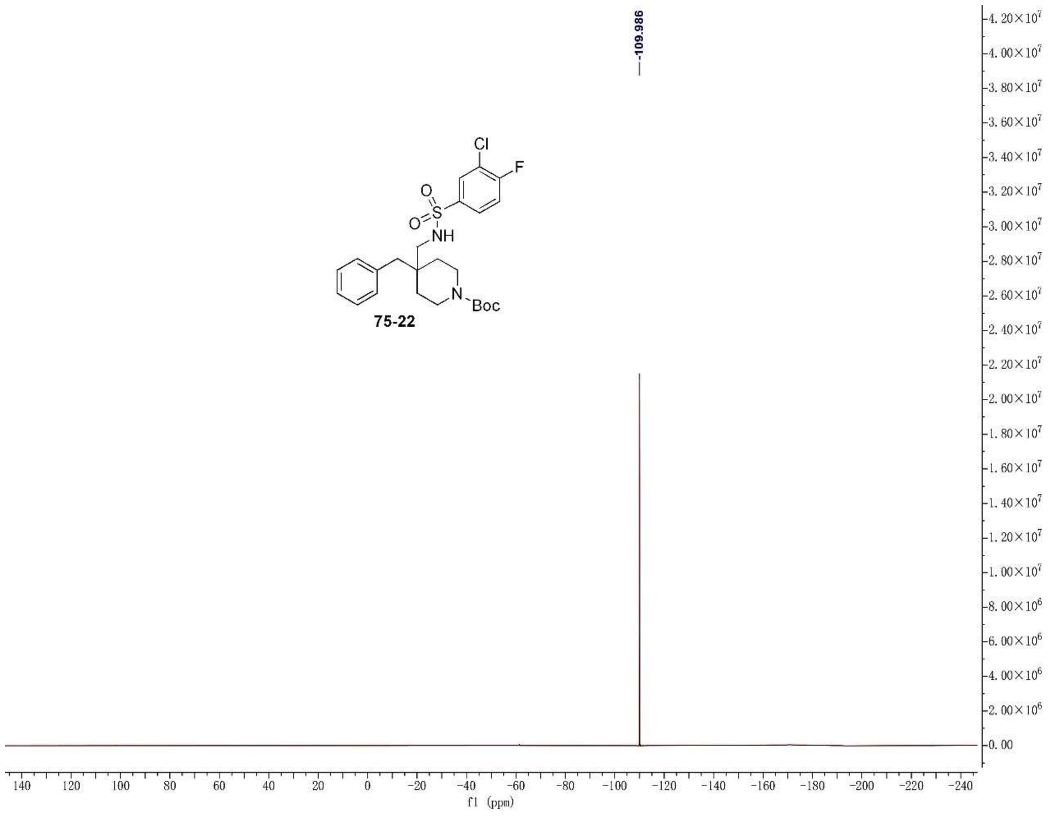

Figure S336: ^{19}F NMR spectrum of **75-22**

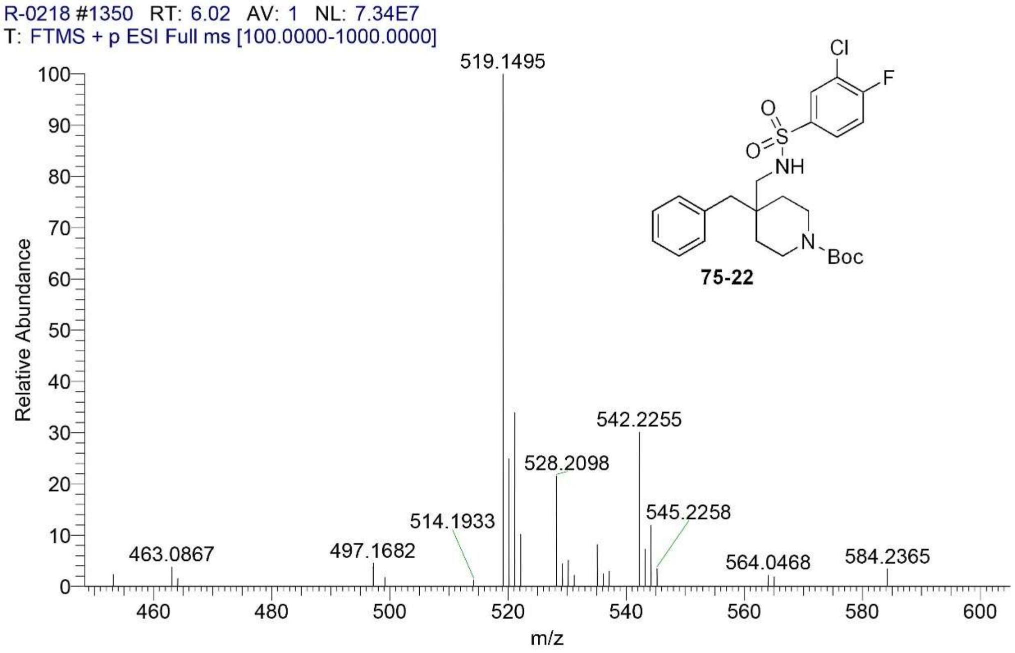

Figure S337: HR-MS (ESI/ion trap) spectrum of **75-22**

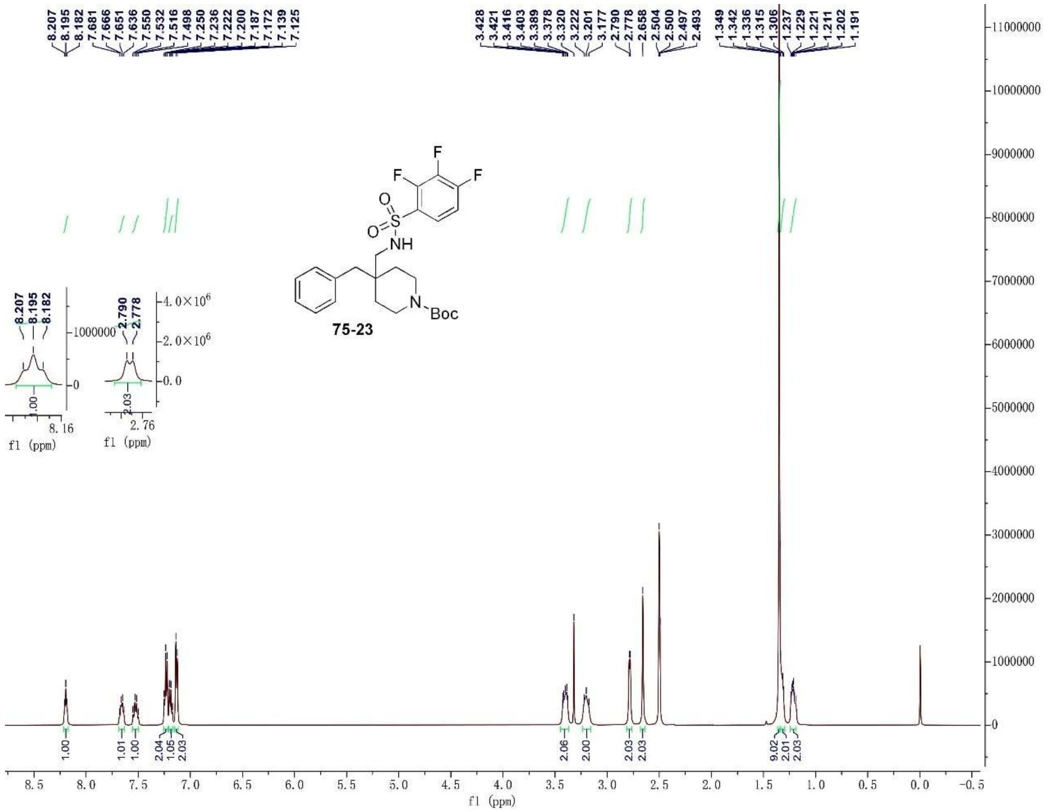

Figure S338: ^{1}H NMR spectrum of **75-23**

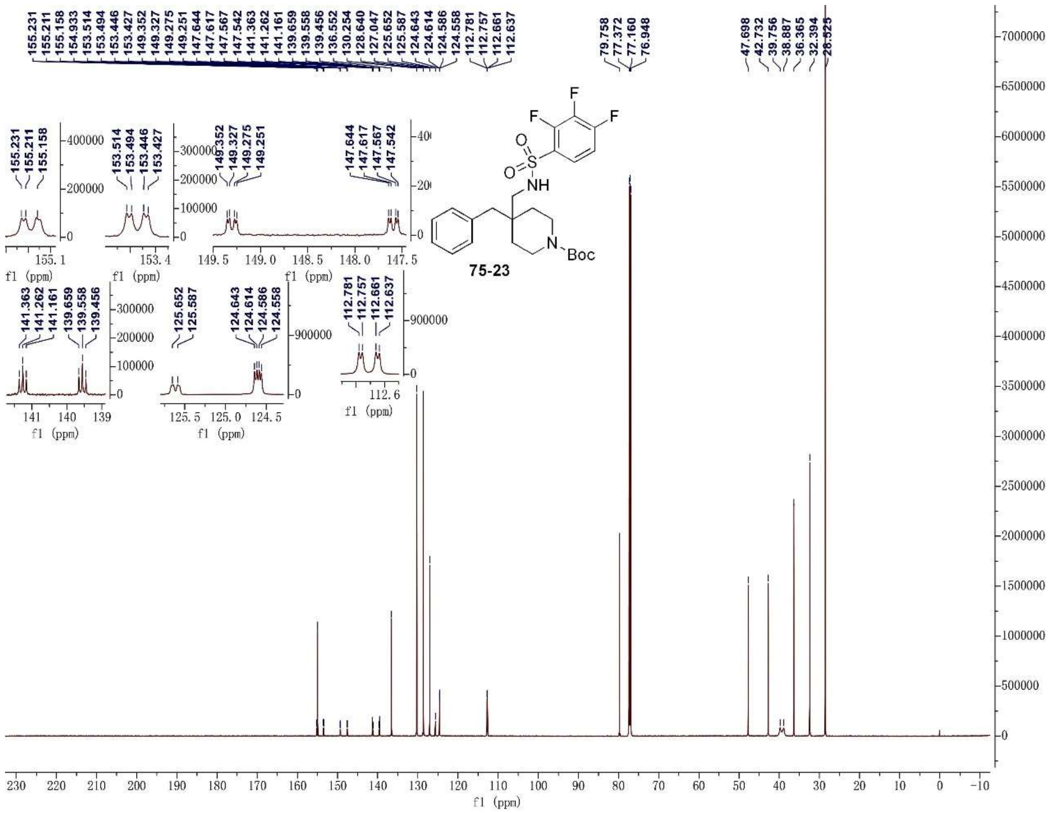

Figure S339: ^{13}C NMR spectrum of **75-23**

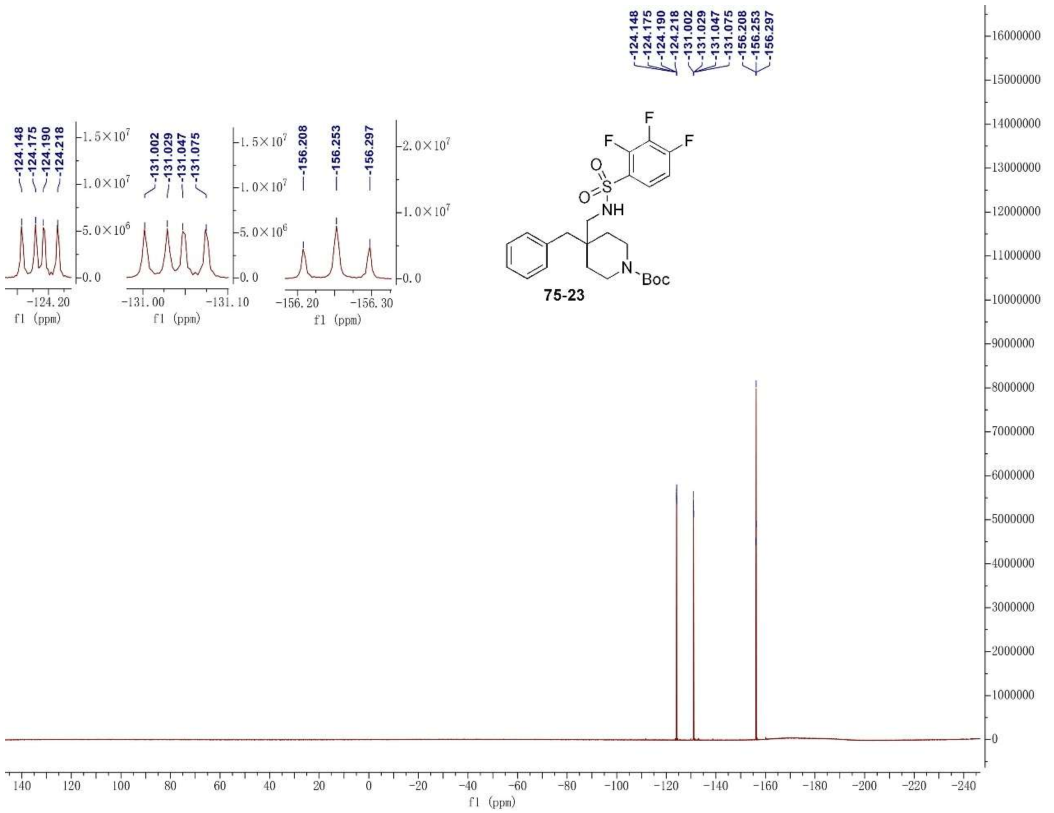

Figure S340: ^{19}F NMR spectrum of **75-23**

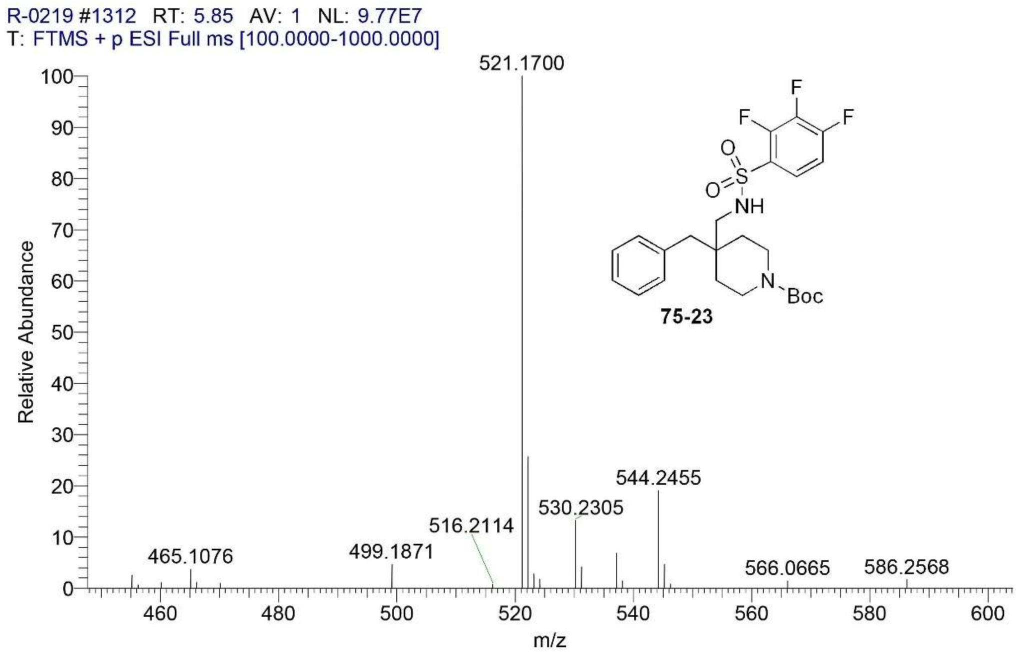

Figure S341: HR-MS (ESI/ion trap) spectrum of **75-23**

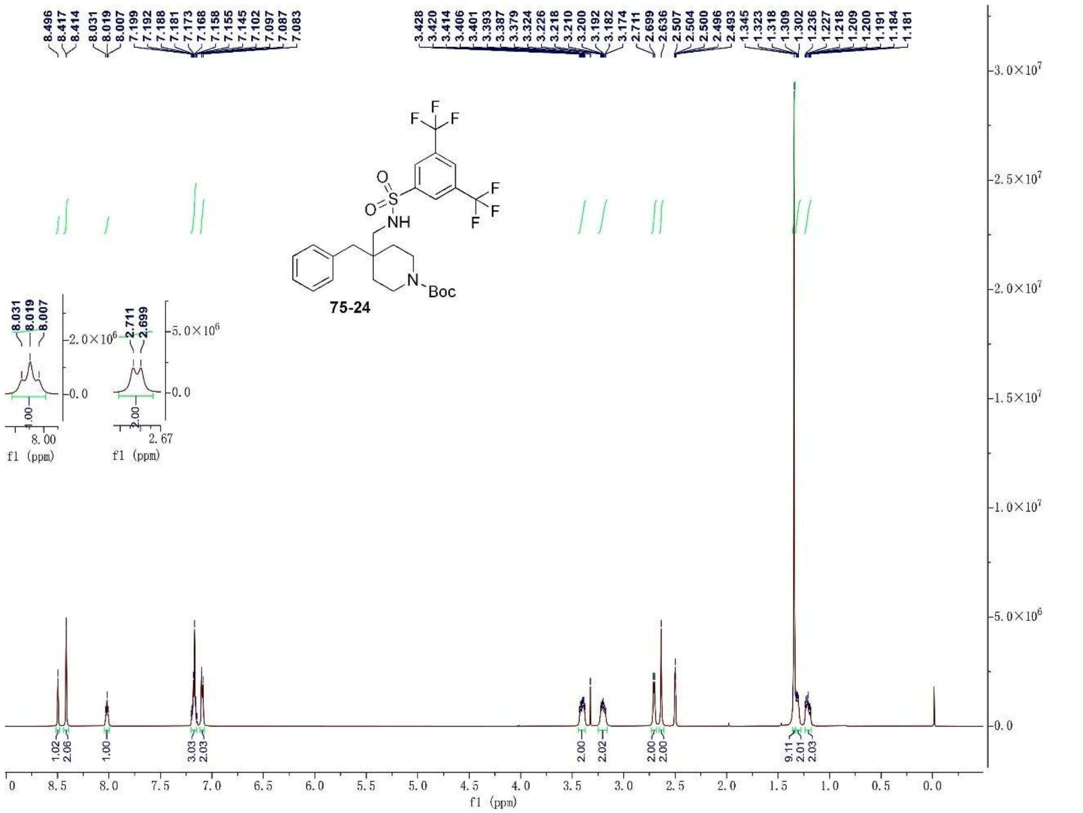

Figure S342: ^{1}H NMR spectrum of **75-24**

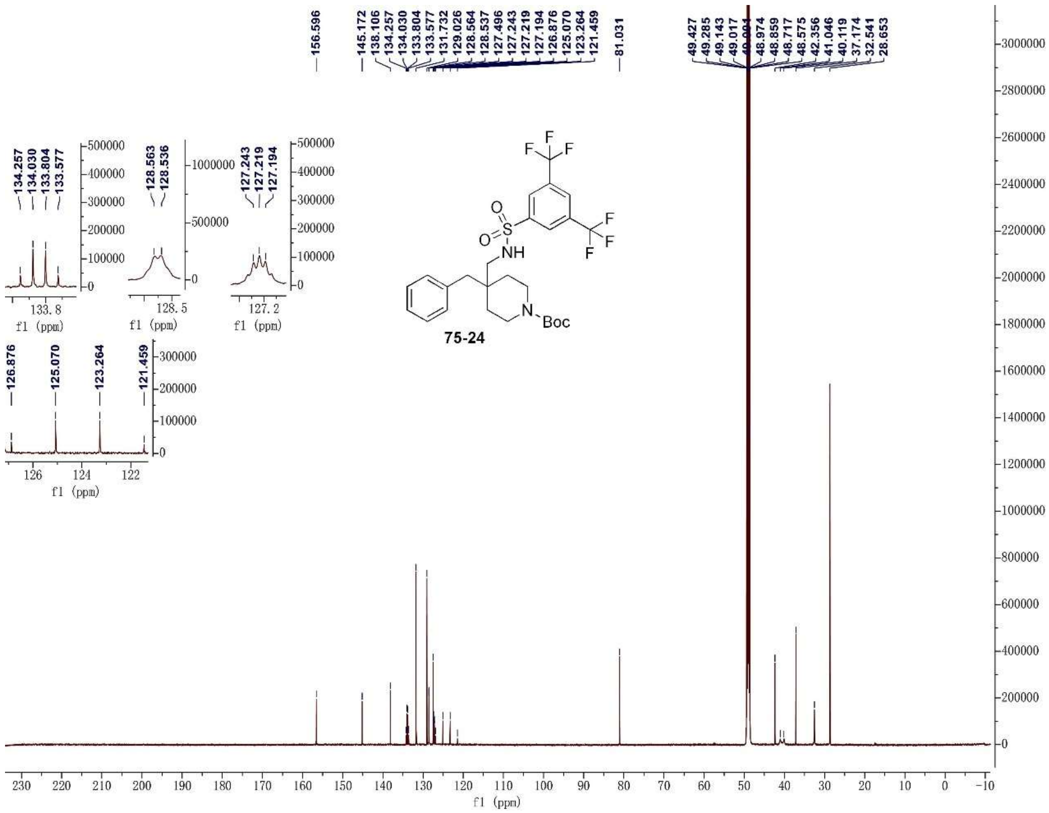

Figure S343: ^{13}C NMR spectrum of **75-24**

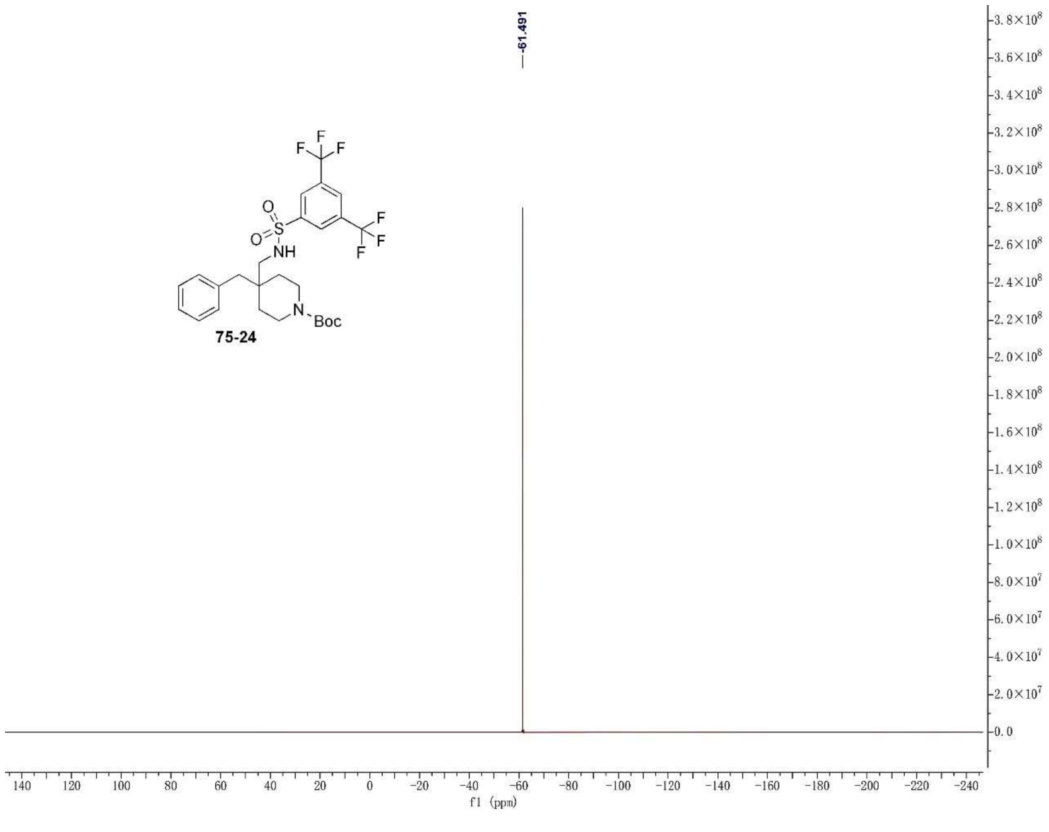

Figure S344: ^{19}F NMR spectrum of **75-24**

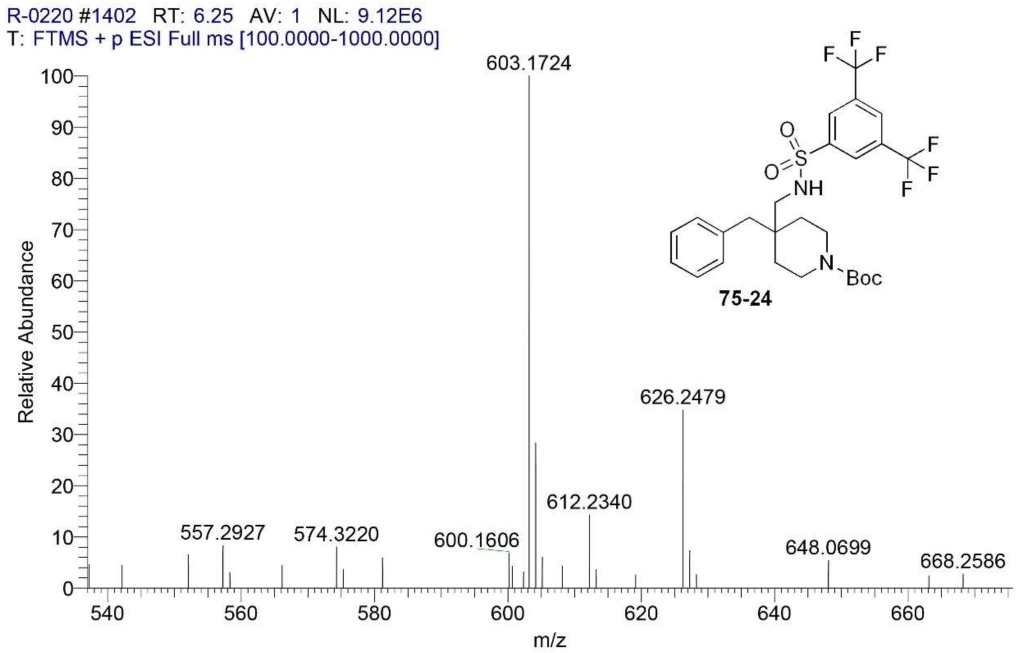

Figure S345: HR-MS (ESI/ion trap) spectrum of **75-24**

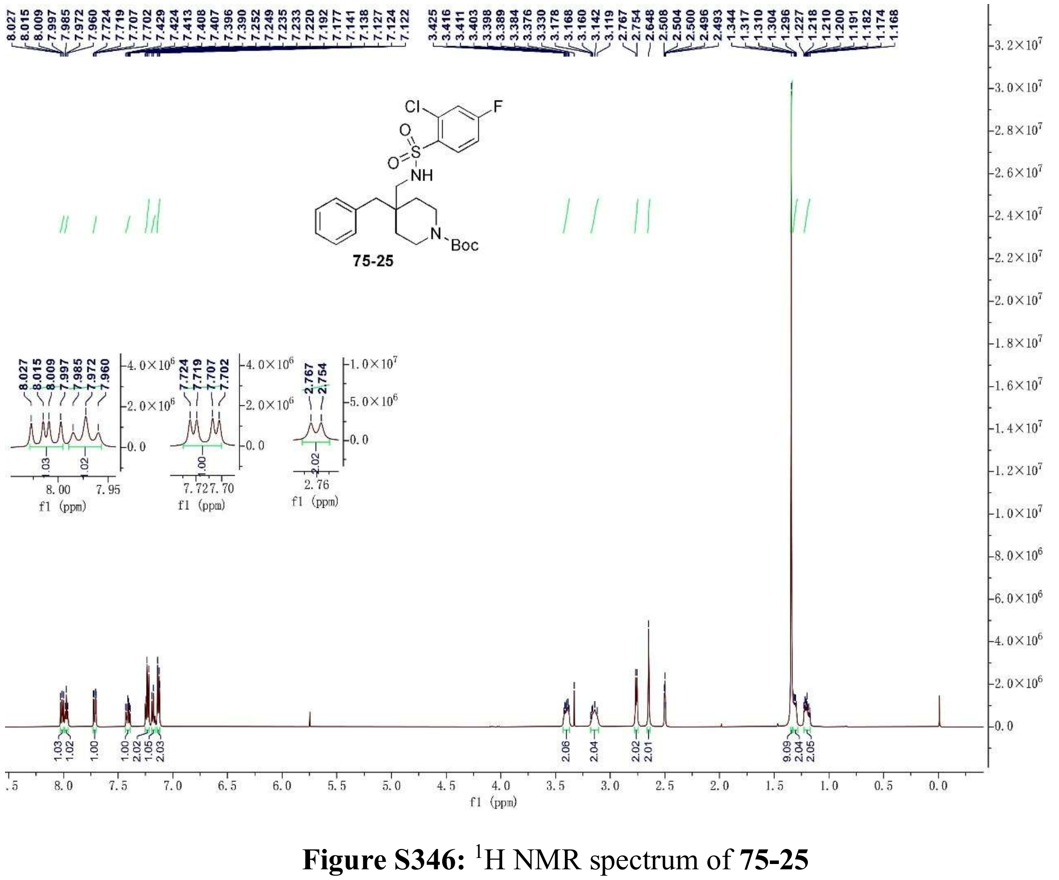

Figure S346: ^{1}H NMR spectrum of **75-25**

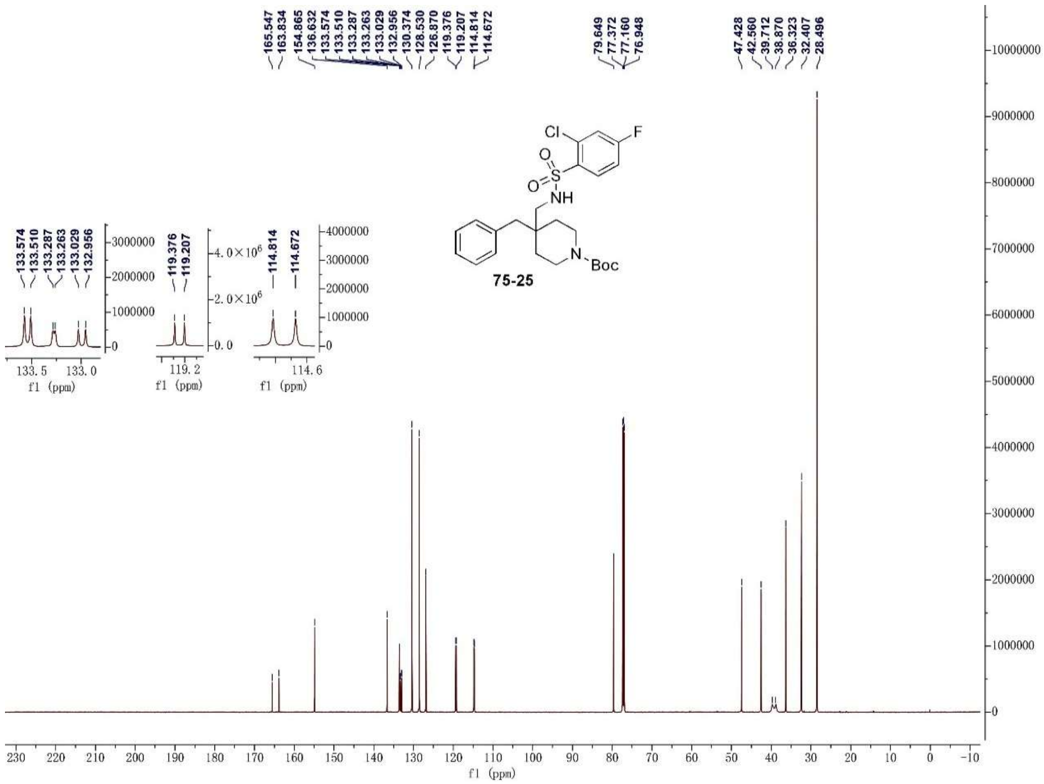

Figure S347: ^{13}C NMR spectrum of **75-25**

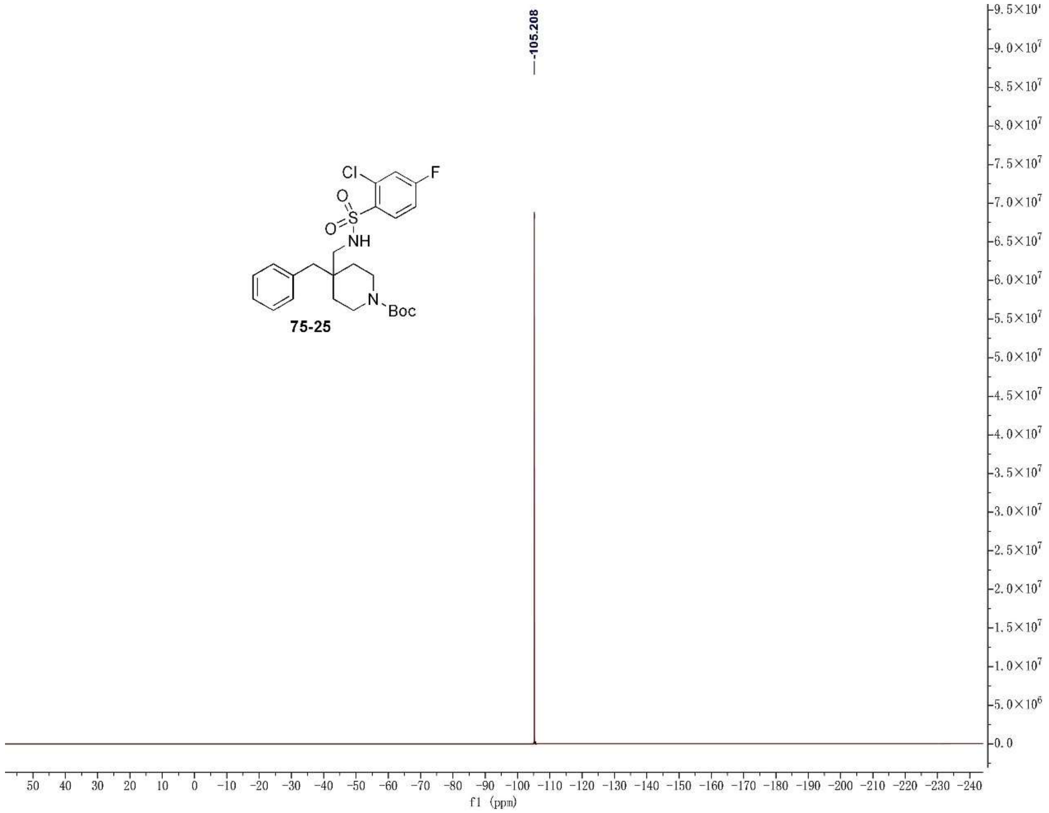

Figure S348: ^{19}F NMR spectrum of **75-25**

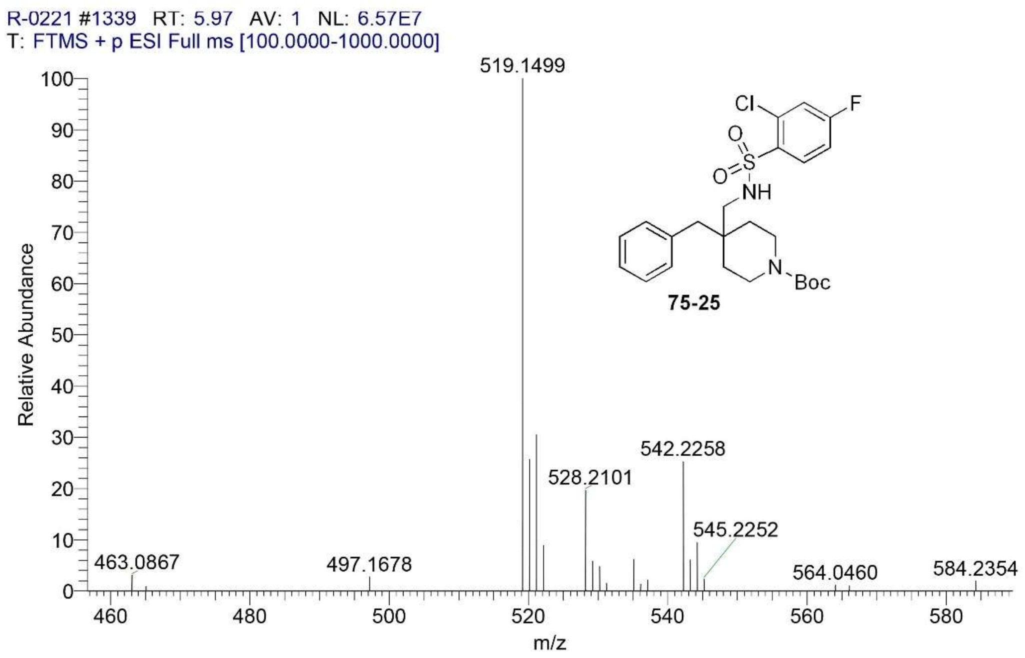

Figure S349: HR-MS (ESI/ion trap) spectrum of **75-25**

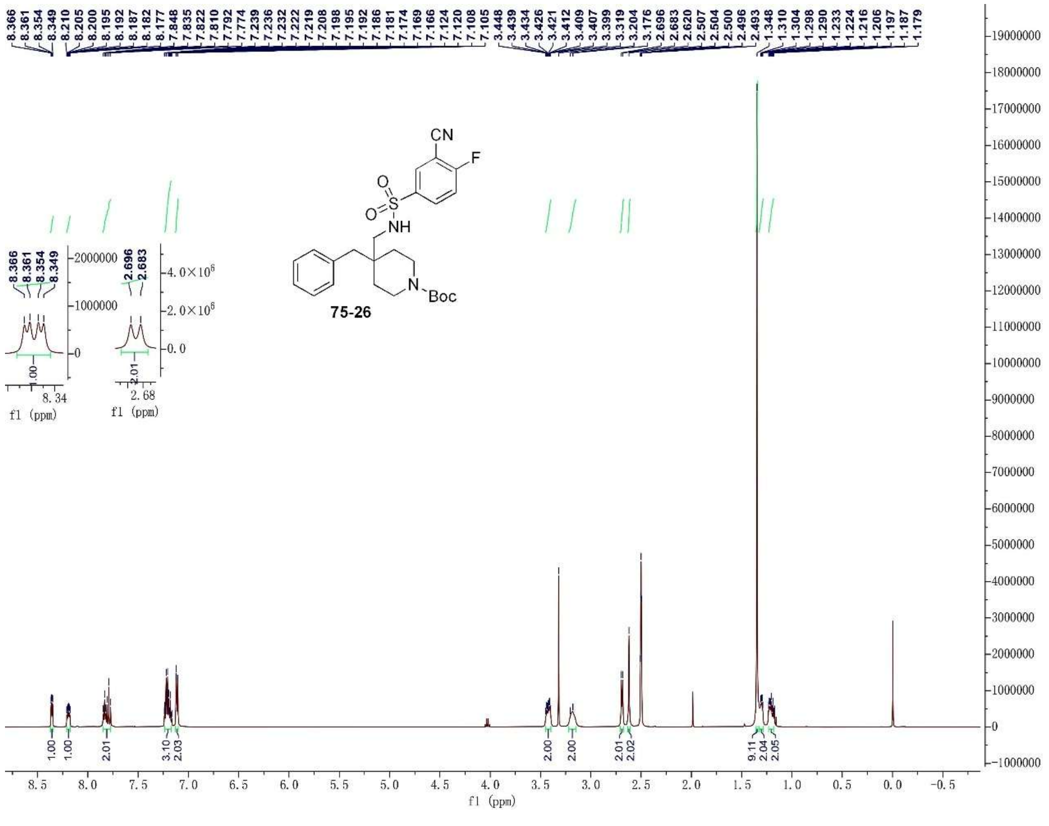

Figure S350: ^{1}H NMR spectrum of **75-26**

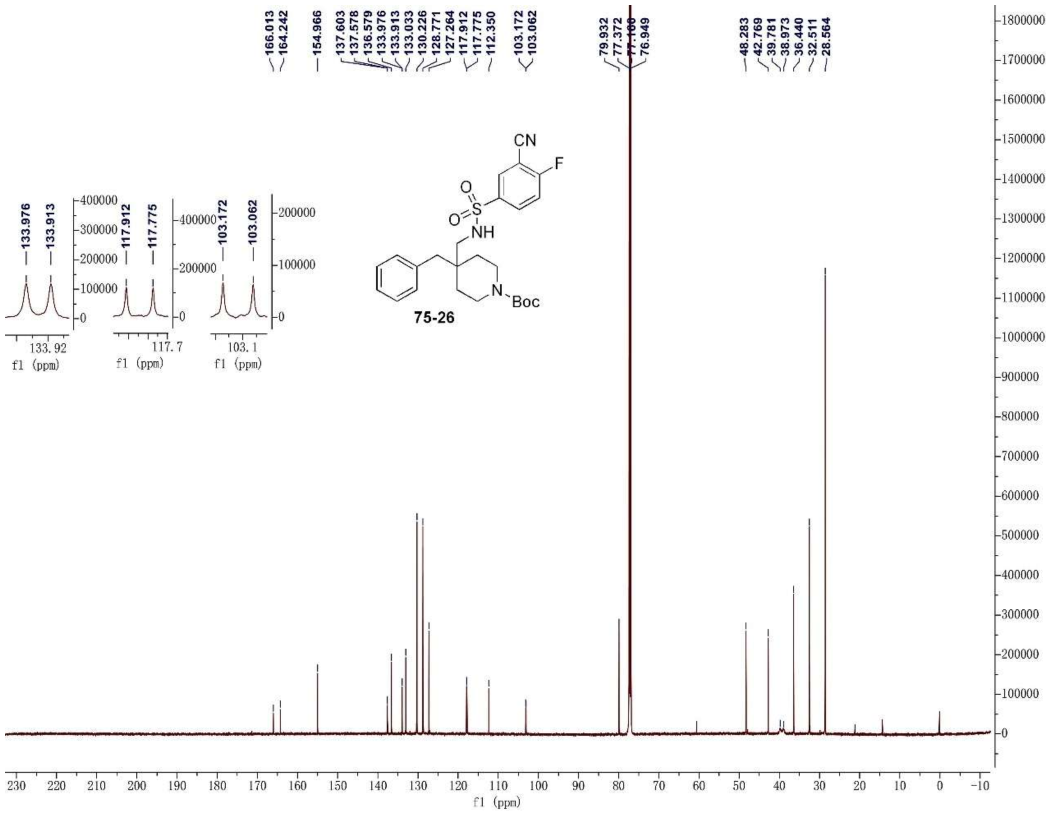

Figure S351: ^{13}C NMR spectrum of **75-26**

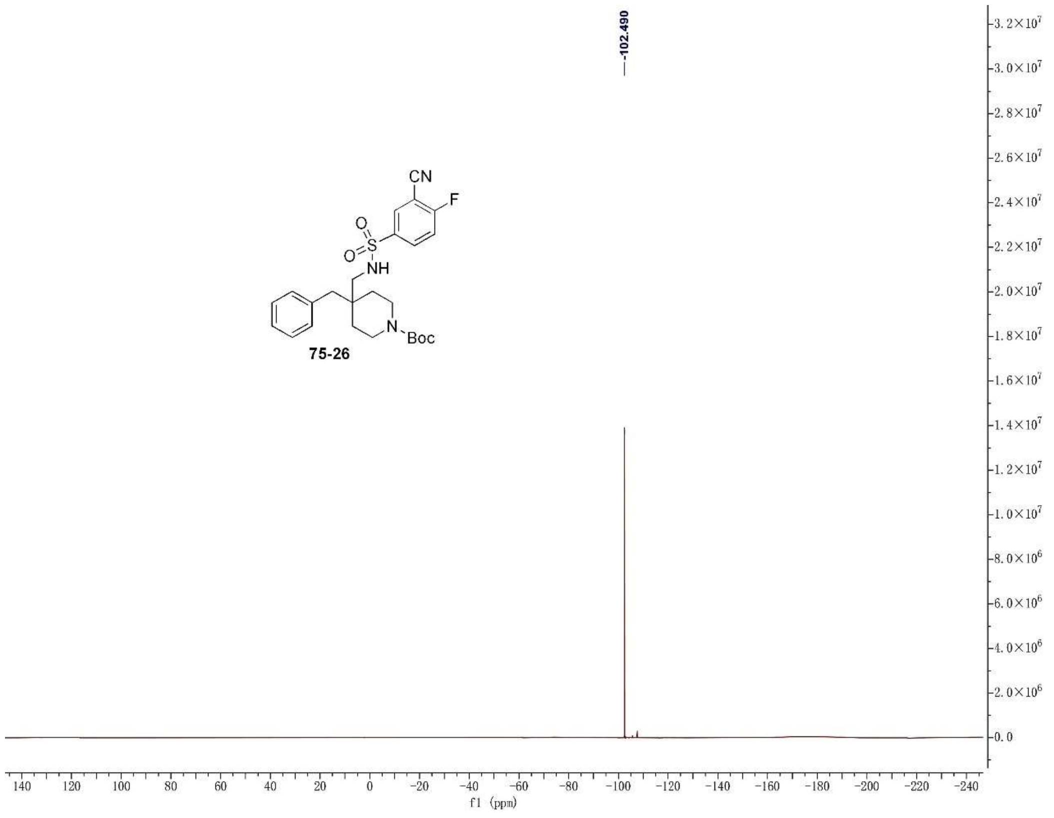

Figure S352: ^{19}F NMR spectrum of **75-26**

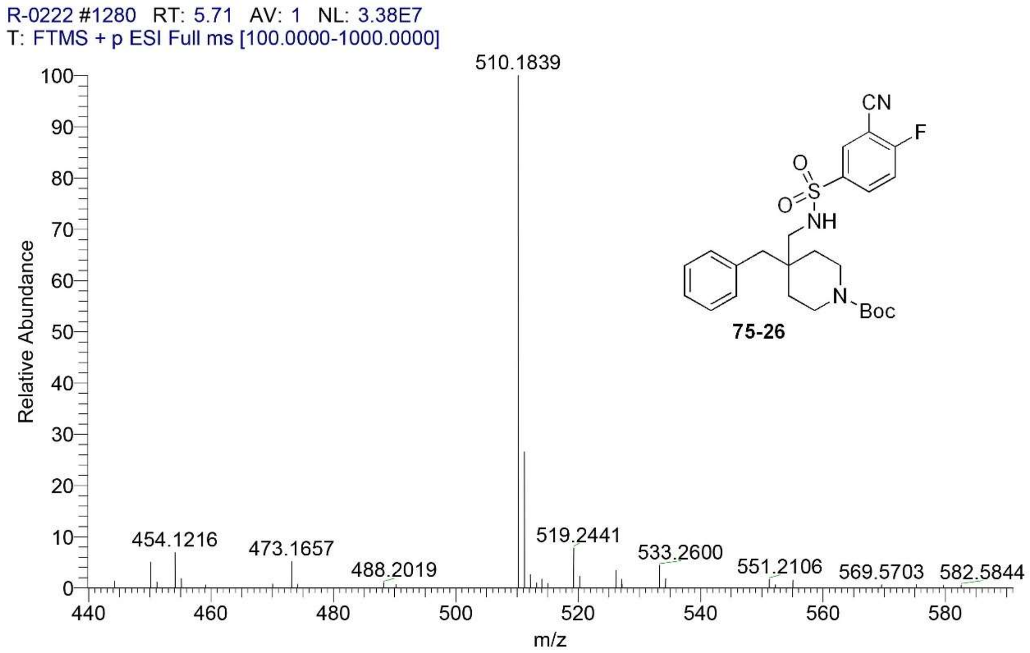

Figure S353: HR-MS (ESI/ion trap) spectrum of **75-26**

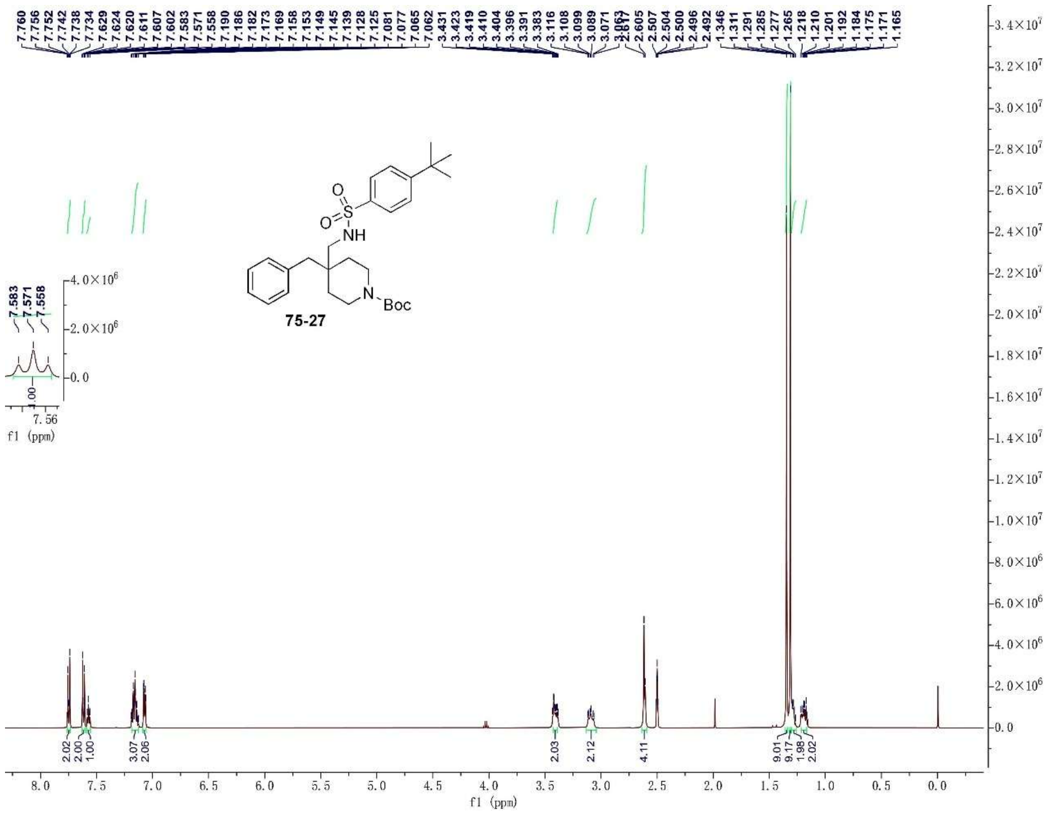

Figure S354: ^{1}H NMR spectrum of **75-27**

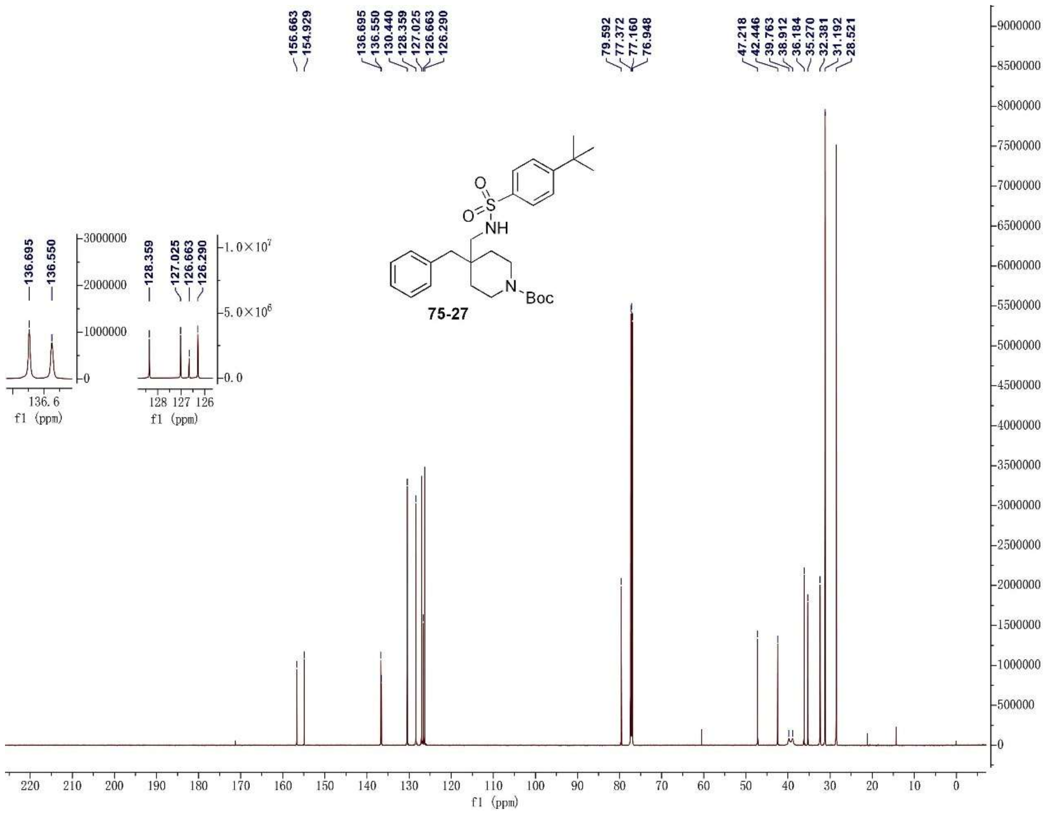

Figure S355: ^{13}C NMR spectrum of **75-27**

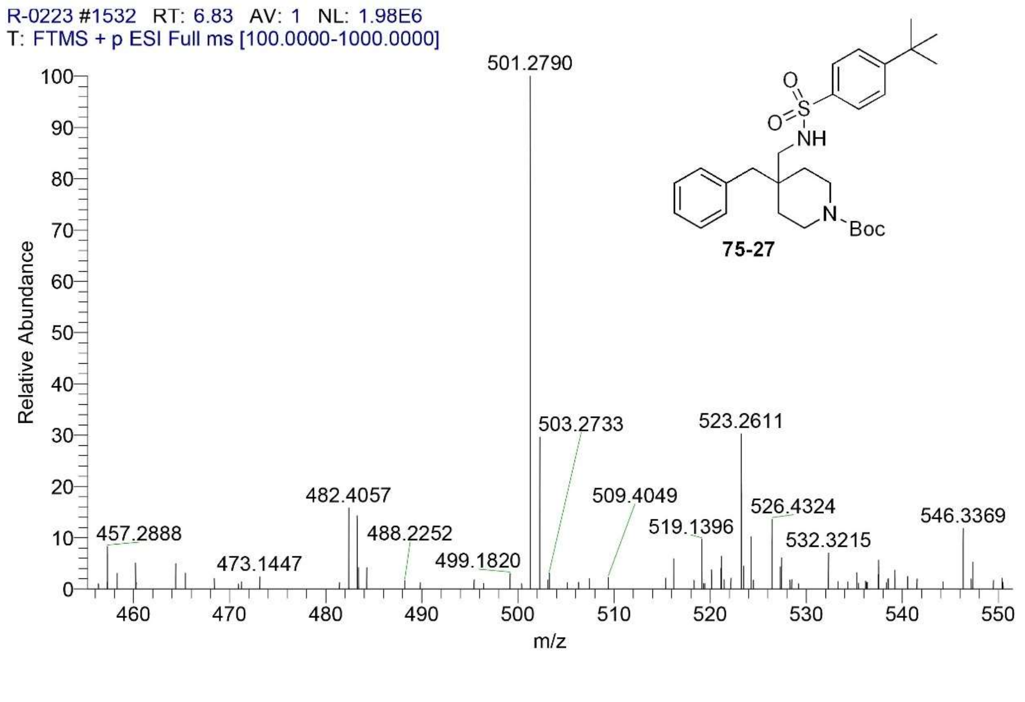

Figure S356: HR-MS (ESI/ion trap) spectrum of **75-27**

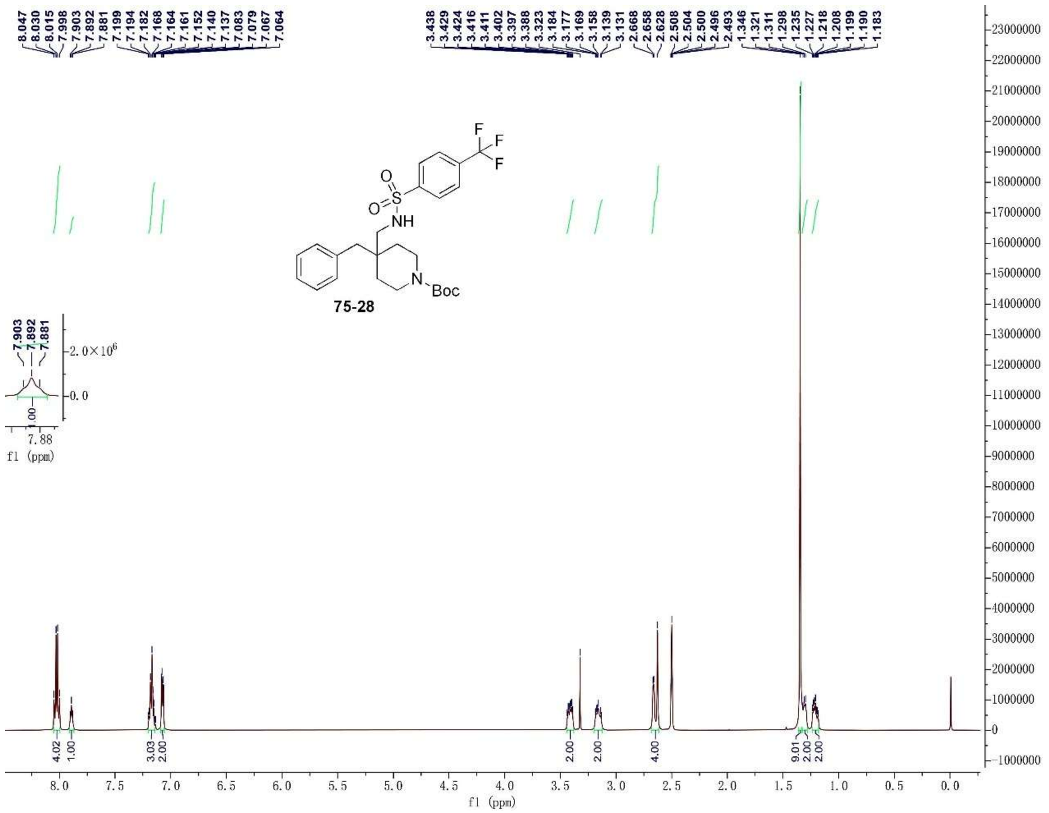

Figure S357: ^{1}H NMR spectrum of **75-28**

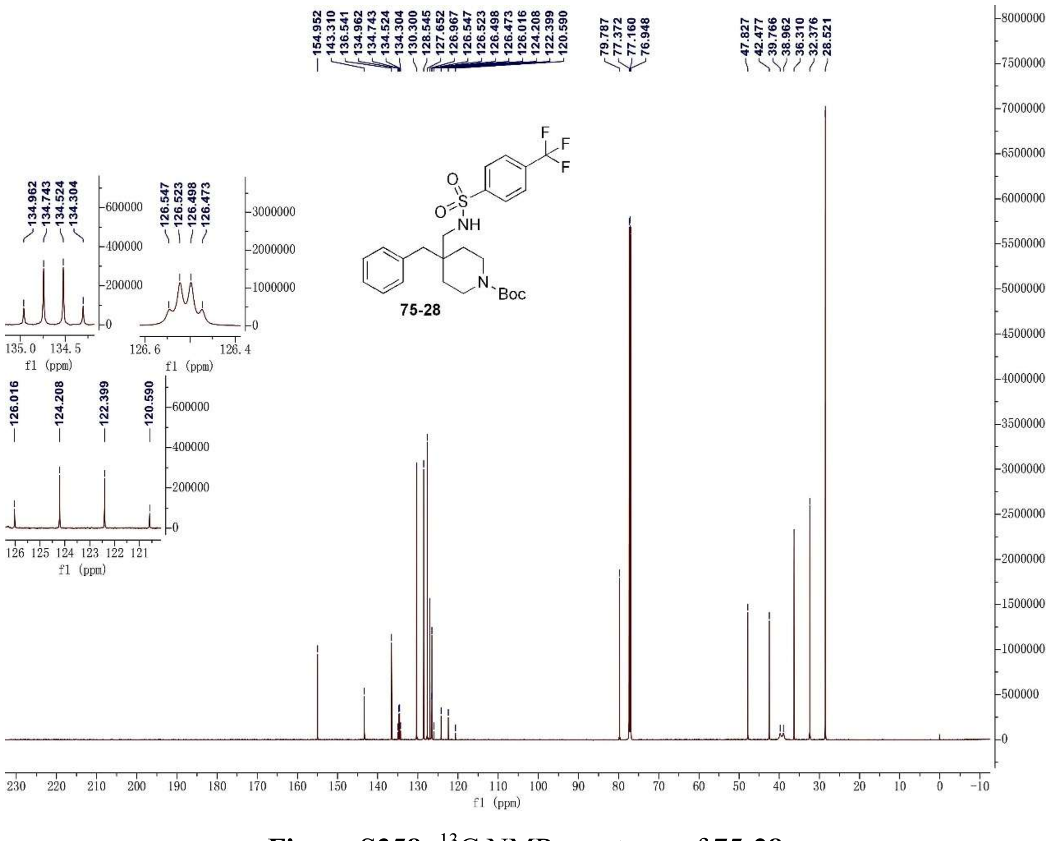

Figure S358: ^{13}C NMR spectrum of **75-28**

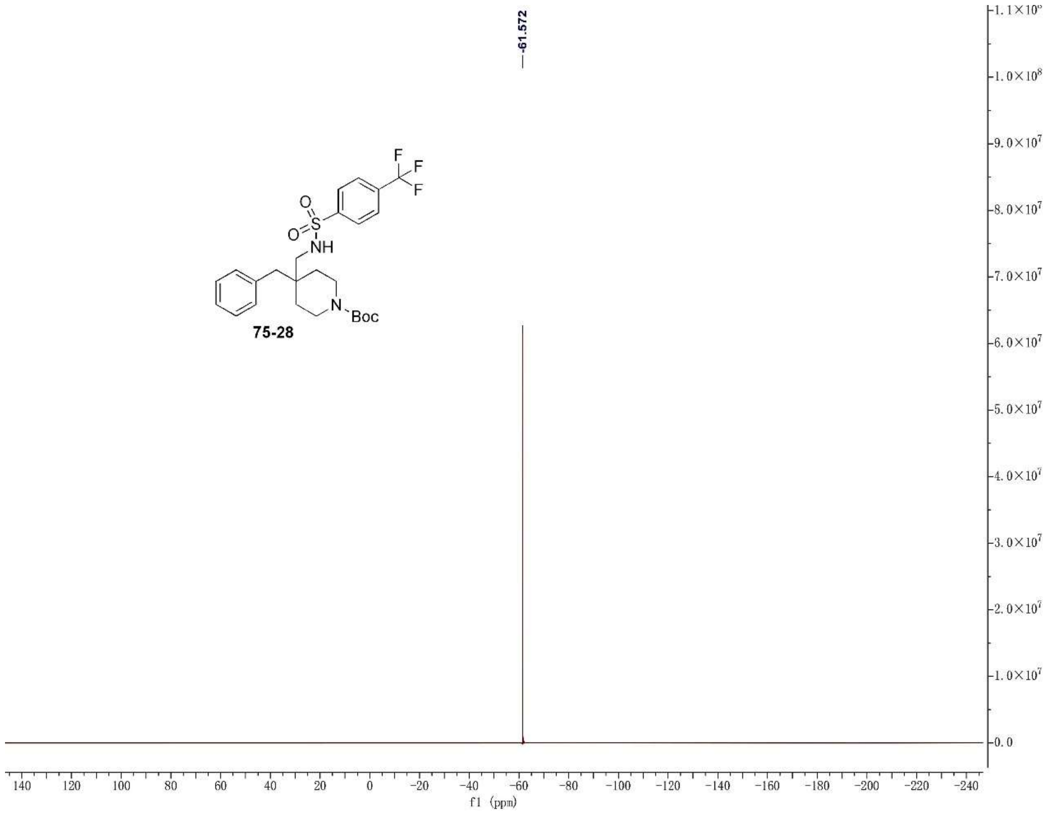

Figure S360: ^{19}F NMR spectrum of **75-28**

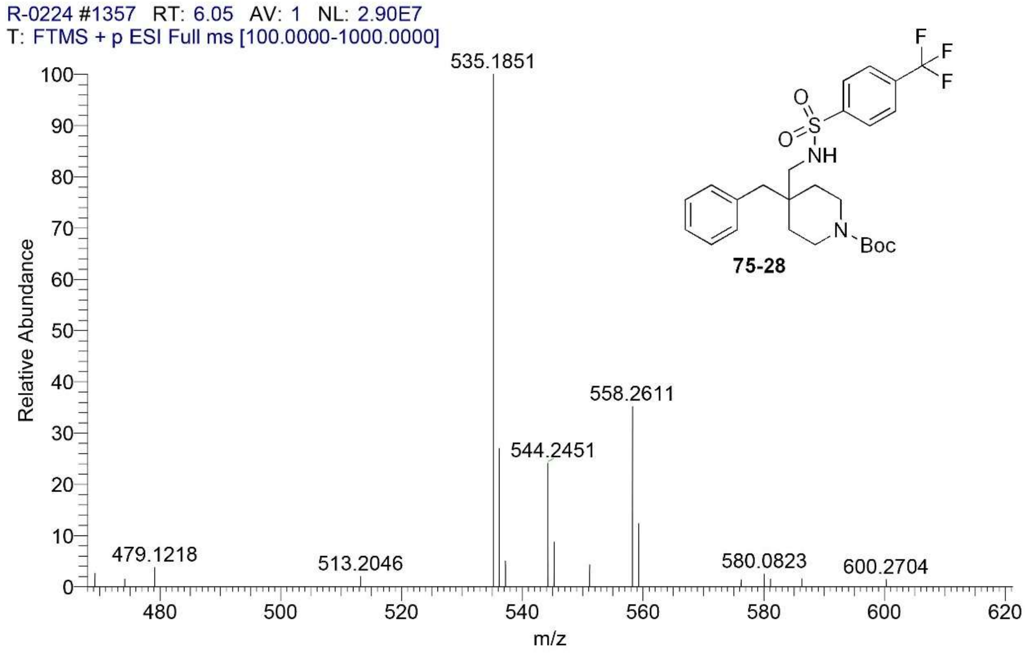

Figure S361: HR-MS (ESI/ion trap) spectrum of **75-28**

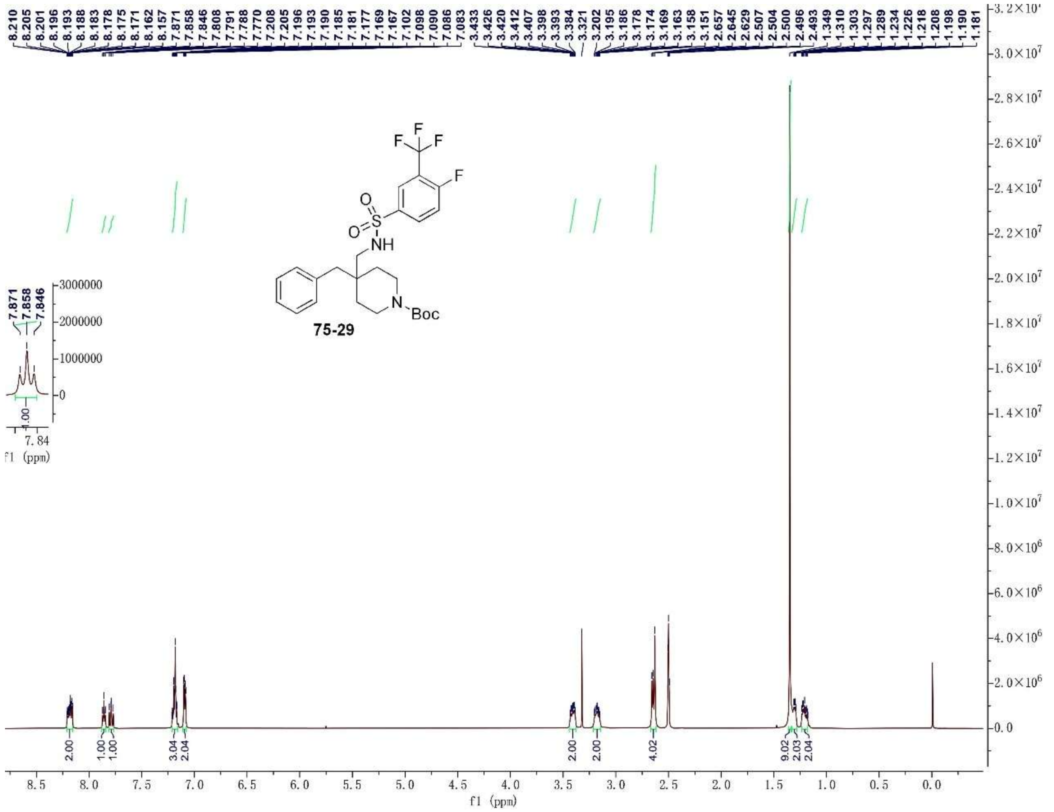

Figure S361: ^{1}H NMR spectrum of **75-29**

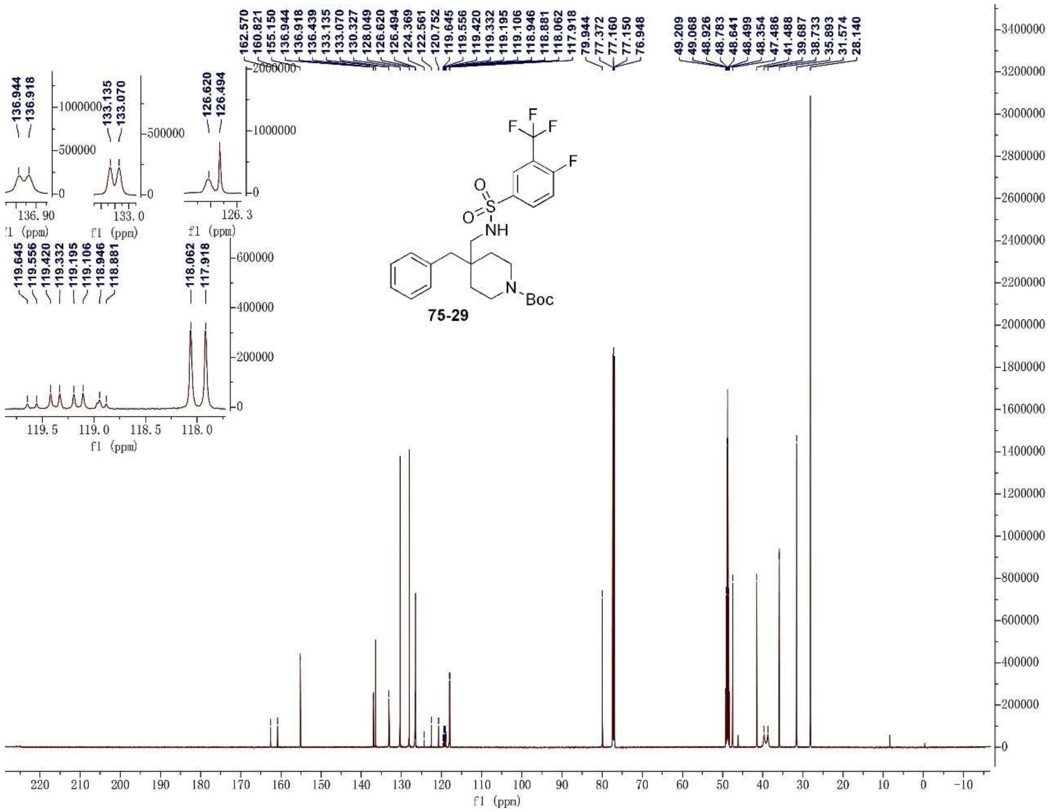

Figure S362: ^{13}C NMR spectrum of **75-29**

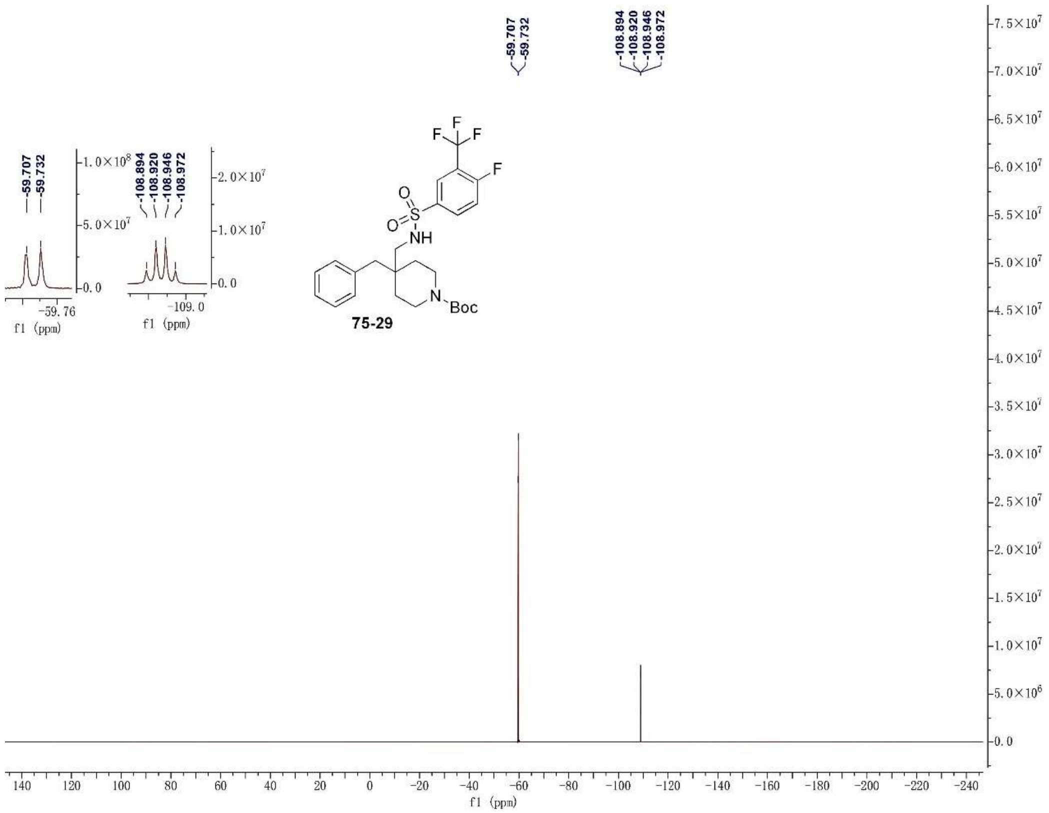

Figure S363: ^{19}F NMR spectrum of **75-29**

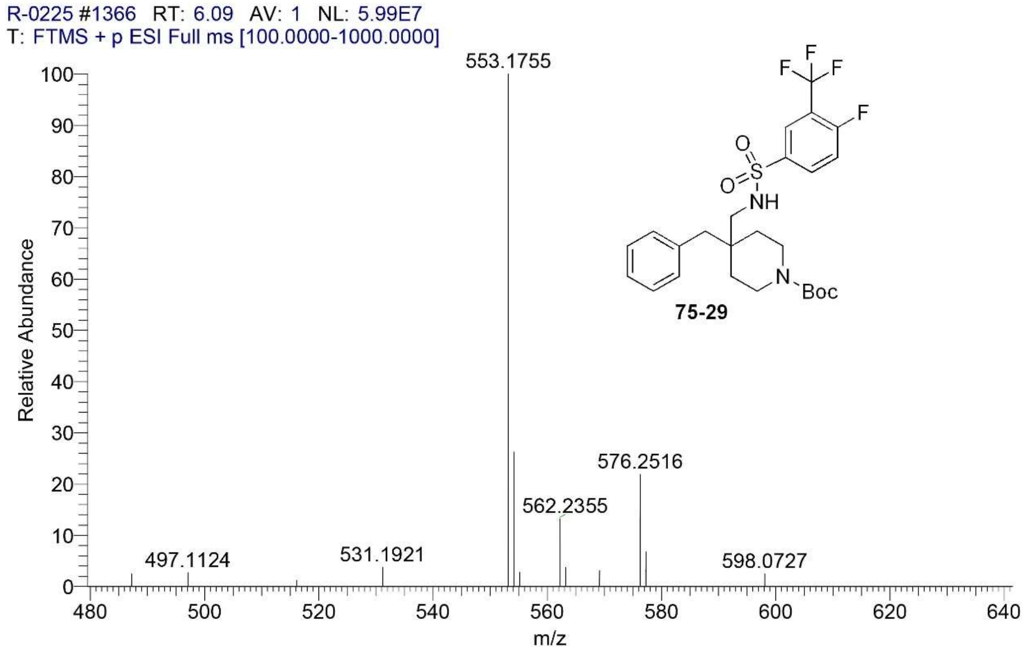

Figure S364: HR-MS (ESI/ion trap) spectrum of **75-29**

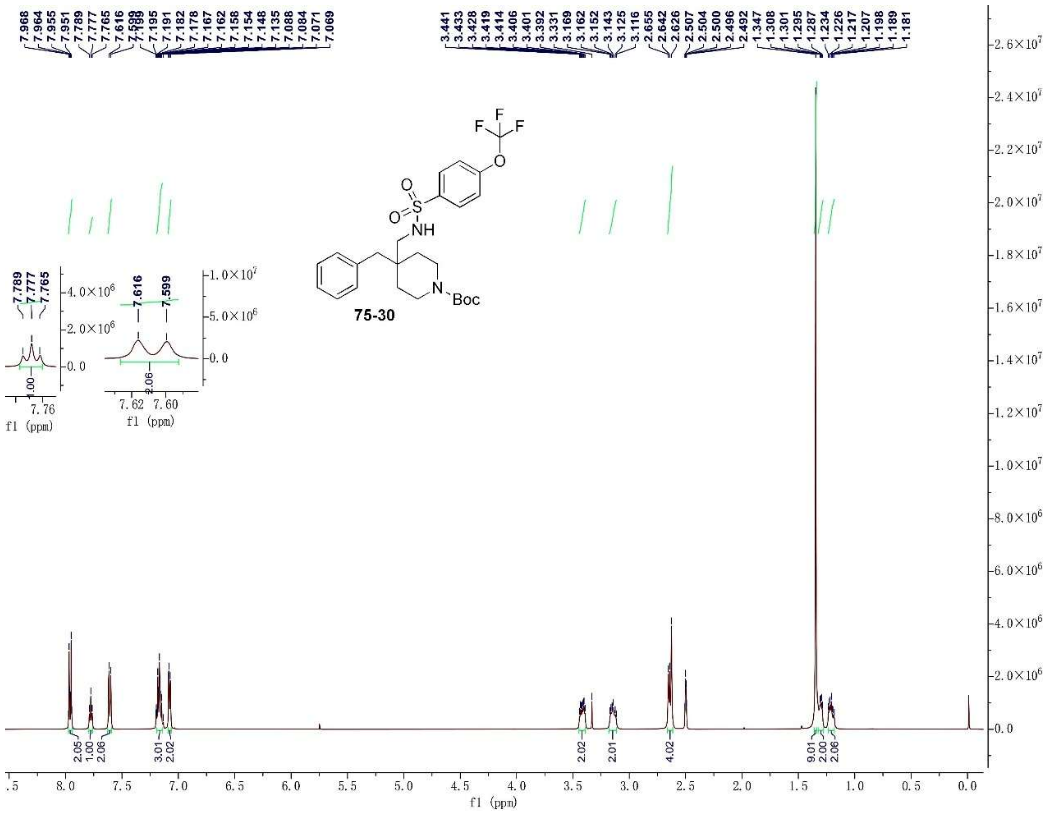

Figure S365: ^{1}H NMR spectrum of **75-30**

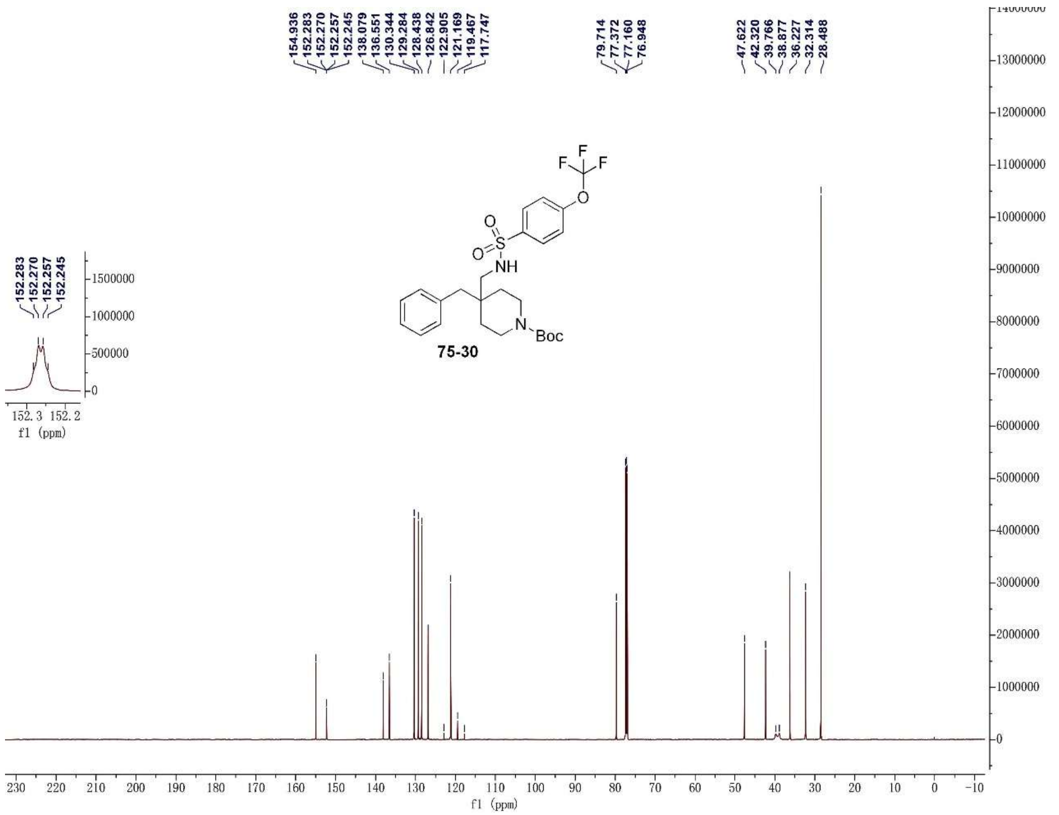

Figure S366: ^{13}C NMR spectrum of **75-30**

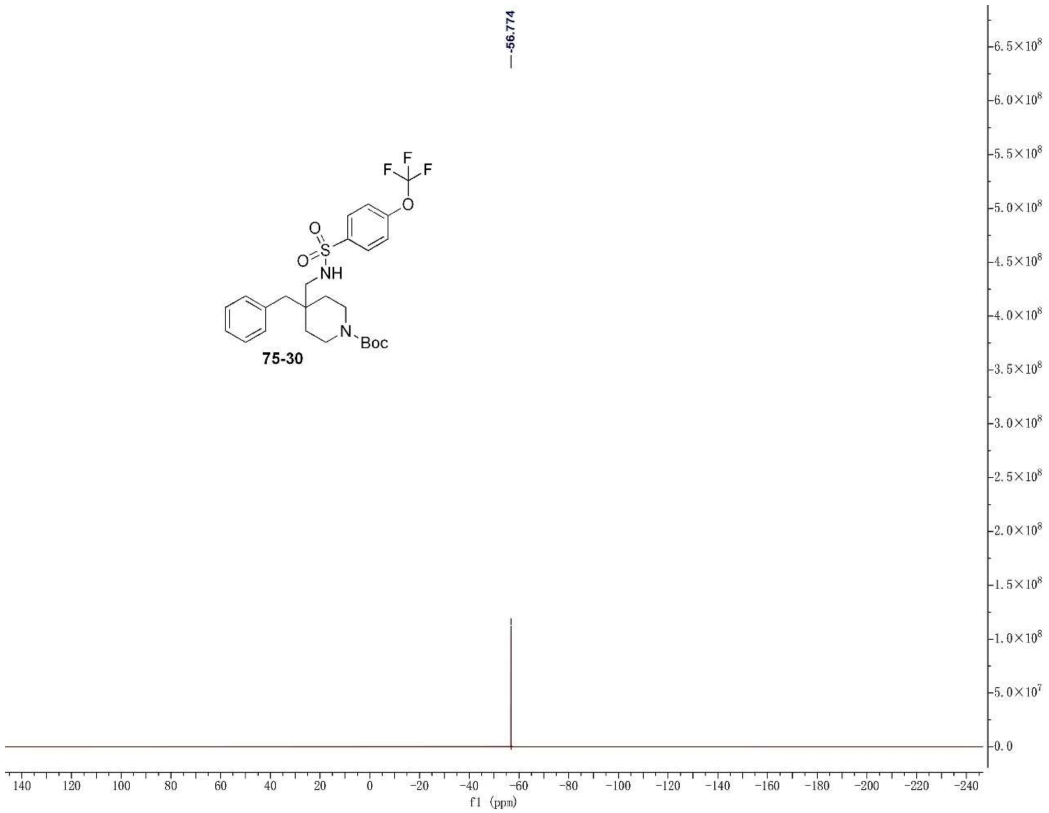

Figure S367: ^{19}F NMR spectrum of **75-30**

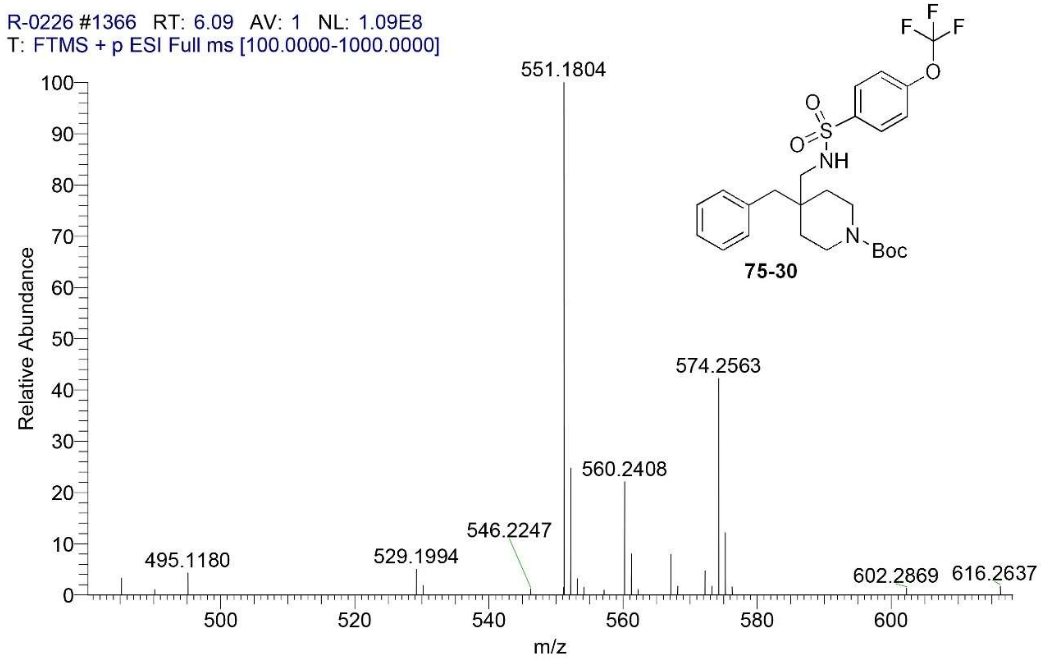

Figure S368: HR-MS (ESI/ion trap) spectrum of **75-30**

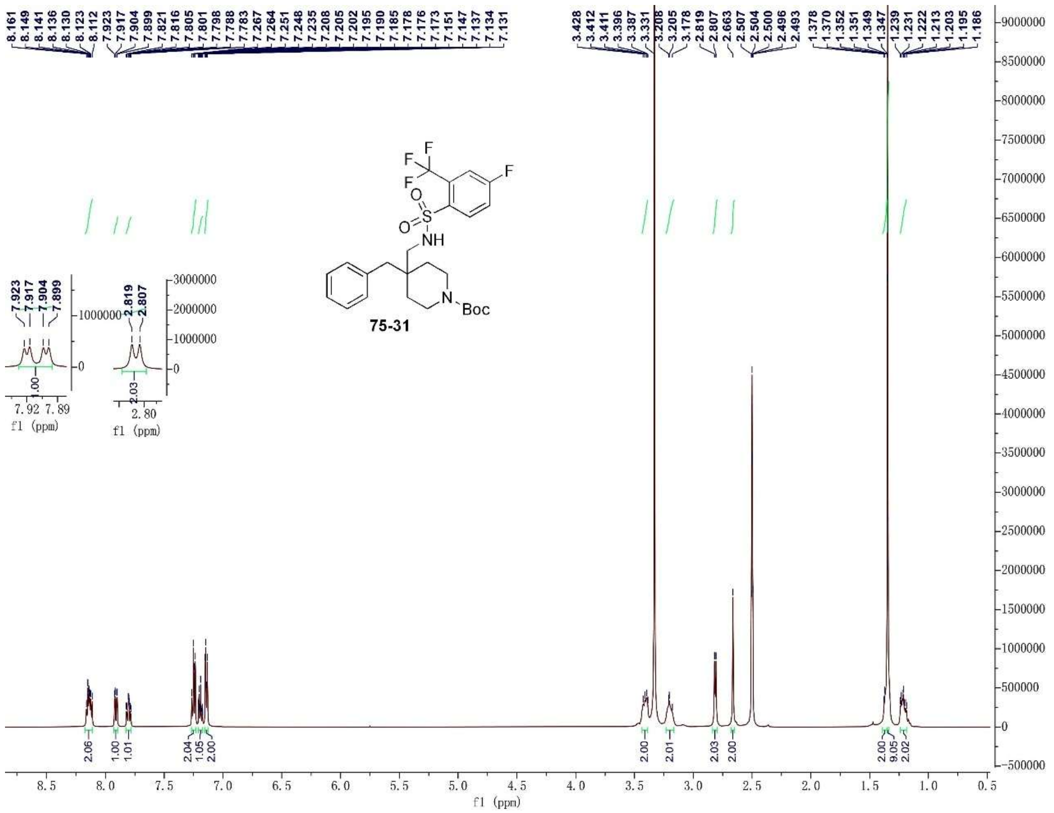

Figure S369: ^{1}H NMR spectrum of **75-31**

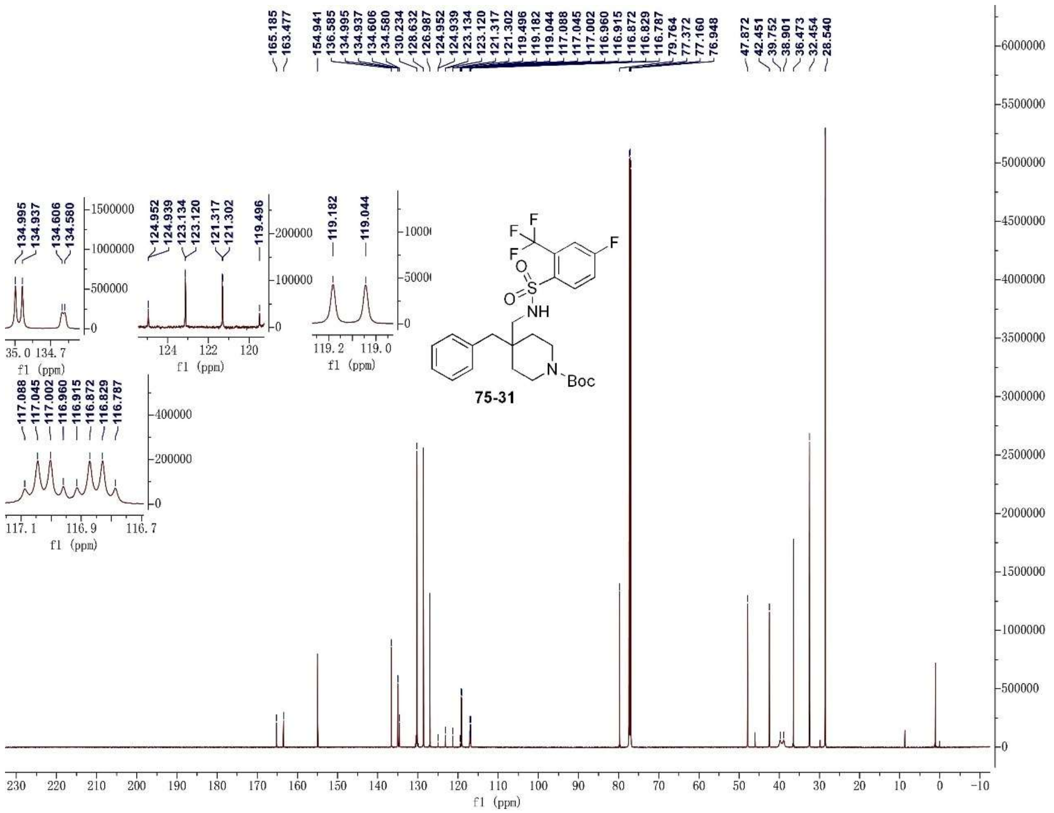

Figure S370: ^{13}C NMR spectrum of **75-31**

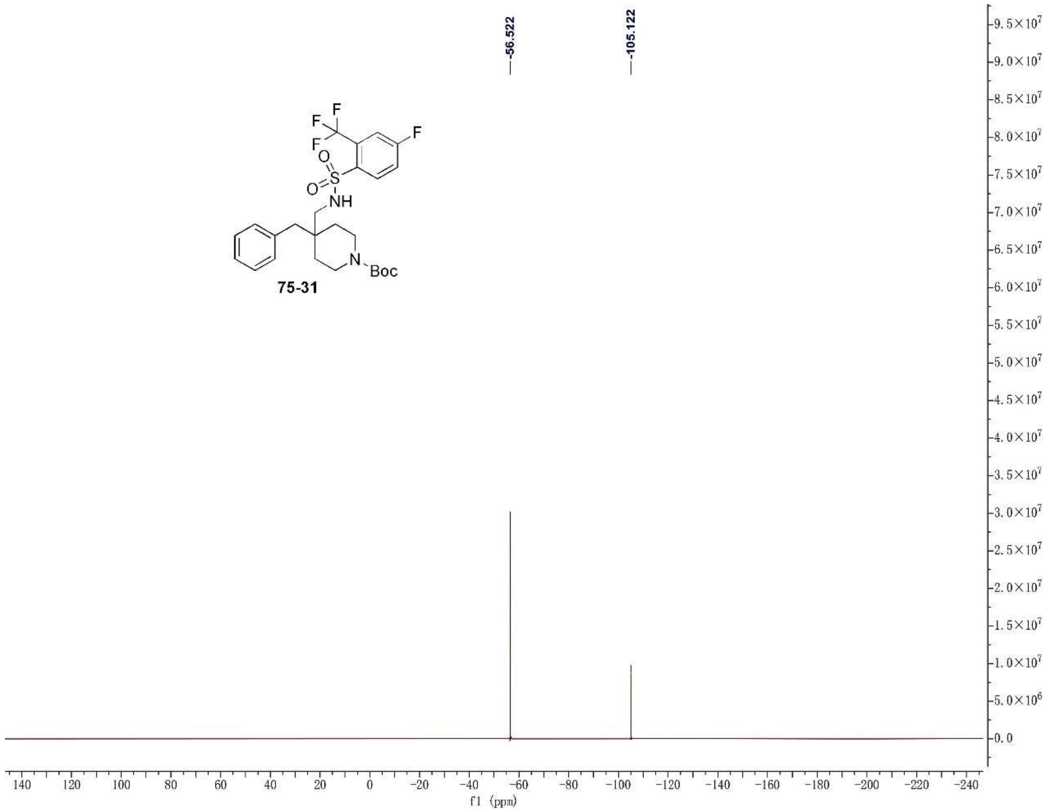

Figure S371: ^{19}F NMR spectrum of **75-31**

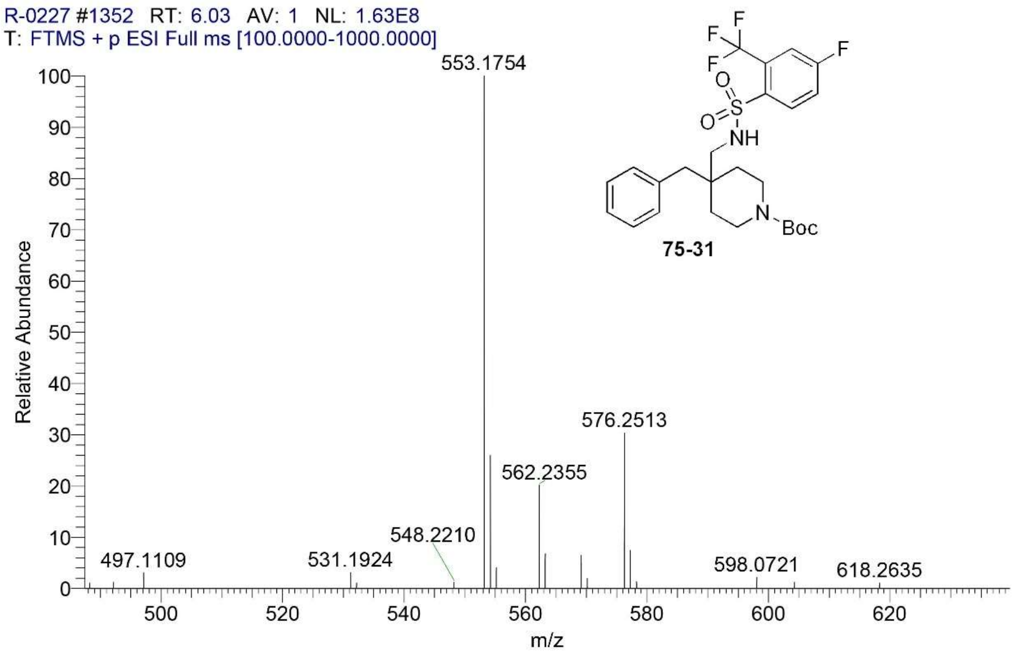

Figure S372: HR-MS (ESI/ion trap) spectrum of **75-31**

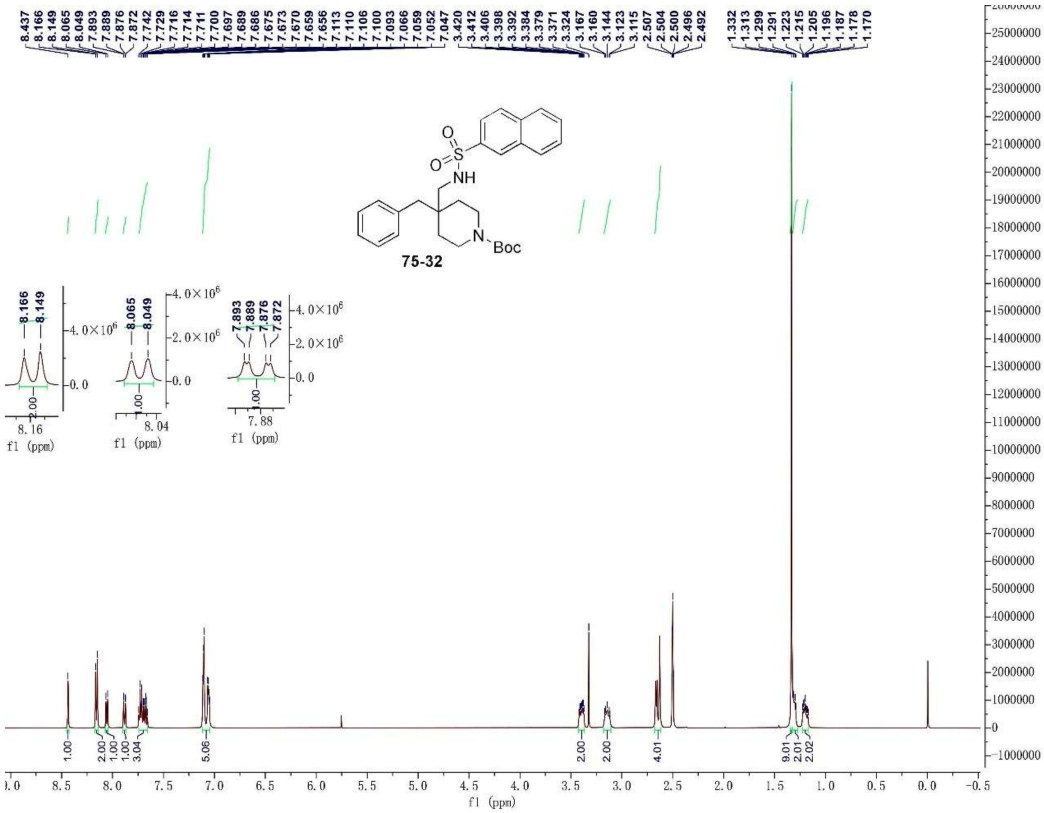

Figure S373: ^{1}H NMR spectrum of **75-32**

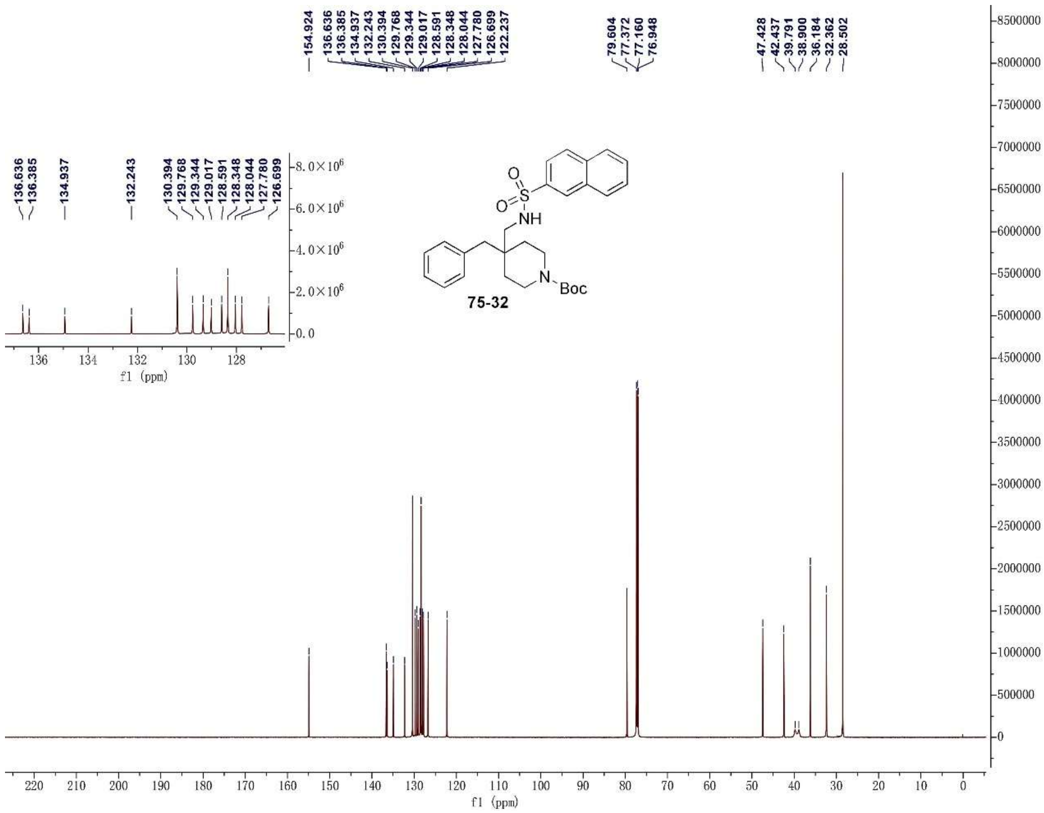

Figure S374: ^{13}C NMR spectrum of **75-32**

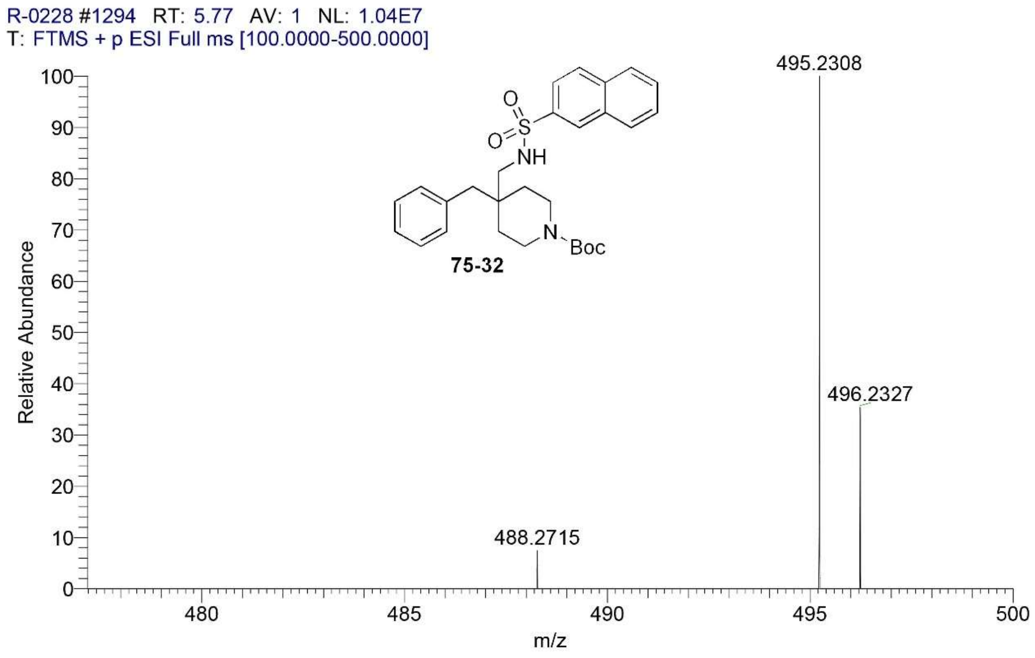

Figure S375: HR-MS (ESI/ion trap) spectrum of **75-32**

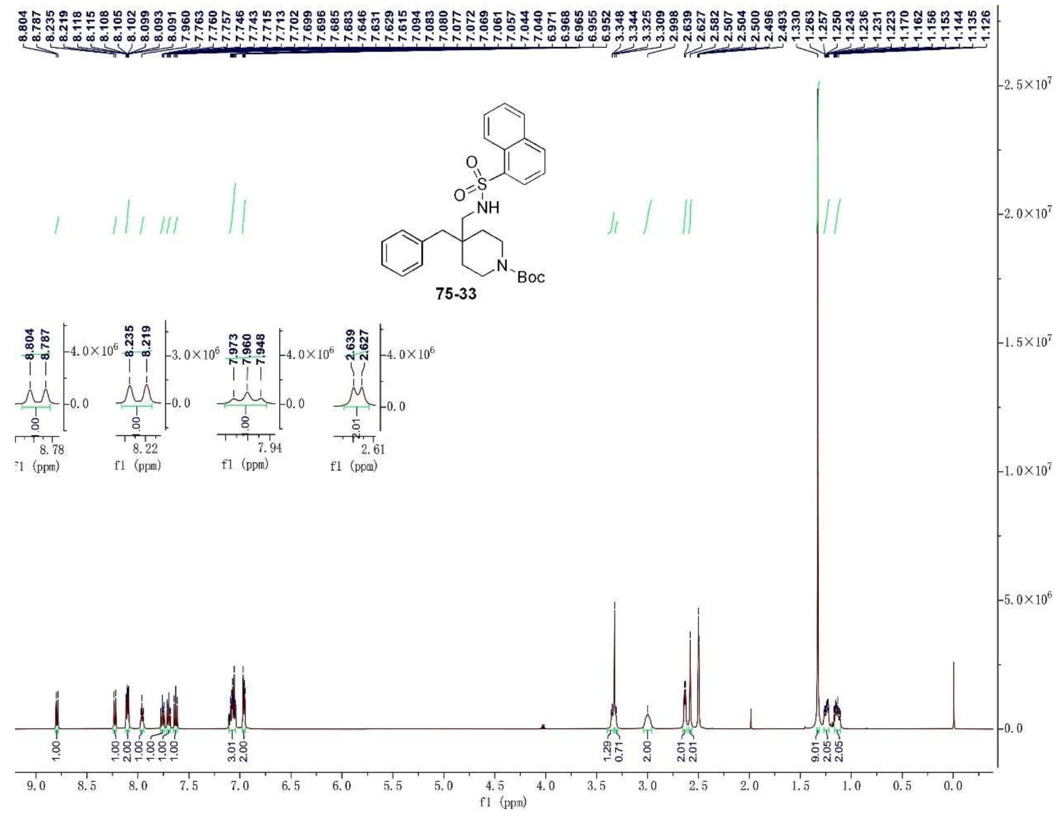

Figure S376: ^{1}H NMR spectrum of **75-33**

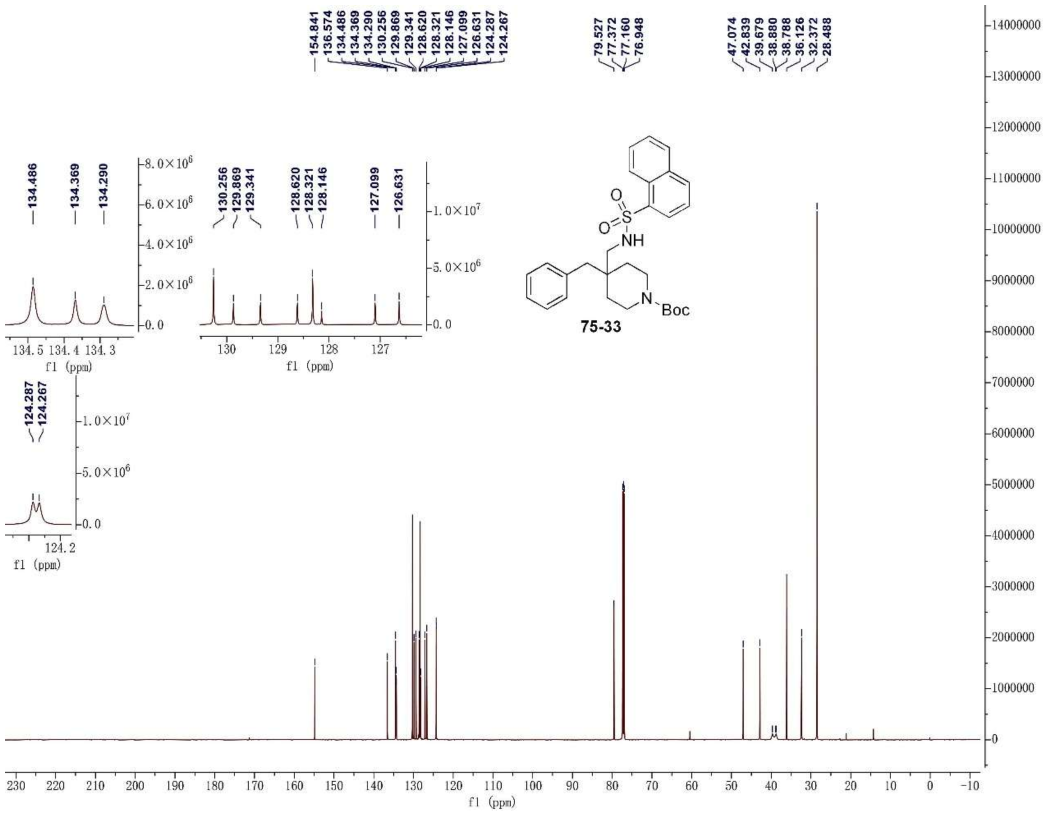

Figure S377: ^{13}C NMR spectrum of **75-33**

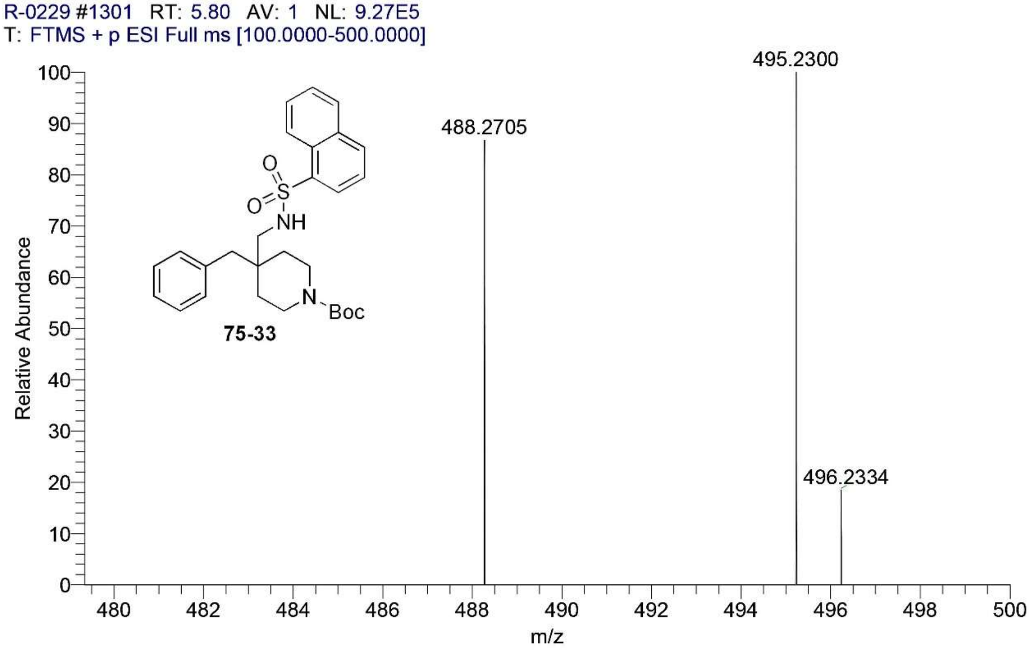

Figure S378: HR-MS (ESI/ion trap) spectrum of **75-33**

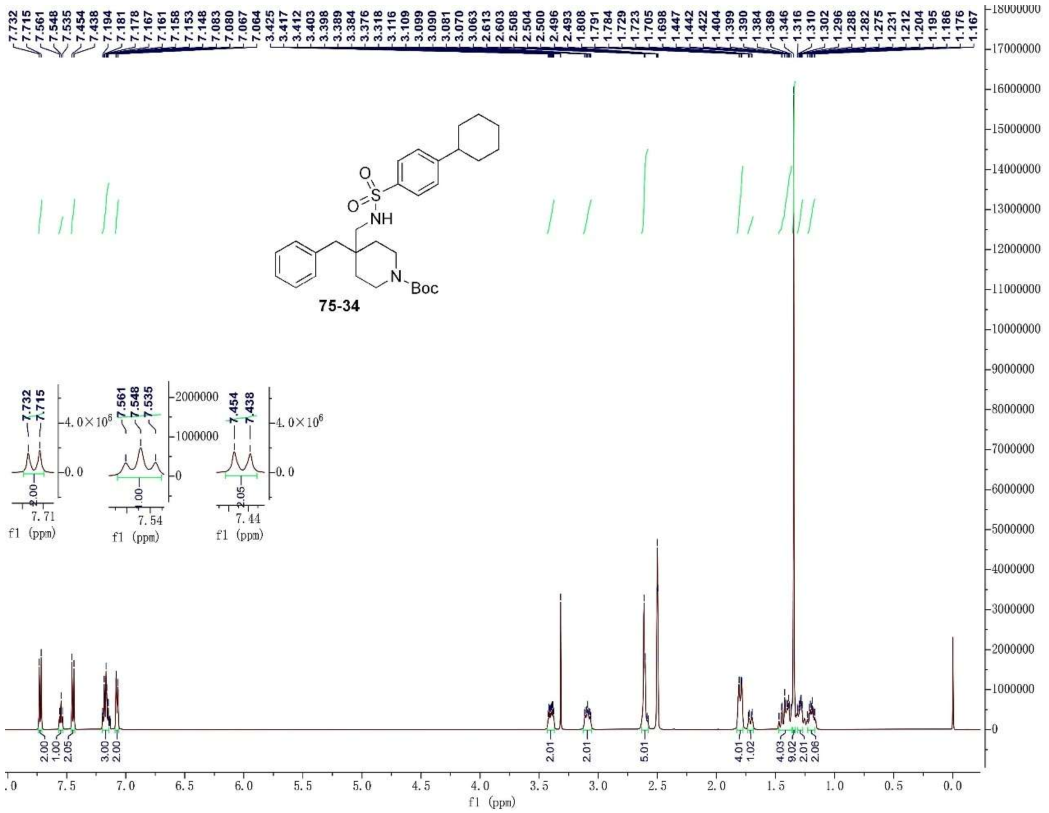

Figure S379: ^{1}H NMR spectrum of **75-34**

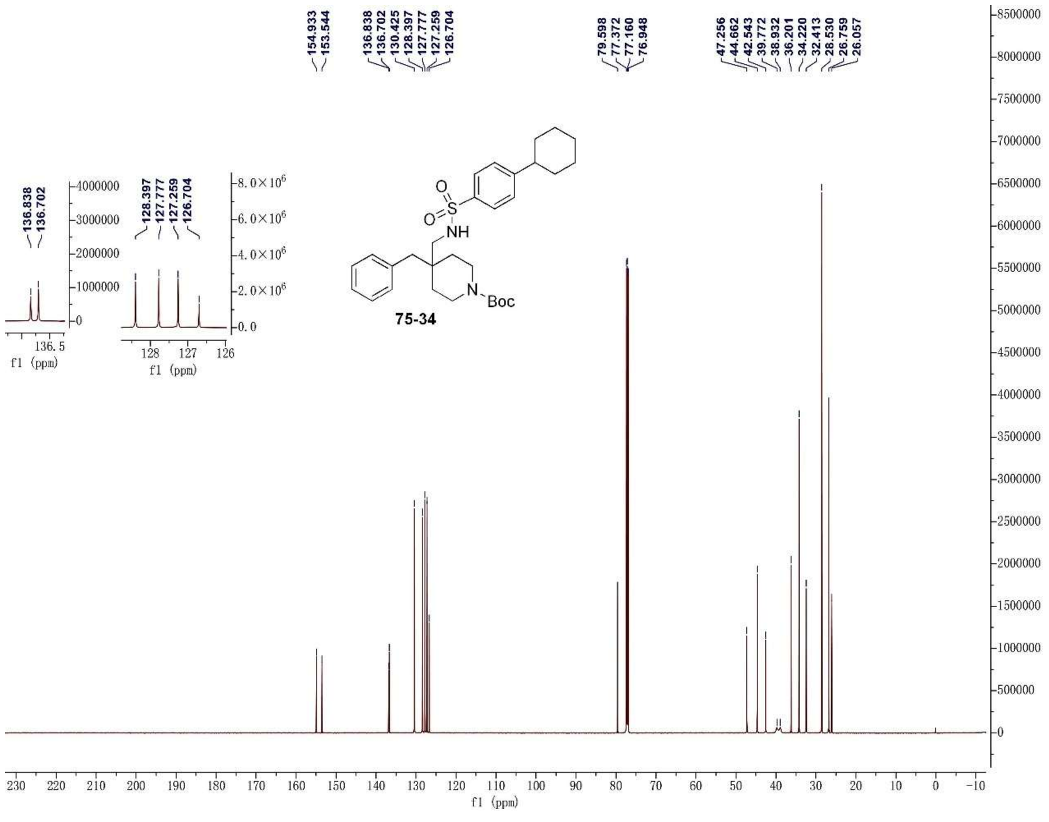

Figure S380: ^{13}C NMR spectrum of **75-34**

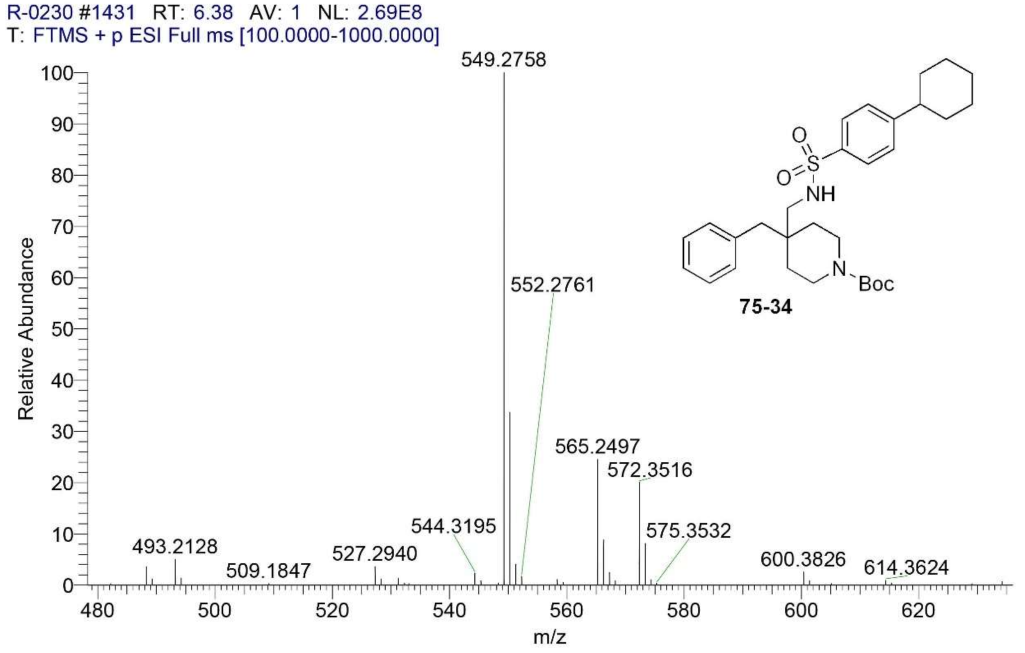

Figure S381: HR-MS (ESI/ion trap) spectrum of **75-34**

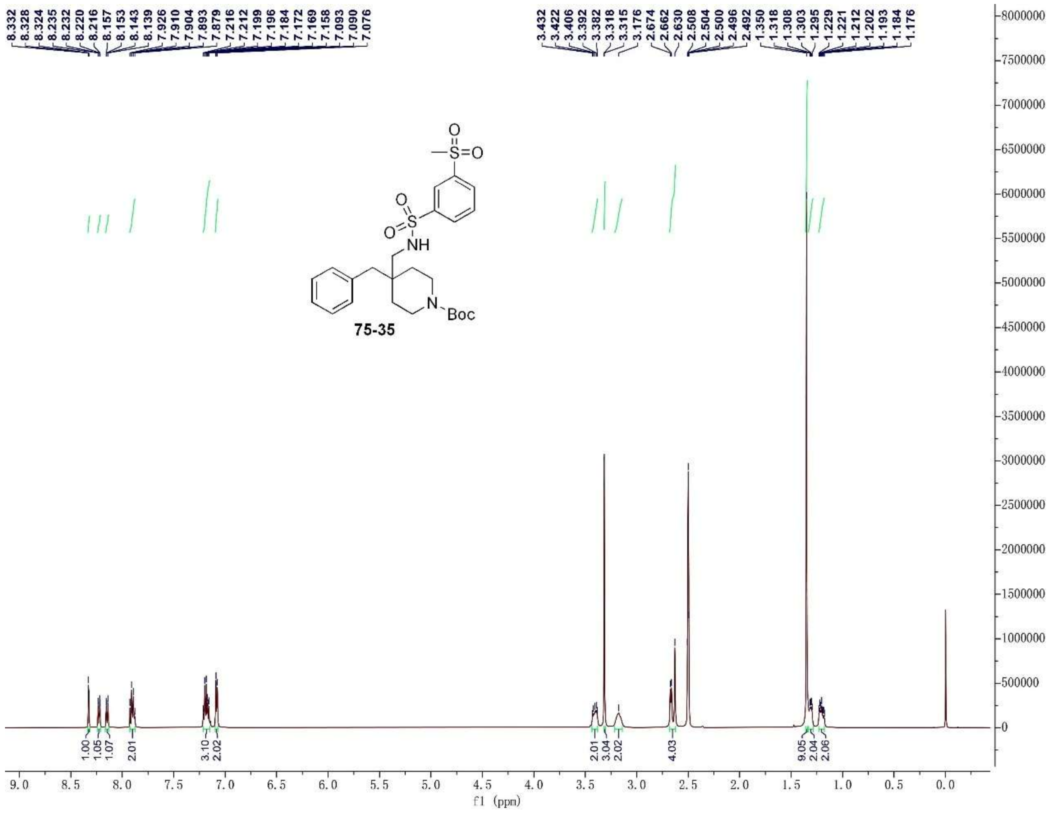

Figure S382: ^{1}H NMR spectrum of **75-35**

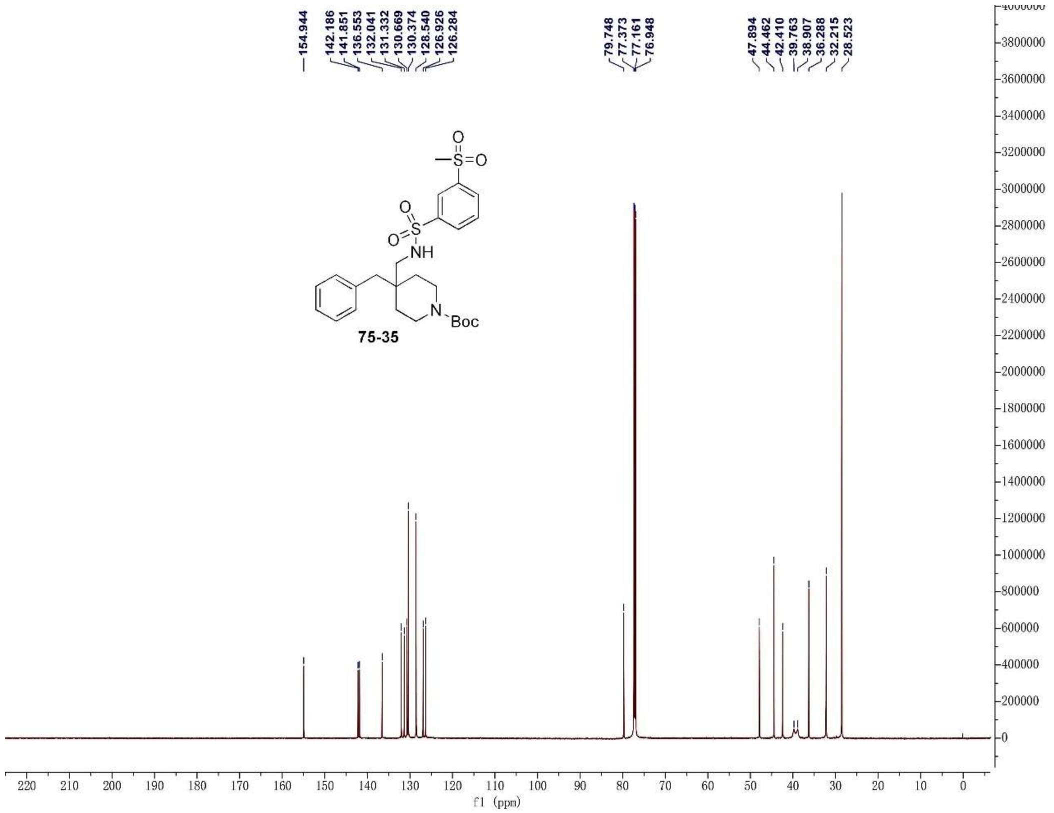

Figure S383: ^{13}C NMR spectrum of **75-35**

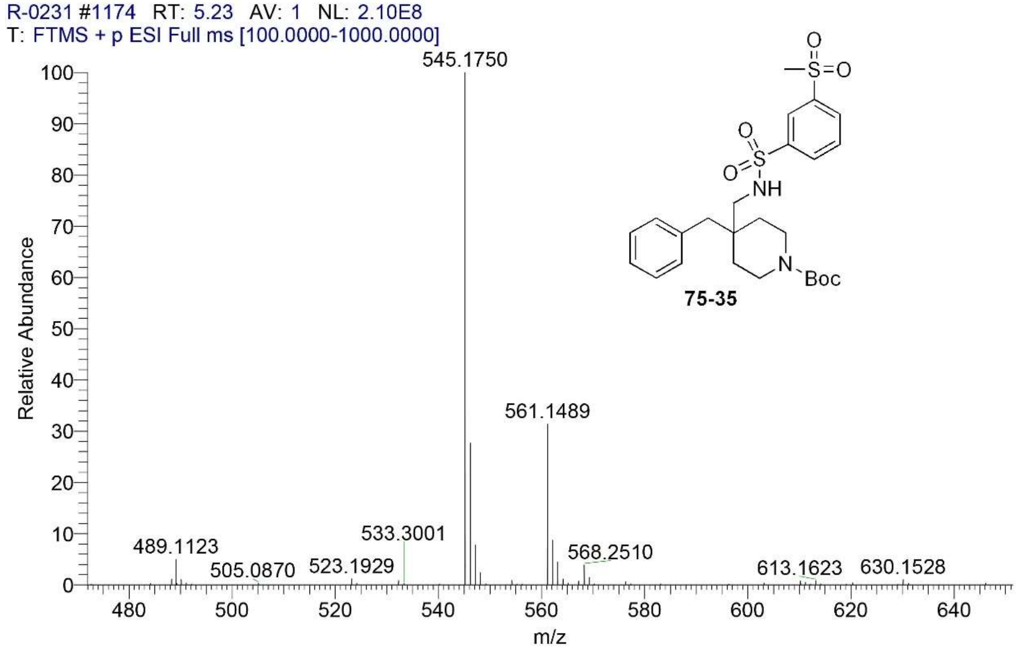

Figure S384: HR-MS (ESI/ion trap) spectrum of **75-35**

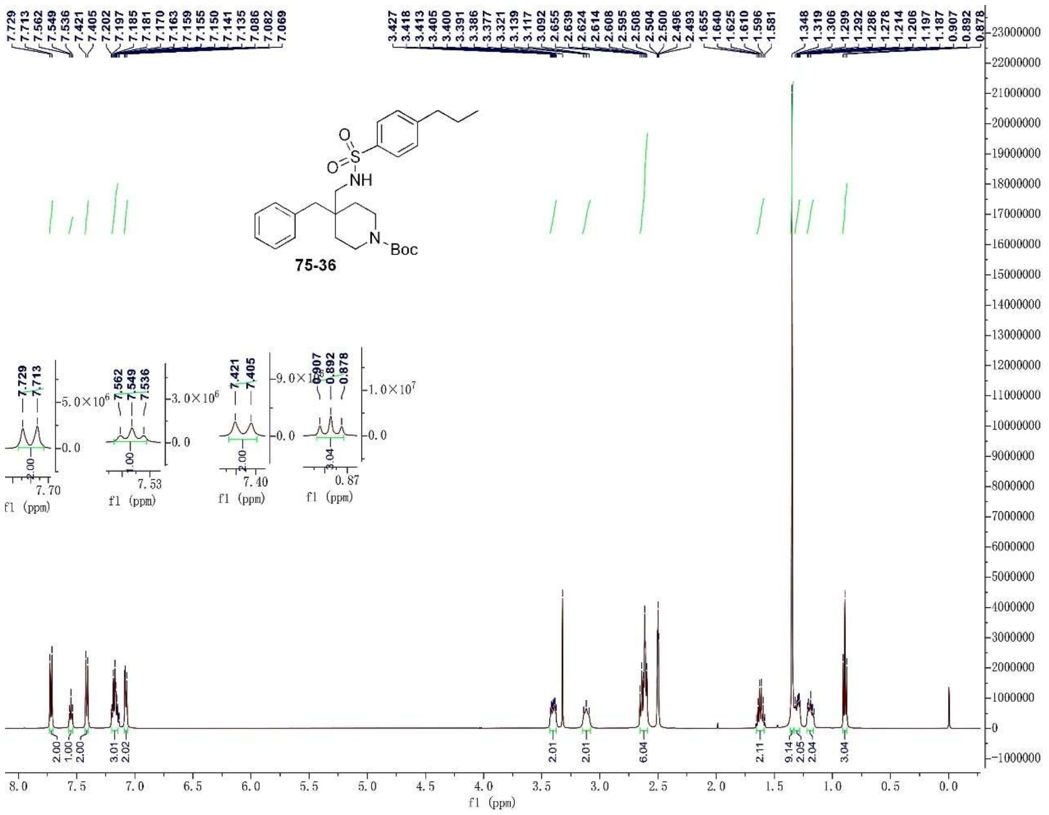

Figure S385: ^{1}H NMR spectrum of **75-36**

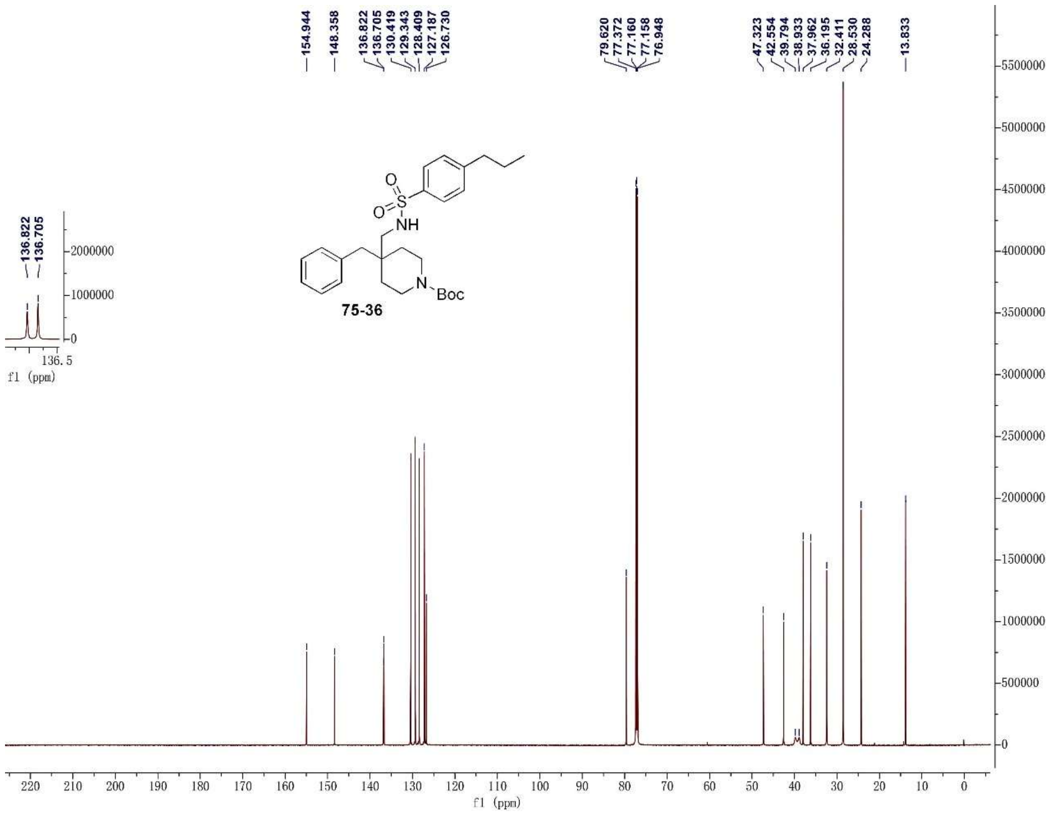

Figure S386: ^{13}C NMR spectrum of **75-36**

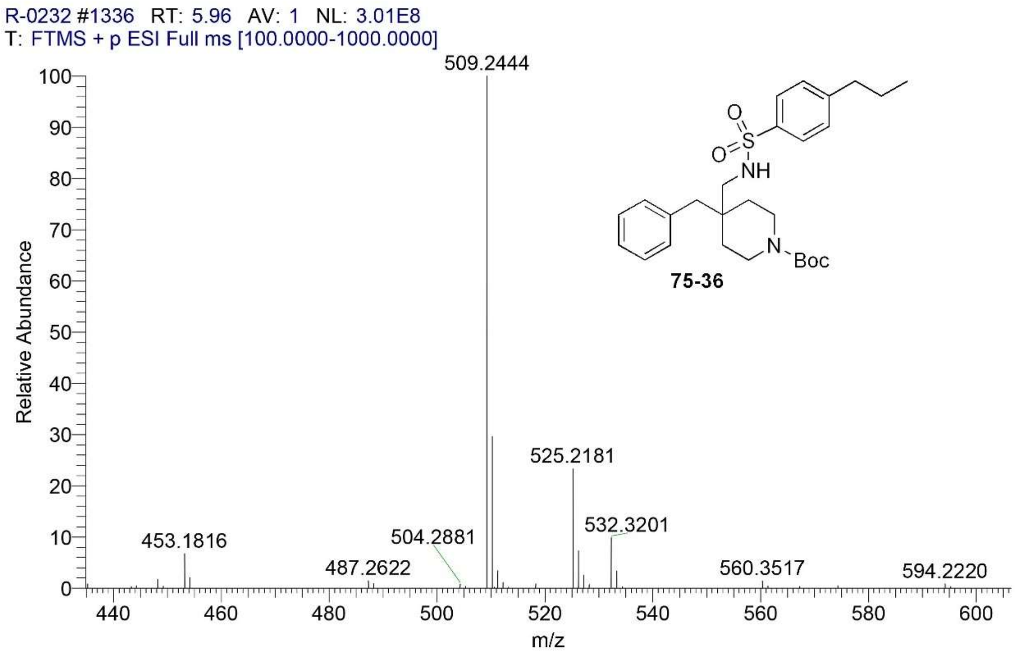

Figure S387: HR-MS (ESI/ion trap) spectrum of **75-36**

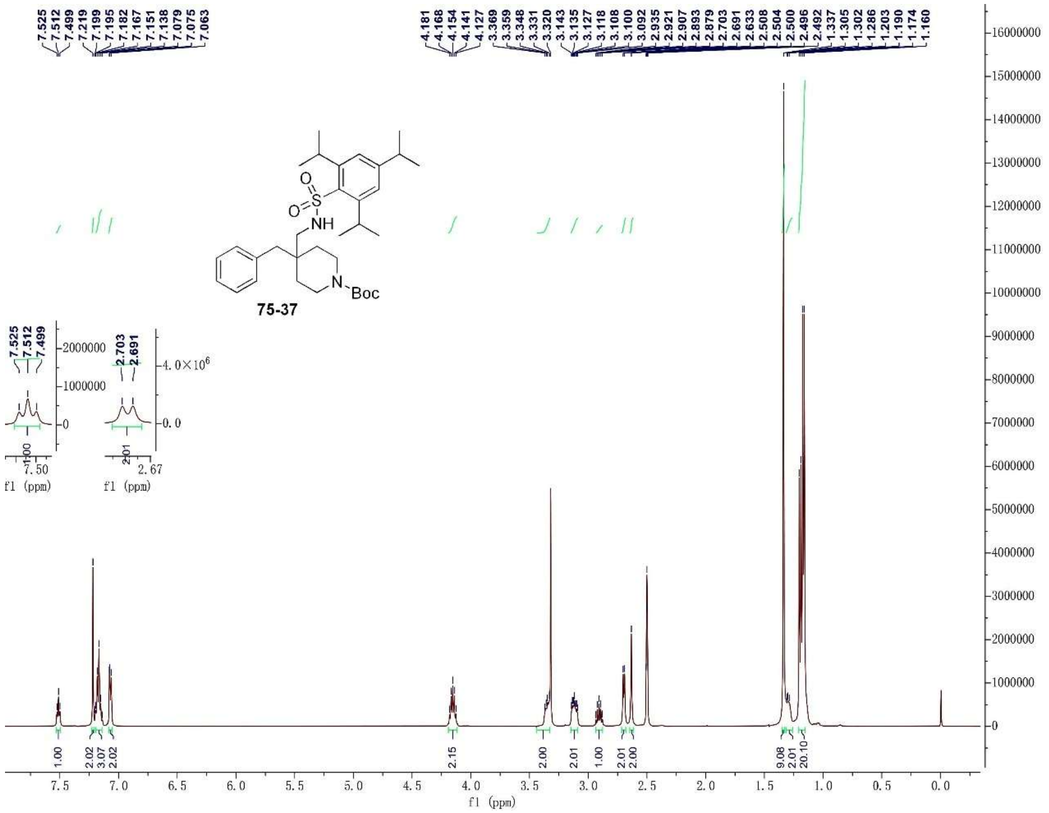

Figure S388: ^{1}H NMR spectrum of **75-37**

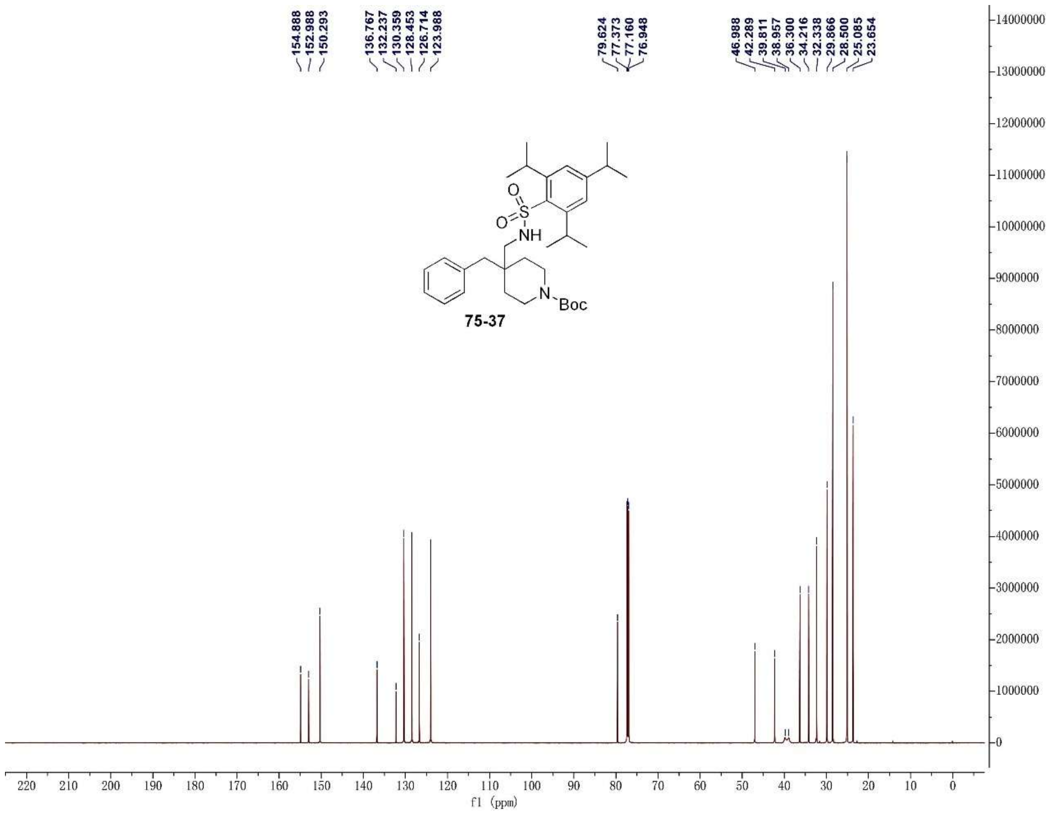

Figure S389: ^{13}C NMR spectrum of **75-37**

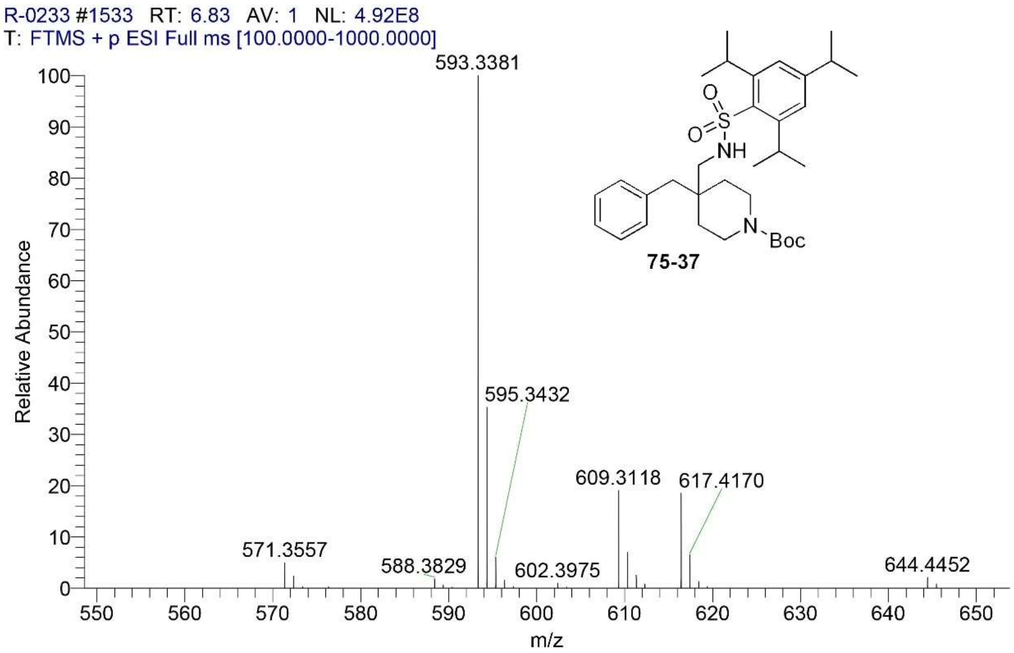

Figure S390: HR-MS (ESI/ion trap) spectrum of **75-37**

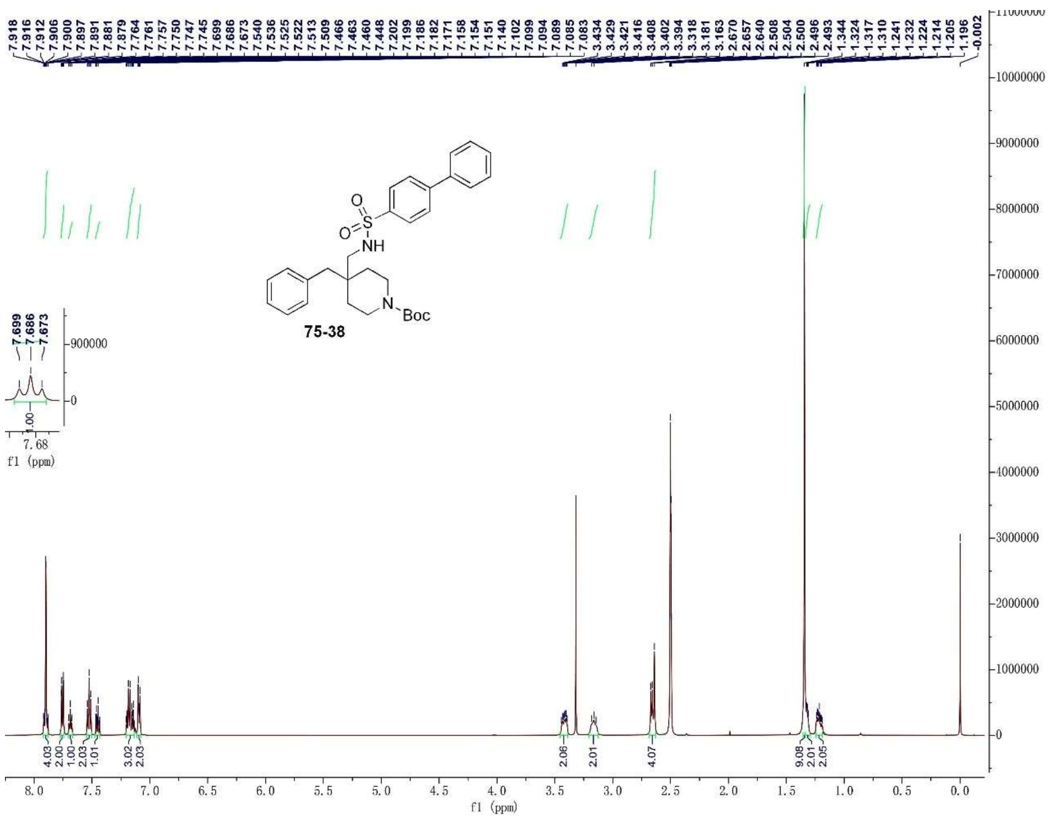

Figure S391: ^{1}H NMR spectrum of **75-38**

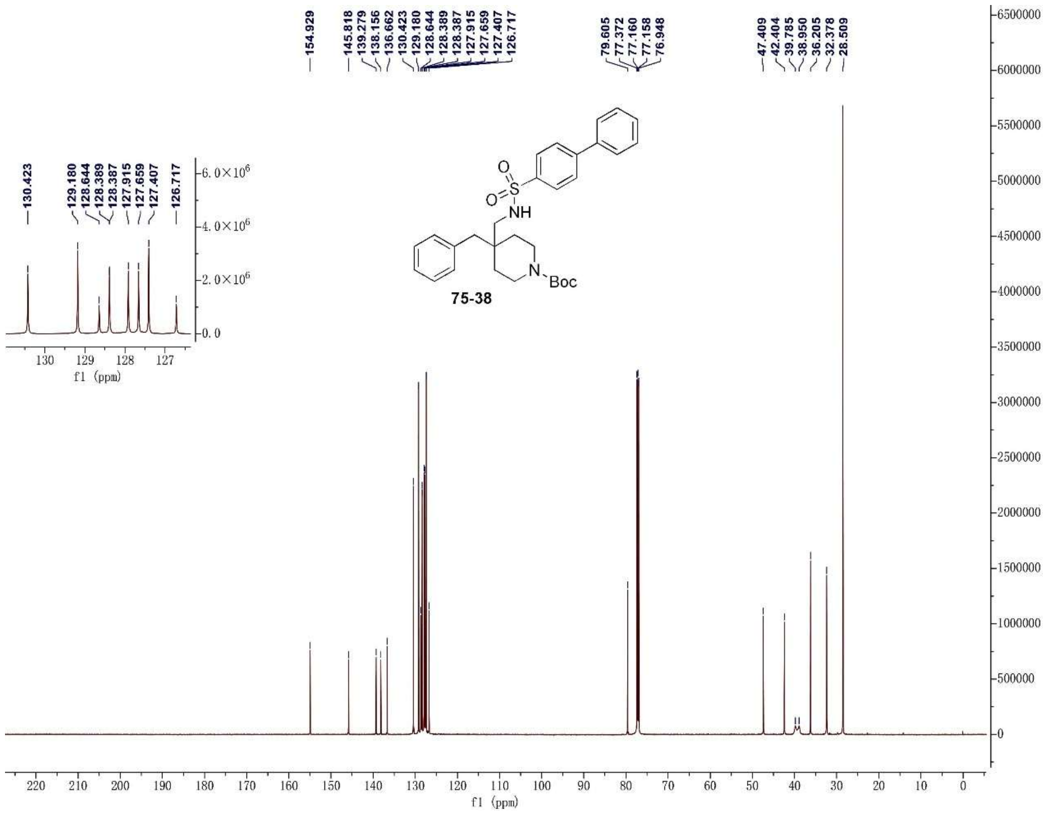

Figure S392: ^{13}C NMR spectrum of **75-38**

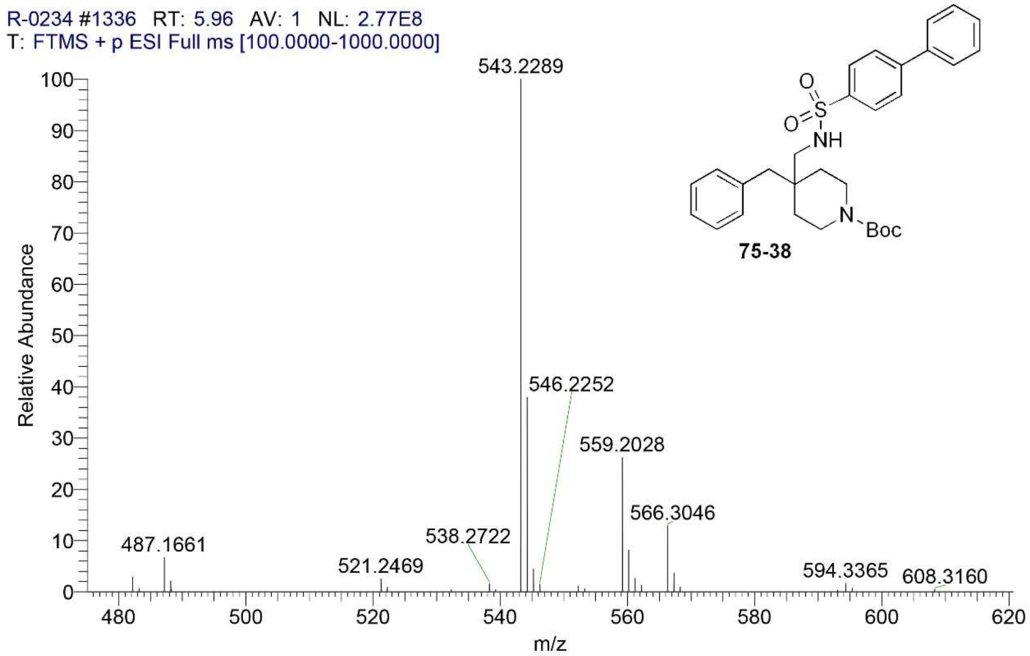

Figure S393: HR-MS (ESI/ion trap) spectrum of **75-38**

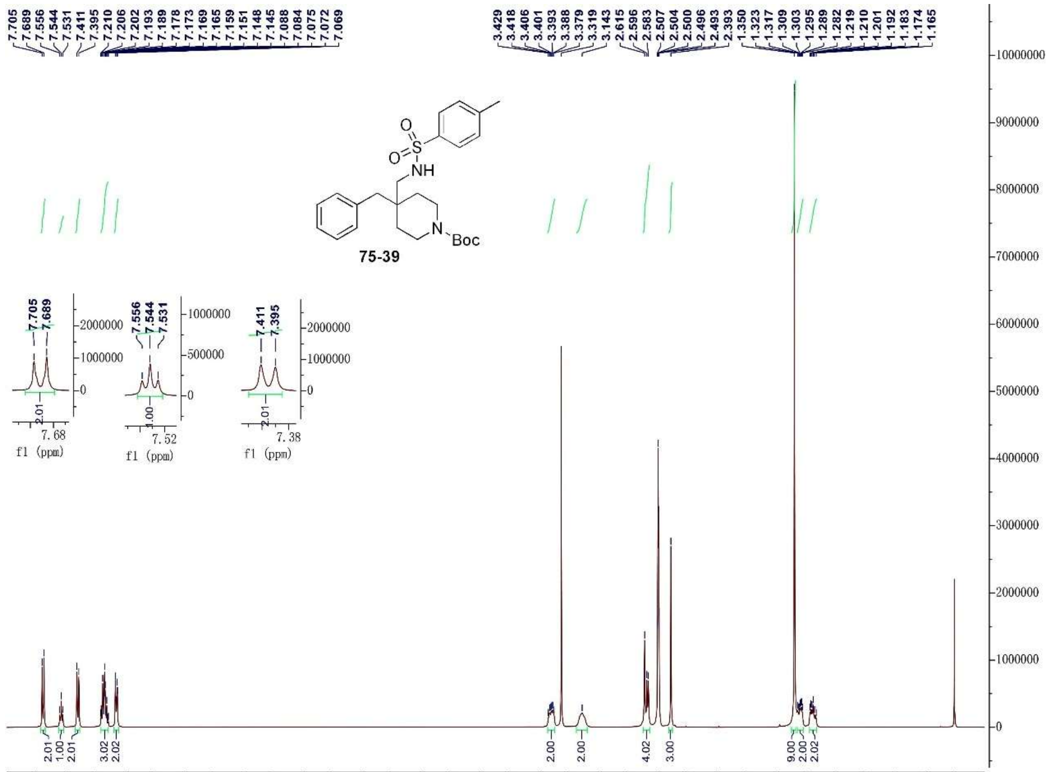

Figure S394: ^{1}H NMR spectrum of **75-39**

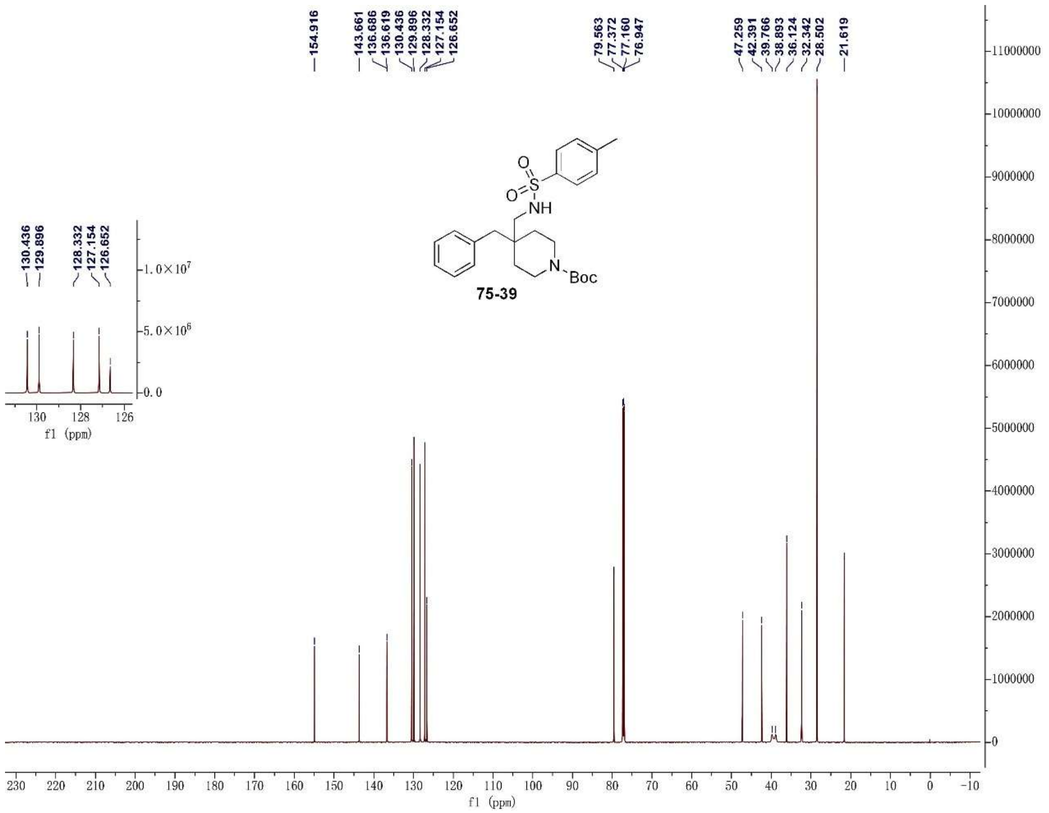

Figure S395: ^{13}C NMR spectrum of **75-39**

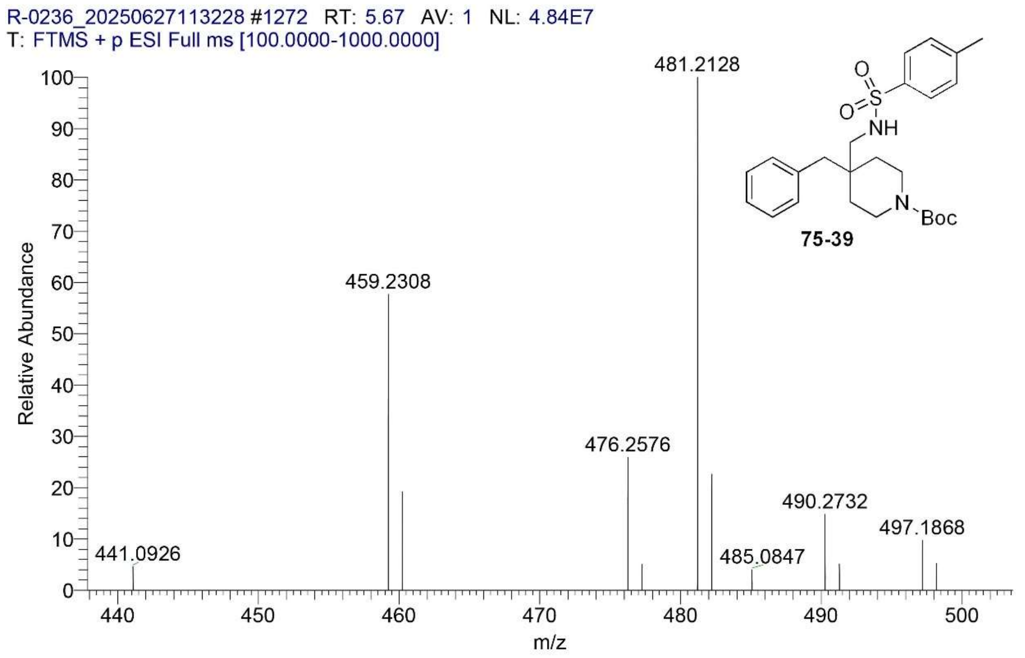

Figure S396: HR-MS (ESI/ion trap) spectrum of **75-39**

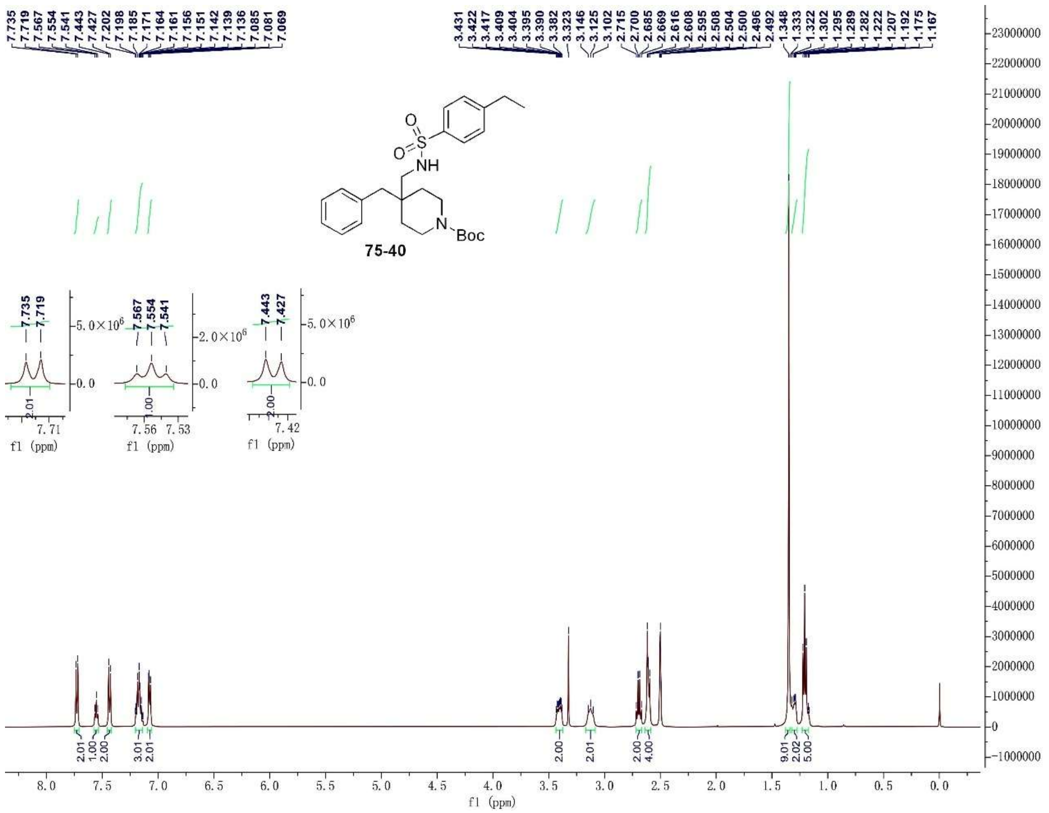

Figure S397: ^{1}H NMR spectrum of **75-40**

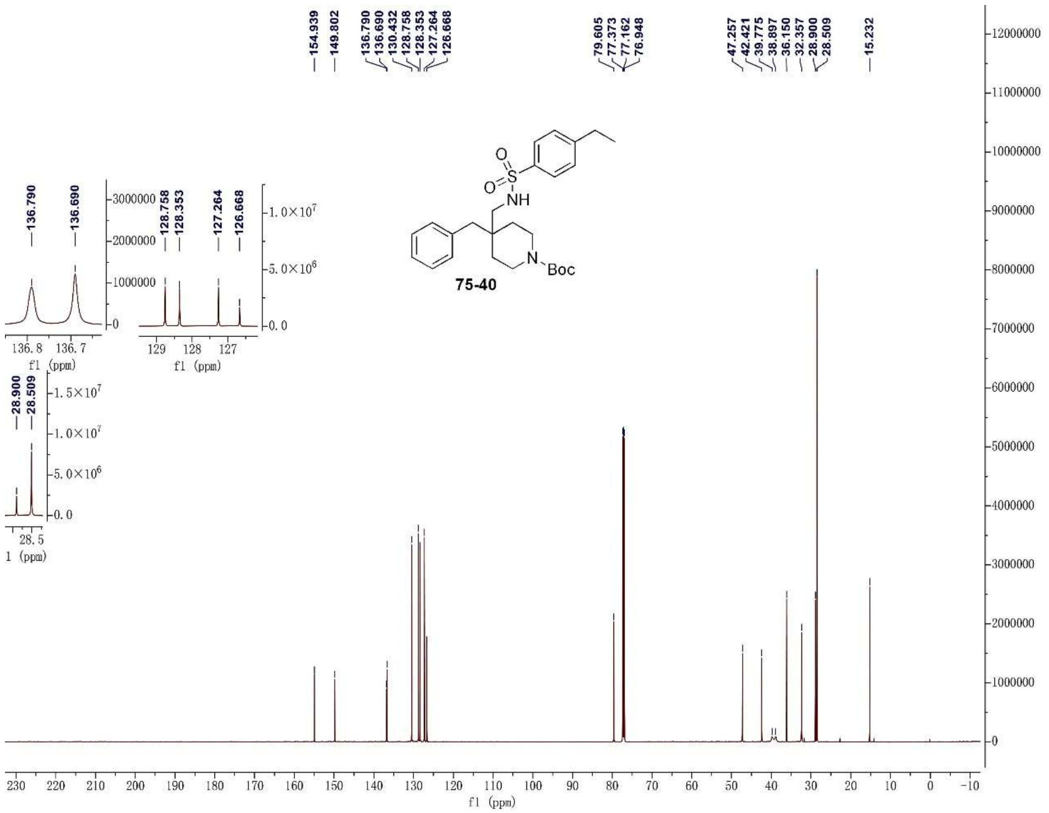

Figure S398: ^{13}C NMR spectrum of **75-40**

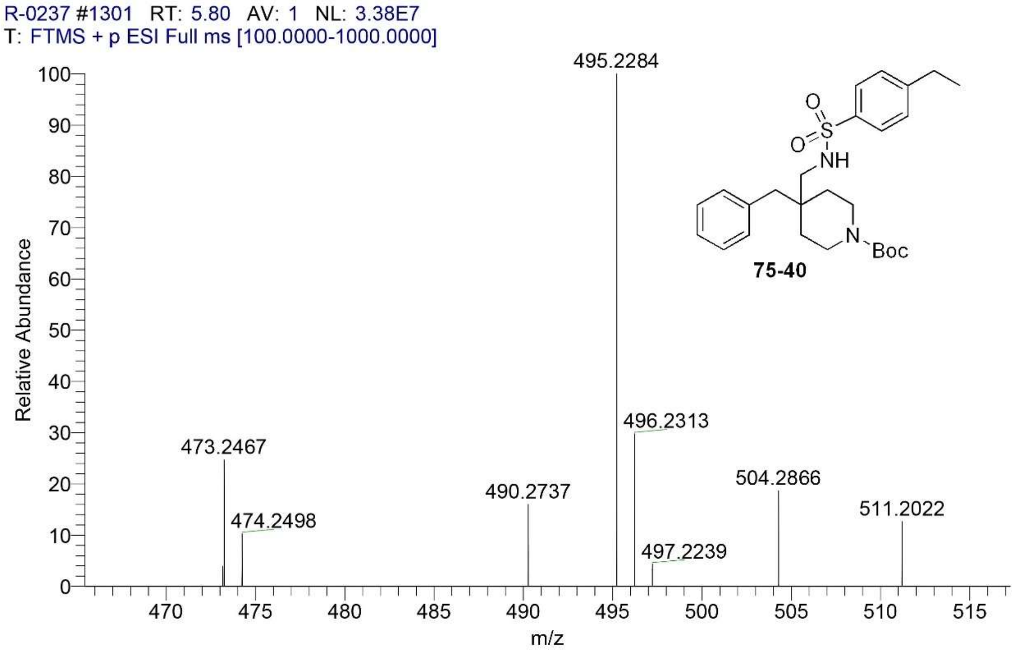

Figure S399: HR-MS (ESI/ion trap) spectrum of **75-40**

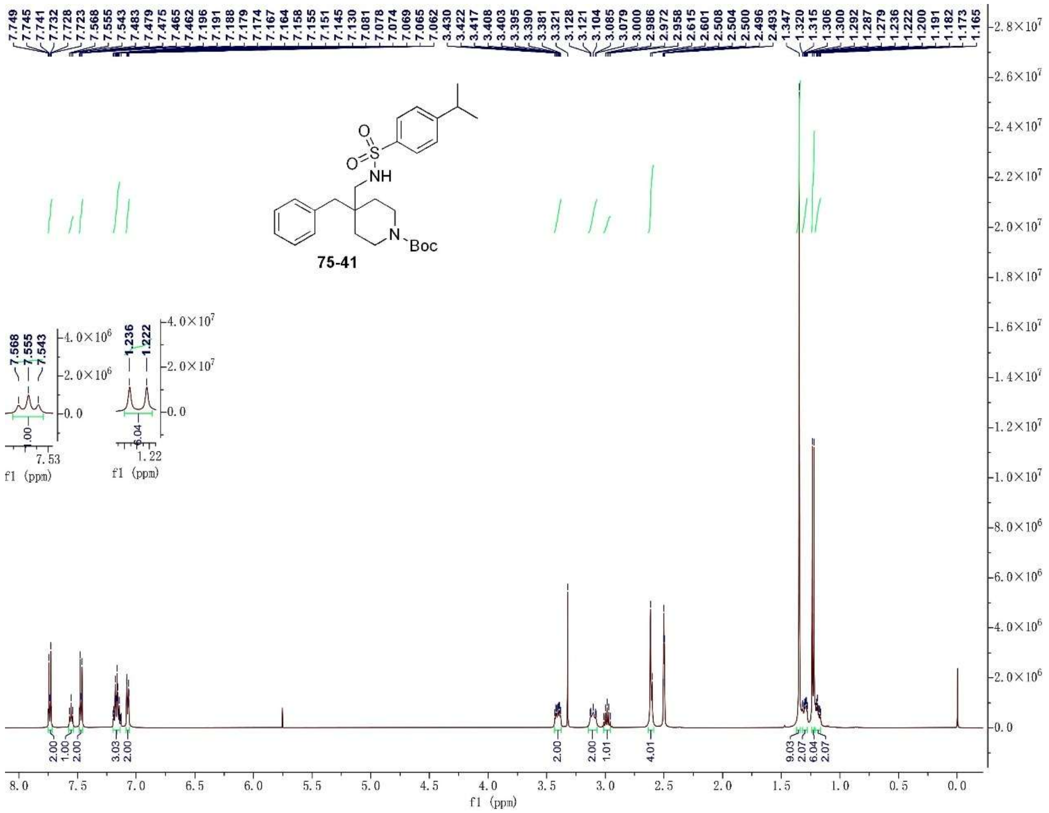

Figure S400: ^{1}H NMR spectrum of **75-41**

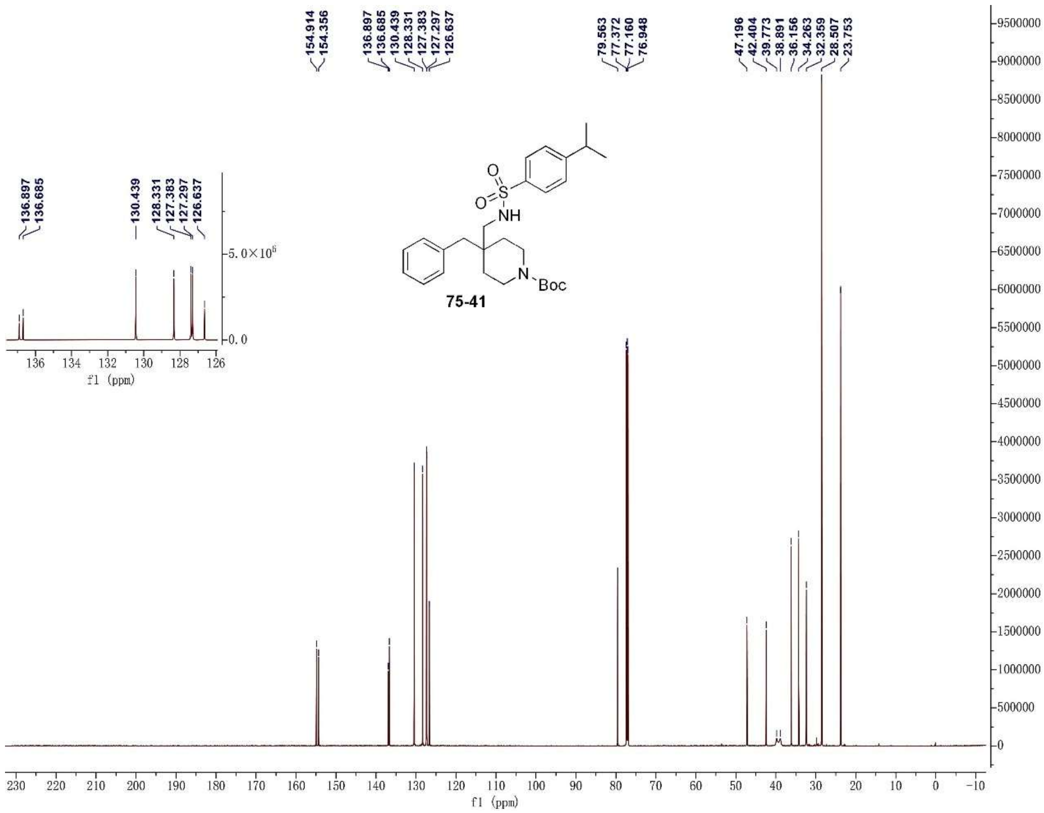

Figure S401: ^{13}C NMR spectrum of **75-41**

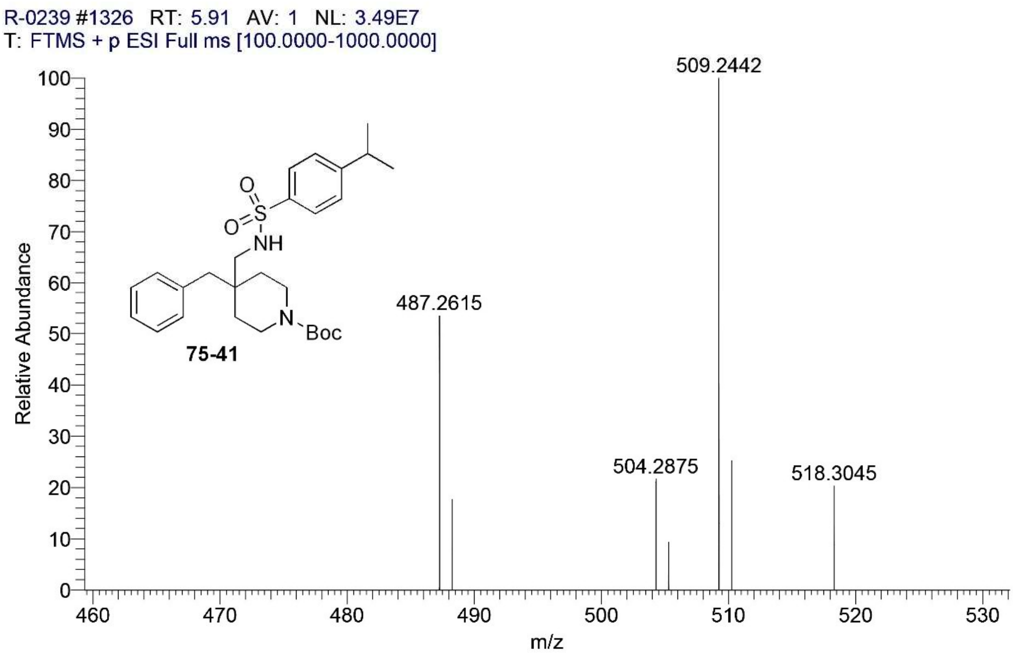

Figure S402: HR-MS (ESI/ion trap) spectrum of **75-41**

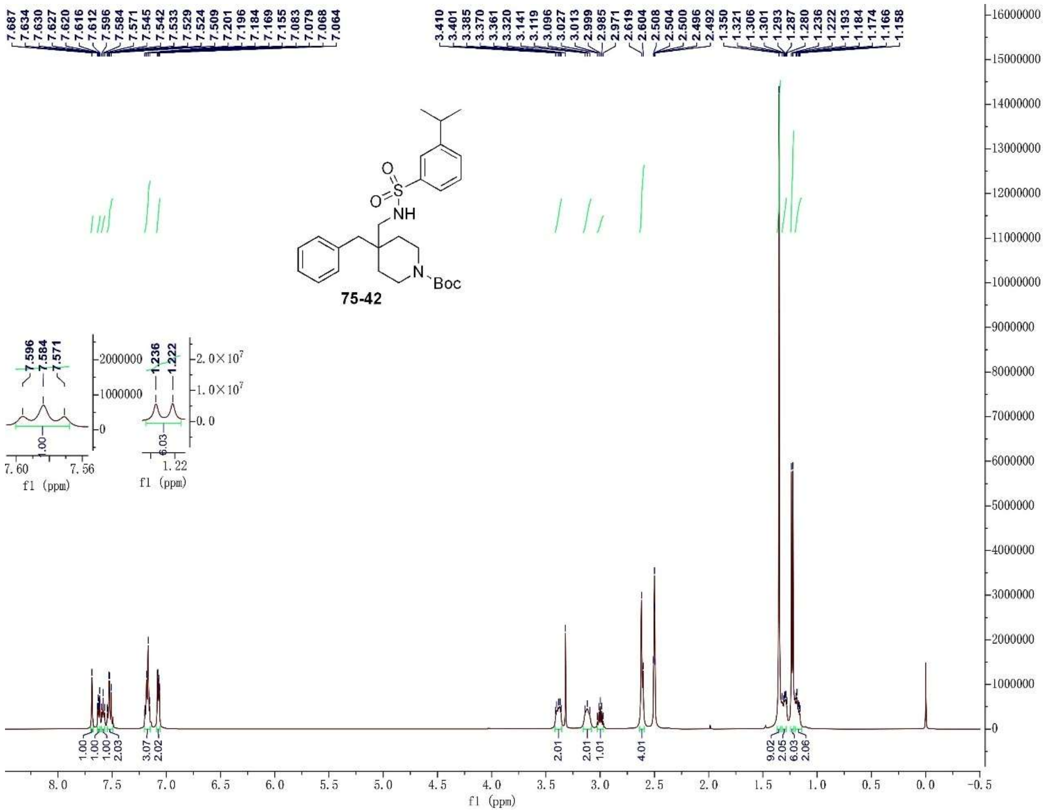

Figure S403: ^{1}H NMR spectrum of **75-42**

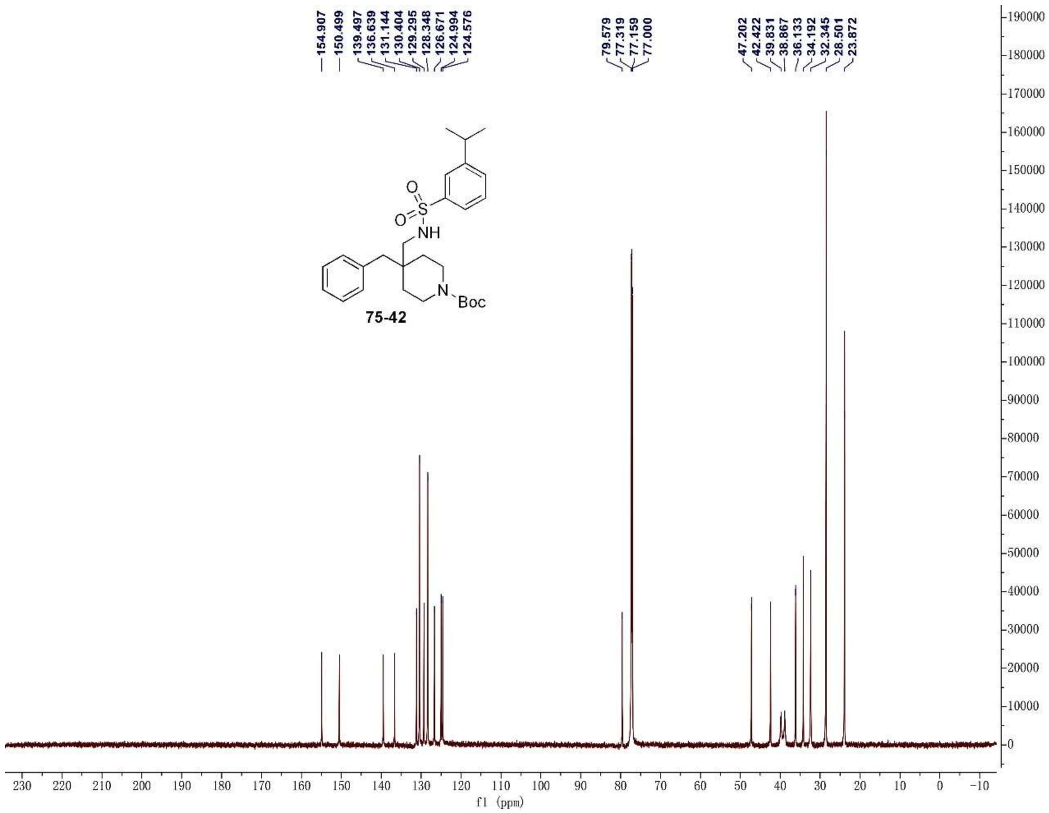

Figure S404: ^{13}C NMR spectrum of **75-42**

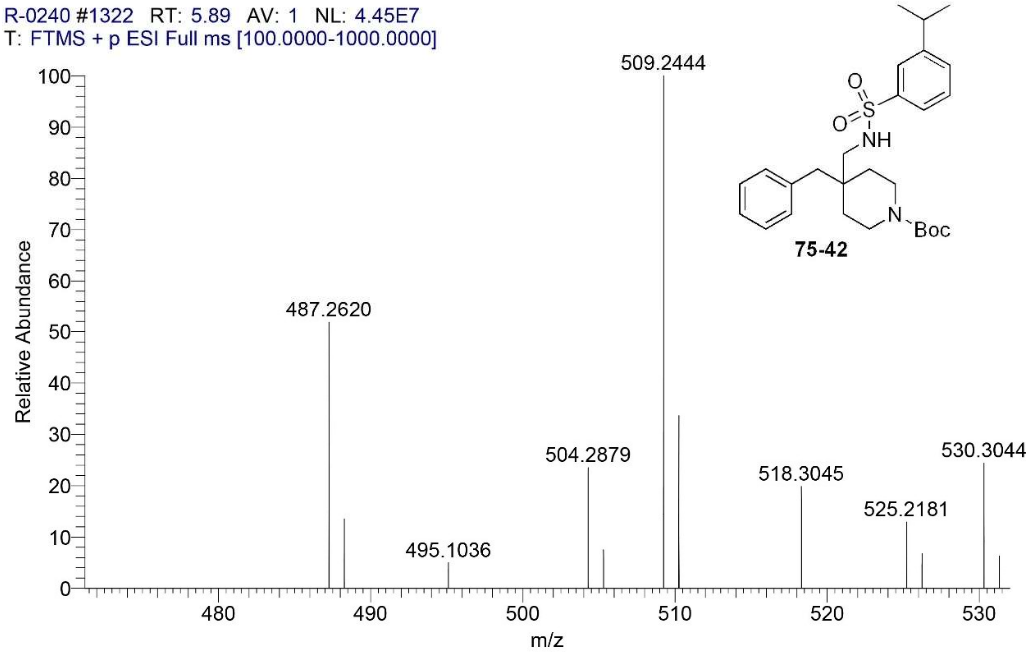

Figure S405: HR-MS (ESI/ion trap) spectrum of **75-42**

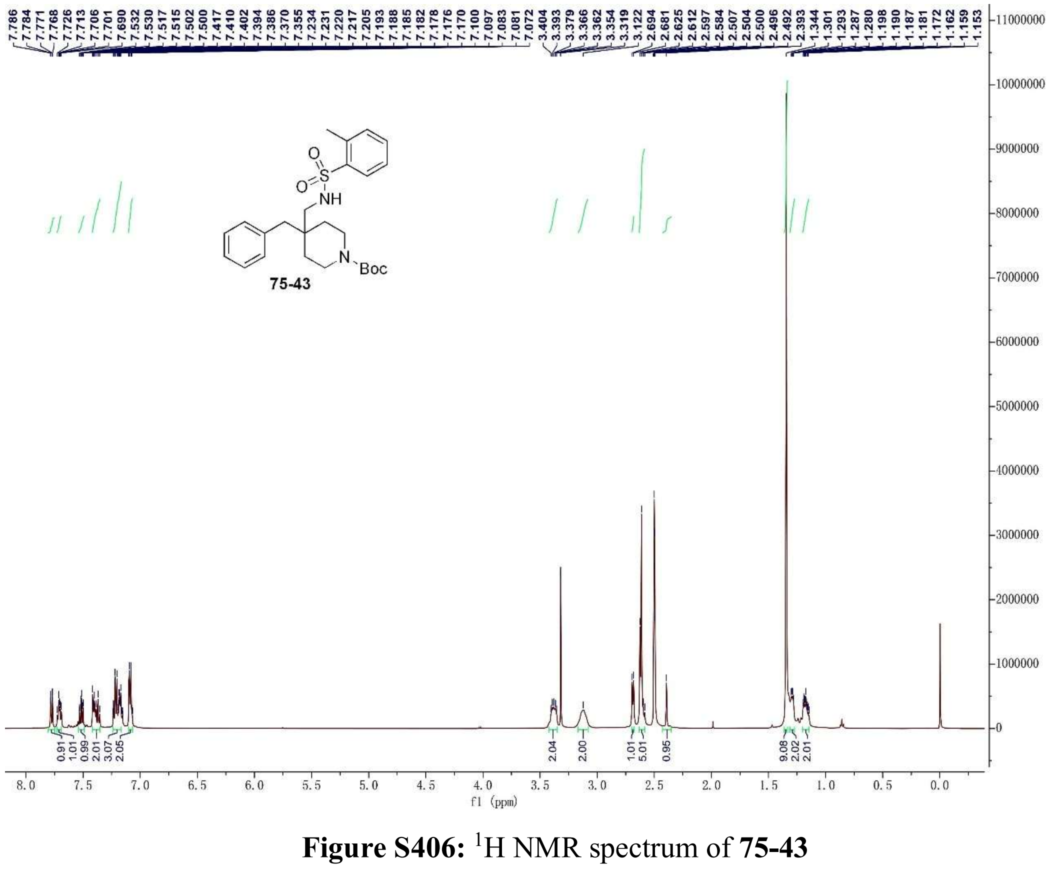

Figure S406: ^{1}H NMR spectrum of **75-43**

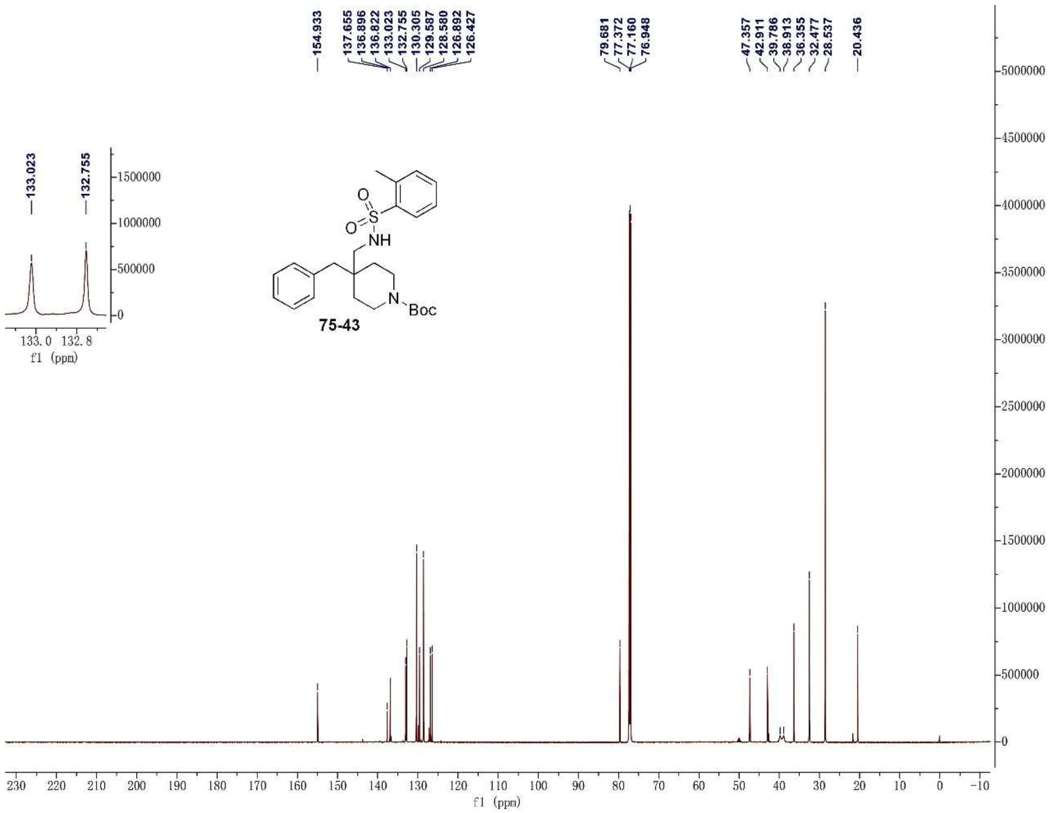

Figure S407: ^{13}C NMR spectrum of **75-43**

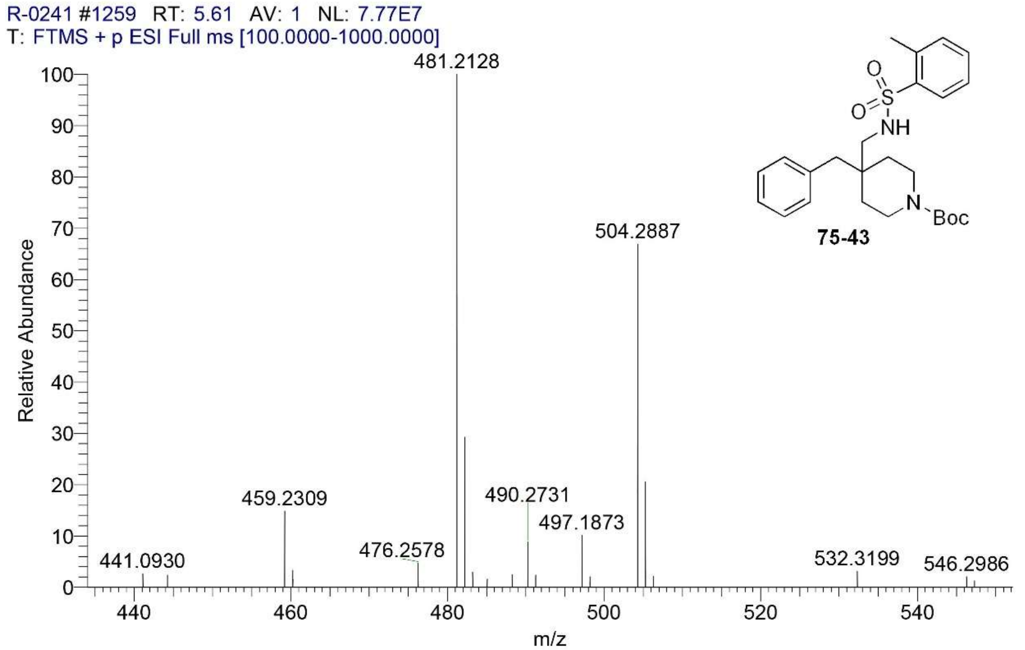

Figure S408: HR-MS (ESI/ion trap) spectrum of **75-43**

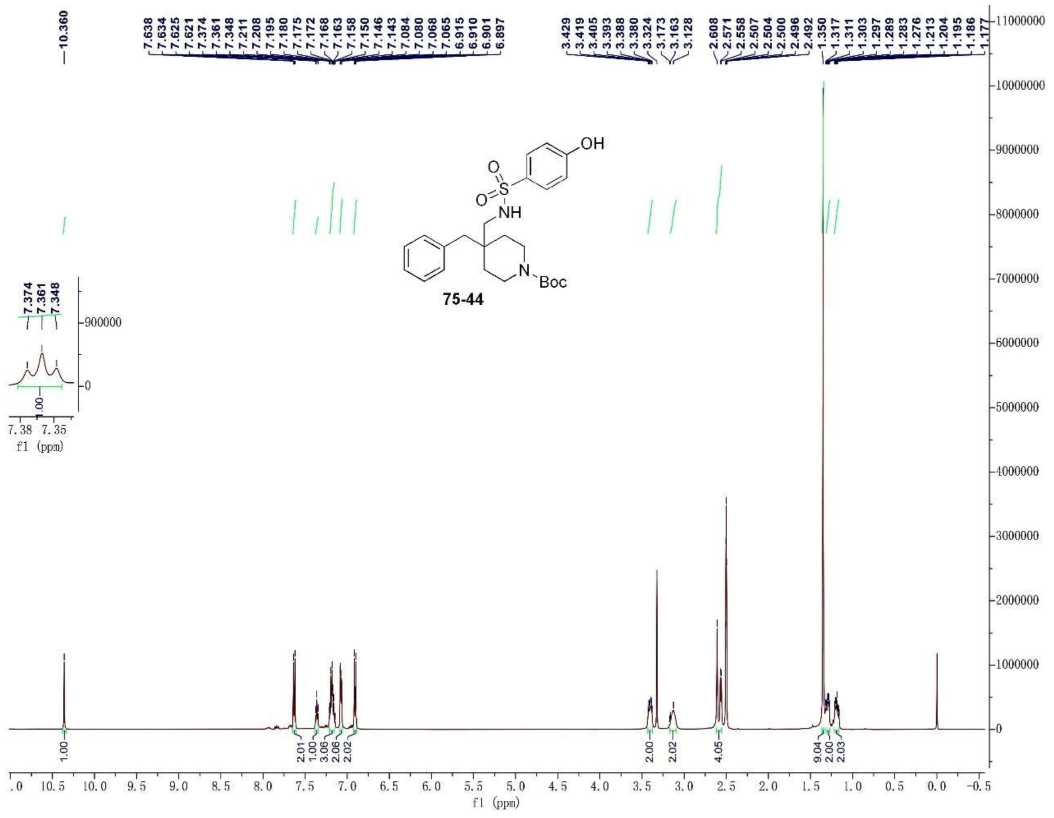

Figure S409: ^{1}H NMR spectrum of 75-44

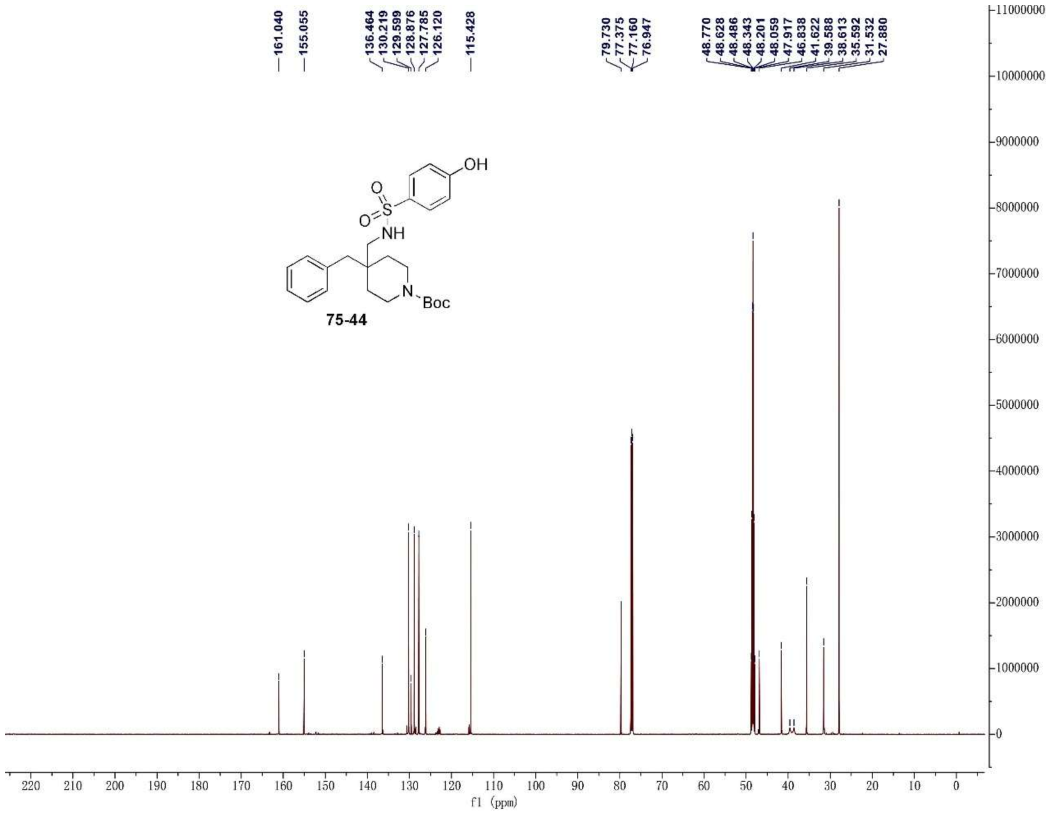

Figure S410: ^{13}C NMR spectrum of 75-44

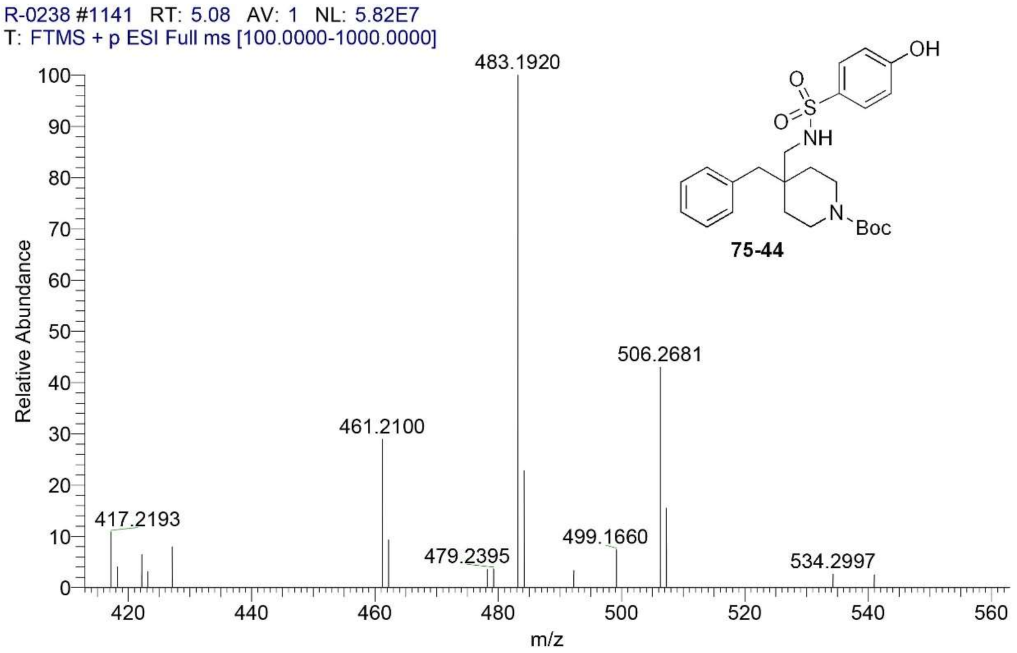

Figure S411: HR-MS (ESI/ion trap) spectrum of **75-44**

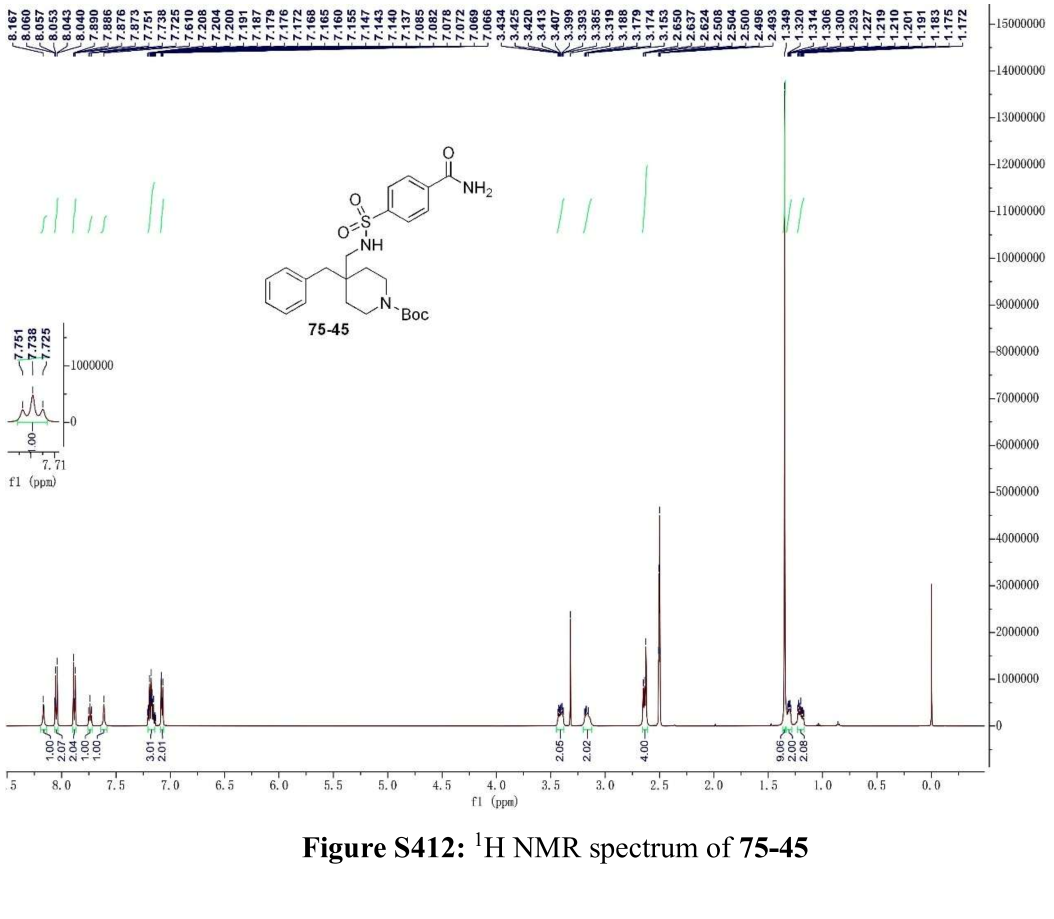

Figure S412: ^{1}H NMR spectrum of **75-45**

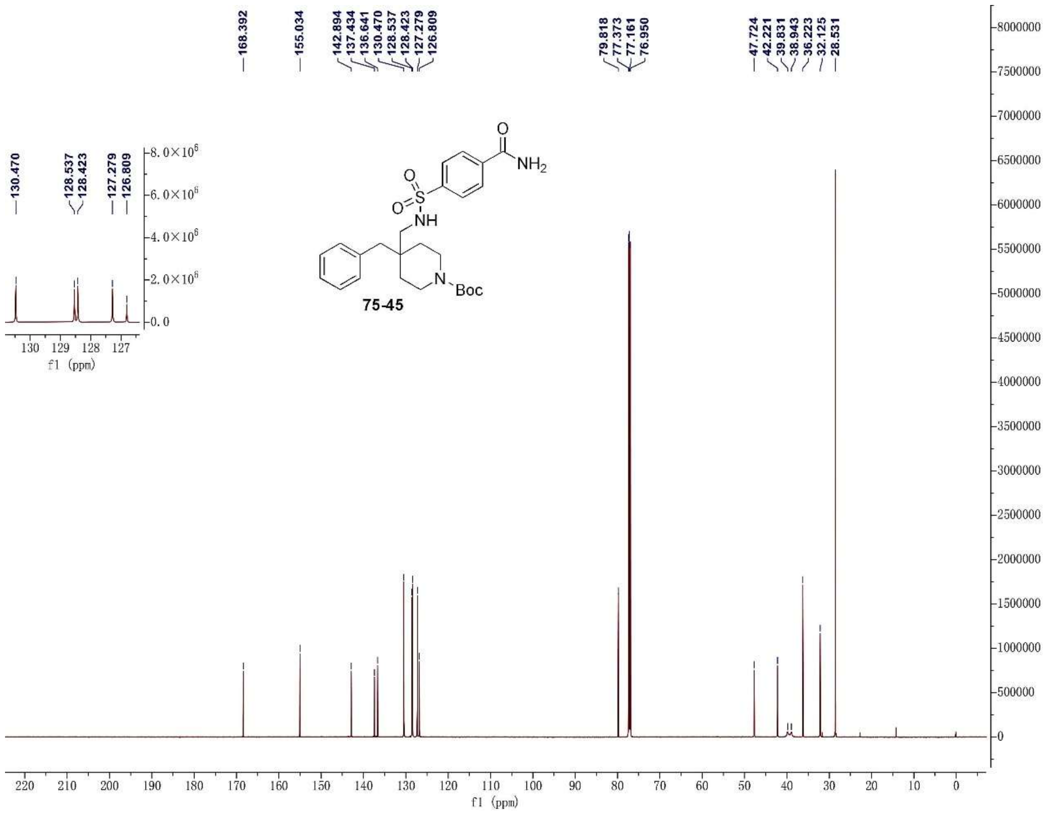

Figure S413: ^{13}C NMR spectrum of **75-45**

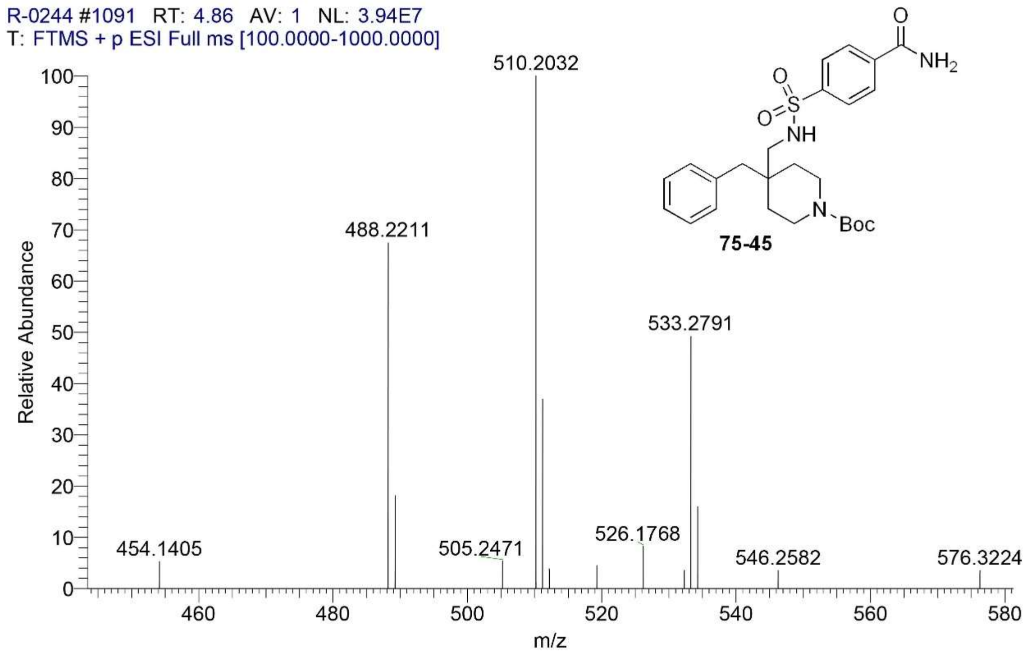

Figure S414: HR-MS (ESI/ion trap) spectrum of **75-45**

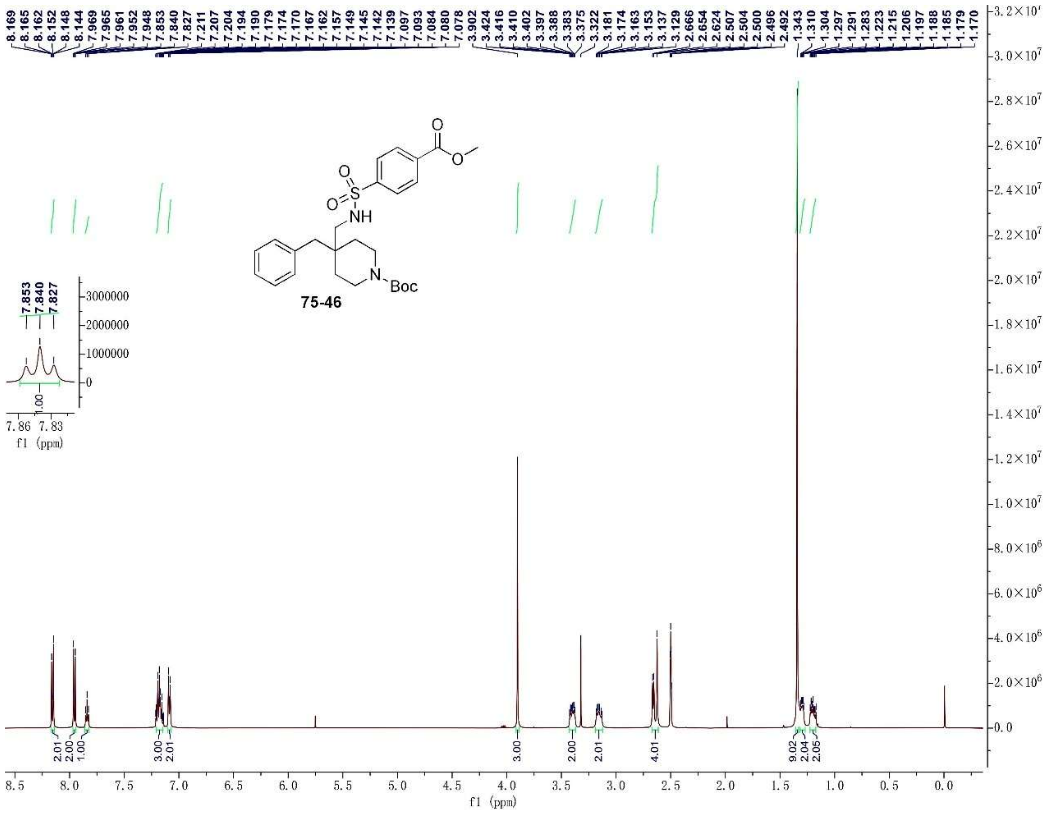

Figure S415: ^{1}H NMR spectrum of **75-46**

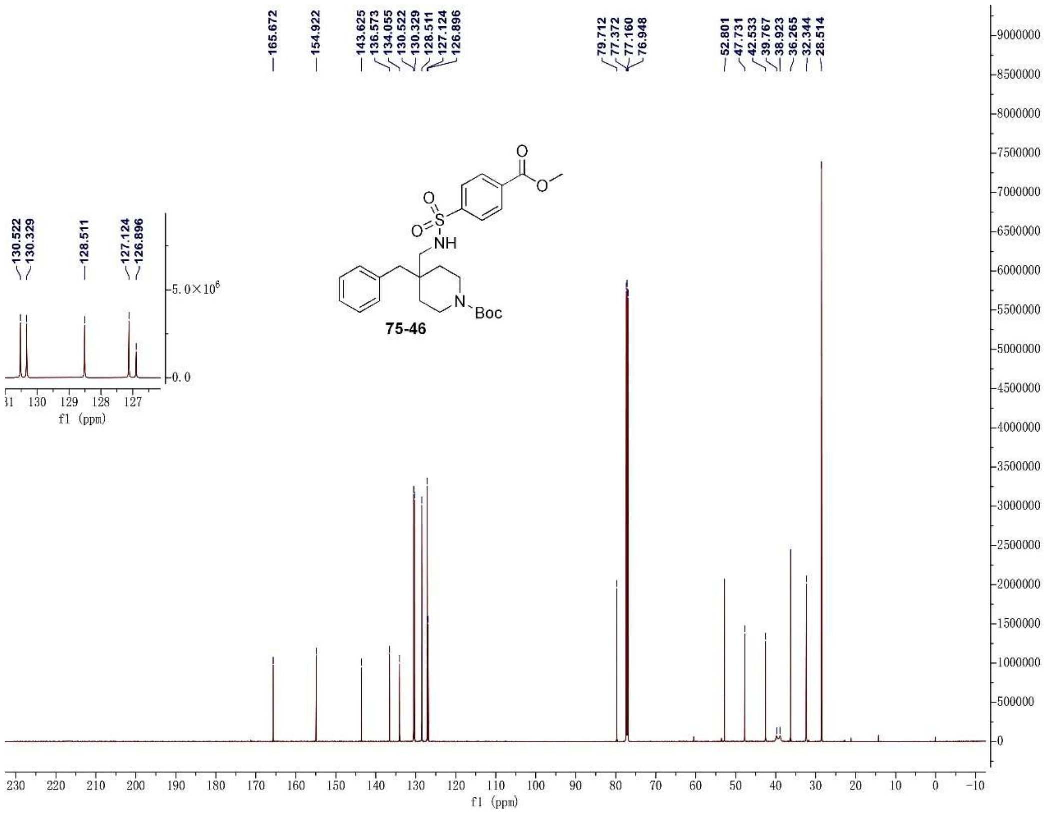

Figure S416: ^{13}C NMR spectrum of **75-46**

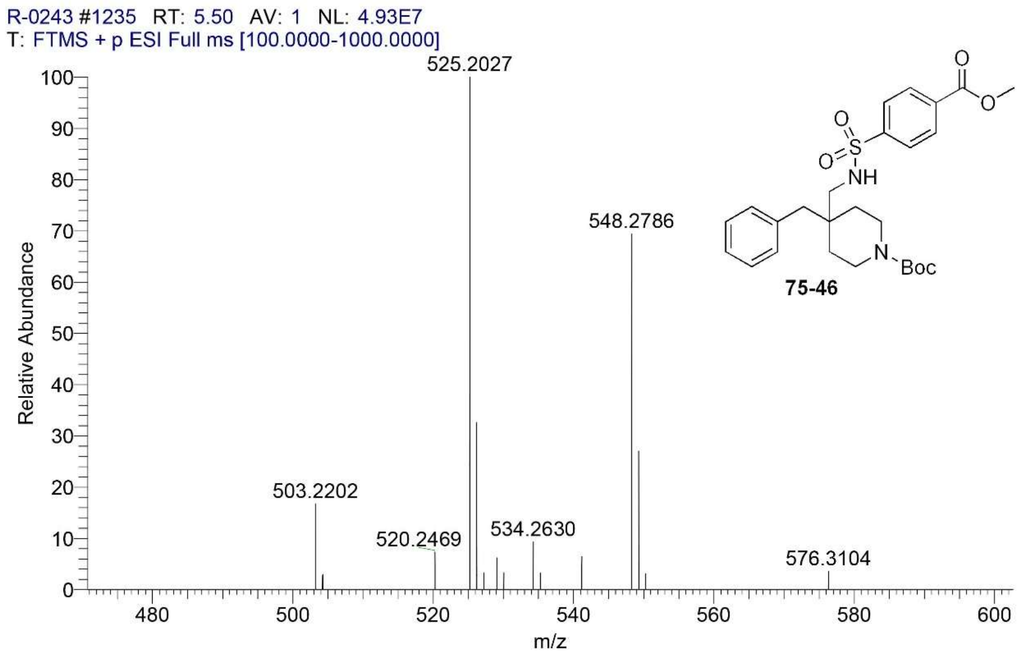

Figure S417: HR-MS (ESI/ion trap) spectrum of **75-46**

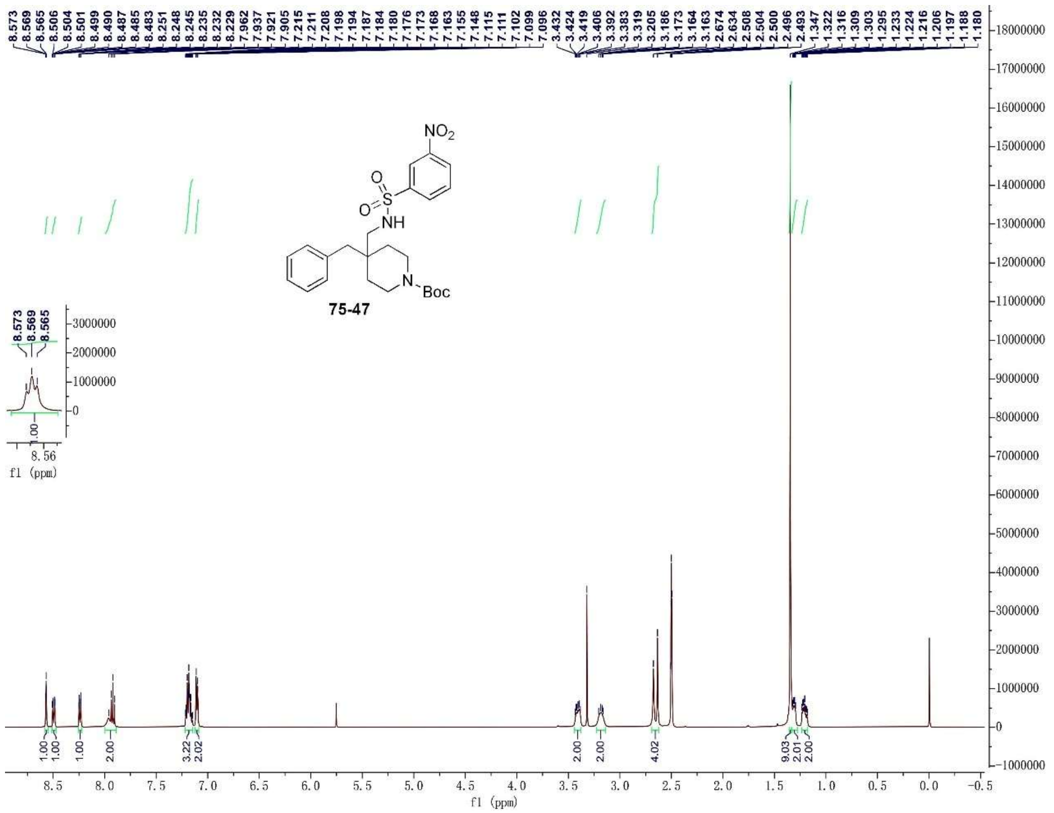

Figure S418: ^{1}H NMR spectrum of **75-47**

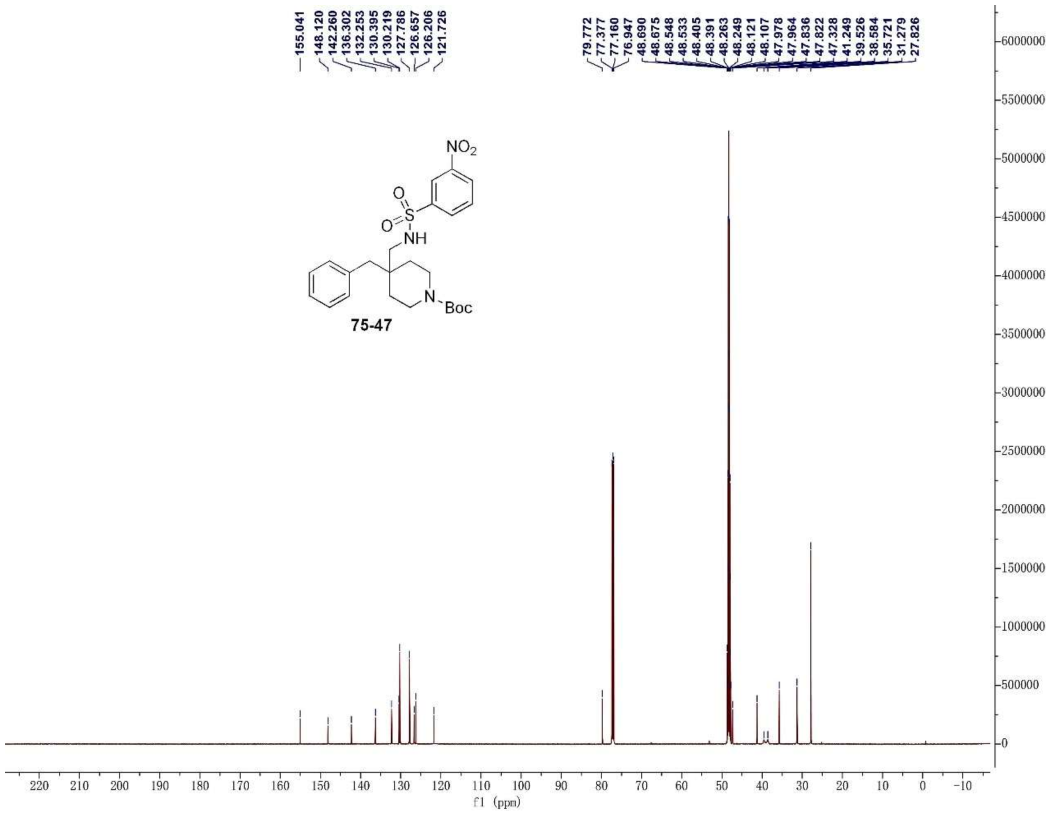

Figure S419: ^{13}C NMR spectrum of **75-47**

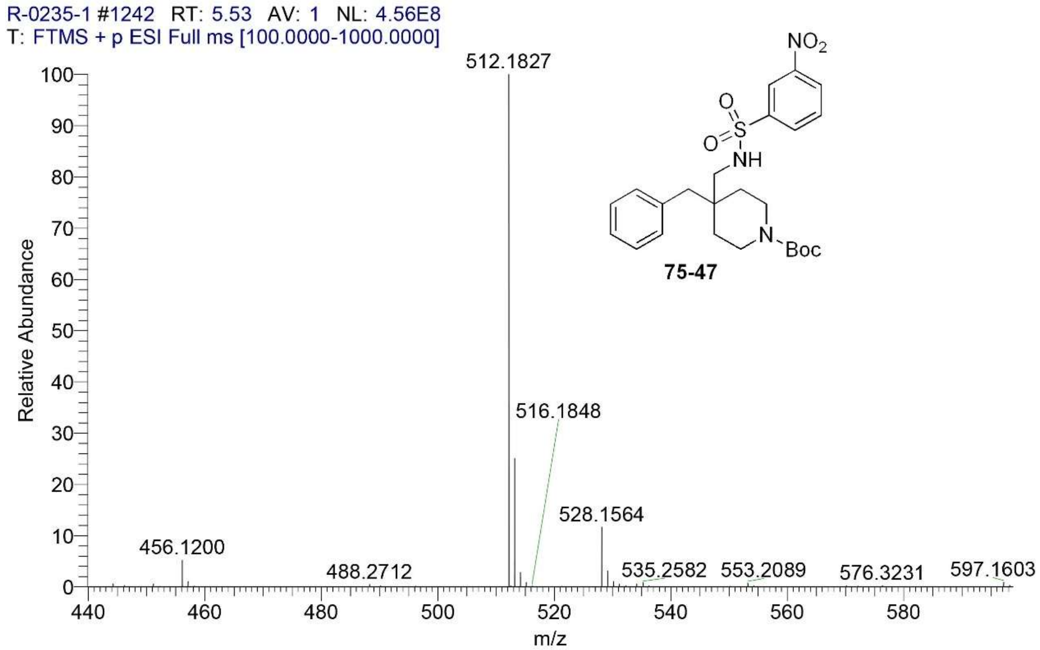

Figure S420: HR-MS (ESI/ion trap) spectrum of **75-47**

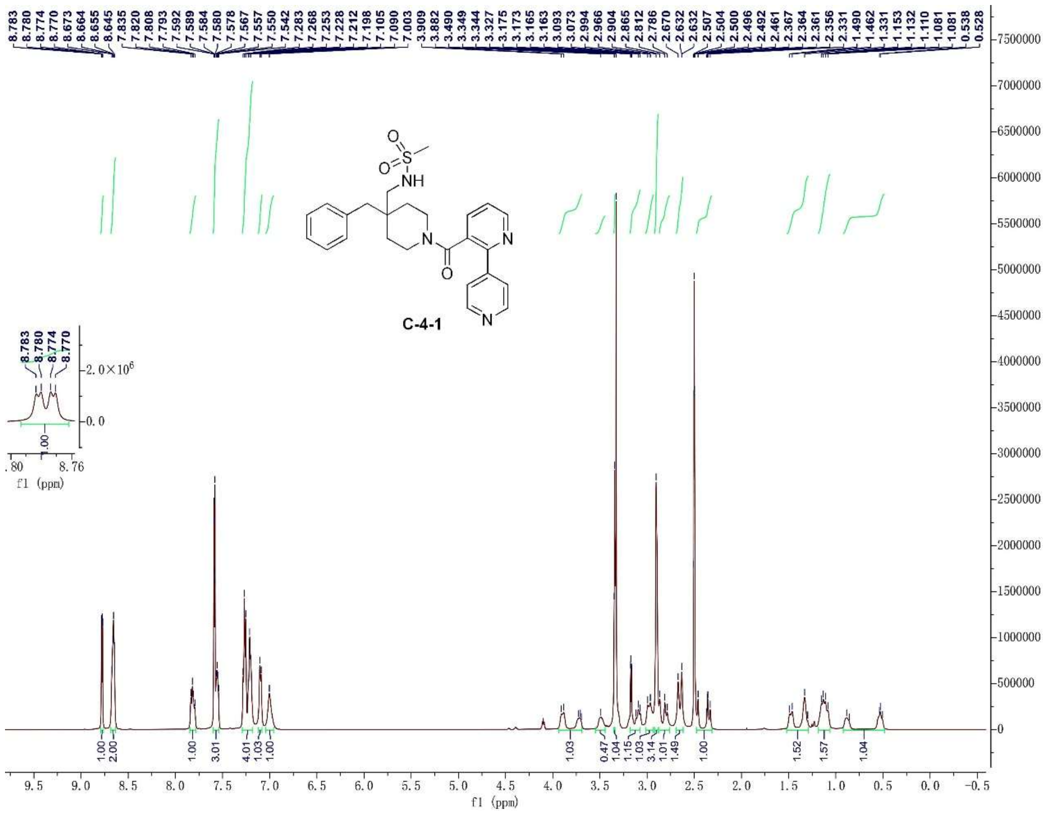

Figure S421: ^{1}H NMR spectrum of **C-4-1**

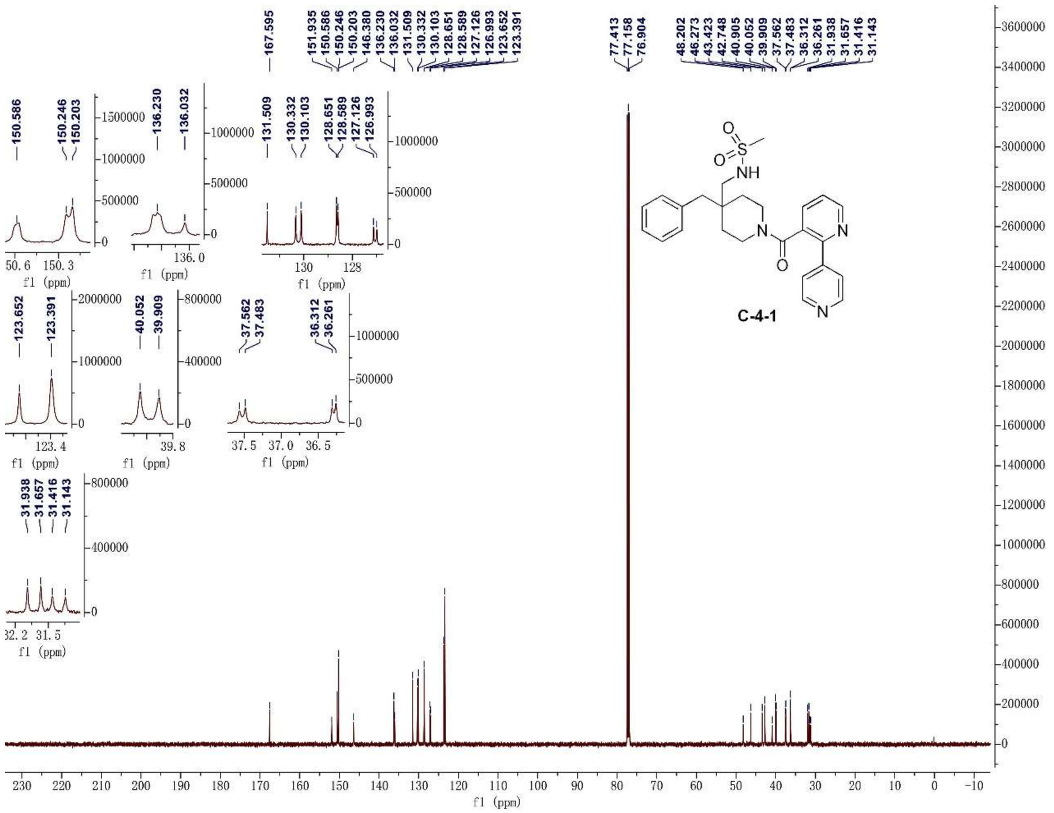

Figure S422: ^{13}C NMR spectrum of **C-4-1**

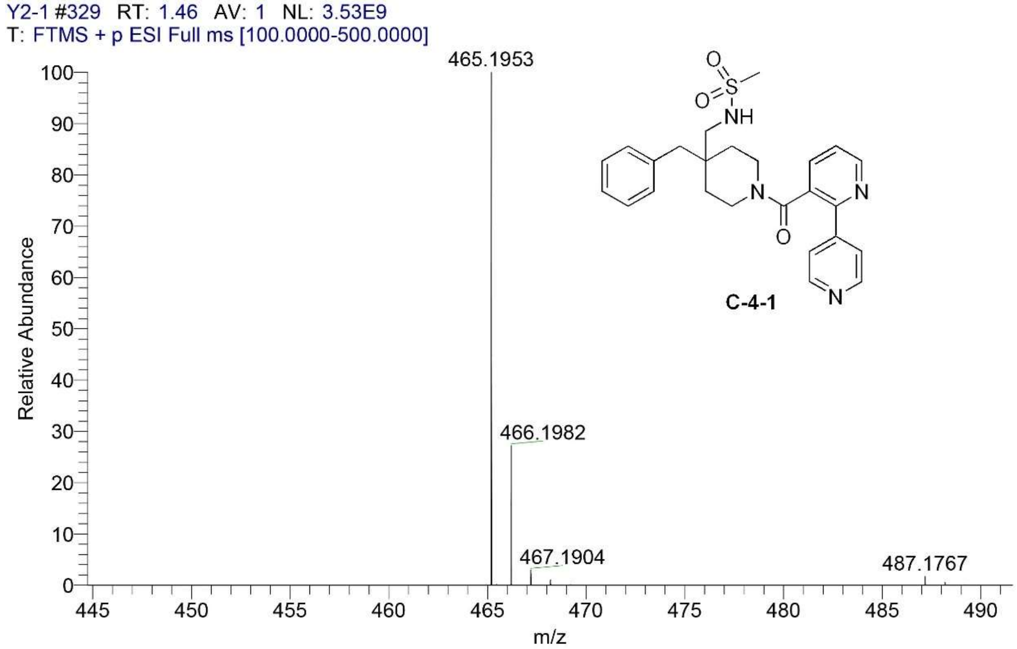

Figure S423: HR-MS (ESI/ion trap) spectrum of **C-4-1**

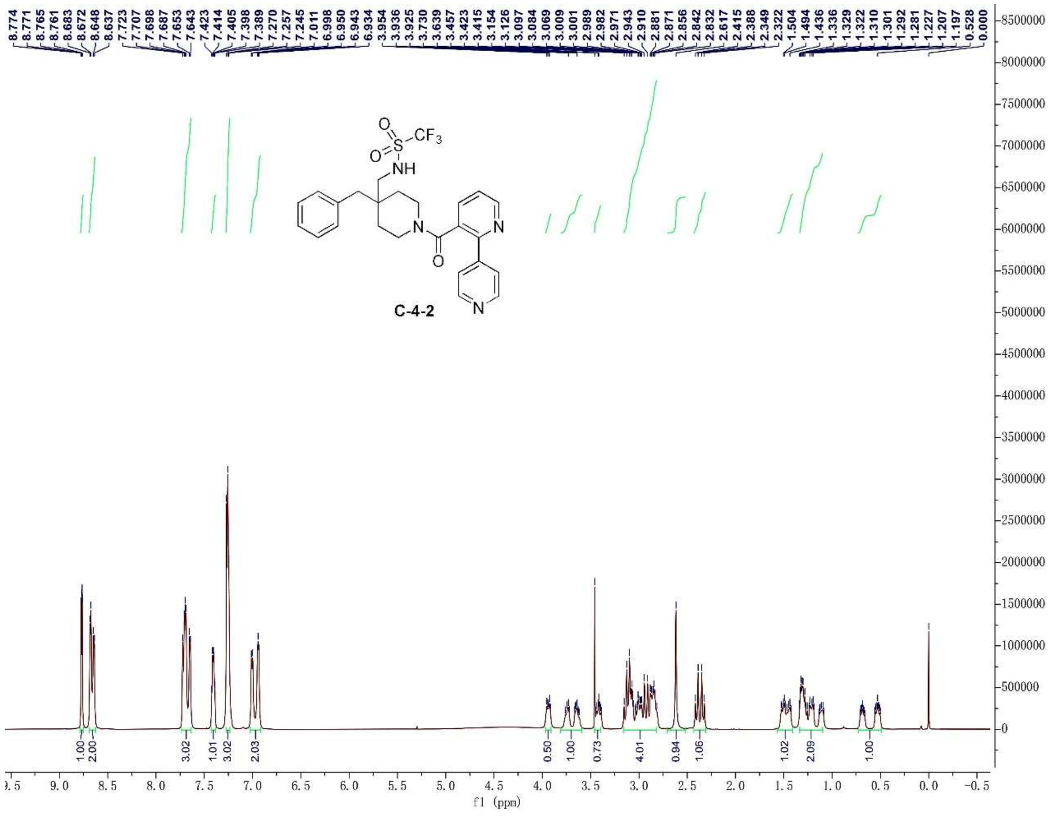

Figure S424: ^{1}H NMR spectrum of **C-4-2**

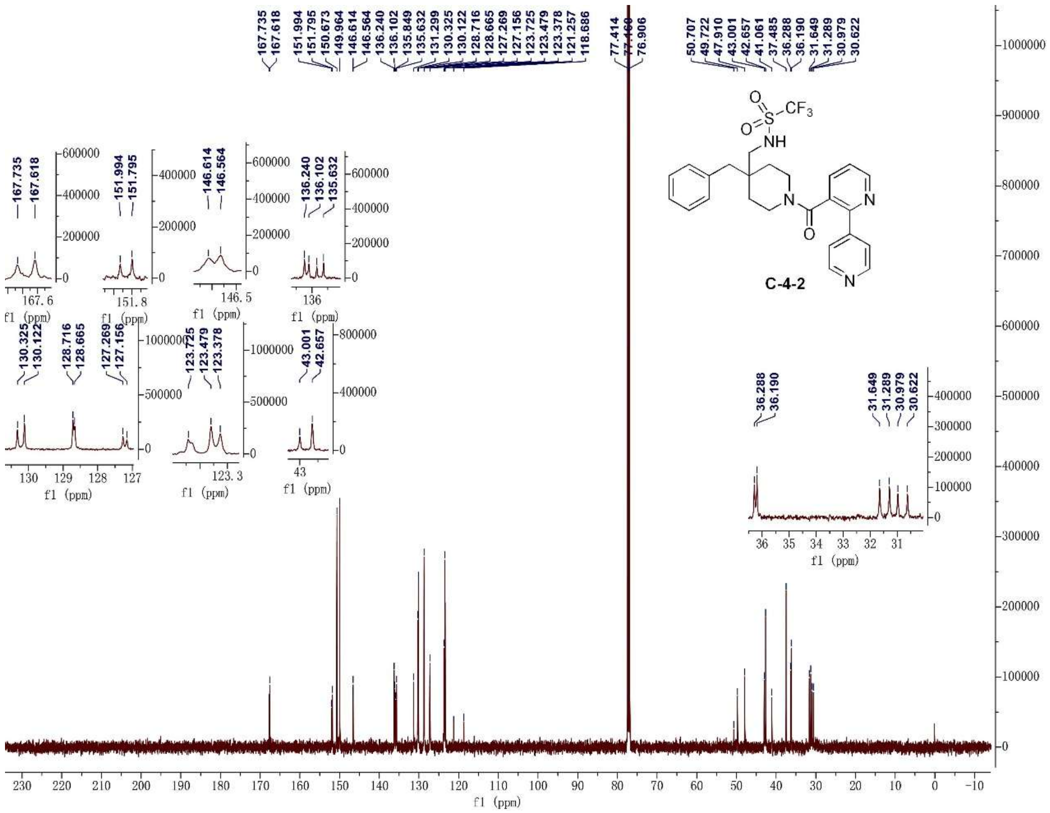

Figure S425: ^{13}C NMR spectrum of **C-4-2**

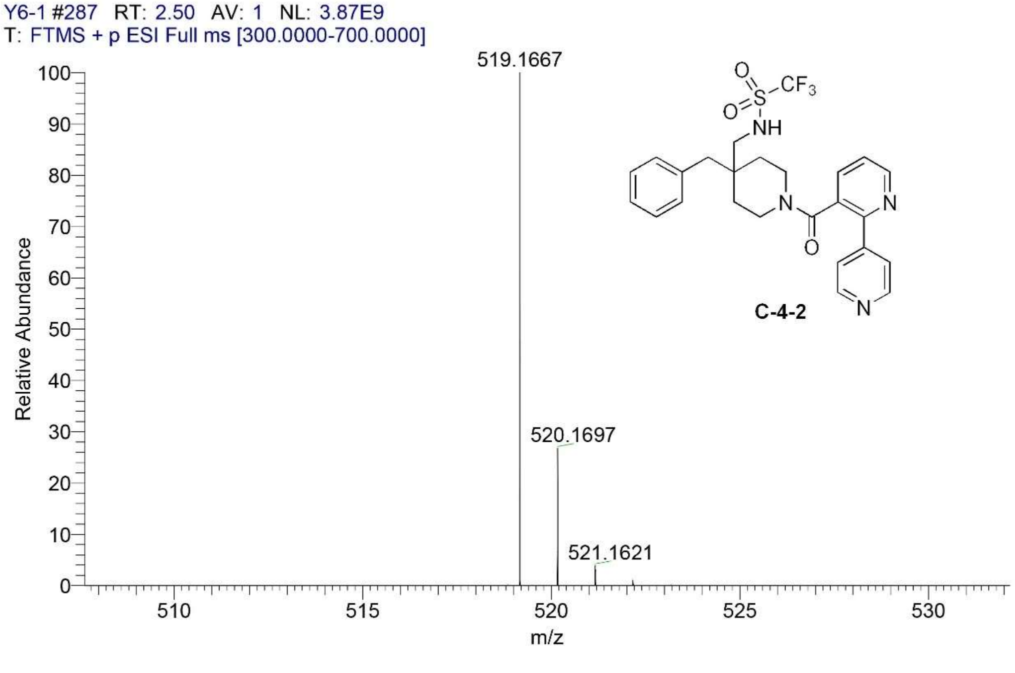

Figure S426: HR-MS (ESI/ion trap) spectrum of **C-4-2**

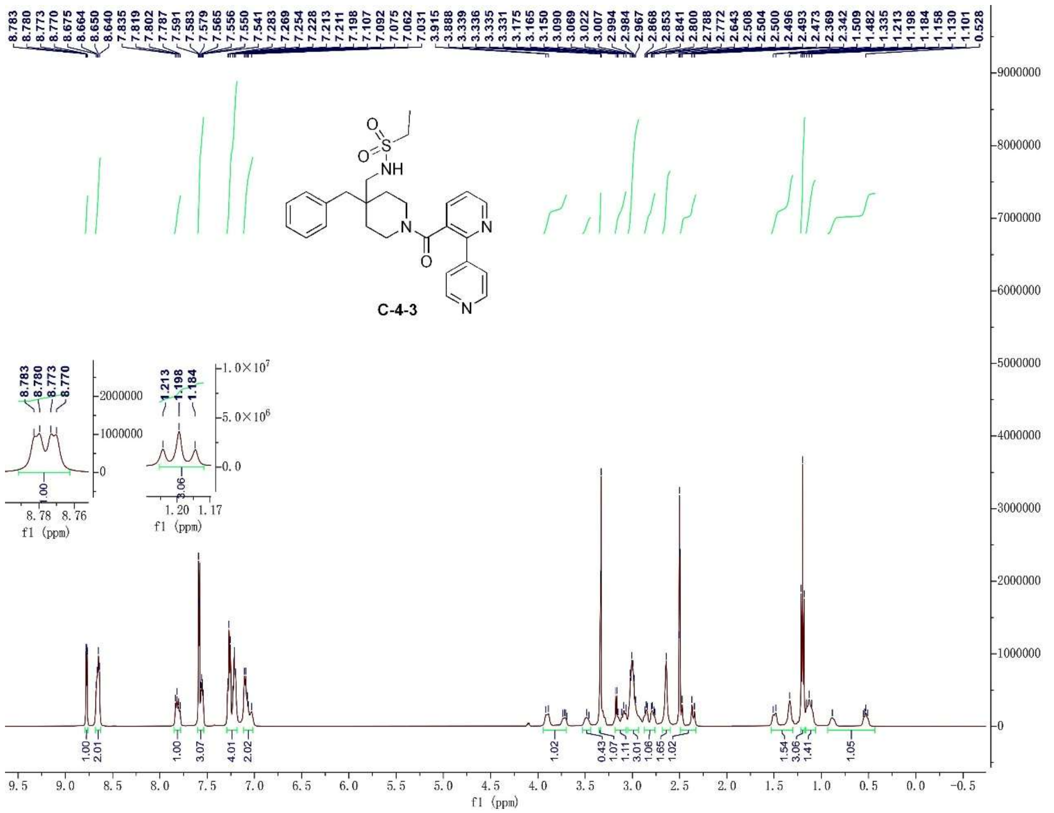

Figure S427: ^{1}H NMR spectrum of **C-4-3**

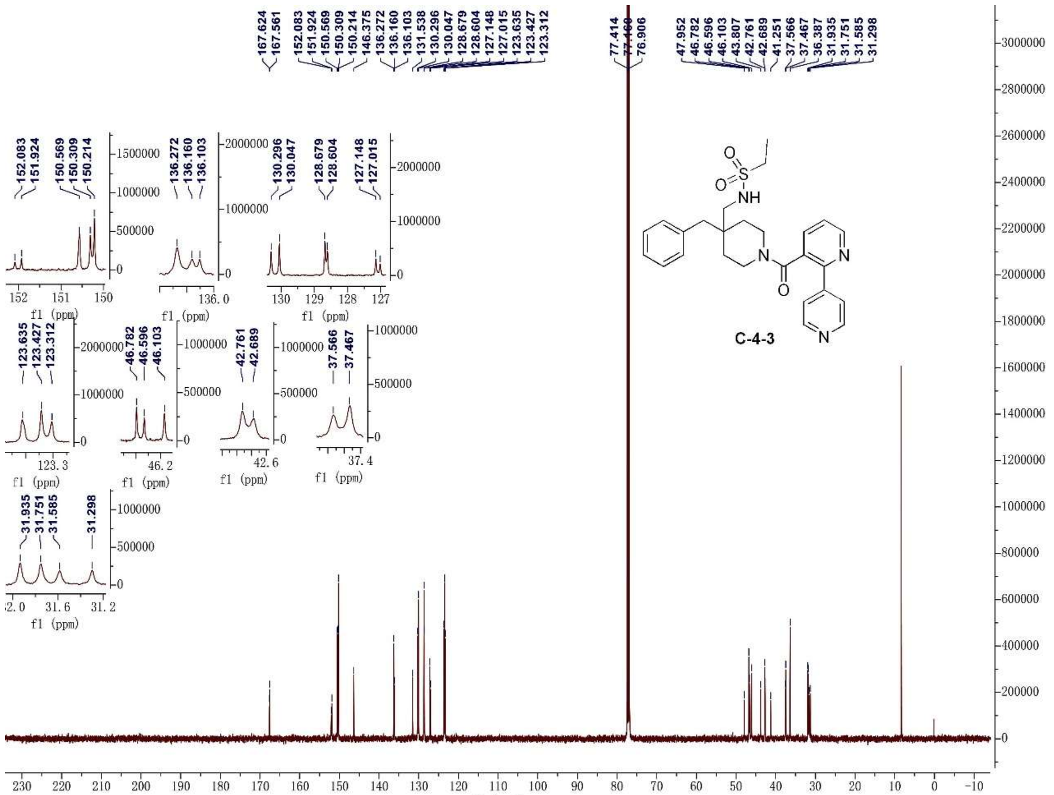

Figure S428: ^{13}C NMR spectrum of **C-4-3**

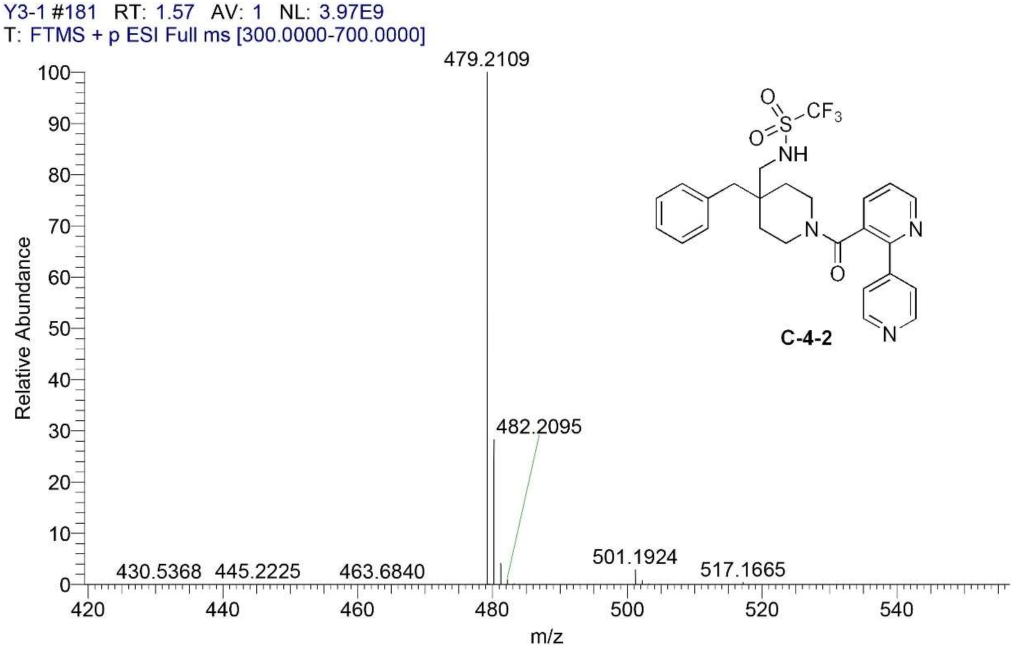

Figure S429: HR-MS (ESI/ion trap) spectrum of **C-4-2**

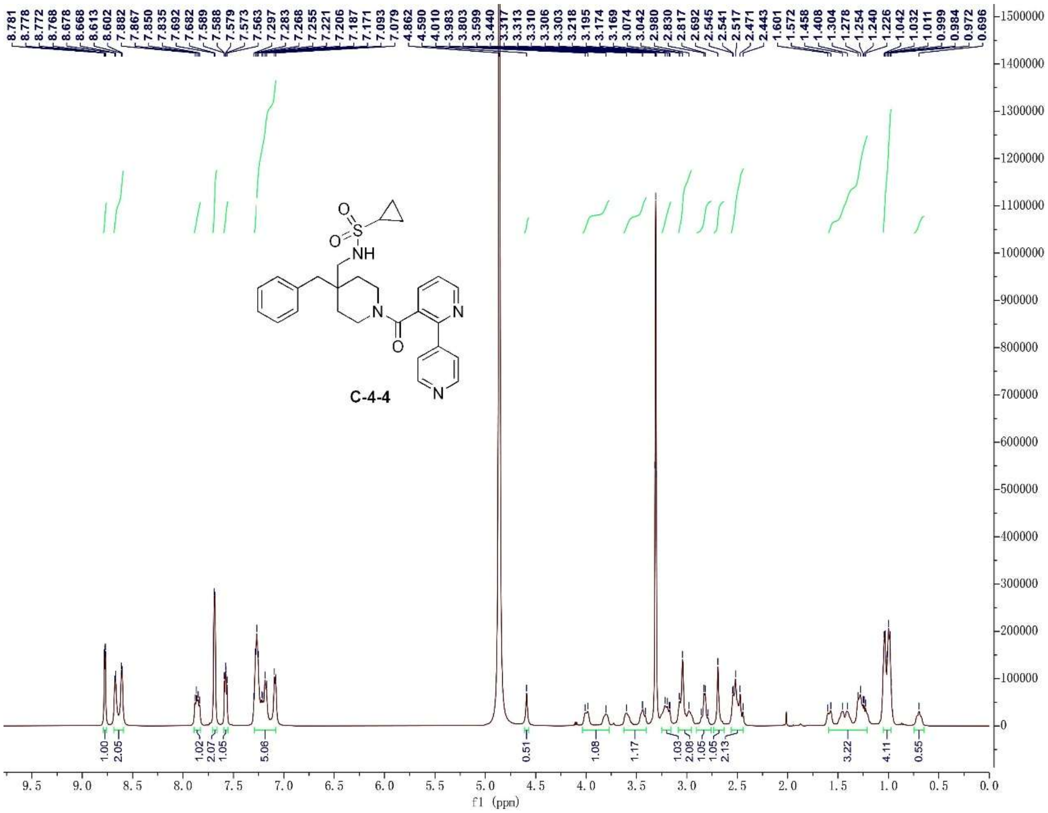

Figure S430: ^{1}H NMR spectrum of **C-4-4**

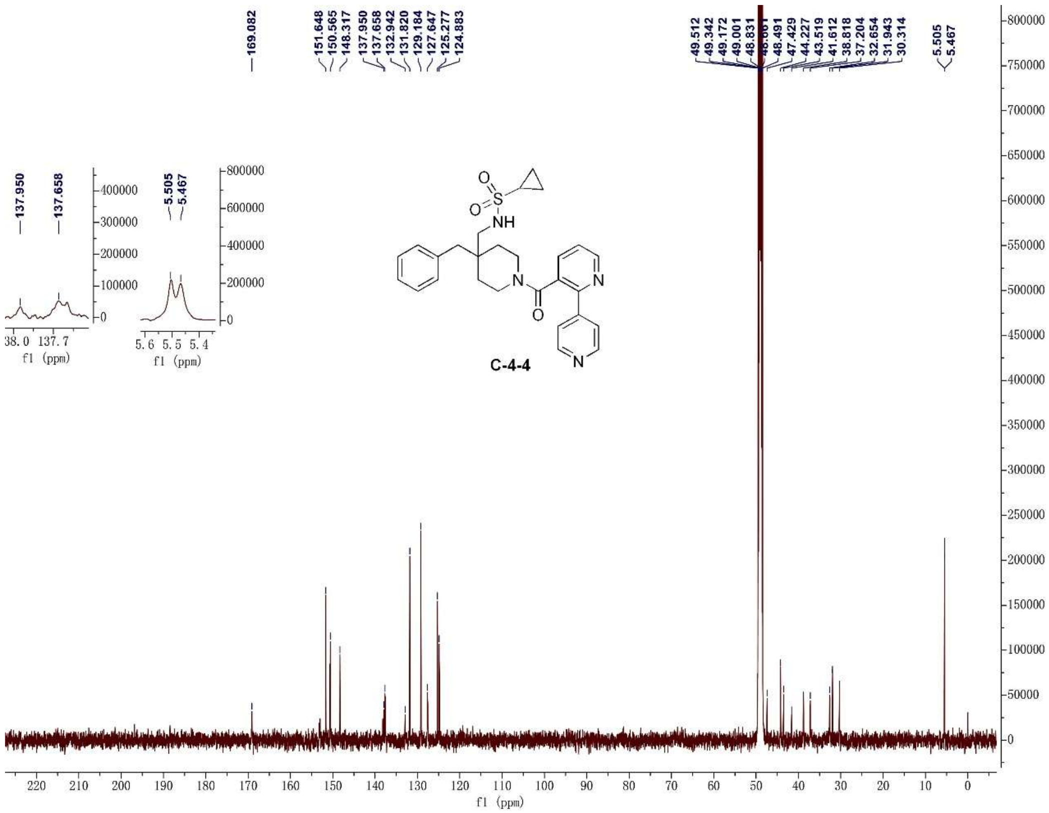

Figure S431: ^{13}C NMR spectrum of **C-4-4**

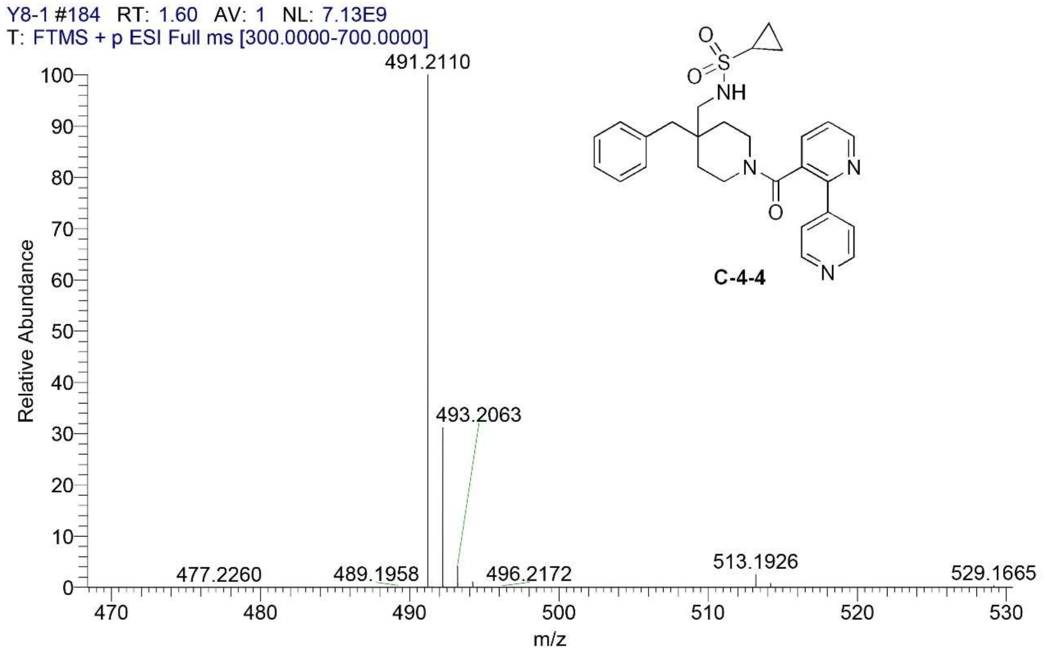

Figure S432: HR-MS (ESI/ion trap) spectrum of **C-4-4**

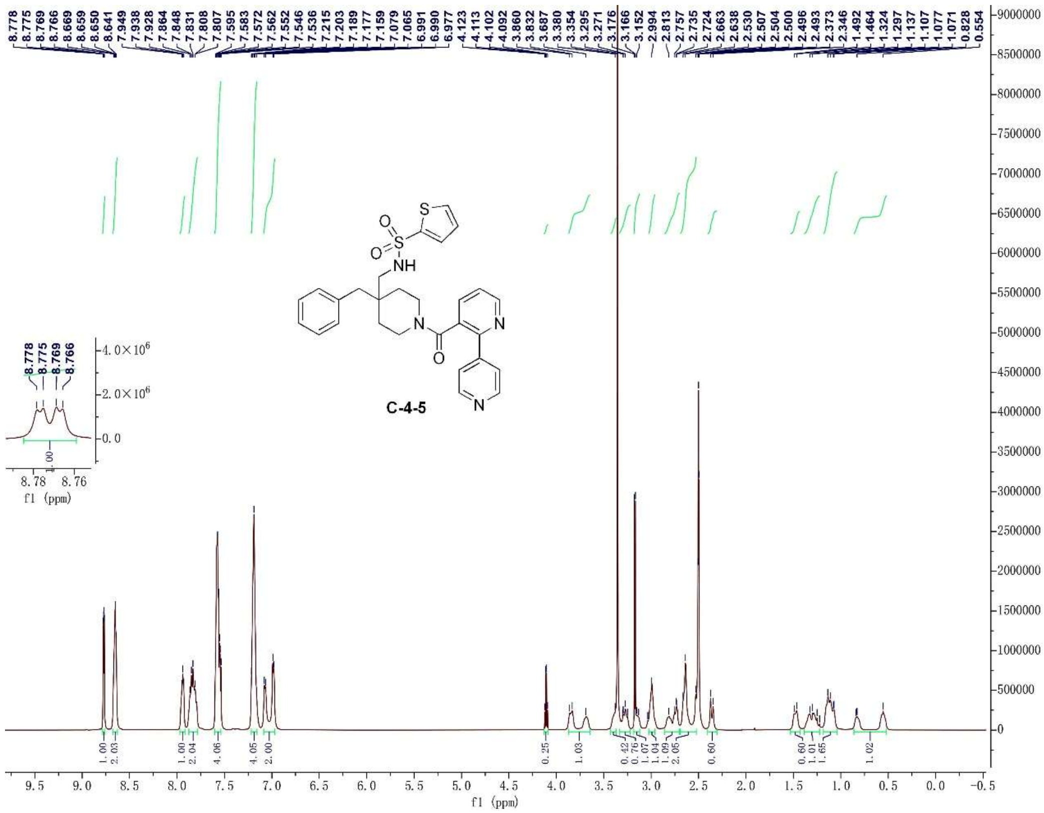

Figure S433: ^{1}H NMR spectrum of **C-4-5**

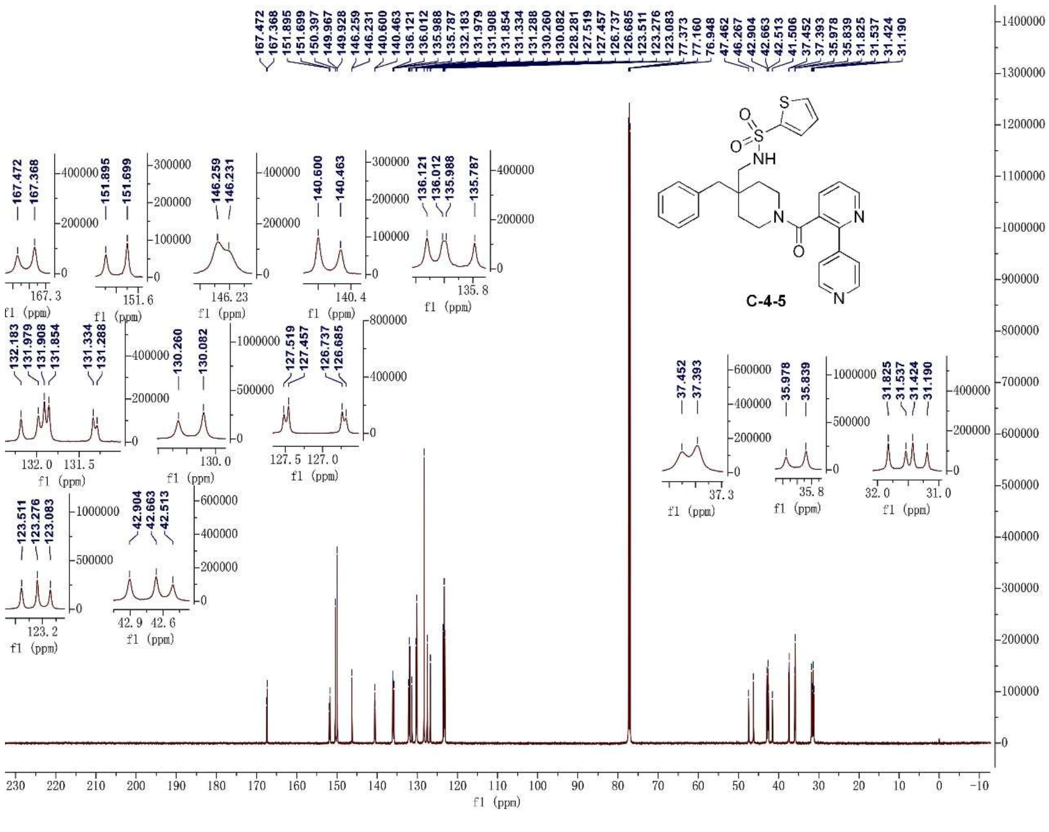

Figure S434: ^{13}C NMR spectrum of **C-4-5**

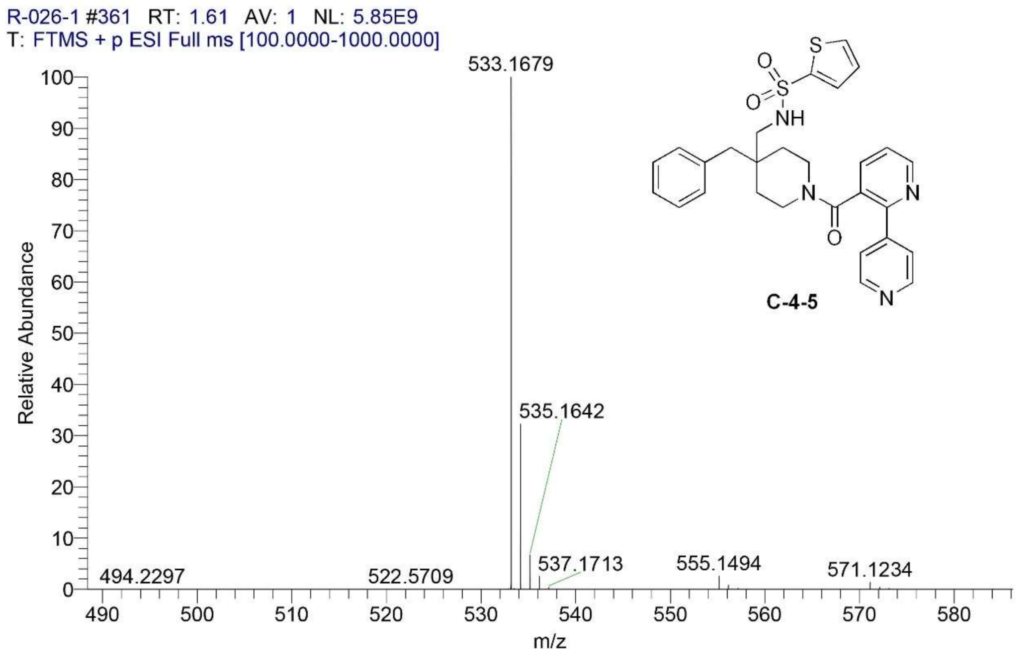

Figure S435: HR-MS (ESI/ion trap) spectrum of **C-4-5**

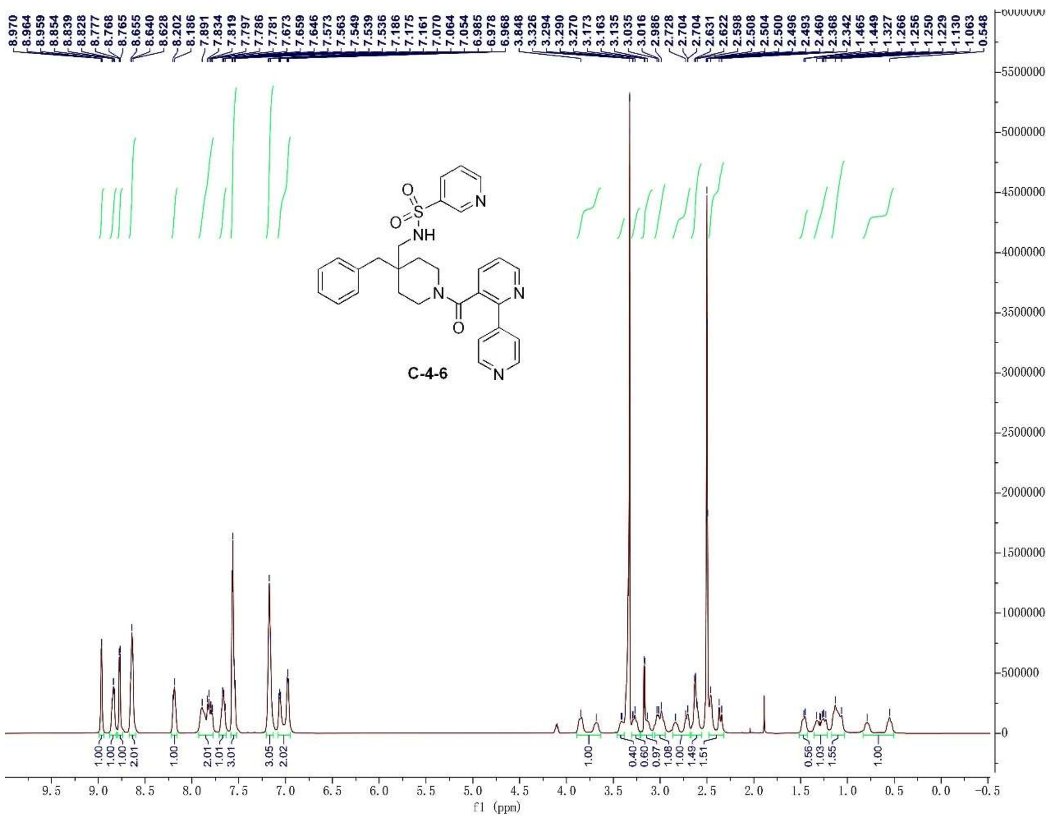

Figure S436: ^{1}H NMR spectrum of **C-4-6**

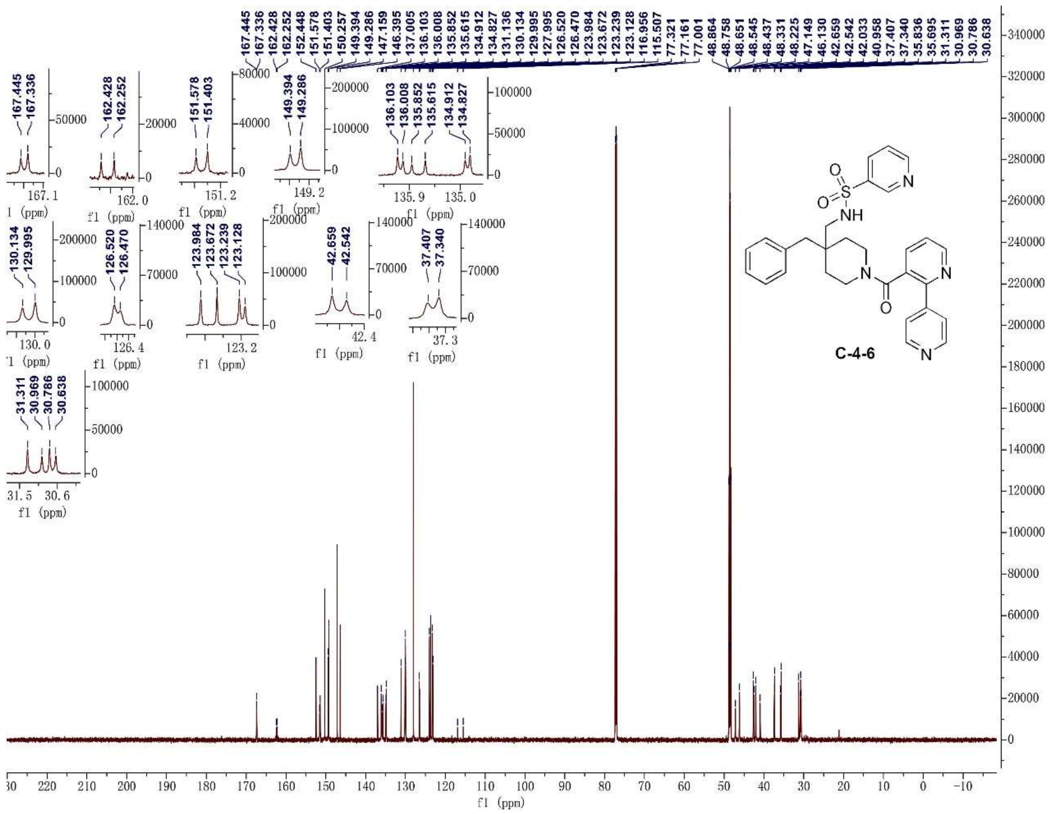

Figure S437: ^{13}C NMR spectrum of **C-4-6**

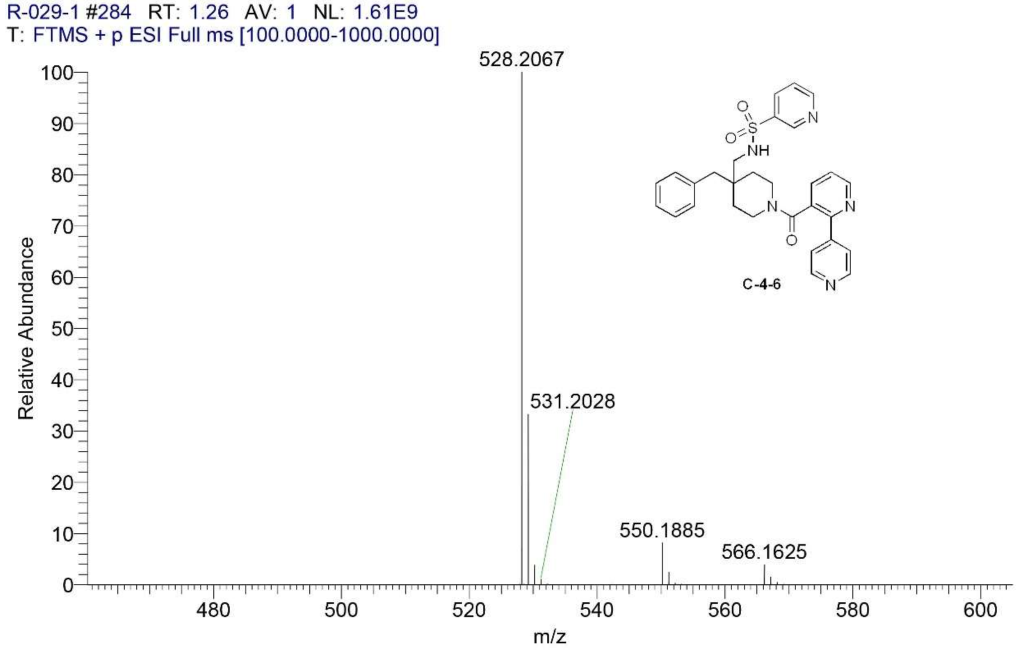

Figure S438: HR-MS (ESI/ion trap) spectrum of **C-4-6**

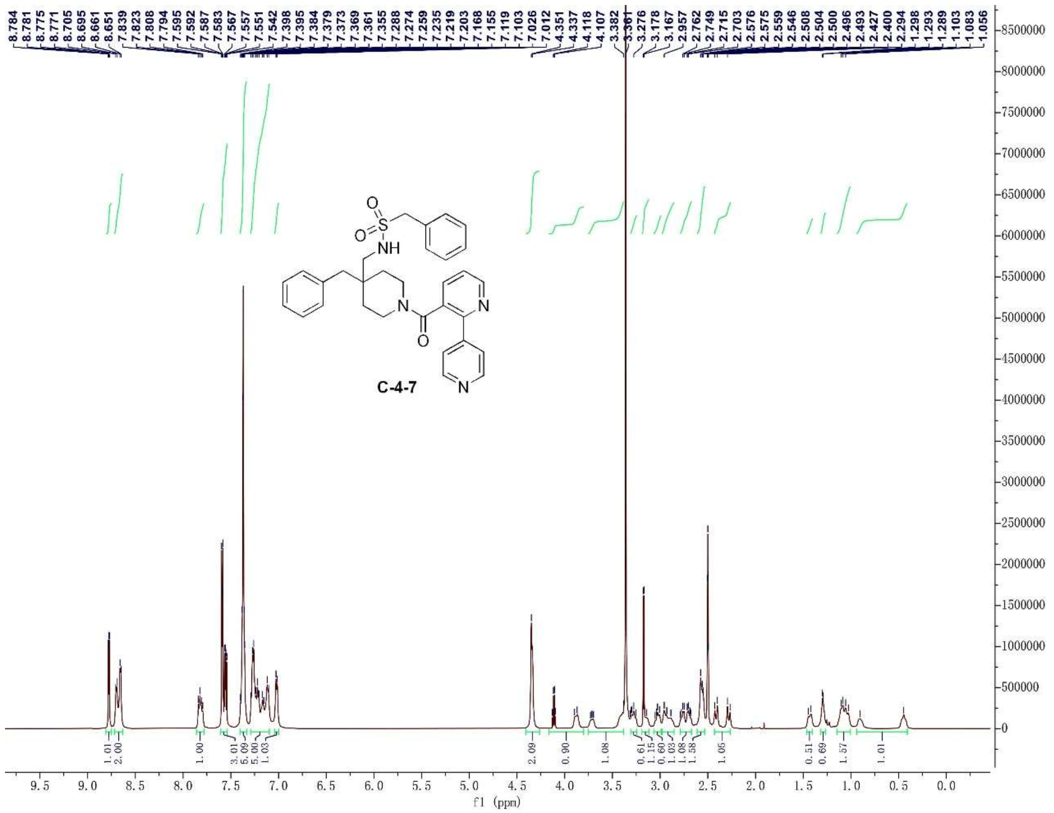

Figure S439: ^{1}H NMR spectrum of **C-4-7**

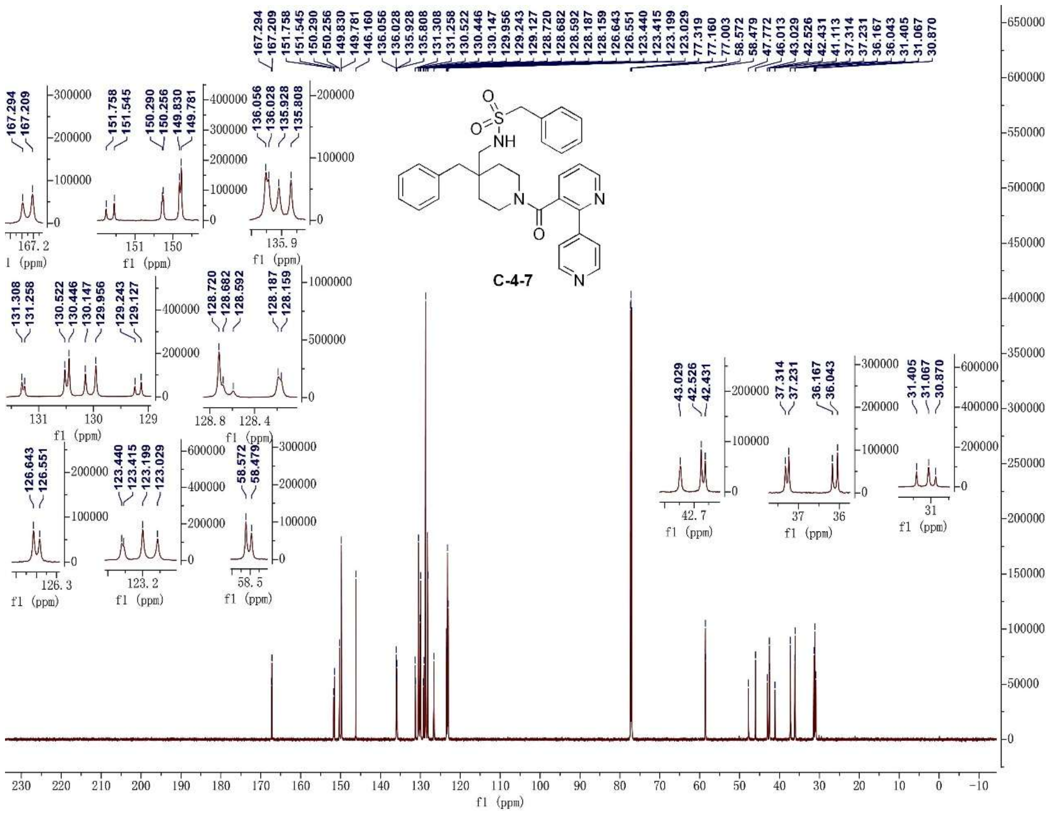

Figure S440: ^{13}C NMR spectrum of **C-4-7**

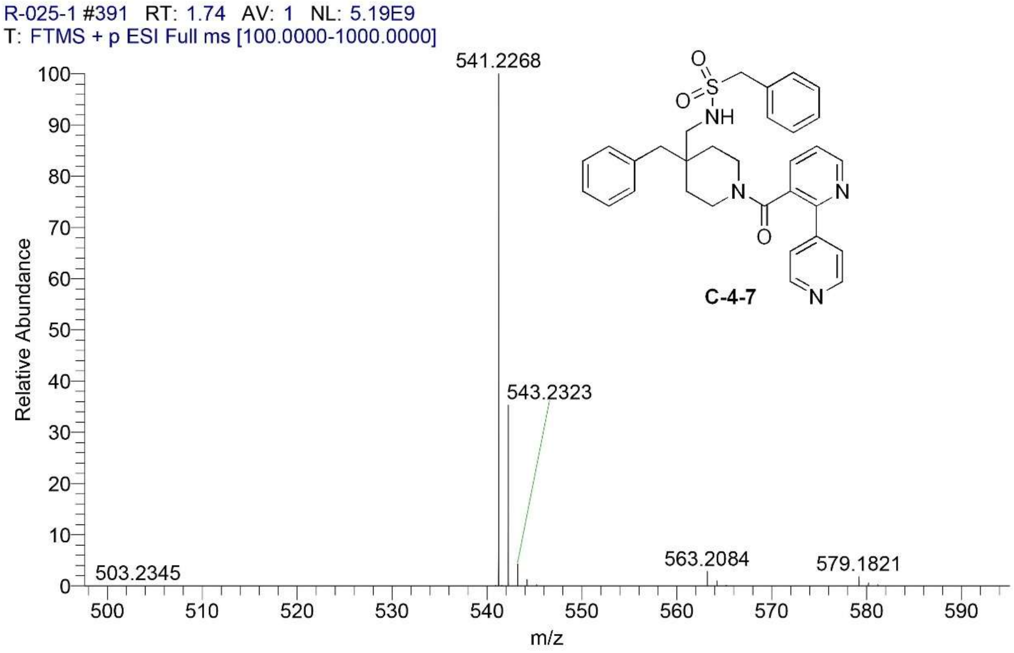

Figure S441: HR-MS (ESI/ion trap) spectrum of **C-4-7**

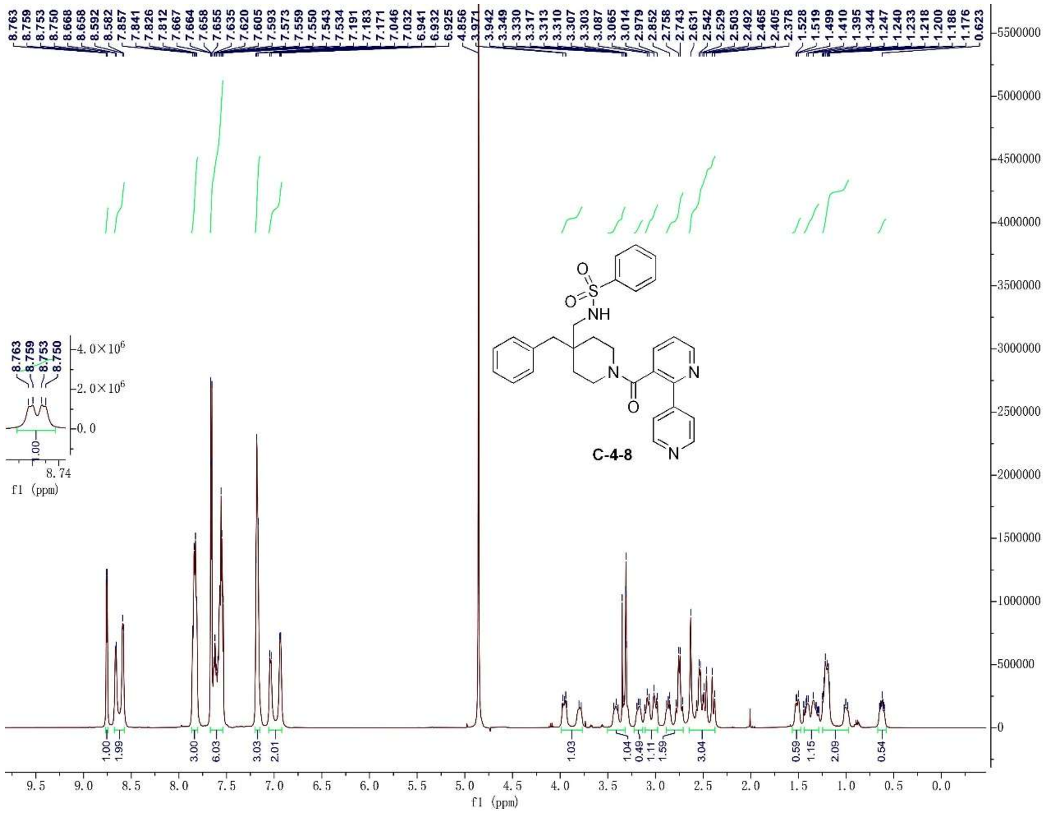

Figure S442: ^{1}H NMR spectrum of **C-4-8**

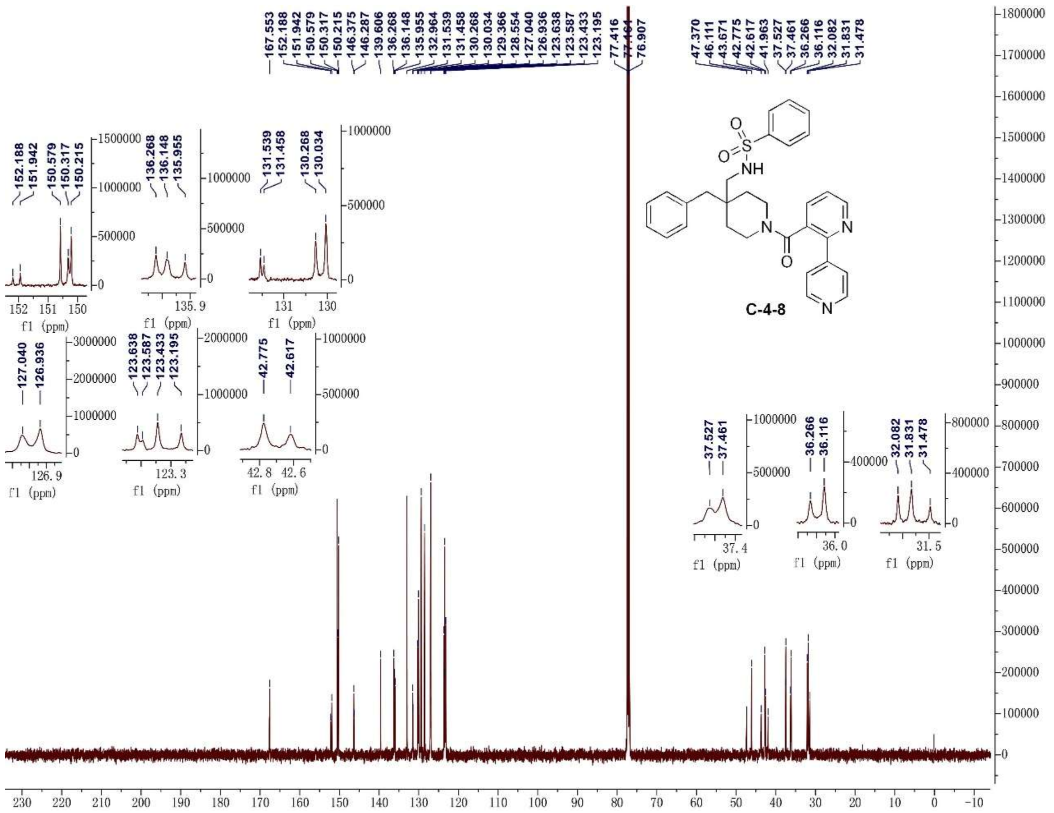

Figure S443: ^{13}C NMR spectrum of **C-4-8**

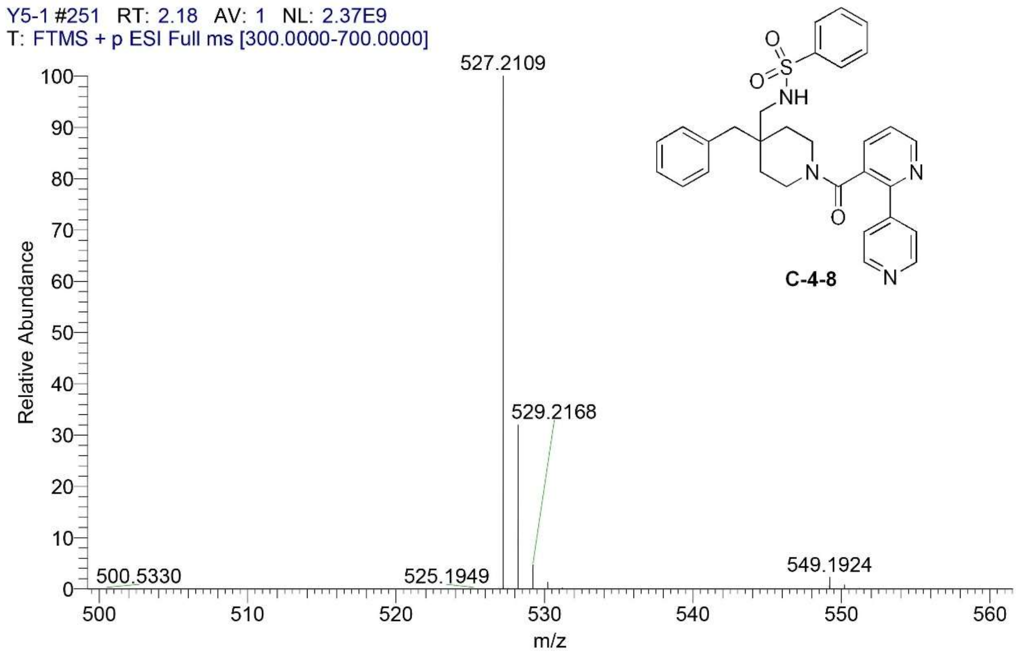

Figure S444: HR-MS (ESI/ion trap) spectrum of **C-4-8**

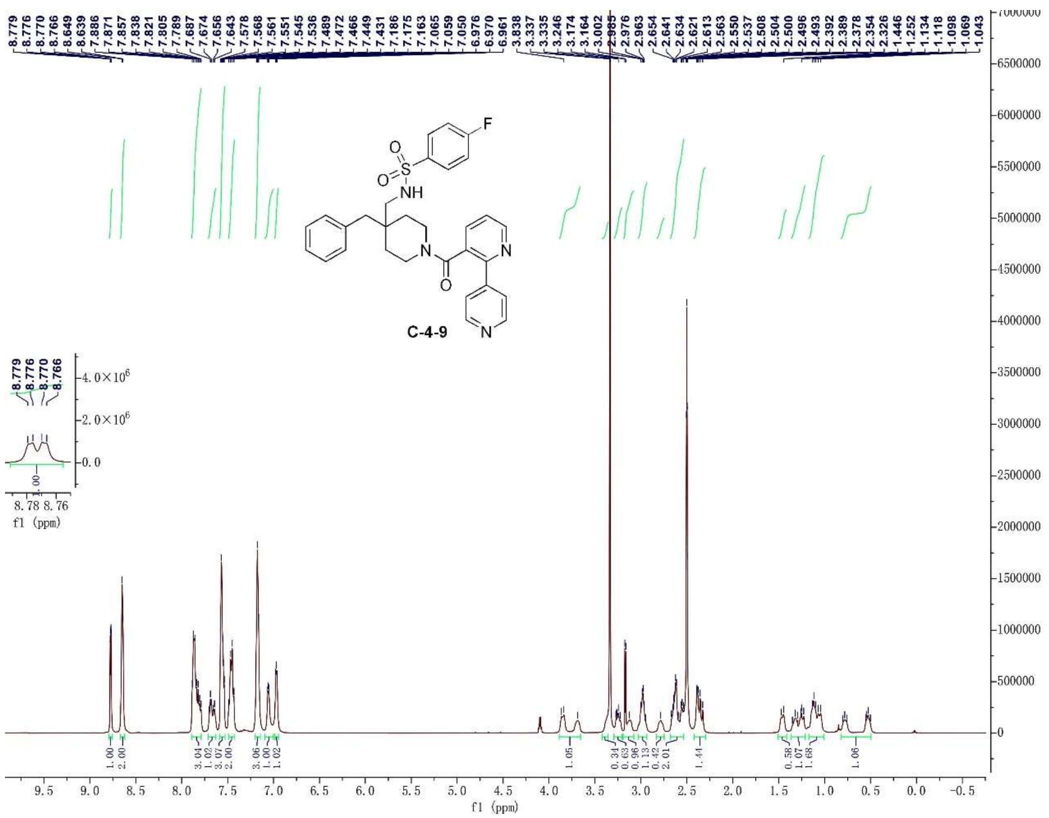

Figure S445: ^{1}H NMR spectrum of **C-4-9**

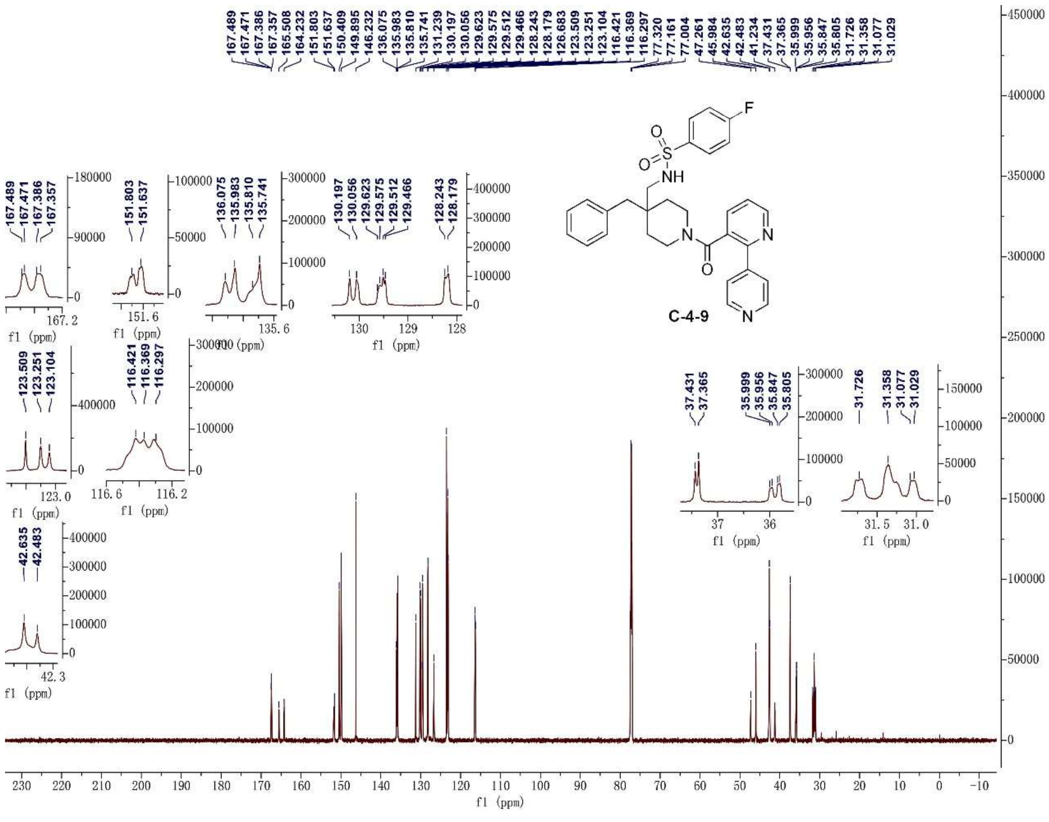

Figure S446: ^{13}C NMR spectrum of **C-4-9**

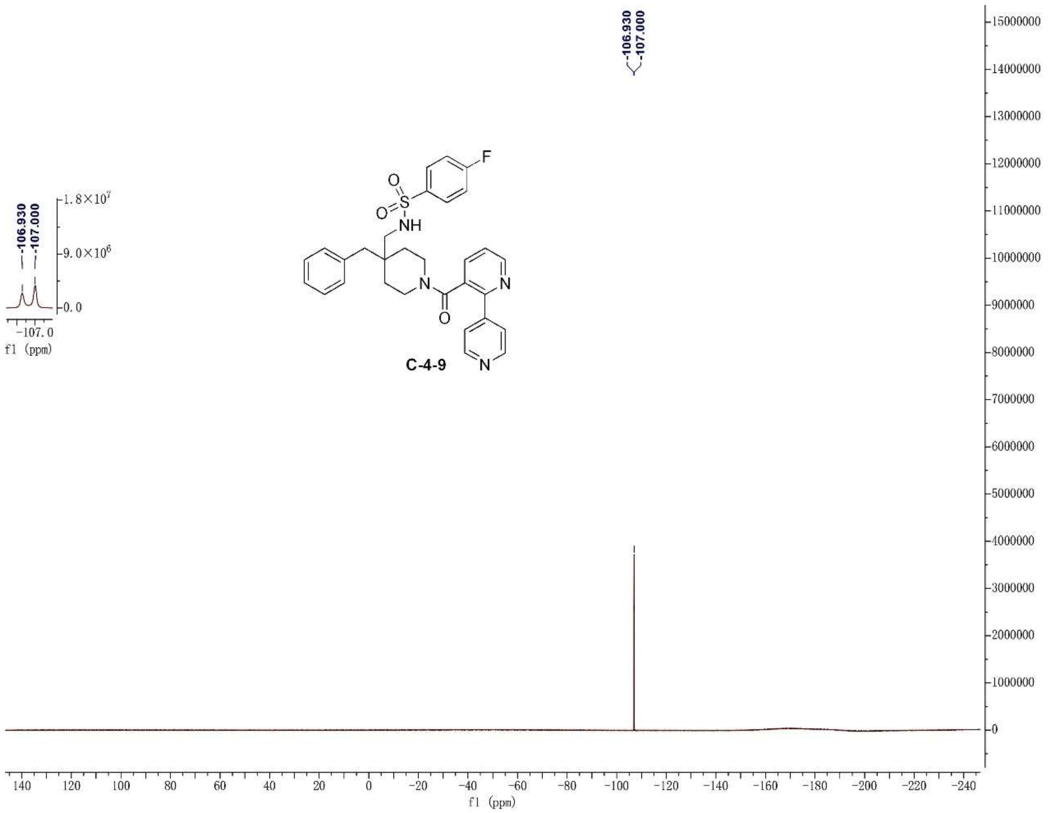

Figure S447: ^{19}F NMR spectrum of **C-4-9**

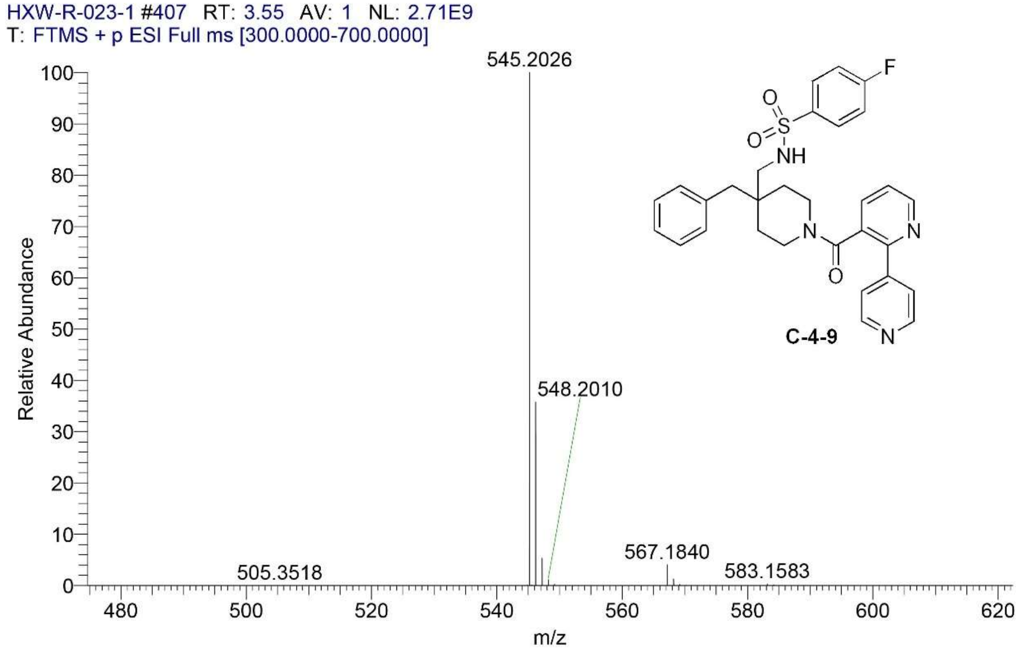

Figure S448: HR-MS (ESI/ion trap) spectrum of **C-4-9**

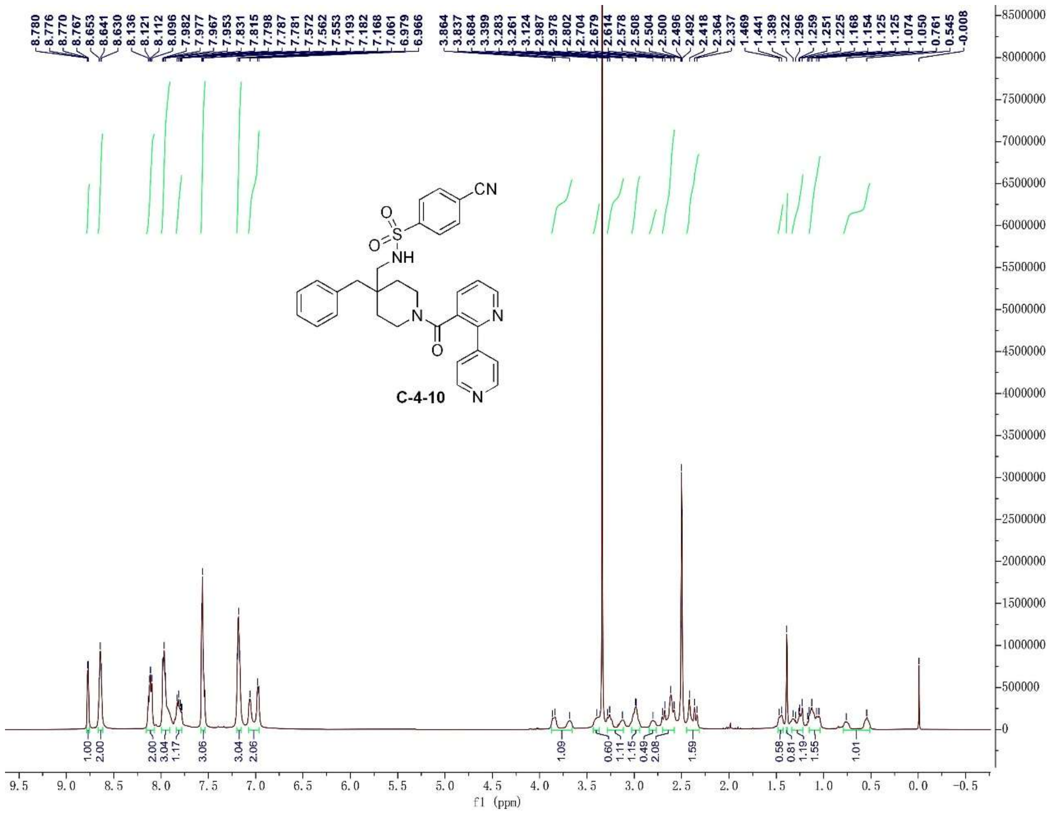

Figure S449: ^{1}H NMR spectrum of **C-4-10**

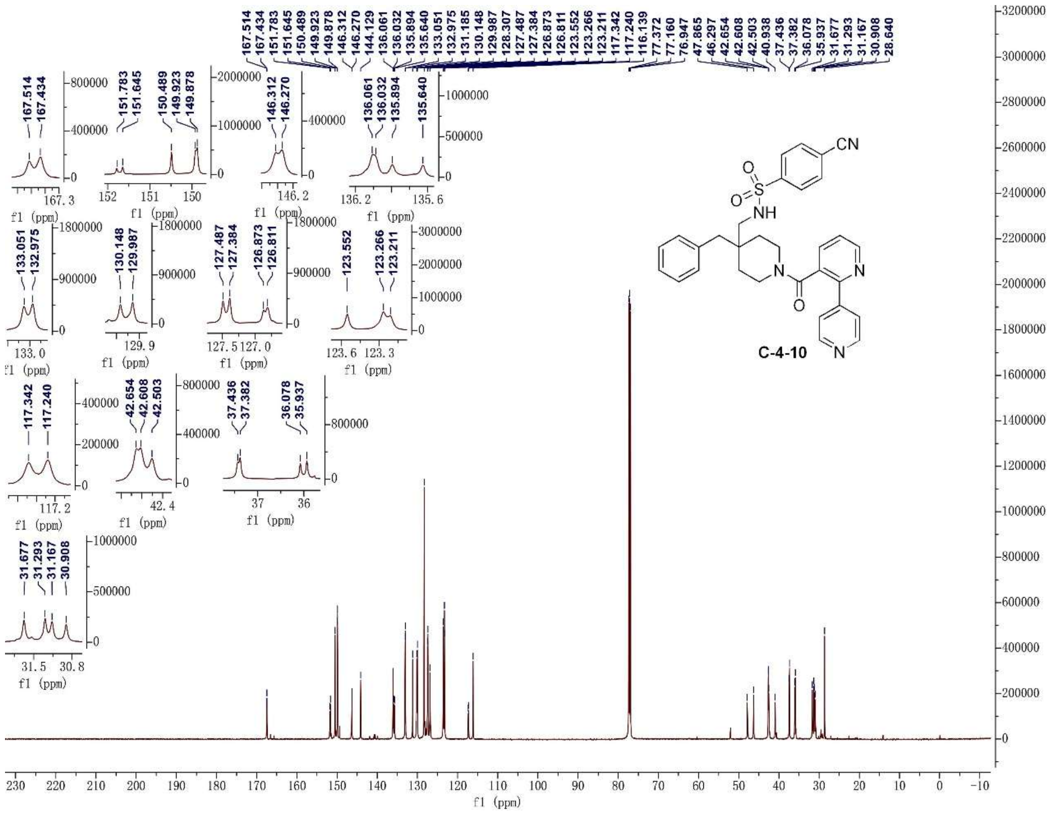

Figure S450: ^{13}C NMR spectrum of **C-4-10**

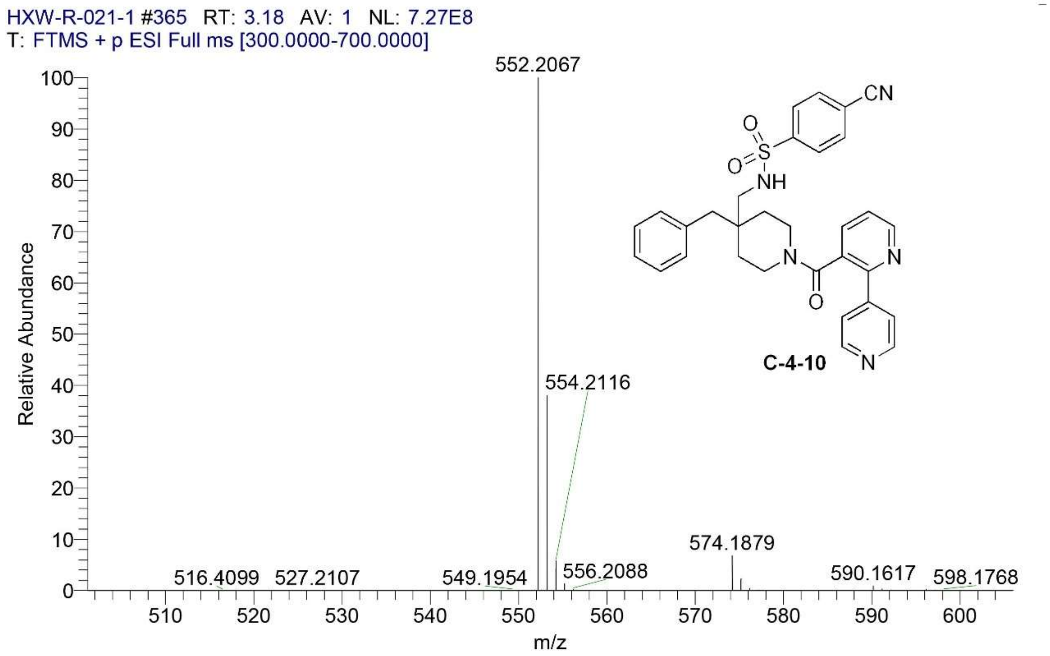

Figure S451: HR-MS (ESI/ion trap) spectrum of **C-4-10**

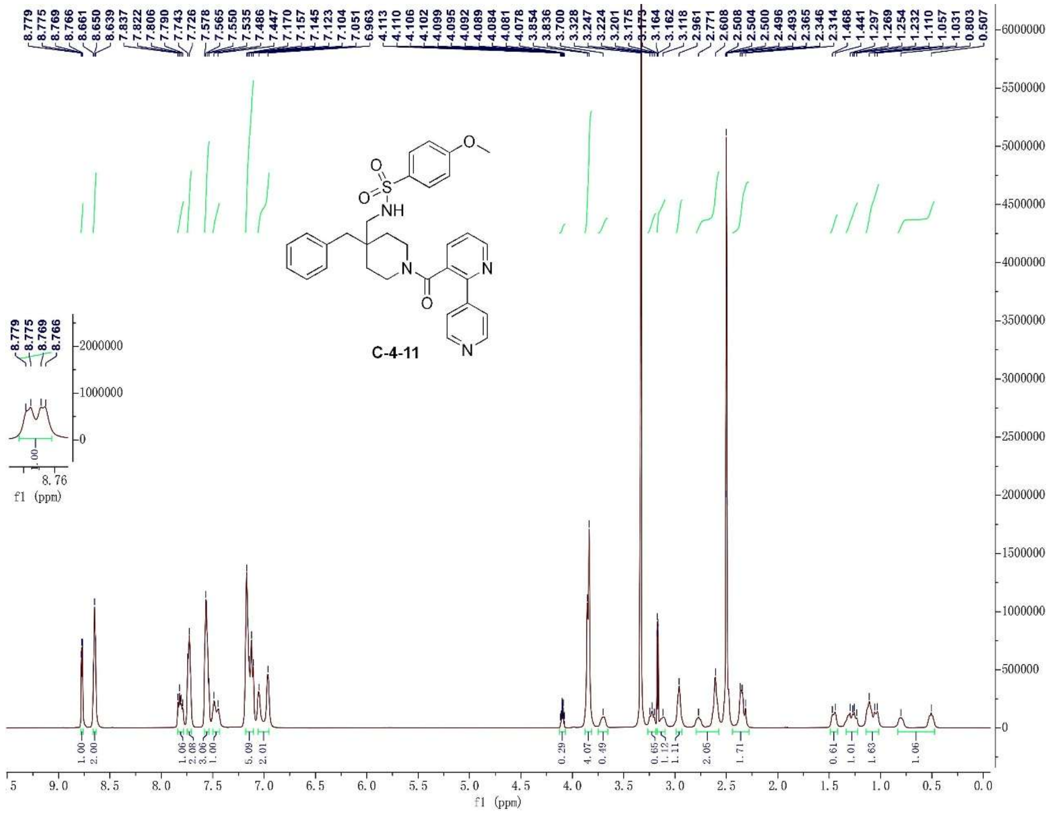

Figure S452: ^{1}H NMR spectrum of **C-4-11**

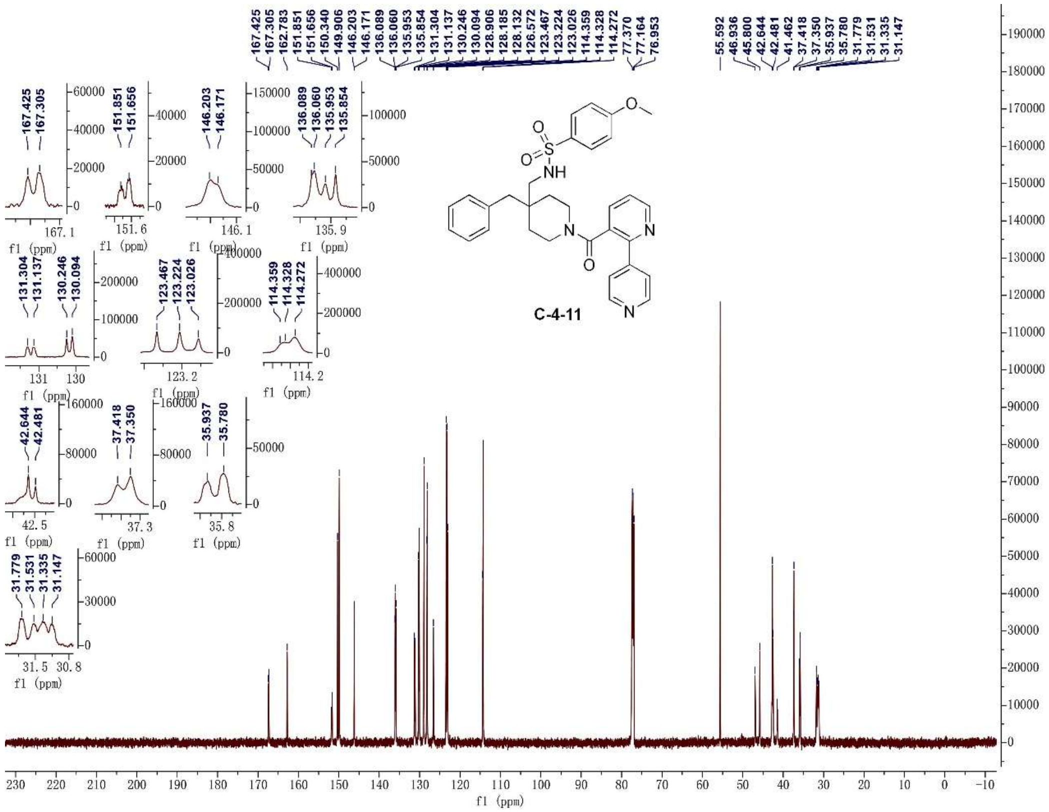

Figure S453: ^{13}C NMR spectrum of **C-4-11**

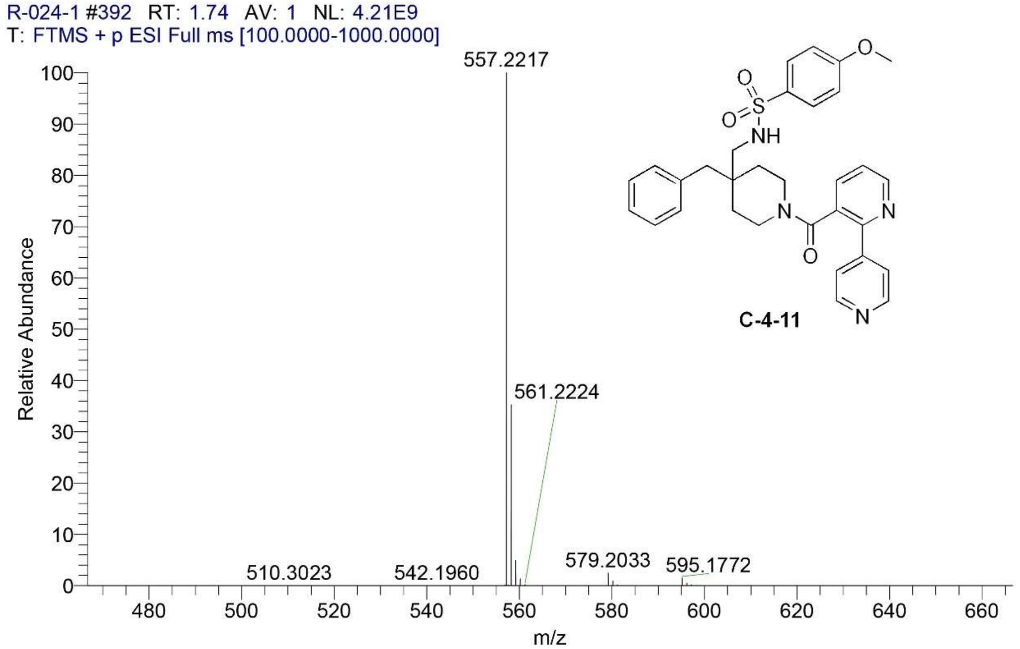

Figure S454: HR-MS (ESI/ion trap) spectrum of **C-4-11**

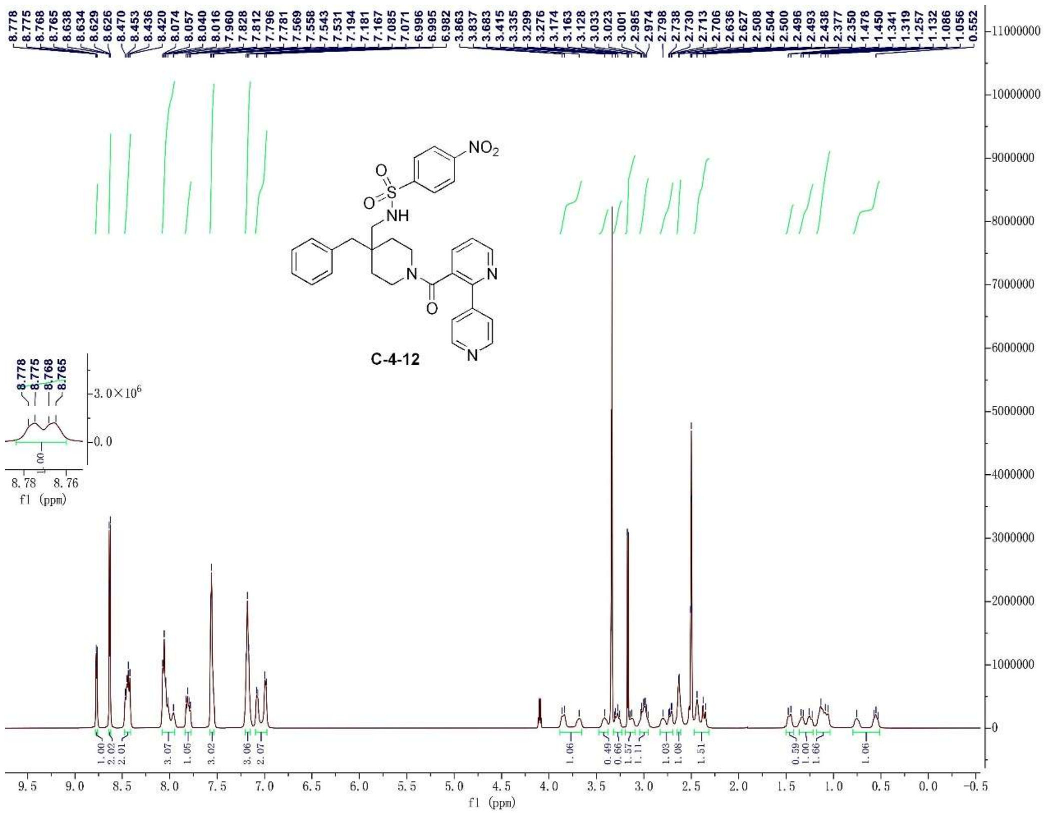

Figure S455: ^{1}H NMR spectrum of **C-4-12**

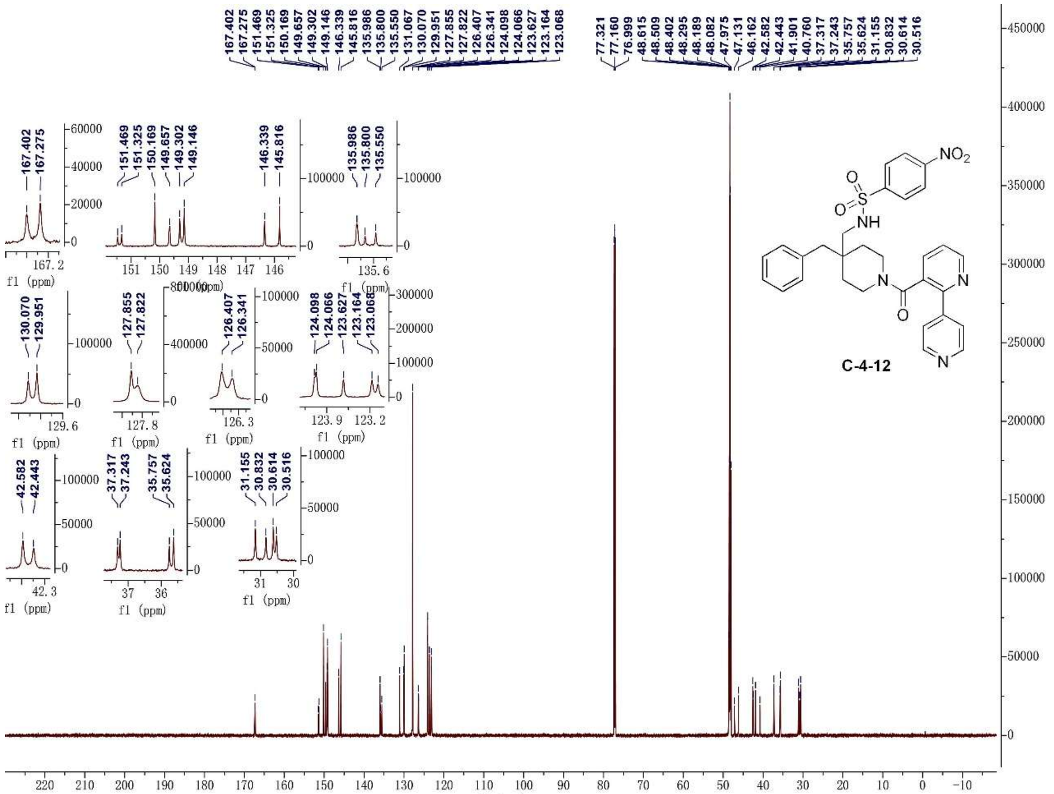

Figure S456: ^{13}C NMR spectrum of **C-4-12**

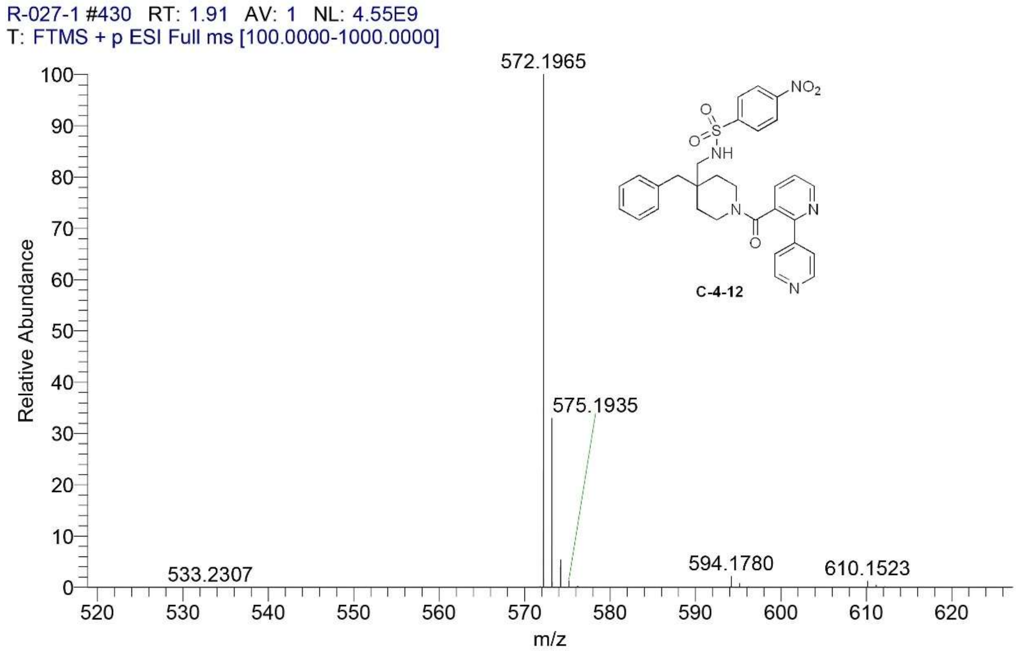

Figure S457: HR-MS (ESI/ion trap) spectrum of **C-4-12**

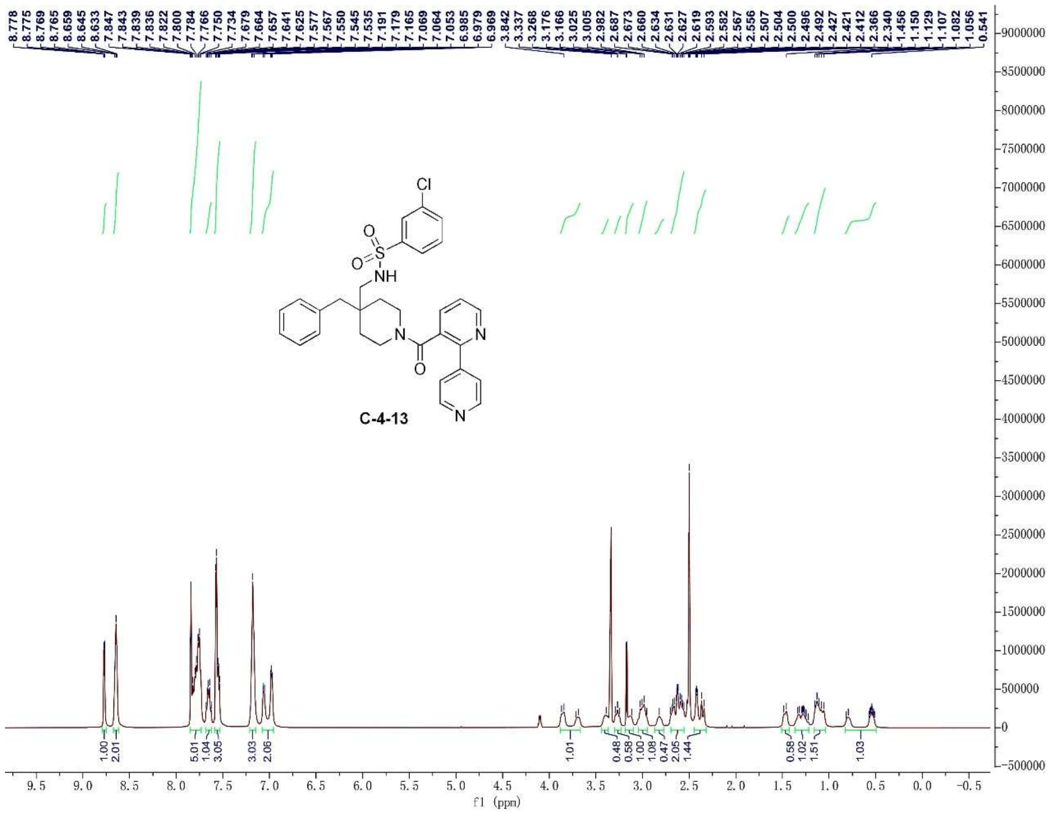

Figure S458: ^{1}H NMR spectrum of **C-4-13**

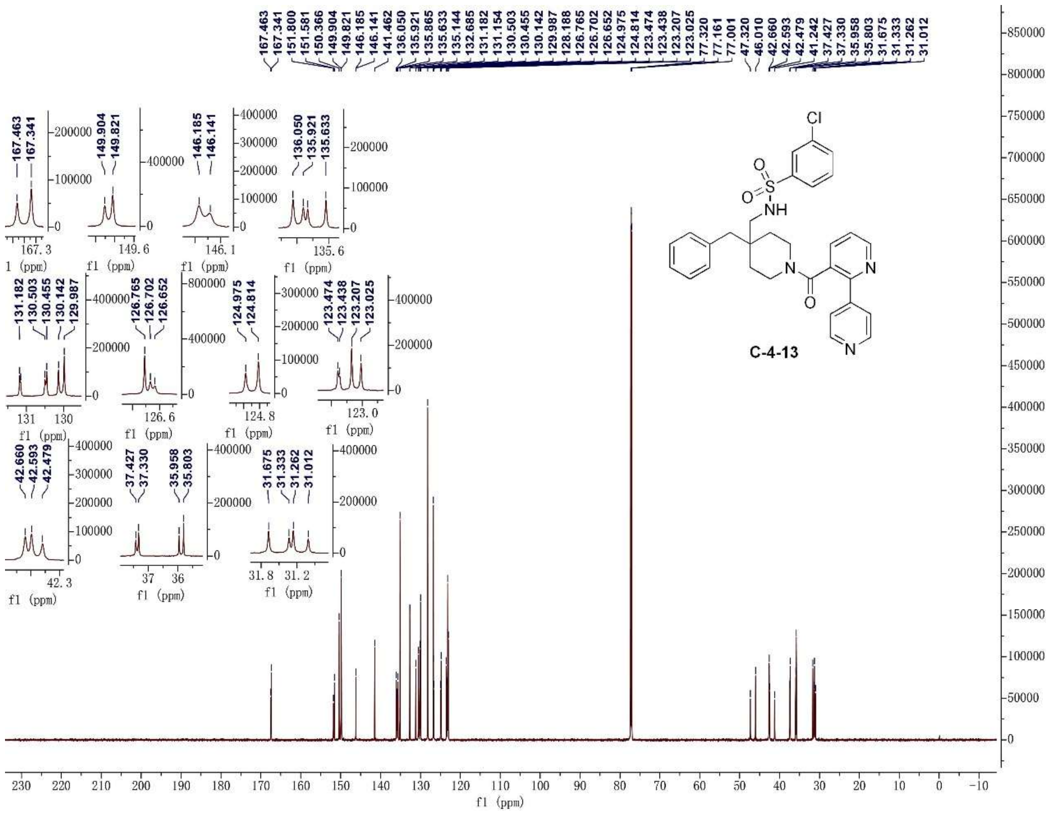

Figure S459: ^{13}C NMR spectrum of **C-4-13**

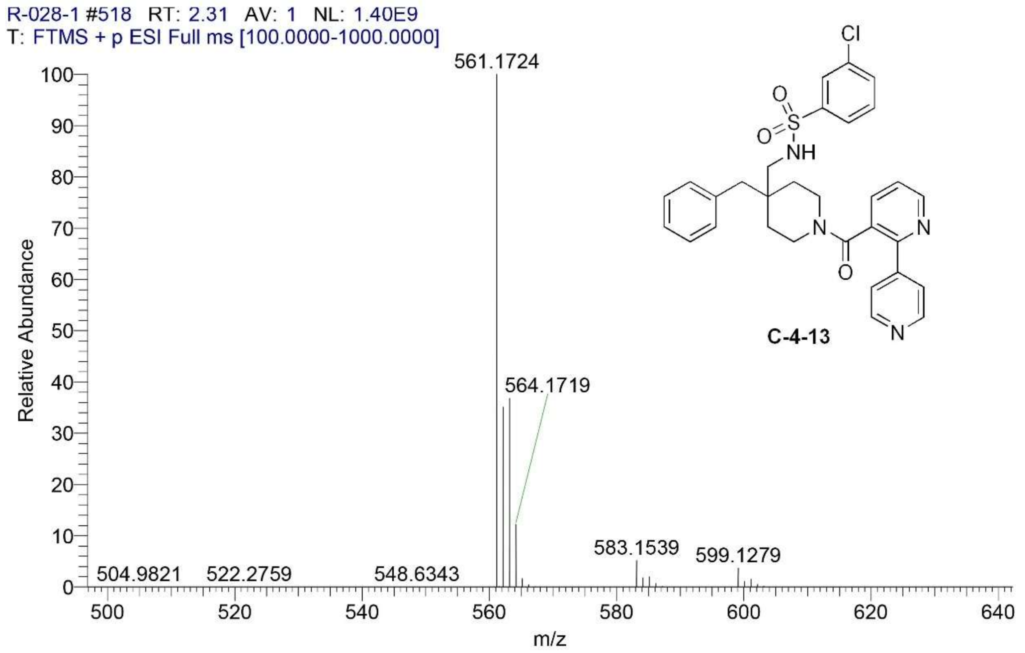

Figure S460: HR-MS (ESI/ion trap) spectrum of **C-4-13**

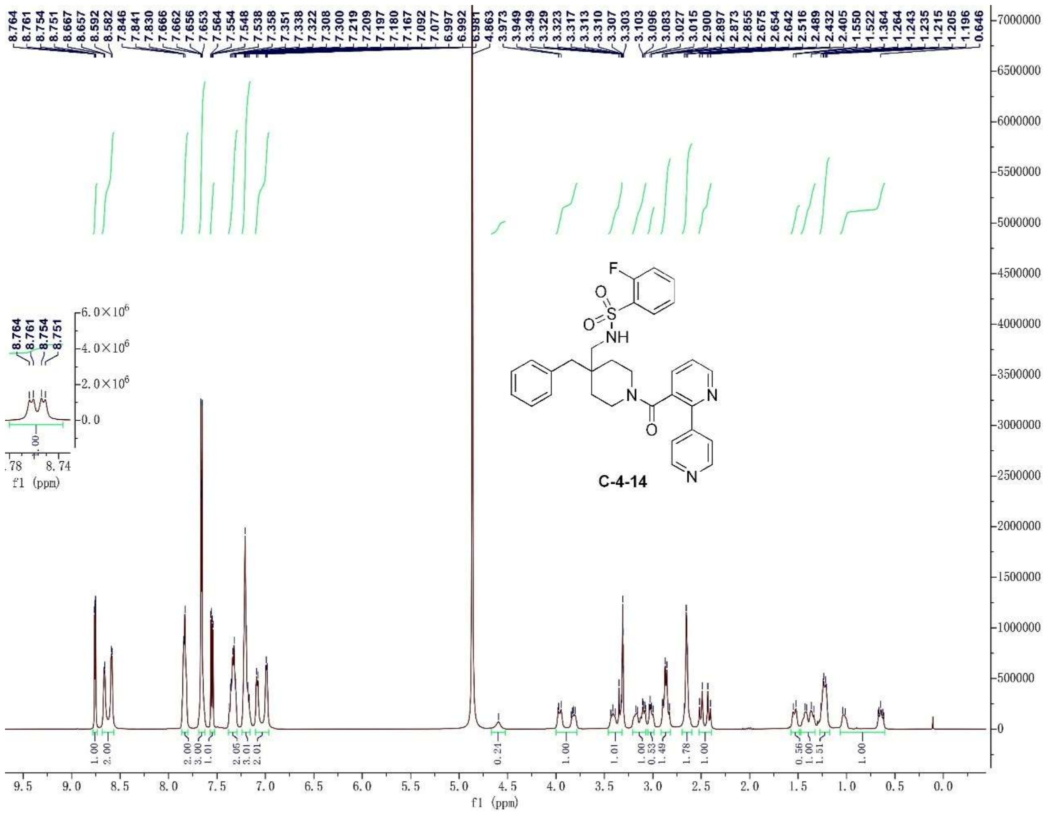

Figure S461: ^{1}H NMR spectrum of **C-4-14**

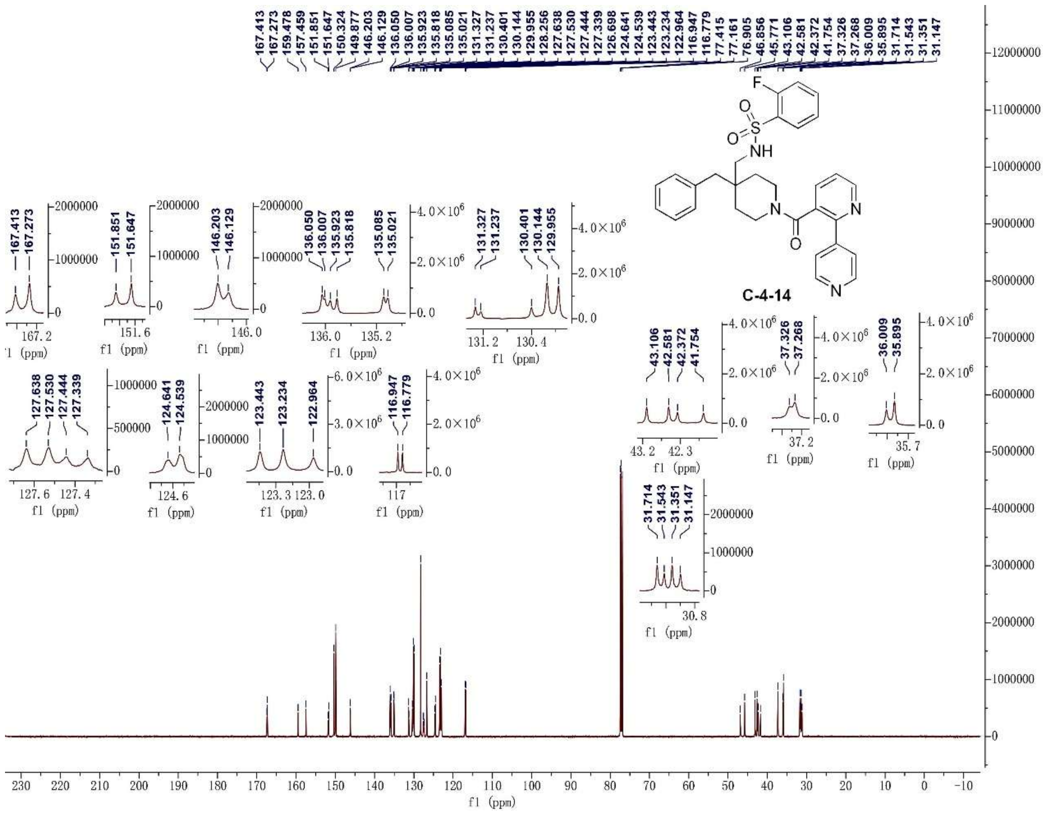

Figure S462: ^{13}C NMR spectrum of **C-4-14**

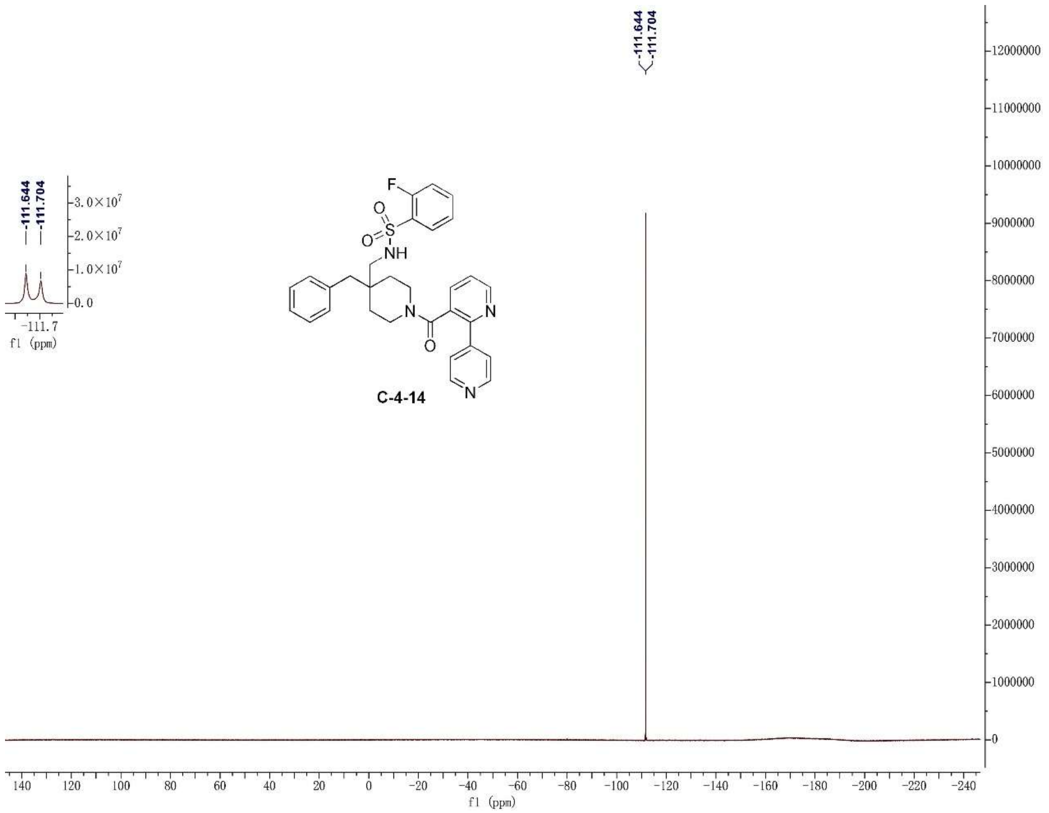

Figure S463: ^{19}F NMR spectrum of **C-4-14**

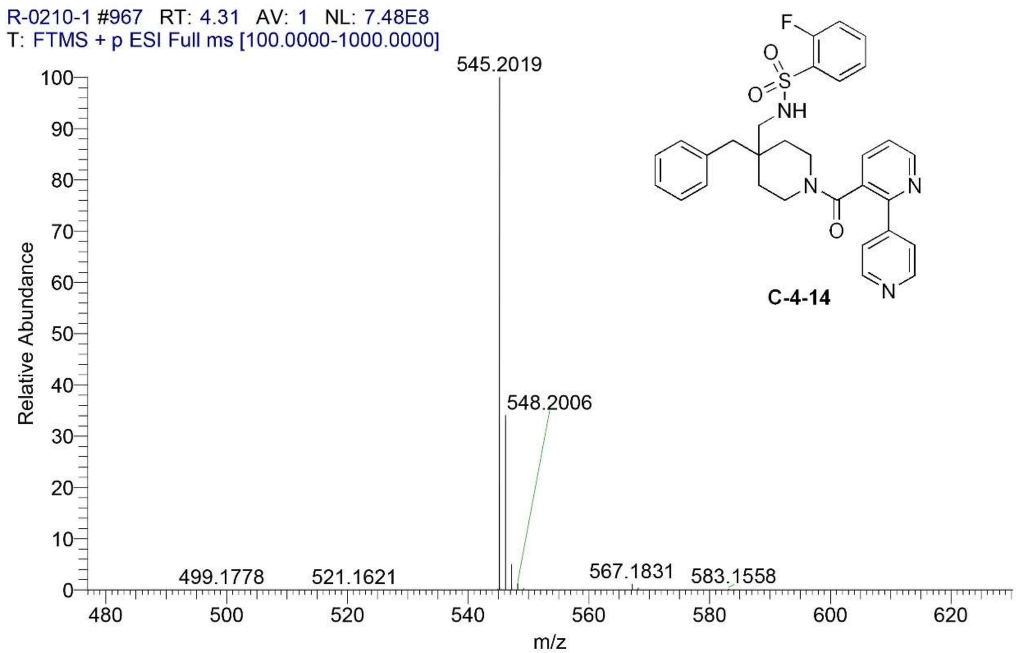

Figure S464: HR-MS (ESI/ion trap) spectrum of **C-4-14**

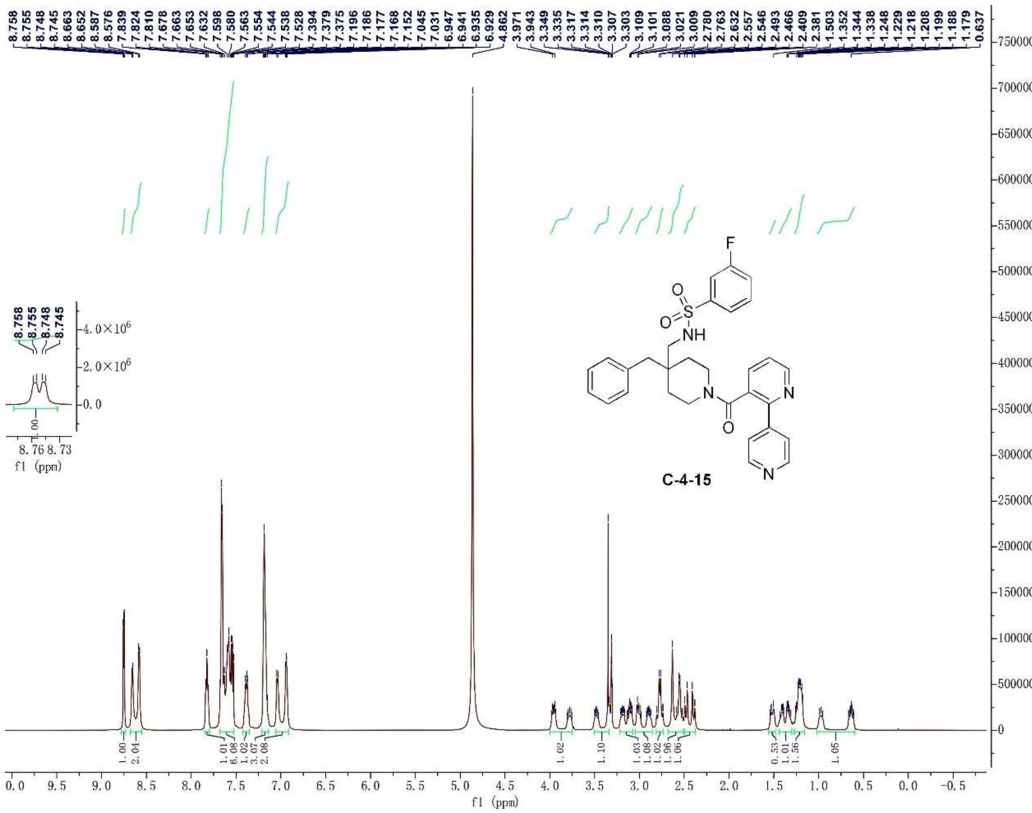

Figure S465: ^{1}H NMR spectrum of **C-4-15**

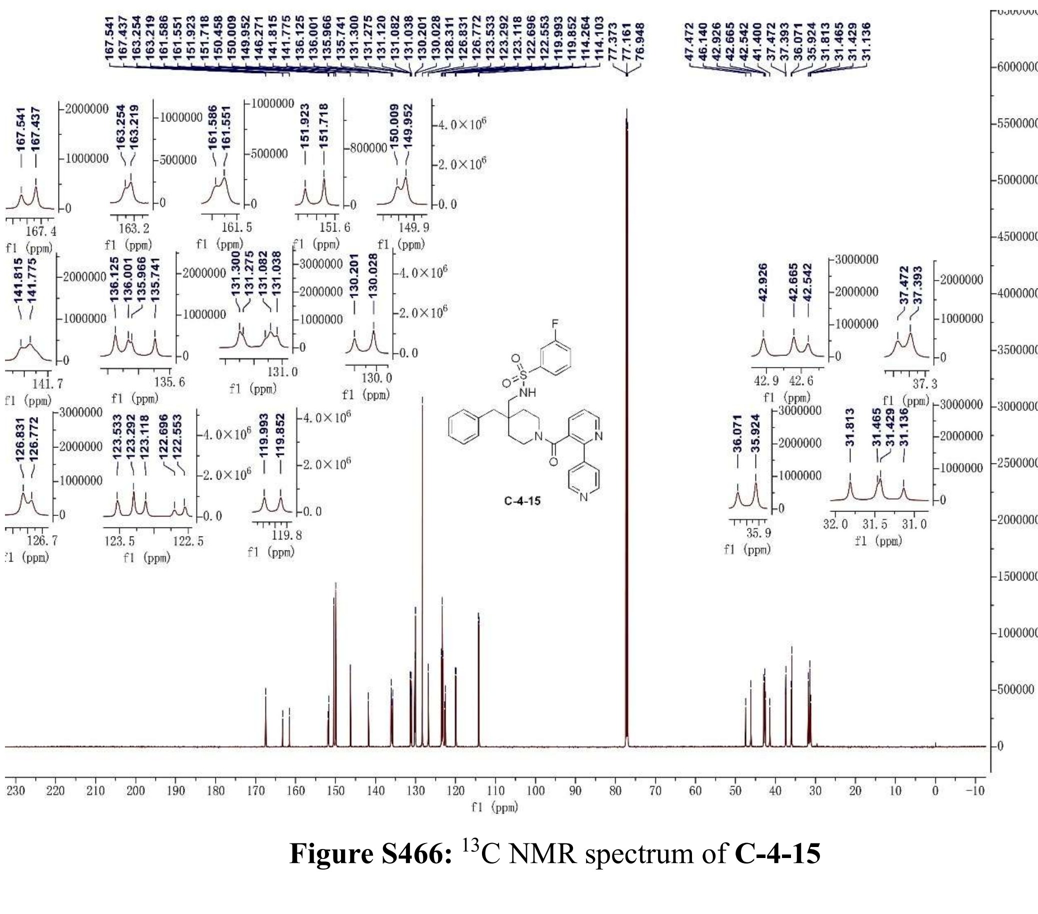

Figure S466: ^{13}C NMR spectrum of **C-4-15**

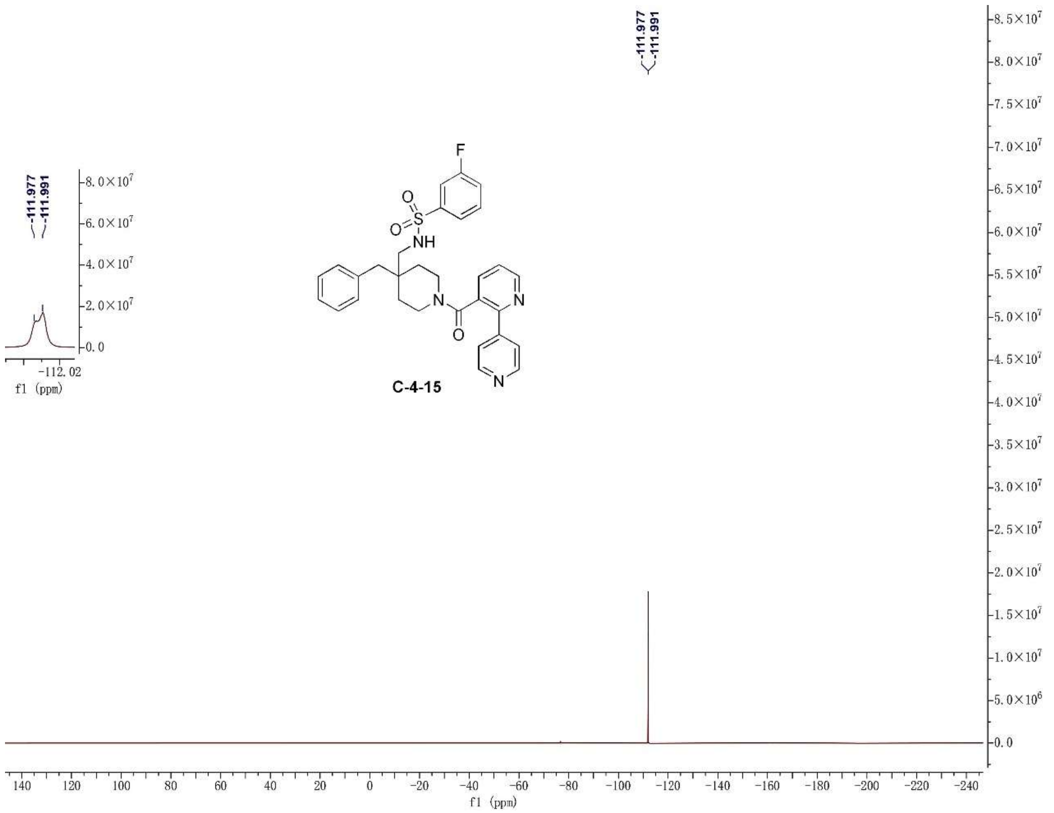

Figure S467: ^{19}F NMR spectrum of **C-4-15**

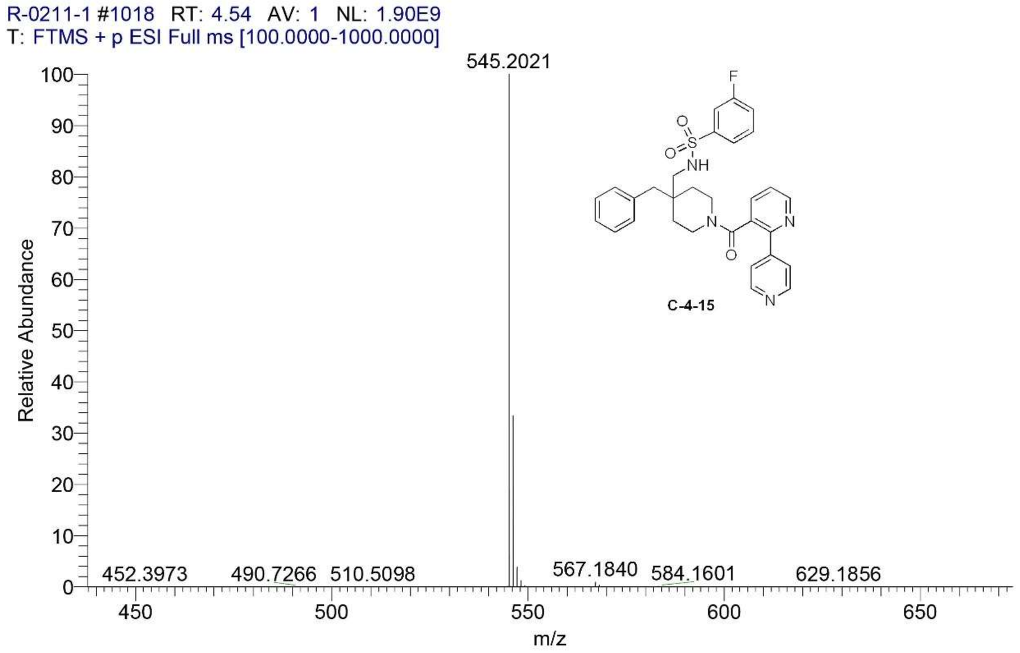

Figure S468: HR-MS (ESI/ion trap) spectrum of **C-4-15**

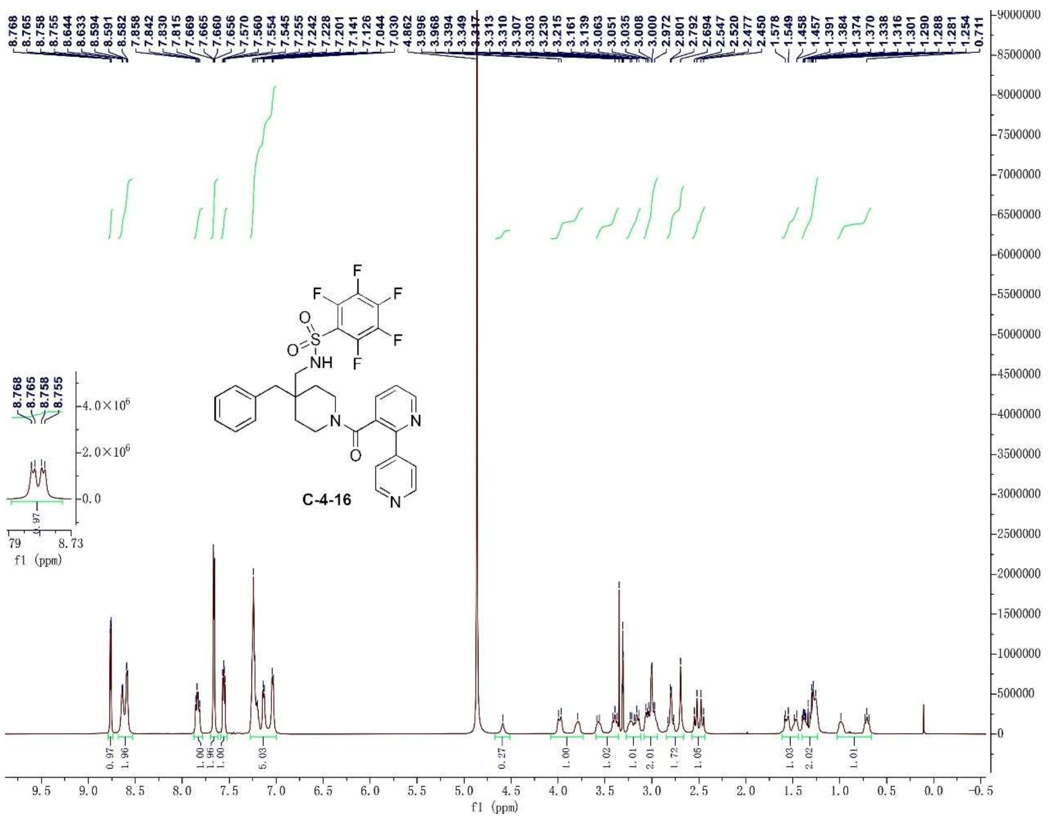

Figure S469: ^{1}H NMR spectrum of **C-4-16**

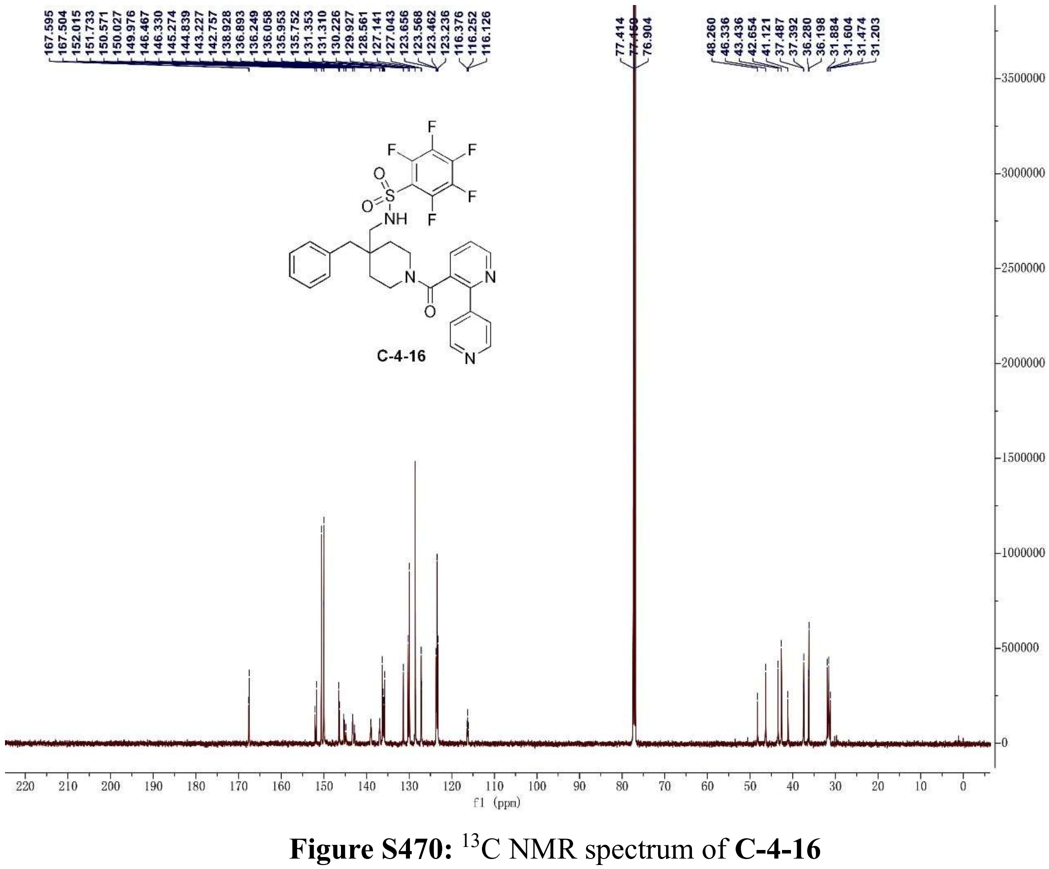

Figure S470: ^{13}C NMR spectrum of **C-4-16**

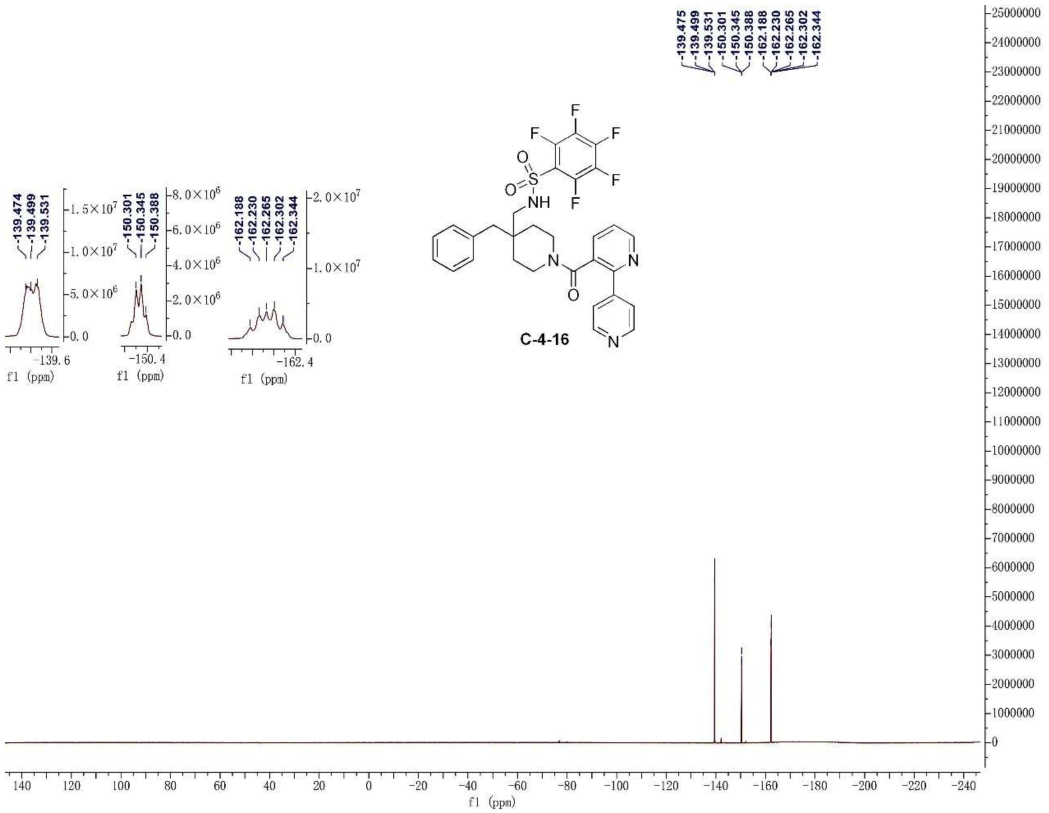

Figure S471: ^{19}F NMR spectrum of **C-4-16**

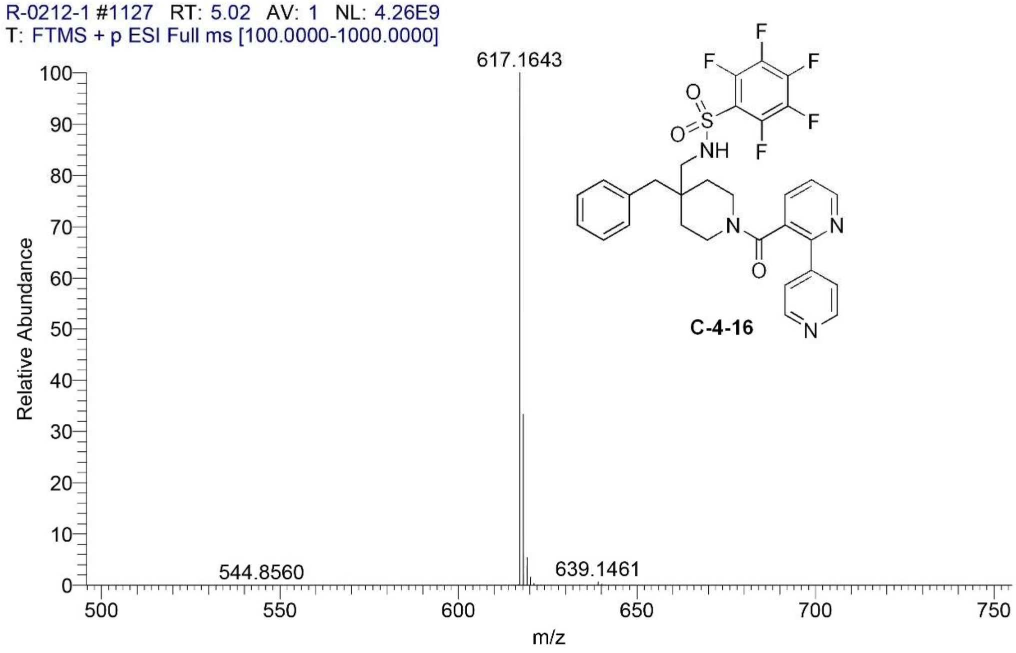

Figure S472: HR-MS (ESI/ion trap) spectrum of **C-4-16**

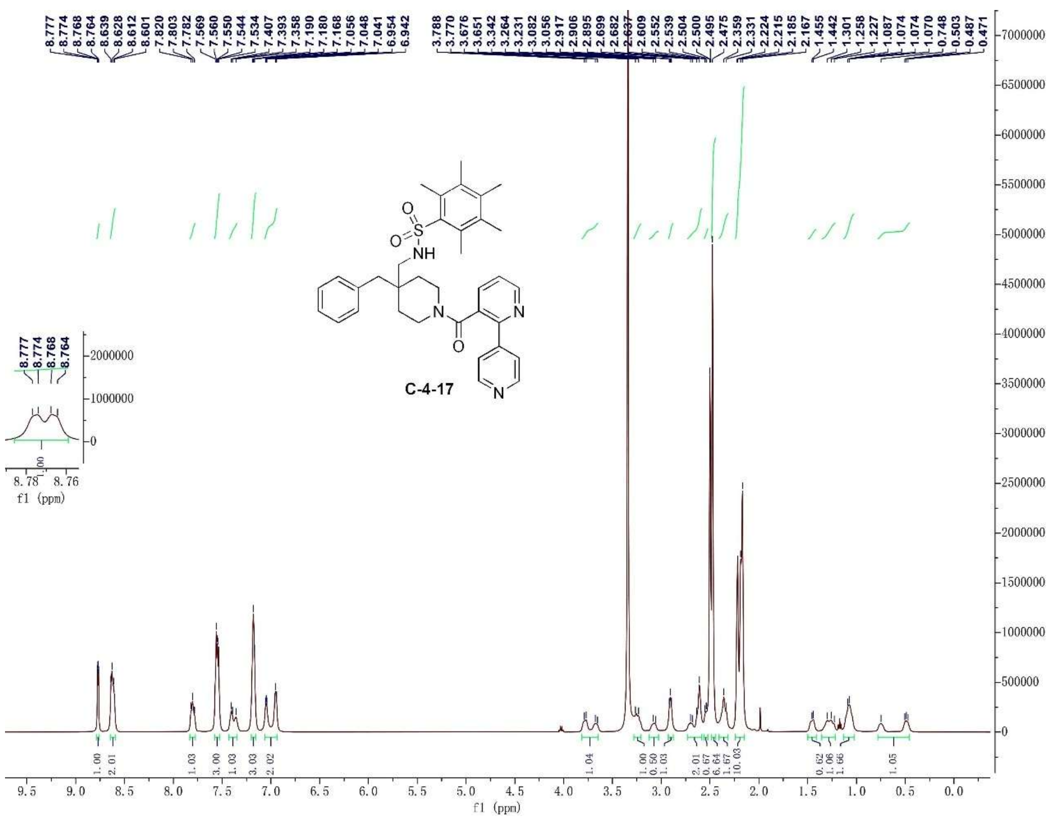

Figure S473: ^{1}H NMR spectrum of **C-4-17**

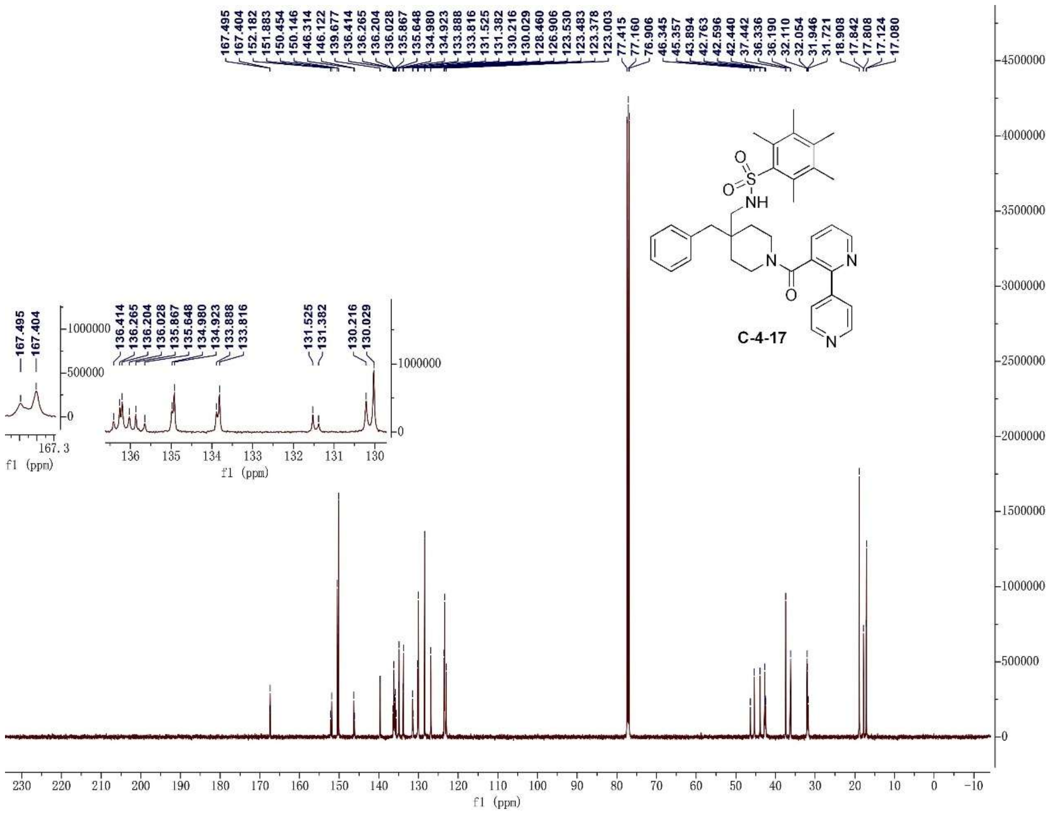

Figure S474: ^{13}C NMR spectrum of **C-4-17**

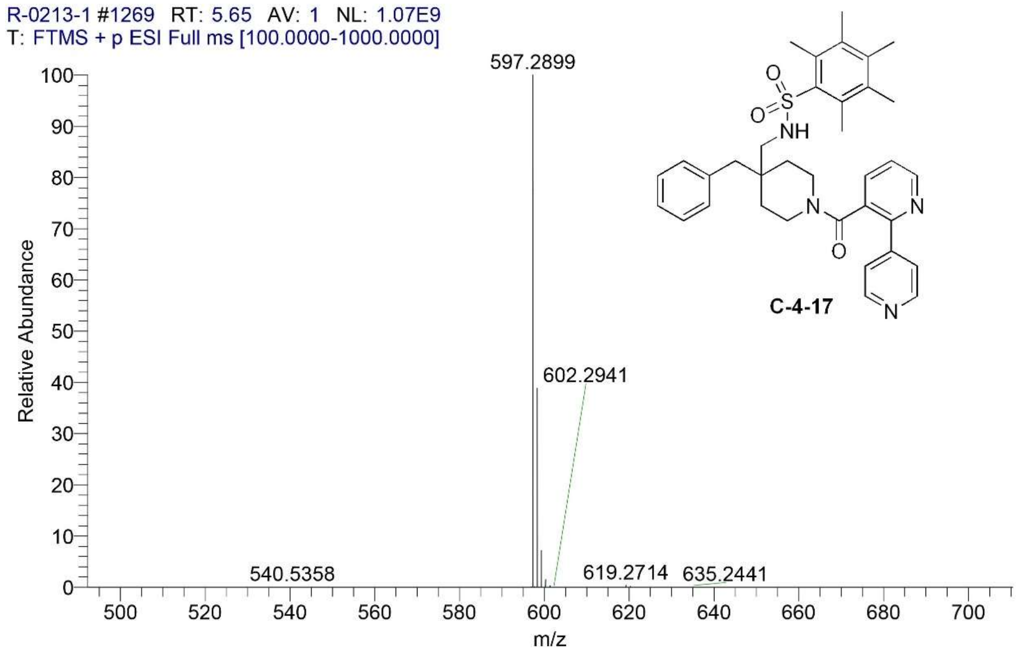

Figure S475: HR-MS (ESI/ion trap) spectrum of **C-4-17**

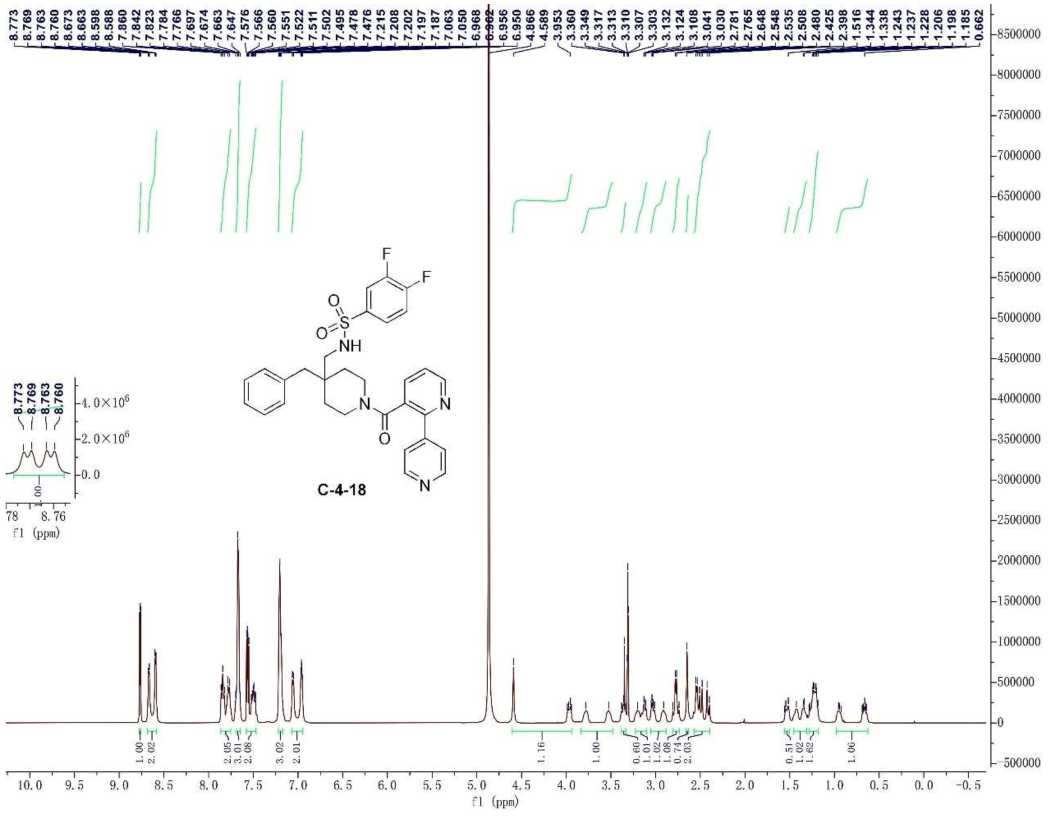

Figure S476: ^{1}H NMR spectrum of **C-4-18**

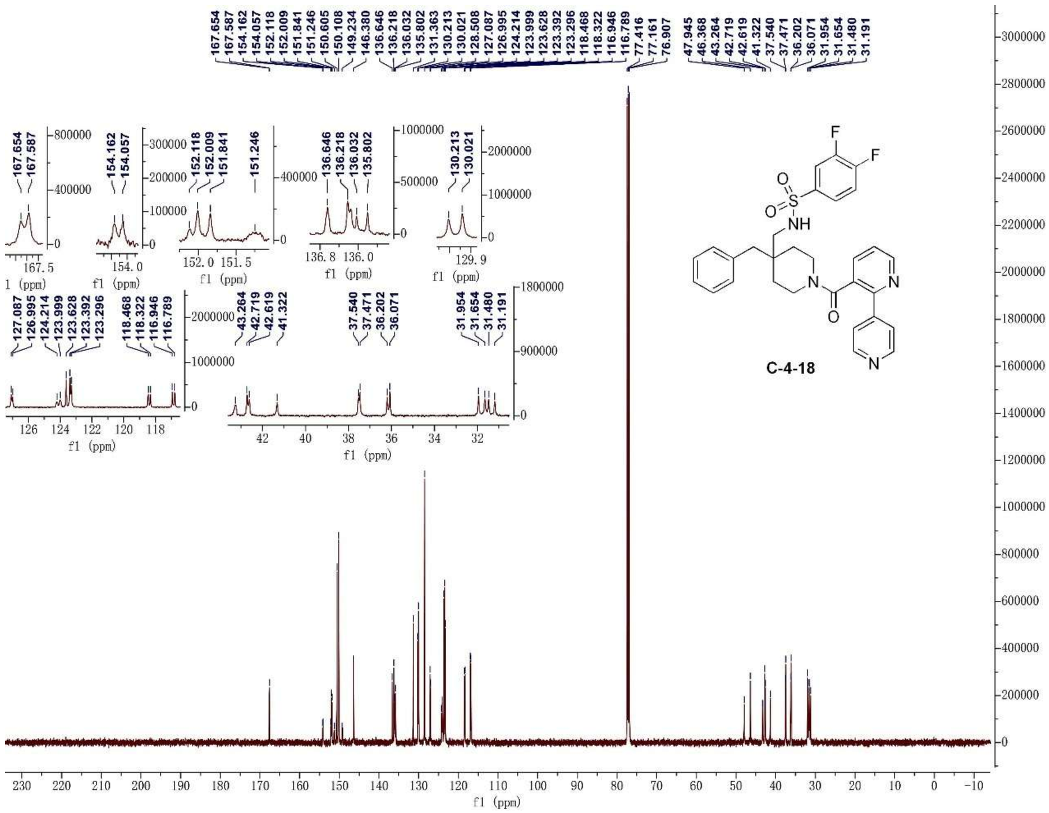

Figure S477: ^{13}C NMR spectrum of **C-4-18**

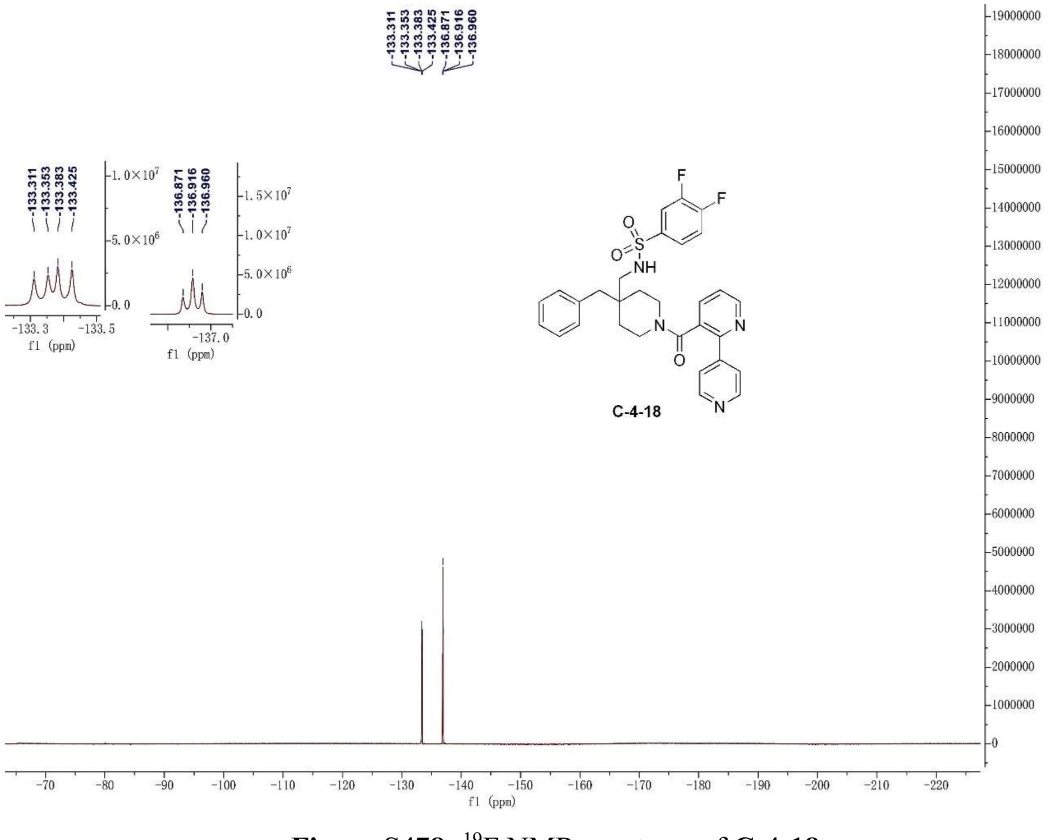

Figure S478: ^{19}F NMR spectrum of **C-4-18**

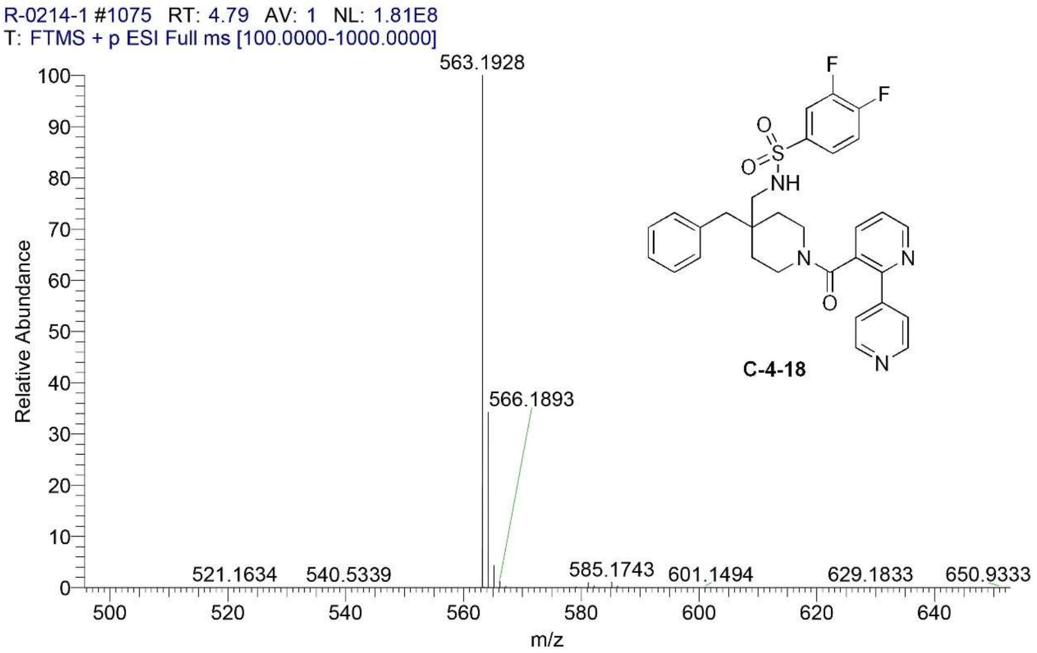

Figure S479: HR-MS (ESI/ion trap) spectrum of **C-4-18**

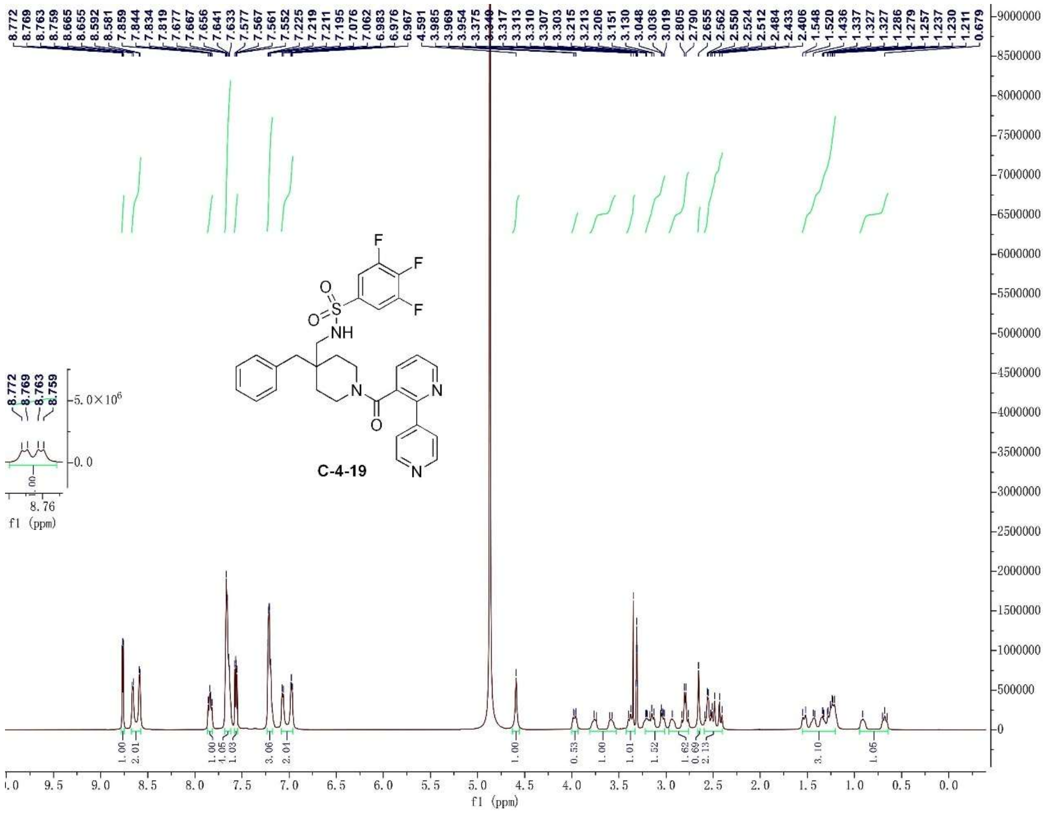

Figure S480: ^{1}H NMR spectrum of **C-4-19**

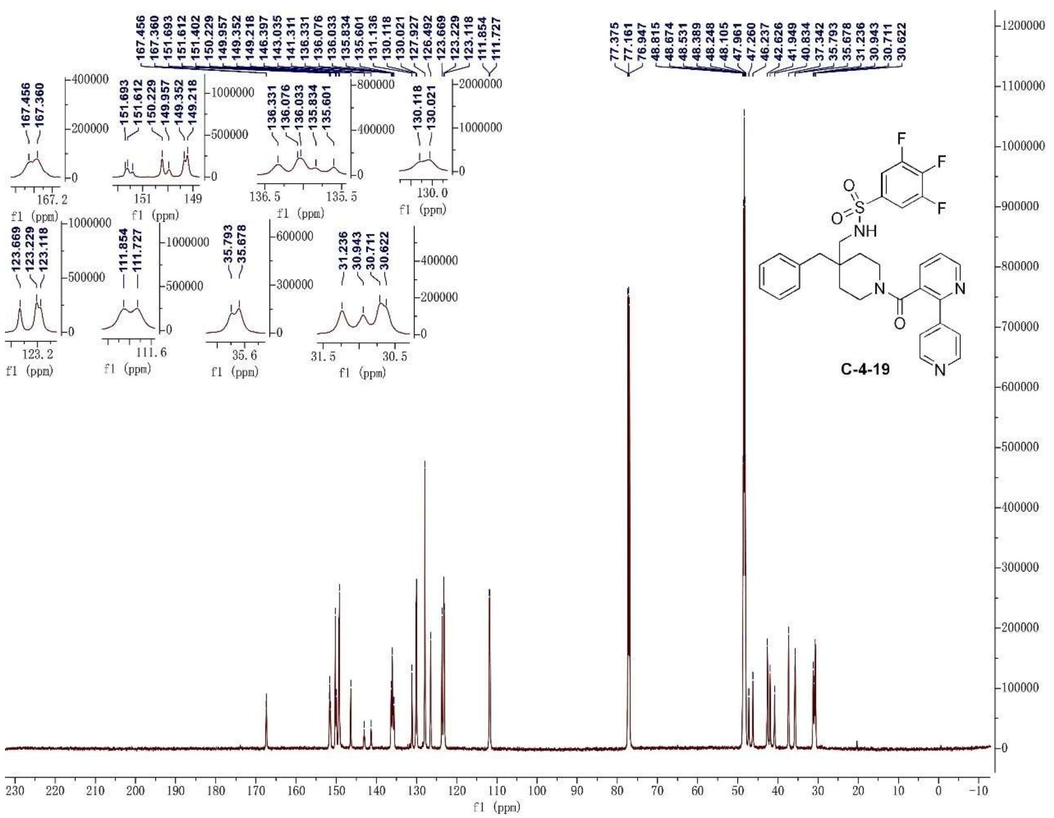

Figure S481: ^{13}C NMR spectrum of **C-4-19**

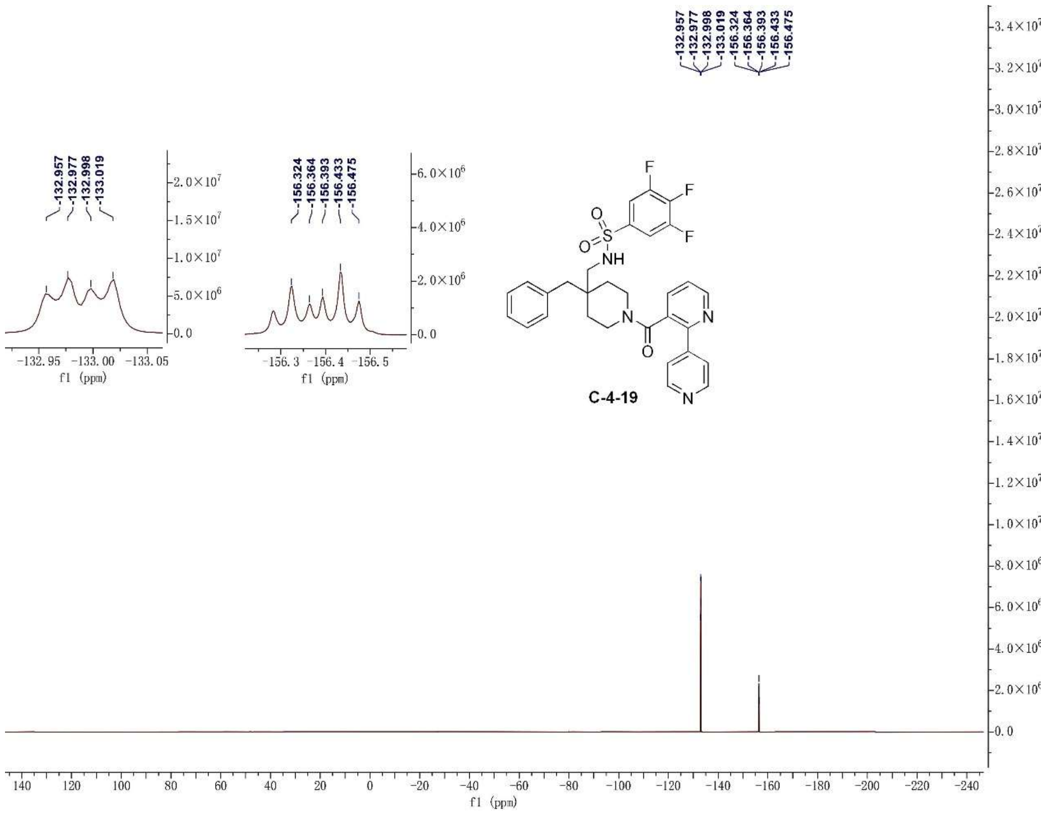

Figure S482: ^{19}F NMR spectrum of **C-4-19**

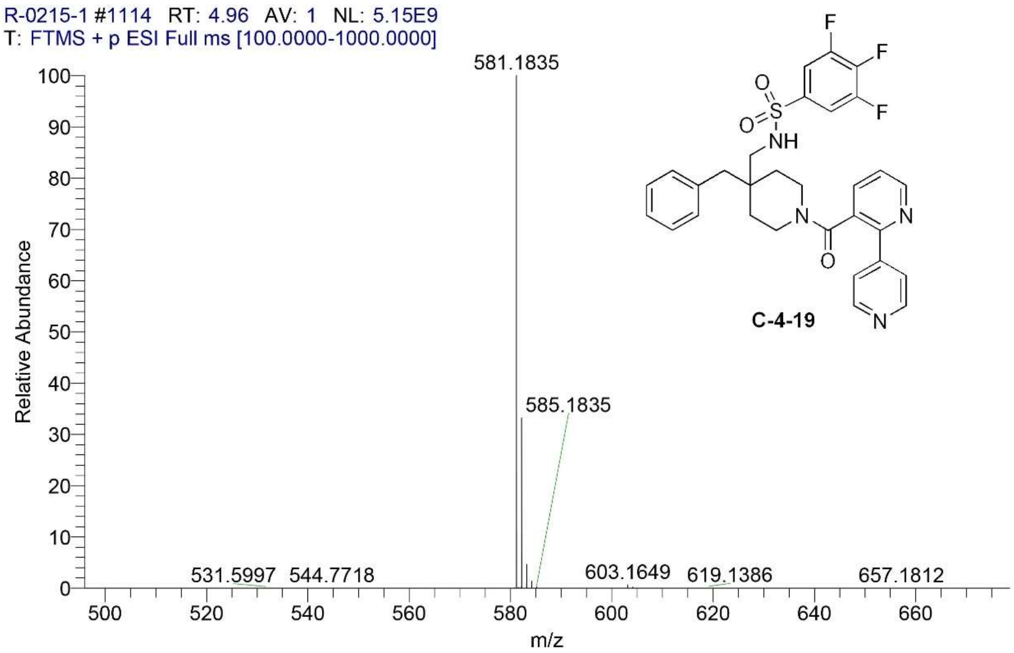

Figure S483: HR-MS (ESI/ion trap) spectrum of **C-4-19**

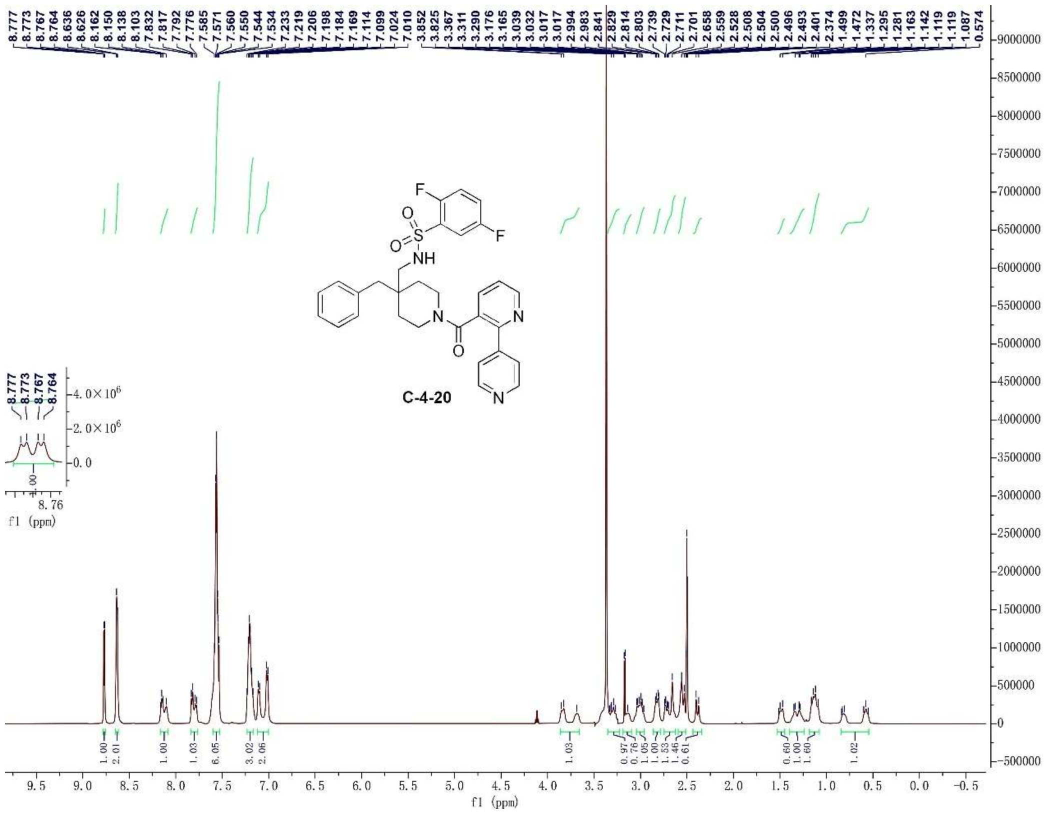

Figure S484: ^{1}H NMR spectrum of **C-4-20**

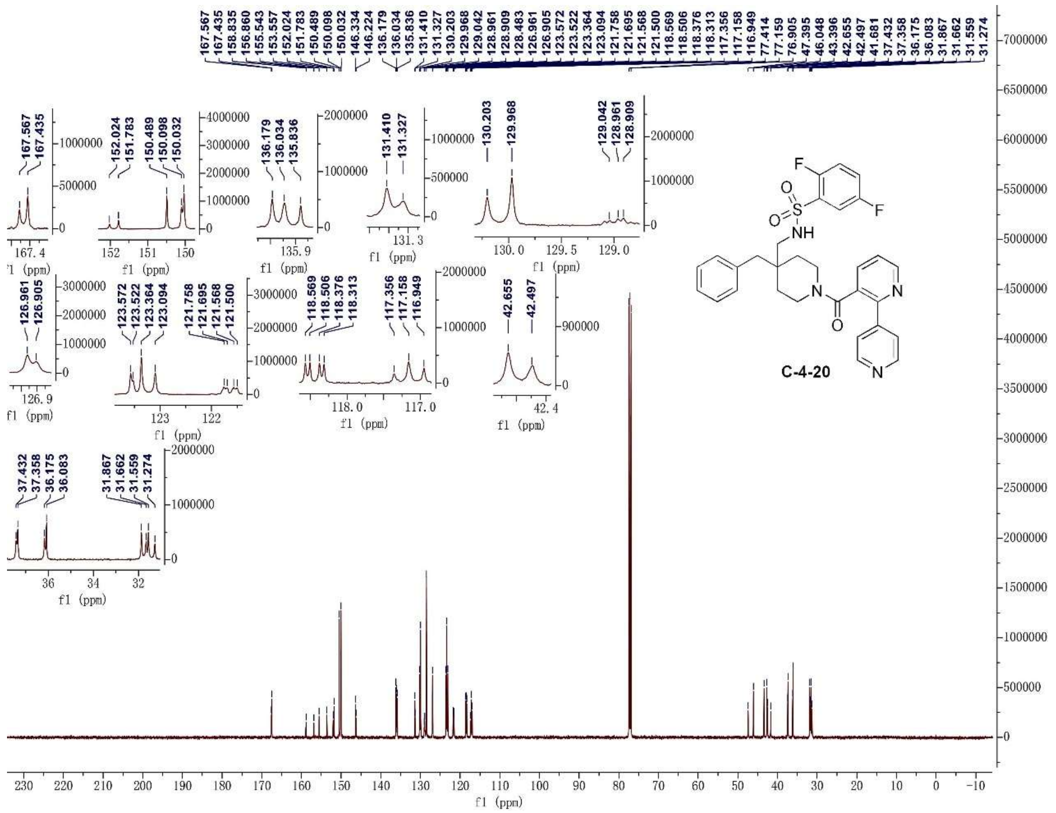

Figure S485: ^{13}C NMR spectrum of **C-4-20**

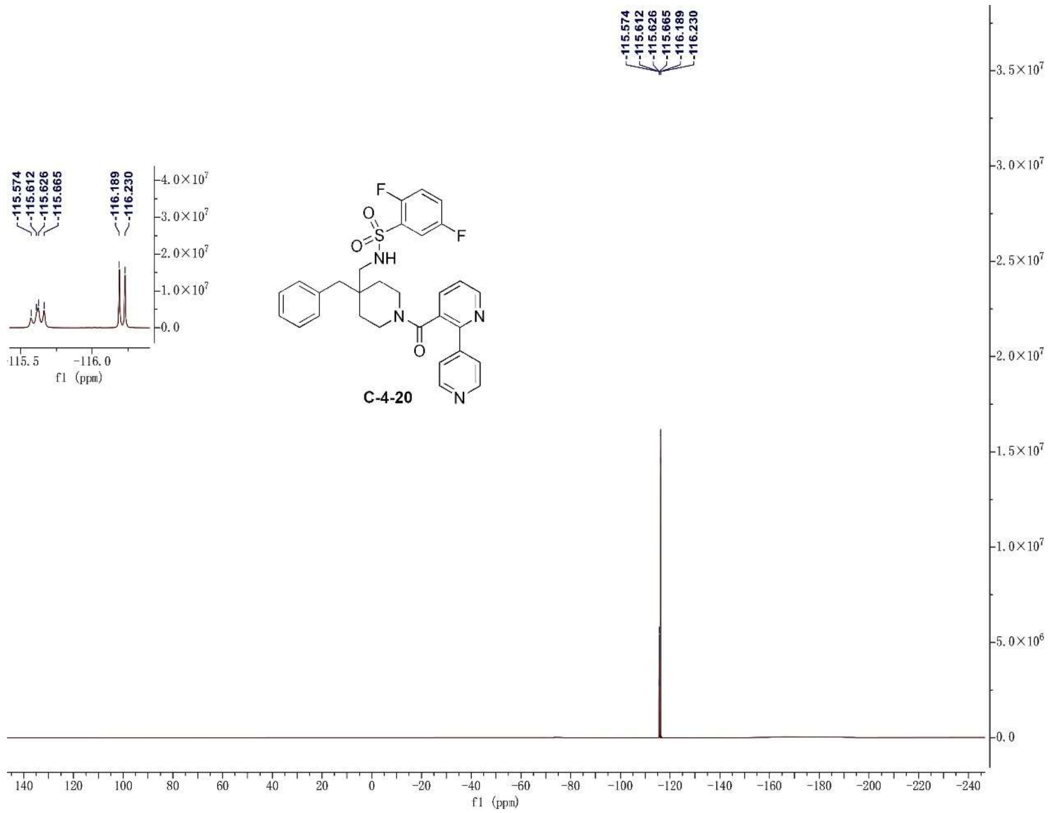

Figure S486: ^{19}F NMR spectrum of **C-4-20**

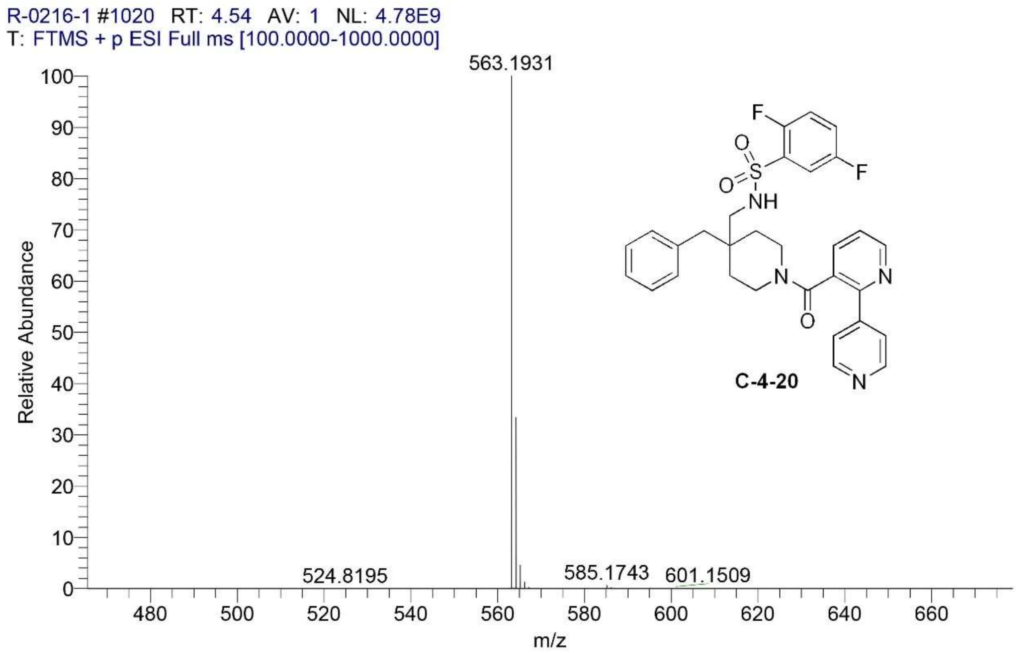

Figure S487: HR-MS (ESI/ion trap) spectrum of **C-4-20**

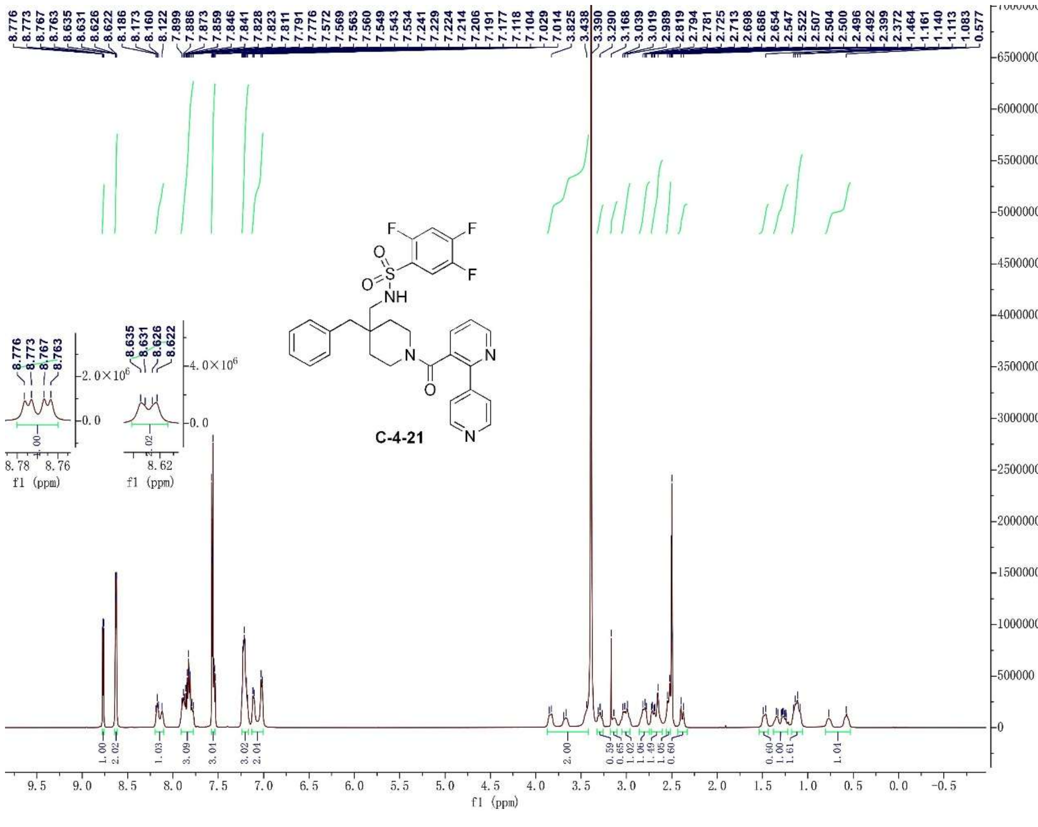

Figure S488: ^{1}H NMR spectrum of **C-4-21**

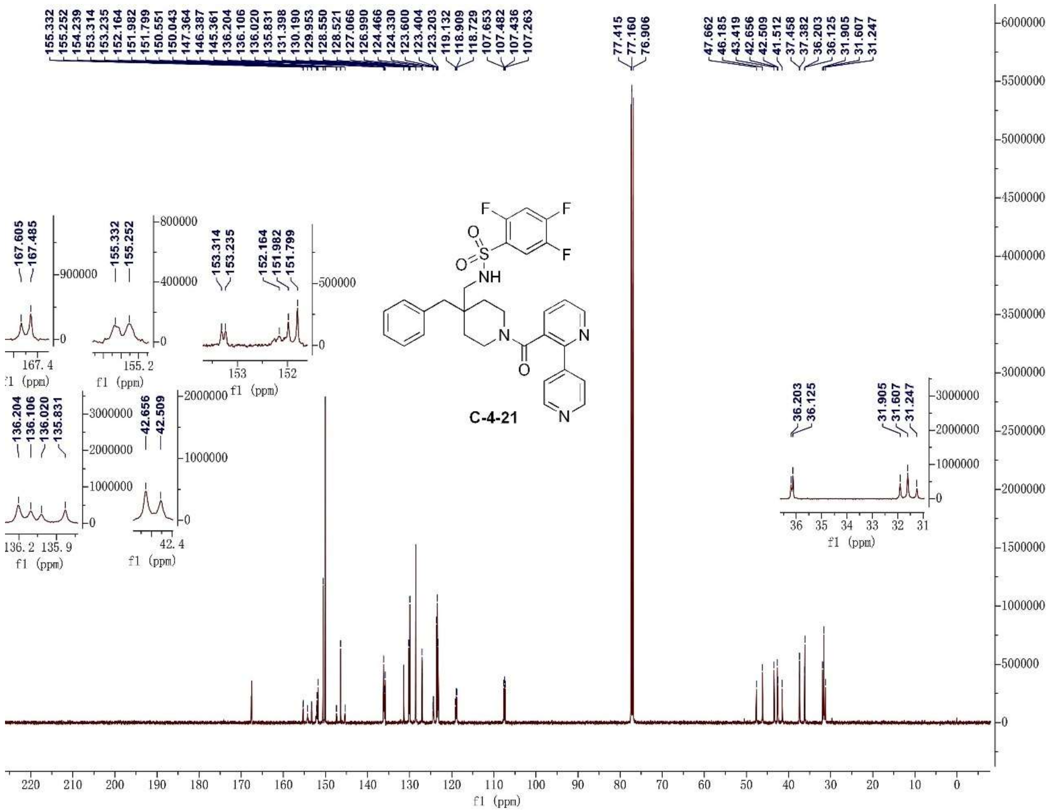

Figure S489: ^{13}C NMR spectrum of **C-4-21**

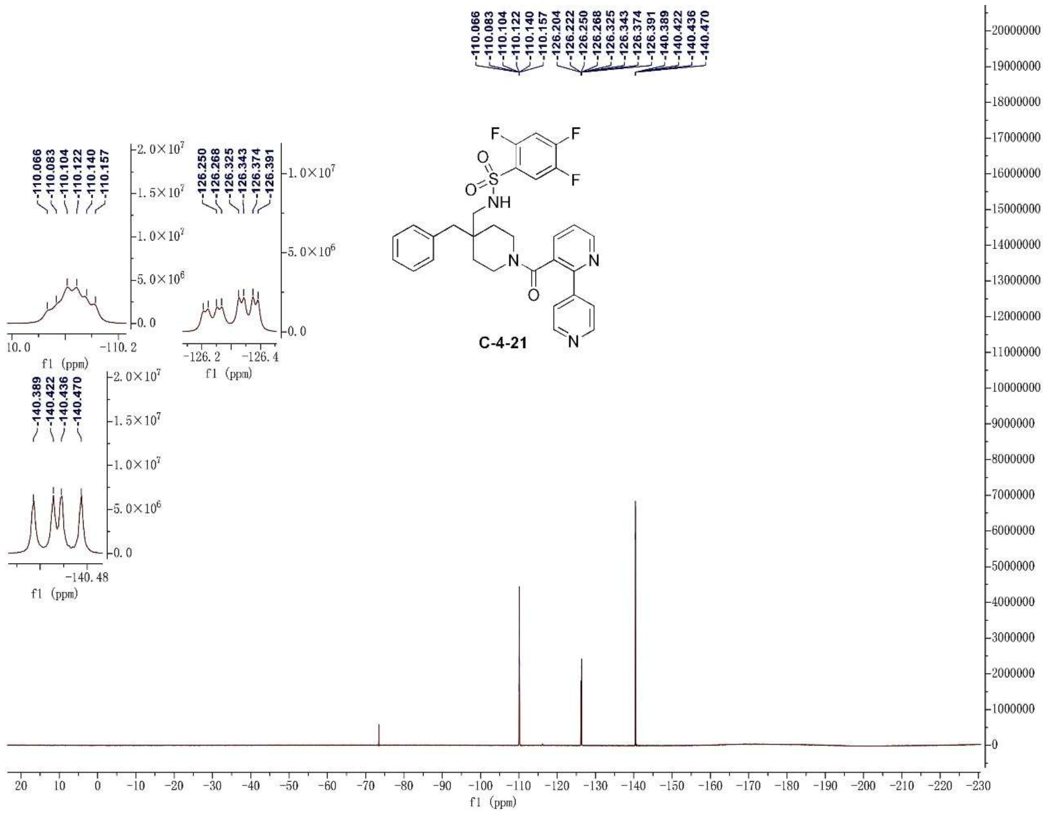

Figure S490: ^{19}F NMR spectrum of **C-4-21**

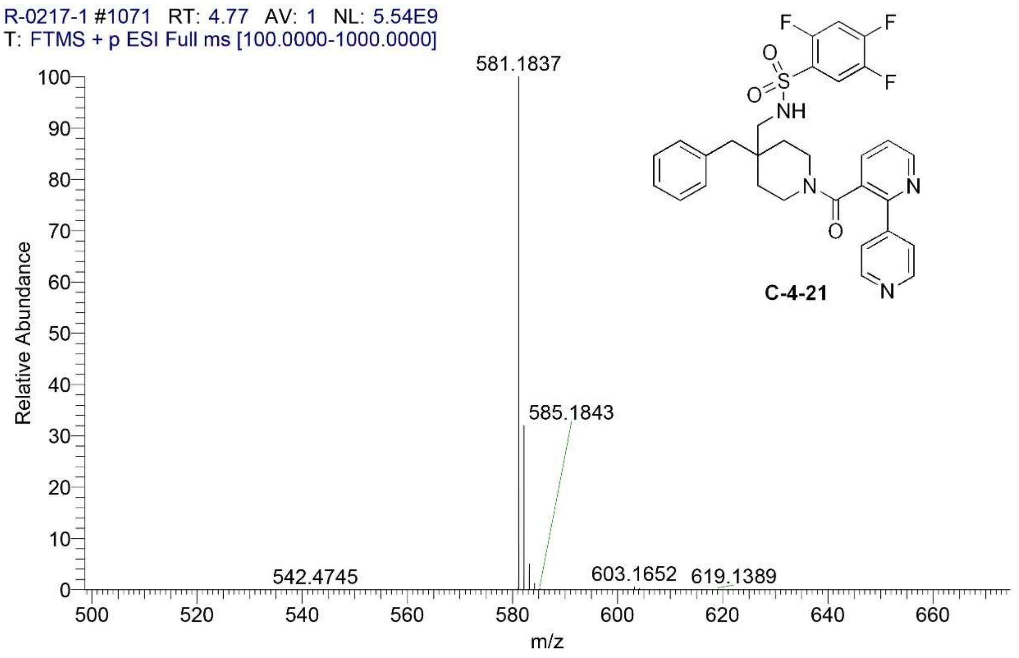

Figure S491: HR-MS (ESI/ion trap) spectrum of **C-4-21**

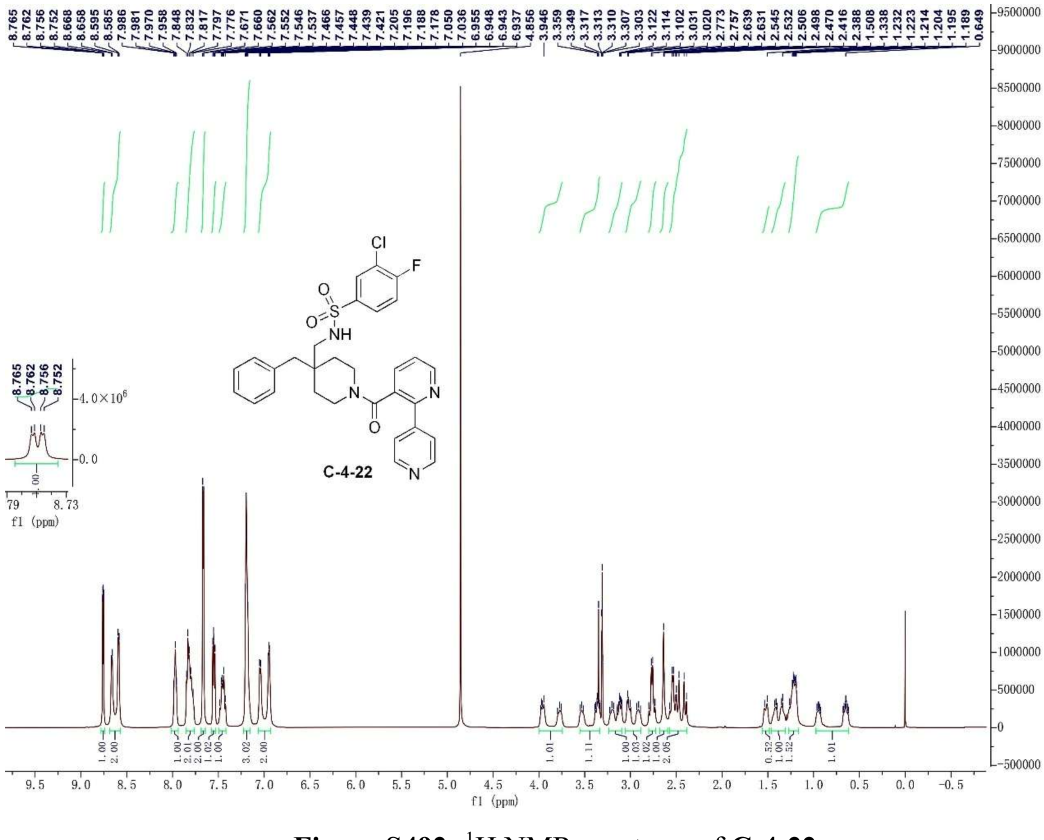

Figure S492: ^{1}H NMR spectrum of **C-4-22**

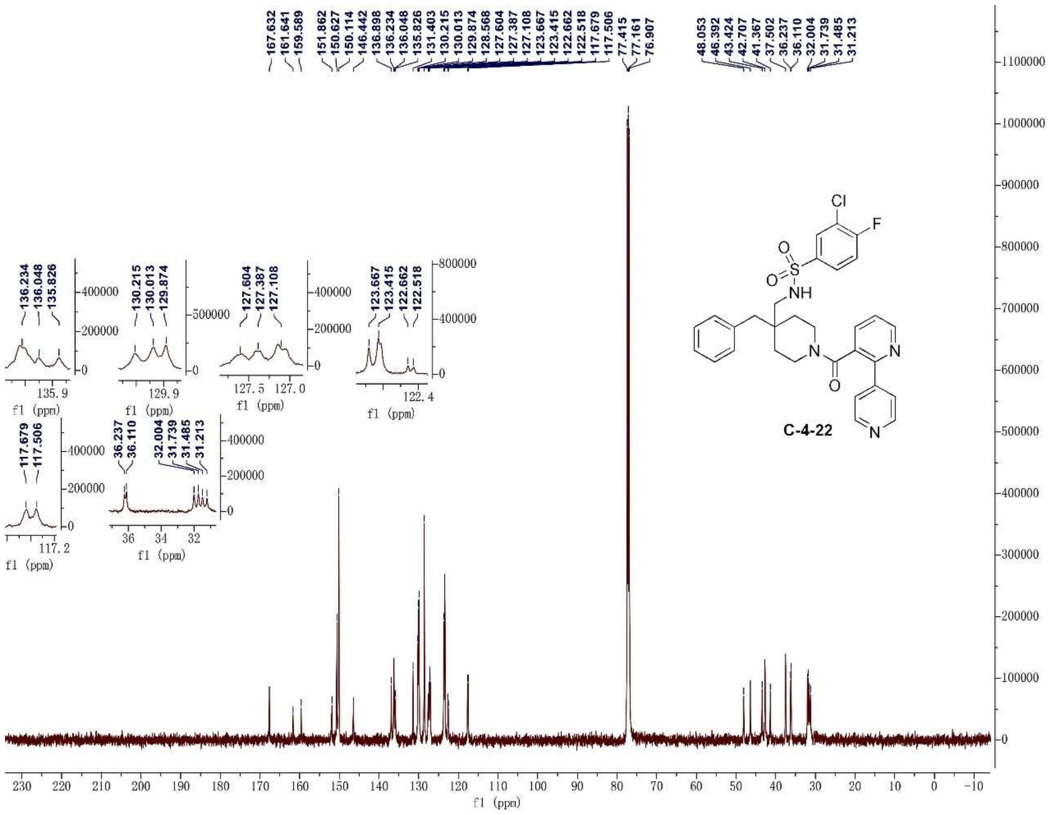

Figure S493: ^{13}C NMR spectrum of **C-4-22**

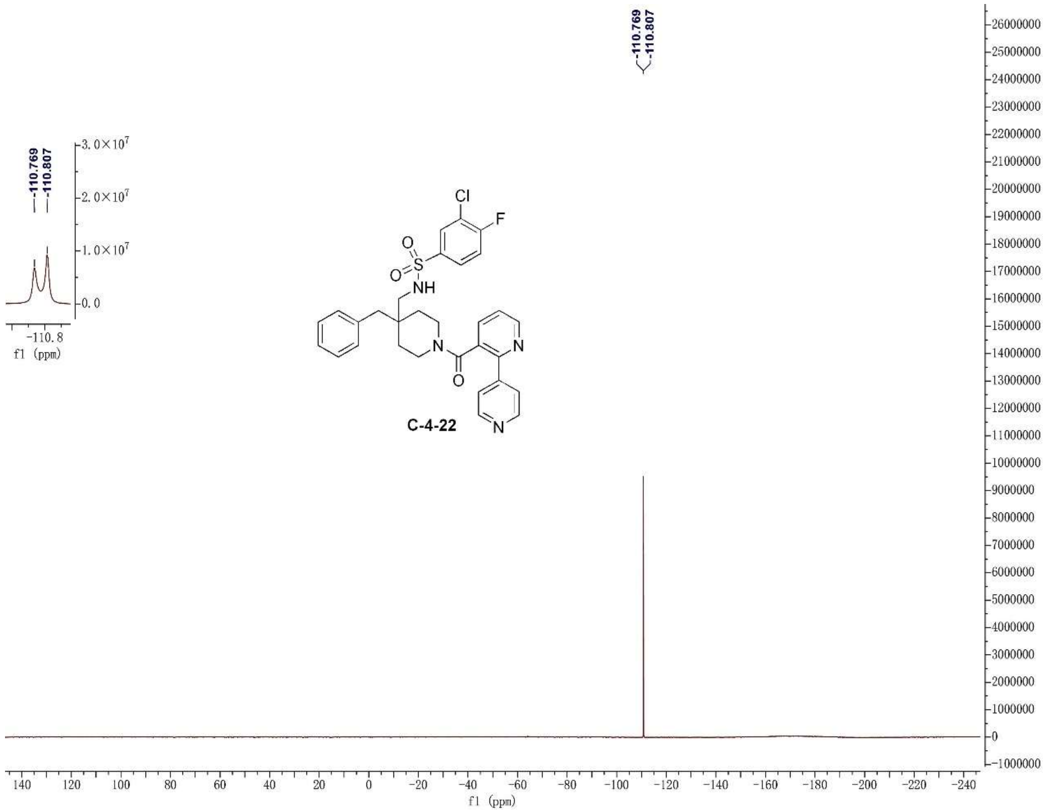

Figure S494: ^{19}F NMR spectrum of **C-4-22**

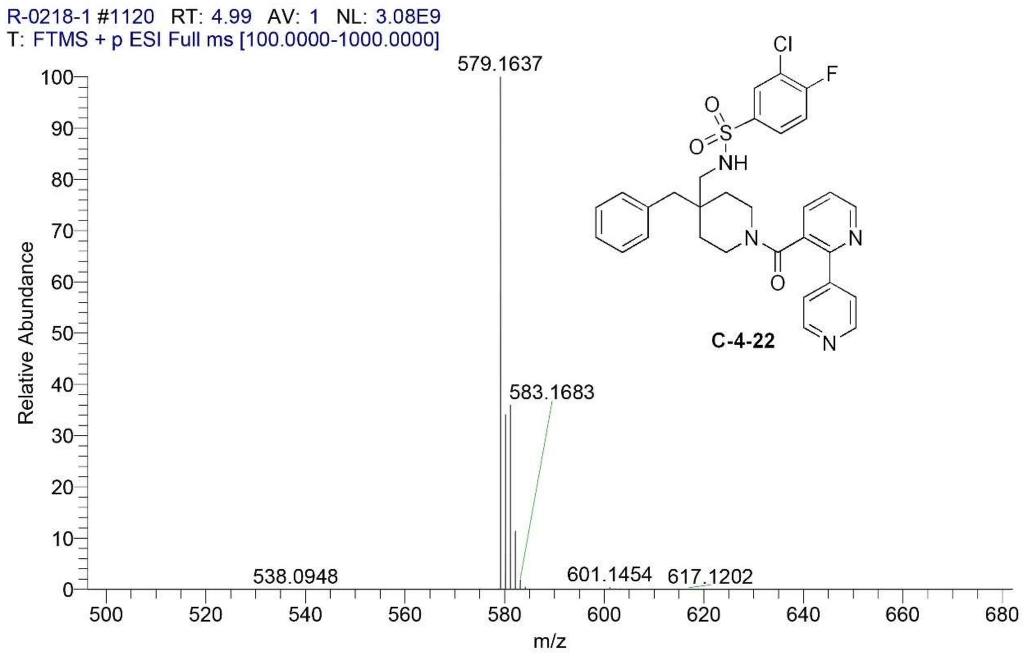

Figure S495: HR-MS (ESI/ion trap) spectrum of **C-4-22**

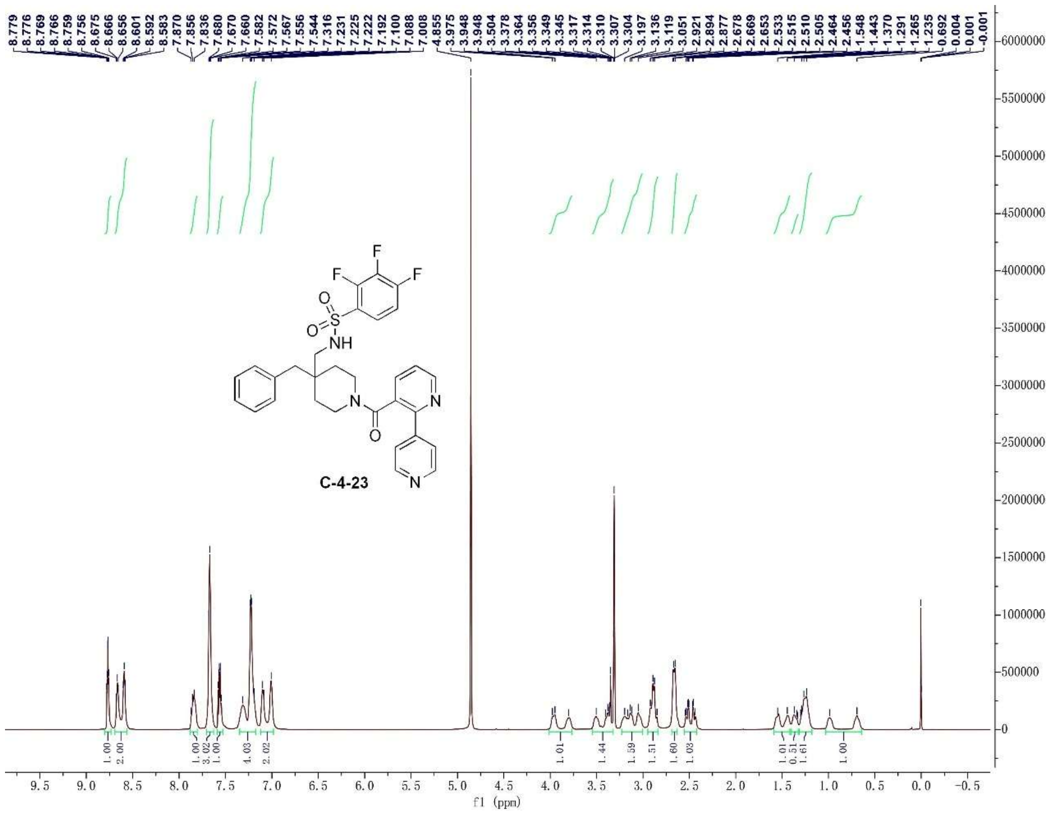

Figure S496: ^{1}H NMR spectrum of **C-4-23**

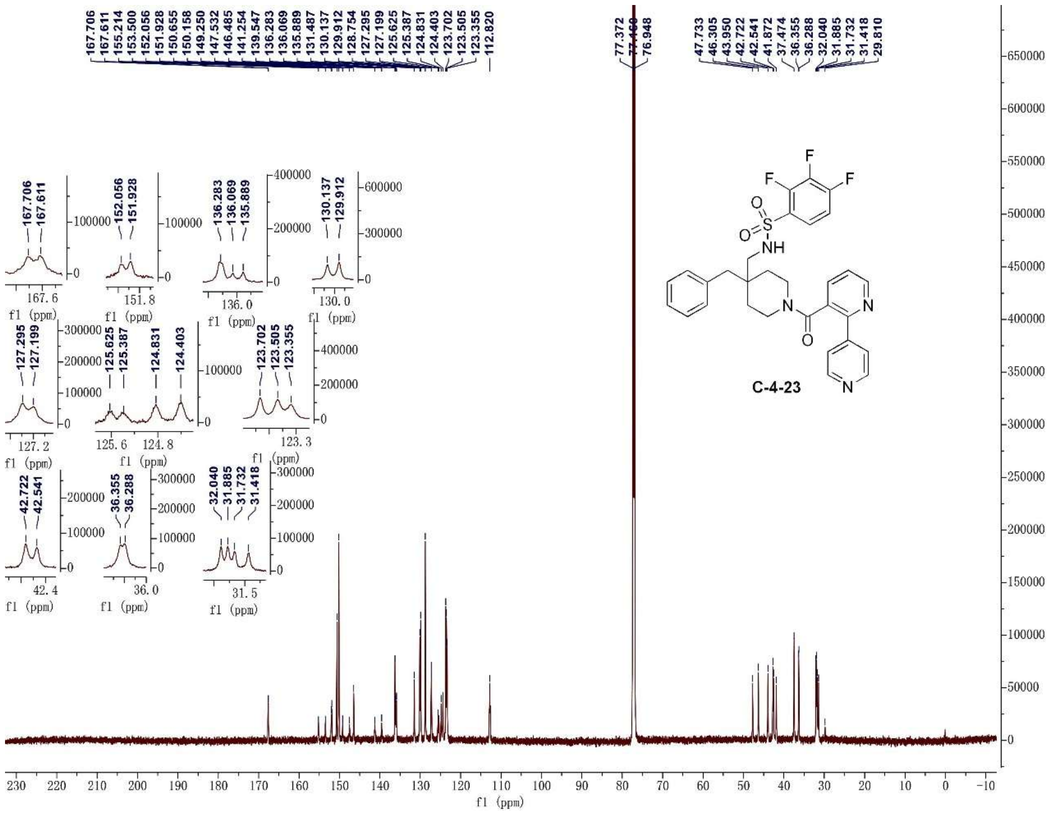

Figure S497: ^{13}C NMR spectrum of **C-4-23**

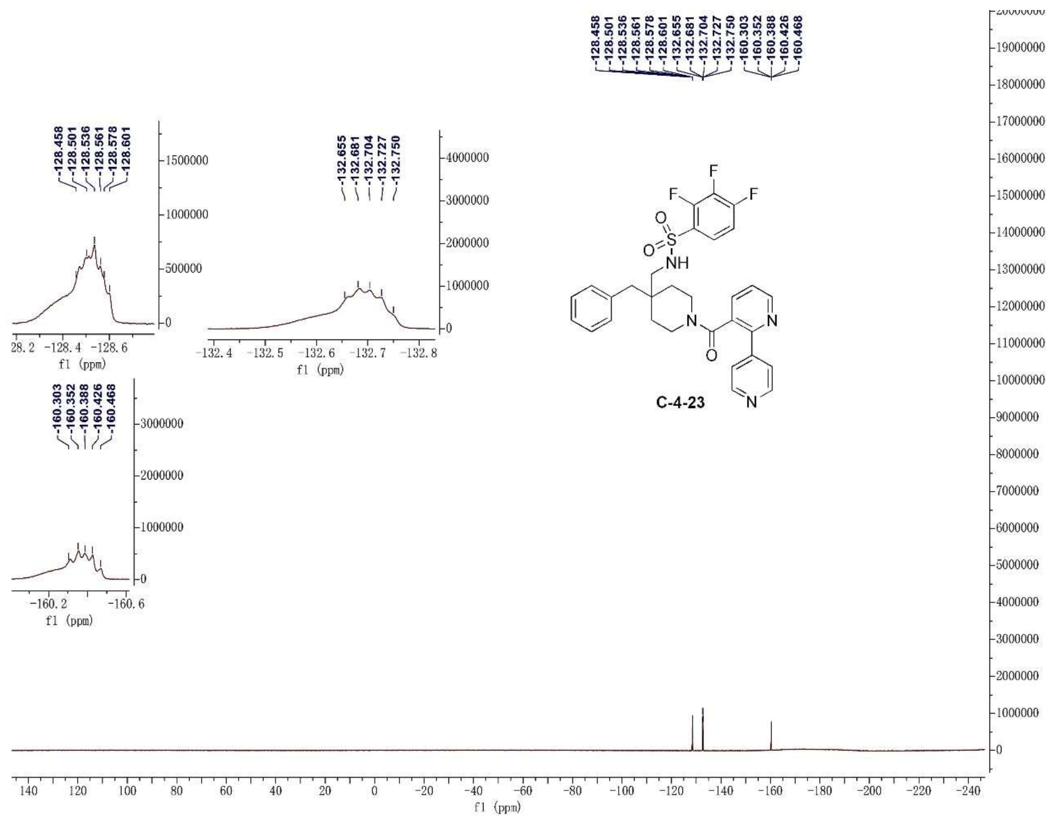

Figure S498: ^{19}F NMR spectrum of **C-4-23**

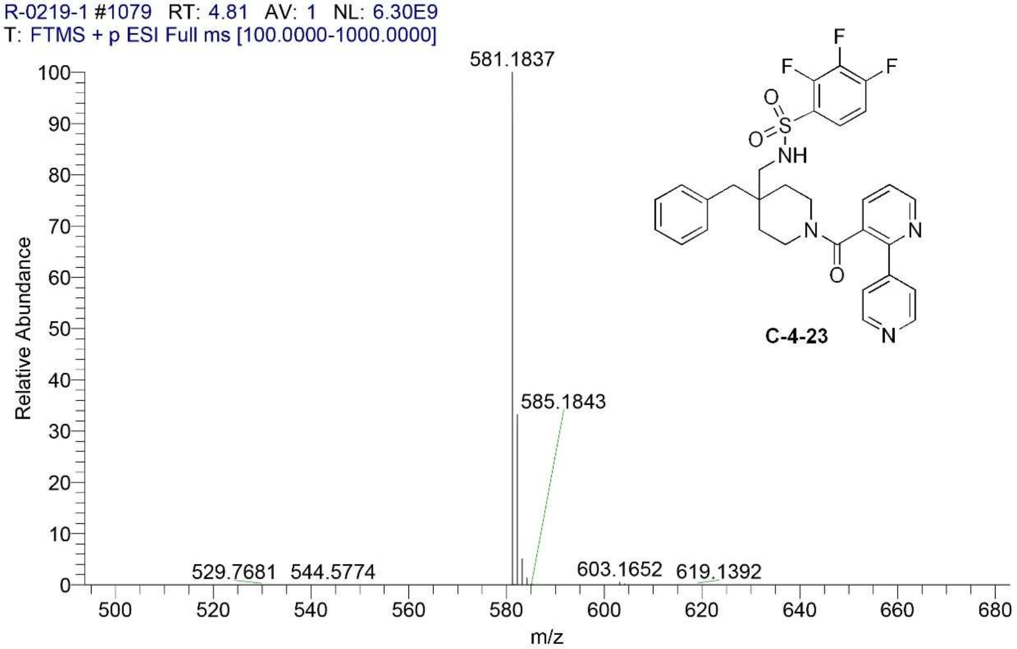

Figure S499: HR-MS (ESI/ion trap) spectrum of **C-4-23**

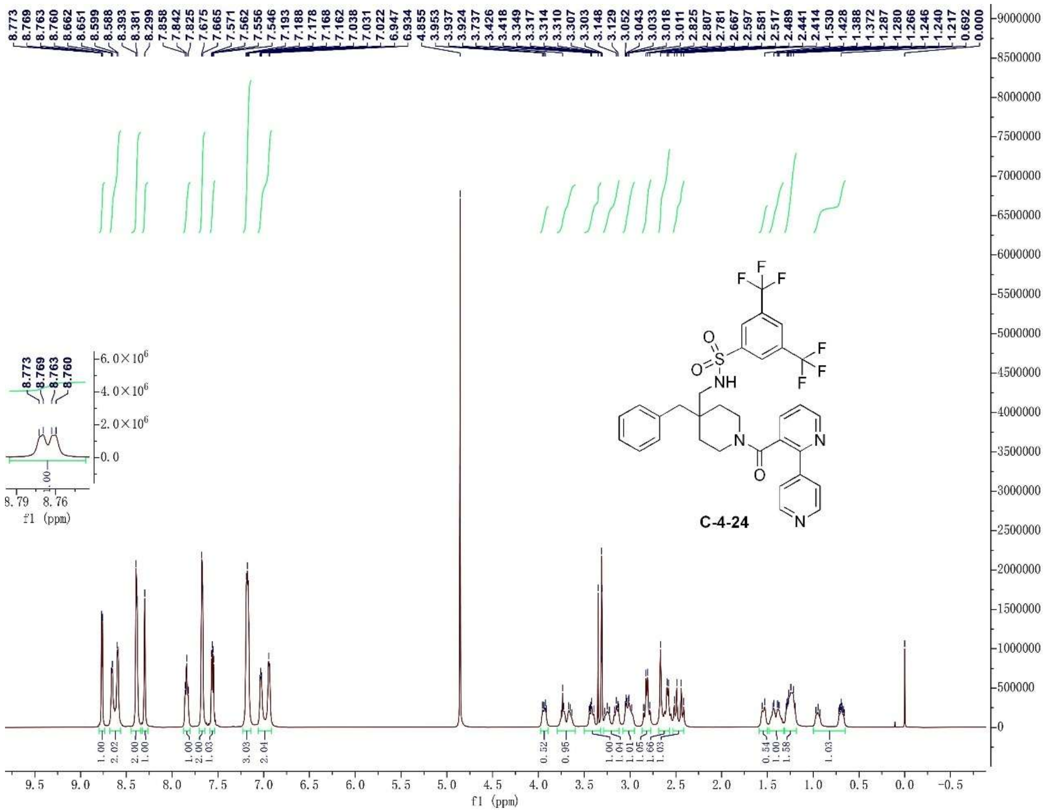

Figure S500: ^{1}H NMR spectrum of **C-4-24**

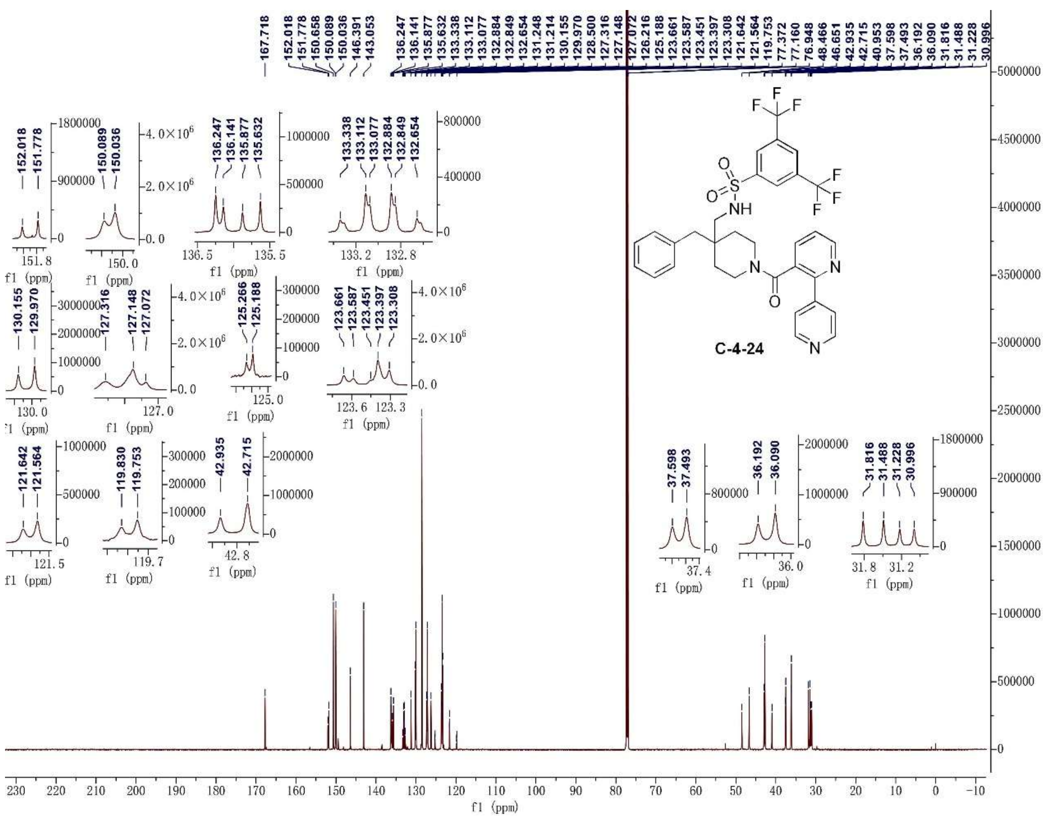

Figure S501: ^{13}C NMR spectrum of **C-4-24**

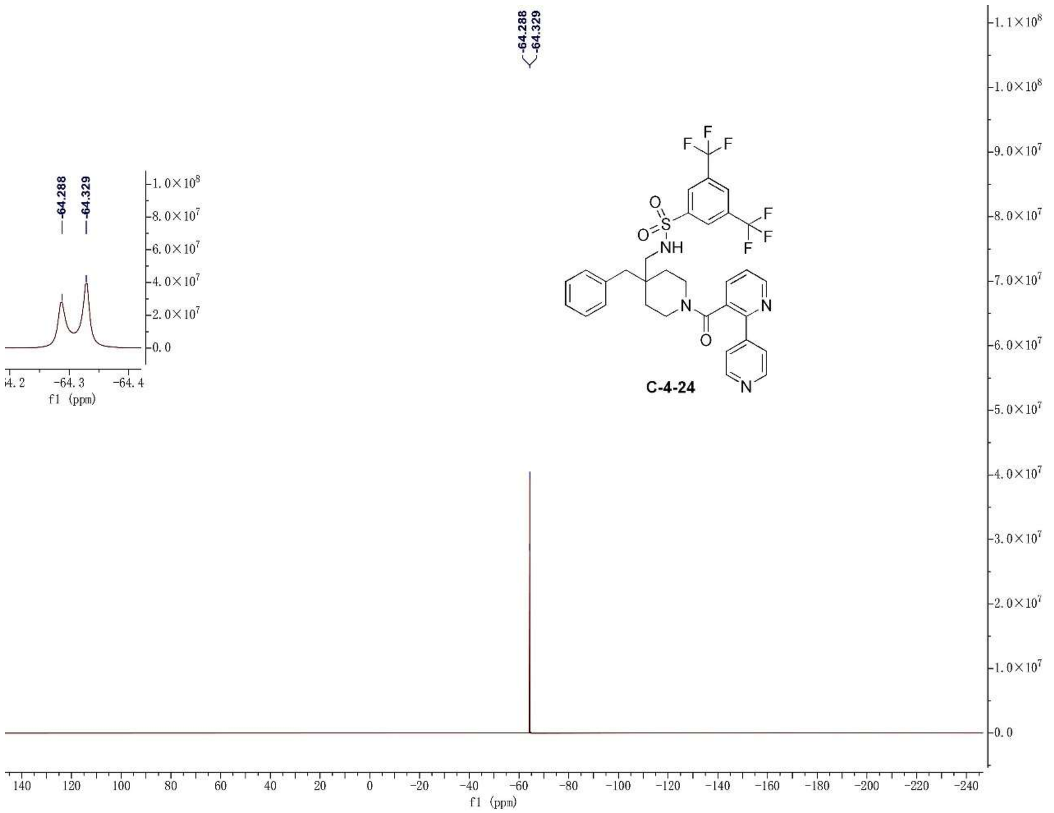

Figure S502: ^{19}F NMR spectrum of **C-4-24**

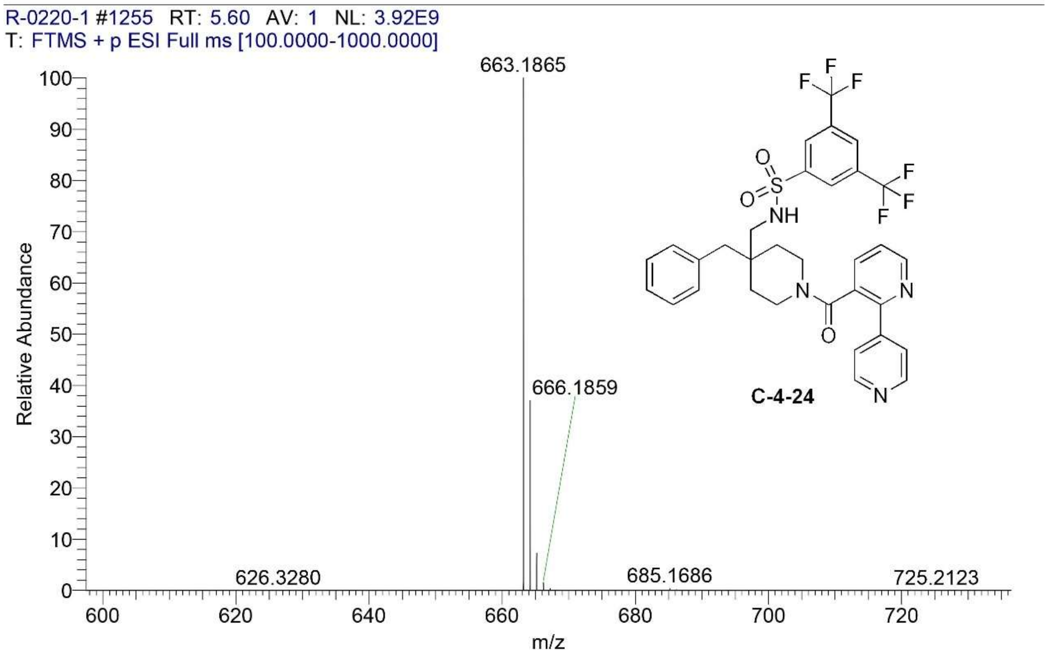

Figure S503: HR-MS (ESI/ion trap) spectrum of **C-4-24**

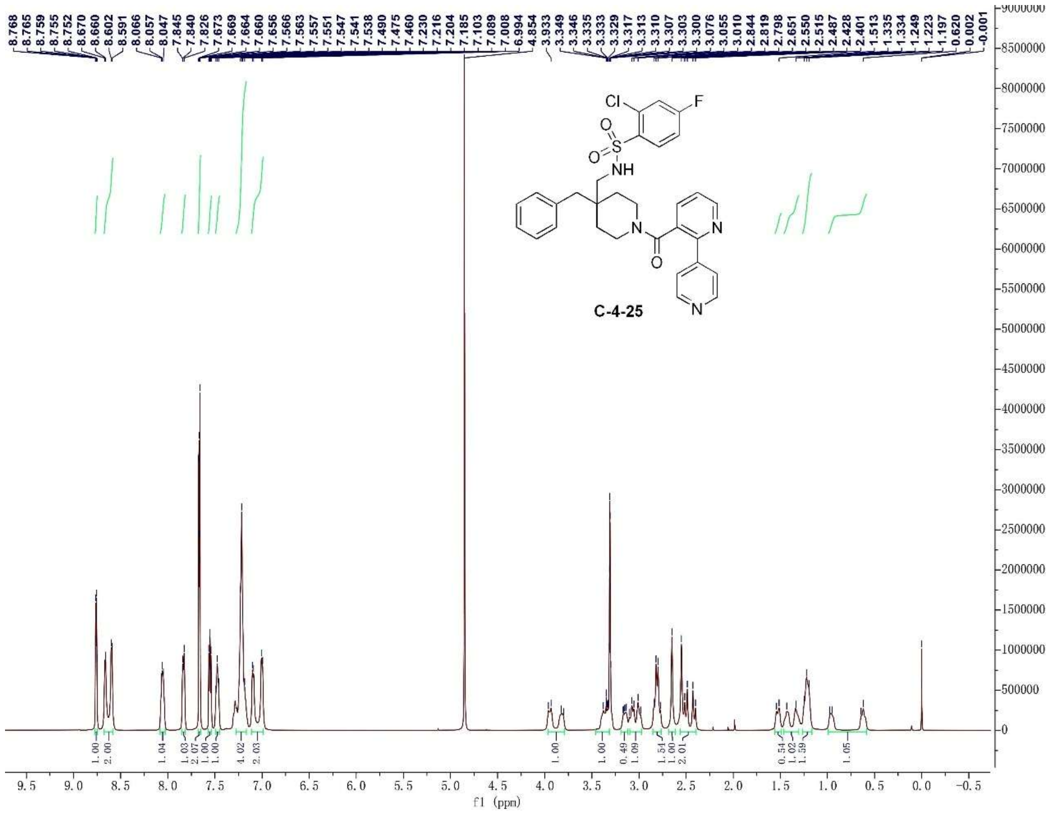

Figure S504: ^{1}H NMR spectrum of **C-4-25**

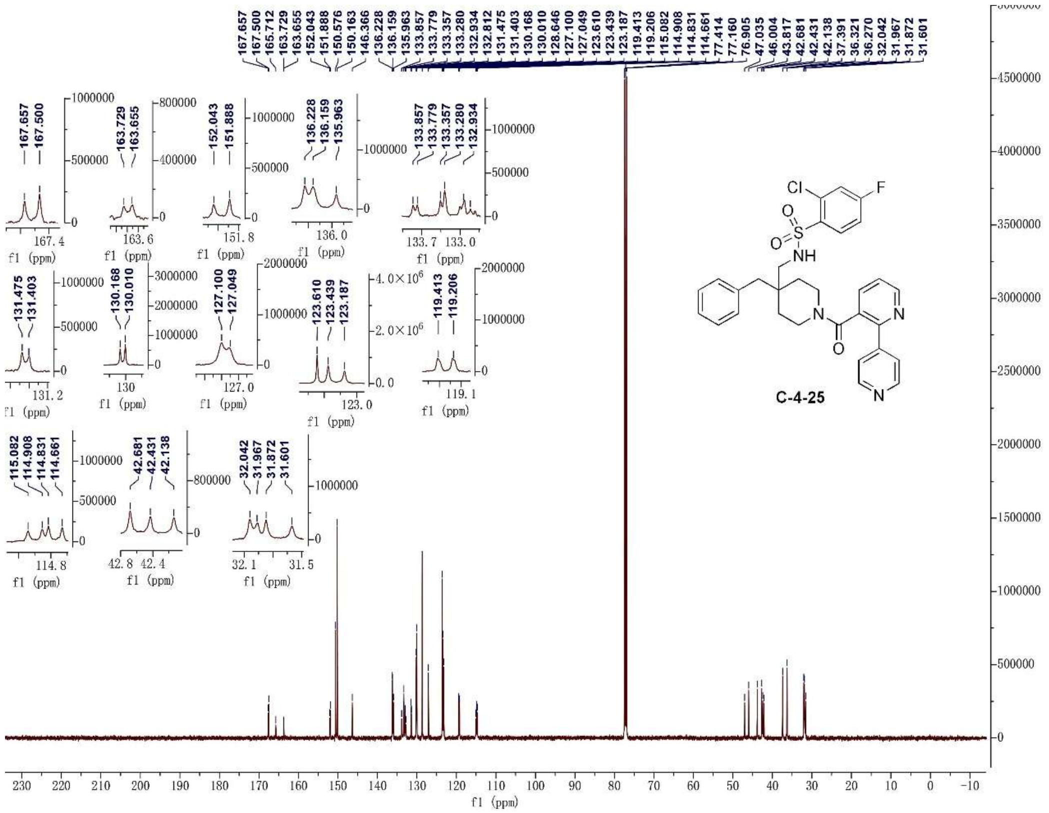

Figure S505: ^{13}C NMR spectrum of **C-4-25**

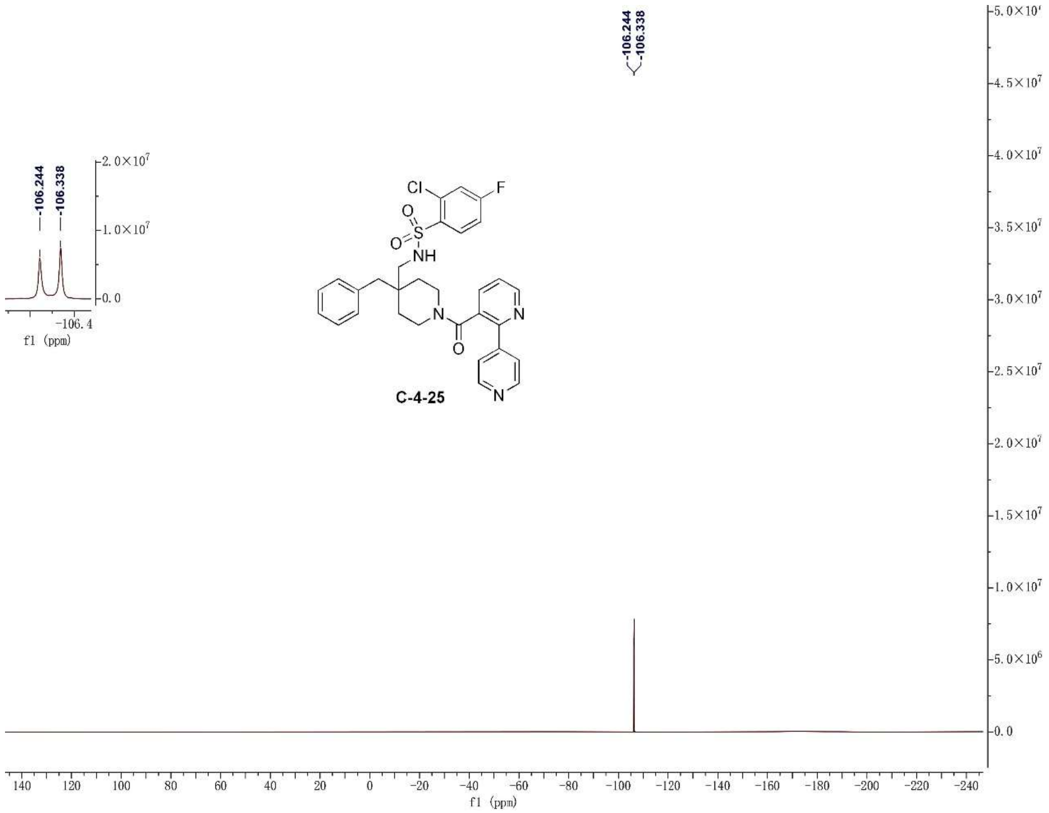

Figure S506: ^{19}F NMR spectrum of **C-4-25**

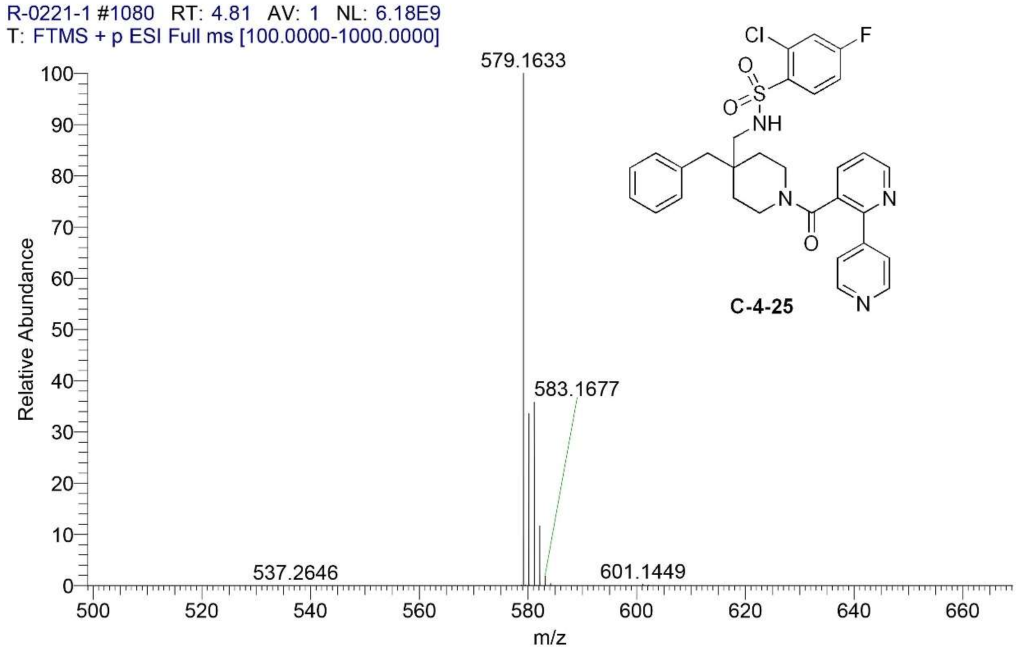

Figure S507: HR-MS (ESI/ion trap) spectrum of **C-4-25**

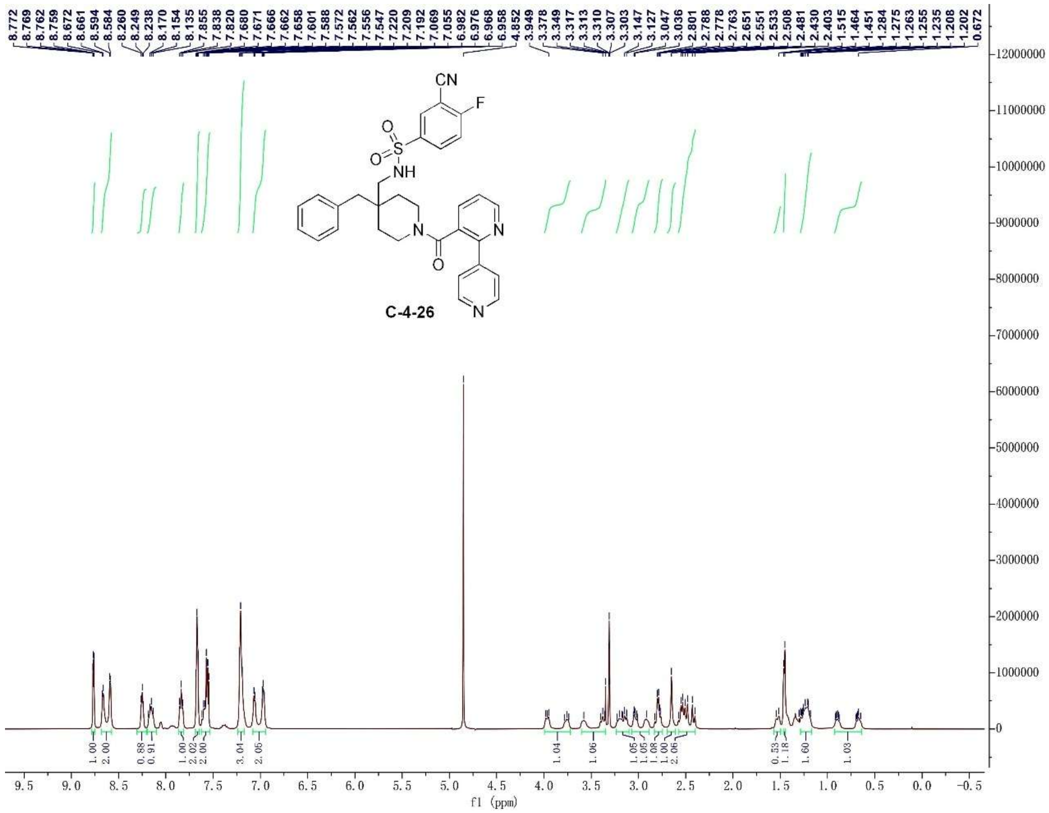

Figure S508: ^{1}H NMR spectrum of **C-4-26**

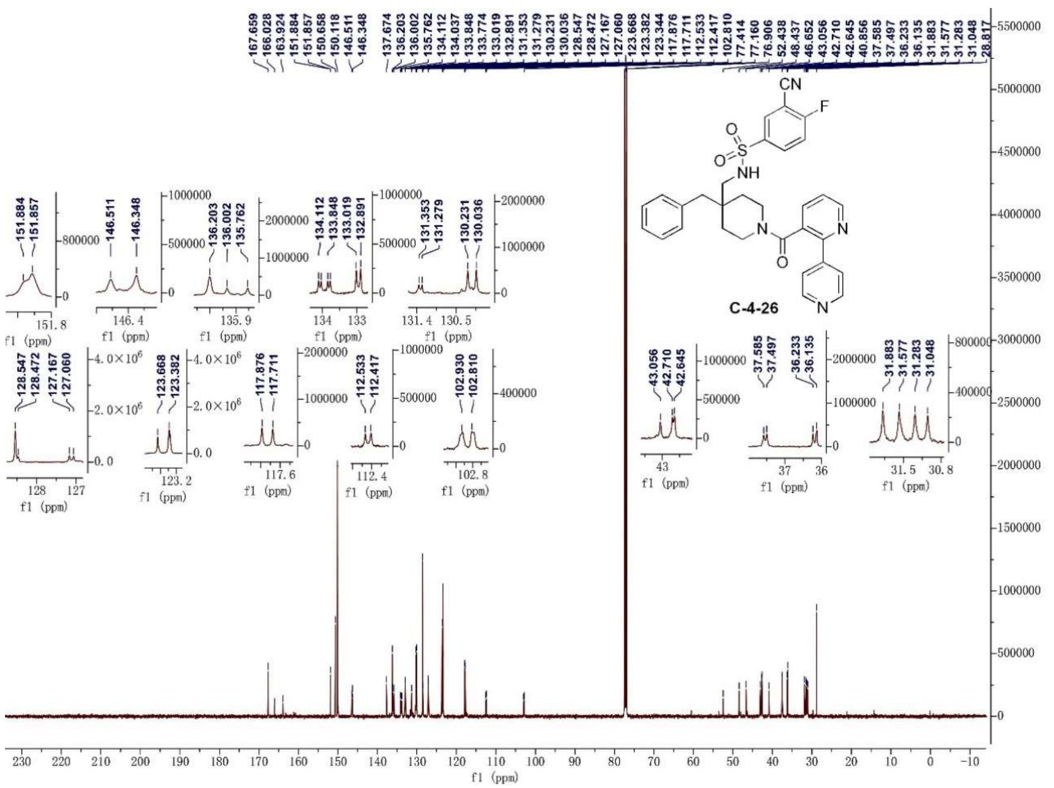

Figure S509: ^{13}C NMR spectrum of **C-4-26**

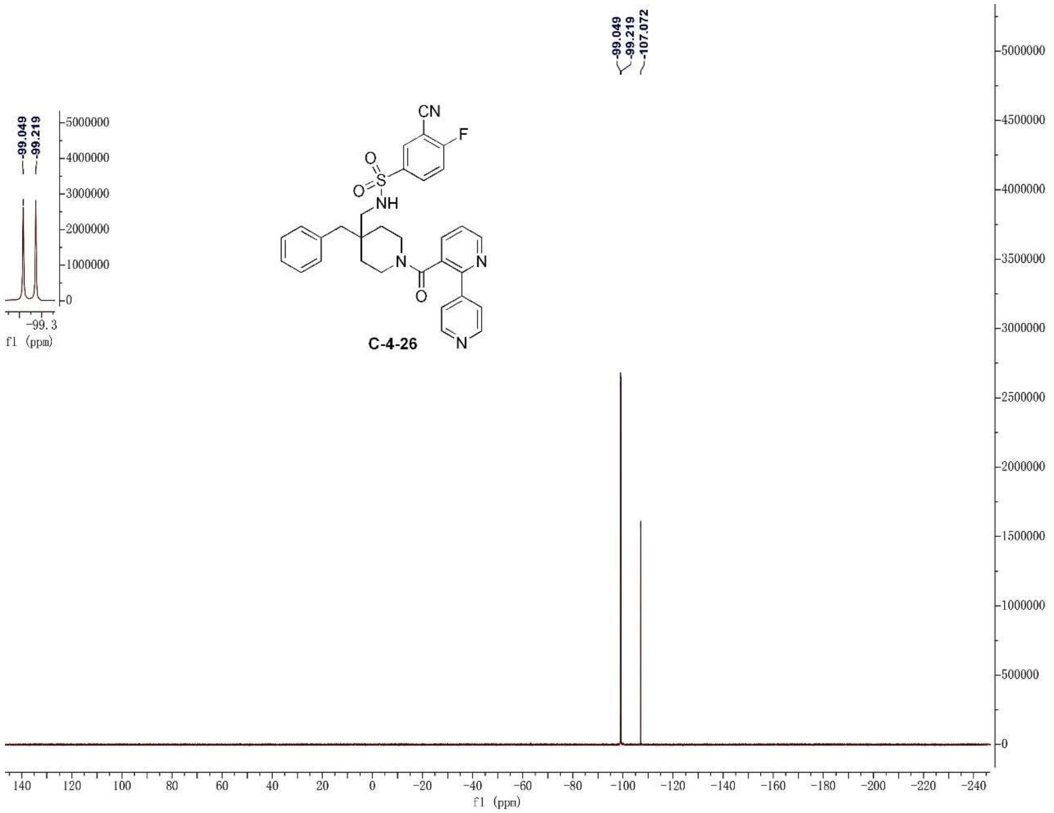

Figure S510: ^{19}F NMR spectrum of **C-4-26**

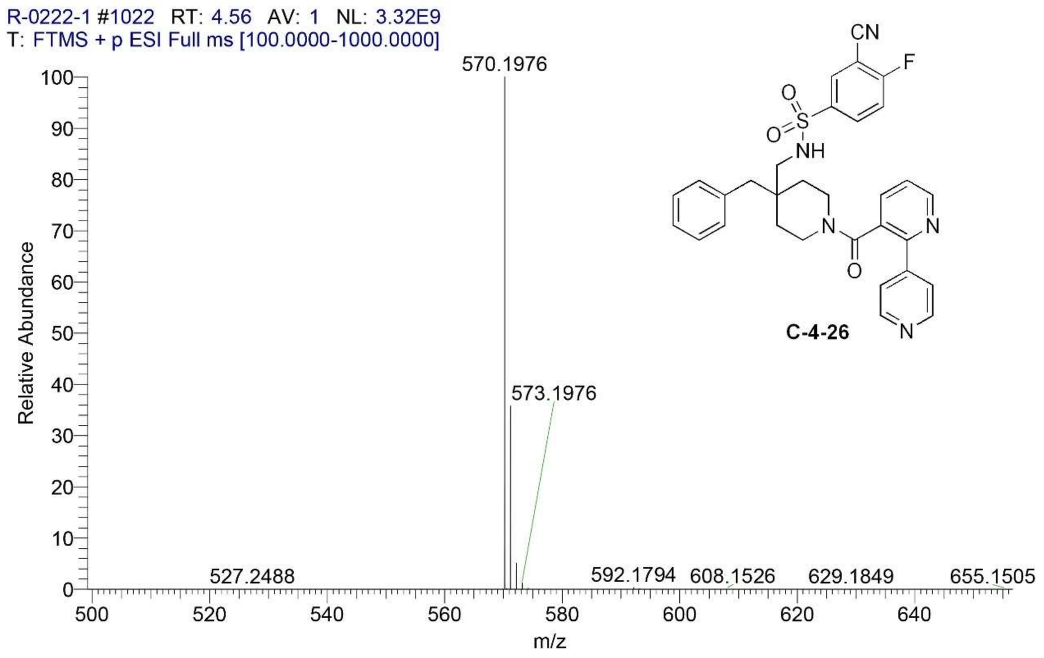

Figure S511: HR-MS (ESI/ion trap) spectrum of **C-4-26**

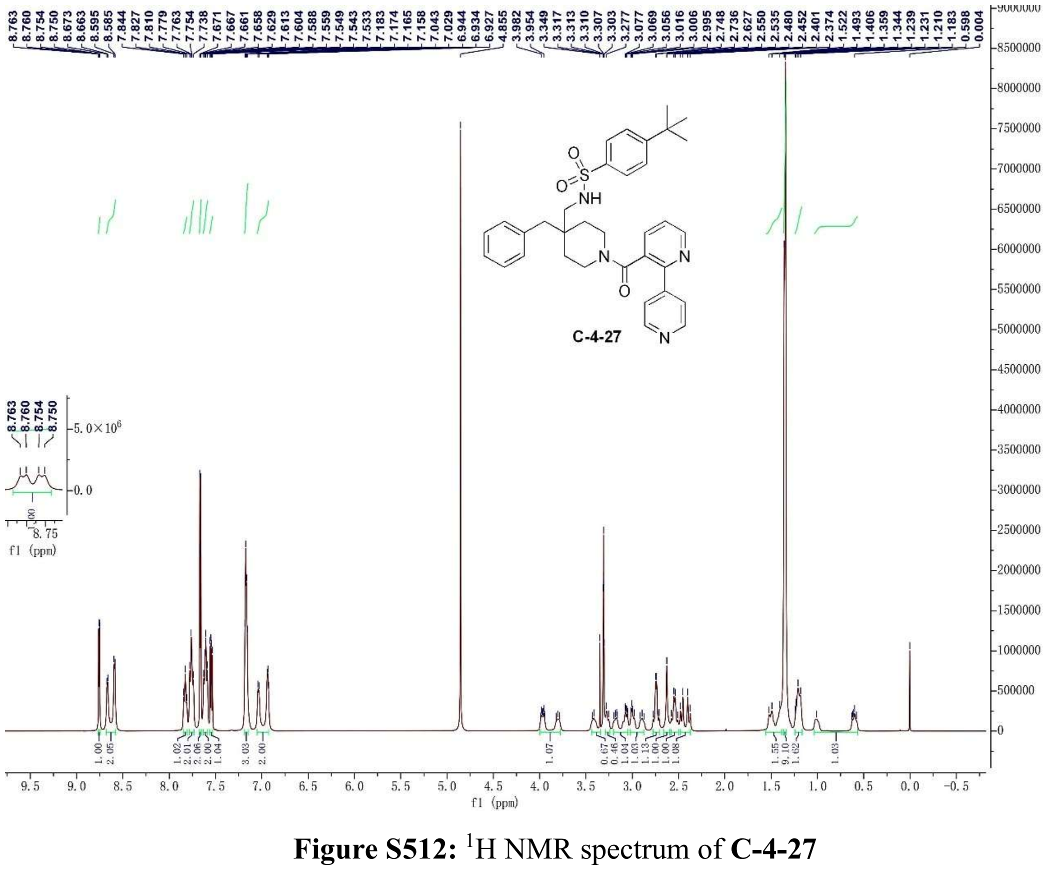

Figure S512: ^{1}H NMR spectrum of **C-4-27**

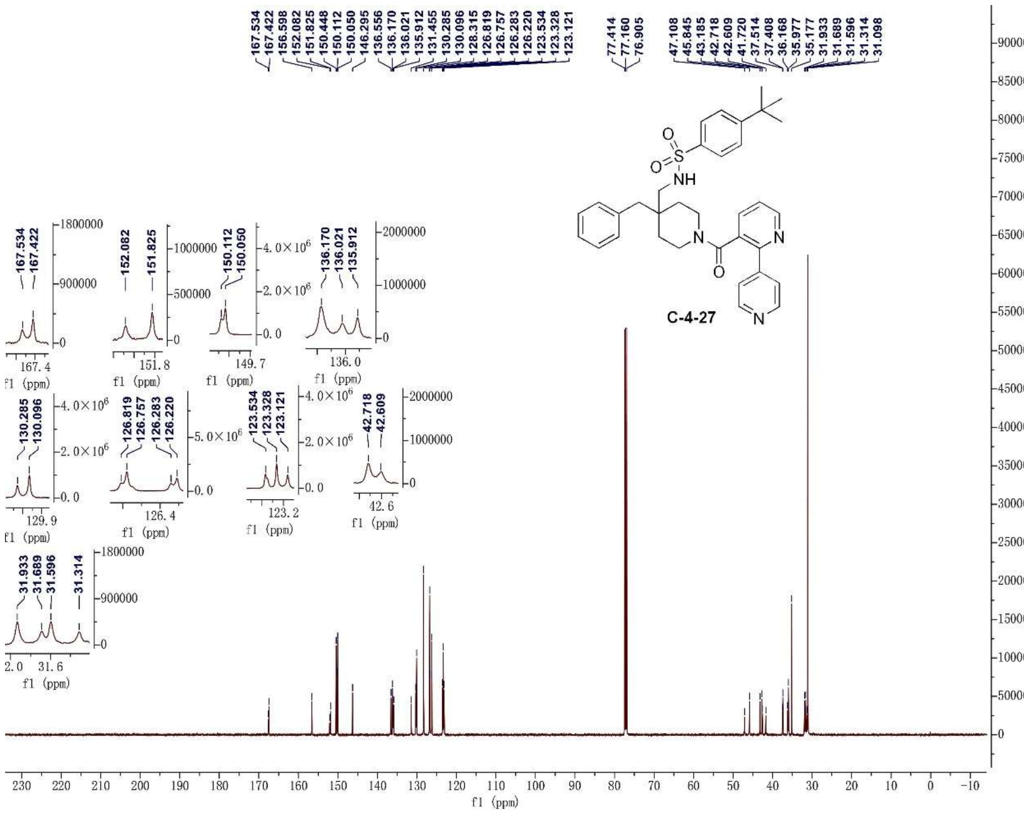

Figure S513: ^{13}C NMR spectrum of **C-4-27**

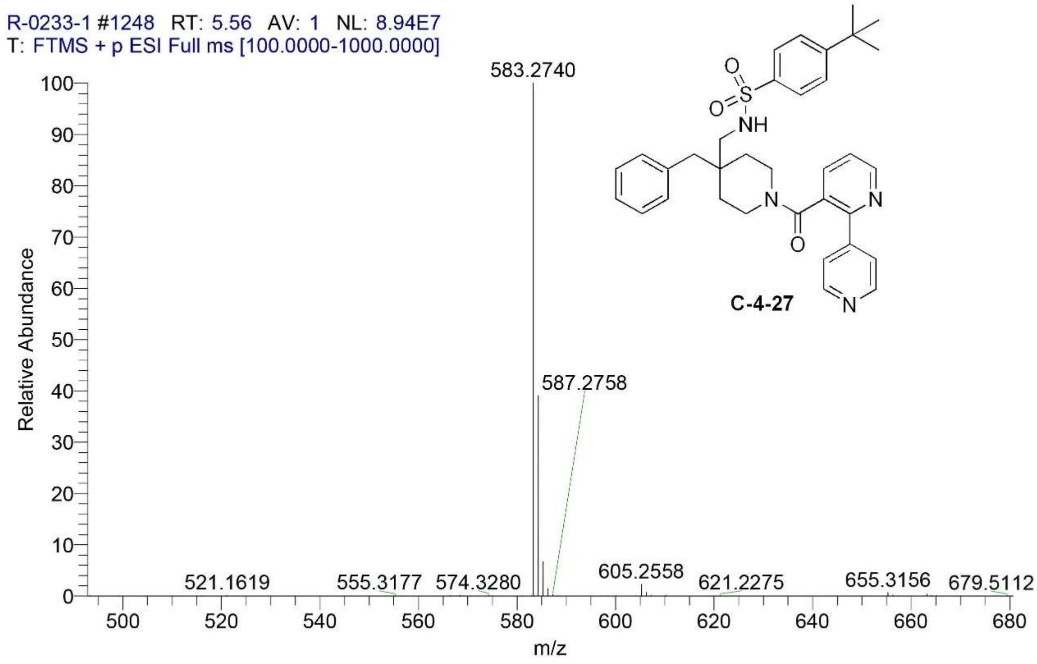

Figure S514: HR-MS (ESI/ion trap) spectrum of **C-4-27**

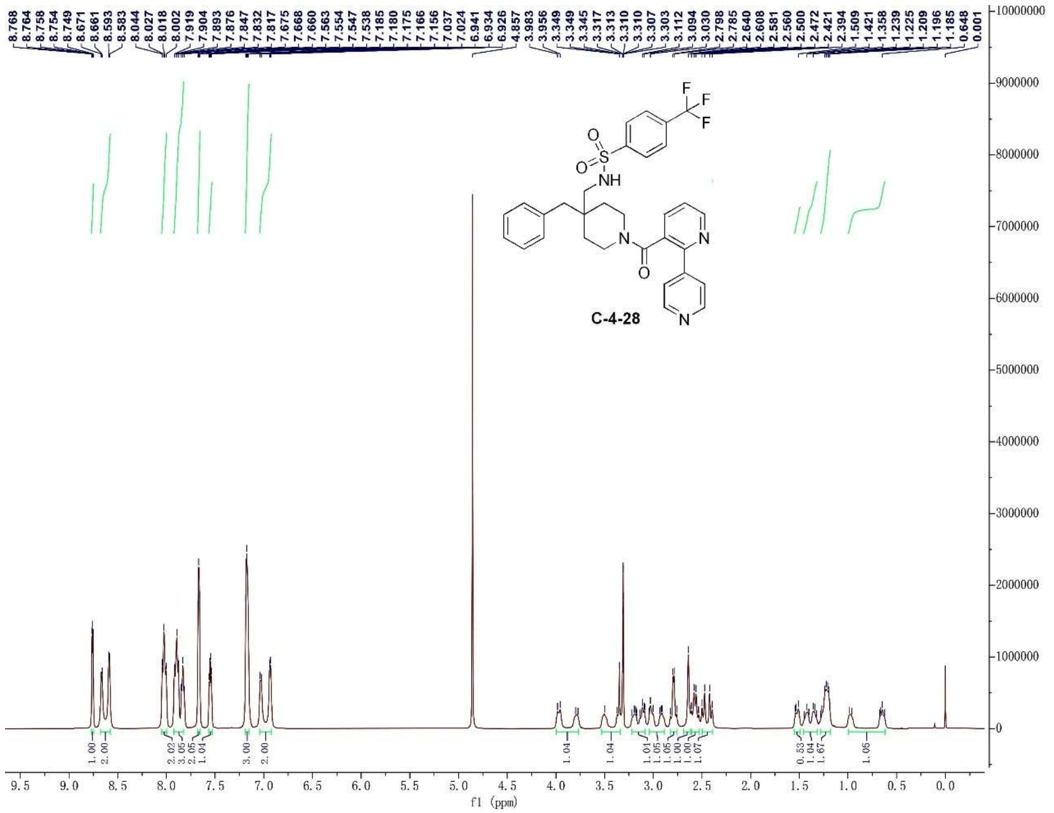

Figure S515: ^{1}H NMR spectrum of **C-4-28**

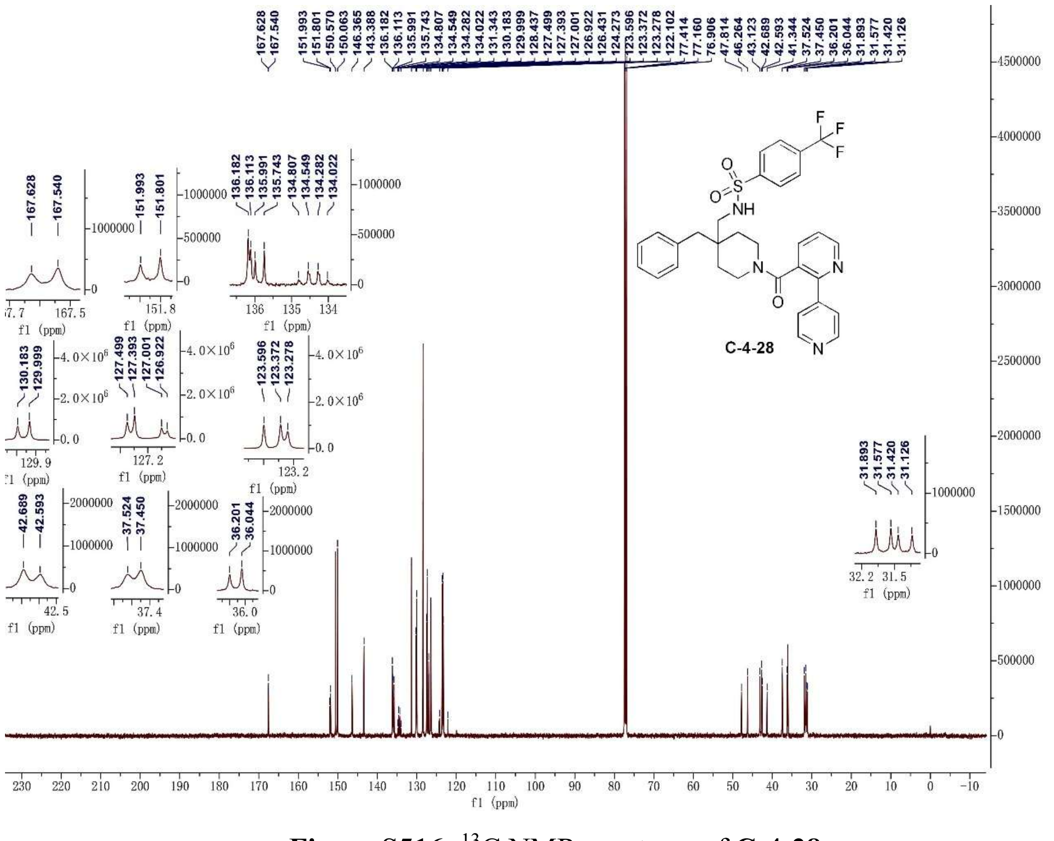

Figure S516: ^{13}C NMR spectrum of **C-4-28**

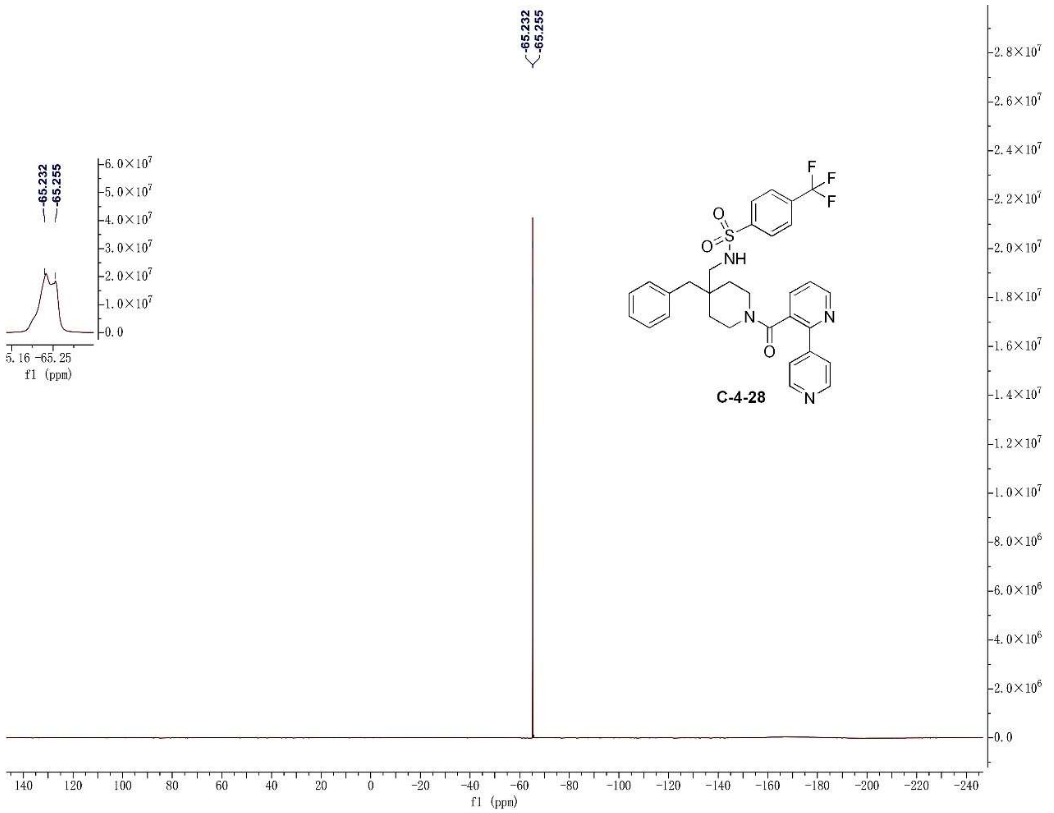

Figure S517: ^{19}F NMR spectrum of **C-4-28**

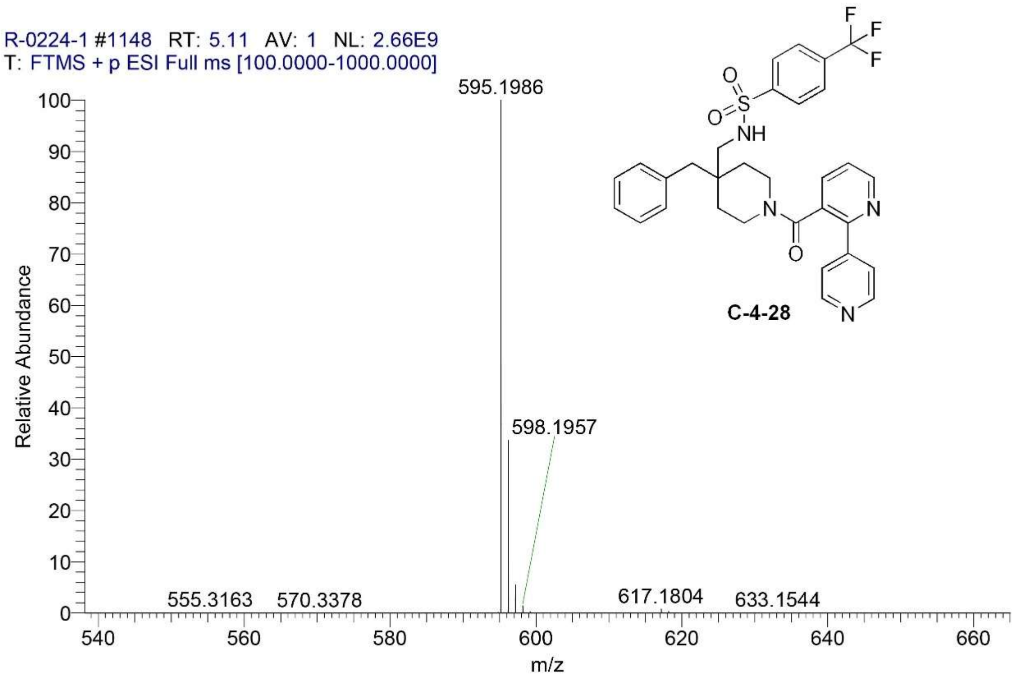

Figure S518: HR-MS (ESI/ion trap) spectrum of **C-4-28**

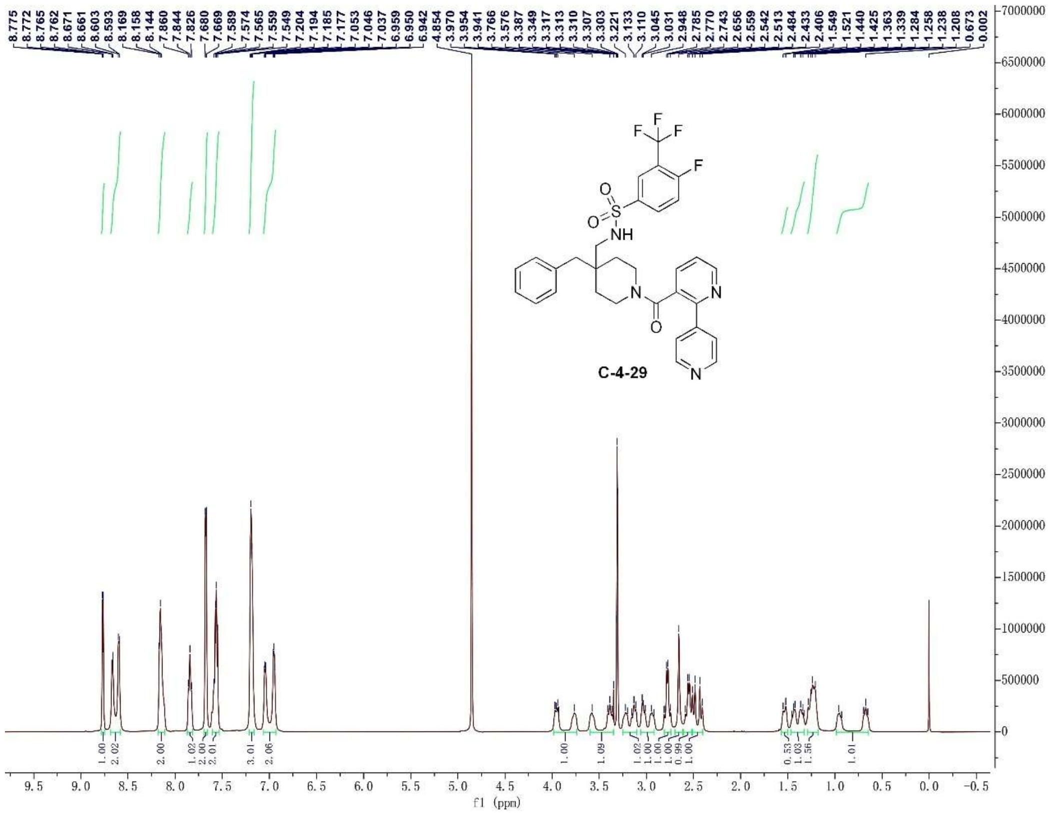

Figure S519: ^{1}H NMR spectrum of **C-4-29**

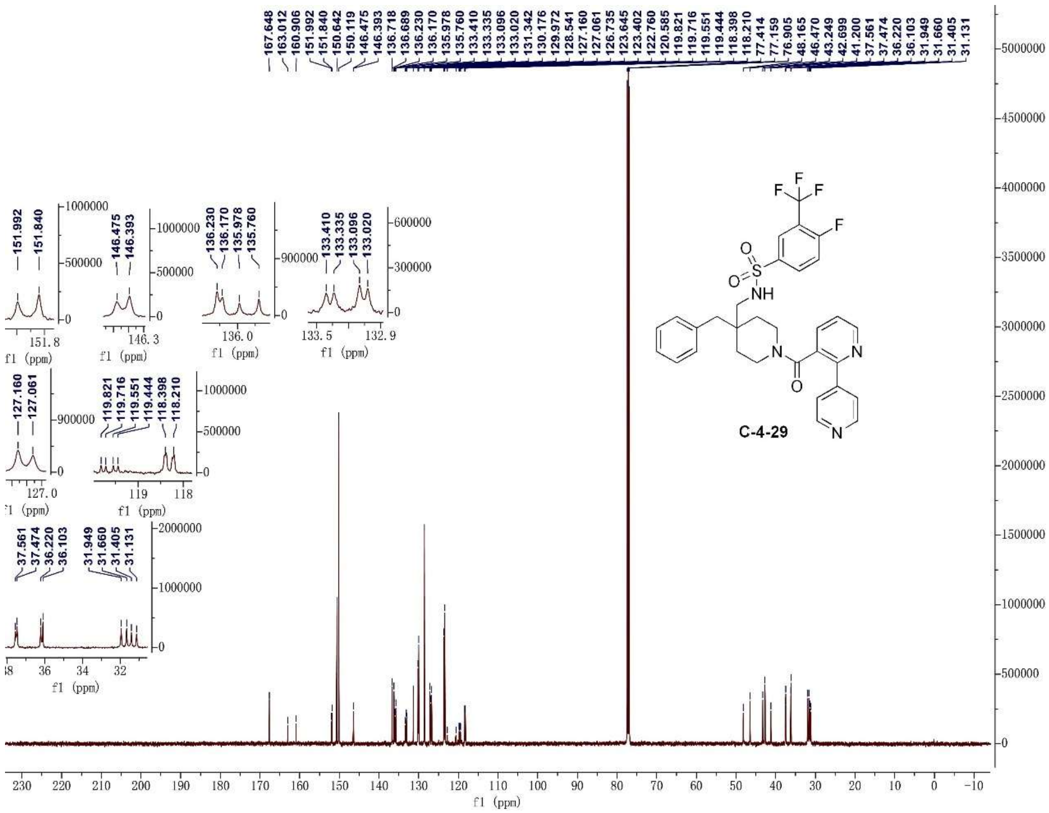

Figure S520: ^{13}C NMR spectrum of **C-4-29**

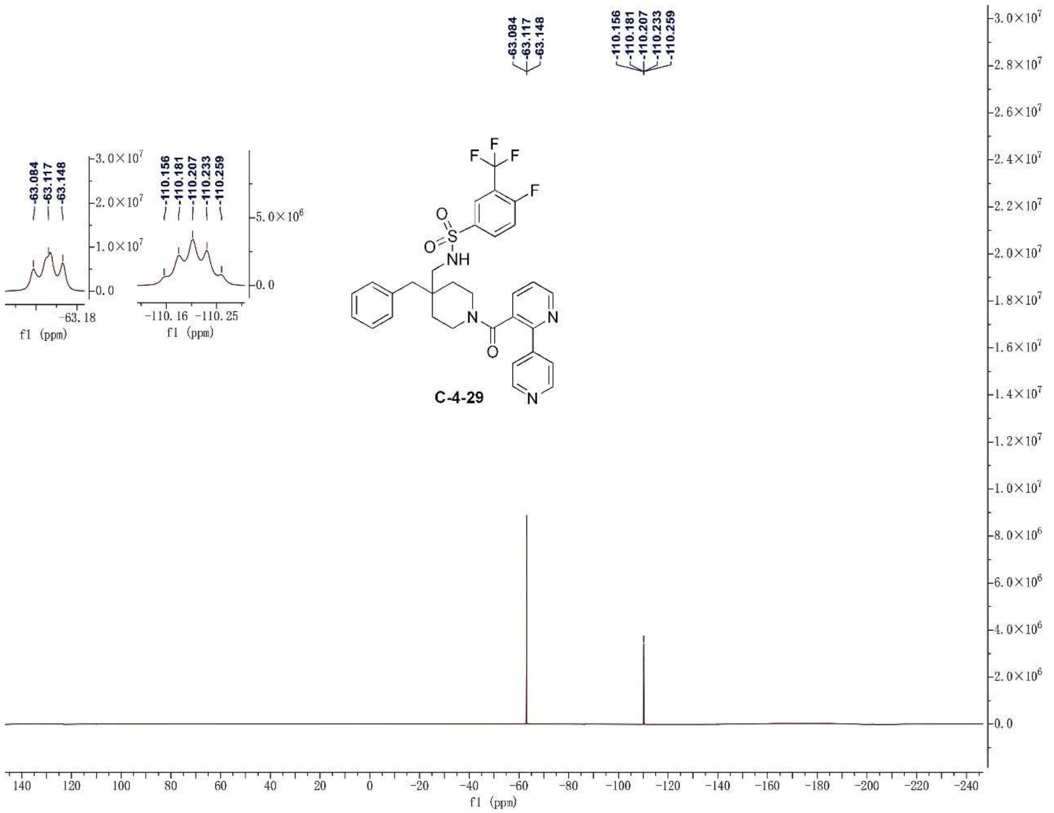

Figure S521: ^{19}F NMR spectrum of **C-4-29**

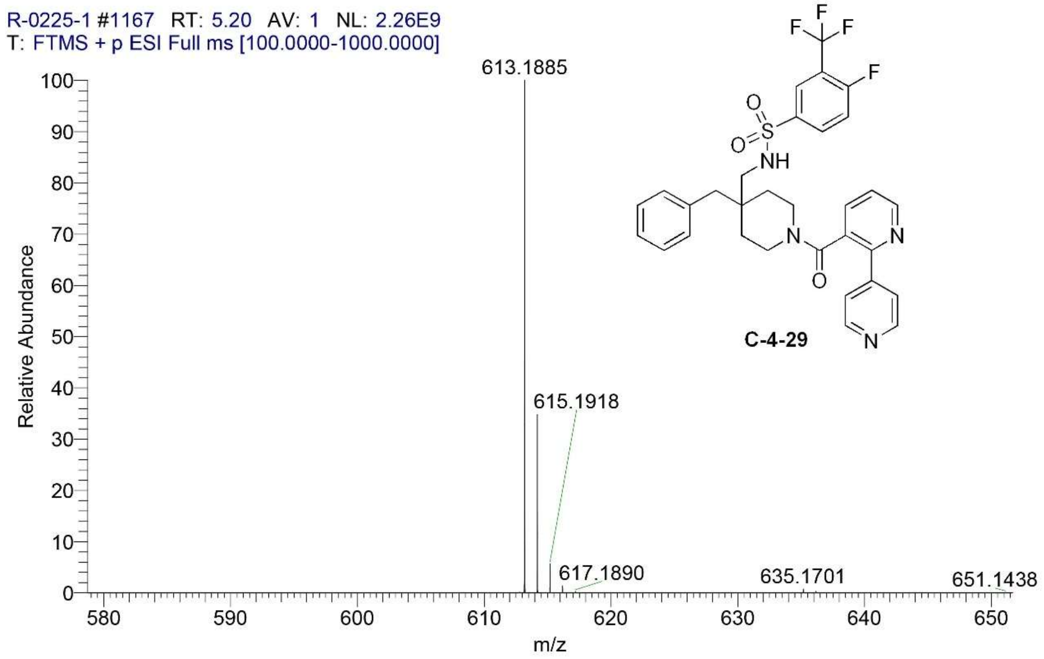

Figure S522: HR-MS (ESI/ion trap) spectrum of **C-4-29**

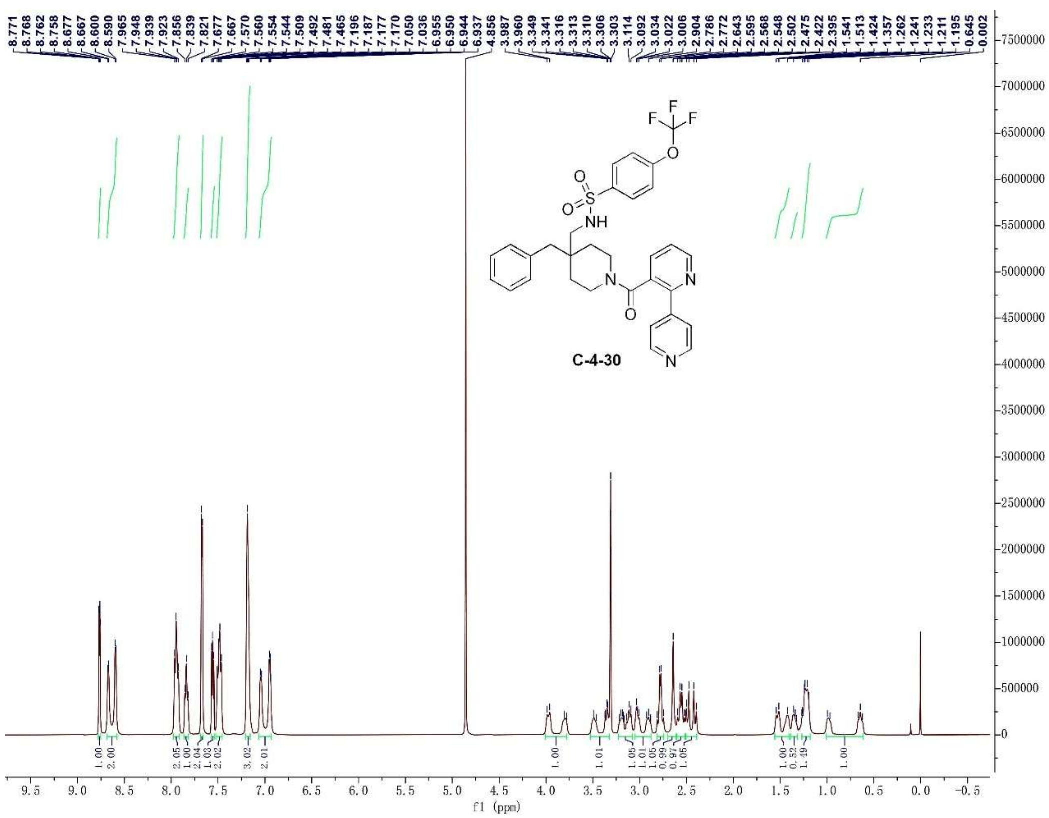

Figure S523: ^{1}H NMR spectrum of **C-4-30**

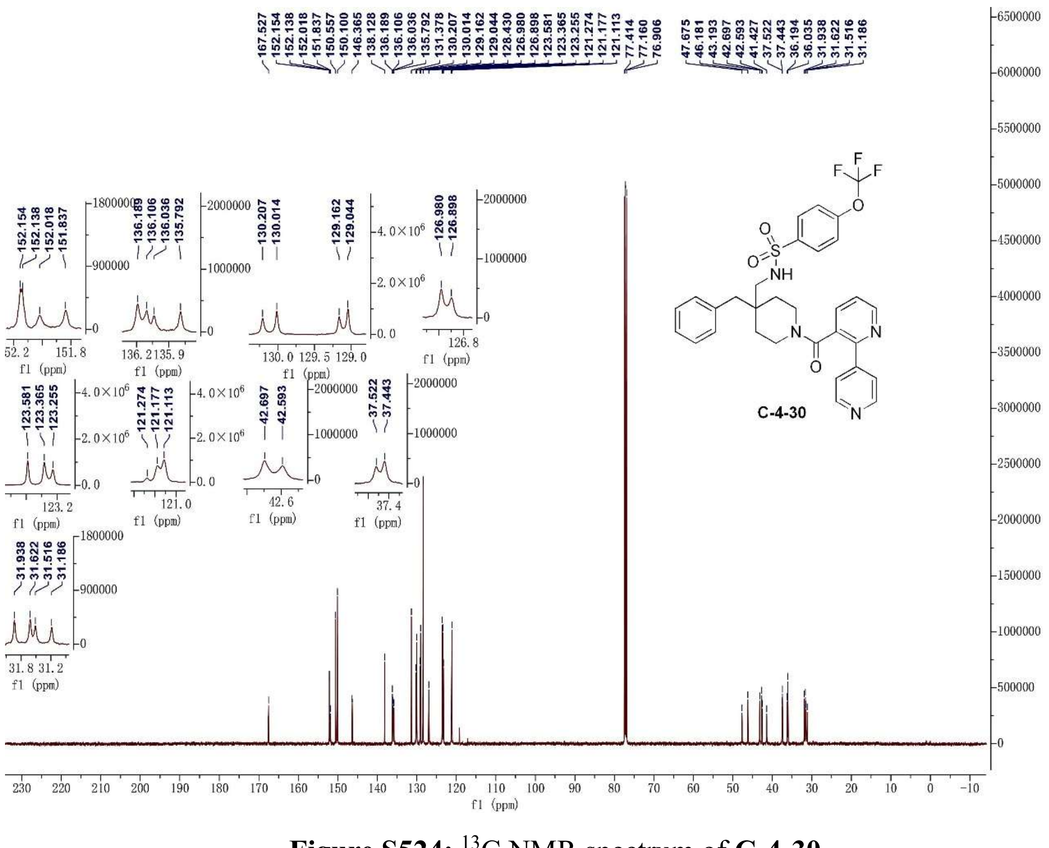

Figure S524: ^{13}C NMR spectrum of **C-4-30**

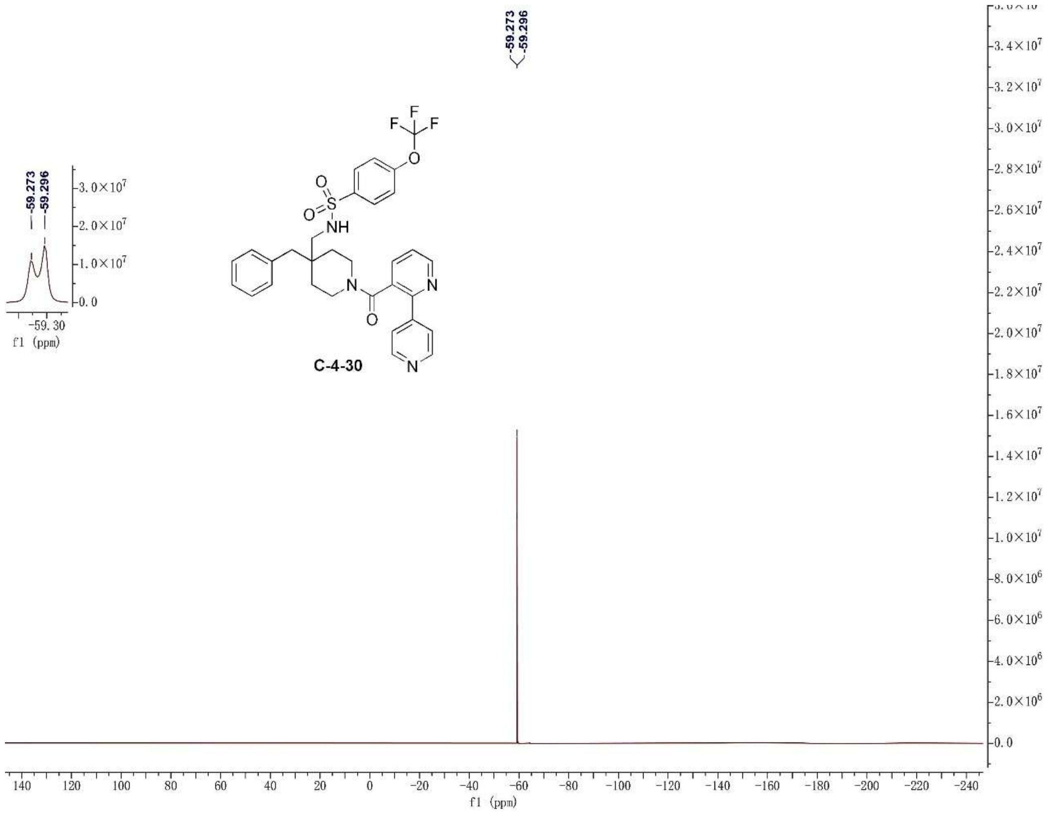

Figure S525: ^{19}F NMR spectrum of **C-4-30**

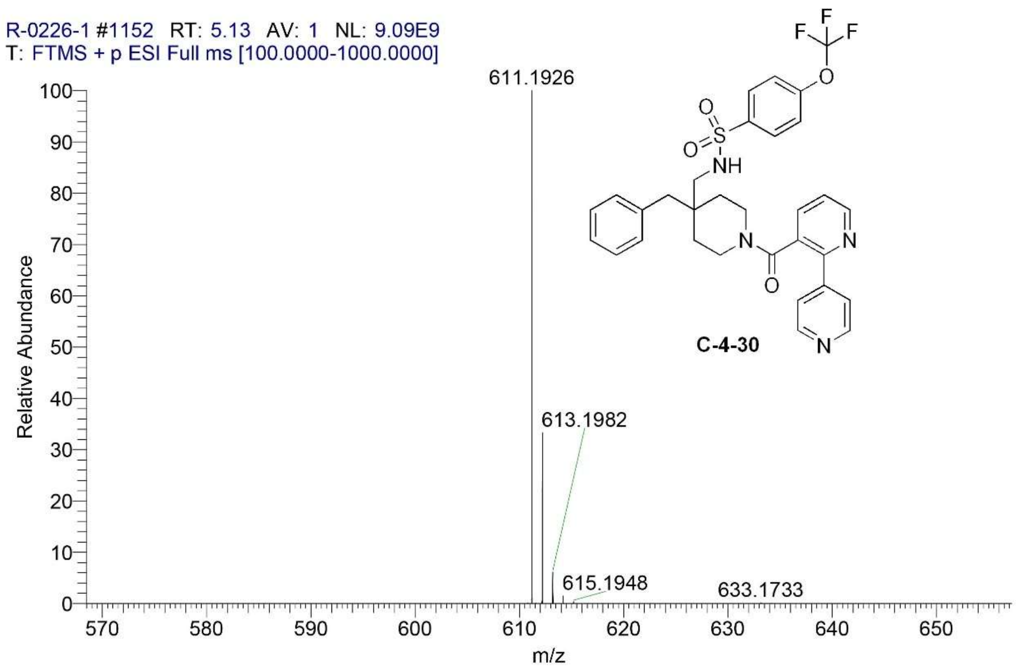

Figure S526: HR-MS (ESI/ion trap) spectrum of **C-4-30**

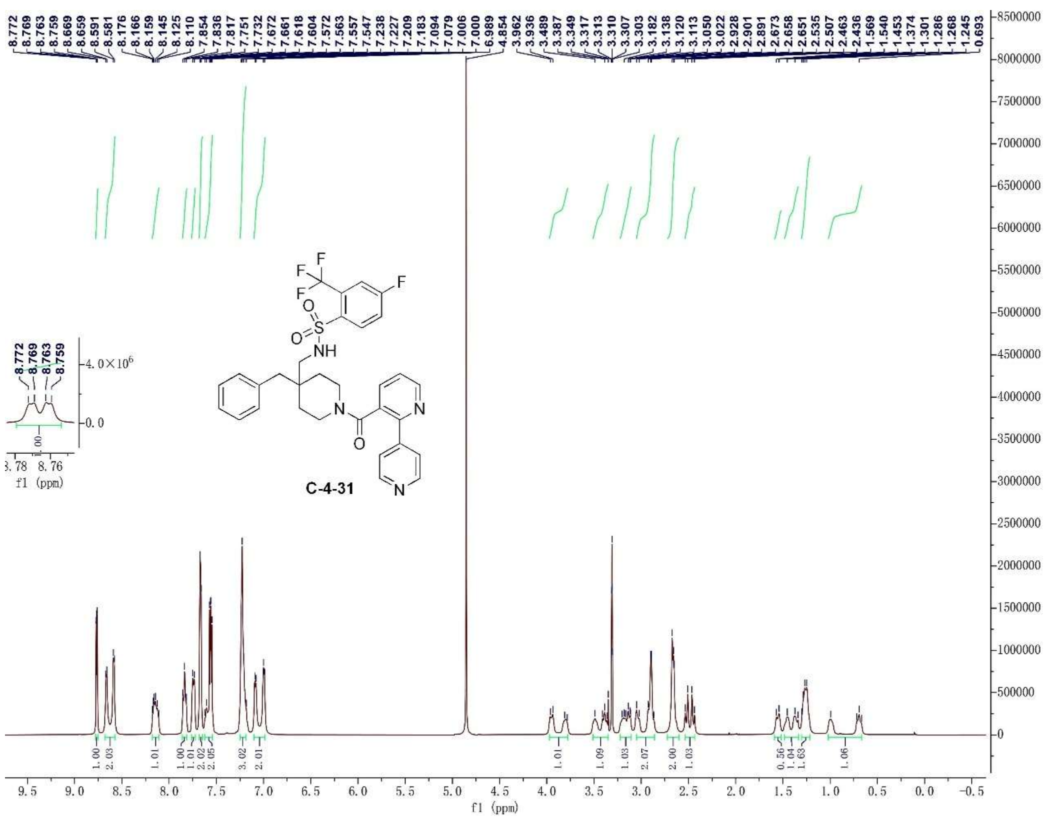

Figure S527: ^{1}H NMR spectrum of **C-4-31**

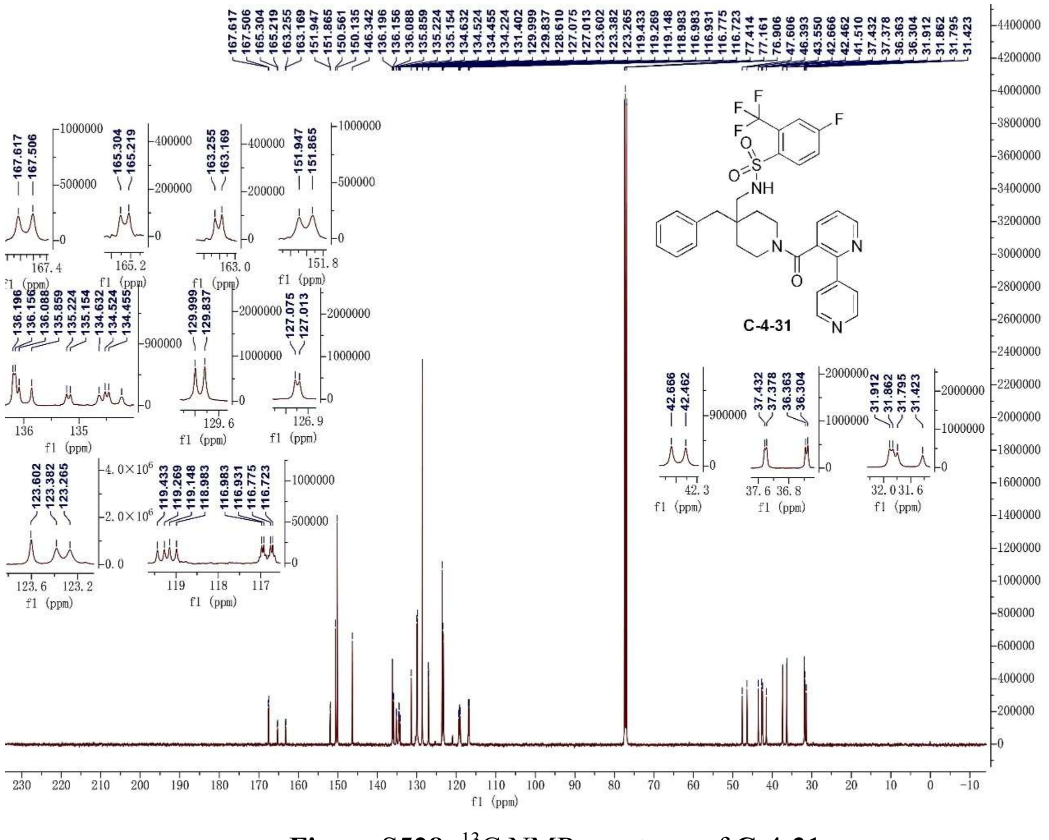

Figure S528: ^{13}C NMR spectrum of **C-4-31**

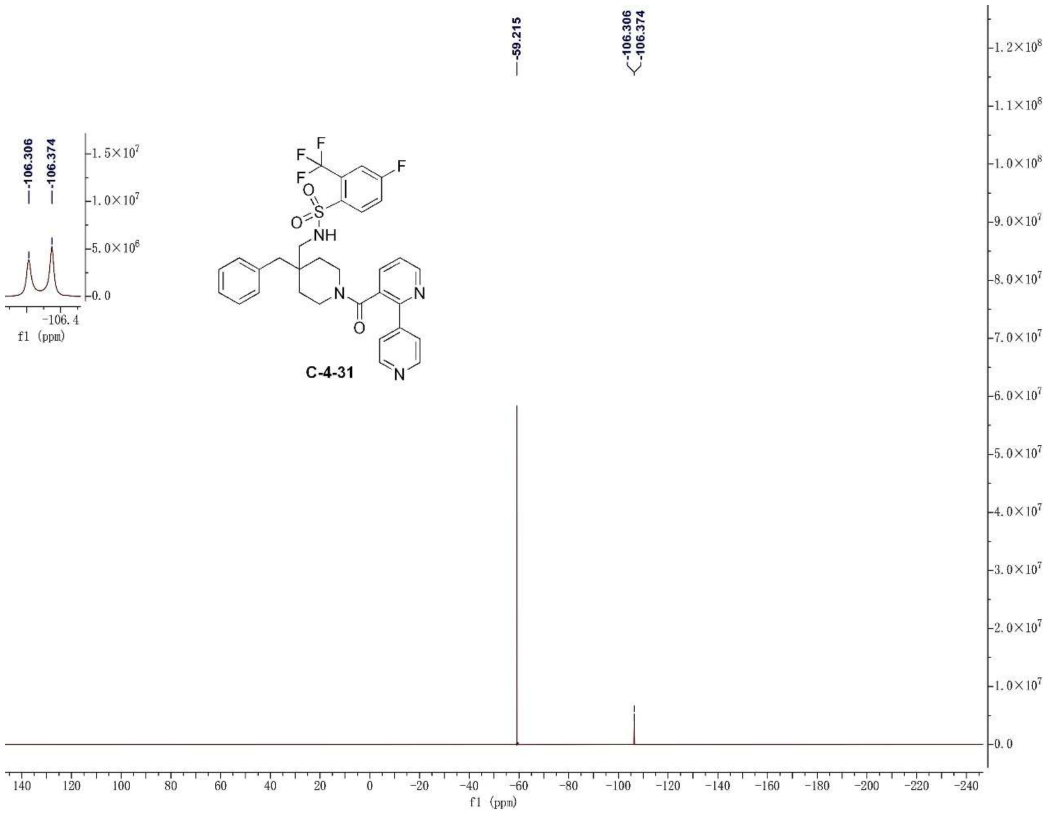

Figure S529: ^{19}F NMR spectrum of **C-4-31**

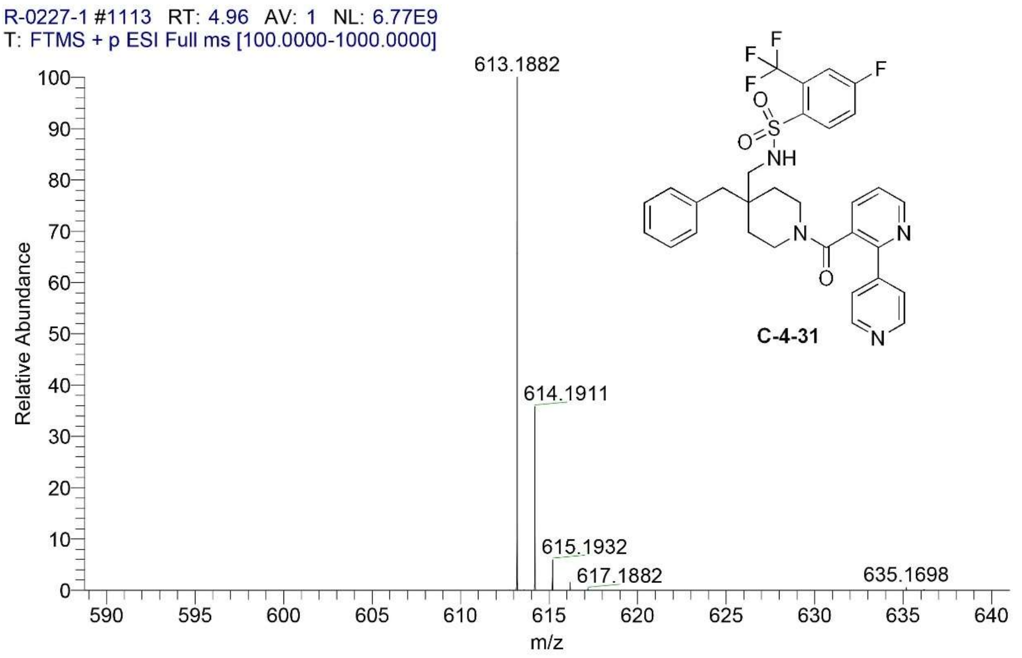

Figure S530: HR-MS (ESI/ion trap) spectrum of **C-4-31**

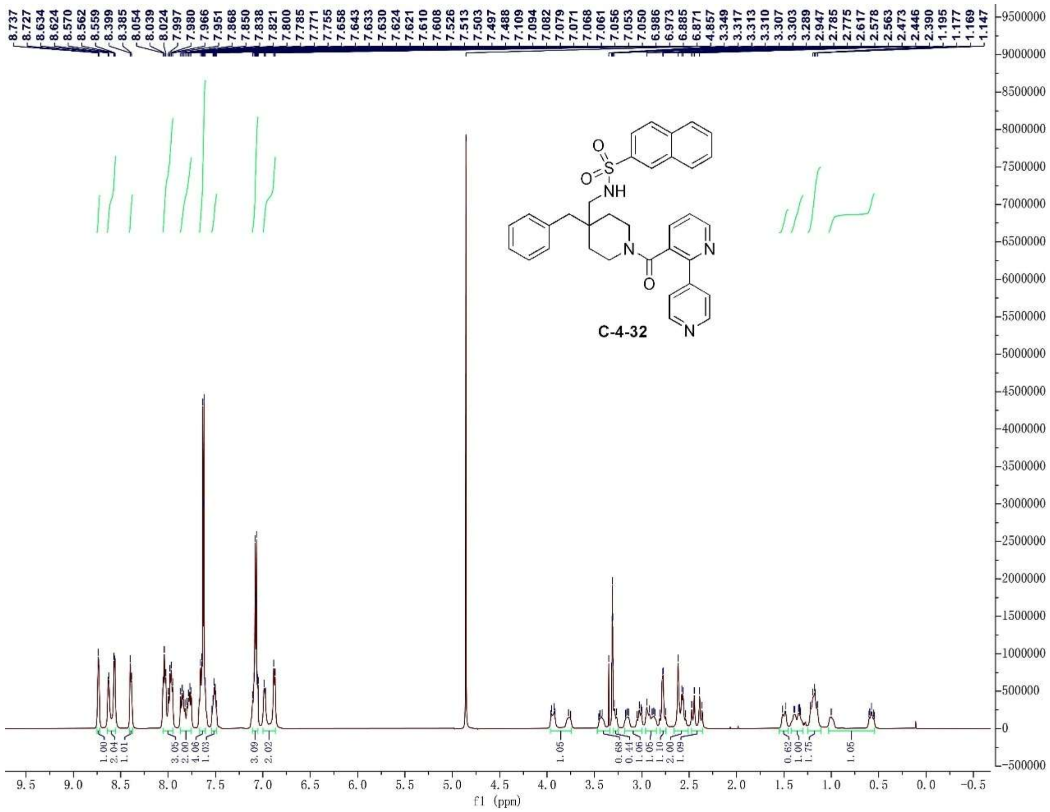

Figure S531: ^{1}H NMR spectrum of **C-4-32**

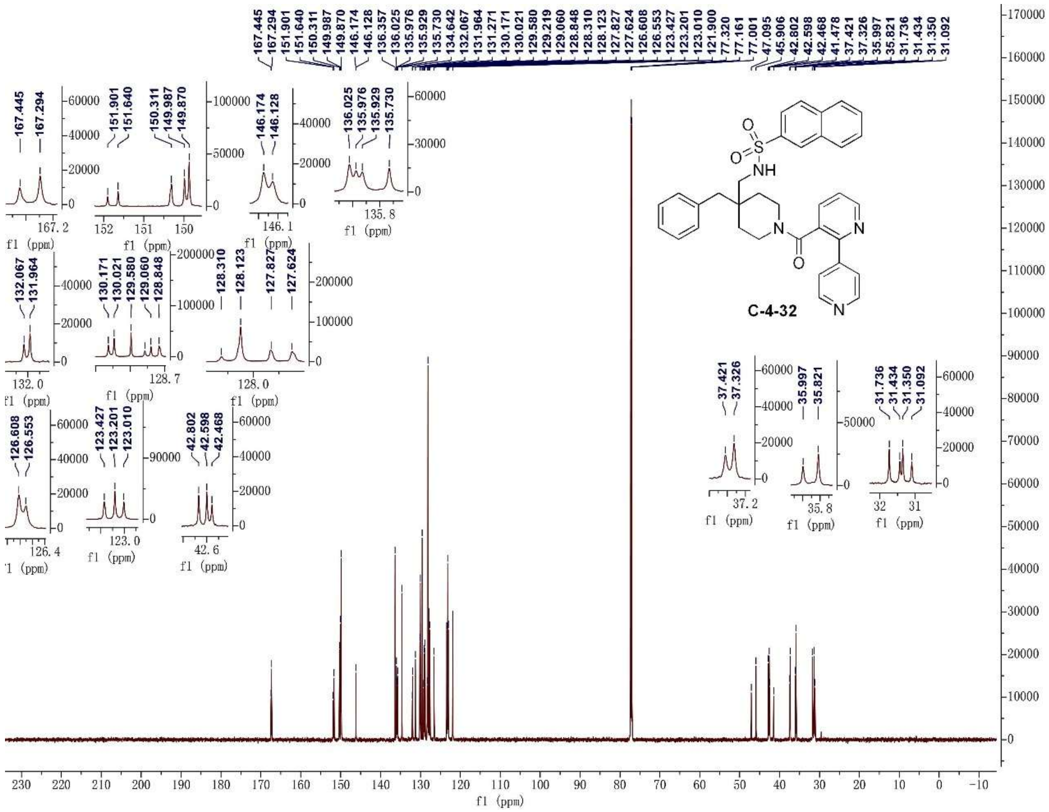

Figure S532: ^{13}C NMR spectrum of **C-4-32**

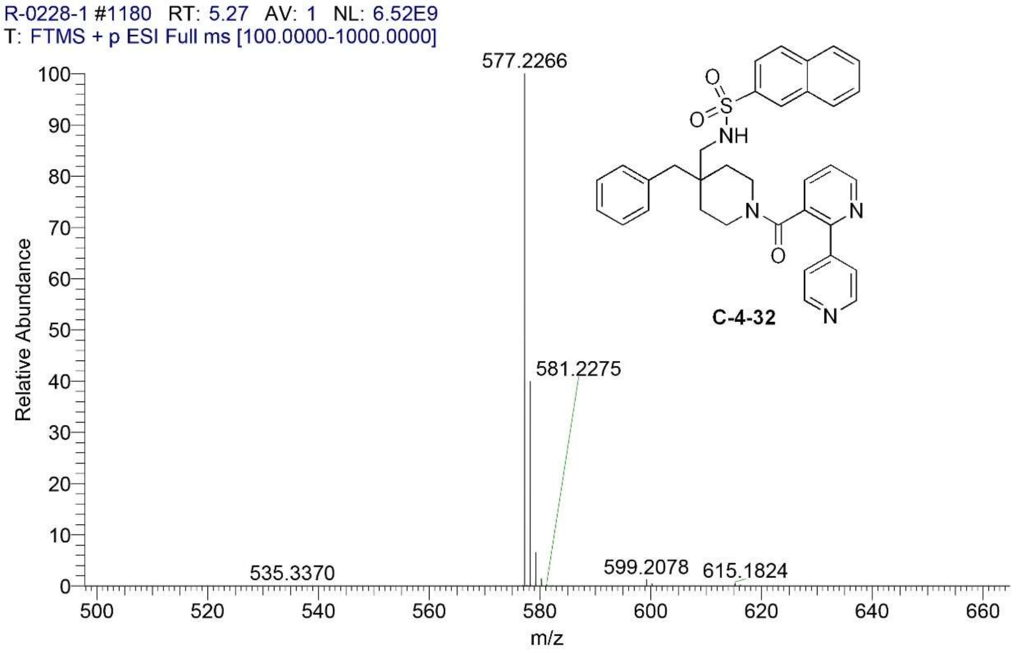

Figure S533: HR-MS (ESI/ion trap) spectrum of **C-4-32**

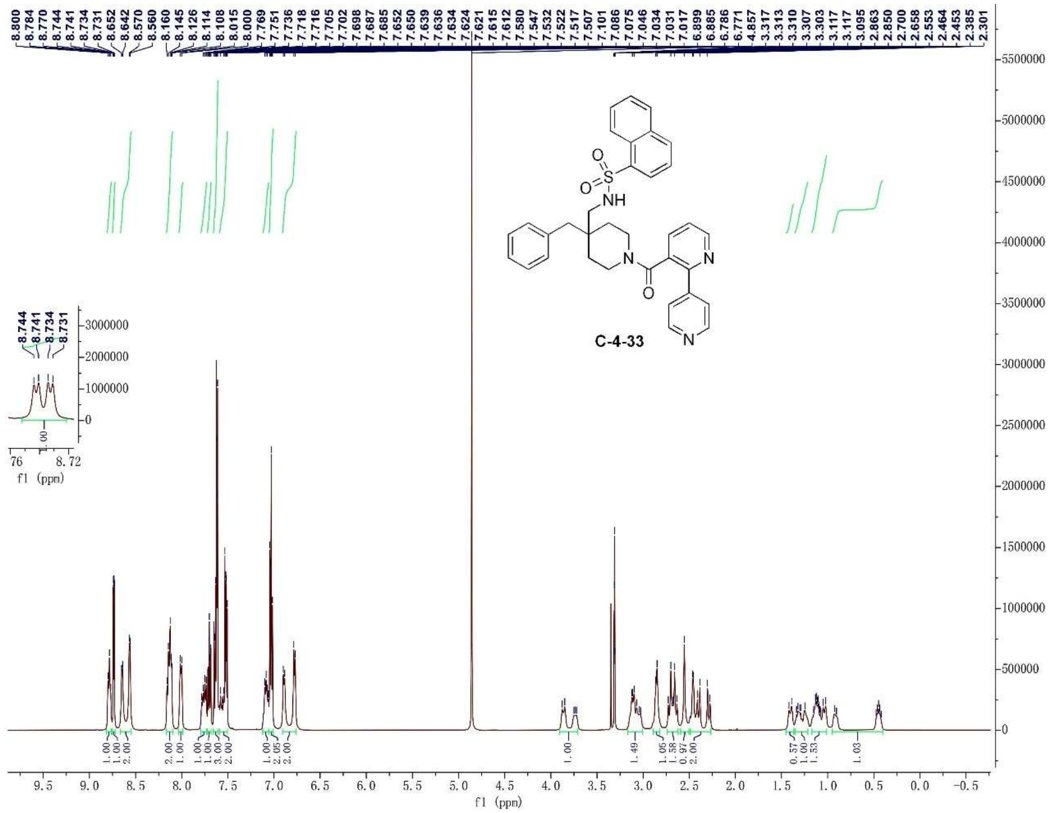

Figure S534: ^{1}H NMR spectrum of **C-4-33**

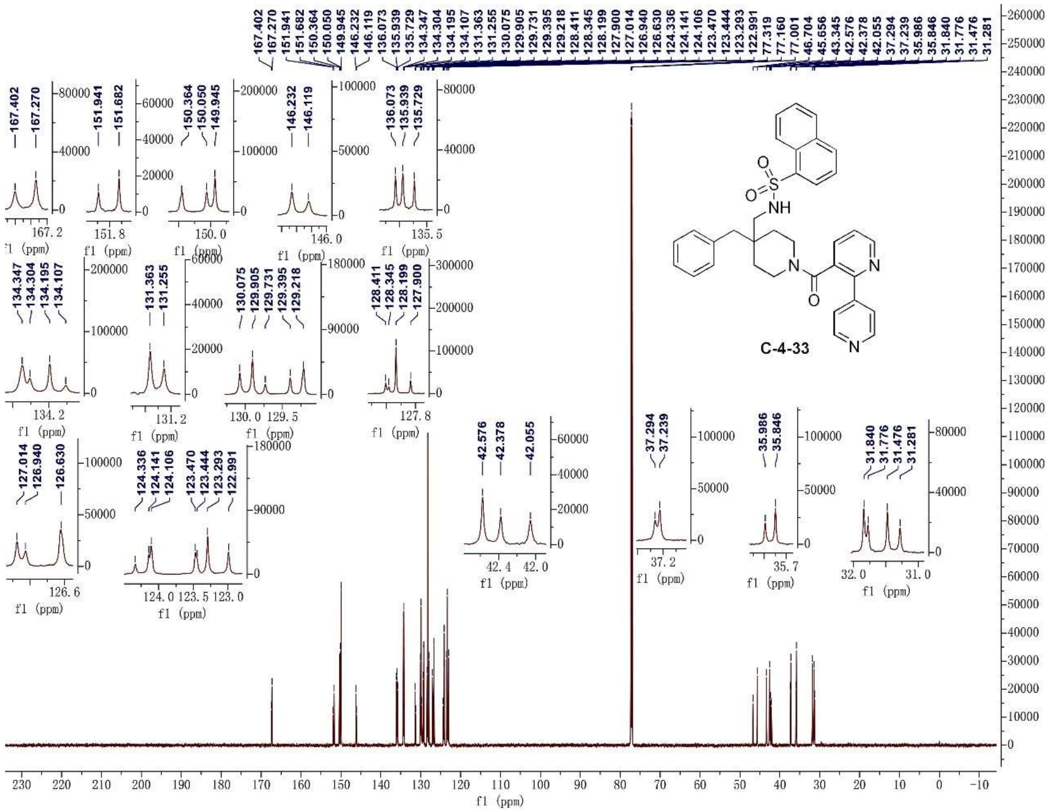

Figure S535: ^{13}C NMR spectrum of **C-4-33**

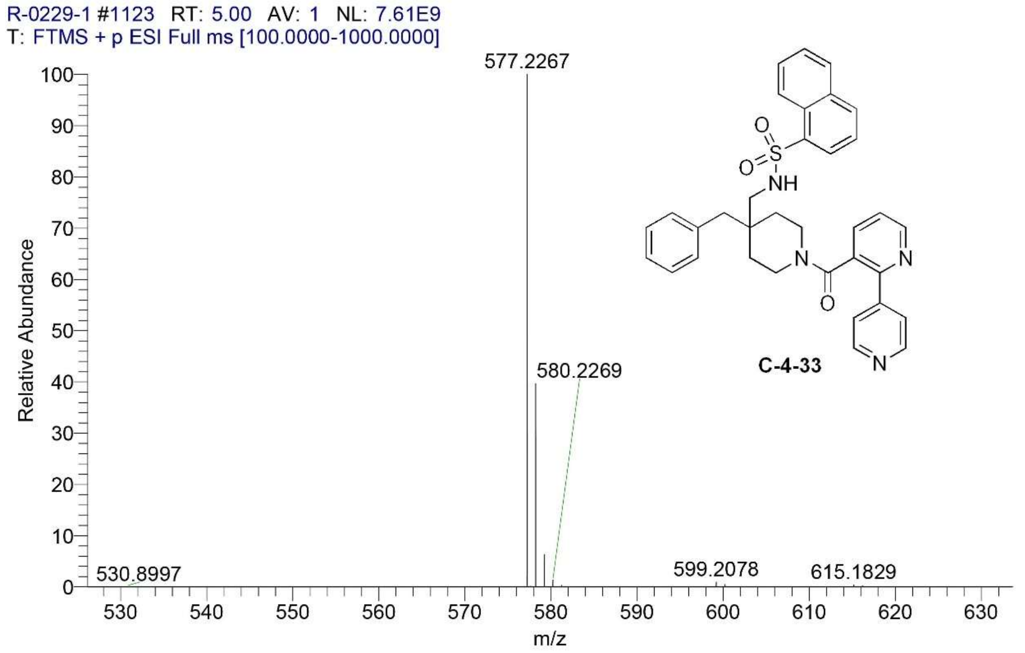

Figure S536: HR-MS (ESI/ion trap) spectrum of **C-4-33**

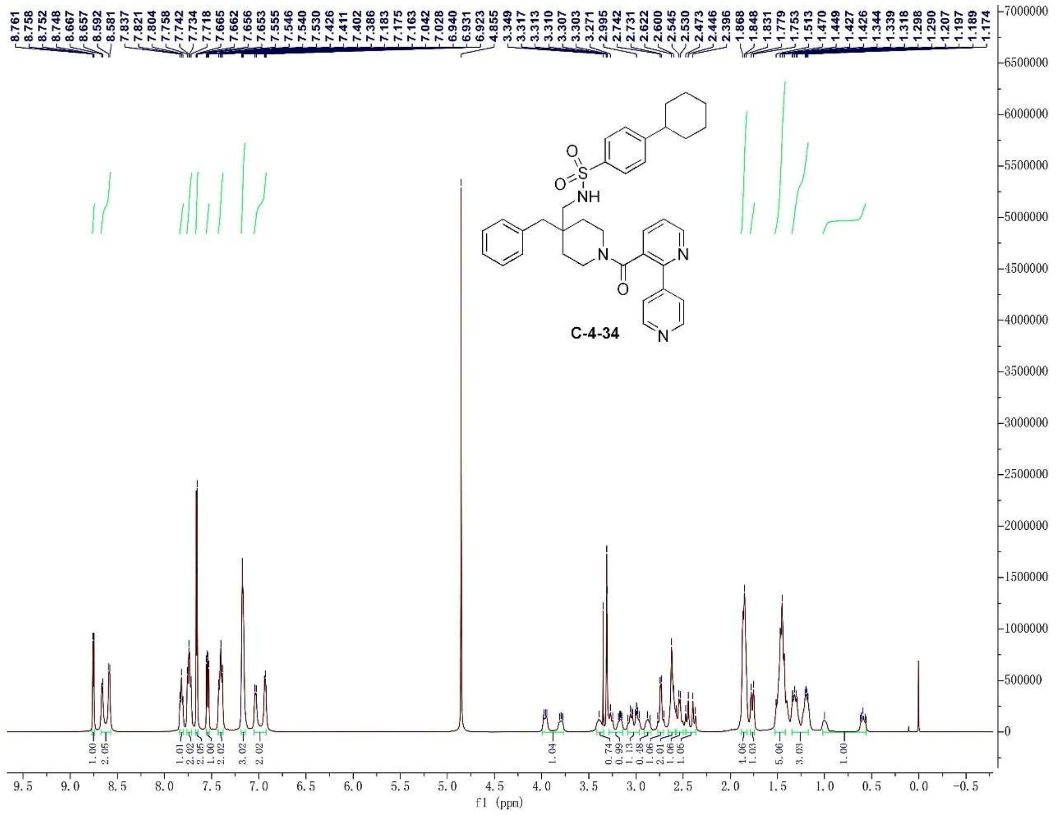

Figure S537: ^{1}H NMR spectrum of **C-4-34**

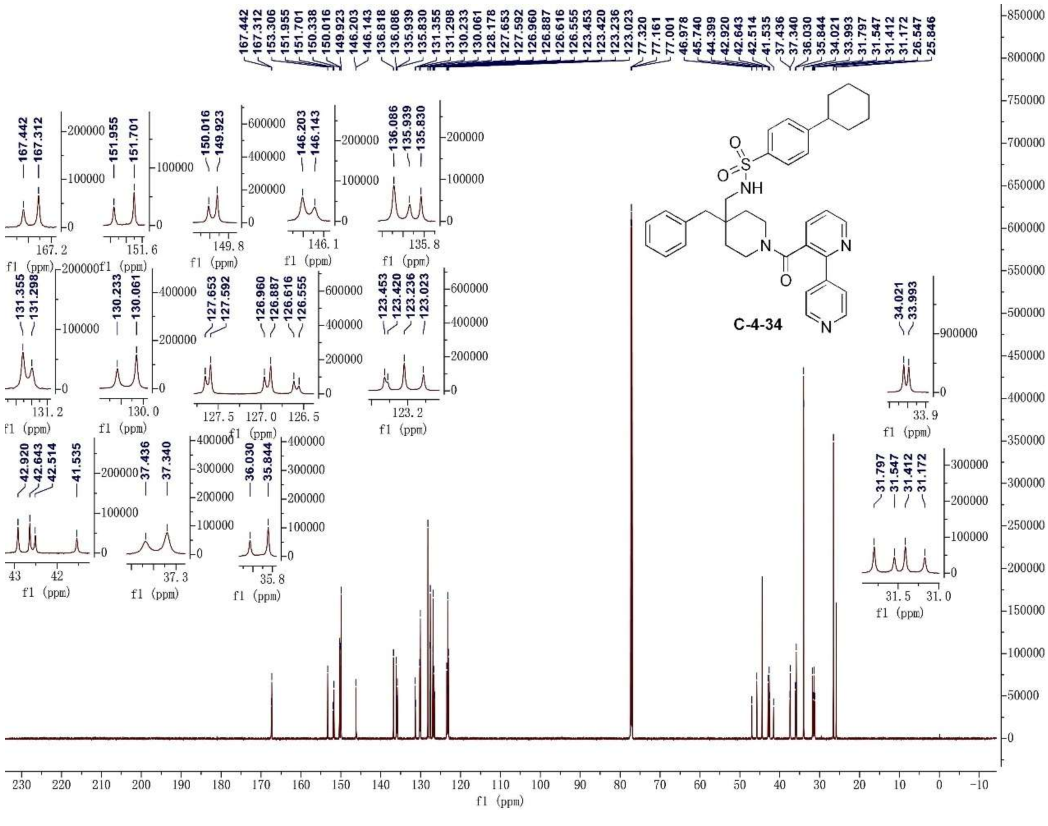

Figure S538: ^{13}C NMR spectrum of **C-4-34**

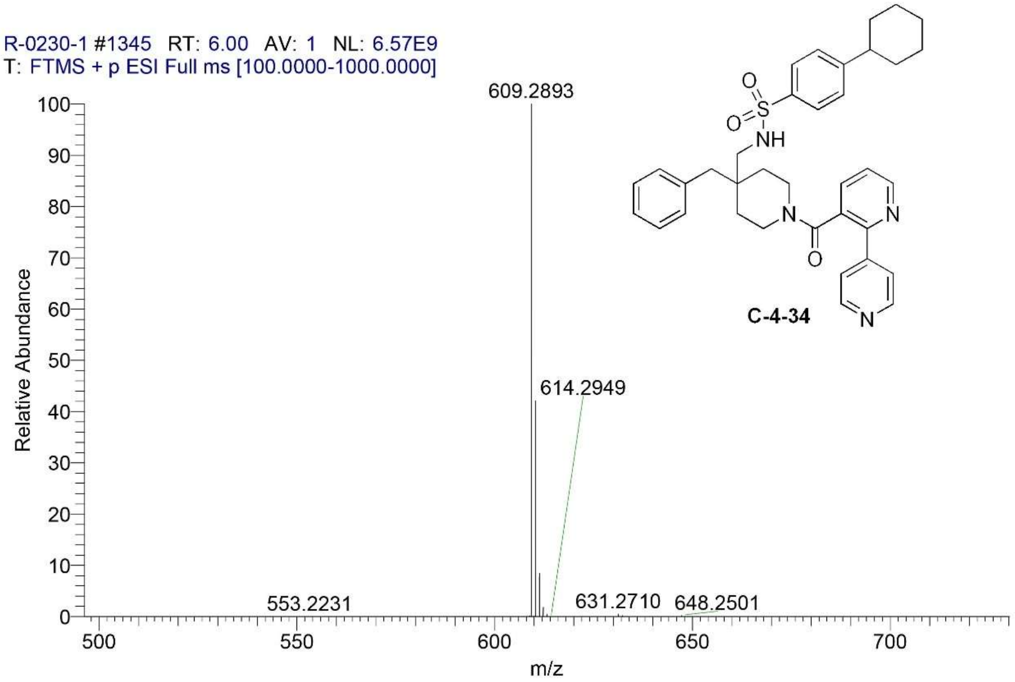

Figure S539: HR-MS (ESI/ion trap) spectrum of **C-4-34**

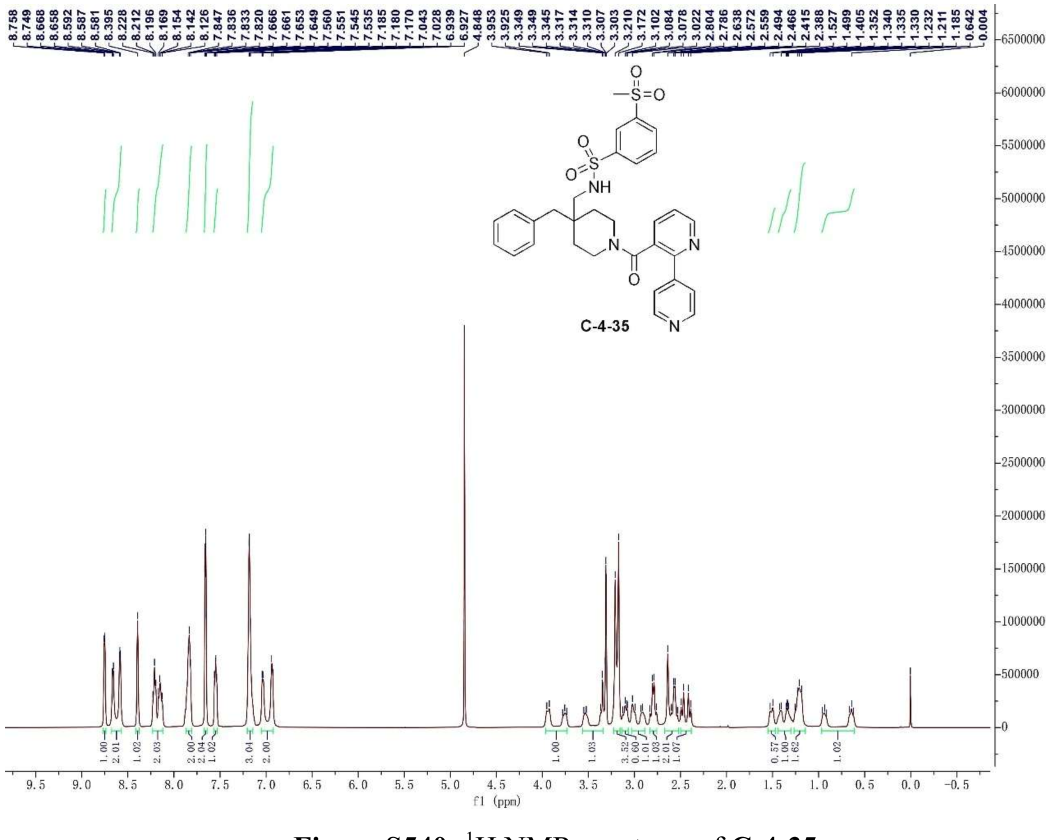

Figure S540: ^{1}H NMR spectrum of **C-4-35**

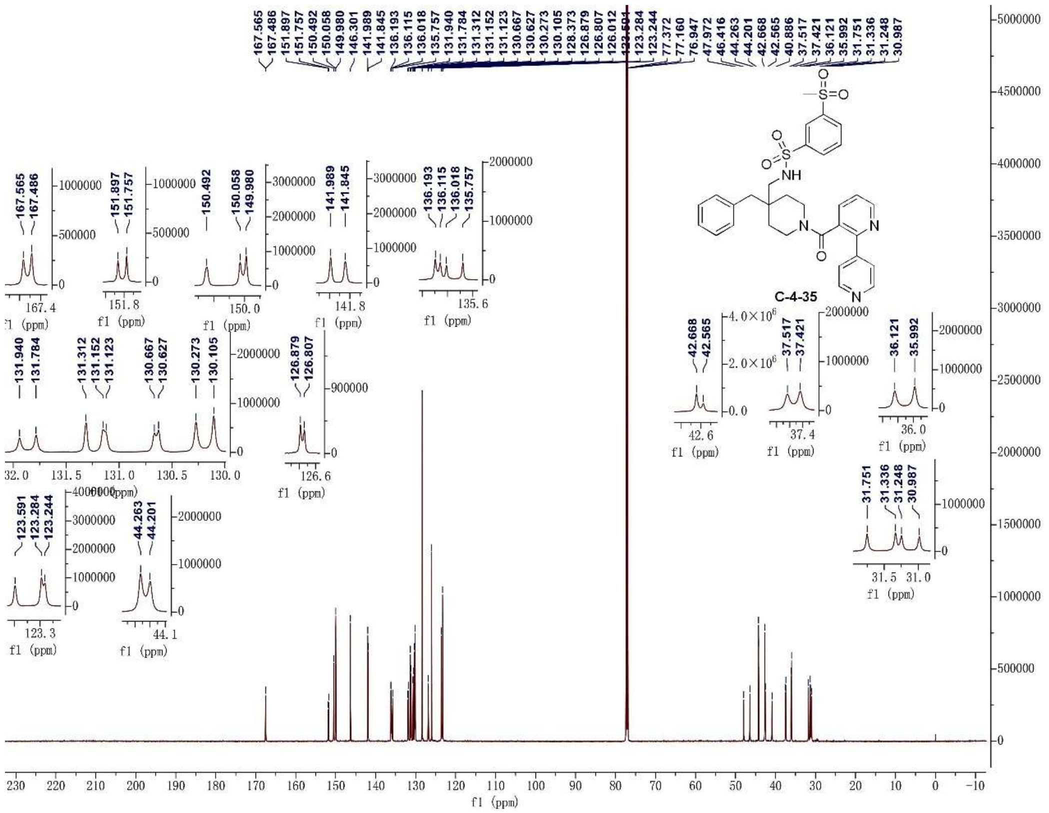

Figure S541: ^{13}C NMR spectrum of **C-4-35**

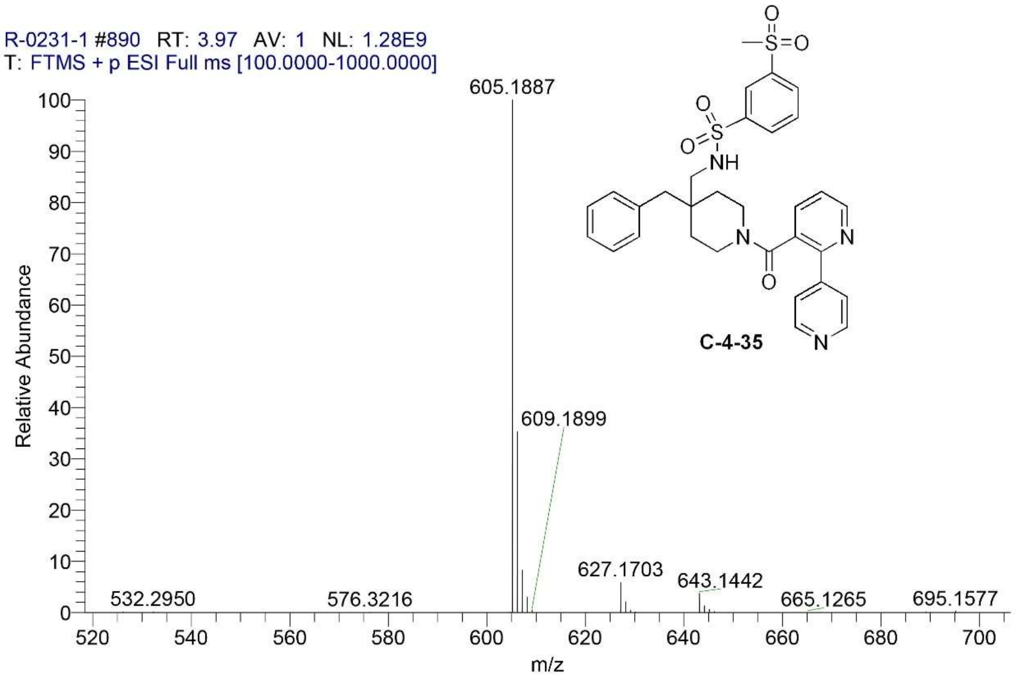

Figure S542: HR-MS (ESI/ion trap) spectrum of **C-4-35**

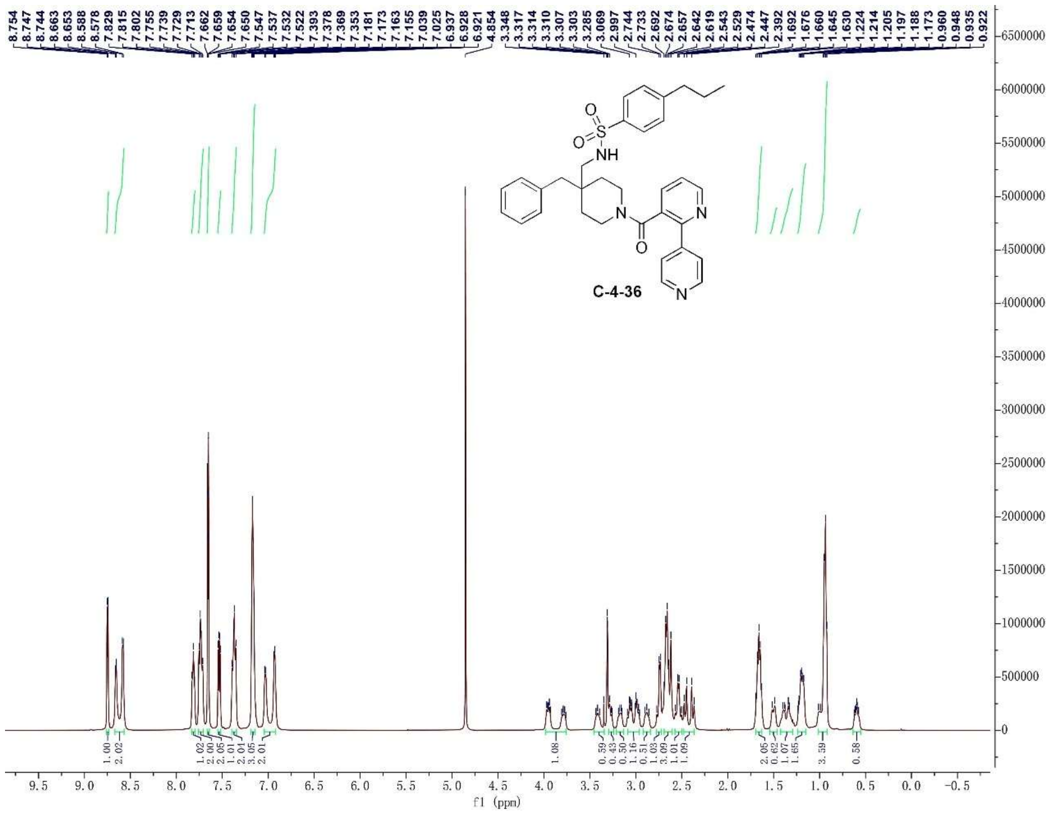

Figure S543: ^{1}H NMR spectrum of **C-4-36**

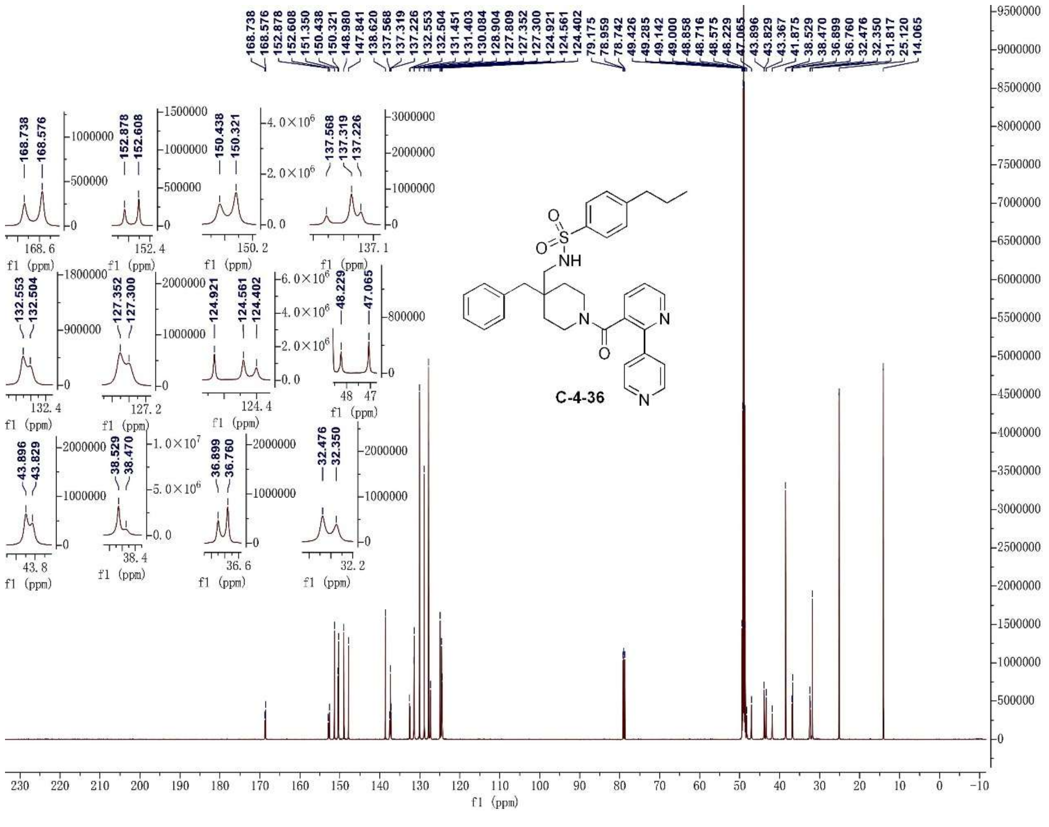

Figure S544: ^{13}C NMR spectrum of **C-4-36**

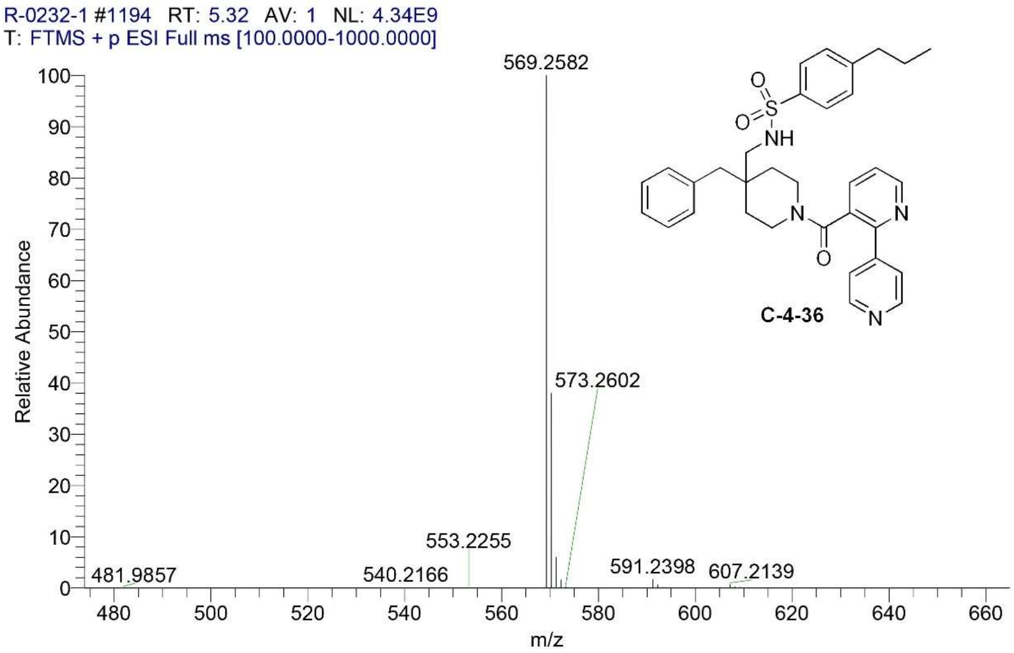

Figure S545: HR-MS (ESI/ion trap) spectrum of **C-4-36**

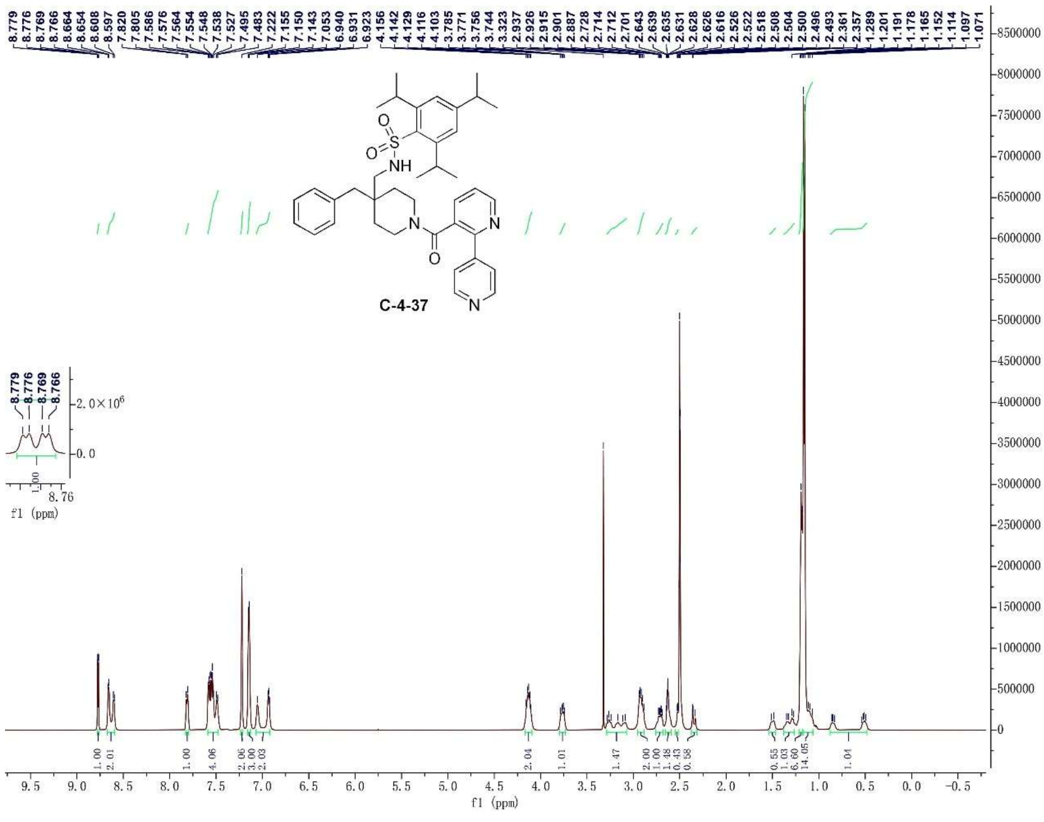

Figure S546: ^{1}H NMR spectrum of **C-4-37**

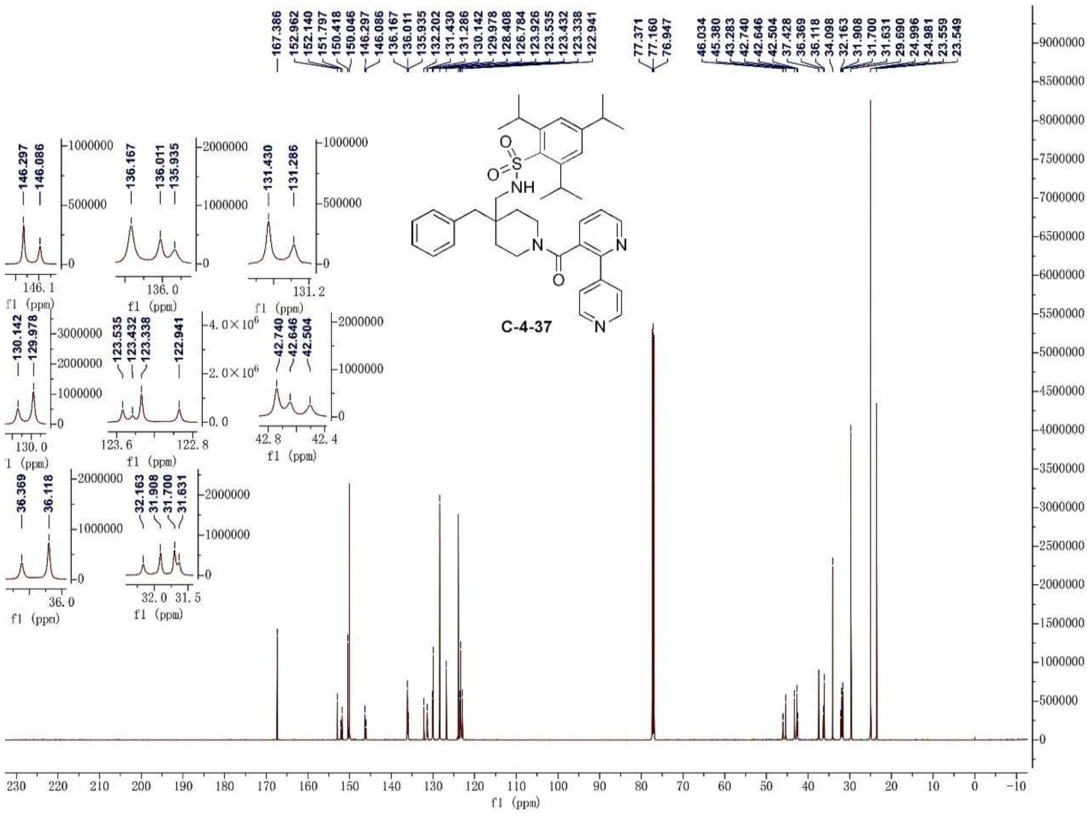

Figure S547: ^{13}C NMR spectrum of **C-4-37**

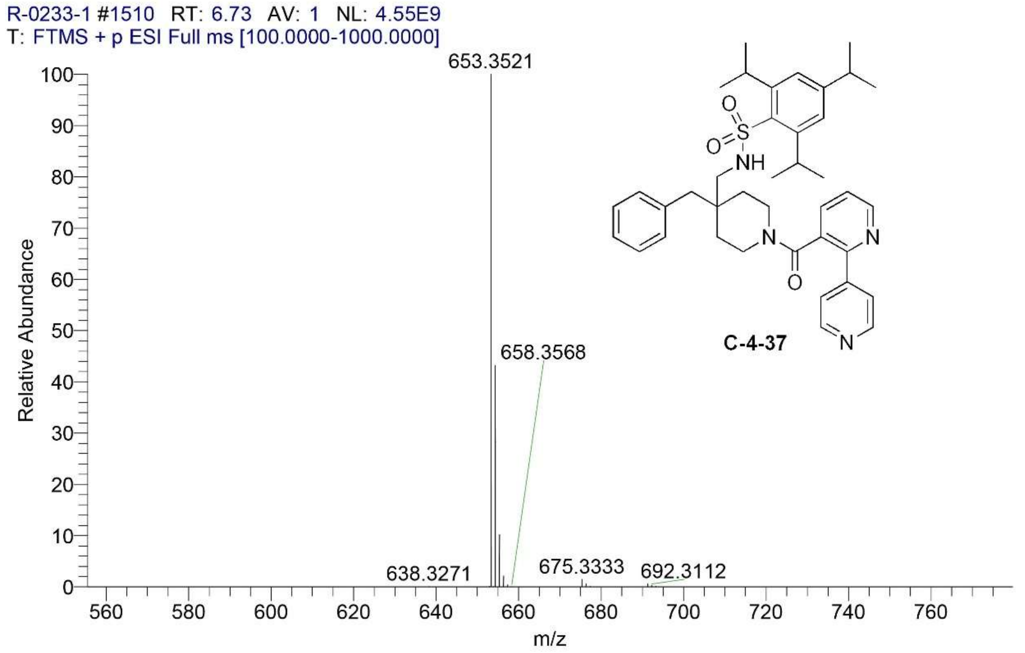

Figure S548: HR-MS (ESI/ion trap) spectrum of **C-4-37**

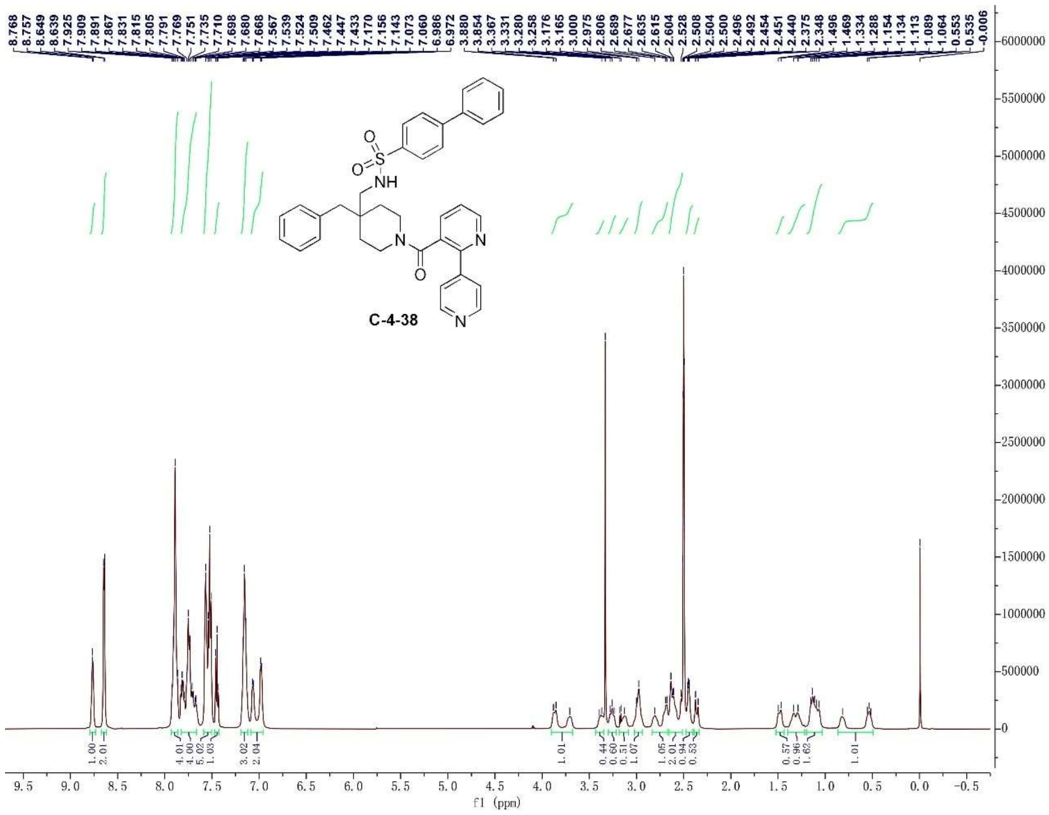

Figure S549: ^{1}H NMR spectrum of **C-4-38**

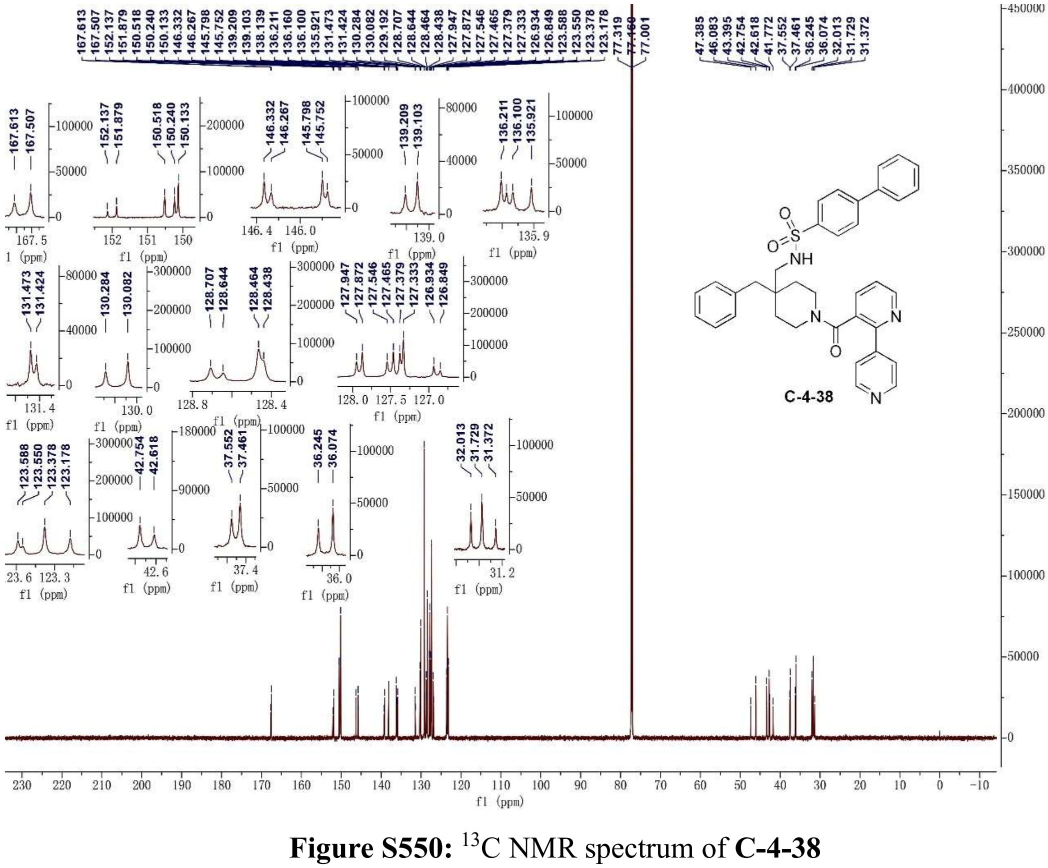

Figure S550: ^{13}C NMR spectrum of **C-4-38**

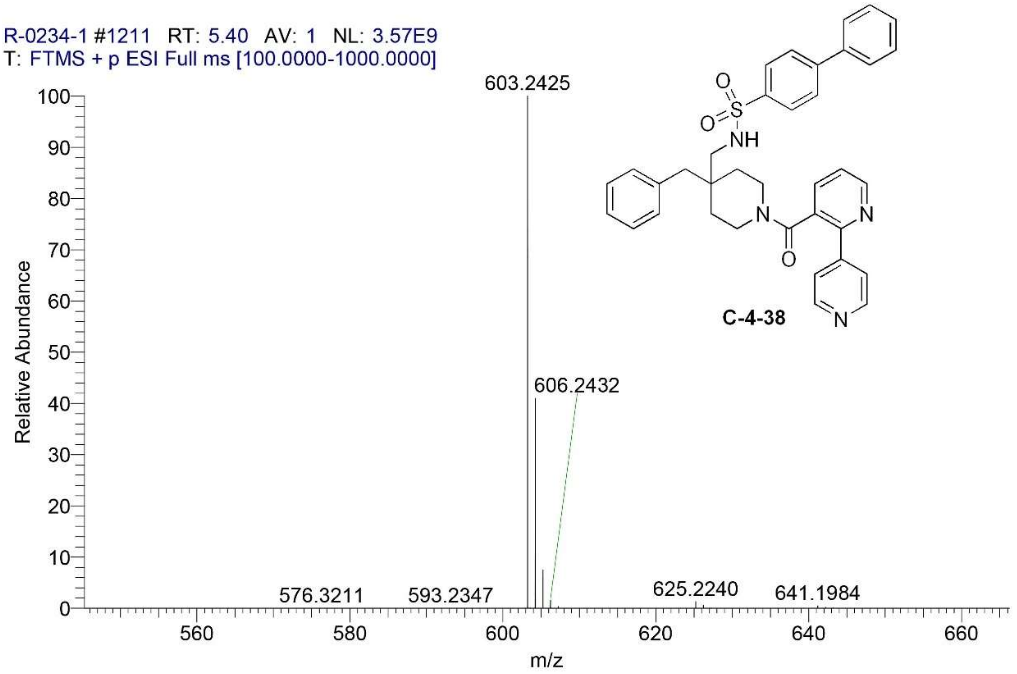

Figure S551: HR-MS (ESI/ion trap) spectrum of **C-4-38**

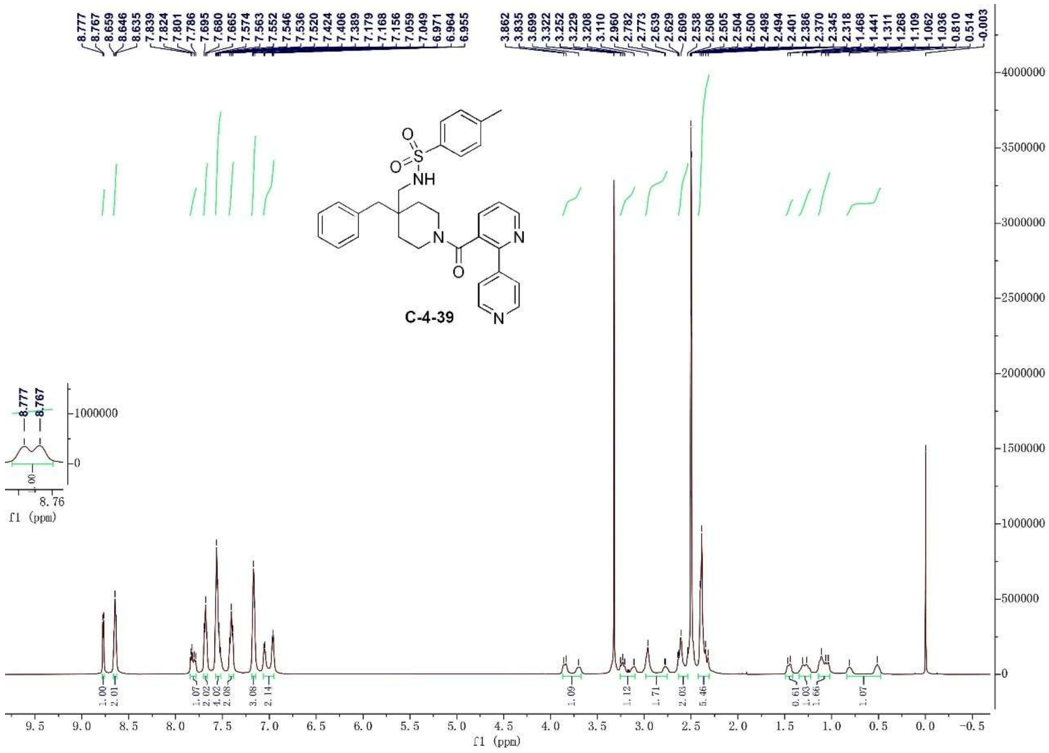

Figure S552: ^{1}H NMR spectrum of **C-4-39**

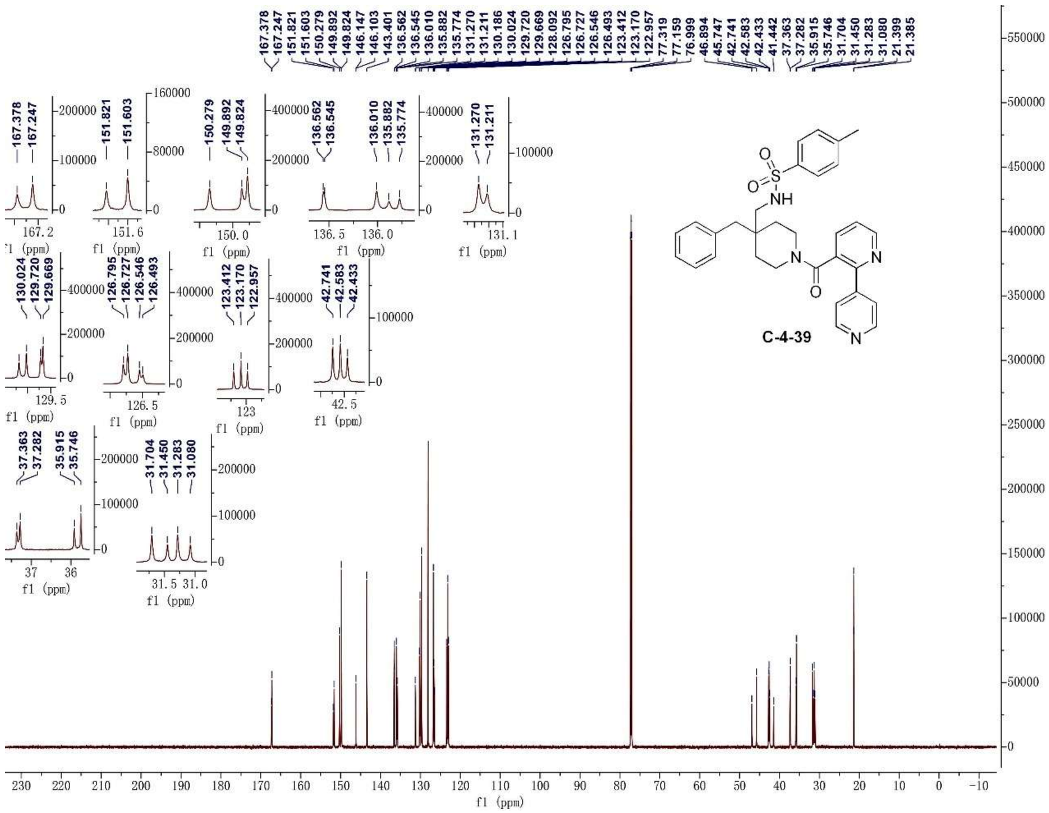

Figure S553: ^{13}C NMR spectrum of **C-4-39**

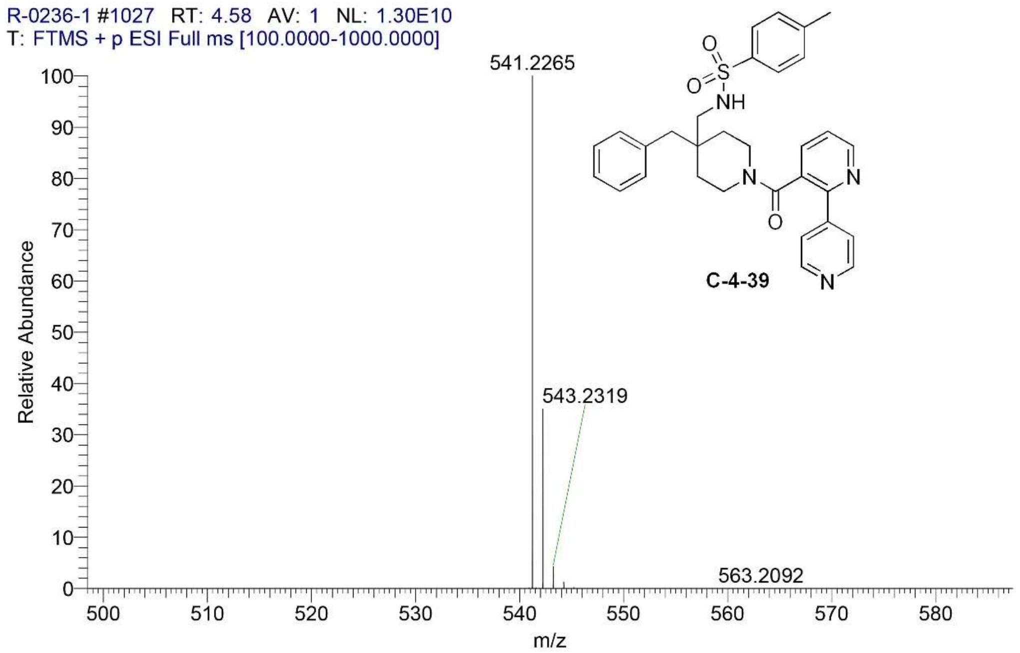

Figure S554: HR-MS (ESI/ion trap) spectrum of **C-4-39**

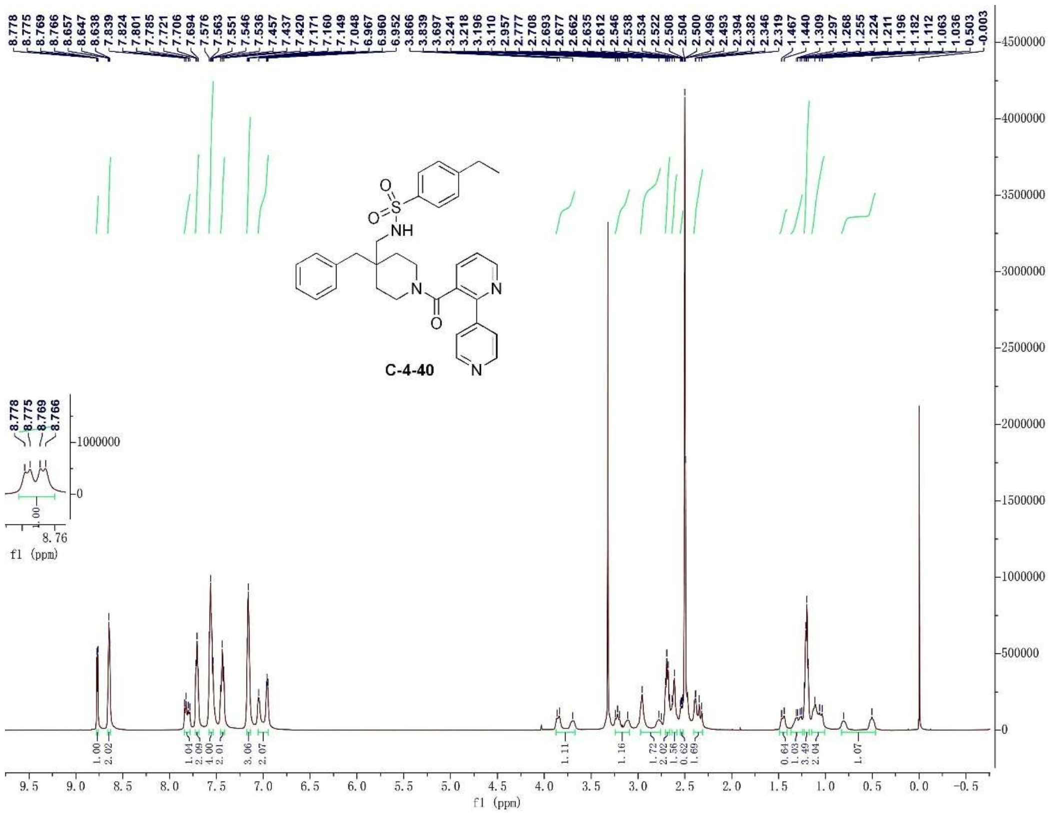

Figure S555: ^{1}H NMR spectrum of **C-4-40**

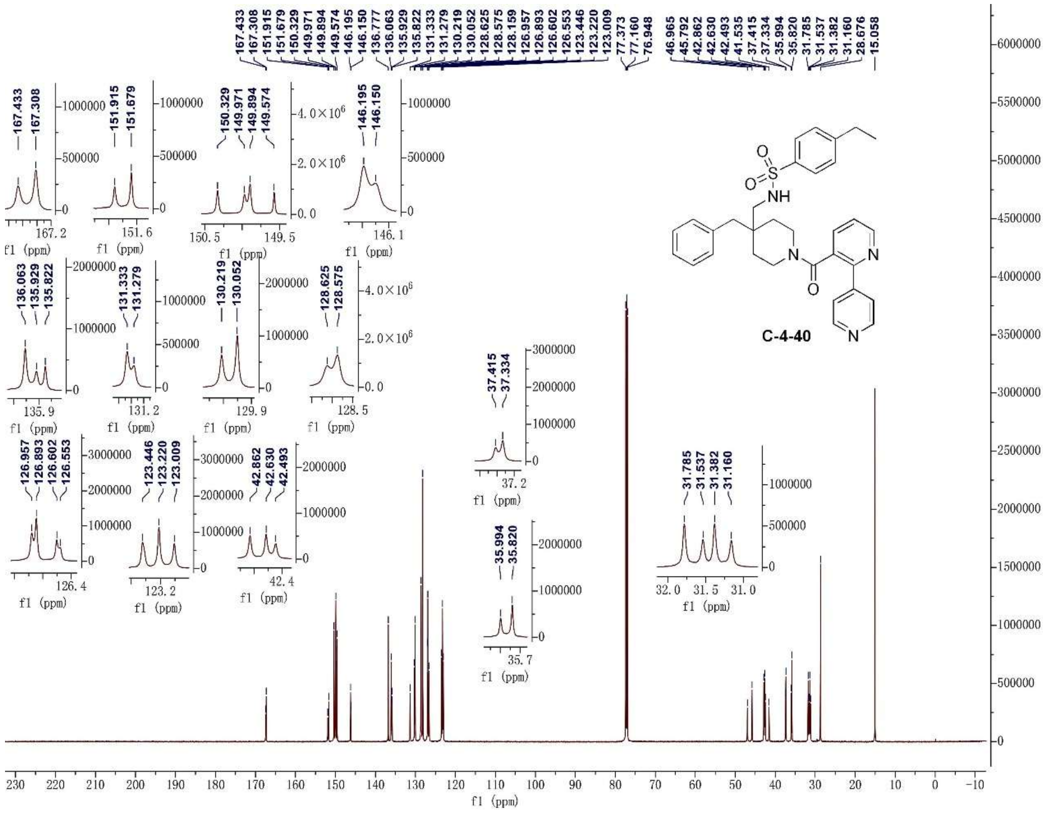

Figure S556: ^{13}C NMR spectrum of **C-4-40**

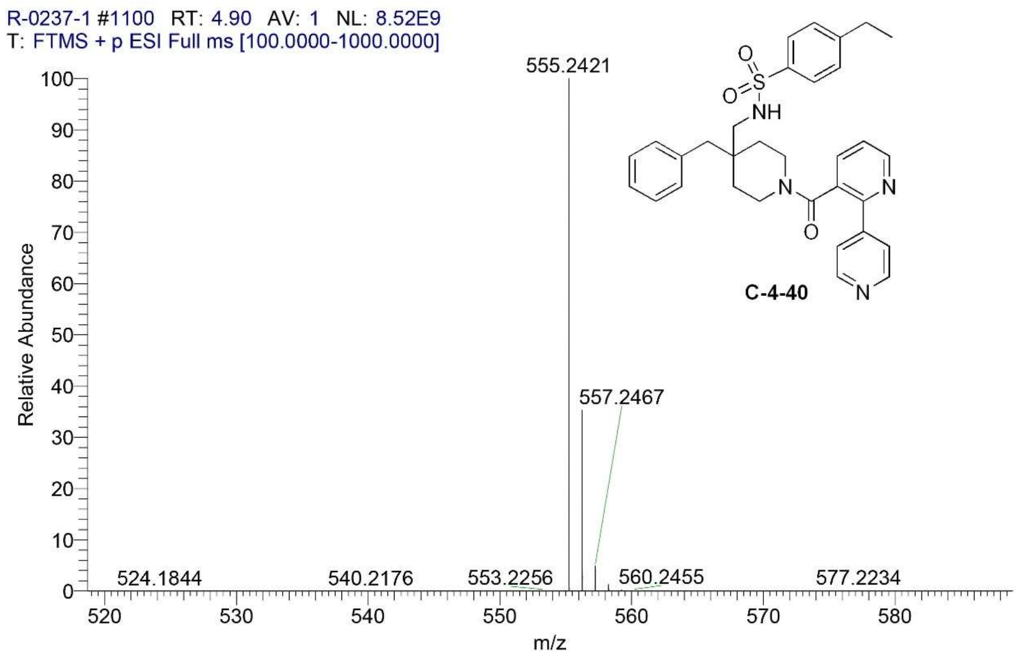

Figure S557: HR-MS (ESI/ion trap) spectrum of **C-4-40**

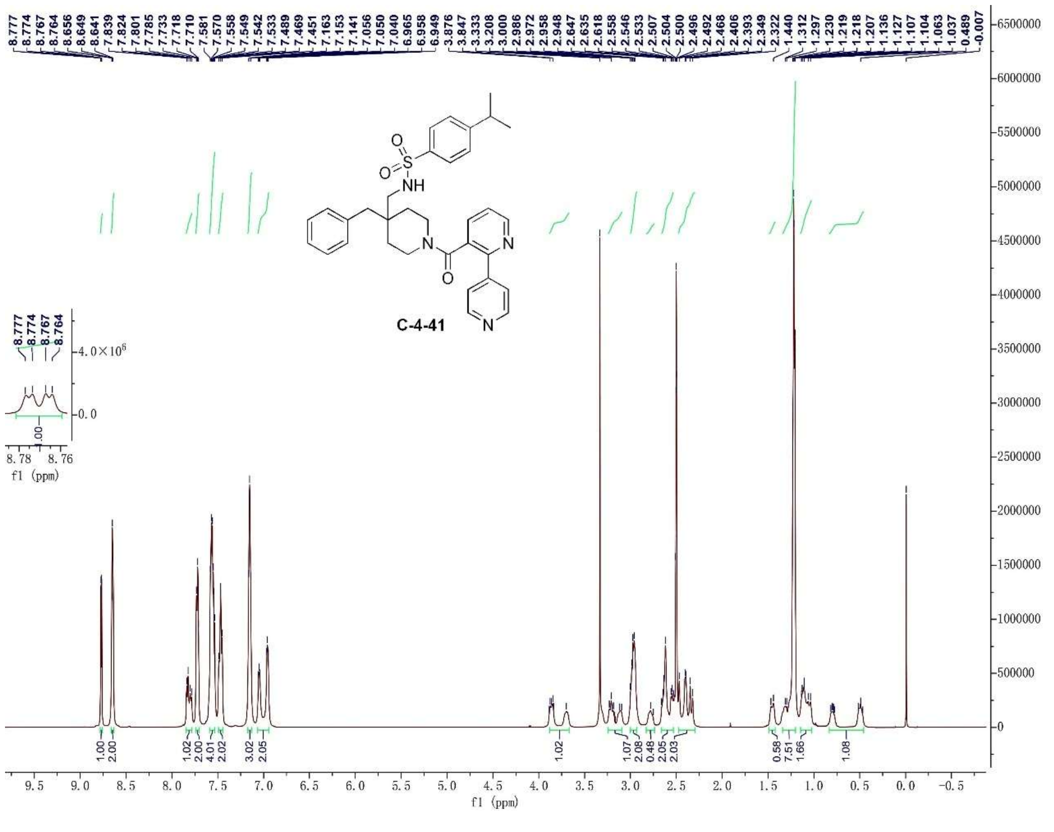

Figure S558: ^{1}H NMR spectrum of **C-4-41**

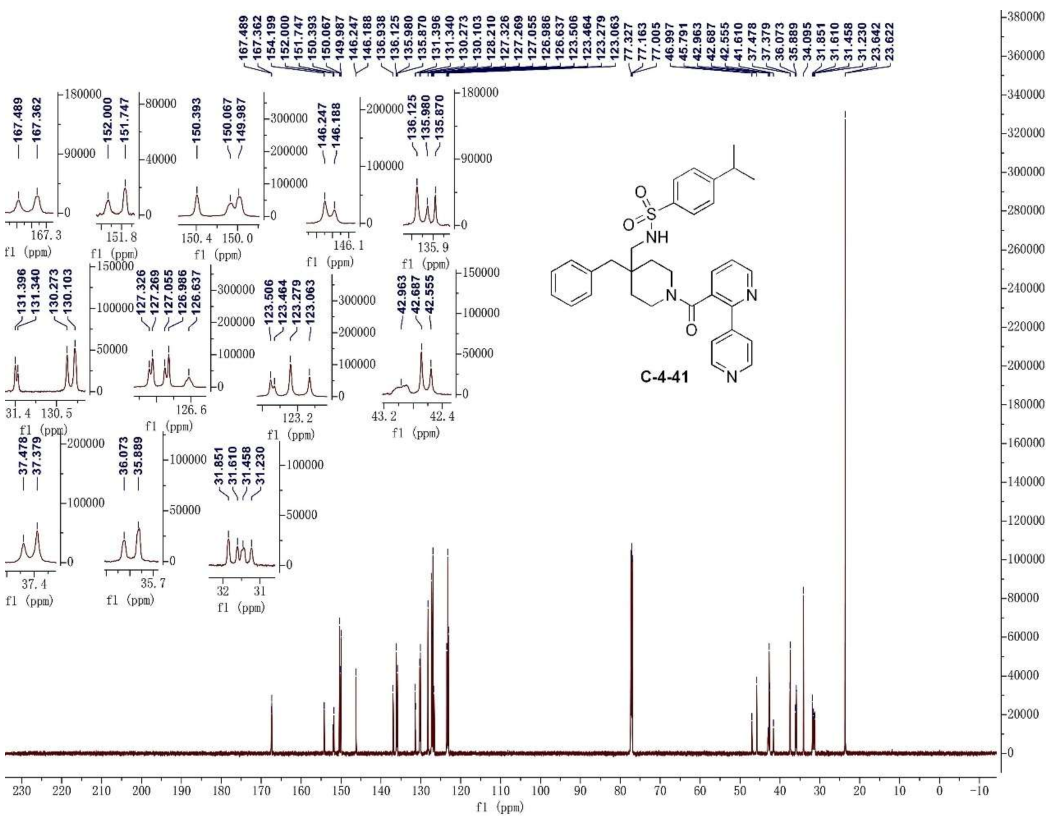

Figure S559: ^{13}C NMR spectrum of **C-4-41**

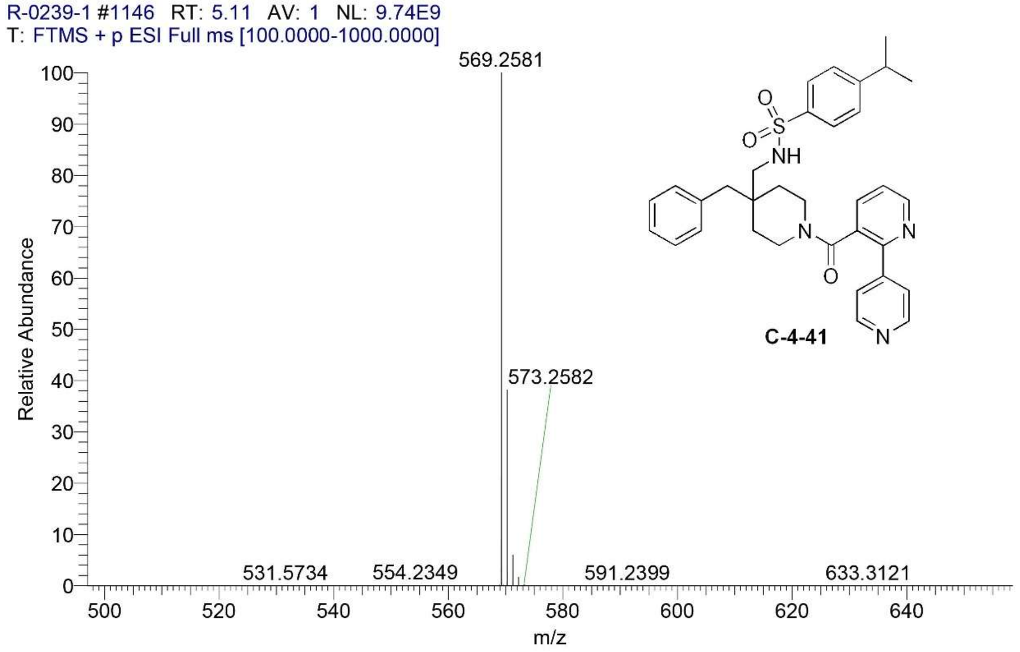

Figure S560: HR-MS (ESI/ion trap) spectrum of **C-4-41**

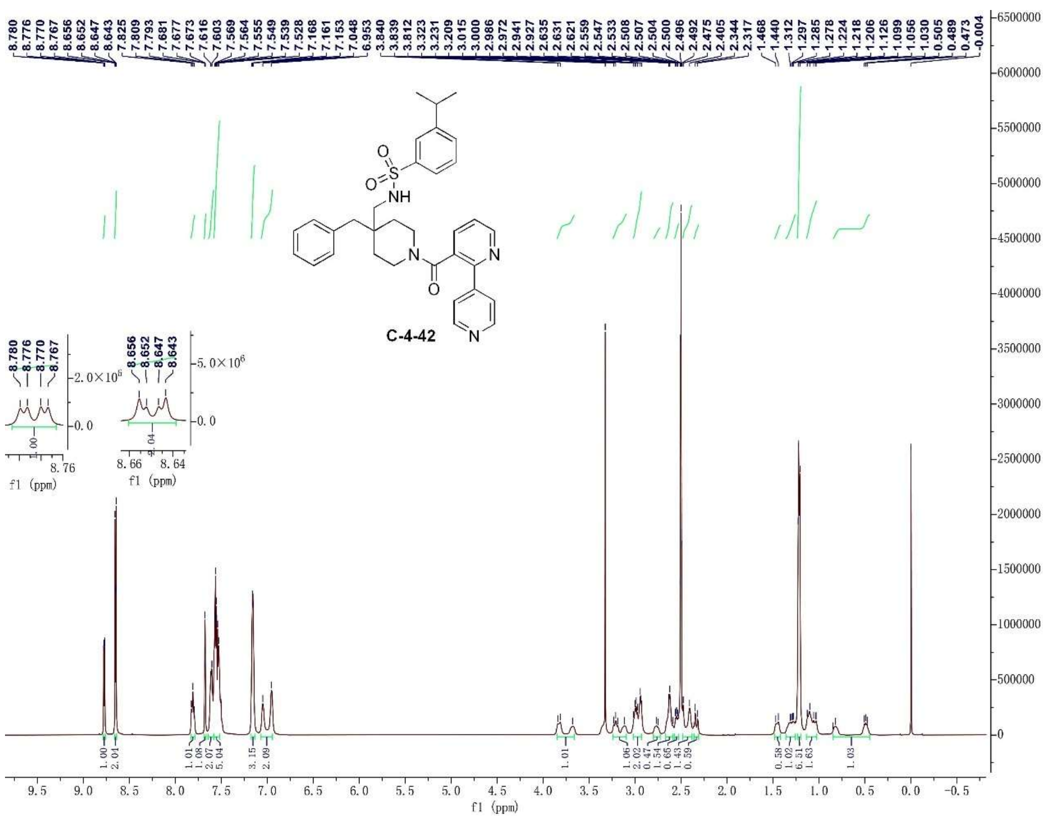

Figure S561: ^{1}H NMR spectrum of **C-4-42**

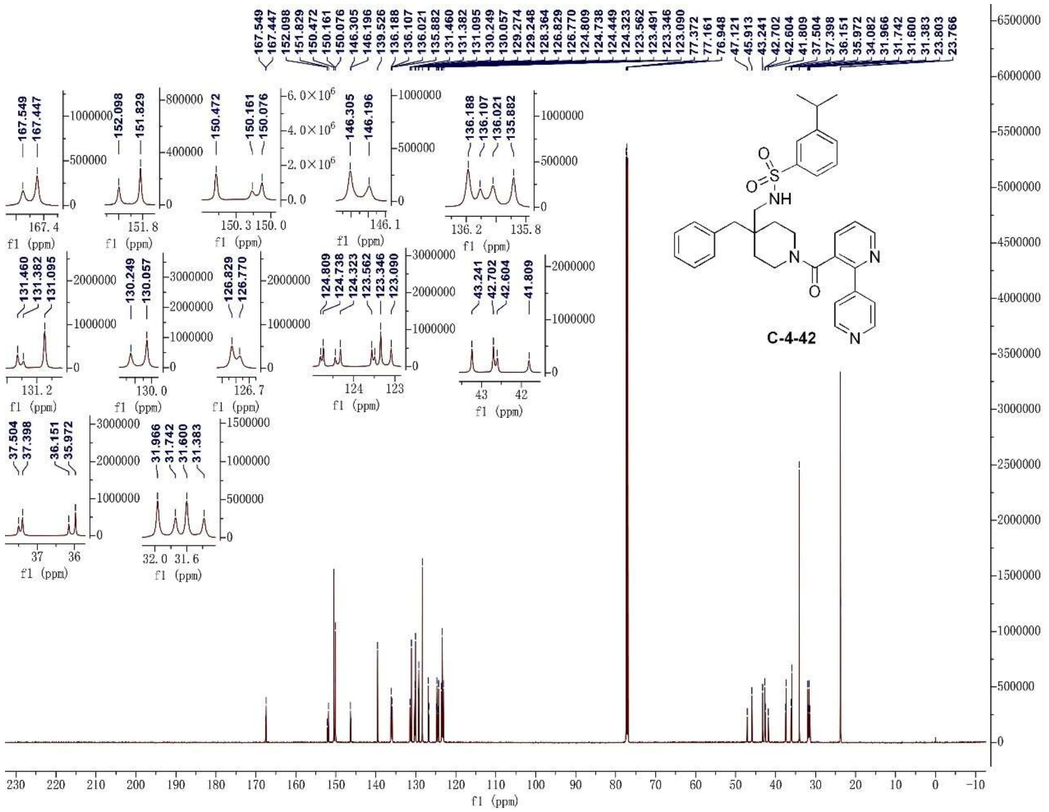

Figure S562: ^{13}C NMR spectrum of **C-4-42**

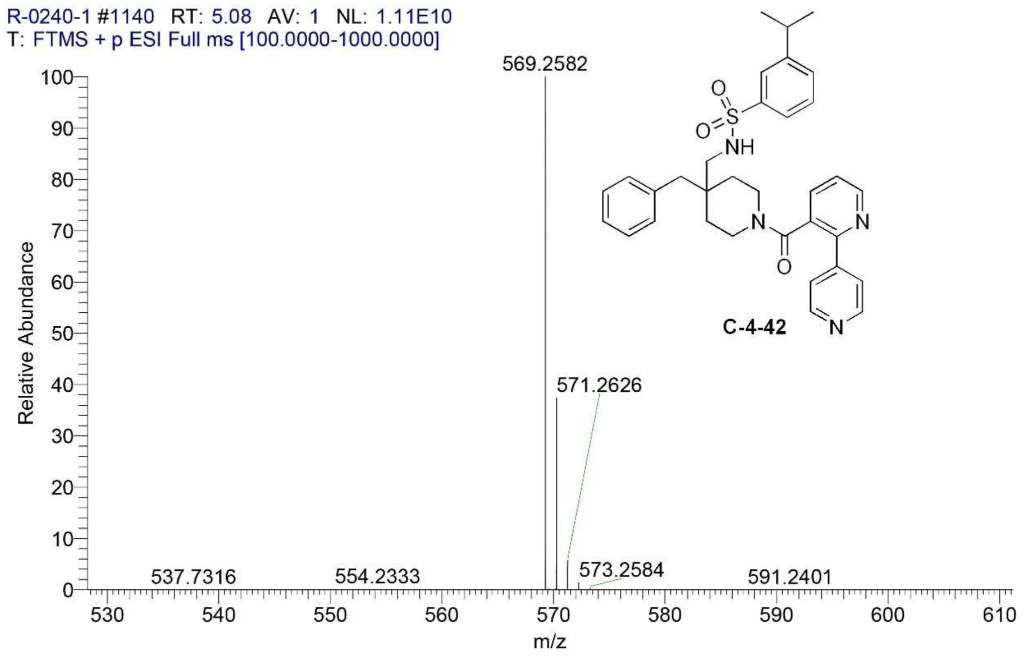

Figure S563: HR-MS (ESI/ion trap) spectrum of **C-4-42**

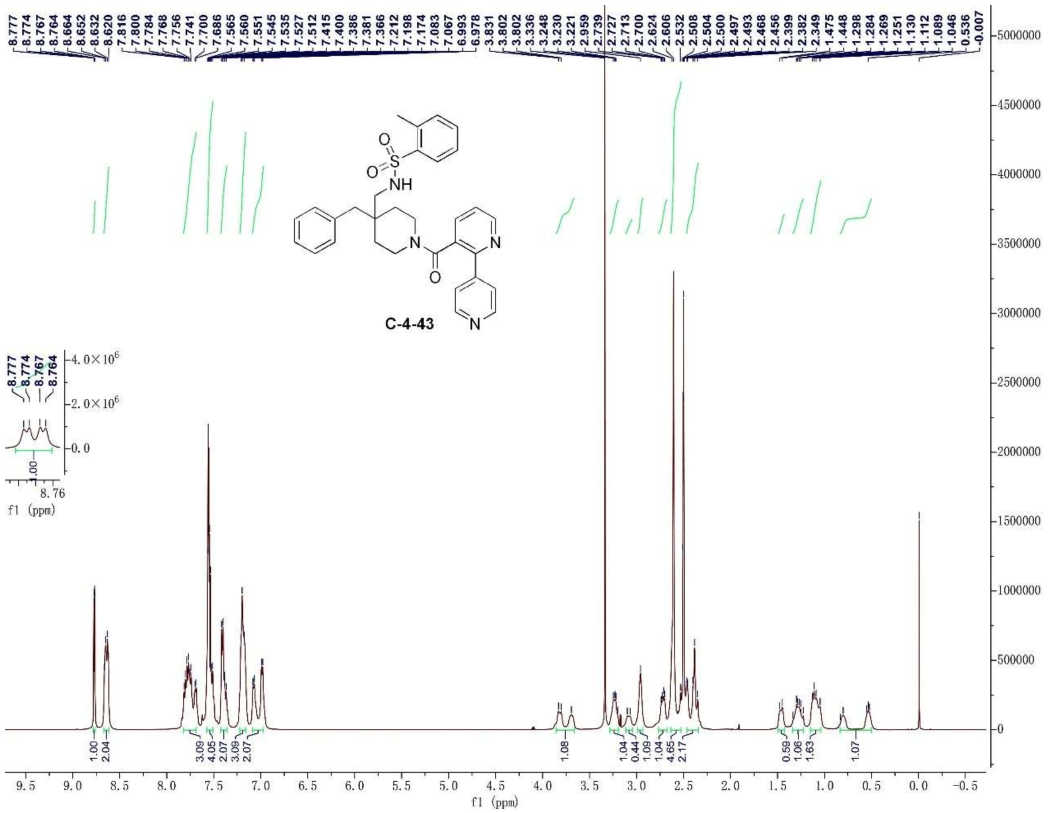

Figure S564: ^{1}H NMR spectrum of **C-4-43**

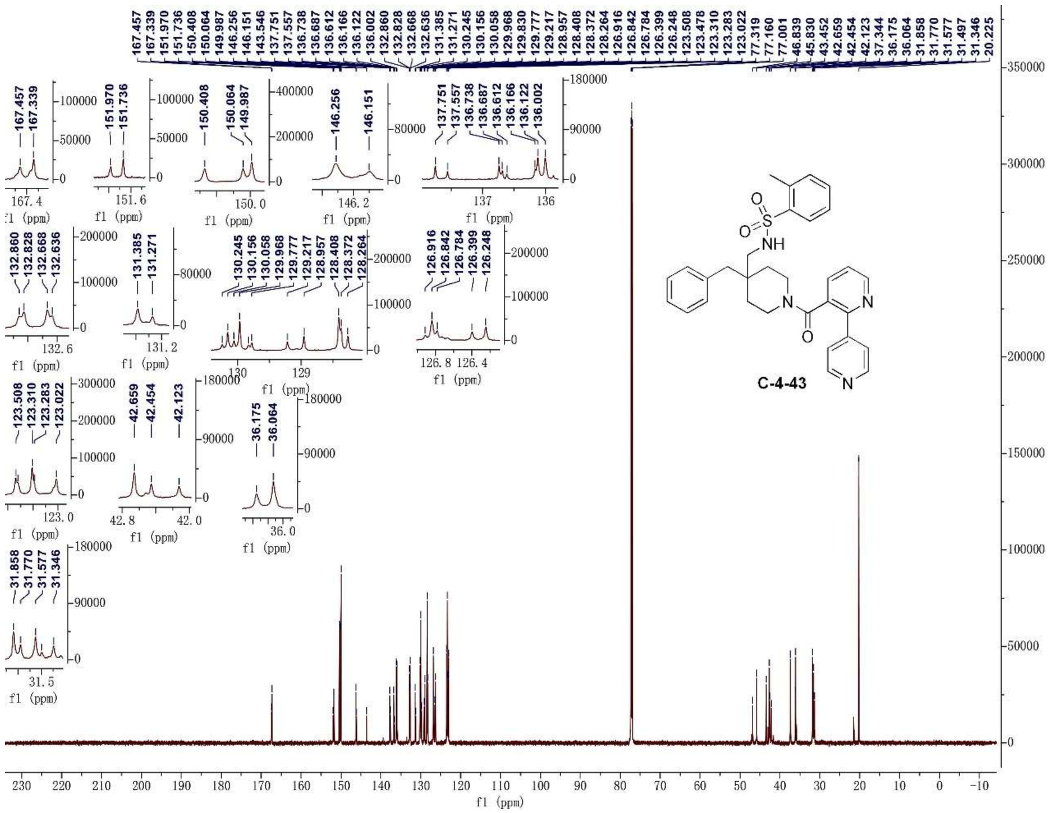

Figure S565: ^{13}C NMR spectrum of **C-4-43**

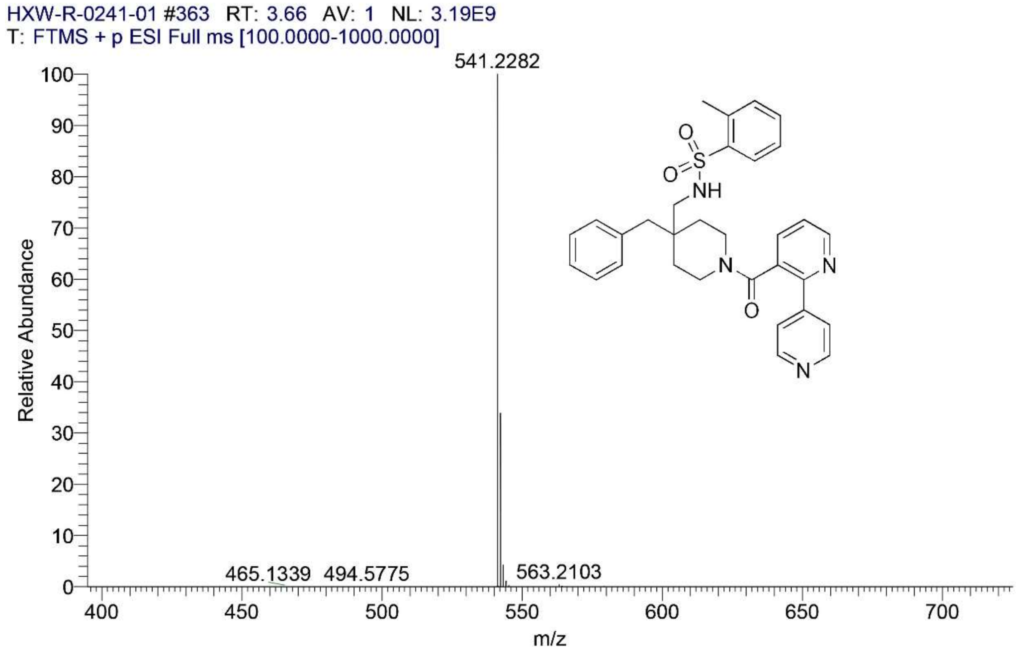

Figure S566: HR-MS (ESI/ion trap) spectrum of **C-4-43**

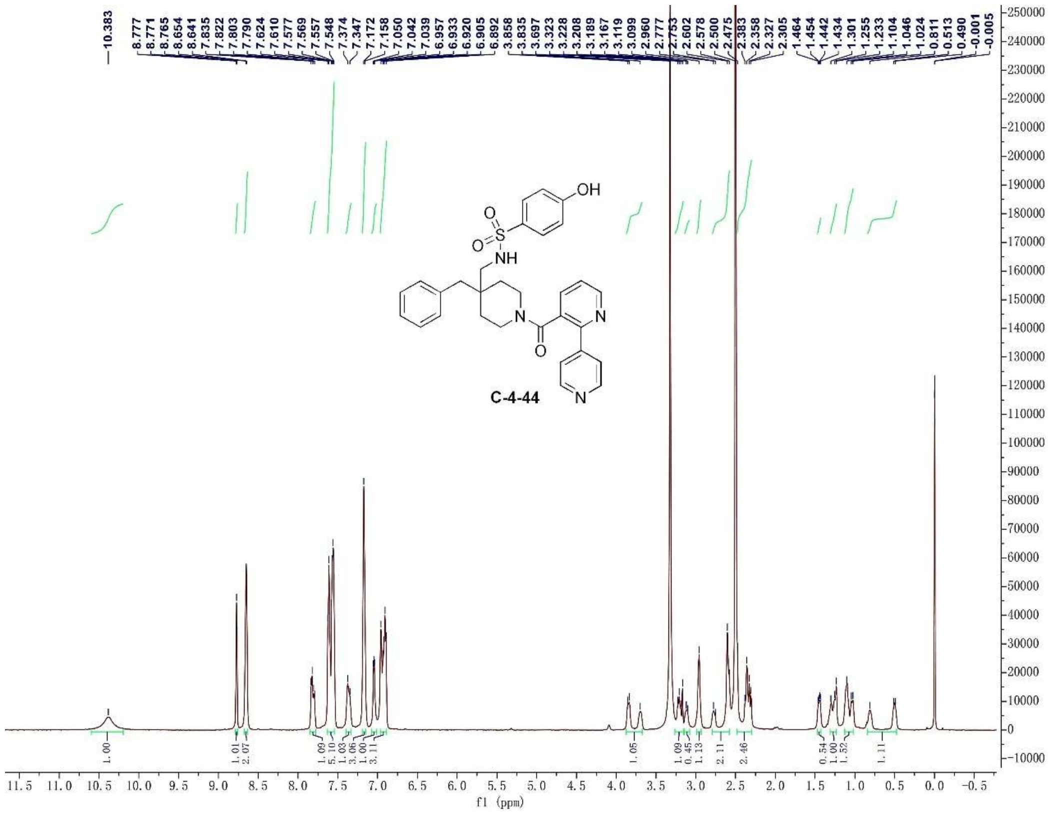

Figure S567: ^{1}H NMR spectrum of **C-4-44**

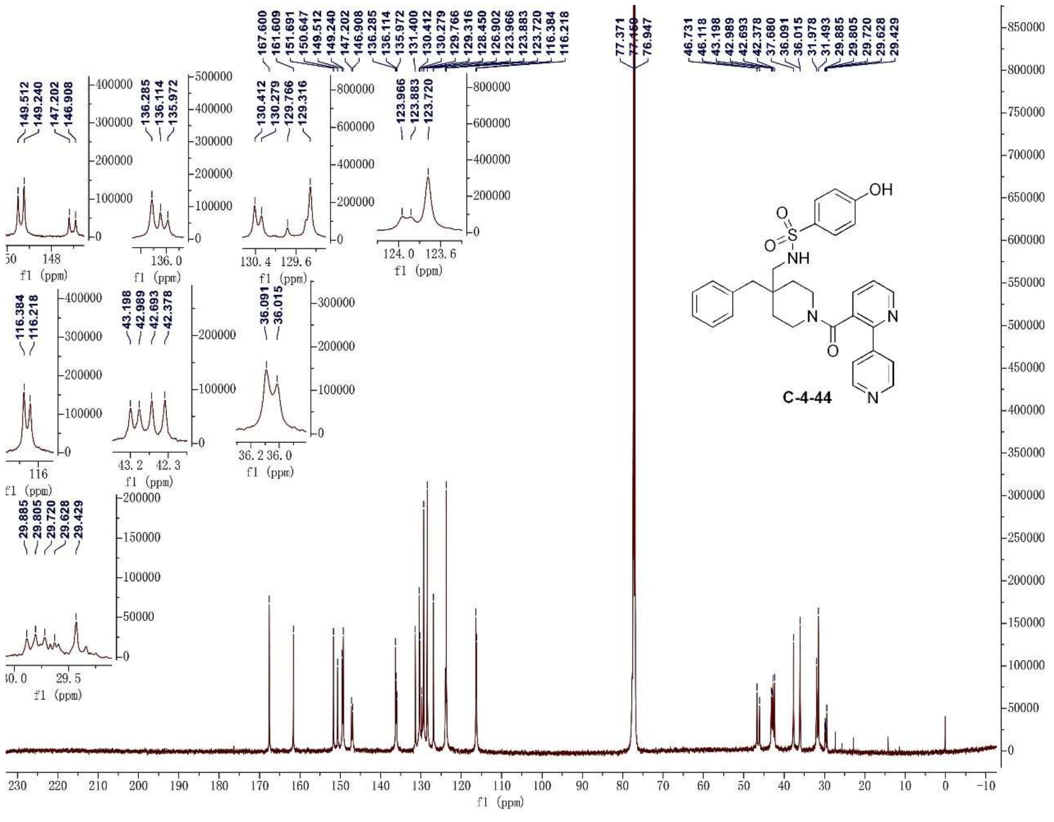

Figure S568: ^{13}C NMR spectrum of **C-4-44**

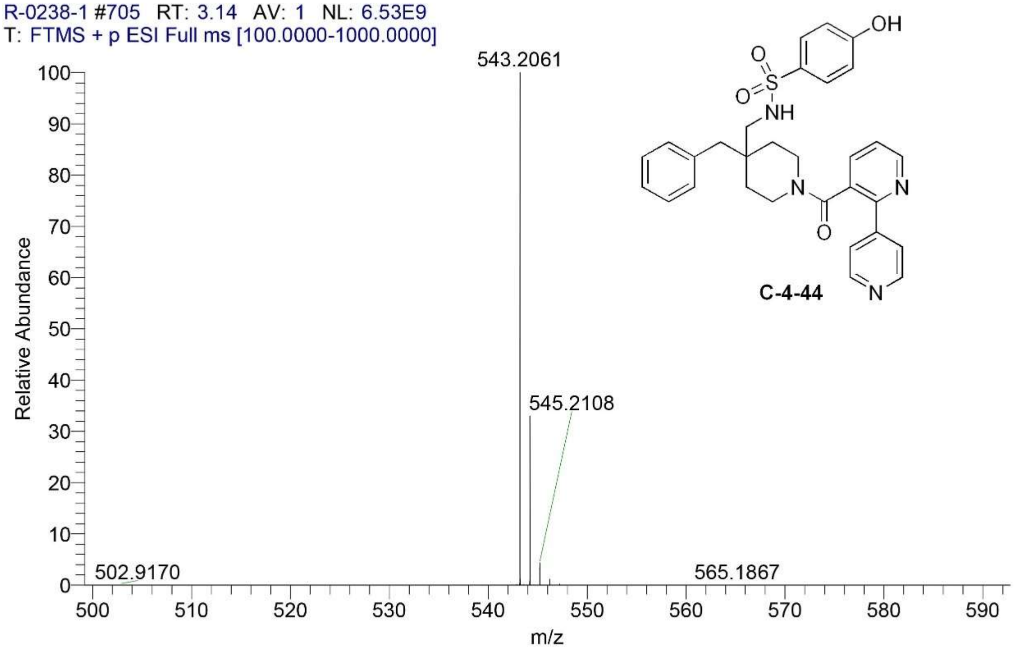

Figure S569: HR-MS (ESI/ion trap) spectrum of **C-4-44**

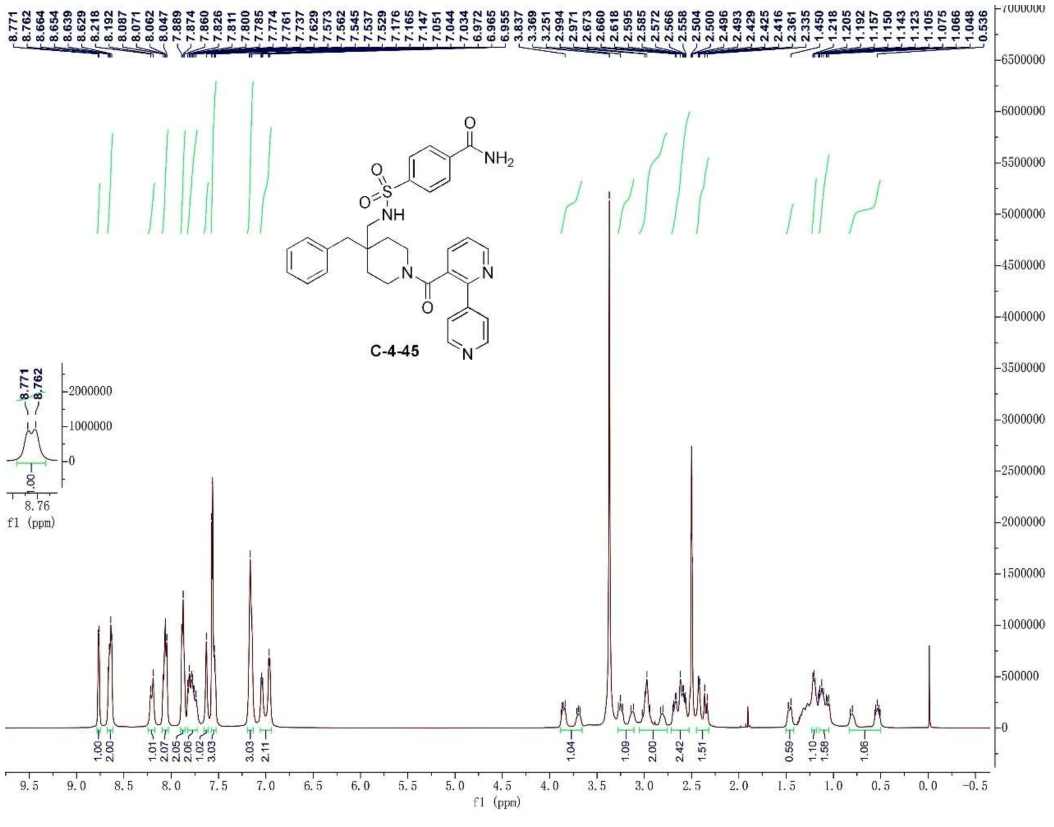

Figure S570: ^{1}H NMR spectrum of **C-4-45**

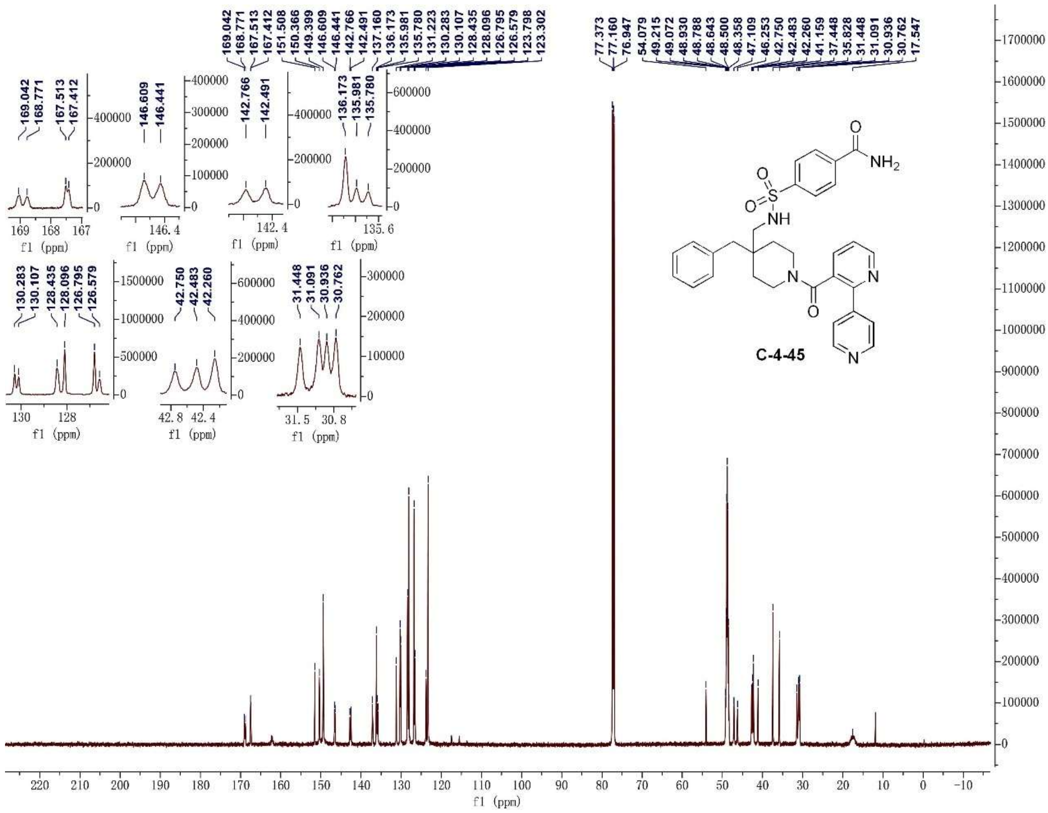

Figure S571: ^{13}C NMR spectrum of **C-4-45**

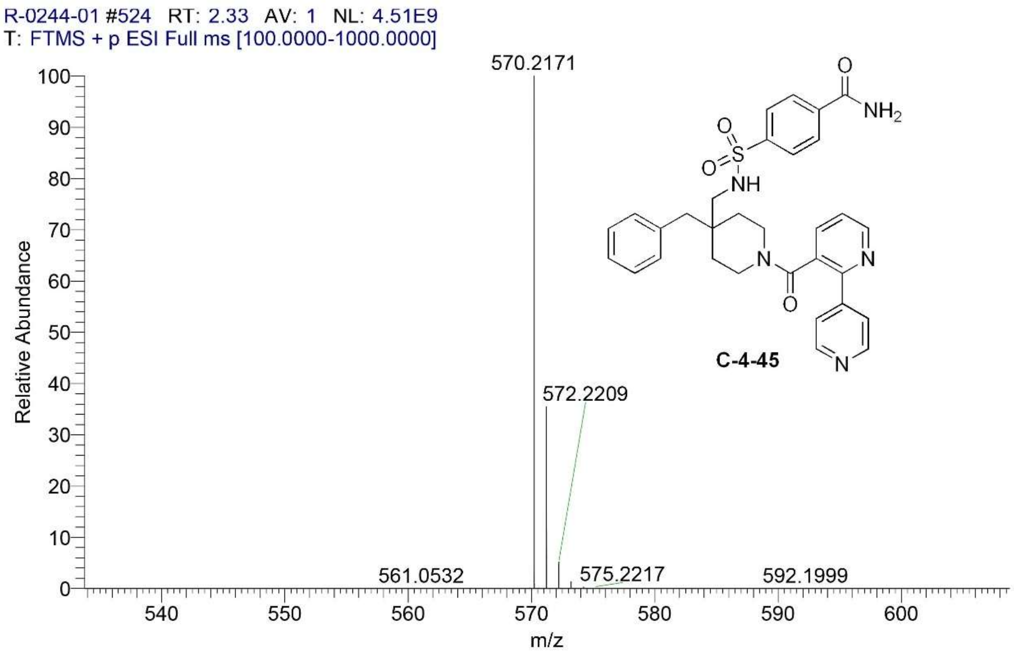

Figure S572: HR-MS (ESI/ion trap) spectrum of **C-4-45**

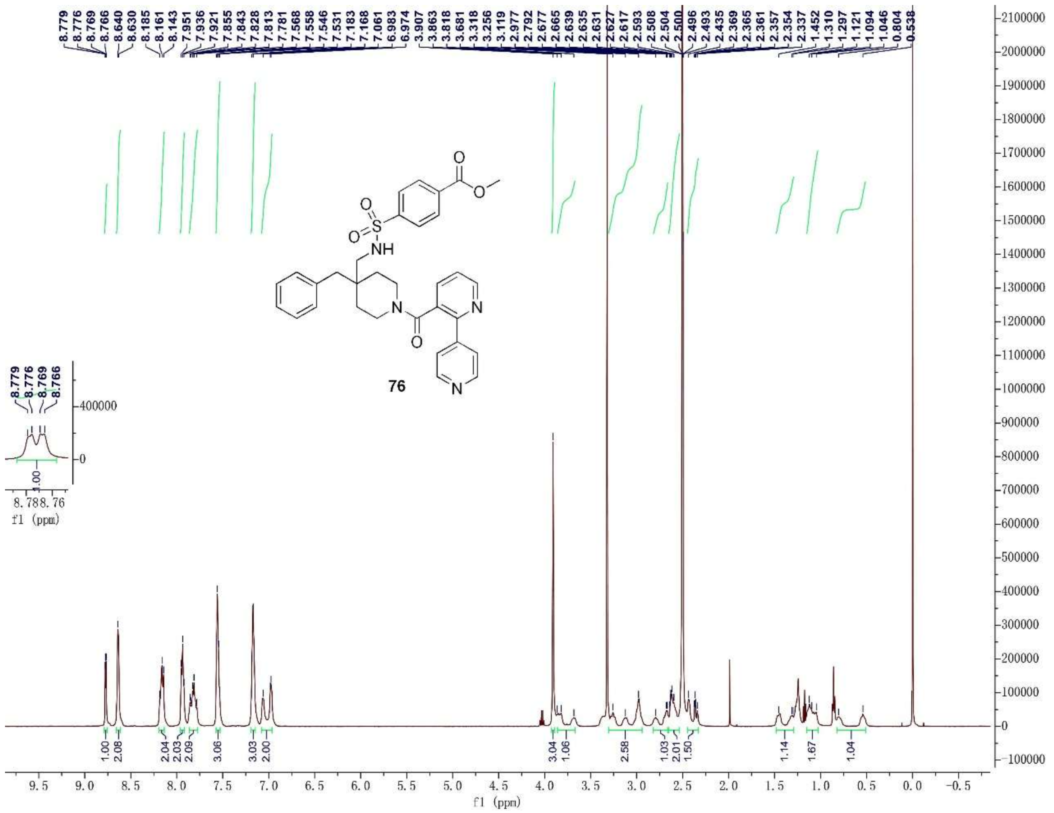

Figure S573: ^{1}H NMR spectrum of **76**

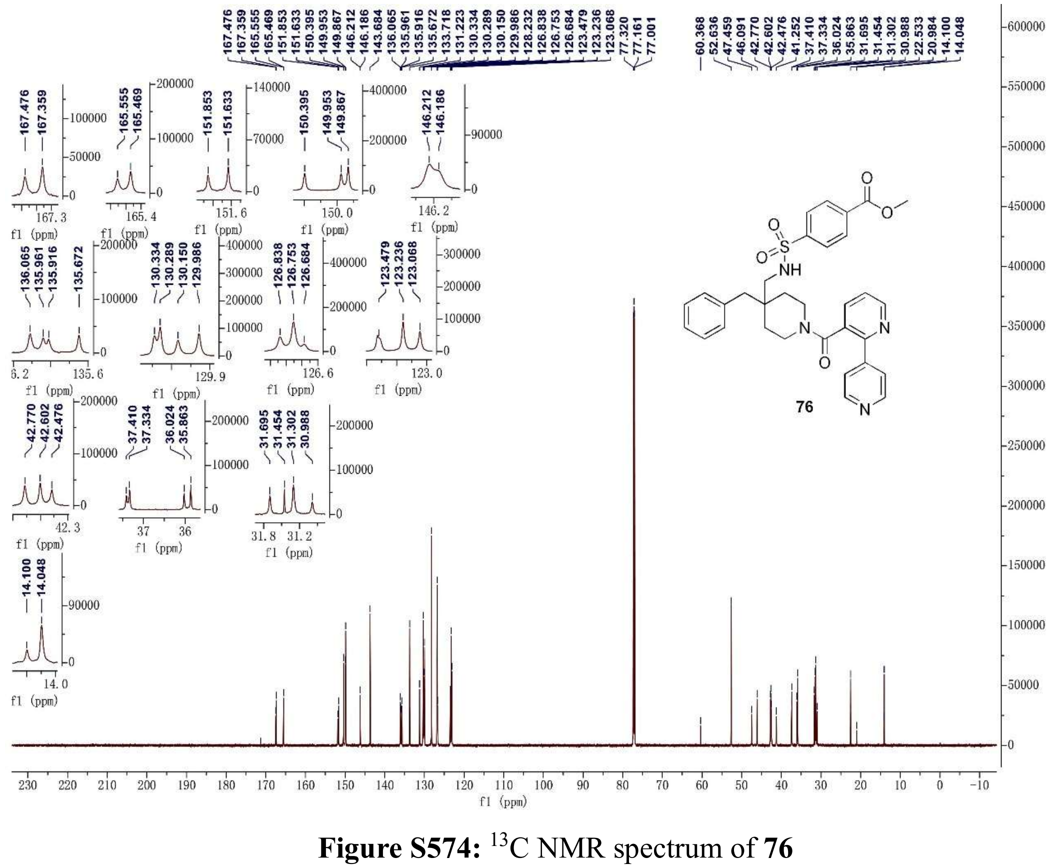

Figure S574: ^{13}C NMR spectrum of **76**

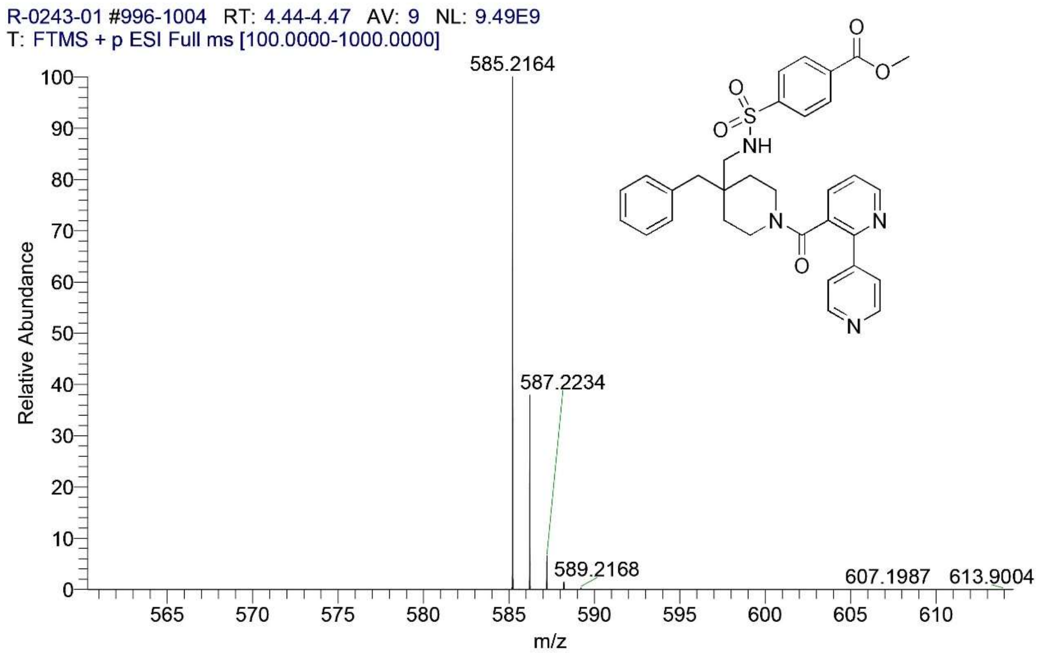

Figure S575: HR-MS (ESI/ion trap) spectrum of **76**

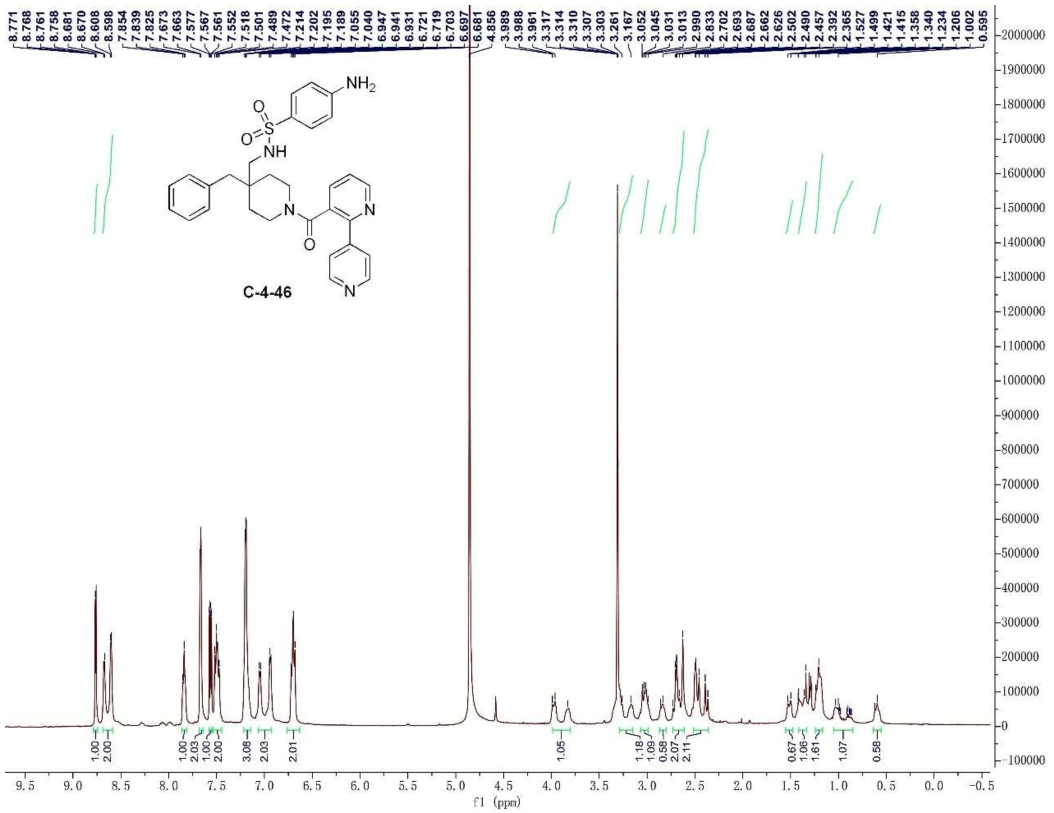

Figure S576: ^{1}H NMR spectrum of **C-4-46**

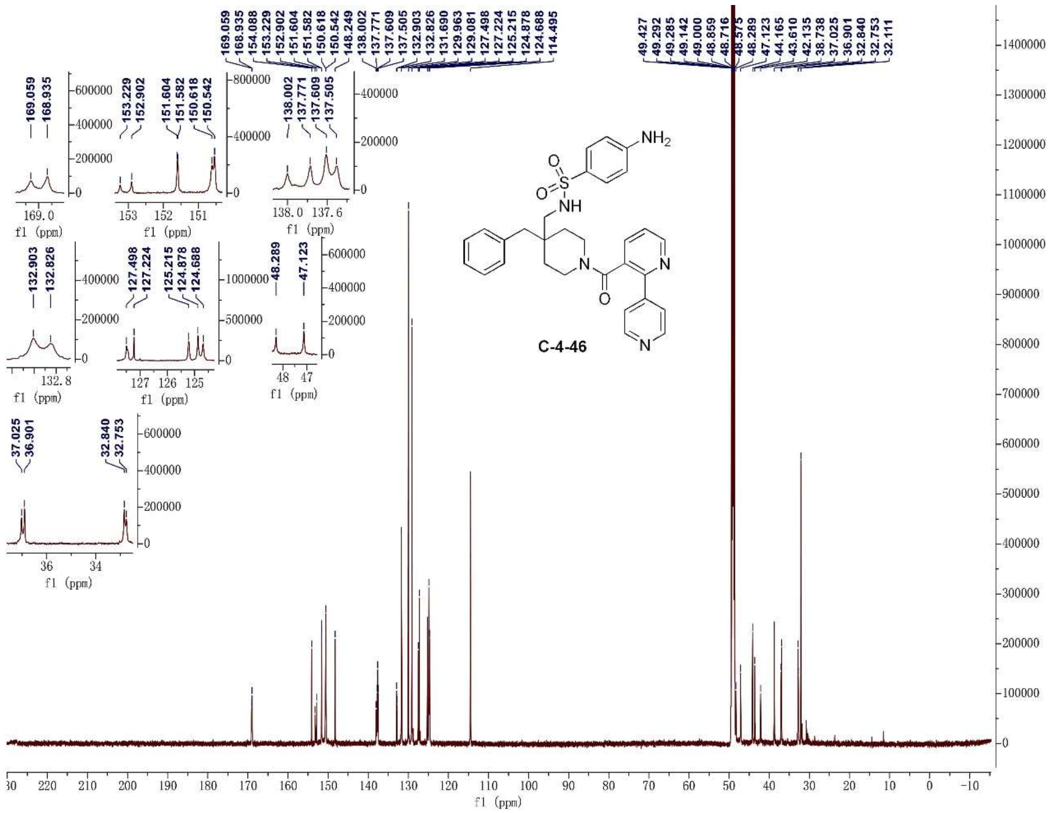

Figure S577: ^{13}C NMR spectrum of **C-4-46**

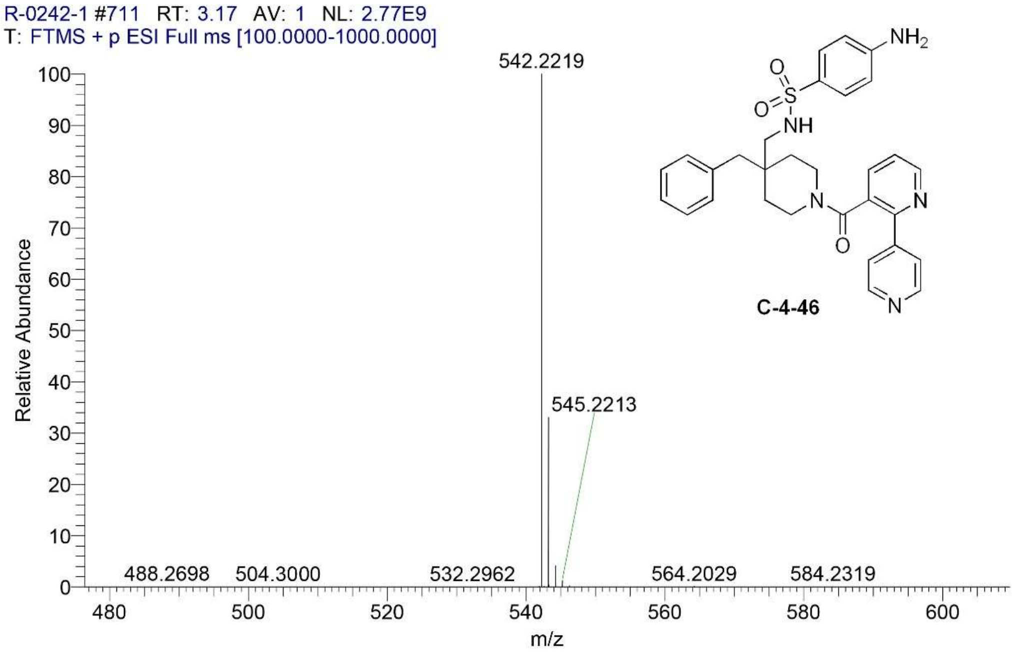

Figure S578: HR-MS (ESI/ion trap) spectrum of **C-4-46**

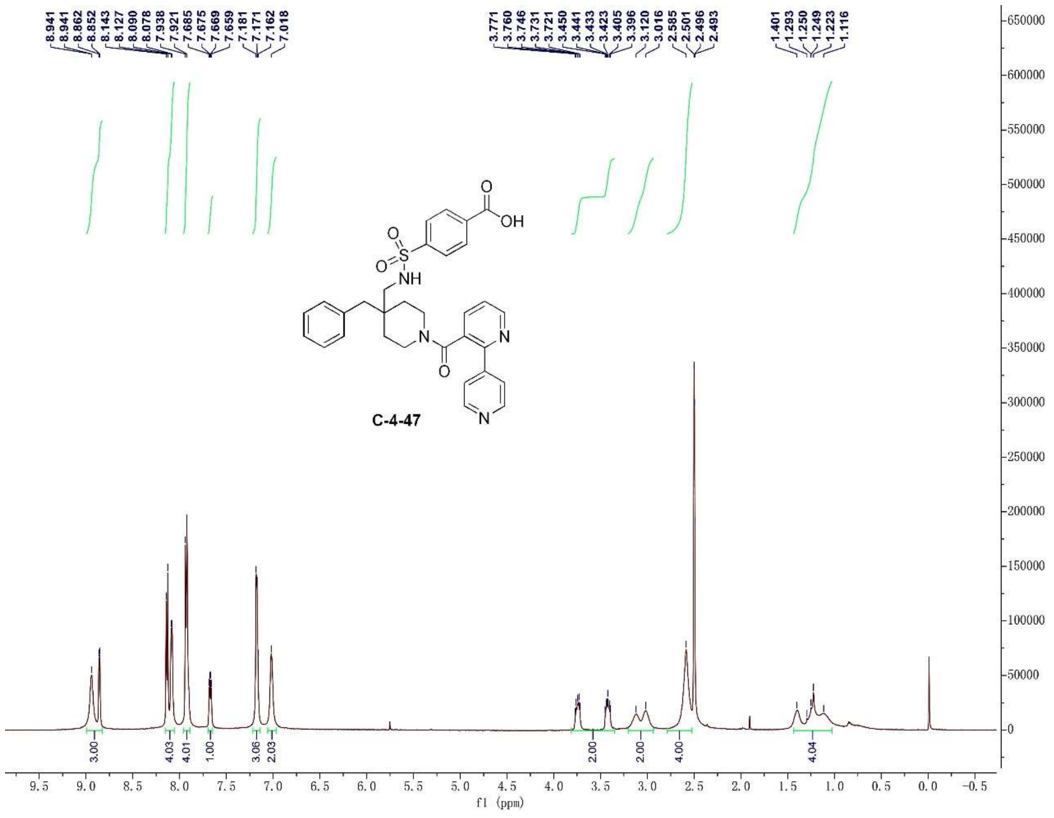

Figure S579: ^{1}H NMR spectrum of **C-4-47**

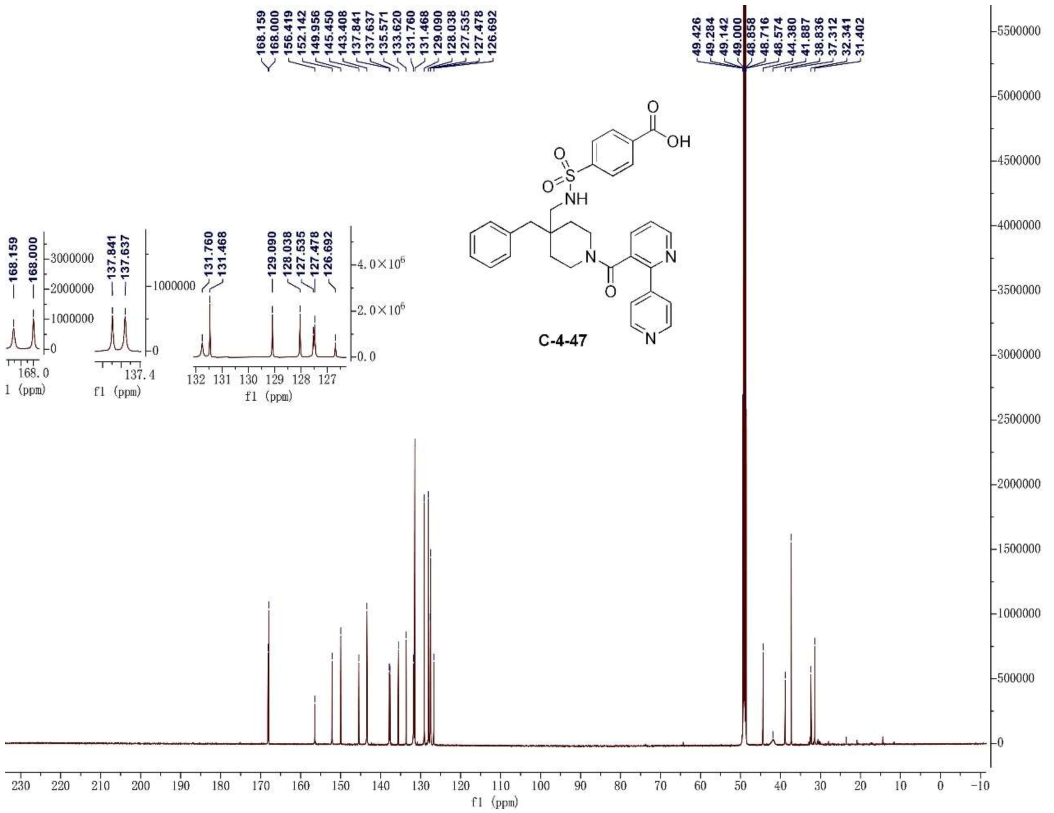

Figure S580: ^{13}C NMR spectrum of **C-4-47**

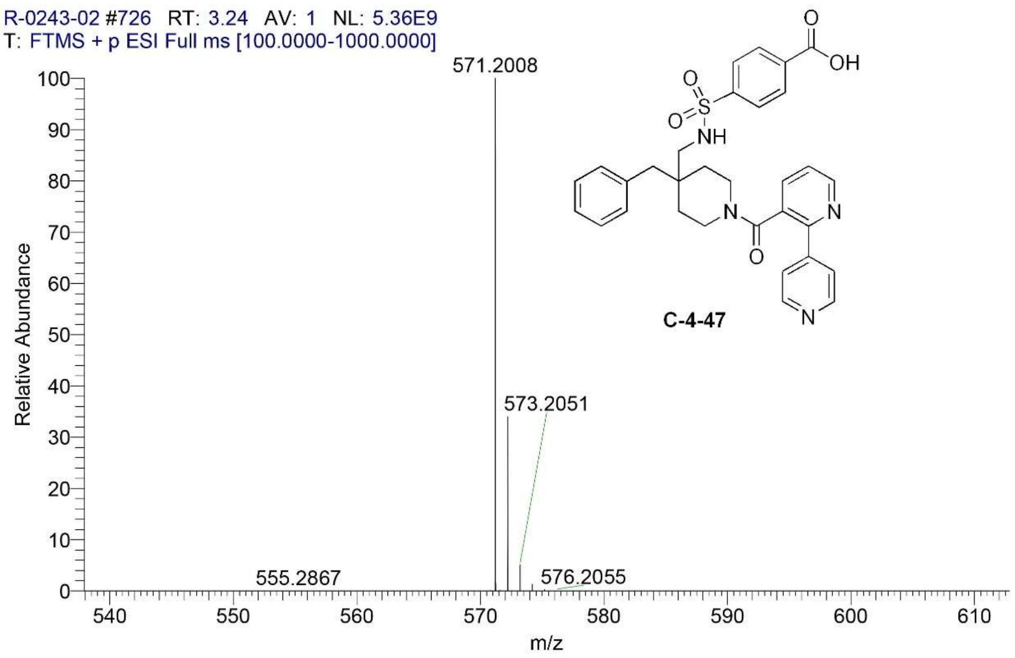

Figure S581: HR-MS (ESI/ion trap) spectrum of **C-4-47**

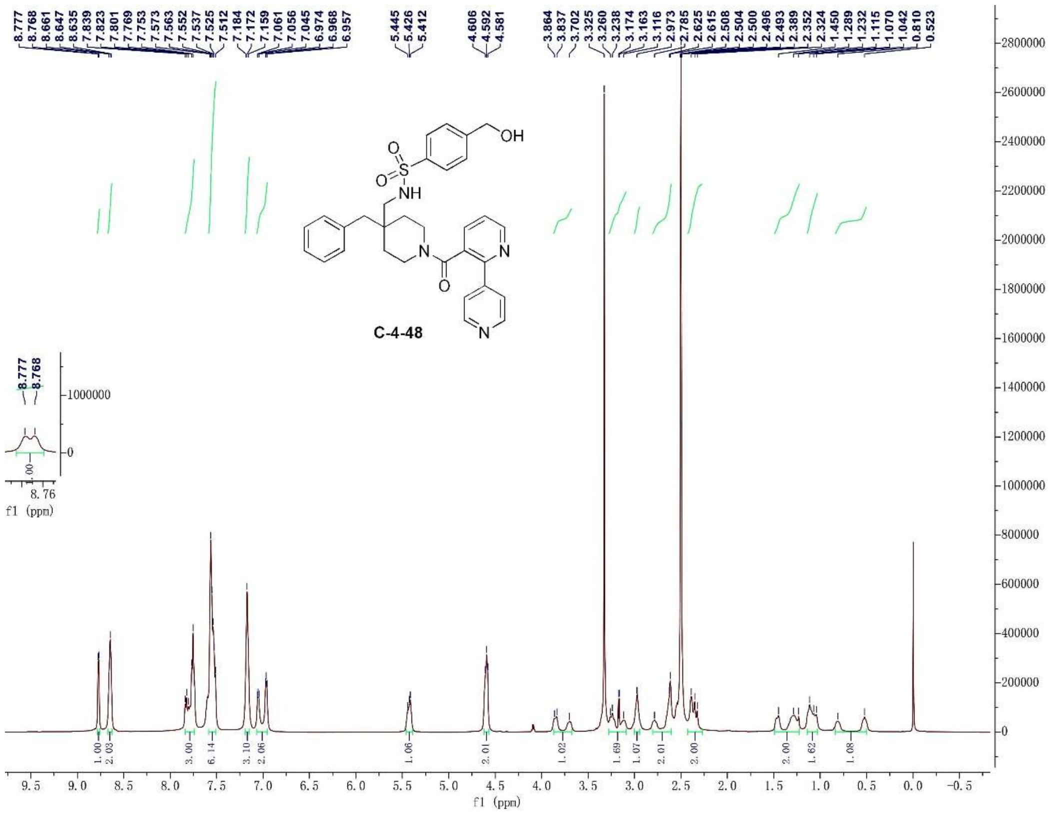

Figure S582: ^{1}H NMR spectrum of **C-4-48**

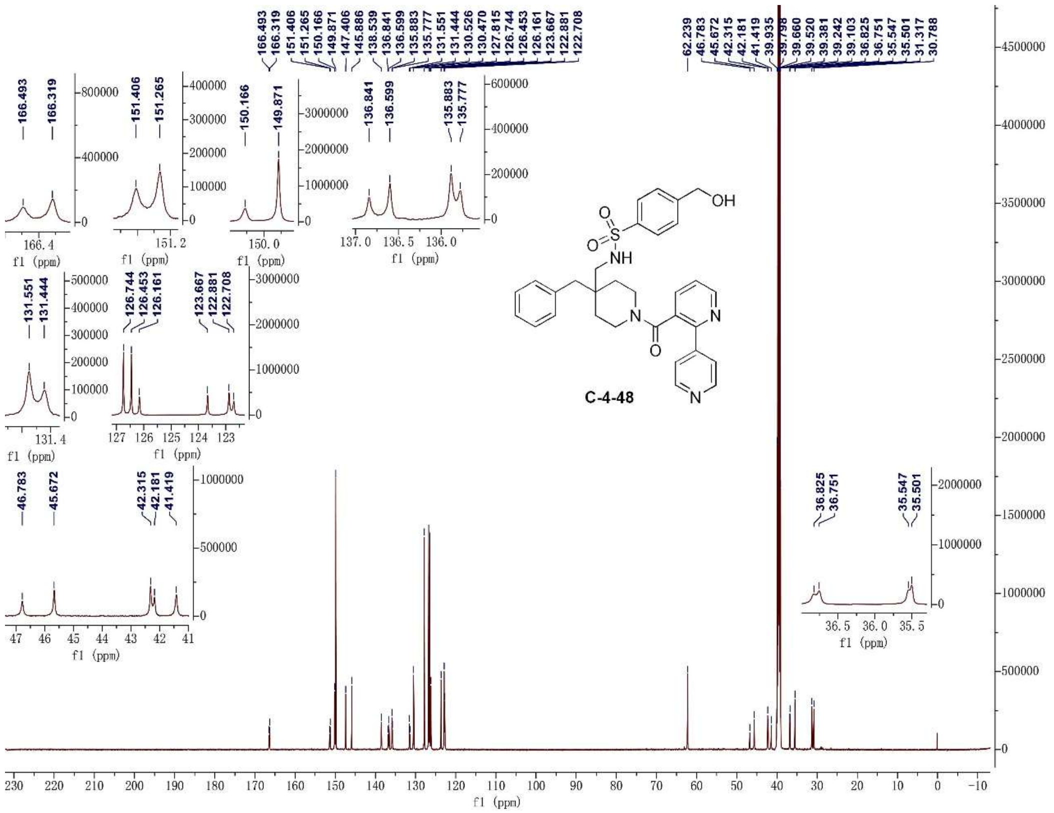

Figure S583: ^{13}C NMR spectrum of **C-4-48**

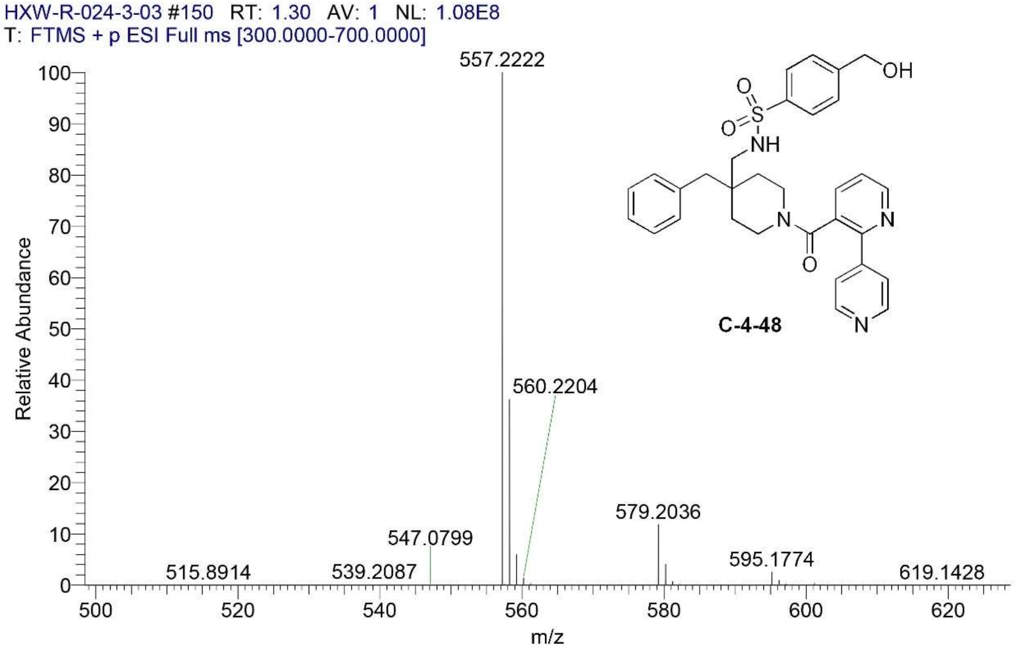

Figure S584: HR-MS (ESI/ion trap) spectrum of **C-4-48**

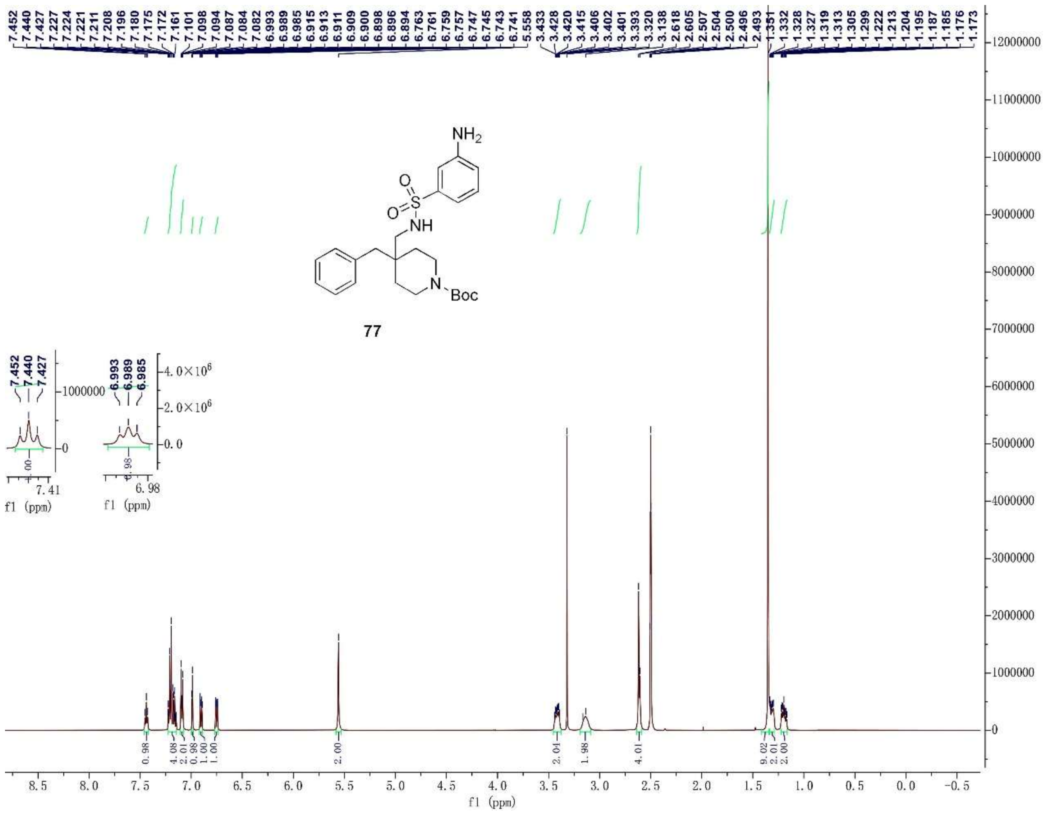

Figure S585: ^{1}H NMR spectrum of **77**

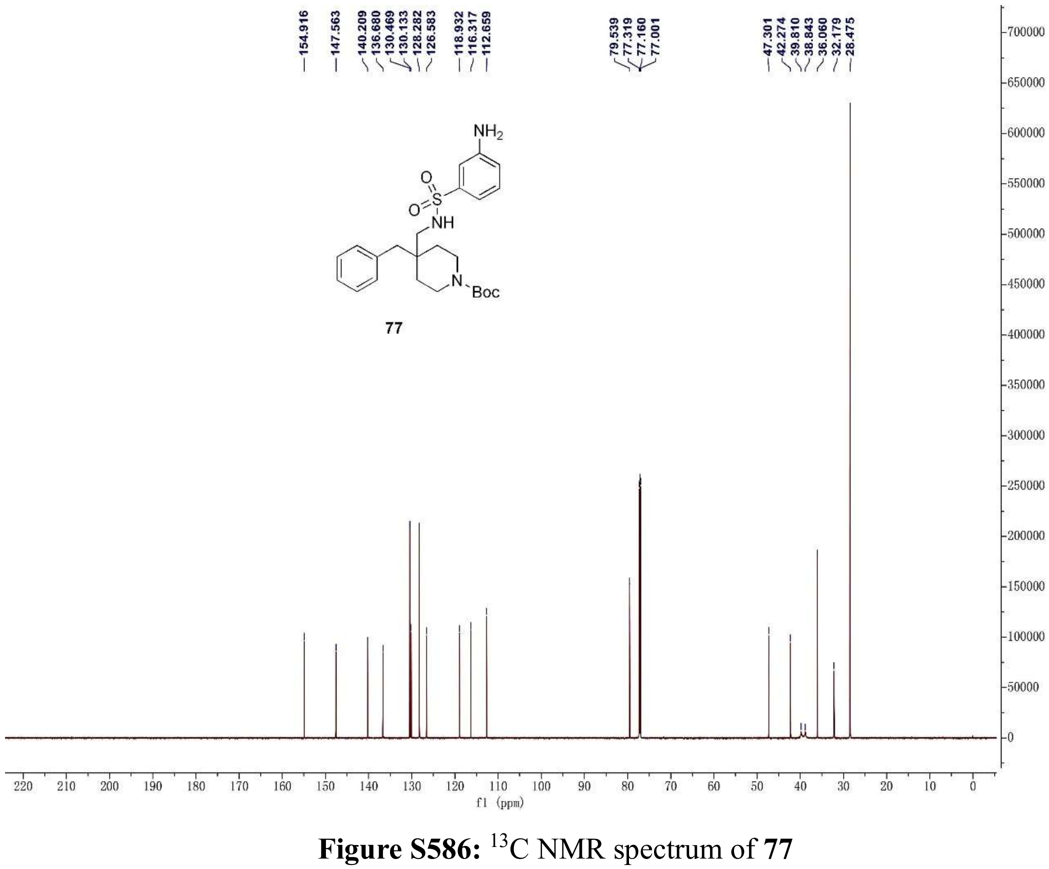

Figure S586: ^{13}C NMR spectrum of **77**

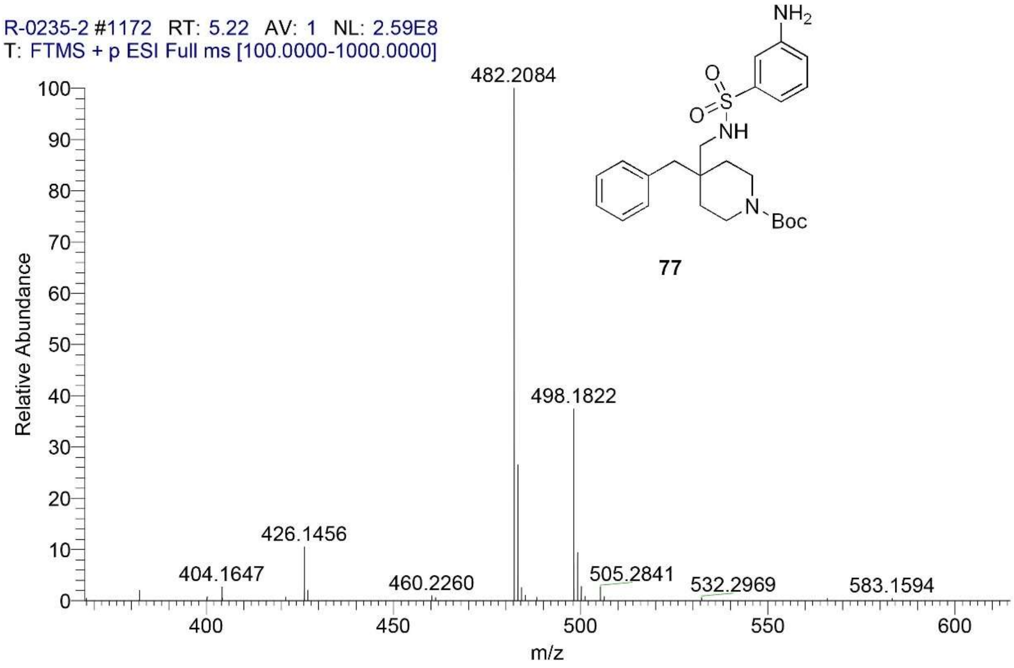

Figure S587: HR-MS (ESI/ion trap) spectrum of **77**

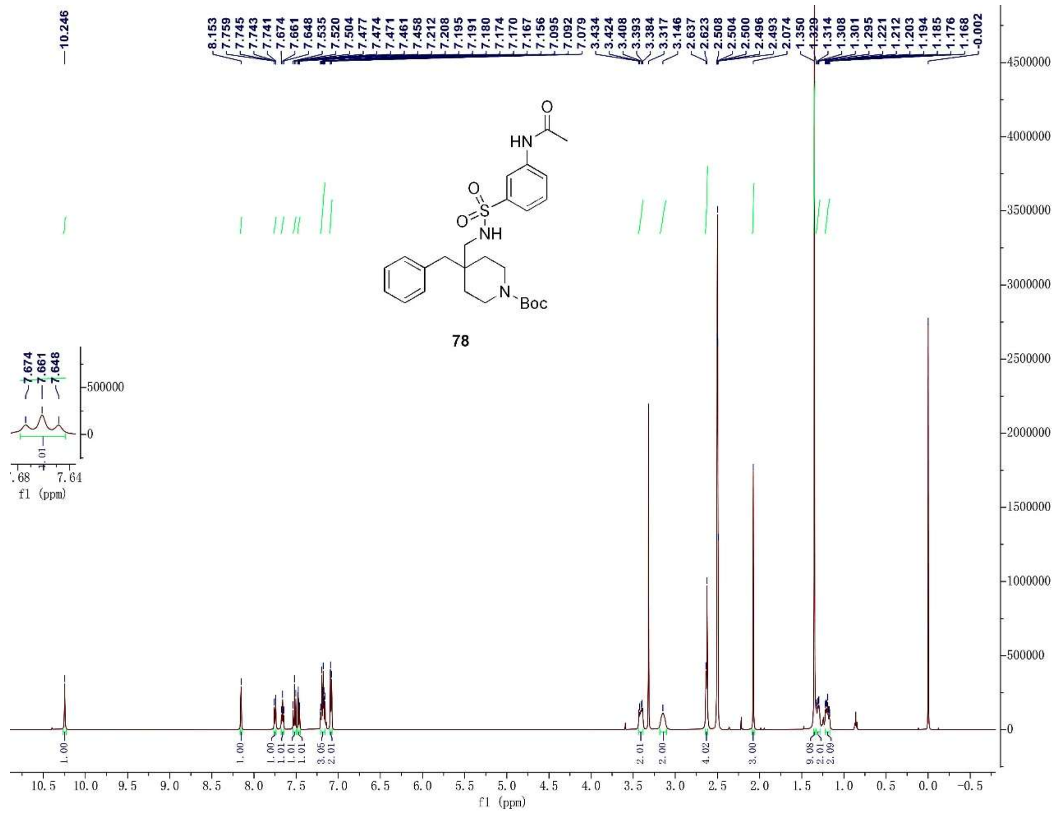

Figure S588: ^{1}H NMR spectrum of **78**

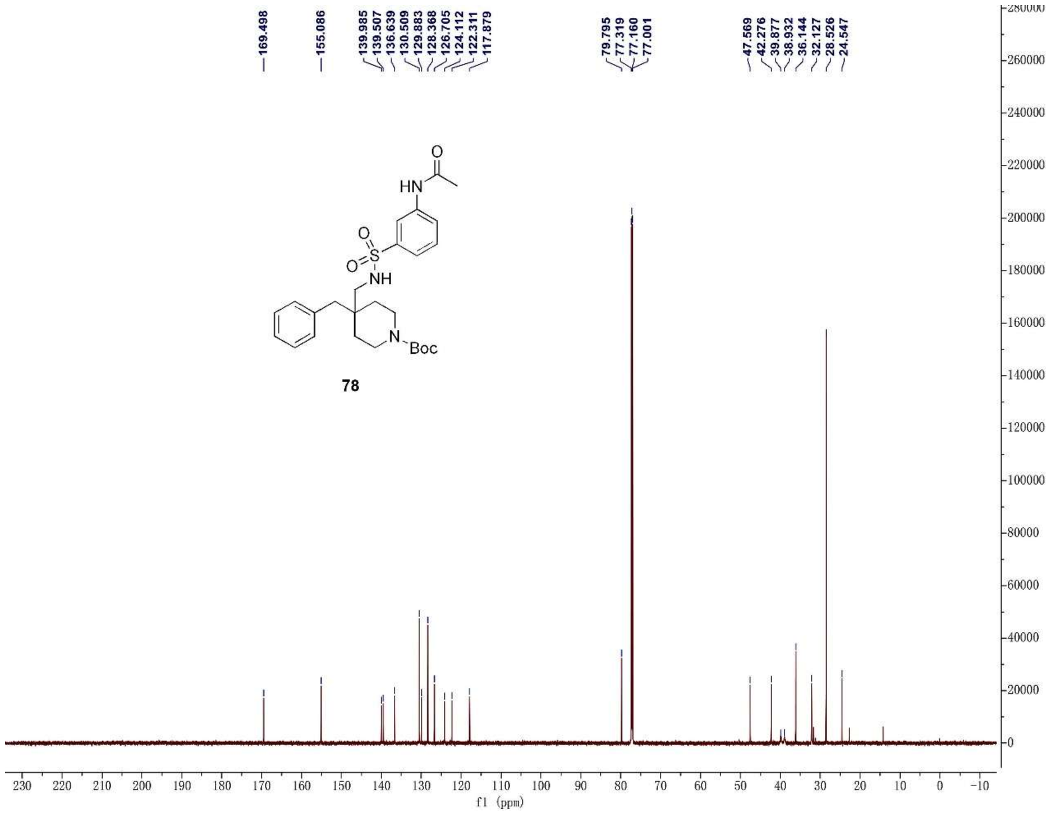

Figure S589: ^{13}C NMR spectrum of **78**

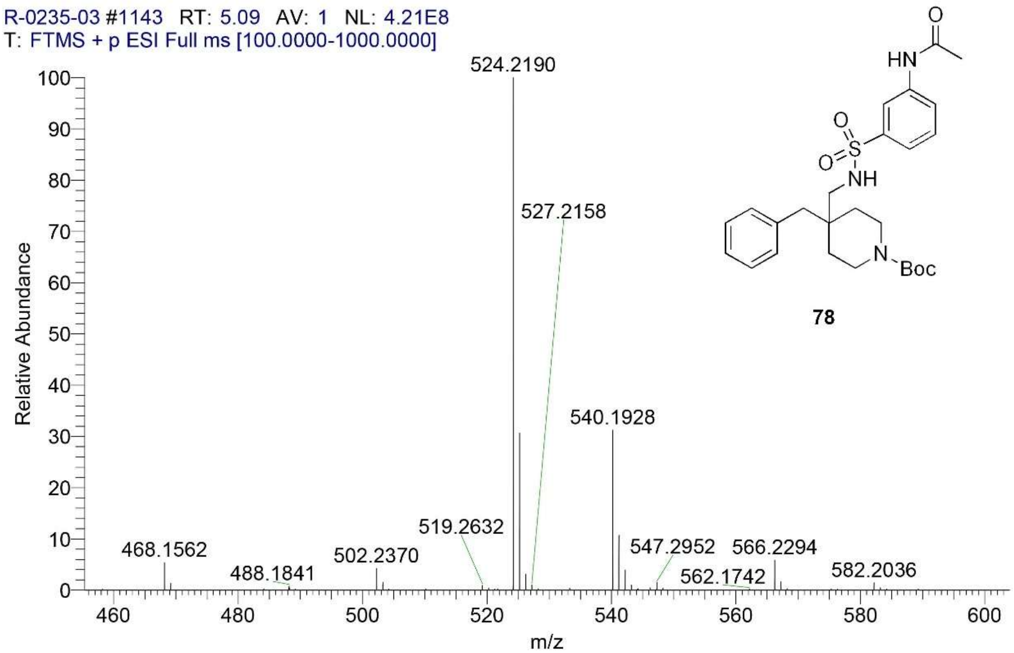

Figure S590: HR-MS (ESI/ion trap) spectrum of **78**

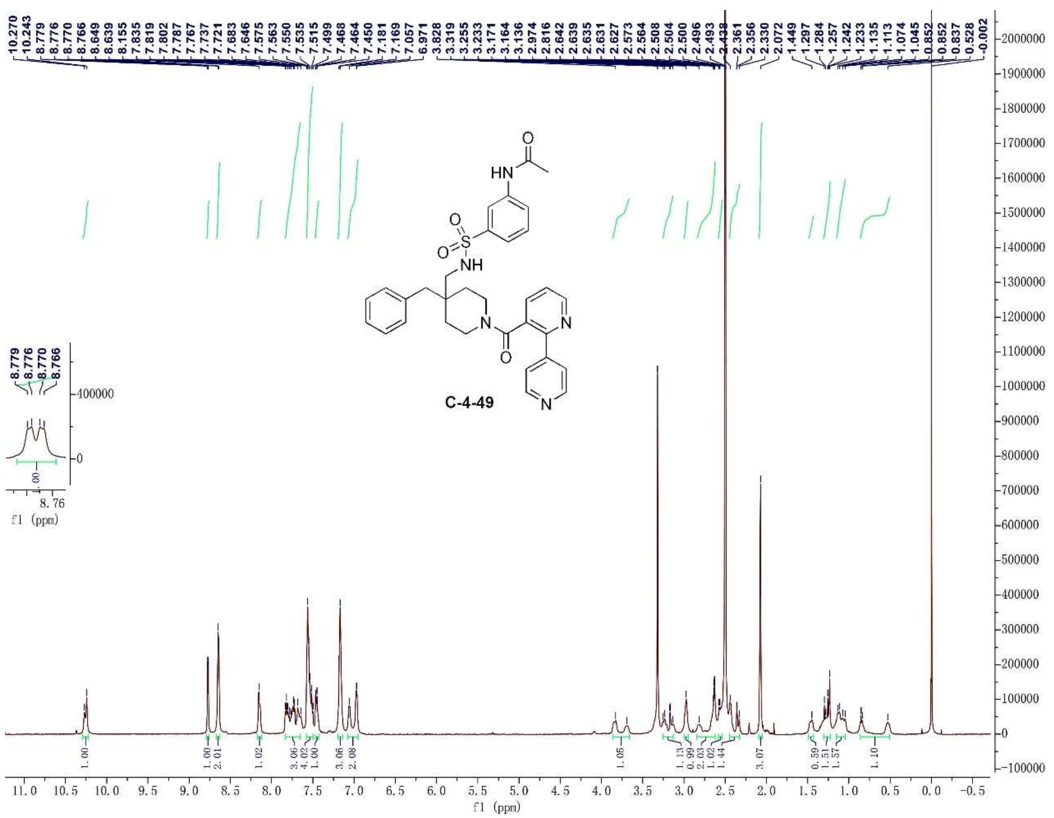

Figure S591: ^{1}H NMR spectrum of **C-4-49**

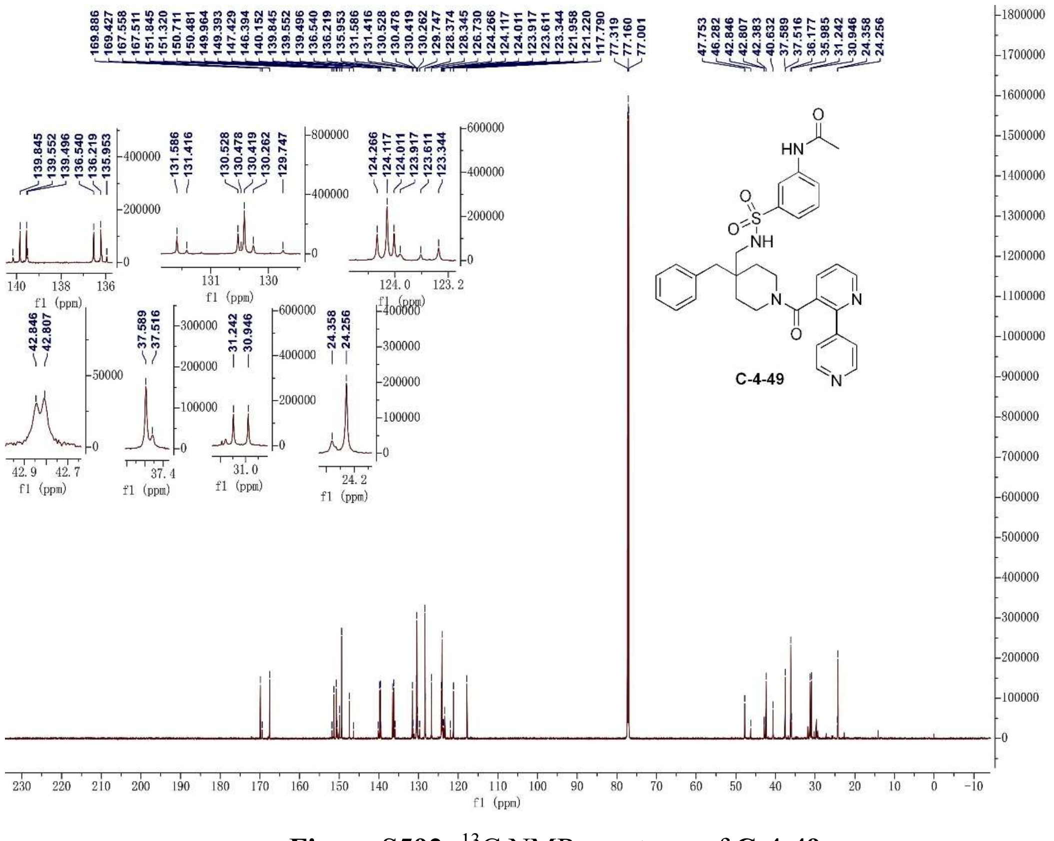

Figure S592: ^{13}C NMR spectrum of **C-4-49**

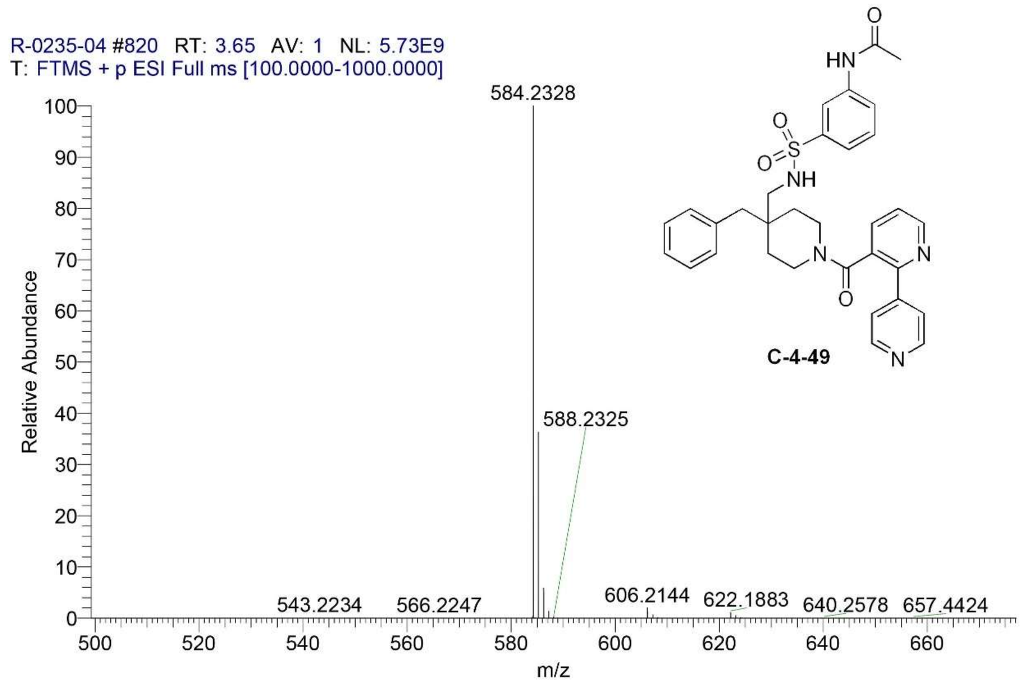

Figure S593: HR-MS (ESI/ion trap) spectrum of **C-4-49**
